# Allogenic and Autogenic Signals in the Stratigraphic Record of the Deep-Sea Bengal Fan

**DOI:** 10.1038/s41598-018-25819-5

**Published:** 2018-05-22

**Authors:** Mike Blum, Kimberly Rogers, James Gleason, Yani Najman, Jarrett Cruz, Lyndsey Fox

**Affiliations:** 1 Department of Geology, University of Kansas, Lawrence, Kansas USA; 2 Institute for Arctic and Alpine Research, University of Colorado, Boulder, Colorado, USA; 3 Department of Earth and Environmental Sciences, University of Michigan, Ann Arbor, Michigan USA; 4 Lancaster Environment Centre, Lancaster University, Lancaster, UK; 5 Department of Earth, Ocean, and Atmospheric Science, Florida State University, Tallahassee, Florida USA; 6 Department of Earth Sciences, The Natural History Museum, London, UK

## Abstract

The Himalayan-sourced Ganges-Brahmaputra river system and the deep-sea Bengal Fan represent Earth’s largest sediment-dispersal system. Here we present detrital zircon U-Pb provenance data from Miocene to middle Pleistocene Bengal Fan turbidites, and evaluate the influence of allogenic forcing vs. autogenic processes on signal propagation from the Himalaya to the deep sea. Our data record the strong tectonic and climatic forcing characteristic of the Himalayan system: after up to 2500 km of river transport, and >1400 km of transport by turbidity currents, the U-Pb record faithfully represents Himalayan sources. Moreover, specific U-Pb populations record Miocene integration of the Brahmaputra drainage with the Asian plate, as well as the rapid Plio-Pleistocene incision through, and exhumation of, the eastern Himalayan syntaxis. The record is, however, biased towards glacial periods when rivers were extended across the shelf in response to climate-forced sea-level fall, and discharged directly to slope canyons. Finally, only part of the record represents a Ganges or Brahmaputra provenance end-member, and most samples represent mixing from the two systems. Mixing or the lack thereof likely represents the fingerprint of autogenic delta-plain avulsions, which result in the two rivers delivering sediment separately to a shelf-margin canyon or merging together as they do today.

## Introduction

Source-to-sink (S2S) concepts have focused attention on coupling between sediment production in orogenic source terrains, routing and storage of sediments through fluvial systems, and accumulation in deltaic to basin-floor sinks^1^. Much of the S2S approach is grounded on insights from modern systems, where rates, processes, and characteristics of source terrains can be quantified. Inverting concepts from the modern world to interpret Earth history from the stratigraphic record in the depositional sink raises a number of important questions. For example, the primary allogenic drivers for erosion and sediment transfer to the land-sea boundary include tectonic and geodynamic processes that build topography, climatically- and tectonically-driven erosion, climate change, and climate-forced sea-level change. But how faithfully are allogenic signals such as these transferred to sinks in the deep sea, and how do we disentangle allogenic forcing from signals of autogenic surface dynamics?

**Figure 1:**
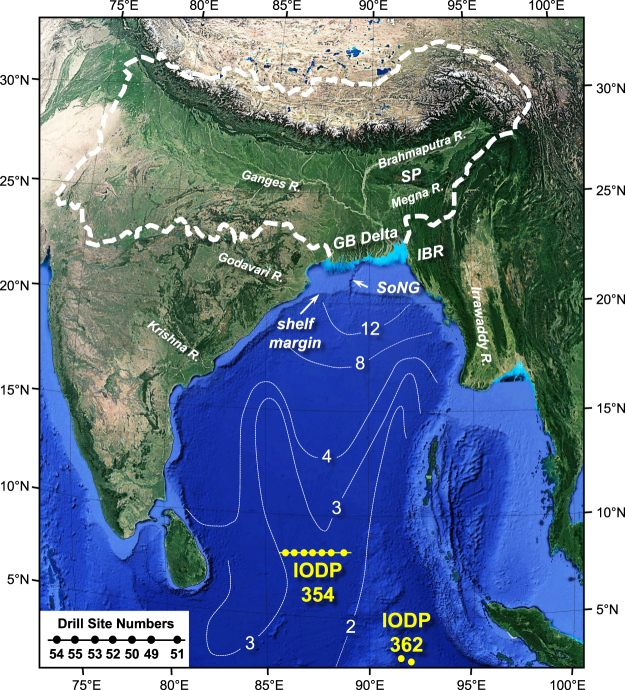
The Ganges-Brahmaputra sediment-dispersal system from Google Earth imagery (Map data: SIO, NOAA, U.S. Navy, NGA, GEBCO; Image; Landsat/Copernicus). Solid white line outlines the combined Ganges-Brahmaputra contributing drainage area. Light blue colors along the delta front roughly correspond to the mud transport system that has produced a muddy subaqueous clinothem^18^. SoNG indicates the “Swatch of No Ground” modern shelf-penetrating canyon. Thin white dashed lines are post-Paleocene Bengal Fan thickness contours (in kilometers)^17,29^. The location of IODP Expedition 354 drill sites are shown within the Bay of Bengal, with specific core numbers shown in lower left inset (formal core numbers identified by U1454, U1455, etc.). SP = Shillong Plateau and IBR = Indo-Burman Range, with the location of IODP Expedition 362 drill sites on the Nicobar Fan as shown^28^.

In February and March 2015, International Ocean Discovery Program Expedition 354 (hereafter IODP 354) drilled a 7-site transect in the Bay of Bengal to collect core from the deep-sea Bengal Fan **(**Fig. 1**)**, the terminal sink for the Himalayan-sourced Ganges and Brahmaputra Rivers. A key objective was to expand the record of source-to-sink sediment transport from the Himalaya to the deep sea^2^. Here we present a new body of detrital-zircon U-Pb (hereafter DZ U-Pb) data from sandy and silty turbidites of the Bengal Fan, and use these data to evaluate the record of signal transfer in the Miocene to middle Pleistocene stratigraphic record.

## Background

**Figure 2:**
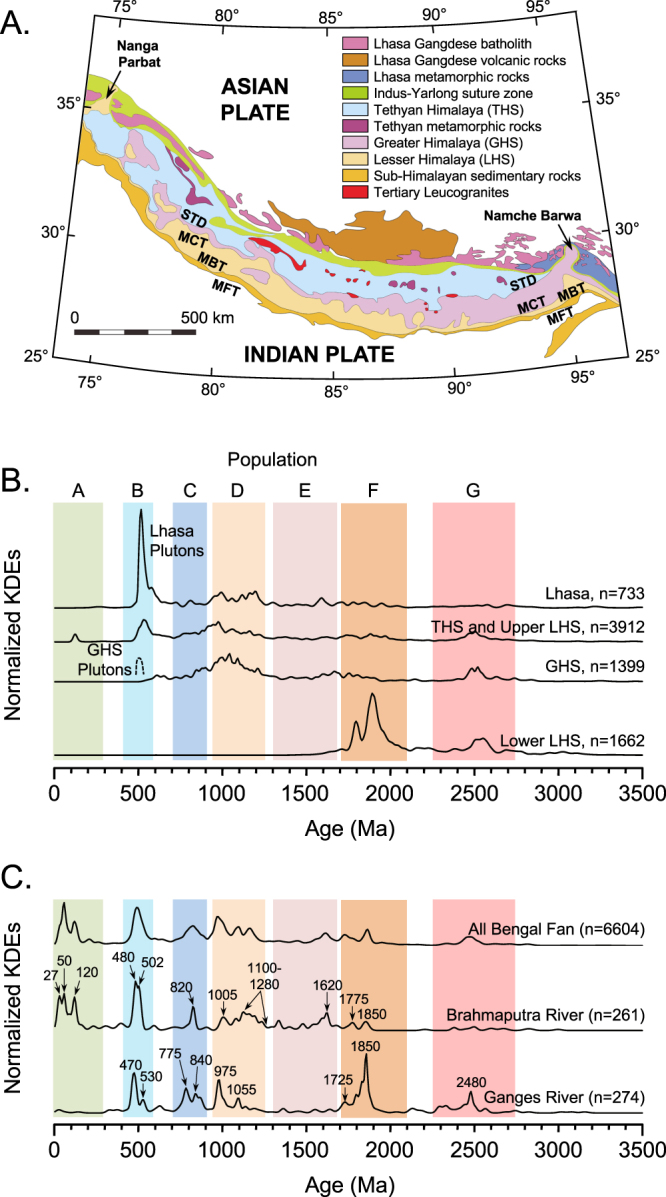
(**A**) Map illustrating major tectonostratigraphic domains and zircon source areas within the Himalaya, which are relevant to this study, modified and simplified from^7^ with data from^3,4,10^. STD = South-Tibetan Detachment, MCT = Main Central Thrust, MBT = Main Boundary Thrust, MFT = Main Frontal Thrust, Nanga Parbat = western syntaxis, Namche Barwa = eastern syntaxis. The Indus-Yarlong suture zone (Indus-Tsangpo or ITS in the text) separates Asia from India. (**B**) Normalized kernel-density estimates (KDEs) for zircon (bedrock and detrital) U-Pb ages from major tectonostratigraphic domains calculated from data published in^37^. Here and in Fig. 3, populations A-G represent informal groups for discussion purposes. GHS plutons are schematic, and plot does not include Mesozoic and Cenozoic ages from Lhasa or the eastern syntaxis. (**C**) Normalized KDEs for the modern Ganges and Brahmaputra Rivers, compared with a composite plot for all Bengal Fan samples. Distinctive peaks as shown for the modern Ganges and Brahmaputra Rivers.

The Himalayan-sourced Ganges-Brahmaputra River system (GB) and the deep-sea Bengal Fan represents Earth’s largest modern-day source-to-sink sediment-dispersal system. Himalayan orogenesis is driven by intercontinetal collision between the Indian and Asian plates, which began as early as ca. 59 Ma. and is still ongoing^3–7^. Headwaters of the Brahmaputra lie within the Lhasa terrane (hereafter Lhasa) on the Tibetan Plateau of the Asian plate, to the north of the Indus-Tsangpo Suture (ITS). Lhasa includes Cambrian-age granites, and Paleozoic and Mesozoic clastics intruded by Jurassic through Paleogene granitoids of the pre-collision Gangdese arc^8,9^. Headwaters of the Ganges, and many tributaries to the Brahmaputra, are restricted to the Himalaya and the Indian plate, which extends from the ITS in Tibet to the Main Frontal Thrust in the south. There is much confusion between geologic and geographic terminology, but Himalayan rocks represent four tectonostratigraphic sequences that extend from west to east, and which are generally separated by north-dipping crustal-scale faults^10^. From north to south, as used here these are: (1) the Tethyan Himalaya Sequence (THS), comprised primarily of Paleozoic and Mesozoic sedimentary rocks, with leucogranites of Neogene age^11^; (2) the Greater Himalaya Sequence (GHS) is separated from the THS by the South Tibetan detachment, a normal fault, and is comprised of Late Neoproterozoic to Ordovician high-grade metamorphic and plutonic rocks, also with leucogranites of Neogene age; (3) the Lesser Himalaya Sequence (LHS) is separated from the GHS by the Main Central Thrust, and is comprised of Paleoproterozoic and older metasedimentary and igneous rocks; and (4) the Sub-Himalaya is separated from the LHS by the Main Boundary Thrust, and is comprised of mostly Neogene foreland-basin sediments **(**Fig. 2**)**. The THS, GHS, and LHS dominate the geographically-defined Tethyan, Greater (or Higher), and Lesser (or Lower) Himalaya, respectively, but the geographic Greater Himalaya includes THS rocks as structural outliers, and the geographic Lesser Himalaya includes THS and GHS structural outliers.

The modern-day Ganges-Brahmaputra system (hereafter the GB) has a contributing drainage area of >2*10^6^ km^2^, which includes much of the high-relief Himalaya and the southern Tibetan Plateau. The Ganges and Brahmaputra emerge from the Sub-Himalaya, route sediment through the foreland basin while gathering tributary inputs from the Himalaya to the north, Peninsular India to the south, and the Indo-Burman Ranges to the East, and then discharge to the delta plain and Bengal basin in the northern Bay of Bengal. Each river has a monsoon-driven discharge regime with 1–3 m of precipitation per year^12^, and the GB system delivers >1 Gt of sediment to the delta plain in India and Bangladesh^13,14^: an estimated 30% of the total modern load comes from the Ganges, whereas up to 70% is derived from the Brahmaputra. Rates of erosion generally follow precipitation trends, and are low in the dry Tethyan Himalaya, then increase to the south and east with increases in monsoon precipitation, as GB tributaries cut through the Greater and Lesser Himalaya^15,16^. The GB system therefore includes strong tectonic and climatic forcing, which drives high rates of sediment production and transfer to the foreland basin, the delta plain and the land-sea boundary.

The Holocene GB delta overlies a post-Paleocene Bengal basin depocenter that is up to 20 km in thickness^17^. The Holocene delta resides on the inner shelf, and includes an aggradational and progradational subaerial topset and foreset, and an active mid-shelf subaqueous clinoform^18^. Large GB sediment loads drive aggradation of the subaerial delta-plain, and high aggradation rates promote frequent avulsions^19,20^. For example, the Ganges and Brahmaputra merge in Bangladesh today, but the Ganges discharged to the Bay of Bengal >250 km west of the present-day river mouths as late as the 1600s^21^: a distributary channel still connects to this course, and may transport a sediment load similar to the Ganges below the bifurcation^22^. Similarly, the Holocene Brahmaputra has avulsed at least 3 times^23^. Avulsions of this kind typically represent inherent, autogenic behavior in aggradational fluvial-deltaic systems^24^ and have produced the ~400 km wide swath of Holocene GB alluvial-deltaic sediments. The delta is connected to the modern Bengal Fan system via the shelf-penetrating “Swatch-of-no-Ground” canyon (SoNG), which is inherited from the last glacial period when rivers were extended across the shelf due to climate-forced sea-level fall^17^.

The Bengal Fan extends ~2500–3000 km south into the Bay of Bengal and Indian Ocean, and is Earth’s largest deep-sea fan. Post-collision Himalayan sediment first appears in the Bengal Basin by 38 Ma^25^, but the Bengal Fan emerged as a distinct entity in the deep Bay of Bengal by the Late Oligocene to Early Miocene^26^, roughly coincident with an increase in Himalayan exhumation^27^. The Nicobar Fan, located in the eastern Bay of Bengal adjacent to the Sunda accretionary prism (Fig. 1), is a lobe within the greater Bengal Fan system and has over time received significant sediment from the GB as well^28^. Thickness of post-Paleocene Bengal Fan sediment tapers from ~12 km near the toe of the continental slope to <1 km over a distance of ~2500 km^17,29^. IODP 354 drill sites at 8°N latitude are located ~1400 km to the south of the modern shelf margin: significant silt- and sand-rich fan deposition at this latitude commenced ca. 18 Ma, with Miocene to present thicknesses of >1100 m [see Fig. SR3]. At ~3°N, where IODP Expedition 362 collected cores through the Nicobar Fan, Miocene to present sediments exceed 1200 m in thickness^28^.

Basic patterns of erosion and source-to-sink sediment transfer have been in place through the Neogene and Quaternary^30^, but details of sediment routing have changed through time. The most significant changes are: (1) continued north-northeast motion of the Indian plate relative to Eurasia^31^ such that the location of IODP 354 drill sites would have been ~950 km and ~400 farther south with respect to the GB delta at ca. 20 and 10 Ma, respectively; (2) paleogeographic changes in the GB drainage area, in particular the Miocene capture of Tibetan drainage by the Brahmaputra and its tributaries^32,33^; (3) the Late Miocene-Pliocene incision of the Brahmaputra through the Tsangpo Gorge in the rapidly uplifting eastern Himalayan syntaxis^34^; and (4) westward migration of the Brahmaputra river and delta due to westward propagation of the IBR and uplift of the Shillong Plateau since 5 Ma^35^, which reduced the east-west dimensions of the Bengal basin by >200 km. Last, the SoNG is the only major shelf-penetrating canyon today, however, older shelf-penetrating canyons are likely common in the subsurface where they have been buried by younger shelf-margin aggradation and progradation.

## Results

Our data consists of U-Pb and Pb-Pb zircon crystallization ages obtained by laser-ablation ICP-MS analyses of detrital-zircon grain interiors: we have 535 concordant ages from samples collected from the modern Ganges and Brahmaputra Rivers, and 6602 concordant ages from twenty-five samples from IODP 354 cores through the middle Bengal Fan **(**Table 1**)**. Modern river samples were collected from sand bars located on the modern Ganges and Brahmaputra ~80–120 km upstream from their present confluence. Bengal Fan samples cover the period of turbidite fan deposition at the location of IODP Expedition 354 and are early Miocene (Burdigalian) to middle Pleistocene in age (ca. 18 to 0.2 Ma) based on shipboard^2^ and post-shipboard biostratigraphic constraints (Table S1 and S2). Silt-rich turbidites were sampled for the ca. 18–10 Ma record, but post-10 Ma samples were fine-grained sand. Our interpretations are guided by previously published bedrock- and detrital-zircon U-Pb age populations from Tibetan and Himalayan tectonostratigraphic sequences **(**Fig. 2B) [e.g.^36–40^], DZ U-Pb signatures of time-equivalent Neogene foreland-basin sediments of the Sub-Himalaya **[**e.g.^41,42^] and ancestral Brahmaputra sediments to the south of Shillong Plateau^32^, and DZ U-Pb data from the modern Ganges, modern Brahmaputra or their tributaries within or proximal to the Himalaya^41,42^.

**Table 1:** Summary of detrital zircon samples from IODP Expedition 354.

Sample #	Drill Site	Hole	Core	Depth (Mbsf)	Numerical Age Estimate (Ma)	Stratigraphic Age	n
1	U1451	A	4H–6H	25–35	0.3	Middle Pleistocene	273
2	U1450	A	6F–8F	30–44	0.4	Middle Pleistocene	263
3	U1452	B	8F	49–51	0.5	Middle Pleistocene	273
4	U1453	A	11F	370–385	0.5	Middle Pleistocene	268
6	U1453	A	26F	467	0.6	Middle Pleistocene	276
5	U1451	A	13F	70–75	0.6	Middle Pleistocene	259
7	U1452	B	38F	190–192	1.3	Early Pleistocene	260
8	U1453	A	32F	24–40	1.3	Early Pleistocene	291
9	U1449	A	29F–31F	74	1.5	Early Pleistocene	278
10	U1450	A	70F	332–338	2.9	Late Pliocene	259
11	U1450	A	78F–80F	360–370	3.2	Late Pliocene	259
12	U1450	A	98F	465–470	3.5	Late Pliocene	273
13	U1450	A	124F	600–605	3.6	Early Pliocene	271
16	U1451	A	37F	225–230	6.3	Late Miocene	267
17	U1451	A	41F	245–250	6.5	Late Miocene	262
14	U1451	A	49F	280–285	7.2	Late Miocene	282
18	U1451	A	60F	335–340	7.5	Late Miocene	264
19	U1451	A	66F	365–370	8.4	Late Miocene	271
20	U1451	A	80F	430–435	8.7	Late Miocene	264
15	U1451	A	102F	535–540	9.8	Late Miocene	269
21	U1451	B	3X	550–555	10.2	Late Miocene	255
22	U1451	B	22R	715–725	11.5	Late Miocene	281
23	U1451	B	41R	900–905	14.0	Middle Miocene	273
24	U1451	B	53–54R	1015–1020	16	Early Miocene	201
25	U1451	B	62R	1085–1090	18	Early Miocene	210
26	M01	modern Ganges River			0	modern	261
27	J03	modern Brahmaputra River			0	modern	274

Depth reported in meters below sea floor (Mbsf), whereas numerical age estimates are based on best-fit interpolations between shipboard and post-cruise biostratigraphic constraints (Tables S1 and S2), and “n” = number of concordant U-Pb or Pb-Pb analyses in that sample. Additional data on sample context is provided in the Supplemental File, Figures S1–S4.

Our new modern river samples represent the integrated DZ U-Pb population for each river system as it enters the Bengal Basin, and define major populations and individual peaks that are diagnostic of the Ganges vs. Brahmaputra drainages as a whole. Figure 2C plots DZ U-Pb populations for the modern Ganges and Brahmaputra Rivers as normalized kernel-density estimates (KDEs), relative to the entire Bengal Fan dataset, which show that modern rivers contain all major Archean to Paleozoic zircon U-Pb populations that characterize the Himalaya. The modern Brahmaputra sample also contains a significant <300 Ma population (22% of the total) that is predominantly derived from the Jurassic through Paleogene Gangdese arc in Tibet **(**Fig. S4**)**, and possibly the GHS within the eastern Himalaya syntaxis (Neogene population only)^36,38,43–45^. All populations from the Himalaya are represented to some degree **(**Fig. 2B,C**)**, but there are significant differences in the representation of the different tectonostratigraphic sequences, and the erosional terrains they represent. The most notable of these is the paucity of the ca. 400–600 Ma population that characterizes the THS^37^ (Fig. S5**)**: in modern river samples this age population is dominated by late Cambrian and Ordovician ages typical of intrusive rocks of the GHS^39,41^ or perhaps Lhasa^8,37^. Moreover, there are differences in peaks between the two river samples: the modern Ganges contains a single prominent peak at ca. 470 Ma, whereas the modern Brahmaputra contains distinct peaks at ca. 480 and 500 Ma. Within the ca. 900–1250 Ma population, the modern Ganges contains a strong peak at ca. 975 Ma, common to the GHS, whereas the Brahmaputra is dominated by ages of ca. 1100–1230 Ma that are likely more typical of Lhasa^34^ **(**Fig. S7**)**. Last, the modern Ganges contains a significant Paleoproterozoic and Archean component, including peaks at ca. 1850 and 2475 Ma, which reflect derivation from Paleoproterozoic strata of the LHS and Peninsular India^39,40^.

**Figure 3:**
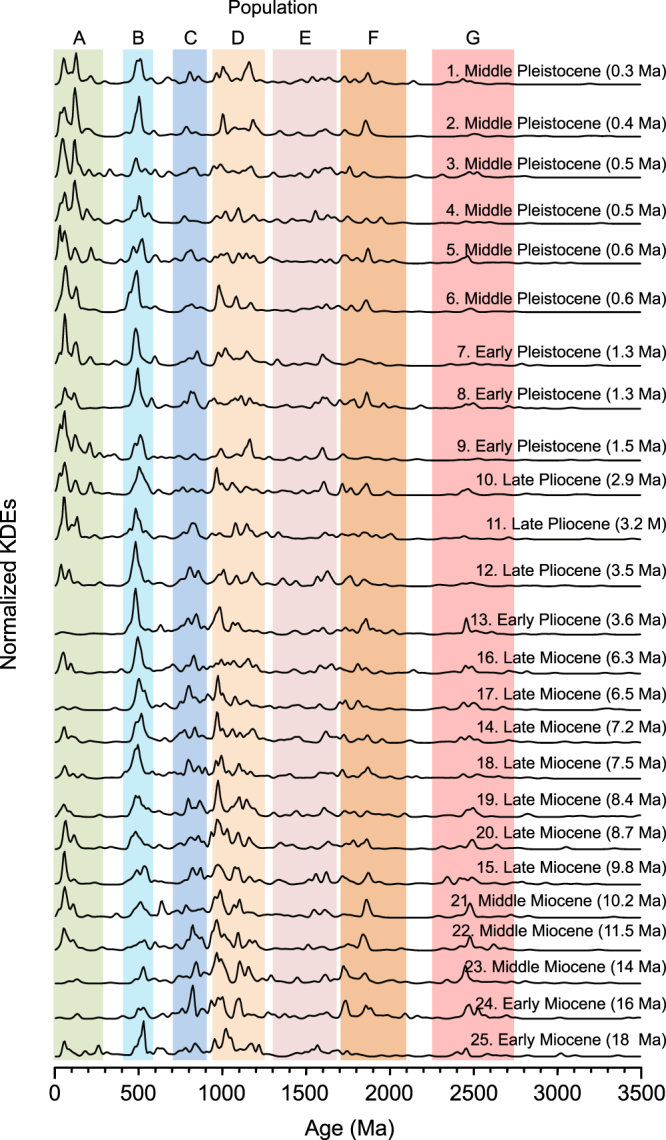
Stacked normalized KDE plots for Bengal Fan DZ samples, illustrating U-Pb age populations in each sample. Additional data, including KDE plots of specific age populations present in the modern Ganges and Brahmaputra Rivers, and the Bengal Fan as a whole, are included in Figures S4–S7, whereas multi-dimensional scaling plots are provided in Figure S8.

Figure 3 Summarizes normalized KDE plots of DZ U-Pb age populations for all Bengal Fan samples. Like modern river samples, each fan sample displays Archean to Paleozoic zircon U-Pb populations that characterize the Himalaya. The largest populations overall are ca. 400–600 Ma and 900–1250 Ma in age, which comprise ~17 and ~27 percent of the total, respectively. Similar to modern river samples, the ca. 400–600 Ma population of the Bengal Fan is dominated by late Cambrian and Ordovician ages typical of the GHS and Lhasa, each of the key peaks from modern river samples are well-represented, and the THS signal is relatively minor. Most Bengal Fan samples also include Mesozoic and Cenozoic populations (<300 Ma) from Lhasa, which comprises ~21% of the entire dataset, with broad peaks at ca. 50–60 and 110–130 Ma^36^ (Fig. S4**)**. Some ~20% of the <300 Ma population, or ~4% of the total, is ca. 14–36 Ma in age, which is consistent with derivation from Lhasa or Tertiary leucogranites of the THS and GHS^11,36^. A small number of grains (<0.5% of the total) from this <40 Ma population are <10 Ma in age, which likely come from the rapidly exhuming Namche Barwa massif of the eastern syntaxis^34,38,44–49^.

**Figure 4:**
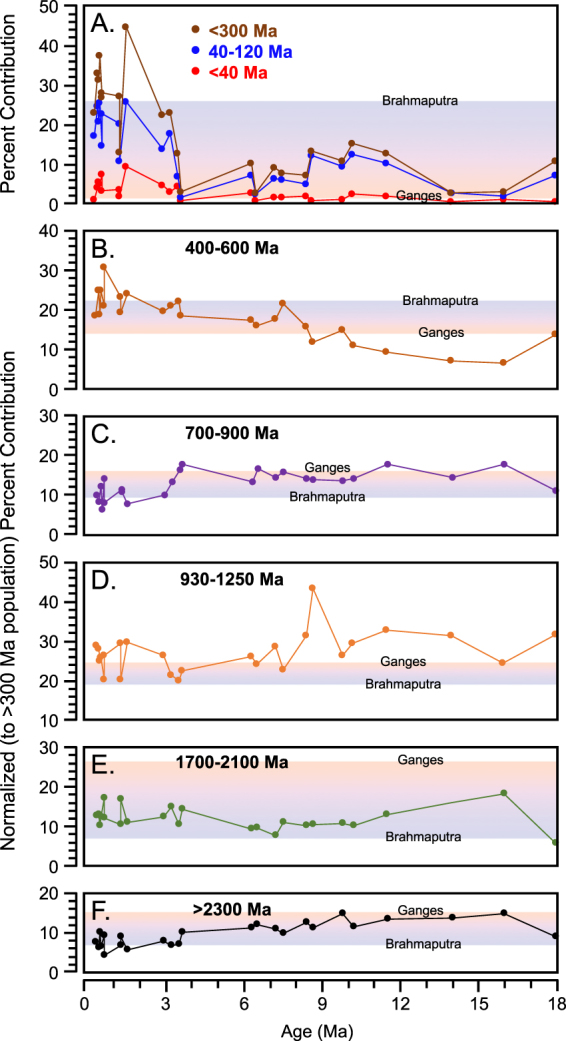
Changes in key DZ U-Pb populations through time. (**A**) The total proportion of mostly Lhasa grains that are <300 Ma within each sample, with additional more specific plots of the Gangdese ca. 40–120 Ma population and the ca. <40 Ma population that can represent Lhasa or Tertiary luecogranites of the THS and GHS. (**B**–**F**) Changes through time in key >300 Ma populations, where proportions for each sample represent values that have been normalized to represent the >300 population only, which is both abundant and highly variable. In all figures, the red-blue gradient represents percent contribution of this population within the modern Ganges (red end-member) and modern Brahmaputra Rivers (blue end-member).

Bengal Fan samples display changes through time in the proportional representation of different populations, which are shown in Fig. 4 relative to modern river values. Most prominent is the two-fold increase in the <300 Ma population during the late Pliocene to early Pleistocene: this population comprises only 4–15% of the total in Miocene through middle Pliocene samples, but ranges from 15–45% in samples of Late Pliocene and Pleistocene age. The ca. 400–600 Ma population also increases through time: Miocene samples have lower proportions of this population than either modern river, whereas Late Pliocene and Pleistocene samples have values that are similar to, or greater than, either modern river. Increases in the <300 and ca. 400–600 Ma populations through time are balanced by decreases in the ca. 700–900 Ma and 900–1250 Ma populations, which could be derived from the GHS, the THS or Lhasa, and the >2300 Ma population, derived from the LHS and possibly peninsular India. Miocene and Pliocene Bengal Fan samples have proportions of the ca. 700–900 Ma population that are similar to the modern Ganges, whereas Pleistocene samples are generally consistent with or outside the range of values found in the modern Ganges and Brahmaputra Rivers. For the ca. 900–1250 Ma population, the most significant observation is that four Pleistocene samples fall within the range defined by the modern Ganges and Brahmaputra Rivers, whereas seven samples have higher proportions than either modern river sample. In aggregate, then, individual DZ U-Pb peaks in modern river samples are well represented in the Bengal Fan, but the proportions of key populations in Bengal Fan samples are different: for Pleistocene samples, proportions of the <300 Ma, 400–600 Ma, 700–900 Ma, 900–1250 Ma, and >2300 Ma populations mostly lie outside the domain of modern river samples.

**Figure 5:**
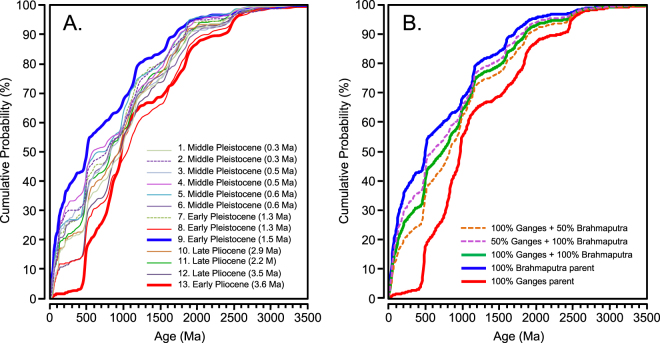
(**A**) Cumulative probability plots for Bengal Fan DZ samples of Plio-Pleistocene age, highlighting the end-member paleo-Ganges (sample 13) and paleo-Brahmaputra (sample 9) provenance signals. (**B**) A simple mixing model that illustrates the Ganges and Brahmaputra provenance end-members compared with mixes of the two signals in various proportions. Mixes are normalized to the modern-day load differential between the two rivers, such that a population that reflects 100% of the Ganges mixed with 100% of the Brahmaputra is comprised of 30% Ganges and 70% Brahmaputra.

There are also significant non-directional (between sample) population changes through time, especially after the Late Pliocene to Early Pleistocene increase in the <300 Ma population. From KDEs, cumulative probability plots, and multi-dimensional scaling analyses (Figs 3 and 5A, DR8), Pliocene sample 13 (ca. 3.6 Ma) and early Pleistocene sample 9 (ca. 1.5 Ma) represent end-member populations within the Late Pliocene and younger Bengal Fan sample set. Sample 13 has a very small <300 Ma population (<2% of the total), which indicates little to no direct contributions from Lhasa, as well as peaks within Proterozoic and Archean populations that are common in our modern Ganges River sample, but uncommon in the modern Brahmaputra, and is therefore interpreted to represent a paleo-Ganges provenance. By contrast, sample 9 contains the highest proportion of the <300 Ma population (~45%), whereas only ~11% is Paleoproterozoic or Archean in age, which is interpreted to represent a paleo-Brahmaputra provenance.

As noted above, there are significant differences between proportions in modern river samples vs. even the youngest Bengal Fan samples of Middle Pleistocene age, suggesting there are differences between the modern sediment-routing system and that which produced Bengal Fan turbidites. We therefore use samples 13 and 9 as Bengal Fan end members and have constructed simple mixing models to assess possible changes in contributions from the paleo-Ganges and paleo-Brahmaputra through the Late Pliocene and Pleistocene **(**Fig. 5B**)**. The first model simulates a completely mixed daughter population, whereas the second and third models simulate daughter populations that mix 100% of the paleo-Ganges load with 50% of the paleo-Brahmaputra, and conversely, 50% of the paleo-Ganges load with 100% of the paleo-Brahmaputra. Apart from samples 13 and 9, about one-third of Plio-Pleistocene Bengal Fan samples resemble a completely mixed GB system, whereas one-third resemble the simulation with 100% of the Ganges and 50% or less of the Brahmaputra, and one-third resemble the simulation with 100% of the Brahmaputra and 50% of the Ganges.

## Discussion

DZ U-Pb data from Bengal Fan turbidites provide a record of Miocene to middle Pleistocene source-to-sink signal transfer in Earth’s largest sediment-dispersal system. IODP 354 drill sites were located up to ~3500 kilometers from source areas in the Himalaya, and ~1400 km from the modern shelf margin, within a mid-fan setting. However, due to northward migration of the Indian plate, distances between the shelf margin and IODP 354 locations were almost 1000 km more during the Early Miocene: what was a very distal fan setting at the beginning of our record evolved to a mid-fan setting by the Plio-Pleistocene. Miocene increases in exhumation coupled with decreases over time in the length scales of sediment transport due to ongoing convergence^27,31^ likely explains increases over time in sand content within IODP 354 cores^2^. Nevertheless, even with transport distances that blend up to 2500 km of river flow with ~1400–2400 km of turbidity currents, the DZ U-Pb record faithfully records Himalayan source terrains. This large-scale signal transfer reflects the high relief and high exhumation rates of the Himalaya, an active sediment transport system driven by monsoon and snowmelt-generated discharge regimes, and the propensity of large river systems like the GB to homogenize sand transport over long distances^50^. We note the modern Mississippi River DZ U-Pb record also integrates the signals of its drainage basin^51^, and the Mississippi fan in the deep Gulf of Mexico also faithfully records the signature of the Mississippi drainage basin^52^.

The timing and style of Brahmaputra integration with the Asian plate is important to SE Asian drainage evolution as well as models that argue for or against strong coupling between tectonics and surface processes [e.g.^32,46–49^]. Our data includes significant Gangdese arc and, more broadly, <300 Ma populations in Early Miocene (ca. 18 Ma) Bengal Fan samples, which shows that Tibetan drainage was at least partially integrated with the Brahmaputra by that time:^32,33^ the <300 Ma populations from Tibet are prominent in Middle Miocene DZ U-Pb data from the Nicobar Fan as well^28^. Equally important, the population from Lhasa doubles in Bengal Fan samples beginning in the late Pliocene, which we speculate may in part reflect increasing integration of the Lhasa terrane into the Brahmaputra drainage. Our data also shows an increase in the ca. 400–600 Ma population through the Plio-Pleistocene, which is consistent with increased sediment flux from Lhasa, as well as from the eastern syntaxis, which was rapidly exhuming at this time^32,49,53–55^.

Bengal Fan samples produced no U-Pb ages that approximate depositional ages from biostratigraphic data, and therefore U-Pb data do not improve geochronological resolution on fan deposition. However, the presence of small numbers of grains with U-Pb ages of ca. 10–4 Ma, which are derived from the anomalously young (<10 Ma) and rapidly exhuming metamorphic core of the eastern syntaxis^38^, provides insights into rates of signal propagation. Young syntaxial grains with U-Pb ages of 6.2 ± 0.1 and 5.0 ± 0.1 Ma appear in Bengal Fan Late Pliocene sample 10, deposited ca. 2.9 Ma, and grains with U-Pb ages of 9.8 ± 0.3 Ma to 4.8 ± 0.2 Ma appear in Early Pleistocene sample 9, deposited ca. 1.3 Ma. The low abundance of this young population makes it difficult to assess the significance of presence or absence in older samples, and fully address alternative models for syntaxis uplift and incision. However, the presence of these young grains indicates crystallization, kilometers of exhumation, and transport ~3000 km to IODP drill sites in the deep-sea fan within a ~2–3 Myr time frame.

Bengal Fan DZ U-Pb data are likely insufficient to serve as a direct proxy for Neogene and Quaternary climate changes and their effects on erosion in the Himalaya, which are best documented by other means. However, large-scale sand transport to the middle Bengal Fan is most likely dependent on climate-forced sea-level change, and corresponding extension of river mouths to the shelf margin^56^. With the exception of the Congo Fan, which is fed by a canyon that penetrates across the shelf and into the river mouth, large rivers with broad shelves like the Amazon and Mississippi are not contributing significant volumes of sand to the deep sea during Holocene interglacial conditions of high sea level^57,58^. Instead, they discharge to inner-shelf shorelines, and the sand fraction is sequestered on the inner shelf. Moreover, river mouths or a sandy shoreline must be within 2–5 km of the canyon head to deliver high concentrations of sand, whereas mud can be delivered if the river mouth, shoreline, or subaqueous clinoform is within 20–50 km^59^. The GB system is in this category as well: Holocene mud transport through the SoNG is well documented **[**e.g.^60,61^**]**, but there has been little if any concentrated sand delivery to the basin floor during this same period. Moreover, the latest Pleistocene to Holocene channel-levee of the Bengal Fan, from IODP 354 Site 1454, lacks distinct sandy turbidite beds^2^. We therefore suggest the sand-rich part of the Late Pliocene to Pleistocene Bengal Fan record is biased towards glacial-periods with low sea level, when rivers were extended across emergent shelves and discharged directly to shelf-margin canyon heads. Hence, whereas strong tectonic and climatic forcing associated with the Himalaya and the GB system drives sediment transfer to the continental margin and the land-sea boundary, large-scale transfer of sand to the deep sea also reflects responses of the Ganges and Brahmaputra Rivers to climate-forced sea-level fall.

Other aspects of the Bengal Fan DZ U-Pb record may reflect climatic controls and climate change. For example, the limited THS signal throughout the Bengal Fan DZ U-Pb record is consistent with views that erosion in that part of the orogen is at least partly precipitation limited^15,16^, and the majority of sediment over time is derived from Lhasa and the GHS^30^. In this context, differences in the proportions of key DZ U-Pb populations between modern river samples and the Bengal Fan may be informative. Pleistocene Bengal Fan DZ U-Pb data include (a) higher proportions of the <300 Ma population, which reflects source areas in Lhasa, (b) higher proportions of the ca. 400–600 Ma population, which can be derived from Lhasa and the GHS, but (c) lower proportions from the LHS and/or peninsular India. We consider Pleistocene Bengal Fan samples to be biased to record turbidite deposition during glacial periods with low sea level. We therefore suggest the differences between modern river samples and the Pleistocene Bengal Fan may reflect differences in the loci of sediment production in the modern interglacial climate with strong monsoon rains, vs. a glacial climate where monsoon strength may be reduced and erosion is more strongly tied to higher-elevation cold-climate and glacial processes.

**Figure 6:**
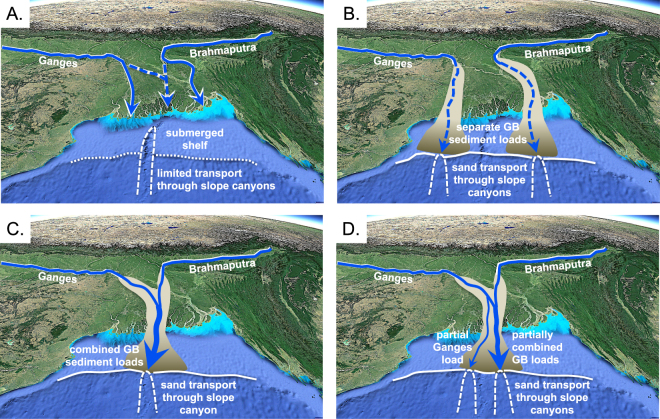
Multi-scenario model for Late Pliocene through Pleistocene sediment dispersal, showing the modern context with sea-level highstand and an inner-shelf delta vs. time periods when climate-forced sea-level fall results in coastal-plain and cross-shelf incised valleys linked to shelf-margin shoreline positions. Different routing scenarios through the terrestrial and shallow marine Bengal basin are as follows. (**A**) The interglacial Ganges and Brahmaputra discharge separately, or together as they do today, to shorelines located in inner shelf positions. Although some mud is transferred to the basin floor through the slope canyon, sand is trapped near the inner shelf shoreline. (**B**) The glacial-period Ganges and Brahmaputra occupy separate coastal-plain and cross-shelf incised valleys, discharge mud and sand to different parts of the shelf margin, and feed separate slope canyons and sand-rich channel-levee systems on the Bengal Fan to produce parent DZ U-Pb populations. (**B**) The Ganges and Brahmaputra join and occupy the same incised valley, and discharge to a common slope canyon to feed a single sand-rich channel-levee system to produce a 50–50 mix in DZ U-Pb populations. (**D**) The Ganges and Brahmaputra occupy the same incised valley, but partial avulsion of the Ganges results in partial discharge of Ganges load to one canyon, and partial discharge of the Ganges plus the complete load of the Brahmaputra to a different canyon. This same scenario could apply to partial avulsion of the Brahmaputra as well. Background image from Google Earth (Map data: SIO, NOAA, U.S. Navy, NGA, GEBCO; Image; Landsat/Copernicus).

Last, we argue that the Plio-Pleistocene Ganges and Brahmaputra delivered sand to IODP 354 sites separately to produce parent samples 13 and 9, but were merged or partially merged when they delivered the mixed populations that comprise most Bengal Fan samples. DZ U-Pb data from the Nicobar Fan show periods of more Ganges-like vs. mixed vs. more Brahmaputra-like populations as well^28^. We recognize there are multiple mechanisms by which populations can be mixed during transport, for example, climate change impacts on sediment composition, tidal and longshore processes, and recycling of older deposits. However, in view of the pervasive nature of mixing, we argue that autogenic delta-plain avulsions represent the most straightforward way to explain the mixing of DZ U-Pb populations or the lack thereof. Avulsions are commonly initiated within a river’s hydraulic backwater reach, which extends hundreds of kilometers upstream from the river mouth in large, deep, low-gradient rivers like the Ganges and Brahmaputra. Hence, even a modest avulsion angle of 15–25° can result in 100s of km of lateral displacement in river-mouth sediment inputs to the active land-sea boundary^56,62^. Moreover, avulsion is an inherent, high-frequency (millennial-scale) process^24^ that can distribute sediment inputs across a broad delta plain like the GB over a short period of time. During periods of high sea level like the Holocene, GB avulsions take place on an inner-shelf delta, and sandy sediments are sequestered on the inner shelf **(**Fig. 6A). With sea-level fall, rivers incise through the highstand deltaic clinothem and extend to the shelf margin, with backwater reaches that shift basinward as well. Avulsion on the shelf-margin delta then plays a fundamental role in the location of sediment discharge to a canyon system and the fan. We therefore infer that most of our DZ U-Pb samples represent times of low sea level when the Ganges and Brahmaputra sometimes discharged separately to the margin and delivered sediments to different canyons **(**Fig. 6B**)**, and at other times merged on the shelf-margin delta plain, and discharged to the same canyon **(**Fig. 6C,D).

It is easy to see how a completely mixed DZ U-Pb population represents an idealized case where both rivers approach peak sediment discharge at approximately the same time, they merge within a common incised valley that transfers sediment through a former highstand delta plain, and they extend to the shelf margin where they feed a single slope canyon and basin-floor channel-levee system **(**Fig. 6c). Cases where 100% of the model Ganges signal is mixed with only part of the model Brahmaputra, or vice versa, are less straightforward, but modern rivers provide two simple process-based alternative models worthy of consideration. One would be a completely merged GB as above, but turbidites recorded by our samples were ignited when one river was at peak sediment discharge and the other was not. Peak discharge for the modern Ganges is almost a month earlier than the Brahmaputra due to west-to-east migration of monsoon rains^12^, and it is not uncommon for large rivers like the Ganges and Brahmaputra to have multiple peaks each year due to out-of-phase tributary inputs. An alternative model assumes that avulsions are not instantaneous but take centuries to complete: the sediment load of either the Ganges or Brahmaputra can be split over that time period if one river has avulsed to join the other, or avulsed away from a merged system to deliver sediments to a different part of the shelf margin, but left behind a distributary that still transports part of the sediment load **(**Fig. 6d). As noted above, this type of split in sediment load has been the case for the Ganges River in historic times^22^.

## Summary

The detrital zircon U-Pb record of the Miocene through Pleistocene Bengal Fan represents an important archive of Himalayan tectonics and climate, climate-forced sea-level change, and autogenic surface dynamics. The general DZ population reflects strong tectonic and climatic forcing, and long-term drainage integration, which drives sediment transfer from Himalayan source terrains to the continental margin and land-sea boundary. Moreover, late Miocene- through Pleistocene-age zircons from the eastern Himalayan syntaxis record unique aspects of Himalayan tectonics and topographic evolution, and show that signal transfer is not only faithful to Himalayan source terrains and surface processes, but geologically rapid. Within this broader context, the large-scale transfer of turbidite sand from the continental margin to distal basin-floor fan settings is inherently episodic because it requires climate-forced sea-level fall associated with global ice volume increases, so that the Ganges and Brahmaputra Rivers extend their courses to the shelf-margin and connect with canyon-feeder systems. Within the boundary conditions and process framework established by tectonic and climatic systems, and climate-forced sea-level change, the record of sediment mixing or the lack thereof represents the fingerprints of autogenic avulsions on signal transfer from source-to-sink.

## Methods

For IODP 354, the research vessel JOIDES Resolution drilled a 7-site transect at 8°N across the middle Bengal submarine fan^2^. A total of 1.7 kilometers of core was obtained from the 7-site transect, resulting in recovery of abundant turbidite sand and silt ranging in stratigraphic age from ~18 Ma to the present **(**Figs 1, S1,S2, Tables S1 and S2**)**.

We collected 25 unconsolidated samples of Bengal Fan turbidite sand and silt from IODP 354 cores, with samples ranging in age from Early Miocene to mid Pleistocene **(**Tables 1, S1 and S2**)**, and two samples from the modern Ganges and Brahmaputra Rivers some 100 km upstream from their confluence. Our IODP 354 turbidite sands mostly sampled individual turbidite units, which were evident from core images and shipboard physical properties that show fining-upward grain-size trends and sedimentary structures on the scale that we sampled or larger. Other samples were collected from thick, structureless sands that were demonstrably continuous between sections of a particular coring interval, but had flowed or no longer retained their original physical stratification. We assign approximate ages to each sample based on existing biostratigraphic constraints (see Tables S1 and S2**)**, and extrapolation between those constraints, although our interpretations do not rely on specific age estimates.

Approximately 1–2 kilograms of sediment from each sample underwent heavy mineral separation, and individual detrital zircon grains were then selected for mounting and imaging. U-Th-Pb laser ablation geochronology was conducted at the Arizona LaserChron Center (more details of the analytical methods are provided in the data repository), using the high-resolution sector ICP-MS at the University of Arizona (Element 2, Thermo Fisher) and a fixed 20 micron laser ablation spot size^63^. We use backscatter-electron (BSE) images to identify grain interiors, so as to avoid younger overgrowths, and where possible we place spots on grain cores to ensure targeting of the original protolith ages. We targeted ~300 individual zircon grains (n = 300) for U-Pb analyses of grain cores^64,65^: U-Pb ages with >10% discordance were discarded (~10% of all zircons analyzed), resulting in a total of 6602 concordant U-Pb ages from the Bengal Fan and 535 concordant ages from the modern Ganges and Brahmaputra Rivers, an average of ~270 numerical ages per sample. For each analysis, we use ^206^Pb/^238^U ages for grains <1 Ga, and ^206^Pb/^207^Pb ages for grains >1 Ga. Analytical details are provided in the Supplemental Data and can be downloaded from the community database http://geochron.org.

We use plots of normalized kernel-density estimates (KDE) and cumulative probability to visualize DZ U-Pb age populations **(**Figs 2B,C, 3 and 5A), whereas we use multi-dimensional scaling (MDS) to identify statistical relationships between samples, or the lack thereof (Fig. S8**)**. Normalized KDEs and MDS plots of zircon U-Pb age spectra were produced using the methodology and scripts developed and described by Vermeesch^66,67^ and available at http://www.ucl.ac.uk/~ucfbpve/provenance/.

We also deploy a simple mixing model to test whether samples represent a mix of Ganges and Brahmaputra sediment. Mixing models can take several forms. One seeks to predict and/or explain the distribution of ages within the population by simulating contributions of different parts of a drainage basin, including geologic units and their areal extent, as well as concentration of zircons in river sands^68–70^. The form we deploy seeks to explain populations that appear to be daughter products derived from 2 or more parent populations^41^. Because modern river DZ U-Pb populations are different from those of the Bengal Fan, as discussed above, we use samples 13 and 9 as the end-member Plio-Pleistocene Ganges and Brahmaputra parent signals, respectively.

Our mixing models utilize normalized KDEs calculated from samples 9 and 13 and exported using DensityPlotter 7.3^66^. Parent populations are simply the KDE plots of samples 9 and 13, whereas daughter populations take the form in equation 1:

1 $${Z}_{i}=a{X}_{i}+b{Y}_{i}$$

where Z_*i*_ = the daughter KDE for age *i*, X_*i*_ = the Ganges parent KDE for age *i*, Y_*i*_ = the Brahmaputra KDE for age *i*, and a and b represent proportional contributions of the Ganges and Brahmaputra, respectively, to the total load. We then plot cumulative probability for Late Pliocene and Pleistocene Bengal Fan samples in Fig. 5A, and parent and daughter mixes in Fig. 5B.

Mixed populations likely reflect autogenic processes operating in the delta region, in particular avulsion, which can result in merging or separating the Ganges and Brahmaputra, such that they discharge one combined population or two separate populations to the coastal ocean. The modern sediment load of the combined GB system is 30% derived from the Ganges, and 70% derived from the Brahmaputra^12^. Hence, for a 100% merge of the two rivers sediment load, the mixing model takes the form of Z_*i*_ = 0.3X_*i*_ + 0.7Y_*i*_. Several important caveats bear on interpretation of mixed populations in the Bengal Fan. First, part of the modern Ganges load may be diverted through distributaries, and not reach the Brahmaputra confluence, such that the load differential we use is a transient phenomenon. By one estimate^21^, the Ganges load that is diverted to distributaries is roughly equal to the current load of the lower Ganges just prior to joining the Brahmaputra, meaning that if the entire Ganges load merged with the entire Brahmaputra load, contributions would approach 50% from each system, or Z_*i*_ = 0.5X_*i*_ + 0.5Y_*i*_. Second, the two rivers peak at slightly different times due to inherent differences in hydrology^12^ (Fig. DR9), including differences in snowpack and time of arrival of the monsoon. It is therefore likely that avulsion produces the mixed signal, but composition of the mixed population may record turbidites that were ignited during a time when one river was reaching its seasonal peak sediment discharge, and the other had yet to reach its peak, or its peak had passed, and its contribution to the total load was diminished.

We model the following scenarios:

A. Base case, 100% of the modern Ganges and Brahmaputra loads, Z_*i*_ = 0.3X_*i*_ + 0.7Y_*i*_.
B. 50% Ganges load plus 100% Brahmaputra, Z_*i*_ = 0.15X_*i*_ + 0.7Y_*i*_.
C. 100% Ganges load plus 50% Brahmaputra, Z_*i*_ = 0.3X_*i*_ + 0.35Y_*i*_.

Scenario A represents the current load differential, whereas Scenario B can represent 50% Ganges load and 100% Brahmaputra, as indicated, but is mathematically similar to a case where the Ganges has not yet reached peak sediment discharge but the Brahmaputra has. Scenario C can represent 100% of the Ganges load and 50% of the Brahmaputra, as indicated, but is mathematically similar to a case where the Brahmaputra has already peaked, and is in recession in terms of its sediment load by the time the Ganges reaches peak sediment discharge, or a case where the Ganges load is actually significantly larger than modern data suggests, perhaps due to a single channel with no distributaries.

## Electronic supplementary material

Supplementary Information
