# Author Correction: Allogenic and Autogenic Signals in the Stratigraphic Record of the Deep-Sea Bengal Fan

**DOI:** 10.1038/s41598-018-30160-y

**Published:** 2018-08-10

**Authors:** Mike Blum, Kimberly Rogers, James Gleason, Yani Najman, Jarrett Cruz, Lyndsey Fox

**Affiliations:** 1 Department of Geology, University of Kansas, Lawrence, Kansas USA; 2 Institute for Arctic and Alpine Research, University of Colorado, Boulder, Colorado USA; 3 Department of Earth and Environmental Sciences, University of Michigan, Ann Arbor, Michigan USA; 4 Lancaster Environment Centre, Lancaster University, Lancaster, UK; 5 Department of Earth, Ocean, and Atmospheric Science, Florida State University, Tallahassee, Florida USA; 6 Department of Earth Sciences, The Natural History Museum, London, UK

Correction to: *Scientific Reports* 10.1038/s41598-018-25819-5, published online 22 May 2018

In the Supplementary Information file originally published with this Article, a dataset containing additional sample results was omitted. This error has now been corrected in the Supplementary Information that now accompanies the Article.

