# Supplementary material for: Allogenic and Autogenic Signals in the Stratigraphic Record of the Deep-Sea Bengal Fan
Source: Sci Rep. 2018 May 22;8:7973. doi: 10.1038/s41598-018-25819-5 (PMC5964172; doi:10.1038/s41598-018-25819-5)
Supplement: Supplementary file 1 — Supplementary Information [file 41598_2018_25819_MOESM1_ESM.pdf]

Allogenic and Autogenic Signals in the Stratigraphic Record of the Deep-Sea Bengal Fan

*[1]Mike Blum, [2]Kimberly Rogers, [3]James Gleason, [4]Yani Najman, [5]Jarrett Cruz, [6]Lyndsey Fox,

[1] Department of Geology, University of Kansas. Lawrence, Kansas USA. mblum@ku.edu
[2] Institute for Arctic and Alpine Research, University of Colorado. Boulder, Colorado USA. kgrogers@colorado.edu
[3] Department of Earth and Environmental Sciences, University of Michigan. Ann Arbor, Michigan USA. jdgleaso@umich.edu
[4] Lancaster Environment Centre, Lancaster University. Lancaster, UK. y.najman@lancaster.ac.uk
[5] Department of Earth, Ocean, and Atmospheric Science, Florida State University. Tallahassee, Florida USA. jwc09e@my.fsu.edu
[6] Department of Earth Sciences, The Natural History Museum. London, UK. lyndsey.fox@nhm.ac.uk

*corresponding author

GENERAL CONTEXT

Figures S1-S3 provide stratigraphic context and locations for IODP 354 drill sites, and sample locations within cores from the drill sites.

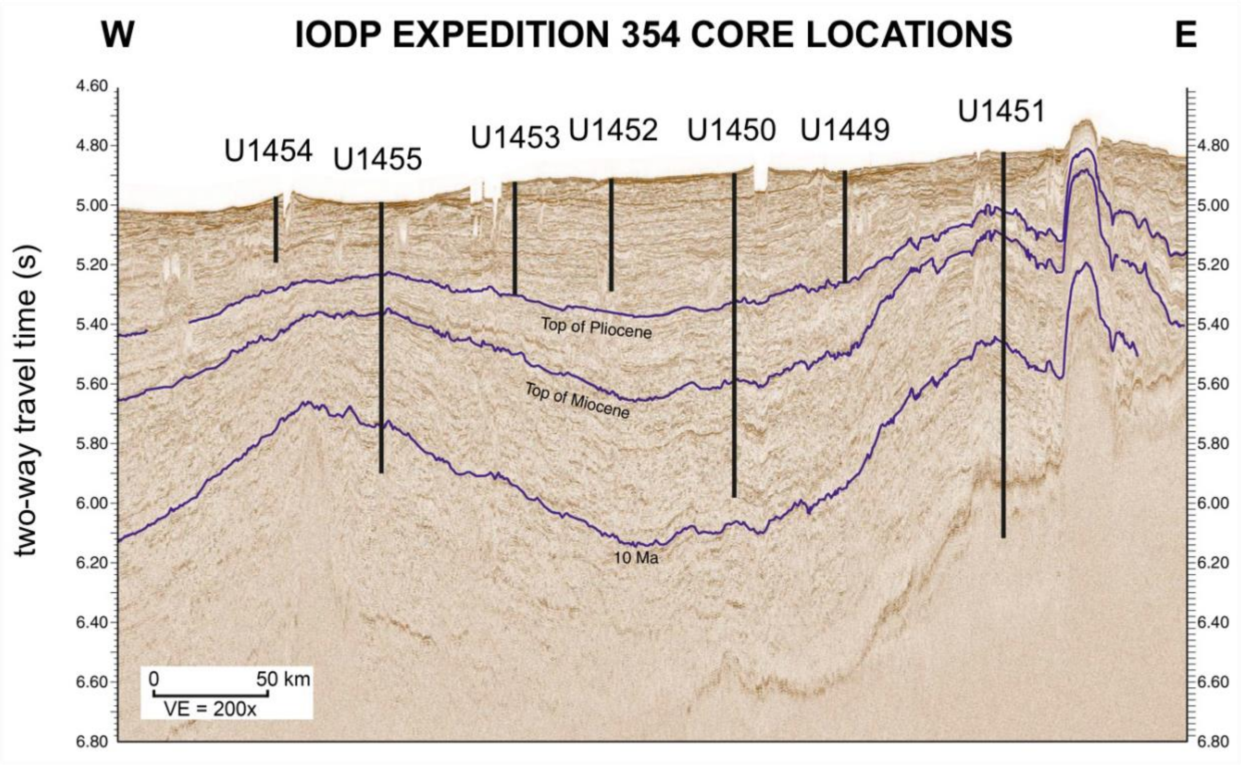

Figure S1. Seismic profile showing the positions of IODP Expedition 354 drill sites in relation to regional fan architecture. From France-Lanord et al. (2016). For the location of this transect, see text Fig. 1 and Fig. S2 below.

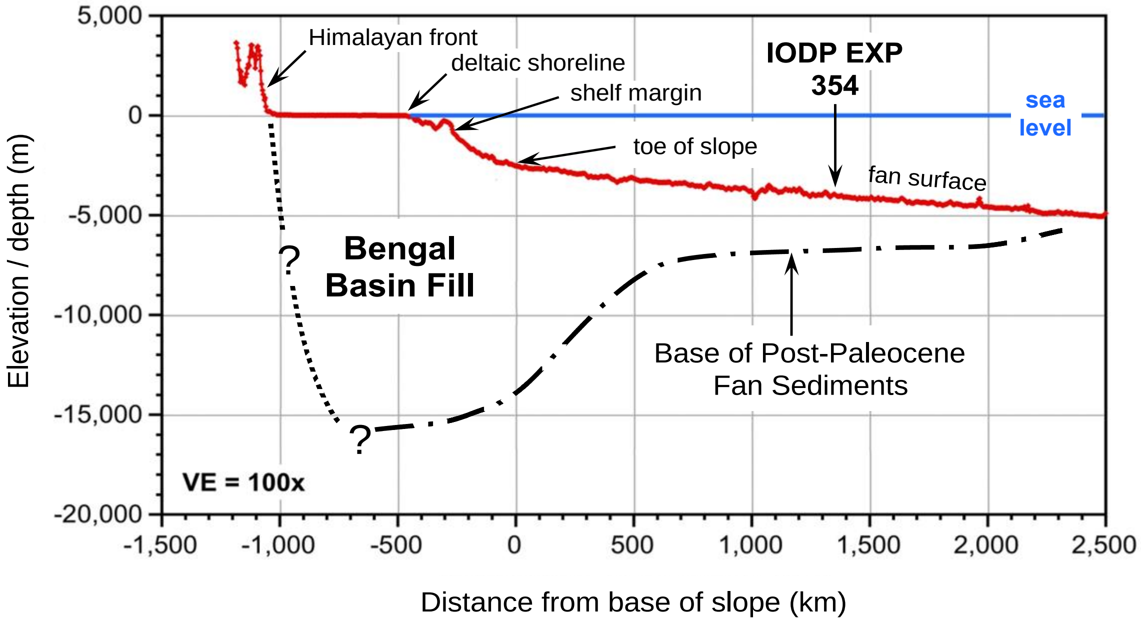

Figure S2. N-S profile through the Himalayan front, the Bengal basin, and the Bay of Bengal, illustrating topography, bathymetry, and sediment thickness, as well as the latitudinal position of IODP Expedition 354 cores. Topography and bathymetry are derived from GeomappApp, available at http://www.geomapapp.org. Base of fan sediments is derived from Curray et al. (2003) and Schwenk and Spieß (2009), with each of those sources drawing on earlier work.

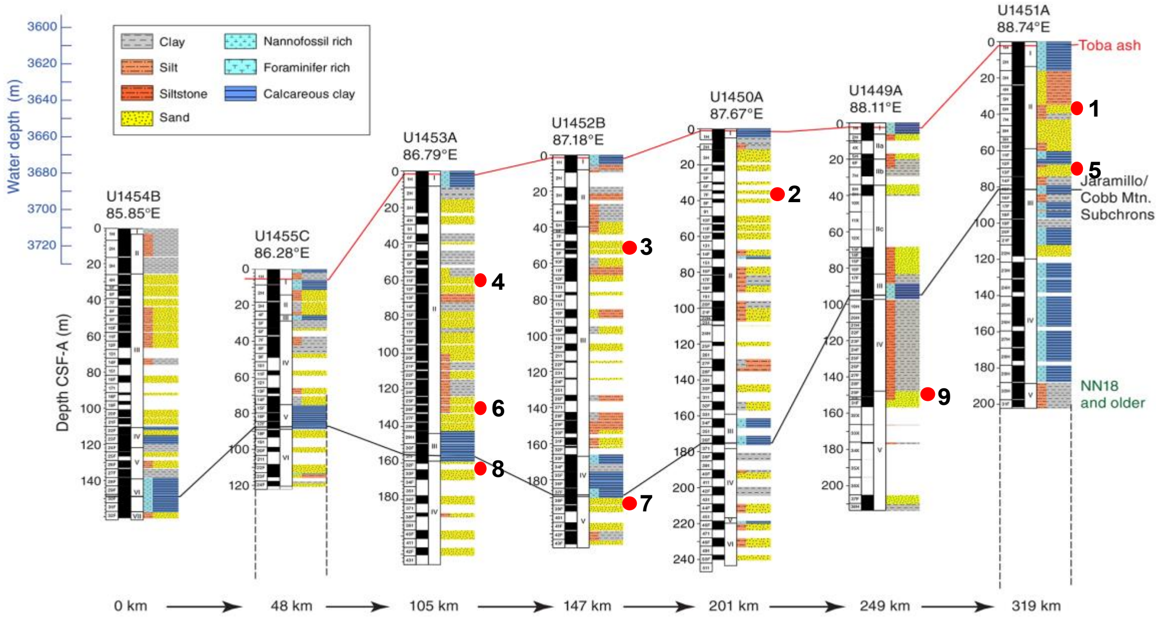

Figure S3. (a) Core descriptions for IODP 354 drill sites, showing positions of Pleistocene-age DZ samples. (b – next page) Core descriptions for IODP 354 drill sites U1450 and 1451, showing positions of DZ samples for the older part of the record. See Figure S1 for locations of drill sites. After France-Lanord et al. (2016).

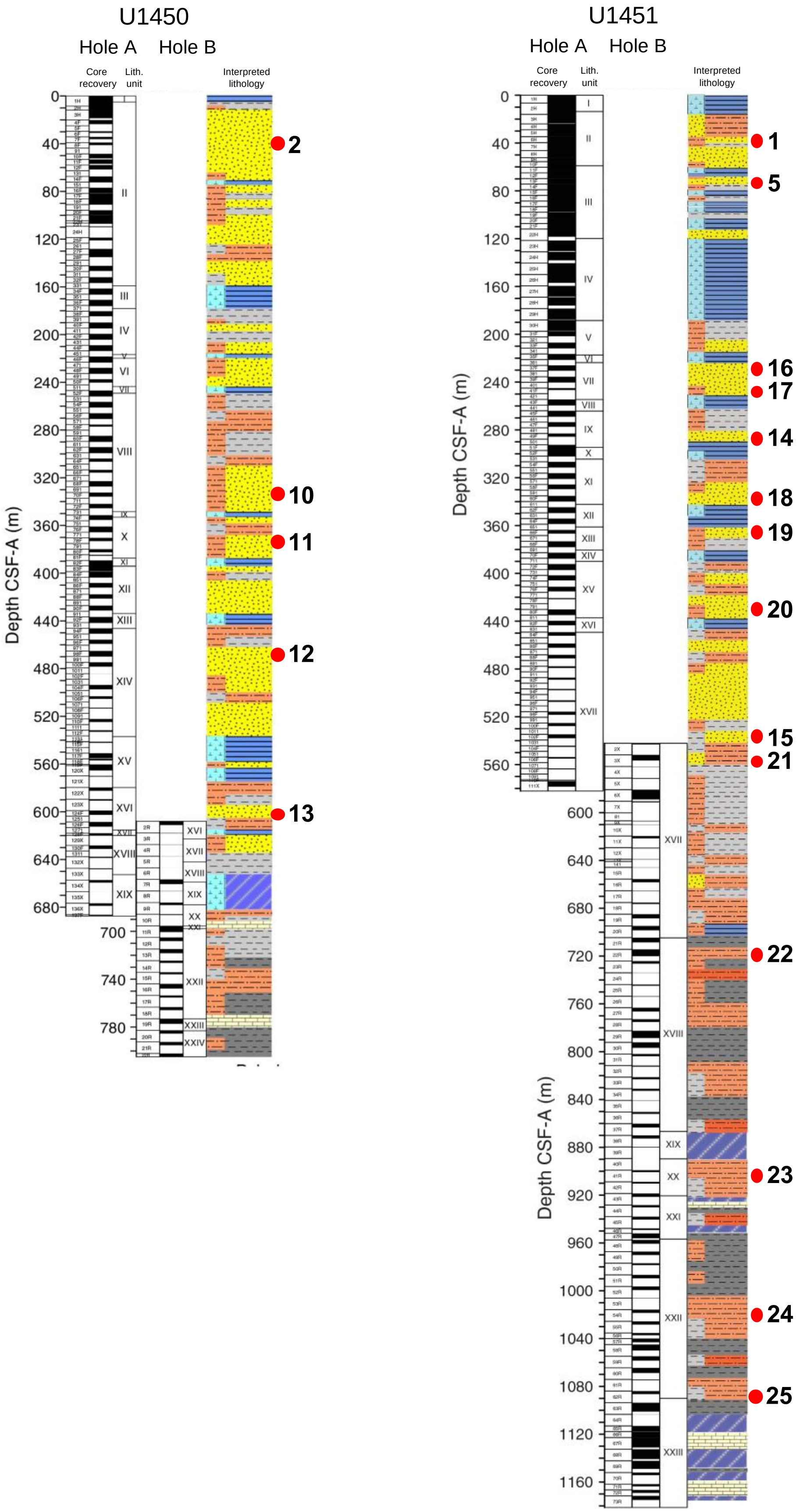
U1450
Hole A
Hole B
Core recovery
Lith. unit
Interpreted lithology
Depth CSF-A (m)
U1451
Hole A
Hole B
Core recovery
Lith. unit
Interpreted lithology
Depth CSF-A (m)
2
10
11
12
13
1
5
16
17
14
18
19
20
15
21
22
23
24
25

REVISED BIOSTRATIGRAPHIC CONSTRAINTS

Initial age constraints on core samples from IODP 354 were based on shipboard analyses of biostratigraphic data, as well as paleomagnetic reversals, and the presence of volcanic ashes (see France-Lanord et al., 2016). Shipboard core descriptions with positions of DZ samples, for U1449, U1452, and U1453 are shown in Figures S3, S6, and S7. Subsequent analyses of biostratigraphic constraints have been completed on U1450 and U1451, as discussed below, and summarized in Table S1 and S2.

For U1450, low-resolution biostratigraphic analyses were conducted shipboard using core catchers, which has been supplemented post-cruise by high resolution sampling of the pelagic layers (20cm intervals). Each sample was first repeatedly split with a microsplitter until a volume of sediment, equivalent to that used for a population analysis (~300 tests), was obtained and evenly spread onto a picking tray and examined under a light microscope (Nikon SMZ25). This smaller but representative sample volume allows for a more accurate visual assessment of species relative abundances and preservation. Using insights gained through SEM studies, we have revised the shipboard range charts for extinct taxa, updated the biostratigraphy to include observations from U1450A and B, and followed the planktonic foraminiferal zonal scheme presented in Wade and others (2011).

For U1451, samples were selected from cores based on visual lithological features, which differs from shipboard sampling where sediment from core catchers were used to provide a general idea of sample ages. Post cruise nannofossil biostratigraphic zonation was completed using a compound light microscope to examine smear slides. Specimens were identified in a semi-quantitative manner from one traverse (100 fields of view) and assigned an abundance code for relative comparisons. Samples were analyzed in accordance with the standard nannofossil zonations and the geochronological framework in Gradstein et al. (2012).

Table S1. Revised biostratigraphic constraints on DZ sample ages from U1450 (see also Fig. S4). Analyses were performed by Lyndsey Fox, The Natural History Museum, London.

U1450 DZ Samples			U1450 Age Constraints					
Sample no.	Full Sample Name	Depth (mbsf)	Sample no.	Depth (mbsf)	Limit	PF Zone	Diagnostic Fossils	Age (Ma)
2	U1450A 6F-8F	30-44	---	0	Upper	---	---	0
			14F-4W, 32-37	72	highest	PT1b	*G. tosaensis*	0.63
10	U1450A 70F	332-338	62F-CC	299	lowest	PL6	*G. tosaensis***	2.6
			92F-CC	422	highest	PL6	*D. altispira*	3.47-3.59
11	U1450A 78F-80F	360-370	62F-CC	299	lowest	PL6	*G. tosaensis***	2.6
			92F-CC	422	highest	PL6	*D. altispira*	3.47-3.59
12	U1450B 98F	465-470	92F-CC	422	highest	PL6	*D. altispira*	3.47-3.59
			5R-CC	637	highest	PL5	*P. primalis*	3.65
13	U1450A 124F	600-605	92F-CC	422	highest	PL6	*D. altispira*	3.47-3.59
			5R-CC	637	highest	PL5	*P. primalis*	3.65

Table S2. Revised biostratigraphic constraints on DZ sample ages from U1451 (see also Fig. S5). Analyses were performed by Jarrett Cruz, Florida State University.

**U1451 DZ Samples**			**U1451 Age Constraints**					
**Sample no.**	**Full Sample Name**	**Depth (mbsf)**	**Sample no.**	**Depth (mbsf)**	**Limit**	**NN zone**	**Diagnostic Fossils**	**Age (Ma)**
1	U1451A 4F-6F	25-35	1H-3 66/67	25-29.6	Upper	NN21	Top *Emiliania huxleyi*	<0.29
			5H-1W 74/75	29.6	Within	NN21/NN20	*Gephyrocapsa spp* with no *Emiliania huxleyi*	0.29
5	U1451A 13F	70-75	11F-3W 108/109	64.38	Upper	NN20/NN19	Top *Pseudoemiliania lacunosa*	0.44
			20F-1 142/143	104	Lower	NN19	Top *Helicosphaera sellii*	1.26
16	U1451A 37F	225-230	30H-2-60/61	189	Upper	NN11	Top *Reticulofenestra rotaria*	6.1
			43F-1 80/81	255.3	Lower	NN11	X *Nicklithus amplificus* to *Triquetrorhabdulus rugosus*	6.79
17	U1451A 41F	245-250	30H-2-60/61	189	Upper	NN11	Top *Reticulofenestra rotaria*	6.1
			43F-1 80/81	255.3	Lower	NN11	X *Nicklithus amplificus* to *Triquetrorhabdulus rugosus*	6.79
14	U1451A 47F-49F	280	43F-4 45/46	259.1	Upper	NN11	Base of *Nickilithus amplificus*	6.91
			52F-3 78/79	301	Lower	NN11	Base *Amaurolithus primus/spp*	7.42
18	U1451A 60F	340-345	52F-3 78/79	301	Upper	NN11	Base *Amaurolithus primus/spp*	7.42
			62F-1 59/60	345.8	Lower	NN11	Top *Discoaster loeblichii*	7.53
19	U1451A 66F	365-370	62F-2 15/16	346.4	Upper	NN11/NN10	Base *Discoaster berggreniil* Top *Discoaster prepentaradiatus*	8.29
			70F-1 65/66	373.4	Lower	NN10	Top *Minylitha convalis*	8.68
20	U1451A 80F	425-430	70F-1 65/66	373.4	Upper	NN10	Top *Minylitha convalis*	8.68
			82F-1 86/87	440.6	Lower	NN10	Base of *Discoaster loeblichii*	8.77
15	U1451A 102F	535-540	86F-1 65/66	449.7	Upper	NN9	Top *Catinaster coalitus*	9.69
			6x-4 56/57	584.95	Lower	NN9/NN8	Base *Discoaster hamatus*	10.55
21	U1451B 3X	550-555	86F-1 65/66	449.7	Upper	NN9	Top *Catinaster coalitus*	9.69
			6x-4 56/57	584.95	Lower	NN9/NN8	Base *Discoaster hamatus*	10.55
22	U1451B 22R	715-725	22R-3-28/29	717.54	Upper	NN7	Top *Cyclicargolithus floridanus*	11.85
			29R-4 58/59	787.96	Lower	NN7/NN6	Base common *Discoaster kugleri*	11.9
23	U1451B 41R	900-905	41R-1 51/52	899.61	Upper	NN5	Top *Discoaster petilaformis*	14.1
			46R-1 108/109	948.88	Lower	NN5/NN4	Top *Helicosphaera ampliaperta*	14.91
24	U1451B 51R-54R	1015-1020	47R-3-2/3	954.23	Upper	NN4	abundant *Discoaster deflandrei* group	15.8
			63R-3 33/34	1096.93	Lower	NN4/NN3	Top *Sphenolithus belemnos*	17.95
25	U1451B 62R	1085-1090	47R-3-2/4	954.23	Upper	NN4	abundant *Discoaster deflandrei* group	15.8
			63R-3 33/35	1096.93	Lower	NN4/NN3	Top *Sphenolithus belemnos*	17.95

Additional Data

Figures S4 through S7 provided additional data on specific U-Pb populations within the Bengal Fan data set, and within parts of the Himalayan and Tibetan source terrain. Figure S8 illustrates the results of multi-dimensional scaling, which defines relationships or the lack thereof between Plio-Pleistocene samples of the Bengal Fan, and Figure S9 illustrates seasonal offsets of flood peaks for the Ganges vs. Brahmaputra, due to the earlier arrival of snowmelt discharge from the Brahmaputra drainage, relative to monsoon rains.

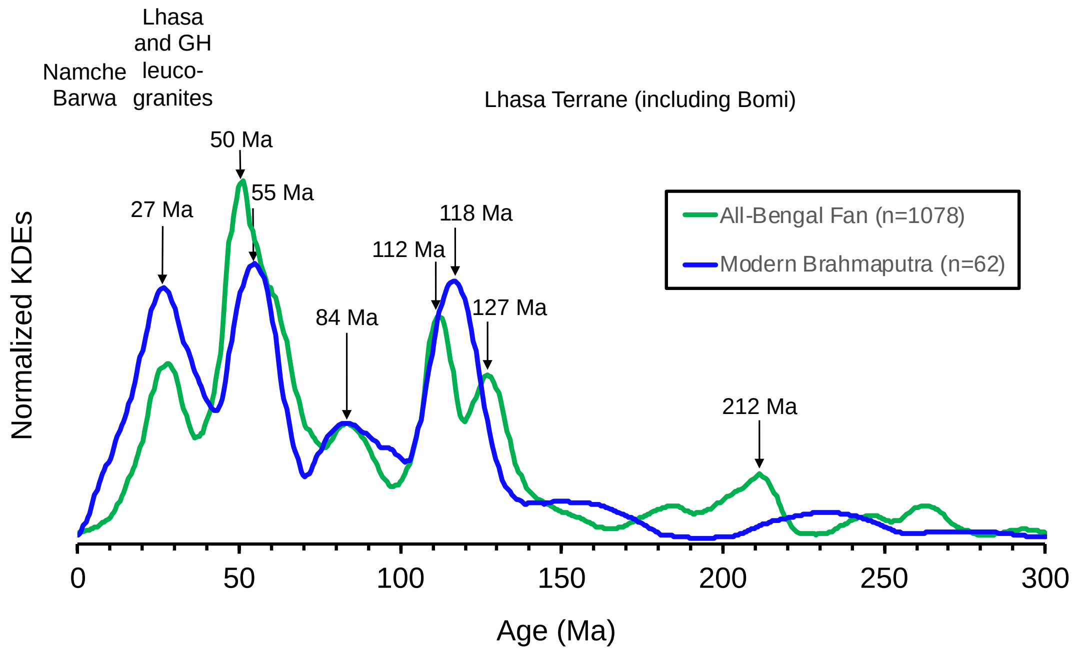

Figure S4. Normalized KDE plot of all Bengal Fan U-Pb ages <300 Ma, as well as the <300 population from the modern Brahmaputra River. Note that while Neogene-age zircons can be derived from the Greater Himalayan leucogranites, they represent a very small part of the Greater Himalaya, and Bengal Fan samples are likely dominated by grains from Lhasa.

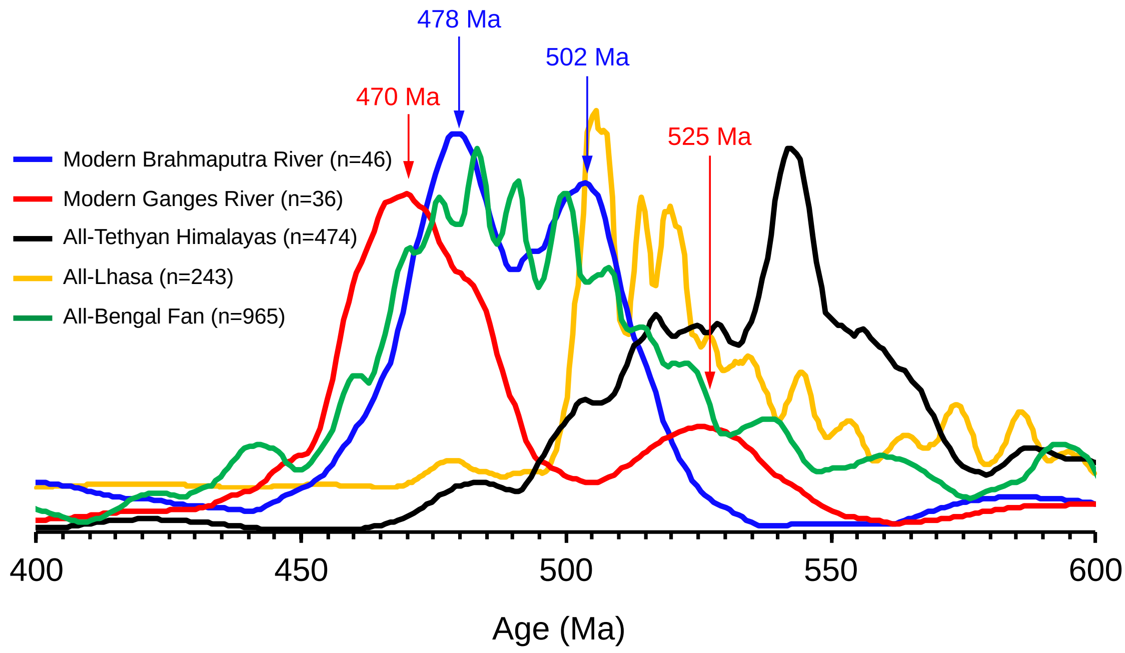

Figure S5. Normalized KDE plots for the 400-600 Ma DZ U-Pb populations from the Tethyan Himalaya and Lhasa Terrane (from Gehrels et al., 2011), for all Bengal Fan data, and for the modern Ganges and Brahmaputra Rivers. The orange shaded block represents analyses from granites of the MCT Zone in Nepal (Cawood et al., 2007).

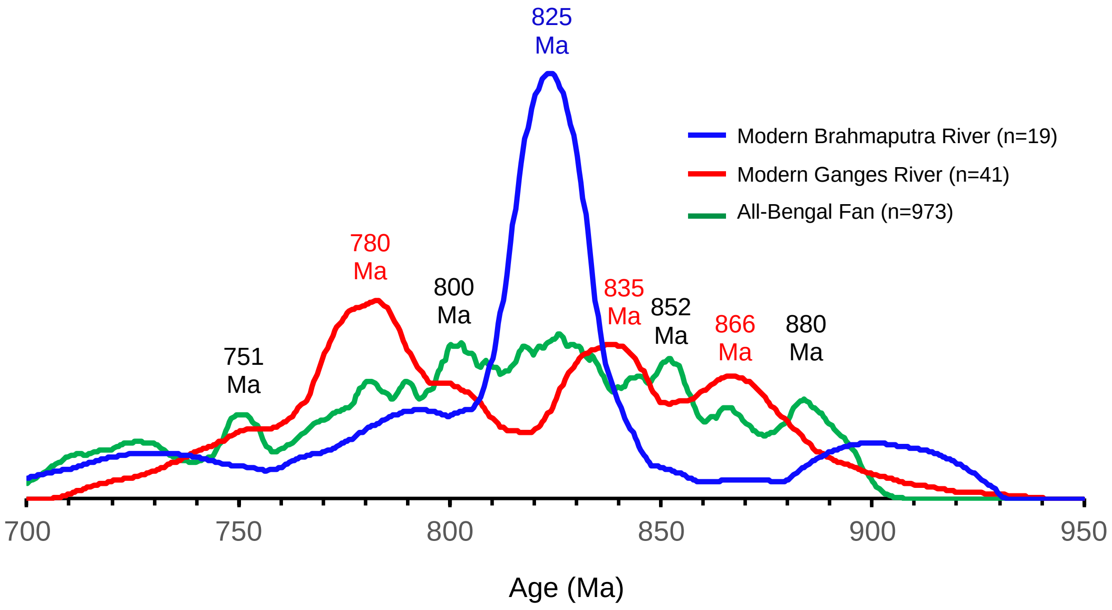

Figure S6. Normalized KDE plots for the 700-930 Ma DZ U-Pb populations for all Bengal Fan data, compared withthe modern Ganges and Brahmaputra Rivers.

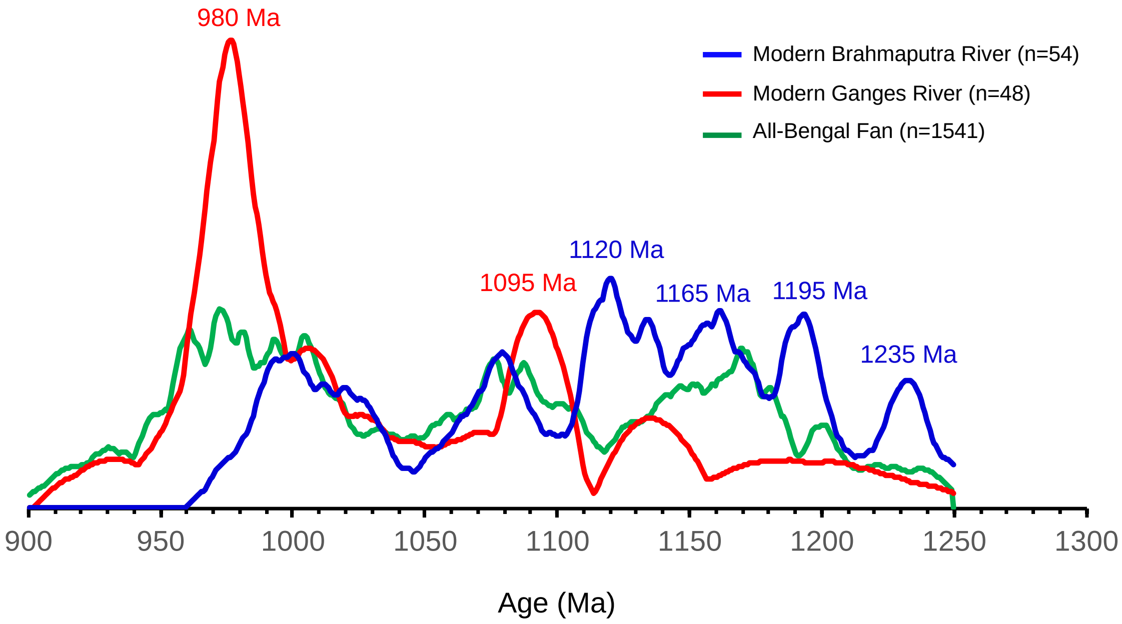

Figure S7. Normalized KDE plots for the 900-1250 Ma DZ U-Pb populations for all Bengal Fan data, compared with the modern Ganges and Brahmaputra Rivers.

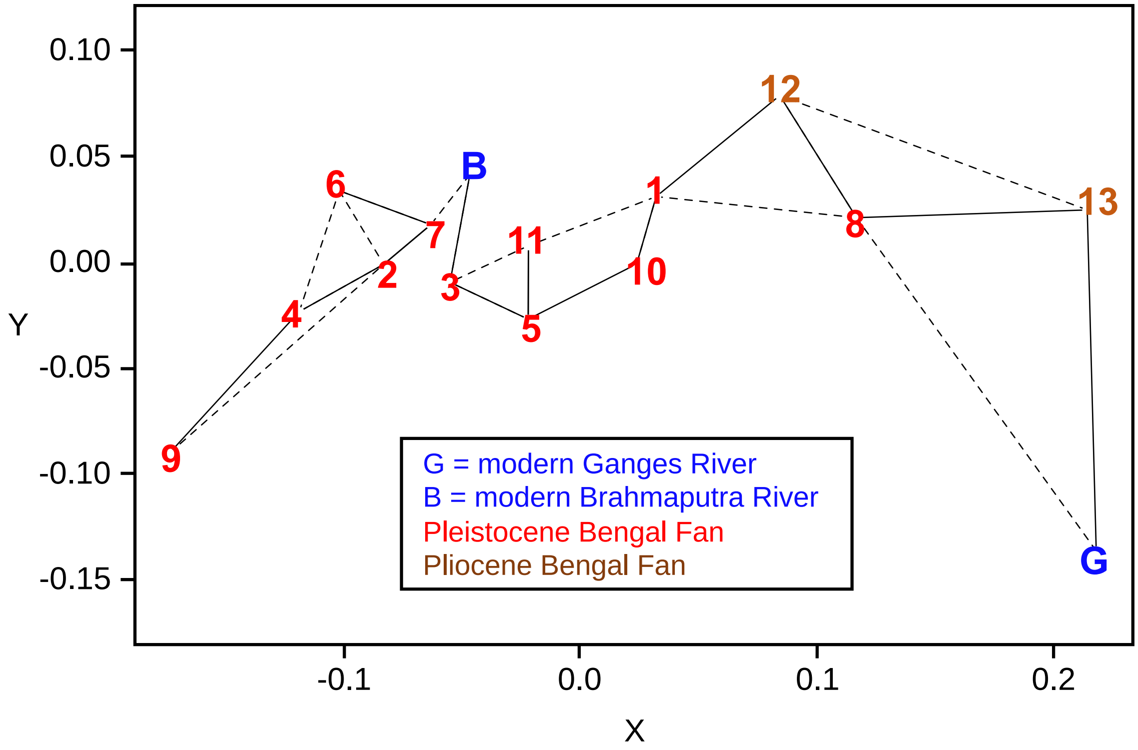

Figure S8. Multi-dimensional scaling plot for the modern Ganges and Brahmaputra Rivers, and Plio-Pleistocene Bengal Fan samples (see Vermeesch et al., 2016 for methods and software). These data illustrate statistical relationships between samples, or the lack thereof, and show that samples 9 and 13 represent end-members within the Plio-Pleistocene Bengal Fan data set.

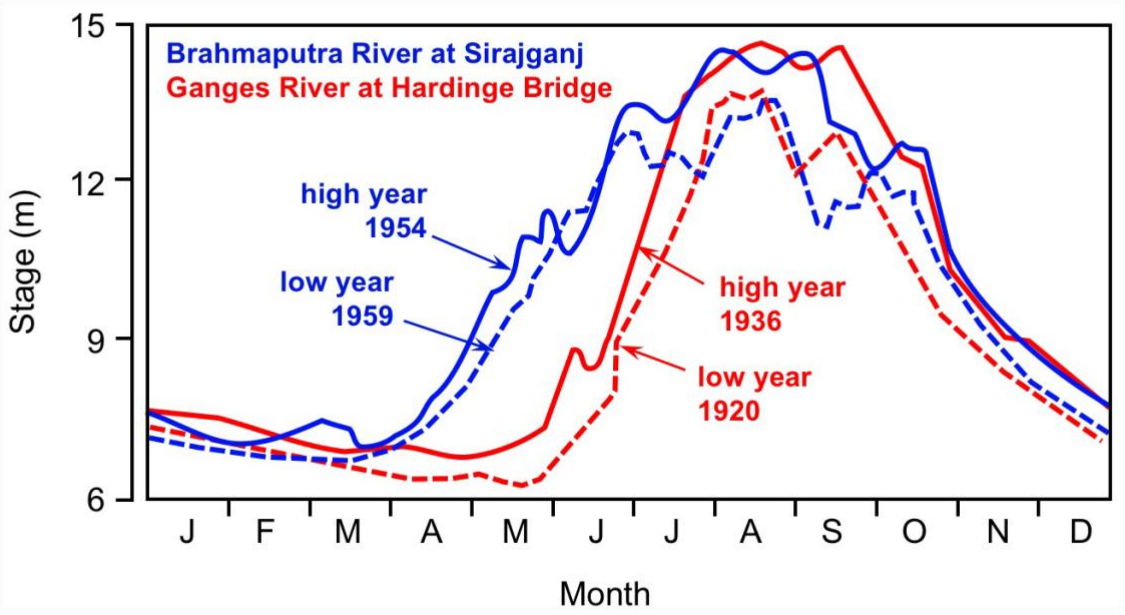

Figure S9. Annual discharge curves for the Ganges and Brahmaputra, showing highest and lowest years of record, and times of peak discharge for the two rivers (after Mirza 1993).

DETRITAL ZIRCON U-PB ANALYSES

This study is based on U-Pb dating of zircons recovered from sand samples collected on the modern Ganges and Brahmaputra Rivers, ~100 km upstream from their confluence, and 25 unconsolidated samples of Bengal Fan turbidite sand and silt from IODP 354 cores, with samples ranging in age from Early Miocene to mid Pleistocene **(Table 1)**. U-Th-Pb detrital zircon laser ablation ICP-MS analysis was conducted at the University of Arizona LaserChron Center (Tucson, AZ) according to methods outlined in Gehrels et al. (2008) and Carrapa et al. (2017).

Initial heavy mineral separations were performed at the Arizona LaserChron Center (ALC) using standard mineral separation techniques. Wilfley wet shaking table, Frantz magnetic separation, and heavy liquid separation were employed to isolate the heavy and detrital zircon (DZ) mineral fractions. For each sample, approximately ~1000 individual DZ grains were mounted on 1” epoxy mounts with fragments of the U-Th-Pb zircon standards Sri Lanka SL2 (563.5 ± 3.2 Ma) and Duluth Complex FC-1 (1099 ± 0.6 Ma). Mounts were polished and imaged using backscatter-electron (**BSE**) and cathode-luminescence (CL) images to identify grain interiors, so as to avoid younger overgrowths, and where possible we target spots on grain cores to ensure targeting of the original protolith ages prior to laser ablation ICP-MS U-Pb analyses. The Sri Lanka SL2 (563.5 ± 3.2 Ma) and Duluth Complex FC-1 (1099 ± 0.6 Ma) U-Th-Pb zircon age standards were analyzed repeatedly during each analytical session, typically once every 5 measurements. Fragments of these large, homogeneous zircon crystals are mounted with DZ grains, allowing for in-run fractionation corrections to be made for Pb/U, Pb isotopes (instrumental mass bias effects) and U-Th abundances. Analytical errors in calculated ages are reported at the 1-sigma level. Analyses that are >10% discordant, or >5% reversely discordant, were discarded and not included in any interpretations.

A Photon Machines Analyte G2 excimer laser system was employed with a 20 micron diameter laser ablation spot size for all analyses, and the sputtered material introduced via purified He gas stream into the argon plasma of an Element 2 (Thermo Fisher) single collector magnetic sector (HR) ICP-MS. Errors in measurement of $^{206}Pb/^{207}Pb$ and $^{206}Pb/^{238}U$ ages are approximately ±1-2% (2-sigma) for zircon ages of >900 Ma and <900 Ma, respectively. ^{204}Hg interference on ^{204}Pb is minimized by use of a gold trap installed directly in the He gas flow stream and monitored by measuring ^{202}Hg continuously during analysis. A correction factor of $^{202}Hg/^{204}Hg = 4.35$ (based on the natural Hg isotopic composition) is employed for data reduction. For most analyses, the Hg correction was insignificant due to uniformly low Hg background (ca. ~150 cps on ^{202}Hg). Pb isotopic compositions are corrected using the Hg-corrected ^{204}Pb measurement and by assuming an initial zircon common Pb isotopic composition derived using the average model Pb crustal evolution curve of Stacy and Kramers (1975).

REFERENCES

Bracciali, L., Parrish, R. R., Najman, Y., Smye, A., Carter, A. & Wijbrans, J. R. (2016) Plio-Pleistocene exhumation of the eastern Himalayan syntaxis and its domal ‘pop-up’. *Earth-Science Reviews*, v. 160, p. 350-385.

Campbell, I. H., Reiners, P. W., Allen, C. M., Nicolescu, S. and Upadhyay, R. (2005) He–Pb double dating of detrital zircons from the Ganges and Indus Rivers: implication for quantifying sediment recycling and provenance studies. *Earth and Planetary Science Letters*, v. 237, p. 402-432.

Carrapa, B., bin Hassim, M. F., Kapp, P. A., DeCelles, P. G., & Gehrels, G. (2017) Tectonic and erosional history of southern Tibet recorded by detrital chronological signatures along the Yarlung River drainage. *Geological Society of America Bulletin*, v. 129, p. 570-581.

Cawood, P.A., Johnson, M.R., & Nemchin, A.A. (2007) Early Palaeozoic orogenesis along the Indian margin of Gondwana: Tectonic response to Gondwana assembly. *Earth and Planetary Science Letters*, v. 255, p. 70-84.

Curray, J. R., Emmel, F. J. & Moore, D. G. (2003) The Bengal Fan: morphology, geometry, stratigraphy, history and processes. *Marine and Petroleum Geology*, v. 19, p. 1191-1223.

Denne, R., Callender, A, and Nault, M. (2005) Applied Biostratigraphix Gulf of Mexico Biostratigraphic Chart Neogene Calcareous Nannofossils, Houston TX.

France-Lanord, C., Spiess, V., Klaus, A., Schwenk, T., and the Expedition 354 Scientists (2016) *Bengal Fan*. Proceedings of the International Ocean Discovery Program, 354: College Station, TX.

Gehrels, G. E., Valencia, V. A., & Ruiz, J. (2008) Enhanced precision, accuracy, efficiency, and spatial resolution of U-Pb ages by laser ablation–multicollector–inductively coupled plasma–mass spectrometry. *Geochemistry, Geophysics, Geosystems,* v. 9, 13 p.

Gehrels, G., Kapp, P., DeCelles, P., Pullen, A., Blakey, R., Weislogel, A., Ding, L., Guynn, J., Martin, A., McQuarrie, N. & Yin, A. (2011) Detrital zircon geochronology of pre-Tertiary strata in the Tibetan-Himalayan orogen. *Tectonics*, v. 30. 27 p.

Gradstein, F.M., Ogg, J.G., Schmitz, M.D. & Ogg, G.M. (2012) *The Geologic Time Scale 2012*. Elsevier.

Mirza, M. M. Q. (2003) Three recent extreme floods in Bangladesh: a hydro-meteorological analysis. In *Flood problem and management in South Asia* 35-64 (Springer Netherlands).

Schwenk, T. & Spieß, V. (2009) Architecture and stratigraphy of the Bengal Fan as response to tectonic and climate revealed from high-resolution seismic data. In *External Controls on Deep-Water Depositional Systems. SEPM Spec. Pub.* **92,** 107-131.

Stacey, J. T., & Kramers, 1. (1975) Approximation of terrestrial lead isotope evolution by a two-stage model. *Earth and Planetary Science Letters,* v. 26, p. 207-221.

Vermeesch, P. (2012) On the visualisation of detrital age distributions. *Chemical Geology,* v. 312, p. 190-194.

Vermeesch, P., Resentini, A. & Garzanti, E. (2016) An R package for statistical provenance analysis. *Sedimentary Geology*, v. 336, p. 14-25.

Wade, B.S., Pearson, P.N., Berggren, W.A. and Pälike, H. (2011) Review and revision of Cenozoic tropical planktonic foraminiferal biostratigraphy and calibration to the geomagnetic polarity and astronomical time scale. *Earth-Science Reviews*, v. 104, p. 111-142.

U-PB DATA FILES

The following contains the raw data files from the Arizona Laserchron Center for DZ U-Pb analyses reported on in this paper. The sample list in Table 1 from the paper is reproduced here.

Table S3 (same as Text Table 1). Summary of detrital zircon samples from IODP Expedition 354. Depth reported in meters below sea floor (Mbsf), whereas numerical age estimates are based on shipboard biostratigraphic interpretations and post-cruise updates (Tables S1 and S2), and "n" = number of concordant U-Pb or Pb-Pb analyses in that sample. Additional data on sample context is provided in Figures S1-S4.

Sample #	Drill Site	Hole	Core	Depth (Mbsf)	Numerical Age Estimate (Ma)	Stratigraphic Age	n
1	U1451	A	4H-6H	25-35	0.3	Middle Pleistocene	273
2	U1450	A	6F-8F	30-44	0.4	Middle Pleistocene	263
3	U1452	B	8F	49-51	0.5	Middle Pleistocene	273
4	U1453	A	11F	370-385	0.5	Middle Pleistocene	268
6	U1453	A	26F	467	0.6	Middle Pleistocene	276
5	U1451	A	13F	70-75	0.6	Middle Pleistocene	259
7	U1452	B	38F	190-192	1.3	Early Pleistocene	260
8	U1453	A	32F	24-40	1.3	Early Pleistocene	291
9	U1449	A	29F-31F	74	1.5	Early Pleistocene	278
10	U1450	A	70F	332-338	2.9	Late Pliocene	259
11	U1450	A	78F-80F	360-370	3.2	Late Pliocene	259
12	U1450	A	98F	465-470	3.5	Late Pliocene	273
13	U1450	A	124F	600-605	3.6	Early Pliocene	271
16	U1451	A	37F	225-230	6.3	Late Miocene	267
17	U1451	A	41F	245-250	6.5	Late Miocene	262
14	U1451	A	49F	280-285	7.2	Late Miocene	282
18	U1451	A	60F	335-340	7.5	Late Miocene	264
19	U1451	A	66F	365-370	8.4	Late Miocene	271
20	U1451	A	80F	430-435	8.7	Late Miocene	264
15	U1451	A	102F	535-540	9.8	Late Miocene	269
21	U1451	B	3X	550-555	10.2	Late Miocene	255
22	U1451	B	22R	715-725	11.5	Late Miocene	281
23	U1451	B	41R	900-905	14.0	Middle Miocene	273
24	U1451	B	53-54R	1015-1020	16	Early Miocene	201
25	U1451	B	62R	1085-1090	18	Early Miocene	210
26	M01	modern Ganges River			0	modern	261
27	J03	modern Brahmaputra River			0	modern	274

Table ______. U-Pb geochronologic analyses.																		
#1 U1451A_4H-6H						Isotope ratios						Apparent ages (Ma)						
Mid Pleistocene 0.3 Ma																		
Analysis	U	206Pb	U/Th	206Pb*	±	207Pb*	±	206Pb*	±	error	206Pb*	±	207Pb*	±	206Pb*	±	Best age	±
	(ppm)	204Pb		207Pb*	(%)	235U*	(%)	238U	(%)	corr.	238U*	(Ma)	235U	(Ma)	207Pb*	(Ma)	(Ma)	(Ma)
-Spot 289	86	728	4.5	3.6201	2.3	0.1713	4.4	0.0045	3.7	0.85	28.9	1.1	160.6	6.5	3341.8	36.7	28.9	1.1
-Spot 309	184	2615	2.3	21.2444	4.3	0.0319	5.1	0.0049	2.7	0.53	31.6	0.8	31.8	1.6	52.8	102.9	31.6	0.8
-Spot 45	5789	56351	22.9	20.9217	0.8	0.0411	1.7	0.0062	1.4	0.87	40.1	0.6	40.9	0.7	89.3	19.5	40.1	0.6
-Spot 229	63	2667	1.4	26.3841	4.1	0.0334	6.4	0.0064	4.9	0.77	41.0	2.0	33.3	2.1	492.1	107.9	41.0	2.0
-Spot 161	527	3529	1.3	21.6705	2.9	0.0428	3.5	0.0067	2.1	0.59	43.2	0.9	42.5	1.5	5.2	68.7	43.2	0.9
-Spot 133	325	12757	1.5	21.0681	2.4	0.0479	3.1	0.0073	1.9	0.62	47.0	0.9	47.5	1.4	72.7	57.1	47.0	0.9
-Spot 1	695	27036	35.5	20.8504	1.4	0.0488	2.9	0.0074	2.5	0.88	47.4	1.2	48.3	1.4	97.3	32.9	47.4	1.2
-Spot 225	257	2305	1.3	14.3006	8.0	0.0715	8.3	0.0074	2.0	0.25	47.6	1.0	70.1	5.6	926.2	164.7	47.6	1.0
-Spot 179	182	942	1.4	25.3119	4.3	0.0405	5.0	0.0074	2.5	0.51	47.8	1.2	40.3	2.0	383.1	111.5	47.8	1.2
-Spot 256	525	15052	289.1	20.6209	1.4	0.0505	2.5	0.0075	2.1	0.83	48.5	1.0	50.0	1.2	123.4	33.0	48.5	1.0
-Spot 313	183	1803	1.4	24.8683	9.0	0.0445	9.2	0.0080	2.2	0.23	51.5	1.1	44.2	4.0	337.3	231.5	51.5	1.1
-Spot 46	1370	27394	0.7	20.5930	1.6	0.0557	2.2	0.0083	1.5	0.68	53.4	0.8	55.0	1.2	126.7	37.8	53.4	0.8
-Spot 235	588	3433	2.5	22.4434	2.3	0.0516	3.1	0.0084	2.1	0.67	53.9	1.1	51.0	1.6	79.8	57.1	53.9	1.1
-Spot 118	851	6885	18.0	19.4133	2.1	0.0617	2.8	0.0087	1.9	0.68	55.8	1.1	60.8	1.7	263.7	47.6	55.8	1.1
-Spot 68	398	13317	1.4	20.6045	1.6	0.0611	3.0	0.0091	2.5	0.85	58.6	1.5	60.2	1.7	125.3	37.0	58.6	1.5
-Spot 250	1397	173909	5.1	20.9230	1.0	0.0627	1.8	0.0095	1.5	0.84	61.0	0.9	61.7	1.1	89.1	23.1	61.0	0.9
-Spot 231	1532	32021	1.1	20.1492	1.3	0.0737	2.9	0.0108	2.6	0.90	69.0	1.8	72.2	2.0	177.7	30.2	69.0	1.8
-Spot 28	198	5923	7.1	20.7624	2.2	0.0716	2.9	0.0108	1.9	0.65	69.1	1.3	70.2	2.0	107.4	52.5	69.1	1.3
-Spot 278	417	16476	1.1	21.0654	1.5	0.0721	2.6	0.0110	2.1	0.81	70.6	1.5	70.7	1.8	73.0	35.9	70.6	1.5
-Spot 20	593	15138	0.8	21.4891	1.4	0.0749	2.0	0.0117	1.5	0.72	74.9	1.1	73.4	1.4	25.4	34.1	74.9	1.1
-Spot 182	72	875	1.4	30.0601	13.6	0.0616	14.0	0.0134	3.2	0.23	86.0	2.7	60.7	8.2	852.1	391.9	86.0	2.7
-Spot 276	89	4305	2.2	19.8740	4.0	0.0942	5.0	0.0136	3.0	0.60	87.0	2.6	91.5	4.3	209.6	92.1	87.0	2.6
-Spot 156	121	2460	1.2	24.4376	5.7	0.0788	6.2	0.0140	2.3	0.38	89.4	2.1	77.0	4.6	292.5	146.2	89.4	2.1
-Spot 264	307	2058	1.7	23.3670	1.5	0.0856	2.6	0.0145	2.2	0.83	92.9	2.0	83.4	2.1	179.5	36.6	92.9	2.0
-Spot 63	51	2143	1.9	17.3278	5.2	0.1189	7.3	0.0149	5.1	0.70	95.6	4.8	114.1	7.8	518.8	114.3	95.6	4.8
-Spot 275	86	704	2.4	28.3921	5.1	0.0757	6.3	0.0156	3.7	0.58	99.7	3.7	74.1	4.5	691.1	142.6	99.7	3.7
-Spot 129	144	3344	0.9	21.2546	2.0	0.1019	3.2	0.0157	2.5	0.78	100.5	2.5	98.5	3.0	51.7	47.8	100.5	2.5
-Spot 36	127	32318	0.9	20.6538	2.3	0.1185	3.6	0.0177	2.7	0.77	113.4	3.1	113.7	3.8	119.7	53.9	113.4	3.1
-Spot 200	662	93101	1.6	20.9454	1.0	0.1173	2.3	0.0178	2.0	0.90	113.9	2.3	112.7	2.4	86.6	23.8	113.9	2.3
-Spot 191	516	29439	1.9	19.5068	1.6	0.1276	2.6	0.0180	2.1	0.79	115.3	2.4	121.9	3.0	252.8	37.0	115.3	2.4
-Spot 76	1830	260691	0.9	20.6506	0.7	0.1212	1.8	0.0181	1.6	0.91	115.9	1.8	116.1	1.9	120.1	16.7	115.9	1.8
-Spot 139	1004	56705	2.0	20.7752	0.8	0.1231	1.9	0.0185	1.7	0.90	118.4	2.0	117.9	2.1	105.9	19.2	118.4	2.0
-Spot 226	2137	40980	2.0	20.1129	1.3	0.1292	2.0	0.0189	1.4	0.73	120.4	1.7	123.4	2.3	181.9	30.9	120.4	1.7
-Spot 209	978	54155	2.8	20.2587	0.9	0.1305	2.1	0.0192	1.9	0.90	122.5	2.3	124.6	2.4	165.0	20.9	122.5	2.3
-Spot 41	3054	43784	6.0	20.6033	0.7	0.1291	1.8	0.0193	1.6	0.92	123.2	2.0	123.3	2.1	125.4	16.6	123.2	2.0
-Spot 271	2764	62313	10.1	20.5345	0.6	0.1303	1.6	0.0194	1.5	0.94	123.9	1.9	124.4	1.9	133.4	13.4	123.9	1.9
-Spot 152	2857	35696	3.7	20.5980	0.9	0.1301	2.0	0.0194	1.8	0.89	124.1	2.2	124.2	2.4	126.1	21.5	124.1	2.2
-Spot 101	496	100730	3.4	20.0082	1.0	0.1370	2.1	0.0199	1.9	0.89	126.9	2.4	130.3	2.6	194.1	22.6	126.9	2.4
-Spot 301	1064	16957	2.1	20.5055	0.9	0.1338	2.3	0.0199	2.1	0.92	127.0	2.6	127.5	2.7	136.6	20.8	127.0	2.6
-Spot 32	182	3819	1.1	21.5103	1.7	0.1277	2.7	0.0199	2.1	0.77	127.1	2.6	122.0	3.1	23.1	41.2	127.1	2.6
-Spot 158	874	12487	5.2	20.9075	1.1	0.1333	2.4	0.0202	2.2	0.89	129.0	2.8	127.1	2.9	90.9	25.9	129.0	2.8
-Spot 299	324	79802	1.1	17.6524	3.4	0.1592	4.9	0.0204	3.6	0.73	130.1	4.6	150.0	6.8	477.9	74.1	130.1	4.6
-Spot 22	866	34899	2.0	20.5839	0.9	0.1428	1.9	0.0213	1.7	0.88	136.0	2.3	135.6	2.5	127.7	21.2	136.0	2.3
-Spot 173	109	2424	3.8	21.6438	4.8	0.1437	5.1	0.0226	1.8	0.36	143.8	2.6	136.4	6.5	8.2	114.6	143.8	2.6
-Spot 69	3631	397973	2.1	20.3638	0.8	0.1546	1.6	0.0228	1.4	0.89	145.6	2.1	146.0	2.2	152.9	17.6	145.6	2.1
-Spot 47	4950	6587696	13.1	17.4050	0.6	0.2113	1.5	0.0267	1.3	0.90	169.7	2.2	194.7	2.6	509.0	14.2	169.7	2.2
-Spot 94	858	22999	2.5	10.5472	0.7	0.3672	1.9	0.0281	1.8	0.93	178.6	3.1	317.6	5.2	1524.3	13.0	178.6	3.1
-Spot 230	149	2859	7.4	21.7450	1.8	0.1787	3.5	0.0282	3.0	0.85	179.2	5.3	167.0	5.4	3.0	44.2	179.2	5.3
-Spot 97	2340	43699	2.8	13.3124	0.5	0.3255	1.4	0.0314	1.3	0.94	199.5	2.6	286.2	3.5	1071.7	9.3	199.5	2.6
-Spot 49	376	93356	2.6	19.5283	0.8	0.2291	2.2	0.0325	2.1	0.93	205.9	4.2	209.5	4.2	250.2	18.4	205.9	4.2
-Spot 48	2306	114150	1.4	19.8035	0.5	0.2263	1.6	0.0325	1.5	0.95	206.2	3.1	207.1	3.0	217.9	12.2	206.2	3.1
-Spot 26	1786	51594	36.0	20.0186	0.7	0.2339	1.5	0.0340	1.3	0.89	215.3	2.8	213.4	2.9	192.9	15.9	215.3	2.8
-Spot 172	395	16806	24.5	20.1174	1.3	0.2342	2.1	0.0342	1.7	0.81	216.6	3.7	213.6	4.1	181.3	29.2	216.6	3.7
-Spot 66	1848	46757	20.9	15.4809	3.4	0.3111	6.5	0.0349	5.6	0.86	221.4	12.1	275.1	15.7	761.2	71.1	221.4	12.1
-Spot 72	350	46928	3.0	17.4263	2.5	0.3015	4.7	0.0381	4.0	0.85	241.1	9.5	267.6	11.1	506.3	55.3	241.1	9.5
-Spot 6	1172	43633	8.1	16.3948	0.6	0.3881	5.7	0.0462	5.7	0.99	290.9	16.2	333.0	16.2	639.1	12.4	290.9	16.2
-Spot 24	6066	91153	6.0	17.3459	0.6	0.3679	4.2	0.0463	4.1	0.99	291.6	11.8	318.1	11.5	516.5	14.2	291.6	11.8
-Spot 121	1979	631520	1.4	19.0609	0.6	0.3511	1.7	0.0485	1.6	0.94	305.6	4.7	305.6	4.4	305.6	13.4	305.6	4.7
-Spot 204	267	6893	1.3	17.4790	0.9	0.4413	2.1	0.0559	1.9	0.89	350.9	6.4	371.1	6.6	499.7	20.8	350.9	6.4
-Spot 259	1637	206035	1.1	17.6026	0.6	0.5066	2.5	0.0647	2.4	0.97	404.0	9.4	416.1	8.5	484.2	14.0	404.0	9.4
-Spot 206	979	21889	1.4	18.0363	0.8	0.5130	2.4	0.0671	2.3	0.95	418.7	9.2	420.5	8.3	430.1	16.8	418.7	9.2
-Spot 254	569	24454	8.8	17.5673	0.7	0.5581	2.2	0.0711	2.1	0.94	442.9	9.1	450.3	8.2	488.6	16.3	442.9	9.1
-Spot 280	1634	235931	2.2	17.5528	0.7	0.5758	2.0	0.0733	1.9	0.95	456.0	8.6	461.8	7.6	490.4	14.4	456.0	8.6
-Spot 251	2425	171984	2.2	17.1915	0.5	0.5926	2.0	0.0739	1.9	0.96	459.5	8.4	472.5	7.4	536.1	11.7	459.5	8.4
-Spot 44	900	80423	7.9	17.6157	0.6	0.5892	1.9	0.0753	1.8	0.95	467.9	8.0	470.4	7.1	482.5	13.1	467.9	8.0
-Spot 61	567	89883	4.1	17.4554	0.6	0.6031	3.4	0.0764	3.4	0.98	474.3	15.5	479.2	13.2	502.7	13.9	474.3	15.5
-Spot 286	341	63011	4.2	17.5855	0.8	0.6052	2.1	0.0772	1.9	0.93	479.3	9.0	480.5	8.1	486.3	17.6	479.3	9.0
-Spot 103	324	134586	0.7	17.4705	0.7	0.6100	1.8	0.0773	1.6	0.93	479.9	7.6	483.6	6.8	500.8	14.4	479.9	7.6
-Spot 263	280	16870	3.1	17.7492	0.8	0.6008	2.6	0.0773	2.4	0.95	480.2	11.3	477.7	9.9	465.8	18.7	480.2	11.3
-Spot 228	1234	99160	16.8	16.9517	0.7	0.6324	1.9	0.0778	1.7	0.92	482.7	8.0	497.6	7.3	566.8	16.0	482.7	8.0
-Spot 292	544	225023	2.1	17.6721	0.8	0.6109	1.8	0.0783	1.6	0.89	486.0	7.5	484.1	6.9	475.4	17.7	486.0	7.5
-Spot 17	186	612824	1.4	17.4038	1.0	0.6204	2.6	0.0783	2.4	0.93	486.0	11.4	490.1	10.2	509.2	21.9	486.0	11.4
-Spot 297	3236	1005546	1.3	17.3000	0.5	0.6259	1.7	0.0785	1.7	0.96	487.4	7.9	493.5	6.8	522.3	10.6	487.4	7.9
-Spot 148	435	34189	0.6	17.5381	0.7	0.6194	1.9	0.0788	1.7	0.93	488.9	8.2	489.5	7.3	492.3	15.7	488.9	8.2
-Spot 60	241	84031	1.8	17.0658	0.8	0.6401	2.2	0.0792	2.0	0.93	491.5	9.7	502.4	8.7	552.1	17.2	491.5	9.7
-Spot 4	393	47184	1.4	17.2786	1.0	0.6323	3.4	0.0792	3.2	0.96	491.6	15.3	497.5	13.3	525.0	21.3	491.6	15.3
-Spot 237	1892	171865	2.1	17.4318	0.6	0.6340	1.8	0.0802	1.6	0.94	497.1	7.8	498.6	6.9	505.6	13.5	497.1	7.8
-Spot 153	1186	76528	3.2	17.4823	0.6	0.6330	1.3	0.0803	1.2	0.88	497.7	5.7	498.0	5.3	499.3	14.0	497.7	5.7
-Spot 143	1036	96995	1.9	17.3927	0.6	0.6376	1.6	0.0804	1.4	0.92	498.7	6.9	500.8	6.2	510.6	13.1	498.7	6.9

-Spot 12	404	29897	1.2	17.4912	0.9	0.6367	2.0	0.0808	1.8	0.90	500.7	8.8	500.3	8.0	498.1	19.1	500.7	8.8
-Spot 23	656	234619	4.4	17.1678	0.6	0.6540	2.0	0.0814	1.9	0.95	504.6	9.1	510.9	7.9	539.1	13.1	504.6	9.1
-Spot 150	222	16910	1.7	16.9216	0.8	0.6688	1.9	0.0821	1.7	0.90	508.5	8.4	520.0	7.8	570.6	18.2	508.5	8.4
-Spot 293	1348	232817	3.2	17.4932	0.4	0.6478	1.6	0.0822	1.5	0.96	509.2	7.6	507.1	6.4	497.9	9.6	509.2	7.6
-Spot 303	544	92680	0.5	17.3815	0.8	0.6525	2.2	0.0823	2.1	0.93	509.5	10.1	510.0	8.9	512.0	17.4	509.5	10.1
-Spot 27	881	217547	2.2	17.4844	0.7	0.6495	2.0	0.0824	1.9	0.94	510.2	9.1	508.1	7.9	499.0	14.6	510.2	9.1
-Spot 180	534	86155	1.0	17.5053	0.6	0.6490	1.8	0.0824	1.7	0.95	510.4	8.5	507.8	7.3	496.4	12.8	510.4	8.5
-Spot 52	209	56940	2.2	17.4510	0.9	0.6544	2.8	0.0828	2.7	0.95	512.9	13.1	511.2	11.3	503.2	19.9	512.9	13.1
-Spot 239	455	142370	29.6	16.8503	0.6	0.6812	1.8	0.0833	1.7	0.95	515.5	8.4	527.5	7.4	579.8	12.4	515.5	8.4
-Spot 199	98	18061	1.2	17.2399	1.1	0.6677	2.5	0.0835	2.2	0.89	516.9	10.9	519.3	10.0	530.0	24.5	516.9	10.9
-Spot 108	249	26928	0.8	17.5157	0.8	0.6574	2.0	0.0835	1.9	0.92	517.1	9.2	513.0	8.1	495.1	17.1	517.1	9.2
-Spot 70	666	45595	2.1	17.3523	0.7	0.6753	2.1	0.0850	2.0	0.95	525.8	10.0	524.0	8.6	515.7	14.8	525.8	10.0
-Spot 168	983	23923	4.2	16.9038	0.6	0.6964	1.9	0.0854	1.8	0.94	528.1	8.9	536.6	7.8	572.9	13.3	528.1	8.9
-Spot 154	187	18947	0.9	17.0887	0.8	0.7036	2.1	0.0872	1.9	0.93	538.9	9.9	540.9	8.6	549.2	16.4	538.9	9.9
-Spot 159	187	436496	1.3	17.0155	0.9	0.7226	2.0	0.0892	1.8	0.88	550.7	9.4	552.2	8.6	558.6	20.6	550.7	9.4
-Spot 155	455	24168	6.8	16.5012	0.7	0.7589	2.3	0.0908	2.2	0.95	560.4	11.9	573.4	10.2	625.1	15.9	560.4	11.9
-Spot 38	578	71855	2.6	17.0153	0.7	0.7468	1.6	0.0922	1.5	0.90	568.3	8.1	566.3	7.2	558.6	15.3	568.3	8.1
-Spot 132	128	9025	0.6	16.9457	1.4	0.7563	2.8	0.0929	2.4	0.86	573.0	13.2	571.9	12.2	567.6	30.5	573.0	13.2
-Spot 59	571	115799	3.4	16.5837	0.8	0.7857	2.5	0.0945	2.4	0.94	582.1	13.4	588.7	11.4	614.3	18.2	582.1	13.4
-Spot 100	355	39102	1.4	16.0573	0.9	0.8135	2.1	0.0947	1.9	0.91	583.5	10.4	604.4	9.4	683.6	18.5	583.5	10.4
-Spot 90	196	27561	0.7	16.3622	1.0	0.8635	2.5	0.1025	2.3	0.92	628.9	13.6	632.0	11.6	643.4	20.8	628.9	13.6
-Spot 8	332	37429	1.5	16.3143	0.8	0.8961	2.1	0.1060	1.9	0.93	649.7	12.0	649.7	10.0	649.7	16.2	649.7	12.0
-Spot 95	151	80225	0.4	16.3413	1.0	0.9101	2.5	0.1079	2.3	0.91	660.4	14.3	657.1	12.1	646.1	22.3	660.4	14.3
-Spot 245	377	1161830	1.6	15.5118	0.9	0.9626	2.5	0.1083	2.3	0.93	662.9	14.7	684.7	12.4	757.0	18.7	662.9	14.7
-Spot 34	151	305039	4.9	15.2545	0.8	0.9889	2.3	0.1094	2.2	0.94	669.3	13.8	698.1	11.7	792.2	16.7	669.3	13.8
-Spot 65	2742	236994	1.5	14.9455	0.6	1.0284	2.0	0.1115	1.9	0.95	681.3	12.5	718.1	10.5	835.0	13.5	681.3	12.5
-Spot 294	82	20126	1.4	15.9469	0.9	0.9668	2.7	0.1118	2.5	0.94	683.3	16.5	686.8	13.5	698.4	19.3	683.3	16.5
-Spot 253	2427	322972	1.8	15.1240	0.5	1.0214	2.0	0.1120	2.0	0.97	684.6	12.7	714.6	10.3	810.2	9.6	684.6	12.7
-Spot 270	1464	307079	1.7	14.9967	0.4	1.0705	1.5	0.1164	1.4	0.95	710.0	9.7	739.0	7.9	827.9	9.4	710.0	9.7
-Spot 165	173	14518	1.2	15.3116	0.7	1.0525	2.1	0.1169	2.0	0.95	712.6	13.7	730.1	11.2	784.3	14.5	712.6	13.7
-Spot 269	96	15504	0.8	15.6683	1.0	1.0368	3.3	0.1178	3.2	0.95	718.0	21.4	722.3	17.1	735.8	21.9	718.0	21.4
-Spot 75	586	2487812	3.3	15.0492	0.7	1.1549	1.8	0.1261	1.6	0.92	765.3	11.9	779.5	9.7	820.6	14.3	765.3	11.9
-Spot 43	83	2621022	1.9	14.4607	1.3	1.2103	3.1	0.1269	2.8	0.91	770.4	20.3	805.3	17.1	903.3	26.0	770.4	20.3
-Spot 57	780	1598330	1.4	15.2676	0.5	1.1924	1.9	0.1320	1.8	0.96	799.5	13.9	797.1	10.6	790.4	11.4	799.5	13.9
-Spot 157	1990	297941	1.5	14.9101	0.5	1.2211	1.8	0.1321	1.7	0.96	799.6	12.6	810.3	9.8	839.9	10.6	799.6	12.6
-Spot 53	941	264105	1.5	14.9360	0.7	1.2194	1.9	0.1321	1.8	0.93	799.8	13.3	809.5	10.6	836.3	15.1	799.8	13.3
-Spot 39	843	111055	1.6	14.7909	0.8	1.2317	2.6	0.1321	2.4	0.95	800.0	18.3	815.1	14.4	856.6	17.0	800.0	18.3
-Spot 262	840	1419356	2.1	15.0002	0.6	1.2153	1.7	0.1322	1.6	0.94	800.5	11.8	807.6	9.3	827.3	12.1	800.5	11.8
-Spot 5	146	14737	1.6	15.1346	0.8	1.2218	2.2	0.1341	2.0	0.93	811.3	15.4	810.6	12.2	808.7	17.1	811.3	15.4
-Spot 232	101	15934	1.2	15.1206	1.0	1.2243	2.7	0.1343	2.5	0.93	812.1	19.4	811.7	15.2	810.6	20.3	812.1	19.4
-Spot 261	2302	112305	2.1	14.8854	0.7	1.2488	1.8	0.1348	1.7	0.93	815.3	13.0	822.9	10.2	843.4	13.7	815.3	13.0
-Spot 181	693	191511	3.4	14.9789	0.7	1.2566	1.8	0.1365	1.6	0.91	824.9	12.4	826.4	9.9	830.3	15.4	824.9	12.4
-Spot 282	902	71629	0.9	14.6479	0.5	1.3206	1.9	0.1403	1.8	0.96	846.3	14.2	854.8	10.8	876.7	11.2	846.3	14.2
-Spot 188	258	47218	1.9	14.9295	0.7	1.3002	2.2	0.1408	2.1	0.95	849.1	17.1	845.8	12.9	837.2	14.0	849.1	17.1
-Spot 187	496	124929	4.3	15.0358	0.6	1.2970	1.7	0.1414	1.6	0.95	852.8	13.0	844.4	9.9	822.4	11.5	852.8	13.0
-Spot 109	381	100025	1.0	14.7557	0.6	1.3278	1.8	0.1421	1.7	0.94	856.5	13.7	857.9	10.5	861.5	12.6	856.5	13.7
-Spot 285	120	71601	1.2	14.6923	0.9	1.3450	2.6	0.1433	2.4	0.94	863.4	19.5	865.4	14.9	870.5	17.8	863.4	19.5
-Spot 215	117	16271	0.9	14.4677	1.3	1.3786	3.0	0.1447	2.7	0.91	871.0	21.9	879.9	17.5	902.3	25.8	871.0	21.9
-Spot 304	308	44794	0.6	14.4611	0.7	1.4055	2.4	0.1474	2.3	0.95	886.4	18.9	891.3	14.3	903.2	15.1	886.4	18.9
-Spot 19	84	19972	2.5	14.5088	0.8	1.4107	2.8	0.1484	2.7	0.96	892.2	22.5	893.4	16.8	896.4	16.8	892.2	22.5
-Spot 35	349	128329	1.9	14.3004	0.7	1.5027	1.8	0.1559	1.7	0.93	933.7	14.6	931.5	11.1	926.2	14.1	926.2	14.1
-Spot 89	201	35327	1.3	14.2958	0.7	1.5280	1.8	0.1584	1.7	0.93	948.0	14.9	941.7	11.2	926.9	14.0	926.9	14.0
-Spot 58	211	75896	1.3	14.1442	0.6	1.5579	1.7	0.1598	1.6	0.93	955.7	14.0	953.6	10.5	948.8	12.9	948.8	12.9
-Spot 25	182	186723	0.8	14.0816	0.7	1.5927	1.8	0.1627	1.7	0.93	971.6	15.4	967.4	11.4	957.8	13.7	957.8	13.7
-Spot 257	271	147622	1.9	14.0552	0.6	1.5709	2.7	0.1601	2.6	0.98	957.5	23.5	958.8	16.8	961.7	12.2	961.7	12.2
-Spot 119	3217	2673883	1.3	14.0432	0.6	1.6114	2.0	0.1641	1.9	0.96	979.6	17.6	974.6	12.7	963.4	11.9	963.4	11.9
-Spot 279	1228	129113	3.4	14.0333	0.7	1.4412	2.0	0.1467	1.9	0.93	882.3	15.4	906.2	12.1	964.9	15.0	964.9	15.0
-Spot 183	102	16393	0.6	14.0311	0.6	1.5056	1.9	0.1532	1.8	0.95	919.0	15.7	932.7	11.8	965.2	12.7	965.2	12.7
-Spot 14	113	15557	3.0	13.9886	0.7	1.3932	2.8	0.1413	2.7	0.97	852.3	21.7	886.0	16.6	971.4	14.8	971.4	14.8
-Spot 249	204	40390	1.2	13.9028	0.9	1.5674	2.7	0.1580	2.5	0.94	945.9	22.1	957.4	16.5	983.9	18.0	983.9	18.0
-Spot 164	168	70243	1.4	13.8682	0.6	1.5886	2.1	0.1598	2.0	0.96	955.6	17.6	965.7	12.9	989.0	12.2	989.0	12.2
-Spot 222	73	50500	1.4	13.8237	0.9	1.6407	3.2	0.1645	3.1	0.96	981.7	28.1	986.0	20.2	995.5	17.8	995.5	17.8
-Spot 146	459	131375	0.6	13.8199	0.5	1.6315	1.7	0.1635	1.6	0.96	976.4	14.4	982.4	10.4	996.1	9.2	996.1	9.2
-Spot 98	380	98062	1.5	13.7821	0.6	1.6416	2.1	0.1641	2.0	0.96	979.4	18.6	986.3	13.4	1001.6	11.6	1001.6	11.6
-Spot 77	167	111113	1.7	13.7797	0.7	1.6999	2.2	0.1699	2.1	0.94	1011.5	19.6	1008.5	14.2	1002.0	15.1	1002.0	15.1
-Spot 233	540	554870	1.0	13.7738	0.7	1.5666	2.0	0.1565	1.8	0.93	937.3	15.8	957.1	12.1	1002.8	14.8	1002.8	14.8
-Spot 116	693	172975	2.4	13.7716	0.4	1.6617	1.4	0.1660	1.4	0.96	989.9	12.8	994.0	9.2	1003.2	8.4	1003.2	8.4
-Spot 96	281	62036	1.2	13.7515	0.8	1.6818	2.2	0.1677	2.0	0.93	999.6	18.7	1001.7	13.8	1006.1	16.2	1006.1	16.2
-Spot 2	181	1480378	1.4	13.7300	0.8	1.6648	2.6	0.1658	2.5	0.95	988.8	22.9	995.2	16.7	1009.3	16.5	1009.3	16.5
-Spot 212	232	224555	1.5	13.6867	0.6	1.6640	2.3	0.1652	2.2	0.97	985.5	20.4	994.9	14.6	1015.7	11.8	1015.7	11.8
-Spot 112	193	28256	2.8	13.6700	0.8	1.6904	2.3	0.1676	2.1	0.94	998.8	19.9	1004.9	14.6	1018.2	16.0	1018.2	16.0
-Spot 93	252	56325	1.9	13.6353	0.8	1.6753	2.7	0.1657	2.6	0.96	988.2	23.6	999.2	17.1	1023.3	16.0	1023.3	16.0
-Spot 151	3953	331629	16.8	13.6020	0.5	1.7536	1.3	0.1730	1.2	0.94	1028.6	11.7	1028.5	8.5	1028.3	9.2	1028.3	9.2
-Spot 315	59	53202	1.1	13.5838	0.9	1.5545	2.4	0.1531	2.2	0.92	918.6	18.7	952.3	14.7	1031.0	19.2	1031.0	19.2
-Spot 283	427	2876338	0.8	13.5551	0.5	1.6976	1.8	0.1669	1.7	0.96	995.0	16.0	1007.6	11.6	1035.2	10.1	1035.2	10.1
-Spot 163	590	21760	1.2	13.5473	0.6	1.5638	2.0	0.1537	1.9	0.95	921.4	16.0	956.0	12.2	1036.4	13.0	1036.4	13.0
-Spot 296	602	132145	6.6	13.4434	0.6	1.6507	1.8	0.1609	1.7	0.94	962.0	15.5	989.8	11.7	1052.0	12.7	1052.0	12.7
-Spot 145	497	160782	4.3	13.3169	0.6	1.8856	2.0	0.1821	1.9	0.96	1078.5	19.0	1076.0	13.3	1071.0	11.8	1071.0	11.8
-Spot 67	606	165605	5.8	13.3008	0.7	1.8692	2.1	0.1803	2.0	0.95	1068.7	19.8	1070.2	14.1	1073.4	13.9	1073.4	13.9
-Spot 134	503	103970	3.6	13.2524	1.3	1.7738	3.4	0.1705	3.1	0.92	1014.8	29.2	1035.9	22.0	1080.7	26.8	1080.7	26.8
-Spot 84	321	61675	1.3	13.1776	0.6	1.9742	2.0	0.1887	1.9	0.95	1114.2	19.3	1106.7	13.4	1092.0	12.7	1092.0	12.7
-Spot 31	466	107499	0.7	13.1396	0.7	1.9966	3.1	0.1903	3.0	0.97	1122.9	31.2	1114.4	21.1	1097.8	14.8	1097.8	14.8
-Spot 248	918	115039	1.7	13.1275	0.5	1.8247	1.5	0.1737	1.4	0.94	1032.6	13.3	1054.4	9.8	1099.7	10.5	1099.7	10.5
-Spot 241	318	126694	2.7	12.9714	0.9	1.7546	2.4	0.1651	2.2	0.92	984.9	20.4	1028.9	15.7	1123.6	18.9	1123.6	18.9
-Spot 124	194	57961	0.7	12.9579	0.6	2.0754	1.9	0.1950	1.8	0.94	1148.6	18.6	1140.7	12.9	1125.7	12.7	1125.7	12.7
-Spot 216	106	21961	1.1	12.9347	0.7	1.9336	2.7	0.1814	2.6	0.96	1074.6	25.3	1092.8	17.8	1129.2	14.4	1129.2	14.4

-Spot 288	47	12992	1.3	12.9164	1.1	2.1036	3.9	0.1971	3.8	0.96	1159.5	39.9	1150.0	27.0	1132.0	22.1	1132.0	22.1
-Spot 114	212	42359	1.3	12.8946	0.6	2.0275	1.9	0.1896	1.8	0.95	1119.2	18.4	1124.8	12.9	1135.4	12.2	1135.4	12.2
-Spot 85	190	33343	1.7	12.8707	0.7	2.0861	2.4	0.1947	2.3	0.96	1147.0	24.2	1144.2	16.6	1139.1	14.2	1139.1	14.2
-Spot 300	108	14841	0.7	12.8704	1.0	2.0740	2.2	0.1936	2.0	0.90	1140.8	21.1	1140.3	15.4	1139.1	19.3	1139.1	19.3
-Spot 272	422	305841	6.9	12.8342	0.6	2.1000	2.4	0.1955	2.4	0.97	1151.0	24.8	1148.8	16.7	1144.7	12.0	1144.7	12.0
-Spot 205	1003	65647	2.1	12.8242	0.6	1.9108	1.6	0.1777	1.5	0.93	1054.5	14.3	1084.9	10.5	1146.3	11.1	1146.3	11.1
-Spot 242	2489	375371	3.9	12.7970	0.6	2.1174	1.5	0.1965	1.3	0.91	1156.6	14.3	1154.5	10.2	1150.5	12.2	1150.5	12.2
-Spot 312	324	218663	2.3	12.7949	0.7	2.1835	2.0	0.2026	1.9	0.93	1189.4	20.1	1175.8	13.8	1150.9	14.1	1150.9	14.1
-Spot 125	20	10542	1.1	12.7675	1.1	2.0432	5.8	0.1892	5.7	0.98	1117.0	58.0	1130.0	39.4	1155.1	22.7	1155.1	22.7
-Spot 314	87	71084	2.0	12.7635	0.9	1.9006	2.4	0.1759	2.2	0.93	1044.8	21.1	1081.3	15.7	1155.7	17.5	1155.7	17.5
-Spot 71	118	30769	1.6	12.7457	0.7	2.0478	2.7	0.1893	2.6	0.96	1117.6	26.7	1131.6	18.4	1158.5	14.1	1158.5	14.1
-Spot 208	406	240514	0.5	12.7313	0.6	2.0481	1.7	0.1891	1.6	0.93	1116.6	16.7	1131.7	11.9	1160.7	12.5	1160.7	12.5
-Spot 7	133	22784	1.0	12.7208	0.8	2.2121	2.4	0.2041	2.2	0.94	1197.2	24.4	1184.9	16.6	1162.4	16.0	1162.4	16.0
-Spot 135	378	198041	2.2	12.7101	0.7	2.0422	1.8	0.1883	1.7	0.93	1111.9	17.4	1129.7	12.4	1164.0	13.1	1164.0	13.1
-Spot 196	253	78329	1.4	12.7055	0.8	1.9392	2.0	0.1787	1.8	0.91	1059.9	17.8	1094.7	13.3	1164.7	16.0	1164.7	16.0
-Spot 120	240	77709	1.1	12.7051	0.8	2.1227	2.3	0.1956	2.1	0.94	1151.6	22.6	1156.2	15.8	1164.8	15.7	1164.8	15.7
-Spot 54	429	81777	1.2	12.6655	0.5	2.0019	2.0	0.1839	1.9	0.96	1088.2	18.8	1116.2	13.2	1171.0	10.6	1171.0	10.6
-Spot 189	88	13434	1.3	12.6434	0.6	2.2657	2.8	0.2078	2.7	0.97	1216.9	29.9	1201.7	19.5	1174.5	12.5	1174.5	12.5
-Spot 62	693	1135011	2.3	12.6294	0.9	2.1371	2.0	0.1957	1.8	0.90	1152.4	19.3	1160.9	14.0	1176.7	17.1	1176.7	17.1
-Spot 79	250	103519	2.4	12.6124	0.7	2.0993	2.3	0.1920	2.2	0.95	1132.4	22.7	1148.6	15.8	1179.3	13.5	1179.3	13.5
-Spot 106	249	11105	0.7	12.5671	2.2	2.0767	3.2	0.1893	2.2	0.71	1117.5	23.0	1141.1	21.7	1186.4	44.1	1186.4	44.1
-Spot 51	30	18279	0.5	12.4424	1.1	2.2244	4.2	0.2007	4.0	0.96	1179.2	43.3	1188.8	29.3	1206.1	22.0	1206.1	22.0
-Spot 258	640	3291423	5.5	12.3871	0.5	1.8244	2.2	0.1639	2.1	0.97	978.4	19.3	1054.3	14.3	1214.8	9.6	1214.8	9.6
-Spot 194	1566	126160	1.1	12.3443	0.7	2.2158	2.2	0.1984	2.1	0.96	1166.6	22.9	1186.1	15.7	1221.6	13.0	1221.6	13.0
-Spot 284	78	16205	9.8	12.2541	1.0	2.2316	3.9	0.1983	3.7	0.97	1166.4	40.0	1191.0	27.2	1236.1	19.4	1236.1	19.4
-Spot 64	609	349965	1.4	12.2116	0.7	2.1955	1.7	0.1944	1.6	0.93	1145.4	17.0	1179.6	12.2	1242.9	12.8	1242.9	12.8
-Spot 166	83	244329	0.8	12.0470	0.5	2.5044	2.7	0.2188	2.6	0.98	1275.6	30.4	1273.3	19.5	1269.4	10.7	1269.4	10.7
-Spot 177	232	57063	0.8	11.9057	0.6	2.5865	2.0	0.2233	1.9	0.96	1299.5	22.6	1296.8	14.7	1292.4	11.3	1292.4	11.3
-Spot 99	123	12196	1.7	11.8392	0.8	2.1422	2.0	0.1839	1.8	0.91	1088.5	18.1	1162.5	13.8	1303.3	16.0	1303.3	16.0
-Spot 88	571	9835887	4.7	11.7435	0.6	2.5028	1.7	0.2132	1.5	0.93	1245.7	17.5	1272.8	12.0	1319.0	11.4	1319.0	11.4
-Spot 80	762	217278	232.7	11.5589	1.0	2.3404	2.3	0.1962	2.1	0.91	1154.9	22.4	1224.6	16.6	1349.7	18.9	1349.7	18.9
-Spot 10	2162	382530	1.3	11.4660	0.5	2.6782	1.7	0.2227	1.6	0.95	1296.2	18.7	1322.5	12.4	1365.2	10.0	1365.2	10.0
-Spot 207	417	41271	1.3	11.4014	0.7	2.4310	2.0	0.2010	1.9	0.93	1180.8	20.2	1251.8	14.4	1376.1	13.9	1376.1	13.9
-Spot 171	116	76625	1.5	11.0662	0.7	3.0848	2.0	0.2476	1.9	0.94	1426.0	24.1	1428.9	15.3	1433.2	12.7	1433.2	12.7
-Spot 273	768	673898	2.3	11.0466	0.6	2.8394	2.0	0.2275	2.0	0.96	1321.3	23.6	1366.0	15.4	1436.6	10.5	1436.6	10.5
-Spot 74	184	29428	1.9	10.9227	0.6	3.1597	4.3	0.2503	4.2	0.99	1440.1	54.2	1447.4	32.8	1458.1	12.2	1458.1	12.2
-Spot 265	358	48438	4.1	10.8701	0.9	2.6362	2.5	0.2078	2.4	0.94	1217.3	26.3	1310.8	18.6	1467.3	17.0	1467.3	17.0
-Spot 9	120	34945	1.6	10.8446	0.8	3.2241	3.2	0.2536	3.1	0.97	1456.9	40.3	1463.0	24.8	1471.7	15.7	1471.7	15.7
-Spot 160	314	2445050	3.5	10.7611	0.7	2.7082	3.4	0.2114	3.3	0.98	1236.1	37.5	1330.7	25.2	1486.4	12.6	1486.4	12.6
-Spot 82	52	62631	1.8	10.7358	0.8	3.4676	3.5	0.2700	3.4	0.97	1540.8	46.9	1519.9	27.7	1490.8	15.0	1490.8	15.0
-Spot 198	734	82095	1.1	10.5475	0.6	2.8630	2.2	0.2190	2.1	0.97	1276.6	24.8	1372.2	16.7	1524.3	10.9	1524.3	10.9
-Spot 202	25	4166	1.4	10.5144	2.0	3.6379	5.4	0.2774	5.0	0.93	1578.3	69.5	1557.9	42.7	1530.2	37.8	1530.2	37.8
-Spot 40	141	24860	1.7	10.4764	0.5	3.5072	2.4	0.2665	2.3	0.97	1522.9	31.4	1528.8	18.8	1537.0	10.3	1537.0	10.3
-Spot 170	236	1714614	8.8	10.4693	1.9	2.9867	3.3	0.2268	2.7	0.82	1317.6	31.8	1404.2	24.8	1538.3	35.4	1538.3	35.4
-Spot 107	292	152490	1.7	10.4329	0.6	3.0765	2.1	0.2328	2.1	0.96	1349.1	25.1	1426.9	16.5	1544.8	11.1	1544.8	11.1
-Spot 281	313	322766	3.3	10.3961	0.7	3.2563	2.6	0.2455	2.5	0.96	1415.4	32.1	1470.7	20.4	1551.5	13.5	1551.5	13.5
-Spot 308	118	46638	2.0	10.3004	0.5	3.7166	2.1	0.2776	2.0	0.97	1579.5	28.5	1574.9	16.8	1568.8	9.4	1568.8	9.4
-Spot 213	132	101954	1.5	10.2335	0.8	3.6899	2.1	0.2739	2.0	0.93	1560.4	27.8	1569.2	17.1	1581.0	14.2	1581.0	14.2
-Spot 104	399	133619	0.7	10.1739	0.5	3.8047	1.6	0.2807	1.5	0.95	1595.1	21.1	1593.7	12.7	1591.9	9.4	1591.9	9.4
-Spot 37	269	47134	1.7	10.1714	0.7	3.5274	2.6	0.2602	2.6	0.97	1490.9	34.0	1533.4	20.9	1592.4	12.4	1592.4	12.4
-Spot 130	63	29672	0.8	10.1180	0.8	3.7832	2.6	0.2776	2.5	0.95	1579.4	34.6	1589.2	21.0	1602.2	15.8	1602.2	15.8
-Spot 83	201	52943	1.1	10.0682	0.6	3.8937	2.0	0.2843	1.9	0.95	1613.1	26.6	1612.4	15.9	1611.4	11.8	1611.4	11.8
-Spot 86	1031	321051	1.4	9.9917	0.6	3.8811	1.7	0.2812	1.7	0.94	1597.6	23.4	1609.7	14.1	1625.6	10.7	1625.6	10.7
-Spot 274	1370	96760	3.0	9.9554	0.5	3.2532	1.4	0.2349	1.3	0.93	1360.1	16.0	1469.9	10.9	1632.4	9.5	1632.4	9.5
-Spot 131	1467	1463945	1.0	9.9422	0.5	3.9597	1.3	0.2855	1.2	0.91	1619.1	16.7	1626.0	10.4	1634.8	9.7	1634.8	9.7
-Spot 122	443	98116	1.6	9.8930	0.6	4.1269	2.3	0.2961	2.2	0.96	1672.0	32.4	1659.6	18.7	1644.0	11.2	1644.0	11.2
-Spot 126	238	3449333	1.6	9.8889	0.8	3.9309	2.1	0.2819	2.0	0.92	1601.1	27.7	1620.1	17.2	1644.8	15.2	1644.8	15.2
-Spot 162	340	86267	1.6	9.7762	0.6	4.1479	2.1	0.2941	2.0	0.96	1662.0	29.0	1663.8	16.8	1666.0	10.3	1666.0	10.3
-Spot 142	368	82511	1.6	9.7719	0.6	4.2018	1.5	0.2978	1.4	0.93	1680.4	20.4	1674.4	12.2	1666.9	10.3	1666.9	10.3
-Spot 111	413	11686	0.9	9.6618	0.6	3.9001	1.7	0.2733	1.6	0.94	1557.5	22.3	1613.7	13.8	1687.8	10.6	1687.8	10.6
-Spot 136	3546	303505	1.6	9.5779	0.6	4.2685	1.9	0.2965	1.8	0.95	1674.0	27.2	1687.3	15.9	1703.9	10.7	1703.9	10.7
-Spot 310	263	70402	1.8	9.5079	0.6	4.2968	2.0	0.2963	2.0	0.96	1672.9	28.9	1692.7	16.9	1717.4	10.7	1717.4	10.7
-Spot 141	1422	815574	1.3	9.5009	0.8	3.7111	2.8	0.2557	2.7	0.96	1467.9	35.1	1573.8	22.3	1718.7	14.4	1718.7	14.4
-Spot 255	385	140371	1.5	9.4350	0.6	4.4467	2.4	0.3043	2.3	0.97	1712.5	35.0	1721.1	20.0	1731.5	11.5	1731.5	11.5
-Spot 311	351	299314	1.0	9.4342	0.5	4.5243	1.5	0.3096	1.5	0.96	1738.6	22.5	1735.4	12.8	1731.7	8.4	1731.7	8.4
-Spot 243	411	1066345	1.1	9.4197	0.5	4.4858	1.8	0.3065	1.8	0.96	1723.3	26.5	1728.3	15.2	1734.5	9.4	1734.5	9.4
-Spot 30	124	315484	2.1	9.3781	0.6	4.6663	2.4	0.3174	2.3	0.97	1776.9	35.4	1761.2	19.7	1742.6	11.1	1742.6	11.1
-Spot 176	368	179756	1.6	9.2769	0.5	4.5326	2.1	0.3050	2.0	0.97	1715.9	30.1	1737.0	17.2	1762.4	9.7	1762.4	9.7
-Spot 211	616	142553	18.5	9.1654	0.6	4.1766	2.1	0.2776	2.0	0.96	1579.4	27.5	1669.4	16.8	1784.5	10.9	1784.5	10.9
-Spot 217	1129	339121	9.5	9.1527	0.7	4.8388	2.1	0.3212	2.0	0.95	1795.6	30.8	1791.7	17.4	1787.0	12.0	1787.0	12.0
-Spot 91	556	1280435	9.9	9.1103	0.5	4.2568	1.6	0.2813	1.5	0.94	1597.7	21.4	1685.0	13.2	1795.5	10.0	1795.5	10.0
-Spot 184	399	32177	0.6	9.0849	0.7	3.9614	2.6	0.2610	2.5	0.96	1495.1	33.0	1626.3	20.9	1800.6	13.4	1800.6	13.4
-Spot 16	184	74248	1.6	8.9248	0.6	5.3401	2.4	0.3457	2.4	0.97	1913.8	39.1	1875.3	20.9	1832.9	11.1	1832.9	11.1
-Spot 29	313	380199	1.2	8.8838	0.4	4.8012	1.7	0.3093	1.7	0.97	1737.5	25.3	1785.1	14.5	1841.2	8.1	1841.2	8.1
-Spot 144	725	194443	2.0	8.8606	0.6	4.2740	2.2	0.2747	2.1	0.97	1564.4	29.4	1688.4	18.0	1845.9	10.0	1845.9	10.0
-Spot 33	183	364869	3.0	8.8017	0.6	5.0013	2.5	0.3193	2.4	0.97	1786.1	37.0	1819.5	20.7	1858.0	11.0	1858.0	11.0
-Spot 287	75	175069	1.3	8.7732	0.7	5.3099	3.4	0.3379	3.3	0.98	1876.4	53.5	1870.5	28.7	1863.9	12.9	1863.9	12.9
-Spot 15	482	137417	2.3	8.7482	0.6	5.2909	1.7	0.3357	1.6	0.94	1865.9	25.9	1867.4	14.6	1869.0	10.9	1869.0	10.9
-Spot 185	885	170195	3.2	8.7459	0.6	5.3549	2.2	0.3397	2.1	0.96	1885.1	33.6	1877.7	18.4	1869.5	11.2	1869.5	11.2
-Spot 244	421	64159	3.0	8.7257	0.7	5.5505	4.1	0.3513	4.0	0.98	1940.6	67.6	1908.4	35.3	1873.7	13.3	1873.7	13.3
-Spot 50	278	175243	2.8	8.7185	0.6	4.6864	2.4	0.2963	2.3	0.96	1673.1	33.3	1764.8	19.7	1875.1	11.7	1875.1	11.7
-Spot 298	271	216935	3.1	8.7093	0.6	5.3147	2.0	0.3357	1.9	0.95	1866.0	30.6	1871.2	17.0	1877.0	11.0	1877.0	11.0
-Spot 78	109	183952	1.7	8.6400	0.7	5.3472	2.8	0.3351	2.7	0.97	1862.9	43.9	1876.4	24.0	1891.4	13.1	1891.4	13.1
-Spot 234	847	84649	8.9	8.4049	0.7	5.4231	2.0	0.3306	1.8	0.94	1841.2	29.6	1888.5	16.8	1940.9	11.8	1940.9	11.8
-Spot 117	165	213754	1.4	8.3623	0.6	5.8021	2.0	0.3519	1.9	0.96	1943.6	32.3	1946.7	17.4	1950.0	10.4	1950.0	10.4
-Spot 55	211	306720	6.4	8.3069	0.5	5.6879	4.0	0.3427	4.0	0.99	1899.6	65.6	1929.5	34.8	1961.9	9.5	1961.9	9.5

-Spot 127	133	194058	1.4	8.1480	0.6	6.2147	2.2	0.3673	2.1	0.96	2016.5	37.2	2006.5	19.6	1996.3	11.1	1996.3	11.1
-Spot 123	358	196031	2.9	7.7860	0.7	6.1900	2.3	0.3495	2.2	0.96	1932.4	37.4	2003.0	20.4	2076.6	11.8	2076.6	11.8
-Spot 218	258	628685	2.7	7.5323	0.6	6.8249	2.2	0.3728	2.1	0.96	2042.8	36.6	2089.0	19.4	2134.8	11.1	2134.8	11.1
-Spot 102	172	121652	1.2	7.4881	0.5	7.5071	2.3	0.4077	2.3	0.98	2204.4	42.2	2173.8	20.7	2145.1	8.0	2145.1	8.0
-Spot 140	2689	1202724	48.3	7.4858	0.3	7.5961	2.1	0.4124	2.0	0.99	2225.9	38.1	2184.4	18.4	2145.6	6.0	2145.6	6.0
-Spot 13	104	4430786	1.6	6.7144	0.8	8.7401	2.4	0.4256	2.3	0.94	2286.0	43.6	2311.3	22.0	2333.7	14.3	2333.7	14.3
-Spot 137	1707	875036	6.7	6.6543	0.6	7.8816	1.8	0.3804	1.7	0.95	2078.1	30.5	2217.6	16.2	2349.1	9.4	2349.1	9.4
-Spot 224	318	57481	1.8	6.5995	0.6	8.0120	2.1	0.3835	2.1	0.97	2092.6	37.0	2232.4	19.3	2363.2	9.6	2363.2	9.6
-Spot 306	388	117332	1.8	6.4788	0.6	7.5801	2.1	0.3562	2.0	0.96	1964.0	34.5	2182.5	19.0	2394.7	9.8	2394.7	9.8
-Spot 290	119	42924	1.6	6.4392	0.6	8.2535	3.1	0.3855	3.0	0.98	2101.7	53.7	2259.2	27.7	2405.1	10.7	2405.1	10.7
-Spot 210	374	106348	1.4	6.3513	0.4	9.0436	1.8	0.4166	1.8	0.97	2244.9	33.8	2342.4	16.8	2428.4	7.6	2428.4	7.6
-Spot 138	630	807896	2.0	6.3167	0.5	8.7963	2.2	0.4030	2.2	0.97	2182.8	40.2	2317.1	20.4	2437.7	8.8	2437.7	8.8
-Spot 260	294	62949	0.8	6.3119	0.6	8.7331	2.1	0.3998	2.0	0.96	2168.1	37.6	2310.5	19.5	2439.0	10.6	2439.0	10.6
-Spot 113	100	73209	0.9	6.2900	0.6	10.1364	2.5	0.4624	2.5	0.97	2450.2	50.4	2447.3	23.5	2444.9	9.9	2444.9	9.9
-Spot 105	435	141170	2.2	6.2016	0.6	9.1643	2.0	0.4122	1.9	0.96	2224.9	36.7	2354.5	18.6	2468.8	9.5	2468.8	9.5
-Spot 174	668	278564	4.2	6.1563	0.7	9.5569	2.1	0.4267	2.0	0.94	2290.9	38.8	2393.0	19.7	2481.2	12.3	2481.2	12.3
-Spot 252	158	111214	0.8	6.1356	0.8	8.1606	2.8	0.3631	2.6	0.96	1997.0	45.5	2249.0	24.9	2486.8	12.7	2486.8	12.7
-Spot 87	608	516383	7.4	6.0228	0.6	8.6033	2.0	0.3758	1.9	0.95	2056.7	32.9	2296.9	18.0	2518.0	10.9	2518.0	10.9
-Spot 227	297	87966	1.4	5.9273	0.4	11.2759	1.6	0.4847	1.6	0.96	2547.9	32.9	2546.2	15.1	2544.9	7.3	2544.9	7.3
-Spot 214	774	242300	2.1	5.5747	0.5	11.8991	1.7	0.4811	1.6	0.95	2532.1	33.2	2596.5	15.7	2647.1	8.9	2647.1	8.9
-Spot 221	109	282582	1.7	5.3945	0.6	12.8388	2.9	0.5023	2.9	0.98	2623.8	61.5	2667.9	27.5	2701.5	10.1	2701.5	10.1
-Spot 178	281	201738	1.2	3.9660	0.6	21.3565	2.3	0.6143	2.2	0.97	3087.2	54.2	3154.9	22.2	3198.3	9.4	3198.3	9.4

Table ______. U-Pb geochronologic analyses.																		
#2 U1450A_6F-8F						Isotope ratios						Apparent ages (Ma)						
Mid Pleistocene 0.4 Ma																		
Analysis	U	206Pb	U/Th	206Pb*	±	207Pb*	±	206Pb*	±	error	206Pb*	±	207Pb*	±	206Pb*	±	Best age	±
	(ppm)	204Pb		207Pb*	(%)	235U*	(%)	238U	(%)	corr.	238U*	(Ma)	235U	(Ma)	207Pb*	(Ma)	(Ma)	(Ma)
IDOP_1-Spot 297	363	8217	1.5	21.0607	5.2	0.0240	5.7	0.0037	2.4	0.41	23.6	0.6	24.1	1.4	73.5	124.2	23.6	0.6
IDOP_1-Spot 68	287	3469	49.2	22.9910	5.2	0.0225	5.7	0.0038	2.3	0.41	24.1	0.6	22.6	1.3	139.2	129.5	24.1	0.6
IDOP_1-Spot 229	773	37807	66.0	21.3326	3.3	0.0245	3.9	0.0038	2.0	0.51	24.4	0.5	24.6	0.9	42.9	79.3	24.4	0.5
IDOP_1-Spot 80	1801	45861	80.9	20.5115	2.6	0.0279	3.0	0.0041	1.5	0.51	26.7	0.4	27.9	0.8	136.0	61.1	26.7	0.4
IDOP_1-Spot 265	890	59932	0.5	20.4014	4.1	0.0281	4.8	0.0042	2.3	0.49	26.8	0.6	28.2	1.3	148.6	97.3	26.8	0.6
IDOP_1-Spot 156	890	17421	1.3	20.9864	3.5	0.0283	4.0	0.0043	2.1	0.51	27.7	0.6	28.4	1.1	81.9	82.3	27.7	0.6
IDOP_1-Spot 52	1301	57317	87.5	20.5525	2.5	0.0294	3.3	0.0044	2.1	0.63	28.2	0.6	29.4	0.9	131.3	59.4	28.2	0.6
IDOP_1-Spot 5	1909	290403	79.3	19.8156	2.9	0.0321	4.7	0.0046	3.8	0.79	29.7	1.1	32.1	1.5	216.5	66.6	29.7	1.1
IDOP_1-Spot 234	328	21939	1.3	21.6965	4.5	0.0294	5.2	0.0046	2.5	0.48	29.8	0.7	29.4	1.5	2.3	109.4	29.8	0.7
IDOP_1-Spot 280	815	159684	1.8	19.5176	2.6	0.0381	3.0	0.0054	1.6	0.53	34.7	0.6	38.0	1.1	251.5	58.7	34.7	0.6
IDOP_1-Spot 226	224	6407	2.7	21.1470	4.6	0.0416	4.9	0.0064	1.7	0.34	41.0	0.7	41.3	2.0	63.8	110.2	41.0	0.7
IDOP_1-Spot 220	2700	79468	2.0	20.6415	2.5	0.0441	3.3	0.0066	2.2	0.66	42.4	0.9	43.8	1.4	121.1	58.2	42.4	0.9
IDOP_1-Spot 187	735	34135	4.5	20.6242	3.0	0.0442	3.4	0.0066	1.5	0.45	42.5	0.6	43.9	1.5	123.1	71.7	42.5	0.6
IDOP_1-Spot 31	340	11286	1.4	21.6821	4.1	0.0450	4.6	0.0071	2.0	0.44	45.4	0.9	44.7	2.0	3.9	99.1	45.4	0.9
IDOP_1-Spot 263	987	31918	13.9	20.8936	2.7	0.0476	3.4	0.0072	1.9	0.58	46.3	0.9	47.2	1.5	92.5	65.0	46.3	0.9
IDOP_1-Spot 72	200	29384	1.7	21.0365	4.5	0.0496	5.2	0.0076	2.6	0.50	48.6	1.2	49.1	2.5	76.2	106.8	48.6	1.2
IDOP_1-Spot 58	1201	35025	1.1	21.1312	2.0	0.0498	2.5	0.0076	1.6	0.63	49.0	0.8	49.3	1.2	65.6	46.8	49.0	0.8
IDOP_1-Spot 218	347	9375	6.4	21.1048	4.0	0.0518	4.4	0.0079	1.7	0.38	50.9	0.8	51.3	2.2	68.6	95.9	50.9	0.8
IDOP_1-Spot 214	363	98547	2.4	20.8972	4.0	0.0552	4.8	0.0084	2.6	0.54	53.7	1.4	54.6	2.5	92.0	94.4	53.7	1.4
IDOP_1-Spot 116	64	2727	4.2	22.8442	7.1	0.0513	7.9	0.0085	3.6	0.45	54.5	2.0	50.8	3.9	123.3	174.8	54.5	2.0
IDOP_1-Spot 175	2311	59965	2.5	20.6157	2.1	0.0570	2.7	0.0085	1.8	0.64	54.7	1.0	56.3	1.5	124.0	49.4	54.7	1.0
IDOP_1-Spot 230	1082	62799	2.1	20.7487	2.0	0.0573	2.7	0.0086	1.7	0.65	55.4	0.9	56.6	1.5	108.9	47.8	55.4	0.9
IDOP_1-Spot 135	220	10915	1.9	21.2787	5.3	0.0573	5.9	0.0088	2.6	0.44	56.8	1.4	56.6	3.2	49.0	125.8	56.8	1.4
IDOP_1-Spot 22	653	17479	2.7	20.6886	3.2	0.0595	3.5	0.0089	1.6	0.45	57.3	0.9	58.7	2.0	115.8	74.9	57.3	0.9
IDOP_1-Spot 86	3515	58633	4.5	19.2685	2.0	0.0685	2.6	0.0096	1.7	0.66	61.4	1.0	67.3	1.7	281.0	45.0	61.4	1.0
IDOP_1-Spot 272	946	51093	1.6	20.1937	2.2	0.0679	2.6	0.0099	1.4	0.54	63.8	0.9	66.7	1.7	172.5	51.8	63.8	0.9
IDOP_1-Spot 292	2305	193646	27.8	21.0175	1.7	0.0670	2.5	0.0102	1.8	0.71	65.5	1.1	65.8	1.6	78.4	41.3	65.5	1.1
IDOP_1-Spot 222	214	19954	0.5	22.2720	4.6	0.0682	4.9	0.0110	1.7	0.35	70.6	1.2	67.0	3.2	61.1	111.2	70.6	1.2
IDOP_1-Spot 271	163	8760	2.4	21.3161	4.5	0.0736	4.9	0.0114	1.8	0.37	73.0	1.3	72.1	3.4	44.8	108.7	73.0	1.3
IDOP_1-Spot 138	30	2470	1.7	20.1718	9.7	0.0831	10.2	0.0122	3.2	0.31	77.9	2.5	81.0	8.0	175.1	227.7	77.9	2.5
IDOP_1-Spot 236	3314	73577	15.3	17.5170	1.1	0.0984	2.5	0.0125	2.2	0.90	80.1	1.8	95.3	2.3	494.9	23.9	80.1	1.8
IDOP_1-Spot 18	802	73545	18.7	20.4181	1.4	0.0871	2.2	0.0129	1.7	0.79	82.7	1.4	84.8	1.8	146.7	32.1	82.7	1.4
IDOP_1-Spot 90	484	35159	17.4	20.7163	2.6	0.0886	3.1	0.0133	1.6	0.51	85.3	1.3	86.2	2.5	112.6	62.0	85.3	1.3
IDOP_1-Spot 4	178	14922	1.2	20.8341	3.7	0.0913	4.1	0.0138	1.6	0.40	88.3	1.4	88.7	3.5	99.2	88.0	88.3	1.4
IDOP_1-Spot 23	94	24251	1.2	20.7105	3.7	0.0964	4.2	0.0145	2.0	0.48	92.7	1.9	93.5	3.8	113.3	87.4	92.7	1.9
IDOP_1-Spot 125	252	58555	2.3	20.8217	3.4	0.1018	4.0	0.0154	2.1	0.52	98.3	2.0	98.4	3.7	100.6	80.7	98.3	2.0
IDOP_1-Spot 231	5682	176854	2.6	19.9042	1.1	0.1108	2.4	0.0160	2.1	0.89	102.3	2.2	106.7	2.4	206.1	24.6	102.3	2.2
IDOP_1-Spot 264	93	37891	2.4	20.7216	5.0	0.1097	5.3	0.0165	1.7	0.33	105.4	1.8	105.7	5.3	112.0	118.8	105.4	1.8
IDOP_1-Spot 114	283	40146	1.3	20.2691	3.2	0.1161	4.4	0.0171	3.1	0.70	109.1	3.3	111.5	4.7	163.8	74.4	109.1	3.3
IDOP_1-Spot 120	204	17205	1.3	20.5008	3.7	0.1171	4.2	0.0174	1.9	0.45	111.2	2.1	112.4	4.4	137.2	87.1	111.2	2.1
IDOP_1-Spot 242	331	57793	109.7	19.4445	2.4	0.1235	3.2	0.0174	2.1	0.66	111.3	2.3	118.3	3.6	260.1	55.1	111.3	2.3
IDOP_1-Spot 30	151	19744	1.2	20.0605	3.6	0.1204	3.9	0.0175	1.6	0.41	111.9	1.8	115.4	4.3	187.9	83.3	111.9	1.8
IDOP_1-Spot 44	170	19840	1.5	20.3230	3.7	0.1189	4.1	0.0175	1.6	0.41	112.0	1.8	114.1	4.4	157.6	86.7	112.0	1.8
IDOP_1-Spot 312	102	9278	2.8	20.8453	4.1	0.1160	4.5	0.0175	1.9	0.41	112.1	2.1	111.5	4.8	97.9	97.2	112.1	2.1
IDOP_1-Spot 186	1233	1839411	0.9	20.4977	1.7	0.1186	2.7	0.0176	2.1	0.77	112.6	2.3	113.8	2.9	137.6	39.9	112.6	2.3
IDOP_1-Spot 246	2122	70493	3.4	20.6966	1.4	0.1181	2.4	0.0177	2.0	0.80	113.3	2.2	113.3	2.6	114.8	34.2	113.3	2.2
IDOP_1-Spot 309	694	149365	3.0	20.6834	2.1	0.1188	2.7	0.0178	1.7	0.62	113.9	1.9	114.0	2.9	116.3	49.8	113.9	1.9
IDOP_1-Spot 170	1540	109158	3.3	20.3258	1.4	0.1215	2.1	0.0179	1.6	0.75	114.5	1.8	116.5	2.3	157.3	32.4	114.5	1.8
IDOP_1-Spot 267	123	12936	1.8	21.8596	4.8	0.1151	5.2	0.0183	2.0	0.38	116.6	2.3	110.7	5.5	15.7	116.4	116.6	2.3
IDOP_1-Spot 161	259	36279	1.6	20.4059	2.8	0.1238	3.3	0.0183	1.8	0.55	117.0	2.1	118.5	3.7	148.1	65.6	117.0	2.1
IDOP_1-Spot 173	486	47930	1.7	20.1324	2.1	0.1257	2.7	0.0184	1.7	0.62	117.3	2.0	120.3	3.1	179.6	49.5	117.3	2.0
IDOP_1-Spot 154	1308	75655	0.6	20.4459	1.5	0.1248	1.9	0.0185	1.1	0.59	118.3	1.3	119.5	2.1	143.5	36.0	118.3	1.3
IDOP_1-Spot 127	511	38423	2.6	20.5735	2.3	0.1246	2.8	0.0186	1.7	0.60	118.7	2.0	119.2	3.2	128.9	53.0	118.7	2.0
IDOP_1-Spot 108	651	42599	3.7	20.9978	2.2	0.1230	2.9	0.0187	1.9	0.67	119.7	2.3	117.8	3.2	80.6	51.1	119.7	2.3
IDOP_1-Spot 109	865	83066	9.7	20.4200	1.5	0.1297	2.5	0.0192	2.1	0.81	122.7	2.5	123.9	3.0	146.5	34.8	122.7	2.5
IDOP_1-Spot 286	536	40560	1.8	18.2903	2.5	0.1449	3.1	0.0192	1.9	0.60	122.7	2.3	137.4	4.0	398.9	56.5	122.7	2.3
IDOP_1-Spot 294	577	45492	1.9	20.3315	1.8	0.1331	2.5	0.0196	1.7	0.70	125.3	2.2	126.9	3.0	156.7	41.8	125.3	2.2
IDOP_1-Spot 207	246	22879	0.9	20.5892	2.2	0.1325	2.6	0.0198	1.4	0.55	126.3	1.8	126.4	3.1	127.1	51.3	126.3	1.8
IDOP_1-Spot 250	400	184662	2.0	19.9009	1.9	0.1374	2.4	0.0198	1.6	0.63	126.6	1.9	130.7	3.0	206.6	44.0	126.6	1.9
IDOP_1-Spot 287	316	62786	1.3	19.5735	2.0	0.1409	2.5	0.0200	1.5	0.60	127.6	1.9	133.8	3.2	244.9	46.8	127.6	1.9
IDOP_1-Spot 115	179	23657	1.1	19.6153	3.5	0.1413	4.1	0.0201	2.0	0.48	128.3	2.5	134.2	5.1	239.9	81.8	128.3	2.5
IDOP_1-Spot 34	558	74945	1.0	20.3391	1.8	0.1417	2.4	0.0209	1.6	0.65	133.4	2.1	134.6	3.1	155.8	43.1	133.4	2.1
IDOP_1-Spot 10	270	147427	2.2	18.7963	2.3	0.1561	2.8	0.0213	1.7	0.59	135.8	2.2	147.3	3.9	337.4	52.0	135.8	2.2
IDOP_1-Spot 206	425	46609	1.9	20.1248	2.5	0.1461	3.0	0.0213	1.5	0.52	136.0	2.1	138.5	3.8	180.5	59.1	136.0	2.1
IDOP_1-Spot 237	117	25238	1.5	19.5330	4.2	0.1515	4.6	0.0215	1.9	0.42	136.9	2.6	143.2	6.1	249.6	95.8	136.9	2.6
IDOP_1-Spot 121	103	5938	1.7	19.8529	4.0	0.1505	4.3	0.0217	1.6	0.38	138.2	2.2	142.3	5.7	212.1	92.0	138.2	2.2
IDOP_1-Spot 93	75	6355	5.9	20.2056	4.2	0.1534	4.6	0.0225	1.9	0.41	143.3	2.7	144.9	6.2	171.2	98.6	143.3	2.7
IDOP_1-Spot 308	214	48507	1.4	18.9916	2.6	0.1865	3.3	0.0257	2.0	0.61	163.5	3.2	173.6	5.2	313.9	59.2	163.5	3.2
IDOP_1-Spot 146	756	77783	24.6	17.4437	3.2	0.2150	8.2	0.0272	7.5	0.92	173.0	12.9	197.7	14.8	504.1	71.5	173.0	12.9
IDOP_1-Spot 165	2041	141793	29.9	19.8306	1.4	0.1939	2.3	0.0279	1.8	0.78	177.3	3.1	179.9	3.8	214.7	33.0	177.3	3.1
IDOP_1-Spot 306	1107	106702	25.9	19.7938	1.7	0.1966	2.8	0.0282	2.3	0.79	179.4	4.0	182.3	4.7	219.0	40.0	179.4	4.0
IDOP_1-Spot 48	255	30071	1.2	19.4933	2.5	0.2086	3.0	0.0295	1.6	0.53	187.4	2.9	192.4	5.2	254.3	58.3	187.4	2.9
IDOP_1-Spot 1	2550	168533	3.0	19.7110	1.3	0.2164	2.8	0.0309	2.5	0.89	196.4	4.9	198.9	5.1	228.7	29.5	196.4	4.9
IDOP_1-Spot 293	343	34741	10.8	19.7095	2.1	0.2227	2.7	0.0318	1.7	0.64	202.1	3.4	204.2	5.0	228.9	48.4	202.1	3.4
IDOP_1-Spot 315	1783	66246	2.0	19.7322	1.3	0.2258	2.9	0.0323	2.6	0.89	205.0	5.3	206.7	5.5	226.2	30.5	205.0	5.3
IDOP_1-Spot 159	399	36722	2.9	19.6651	1.8	0.2370	2.5	0.0338	1.7	0.70	214.3	3.7	216.0	4.8	234.1	41.1	214.3	3.7
IDOP_1-Spot 143	802	223437	27.0	19.2955	1.8	0.2444	2.5	0.0342	1.7	0.70	216.8	3.7	222.0	5.0	277.7	40.5	216.8	3.7
IDOP_1-Spot 203	218	41054	0.7	19.2364	2.4	0.2647	2.8	0.0369	1.5	0.53	233.8	3.4	238.5	5.9	284.7	54.1	233.8	3.4
IDOP_1-Spot 35	258	66508	1.6	19.8092	1.8	0.2763	2.3	0.0397	1.5	0.64	250.9	3.6	247.7	5.1	217.2	41.3	250.9	3.6

IDOP_1-Spot 7	4403	3761471	4.3	17.2608	1.0	0.5075	2.2	0.0635	2.0	0.89	397.1	7.6	416.8	7.5	527.3	22.0	397.1	7.6
IDOP_1-Spot 302	85	46752	1.0	17.8235	2.8	0.5131	3.4	0.0663	2.0	0.57	414.0	7.9	420.5	11.8	456.5	62.6	414.0	7.9
IDOP_1-Spot 98	5600	716030	4.7	17.3080	1.2	0.5432	2.6	0.0682	2.4	0.89	425.2	9.7	440.5	9.4	521.3	26.0	425.2	9.7
IDOP_1-Spot 179	505	165182	2.9	17.5992	1.3	0.5346	2.5	0.0682	2.1	0.84	425.5	8.7	434.9	8.9	484.6	29.7	425.5	8.7
IDOP_1-Spot 79	1826	85499	4.9	17.3040	0.9	0.5735	2.4	0.0720	2.2	0.92	448.0	9.6	460.2	8.9	521.8	20.5	448.0	9.6
IDOP_1-Spot 152	1998	66579	17.1	17.4056	1.2	0.5836	2.2	0.0737	1.8	0.84	458.3	8.1	466.8	8.1	509.0	26.2	458.3	8.1
IDOP_1-Spot 183	2516	529665	51.3	17.4588	0.8	0.5916	1.9	0.0749	1.7	0.89	465.7	7.5	471.9	7.0	502.2	18.4	465.7	7.5
IDOP_1-Spot 171	375	59544	3.8	17.4610	1.5	0.5970	3.1	0.0756	2.8	0.88	469.8	12.6	475.3	11.9	502.0	32.7	469.8	12.6
IDOP_1-Spot 131	188	31691	2.5	17.7436	1.3	0.5933	2.0	0.0764	1.5	0.77	474.3	7.1	473.0	7.6	466.5	28.8	474.3	7.1
IDOP_1-Spot 185	442	1371385	3.5	17.3753	1.2	0.6068	2.2	0.0765	1.9	0.84	475.0	8.5	481.6	8.5	512.8	26.7	475.0	8.5
IDOP_1-Spot 15	362	61178	1.2	17.5572	1.3	0.6043	2.2	0.0769	1.8	0.81	477.9	8.2	480.0	8.4	489.9	28.0	477.9	8.2
IDOP_1-Spot 92	364	132225	4.1	17.0550	1.6	0.6228	2.6	0.0770	2.0	0.77	478.4	9.1	491.6	10.0	553.5	35.6	478.4	9.1
IDOP_1-Spot 314	120	36062	1.1	16.8747	2.3	0.6357	3.5	0.0778	2.6	0.75	483.0	12.2	499.7	13.9	576.6	50.6	483.0	12.2
IDOP_1-Spot 249	150	116179	1.0	17.1050	2.1	0.6279	3.0	0.0779	2.2	0.73	483.5	10.1	494.8	11.7	547.1	44.8	483.5	10.1
IDOP_1-Spot 78	306	175731	1.3	17.3885	1.5	0.6184	2.2	0.0780	1.7	0.75	484.1	7.7	488.8	8.6	511.1	32.3	484.1	7.7
IDOP_1-Spot 102	155	55294	1.0	17.1626	1.8	0.6270	2.6	0.0780	1.9	0.72	484.4	8.9	494.2	10.4	539.8	40.3	484.4	8.9
IDOP_1-Spot 177	673	153283	2.9	17.3446	0.9	0.6246	1.7	0.0786	1.5	0.85	487.6	6.8	492.8	6.7	516.7	19.6	487.6	6.8
IDOP_1-Spot 17	340	75012	1.5	17.5235	1.6	0.6230	2.3	0.0792	1.6	0.70	491.2	7.4	491.7	8.8	494.1	35.6	491.2	7.4
IDOP_1-Spot 169	627	116704	3.8	17.6257	1.4	0.6226	2.4	0.0796	2.0	0.81	493.7	9.3	491.5	9.4	481.2	31.3	493.7	9.3
IDOP_1-Spot 97	586	129025	0.8	17.1187	1.1	0.6435	1.8	0.0799	1.4	0.80	495.5	6.9	504.5	7.2	545.4	23.7	495.5	6.9
IDOP_1-Spot 37	632	133813	3.6	17.2517	1.0	0.6409	2.0	0.0802	1.7	0.85	497.3	8.0	502.9	7.8	528.5	22.7	497.3	8.0
IDOP_1-Spot 54	365	68544	2.0	16.8291	1.6	0.6573	2.5	0.0802	1.8	0.74	497.5	8.7	513.0	9.9	582.6	35.6	497.5	8.7
IDOP_1-Spot 193	123	27609	1.7	17.4940	1.8	0.6330	2.7	0.0803	2.0	0.73	498.0	9.4	498.0	10.6	497.8	40.5	498.0	9.4
IDOP_1-Spot 221	259	91634	1.9	17.5147	1.7	0.6334	2.3	0.0805	1.6	0.69	498.9	7.6	498.2	9.0	495.2	36.5	498.9	7.6
IDOP_1-Spot 76	447	213753	1.1	17.1634	1.4	0.6470	2.0	0.0805	1.4	0.72	499.4	6.8	506.7	7.9	539.7	30.2	499.4	6.8
IDOP_1-Spot 261	206	67443	1.8	17.2531	1.7	0.6450	2.5	0.0807	1.8	0.74	500.3	8.8	505.4	9.9	528.3	36.7	500.3	8.8
IDOP_1-Spot 16	89	1422527	0.5	16.7411	2.1	0.6679	2.9	0.0811	2.0	0.70	502.7	9.9	519.5	11.9	593.9	45.7	502.7	9.9
IDOP_1-Spot 168	548	81819	1.8	17.1905	1.2	0.6505	2.0	0.0811	1.6	0.81	502.7	8.0	508.8	8.1	536.3	26.0	502.7	8.0
IDOP_1-Spot 81	596	244978	2.6	17.0317	1.1	0.6570	1.9	0.0812	1.6	0.81	503.0	7.6	512.8	7.8	556.5	25.1	503.0	7.6
IDOP_1-Spot 106	89	35608	1.7	16.7994	2.3	0.6684	3.0	0.0814	1.9	0.63	504.7	9.2	519.7	12.3	586.4	51.0	504.7	9.2
IDOP_1-Spot 282	944	54502	73.0	17.4347	1.3	0.6446	1.9	0.0815	1.4	0.74	505.1	6.9	505.2	7.6	505.3	28.3	505.1	6.9
IDOP_1-Spot 190	289	127096	4.0	17.0737	1.7	0.6613	2.2	0.0819	1.4	0.66	507.4	7.1	515.4	8.9	551.2	36.3	507.4	7.1
IDOP_1-Spot 57	675	57149	6.3	17.4536	1.3	0.6505	2.1	0.0823	1.7	0.81	510.1	8.4	508.8	8.5	502.9	27.7	510.1	8.4
IDOP_1-Spot 59	557	94497	1.3	17.1372	1.2	0.6635	2.3	0.0825	2.0	0.85	510.8	9.7	516.8	9.4	543.0	26.7	510.8	9.7
IDOP_1-Spot 67	98	26702	0.8	17.2785	2.6	0.6602	3.3	0.0827	2.0	0.60	512.4	9.8	514.7	13.3	525.0	57.5	512.4	9.8
IDOP_1-Spot 49	359	58874	1.8	17.3864	1.3	0.6603	2.3	0.0833	1.9	0.81	515.5	9.2	514.8	9.3	511.4	29.4	515.5	9.2
IDOP_1-Spot 160	419	188477	1.4	17.2492	1.2	0.6662	2.2	0.0833	1.8	0.82	516.1	8.9	518.4	8.9	528.8	27.3	516.1	8.9
IDOP_1-Spot 274	407	159493	2.4	16.7774	1.4	0.7023	2.1	0.0855	1.6	0.74	528.6	7.9	540.2	8.9	589.2	31.3	528.6	7.9
IDOP_1-Spot 248	1593	780787	61.2	16.9146	0.8	0.7085	1.9	0.0869	1.8	0.91	537.3	9.1	543.9	8.2	571.5	17.5	537.3	9.1
IDOP_1-Spot 12	821	188584	6.5	16.8981	1.1	0.7157	2.1	0.0877	1.8	0.85	542.0	9.3	548.1	8.9	573.7	24.1	542.0	9.3
IDOP_1-Spot 235	325	45129	3.0	16.9850	1.4	0.7400	2.3	0.0912	1.9	0.81	562.4	10.0	562.4	10.0	562.5	29.7	562.4	10.0
IDOP_1-Spot 172	618	7311852	0.9	16.7561	1.4	0.7817	2.3	0.0950	1.8	0.80	585.0	10.1	586.5	10.1	592.0	29.7	585.0	10.1
IDOP_1-Spot 50	866	203400	1.2	16.5836	1.3	0.7916	2.2	0.0952	1.8	0.81	586.3	10.1	592.1	10.0	614.4	28.4	586.3	10.1
IDOP_1-Spot 196	101	42551	131.1	16.7126	2.5	0.7888	3.5	0.0956	2.4	0.70	588.6	13.7	590.5	15.5	597.6	53.3	588.6	13.7
IDOP_1-Spot 247	255	62701	6.7	16.6565	1.5	0.8007	2.3	0.0967	1.7	0.75	595.2	9.8	597.2	10.3	604.9	32.4	595.2	9.8
IDOP_1-Spot 181	281	186357	1.4	16.1472	1.4	0.8850	2.0	0.1036	1.4	0.71	635.7	8.7	643.7	9.6	671.7	30.6	635.7	8.7
IDOP_1-Spot 14	328	143848	22.1	15.9501	1.3	0.9437	2.0	0.1092	1.6	0.78	667.9	10.1	674.8	10.1	697.9	27.2	667.9	10.1
IDOP_1-Spot 6	908	60063	0.5	15.4370	1.2	1.0117	2.7	0.1133	2.4	0.89	691.7	15.5	709.7	13.6	767.2	26.0	691.7	15.5
IDOP_1-Spot 228	194	41491	1.8	15.7615	1.4	1.0065	2.1	0.1151	1.6	0.75	702.1	10.6	707.1	10.8	723.2	29.7	702.1	10.6
IDOP_1-Spot 96	105	28545	0.8	15.3800	1.9	1.0957	2.6	0.1222	1.8	0.69	743.3	12.9	751.2	14.0	774.9	40.1	743.3	12.9
IDOP_1-Spot 283	321	122229	7.9	14.5657	1.3	1.1658	2.0	0.1232	1.6	0.78	748.7	11.2	784.7	11.1	888.4	26.2	748.7	11.2
IDOP_1-Spot 162	196	148338	1.3	14.8314	1.4	1.1829	2.2	0.1272	1.7	0.77	772.1	12.3	792.6	12.1	850.9	29.2	772.1	12.3
IDOP_1-Spot 85	794	63329	7.6	14.9914	1.1	1.1728	2.0	0.1275	1.7	0.83	773.7	12.2	788.0	11.1	828.6	23.8	773.7	12.2
IDOP_1-Spot 210	224	85320	0.7	15.0866	1.3	1.1733	2.0	0.1284	1.6	0.78	778.7	11.5	788.2	11.1	815.4	26.5	778.7	11.5
IDOP_1-Spot 239	122	35784	0.7	15.1557	1.6	1.1719	2.4	0.1288	1.8	0.74	781.1	12.9	787.5	12.9	805.8	33.0	781.1	12.9
IDOP_1-Spot 313	129	53372	1.5	14.3167	2.0	1.2524	4.2	0.1300	3.7	0.88	788.1	27.2	824.5	23.5	923.9	40.2	788.1	27.2
IDOP_1-Spot 20	235	149357	3.2	14.3853	1.0	1.2486	1.9	0.1303	1.5	0.83	789.4	11.5	822.8	10.5	914.1	21.5	789.4	11.5
IDOP_1-Spot 298	653	255448	1.2	14.7336	1.3	1.2325	2.3	0.1317	1.9	0.84	797.6	14.5	815.5	12.9	864.7	26.3	797.6	14.5
IDOP_1-Spot 8	178	67330	1.2	14.4832	1.4	1.2573	2.7	0.1321	2.3	0.86	799.6	17.3	826.7	15.1	900.1	27.9	799.6	17.3
IDOP_1-Spot 310	35	23366	1.4	14.9470	2.5	1.2625	3.2	0.1369	2.0	0.63	826.9	15.8	829.0	18.4	834.8	52.7	826.9	15.8
IDOP_1-Spot 75	453	106018	2.3	14.8939	1.1	1.2825	2.2	0.1385	2.0	0.87	836.4	15.3	838.0	12.8	842.2	23.2	836.4	15.3
IDOP_1-Spot 223	299	160095	1.1	14.8215	1.3	1.2925	2.3	0.1389	1.9	0.84	838.7	15.1	842.4	13.2	852.3	26.2	838.7	15.1
IDOP_1-Spot 259	212	64742	1.9	14.6843	1.5	1.3270	2.4	0.1413	1.9	0.78	852.2	15.0	857.6	13.9	871.6	30.8	852.2	15.0
IDOP_1-Spot 216	190	52398	1.4	14.5913	1.2	1.3802	1.7	0.1461	1.2	0.68	878.8	9.5	880.5	9.9	884.7	25.5	878.8	9.5
IDOP_1-Spot 38	68	88588	0.6	14.4448	1.6	1.4492	2.1	0.1518	1.3	0.64	911.2	11.3	909.5	12.4	905.6	32.8	905.6	32.8
IDOP_1-Spot 41	35	75040	2.0	14.3102	2.5	1.4859	3.0	0.1542	1.7	0.56	924.6	14.6	924.7	18.2	924.8	51.0	924.8	51.0
IDOP_1-Spot 305	146	85764	1.8	14.0567	1.3	1.5719	1.9	0.1603	1.3	0.72	958.2	11.8	959.2	11.5	961.5	26.3	961.5	26.3
IDOP_1-Spot 205	366	96907	2.8	13.9704	1.2	1.5737	2.2	0.1595	1.9	0.84	953.7	16.4	959.9	13.8	974.0	24.8	974.0	24.8
IDOP_1-Spot 199	241	151120	2.9	13.9346	1.0	1.5871	2.1	0.1604	1.9	0.87	959.0	16.6	965.1	13.3	979.2	21.0	979.2	21.0
IDOP_1-Spot 194	120	299214	1.3	13.8493	1.5	1.4450	2.3	0.1451	1.7	0.73	873.7	13.5	907.8	13.6	991.7	31.3	991.7	31.3
IDOP_1-Spot 277	1387	143060	9.5	13.8245	1.0	1.3953	1.9	0.1399	1.6	0.85	844.1	12.8	886.9	11.2	995.4	20.0	995.4	20.0
IDOP_1-Spot 11	414	752103	1.5	13.7980	1.2	1.5092	2.4	0.1510	2.0	0.86	906.7	17.2	934.1	14.5	999.3	25.0	999.3	25.0
IDOP_1-Spot 147	672	273902	4.2	13.7911	1.2	1.4866	2.4	0.1487	2.1	0.88	893.6	17.6	924.9	14.6	1000.3	23.4	1000.3	23.4
IDOP_1-Spot 227	315	465475	2.4	13.7840	1.3	1.6248	2.0	0.1624	1.5	0.75	970.3	13.4	979.9	12.5	1001.3	26.7	1001.3	26.7
IDOP_1-Spot 219	700	205611	1.8	13.7823	1.4	1.6556	2.3	0.1655	1.8	0.79	987.2	16.5	991.7	14.5	1001.6	28.8	1001.6	28.8
IDOP_1-Spot 82	140	66848	1.4	13.7739	1.6	1.7416	2.3	0.1740	1.7	0.73	1034.0	16.0	1024.1	14.8	1002.8	31.6	1002.8	31.6
IDOP_1-Spot 107	1103	334499	3.3	13.7592	1.1	1.5029	2.4	0.1500	2.1	0.88	900.8	17.9	931.6	14.7	1005.0	23.1	1005.0	23.1
IDOP_1-Spot 122	110	66151	1.8	13.7464	1.6	1.6301	2.2	0.1625	1.6	0.70	970.7	14.1	981.9	14.1	1006.9	32.5	1006.9	32.5
IDOP_1-Spot 137	353	215607	3.1	13.7420	1.0	1.6200	1.7	0.1615	1.4	0.81	964.9	12.3	978.0	10.6	1007.5	19.9	1007.5	19.9
IDOP_1-Spot 91	245	83670	1.6	13.7325	1.3	1.6291	1.9	0.1623	1.4	0.73	969.3	12.8	981.5	12.2	1008.9	26.6	1008.9	26.6
IDOP_1-Spot 176	95	170977	1.7	13.7172	1.6	1.6499	2.4	0.1641	1.7	0.73	979.8	15.6	989.5	14.9	1011.2	32.8	1011.2	32.8
IDOP_1-Spot 136	1650	120362	67.8	13.5385	1.0	1.6274	2.0	0.1598	1.8	0.88	955.6	16.0	980.8	12.9	1037.7	19.7	1037.7	19.7
IDOP_1-Spot 141	1252	168320	1.2	13.4702	1.2	1.4965	1.9	0.1462	1.5	0.78	879.6	12.2	929.0	11.5	1048.0	23.7	1048.0	23.7
IDOP_1-Spot 255	282	134501	1.1	13.4683	1.1	1.7384	1.9	0.1698	1.5	0.80	1011.1	14.1	1022.9	12.1	1048.2	22.8	1048.2	22.8
IDOP_1-Spot 19	521	71620	8.0	13.4092	1.2	1.5329	2.1	0.1491	1.8	0.83	895.8	14.7	943.7	13.0	1057.1	23.5	1057.1	23.5
IDOP_1-Spot 163	1071	669382	1.1	13.3926	0.8	1.7823	1.8	0.1731	1.6	0.90	1029.3	15.4	1039.0	11.7	1059.6	15.7	1059.6	15.7

IDOP_1-Spot 300	618	172681	3.5	13.3062	1.1	1.8320	2.1	0.1768	1.8	0.85	1049.4	17.6	1057.0	14.0	1072.6	22.6	1072.6	22.6
IDOP_1-Spot 180	198	153518	1.6	13.2999	1.2	1.8549	2.1	0.1789	1.7	0.83	1061.1	16.9	1065.2	13.8	1073.6	23.4	1073.6	23.4
IDOP_1-Spot 273	261	194650	2.6	13.2924	1.0	1.8347	2.0	0.1769	1.8	0.87	1049.9	17.2	1057.9	13.4	1074.7	20.0	1074.7	20.0
IDOP_1-Spot 100	242	86148	4.1	13.2286	1.3	1.6710	2.4	0.1603	2.0	0.84	958.6	17.8	997.6	15.2	1084.4	26.2	1084.4	26.2
IDOP_1-Spot 70	475	22688784	0.6	13.2282	1.1	1.7301	2.0	0.1660	1.6	0.82	990.0	15.1	1019.8	12.9	1084.4	22.9	1084.4	22.9
IDOP_1-Spot 254	127	165456	0.9	13.1252	1.0	1.8691	1.9	0.1779	1.7	0.85	1055.6	16.1	1070.2	12.9	1100.1	20.4	1100.1	20.4
IDOP_1-Spot 270	448	55586	8.3	13.1087	0.9	1.9765	1.9	0.1879	1.7	0.89	1110.1	17.5	1107.5	13.0	1102.6	17.3	1102.6	17.3
IDOP_1-Spot 55	421	53626	8.5	13.0848	1.3	1.9364	1.9	0.1838	1.5	0.75	1087.5	14.6	1093.7	13.0	1106.2	25.8	1106.2	25.8
IDOP_1-Spot 130	402	1310792	6.1	13.0507	1.1	2.0397	2.2	0.1931	1.9	0.86	1137.9	19.5	1128.9	14.8	1111.4	22.0	1111.4	22.0
IDOP_1-Spot 126	336	134294	2.5	12.9612	1.2	2.0634	1.9	0.1940	1.6	0.80	1142.8	16.3	1136.7	13.3	1125.1	23.1	1125.1	23.1
IDOP_1-Spot 238	543	186610	21.6	12.9606	1.2	1.6864	2.5	0.1585	2.2	0.87	948.5	19.1	1003.4	15.8	1125.2	24.1	1125.2	24.1
IDOP_1-Spot 182	324	157097	4.6	12.9416	1.4	1.8359	3.4	0.1723	3.1	0.91	1024.9	29.1	1058.4	22.1	1128.2	27.1	1128.2	27.1
IDOP_1-Spot 150	522	50380	1.7	12.8809	1.0	1.9678	1.8	0.1838	1.4	0.82	1087.9	14.4	1104.5	11.8	1137.5	20.2	1137.5	20.2
IDOP_1-Spot 164	339	84526	2.1	12.8277	1.1	1.9139	2.0	0.1781	1.7	0.84	1056.4	16.4	1086.0	13.3	1145.8	21.4	1145.8	21.4
IDOP_1-Spot 45	194	142325	1.3	12.8042	1.4	2.1560	3.8	0.2002	3.5	0.93	1176.5	38.0	1167.0	26.5	1149.4	28.7	1149.4	28.7
IDOP_1-Spot 197	120	90879	1.4	12.7930	1.2	2.0928	1.9	0.1942	1.5	0.78	1144.0	15.8	1146.4	13.2	1151.1	23.8	1151.1	23.8
IDOP_1-Spot 69	50	33071	1.2	12.6835	2.4	1.9498	4.5	0.1794	3.8	0.85	1063.5	37.3	1098.4	30.0	1168.2	46.7	1168.2	46.7
IDOP_1-Spot 208	279	143561	1.9	12.6363	1.2	1.7816	2.2	0.1633	1.9	0.84	975.0	17.0	1038.8	14.5	1175.6	23.5	1175.6	23.5
IDOP_1-Spot 258	247	188814	0.5	12.6256	1.1	2.1569	2.0	0.1975	1.7	0.83	1161.9	17.7	1167.3	13.8	1177.3	21.8	1177.3	21.8
IDOP_1-Spot 62	65	91360	0.7	12.6234	1.3	2.1029	2.0	0.1925	1.6	0.78	1135.0	16.4	1149.8	13.9	1177.6	25.2	1177.6	25.2
IDOP_1-Spot 104	121	63449	2.3	12.5935	1.3	2.1097	2.0	0.1927	1.6	0.78	1135.9	16.5	1152.0	14.0	1182.3	25.4	1182.3	25.4
IDOP_1-Spot 73	4836	1437568	9.9	12.5897	1.1	1.9716	2.5	0.1800	2.2	0.90	1067.1	21.9	1105.9	16.7	1182.9	21.6	1182.9	21.6
IDOP_1-Spot 232	140	143934	1.9	12.5847	1.6	2.0602	2.2	0.1880	1.5	0.67	1110.7	15.2	1135.7	15.2	1183.7	32.6	1183.7	32.6
IDOP_1-Spot 240	841	246307	24.7	12.5847	0.9	1.8477	1.9	0.1686	1.6	0.87	1004.6	15.1	1062.6	12.3	1183.7	18.5	1183.7	18.5
IDOP_1-Spot 260	128	105462	1.0	12.5807	1.8	2.1375	2.4	0.1950	1.6	0.68	1148.6	17.2	1161.0	16.6	1184.3	34.7	1184.3	34.7
IDOP_1-Spot 13	317	140755	2.0	12.4944	1.1	2.2660	2.1	0.2053	1.7	0.85	1204.0	19.2	1201.8	14.6	1197.9	21.8	1197.9	21.8
IDOP_1-Spot 25	92	96565	1.6	12.4785	1.2	2.1732	2.1	0.1967	1.7	0.81	1157.5	18.2	1172.5	14.7	1200.4	24.2	1200.4	24.2
IDOP_1-Spot 291	86	95750	0.6	12.4575	1.5	1.9266	2.1	0.1741	1.5	0.72	1034.5	14.6	1090.4	14.2	1203.7	29.0	1203.7	29.0
IDOP_1-Spot 225	57	108565	0.7	12.4364	1.9	2.1783	2.6	0.1965	1.7	0.66	1156.4	17.8	1174.1	17.8	1207.1	37.9	1207.1	37.9
IDOP_1-Spot 36	299	130047	18.9	12.3958	1.1	2.3467	1.7	0.2110	1.3	0.75	1234.0	14.4	1226.5	12.2	1213.5	22.1	1213.5	22.1
IDOP_1-Spot 192	37	43454	0.4	12.3285	2.3	2.1942	3.2	0.1962	2.2	0.68	1154.8	22.9	1179.2	22.2	1224.2	45.8	1224.2	45.8
IDOP_1-Spot 2	136	61899	1.6	12.3244	1.3	2.1956	1.9	0.1963	1.4	0.71	1155.2	14.3	1179.7	13.3	1224.8	26.1	1224.8	26.1
IDOP_1-Spot 281	648	129857	4.8	12.1462	1.2	2.2908	2.5	0.2018	2.2	0.87	1185.0	23.4	1209.4	17.6	1253.4	24.1	1253.4	24.1
IDOP_1-Spot 99	478	203373	1.0	12.0195	1.2	2.5492	2.2	0.2222	1.8	0.85	1293.6	21.7	1286.2	15.9	1273.9	22.4	1273.9	22.4
IDOP_1-Spot 155	432	1039719	11.6	11.8113	1.3	2.1217	2.5	0.1818	2.1	0.85	1076.6	21.2	1155.9	17.4	1307.8	25.7	1307.8	25.7
IDOP_1-Spot 200	40	59042	1.2	11.7546	1.6	2.7053	2.5	0.2306	1.9	0.77	1337.8	23.3	1329.9	18.6	1317.2	31.1	1317.2	31.1
IDOP_1-Spot 153	687	118757	4.3	11.6103	1.2	2.4522	2.4	0.2065	2.1	0.87	1210.1	23.1	1258.1	17.3	1341.1	22.4	1341.1	22.4
IDOP_1-Spot 124	502	151755	8.2	11.6094	1.0	2.7063	1.7	0.2279	1.4	0.82	1323.3	16.7	1330.2	12.7	1341.2	19.1	1341.2	19.1
IDOP_1-Spot 24	594	53926	2.1	11.5979	0.8	2.8096	1.5	0.2363	1.3	0.83	1367.6	15.5	1358.1	11.3	1343.2	16.2	1343.2	16.2
IDOP_1-Spot 43	501	84366	5.8	11.5145	1.1	2.4298	2.5	0.2029	2.3	0.90	1191.0	24.5	1251.5	18.0	1357.1	21.1	1357.1	21.1
IDOP_1-Spot 184	1049	70992	1.4	11.4315	1.0	2.6630	2.0	0.2208	1.8	0.88	1286.0	21.0	1318.3	15.1	1371.0	18.7	1371.0	18.7
IDOP_1-Spot 198	135	64970	2.2	11.1940	1.1	3.0448	2.0	0.2472	1.7	0.85	1424.0	21.6	1418.9	15.3	1411.3	20.4	1411.3	20.4
IDOP_1-Spot 149	518	63484	5.5	11.0797	1.0	3.0025	1.8	0.2413	1.5	0.82	1393.3	18.6	1408.3	13.7	1430.9	19.5	1430.9	19.5
IDOP_1-Spot 64	71	49641	2.0	10.9636	1.4	3.1147	2.0	0.2477	1.4	0.71	1426.5	18.4	1436.3	15.4	1451.0	26.8	1451.0	26.8
IDOP_1-Spot 245	105	110399	1.0	10.9482	1.0	3.3029	1.8	0.2623	1.5	0.83	1501.4	19.7	1481.7	13.8	1453.7	19.0	1453.7	19.0
IDOP_1-Spot 39	666	57717	0.9	10.8998	1.2	3.2602	2.2	0.2577	1.8	0.85	1478.2	24.2	1471.6	16.8	1462.1	21.9	1462.1	21.9
IDOP_1-Spot 60	907	8208122	16.5	10.6931	0.9	3.1847	1.8	0.2470	1.6	0.87	1422.9	20.6	1453.5	14.2	1498.4	17.0	1498.4	17.0
IDOP_1-Spot 201	239	162904	1.4	10.4463	1.2	3.5016	1.9	0.2653	1.6	0.80	1516.9	21.0	1527.6	15.4	1542.4	22.0	1542.4	22.0
IDOP_1-Spot 209	524	58595	7.8	10.2876	1.1	3.5733	1.8	0.2666	1.4	0.79	1523.6	19.5	1543.6	14.4	1571.2	20.7	1571.2	20.7
IDOP_1-Spot 117	129	79510	1.2	10.2652	1.3	3.6195	2.1	0.2695	1.7	0.81	1538.1	23.6	1553.8	17.0	1575.2	23.6	1575.2	23.6
IDOP_1-Spot 95	321	176976	0.7	10.2358	1.2	3.6855	1.8	0.2736	1.3	0.73	1559.1	18.1	1568.2	14.4	1580.6	23.2	1580.6	23.2
IDOP_1-Spot 266	269	140010	1.7	10.1775	1.1	3.6063	2.0	0.2662	1.7	0.84	1521.5	23.0	1550.9	16.1	1591.3	20.7	1591.3	20.7
IDOP_1-Spot 174	403	285279	1.4	10.1269	1.1	3.9255	1.9	0.2883	1.5	0.80	1633.1	21.6	1618.9	15.2	1600.6	21.0	1600.6	21.0
IDOP_1-Spot 139	140	188519	2.0	10.0579	1.3	3.8383	2.0	0.2800	1.6	0.78	1591.3	22.0	1600.8	16.1	1613.3	23.4	1613.3	23.4
IDOP_1-Spot 303	166	201566	1.5	10.0495	0.9	3.9655	1.7	0.2890	1.4	0.85	1636.7	20.7	1627.2	13.6	1614.9	16.3	1614.9	16.3
IDOP_1-Spot 299	332	163095	4.3	10.0495	1.4	3.2819	2.1	0.2392	1.6	0.75	1382.5	19.5	1476.8	16.3	1614.9	25.7	1614.9	25.7
IDOP_1-Spot 53	278	109974	1.7	9.9919	0.9	3.9019	1.7	0.2828	1.5	0.86	1605.3	21.1	1614.1	14.0	1625.6	16.5	1625.6	16.5
IDOP_1-Spot 256	187	91650	3.7	9.9225	1.2	3.3859	1.9	0.2437	1.5	0.79	1405.7	19.3	1501.1	15.2	1638.5	22.0	1638.5	22.0
IDOP_1-Spot 133	165	193938	1.3	9.9063	1.1	4.1077	1.8	0.2951	1.4	0.79	1667.1	20.8	1655.8	14.7	1641.6	20.5	1641.6	20.5
IDOP_1-Spot 285	824	1418775	4.8	9.7883	0.9	3.5060	2.1	0.2489	1.9	0.91	1432.8	24.1	1528.6	16.4	1663.8	16.3	1663.8	16.3
IDOP_1-Spot 191	507	335380	2.9	9.6909	0.9	4.3646	1.7	0.3068	1.4	0.85	1724.8	21.4	1705.7	13.7	1682.2	15.9	1682.2	15.9
IDOP_1-Spot 195	344	210340	3.2	9.5019	1.1	4.4419	1.8	0.3061	1.5	0.80	1721.5	22.2	1720.2	15.2	1718.5	20.1	1718.5	20.1
IDOP_1-Spot 167	234	107672	2.9	9.4520	1.2	4.5598	2.3	0.3126	1.9	0.84	1753.4	29.6	1741.9	19.1	1728.2	22.9	1728.2	22.9
IDOP_1-Spot 144	97	81606	1.1	9.4451	1.1	4.5138	1.9	0.3092	1.5	0.82	1736.8	23.3	1733.5	15.5	1729.6	19.4	1729.6	19.4
IDOP_1-Spot 151	53	50909	0.8	9.4376	1.4	4.3843	2.0	0.3001	1.5	0.74	1691.8	22.3	1709.4	16.7	1731.0	24.9	1731.0	24.9
IDOP_1-Spot 74	66	110414	1.4	9.3705	1.4	4.4235	2.1	0.3006	1.6	0.77	1694.4	24.1	1716.8	17.5	1744.1	24.9	1744.1	24.9
IDOP_1-Spot 166	295	68834	4.3	9.2976	1.1	4.4311	2.1	0.2988	1.7	0.85	1685.4	25.8	1718.2	17.0	1758.4	19.8	1758.4	19.8
IDOP_1-Spot 51	116	124051	1.7	9.2322	1.0	4.6606	1.7	0.3121	1.3	0.81	1750.9	20.6	1760.2	13.8	1771.3	17.7	1771.3	17.7
IDOP_1-Spot 42	194	81738	3.1	9.1042	1.1	4.8627	1.9	0.3211	1.6	0.81	1795.0	24.5	1795.8	16.3	1796.7	20.9	1796.7	20.9
IDOP_1-Spot 262	525	155892	6.2	8.9133	0.9	4.9555	2.2	0.3204	2.0	0.92	1791.4	31.9	1811.8	18.8	1835.2	16.0	1835.2	16.0
IDOP_1-Spot 3	341	245779	5.9	8.8814	1.0	4.6108	2.0	0.2970	1.7	0.87	1676.4	25.7	1751.2	16.7	1841.7	17.7	1841.7	17.7
IDOP_1-Spot 275	220	130575	2.2	8.8551	1.1	5.0236	2.1	0.3226	1.8	0.85	1802.6	28.1	1823.3	17.8	1847.1	20.0	1847.1	20.0
IDOP_1-Spot 65	593	22760687	1.8	8.8456	1.2	4.9123	2.1	0.3151	1.7	0.83	1766.0	26.9	1804.4	17.7	1849.0	21.3	1849.0	21.3
IDOP_1-Spot 289	208	93891	5.7	8.8319	1.1	5.2850	2.0	0.3385	1.6	0.83	1879.6	26.7	1866.4	16.8	1851.8	19.7	1851.8	19.7
IDOP_1-Spot 89	190	50375	2.2	8.8214	1.0	5.1742	1.8	0.3310	1.5	0.84	1843.4	24.8	1848.4	15.7	1854.0	18.1	1854.0	18.1
IDOP_1-Spot 63	124	83536	3.3	8.8176	0.9	5.3123	1.6	0.3397	1.2	0.79	1885.4	20.3	1870.8	13.3	1854.7	17.1	1854.7	17.1
IDOP_1-Spot 83	502	375039	5.0	8.8099	1.0	5.3894	2.4	0.3444	2.2	0.92	1907.6	36.3	1883.2	20.5	1856.3	17.2	1856.3	17.2
IDOP_1-Spot 29	399	3343829	6.3	8.7750	1.0	5.3119	2.2	0.3381	1.9	0.89	1877.3	31.6	1870.8	18.6	1863.5	17.6	1863.5	17.6
IDOP_1-Spot 56	215	142672	2.2	8.7613	1.2	5.1798	2.1	0.3291	1.8	0.83	1834.2	28.4	1849.3	18.3	1866.3	21.6	1866.3	21.6
IDOP_1-Spot 112	415	111905	2.3	8.7528	1.1	5.3126	2.0	0.3372	1.7	0.83	1873.4	27.1	1870.9	17.2	1868.1	20.3	1868.1	20.3
IDOP_1-Spot 113	345	233458	8.7	8.7301	1.0	5.2660	2.0	0.3334	1.8	0.87	1855.0	28.5	1863.4	17.4	1872.7	18.2	1872.7	18.2
IDOP_1-Spot 243	173	244214	5.0	8.7216	1.0	5.2928	2.0	0.3348	1.8	0.88	1861.6	28.6	1867.7	17.2	1874.5	17.3	1874.5	17.3
IDOP_1-Spot 215	196	98830	3.0	8.6375	1.2	5.3134	1.9	0.3329	1.4	0.77	1852.2	23.0	1871.0	15.9	1891.9	21.5	1891.9	21.5
IDOP_1-Spot 46	61	161830	1.8	8.6280	1.2	5.3188	1.8	0.3328	1.4	0.75	1852.1	21.8	1871.9	15.4	1893.9	21.5	1893.9	21.5
IDOP_1-Spot 213	109	88070	1.9	8.4300	0.8	5.9174	1.9	0.3618	1.7	0.91	1990.7	29.4	1963.8	16.3	1935.6	13.6	1935.6	13.6
IDOP_1-Spot 268	942	136573	10.8	7.3666	0.8	6.6570	2.4	0.3557	2.3	0.94	1961.6	38.2	2066.9	21.1	2173.6	13.7	2173.6	13.7

IDOP_1-Spot 47	523	709171	2.8	6.3362	1.0	8.6658	1.7	0.3982	1.4	0.81	2160.9	24.8	2303.5	15.1	2432.5	16.3	2432.5	16.3
IDOP_1-Spot 66	229	375878	2.4	6.1080	1.1	9.8962	2.0	0.4384	1.7	0.84	2343.5	33.7	2425.2	18.7	2494.4	18.3	2494.4	18.3
IDOP_1-Spot 33	112	279418	1.7	6.0996	1.0	10.4320	1.7	0.4615	1.4	0.81	2446.2	28.0	2473.9	15.7	2496.7	16.7	2496.7	16.7
IDOP_1-Spot 87	67	82608	1.5	5.9952	1.1	11.0643	1.8	0.4811	1.5	0.80	2532.0	30.8	2528.6	17.2	2525.8	18.6	2525.8	18.6
IDOP_1-Spot 304	560	1019098	4.2	5.9694	1.0	10.2822	1.9	0.4452	1.7	0.87	2373.7	33.2	2460.5	17.9	2533.0	16.1	2533.0	16.1
IDOP_1-Spot 244	686	249465	1.5	5.5560	1.1	11.1910	2.2	0.4510	1.9	0.86	2399.5	38.2	2539.2	20.7	2652.7	18.9	2652.7	18.9
IDOP_1-Spot 94	278	234915	2.5	5.0652	0.9	14.6832	2.1	0.5394	1.9	0.90	2781.0	42.5	2794.9	19.8	2805.0	14.5	2805.0	14.5
IDOP_1-Spot 110	66	251130	2.1	4.9100	1.3	15.2932	2.1	0.5446	1.7	0.80	2802.7	37.6	2833.7	19.8	2855.8	20.5	2855.8	20.5
IDOP_1-Spot 288	325	342598	5.6	4.6919	1.0	14.0190	1.7	0.4770	1.3	0.80	2514.4	27.5	2751.0	15.7	2929.5	16.0	2929.5	16.0
IDOP_1-Spot 71	84	70026	1.2	4.0132	1.0	20.7939	2.2	0.6052	2.0	0.90	3050.9	48.7	3129.0	21.5	3179.5	15.3	3179.5	15.3
IDOP_1-Spot 284	311	148334	4.8	3.8757	1.4	20.6575	2.2	0.5807	1.8	0.80	2951.5	42.3	3122.7	21.7	3234.6	21.3	3234.6	21.3
IDOP_1-Spot 224	267	103401	1.5	3.6955	1.3	22.5143	2.3	0.6034	1.9	0.83	3043.7	46.8	3206.2	22.5	3309.5	20.1	3309.5	20.1

Table _____. U-Pb geochronologic analyses.																		
#3 U1452B_8F						Isotope ratios						Apparent ages (Ma)						
Mid Pleistocene 0.5 Ma																		
Analysis	U	206Pb	U/Th	206Pb*	±	207Pb*	±	206Pb*	±	error	206Pb*	±	207Pb*	±	206Pb*	±	Best age	±
	(ppm)	204Pb		207Pb*	(%)	235U*	(%)	238U	(%)	corr.	238U*	(Ma)	235U	(Ma)	207Pb*	(Ma)	(Ma)	(Ma)
Gleason-354-U1452B-8F-3W-85-150_9May16-Spot 249	397	624	1.5	39.7275	7.1	0.0081	7.4	0.0023	2.2	0.30	15.0	0.3	8.2	0.6	1734.8	249.3	15.0	0.3
Gleason-354-U1452B-8F-3W-85-150_9May16-Spot 249	3087	19314	31.2	21.3684	1.3	0.0212	1.9	0.0033	1.4	0.73	21.1	0.3	21.3	0.4	38.9	31.0	21.1	0.3
Gleason-354-U1452B-8F-3W-85-150_9May16-Spot 67	479	1074	2.4	26.2517	5.0	0.0185	5.3	0.0035	2.0	0.37	22.7	0.4	18.7	1.0	478.7	131.7	22.7	0.4
Gleason-354-U1452B-8F-3W-85-150_9May16-Spot 296	582	2052	0.4	24.3425	4.9	0.0210	5.4	0.0037	2.3	0.42	23.8	0.5	21.1	1.1	282.6	125.8	23.8	0.5
Gleason-354-U1452B-8F-3W-85-150_9May16-Spot 134	6560	23353	70.2	21.3010	0.8	0.0248	1.7	0.0038	1.6	0.90	24.7	0.4	24.9	0.4	46.5	18.0	24.7	0.4
Gleason-354-U1452B-8F-3W-85-150_9May16-Spot 177	2233	20856	0.5	19.8142	1.4	0.0316	2.2	0.0045	1.6	0.75	29.2	0.5	31.6	0.7	216.6	33.3	29.2	0.5
Gleason-354-U1452B-8F-3W-85-150_9May16-Spot 165	441	258	0.6	4.8739	14.5	0.1305	15.0	0.0046	4.1	0.27	29.7	1.2	124.5	17.6	2867.8	236.8	29.7	1.2
Gleason-354-U1452B-8F-3W-85-150_9May16-Spot 19	6919	14324	1.8	19.9048	1.4	0.0323	1.9	0.0047	1.4	0.71	30.0	0.4	32.3	0.6	206.1	31.4	30.0	0.4
Gleason-354-U1452B-8F-3W-85-150_9May16-Spot 90	984	2234	0.6	23.6433	1.7	0.0287	2.4	0.0049	1.6	0.69	31.7	0.5	28.8	0.7	208.9	42.8	31.7	0.5
Gleason-354-U1452B-8F-3W-85-150_9May16-Spot 192	341	2230	0.5	23.7204	5.6	0.0295	6.4	0.0051	3.0	0.47	32.6	1.0	29.5	1.8	217.1	141.1	32.6	1.0
Gleason-354-U1452B-8F-3W-85-150_9May16-Spot 30	1485	47395	0.8	21.1044	1.4	0.0364	2.0	0.0056	1.5	0.73	35.8	0.5	36.3	0.7	68.6	32.9	35.8	0.5
Gleason-354-U1452B-8F-3W-85-150_9May16-Spot 126	3217	460375	178.5	20.9505	1.2	0.0372	1.9	0.0057	1.5	0.79	36.3	0.6	37.1	0.7	86.0	28.2	36.3	0.6
Gleason-354-U1452B-8F-3W-85-150_9May16-Spot 132	285	1119	1.0	28.8430	19.6	0.0275	19.7	0.0058	2.6	0.13	37.0	1.0	27.5	5.4	735.0	551.3	37.0	1.0
Gleason-354-U1452B-8F-3W-85-150_9May16-Spot 150	675	4628	1.5	16.9582	3.1	0.0495	3.7	0.0061	2.1	0.56	39.1	0.8	49.0	1.8	566.0	67.3	39.1	0.8
Gleason-354-U1452B-8F-3W-85-150_9May16-Spot 278	810	14091	6.2	20.9445	1.8	0.0423	2.7	0.0064	2.0	0.75	41.3	0.8	42.1	1.1	86.7	41.8	41.3	0.8
Gleason-354-U1452B-8F-3W-85-150_9May16-Spot 93	366	1557	1.8	25.2606	4.2	0.0353	4.6	0.0065	2.0	0.43	41.6	0.8	35.2	1.6	377.8	108.4	41.6	0.8
Gleason-354-U1452B-8F-3W-85-150_9May16-Spot 181	4044	14239	0.5	21.1833	0.8	0.0423	3.1	0.0065	3.0	0.96	41.8	1.2	42.1	1.3	59.7	19.8	41.8	1.2
Gleason-354-U1452B-8F-3W-85-150_9May16-Spot 103	1189	4776	1.7	21.8737	2.0	0.0441	2.6	0.0070	1.6	0.64	45.0	0.7	43.9	1.1	17.3	47.5	45.0	0.7
Gleason-354-U1452B-8F-3W-85-150_9May16-Spot 23	1334	30121	2.1	21.2536	1.2	0.0465	2.0	0.0072	1.6	0.81	46.0	0.7	46.1	0.9	51.8	27.9	46.0	0.7
Gleason-354-U1452B-8F-3W-85-150_9May16-Spot 36	1717	23385	2.6	20.7007	1.3	0.0483	3.0	0.0073	2.7	0.90	46.6	1.2	47.9	1.4	114.4	31.2	46.6	1.2
Gleason-354-U1452B-8F-3W-85-150_9May16-Spot 52	548	15558	12.5	20.6297	1.9	0.0492	3.3	0.0074	2.7	0.81	47.3	1.3	48.7	1.6	122.4	45.5	47.3	1.3
Gleason-354-U1452B-8F-3W-85-150_9May16-Spot 88	1007	51380	2.1	20.8428	1.5	0.0487	2.5	0.0074	2.0	0.80	47.3	0.9	48.3	1.2	98.2	35.4	47.3	0.9
Gleason-354-U1452B-8F-3W-85-150_9May16-Spot 71	7728	206471	0.0	20.9706	0.9	0.0491	3.4	0.0075	3.3	0.97	48.0	1.6	48.7	1.6	83.7	20.4	48.0	1.6
Gleason-354-U1452B-8F-3W-85-150_9May16-Spot 189	1097	32230	0.8	20.0103	1.6	0.0518	2.3	0.0075	1.7	0.73	48.3	0.8	51.3	1.1	193.8	36.4	48.3	0.8
Gleason-354-U1452B-8F-3W-85-150_9May16-Spot 290	1447	150449	3.8	20.1305	1.1	0.0523	1.9	0.0076	1.5	0.81	49.0	0.7	51.7	0.9	179.8	25.5	49.0	0.7
Gleason-354-U1452B-8F-3W-85-150_9May16-Spot 297	591	68522	1.0	20.3464	1.4	0.0556	2.1	0.0082	1.5	0.73	52.7	0.8	55.0	1.1	155.0	33.7	52.7	0.8
Gleason-354-U1452B-8F-3W-85-150_9May16-Spot 135	344	4089	1.8	21.4001	2.6	0.0538	2.9	0.0084	1.3	0.46	53.6	0.7	53.2	1.5	35.4	61.8	53.6	0.7
Gleason-354-U1452B-8F-3W-85-150_9May16-Spot 28	1173	3539	5.8	20.0002	1.3	0.0625	2.3	0.0091	1.9	0.84	58.2	1.1	61.6	1.4	195.0	29.3	58.2	1.1
Gleason-354-U1452B-8F-3W-85-150_9May16-Spot 144	11186	370447	3.4	20.8389	0.8	0.0619	4.3	0.0093	4.3	0.98	60.0	2.6	60.9	2.6	98.6	18.5	60.0	2.6
Gleason-354-U1452B-8F-3W-85-150_9May16-Spot 129	1161	28912	1.3	20.9212	1.1	0.0629	1.8	0.0095	1.5	0.80	61.2	0.9	61.9	1.1	89.3	25.4	61.2	0.9
Gleason-354-U1452B-8F-3W-85-150_9May16-Spot 145	661	74474	1.4	19.7332	1.3	0.0703	2.1	0.0101	1.7	0.79	64.5	1.1	68.9	1.4	226.1	30.5	64.5	1.1
Gleason-354-U1452B-8F-3W-85-150_9May16-Spot 260	5138	54195	3.2	21.0326	0.8	0.0668	1.8	0.0102	1.5	0.88	65.3	1.0	65.6	1.1	76.7	20.1	65.3	1.0
Gleason-354-U1452B-8F-3W-85-150_9May16-Spot 244	111	1257	1.9	27.7212	3.8	0.0507	5.1	0.0102	3.4	0.67	65.4	2.2	50.3	2.5	625.3	104.4	65.4	2.2
Gleason-354-U1452B-8F-3W-85-150_9May16-Spot 279	1448	27885	1.8	20.8239	1.0	0.0691	1.5	0.0104	1.1	0.74	66.9	0.7	67.8	1.0	100.3	23.6	66.9	0.7
Gleason-354-U1452B-8F-3W-85-150_9May16-Spot 251	1118	12026	0.5	19.5921	1.8	0.0758	2.6	0.0108	1.9	0.72	69.1	1.3	74.2	1.8	242.7	41.0	69.1	1.3
Gleason-354-U1452B-8F-3W-85-150_9May16-Spot 108	726	34525	0.9	17.9999	1.8	0.0894	2.8	0.0117	2.1	0.76	74.8	1.6	87.0	2.3	434.6	39.6	74.8	1.6
Gleason-354-U1452B-8F-3W-85-150_9May16-Spot 164	3519	23342	50.7	16.2055	2.8	0.1182	7.4	0.0139	6.9	0.93	88.9	6.1	113.4	8.0	664.0	60.2	88.9	6.1
Gleason-354-U1452B-8F-3W-85-150_9May16-Spot 172	661	20742	3.1	17.9785	2.4	0.1171	3.0	0.0153	1.8	0.61	97.7	1.8	112.5	3.2	437.3	52.4	97.7	1.8
Gleason-354-U1452B-8F-3W-85-150_9May16-Spot 74	1527	153424	1.1	20.2066	0.9	0.1140	2.2	0.0167	2.0	0.92	106.8	2.1	109.6	2.3	171.0	20.2	106.8	2.1
Gleason-354-U1452B-8F-3W-85-150_9May16-Spot 306	433	11644	1.8	20.7435	1.6	0.1111	2.2	0.0167	1.6	0.72	106.9	1.7	107.0	2.3	109.5	36.8	106.9	1.7
Gleason-354-U1452B-8F-3W-85-150_9May16-Spot 268	2137	28468	39.2	12.1063	0.8	0.1921	2.0	0.0169	1.8	0.93	107.8	2.0	178.4	3.3	1259.8	14.7	107.8	2.0
Gleason-354-U1452B-8F-3W-85-150_9May16-Spot 200	901	13610	21.6	19.1203	1.5	0.1238	4.6	0.0172	4.3	0.95	109.7	4.7	118.5	5.1	298.5	33.4	109.7	4.7
Gleason-354-U1452B-8F-3W-85-150_9May16-Spot 205	1485	30832	0.5	20.6039	0.7	0.1159	1.8	0.0173	1.6	0.92	110.7	1.8	111.4	1.9	125.4	16.8	110.7	1.8
Gleason-354-U1452B-8F-3W-85-150_9May16-Spot 303	1946	75350	0.8	20.5061	0.8	0.1173	1.7	0.0174	1.5	0.88	111.5	1.6	112.6	1.8	136.6	18.6	111.5	1.6
Gleason-354-U1452B-8F-3W-85-150_9May16-Spot 13	7454	256957	3.0	20.4703	0.9	0.1179	2.2	0.0175	2.0	0.91	111.9	2.3	113.2	2.4	140.7	21.7	111.9	2.3
Gleason-354-U1452B-8F-3W-85-150_9May16-Spot 10	755	41455	0.9	20.2366	1.2	0.1198	2.2	0.0176	1.9	0.84	112.4	2.1	114.9	2.4	167.6	28.3	112.4	2.1
Gleason-354-U1452B-8F-3W-85-150_9May16-Spot 70	274	3425	1.7	21.7192	4.9	0.1123	5.6	0.0177	2.8	0.50	113.0	3.1	108.0	5.8	0.1	117.5	113.0	3.1
Gleason-354-U1452B-8F-3W-85-150_9May16-Spot 6	731	35179	0.9	20.5204	1.1	0.1192	1.8	0.0177	1.4	0.78	113.4	1.6	114.4	1.9	135.0	25.9	113.4	1.6
Gleason-354-U1452B-8F-3W-85-150_9May16-Spot 141	632	18106	1.5	20.3615	1.1	0.1222	2.1	0.0180	1.7	0.84	115.3	2.0	117.1	2.3	153.2	26.1	115.3	2.0
Gleason-354-U1452B-8F-3W-85-150_9May16-Spot 196	972	17323	1.2	20.7321	1.3	0.1229	2.2	0.0185	1.7	0.80	118.0	2.0	117.7	2.4	110.8	31.0	118.0	2.0
Gleason-354-U1452B-8F-3W-85-150_9May16-Spot 230	328	27242	1.2	18.6002	2.1	0.1372	3.1	0.0185	2.4	0.76	118.2	2.8	130.5	3.9	361.1	46.4	118.2	2.8
Gleason-354-U1452B-8F-3W-85-150_9May16-Spot 50	394	35998	0.7	20.2408	1.3	0.1266	2.4	0.0186	2.1	0.85	118.7	2.5	121.0	2.8	167.1	29.9	118.7	2.5
Gleason-354-U1452B-8F-3W-85-150_9May16-Spot 117	230	2007	0.6	24.0979	4.6	0.1071	5.2	0.0187	2.5	0.48	119.6	2.9	103.3	5.1	256.9	115.5	119.6	2.9
Gleason-354-U1452B-8F-3W-85-150_9May16-Spot 155	840	135716	1.8	20.0311	1.0	0.1331	2.0	0.0193	1.7	0.87	123.5	2.1	126.9	2.4	191.4	23.2	123.5	2.1
Gleason-354-U1452B-8F-3W-85-150_9May16-Spot 285	950	111586	0.8	19.7088	1.1	0.1359	2.0	0.0194	1.7	0.85	124.1	2.1	129.4	2.5	229.0	25.1	124.1	2.1
Gleason-354-U1452B-8F-3W-85-150_9May16-Spot 237	624	8954	1.8	17.8023	3.9	0.1511	4.5	0.0195	2.2	0.50	124.5	2.7	142.9	6.0	459.2	86.6	124.5	2.7
Gleason-354-U1452B-8F-3W-85-150_9May16-Spot 287	1567	47151	1.2	19.7856	0.9	0.1370	1.6	0.0197	1.4	0.83	125.5	1.7	130.4	2.0	220.0	21.3	125.5	1.7
Gleason-354-U1452B-8F-3W-85-150_9May16-Spot 186	848	117847	2.0	20.5061	1.0	0.1421	2.3	0.0211	2.1	0.90	134.8	2.8	134.9	2.9	136.6	23.3	134.8	2.8
Gleason-354-U1452B-8F-3W-85-150_9May16-Spot 65	544	9340	1.8	20.6512	0.9	0.1420	1.9	0.0213	1.7	0.90	135.7	2.3	134.8	2.4	120.0	20.1	135.7	2.3
Gleason-354-U1452B-8F-3W-85-150_9May16-Spot 309	1131	693436	2.7	17.5903	0.7	0.1743	6.7	0.0222	6.7	0.99	141.8	9.4	163.1	10.2	485.7	15.9	141.8	9.4
Gleason-354-U1452B-8F-3W-85-150_9May16-Spot 24	2364	28962	0.9	19.6824	0.8	0.1561	2.0	0.0223	1.8	0.92	142.0	2.5	147.3	2.7	232.1	17.7	142.0	2.5
Gleason-354-U1452B-8F-3W-85-150_9May16-Spot 136	418	25573	1.6	18.5418	1.6	0.1729	3.2	0.0232	2.8	0.87	148.1	4.1	161.9	4.8	368.2	34.9	148.1	4.1
Gleason-354-U1452B-8F-3W-85-150_9May16-Spot 53	828	29706	2.7	18.6144	1.1	0.1722	1.9	0.0232	1.6	0.83	148.1	2.3	161.3	2.9	359.4	24.2	148.1	2.3
Gleason-354-U1452B-8F-3W-85-150_9May16-Spot 143	600	45392	3.0	19.5941	1.0	0.1702	2.8	0.0242	2.6	0.94	154.1	4.0	159.6	4.1	242.4	22.4	154.1	4.0
Gleason-354-U1452B-8F-3W-85-150_9May16-Spot 232	56	23277	1.9	19.3329	3.0	0.1750	5.0	0.0245	4.0	0.80	156.2	6.2	163.7	7.5	273.3	68.6	156.2	6.2
Gleason-354-U1452B-8F-3W-85-150_9May16-Spot 163	1213	160784	1.6	19.5595	0.9	0.2101	1.8	0.0298	1.6	0.88	189.3	3.0	193.6	3.2	246.5	20.2	189.3	3.0
Gleason-354-U1452B-8F-3W-85-150_9May16-Spot 171	177	4571	1.6	20.4315	3.0	0.2048	4.1	0.0303	2.8	0.69	192.7	5.3	189.2	7.0	145.1	69.6	192.7	5.3
Gleason-354-U1452B-8F-3W-85-150_9May16-Spot 147	1082	166810	1.8	19.6083	0.7	0.2194	1.5	0.0312	1.3	0.90	198.0	2.6	201.4	2.7	240.8	15.2	198.0	2.6
Gleason-354-U1452B-8F-3W-85-150_9May16-Spot 313	756	38640	1.8	19.7101	0.6	0.2234	1.6	0.0319	1.4	0.93	202.6	2.9	204.7	2.9	228.8	13.1	202.6	2.9
Gleason-354-U1452B-8F-3W-85-150_9May16-Spot 33	438	6275	1.6	20.1827	1.0	0.2201	2.3	0.0322	2.1	0.91	204.4	4.2	202.0	4.2	173.8	22.7	204.4	4.2
Gleason-354-U1452B-8F-3W-85-150_9May16-Spot 236	3176	29740	3.1	18.6042	1.7	0.2473	2.2	0.0334	1.5	0.67	211.6	3.1	224.4	4.5	360.6	37.6	211.6	3.1
Gleason-354-U1452B-8F-3W-85-150_9May16-Spot 77	603	54724	3.7	17.8450	1.1	0.3036	2.7	0.0393	2.5	0.92	248.4	6.0	269.2	6.3	453.9	23.4	248.4	6.0
Gleason-354-U1452B-8F-3W-85-150_9May16-Spot 138	2443	2190003	0.6	19.3603	0.6	0.2907	1.5	0.0408	1.4	0.90	257.9	3.4	259.1	3.4	270.0	14.7	257.9	3.4
Gleason-354-U1452B-8F-3W-85-150_9May16-Spot 198	458	221380	1.1	18.9939	0.9	0.3025	2.0	0.0417	1.7	0.88	263.2	4.5	268.3	4.6	313.7	21.3	263.2	4.5
Gleason-354-U1452B-8F-3W-85-150_9May16-Spot 82	565	21603	5.8	18.5194	0.9	0.3197	4.8	0.0429	4.7	0.98	271.1	12.5	281.7	11.8	370.9	21.3	271.1	12.5
Gleason-354-U1452B-8F-3W-85-150_9May16-Spot 17	951	72272	3.5	17.4135	1.0	0.3937	3.4	0.0497	3.2	0.96	312.8	9.8	337.1	9.6	508.0	21.4	312.8	9.8
Gleason-354-U1452B-8F-3W-85-150_9May16-Spot 41	8508	36460	10.2	9.3409	0.8	0.7456	2.5	0.0505	2.4	0.95	317.7	7.4	565.7	10.9	1749.9	14.4	317.7	7.4
Gleason-354-U1452B-8F-3W-85-150_9May16-Spot 294	669	54211	3.7	10.1662	0.5	0.6977	5.0	0.0514	5.0	0.99	323.4	15.6	537.4	20.8	1593.3	9.7	323.4	15.6
Gleason-354-U1452B-8F-3W-85-150_9May16-Spot 178	3235	551664	5.2	17.3750	0.6	0.4106	2.4	0.0517	2.3	0.96	325.2	7.2	349.3	7.0	512.8	13.9	325.2	7.2
Gleason-354-U1452B-8F-3W-85-150_9May16-Spot 312	988	146727	6.7	17.2889	0.7	0.4172	6.7	0.0523	6.7	1.00	328.7	21.5	354.0	20.2	523.7	14.5	328.7	21.5
Gleason-354-U1452B-8F-3W-85-150_9May16-Spot 1	2866	319709	2.3	17.9908	0.7	0.4122	1.9	0.0538	1.7	0.94	337.7	5.8	350.5	5.5	435.8	14.5	337.7	5.8
Gleason-354-U1452B-8F-3W-85-150_9May16-Spot 169	1857	1100255	5.3	18.7061	0.5	0.4087	1.6	0.0554	1.5	0.95	347.8	5.1	347.9	4.6	348.3	10.6	347.8	5.1
Gleason-354-U1452B-8F-3W-85-150_9May16-Spot 240	474	145409	2.2	12.7205	0.9	0.6566	2.3	0.0606	2.2	0.92	379.2	7.9	512.6	9.4	1162.4	18.5	379.2	7.9
Gleason-354-U1452B-8F-3W-85-150_9May16-Spot 168	2156	4016544	9.6	13.7525	0.6	0.6338	2.0	0.0632	1.9	0.96	395.1	7.4	498.4	7.9	1006.0	11.7	395.1	7.4
Gleason-354-U1452B-8F-3W-85-150_9May16-Spot 57	307	70409	4.6	14.8550	1.0	0.5904	3.3	0.0636	3.2	0.96	397.5	12.2	471.1	12.4	847.6	20.2	397.5	12.2
Gleason-354-U1452B-8F-3W-85-150_9May16-Spot 25	1041	112951	2.4	17.3181	0.9	0.5572	4.7	0.0700	4.6	0.98	436.1	19.4	449.7	17.0	520.0	19.4	436.1	19.4
Gleason-354-U1452B-8F-3W-85-150_9May16-Spot 151	447	12668	2.3	17.0266	0.9	0.5930	1.8	0.0732	1.6	0.87	455.6	7.0	472.8	7.0	557.2	20.0	455.6	7.0
Gleason-354-U1452B-8F-3W-85-150_9May16-Spot 85	565	144540	1.3	17.4018	0.9	0.5923	2.0	0.0748	1.8	0.90	464.7	7.9	472.3	7.4	509.4	18.8	464.7	7.9
Gleason-354-U1452B-8F-3W-85-150_9May16-Spot 79	2126	563610	2.2	17.4104	0.5	0.5950	1.4	0.0751	1.4	0.93	467.0	6.1	474.0	5.5	508.4	11.4	467.0	6.1
Gleason-354-U1452B-8F-3W-85-150_9May16-Spot 161	576	108588	1.9	17.1997	0.7	0.6034	2.2	0.0753	2.1	0.95	467.8	9.4	479.4	8.4	535.1	15.2	467.8	9.4
Gleason-354-U1452B-8F-3W-85-150_9May16-Spot 217	537	100296	2.0	17.2150	0.8	0.6077	1.8	0.0759	1.6	0.91	471.4	7.4	482.1	6.9	533.1	16.5	471.4	7.4
Gleason-354-U1452B-8F-3W-85-150_9May16-Spot 119	1256	332631	1.6	17.4209	0.6	0.6056	1.4	0.0765	1.3	0.92	475.3	5.9	480.8	5.3	507.0	12.2	475.3	5.9
Gleason-354-U1452B-8F-3W-85-150_9May16-Spot 212	1174	36803	4.2	17.5557	0.6	0.6019	1.6	0.0766	1.5	0.92	476.0	7.0	478.4	6.3	490.0	14.0	476.0	7.0
Gleason-354-U1452B-8F-3W-85-150_9May16-Spot 227	954	64079	6.8	17.5687	0.7	0.6098	1.7	0.0777	1.6	0.92	482.4	7.2	483.4	6.5	488.4	14.6	482.4	7.2
Gleason-354-U1452B-8F-3W-85-150_9May16-Spot 27	1187	43149	1.3	17.7403	0.7	0.6059	1.5	0.0780	1.3	0.87	483.9	6.0	481.0	5.7	466.9	16.3	483.9	6.0
Gleason-354-U1452B-8F-3W-85-150_9May16-Spot 154	1801	146088	1.7	17.3229	0.5	0.6215	1.4	0.0781	1.3	0.92	484.7	6.1	490.8	5.5	519.4	11.9	484.7	6.1
Gleason-354-U1452B-8F-3W-85-150_9May16-Spot 221	1513	214063	1.0	17.2841	0.8	0.6239	2.4	0.0782	2.3	0.94	485.4	10.7	492.3	9.5	524.3	18.1	485.4	10.7
Gleason-354-U1452B-8F-3W-85-150_9May16-Spot 252	1040	140921	1.9	17.3384	0.6	0.6221	1.4	0.0782	1.3	0.90	485.5	6.0	491.2	5.6	517.4	13.7	485.5	6.0
Gleason-354-U1452B-8F-3W-85-150_9May16-Spot 95	631	41219	2.5	17.3938	0.8	0.6242	2.0	0.0787	1.8	0.91	488.6	8.6	492.5	7.8	510.5	17.7	488.6	8.6
Gleason-354-U1452B-8F-3W-85-150_9May16-Spot 58	2013	284521	4.7	17.4279	0.6	0.6314	1.7	0.0798	1.6	0.94	495.0	7.8	496.9	6.9	506.1	13.0	495.0	7.8

Gleason-354-U1452B-8F-3W-85-150_9May16-Spot 166	502	321594	0.4	17.1861	0.7	0.6481	2.3	0.0808	2.2	0.96	500.8	10.5	507.3	9.1	536.8	14.2	500.8	10.5
Gleason-354-U1452B-8F-3W-85-150_9May16-Spot 210	1022	34301	6.1	16.5025	0.9	0.6760	2.1	0.0809	1.9	0.91	501.6	9.1	524.4	8.5	624.9	18.4	501.6	9.1
Gleason-354-U1452B-8F-3W-85-150_9May16-Spot 226	1293	254547	9.7	17.2618	0.7	0.6641	1.7	0.0831	1.5	0.91	514.8	7.6	517.1	6.8	527.2	15.6	514.8	7.6
Gleason-354-U1452B-8F-3W-85-150_9May16-Spot 229	2157	338575	0.9	17.3065	0.5	0.6627	1.2	0.0832	1.1	0.90	515.1	5.3	516.3	4.8	521.5	11.2	515.1	5.3
Gleason-354-U1452B-8F-3W-85-150_9May16-Spot 315	526	25245	0.7	16.7343	0.9	0.7087	1.8	0.0860	1.6	0.88	531.9	8.2	544.0	7.7	594.8	18.9	531.9	8.2
Gleason-354-U1452B-8F-3W-85-150_9May16-Spot 258	131	21748	0.6	16.7706	1.1	0.7080	3.8	0.0861	3.6	0.96	532.5	18.4	543.6	15.8	590.1	22.9	532.5	18.4
Gleason-354-U1452B-8F-3W-85-150_9May16-Spot 253	102	10711	0.5	17.3166	1.2	0.6911	2.8	0.0868	2.6	0.91	536.5	13.1	533.4	11.6	520.2	25.5	536.5	13.1
Gleason-354-U1452B-8F-3W-85-150_9May16-Spot 170	378	30819	1.7	17.0422	0.7	0.7049	1.9	0.0871	1.8	0.94	538.6	9.4	541.7	8.1	555.1	14.5	538.6	9.4
Gleason-354-U1452B-8F-3W-85-150_9May16-Spot 69	1384	114198	30.3	16.9462	0.5	0.7240	1.5	0.0890	1.5	0.94	549.5	7.6	553.0	6.6	567.5	11.2	549.5	7.6
Gleason-354-U1452B-8F-3W-85-150_9May16-Spot 60	699	77887	2.0	17.0009	0.9	0.7285	2.1	0.0898	1.9	0.90	554.5	9.9	555.7	8.9	560.4	20.0	554.5	9.9
Gleason-354-U1452B-8F-3W-85-150_9May16-Spot 124	410	38009	1.5	16.8912	0.8	0.7496	2.1	0.0918	2.0	0.93	566.4	10.6	568.0	9.1	574.5	16.5	566.4	10.6
Gleason-354-U1452B-8F-3W-85-150_9May16-Spot 231	189	43092	6.5	16.9406	1.1	0.7711	2.3	0.0947	2.1	0.89	583.5	11.7	580.4	10.4	568.2	23.1	583.5	11.7
Gleason-354-U1452B-8F-3W-85-150_9May16-Spot 43	1611	206397	9.3	16.1076	0.8	0.8262	1.8	0.0965	1.6	0.90	594.0	9.0	611.5	8.1	677.0	16.4	594.0	9.0
Gleason-354-U1452B-8F-3W-85-150_9May16-Spot 5	298	33119	1.0	16.4981	0.9	0.8107	2.3	0.0970	2.1	0.93	596.8	12.1	602.8	10.4	625.5	18.7	596.8	12.1
Gleason-354-U1452B-8F-3W-85-150_9May16-Spot 139	205	39556	2.8	16.5193	0.9	0.8130	2.0	0.0974	1.8	0.90	599.2	10.3	604.2	9.1	622.7	18.7	599.2	10.3
Gleason-354-U1452B-8F-3W-85-150_9May16-Spot 180	202	28182	1.2	16.6491	0.9	0.8068	2.2	0.0974	2.0	0.91	599.3	11.4	600.7	9.9	605.8	19.1	599.3	11.4
Gleason-354-U1452B-8F-3W-85-150_9May16-Spot 18	229	92112	2.2	15.9434	1.0	0.8685	3.5	0.1004	3.4	0.96	616.9	19.9	634.7	16.6	698.8	21.2	616.9	19.9
Gleason-354-U1452B-8F-3W-85-150_9May16-Spot 276	644	117808	6.7	15.8003	0.8	0.9515	3.4	0.1090	3.3	0.97	667.1	21.0	678.9	16.9	718.0	17.2	667.1	21.0
Gleason-354-U1452B-8F-3W-85-150_9May16-Spot 216	1973	280162	6.8	15.3907	0.6	0.9789	1.6	0.1093	1.5	0.92	668.5	9.4	693.1	8.1	773.5	13.6	668.5	9.4
Gleason-354-U1452B-8F-3W-85-150_9May16-Spot 241	221	66459	1.2	15.8219	0.8	0.9556	2.5	0.1097	2.4	0.95	670.7	15.0	681.0	12.3	715.1	16.4	670.7	15.0
Gleason-354-U1452B-8F-3W-85-150_9May16-Spot 213	644	52829	3.6	14.9308	0.8	1.0347	2.2	0.1120	2.0	0.94	684.6	13.3	721.3	11.2	837.0	15.7	684.6	13.3
Gleason-354-U1452B-8F-3W-85-150_9May16-Spot 265	76	9955	1.2	15.8216	1.4	0.9868	3.1	0.1132	2.8	0.90	691.5	18.1	697.1	15.6	715.1	29.3	691.5	18.1
Gleason-354-U1452B-8F-3W-85-150_9May16-Spot 120	70	5423	1.2	16.1679	2.6	0.9717	3.8	0.1139	2.8	0.74	695.6	18.7	689.3	19.2	669.0	55.1	695.6	18.7
Gleason-354-U1452B-8F-3W-85-150_9May16-Spot 123	619	372403	10.9	14.8807	0.9	1.0962	2.2	0.1183	2.1	0.92	720.8	14.0	751.5	11.9	844.0	18.4	720.8	14.0
Gleason-354-U1452B-8F-3W-85-150_9May16-Spot 250	1004	140065	2.2	14.9340	0.8	1.1308	1.9	0.1225	1.7	0.91	744.8	11.9	768.1	10.0	836.6	15.7	744.8	11.9
Gleason-354-U1452B-8F-3W-85-150_9May16-Spot 259	456	375873	1.6	14.5983	1.0	1.1684	2.0	0.1237	1.8	0.88	751.8	12.6	785.9	11.0	883.8	19.9	751.8	12.6
Gleason-354-U1452B-8F-3W-85-150_9May16-Spot 282	335	131316	1.2	14.6001	0.7	1.1980	2.0	0.1269	1.9	0.93	769.9	13.5	799.7	11.1	883.5	15.2	769.9	13.5
Gleason-354-U1452B-8F-3W-85-150_9May16-Spot 304	522	49204	3.9	15.4360	0.8	1.1347	1.6	0.1270	1.4	0.89	770.9	10.5	770.0	8.8	767.3	15.9	770.9	10.5
Gleason-354-U1452B-8F-3W-85-150_9May16-Spot 72	167	13316	1.4	15.1558	0.8	1.1726	2.5	0.1289	2.3	0.95	781.5	17.2	787.9	13.5	805.8	16.4	781.5	17.2
Gleason-354-U1452B-8F-3W-85-150_9May16-Spot 34	513	59823	12.9	15.1549	0.8	1.1951	2.0	0.1314	1.8	0.92	795.6	13.7	798.3	10.9	805.9	15.9	795.6	13.7
Gleason-354-U1452B-8F-3W-85-150_9May16-Spot 271	761	427825	9.9	14.7117	0.9	1.2379	1.7	0.1321	1.5	0.86	799.8	11.2	817.9	9.7	867.8	18.1	799.8	11.2
Gleason-354-U1452B-8F-3W-85-150_9May16-Spot 149	245	370451	1.9	14.3231	0.7	1.2721	2.3	0.1321	2.1	0.95	800.1	16.1	833.3	12.9	923.0	14.9	800.1	16.1
Gleason-354-U1452B-8F-3W-85-150_9May16-Spot 96	204	112474	1.1	15.1044	0.9	1.2134	2.7	0.1329	2.5	0.94	804.5	19.1	806.8	14.9	812.9	18.5	804.5	19.1
Gleason-354-U1452B-8F-3W-85-150_9May16-Spot 248	181	42701	0.9	14.9189	0.8	1.2404	2.3	0.1342	2.2	0.94	811.9	16.7	819.1	13.1	838.7	16.6	811.9	16.7
Gleason-354-U1452B-8F-3W-85-150_9May16-Spot 292	841	72707	3.2	14.8514	0.6	1.2569	1.5	0.1354	1.4	0.92	818.5	10.7	826.5	8.5	848.1	12.0	818.5	10.7
Gleason-354-U1452B-8F-3W-85-150_9May16-Spot 162	2175	332066	2.7	14.4103	1.0	1.3054	2.6	0.1364	2.4	0.92	824.4	18.7	848.1	15.1	910.5	21.6	824.4	18.7
Gleason-354-U1452B-8F-3W-85-150_9May16-Spot 22	342	117975	0.9	14.9949	0.8	1.2569	2.2	0.1367	2.1	0.94	826.0	16.2	826.5	12.6	828.1	16.2	826.0	16.2
Gleason-354-U1452B-8F-3W-85-150_9May16-Spot 133	1887	202034	9.7	14.1366	0.6	1.3411	1.4	0.1375	1.3	0.91	830.5	10.1	863.7	8.3	949.9	11.9	830.5	10.1
Gleason-354-U1452B-8F-3W-85-150_9May16-Spot 228	270	125576	3.0	14.5074	0.7	1.3217	2.2	0.1391	2.1	0.95	839.4	16.5	855.2	12.8	896.7	14.3	839.4	16.5
Gleason-354-U1452B-8F-3W-85-150_9May16-Spot 220	271	78350	2.8	14.3516	0.5	1.3407	1.9	0.1395	1.9	0.96	842.1	14.7	863.5	11.3	918.9	10.9	842.1	14.7
Gleason-354-U1452B-8F-3W-85-150_9May16-Spot 214	955	71776	1.6	14.9487	0.5	1.2871	1.6	0.1395	1.6	0.96	842.1	12.3	840.0	9.3	834.5	9.5	842.1	12.3
Gleason-354-U1452B-8F-3W-85-150_9May16-Spot 207	190	63229	1.7	14.6855	0.9	1.3199	2.2	0.1406	2.0	0.91	847.9	16.0	854.5	12.7	871.4	18.5	847.9	16.0
Gleason-354-U1452B-8F-3W-85-150_9May16-Spot 40	35	23851	0.8	14.6885	1.2	1.3772	4.7	0.1467	4.5	0.97	882.5	37.5	879.2	27.6	871.0	24.3	882.5	37.5
Gleason-354-U1452B-8F-3W-85-150_9May16-Spot 275	536	114640	5.9	14.5312	0.6	1.4104	2.2	0.1486	2.1	0.96	893.4	17.7	893.3	13.1	893.3	12.3	893.4	17.7
Gleason-354-U1452B-8F-3W-85-150_9May16-Spot 307	297	74374	0.8	14.4734	0.6	1.4697	2.3	0.1543	2.3	0.96	924.9	19.5	918.0	14.2	901.5	12.8	901.5	12.8
Gleason-354-U1452B-8F-3W-85-150_9May16-Spot 199	929	263822	5.3	14.2585	0.8	1.4351	2.5	0.1484	2.4	0.95	892.0	19.9	903.7	15.0	932.3	15.7	932.3	15.7
Gleason-354-U1452B-8F-3W-85-150_9May16-Spot 125	477	69621	1.1	14.2270	0.7	1.4687	1.5	0.1515	1.4	0.90	909.6	11.7	917.6	9.2	936.8	13.3	936.8	13.3
Gleason-354-U1452B-8F-3W-85-150_9May16-Spot 191	136	20407	0.5	14.1936	0.8	1.6048	2.5	0.1652	2.4	0.94	985.6	21.7	972.1	15.8	941.6	17.3	941.6	17.3
Gleason-354-U1452B-8F-3W-85-150_9May16-Spot 173	364	68626	1.7	14.1830	0.7	1.5154	1.9	0.1559	1.8	0.92	933.8	15.5	936.6	11.8	943.2	15.0	943.2	15.0
Gleason-354-U1452B-8F-3W-85-150_9May16-Spot 26	1157	176359	4.6	14.1602	0.6	1.4422	1.6	0.1481	1.5	0.92	890.4	12.2	906.6	9.5	946.5	12.5	946.5	12.5
Gleason-354-U1452B-8F-3W-85-150_9May16-Spot 68	445	61751	1.8	14.1458	0.6	1.6046	1.7	0.1646	1.6	0.94	982.4	14.7	972.0	10.7	948.5	12.1	948.5	12.1
Gleason-354-U1452B-8F-3W-85-150_9May16-Spot 152	1236	241940	4.7	14.0847	0.7	1.4929	1.9	0.1525	1.7	0.92	915.0	14.6	927.5	11.3	957.4	14.9	957.4	14.9
Gleason-354-U1452B-8F-3W-85-150_9May16-Spot 209	207	40086	2.4	14.0713	0.6	1.6110	2.2	0.1644	2.2	0.96	981.2	19.6	974.5	14.0	959.3	12.3	959.3	12.3
Gleason-354-U1452B-8F-3W-85-150_9May16-Spot 80	383	42583504	4.3	14.0568	0.6	1.5919	1.7	0.1623	1.6	0.92	969.5	14.0	967.0	10.5	961.5	13.2	961.5	13.2
Gleason-354-U1452B-8F-3W-85-150_9May16-Spot 299	448	498364	2.1	13.9797	0.6	1.5755	1.5	0.1597	1.3	0.90	955.3	11.7	960.6	9.1	972.7	12.8	972.7	12.8
Gleason-354-U1452B-8F-3W-85-150_9May16-Spot 16	99	6600	1.1	13.9419	1.2	1.5098	3.3	0.1527	3.1	0.93	915.9	26.5	934.4	20.4	978.2	24.9	978.2	24.9
Gleason-354-U1452B-8F-3W-85-150_9May16-Spot 81	665	63187	4.0	13.9417	0.7	1.6209	1.6	0.1639	1.5	0.92	978.4	13.7	978.3	10.3	978.2	13.5	978.2	13.5
Gleason-354-U1452B-8F-3W-85-150_9May16-Spot 219	948	2456351	11.4	13.9332	0.5	1.5189	1.8	0.1535	1.7	0.96	920.5	15.0	938.0	11.1	979.4	10.5	979.4	10.5
Gleason-354-U1452B-8F-3W-85-150_9May16-Spot 29	289	126349	1.8	13.8915	0.8	1.7273	3.0	0.1740	2.9	0.96	1034.3	27.3	1018.7	19.2	985.6	17.2	985.6	17.2
Gleason-354-U1452B-8F-3W-85-150_9May16-Spot 246	979	155366	2.6	13.8529	0.7	1.6460	2.5	0.1654	2.4	0.96	986.6	21.5	988.0	15.5	991.2	14.8	991.2	14.8
Gleason-354-U1452B-8F-3W-85-150_9May16-Spot 8	2360	262198	1.2	13.8381	0.6	1.5996	1.8	0.1605	1.7	0.93	959.8	14.9	970.0	11.2	993.4	13.0	993.4	13.0
Gleason-354-U1452B-8F-3W-85-150_9May16-Spot 159	398	84090	1.3	13.8198	0.6	1.5702	2.1	0.1574	2.0	0.95	942.2	17.3	958.5	12.9	996.1	12.8	996.1	12.8
Gleason-354-U1452B-8F-3W-85-150_9May16-Spot 239	28	4079	1.0	13.8182	2.8	1.5939	5.5	0.1597	4.8	0.87	955.3	42.7	967.8	34.6	996.3	56.2	996.3	56.2
Gleason-354-U1452B-8F-3W-85-150_9May16-Spot 42	359	189373	1.7	13.7645	0.8	1.6388	1.7	0.1636	1.5	0.89	976.8	13.8	985.3	10.8	1004.2	15.9	1004.2	15.9
Gleason-354-U1452B-8F-3W-85-150_9May16-Spot 197	475	125607	4.2	13.7207	0.8	1.5268	1.9	0.1519	1.7	0.90	911.8	14.5	941.2	11.7	1010.7	17.0	1010.7	17.0
Gleason-354-U1452B-8F-3W-85-150_9May16-Spot 153	491	40096	1.3	13.6943	0.7	1.4755	1.8	0.1465	1.6	0.92	881.6	13.3	920.4	10.6	1014.6	13.9	1014.6	13.9
Gleason-354-U1452B-8F-3W-85-150_9May16-Spot 49	81	15054	3.9	13.6630	0.9	1.5970	3.3	0.1583	3.2	0.96	947.1	28.2	969.0	20.8	1019.2	19.2	1019.2	19.2
Gleason-354-U1452B-8F-3W-85-150_9May16-Spot 137	546	63503	2.6	13.6052	0.7	1.6246	1.9	0.1603	1.8	0.94	958.5	16.3	979.8	12.2	1027.8	13.6	1027.8	13.6
Gleason-354-U1452B-8F-3W-85-150_9May16-Spot 140	726	98862	9.8	13.5008	0.6	1.7920	1.8	0.1755	1.7	0.94	1042.2	16.3	1042.5	11.8	1043.3	12.6	1043.3	12.6
Gleason-354-U1452B-8F-3W-85-150_9May16-Spot 188	468	221527	2.9	13.4807	0.7	1.7991	2.0	0.1759	1.8	0.93	1044.6	17.5	1045.1	12.8	1046.4	14.8	1046.4	14.8
Gleason-354-U1452B-8F-3W-85-150_9May16-Spot 183	188	394827	2.0	13.4261	0.7	1.5977	2.5	0.1556	2.4	0.96	932.1	20.8	969.3	15.6	1054.6	14.3	1054.6	14.3
Gleason-354-U1452B-8F-3W-85-150_9May16-Spot 31	226	27950	0.9	13.3702	0.9	1.8940	2.5	0.1837	2.4	0.94	1086.9	23.7	1079.0	16.8	1063.0	18.0	1063.0	18.0
Gleason-354-U1452B-8F-3W-85-150_9May16-Spot 283	233	166148	1.6	13.3626	0.7	1.8508	2.6	0.1794	2.5	0.97	1063.6	24.3	1063.7	16.9	1064.1	13.3	1064.1	13.3
Gleason-354-U1452B-8F-3W-85-150_9May16-Spot 54	281	156347	1.6	13.2750	0.7	1.8994	2.2	0.1829	2.1	0.94	1082.6	20.9	1080.9	14.8	1077.3	14.7	1077.3	14.7
Gleason-354-U1452B-8F-3W-85-150_9May16-Spot 277	431	393685	1.1	13.2343	0.6	1.8881	2.3	0.1812	2.2	0.96	1073.7	21.5	1076.9	15.0	1083.5	12.6	1083.5	12.6
Gleason-354-U1452B-8F-3W-85-150_9May16-Spot 38	552	33373	3.1	13.1903	0.9	2.0103	3.1	0.1923	2.9	0.96	1133.9	30.6	1119.0	20.8	1090.2	17.6	1090.2	17.6
Gleason-354-U1452B-8F-3W-85-150_9May16-Spot 121	130	56719	1.3	13.0924	1.2	1.9516	2.4	0.1853	2.1	0.87	1095.9	21.5	1099.0	16.4	1105.0	23.6	1105.0	23.6
Gleason-354-U1452B-8F-3W-85-150_9May16-Spot 14	368	85333	2.1	13.0259	0.7	1.9671	1.7	0.1858	1.6	0.91	1098.8	15.9	1104.3	11.6	1115.2	14.3	1115.2	14.3
Gleason-354-U1452B-8F-3W-85-150_9May16-Spot 63	507	41043	6.7	12.9906	0.9	1.8256	2.5	0.1720	2.3	0.93	1023.1	21.9	1054.7	16.3	1120.7	18.4	1120.7	18.4
Gleason-354-U1452B-8F-3W-85-150_9May16-Spot 190	949	1459077	2.7	12.9778	0.6	1.8738	1.5	0.1764	1.4	0.93	1047.1	13.7	1071.9	10.1	1122.6	11.4	1122.6	11.4
Gleason-354-U1452B-8F-3W-85-150_9May16-Spot 269	148	43051	1.3	12.9353	0.8	2.0142	2.7	0.1890	2.6	0.96	1115.8	26.1	1120.3	18.1	1129.1	15.2	1129.1	15.2
Gleason-354-U1452B-8F-3W-85-150_9May16-Spot 235	52	16700	0.9	12.8654	0.9	1.9487	3.3	0.1818	3.1	0.96	1077.0	31.1	1098.0	21.8	1139.9	17.6	1139.9	17.6
Gleason-354-U1452B-8F-3W-85-150_9May16-Spot 254	341	43669	1.2	12.8145	0.7	2.0073	1.7	0.1866	1.6	0.91	1102.7	15.7	1118.0	11.5	1147.8	13.8	1147.8	13.8
Gleason-354-U1452B-8F-3W-85-150_9May16-Spot 245	1457	449745	3.1	12.7895	0.7	1.8787	1.6	0.1743	1.4	0.90	1035.5	13.4	1073.6	10.3	1151.7	13.5	1151.7	13.5
Gleason-354-U1452B-8F-3W-85-150_9May16-Spot 111	273	60460	7.1	12.7775	0.7	2.0810	2.1	0.1928	1.9	0.94	1136.8	20.3	1142.6	14.2	1153.5	14.0	1153.5	14.0
Gleason-354-U1452B-8F-3W-85-150_9May16-Spot 148	130	16040	1.4	12.7658	0.8	1.8953	2.5	0.1755	2.4	0.94	1042.2	22.9	1079.5	16.8	1155.3	16.6	1155.3	16.6
Gleason-354-U1452B-8F-3W-85-150_9May16-Spot 112	69	6740	1.1	12.6681	0.8	2.0578	3.5	0.1891	3.4	0.98	1116.3	34.8	1134.9	23.8	1170.6	15.2	1170.6	15.2
Gleason-354-U1452B-8F-3W-85-150_9May16-Spot 286	150	332045	2.0	12.6652	0.8	1.9663	2.3	0.1806	2.1	0.94	1070.3	20.9	1104.0	15.2	1171.1	15.4	1171.1	15.4
Gleason-354-U1452B-8F-3W-85-150_9May16-Spot 211	135	64948	1.5	12.6619	0.8	2.1054	2.5	0.1933	2.3	0.94	1139.4	24.2	1150.6	17.0	1171.6	16.7	1171.6	16.7
Gleason-354-U1452B-8F-3W-85-150_9May16-Spot 193	375	21542	1.9	12.6532	0.6	1.8028	1.6	0.1654	1.5	0.92	986.9	13.6	1046.5	10.5	1172.9	12.1	1172.9	12.1
Gleason-354-U1452B-8F-3W-85-150_9May16-Spot 9	427	112341	2.5	12.6270	0.7	2.0130	2.5	0.1843	2.4	0.96	1090.7	24.4	1119.9	17.2	1177.0	14.7	1177.0	14.7
Gleason-354-U1452B-8F-3W-85-150_9May16-Spot 266	73	15745	2.9	12.5548	1.2	2.1712	3.0	0.1977	2.8	0.92	1163.0	29.9	1171.9	21.2	1188.4	23.3	1188.4	23.3
Gleason-354-U1452B-8F-3W-85-150_9May16-Spot 56	752	208537	1.6	12.5365	0.7	2.2192	1.6	0.2018	1.4	0.91	1184.8	15.4	1187.1	11.0	1191.2	13.1	1191.2	13.1
Gleason-354-U1452B-8F-3W-85-150_9May16-Spot 15	1127	174555	26.5	12.0953	0.7	2.2397	1.9	0.1965	1.8	0.93	1156.3	18.6	1193.6	13.3	1261.6	13.8	1261.6	13.8
Gleason-354-U1452B-8F-3W-85-150_9May16-Spot 281	368	443192	1.2	11.8722	0.7	2.5067	1.7	0.2158	1.6	0.92	1259.9	18.0	1274.0	12.4	1297.9	12.7	1297.9	12.7
Gleason-354-U1452B-8F-3W-85-150_9May16-Spot 75	1733	314432	0.7	11.8513	0.7	2.1447	2.1	0.1843	1.9	0.94	1090.7	19.3	1163.3	14.2	1301.3	14.0	1301.3	14.0
Gleason-354-U1452B-8F-3W-85-150_9May16-Spot 194	229	256959	2.5	11.8086	0.6	2.7204	2.0	0.2330	1.9	0.95	1350.2	22.9	1334.1	14.7	1308.3	11.5	1308.3	11.5
Gleason-354-U1452B-8F-3W-85-150_9May16-Spot 270	349	52133	2.1	11.7775	0.7	2.5997	2.6	0.2221	2.5	0.96	1292.8	29.1	1300.6	19.0	1313.4	13.9	1313.4	13.9
Gleason-354-U1452B-8F-3W-85-150_9May16-Spot 109	488	80372	1.2	11.4980	0.9	2.2655	2.4	0.1889	2.3	0.94	1115.6	23.5	1201.6	17.2	1359.9	16.5	1359.9	16.5
Gleason-354-U1452B-8F-3W-85-150_9May16-Spot 37	776	288310	0.9	11.2406	0.6	2.9405	1.8	0.2397	1.7	0.95	1385.3	20.8	1392.4	13.3	1403.3	10.7	1403.3	10.7
Gleason-354-U1452B-8F-3W-85-150_9May16-Spot 179	500	259096	1.0	11.1980	0.5	3.0104	1.7	0.2445	1.6	0.95	1410.0	20.8	1410.3	13.1	1410.6	9.9	1410.6	9.9
Gleason-354-U1452B-8F-3W-85-150_9May16-Spot 130	348	227566	3.2	10.9575	0.7	2.8262	1.6	0.2246	1.5	0.91	1306.1	17.3	1362.5	12.1	1452.0	13.0	1452.0	13.0
Gleason-354-U1452B-8F-3W-85-150_9May16-Spot 293	703	1915474	1.3	10.9318	0.6	2.9193	1.9	0.2315	1.8	0.94	1342.1	21.8	1386.9	14.4	1456.5	11.8	1456.5	11.8
Gleason-354-U1452B-8F-3W-85-150_9May16-Spot 7	146	17264	1.6	10.8557	0.8	3.2297	2.9	0.2543	2.8	0.97	1460.5	36.9	1464.3	22.7	1469.8	14.5	1469.8	14.5
Gleason-354-U1452B-8F-3W-85-150_9May16-Spot 21	123	21897	1.3	10.8467	0.6	3.1616	2.8	0.2487	2.8	0.97	1431.8	35.6	1447.8	21.9	1471.4	12.2	1471.4	12.2
Gleason-354-U1452B-8F-3W-85-150_9May16-Spot 264	130	71479	0.5	10.4554	0.7	3.4817	2.2	0.2640	2.1	0.95	1510.3	28.4	1523.1	17.5	1540.8	12.6	1540.8	12.6
Gleason-354-U1452B-8F-3W-85-150_9May16-Spot 127	192	91239	0.8	10.4296	0.9	3.3761	2.6	0.2554	2.4	0.93	1466.2	31.2	1498.9	20.0	1545.4	17.5	1545.4	17.5

Gleason-354-U1452B-8F-3W-85-150_9May16-Spot 84	112	19610	0.7	10.3950	0.7	3.0989	3.6	0.2336	3.5	0.98	1353.5	42.7	1432.4	27.5	1551.7	13.9	1551.7	13.9
Gleason-354-U1452B-8F-3W-85-150_9May16-Spot 59	1628	507666	8.8	10.3809	0.8	3.6177	2.1	0.2724	1.9	0.92	1552.8	26.1	1553.4	16.4	1554.2	15.3	1554.2	15.3
Gleason-354-U1452B-8F-3W-85-150_9May16-Spot 92	56	9832	0.4	10.3730	1.0	3.6377	3.9	0.2737	3.8	0.97	1559.4	52.2	1557.8	31.1	1555.6	19.2	1555.6	19.2
Gleason-354-U1452B-8F-3W-85-150_9May16-Spot 242	48	8535	1.1	10.3310	1.6	3.6434	3.5	0.2730	3.1	0.88	1556.0	42.6	1559.1	27.7	1563.3	30.5	1563.3	30.5
Gleason-354-U1452B-8F-3W-85-150_9May16-Spot 107	375	98869	1.2	10.2033	0.6	3.6797	1.8	0.2723	1.7	0.95	1552.5	23.9	1567.0	14.6	1586.5	10.9	1586.5	10.9
Gleason-354-U1452B-8F-3W-85-150_9May16-Spot 73	108	52403	1.7	10.1997	0.6	3.7904	2.7	0.2804	2.7	0.97	1593.4	37.5	1590.7	21.9	1587.2	11.9	1587.2	11.9
Gleason-354-U1452B-8F-3W-85-150_9May16-Spot 102	228	46854	1.7	10.1754	0.7	3.7545	2.0	0.2771	1.9	0.94	1576.6	27.0	1583.1	16.4	1591.6	12.7	1591.6	12.7
Gleason-354-U1452B-8F-3W-85-150_9May16-Spot 182	4815	246899	4.7	10.1656	0.6	3.1161	5.2	0.2297	5.2	0.99	1333.1	62.1	1436.7	39.9	1593.5	10.7	1593.5	10.7
Gleason-354-U1452B-8F-3W-85-150_9May16-Spot 295	307	55301	1.2	10.1156	0.7	3.4846	1.9	0.2557	1.8	0.94	1467.6	23.6	1523.7	15.2	1602.6	12.6	1602.6	12.6
Gleason-354-U1452B-8F-3W-85-150_9May16-Spot 238	359	202156	1.8	10.0667	0.8	3.6813	1.9	0.2688	1.7	0.92	1534.6	23.6	1567.3	15.1	1611.7	14.2	1611.7	14.2
Gleason-354-U1452B-8F-3W-85-150_9May16-Spot 234	194	3850893	1.4	10.0656	0.7	3.9216	2.4	0.2863	2.2	0.95	1622.9	32.2	1618.1	19.1	1611.9	13.5	1611.9	13.5
Gleason-354-U1452B-8F-3W-85-150_9May16-Spot 280	81	32132	1.4	10.0387	0.8	3.8362	3.1	0.2793	3.0	0.96	1587.9	41.7	1600.4	24.8	1616.9	15.3	1616.9	15.3
Gleason-354-U1452B-8F-3W-85-150_9May16-Spot 273	224	76149	1.3	10.0273	0.7	3.6557	2.1	0.2659	2.0	0.95	1519.8	26.9	1561.8	16.7	1619.0	12.4	1619.0	12.4
Gleason-354-U1452B-8F-3W-85-150_9May16-Spot 62	236	149861	1.7	9.9807	0.9	3.6745	8.1	0.2660	8.0	0.99	1520.4	108.8	1565.8	64.6	1627.7	16.1	1627.7	16.1
Gleason-354-U1452B-8F-3W-85-150_9May16-Spot 97	316	131351	1.1	9.9617	0.6	3.9246	2.7	0.2835	2.6	0.97	1609.2	37.3	1618.8	21.7	1631.2	11.2	1631.2	11.2
Gleason-354-U1452B-8F-3W-85-150_9May16-Spot 122	773	279708	5.8	9.9038	0.6	3.6862	1.6	0.2648	1.5	0.93	1514.3	19.7	1568.4	12.5	1642.0	10.3	1642.0	10.3
Gleason-354-U1452B-8F-3W-85-150_9May16-Spot 94	312	278354	2.8	9.8930	0.7	3.9365	2.2	0.2824	2.0	0.94	1603.7	29.0	1621.2	17.5	1644.1	13.4	1644.1	13.4
Gleason-354-U1452B-8F-3W-85-150_9May16-Spot 98	1325	409928	0.9	9.8903	0.8	3.8979	1.9	0.2796	1.8	0.92	1589.3	24.9	1613.2	15.5	1644.6	14.2	1644.6	14.2
Gleason-354-U1452B-8F-3W-85-150_9May16-Spot 224	191	149747	1.6	9.8858	0.6	3.9793	2.5	0.2853	2.4	0.97	1618.1	34.3	1630.0	20.1	1645.4	11.4	1645.4	11.4
Gleason-354-U1452B-8F-3W-85-150_9May16-Spot 298	155	519093	1.6	9.8067	0.5	3.9946	2.5	0.2841	2.5	0.98	1612.1	35.3	1633.1	20.5	1660.3	9.5	1660.3	9.5
Gleason-354-U1452B-8F-3W-85-150_9May16-Spot 262	387	42407	4.4	9.8028	0.6	4.2634	2.0	0.3031	1.9	0.96	1706.7	29.0	1686.3	16.6	1661.0	10.3	1661.0	10.3
Gleason-354-U1452B-8F-3W-85-150_9May16-Spot 142	616	132553	14.7	9.7805	0.8	3.9896	1.7	0.2830	1.6	0.89	1606.4	22.2	1632.1	14.2	1665.2	14.5	1665.2	14.5
Gleason-354-U1452B-8F-3W-85-150_9May16-Spot 310	311	43999	2.8	9.7027	0.6	3.9991	2.4	0.2814	2.4	0.97	1598.5	33.5	1634.0	19.8	1680.0	11.2	1680.0	11.2
Gleason-354-U1452B-8F-3W-85-150_9May16-Spot 167	193	40039	1.8	9.5910	0.6	3.8295	3.0	0.2664	3.0	0.98	1522.4	40.2	1599.0	24.4	1701.4	10.9	1701.4	10.9
Gleason-354-U1452B-8F-3W-85-150_9May16-Spot 204	1484	323078	2.1	9.5718	0.6	4.0733	1.7	0.2828	1.6	0.94	1605.3	23.2	1649.0	14.1	1705.1	10.4	1705.1	10.4
Gleason-354-U1452B-8F-3W-85-150_9May16-Spot 158	166	67058	0.6	9.5256	0.6	4.1891	2.2	0.2894	2.1	0.97	1638.6	31.1	1671.9	18.2	1714.0	10.4	1714.0	10.4
Gleason-354-U1452B-8F-3W-85-150_9May16-Spot 44	985	690223	1.2	9.4963	0.6	4.2858	1.5	0.2952	1.3	0.90	1667.4	19.5	1690.6	12.1	1719.6	11.6	1719.6	11.6
Gleason-354-U1452B-8F-3W-85-150_9May16-Spot 101	279	39562	2.3	9.4309	0.6	3.8870	1.5	0.2659	1.4	0.93	1519.8	18.6	1611.0	12.0	1732.3	10.3	1732.3	10.3
Gleason-354-U1452B-8F-3W-85-150_9May16-Spot 291	156	186204	0.8	9.3952	0.7	4.6707	2.3	0.3183	2.2	0.96	1781.3	34.9	1762.0	19.6	1739.3	12.7	1739.3	12.7
Gleason-354-U1452B-8F-3W-85-150_9May16-Spot 110	1148	512423	1.3	9.3170	0.6	4.4899	1.7	0.3034	1.6	0.94	1708.1	24.6	1729.1	14.5	1754.6	10.6	1754.6	10.6
Gleason-354-U1452B-8F-3W-85-150_9May16-Spot 114	391	197169	1.3	9.3019	0.5	4.6638	1.8	0.3146	1.7	0.95	1763.5	25.8	1760.8	14.7	1757.5	9.9	1757.5	9.9
Gleason-354-U1452B-8F-3W-85-150_9May16-Spot 195	242	304538	2.0	9.2960	0.5	4.7429	2.0	0.3198	1.9	0.96	1788.6	29.5	1774.8	16.4	1758.7	9.6	1758.7	9.6
Gleason-354-U1452B-8F-3W-85-150_9May16-Spot 157	255	47108	3.6	9.2848	0.5	4.4458	2.0	0.2994	2.0	0.96	1688.2	29.2	1720.9	16.9	1760.9	10.0	1760.9	10.0
Gleason-354-U1452B-8F-3W-85-150_9May16-Spot 300	170	48125	1.1	9.2670	0.7	4.0110	2.5	0.2696	2.4	0.96	1538.7	32.2	1636.4	19.9	1764.4	12.4	1764.4	12.4
Gleason-354-U1452B-8F-3W-85-150_9May16-Spot 39	1313	314450	4.7	9.2506	0.5	4.4644	1.8	0.2995	1.7	0.96	1689.0	25.4	1724.4	14.7	1767.6	8.7	1767.6	8.7
Gleason-354-U1452B-8F-3W-85-150_9May16-Spot 314	105	794527	1.3	9.1525	0.7	4.6106	2.5	0.3061	2.4	0.96	1721.3	36.4	1751.2	21.0	1787.1	13.6	1787.1	13.6
Gleason-354-U1452B-8F-3W-85-150_9May16-Spot 118	820	1510464	4.7	8.9215	0.5	4.3161	2.0	0.2793	1.9	0.97	1587.7	26.7	1696.4	16.2	1833.5	8.8	1833.5	8.8
Gleason-354-U1452B-8F-3W-85-150_9May16-Spot 51	677	142724	2.2	8.9066	0.6	4.4916	1.6	0.2901	1.5	0.93	1642.2	21.8	1729.4	13.4	1836.6	10.6	1836.6	10.6
Gleason-354-U1452B-8F-3W-85-150_9May16-Spot 106	433	258928	2.1	8.8557	0.7	5.2748	2.0	0.3388	1.9	0.94	1880.9	31.2	1864.8	17.4	1846.9	12.6	1846.9	12.6
Gleason-354-U1452B-8F-3W-85-150_9May16-Spot 187	920	294085	8.5	8.8243	0.9	4.8908	2.1	0.3130	1.8	0.89	1755.5	28.0	1800.7	17.3	1853.4	17.1	1853.4	17.1
Gleason-354-U1452B-8F-3W-85-150_9May16-Spot 203	871	390965	5.5	8.7808	0.7	5.0417	1.8	0.3211	1.7	0.92	1795.0	26.7	1826.4	15.7	1862.3	13.0	1862.3	13.0
Gleason-354-U1452B-8F-3W-85-150_9May16-Spot 45	546	302853	3.3	8.6695	0.8	5.4477	2.1	0.3425	2.0	0.93	1898.8	32.5	1892.4	18.1	1885.3	13.6	1885.3	13.6
Gleason-354-U1452B-8F-3W-85-150_9May16-Spot 100	1723	1220935	15.9	8.1773	0.5	5.7157	1.6	0.3390	1.5	0.94	1881.8	24.4	1933.7	13.7	1989.9	9.7	1989.9	9.7
Gleason-354-U1452B-8F-3W-85-150_9May16-Spot 206	974	291044	2.5	7.4978	0.5	6.9728	1.5	0.3792	1.4	0.94	2072.4	24.5	2108.0	13.1	2142.8	8.9	2142.8	8.9
Gleason-354-U1452B-8F-3W-85-150_9May16-Spot 289	293	149841	2.2	7.4312	0.7	7.5396	2.2	0.4064	2.1	0.95	2198.2	38.8	2177.7	19.6	2158.4	11.4	2158.4	11.4
Gleason-354-U1452B-8F-3W-85-150_9May16-Spot 223	640	968447	2.2	7.3699	0.6	6.8237	1.8	0.3647	1.7	0.94	2004.6	29.6	2088.8	16.2	2172.9	11.0	2172.9	11.0
Gleason-354-U1452B-8F-3W-85-150_9May16-Spot 288	1253	1267196	3.6	6.8396	0.5	7.9393	2.1	0.3938	2.0	0.97	2140.6	37.3	2224.2	19.0	2302.0	8.4	2302.0	8.4
Gleason-354-U1452B-8F-3W-85-150_9May16-Spot 184	278	90321	1.1	6.7847	0.4	8.7937	1.9	0.4327	1.8	0.97	2317.9	36.0	2316.8	17.3	2315.8	7.6	2315.8	7.6
Gleason-354-U1452B-8F-3W-85-150_9May16-Spot 215	2088	7573	2.7	6.7679	1.6	7.7715	2.5	0.3815	1.9	0.77	2083.1	34.2	2204.9	22.4	2320.1	27.2	2320.1	27.2
Gleason-354-U1452B-8F-3W-85-150_9May16-Spot 91	190	267113	1.7	6.3392	0.7	7.8995	2.5	0.3632	2.4	0.96	1997.3	41.3	2219.6	22.5	2431.6	11.1	2431.6	11.1
Gleason-354-U1452B-8F-3W-85-150_9May16-Spot 202	129	34949	1.1	6.2954	0.7	10.2429	2.7	0.4677	2.7	0.97	2473.3	54.8	2457.0	25.4	2443.4	11.2	2443.4	11.2
Gleason-354-U1452B-8F-3W-85-150_9May16-Spot 86	778	531227	1.5	6.2259	0.9	8.8864	3.1	0.4013	2.9	0.95	2174.8	54.4	2326.4	28.2	2462.2	15.8	2462.2	15.8
Gleason-354-U1452B-8F-3W-85-150_9May16-Spot 218	994	227151	2.6	6.2041	0.7	10.3945	2.0	0.4677	1.9	0.95	2473.5	39.8	2470.6	19.0	2468.1	11.1	2468.1	11.1
Gleason-354-U1452B-8F-3W-85-150_9May16-Spot 222	1007	307226	24.2	6.1598	0.7	9.6189	1.9	0.4297	1.7	0.93	2304.5	33.4	2399.0	17.1	2480.2	11.7	2480.2	11.7
Gleason-354-U1452B-8F-3W-85-150_9May16-Spot 175	143	72570	2.5	6.1521	0.9	10.0813	2.9	0.4498	2.8	0.95	2394.5	55.1	2442.3	26.9	2482.3	15.8	2482.3	15.8
Gleason-354-U1452B-8F-3W-85-150_9May16-Spot 116	55	26664	0.7	6.1232	0.7	11.2603	3.8	0.5001	3.7	0.98	2614.1	80.4	2544.9	35.5	2490.2	11.7	2490.2	11.7
Gleason-354-U1452B-8F-3W-85-150_9May16-Spot 131	130	33954	1.2	6.0443	0.7	10.5045	2.4	0.4605	2.3	0.96	2441.7	45.9	2480.3	21.8	2512.1	11.1	2512.1	11.1
Gleason-354-U1452B-8F-3W-85-150_9May16-Spot 87	319	3792362	1.5	6.0110	0.8	10.3110	2.2	0.4495	2.1	0.93	2393.1	41.7	2463.1	20.7	2521.3	13.6	2521.3	13.6
Gleason-354-U1452B-8F-3W-85-150_9May16-Spot 105	398	16091	1.4	6.0002	1.1	10.3115	1.8	0.4487	1.5	0.80	2389.6	29.0	2463.1	16.8	2524.4	18.1	2524.4	18.1
Gleason-354-U1452B-8F-3W-85-150_9May16-Spot 302	699	31317	9.2	5.9695	0.5	10.3370	1.8	0.4475	1.7	0.96	2384.3	33.6	2465.4	16.2	2533.0	8.0	2533.0	8.0
Gleason-354-U1452B-8F-3W-85-150_9May16-Spot 267	504	913110	3.2	5.9330	0.9	10.5656	2.0	0.4546	1.8	0.90	2415.8	35.7	2485.7	18.3	2543.3	14.5	2543.3	14.5
Gleason-354-U1452B-8F-3W-85-150_9May16-Spot 208	279	61963	2.5	5.7791	0.7	11.6042	2.7	0.4864	2.6	0.96	2555.0	54.9	2573.0	25.3	2587.2	12.4	2587.2	12.4
Gleason-354-U1452B-8F-3W-85-150_9May16-Spot 48	194	653262	1.6	5.3736	0.7	12.6049	2.2	0.4913	2.1	0.95	2576.1	45.4	2650.6	21.2	2707.9	11.4	2707.9	11.4
Gleason-354-U1452B-8F-3W-85-150_9May16-Spot 113	239	74772	1.2	5.2205	0.5	14.0711	2.2	0.5328	2.1	0.97	2753.1	47.8	2754.5	20.9	2755.5	9.0	2755.5	9.0
Gleason-354-U1452B-8F-3W-85-150_9May16-Spot 2	553	263687	1.2	5.0875	0.7	14.3253	2.0	0.5286	1.8	0.93	2735.5	40.5	2771.5	18.6	2797.8	12.1	2797.8	12.1
Gleason-354-U1452B-8F-3W-85-150_9May16-Spot 257	159	145035	2.0	5.0172	0.7	15.2239	2.2	0.5540	2.0	0.94	2841.7	46.7	2829.3	20.6	2820.6	11.8	2820.6	11.8

Table ______. U-Pb geochronologic analyses.

#4 U1453A_11F

Mid Pleistocene 0.5 Ma

Analysis	U (ppm)	206Pb/204Pb	U/Th	206Pb*/207Pb*	± (%)	207Pb*/235U*	± (%)	206Pb*/238U	± (%)	error corr.	206Pb*/238U*	± (Ma)	207Pb*/235U	± (Ma)	206Pb*/207Pb*	± (Ma)	Best age (Ma)	± (Ma)
						Isotope ratios						Apparent ages (Ma)						
Gleason-354-U1453A-11F-1W-88-135-Spot 31	831	924	260.5	23.6819	20.1	0.0036	20.4	0.0006	3.2	0.16	4.0	0.1	3.7	0.8	213.0	510.3	4.0	0.1
Gleason-354-U1453A-11F-1W-88-135-Spot 197	2679	13277	109.9	20.7342	1.7	0.0083	2.7	0.0013	2.1	0.76	8.1	0.2	8.4	0.2	110.6	41.3	8.1	0.2
Gleason-354-U1453A-11F-1W-88-135-Spot 199	841	1225	16.6	28.1376	3.0	0.0154	4.3	0.0031	3.1	0.72	20.2	0.6	15.5	0.7	666.3	82.6	20.2	0.6
Gleason-354-U1453A-11F-1W-88-135-Spot 124	1605	11282	83.5	21.6964	1.8	0.0203	2.4	0.0032	1.7	0.68	20.6	0.3	20.4	0.5	2.4	43.1	20.6	0.3
Gleason-354-U1453A-11F-1W-88-135-Spot 1	613	1145	14.3	29.8486	3.9	0.0159	4.5	0.0034	2.3	0.50	22.2	0.5	16.0	0.7	831.9	112.2	22.2	0.5
Gleason-354-U1453A-11F-1W-88-135-Spot 244	465	6953	2.0	21.9226	2.3	0.0256	3.0	0.0041	1.8	0.62	26.2	0.5	25.7	0.8	22.7	56.2	26.2	0.5
Gleason-354-U1453A-11F-1W-88-135-Spot 209	767	8440	1.4	21.0823	1.6	0.0287	2.6	0.0044	2.0	0.78	28.2	0.6	28.7	0.7	71.1	38.7	28.2	0.6
Gleason-354-U1453A-11F-1W-88-135-Spot 64	7594	37665	0.1	21.2822	0.7	0.0285	3.4	0.0044	3.4	0.98	28.3	0.9	28.5	1.0	48.6	17.0	28.3	0.9
Gleason-354-U1453A-11F-1W-88-135-Spot 78	998	4629	2.1	22.3896	2.2	0.0277	2.9	0.0045	1.9	0.64	28.9	0.5	27.7	0.8	73.9	54.5	28.9	0.5
Gleason-354-U1453A-11F-1W-88-135-Spot 129	1432	21825	1.7	20.9962	1.4	0.0336	2.9	0.0051	2.5	0.87	32.9	0.8	33.6	0.9	80.8	33.4	32.9	0.8
Gleason-354-U1453A-11F-1W-88-135-Spot 216	1701	5041	165.1	22.6502	1.3	0.0354	2.4	0.0058	2.0	0.84	37.3	0.7	35.3	0.8	102.3	31.3	37.3	0.7
Gleason-354-U1453A-11F-1W-88-135-Spot 35	452	1580	1.3	24.4899	5.8	0.0331	6.1	0.0059	1.8	0.29	37.7	0.7	33.0	2.0	298.0	148.7	37.7	0.7
Gleason-354-U1453A-11F-1W-88-135-Spot 226	189	12532	8.4	21.6314	3.3	0.0397	4.8	0.0062	3.5	0.73	40.0	1.4	39.5	1.9	9.6	79.1	40.0	1.4
Gleason-354-U1453A-11F-1W-88-135-Spot 261	2193	12784	18.6	21.3990	0.9	0.0471	2.3	0.0073	2.1	0.92	47.0	1.0	46.8	1.0	35.5	21.8	47.0	1.0
Gleason-354-U1453A-11F-1W-88-135-Spot 313	1549	110774	2.3	21.0686	1.1	0.0479	4.7	0.0073	4.6	0.97	47.1	2.1	47.6	2.2	72.6	25.0	47.1	2.1
Gleason-354-U1453A-11F-1W-88-135-Spot 234	267	2002	0.5	22.6913	4.1	0.0457	4.6	0.0075	2.1	0.46	48.3	1.0	45.4	2.0	106.8	100.2	48.3	1.0
Gleason-354-U1453A-11F-1W-88-135-Spot 282	1195	10916	0.9	21.3476	1.3	0.0496	2.7	0.0077	2.4	0.88	49.3	1.2	49.1	1.3	41.3	30.4	49.3	1.2
Gleason-354-U1453A-11F-1W-88-135-Spot 52	599	3719	9.2	21.4754	1.8	0.0494	3.2	0.0077	2.6	0.82	49.5	1.3	49.0	1.5	26.9	44.3	49.5	1.3
Gleason-354-U1453A-11F-1W-88-135-Spot 183	303	2079	0.9	24.6463	2.5	0.0439	3.8	0.0079	2.8	0.74	50.4	1.4	43.7	1.6	314.3	65.0	50.4	1.4
Gleason-354-U1453A-11F-1W-88-135-Spot 38	2645	20753	1.8	20.2530	1.8	0.0573	2.5	0.0084	1.7	0.69	54.1	0.9	56.6	1.4	165.7	42.1	54.1	0.9
Gleason-354-U1453A-11F-1W-88-135-Spot 65	288	8339	1.1	20.3908	1.8	0.0605	3.4	0.0090	2.8	0.84	57.5	1.6	59.7	1.9	149.8	42.7	57.5	1.6
Gleason-354-U1453A-11F-1W-88-135-Spot 270	465	7410	6.2	20.4448	2.3	0.0611	4.2	0.0091	3.5	0.84	58.1	2.0	60.2	2.4	143.6	53.0	58.1	2.0
Gleason-354-U1453A-11F-1W-88-135-Spot 205	429	20154	1.8	20.6897	1.9	0.0606	2.8	0.0091	2.1	0.74	58.4	1.2	59.8	1.7	115.6	44.9	58.4	1.2
Gleason-354-U1453A-11F-1W-88-135-Spot 51	2856	18701	1.4	20.9081	0.8	0.0602	2.4	0.0091	2.3	0.94	58.6	1.3	59.3	1.4	90.8	20.0	58.6	1.3
Gleason-354-U1453A-11F-1W-88-135-Spot 173	1132	17542	4.2	21.1340	1.6	0.0602	2.2	0.0092	1.6	0.70	59.2	0.9	59.3	1.3	65.3	37.6	59.2	0.9
Gleason-354-U1453A-11F-1W-88-135-Spot 175	152	3697	1.5	21.5411	2.9	0.0603	4.2	0.0094	3.0	0.71	60.4	1.8	59.5	2.4	19.6	70.7	60.4	1.8
Gleason-354-U1453A-11F-1W-88-135-Spot 42	844	5336	1.4	19.1091	2.3	0.0685	3.3	0.0095	2.4	0.73	60.9	1.5	67.3	2.2	299.9	52.3	60.9	1.5
Gleason-354-U1453A-11F-1W-88-135-Spot 239	3652	324352	1.9	20.6998	0.8	0.0649	2.3	0.0097	2.2	0.94	62.5	1.3	63.8	1.4	114.5	19.2	62.5	1.3
Gleason-354-U1453A-11F-1W-88-135-Spot 269	327	4078	1.2	14.4346	4.5	0.1002	5.0	0.0105	2.1	0.42	67.3	1.4	96.9	4.6	907.0	93.5	67.3	1.4
Gleason-354-U1453A-11F-1W-88-135-Spot 228	2501	26462	1.6	20.6015	0.9	0.0764	2.4	0.0114	2.3	0.93	73.2	1.6	74.8	1.8	125.7	21.4	73.2	1.6
Gleason-354-U1453A-11F-1W-88-135-Spot 87	1347	13692	1.9	21.4786	1.2	0.0758	2.5	0.0118	2.1	0.87	75.6	1.6	74.2	1.8	26.6	28.7	75.6	1.6
Gleason-354-U1453A-11F-1W-88-135-Spot 277	105	1872	1.5	20.6016	3.0	0.0807	4.9	0.0121	3.9	0.79	77.2	3.0	78.8	3.7	125.6	71.1	77.2	3.0
Gleason-354-U1453A-11F-1W-88-135-Spot 184	407	4658	1.9	22.3120	1.7	0.0810	2.7	0.0131	2.0	0.76	83.9	1.7	79.0	2.0	65.5	42.5	83.9	1.7
Gleason-354-U1453A-11F-1W-88-135-Spot 6	236	1414	1.5	26.3273	1.9	0.0704	3.2	0.0134	2.6	0.82	86.1	2.3	69.1	2.2	486.4	49.7	86.1	2.3
Gleason-354-U1453A-11F-1W-88-135-Spot 257	346	1894	1.2	19.0195	4.3	0.0978	4.9	0.0135	2.3	0.46	86.4	1.9	94.8	4.4	310.6	98.7	86.4	1.9
Gleason-354-U1453A-11F-1W-88-135-Spot 161	589	7250	2.3	21.4858	1.4	0.0893	3.1	0.0139	2.7	0.89	89.1	2.4	86.9	2.6	25.8	33.6	89.1	2.4
Gleason-354-U1453A-11F-1W-88-135-Spot 170	79	8076	1.1	17.9253	3.3	0.1145	4.8	0.0149	3.5	0.73	95.2	3.3	110.1	5.0	443.9	72.4	95.2	3.3
Gleason-354-U1453A-11F-1W-88-135-Spot 231	162	4094	1.1	17.4809	5.0	0.1190	5.9	0.0151	3.1	0.53	96.5	3.0	114.1	6.4	499.4	110.3	96.5	3.0
Gleason-354-U1453A-11F-1W-88-135-Spot 110	89	1196	1.2	26.8768	13.8	0.0795	14.2	0.0155	3.3	0.24	99.1	3.3	77.7	10.6	541.5	371.7	99.1	3.3
Gleason-354-U1453A-11F-1W-88-135-Spot 215	867	15250	10.4	20.1003	1.1	0.1110	2.5	0.0162	2.3	0.90	103.5	2.3	106.9	2.6	183.3	26.0	103.5	2.3
Gleason-354-U1453A-11F-1W-88-135-Spot 179	844	3642	1.2	19.7789	2.5	0.1134	3.2	0.0163	2.1	0.64	104.0	2.1	109.0	3.3	220.8	57.4	104.0	2.1
Gleason-354-U1453A-11F-1W-88-135-Spot 136	47	2676	1.7	21.3303	3.2	0.1074	5.9	0.0166	4.9	0.84	106.2	5.2	103.6	5.8	43.2	77.7	106.2	5.2
Gleason-354-U1453A-11F-1W-88-135-Spot 83	2531	37692	0.6	20.3849	0.9	0.1163	2.3	0.0172	2.1	0.91	109.9	2.2	111.8	2.4	150.5	21.7	109.9	2.2
Gleason-354-U1453A-11F-1W-88-135-Spot 210	200	5292	1.1	21.2807	1.7	0.1136	2.6	0.0175	1.9	0.74	112.0	2.1	109.2	2.6	48.7	41.0	112.0	2.1
Gleason-354-U1453A-11F-1W-88-135-Spot 229	293	17200	1.5	20.7142	1.7	0.1169	2.9	0.0176	2.3	0.80	112.2	2.6	112.2	3.1	112.8	40.9	112.2	2.6
Gleason-354-U1453A-11F-1W-88-135-Spot 174	763	8055	0.8	19.6116	2.6	0.1243	3.3	0.0177	2.1	0.63	112.9	2.3	118.9	3.8	240.4	60.2	112.9	2.3
Gleason-354-U1453A-11F-1W-88-135-Spot 90	1024	21041	0.9	20.5501	1.1	0.1187	2.2	0.0177	2.0	0.88	113.0	2.2	113.9	2.4	131.6	24.8	113.0	2.2
Gleason-354-U1453A-11F-1W-88-135-Spot 238	1014	6412	1.1	22.0181	1.0	0.1108	2.2	0.0177	1.9	0.88	113.1	2.2	106.7	2.2	33.2	24.9	113.1	2.2
Gleason-354-U1453A-11F-1W-88-135-Spot 172	503	139586	0.6	20.4757	1.3	0.1194	2.4	0.0177	2.1	0.86	113.3	2.3	114.5	2.6	140.1	29.4	113.3	2.3
Gleason-354-U1453A-11F-1W-88-135-Spot 268	1307	23818	1.5	20.6169	0.9	0.1192	2.0	0.0178	1.8	0.89	113.9	2.0	114.4	2.2	123.9	21.8	113.9	2.0
Gleason-354-U1453A-11F-1W-88-135-Spot 86	691	22375	1.5	20.5568	1.5	0.1199	2.5	0.0179	2.0	0.80	114.2	2.3	114.9	2.7	130.8	35.4	114.2	2.3
Gleason-354-U1453A-11F-1W-88-135-Spot 258	607	8192	1.3	21.1266	1.3	0.1166	2.4	0.0179	1.9	0.83	114.2	2.2	112.0	2.5	66.1	31.5	114.2	2.2
Gleason-354-U1453A-11F-1W-88-135-Spot 24	1148	102101	1.2	20.1834	1.1	0.1250	2.6	0.0183	2.3	0.90	116.9	2.7	119.6	2.9	173.7	25.7	116.9	2.7
Gleason-354-U1453A-11F-1W-88-135-Spot 211	823	7909	1.5	19.6431	1.9	0.1302	2.4	0.0185	1.5	0.63	118.4	1.8	124.2	2.8	236.7	43.3	118.4	1.8
Gleason-354-U1453A-11F-1W-88-135-Spot 66	445	39690	1.6	19.8519	1.3	0.1289	2.1	0.0186	1.7	0.79	118.6	2.0	123.1	2.5	212.2	30.7	118.6	2.0
Gleason-354-U1453A-11F-1W-88-135-Spot 58	1495	24312	4.1	20.6605	0.9	0.1268	2.1	0.0190	1.9	0.91	121.3	2.3	121.2	2.4	118.9	20.8	121.3	2.3
Gleason-354-U1453A-11F-1W-88-135-Spot 11	617	13328	0.9	20.6569	1.1	0.1270	2.6	0.0190	2.4	0.91	121.5	2.9	121.4	3.0	119.4	26.2	121.5	2.9
Gleason-354-U1453A-11F-1W-88-135-Spot 37	229	11973	0.7	20.8726	1.8	0.1267	2.7	0.0192	2.0	0.73	122.4	2.4	121.1	3.1	94.8	43.4	122.4	2.4
Gleason-354-U1453A-11F-1W-88-135-Spot 251	231	6068	0.8	21.5454	2.5	0.1245	4.2	0.0194	3.3	0.79	124.2	4.1	119.1	4.7	19.2	61.1	124.2	4.1
Gleason-354-U1453A-11F-1W-88-135-Spot 63	568	8062	1.9	21.2133	1.1	0.1282	2.7	0.0197	2.4	0.90	125.9	3.0	122.5	3.1	56.3	27.3	125.9	3.0
Gleason-354-U1453A-11F-1W-88-135-Spot 189	491	26592	1.2	20.8121	1.2	0.1329	2.6	0.0201	2.4	0.90	128.0	3.0	126.7	3.2	101.7	27.6	128.0	3.0
Gleason-354-U1453A-11F-1W-88-135-Spot 163	484	5565	1.0	20.8348	1.4	0.1340	2.5	0.0202	2.0	0.83	129.2	2.6	127.7	2.9	99.1	32.8	129.2	2.6
Gleason-354-U1453A-11F-1W-88-135-Spot 203	468	5930	1.9	21.1891	2.8	0.1368	3.6	0.0210	2.3	0.64	134.1	3.0	130.2	4.4	59.1	66.1	134.1	3.0
Gleason-354-U1453A-11F-1W-88-135-Spot 67	536	13746	1.2	19.1604	1.4	0.1521	2.5	0.0211	2.1	0.82	134.8	2.8	143.8	3.4	293.8	32.9	134.8	2.8
Gleason-354-U1453A-11F-1W-88-135-Spot 8	689	12539	33.9	11.9111	5.7	0.2502	8.6	0.0216	6.4	0.75	137.9	8.8	226.8	17.4	1291.5	110.6	137.9	8.8
Gleason-354-U1453A-11F-1W-88-135-Spot 57	1438	30938	11.8	19.0279	1.5	0.1576	2.6	0.0218	2.1	0.81	138.7	2.8	148.6	3.5	309.6	33.9	138.7	2.8
Gleason-354-U1453A-11F-1W-88-135-Spot 263	1174	21481	2.3	20.4687	0.7	0.1475	2.1	0.0219	1.9	0.94	139.6	2.7	139.7	2.7	140.9	16.3	139.6	2.7
Gleason-354-U1453A-11F-1W-88-135-Spot 62	581	35931	4.5	19.8451	0.8	0.1529	2.0	0.0220	1.9	0.92	140.3	2.6	144.5	2.7	213.0	18.5	140.3	2.6
Gleason-354-U1453A-11F-1W-88-135-Spot 59	717	12530	0.8	20.7734	1.2	0.1525	2.4	0.0230	2.1	0.87	146.5	3.0	144.1	3.2	106.1	27.8	146.5	3.0
Gleason-354-U1453A-11F-1W-88-135-Spot 47	190	3951	1.2	20.2395	2.4	0.1582	3.5	0.0232	2.5	0.72	147.9	3.6	149.1	4.8	167.2	56.7	147.9	3.6
Gleason-354-U1453A-11F-1W-88-135-Spot 299	2148	71595	2.1	20.1259	0.6	0.1620	1.8	0.0236	1.7	0.93	150.6	2.5	152.4	2.5	180.4	14.7	150.6	2.5
Gleason-354-U1453A-11F-1W-88-135-Spot 74	417	19052	1.4	20.4066	1.0	0.1874	2.5	0.0277	2.3	0.91	176.3	4.0	174.4	4.1	148.0	24.6	176.3	4.0
Gleason-354-U1453A-11F-1W-88-135-Spot 188	1391	37104	1.2	20.1468	0.5	0.1916	1.8	0.0280	1.7	0.96	178.0	3.0	178.0	3.0	177.9	11.8	178.0	3.0
Gleason-354-U1453A-11F-1W-88-135-Spot 166	19	679	2.9	27.4227	28.1	0.1439	28.6	0.0286	5.4	0.19	181.9	9.8	136.5	36.6	595.8	775.2	181.9	9.8
Gleason-354-U1453A-11F-1W-88-135-Spot 49	388	9446	1.0	20.5104	1.1	0.1925	2.5	0.0286	2.2	0.90	182.0	4.0	178.7	4.1	136.1	25.2	182.0	4.0
Gleason-354-U1453A-11F-1W-88-135-Spot 201	94	467	0.5	5.5048	1.0	0.7294	3.0	0.0291	2.8	0.94	185.0	5.2	556.2	12.9	2668.1	16.5	185.0	5.2
Gleason-354-U1453A-11F-1W-88-135-Spot 55	345	9918	1.3	19.9165	1.1	0.2054	2.7	0.0297	2.5	0.91	188.5	4.6	189.7	4.7	204.7	26.1	188.5	4.6
Gleason-354-U1453A-11F-1W-88-135-Spot 15	3741	######	43.8	19.3352	1.1	0.2123	7.7	0.0298	7.6	0.99	189.1	14.3	195.5	13.7	273.0	25.3	189.1	14.3
Gleason-354-U1453A-11F-1W-88-135-Spot 266	859	22400	1.8	20.0417	0.8	0.2116	2.5	0.0308	2.4	0.95	195.3	4.6	194.9	4.5	190.2	18.1	195.3	4.6

Gleason-354-U1453A-11F-1W-88-135-Spot 240	589	18474	7.6	19.8465	2.4	0.2179	4.5	0.0314	3.8	0.85	199.1	7.5	200.2	8.2	212.9	54.8	199.1	7.5
Gleason-354-U1453A-11F-1W-88-135-Spot 279	673	19397	4.5	19.9570	0.8	0.2212	2.1	0.0320	1.9	0.92	203.2	3.8	202.9	3.8	200.0	18.3	203.2	3.8
Gleason-354-U1453A-11F-1W-88-135-Spot 122	1768	123188	4.2	17.1554	0.6	0.2674	2.7	0.0333	2.6	0.97	211.0	5.4	240.6	5.7	540.7	13.4	211.0	5.4
Gleason-354-U1453A-11F-1W-88-135-Spot 116	380	10168	1.7	19.9904	1.4	0.2333	2.3	0.0338	1.8	0.79	214.5	3.8	212.9	4.4	196.1	32.2	214.5	3.8
Gleason-354-U1453A-11F-1W-88-135-Spot 223	1199	62932	53.7	17.6706	1.0	0.2648	3.9	0.0339	3.8	0.97	215.2	8.0	238.6	8.2	475.6	21.3	215.2	8.0
Gleason-354-U1453A-11F-1W-88-135-Spot 54	250	7115	1.5	19.8949	1.2	0.2608	2.8	0.0376	2.5	0.90	238.1	5.9	235.3	5.9	207.2	28.4	238.1	5.9
Gleason-354-U1453A-11F-1W-88-135-Spot 34	876	14031	1.5	18.8006	1.0	0.2879	2.1	0.0392	1.8	0.88	248.2	4.5	256.9	4.8	336.9	22.7	248.2	4.5
Gleason-354-U1453A-11F-1W-88-135-Spot 5	1365	33350	32.4	17.3649	0.7	0.3201	2.0	0.0403	1.9	0.94	254.8	4.8	282.0	5.0	514.1	15.2	254.8	4.8
Gleason-354-U1453A-11F-1W-88-135-Spot 202	850	######	3.4	17.4784	0.9	0.3281	3.3	0.0416	3.2	0.96	262.7	8.2	288.1	8.3	499.8	20.8	262.7	8.2
Gleason-354-U1453A-11F-1W-88-135-Spot 26	1121	39126	6.4	18.8344	0.6	0.3134	2.6	0.0428	2.6	0.97	270.2	6.8	276.8	6.4	332.8	14.0	270.2	6.8
Gleason-354-U1453A-11F-1W-88-135-Spot 293	2661	347133	2.3	17.5681	0.7	0.4355	5.1	0.0555	5.1	0.99	348.2	17.3	367.1	15.8	488.5	15.5	348.2	17.3
Gleason-354-U1453A-11F-1W-88-135-Spot 25	1176	52294	1.2	17.5045	0.7	0.4489	3.9	0.0570	3.8	0.98	357.3	13.4	376.5	12.3	496.5	15.7	357.3	13.4
Gleason-354-U1453A-11F-1W-88-135-Spot 314	1098	34448	1.4	17.2281	0.8	0.4683	2.4	0.0585	2.3	0.95	366.6	8.1	390.0	7.7	531.5	16.8	366.6	8.1
Gleason-354-U1453A-11F-1W-88-135-Spot 61	2364	238496	1.0	17.4496	0.7	0.4807	3.1	0.0608	3.1	0.98	380.7	11.4	398.6	10.4	503.4	14.6	380.7	11.4
Gleason-354-U1453A-11F-1W-88-135-Spot 213	1098	590483	5.6	16.8011	0.5	0.5228	2.2	0.0637	2.1	0.97	398.1	8.1	427.0	7.5	586.2	11.9	398.1	8.1
Gleason-354-U1453A-11F-1W-88-135-Spot 255	6725	326903	6.7	17.5178	0.5	0.5373	2.1	0.0683	2.0	0.97	425.7	8.4	436.6	7.4	494.8	10.5	425.7	8.4
Gleason-354-U1453A-11F-1W-88-135-Spot 125	282	13748	2.4	17.2978	1.1	0.5552	5.0	0.0697	4.9	0.97	434.1	20.6	448.4	18.3	522.6	24.9	434.1	20.6
Gleason-354-U1453A-11F-1W-88-135-Spot 88	1859	179914	1.6	17.2393	0.7	0.5576	2.3	0.0697	2.2	0.95	434.4	9.4	449.9	8.5	530.0	15.9	434.4	9.4
Gleason-354-U1453A-11F-1W-88-135-Spot 131	771	81279	2.6	17.3261	0.8	0.5817	2.1	0.0731	1.9	0.93	454.8	8.4	465.5	7.7	519.0	17.0	454.8	8.4
Gleason-354-U1453A-11F-1W-88-135-Spot 137	1253	148372	1.7	17.4893	0.7	0.5815	2.3	0.0738	2.2	0.95	458.8	9.6	465.4	8.5	498.4	15.6	458.8	9.6
Gleason-354-U1453A-11F-1W-88-135-Spot 127	1517	190266	2.2	17.5198	0.6	0.5833	1.8	0.0741	1.7	0.95	460.9	7.4	466.6	6.6	494.5	12.3	460.9	7.4
Gleason-354-U1453A-11F-1W-88-135-Spot 80	1750	87353	1.1	17.1723	0.5	0.5958	1.7	0.0742	1.6	0.95	461.4	7.1	474.6	6.3	538.6	11.5	461.4	7.1
Gleason-354-U1453A-11F-1W-88-135-Spot 45	1956	89622	3.9	16.9351	1.0	0.6115	2.0	0.0751	1.8	0.87	466.9	8.0	484.5	7.8	568.9	21.7	466.9	8.0
Gleason-354-U1453A-11F-1W-88-135-Spot 33	525	335332	1.8	17.2631	0.7	0.6032	2.3	0.0755	2.2	0.95	469.4	9.8	479.3	8.7	527.0	16.2	469.4	9.8
Gleason-354-U1453A-11F-1W-88-135-Spot 28	363	23359	4.3	17.6043	0.8	0.5986	2.1	0.0764	1.9	0.91	474.8	8.6	476.4	7.8	484.0	18.7	474.8	8.6
Gleason-354-U1453A-11F-1W-88-135-Spot 273	438	20053	1.2	17.4949	0.9	0.6054	2.2	0.0768	2.0	0.92	477.1	9.4	480.7	8.5	497.7	19.0	477.1	9.4
Gleason-354-U1453A-11F-1W-88-135-Spot 220	1867	96650	1.1	17.3435	0.7	0.6165	1.8	0.0775	1.6	0.92	481.4	7.6	487.6	6.9	516.8	15.4	481.4	7.6
Gleason-354-U1453A-11F-1W-88-135-Spot 160	185	53864	0.7	17.1230	0.9	0.6259	2.7	0.0777	2.6	0.94	482.6	12.0	493.6	10.7	544.8	20.2	482.6	12.0
Gleason-354-U1453A-11F-1W-88-135-Spot 142	1295	501405	2.5	17.2600	0.6	0.6243	1.7	0.0782	1.6	0.94	485.1	7.3	492.6	6.5	527.4	12.9	485.1	7.3
Gleason-354-U1453A-11F-1W-88-135-Spot 206	363	88983	1.0	17.6510	0.9	0.6219	2.5	0.0796	2.3	0.93	493.8	11.1	491.0	9.8	478.1	20.0	493.8	11.1
Gleason-354-U1453A-11F-1W-88-135-Spot 141	751	68989	5.6	17.3188	0.8	0.6377	2.6	0.0801	2.5	0.95	496.7	11.7	500.9	10.2	519.9	17.6	496.7	11.7
Gleason-354-U1453A-11F-1W-88-135-Spot 146	1787	132651	3.4	17.3631	0.6	0.6393	2.2	0.0805	2.1	0.95	499.2	9.9	501.9	8.5	514.3	14.1	499.2	9.9
Gleason-354-U1453A-11F-1W-88-135-Spot 219	196	23546	0.2	17.2616	0.9	0.6433	2.5	0.0805	2.3	0.93	499.3	11.0	504.4	9.8	527.2	19.5	499.3	11.0
Gleason-354-U1453A-11F-1W-88-135-Spot 60	3124	61244	78.2	16.6750	0.7	0.6662	1.9	0.0806	1.8	0.92	499.5	8.6	518.4	7.9	602.5	16.1	499.5	8.6
Gleason-354-U1453A-11F-1W-88-135-Spot 218	588	41721	1.9	17.3761	0.7	0.6405	2.3	0.0807	2.2	0.95	500.4	10.6	502.6	9.2	512.7	15.3	500.4	10.6
Gleason-354-U1453A-11F-1W-88-135-Spot 248	556	28345	4.8	17.5985	1.0	0.6328	2.3	0.0808	2.1	0.90	500.7	10.1	497.8	9.1	484.7	21.7	500.7	10.1
Gleason-354-U1453A-11F-1W-88-135-Spot 283	791	59598	1.2	17.2376	0.8	0.6494	2.2	0.0812	2.0	0.93	503.2	9.7	508.1	8.6	530.3	17.6	503.2	9.7
Gleason-354-U1453A-11F-1W-88-135-Spot 155	464	31500	2.6	17.4164	0.6	0.6434	2.4	0.0813	2.3	0.97	503.7	11.2	504.4	9.5	507.6	13.2	503.7	11.2
Gleason-354-U1453A-11F-1W-88-135-Spot 235	481	60813	3.3	17.4156	0.6	0.6459	2.4	0.0816	2.3	0.97	505.6	11.1	506.0	9.4	507.7	13.4	505.6	11.1
Gleason-354-U1453A-11F-1W-88-135-Spot 290	258	24718	2.6	17.2350	0.8	0.6547	1.5	0.0818	1.3	0.87	507.1	6.5	511.4	6.2	530.6	16.5	507.1	6.5
Gleason-354-U1453A-11F-1W-88-135-Spot 233	1021	737884	1.5	17.2230	0.6	0.6552	1.7	0.0818	1.6	0.94	507.1	7.6	511.7	6.7	532.1	12.4	507.1	7.6
Gleason-354-U1453A-11F-1W-88-135-Spot 250	1169	50923	2.1	17.5279	0.7	0.6496	1.9	0.0826	1.8	0.94	511.5	9.0	508.2	7.8	493.5	14.6	511.5	9.0
Gleason-354-U1453A-11F-1W-88-135-Spot 191	122	259791	0.4	16.5484	1.1	0.6912	2.7	0.0830	2.4	0.92	513.8	12.1	533.5	11.0	618.9	22.8	513.8	12.1
Gleason-354-U1453A-11F-1W-88-135-Spot 70	180	######	1.6	17.4084	0.9	0.6608	2.5	0.0834	2.4	0.94	516.5	11.8	515.1	10.2	508.6	19.5	516.5	11.8
Gleason-354-U1453A-11F-1W-88-135-Spot 123	672	765262	1.5	17.2159	0.6	0.6767	2.0	0.0845	1.9	0.95	522.9	9.3	524.8	8.1	533.0	14.0	522.9	9.3
Gleason-354-U1453A-11F-1W-88-135-Spot 187	562	176442	2.0	17.3187	0.7	0.6833	1.8	0.0858	1.7	0.93	530.8	8.7	528.8	7.6	519.9	14.5	530.8	8.7
Gleason-354-U1453A-11F-1W-88-135-Spot 190	954	36377	1.4	17.2860	0.5	0.6904	1.9	0.0866	1.8	0.96	535.2	9.4	533.1	7.9	524.1	11.9	535.2	9.4
Gleason-354-U1453A-11F-1W-88-135-Spot 154	944	150890	53.8	16.7695	0.7	0.7193	1.7	0.0875	1.5	0.91	540.6	8.0	550.3	7.2	590.3	14.9	540.6	8.0
Gleason-354-U1453A-11F-1W-88-135-Spot 77	444	######	2.9	16.9870	1.0	0.7246	2.4	0.0893	2.1	0.91	551.2	11.3	553.4	10.1	562.2	21.7	551.2	11.3
Gleason-354-U1453A-11F-1W-88-135-Spot 272	227	######	2.0	16.5806	0.6	0.7445	2.3	0.0895	2.3	0.96	552.8	12.0	565.1	10.2	614.7	13.5	552.8	12.0
Gleason-354-U1453A-11F-1W-88-135-Spot 297	444	26460	3.2	16.7225	0.8	0.7452	2.6	0.0904	2.5	0.95	557.8	13.1	565.5	11.2	596.3	17.7	557.8	13.1
Gleason-354-U1453A-11F-1W-88-135-Spot 93	343	81620	1.4	16.6786	0.6	0.7490	2.2	0.0906	2.2	0.97	559.1	11.5	567.7	9.7	602.0	12.3	559.1	11.5
Gleason-354-U1453A-11F-1W-88-135-Spot 247	543	148538	10.3	17.0518	0.9	0.7368	2.6	0.0911	2.4	0.94	562.2	13.1	560.5	11.2	553.9	19.2	562.2	13.1
Gleason-354-U1453A-11F-1W-88-135-Spot 108	401	37317	19.5	16.1857	0.8	0.7844	1.9	0.0921	1.7	0.90	567.8	9.0	588.0	8.3	666.6	17.7	567.8	9.0
Gleason-354-U1453A-11F-1W-88-135-Spot 300	262	21789	1.4	17.0055	1.0	0.7572	2.3	0.0934	2.1	0.91	575.6	11.6	572.4	10.1	559.8	20.7	575.6	11.6
Gleason-354-U1453A-11F-1W-88-135-Spot 130	1093	######	2.7	16.5134	0.8	0.8076	2.0	0.0967	1.9	0.92	595.2	10.6	601.1	9.2	623.5	17.4	595.2	10.6
Gleason-354-U1453A-11F-1W-88-135-Spot 3	351	57871	1.6	15.7890	0.8	0.8460	3.1	0.0969	2.9	0.96	596.1	16.7	622.4	14.2	719.5	17.7	596.1	16.7
Gleason-354-U1453A-11F-1W-88-135-Spot 180	597	92245	7.2	16.3325	0.7	0.8774	2.0	0.1039	1.9	0.94	637.4	11.3	639.6	9.3	647.3	14.2	637.4	11.3
Gleason-354-U1453A-11F-1W-88-135-Spot 253	912	276020	3.1	16.0582	0.8	0.9498	2.4	0.1106	2.2	0.95	676.4	14.3	678.0	11.6	683.5	16.3	676.4	14.3
Gleason-354-U1453A-11F-1W-88-135-Spot 151	585	230663	4.2	15.4947	0.7	1.0739	2.3	0.1207	2.2	0.94	734.5	14.9	740.7	12.0	759.3	15.7	734.5	14.9
Gleason-354-U1453A-11F-1W-88-135-Spot 309	3375	453616	0.9	15.0182	0.6	1.1542	1.7	0.1257	1.6	0.93	763.4	11.7	779.2	9.5	824.9	13.3	763.4	11.7
Gleason-354-U1453A-11F-1W-88-135-Spot 186	1854	207773	5.3	15.2899	0.6	1.1375	1.9	0.1261	1.8	0.95	765.8	13.0	771.3	10.2	787.3	12.3	765.8	13.0
Gleason-354-U1453A-11F-1W-88-135-Spot 101	966	61767	1.5	15.3554	0.6	1.1407	1.9	0.1270	1.8	0.95	771.0	13.0	772.9	10.2	778.3	11.8	771.0	13.0
Gleason-354-U1453A-11F-1W-88-135-Spot 167	234	23240	3.2	14.3449	0.8	1.2283	2.2	0.1278	2.1	0.93	775.2	15.2	813.5	12.6	919.9	17.2	775.2	15.2
Gleason-354-U1453A-11F-1W-88-135-Spot 36	199	37119	2.5	15.0944	1.1	1.1729	2.9	0.1284	2.7	0.93	778.7	19.5	788.0	15.7	814.3	22.2	778.7	19.5
Gleason-354-U1453A-11F-1W-88-135-Spot 196	507	118816	1.1	15.0696	0.7	1.1952	2.3	0.1306	2.2	0.96	791.4	16.5	798.4	12.7	817.7	13.6	791.4	16.5
Gleason-354-U1453A-11F-1W-88-135-Spot 222	503	34089	2.1	14.0184	1.2	1.3273	2.6	0.1349	2.3	0.89	816.0	17.5	857.7	15.0	967.0	24.4	816.0	17.5
Gleason-354-U1453A-11F-1W-88-135-Spot 254	304	27905	1.6	14.9649	0.6	1.2479	1.9	0.1354	1.8	0.96	818.8	13.8	822.4	10.6	832.3	11.5	818.8	13.8
Gleason-354-U1453A-11F-1W-88-135-Spot 39	996	63567	16.6	14.3297	0.7	1.3525	2.4	0.1406	2.3	0.96	847.8	18.0	868.6	13.9	922.1	14.2	847.8	18.0
Gleason-354-U1453A-11F-1W-88-135-Spot 97	895	69942	1.4	14.5373	0.6	1.3420	2.3	0.1415	2.2	0.96	853.1	17.7	864.1	13.4	892.4	13.4	853.1	17.7
Gleason-354-U1453A-11F-1W-88-135-Spot 53	389	35818	5.9	14.4580	0.7	1.5041	2.2	0.1577	2.0	0.94	944.1	17.9	932.1	13.2	903.7	15.2	903.7	15.2
Gleason-354-U1453A-11F-1W-88-135-Spot 73	1265	236111	2.7	14.1607	0.5	1.4344	1.8	0.1473	1.7	0.95	885.9	13.8	903.4	10.5	946.4	11.2	946.4	11.2
Gleason-354-U1453A-11F-1W-88-135-Spot 4	328	108140	1.7	14.1494	0.7	1.3812	1.7	0.1417	1.5	0.91	854.5	12.3	880.9	9.9	948.0	14.0	948.0	14.0
Gleason-354-U1453A-11F-1W-88-135-Spot 168	814	228527	3.6	14.0805	0.7	1.5636	1.9	0.1597	1.8	0.92	955.0	15.8	955.9	11.9	958.0	15.0	958.0	15.0
Gleason-354-U1453A-11F-1W-88-135-Spot 224	221	57213	1.1	14.0415	0.7	1.5179	3.2	0.1546	3.1	0.97	926.6	26.8	937.6	19.6	963.7	15.3	963.7	15.3
Gleason-354-U1453A-11F-1W-88-135-Spot 307	110	38141	1.2	14.0204	1.1	1.4676	2.8	0.1492	2.6	0.92	896.6	21.6	917.1	16.9	966.7	22.6	966.7	22.6
Gleason-354-U1453A-11F-1W-88-135-Spot 276	727	53846	1.8	13.9626	0.7	1.5623	1.9	0.1582	1.8	0.94	946.8	16.0	955.4	12.0	975.2	13.6	975.2	13.6
Gleason-354-U1453A-11F-1W-88-135-Spot 278	144	104236	1.0	13.9301	0.8	1.5206	2.8	0.1536	2.7	0.96	921.3	22.9	938.7	17.1	979.9	16.3	979.9	16.3
Gleason-354-U1453A-11F-1W-88-135-Spot 48	190	540491	0.8	13.8657	0.8	1.6570	2.5	0.1666	2.3	0.94	993.5	21.6	992.2	15.8	989.3	17.1	989.3	17.1
Gleason-354-U1453A-11F-1W-88-135-Spot 68	456	550970	0.9	13.7824	0.6	1.6111	2.4	0.1610	2.3	0.97	962.6	20.6	974.5	14.9	1001.6	12.4	1001.6	12.4
Gleason-354-U1453A-11F-1W-88-135-Spot 128	577	205967	14.4	13.7363	0.7	1.6293	2.1	0.1623	2.0	0.94	969.7	17.8	981.6	13.2	1008.4	14.9	1008.4	14.9
Gleason-354-U1453A-11F-1W-88-135-Spot 16	269	367423	0.6	13.7285	0.7	1.6712	2.1	0.1664	2.0	0.94	992.2	18.3	997.6	13.4	1009.5	14.8	1009.5	14.8
Gleason-354-U1453A-11F-1W-88-135-Spot 169	761	75103	24.9	13.7237	0.6	1.5461	3.4	0.1539	3.4	0.98	922.7	28.8	948.9	21.0	1010.2	12.1	1010.2	12.1
Gleason-354-U1453A-11F-1W-88-135-Spot 296	203	91883	1.0	13.6502	0.7	1.6746	2.0	0.1658	1.9	0.95	988.9	17.5	998.9	12.8	1021.1	13.3	1021.1	13.3
Gleason-354-U1453A-11F-1W-88-135-Spot 143	162	384193	2.6	13.6421	0.8	1.6955	2.6	0.1678	2.5	0.96	999.7	23.2	1006.8	16.7	1022.3	15.2	1022.3	15.2
Gleason-354-U1453A-11F-1W-88-135-Spot 157	357	76185	1.6	13.6414	0.6	1.8075	2.5	0.1788	2.4	0.97	1060.6	23.5	1048.2	16.2	1022.4	11.8	1022.4	11.8

Gleason-354-U1453A-11F-1W-88-135-Spot 27	1473	703391	1.2	13.6371	0.7	1.6331	2.0	0.1615	1.9	0.93	965.2	16.6	983.0	12.6	1023.0	15.1	1023.0	15.1
Gleason-354-U1453A-11F-1W-88-135-Spot 106	863	70309	6.3	13.5668	0.5	1.5389	2.0	0.1514	1.9	0.96	908.9	16.0	946.1	12.1	1033.5	10.9	1033.5	10.9
Gleason-354-U1453A-11F-1W-88-135-Spot 265	438	370809	3.7	13.5307	0.7	1.6598	2.0	0.1629	1.8	0.93	972.8	16.6	993.3	12.5	1038.9	14.2	1038.9	14.2
Gleason-354-U1453A-11F-1W-88-135-Spot 252	2860	573540	6.7	13.3453	0.5	1.8727	1.8	0.1813	1.7	0.96	1073.8	17.1	1071.5	12.0	1066.7	10.3	1066.7	10.3
Gleason-354-U1453A-11F-1W-88-135-Spot 208	492	175274	5.0	13.3146	0.8	1.8929	2.5	0.1828	2.4	0.95	1082.2	24.0	1078.6	16.8	1071.4	15.2	1071.4	15.2
Gleason-354-U1453A-11F-1W-88-135-Spot 133	1117	131699	6.1	13.2905	0.6	1.8366	1.7	0.1770	1.6	0.93	1050.7	15.3	1058.6	11.2	1075.0	12.5	1075.0	12.5
Gleason-354-U1453A-11F-1W-88-135-Spot 152	134	27639	1.3	13.2785	0.5	1.8901	2.6	0.1820	2.5	0.98	1078.0	25.1	1077.6	17.2	1076.8	10.9	1076.8	10.9
Gleason-354-U1453A-11F-1W-88-135-Spot 32	423	352709	0.8	13.2073	0.7	1.8988	2.2	0.1819	2.1	0.95	1077.3	20.8	1080.7	14.6	1087.5	13.6	1087.5	13.6
Gleason-354-U1453A-11F-1W-88-135-Spot 140	775	539576	19.1	13.2005	0.8	1.8818	2.6	0.1802	2.5	0.96	1067.9	24.2	1074.7	17.0	1088.6	15.0	1088.6	15.0
Gleason-354-U1453A-11F-1W-88-135-Spot 23	129	29158	0.7	13.1730	0.8	1.9359	2.8	0.1850	2.7	0.96	1094.0	27.1	1093.6	18.9	1092.8	16.5	1092.8	16.5
Gleason-354-U1453A-11F-1W-88-135-Spot 232	991	299329	2.0	13.1667	0.6	1.5287	3.3	0.1460	3.2	0.98	878.4	26.3	942.0	20.0	1093.7	11.4	1093.7	11.4
Gleason-354-U1453A-11F-1W-88-135-Spot 259	1147	147717	0.9	13.1422	0.6	1.9207	1.6	0.1831	1.5	0.93	1083.7	14.5	1088.3	10.4	1097.5	11.4	1097.5	11.4
Gleason-354-U1453A-11F-1W-88-135-Spot 305	115	31730	2.0	13.1403	0.7	1.9611	3.0	0.1869	2.9	0.97	1104.6	29.9	1102.3	20.4	1097.8	13.8	1097.8	13.8
Gleason-354-U1453A-11F-1W-88-135-Spot 308	478	43062	1.4	13.1092	0.6	1.8416	2.3	0.1751	2.2	0.96	1040.1	20.9	1060.4	14.9	1102.5	12.1	1102.5	12.1
Gleason-354-U1453A-11F-1W-88-135-Spot 156	505	168958	1.0	13.1059	0.8	1.8267	1.8	0.1736	1.7	0.90	1032.1	15.8	1055.1	12.0	1103.0	15.7	1103.0	15.7
Gleason-354-U1453A-11F-1W-88-135-Spot 92	179	94211	1.6	13.0717	0.8	2.0169	2.7	0.1912	2.6	0.96	1127.9	27.3	1121.2	18.6	1108.2	15.6	1108.2	15.6
Gleason-354-U1453A-11F-1W-88-135-Spot 171	528	76247	1.4	12.9002	0.5	2.1021	2.0	0.1967	1.9	0.96	1157.4	20.4	1149.5	13.8	1134.5	10.8	1134.5	10.8
Gleason-354-U1453A-11F-1W-88-135-Spot 113	82	49400	0.4	12.7788	0.9	2.0752	2.6	0.1923	2.4	0.94	1134.0	25.2	1140.6	17.7	1153.3	18.0	1153.3	18.0
Gleason-354-U1453A-11F-1W-88-135-Spot 195	558	62117	1.8	12.7194	0.6	1.9200	2.3	0.1771	2.2	0.97	1051.2	21.4	1088.1	15.2	1162.6	11.3	1162.6	11.3
Gleason-354-U1453A-11F-1W-88-135-Spot 7	99	######	0.8	12.7037	0.9	1.9729	3.8	0.1818	3.7	0.97	1076.6	36.8	1106.3	25.8	1165.0	18.2	1165.0	18.2
Gleason-354-U1453A-11F-1W-88-135-Spot 139	923	131049	0.9	12.6068	0.7	1.9254	2.3	0.1760	2.2	0.95	1045.3	21.2	1089.9	15.4	1180.2	13.8	1180.2	13.8
Gleason-354-U1453A-11F-1W-88-135-Spot 198	120	99861	0.7	12.5799	0.7	2.1728	2.8	0.1982	2.7	0.97	1165.9	28.6	1172.4	19.2	1184.4	13.0	1184.4	13.0
Gleason-354-U1453A-11F-1W-88-135-Spot 264	478	170270	0.8	12.5763	0.7	2.1041	2.1	0.1919	2.0	0.95	1131.8	20.8	1150.2	14.5	1185.0	13.5	1185.0	13.5
Gleason-354-U1453A-11F-1W-88-135-Spot 286	567	######	2.0	12.5339	0.8	2.1088	2.6	0.1917	2.4	0.95	1130.6	25.1	1151.7	17.6	1191.6	16.3	1191.6	16.3
Gleason-354-U1453A-11F-1W-88-135-Spot 267	181	159903	0.7	12.4992	0.8	2.2502	2.4	0.2040	2.3	0.95	1196.7	24.6	1196.8	16.7	1197.1	15.0	1197.1	15.0
Gleason-354-U1453A-11F-1W-88-135-Spot 96	107	139244	1.0	12.4601	1.0	2.0909	2.8	0.1890	2.6	0.93	1115.7	26.4	1145.8	18.9	1203.3	19.2	1203.3	19.2
Gleason-354-U1453A-11F-1W-88-135-Spot 145	114	20092	1.0	12.3238	0.8	2.3771	2.8	0.2125	2.7	0.96	1242.0	30.3	1235.8	19.9	1224.9	14.9	1224.9	14.9
Gleason-354-U1453A-11F-1W-88-135-Spot 134	821	84330	1.9	12.2351	0.7	2.0658	2.2	0.1833	2.1	0.94	1085.1	21.1	1137.6	15.3	1239.1	14.6	1239.1	14.6
Gleason-354-U1453A-11F-1W-88-135-Spot 292	1400	93483	3.2	12.2066	0.6	2.1067	1.6	0.1865	1.5	0.92	1102.4	15.2	1151.0	11.3	1243.7	12.7	1243.7	12.7
Gleason-354-U1453A-11F-1W-88-135-Spot 311	220	50127	2.0	12.1282	0.8	2.4595	2.1	0.2163	2.0	0.92	1262.5	22.7	1260.2	15.5	1256.3	16.2	1256.3	16.2
Gleason-354-U1453A-11F-1W-88-135-Spot 82	255	36815	3.0	11.9946	0.8	2.5247	2.7	0.2196	2.6	0.96	1279.9	30.6	1279.2	19.9	1277.9	14.7	1277.9	14.7
Gleason-354-U1453A-11F-1W-88-135-Spot 158	326	######	1.9	11.8231	0.6	2.6790	2.2	0.2297	2.1	0.96	1333.1	25.7	1322.7	16.5	1305.9	12.3	1305.9	12.3
Gleason-354-U1453A-11F-1W-88-135-Spot 119	258	129365	2.0	11.7763	0.7	2.5924	2.5	0.2214	2.4	0.96	1289.4	27.5	1298.5	18.0	1313.6	13.8	1313.6	13.8
Gleason-354-U1453A-11F-1W-88-135-Spot 18	580	65935	1.1	11.6769	0.7	2.4722	2.2	0.2094	2.1	0.95	1225.5	23.3	1264.0	15.9	1330.0	13.7	1330.0	13.7
Gleason-354-U1453A-11F-1W-88-135-Spot 256	1056	211029	3.0	11.6641	0.6	2.7420	1.7	0.2320	1.6	0.94	1344.8	19.1	1339.9	12.4	1332.1	10.7	1332.1	10.7
Gleason-354-U1453A-11F-1W-88-135-Spot 69	381	######	2.1	11.5890	0.8	2.3667	2.2	0.1989	2.0	0.94	1169.5	21.7	1232.6	15.4	1344.6	14.7	1344.6	14.7
Gleason-354-U1453A-11F-1W-88-135-Spot 135	457	82681	1.0	11.2636	0.6	2.8127	2.1	0.2298	2.0	0.95	1333.3	23.9	1358.9	15.6	1399.4	12.2	1399.4	12.2
Gleason-354-U1453A-11F-1W-88-135-Spot 21	2398	789672	2.6	11.2010	0.6	2.5999	1.7	0.2112	1.6	0.93	1235.3	17.5	1300.6	12.2	1410.1	11.5	1410.1	11.5
Gleason-354-U1453A-11F-1W-88-135-Spot 315	230	206187	4.5	11.1494	0.6	3.1286	2.4	0.2530	2.4	0.97	1453.9	30.7	1439.8	18.8	1418.9	12.0	1418.9	12.0
Gleason-354-U1453A-11F-1W-88-135-Spot 44	123	111448	2.1	11.1389	0.7	2.9790	2.9	0.2407	2.8	0.97	1390.1	35.4	1402.3	22.1	1420.7	13.2	1420.7	13.2
Gleason-354-U1453A-11F-1W-88-135-Spot 148	351	49785	3.8	11.0423	0.7	3.1011	2.6	0.2484	2.5	0.96	1430.0	31.9	1433.0	19.9	1437.4	13.9	1437.4	13.9
Gleason-354-U1453A-11F-1W-88-135-Spot 176	454	202181	1.6	10.9179	0.6	2.8145	2.1	0.2229	2.0	0.95	1297.0	23.6	1359.4	15.8	1458.9	12.1	1458.9	12.1
Gleason-354-U1453A-11F-1W-88-135-Spot 275	253	357457	2.9	10.8907	1.2	2.9386	3.5	0.2321	3.3	0.94	1345.5	39.8	1391.9	26.4	1463.7	22.4	1463.7	22.4
Gleason-354-U1453A-11F-1W-88-135-Spot 303	353	213373	0.8	10.7744	0.8	3.3045	2.1	0.2582	1.9	0.93	1480.7	25.7	1482.1	16.2	1484.0	14.3	1484.0	14.3
Gleason-354-U1453A-11F-1W-88-135-Spot 214	8941	######	4.3	10.7144	0.6	3.0886	4.8	0.2400	4.7	0.99	1386.7	58.7	1429.9	36.5	1494.6	12.3	1494.6	12.3
Gleason-354-U1453A-11F-1W-88-135-Spot 302	355	306555	7.3	10.6744	0.7	2.8353	1.9	0.2195	1.8	0.93	1279.3	20.9	1364.9	14.5	1501.7	13.6	1501.7	13.6
Gleason-354-U1453A-11F-1W-88-135-Spot 236	215	42239	1.7	10.4986	0.7	3.5810	2.2	0.2727	2.1	0.95	1554.3	29.3	1545.3	17.7	1533.0	13.0	1533.0	13.0
Gleason-354-U1453A-11F-1W-88-135-Spot 177	4387	302722	1.2	10.4149	0.7	2.9007	4.6	0.2191	4.6	0.99	1277.2	53.2	1382.1	35.0	1548.1	12.6	1548.1	12.6
Gleason-354-U1453A-11F-1W-88-135-Spot 193	166	38477	1.2	10.4023	0.7	3.3729	2.8	0.2545	2.7	0.97	1461.5	35.2	1498.1	21.8	1550.3	12.8	1550.3	12.8
Gleason-354-U1453A-11F-1W-88-135-Spot 285	349	320111	0.9	10.3784	0.8	3.5176	1.9	0.2648	1.8	0.92	1514.2	23.9	1531.2	15.3	1554.7	14.4	1554.7	14.4
Gleason-354-U1453A-11F-1W-88-135-Spot 221	374	132154	2.4	10.3747	0.7	2.9825	2.7	0.2244	2.6	0.96	1305.2	31.1	1403.2	20.8	1555.3	13.8	1555.3	13.8
Gleason-354-U1453A-11F-1W-88-135-Spot 138	358	60883	1.0	10.3706	0.5	3.3496	2.3	0.2519	2.3	0.98	1448.5	29.5	1492.7	18.2	1556.1	9.4	1556.1	9.4
Gleason-354-U1453A-11F-1W-88-135-Spot 144	162	28815	1.0	10.3608	0.8	3.8102	2.3	0.2863	2.2	0.94	1623.1	31.1	1594.9	18.6	1557.9	15.1	1557.9	15.1
Gleason-354-U1453A-11F-1W-88-135-Spot 260	308	340083	2.1	10.3353	0.7	3.3757	3.2	0.2530	3.1	0.97	1454.1	40.1	1498.8	24.8	1562.5	13.6	1562.5	13.6
Gleason-354-U1453A-11F-1W-88-135-Spot 245	214	62424	1.0	10.2660	0.8	3.5732	2.3	0.2660	2.1	0.94	1520.7	29.1	1543.6	18.0	1575.1	14.1	1575.1	14.1
Gleason-354-U1453A-11F-1W-88-135-Spot 153	643	63141	2.6	10.2403	0.8	3.6185	1.9	0.2687	1.8	0.92	1534.4	24.2	1553.6	15.4	1579.8	14.3	1579.8	14.3
Gleason-354-U1453A-11F-1W-88-135-Spot 162	100	35621	1.7	10.1537	0.7	3.7916	2.3	0.2792	2.2	0.96	1587.5	30.4	1591.0	18.2	1595.6	12.5	1595.6	12.5
Gleason-354-U1453A-11F-1W-88-135-Spot 227	1002	94462	0.9	10.1414	0.6	3.6139	1.8	0.2658	1.7	0.94	1519.5	22.5	1552.6	14.1	1597.9	11.3	1597.9	11.3
Gleason-354-U1453A-11F-1W-88-135-Spot 71	198	181428	0.7	10.0486	0.8	3.6509	2.2	0.2661	2.1	0.94	1520.9	28.4	1560.7	17.8	1615.0	14.1	1615.0	14.1
Gleason-354-U1453A-11F-1W-88-135-Spot 212	260	104705	1.0	9.9975	0.7	3.9433	2.9	0.2859	2.8	0.97	1621.1	40.6	1622.6	23.7	1624.5	13.5	1624.5	13.5
Gleason-354-U1453A-11F-1W-88-135-Spot 242	252	137537	0.6	9.9846	0.5	4.0557	3.3	0.2937	3.3	0.99	1660.0	47.6	1645.4	26.9	1626.9	10.2	1626.9	10.2
Gleason-354-U1453A-11F-1W-88-135-Spot 50	109	43098	0.8	9.9712	0.9	4.0137	3.0	0.2903	2.8	0.95	1642.9	40.8	1637.0	24.0	1629.4	16.4	1629.4	16.4
Gleason-354-U1453A-11F-1W-88-135-Spot 20	155	40100	1.3	9.9669	0.8	3.9311	3.2	0.2842	3.1	0.97	1612.3	44.4	1620.1	25.9	1630.2	14.0	1630.2	14.0
Gleason-354-U1453A-11F-1W-88-135-Spot 249	194	47094	2.6	9.8829	0.7	4.0428	2.3	0.2898	2.2	0.96	1640.4	32.1	1642.9	18.8	1646.0	12.1	1646.0	12.1
Gleason-354-U1453A-11F-1W-88-135-Spot 89	766	157815	3.7	9.8827	0.7	3.6873	2.3	0.2643	2.2	0.95	1511.7	29.1	1568.6	18.1	1646.0	12.5	1646.0	12.5
Gleason-354-U1453A-11F-1W-88-135-Spot 280	404	110945	1.7	9.8032	0.5	3.9874	2.1	0.2835	2.1	0.97	1609.0	29.6	1631.6	17.4	1660.9	9.9	1660.9	9.9
Gleason-354-U1453A-11F-1W-88-135-Spot 85	105	37094	1.4	9.7807	0.9	3.7099	3.3	0.2632	3.1	0.96	1506.0	42.0	1573.5	26.0	1665.2	16.9	1665.2	16.9
Gleason-354-U1453A-11F-1W-88-135-Spot 312	215	58459	1.5	9.7420	0.6	3.9740	2.5	0.2808	2.4	0.97	1595.3	33.7	1628.9	19.9	1672.5	11.1	1672.5	11.1
Gleason-354-U1453A-11F-1W-88-135-Spot 30	182	32335	2.7	9.7175	0.7	3.9085	2.3	0.2755	2.2	0.95	1568.5	31.1	1615.4	19.0	1677.2	13.3	1677.2	13.3
Gleason-354-U1453A-11F-1W-88-135-Spot 194	742	56908	3.8	9.6681	0.7	3.5972	2.1	0.2522	2.0	0.95	1450.0	26.3	1548.9	17.0	1686.6	12.5	1686.6	12.5
Gleason-354-U1453A-11F-1W-88-135-Spot 14	221	275914	1.6	9.6034	0.8	3.4708	2.7	0.2417	2.5	0.95	1395.8	31.8	1520.6	20.9	1699.0	14.8	1699.0	14.8
Gleason-354-U1453A-11F-1W-88-135-Spot 165	154	526740	1.3	9.4640	0.6	4.4247	2.6	0.3037	2.5	0.97	1709.7	37.5	1717.0	21.3	1725.9	11.5	1725.9	11.5
Gleason-354-U1453A-11F-1W-88-135-Spot 225	273	420135	1.1	9.4275	0.7	4.3585	2.4	0.2980	2.3	0.95	1681.5	33.4	1704.5	19.5	1733.0	13.1	1733.0	13.1
Gleason-354-U1453A-11F-1W-88-135-Spot 298	329	104549	1.4	9.3748	0.5	4.4547	2.1	0.3029	2.1	0.97	1705.6	31.0	1722.6	17.7	1743.2	9.6	1743.2	9.6
Gleason-354-U1453A-11F-1W-88-135-Spot 287	494	89992	3.0	9.3541	0.6	4.6205	1.9	0.3135	1.8	0.95	1757.7	27.8	1753.0	16.0	1747.3	11.5	1747.3	11.5
Gleason-354-U1453A-11F-1W-88-135-Spot 204	161	31844	1.9	9.3191	0.5	4.6141	2.4	0.3119	2.4	0.98	1749.9	36.7	1751.8	20.4	1754.2	8.9	1754.2	8.9
Gleason-354-U1453A-11F-1W-88-135-Spot 262	669	######	0.9	9.3143	0.5	4.4339	2.1	0.2995	2.0	0.97	1689.0	30.0	1718.7	17.3	1755.1	9.9	1755.1	9.9
Gleason-354-U1453A-11F-1W-88-135-Spot 288	395	210335	7.3	9.2207	0.5	4.5404	2.1	0.3036	2.0	0.97	1709.3	30.7	1738.4	17.5	1773.6	8.7	1773.6	8.7
Gleason-354-U1453A-11F-1W-88-135-Spot 2	235	53862	2.0	9.2119	0.7	4.7756	2.5	0.3191	2.4	0.96	1785.1	37.4	1780.6	20.9	1775.3	12.3	1775.3	12.3
Gleason-354-U1453A-11F-1W-88-135-Spot 99	1349	945260	1.5	9.0251	0.6	4.8638	1.8	0.3184	1.7	0.94	1781.7	26.7	1796.0	15.4	1812.6	11.6	1812.6	11.6
Gleason-354-U1453A-11F-1W-88-135-Spot 104	514	######	5.0	8.8105	0.7	4.3851	2.0	0.2802	1.9	0.95	1592.4	26.9	1709.5	16.7	1856.2	11.8	1856.2	11.8
Gleason-354-U1453A-11F-1W-88-135-Spot 281	483	138024	2.6	8.7991	0.8	5.1332	2.8	0.3276	2.7	0.96	1826.7	42.3	1841.6	23.5	1858.5	13.9	1858.5	13.9
Gleason-354-U1453A-11F-1W-88-135-Spot 81	464	92089	2.2	8.7824	0.6	5.0691	1.9	0.3229	1.8	0.94	1803.8	27.7	1831.0	15.8	1862.0	11.4	1862.0	11.4
Gleason-354-U1453A-11F-1W-88-135-Spot 291	362	234766	1.8	8.7390	0.6	5.1262	2.2	0.3249	2.1	0.96	1813.6	33.7	1840.5	18.8	1870.9	10.9	1870.9	10.9
Gleason-354-U1453A-11F-1W-88-135-Spot 120	226	168828	2.2	8.5563	0.7	5.6352	2.3	0.3497	2.2	0.95	1933.2	36.7	1921.5	19.9	1908.9	12.6	1908.9	12.6
Gleason-354-U1453A-11F-1W-88-135-Spot 72	979	234278	40.3	8.4151	0.6	5.0516	1.9	0.3083	1.8	0.96	1732.4	28.0	1828.0	16.3	1938.8	10.0	1938.8	10.0

Gleason-354-U1453A-11F-1W-88-135-Spot 91	232	43796	4.8	8.3869	0.7	4.9170	2.4	0.2991	2.3	0.95	1686.8	34.1	1805.2	20.4	1944.8	13.1	1944.8	13.1
Gleason-354-U1453A-11F-1W-88-135-Spot 132	960	391553	3.2	8.3664	0.7	5.7806	2.4	0.3508	2.3	0.96	1938.2	38.1	1943.5	20.6	1949.1	12.3	1949.1	12.3
Gleason-354-U1453A-11F-1W-88-135-Spot 237	1405	101213	1.9	8.3398	0.6	5.0272	2.2	0.3041	2.1	0.97	1711.5	31.7	1823.9	18.5	1954.8	10.2	1954.8	10.2
Gleason-354-U1453A-11F-1W-88-135-Spot 192	590	114084	7.6	8.3158	0.5	5.9980	1.7	0.3617	1.6	0.95	1990.5	28.2	1975.5	15.1	1960.0	9.8	1960.0	9.8
Gleason-354-U1453A-11F-1W-88-135-Spot 75	796	281543	14.5	7.8566	0.7	6.0105	2.4	0.3425	2.3	0.96	1898.6	38.2	1977.4	21.2	2060.8	12.5	2060.8	12.5
Gleason-354-U1453A-11F-1W-88-135-Spot 118	158	299264	0.9	7.7051	0.5	6.9101	2.4	0.3862	2.4	0.98	2105.0	42.7	2100.0	21.6	2095.0	9.3	2095.0	9.3
Gleason-354-U1453A-11F-1W-88-135-Spot 41	517	260633	7.8	6.8181	0.5	8.7647	2.1	0.4334	2.1	0.97	2321.1	40.1	2313.8	19.3	2307.4	8.7	2307.4	8.7
Gleason-354-U1453A-11F-1W-88-135-Spot 164	1199	348971	7.9	6.6090	0.8	9.5757	2.3	0.4590	2.1	0.94	2435.1	43.4	2394.8	21.0	2360.8	13.3	2360.8	13.3
Gleason-354-U1453A-11F-1W-88-135-Spot 117	558	254327	0.5	6.2942	0.7	9.1595	2.2	0.4181	2.1	0.95	2252.0	40.3	2354.1	20.3	2443.7	11.4	2443.7	11.4
Gleason-354-U1453A-11F-1W-88-135-Spot 10	408	171278	1.9	6.2418	0.7	10.1557	3.9	0.4597	3.8	0.98	2438.4	77.4	2449.1	35.8	2457.9	11.4	2457.9	11.4
Gleason-354-U1453A-11F-1W-88-135-Spot 147	54	13240	1.4	6.1760	0.8	10.2257	3.6	0.4580	3.5	0.98	2430.9	70.8	2455.4	33.2	2475.8	13.3	2475.8	13.3
Gleason-354-U1453A-11F-1W-88-135-Spot 102	433	86594	3.5	5.9971	0.8	9.1162	9.5	0.3965	9.5	1.00	2152.9	173.7	2349.7	87.4	2525.2	14.1	2525.2	14.1
Gleason-354-U1453A-11F-1W-88-135-Spot 40	230	276178	1.3	5.9397	0.8	10.8384	2.8	0.4669	2.7	0.96	2470.0	54.5	2509.4	25.7	2541.4	13.2	2541.4	13.2
Gleason-354-U1453A-11F-1W-88-135-Spot 22	1223	######	5.9	5.7649	0.8	10.0094	2.4	0.4185	2.2	0.94	2253.7	42.4	2435.7	21.8	2591.3	13.2	2591.3	13.2
Gleason-354-U1453A-11F-1W-88-135-Spot 109	203	353736	1.0	5.7038	0.6	11.3663	2.1	0.4702	2.1	0.96	2484.4	42.4	2553.7	20.0	2609.1	9.9	2609.1	9.9
Gleason-354-U1453A-11F-1W-88-135-Spot 111	79	32620	1.6	4.9560	0.7	15.0027	3.2	0.5393	3.2	0.98	2780.4	71.3	2815.4	30.7	2840.6	10.8	2840.6	10.8
Gleason-354-U1453A-11F-1W-88-135-Spot 29	91	######	2.2	3.8027	0.5	23.3284	2.5	0.6434	2.4	0.98	3202.4	61.6	3240.7	24.3	3264.6	8.0	3264.6	8.0

Table ______. U-Pb geochronologic analyses.																		
#5 U1451A_13F_combined						Isotope ratios					Apparent ages (Ma)							
Mid Pleistocene 0.6 Ma																		
Analysis	U	206Pb	U/Th	206Pb*	±	207Pb*	±	206Pb*	±	error	206Pb*	±	207Pb*	±	206Pb*	±	Best age	±
	(ppm)	204Pb		207Pb*	(%)	235U*	(%)	238U	(%)	corr.	238U*	(Ma)	235U	(Ma)	207Pb*	(Ma)	(Ma)	(Ma)
Gleason-354-U1451A-13F-4W-39-89-Spot 293	259	271	0.6	82.6892	77.1	0.0019	77.1	0.0012	3.2	0.04	7.4	0.2	2.0	1.5	0.0	158.3	7.4	0.2
Gleason-354-U1451A-13F-4W-39-89-Spot 196	5655	7367	75.3	21.0812	1.3	0.0077	2.0	0.0012	1.5	0.75	7.6	0.1	7.8	0.2	71.2	31.3	7.6	0.1
Gleason-354-U1451A-13F-4W-39-89-Spot 270	254	412	0.6	11.5933	15.7	0.0191	16.1	0.0016	3.5	0.22	10.3	0.4	19.2	3.1	1343.9	304.7	10.3	0.4
Gleason-354-U1451A-13F-4W-39-89-Spot 177	228	3868	4.8	5.8685	2.0	0.0701	3.2	0.0030	2.4	0.76	19.2	0.5	68.8	2.1	2561.6	34.2	19.2	0.5
Gleason-354-U1451A-13F-4W-39-89-Spot 253	246	371	0.7	3.7214	3.0	0.1277	4.0	0.0034	2.7	0.66	22.2	0.6	122.1	4.6	3298.5	47.6	22.2	0.6
Gleason-354-U1451A-13F-4W-39-89-Spot 227	530	471	2.0	8.0791	1.3	0.0597	2.4	0.0035	2.1	0.85	22.5	0.5	58.9	1.4	2011.4	22.9	22.5	0.5
Gleason-354-U1451A-13F-4W-39-89-Spot 310	142	476	0.5	4.6719	2.7	0.1058	4.4	0.0036	3.4	0.79	23.1	0.8	102.1	4.2	2936.4	43.7	23.1	0.8
Gleason-354-U1451A-13F-4W-39-89-Spot 261	380	3798	5.6	22.2471	3.1	0.0236	3.7	0.0038	1.9	0.53	24.5	0.5	23.6	0.9	58.4	75.3	24.5	0.5
Gleason-354-U1451A-13F-4W-39-89-Spot 159	539	343	1.3	5.6302	1.4	0.0961	2.6	0.0039	2.2	0.84	25.3	0.5	93.2	2.3	2630.7	23.3	25.3	0.5
Gleason-354-U1451A-13F-4W-39-89-Spot 240	111	586	3.7	4.9405	5.4	0.1099	6.1	0.0039	2.9	0.47	25.3	0.7	105.9	6.2	2845.7	88.0	25.3	0.7
Gleason-354-U1451A-13F-4W-39-89-Spot 84	131	369	1.2	5.4617	8.0	0.0997	8.4	0.0039	2.6	0.31	25.4	0.7	96.5	7.7	2681.1	132.2	25.4	0.7
Gleason-354-U1451A-13F-4W-39-89-Spot 76	220	239	0.4	2.8390	0.0	0.1940	2.2	0.0040	2.2	1.00	25.7	0.6	180.1	3.7	3716.7	0.0	25.7	0.6
Gleason-354-U1451A-13F-4W-39-89-Spot 15	119	1638	0.4	7.6304	4.5	0.0740	5.2	0.0041	2.6	0.51	26.4	0.7	72.5	3.6	2112.1	78.5	26.4	0.7
Gleason-354-U1451A-13F-4W-39-89-Spot 41	667	7705	32.7	22.1005	2.6	0.0258	3.3	0.0041	2.1	0.63	26.6	0.6	25.9	0.8	42.3	62.3	26.6	0.6
Gleason-354-U1451A-13F-4W-39-89-Spot 200	110	606	0.7	2.2111	0.2	0.2653	3.5	0.0043	3.5	1.00	27.4	1.0	239.0	7.5	4092.7	2.3	27.4	1.0
Gleason-354-U1451A-13F-4W-39-89-Spot 308	128	384	0.2	4.6135	2.2	0.1576	3.2	0.0053	2.3	0.72	33.9	0.8	148.6	4.4	2956.7	35.2	33.9	0.8
Gleason-354-U1451A-13F-4W-39-89-Spot 88	103	207	3.5	2.4298	0.2	0.3557	3.0	0.0063	3.0	1.00	40.3	1.2	309.0	8.1	3951.8	2.4	40.3	1.2
Gleason-354-U1451A-13F-4W-39-89-Spot 64	66	454	8.2	3.2979	3.1	0.2750	8.2	0.0066	7.6	0.93	42.3	3.2	246.7	18.0	3486.8	47.5	42.3	3.2
Gleason-354-U1451A-13F-4W-39-89-Spot 172	204	1282	4.9	10.4674	6.0	0.0889	6.5	0.0068	2.6	0.40	43.4	1.1	86.5	5.4	1538.6	112.1	43.4	1.1
Gleason-354-U1451A-13F-4W-39-89-Spot 160	56	441	1.8	4.8745	2.6	0.1952	5.2	0.0069	4.4	0.86	44.3	2.0	181.0	8.6	2867.6	43.1	44.3	2.0
Gleason-354-U1451A-13F-4W-39-89-Spot 45	844	2283	0.6	23.1893	1.5	0.0414	2.3	0.0070	1.7	0.76	44.7	0.8	41.2	0.9	160.5	36.9	44.7	0.8
Gleason-354-U1451A-13F-4W-39-89-Spot 239	126	1590	0.9	24.0430	3.9	0.0417	4.7	0.0073	2.7	0.56	46.7	1.2	41.5	1.9	251.2	98.7	46.7	1.2
Gleason-354-U1451A-13F-4W-39-89-Spot 139	876	9655	7.0	21.4299	1.3	0.0519	2.1	0.0081	1.7	0.81	51.8	0.9	51.3	1.1	32.1	30.4	51.8	0.9
Gleason-354-U1451A-13F-4W-39-89-Spot 195	153	474	0.2	8.6829	1.6	0.1298	2.9	0.0082	2.4	0.83	52.5	1.3	123.9	3.4	1882.5	29.7	52.5	1.3
Gleason-354-U1451A-13F-4W-39-89-Spot 145	46	295	0.8	6.4282	1.7	0.1800	4.8	0.0084	4.5	0.94	53.9	2.4	168.1	7.4	2408.0	28.2	53.9	2.4
Gleason-354-U1451A-13F-4W-39-89-Spot 246	2123	9732	2.6	20.7908	1.0	0.0566	1.9	0.0085	1.7	0.85	54.8	0.9	55.9	1.1	104.1	24.0	54.8	0.9
Gleason-354-U1451A-13F-4W-39-89-Spot 260	262	1738	5.3	24.7338	3.1	0.0484	4.3	0.0087	2.9	0.68	55.7	1.6	48.0	2.0	323.4	80.3	55.7	1.6
Gleason-354-U1451A-13F-4W-39-89-Spot 229	72	3567	1.8	18.0792	4.9	0.0715	5.6	0.0094	2.7	0.49	60.1	1.6	70.1	3.8	424.8	109.2	60.1	1.6
Gleason-354-U1451A-13F-4W-39-89-Spot 163	32	561	1.8	25.9260	7.9	0.0499	9.5	0.0094	5.3	0.56	60.3	3.2	49.5	4.6	445.8	207.6	60.3	3.2
Gleason-354-U1451A-13F-4W-39-89-Spot 73	285	4406	1.5	20.3979	3.8	0.0654	4.5	0.0097	2.4	0.54	62.1	1.5	64.3	2.8	149.0	89.5	62.1	1.5
Gleason-354-U1451A-13F-4W-39-89-Spot 151	66	205	0.3	2.6176	0.0	0.5397	4.0	0.0102	4.0	1.00	65.7	2.6	438.2	14.3	3839.8	0.0	65.7	2.6
Gleason-354-U1451A-13F-4W-39-89-Spot 238	878	8570	0.9	21.4219	1.2	0.0676	2.1	0.0105	1.8	0.84	67.3	1.2	66.4	1.4	32.9	28.0	67.3	1.2
Gleason-354-U1451A-13F-4W-39-89-Spot 95	152	8117	0.9	20.6712	2.4	0.0703	3.5	0.0105	2.5	0.72	67.6	1.7	69.0	2.3	117.7	57.1	67.6	1.7
Gleason-354-U1451A-13F-4W-39-89-Spot 111	321	4960	1.0	18.7117	1.6	0.0799	2.8	0.0108	2.2	0.81	69.5	1.6	78.0	2.1	347.6	36.7	69.5	1.6
Gleason-354-U1451A-13F-4W-39-89-Spot 175	159	271	0.6	3.0867	0.2	0.5091	2.6	0.0114	2.6	1.00	73.0	1.9	417.8	9.0	3588.8	2.4	73.0	1.9
Gleason-354-U1451A-13F-4W-39-89-Spot 242	367	5190	1.5	21.7533	1.7	0.0724	2.4	0.0114	1.7	0.70	73.2	1.2	71.0	1.7	3.9	41.6	73.2	1.2
Gleason-354-U1451A-13F-4W-39-89-Spot 204	157	11943	1.2	20.6668	2.4	0.1099	3.5	0.0165	2.6	0.75	105.3	2.7	105.9	3.5	118.2	55.5	105.3	2.7
Gleason-354-U1451A-13F-4W-39-89-Spot 23	358	4440	1.3	21.7572	1.4	0.1118	2.4	0.0176	2.0	0.81	112.7	2.2	107.6	2.5	4.4	34.4	112.7	2.2
Gleason-354-U1451A-13F-4W-39-89-Spot 16	1216	24302	1.6	20.6016	1.0	0.1181	2.3	0.0177	2.0	0.90	112.8	2.3	113.4	2.4	125.6	23.3	112.8	2.3
Gleason-354-U1451A-13F-4W-39-89-Spot 166	1948	584720	0.9	20.6915	0.8	0.1206	2.1	0.0181	1.9	0.93	115.6	2.2	115.6	2.3	115.4	18.1	115.6	2.2
Gleason-354-U1451A-13F-4W-39-89-Spot 167	213	12050	1.2	18.6792	2.9	0.1350	3.9	0.0183	2.6	0.66	116.8	3.0	128.6	4.7	351.5	65.3	116.8	3.0
Gleason-354-U1451A-13F-4W-39-89-Spot 267	905	47252	1.7	20.2864	0.9	0.1354	1.9	0.0199	1.7	0.88	127.2	2.1	129.0	2.3	161.8	20.7	127.2	2.1
Gleason-354-U1451A-13F-4W-39-89-Spot 143	1689	60884	1.4	18.5328	1.4	0.1493	3.8	0.0201	3.5	0.93	128.1	4.5	141.3	5.0	369.3	31.3	128.1	4.5
Gleason-354-U1451A-13F-4W-39-89-Spot 300	1207	47823	2.3	20.5907	0.9	0.1383	2.0	0.0207	1.8	0.90	131.8	2.4	131.6	2.5	126.9	20.7	131.8	2.4
Gleason-354-U1451A-13F-4W-39-89-Spot 86	3176	80720	3.9	20.4887	0.8	0.1467	2.2	0.0218	2.0	0.93	139.0	2.8	139.0	2.8	138.6	17.9	139.0	2.8
Gleason-354-U1451A-13F-4W-39-89-Spot 176	849	85545	2.3	18.6920	1.8	0.2213	4.8	0.0300	4.5	0.93	190.5	8.5	203.0	8.9	350.0	40.0	190.5	8.5
Gleason-354-U1451A-13F-4W-39-89-Spot 107	67	3001	1.3	12.0672	3.5	0.3714	5.0	0.0325	3.5	0.71	206.2	7.2	320.7	13.7	1266.1	68.1	206.2	7.2
Gleason-354-U1451A-13F-4W-39-89-Spot 244	335	17073	2.6	19.8222	1.0	0.2278	2.5	0.0327	2.3	0.92	207.7	4.7	208.3	4.7	215.7	22.4	207.7	4.7
Gleason-354-U1451A-13F-4W-39-89-Spot 119	1857	595	0.5	6.1473	5.2	0.7484	6.6	0.0334	4.0	0.61	211.6	8.3	567.3	28.5	2483.6	87.9	211.6	8.3
Gleason-354-U1451A-13F-4W-39-89-Spot 133	37	639	0.9	7.1926	7.6	0.6434	8.4	0.0336	3.6	0.43	212.8	7.6	504.4	33.4	2215.2	131.6	212.8	7.6
Gleason-354-U1451A-13F-4W-39-89-Spot 48	2574	130811	0.0	19.6651	1.0	0.2460	3.0	0.0351	2.8	0.95	222.3	6.2	223.3	6.0	234.1	22.5	222.3	6.2
Gleason-354-U1451A-13F-4W-39-89-Spot 134	69	680	0.4	6.5853	2.2	0.7383	4.4	0.0353	3.9	0.87	223.4	8.5	561.4	19.1	2366.9	37.1	223.4	8.5
Gleason-354-U1451A-13F-4W-39-89-Spot 287	16	301	5.4	3.4386	2.1	1.4669	6.3	0.0366	6.0	0.95	231.6	13.6	916.9	38.1	3422.0	32.0	231.6	13.6
Gleason-354-U1451A-13F-4W-39-89-Spot 276	623	35874	3.0	17.5986	0.7	0.3268	3.3	0.0417	3.2	0.98	263.4	8.3	287.1	8.2	484.7	15.0	263.4	8.3
Gleason-354-U1451A-13F-4W-39-89-Spot 198	3794	16286	5.4	11.7808	0.5	0.4891	1.6	0.0418	1.5	0.94	263.9	3.9	404.3	5.4	1312.9	10.3	263.9	3.9
Gleason-354-U1451A-13F-4W-39-89-Spot 180	882	100679	9.2	7.6671	0.8	1.1085	3.8	0.0616	3.7	0.98	385.6	13.9	757.4	20.3	2103.7	14.5	385.6	13.9
Gleason-354-U1451A-13F-4W-39-89-Spot 257	296	66760	1.5	18.1290	0.8	0.5327	2.6	0.0700	2.5	0.95	436.4	10.6	433.6	9.3	418.7	18.2	436.4	10.6
Gleason-354-U1451A-13F-4W-39-89-Spot 31	601	39394	4.6	17.5881	0.8	0.5745	2.4	0.0733	2.3	0.94	455.9	9.9	460.9	8.9	486.0	18.6	455.9	9.9
Gleason-354-U1451A-13F-4W-39-89-Spot 89	526	106624	0.6	16.9661	0.5	0.5972	3.9	0.0735	3.9	0.99	457.1	17.3	475.5	15.0	564.9	11.7	457.1	17.3
Gleason-354-U1451A-13F-4W-39-89-Spot 154	206	278367	1.0	17.6293	0.7	0.5803	2.2	0.0742	2.1	0.96	461.4	9.6	464.7	8.4	480.8	14.6	461.4	9.6
Gleason-354-U1451A-13F-4W-39-89-Spot 182	533	139087	1.7	17.5250	0.7	0.5890	2.1	0.0749	1.9	0.94	465.4	8.7	470.2	7.8	493.9	15.6	465.4	8.7
Gleason-354-U1451A-13F-4W-39-89-Spot 306	318	27969	1.0	17.7437	0.8	0.5836	2.0	0.0751	1.9	0.92	466.8	8.5	466.8	7.6	466.5	17.1	466.8	8.5
Gleason-354-U1451A-13F-4W-39-89-Spot 256	259	27228	3.4	17.6991	0.9	0.5883	2.5	0.0755	2.3	0.93	469.3	10.4	469.8	9.3	472.1	19.9	469.3	10.4
Gleason-354-U1451A-13F-4W-39-89-Spot 83	666	######	3.6	17.1760	0.7	0.6169	2.2	0.0769	2.1	0.94	477.3	9.5	487.9	8.5	538.1	15.9	477.3	9.5
Gleason-354-U1451A-13F-4W-39-89-Spot 90	337	50224	1.0	17.3016	0.7	0.6268	2.2	0.0787	2.1	0.95	488.1	9.9	494.1	8.7	522.1	15.5	488.1	9.9
Gleason-354-U1451A-13F-4W-39-89-Spot 235	161	19830	1.0	17.2468	1.1	0.6294	2.7	0.0787	2.5	0.91	488.5	11.8	495.7	10.8	529.1	24.5	488.5	11.8
Gleason-354-U1451A-13F-4W-39-89-Spot 106	625	27347	6.3	17.6737	0.7	0.6242	2.0	0.0800	1.8	0.93	496.2	8.8	492.5	7.7	475.3	15.5	496.2	8.8
Gleason-354-U1451A-13F-4W-39-89-Spot 181	258	74056	1.9	17.3355	0.7	0.6397	2.0	0.0804	1.8	0.94	498.7	8.8	502.1	7.7	517.8	14.8	498.7	8.8
Gleason-354-U1451A-13F-4W-39-89-Spot 194	855	111048	2.1	17.3030	0.7	0.6504	2.0	0.0816	1.9	0.94	505.8	9.2	508.7	8.1	521.9	15.1	505.8	9.2
Gleason-354-U1451A-13F-4W-39-89-Spot 142	708	327869	3.4	17.4596	0.8	0.6456	1.9	0.0818	1.8	0.92	506.6	8.6	505.8	7.7	502.2	17.1	506.6	8.6
Gleason-354-U1451A-13F-4W-39-89-Spot 53	294	72438	0.4	16.9947	0.9	0.6667	2.1	0.0822	2.0	0.91	509.1	9.6	518.7	8.7	561.3	19.0	509.1	9.6
Gleason-354-U1451A-13F-4W-39-89-Spot 179	102	13335	1.1	17.3746	1.1	0.6606	2.9	0.0832	2.6	0.92	515.4	13.1	515.0	11.5	512.9	23.9	515.4	13.1
Gleason-354-U1451A-13F-4W-39-89-Spot 233	414	47039	1.0	17.4147	0.8	0.6611	2.3	0.0835	2.2	0.94	517.0	10.9	515.3	9.4	507.8	17.2	517.0	10.9
Gleason-354-U1451A-13F-4W-39-89-Spot 104	481	99509	2.3	17.0257	0.8	0.6771	1.9	0.0836	1.7	0.89	517.7	8.2	525.0	7.6	557.3	18.4	517.7	8.2
Gleason-354-U1451A-13F-4W-39-89-Spot 3	300	334243	2.1	17.2842	0.9	0.6730	2.4	0.0844	2.2	0.93	522.1	11.0	522.5	9.6	524.3	19.2	522.1	11.0
Gleason-354-U1451A-13F-4W-39-89-Spot 222	474	102085	2.6	17.2702	0.6	0.6743	1.7	0.0845	1.6	0.94	522.7	8.1	523.3	7.0	526.1	13.2	522.7	8.1
Gleason-354-U1451A-13F-4W-39-89-Spot 254	438	321461	12.6	16.4530	1.0	0.7096	2.5	0.0847	2.3	0.91	524.0	11.5	544.5	10.6	631.5	22.2	524.0	11.5
Gleason-354-U1451A-13F-4W-39-89-Spot 161	424	105175	4.3	17.0290	0.5	0.6857	2.0	0.0847	1.9	0.97	524.0	9.7	530.2	8.3	556.8	11.4	524.0	9.7
Gleason-354-U1451A-13F-4W-39-89-Spot 56	498	33013	2.7	17.2680	0.8	0.6814	1.8	0.0853	1.7	0.91	527.9	8.5	527.6	7.6	526.4	16.8	527.9	8.5

Gleason-354-U1451A-13F-4W-39-89-Spot 98	158	21633	0.4	16.9970	0.8	0.6945	2.8	0.0856	2.7	0.96	529.6	13.7	535.5	11.7	561.0	17.2	529.6	13.7
Gleason-354-U1451A-13F-4W-39-89-Spot 34	237	26613	0.9	17.2721	0.9	0.6963	2.1	0.0872	1.9	0.91	539.1	10.1	536.6	8.9	525.8	19.6	539.1	10.1
Gleason-354-U1451A-13F-4W-39-89-Spot 236	93	6370	1.5	17.0301	2.1	0.7566	3.6	0.0935	2.9	0.81	575.9	16.0	572.1	15.7	556.7	46.0	575.9	16.0
Gleason-354-U1451A-13F-4W-39-89-Spot 63	327	376512	1.6	16.5169	0.9	0.8000	2.1	0.0958	1.8	0.89	590.0	10.4	596.8	9.3	623.1	19.8	590.0	10.4
Gleason-354-U1451A-13F-4W-39-89-Spot 1	89	8657	1.1	16.7576	1.5	0.8115	2.7	0.0986	2.3	0.84	606.4	13.1	603.3	12.3	591.8	32.0	606.4	13.1
Gleason-354-U1451A-13F-4W-39-89-Spot 93	520	377977	8.4	15.2561	0.8	1.0457	2.1	0.1157	2.0	0.94	705.8	13.2	726.7	11.0	791.9	15.8	705.8	13.2
Gleason-354-U1451A-13F-4W-39-89-Spot 248	452	75953	2.8	15.4993	0.7	1.0439	2.4	0.1173	2.3	0.96	715.2	15.5	725.8	12.4	758.7	14.7	715.2	15.5
Gleason-354-U1451A-13F-4W-39-89-Spot 237	712	######	2.7	14.9604	0.6	1.1355	2.1	0.1232	2.1	0.96	749.0	14.6	770.4	11.6	832.9	11.7	749.0	14.6
Gleason-354-U1451A-13F-4W-39-89-Spot 103	295	37193	3.8	14.7661	0.7	1.1792	2.5	0.1263	2.4	0.96	766.6	17.3	790.9	13.8	860.1	15.4	766.6	17.3
Gleason-354-U1451A-13F-4W-39-89-Spot 147	409	50245	25.8	15.1986	0.7	1.1647	1.9	0.1284	1.8	0.93	778.6	13.2	784.2	10.6	799.9	15.0	778.6	13.2
Gleason-354-U1451A-13F-4W-39-89-Spot 149	611	81822	1.0	14.9890	0.7	1.1816	2.2	0.1284	2.0	0.94	779.0	15.0	792.0	11.9	828.9	15.0	779.0	15.0
Gleason-354-U1451A-13F-4W-39-89-Spot 230	518	152007	19.2	14.0395	1.8	1.2880	3.4	0.1311	2.9	0.85	794.4	21.3	840.4	19.3	964.0	36.6	794.4	21.3
Gleason-354-U1451A-13F-4W-39-89-Spot 174	775	189896	2.9	14.8446	0.7	1.2219	2.3	0.1316	2.2	0.95	796.7	16.6	810.6	13.0	849.0	15.1	796.7	16.6
Gleason-354-U1451A-13F-4W-39-89-Spot 302	276	55327	1.2	14.5487	1.2	1.2493	2.5	0.1318	2.2	0.88	798.3	16.5	823.1	14.1	890.8	24.5	798.3	16.5
Gleason-354-U1451A-13F-4W-39-89-Spot 61	478	44207	1.2	14.7880	0.8	1.2453	2.3	0.1336	2.2	0.94	808.1	16.3	821.3	12.9	857.0	16.6	808.1	16.3
Gleason-354-U1451A-13F-4W-39-89-Spot 78	90	18433	0.9	14.1326	0.7	1.3071	3.0	0.1340	3.0	0.97	810.5	22.5	848.8	17.5	950.4	14.1	810.5	22.5
Gleason-354-U1451A-13F-4W-39-89-Spot 202	1488	88813	9.3	14.7971	0.6	1.2521	1.4	0.1344	1.3	0.91	812.8	10.0	824.3	8.1	855.7	12.2	812.8	10.0
Gleason-354-U1451A-13F-4W-39-89-Spot 278	681	97794	1.2	14.0457	0.6	1.3363	3.2	0.1361	3.2	0.98	822.7	24.5	861.6	18.8	963.1	11.6	822.7	24.5
Gleason-354-U1451A-13F-4W-39-89-Spot 309	378	######	3.3	14.3851	0.7	1.3367	2.1	0.1395	2.0	0.94	841.6	15.6	861.8	12.3	914.1	15.3	841.6	15.6
Gleason-354-U1451A-13F-4W-39-89-Spot 185	79	209546	1.2	14.4177	0.9	1.3924	2.7	0.1456	2.6	0.95	876.2	21.2	885.7	16.2	909.4	18.5	876.2	21.2
Gleason-354-U1451A-13F-4W-39-89-Spot 313	370	50582	0.8	14.5730	0.8	1.3892	2.1	0.1468	1.9	0.92	883.2	15.8	884.4	12.3	887.4	16.7	883.2	15.8
Gleason-354-U1451A-13F-4W-39-89-Spot 288	281	47515	3.8	14.5988	0.6	1.4057	1.6	0.1488	1.5	0.93	894.4	12.4	891.4	9.5	883.7	12.0	894.4	12.4
Gleason-354-U1451A-13F-4W-39-89-Spot 178	486	52245	1.6	14.2745	0.6	1.4861	1.9	0.1538	1.8	0.95	922.5	15.2	924.7	11.3	930.0	11.6	930.0	11.6
Gleason-354-U1451A-13F-4W-39-89-Spot 127	375	135133	2.9	14.0955	0.7	1.5703	2.0	0.1605	1.9	0.95	959.7	17.3	958.5	12.7	955.8	13.4	955.8	13.4
Gleason-354-U1451A-13F-4W-39-89-Spot 223	2634	805272	25.8	14.0903	0.8	1.6334	2.1	0.1669	2.0	0.92	995.1	18.1	983.2	13.4	956.6	16.9	956.6	16.9
Gleason-354-U1451A-13F-4W-39-89-Spot 75	680	55370	1.1	13.8994	0.8	1.6063	2.0	0.1619	1.9	0.92	967.5	17.0	972.7	12.8	984.4	16.0	984.4	16.0
Gleason-354-U1451A-13F-4W-39-89-Spot 220	1412	284056	2.2	13.7105	0.7	1.6395	2.2	0.1630	2.1	0.94	973.6	18.7	985.5	13.8	1012.2	14.5	1012.2	14.5
Gleason-354-U1451A-13F-4W-39-89-Spot 123	154	111043	0.9	13.6961	0.7	1.6990	2.2	0.1688	2.1	0.95	1005.3	19.5	1008.1	14.0	1014.3	13.3	1014.3	13.3
Gleason-354-U1451A-13F-4W-39-89-Spot 37	312	38649	1.6	13.6938	0.9	1.6120	2.4	0.1601	2.2	0.93	957.3	20.0	974.9	15.1	1014.7	18.0	1014.7	18.0
Gleason-354-U1451A-13F-4W-39-89-Spot 193	900	185155	5.8	13.6109	1.2	1.6252	2.6	0.1604	2.3	0.88	959.2	20.4	980.0	16.3	1026.9	24.9	1026.9	24.9
Gleason-354-U1451A-13F-4W-39-89-Spot 209	231	37368	1.4	13.5601	0.8	1.7653	2.5	0.1736	2.3	0.94	1032.0	22.1	1032.8	16.0	1034.5	17.0	1034.5	17.0
Gleason-354-U1451A-13F-4W-39-89-Spot 35	260	65609	1.4	13.5573	0.7	1.7185	2.1	0.1690	2.0	0.94	1006.5	18.7	1015.5	13.7	1034.9	14.7	1034.9	14.7
Gleason-354-U1451A-13F-4W-39-89-Spot 264	666	45286	1.9	13.5500	0.7	1.5329	2.4	0.1506	2.3	0.96	904.6	19.7	943.7	14.9	1036.0	13.6	1036.0	13.6
Gleason-354-U1451A-13F-4W-39-89-Spot 213	266	371316	2.0	13.2242	0.7	1.8421	2.3	0.1767	2.2	0.96	1048.8	20.8	1060.6	14.8	1085.0	13.3	1085.0	13.3
Gleason-354-U1451A-13F-4W-39-89-Spot 6	305	207305	0.9	13.1801	0.6	1.9808	2.4	0.1893	2.3	0.96	1117.8	23.5	1109.0	16.0	1091.7	12.9	1091.7	12.9
Gleason-354-U1451A-13F-4W-39-89-Spot 138	167	57938	0.7	13.1585	0.8	1.9154	2.9	0.1828	2.8	0.96	1082.2	27.5	1086.5	19.2	1095.0	15.7	1095.0	15.7
Gleason-354-U1451A-13F-4W-39-89-Spot 311	431	54515	1.4	13.1115	0.7	1.7122	2.2	0.1628	2.1	0.95	972.4	19.2	1013.1	14.4	1102.2	14.7	1102.2	14.7
Gleason-354-U1451A-13F-4W-39-89-Spot 85	294	598427	0.4	13.0931	0.8	1.8929	2.4	0.1798	2.3	0.95	1065.6	22.7	1078.6	16.1	1104.9	15.2	1104.9	15.2
Gleason-354-U1451A-13F-4W-39-89-Spot 279	68	18345	0.6	12.9775	0.8	2.0293	2.7	0.1910	2.6	0.95	1126.8	26.6	1125.4	18.4	1122.6	16.7	1122.6	16.7
Gleason-354-U1451A-13F-4W-39-89-Spot 187	259	50995	6.4	12.8547	0.7	1.9709	2.3	0.1837	2.2	0.96	1087.4	22.4	1105.6	15.8	1141.6	13.8	1141.6	13.8
Gleason-354-U1451A-13F-4W-39-89-Spot 307	40	43775	0.9	12.8397	1.0	2.0031	4.1	0.1865	3.9	0.97	1102.6	39.7	1116.6	27.5	1143.9	20.6	1143.9	20.6
Gleason-354-U1451A-13F-4W-39-89-Spot 170	1781	686515	41.8	12.6656	0.7	2.1751	2.3	0.1998	2.2	0.96	1174.3	23.9	1173.1	16.1	1171.0	13.1	1171.0	13.1
Gleason-354-U1451A-13F-4W-39-89-Spot 165	107	48394	1.3	12.5335	1.0	2.2466	2.9	0.2042	2.7	0.94	1197.9	29.6	1195.7	20.2	1191.7	18.8	1191.7	18.8
Gleason-354-U1451A-13F-4W-39-89-Spot 144	566	59606	4.1	12.5134	0.7	2.1031	2.0	0.1909	1.9	0.93	1126.1	19.3	1149.8	13.8	1194.9	14.4	1194.9	14.4
Gleason-354-U1451A-13F-4W-39-89-Spot 212	737	157863	4.2	12.2078	1.0	2.2091	2.3	0.1956	2.1	0.90	1151.6	22.3	1183.9	16.4	1243.5	20.0	1243.5	20.0
Gleason-354-U1451A-13F-4W-39-89-Spot 140	1559	336613	0.7	12.0326	0.7	2.4658	2.3	0.2152	2.2	0.95	1256.4	25.3	1262.1	16.9	1271.8	14.3	1271.8	14.3
Gleason-354-U1451A-13F-4W-39-89-Spot 113	156	######	1.3	11.9571	0.6	2.4995	1.8	0.2168	1.7	0.94	1264.7	20.0	1271.9	13.4	1284.0	12.0	1284.0	12.0
Gleason-354-U1451A-13F-4W-39-89-Spot 9	847	126033	3.9	11.9559	1.4	2.2130	2.6	0.1919	2.2	0.84	1131.6	22.6	1185.2	18.1	1284.2	27.1	1284.2	27.1
Gleason-354-U1451A-13F-4W-39-89-Spot 295	81	8551	0.5	11.9052	2.1	2.2953	4.3	0.1982	3.8	0.87	1165.6	40.1	1210.8	30.4	1292.5	40.7	1292.5	40.7
Gleason-354-U1451A-13F-4W-39-89-Spot 275	131	108974	2.1	11.8077	0.7	2.5937	2.4	0.2221	2.3	0.96	1293.0	26.7	1298.9	17.4	1308.5	13.0	1308.5	13.0
Gleason-354-U1451A-13F-4W-39-89-Spot 49	765	21233	10.0	11.7868	1.2	2.1470	3.4	0.1835	3.2	0.94	1086.3	31.8	1164.1	23.6	1311.9	23.3	1311.9	23.3
Gleason-354-U1451A-13F-4W-39-89-Spot 221	53	12739	1.0	11.6782	0.9	2.6283	3.2	0.2226	3.1	0.96	1295.7	36.7	1308.6	23.8	1329.8	16.6	1329.8	16.6
Gleason-354-U1451A-13F-4W-39-89-Spot 28	1239	669673	10.4	11.3594	1.0	2.5228	2.6	0.2078	2.4	0.92	1217.3	26.1	1278.6	18.6	1383.2	19.1	1383.2	19.1
Gleason-354-U1451A-13F-4W-39-89-Spot 57	1179	394667	4.9	11.1264	0.8	2.9752	2.8	0.2401	2.7	0.96	1387.2	33.3	1401.3	21.2	1422.9	15.7	1422.9	15.7
Gleason-354-U1451A-13F-4W-39-89-Spot 105	345	532088	2.4	10.8584	0.6	3.2258	1.7	0.2540	1.6	0.95	1459.3	21.5	1463.4	13.5	1469.3	10.7	1469.3	10.7
Gleason-354-U1451A-13F-4W-39-89-Spot 241	236	383942	2.6	10.1708	0.6	3.7947	1.7	0.2799	1.6	0.94	1591.0	23.1	1591.6	13.9	1592.5	10.7	1592.5	10.7
Gleason-354-U1451A-13F-4W-39-89-Spot 255	220	290713	1.2	9.9110	0.7	4.2083	2.6	0.3025	2.5	0.97	1703.7	37.7	1675.6	21.4	1640.7	12.5	1640.7	12.5
Gleason-354-U1451A-13F-4W-39-89-Spot 284	1699	######	7.8	9.9016	0.8	3.1513	1.8	0.2263	1.7	0.90	1315.1	19.8	1445.3	14.2	1642.4	14.8	1642.4	14.8
Gleason-354-U1451A-13F-4W-39-89-Spot 286	323	182180	0.7	9.5191	0.7	3.9768	2.2	0.2746	2.1	0.95	1563.9	28.9	1629.5	17.7	1715.2	12.2	1715.2	12.2
Gleason-354-U1451A-13F-4W-39-89-Spot 24	313	294010	2.0	9.5110	0.7	4.4405	2.1	0.3063	2.0	0.95	1722.5	30.5	1719.9	17.7	1716.8	12.8	1716.8	12.8
Gleason-354-U1451A-13F-4W-39-89-Spot 122	608	121748	2.0	9.4566	0.5	4.3478	1.5	0.2982	1.5	0.95	1682.4	21.8	1702.5	12.8	1727.3	8.6	1727.3	8.6
Gleason-354-U1451A-13F-4W-39-89-Spot 168	2049	987288	28.0	9.3880	0.6	4.7523	2.5	0.3236	2.4	0.97	1807.2	37.6	1776.5	20.7	1740.7	11.7	1740.7	11.7
Gleason-354-U1451A-13F-4W-39-89-Spot 303	231	140561	1.5	9.3710	0.6	4.1382	2.1	0.2813	2.0	0.96	1597.7	28.3	1661.9	17.0	1744.0	10.6	1744.0	10.6
Gleason-354-U1451A-13F-4W-39-89-Spot 100	186	153612	1.4	9.3219	0.6	4.7092	2.0	0.3184	1.9	0.95	1781.8	30.2	1768.9	17.1	1753.6	11.7	1753.6	11.7
Gleason-354-U1451A-13F-4W-39-89-Spot 97	248	87386	6.0	9.2840	0.7	4.6816	1.9	0.3152	1.8	0.94	1766.4	28.0	1764.0	16.1	1761.0	11.9	1761.0	11.9
Gleason-354-U1451A-13F-4W-39-89-Spot 208	361	######	4.4	9.1935	0.8	4.6479	2.6	0.3099	2.5	0.96	1740.3	38.4	1757.9	22.0	1778.9	14.0	1778.9	14.0
Gleason-354-U1451A-13F-4W-39-89-Spot 102	444	606956	6.3	9.1275	0.6	4.7486	2.2	0.3144	2.1	0.96	1762.1	32.2	1775.9	18.3	1792.1	11.7	1792.1	11.7
Gleason-354-U1451A-13F-4W-39-89-Spot 81	1602	######	10.8	9.0629	0.6	4.1460	2.3	0.2725	2.2	0.97	1553.6	31.0	1663.4	19.0	1805.0	10.9	1805.0	10.9
Gleason-354-U1451A-13F-4W-39-89-Spot 135	355	63461	0.8	9.0214	1.0	4.0092	2.5	0.2623	2.3	0.92	1501.7	30.4	1636.1	20.0	1813.3	17.6	1813.3	17.6
Gleason-354-U1451A-13F-4W-39-89-Spot 121	651	420676	2.4	8.9046	0.8	4.6035	2.7	0.2973	2.5	0.96	1677.9	37.6	1749.9	22.2	1837.0	14.2	1837.0	14.2
Gleason-354-U1451A-13F-4W-39-89-Spot 280	767	825777	12.3	8.8096	0.6	4.7699	2.3	0.3048	2.2	0.96	1714.9	33.5	1779.6	19.4	1856.4	11.6	1856.4	11.6
Gleason-354-U1451A-13F-4W-39-89-Spot 59	1003	254042	2.2	8.7750	0.6	5.0838	1.5	0.3235	1.4	0.91	1807.0	21.8	1833.4	12.9	1863.5	11.5	1863.5	11.5
Gleason-354-U1451A-13F-4W-39-89-Spot 299	440	163157	2.3	8.7721	0.6	5.0888	2.1	0.3238	2.0	0.95	1808.0	31.8	1834.2	17.9	1864.1	11.3	1864.1	11.3
Gleason-354-U1451A-13F-4W-39-89-Spot 141	405	######	1.7	8.7715	0.6	5.1981	2.3	0.3307	2.2	0.96	1841.7	36.0	1852.3	19.9	1864.2	11.7	1864.2	11.7
Gleason-354-U1451A-13F-4W-39-89-Spot 225	508	199401	3.1	8.7424	0.6	5.2190	2.1	0.3309	2.0	0.96	1842.8	32.7	1855.7	18.1	1870.2	10.2	1870.2	10.2
Gleason-354-U1451A-13F-4W-39-89-Spot 5	391	288247	3.2	8.4393	0.6	5.7039	1.8	0.3491	1.7	0.94	1930.4	27.7	1932.0	15.2	1933.6	10.6	1933.6	10.6
Gleason-354-U1451A-13F-4W-39-89-Spot 110	1138	785722	12.7	8.3189	2.3	5.6899	3.6	0.3433	2.8	0.77	1902.5	45.9	1929.8	31.2	1959.3	41.0	1959.3	41.0
Gleason-354-U1451A-13F-4W-39-89-Spot 153	38	10973	1.9	8.2429	0.9	5.3187	4.0	0.3180	3.9	0.97	1779.8	60.7	1871.9	34.3	1975.7	16.2	1975.7	16.2
Gleason-354-U1451A-13F-4W-39-89-Spot 245	291	70897	1.2	8.0420	0.7	5.8180	2.5	0.3393	2.4	0.96	1883.5	39.4	1949.1	21.7	2019.5	11.8	2019.5	11.8
Gleason-354-U1451A-13F-4W-39-89-Spot 294	936	181624	2.5	7.9693	0.6	6.1467	2.2	0.3553	2.1	0.96	1959.7	35.5	1996.9	19.2	2035.6	11.4	2035.6	11.4
Gleason-354-U1451A-13F-4W-39-89-Spot 158	27	9043	1.8	7.0778	0.8	8.0257	4.3	0.4120	4.2	0.98	2224.0	78.8	2233.9	38.5	2243.0	13.4	2243.0	13.4
Gleason-354-U1451A-13F-4W-39-89-Spot 17	529	176926	0.6	6.7478	0.6	8.7677	2.3	0.4291	2.2	0.96	2301.6	43.0	2314.1	21.0	2325.2	10.8	2325.2	10.8
Gleason-354-U1451A-13F-4W-39-89-Spot 217	461	132758	3.4	6.4213	0.7	8.1983	2.0	0.3818	1.9	0.95	2084.7	34.5	2253.2	18.5	2409.8	11.1	2409.8	11.1
Gleason-354-U1451A-13F-4W-39-89-Spot 247	239	231337	1.7	6.3862	0.6	9.7601	1.8	0.4521	1.7	0.94	2404.4	33.1	2412.4	16.1	2419.1	9.9	2419.1	9.9
Gleason-354-U1451A-13F-4W-39-89-Spot 301	1417	######	34.9	6.3214	0.5	10.2843	1.7	0.4715	1.6	0.95	2490.2	32.8	2460.7	15.5	2436.4	8.8	2436.4	8.8
Gleason-354-U1451A-13F-4W-39-89-Spot 226	376	195611	1.2	6.2029	0.7	8.5830	3.2	0.3861	3.1	0.98	2104.9	55.9	2294.8	29.0	2468.4	11.7	2468.4	11.7

Gleason-354-U1451A-13F-4W-39-89-Spot 263	30	9493	0.8	6.1186	0.7	10.4459	5.6	0.4636	5.6	0.99	2455.2	114.2	2475.1	52.3	2491.5	12.2	2491.5	12.2
Gleason-354-U1451A-13F-4W-39-89-Spot 99	160	105458	1.4	5.6559	0.6	10.5820	2.5	0.4341	2.4	0.97	2324.1	47.0	2487.1	23.0	2623.1	9.8	2623.1	9.8
Gleason-354-U1451A-13F-4W-39-89-Spot 201	98	32612	2.1	5.4899	0.8	13.0093	3.2	0.5180	3.1	0.97	2690.6	68.8	2680.3	30.6	2672.6	14.0	2672.6	14.0
Gleason-354-U1451A-13F-4W-39-89-Spot 296	336	588466	1.5	5.3404	0.6	13.2889	2.1	0.5147	2.0	0.95	2676.7	44.3	2700.4	20.1	2718.2	10.6	2718.2	10.6
Gleason-354-U1451A-13F-4W-39-89-Spot 12	84	136035	1.4	5.0123	0.6	15.0915	2.7	0.5486	2.6	0.97	2819.4	60.4	2821.0	25.8	2822.2	10.1	2822.2	10.1
Gleason-354-U1451A-13F-4W-39-89-Spot 162	217	999141	1.3	4.5219	0.5	17.2735	2.2	0.5665	2.2	0.97	2893.4	50.8	2950.1	21.6	2989.1	8.8	2989.1	8.8
-Spot 4	568	2451	196.7	17.9471	5.2	0.0245	5.7	0.0032	2.3	0.40	20.5	0.5	24.6	1.4	441.2	115.2	20.5	0.5
-Spot 98	260	17992	4.9	21.5256	2.5	0.0454	3.3	0.0071	2.2	0.65	45.6	1.0	45.1	1.5	21.4	61.0	45.6	1.0
-Spot 31	868	4750	3.7	21.7263	1.8	0.0524	2.7	0.0083	2.0	0.75	53.0	1.0	51.9	1.3	0.9	42.6	53.0	1.0
-Spot 97	1468	11275	36.2	21.5255	1.0	0.0720	1.7	0.0112	1.4	0.81	72.0	1.0	70.6	1.1	21.4	23.5	72.0	1.0
-Spot 19	186	1614	1.3	24.9093	11.8	0.0736	12.0	0.0133	2.3	0.20	85.1	2.0	72.1	8.3	341.6	303.8	85.1	2.0
-Spot 100	2760	116976	4.1	20.5374	0.8	0.1136	2.0	0.0169	1.8	0.92	108.2	1.9	109.3	2.0	133.0	17.7	108.2	1.9
-Spot 12	806	32272	0.9	20.8464	1.2	0.1125	2.1	0.0170	1.7	0.82	108.8	1.8	108.3	2.1	97.8	28.3	108.8	1.8
-Spot 47	1093	38619	37.1	14.7300	1.6	0.1958	3.7	0.0209	3.3	0.90	133.4	4.4	181.5	6.1	865.2	32.6	133.4	4.4
-Spot 2	155	3253	2.1	22.1597	1.5	0.1772	3.2	0.0285	2.8	0.88	181.0	5.0	165.6	4.9	48.8	36.2	181.0	5.0
-Spot 1	4731	47993	0.2	15.4042	0.6	0.2964	4.1	0.0331	4.1	0.99	210.0	8.4	263.6	9.6	771.6	13.1	210.0	8.4
-Spot 72	608	111476	1.7	19.5927	0.9	0.2362	1.9	0.0336	1.7	0.89	212.8	3.6	215.3	3.8	242.6	20.0	212.8	3.6
-Spot 108	1693	530988	9.6	14.2152	2.1	0.3886	9.6	0.0401	9.4	0.98	253.2	23.4	333.3	27.4	938.5	42.4	253.2	23.4
-Spot 41	2015	173163	33.6	8.0469	1.8	0.8064	9.8	0.0471	9.6	0.98	296.5	27.9	600.5	44.4	2018.4	31.8	296.5	27.9
-Spot 65	80	5245	2.2	16.0735	1.4	0.5360	9.7	0.0625	9.6	0.99	390.7	36.2	435.8	34.3	681.5	30.8	390.7	36.2
-Spot 20	641	42708	1.7	17.7395	0.9	0.4887	3.6	0.0629	3.5	0.97	393.1	13.3	404.0	12.0	467.0	19.5	393.1	13.3
-Spot 5	218	178496	1.6	17.3763	1.0	0.5611	3.0	0.0707	2.8	0.95	440.4	12.1	452.2	10.9	512.6	21.1	440.4	12.1
-Spot 76	549	######	3.3	17.7290	0.8	0.5741	1.8	0.0738	1.7	0.91	459.1	7.3	460.6	6.7	468.3	16.6	459.1	7.3
-Spot 94	635	140707	17.0	17.5968	0.6	0.5969	2.0	0.0762	1.9	0.96	473.3	8.6	475.2	7.5	484.9	12.4	473.3	8.6
-Spot 79	622	124141	1.7	17.2818	0.7	0.6097	1.9	0.0764	1.8	0.93	474.7	8.3	483.4	7.5	524.6	15.9	474.7	8.3
-Spot 52	414	35235	9.5	17.6103	0.8	0.6268	2.6	0.0801	2.4	0.95	496.5	11.7	494.1	10.1	483.2	18.2	496.5	11.7
-Spot 75	186	10245	0.5	17.4184	1.2	0.6464	2.7	0.0817	2.5	0.90	506.0	12.0	506.2	10.9	507.3	25.7	506.0	12.0
-Spot 21	1011	168342	1.8	17.4280	0.8	0.6477	2.5	0.0819	2.4	0.95	507.2	11.8	507.0	10.1	506.1	17.4	507.2	11.8
-Spot 103	249	29878	0.9	17.2504	0.9	0.6709	2.6	0.0839	2.5	0.93	519.5	12.3	521.2	10.8	528.6	20.7	519.5	12.3
-Spot 26	121	25526	1.9	16.6872	1.4	0.7832	2.6	0.0948	2.2	0.85	583.8	12.4	587.3	11.7	600.9	30.2	583.8	12.4
-Spot 81	1425	375149	29.4	16.3403	0.8	0.8074	3.0	0.0957	2.9	0.97	589.1	16.4	601.0	13.7	646.2	16.2	589.1	16.4
-Spot 23	612	62582	5.6	16.2801	0.8	0.8303	2.1	0.0980	1.9	0.92	602.9	11.0	613.8	9.6	654.1	17.6	602.9	11.0
-Spot 37	327	22987	3.5	16.3299	0.7	0.8361	2.3	0.0990	2.2	0.95	608.7	13.0	617.0	10.8	647.6	15.4	608.7	13.0
-Spot 10	530	67044	1.9	15.5030	0.7	0.8877	2.1	0.0998	1.9	0.95	613.3	11.4	645.1	9.8	758.2	14.0	613.3	11.4
-Spot 39	117	6071	6.8	15.5211	0.9	0.9454	2.4	0.1064	2.2	0.93	651.9	13.8	675.7	11.8	755.7	18.3	651.9	13.8
-Spot 49	2298	178641	4.5	15.3960	0.8	0.9584	3.0	0.1070	2.9	0.96	655.4	18.3	682.5	15.1	772.8	17.1	655.4	18.3
-Spot 71	313	34538	0.9	15.5348	0.7	1.0132	2.0	0.1142	1.9	0.93	696.9	12.6	710.5	10.4	753.8	15.5	696.9	12.6
-Spot 80	653	72867	4.5	15.1351	0.6	1.0790	1.7	0.1184	1.6	0.93	721.6	11.1	743.1	9.2	808.6	13.3	721.6	11.1
-Spot 85	970	66017	23.2	15.2959	0.6	1.0888	1.6	0.1208	1.5	0.92	735.1	10.2	747.9	8.4	786.5	13.3	735.1	10.2
-Spot 101	760	######	1.4	15.0119	0.7	1.1867	1.8	0.1292	1.6	0.92	783.3	11.9	794.4	9.7	825.7	14.2	783.3	11.9
-Spot 11	899	154824	4.4	14.5835	0.6	1.2258	2.0	0.1296	1.9	0.96	785.9	14.2	812.4	11.3	885.8	12.0	785.9	14.2
-Spot 64	2078	24351	4.7	13.9004	1.4	1.3264	4.0	0.1337	3.8	0.94	809.1	28.9	857.3	23.4	984.2	28.0	809.1	28.9
-Spot 58	702	22270	1.5	14.5439	0.7	1.2871	1.7	0.1358	1.5	0.90	820.7	11.5	840.0	9.5	891.5	15.1	820.7	11.5
-Spot 107	3518	238964	0.8	15.1003	0.5	1.2404	1.5	0.1358	1.4	0.94	821.1	10.7	819.1	8.3	813.5	10.6	821.1	10.7
-Spot 48	1272	307021	74.7	14.7607	0.6	1.2926	1.6	0.1384	1.5	0.93	835.5	11.7	842.5	9.2	860.8	12.1	835.5	11.7
-Spot 87	189	98208	2.5	14.5476	0.7	1.3627	2.5	0.1438	2.3	0.95	866.0	19.0	873.0	14.4	891.0	15.4	866.0	19.0
-Spot 53	151	9898	2.3	14.4836	1.7	1.4249	3.4	0.1497	2.9	0.86	899.2	24.2	899.4	20.0	900.0	35.7	899.2	24.2
-Spot 7	340	108525	10.1	14.1388	0.8	1.5056	2.6	0.1544	2.5	0.95	925.5	21.6	932.7	16.1	949.5	16.8	949.5	16.8
-Spot 14	567	43984	6.5	14.0934	0.8	1.5019	2.2	0.1535	2.0	0.92	920.7	17.1	931.2	13.2	956.1	17.3	956.1	17.3
-Spot 89	495	200947	0.8	13.9260	0.5	1.5215	1.7	0.1537	1.6	0.96	921.5	13.9	939.1	10.4	980.5	10.2	980.5	10.2
-Spot 6	201	14187	1.6	13.8718	0.7	1.5655	2.1	0.1575	2.0	0.94	942.9	17.3	956.6	13.1	988.4	14.9	988.4	14.9
-Spot 33	76	44075	1.2	13.8629	1.2	1.3532	2.8	0.1361	2.5	0.90	822.3	19.7	869.0	16.5	989.7	24.6	989.7	24.6
-Spot 73	196	107648	1.8	13.8545	0.6	1.6108	2.1	0.1619	2.0	0.96	967.1	17.9	974.4	13.0	991.0	11.7	991.0	11.7
-Spot 92	217	58464	1.1	13.7654	0.7	1.6635	2.3	0.1661	2.2	0.96	990.5	20.1	994.7	14.5	1004.1	13.2	1004.1	13.2
-Spot 22	644	870300	10.0	13.5939	0.8	1.6553	3.1	0.1632	3.0	0.97	974.5	26.9	991.6	19.4	1029.5	15.9	1029.5	15.9
-Spot 27	869	60555	19.2	13.1965	0.6	1.7528	2.5	0.1678	2.4	0.96	999.8	22.0	1028.2	15.9	1089.2	12.9	1089.2	12.9
-Spot 29	353	105005	1.2	13.0714	1.0	1.7983	2.6	0.1705	2.3	0.91	1014.8	21.9	1044.8	16.7	1108.2	20.8	1108.2	20.8
-Spot 91	661	265313	1.7	12.9095	0.5	1.9716	1.5	0.1846	1.4	0.94	1092.0	14.0	1105.8	9.9	1133.1	9.6	1133.1	9.6
-Spot 68	282	72270	1.3	12.8558	0.7	1.9541	2.5	0.1822	2.4	0.96	1079.0	24.1	1099.9	16.9	1141.4	13.2	1141.4	13.2
-Spot 90	899	884849	0.9	12.8456	0.6	2.0836	2.0	0.1941	1.9	0.96	1143.6	19.9	1143.4	13.6	1143.0	11.4	1143.0	11.4
-Spot 84	161	######	0.8	12.7595	0.9	1.9645	2.4	0.1818	2.2	0.92	1076.8	22.0	1103.4	16.2	1156.3	18.3	1156.3	18.3
-Spot 66	1132	245222	4.4	12.6593	0.5	2.0828	1.5	0.1912	1.4	0.95	1128.0	14.9	1143.1	10.4	1172.0	9.8	1172.0	9.8
-Spot 34	104	34775	1.6	12.5780	0.9	2.2456	3.5	0.2049	3.4	0.97	1201.4	37.5	1195.4	24.9	1184.7	17.6	1184.7	17.6
-Spot 50	1531	181262	27.0	12.5685	0.9	2.1205	2.0	0.1933	1.8	0.90	1139.2	19.0	1155.5	14.0	1186.2	17.8	1186.2	17.8
-Spot 44	1211	70599	3.4	12.0752	0.5	2.1343	1.5	0.1869	1.4	0.94	1104.7	14.7	1160.0	10.6	1264.8	10.1	1264.8	10.1
-Spot 105	297	40771	2.8	11.6302	0.5	2.6293	1.5	0.2218	1.4	0.95	1291.3	16.9	1308.9	11.2	1337.8	9.6	1337.8	9.6
-Spot 95	2261	112325	3.5	11.3541	0.5	2.3124	1.8	0.1904	1.7	0.95	1123.7	17.8	1216.1	12.8	1384.1	10.3	1384.1	10.3
-Spot 9	267	71074	1.9	11.0389	1.1	2.8520	2.6	0.2283	2.3	0.91	1325.8	28.0	1369.3	19.3	1437.9	20.2	1437.9	20.2
-Spot 24	1093	183484	2.5	10.6879	0.7	3.2233	1.6	0.2499	1.5	0.90	1437.7	19.1	1462.8	12.7	1499.3	13.5	1499.3	13.5
-Spot 102	201	395346	1.1	10.5553	0.6	3.2392	1.9	0.2480	1.8	0.95	1428.0	23.4	1466.6	15.0	1522.9	11.5	1522.9	11.5
-Spot 18	822	170349	3.1	10.4766	0.7	3.5348	2.0	0.2686	1.8	0.94	1533.6	25.1	1535.0	15.5	1537.0	12.8	1537.0	12.8
-Spot 35	1594	247251	1.2	10.2178	0.7	3.2165	2.0	0.2384	1.9	0.94	1378.2	23.2	1461.1	15.4	1583.9	12.5	1583.9	12.5
-Spot 62	287	60436	1.7	10.1861	0.6	3.6987	2.0	0.2732	1.9	0.96	1557.3	26.0	1571.1	15.7	1589.7	10.6	1589.7	10.6
-Spot 56	194	101587	2.0	9.8767	0.6	4.0538	2.4	0.2904	2.3	0.97	1643.5	33.6	1645.1	19.4	1647.1	10.9	1647.1	10.9
-Spot 32	161	19614	1.5	9.5627	0.8	4.2378	2.1	0.2939	1.9	0.93	1661.1	27.9	1681.4	16.9	1706.8	14.0	1706.8	14.0
-Spot 15	1483	415928	1.7	9.4837	0.7	3.8981	2.6	0.2681	2.5	0.96	1531.2	34.1	1613.3	21.0	1722.1	13.0	1722.1	13.0
-Spot 88	213	155653	3.2	9.3921	0.6	4.4406	2.7	0.3025	2.7	0.98	1703.6	40.0	1719.9	22.7	1739.9	10.4	1739.9	10.4
-Spot 93	95	119019	0.9	9.0589	0.7	4.8427	2.9	0.3182	2.8	0.97	1780.8	43.4	1792.4	24.2	1805.8	12.9	1805.8	12.9
-Spot 54	123	33396	1.6	8.7328	0.7	5.2404	2.6	0.3319	2.5	0.96	1847.6	40.1	1859.2	22.2	1872.2	12.8	1872.2	12.8
-Spot 77	479	73436	3.0	8.7107	0.5	4.7521	2.4	0.3002	2.3	0.98	1692.4	34.8	1776.5	20.0	1876.8	8.3	1876.8	8.3
-Spot 86	611	694424	4.8	8.7082	0.7	5.0610	1.4	0.3196	1.3	0.88	1788.0	19.7	1829.6	12.2	1877.3	12.4	1877.3	12.4
-Spot 70	287	41043	2.7	8.7008	0.5	4.5040	2.3	0.2842	2.2	0.98	1612.6	31.8	1731.7	18.9	1878.8	8.2	1878.8	8.2
-Spot 40	343	######	5.6	8.6573	0.5	5.1120	1.9	0.3210	1.8	0.96	1794.5	27.9	1838.1	15.8	1887.8	9.3	1887.8	9.3
-Spot 28	314	78408	2.2	8.3975	0.7	5.5652	2.5	0.3389	2.4	0.95	1881.6	38.9	1910.7	21.5	1942.5	13.3	1942.5	13.3
-Spot 42	667	55845	1.9	8.0529	0.6	5.0692	1.7	0.2961	1.6	0.93	1671.8	24.0	1831.0	14.8	2017.1	11.1	2017.1	11.1
-Spot 30	118	113323	2.1	7.8346	0.7	6.6706	2.9	0.3790	2.9	0.97	2071.8	50.7	2068.7	26.0	2065.7	12.1	2065.7	12.1

-Spot 74	835	143402	4.6	6.5390	0.5	7.5231	2.4	0.3568	2.3	0.97	1966.9	39.6	2175.7	21.5	2378.9	9.3	2378.9	9.3
-Spot 83	276	63505	0.9	6.2996	0.5	8.7736	2.1	0.4009	2.0	0.97	2173.0	37.7	2314.8	19.2	2442.3	9.0	2442.3	9.0
-Spot 17	391	######	1.6	6.2924	0.5	8.8652	1.9	0.4046	1.8	0.97	2190.1	33.8	2324.2	17.1	2444.2	7.9	2444.2	7.9
-Spot 96	493	238451	1.4	6.2147	0.5	9.8078	1.9	0.4421	1.8	0.97	2359.9	35.9	2416.9	17.3	2465.2	8.1	2465.2	8.1
-Spot 3	370	329926	3.0	6.2054	0.5	9.3021	1.7	0.4186	1.6	0.95	2254.3	30.4	2368.2	15.4	2467.7	8.6	2467.7	8.6
-Spot 99	229	593554	1.4	6.1957	0.6	9.8584	2.2	0.4430	2.1	0.96	2364.0	41.0	2421.6	20.0	2470.4	10.7	2470.4	10.7
-Spot 38	238	136527	1.9	5.8185	0.4	11.7527	2.2	0.4960	2.1	0.98	2596.4	45.4	2584.9	20.3	2575.9	7.3	2575.9	7.3
-Spot 55	292	177453	6.1	4.2672	0.6	16.8776	2.3	0.5223	2.2	0.97	2709.1	49.7	2927.9	22.3	3082.0	9.7	3082.0	9.7

Table _____. U-Pb geochronologic analyses.																		
#6 U1453A_26F						Isotope ratios							Apparent ages (Ma)					
Mid Pleistocene 0.6 Ma																		
Analysis	U	206Pb	U/Th	206Pb*	±	207Pb*	±	206Pb*	±	error	206Pb*	±	207Pb*	±	206Pb*	±	Best age	±
	(ppm)	204Pb		207Pb*	(%)	235U*	(%)	238U	(%)	corr.	238U*	(Ma)	235U	(Ma)	207Pb*	(Ma)	(Ma)	(Ma)
354-U1453A-26F-1,2W_combo-Spot 35	1420	1379	84.4	26.6779	2.5	0.0118	7.2	0.0023	6.8	0.94	14.7	1.0	11.9	0.9	521.6	68.2	14.7	1.0
354-U1453A-26F-1,2W_combo-Spot 285	1382	7975	108.5	22.0010	1.7	0.0195	2.3	0.0031	1.6	0.69	20.0	0.3	19.6	0.5	31.3	41.0	20.0	0.3
354-U1453A-26F-1,2W_combo-Spot 167	764	16040	165.5	20.9474	2.6	0.0213	4.4	0.0032	3.6	0.81	20.8	0.7	21.4	0.9	86.4	61.9	20.8	0.7
354-U1453A-26F-1,2W_combo-Spot 241	2154	8612	1.6	21.7475	1.6	0.0229	2.1	0.0036	1.4	0.68	23.3	0.3	23.0	0.5	3.3	38.0	23.3	0.3
354-U1453A-26F-1,2W_combo-Spot 204	188	10603	9.6	23.3155	4.4	0.0245	5.5	0.0041	3.3	0.60	26.7	0.9	24.6	1.3	174.0	110.5	26.7	0.9
354-U1453A-26F-1,2W_combo-Spot 290	445	1286	1.9	27.8180	4.5	0.0250	4.9	0.0050	1.9	0.39	32.5	0.6	25.1	1.2	634.9	122.6	32.5	0.6
354-U1453A-26F-1,2W_combo-Spot 291	364	2330	2.0	23.9928	2.5	0.0296	3.2	0.0051	2.0	0.62	33.1	0.6	29.6	0.9	245.9	62.6	33.1	0.6
354-U1453A-26F-1,2W_combo-Spot 134	133	7147	2.1	20.1844	4.2	0.0420	6.3	0.0061	4.7	0.75	39.5	1.9	41.8	2.6	173.6	98.2	39.5	1.9
354-U1453A-26F-1,2W_combo-Spot 40	479	4819	5.8	22.2053	1.7	0.0421	2.6	0.0068	2.0	0.75	43.6	0.9	41.9	1.1	53.8	42.2	43.6	0.9
354-U1453A-26F-1,2W_combo-Spot 313	393	2224	1.6	25.7738	3.8	0.0367	4.5	0.0069	2.5	0.54	44.1	1.1	36.6	1.6	430.3	99.8	44.1	1.1
354-U1453A-26F-1,2W_combo-Spot 75	524	798125	1.7	21.2993	2.1	0.0446	2.9	0.0069	2.0	0.68	44.3	0.9	44.3	1.3	46.7	51.2	44.3	0.9
354-U1453A-26F-1,2W_combo-Spot 130	640	13367	1.0	22.4745	1.3	0.0449	2.4	0.0073	2.0	0.83	47.0	0.9	44.6	1.0	83.2	33.0	47.0	0.9
354-U1453A-26F-1,2W_combo-Spot 308	1005	6455	2.1	18.1759	2.6	0.0568	3.1	0.0075	1.8	0.58	48.1	0.9	56.1	1.7	412.9	57.1	48.1	0.9
354-U1453A-26F-1,2W_combo-Spot 118	618	12213	1.7	21.3532	1.3	0.0486	2.3	0.0075	2.0	0.83	48.4	0.9	48.2	1.1	40.7	31.2	48.4	0.9
354-U1453A-26F-1,2W_combo-Spot 37	149	3697	1.3	24.3726	3.0	0.0435	4.2	0.0077	2.9	0.69	49.3	1.4	43.2	1.8	285.7	76.2	49.3	1.4
354-U1453A-26F-1,2W_combo-Spot 67	562	2272	2.4	24.3396	1.7	0.0442	2.5	0.0078	1.9	0.74	50.1	0.9	43.9	1.1	282.3	43.5	50.1	0.9
354-U1453A-26F-1,2W_combo-Spot 61	2855	9825	1.7	21.8223	0.9	0.0496	2.0	0.0078	1.7	0.88	50.4	0.9	49.1	0.9	11.6	22.4	50.4	0.9
354-U1453A-26F-1,2W_combo-Spot 10	1503	11335	5.8	21.4737	1.2	0.0512	2.4	0.0080	2.1	0.87	51.2	1.1	50.7	1.2	27.1	28.2	51.2	1.1
354-U1453A-26F-1,2W_combo-Spot 183	363	4985	1.7	16.5010	6.2	0.0701	6.8	0.0084	3.0	0.44	53.9	1.6	68.8	4.6	625.1	132.9	53.9	1.6
354-U1453A-26F-1,2W_combo-Spot 29	660	8024	2.5	22.5429	2.1	0.0532	3.2	0.0087	2.5	0.77	55.9	1.4	52.7	1.7	90.7	50.4	55.9	1.4
354-U1453A-26F-1,2W_combo-Spot 140	201	591	1.2	43.8268	5.3	0.0274	5.8	0.0087	2.4	0.41	55.9	1.3	27.4	1.6	2095.2	202.5	55.9	1.3
354-U1453A-26F-1,2W_combo-Spot 79	98	23731	2.9	20.1305	3.8	0.0597	5.2	0.0087	3.6	0.69	55.9	2.0	58.8	3.0	179.8	88.5	55.9	2.0
354-U1453A-26F-1,2W_combo-Spot 288	365	375925	2.1	21.4591	1.6	0.0579	2.7	0.0090	2.2	0.81	57.9	1.2	57.2	1.5	28.8	38.0	57.9	1.2
354-U1453A-26F-1,2W_combo-Spot 3	1729	26873	35.6	19.1424	1.5	0.0661	5.9	0.0092	5.7	0.97	58.9	3.3	65.0	3.7	295.9	34.9	58.9	3.3
354-U1453A-26F-1,2W_combo-Spot 112	2968	112986	5.7	20.8519	0.7	0.0622	1.7	0.0094	1.6	0.92	60.3	1.0	61.3	1.0	97.1	16.5	60.3	1.0
354-U1453A-26F-1,2W_combo-Spot 95	375	2970	4.5	15.4499	2.0	0.0840	3.9	0.0094	3.4	0.86	60.4	2.0	81.9	3.1	765.4	41.7	60.4	2.0
354-U1453A-26F-1,2W_combo-Spot 22	735	16261	4.3	21.4745	1.6	0.0618	2.8	0.0096	2.2	0.81	61.8	1.4	60.9	1.6	27.0	38.4	61.8	1.4
354-U1453A-26F-1,2W_combo-Spot 47	1800	11746	1.8	21.2023	1.0	0.0635	1.9	0.0098	1.6	0.86	62.6	1.0	62.5	1.1	57.6	22.8	62.6	1.0
354-U1453A-26F-1,2W_combo-Spot 56	1096	11021	2.8	21.3298	1.2	0.0642	2.4	0.0099	2.0	0.87	63.7	1.3	63.2	1.4	43.2	28.0	63.7	1.3
354-U1453A-26F-1,2W_combo-Spot 155	874	8091	1.6	21.5746	1.6	0.0637	2.2	0.0100	1.5	0.69	64.0	1.0	62.7	1.3	15.9	38.7	64.0	1.0
354-U1453A-26F-1,2W_combo-Spot 260	180	1801	1.1	24.2052	3.5	0.0578	4.3	0.0101	2.5	0.58	65.1	1.6	57.1	2.4	268.2	89.0	65.1	1.6
354-U1453A-26F-1,2W_combo-Spot 164	119	81986	1.7	20.8521	2.7	0.0694	4.1	0.0105	3.1	0.76	67.3	2.1	68.2	2.7	97.1	63.1	67.3	2.1
354-U1453A-26F-1,2W_combo-Spot 25	248	2289	0.6	24.1527	7.8	0.0602	8.1	0.0105	2.2	0.27	67.6	1.5	59.3	4.6	262.7	197.2	67.6	1.5
354-U1453A-26F-1,2W_combo-Spot 174	383	5434	0.6	22.3463	2.5	0.0659	3.4	0.0107	2.2	0.66	68.5	1.5	64.8	2.1	69.2	61.6	68.5	1.5
354-U1453A-26F-1,2W_combo-Spot 129	189	8880	1.0	18.8836	3.8	0.0781	4.4	0.0107	2.2	0.51	68.6	1.5	76.4	3.2	326.9	85.7	68.6	1.5
354-U1453A-26F-1,2W_combo-Spot 146	194	2021	1.0	17.9815	7.6	0.0822	7.9	0.0107	2.3	0.29	68.8	1.6	80.2	6.1	436.9	169.2	68.8	1.6
354-U1453A-26F-1,2W_combo-Spot 120	334	2879	1.7	21.9662	2.1	0.0687	3.3	0.0109	2.6	0.78	70.1	1.8	67.4	2.2	27.5	50.7	70.1	1.8
354-U1453A-26F-1,2W_combo-Spot 81	297	3263	1.6	15.3017	4.1	0.1020	4.5	0.0113	1.9	0.42	72.6	1.4	98.6	4.2	785.7	85.8	72.6	1.4
354-U1453A-26F-1,2W_combo-Spot 270	494	21628	0.5	19.5399	1.3	0.0812	2.1	0.0115	1.6	0.78	73.7	1.2	79.2	1.6	248.8	29.5	73.7	1.2
354-U1453A-26F-1,2W_combo-Spot 33	1417	58685	16.1	21.1742	1.0	0.0806	2.1	0.0124	1.9	0.88	79.3	1.5	78.7	1.6	60.7	23.9	79.3	1.5
354-U1453A-26F-1,2W_combo-Spot 293	238	1642	2.3	25.5258	2.7	0.0681	3.6	0.0126	2.4	0.67	80.8	1.9	66.9	2.3	405.0	70.7	80.8	1.9
354-U1453A-26F-1,2W_combo-Spot 53	88	1444	1.4	24.9046	4.9	0.0704	7.6	0.0127	5.8	0.76	81.4	4.7	69.0	5.1	341.1	127.4	81.4	4.7
354-U1453A-26F-1,2W_combo-Spot 72	134	2245	1.6	24.9398	3.3	0.0715	4.5	0.0129	3.1	0.69	82.8	2.6	70.1	3.1	344.7	84.4	82.8	2.6
354-U1453A-26F-1,2W_combo-Spot 165	280	42150	2.9	21.7535	1.8	0.0840	3.1	0.0132	2.5	0.81	84.8	2.1	81.9	2.4	4.0	43.0	84.8	2.1
354-U1453A-26F-1,2W_combo-Spot 180	170	1277	2.2	27.4169	16.4	0.0675	16.7	0.0134	2.9	0.17	86.0	2.4	66.3	10.7	595.2	448.5	86.0	2.4
354-U1453A-26F-1,2W_combo-Spot 111	7472	98232	2.2	20.7735	0.7	0.0983	1.7	0.0148	1.5	0.91	94.8	1.4	95.2	1.5	106.0	16.2	94.8	1.4
354-U1453A-26F-1,2W_combo-Spot 162	184	4436	1.2	22.6109	1.8	0.0920	3.0	0.0151	2.4	0.80	96.5	2.3	89.4	2.6	98.0	43.8	96.5	2.3
354-U1453A-26F-1,2W_combo-Spot 280	219	6154	3.2	20.7626	1.9	0.1017	3.2	0.0153	2.6	0.80	97.9	2.5	98.3	3.0	107.3	45.5	97.9	2.5
354-U1453A-26F-1,2W_combo-Spot 151	265	7761	1.0	21.5199	2.0	0.1025	3.1	0.0160	2.4	0.78	102.3	2.5	99.1	3.0	22.0	47.3	102.3	2.5
354-U1453A-26F-1,2W_combo-Spot 219	50	33504	2.0	19.0263	4.4	0.1198	6.5	0.0165	4.7	0.73	105.7	4.9	114.9	7.0	309.8	101.1	105.7	4.9
354-U1453A-26F-1,2W_combo-Spot 121	77	1261	2.0	27.9064	3.9	0.0825	4.9	0.0167	2.9	0.60	106.7	3.1	80.5	3.8	643.6	107.4	106.7	3.1
354-U1453A-26F-1,2W_combo-Spot 283	308	15180	1.6	20.5840	1.5	0.1146	3.1	0.0171	2.8	0.88	109.4	3.0	110.2	3.3	127.7	34.7	109.4	3.0
354-U1453A-26F-1,2W_combo-Spot 170	286	6293	1.5	20.8846	1.8	0.1180	2.6	0.0179	1.8	0.70	114.2	2.0	113.2	2.7	93.4	43.0	114.2	2.0
354-U1453A-26F-1,2W_combo-Spot 296	1586	31070	15.4	17.5764	0.8	0.1445	2.2	0.0184	2.1	0.93	117.6	2.4	137.0	2.8	487.5	17.5	117.6	2.4
354-U1453A-26F-1,2W_combo-Spot 233	195	3781	0.5	22.0209	3.9	0.1186	4.7	0.0189	2.6	0.56	121.0	3.1	113.8	5.0	33.5	94.0	121.0	3.1
354-U1453A-26F-1,2W_combo-Spot 127	230	16268	1.3	20.6561	1.6	0.1278	2.7	0.0191	2.2	0.81	122.2	2.7	122.1	3.1	119.5	37.2	122.2	2.7
354-U1453A-26F-1,2W_combo-Spot 193	410	62639	0.9	21.0322	1.4	0.1260	2.5	0.0192	2.1	0.84	122.7	2.6	120.5	2.9	76.7	32.1	122.7	2.6
354-U1453A-26F-1,2W_combo-Spot 286	339	27557	1.2	20.5601	1.4	0.1295	2.2	0.0193	1.7	0.78	123.3	2.1	123.7	2.6	130.4	33.1	123.3	2.1
354-U1453A-26F-1,2W_combo-Spot 24	2287	48414	3.6	20.2687	0.6	0.1314	1.7	0.0193	1.6	0.94	123.3	2.0	125.4	2.0	163.9	13.2	123.3	2.0
354-U1453A-26F-1,2W_combo-Spot 68	3472	21900	1.7	20.0767	0.8	0.1368	2.2	0.0199	2.0	0.92	127.1	2.5	130.2	2.7	186.1	19.5	127.1	2.5
354-U1453A-26F-1,2W_combo-Spot 224	98	2344	2.0	22.5344	4.9	0.1235	6.0	0.0202	3.4	0.57	128.8	4.3	118.2	6.7	89.7	121.2	128.8	4.3
354-U1453A-26F-1,2W_combo-Spot 23	835	11547	2.1	21.0777	1.1	0.1320	2.0	0.0202	1.7	0.83	128.8	2.2	125.9	2.4	71.6	27.3	128.8	2.2
354-U1453A-26F-1,2W_combo-Spot 86	362	126212	4.2	20.3178	1.2	0.1371	2.2	0.0202	1.8	0.83	129.0	2.3	130.5	2.7	158.2	29.0	129.0	2.3
354-U1453A-26F-1,2W_combo-Spot 234	224	7349	0.9	21.1750	2.2	0.1330	3.0	0.0204	2.0	0.68	130.3	2.6	126.8	3.5	60.7	51.8	130.3	2.6
354-U1453A-26F-1,2W_combo-Spot 307	508	8302	1.0	21.5478	1.2	0.1335	2.2	0.0209	1.9	0.84	133.1	2.5	127.2	2.7	18.9	28.9	133.1	2.5
354-U1453A-26F-1,2W_combo-Spot 236	6894	109749	1.9	19.7771	0.6	0.1658	6.2	0.0238	6.2	1.00	151.5	9.3	155.8	9.0	221.0	14.2	151.5	9.3
354-U1453A-26F-1,2W_combo-Spot 306	3078	83582	22.8	17.9222	0.6	0.1831	2.6	0.0238	2.5	0.97	151.6	3.7	170.7	4.1	444.3	13.9	151.6	3.7
354-U1453A-26F-1,2W_combo-Spot 148	237	22814	3.6	17.2744	3.9	0.2165	9.5	0.0271	8.7	0.91	172.6	14.8	199.0	17.2	525.5	84.5	172.6	14.8
354-U1453A-26F-1,2W_combo-Spot 150	397	7446	3.0	21.3236	1.1	0.1773	2.1	0.0274	1.9	0.87	174.3	3.2	165.7	3.3	44.0	25.3	174.3	3.2
354-U1453A-26F-1,2W_combo-Spot 277	758	8332	6.2	20.7392	0.7	0.2102	1.9	0.0316	1.7	0.92	200.6	3.4	193.7	3.3	110.0	17.2	200.6	3.4
354-U1453A-26F-1,2W_combo-Spot 254	672	40113	1.3	19.9305	0.7	0.2327	1.8	0.0336	1.7	0.93	213.3	3.5	212.4	3.5	203.1	15.6	213.3	3.5
354-U1453A-26F-1,2W_combo-Spot 2	1216	34070	13.4	15.6186	2.4	0.3327	8.4	0.0377	8.0	0.96	238.5	18.8	291.7	21.3	742.5	50.6	238.5	18.8
354-U1453A-26F-1,2W_combo-Spot 282	240	9470	5.1	18.1201	1.6	0.2871	7.6	0.0377	7.5	0.98	238.7	17.5	256.3	17.2	419.8	34.6	238.7	17.5
354-U1453A-26F-1,2W_combo-Spot 226	647	18753	8.7	17.4489	0.7	0.4983	2.9	0.0631	2.9	0.97	394.2	10.9	410.5	10.0	503.5	16.4	394.2	10.9
354-U1453A-26F-1,2W_combo-Spot 203	660	109989	4.6	13.6720	0.5	0.6394	1.7	0.0634	1.6	0.95	396.3	6.2	501.9	6.7	1017.9	10.8	396.3	6.2
354-U1453A-26F-1,2W_combo-Spot 96	1700	48681	1.4	17.4047	0.5	0.5358	1.5	0.0676	1.4	0.93	421.9	5.6	435.6	5.2	509.1	11.7	421.9	5.6
354-U1453A-26F-1,2W_combo-Spot 161	1732	68985	34.6	17.6488	0.6	0.5404	1.6	0.0692	1.5	0.93	431.2	6.1	438.7	5.6	478.3	12.6	431.2	6.1
354-U1453A-26F-1,2W_combo-Spot 196	789	590026	7.3	17.5120	0.7	0.5483	2.1	0.0696	2.0	0.94	434.0	8.4	443.9	7.6	495.6	16.1	434.0	8.4
354-U1453A-26F-1,2W_combo-Spot 88	1370	221585	10.6	17.5688	0.7	0.5520	1.9	0.0703	1.8	0.94	438.2	7.4	446.3	6.8	488.4	14.7	438.2	7.4
354-U1453A-26F-1,2W_combo-Spot 52	2333	86213	19.0	17.8397	0.7	0.5462	1.9	0.0707	1.8	0.94	440.2	7.8	442.5	7.0	454.5	14.9	440.2	7.8
354-U1453A-26F-1,2W_combo-Spot 284	3132	138275	8.0	17.4024	0.5	0.5607	1.5	0.0708	1.4	0.93	440.8	5.8	452.0	5.3	509.4	11.6	440.8	5.8
354-U1453A-26F-1,2W_combo-Spot 110	1688	91560	2.4	17.3202	0.7	0.5642	1.9	0.0709	1.8	0.93	441.4	7.5	454.2	6.9	519.7	15.5	441.4	7.5
354-U1453A-26F-1,2W_combo-Spot 279	2885	142487	23.4	17.2328	0.5	0.5673	1.5	0.0709	1.4	0.95	441.6	6.2	456.2	5.6	530.9	10.9	441.6	6.2
354-U1453A-26F-1,2W_combo-Spot 273	2001	1371672	3.5	17.4272	0.6	0.5612	1.8	0.0709	1.7	0.93	441.8	7.1	452.3	6.5	506.3	14.1	441.8	7.1
354-U1453A-26F-1,2W_combo-Spot 201	712	66402	30.7	17.6523	0.7	0.5599	1.8	0.0717	1.7	0.93	446.3	7.4	451.5	6.7	477.9	14.6	446.3	7.4
354-U1453A-26F-1,2W_combo-Spot 50	1512	563740	6.6	17.4298	0.7	0.5686	2.2	0.0719	2.0	0.94	447.5	8.8	457.1	8.0	505.9	16.0	447.5	8.8
354-U1453A-26F-1,2W_combo-Spot 301	394	78075	1.8	17.4129	0.8	0.5742	2.2	0.0725	2.1	0.93	451.3	9.0	460.7	8.2	508.1	18.1	451.3	9.0
354-U1453A-26F-1,2W_combo-Spot 297	604	98785	0.8	17.6283	0.5	0.5761	1.4	0.0737	1.3	0.93	458.1	5.8	461.9	5.3	480.9	11.8	458.1	5.8

354-U1453A-26F-1,2W_combo-Spot 314	273	17384	1.2	17.8019	0.9	0.5722	2.5	0.0739	2.3	0.94	459.5	10.4	459.4	9.2	459.2	19.4	459.5	10.4
354-U1453A-26F-1,2W_combo-Spot 59	958	45292	5.0	17.5627	0.8	0.5830	2.1	0.0743	2.0	0.93	461.8	8.9	466.4	8.0	489.2	17.0	461.8	8.9
354-U1453A-26F-1,2W_combo-Spot 210	1694	82022	8.8	17.4979	0.6	0.5861	1.8	0.0744	1.7	0.94	462.5	7.7	468.4	6.9	497.3	13.4	462.5	7.7
354-U1453A-26F-1,2W_combo-Spot 305	1679	125448	16.1	17.3629	0.6	0.5918	1.9	0.0745	1.8	0.95	463.3	8.2	472.0	7.3	514.3	13.0	463.3	8.2
354-U1453A-26F-1,2W_combo-Spot 274	2913	542001	5.1	17.5135	0.6	0.5893	1.8	0.0748	1.7	0.95	465.3	7.8	470.4	6.9	495.4	12.9	465.3	7.8
354-U1453A-26F-1,2W_combo-Spot 89	311	38698	0.9	17.5165	0.7	0.5910	2.4	0.0751	2.3	0.95	466.7	10.4	471.5	9.2	495.0	16.5	466.7	10.4
354-U1453A-26F-1,2W_combo-Spot 311	299	21528	0.9	17.8057	1.0	0.5822	2.5	0.0752	2.3	0.92	467.3	10.3	465.9	9.3	458.8	21.4	467.3	10.3
354-U1453A-26F-1,2W_combo-Spot 30	503	46892	12.6	17.7459	0.7	0.5880	2.3	0.0757	2.2	0.95	470.3	9.9	469.6	8.7	466.2	16.5	470.3	9.9
354-U1453A-26F-1,2W_combo-Spot 269	3028	176512	2.8	17.4497	0.5	0.5982	1.3	0.0757	1.2	0.92	470.4	5.6	476.1	5.1	503.4	11.5	470.4	5.6
354-U1453A-26F-1,2W_combo-Spot 294	694	71750	0.9	17.7454	0.7	0.5897	1.8	0.0759	1.6	0.93	471.5	7.5	470.6	6.7	466.3	14.5	471.5	7.5
354-U1453A-26F-1,2W_combo-Spot 295	3377	663802	1.5	17.2603	0.6	0.6084	1.7	0.0762	1.6	0.94	473.2	7.2	482.6	6.5	527.4	12.3	473.2	7.2
354-U1453A-26F-1,2W_combo-Spot 228	1204	120494	2.5	17.4489	0.6	0.6027	1.7	0.0763	1.6	0.92	473.8	7.1	479.0	6.4	503.5	14.1	473.8	7.1
354-U1453A-26F-1,2W_combo-Spot 246	1016	257765	1.3	17.5921	0.6	0.6005	1.9	0.0766	1.8	0.95	475.9	8.1	477.5	7.1	485.5	12.7	475.9	8.1
354-U1453A-26F-1,2W_combo-Spot 256	410	33431	1.5	17.6973	0.8	0.5985	1.9	0.0768	1.8	0.92	477.1	8.1	476.3	7.3	472.3	16.9	477.1	8.1
354-U1453A-26F-1,2W_combo-Spot 199	256	26371	1.4	17.6063	0.9	0.6018	2.2	0.0768	2.0	0.92	477.3	9.2	478.4	8.3	483.7	19.0	477.3	9.2
354-U1453A-26F-1,2W_combo-Spot 303	288	35181	1.1	17.5399	0.8	0.6065	1.8	0.0772	1.6	0.89	479.1	7.4	481.4	6.9	492.1	17.7	479.1	7.4
354-U1453A-26F-1,2W_combo-Spot 153	816	37953	8.5	17.4665	0.7	0.6108	1.7	0.0774	1.5	0.91	480.4	7.1	484.0	6.5	501.3	15.5	480.4	7.1
354-U1453A-26F-1,2W_combo-Spot 144	1309	68479	0.8	17.3452	0.7	0.6154	1.8	0.0774	1.6	0.92	480.7	7.5	486.9	6.8	516.6	15.0	480.7	7.5
354-U1453A-26F-1,2W_combo-Spot 205	1492	154988	0.9	17.5082	0.6	0.6121	1.8	0.0777	1.7	0.95	482.5	7.9	484.9	6.9	496.0	12.4	482.5	7.9
354-U1453A-26F-1,2W_combo-Spot 45	1851	87246	3.4	17.2961	0.7	0.6217	1.9	0.0780	1.8	0.93	484.1	8.2	490.9	7.3	522.8	14.9	484.1	8.2
354-U1453A-26F-1,2W_combo-Spot 136	1916	163750	1.6	17.4830	0.7	0.6162	1.9	0.0781	1.7	0.93	485.0	8.0	487.5	7.2	499.2	15.4	485.0	8.0
354-U1453A-26F-1,2W_combo-Spot 8	390	24838	1.9	17.5820	0.8	0.6138	2.3	0.0783	2.1	0.93	485.8	10.0	485.9	8.8	486.8	17.9	485.8	10.0
354-U1453A-26F-1,2W_combo-Spot 158	687	35145	1.3	17.5614	0.8	0.6182	2.3	0.0787	2.1	0.93	488.6	9.9	488.7	8.8	489.3	18.4	488.6	9.9
354-U1453A-26F-1,2W_combo-Spot 169	316	30003	1.7	17.7331	0.6	0.6139	2.0	0.0790	1.9	0.95	489.9	9.0	486.0	7.7	467.8	14.3	489.9	9.0
354-U1453A-26F-1,2W_combo-Spot 82	748	495355	1.8	17.2395	0.6	0.6328	1.7	0.0791	1.6	0.93	490.9	7.5	497.8	6.8	530.0	14.0	490.9	7.5
354-U1453A-26F-1,2W_combo-Spot 97	199	57174	0.9	17.5794	0.9	0.6208	2.1	0.0791	1.9	0.89	491.0	8.8	490.3	8.1	487.1	20.6	491.0	8.8
354-U1453A-26F-1,2W_combo-Spot 93	654	56495	0.7	17.6049	0.6	0.6202	1.9	0.0792	1.8	0.94	491.3	8.5	490.0	7.4	483.9	14.0	491.3	8.5
354-U1453A-26F-1,2W_combo-Spot 192	381	186250	1.5	17.4150	0.8	0.6281	1.9	0.0793	1.8	0.91	492.1	8.3	494.9	7.6	507.8	17.6	492.1	8.3
354-U1453A-26F-1,2W_combo-Spot 145	1404	1746463	8.3	17.4294	0.7	0.6278	2.2	0.0794	2.1	0.95	492.3	9.8	494.7	8.5	505.9	14.3	492.3	9.8
354-U1453A-26F-1,2W_combo-Spot 281	244	30161	1.9	17.5020	1.0	0.6259	2.7	0.0794	2.5	0.92	492.8	11.6	493.5	10.4	496.8	22.3	492.8	11.6
354-U1453A-26F-1,2W_combo-Spot 218	940	295559	2.5	17.5168	0.6	0.6260	1.6	0.0795	1.5	0.92	493.3	7.1	493.6	6.3	494.9	13.7	493.3	7.1
354-U1453A-26F-1,2W_combo-Spot 114	4417	774678	1.4	17.2387	0.4	0.6366	1.5	0.0796	1.5	0.96	493.7	6.9	500.2	6.0	530.1	9.5	493.7	6.9
354-U1453A-26F-1,2W_combo-Spot 147	555	40432	0.9	17.5089	0.7	0.6298	2.1	0.0800	2.0	0.94	495.9	9.5	495.9	8.3	495.9	16.3	495.9	9.5
354-U1453A-26F-1,2W_combo-Spot 292	828	484689	1.3	17.2866	0.5	0.6470	1.6	0.0811	1.5	0.96	502.8	7.4	506.6	6.4	524.0	9.9	502.8	7.4
354-U1453A-26F-1,2W_combo-Spot 113	989	73966	2.2	17.4937	0.6	0.6396	1.7	0.0812	1.6	0.93	503.0	7.8	502.1	6.9	497.8	14.0	503.0	7.8
354-U1453A-26F-1,2W_combo-Spot 245	447	119274	1.8	17.2556	0.8	0.6630	2.1	0.0830	1.9	0.92	513.9	9.4	516.5	8.4	528.0	17.5	513.9	9.4
354-U1453A-26F-1,2W_combo-Spot 115	1240	135417	7.0	17.2660	0.6	0.6641	1.4	0.0832	1.3	0.90	515.0	6.4	517.1	5.8	526.7	13.6	515.0	6.4
354-U1453A-26F-1,2W_combo-Spot 223	1173	3707224	1.5	16.7550	0.7	0.7127	2.3	0.0866	2.2	0.95	535.4	11.4	546.3	9.8	592.1	15.0	535.4	11.4
354-U1453A-26F-1,2W_combo-Spot 73	878	138390	2.1	17.1895	0.6	0.6999	1.5	0.0873	1.4	0.91	539.3	7.1	538.8	6.3	536.4	13.8	539.3	7.1
354-U1453A-26F-1,2W_combo-Spot 216	667	122399	1.5	17.0033	0.6	0.7127	1.9	0.0879	1.8	0.94	543.1	9.5	546.4	8.1	560.1	13.8	543.1	9.5
354-U1453A-26F-1,2W_combo-Spot 107	1610	190106	34.7	16.5747	0.6	0.7408	2.0	0.0890	1.9	0.95	549.9	9.8	562.9	8.4	615.5	13.5	549.9	9.8
354-U1453A-26F-1,2W_combo-Spot 244	338	35845	1.0	16.9061	0.8	0.7508	2.4	0.0921	2.2	0.94	567.7	12.0	568.7	10.3	572.6	18.2	567.7	12.0
354-U1453A-26F-1,2W_combo-Spot 51	497	201917	4.7	16.9957	0.6	0.7588	2.1	0.0935	2.0	0.95	576.4	11.2	573.3	9.3	561.1	13.8	576.4	11.2
354-U1453A-26F-1,2W_combo-Spot 300	549	916643	3.3	16.7640	0.7	0.7928	2.2	0.0964	2.1	0.95	593.2	11.7	592.7	9.7	590.9	14.2	593.2	11.7
354-U1453A-26F-1,2W_combo-Spot 11	146	20928	0.8	16.8874	1.2	0.7879	2.8	0.0965	2.6	0.91	593.9	14.5	590.0	12.6	575.0	25.3	593.9	14.5
354-U1453A-26F-1,2W_combo-Spot 138	692	503044	1.2	15.7018	0.8	0.8556	2.3	0.0974	2.2	0.94	599.4	12.7	627.7	11.0	731.3	16.8	599.4	12.7
354-U1453A-26F-1,2W_combo-Spot 41	161	56020	1.5	16.2757	1.2	0.8200	2.7	0.0986	2.5	0.91	606.0	14.2	608.0	12.4	615.4	24.9	606.0	14.2
354-U1453A-26F-1,2W_combo-Spot 198	697	433904	5.9	16.3098	0.7	0.8676	3.1	0.1026	3.0	0.97	629.8	17.9	634.2	14.5	650.2	15.4	629.8	17.9
354-U1453A-26F-1,2W_combo-Spot 15	82	14930	1.0	16.5865	1.4	0.8656	3.3	0.1041	3.0	0.91	638.5	18.0	633.2	15.3	614.0	29.4	638.5	18.0
354-U1453A-26F-1,2W_combo-Spot 57	156	9097	0.7	14.3175	1.2	1.2132	3.6	0.1260	3.3	0.94	764.9	24.1	806.7	19.8	923.8	25.5	764.9	24.1
354-U1453A-26F-1,2W_combo-Spot 117	626	65838	5.1	14.6755	0.6	1.2044	3.0	0.1282	3.0	0.98	777.5	21.8	802.6	16.8	872.8	11.8	777.5	21.8
354-U1453A-26F-1,2W_combo-Spot 103	149	36456	1.5	15.4525	1.2	1.1490	2.9	0.1288	2.7	0.91	780.8	19.6	776.8	15.8	765.0	25.0	780.8	19.6
354-U1453A-26F-1,2W_combo-Spot 19	1259	248664	0.8	14.9275	0.6	1.1917	1.7	0.1290	1.5	0.93	782.3	11.3	796.8	9.1	837.5	12.7	782.3	11.3
354-U1453A-26F-1,2W_combo-Spot 261	125	38645	0.7	14.8295	1.0	1.2228	2.4	0.1315	2.2	0.92	796.5	16.7	811.1	13.6	851.2	20.3	796.5	16.7
354-U1453A-26F-1,2W_combo-Spot 87	584	175554	1.9	15.3919	0.6	1.1855	1.6	0.1323	1.5	0.94	801.2	11.5	793.9	8.9	773.3	11.8	801.2	11.5
354-U1453A-26F-1,2W_combo-Spot 253	220	349763	0.5	14.6460	0.6	1.2621	2.0	0.1341	1.9	0.96	811.0	14.6	828.8	11.3	877.0	11.8	811.0	14.6
354-U1453A-26F-1,2W_combo-Spot 230	344	37360	1.0	15.3051	0.5	1.2154	2.1	0.1349	2.0	0.97	815.9	15.7	807.7	11.8	785.2	11.2	815.9	15.7
354-U1453A-26F-1,2W_combo-Spot 227	96	8323	1.5	15.1392	1.1	1.2317	3.0	0.1352	2.8	0.93	817.7	21.2	815.1	16.6	808.1	22.8	817.7	21.2
354-U1453A-26F-1,2W_combo-Spot 69	549	146114	2.1	14.8630	0.6	1.2652	2.0	0.1364	1.9	0.96	824.2	14.5	830.3	11.1	846.5	11.5	824.2	14.5
354-U1453A-26F-1,2W_combo-Spot 208	2569	282457	1.3	15.0517	0.6	1.2517	1.9	0.1366	1.8	0.94	825.7	13.7	824.2	10.7	820.2	13.5	825.7	13.7
354-U1453A-26F-1,2W_combo-Spot 26	116	19207	1.1	15.0801	0.8	1.2935	2.9	0.1415	2.7	0.96	853.0	21.9	842.8	16.4	816.3	16.6	853.0	21.9
354-U1453A-26F-1,2W_combo-Spot 278	358	25018	1.7	14.7745	0.7	1.3213	1.8	0.1416	1.7	0.93	853.6	13.4	855.1	10.5	858.9	14.3	853.6	13.4
354-U1453A-26F-1,2W_combo-Spot 229	202	19071	0.9	14.8413	0.7	1.3316	2.0	0.1433	1.9	0.93	863.5	15.4	859.6	11.9	849.5	15.6	863.5	15.4
354-U1453A-26F-1,2W_combo-Spot 116	748	292991	23.5	14.5514	0.6	1.3773	1.8	0.1454	1.7	0.95	874.9	14.3	879.3	10.8	890.4	11.7	874.9	14.3
354-U1453A-26F-1,2W_combo-Spot 119	258	126272	1.7	14.4923	0.7	1.3981	2.3	0.1470	2.1	0.95	883.9	17.7	888.1	13.3	898.8	14.6	883.9	17.7
354-U1453A-26F-1,2W_combo-Spot 272	259	148223	1.3	14.6340	0.7	1.3958	2.1	0.1481	2.0	0.94	890.5	16.7	887.1	12.6	878.7	14.5	890.5	16.7
354-U1453A-26F-1,2W_combo-Spot 14	318	53152	2.3	14.4078	0.6	1.4782	1.8	0.1545	1.7	0.94	925.9	14.7	921.5	11.0	910.8	13.2	910.8	13.2
354-U1453A-26F-1,2W_combo-Spot 176	218	70049	1.5	14.1404	0.6	1.4816	2.6	0.1519	2.5	0.97	911.9	21.4	922.9	15.7	949.3	13.0	949.3	13.0
354-U1453A-26F-1,2W_combo-Spot 214	445	130615	1.7	14.0730	0.8	1.5940	2.5	0.1627	2.4	0.95	971.7	21.7	967.8	15.8	959.1	15.8	959.1	15.8
354-U1453A-26F-1,2W_combo-Spot 188	224	108042	1.2	14.0163	0.7	1.6504	2.2	0.1678	2.0	0.95	999.8	18.9	989.7	13.6	967.3	13.8	967.3	13.8
354-U1453A-26F-1,2W_combo-Spot 91	509	75514	4.8	13.9878	0.6	1.6274	2.1	0.1651	2.0	0.95	985.1	18.2	980.9	13.1	971.5	12.9	971.5	12.9
354-U1453A-26F-1,2W_combo-Spot 276	65	13030	1.2	13.9873	1.1	1.6139	3.4	0.1637	3.2	0.95	977.4	29.4	975.6	21.5	971.5	22.6	971.5	22.6
354-U1453A-26F-1,2W_combo-Spot 312	768	80797	3.0	13.9735	0.6	1.5776	1.9	0.1599	1.8	0.95	956.1	16.3	961.4	12.0	973.6	12.4	973.6	12.4
354-U1453A-26F-1,2W_combo-Spot 100	357	2280538	1.3	13.9685	0.7	1.6338	2.3	0.1655	2.3	0.96	987.4	20.6	983.3	14.8	974.3	13.4	974.3	13.4
354-U1453A-26F-1,2W_combo-Spot 7	72	8019	0.7	13.9628	1.2	1.6210	4.8	0.1642	4.6	0.97	979.8	41.9	978.4	29.9	975.1	24.5	975.1	24.5
354-U1453A-26F-1,2W_combo-Spot 249	3427	616097	1.6	13.9559	0.5	1.4733	2.3	0.1491	2.3	0.97	896.0	19.0	919.5	14.1	976.2	10.8	976.2	10.8
354-U1453A-26F-1,2W_combo-Spot 122	522	22866	1.9	13.9519	1.1	1.3925	2.1	0.1409	1.8	0.86	849.8	14.2	885.8	12.3	976.7	22.0	976.7	22.0
354-U1453A-26F-1,2W_combo-Spot 90	194	13991	1.3	13.9425	0.8	1.6057	2.1	0.1624	2.0	0.93	969.9	18.0	972.4	13.4	978.1	16.0	978.1	16.0
354-U1453A-26F-1,2W_combo-Spot 287	118	17367	1.8	13.9388	0.9	1.6403	2.3	0.1658	2.1	0.92	989.1	19.7	985.8	14.7	978.6	18.2	978.6	18.2
354-U1453A-26F-1,2W_combo-Spot 225	1275	122268	4.6	13.9379	0.6	1.6552	1.9	0.1673	1.8	0.95	997.3	16.5	991.5	11.9	978.7	12.5	978.7	12.5
354-U1453A-26F-1,2W_combo-Spot 83	603	89244	2.3	13.9082	0.5	1.6383	1.9	0.1653	1.8	0.96	985.9	16.8	985.0	12.0	983.1	10.7	983.1	10.7
354-U1453A-26F-1,2W_combo-Spot 248	1090	264201	9.1	13.8973	0.7	1.5910	1.8	0.1604	1.6	0.91	958.8	14.3	966.7	11.1	984.7	15.1	984.7	15.1
354-U1453A-26F-1,2W_combo-Spot 94	1714	102988	7.0	13.8752	0.6	1.5947	1.8	0.1605	1.7	0.94	959.5	15.5	968.1	11.5	988.0	12.7	988.0	12.7
354-U1453A-26F-1,2W_combo-Spot 189	64	14746	1.0	13.8314	1.0	1.4915	4.2	0.1496	4.1	0.97	898.8	34.0	926.9	25.5	994.4	21.2	994.4	21.2
354-U1453A-26F-1,2W_combo-Spot 163	312	143886	1.0	13.8169	0.6	1.6748	2.0	0.1678	1.9	0.95	1000.2	17.3	999.0	12.4	996.5	12.3	996.5	12.3
354-U1453A-26F-1,2W_combo-Spot 235	365	49255	2.3	13.8141	0.6	1.6438	1.9	0.1647	1.8	0.95	982.8	16.7	987.2	12.2	996.9	12.5	996.9	12.5
354-U1453A-26F-1,2W_combo-Spot 304	141	183867	1.3	13.8135	0.7	1.7096	2.6	0.1713	2.5	0.96	1019.2	23.3	1012.1	16.5	997.0	15.2	997.0	15.2
354-U1453A-26F-1,2W_combo-Spot 252	119	27593	0.7	13.8013	0.7	1.6282	2.8	0.1630	2.7	0.97	973.3	24.8	981.2	17.8	998.8	14.5	998.8	14.5
354-U1453A-26F-1,2W_combo-Spot 262	210	64894	1.7	13.7849	0.6	1.6346	1.9	0.1634	1.8	0.95	975.8	16.3	983.6	11.9	1001.2	11.7	1001.2	11.7
354-U1453A-26F-1,2W_combo-Spot 237	288	35709	1.6	13.6858	0.6	1.6313	1.9	0.1619	1.8	0.95	967.4	16.1	982.4	11.9	1015.8	12.0	1015.8	12.0
354-U1453A-26F-1,2W_combo-Spot 49	2057	710711	2.9	13.6827	0.5	1.4374	1.9	0.1426	1.8	0.96	859.6	14.4	904.6	11.2	1016.3	11.0	1016.3	11.0
354-U1453A-26F-1,2W_combo-Spot 168	427	365510	1.5	13.6686	0.7	1.5902	1.9	0.1576	1.7	0.92	943.7	15.0	966.4	11.6	1018.4	14.8	1018.4	14.8
354-U1453A-26F-1,2W_combo-Spot 190	1164	10267082	11.1	13.5647	0.5	1.6100	1.8	0.1584	1.8	0.96	947.8	15.7	974.1	11.6	1033.8	10.0	1033.8	10.0
354-U1453A-26F-1,2W_combo-Spot 102	601	71367	12.5	13.5090	0.6	1.6947	2.3	0.1660	2.2	0.96	990.3	20.1	1006.5	14.5	1042.1	12.4	1042.1	12.4
354-U1453A-26F-1,2W_combo-Spot 197	1309	348602	13.9	13.5031	0.6	1.5552	1.8	0.1523	1.7	0.95	913.9	14.7	952.6	11.2	1043.0	11.3	1043.0	11.3

354-U1453A-26F-1,2W_combo-Spot 298	315	31293	1.8	13.4169	0.6	1.7530	2.4	0.1706	2.3	0.97	1015.3	21.4	1028.3	15.3	1056.0	12.3	1056.0	12.3
354-U1453A-26F-1,2W_combo-Spot 20	2889	593966	2.5	13.3455	0.6	1.8575	1.8	0.1798	1.7	0.94	1065.8	16.9	1066.1	12.1	1066.7	12.3	1066.7	12.3
354-U1453A-26F-1,2W_combo-Spot 34	690	74412	0.9	13.3454	0.6	1.9165	1.9	0.1855	1.8	0.95	1097.0	17.8	1086.9	12.4	1066.7	11.6	1066.7	11.6
354-U1453A-26F-1,2W_combo-Spot 166	200	113156	1.2	13.2951	0.6	1.8335	2.5	0.1768	2.5	0.97	1049.4	23.8	1057.5	16.6	1074.3	12.6	1074.3	12.6
354-U1453A-26F-1,2W_combo-Spot 255	61	5281	0.8	13.2739	1.0	1.9872	3.2	0.1913	3.1	0.95	1128.5	32.1	1111.2	21.9	1077.5	19.6	1077.5	19.6
354-U1453A-26F-1,2W_combo-Spot 16	201	184082	1.7	13.2571	0.7	1.8861	2.6	0.1813	2.5	0.96	1074.3	24.4	1076.2	17.1	1080.0	15.0	1080.0	15.0
354-U1453A-26F-1,2W_combo-Spot 200	334	35618	1.5	13.2503	0.7	1.7189	2.2	0.1652	2.0	0.95	985.5	18.7	1015.6	13.8	1081.0	13.4	1081.0	13.4
354-U1453A-26F-1,2W_combo-Spot 231	535	95530	1.7	13.2263	0.6	1.8862	2.0	0.1809	1.9	0.95	1072.1	18.5	1076.2	13.1	1084.7	12.2	1084.7	12.2
354-U1453A-26F-1,2W_combo-Spot 173	346	200858	3.0	13.2212	0.7	1.7755	2.2	0.1703	2.1	0.95	1013.5	19.7	1036.5	14.3	1085.5	13.6	1085.5	13.6
354-U1453A-26F-1,2W_combo-Spot 99	239	202503	1.3	13.2106	0.6	1.6931	2.3	0.1622	2.2	0.96	969.1	20.0	1005.9	14.8	1087.1	12.7	1087.1	12.7
354-U1453A-26F-1,2W_combo-Spot 142	1198	587512	6.8	13.2077	0.5	1.9076	1.3	0.1827	1.2	0.92	1081.9	11.6	1083.8	8.4	1087.5	9.7	1087.5	9.7
354-U1453A-26F-1,2W_combo-Spot 39	811	125038	4.0	13.1121	0.7	1.8544	2.4	0.1764	2.3	0.96	1047.0	21.8	1065.0	15.6	1102.1	13.9	1102.1	13.9
354-U1453A-26F-1,2W_combo-Spot 143	318	93109	3.0	13.0583	1.0	1.7160	4.6	0.1625	4.5	0.98	970.8	40.8	1014.5	29.7	1110.3	19.8	1110.3	19.8
354-U1453A-26F-1,2W_combo-Spot 263	71	12085	1.6	12.9449	1.1	2.0509	3.4	0.1925	3.2	0.94	1135.2	33.2	1132.6	23.1	1127.6	22.3	1127.6	22.3
354-U1453A-26F-1,2W_combo-Spot 13	4682	794647	8.6	12.9253	0.6	1.9918	1.8	0.1867	1.7	0.95	1103.6	17.4	1112.7	12.2	1130.7	11.7	1130.7	11.7
354-U1453A-26F-1,2W_combo-Spot 217	803	397371	1.8	12.8418	0.4	2.0586	1.7	0.1917	1.7	0.97	1130.8	17.2	1135.2	11.7	1143.6	8.9	1143.6	8.9
354-U1453A-26F-1,2W_combo-Spot 310	862	292398	18.9	12.7595	0.5	2.0634	2.0	0.1910	2.0	0.97	1126.5	20.4	1136.8	14.0	1156.3	10.5	1156.3	10.5
354-U1453A-26F-1,2W_combo-Spot 48	153	4481972	1.4	12.7191	0.7	2.1100	2.6	0.1946	2.5	0.96	1146.5	26.7	1152.1	18.2	1162.6	13.9	1162.6	13.9
354-U1453A-26F-1,2W_combo-Spot 9	1192	92497	2.5	12.7033	0.6	2.0338	1.9	0.1874	1.8	0.95	1107.2	18.7	1126.9	13.1	1165.1	11.5	1165.1	11.5
354-U1453A-26F-1,2W_combo-Spot 186	595	51059	1.8	12.6668	0.5	1.9294	1.6	0.1772	1.5	0.94	1051.9	15.0	1091.3	10.9	1170.8	10.9	1170.8	10.9
354-U1453A-26F-1,2W_combo-Spot 211	342	789942	2.1	12.6657	0.8	2.1536	2.6	0.1978	2.5	0.96	1163.7	26.3	1166.2	17.9	1171.0	14.9	1171.0	14.9
354-U1453A-26F-1,2W_combo-Spot 250	1062	130192	1.9	12.6254	0.5	2.0777	1.6	0.1902	1.5	0.95	1122.7	15.7	1141.5	11.0	1177.3	10.1	1177.3	10.1
354-U1453A-26F-1,2W_combo-Spot 194	90	83962	0.3	12.5877	0.7	2.1288	3.3	0.1943	3.3	0.98	1144.9	34.2	1158.2	23.1	1183.2	14.7	1183.2	14.7
354-U1453A-26F-1,2W_combo-Spot 70	708	148243	3.3	12.4352	0.7	2.0374	2.3	0.1838	2.2	0.96	1087.4	21.9	1128.1	15.6	1207.2	13.0	1207.2	13.0
354-U1453A-26F-1,2W_combo-Spot 242	95	48171	1.9	12.2864	0.9	2.2958	3.0	0.2046	2.8	0.95	1199.9	30.9	1211.0	21.0	1230.9	17.5	1230.9	17.5
354-U1453A-26F-1,2W_combo-Spot 178	283	213684	2.2	12.2259	0.6	2.3490	2.1	0.2083	2.0	0.95	1219.7	22.1	1227.3	14.9	1240.6	12.5	1240.6	12.5
354-U1453A-26F-1,2W_combo-Spot 101	598	140873	1.5	11.9427	0.6	2.4108	2.1	0.2088	2.0	0.96	1222.5	22.3	1245.8	15.0	1286.3	11.7	1286.3	11.7
354-U1453A-26F-1,2W_combo-Spot 131	225	36147	2.1	11.8362	1.0	2.3577	2.8	0.2024	2.6	0.93	1188.2	27.9	1229.9	19.7	1303.8	19.4	1303.8	19.4
354-U1453A-26F-1,2W_combo-Spot 12	2147	956618	2.8	11.7177	0.6	2.7443	1.9	0.2332	1.8	0.95	1351.4	22.2	1340.5	14.3	1323.3	11.8	1323.3	11.8
354-U1453A-26F-1,2W_combo-Spot 221	374	1717008	8.0	11.6099	1.9	2.5598	3.6	0.2155	3.0	0.84	1258.3	34.3	1289.2	26.0	1341.2	37.1	1341.2	37.1
354-U1453A-26F-1,2W_combo-Spot 105	616	307210	8.1	11.3351	0.7	2.8095	2.2	0.2310	2.0	0.94	1339.6	24.5	1358.1	16.1	1387.3	14.1	1387.3	14.1
354-U1453A-26F-1,2W_combo-Spot 32	193	18469	1.4	11.3219	0.7	2.7647	2.7	0.2270	2.6	0.96	1318.9	30.9	1346.1	20.1	1389.5	13.9	1389.5	13.9
354-U1453A-26F-1,2W_combo-Spot 55	490	96119	17.0	10.9083	0.7	2.9449	2.1	0.2330	2.0	0.94	1350.1	24.3	1393.5	16.1	1460.6	14.1	1460.6	14.1
354-U1453A-26F-1,2W_combo-Spot 232	206	111059	0.9	10.8927	0.7	3.1759	2.3	0.2509	2.2	0.96	1443.1	28.6	1451.3	17.8	1463.3	12.6	1463.3	12.6
354-U1453A-26F-1,2W_combo-Spot 187	756	133583	1.5	10.7628	0.7	3.1002	1.8	0.2420	1.6	0.92	1397.1	20.3	1432.7	13.5	1486.1	12.9	1486.1	12.9
354-U1453A-26F-1,2W_combo-Spot 63	308	65124	1.6	10.7503	0.5	3.0496	2.3	0.2378	2.2	0.97	1375.1	27.1	1420.1	17.2	1488.3	9.9	1488.3	9.9
354-U1453A-26F-1,2W_combo-Spot 135	96	23894	2.6	10.6475	0.8	3.4068	3.0	0.2631	2.9	0.96	1505.6	38.5	1506.0	23.4	1506.5	15.5	1506.5	15.5
354-U1453A-26F-1,2W_combo-Spot 184	1312	170151	1.7	10.5822	0.7	3.2343	1.8	0.2482	1.7	0.92	1429.3	21.8	1465.4	14.3	1518.1	13.6	1518.1	13.6
354-U1453A-26F-1,2W_combo-Spot 207	556	345794	1.3	10.4696	0.5	3.0617	1.9	0.2325	1.8	0.96	1347.5	22.4	1423.2	14.7	1538.2	10.1	1538.2	10.1
354-U1453A-26F-1,2W_combo-Spot 77	365	315642	0.6	10.4523	0.8	3.0051	2.9	0.2278	2.8	0.96	1323.0	33.1	1408.9	21.8	1541.3	14.2	1541.3	14.2
354-U1453A-26F-1,2W_combo-Spot 259	165	22840	1.4	10.3715	0.5	3.5156	2.4	0.2644	2.3	0.98	1512.6	31.1	1530.7	18.7	1555.9	9.5	1555.9	9.5
354-U1453A-26F-1,2W_combo-Spot 54	218	16411	0.8	10.3170	0.8	3.8120	2.6	0.2852	2.5	0.96	1617.7	35.9	1595.3	21.1	1565.8	14.6	1565.8	14.6
354-U1453A-26F-1,2W_combo-Spot 251	1150	358118	7.9	10.2800	0.6	3.0502	2.1	0.2274	2.0	0.95	1321.0	23.5	1420.3	15.8	1572.5	11.5	1572.5	11.5
354-U1453A-26F-1,2W_combo-Spot 1	125	49067	1.4	10.2682	0.8	3.6317	2.8	0.2705	2.7	0.96	1543.1	36.9	1556.5	22.2	1574.7	14.1	1574.7	14.1
354-U1453A-26F-1,2W_combo-Spot 257	219	64750	1.1	10.2163	0.6	3.6396	2.2	0.2697	2.1	0.97	1539.2	28.9	1558.2	17.4	1584.2	10.7	1584.2	10.7
354-U1453A-26F-1,2W_combo-Spot 43	132	46638	1.6	10.0952	0.7	3.7115	2.7	0.2717	2.6	0.97	1549.7	35.4	1573.9	21.3	1606.4	12.8	1606.4	12.8
354-U1453A-26F-1,2W_combo-Spot 267	246	56147	2.2	10.0539	0.5	3.4518	2.2	0.2517	2.2	0.98	1447.2	28.5	1516.3	17.7	1614.1	8.7	1614.1	8.7
354-U1453A-26F-1,2W_combo-Spot 85	292	105011	1.2	10.0295	0.8	3.7205	2.3	0.2706	2.2	0.95	1544.0	30.0	1575.8	18.5	1618.6	14.0	1618.6	14.0
354-U1453A-26F-1,2W_combo-Spot 271	1158	3531414	1.9	10.0063	0.6	3.6354	1.3	0.2638	1.2	0.90	1509.4	16.4	1557.3	10.7	1622.9	10.7	1622.9	10.7
354-U1453A-26F-1,2W_combo-Spot 149	367	198122	1.3	10.0055	0.6	3.8628	2.1	0.2803	2.0	0.96	1592.9	28.2	1605.9	16.9	1623.0	11.3	1623.0	11.3
354-U1453A-26F-1,2W_combo-Spot 159	958	357871	5.6	9.9824	0.6	3.7752	2.1	0.2733	2.0	0.95	1557.6	27.5	1587.5	16.7	1627.3	11.6	1627.3	11.6
354-U1453A-26F-1,2W_combo-Spot 315	494	133080	0.9	9.8682	0.7	3.9608	2.2	0.2835	2.1	0.95	1608.9	30.3	1626.2	18.1	1648.7	12.5	1648.7	12.5
354-U1453A-26F-1,2W_combo-Spot 17	299	54485	1.2	9.8161	0.6	4.0813	2.1	0.2906	2.0	0.96	1644.3	28.9	1650.6	17.0	1658.5	11.0	1658.5	11.0
354-U1453A-26F-1,2W_combo-Spot 309	346	216474	1.6	9.7638	0.5	4.0057	1.4	0.2837	1.3	0.94	1609.8	19.1	1635.3	11.6	1668.4	9.1	1668.4	9.1
354-U1453A-26F-1,2W_combo-Spot 156	942	251974	2.1	9.7094	0.5	4.1204	1.9	0.2902	1.9	0.97	1642.3	27.3	1658.3	15.8	1678.7	8.8	1678.7	8.8
354-U1453A-26F-1,2W_combo-Spot 181	556	179709	2.4	9.6486	0.5	4.1660	1.9	0.2915	1.8	0.96	1649.2	26.2	1667.4	15.4	1690.3	9.8	1690.3	9.8
354-U1453A-26F-1,2W_combo-Spot 38	325	165485	1.3	9.6381	0.6	4.1407	1.9	0.2894	1.8	0.95	1638.7	26.3	1662.4	15.6	1692.3	10.6	1692.3	10.6
354-U1453A-26F-1,2W_combo-Spot 126	1173	373399	2.8	9.6246	0.5	3.8488	1.7	0.2687	1.7	0.95	1534.0	22.6	1603.0	14.0	1694.9	9.9	1694.9	9.9
354-U1453A-26F-1,2W_combo-Spot 31	1504	394008	8.5	9.5508	0.5	4.2773	1.9	0.2963	1.9	0.97	1672.9	27.4	1689.0	15.8	1709.1	8.9	1709.1	8.9
354-U1453A-26F-1,2W_combo-Spot 177	338	46039	0.9	9.5116	0.6	4.4331	2.0	0.3058	2.0	0.96	1720.1	29.7	1718.6	16.9	1716.7	10.5	1716.7	10.5
354-U1453A-26F-1,2W_combo-Spot 98	877	2599230	1.7	9.4749	0.6	4.3337	1.7	0.2978	1.6	0.94	1680.4	24.2	1699.8	14.3	1723.8	10.7	1723.8	10.7
354-U1453A-26F-1,2W_combo-Spot 264	399	716515	2.9	9.2940	0.5	4.2888	2.4	0.2891	2.3	0.98	1637.0	33.4	1691.2	19.5	1759.1	9.4	1759.1	9.4
354-U1453A-26F-1,2W_combo-Spot 247	627	183242	2.0	9.2657	0.6	4.5294	1.8	0.3044	1.6	0.93	1713.0	24.6	1736.4	14.6	1764.7	11.8	1764.7	11.8
354-U1453A-26F-1,2W_combo-Spot 60	504	161744	1.8	9.2327	0.6	4.4311	2.3	0.2967	2.2	0.96	1675.0	32.6	1718.2	19.0	1771.2	11.0	1771.2	11.0
354-U1453A-26F-1,2W_combo-Spot 46	238	110827	1.5	9.2309	0.7	4.7204	2.5	0.3160	2.4	0.96	1770.3	36.7	1770.9	20.7	1771.5	12.9	1771.5	12.9
354-U1453A-26F-1,2W_combo-Spot 58	1212	770267	12.0	9.2038	0.6	3.8076	1.6	0.2542	1.5	0.93	1459.9	19.3	1594.3	12.8	1776.9	10.9	1776.9	10.9
354-U1453A-26F-1,2W_combo-Spot 137	754	104795	3.8	9.1333	0.5	4.7467	2.0	0.3144	1.9	0.97	1762.4	29.9	1775.5	16.8	1790.9	8.6	1790.9	8.6
354-U1453A-26F-1,2W_combo-Spot 160	625	579883	3.0	8.9092	0.5	4.8851	1.5	0.3157	1.4	0.95	1768.5	22.0	1799.7	12.6	1836.0	8.4	1836.0	8.4
354-U1453A-26F-1,2W_combo-Spot 195	1581	1173834	15.6	8.9086	0.5	4.1180	1.9	0.2661	1.8	0.96	1520.8	24.5	1657.9	15.3	1836.2	9.0	1836.2	9.0
354-U1453A-26F-1,2W_combo-Spot 220	438	54635	2.3	8.8670	0.5	4.7783	2.1	0.3073	2.1	0.97	1727.4	31.1	1781.1	17.8	1844.6	9.4	1844.6	9.4
354-U1453A-26F-1,2W_combo-Spot 4	989	136238	2.0	8.8432	0.6	4.8689	2.5	0.3123	2.4	0.97	1751.9	37.4	1796.9	21.1	1849.5	10.5	1849.5	10.5
354-U1453A-26F-1,2W_combo-Spot 104	1232	215942	4.5	8.8248	0.5	4.8738	1.7	0.3119	1.6	0.95	1750.3	24.1	1797.7	14.0	1853.3	9.3	1853.3	9.3
354-U1453A-26F-1,2W_combo-Spot 108	392	106350	1.8	8.8172	0.6	5.1180	2.0	0.3273	1.9	0.95	1825.2	29.9	1839.1	16.8	1854.8	10.9	1854.8	10.9
354-U1453A-26F-1,2W_combo-Spot 66	161	189257	1.3	8.7734	0.8	5.1351	2.6	0.3267	2.5	0.95	1822.6	39.9	1841.9	22.4	1863.8	14.4	1863.8	14.4
354-U1453A-26F-1,2W_combo-Spot 266	535	30140	4.9	8.7685	0.7	5.5208	4.2	0.3511	4.1	0.99	1939.8	68.8	1903.8	35.8	1864.8	12.6	1864.8	12.6
354-U1453A-26F-1,2W_combo-Spot 213	225	186444	1.8	8.7669	0.6	5.3440	2.2	0.3398	2.1	0.97	1885.7	34.4	1875.9	18.7	1865.2	10.3	1865.2	10.3
354-U1453A-26F-1,2W_combo-Spot 175	561	101789	6.9	8.7612	0.5	4.9660	2.3	0.3156	2.2	0.98	1768.0	34.7	1813.6	19.4	1866.3	8.3	1866.3	8.3
354-U1453A-26F-1,2W_combo-Spot 238	428	134119	3.5	8.6972	0.4	5.2885	2.2	0.3336	2.1	0.98	1855.7	34.3	1867.0	18.5	1879.6	7.7	1879.6	7.7
354-U1453A-26F-1,2W_combo-Spot 268	906	1859557	3.9	8.6932	0.5	5.3842	1.9	0.3395	1.8	0.96	1884.1	29.7	1882.3	16.2	1880.4	9.0	1880.4	9.0
354-U1453A-26F-1,2W_combo-Spot 80	412	308447	2.0	8.3464	0.7	4.8984	2.5	0.2965	2.4	0.95	1674.0	34.9	1802.0	20.9	1953.4	13.2	1953.4	13.2
354-U1453A-26F-1,2W_combo-Spot 243	191	53263	1.0	8.2149	0.6	5.9862	2.6	0.3567	2.5	0.98	1966.3	42.7	1973.8	22.5	1981.7	9.9	1981.7	9.9
354-U1453A-26F-1,2W_combo-Spot 302	476	183842	1.0	7.7504	0.5	5.2865	1.7	0.2972	1.6	0.96	1677.2	24.1	1866.7	14.5	2084.7	8.4	2084.7	8.4
354-U1453A-26F-1,2W_combo-Spot 92	366	755490	1.8	7.3545	0.5	6.4984	1.7	0.3466	1.6	0.95	1918.5	26.8	2045.7	15.0	2176.5	9.4	2176.5	9.4
354-U1453A-26F-1,2W_combo-Spot 74	98	80002	1.1	6.6921	0.6	8.7191	2.6	0.4232	2.5	0.97	2274.9	48.1	2309.1	23.5	2339.4	10.0	2339.4	10.0
354-U1453A-26F-1,2W_combo-Spot 84	91	49029	2.8	6.2407	0.6	10.5811	2.6	0.4789	2.5	0.97	2522.6	52.6	2487.1	24.1	2458.2	10.5	2458.2	10.5
354-U1453A-26F-1,2W_combo-Spot 157	300	1175610	1.5	6.2112	0.5	10.4635	2.4	0.4714	2.4	0.97	2489.5	48.9	2476.7	22.5	2466.2	9.2	2466.2	9.2
354-U1453A-26F-1,2W_combo-Spot 209	284	119437	1.6	6.1434	0.6	10.2910	2.0	0.4585	1.9	0.96	2433.1	38.8	2461.3	18.5	2484.7	9.4	2484.7	9.4
354-U1453A-26F-1,2W_combo-Spot 128	123	31248	0.5	6.1151	0.6	10.1691	2.5	0.4510	2.5	0.98	2399.7	49.6	2450.3	23.5	2492.5	9.5	2492.5	9.5
354-U1453A-26F-1,2W_combo-Spot 212	131	36690	0.9	6.0831	0.7	10.7085	2.3	0.4724	2.2	0.95	2494.3	45.9	2498.2	21.7	2501.3	12.4	2501.3	12.4
354-U1453A-26F-1,2W_combo-Spot 6	1226	636083	3.4	5.6744	0.6	12.4417	2.2	0.5120	2.1	0.97	2665.3	46.0	2638.3	20.5	2617.7	9.4	2617.7	9.4
354-U1453A-26F-1,2W_combo-Spot 76	98	75165	1.4	5.4887	0.5	11.6378	3.3	0.4633	3.2	0.99	2454.0	66.3	2575.7	30.8	2672.9	9.1	2672.9	9.1
354-U1453A-26F-1,2W_combo-Spot 239	163	131221	1.9	5.2467	0.7	12.5147	3.4	0.4762	3.3	0.98	2510.8	69.0	2643.8	31.8	2747.3	10.7	2747.3	10.7

Table ______. U-Pb geochronologic analyses.																		
#7 U1452B_38F						Isotope ratios						Apparent ages (Ma)						
Early Pleistocene 1.3 Ma																		
Analysis	U	206Pb	U/Th	206Pb*	±	207Pb*	±	206Pb*	±	error	206Pb*	±	207Pb*	±	206Pb*	±	Best age	±
	(ppm)	204Pb		207Pb*	(%)	235U*	(%)	238U	(%)	corr.	238U*	(Ma)	235U	(Ma)	207Pb*	(Ma)	(Ma)	(Ma)
Gleason_354-U1452B-38F-3 and 4-Spot 313	258	677	0.8	8.4552	3.5	0.0165	4.8	0.0010	3.2	0.67	6.5	0.2	16.6	0.8	1930.2	63.5	6.5	0.2
Gleason_354-U1452B-38F-3 and 4-Spot 82	258	359	2.4	70.0301	5.7	0.0043	7.1	0.0022	4.3	0.60	14.2	0.6	4.4	0.3	0.0	0.0	14.2	0.6
Gleason_354-U1452B-38F-3 and 4-Spot 97	10338	22410	42.8	20.6333	0.9	0.0192	2.5	0.0029	2.4	0.94	18.5	0.4	19.3	0.5	122.0	20.7	18.5	0.4
Gleason_354-U1452B-38F-3 and 4-Spot 13	1938	11121	2.4	21.6804	1.2	0.0242	2.3	0.0038	1.9	0.85	24.5	0.5	24.2	0.5	4.2	29.2	24.5	0.5
Gleason_354-U1452B-38F-3 and 4-Spot 190	57	271	1.0	270.7552	57.1	0.0020	57.2	0.0040	3.5	0.06	25.9	0.9	2.1	1.2	0.0	0.0	25.9	0.9
Gleason_354-U1452B-38F-3 and 4-Spot 4	9808	200885	109.2	21.0376	0.7	0.0279	1.7	0.0043	1.5	0.92	27.4	0.4	28.0	0.5	76.1	15.7	27.4	0.4
Gleason_354-U1452B-38F-3 and 4-Spot 249	1071	3111	162.6	23.2895	1.3	0.0261	2.5	0.0044	2.1	0.84	28.3	0.6	26.2	0.6	171.2	33.3	28.3	0.6
Gleason_354-U1452B-38F-3 and 4-Spot 31	123	241	1.5	4.3156	18.2	0.1600	19.4	0.0050	6.9	0.36	32.2	2.2	150.7	27.2	3063.9	293.1	32.2	2.2
Gleason_354-U1452B-38F-3 and 4-Spot 39	176	1852	1.5	22.3893	3.3	0.0405	4.7	0.0066	3.4	0.72	42.3	1.4	40.3	1.9	73.9	80.5	42.3	1.4
Gleason_354-U1452B-38F-3 and 4-Spot 310	1421	7378	1.2	22.0114	0.9	0.0429	2.1	0.0068	2.0	0.92	44.0	0.9	42.7	0.9	32.5	20.9	44.0	0.9
Gleason_354-U1452B-38F-3 and 4-Spot 121	92	622	1.8	36.7769	6.2	0.0287	7.0	0.0077	3.3	0.48	49.2	1.6	28.8	2.0	1471.9	204.7	49.2	1.6
Gleason_354-U1452B-38F-3 and 4-Spot 55	221	7052	1.4	21.3683	2.0	0.0497	3.1	0.0077	2.3	0.76	49.5	1.1	49.3	1.5	38.9	47.4	49.5	1.1
Gleason_354-U1452B-38F-3 and 4-Spot 284	86	313	1.9	3.3296	1.0	0.3215	3.1	0.0078	3.0	0.95	49.9	1.5	283.1	7.7	3472.0	15.6	49.9	1.5
Gleason_354-U1452B-38F-3 and 4-Spot 174	243	4004	1.8	23.2051	2.3	0.0465	3.3	0.0078	2.4	0.73	50.3	1.2	46.2	1.5	162.2	56.3	50.3	1.2
Gleason_354-U1452B-38F-3 and 4-Spot 300	1436	35773	2.5	20.6743	1.1	0.0539	1.8	0.0081	1.4	0.79	51.9	0.7	53.3	0.9	117.3	25.2	51.9	0.7
Gleason_354-U1452B-38F-3 and 4-Spot 57	430	6094	1.9	21.3569	1.5	0.0529	2.8	0.0082	2.4	0.84	52.6	1.3	52.4	1.5	40.2	36.7	52.6	1.3
Gleason_354-U1452B-38F-3 and 4-Spot 44	45	519	1.6	38.7482	19.5	0.0292	20.0	0.0082	4.4	0.22	52.7	2.3	29.2	5.8	1647.9	676.6	52.7	2.3
Gleason_354-U1452B-38F-3 and 4-Spot 217	304	3174	2.1	23.0280	1.4	0.0494	2.6	0.0082	2.1	0.83	53.0	1.1	48.9	1.2	143.1	35.2	53.0	1.1
Gleason_354-U1452B-38F-3 and 4-Spot 2	530	3811	2.6	22.4835	1.1	0.0514	2.7	0.0084	2.4	0.90	53.8	1.3	50.9	1.3	84.2	27.9	53.8	1.3
Gleason_354-U1452B-38F-3 and 4-Spot 170	183	10776	1.4	20.9237	1.8	0.0568	2.8	0.0086	2.2	0.77	55.3	1.2	56.1	1.5	89.0	42.3	55.3	1.2
Gleason_354-U1452B-38F-3 and 4-Spot 72	38	1917	1.9	24.9361	6.4	0.0487	8.0	0.0088	4.8	0.61	56.5	2.7	48.3	3.8	344.3	164.1	56.5	2.7
Gleason_354-U1452B-38F-3 and 4-Spot 69	92	954	2.8	30.3044	9.4	0.0403	9.8	0.0089	2.8	0.28	56.9	1.6	40.2	3.9	875.4	272.2	56.9	1.6
Gleason_354-U1452B-38F-3 and 4-Spot 163	344	14044	1.4	20.5677	1.5	0.0606	2.7	0.0090	2.3	0.83	58.0	1.3	59.7	1.6	129.6	35.9	58.0	1.3
Gleason_354-U1452B-38F-3 and 4-Spot 94	709	45932	3.0	20.3103	1.1	0.0624	1.9	0.0092	1.6	0.82	59.0	0.9	61.4	1.1	159.1	26.1	59.0	0.9
Gleason_354-U1452B-38F-3 and 4-Spot 70	1006	13367	3.2	21.0008	1.1	0.0607	2.1	0.0092	1.8	0.86	59.3	1.0	59.8	1.2	80.3	25.2	59.3	1.0
Gleason_354-U1452B-38F-3 and 4-Spot 88	1898	18882	3.4	21.1083	0.9	0.0607	2.4	0.0093	2.2	0.93	59.7	1.3	59.9	1.4	68.1	20.4	59.7	1.3
Gleason_354-U1452B-38F-3 and 4-Spot 308	514	46223	1.9	20.5039	1.2	0.0641	2.0	0.0095	1.7	0.82	61.2	1.0	63.1	1.2	136.8	27.5	61.2	1.0
Gleason_354-U1452B-38F-3 and 4-Spot 261	233	21457	1.2	20.4119	2.3	0.0646	3.3	0.0096	2.3	0.71	61.3	1.4	63.5	2.0	147.4	54.2	61.3	1.4
Gleason_354-U1452B-38F-3 and 4-Spot 245	1505	21759	1.4	19.9399	1.6	0.0664	2.3	0.0096	1.6	0.71	61.6	1.0	65.3	1.4	202.0	37.5	61.6	1.0
Gleason_354-U1452B-38F-3 and 4-Spot 119	72	677	1.7	4.0165	3.6	0.3328	4.3	0.0097	2.4	0.55	62.2	1.5	291.7	10.8	3178.3	56.3	62.2	1.5
Gleason_354-U1452B-38F-3 and 4-Spot 241	368	12630	1.6	21.2812	1.4	0.0646	2.4	0.0100	2.0	0.82	63.9	1.2	63.5	1.5	48.7	32.6	63.9	1.2
Gleason_354-U1452B-38F-3 and 4-Spot 303	109	1386	1.5	24.6198	3.3	0.0578	4.2	0.0103	2.6	0.62	66.2	1.7	57.1	2.3	311.5	85.2	66.2	1.7
Gleason_354-U1452B-38F-3 and 4-Spot 280	5174	3126	1.3	11.9099	2.0	0.1203	8.2	0.0104	8.0	0.97	66.6	5.3	115.3	9.0	1291.7	39.3	66.6	5.3
Gleason_354-U1452B-38F-3 and 4-Spot 35	253	8206	0.9	20.0297	2.5	0.0747	3.6	0.0109	2.6	0.73	69.6	1.8	73.2	2.6	191.6	58.2	69.6	1.8
Gleason_354-U1452B-38F-3 and 4-Spot 127	413	4105	6.0	21.6959	1.5	0.0812	2.4	0.0128	1.9	0.79	81.8	1.6	79.3	1.8	2.4	35.6	81.8	1.6
Gleason_354-U1452B-38F-3 and 4-Spot 101	81	701	1.6	35.7314	3.3	0.0502	4.6	0.0130	3.2	0.69	83.4	2.6	49.8	2.2	1377.7	106.9	83.4	2.6
Gleason_354-U1452B-38F-3 and 4-Spot 291	57	1320	2.2	24.9515	6.2	0.0732	6.9	0.0133	3.2	0.46	84.9	2.7	71.8	4.8	345.9	159.2	84.9	2.7
Gleason_354-U1452B-38F-3 and 4-Spot 129	90	1255	1.2	24.8489	3.1	0.0740	4.6	0.0133	3.4	0.74	85.3	2.9	72.4	3.2	335.3	79.2	85.3	2.9
Gleason_354-U1452B-38F-3 and 4-Spot 93	225	3380	1.2	21.6999	3.4	0.0863	4.0	0.0136	2.2	0.53	87.0	1.9	84.1	3.3	2.0	82.2	87.0	1.9
Gleason_354-U1452B-38F-3 and 4-Spot 221	86	969	1.5	30.4588	3.3	0.0621	4.4	0.0137	2.9	0.66	87.9	2.6	61.2	2.6	890.1	96.6	87.9	2.6
Gleason_354-U1452B-38F-3 and 4-Spot 95	126	32220	3.0	19.9737	1.8	0.1155	3.3	0.0167	2.8	0.84	107.0	3.0	111.0	3.5	198.1	41.3	107.0	3.0
Gleason_354-U1452B-38F-3 and 4-Spot 239	981	25735	0.8	20.4540	0.8	0.1137	2.0	0.0169	1.8	0.91	107.8	1.9	109.3	2.1	142.6	19.5	107.8	1.9
Gleason_354-U1452B-38F-3 and 4-Spot 169	3082	5614	1.8	15.2542	3.9	0.1547	4.4	0.0171	1.9	0.44	109.4	2.1	146.1	6.0	792.2	82.4	109.4	2.1
Gleason_354-U1452B-38F-3 and 4-Spot 160	529	12296	1.7	20.5345	1.4	0.1170	2.3	0.0174	1.8	0.80	111.4	2.0	112.4	2.4	133.4	32.5	111.4	2.0
Gleason_354-U1452B-38F-3 and 4-Spot 311	179	3658	7.6	16.8043	1.8	0.1441	3.4	0.0176	2.9	0.85	112.2	3.2	136.7	4.3	585.8	38.1	112.2	3.2
Gleason_354-U1452B-38F-3 and 4-Spot 225	452	17479	2.5	20.4795	1.3	0.1195	2.5	0.0177	2.2	0.86	113.4	2.4	114.6	2.7	139.7	30.2	113.4	2.4
Gleason_354-U1452B-38F-3 and 4-Spot 216	665	9425	1.5	21.0039	0.8	0.1167	1.9	0.0178	1.7	0.91	113.6	1.9	112.0	2.0	79.9	17.9	113.6	1.9
Gleason_354-U1452B-38F-3 and 4-Spot 135	594	155766	1.5	20.1250	0.9	0.1236	1.7	0.0180	1.5	0.85	115.3	1.7	118.4	1.9	180.5	21.6	115.3	1.7
Gleason_354-U1452B-38F-3 and 4-Spot 155	63	11657	9.1	19.7318	3.1	0.1355	4.9	0.0194	3.8	0.77	123.8	4.6	129.1	5.9	226.3	72.4	123.8	4.6
Gleason_354-U1452B-38F-3 and 4-Spot 80	377	23370	3.9	20.3924	1.2	0.1333	2.6	0.0197	2.3	0.89	125.8	2.8	127.0	3.1	149.7	27.6	125.8	2.8
Gleason_354-U1452B-38F-3 and 4-Spot 254	760	20460	7.9	20.6132	0.9	0.1319	2.3	0.0197	2.1	0.92	125.9	2.6	125.8	2.7	124.3	21.3	125.9	2.6
Gleason_354-U1452B-38F-3 and 4-Spot 25	824	9839	2.2	20.9839	0.9	0.1296	1.9	0.0197	1.6	0.87	125.9	2.0	123.7	2.2	82.2	21.8	125.9	2.0
Gleason_354-U1452B-38F-3 and 4-Spot 252	224	3883	0.9	21.2741	1.8	0.1295	3.0	0.0200	2.3	0.78	127.6	2.9	123.7	3.4	49.5	43.9	127.6	2.9
Gleason_354-U1452B-38F-3 and 4-Spot 273	95	7470	1.6	20.2258	1.9	0.1379	3.5	0.0202	3.0	0.84	129.1	3.8	131.2	4.4	168.8	44.7	129.1	3.8
Gleason_354-U1452B-38F-3 and 4-Spot 7	206	539732	2.0	19.4653	1.6	0.1445	2.8	0.0204	2.3	0.82	130.2	2.9	137.1	3.6	257.6	36.9	130.2	2.9
Gleason_354-U1452B-38F-3 and 4-Spot 110	676	49632	2.1	20.0813	0.9	0.1416	2.2	0.0206	2.0	0.91	131.6	2.6	134.4	2.7	185.5	20.7	131.6	2.6
Gleason_354-U1452B-38F-3 and 4-Spot 187	321	24876	1.6	19.4891	1.2	0.1508	2.6	0.0213	2.3	0.89	136.0	3.2	142.6	3.5	254.8	27.9	136.0	3.2
Gleason_354-U1452B-38F-3 and 4-Spot 14	96	760	1.1	7.8950	1.4	0.4134	2.6	0.0237	2.2	0.85	150.8	3.3	351.3	7.8	2052.1	24.4	150.8	3.3
Gleason_354-U1452B-38F-3 and 4-Spot 43	4371	#######	474.8	19.9387	0.7	0.2060	2.1	0.0298	2.0	0.94	189.2	3.7	190.2	3.6	202.1	16.2	189.2	3.7
Gleason_354-U1452B-38F-3 and 4-Spot 116	283	62899	1.9	19.5041	1.0	0.2127	2.1	0.0301	1.8	0.88	191.1	3.5	195.8	3.7	253.1	22.9	191.1	3.5
Gleason_354-U1452B-38F-3 and 4-Spot 49	858	25451	3.1	19.8536	0.7	0.2225	1.8	0.0320	1.7	0.92	203.3	3.3	204.0	3.3	212.0	16.4	203.3	3.3
Gleason_354-U1452B-38F-3 and 4-Spot 117	1180	227345	6.9	19.6553	0.7	0.2285	2.1	0.0326	1.9	0.93	206.6	3.9	208.9	3.9	235.3	16.9	206.6	3.9
Gleason_354-U1452B-38F-3 and 4-Spot 177	115	1465	1.3	9.4497	1.1	0.4842	2.5	0.0332	2.2	0.90	210.5	4.6	401.0	8.2	1728.7	19.6	210.5	4.6
Gleason_354-U1452B-38F-3 and 4-Spot 212	788	44330	1.7	19.6968	0.8	0.2358	2.0	0.0337	1.9	0.93	213.5	4.0	215.0	3.9	230.4	17.6	213.5	4.0
Gleason_354-U1452B-38F-3 and 4-Spot 259	1074	93100	4.9	20.0344	0.9	0.2358	2.2	0.0343	2.0	0.91	217.1	4.3	214.9	4.3	191.0	21.4	217.1	4.3
Gleason_354-U1452B-38F-3 and 4-Spot 90	953	67679	21.5	15.4302	2.6	0.3389	4.1	0.0379	3.1	0.77	240.0	7.4	296.4	10.6	768.1	55.6	240.0	7.4
Gleason_354-U1452B-38F-3 and 4-Spot 48	960	109013	9.8	17.8691	0.9	0.3373	2.5	0.0437	2.4	0.94	275.8	6.4	295.1	6.5	450.9	19.5	275.8	6.4
Gleason_354-U1452B-38F-3 and 4-Spot 173	285	7280152	3.2	16.6191	1.5	0.3833	3.1	0.0462	2.7	0.87	291.1	7.6	329.4	8.6	609.7	32.2	291.1	7.6
Gleason_354-U1452B-38F-3 and 4-Spot 211	1519	119862	118.9	17.7835	0.7	0.4208	2.1	0.0543	2.0	0.95	340.7	6.7	356.6	6.4	461.5	14.5	340.7	6.7
Gleason_354-U1452B-38F-3 and 4-Spot 137	218	27344	3.0	17.1097	0.8	0.4544	2.1	0.0564	1.9	0.93	353.6	6.7	380.3	6.6	546.5	16.6	353.6	6.7
Gleason_354-U1452B-38F-3 and 4-Spot 6	3418	177369	18.9	17.4968	0.7	0.4463	2.2	0.0566	2.1	0.96	355.2	7.4	374.7	7.0	497.5	14.6	355.2	7.4
Gleason_354-U1452B-38F-3 and 4-Spot 53	335	25303	3.8	17.5606	0.7	0.4631	2.8	0.0590	2.7	0.97	369.5	9.7	386.4	9.0	489.4	15.6	369.5	9.7
Gleason_354-U1452B-38F-3 and 4-Spot 91	676	26154	5.7	12.1078	0.7	0.6871	2.0	0.0603	1.9	0.94	377.7	7.0	531.0	8.4	1259.6	13.5	377.7	7.0
Gleason_354-U1452B-38F-3 and 4-Spot 309	179	42071	2.2	16.8205	0.8	0.5192	2.5	0.0633	2.3	0.94	395.9	9.0	424.6	8.6	583.6	17.9	395.9	9.0
Gleason_354-U1452B-38F-3 and 4-Spot 11	1390	369367	2.7	17.3456	0.7	0.5681	1.8	0.0715	1.7	0.92	445.0	7.1	456.8	6.6	516.5	14.9	445.0	7.1
Gleason_354-U1452B-38F-3 and 4-Spot 74	255	40115	1.6	17.3725	0.8	0.5710	2.6	0.0719	2.5	0.95	447.9	10.9	458.7	9.8	513.1	17.8	447.9	10.9
Gleason_354-U1452B-38F-3 and 4-Spot 238	432	159712	1.7	17.5581	0.8	0.5786	2.5	0.0737	2.3	0.94	458.3	10.3	463.5	9.2	489.7	18.4	458.3	10.3
Gleason_354-U1452B-38F-3 and 4-Spot 79	913	90734	1.2	17.5837	0.7	0.5852	2.4	0.0746	2.3	0.95	464.0	10.1	467.8	8.9	486.6	15.6	464.0	10.1
Gleason_354-U1452B-38F-3 and 4-Spot 54	58	7473	0.8	17.5643	1.4	0.5910	3.8	0.0753	3.5	0.93	467.9	15.8	471.5	14.2	489.0	29.9	467.9	15.8
Gleason_354-U1452B-38F-3 and 4-Spot 138	1115	51334	17.1	17.5174	0.5	0.5930	1.5	0.0753	1.4	0.94	468.2	6.3	472.8	5.6	494.8	11.1	468.2	6.3
Gleason_354-U1452B-38F-3 and 4-Spot 276	679	39618	3.5	17.6559	0.6	0.5916	1.8	0.0758	1.6	0.93	470.7	7.4	471.9	6.6	477.5	14.0	470.7	7.4
Gleason_354-U1452B-38F-3 and 4-Spot 15	205	29991	1.7	17.3336	0.8	0.6032	2.2	0.0758	2.0	0.93	471.2	9.2	479.2	8.3	518.0	17.2	471.2	9.2
Gleason_354-U1452B-38F-3 and 4-Spot 272	447	1379200	1.6	17.5516	0.8	0.5969	2.0	0.0760	1.8	0.92	472.1	8.3	475.3	7.5	490.6	16.7	472.1	8.3
Gleason_354-U1452B-38F-3 and 4-Spot 37	637	34175	9.0	17.6319	0.6	0.5951	1.9	0.0761	1.8	0.95	472.8	8.3	474.1	7.2	480.5	13.0	472.8	8.3
Gleason_354-U1452B-38F-3 and 4-Spot 157	359	51082	3.0	17.4958	0.7	0.6003	2.0	0.0762	1.8	0.93	473.2	8.4	477.4	7.6	497.6	16.5	473.2	8.4
Gleason_354-U1452B-38F-3 and 4-Spot 52	263	17937	1.5	17.5652	0.8	0.6012	2.4	0.0766	2.2	0.94	475.7	10.1	478.0	9.0	488.8	18.4	475.7	10.1
Gleason_354-U1452B-38F-3 and 4-Spot 3	765	440960	9.9	17.5417	0.6	0.6023	2.0	0.0766	1.8	0.94	475.9	8.4	478.7	7.4	491.8	14.3	475.9	8.4
Gleason_354-U1452B-38F-3 and 4-Spot 214	222	9205	1.4	17.1381	1.8	0.6173	3.3	0.0767	2.7	0.83	476.6	12.4	488.2	12.6	542.9	39.9	476.6	12.4

Gleason_354-U1452B-38F-3 and 4-Spot 294	203	11823	1.5	17.5269	1.1	0.6043	2.4	0.0768	2.2	0.90	477.1	10.0	480.0	9.2	493.7	23.7	477.1	10.0
Gleason_354-U1452B-38F-3 and 4-Spot 196	2280	611823	1.0	17.5062	0.5	0.6058	2.0	0.0769	1.9	0.96	477.7	8.9	480.9	7.7	496.3	12.0	477.7	8.9
Gleason_354-U1452B-38F-3 and 4-Spot 258	624	31170	1.5	17.6575	0.7	0.6062	1.9	0.0776	1.8	0.93	482.0	8.3	481.2	7.3	477.3	15.2	482.0	8.3
Gleason_354-U1452B-38F-3 and 4-Spot 200	107	30213	1.7	17.4532	1.0	0.6142	2.6	0.0778	2.4	0.92	482.7	11.0	486.2	9.9	503.0	21.8	482.7	11.0
Gleason_354-U1452B-38F-3 and 4-Spot 191	76	35864	1.4	17.3315	1.0	0.6245	2.5	0.0785	2.3	0.92	487.2	10.6	492.7	9.6	518.3	21.7	487.2	10.6
Gleason_354-U1452B-38F-3 and 4-Spot 120	452	56908	3.1	17.1706	0.7	0.6311	2.5	0.0786	2.4	0.97	487.7	11.5	496.8	10.0	538.8	14.3	487.7	11.5
Gleason_354-U1452B-38F-3 and 4-Spot 112	4970	233089	4.2	17.6918	0.7	0.6141	2.3	0.0788	2.2	0.96	488.9	10.2	486.1	8.8	473.0	14.5	488.9	10.2
Gleason_354-U1452B-38F-3 and 4-Spot 205	1407	343412	1.7	17.2106	0.7	0.6318	2.1	0.0789	1.9	0.93	489.4	9.1	497.2	8.1	533.7	16.3	489.4	9.1
Gleason_354-U1452B-38F-3 and 4-Spot 124	660	683098	6.1	17.4846	0.7	0.6223	2.4	0.0789	2.3	0.95	489.6	10.8	491.3	9.3	499.0	15.6	489.6	10.8
Gleason_354-U1452B-38F-3 and 4-Spot 50	981	145232	0.6	17.4548	0.7	0.6241	1.6	0.0790	1.4	0.89	490.2	6.6	492.4	6.2	502.8	16.0	490.2	6.6
Gleason_354-U1452B-38F-3 and 4-Spot 107	594	47755	4.0	17.5022	0.6	0.6229	1.9	0.0791	1.8	0.96	490.6	8.7	491.7	7.5	496.8	12.3	490.6	8.7
Gleason_354-U1452B-38F-3 and 4-Spot 179	1098	117676	295.4	17.2518	0.6	0.6321	1.8	0.0791	1.7	0.94	490.7	8.1	497.4	7.2	528.5	13.7	490.7	8.1
Gleason_354-U1452B-38F-3 and 4-Spot 41	4742	307497	22.1	17.4138	0.5	0.6306	1.7	0.0796	1.7	0.96	494.0	7.9	496.5	6.8	507.9	10.3	494.0	7.9
Gleason_354-U1452B-38F-3 and 4-Spot 290	649	87476	1.9	17.5084	0.8	0.6305	2.4	0.0801	2.3	0.95	496.5	10.9	496.4	9.5	496.0	17.2	496.5	10.9
Gleason_354-U1452B-38F-3 and 4-Spot 271	511	43339	1.8	17.4500	0.6	0.6355	2.1	0.0804	2.0	0.95	498.7	9.4	499.5	8.1	503.4	13.9	498.7	9.4
Gleason_354-U1452B-38F-3 and 4-Spot 29	3952	224559	8.2	17.4173	0.7	0.6428	1.8	0.0812	1.7	0.92	503.3	8.2	504.0	7.3	507.5	15.6	503.3	8.2
Gleason_354-U1452B-38F-3 and 4-Spot 36	426	37768	2.1	17.2816	0.9	0.6548	2.4	0.0821	2.2	0.93	508.5	11.0	511.5	9.7	524.6	20.1	508.5	11.0
Gleason_354-U1452B-38F-3 and 4-Spot 60	164	131452	1.7	16.9152	1.2	0.6740	2.5	0.0827	2.2	0.87	512.1	10.6	523.1	10.1	571.4	26.6	512.1	10.6
Gleason_354-U1452B-38F-3 and 4-Spot 244	993	1779155	1.6	17.3494	0.5	0.6685	1.6	0.0841	1.5	0.94	520.7	7.4	519.8	6.4	516.0	12.0	520.7	7.4
Gleason_354-U1452B-38F-3 and 4-Spot 45	329	87077	1.3	17.2936	0.7	0.6731	1.8	0.0844	1.6	0.91	522.5	8.1	522.6	7.2	523.1	15.7	522.5	8.1
Gleason_354-U1452B-38F-3 and 4-Spot 162	896	103194	1.9	17.3183	0.8	0.6864	1.9	0.0862	1.7	0.91	533.1	8.7	530.7	7.7	520.0	17.0	533.1	8.7
Gleason_354-U1452B-38F-3 and 4-Spot 278	2980	660618	5.2	17.1531	0.8	0.7023	2.1	0.0874	1.9	0.92	539.9	9.8	540.1	8.7	541.0	18.3	539.9	9.8
Gleason_354-U1452B-38F-3 and 4-Spot 47	133	10730	2.6	16.8968	0.9	0.7342	2.4	0.0900	2.2	0.93	555.4	11.7	559.0	10.1	573.9	18.6	555.4	11.7
Gleason_354-U1452B-38F-3 and 4-Spot 178	2980	329977	11.4	16.7984	0.7	0.7535	2.4	0.0918	2.3	0.96	566.2	12.3	570.2	10.4	586.5	14.8	566.2	12.3
Gleason_354-U1452B-38F-3 and 4-Spot 209	84	24592	0.8	16.4884	0.9	0.8009	3.1	0.0958	3.0	0.96	589.6	17.0	597.4	14.2	626.8	19.1	589.6	17.0
Gleason_354-U1452B-38F-3 and 4-Spot 158	115	12662	1.5	16.7477	1.1	0.7944	3.0	0.0965	2.8	0.93	593.8	15.8	593.7	13.4	593.1	23.1	593.8	15.8
Gleason_354-U1452B-38F-3 and 4-Spot 46	982	47566	16.9	15.6665	0.7	0.8509	2.0	0.0967	1.9	0.93	594.9	10.6	625.1	9.4	736.0	15.8	594.9	10.6
Gleason_354-U1452B-38F-3 and 4-Spot 189	161	36262	0.7	16.6031	0.8	0.8036	2.7	0.0968	2.6	0.95	595.4	14.6	598.9	12.2	611.8	18.0	595.4	14.6
Gleason_354-U1452B-38F-3 and 4-Spot 226	787	102996	4.4	15.6965	0.8	0.8527	3.1	0.0971	3.0	0.97	597.2	17.2	626.1	14.6	732.0	17.2	597.2	17.2
Gleason_354-U1452B-38F-3 and 4-Spot 213	98	33158	1.4	15.9183	1.2	0.8622	3.2	0.0995	2.9	0.93	611.7	17.1	631.3	14.8	702.2	24.7	611.7	17.1
Gleason_354-U1452B-38F-3 and 4-Spot 243	329	128179	7.1	15.6462	1.2	0.9134	3.8	0.1037	3.6	0.95	635.8	21.9	658.9	18.4	738.7	24.4	635.8	21.9
Gleason_354-U1452B-38F-3 and 4-Spot 274	769	454997	14.2	15.0693	1.0	1.0892	2.2	0.1190	2.0	0.90	725.0	13.7	748.1	11.8	817.8	20.6	725.0	13.7
Gleason_354-U1452B-38F-3 and 4-Spot 92	2362	121434	6.3	14.7544	0.7	1.1527	1.9	0.1233	1.7	0.94	749.8	12.4	778.5	10.2	861.7	13.5	749.8	12.4
Gleason_354-U1452B-38F-3 and 4-Spot 256	802	40803	4.4	14.9183	1.0	1.1567	2.0	0.1252	1.7	0.87	760.2	12.3	780.4	10.8	838.7	20.5	760.2	12.3
Gleason_354-U1452B-38F-3 and 4-Spot 12	97	17923	1.6	15.1690	1.1	1.1427	3.3	0.1257	3.1	0.94	763.4	22.1	773.8	17.7	803.9	23.0	763.4	22.1
Gleason_354-U1452B-38F-3 and 4-Spot 59	626	721911	2.1	14.9152	0.6	1.2013	2.6	0.1300	2.5	0.97	787.6	18.9	801.2	14.6	839.2	13.4	787.6	18.9
Gleason_354-U1452B-38F-3 and 4-Spot 253	1293	181956	3.6	14.7958	0.6	1.2137	1.8	0.1302	1.7	0.94	789.3	12.9	806.9	10.3	855.9	13.0	789.3	12.9
Gleason_354-U1452B-38F-3 and 4-Spot 122	495	336188	2.8	14.8425	0.7	1.2159	1.9	0.1309	1.8	0.93	792.9	13.5	807.9	10.8	849.3	14.3	792.9	13.5
Gleason_354-U1452B-38F-3 and 4-Spot 266	304	61769	3.6	15.0959	0.7	1.2111	2.2	0.1326	2.0	0.94	802.6	15.4	805.7	12.0	814.1	15.5	802.6	15.4
Gleason_354-U1452B-38F-3 and 4-Spot 176	170	644383	3.6	15.0447	0.7	1.2197	2.4	0.1331	2.3	0.95	805.4	17.5	809.6	13.5	821.2	15.1	805.4	17.5
Gleason_354-U1452B-38F-3 and 4-Spot 301	200	97494	1.5	14.9596	0.7	1.2398	2.0	0.1345	1.9	0.94	813.6	14.3	818.8	11.1	833.0	14.0	813.6	14.3
Gleason_354-U1452B-38F-3 and 4-Spot 134	585	44906	4.6	14.5171	0.6	1.2948	1.7	0.1363	1.6	0.93	823.8	12.5	843.4	9.9	895.3	13.0	823.8	12.5
Gleason_354-U1452B-38F-3 and 4-Spot 152	580	868562	2.4	14.7470	0.6	1.2749	2.3	0.1364	2.2	0.96	824.0	16.9	834.6	12.9	862.8	13.2	824.0	16.9
Gleason_354-U1452B-38F-3 and 4-Spot 237	794	943287	2.9	14.9930	0.5	1.2679	1.8	0.1379	1.8	0.97	832.6	13.8	831.5	10.4	828.4	9.7	832.6	13.8
Gleason_354-U1452B-38F-3 and 4-Spot 30	91	32445	1.5	14.7074	0.8	1.3093	2.7	0.1397	2.6	0.96	842.7	20.5	849.8	15.6	868.3	16.7	842.7	20.5
Gleason_354-U1452B-38F-3 and 4-Spot 32	3102	414405	1.2	15.1156	0.6	1.2746	1.8	0.1397	1.7	0.94	843.2	13.6	834.5	10.3	811.4	12.4	843.2	13.6
Gleason_354-U1452B-38F-3 and 4-Spot 240	104	72318	1.0	14.7885	0.7	1.3041	2.9	0.1399	2.8	0.97	843.9	22.5	847.5	16.9	856.9	15.3	843.9	22.5
Gleason_354-U1452B-38F-3 and 4-Spot 141	236	35399	2.7	14.7139	0.7	1.3196	2.1	0.1408	1.9	0.94	849.3	15.3	854.4	11.8	867.4	14.5	849.3	15.3
Gleason_354-U1452B-38F-3 and 4-Spot 172	194	18291	1.9	14.9318	0.7	1.3024	2.2	0.1410	2.1	0.95	850.6	16.9	846.8	12.7	836.9	13.7	850.6	16.9
Gleason_354-U1452B-38F-3 and 4-Spot 102	306	33401	2.5	14.7731	0.6	1.3200	2.1	0.1414	2.0	0.96	852.8	16.2	854.5	12.3	859.1	12.9	852.8	16.2
Gleason_354-U1452B-38F-3 and 4-Spot 281	341	414811	2.0	14.6927	0.6	1.3318	2.0	0.1419	1.9	0.95	855.5	14.9	859.7	11.4	870.4	12.5	855.5	14.9
Gleason_354-U1452B-38F-3 and 4-Spot 85	460	71347	4.2	14.6758	0.5	1.3413	2.1	0.1428	2.0	0.97	860.3	16.3	863.8	12.1	872.8	10.8	860.3	16.3
Gleason_354-U1452B-38F-3 and 4-Spot 199	183	16579	0.9	14.4383	0.7	1.3866	2.5	0.1452	2.4	0.96	874.0	19.5	883.3	14.6	906.5	13.7	874.0	19.5
Gleason_354-U1452B-38F-3 and 4-Spot 283	482	425053	2.4	14.4043	0.7	1.4112	2.0	0.1474	1.9	0.94	886.5	15.5	893.7	11.9	911.3	14.5	886.5	15.5
Gleason_354-U1452B-38F-3 and 4-Spot 184	323	3842835	0.7	14.0801	0.8	1.5406	2.1	0.1573	2.0	0.93	941.9	17.5	946.7	13.2	958.0	15.9	958.0	15.9
Gleason_354-U1452B-38F-3 and 4-Spot 20	350	75902	2.2	14.0180	0.8	1.6029	2.3	0.1630	2.2	0.95	973.2	20.1	971.4	14.7	967.1	15.3	967.1	15.3
Gleason_354-U1452B-38F-3 and 4-Spot 98	108	64830	1.4	14.0151	0.7	1.5674	2.9	0.1593	2.9	0.97	953.0	25.3	957.4	18.2	967.5	13.8	967.5	13.8
Gleason_354-U1452B-38F-3 and 4-Spot 185	195	35464	1.5	13.9994	0.8	1.6675	3.0	0.1693	2.9	0.96	1008.3	27.0	996.2	19.1	969.8	17.1	969.8	17.1
Gleason_354-U1452B-38F-3 and 4-Spot 242	487	619992	3.2	13.9709	0.6	1.5634	2.1	0.1584	2.0	0.96	947.9	17.4	955.8	12.7	973.9	11.7	973.9	11.7
Gleason_354-U1452B-38F-3 and 4-Spot 219	365	3748729	3.5	13.9566	0.6	1.4179	3.3	0.1435	3.2	0.99	864.5	26.3	896.5	19.6	976.1	11.2	976.1	11.2
Gleason_354-U1452B-38F-3 and 4-Spot 236	298	179440	2.0	13.9384	0.6	1.5915	2.0	0.1609	1.9	0.95	961.7	17.2	966.9	12.6	978.7	12.2	978.7	12.2
Gleason_354-U1452B-38F-3 and 4-Spot 268	140	14903	1.9	13.9184	0.8	1.6193	2.3	0.1635	2.1	0.94	976.0	19.3	977.7	14.2	981.6	15.3	981.6	15.3
Gleason_354-U1452B-38F-3 and 4-Spot 206	336	893463	2.1	13.8671	0.6	1.6346	1.9	0.1644	1.8	0.95	981.2	16.2	983.6	11.8	989.1	12.3	989.1	12.3
Gleason_354-U1452B-38F-3 and 4-Spot 154	692	547004	18.3	13.8174	0.7	1.6552	2.5	0.1659	2.4	0.96	989.3	22.3	991.5	16.0	996.5	14.4	996.5	14.4
Gleason_354-U1452B-38F-3 and 4-Spot 81	100	29163	3.8	13.7885	0.7	1.6330	2.7	0.1633	2.6	0.96	975.1	23.7	983.0	17.2	1000.7	15.2	1000.7	15.2
Gleason_354-U1452B-38F-3 and 4-Spot 114	174	223090	1.2	13.7512	0.7	1.7091	2.7	0.1705	2.6	0.97	1014.6	24.4	1012.0	17.2	1006.2	13.5	1006.2	13.5
Gleason_354-U1452B-38F-3 and 4-Spot 230	68	20800	1.4	13.7370	0.8	1.6188	2.7	0.1613	2.5	0.95	963.9	22.7	977.5	16.8	1008.3	17.0	1008.3	17.0
Gleason_354-U1452B-38F-3 and 4-Spot 61	476	121477	2.0	13.7123	0.7	1.6577	1.8	0.1649	1.7	0.93	983.7	15.6	992.5	11.7	1011.9	13.9	1011.9	13.9
Gleason_354-U1452B-38F-3 and 4-Spot 132	90	12662	2.2	13.7110	0.8	1.6419	2.6	0.1633	2.5	0.96	974.9	22.7	986.4	16.5	1012.1	15.4	1012.1	15.4
Gleason_354-U1452B-38F-3 and 4-Spot 38	1491	234446	100.2	13.6829	0.7	1.4183	2.1	0.1407	2.0	0.94	848.9	16.0	896.6	12.8	1016.3	14.8	1016.3	14.8
Gleason_354-U1452B-38F-3 and 4-Spot 197	585	27224	3.7	13.6668	0.9	1.5161	2.0	0.1503	1.8	0.90	902.5	14.9	936.9	12.1	1018.7	17.6	1018.7	17.6
Gleason_354-U1452B-38F-3 and 4-Spot 103	80	515138	1.9	13.6453	0.7	1.6458	3.0	0.1629	2.9	0.97	972.7	26.2	987.9	18.9	1021.8	14.8	1021.8	14.8
Gleason_354-U1452B-38F-3 and 4-Spot 182	129	298605	2.1	13.6343	0.7	1.6741	2.9	0.1655	2.9	0.97	987.5	26.3	998.7	18.7	1023.5	13.8	1023.5	13.8
Gleason_354-U1452B-38F-3 and 4-Spot 104	746	310932	12.3	13.6227	0.7	1.7526	2.0	0.1732	1.9	0.94	1029.5	18.2	1028.1	13.1	1025.2	13.7	1025.2	13.7
Gleason_354-U1452B-38F-3 and 4-Spot 16	256	28253	11.6	13.6007	0.7	1.7418	1.8	0.1718	1.7	0.93	1022.1	15.9	1024.1	11.7	1028.5	13.8	1028.5	13.8
Gleason_354-U1452B-38F-3 and 4-Spot 183	344	65602	1.3	13.5480	0.7	1.6102	2.1	0.1582	2.0	0.94	946.8	17.2	974.2	13.0	1036.3	14.5	1036.3	14.5
Gleason_354-U1452B-38F-3 and 4-Spot 306	46	263741	2.2	13.5236	0.9	1.5163	2.7	0.1487	2.5	0.94	893.8	21.0	937.0	16.3	1040.0	17.9	1040.0	17.9
Gleason_354-U1452B-38F-3 and 4-Spot 68	172	123526	1.9	13.4878	0.9	1.7416	2.5	0.1704	2.3	0.93	1014.2	21.6	1024.1	16.1	1045.3	19.1	1045.3	19.1
Gleason_354-U1452B-38F-3 and 4-Spot 251	456	1071872	23.0	13.4681	0.5	1.7902	2.0	0.1749	1.9	0.96	1038.9	18.7	1041.9	13.2	1048.3	10.9	1048.3	10.9
Gleason_354-U1452B-38F-3 and 4-Spot 223	353	38514	2.7	13.4589	0.9	1.4457	2.7	0.1411	2.5	0.94	851.0	20.1	908.1	16.1	1049.7	18.2	1049.7	18.2
Gleason_354-U1452B-38F-3 and 4-Spot 288	344	76938	9.3	13.4050	0.6	1.8403	1.6	0.1789	1.4	0.92	1061.1	14.1	1060.0	10.3	1057.7	12.2	1057.7	12.2
Gleason_354-U1452B-38F-3 and 4-Spot 86	143	303772	1.1	13.3437	1.0	1.6510	2.8	0.1598	2.6	0.94	955.5	23.5	989.9	17.8	1067.0	19.5	1067.0	19.5
Gleason_354-U1452B-38F-3 and 4-Spot 89	344	127727	2.8	13.2652	0.7	1.7805	2.5	0.1713	2.4	0.97	1019.3	23.0	1038.4	16.4	1078.8	13.1	1078.8	13.1
Gleason_354-U1452B-38F-3 and 4-Spot 304	796	138021	2.6	13.2493	0.6	1.8256	2.5	0.1754	2.4	0.97	1042.0	23.0	1054.7	16.2	1081.2	12.3	1081.2	12.3
Gleason_354-U1452B-38F-3 and 4-Spot 87	875	718326	17.4	13.2140	0.6	1.6854	1.7	0.1615	1.6	0.95	965.3	14.7	1003.0	11.1	1086.5	11.4	1086.5	11.4
Gleason_354-U1452B-38F-3 and 4-Spot 298	675	788918	24.8	13.1984	0.6	1.7659	1.9	0.1690	1.8	0.95	1006.8	16.8	1033.0	12.3	1088.9	12.3	1088.9	12.3
Gleason_354-U1452B-38F-3 and 4-Spot 123	19	3814	0.8	13.0833	1.4	2.0490	4.8	0.1944	4.6	0.96	1145.3	48.0	1132.0	32.6	1106.5	27.4	1106.5	27.4
Gleason_354-U1452B-38F-3 and 4-Spot 146	1372	216729	9.3	13.0541	0.6	1.8744	1.9	0.1775	1.8	0.95	1053.1	17.7	1072.1	12.7	1110.9	11.6	1110.9	11.6
Gleason_354-U1452B-38F-3 and 4-Spot 161	105	62203	1.0	13.0512	0.7	1.9219	2.5	0.1819	2.4	0.96	1077.5	23.6	1088.7	16.6	1111.3	14.3	1111.3	14.3
Gleason_354-U1452B-38F-3 and 4-Spot 142	569	86045	6.6	12.9733	0.5	1.7314	1.7	0.1629	1.7	0.95	972.9	15.0	1020.3	11.2	1123.3	10.6	1123.3	10.6
Gleason_354-U1452B-38F-3 and 4-Spot 250	157	21555	0.4	12.9714	0.8	1.9654	2.0	0.1849	1.9	0.92	1093.7	18.8	1103.7	13.6	1123.6	15.4	1123.6	15.4
Gleason_354-U1452B-38F-3 and 4-Spot 71	1920	192601	8.9	12.9140	0.7	1.8548	2.0	0.1737	1.9	0.94	1032.6	18.4	1065.2	13.5	1132.4	13.5	1132.4	13.5
Gleason_354-U1452B-38F-3 and 4-Spot 10	38	7788	1.9	12.8650	0.9	2.0628	3.1	0.1925	2.9	0.96	1134.7	30.4	1136.5	20.9	1140.0	17.3	1140.0	17.3
Gleason_354-U1452B-38F-3 and 4-Spot 64	295	29932	0.6	12.8616	0.6	2.0313	1.9	0.1895	1.8	0.94	1118.6	18.1	1126.1	12.8	1140.5	12.7	1140.5	12.7
Gleason_354-U1452B-38F-3 and 4-Spot 227	1490	467108	9.1	12.8440	0.6	2.0418	1.9	0.1902	1.8	0.95	1122.5	18.5	1129.6	12.9	1143.2	12.1	1143.2	12.1

Gleason_354-U1452B-38F-3 and 4-Spot 108	158	97740	1.6	12.8233	0.7	1.9242	2.4	0.1790	2.3	0.96	1061.3	22.4	1089.5	16.0	1146.4	14.0	1146.4	14.0
Gleason_354-U1452B-38F-3 and 4-Spot 65	131	67960	1.9	12.8170	0.9	2.0116	2.6	0.1870	2.5	0.94	1105.1	25.4	1119.4	18.0	1147.4	17.4	1147.4	17.4
Gleason_354-U1452B-38F-3 and 4-Spot 24	39	4320	2.4	12.8078	1.2	1.7801	3.9	0.1654	3.8	0.95	986.5	34.3	1038.2	25.7	1148.9	24.3	1148.9	24.3
Gleason_354-U1452B-38F-3 and 4-Spot 51	213	27937	2.7	12.7537	0.8	1.6904	2.4	0.1564	2.2	0.95	936.5	19.5	1004.9	15.0	1157.2	15.1	1157.2	15.1
Gleason_354-U1452B-38F-3 and 4-Spot 40	591	94261	2.0	12.7166	0.5	1.9314	1.8	0.1781	1.7	0.96	1056.8	16.5	1092.0	11.9	1163.0	10.3	1163.0	10.3
Gleason_354-U1452B-38F-3 and 4-Spot 275	313	119618	1.2	12.7157	0.8	2.1472	2.4	0.1980	2.3	0.95	1164.7	24.4	1164.1	16.7	1163.1	15.3	1163.1	15.3
Gleason_354-U1452B-38F-3 and 4-Spot 293	500	76779	3.9	12.6831	0.9	2.0744	2.0	0.1908	1.8	0.89	1125.8	18.7	1140.4	13.9	1168.2	18.3	1168.2	18.3
Gleason_354-U1452B-38F-3 and 4-Spot 314	142	98870	1.0	12.6388	0.8	2.0686	2.7	0.1896	2.5	0.95	1119.3	26.1	1138.5	18.3	1175.2	16.5	1175.2	16.5
Gleason_354-U1452B-38F-3 and 4-Spot 78	144	84705	0.7	12.6155	0.7	2.0397	2.3	0.1866	2.2	0.95	1103.1	22.1	1128.9	15.6	1178.8	14.0	1178.8	14.0
Gleason_354-U1452B-38F-3 and 4-Spot 139	265	1558072	1.3	12.5317	0.7	2.2010	2.6	0.2000	2.5	0.96	1175.5	27.1	1181.3	18.4	1192.0	14.7	1192.0	14.7
Gleason_354-U1452B-38F-3 and 4-Spot 149	403	359072	2.3	12.4905	0.7	2.0888	1.9	0.1892	1.8	0.94	1117.2	18.3	1145.1	13.0	1198.5	12.9	1198.5	12.9
Gleason_354-U1452B-38F-3 and 4-Spot 28	922	90429	1.6	12.4327	0.6	2.1808	1.9	0.1966	1.8	0.94	1157.3	18.6	1174.9	13.0	1207.7	12.6	1207.7	12.6
Gleason_354-U1452B-38F-3 and 4-Spot 181	278	64402	1.8	12.3432	0.8	2.3016	2.8	0.2060	2.7	0.95	1207.7	29.3	1212.8	19.8	1221.8	16.6	1221.8	16.6
Gleason_354-U1452B-38F-3 and 4-Spot 111	330	327680	2.9	12.2649	0.6	2.3229	2.1	0.2066	2.0	0.96	1210.9	22.1	1219.3	14.8	1234.4	11.7	1234.4	11.7
Gleason_354-U1452B-38F-3 and 4-Spot 302	447	59397	2.0	12.1604	0.5	1.9868	1.6	0.1752	1.6	0.96	1040.8	15.0	1111.0	11.0	1251.1	9.2	1251.1	9.2
Gleason_354-U1452B-38F-3 and 4-Spot 100	44	6990	0.8	12.1278	1.8	2.1460	4.5	0.1888	4.1	0.91	1114.7	41.9	1163.8	31.0	1256.4	35.4	1256.4	35.4
Gleason_354-U1452B-38F-3 and 4-Spot 297	222	7187	2.1	12.0256	1.5	1.9775	2.7	0.1725	2.2	0.82	1025.7	20.8	1107.9	18.0	1272.9	29.5	1272.9	29.5
Gleason_354-U1452B-38F-3 and 4-Spot 229	98	16412	2.0	11.7291	0.9	2.6626	2.9	0.2265	2.7	0.95	1316.1	32.4	1318.2	21.1	1321.4	17.2	1321.4	17.2
Gleason_354-U1452B-38F-3 and 4-Spot 186	908	358156	2.9	11.7160	0.6	2.4738	1.7	0.2102	1.6	0.94	1229.9	18.0	1264.4	12.3	1323.6	10.9	1323.6	10.9
Gleason_354-U1452B-38F-3 and 4-Spot 109	22	5405	2.7	11.7103	1.2	2.6258	4.8	0.2230	4.7	0.97	1297.8	54.8	1307.9	35.5	1324.5	23.8	1324.5	23.8
Gleason_354-U1452B-38F-3 and 4-Spot 279	69	31311	1.7	11.6553	0.8	2.7715	3.0	0.2343	2.9	0.97	1356.9	35.9	1347.9	22.6	1333.6	14.9	1333.6	14.9
Gleason_354-U1452B-38F-3 and 4-Spot 128	140	24471	1.6	11.6118	0.7	2.7434	2.6	0.2310	2.5	0.96	1339.9	30.8	1340.3	19.7	1340.8	14.1	1340.8	14.1
Gleason_354-U1452B-38F-3 and 4-Spot 282	723	127952	1.7	11.1244	1.1	2.5379	1.9	0.2048	1.6	0.82	1200.9	17.3	1283.0	14.0	1423.2	21.0	1423.2	21.0
Gleason_354-U1452B-38F-3 and 4-Spot 67	144	15208	3.6	11.0917	1.0	2.6696	3.0	0.2148	2.8	0.94	1254.1	32.4	1320.1	22.3	1428.8	19.0	1428.8	19.0
Gleason_354-U1452B-38F-3 and 4-Spot 1	134	53104	3.8	10.7716	1.5	2.8609	3.8	0.2235	3.5	0.91	1300.4	40.8	1371.7	28.5	1484.5	29.0	1484.5	29.0
Gleason_354-U1452B-38F-3 and 4-Spot 151	230	81147	2.0	10.6986	0.7	3.1384	2.7	0.2435	2.6	0.97	1405.0	33.1	1442.2	20.8	1497.4	12.6	1497.4	12.6
Gleason_354-U1452B-38F-3 and 4-Spot 175	95	46308	1.6	10.6015	0.6	3.5012	2.1	0.2692	2.0	0.96	1536.8	27.6	1527.5	16.6	1514.6	11.3	1514.6	11.3
Gleason_354-U1452B-38F-3 and 4-Spot 286	398	81998	2.7	10.4672	0.8	3.1092	1.6	0.2360	1.4	0.88	1366.1	17.8	1435.0	12.6	1538.6	14.5	1538.6	14.5
Gleason_354-U1452B-38F-3 and 4-Spot 195	172	101875	1.8	10.2253	0.7	3.2953	2.2	0.2444	2.1	0.95	1409.4	26.6	1479.9	17.2	1582.5	12.4	1582.5	12.4
Gleason_354-U1452B-38F-3 and 4-Spot 198	2030	361848	35.8	10.1701	0.5	3.8886	1.8	0.2868	1.7	0.96	1625.6	24.2	1611.3	14.2	1592.6	9.6	1592.6	9.6
Gleason_354-U1452B-38F-3 and 4-Spot 153	1303	408833	4.1	10.1584	0.6	3.2829	2.2	0.2419	2.1	0.96	1396.4	25.9	1477.0	16.8	1594.8	11.7	1594.8	11.7
Gleason_354-U1452B-38F-3 and 4-Spot 228	372	55133	1.6	10.1476	0.6	3.3665	1.8	0.2478	1.7	0.94	1427.0	21.3	1496.6	13.8	1596.8	11.1	1596.8	11.1
Gleason_354-U1452B-38F-3 and 4-Spot 144	1809	2777558	14.6	10.1444	0.5	3.1886	1.7	0.2346	1.6	0.95	1358.5	20.0	1454.4	13.2	1597.3	9.6	1597.3	9.6
Gleason_354-U1452B-38F-3 and 4-Spot 83	781	134499	1.9	10.1318	0.6	3.8672	1.9	0.2842	1.9	0.95	1612.3	26.5	1606.9	15.7	1599.7	10.8	1599.7	10.8
Gleason_354-U1452B-38F-3 and 4-Spot 130	331	113345	1.3	10.0792	0.6	3.7155	2.4	0.2716	2.3	0.97	1549.0	31.8	1574.7	19.0	1609.4	10.5	1609.4	10.5
Gleason_354-U1452B-38F-3 and 4-Spot 99	2094	3490732	6.3	10.0365	0.6	3.1816	2.1	0.2316	2.1	0.96	1342.9	24.9	1452.7	16.6	1617.3	11.3	1617.3	11.3
Gleason_354-U1452B-38F-3 and 4-Spot 215	317	3098824	1.9	10.0151	0.5	3.6151	1.8	0.2626	1.7	0.96	1503.1	22.9	1552.9	14.2	1621.3	9.7	1621.3	9.7
Gleason_354-U1452B-38F-3 and 4-Spot 231	249	57673	1.7	9.9654	0.7	3.9737	2.2	0.2872	2.1	0.95	1627.5	29.9	1628.8	17.8	1630.5	13.3	1630.5	13.3
Gleason_354-U1452B-38F-3 and 4-Spot 224	405	266912	2.2	9.9581	0.5	3.8236	1.6	0.2761	1.5	0.94	1571.9	21.1	1597.7	12.9	1631.9	9.9	1631.9	9.9
Gleason_354-U1452B-38F-3 and 4-Spot 133	395	990879	3.1	9.8524	0.6	3.3621	2.4	0.2402	2.4	0.97	1388.0	29.4	1495.6	19.1	1651.7	11.6	1651.7	11.6
Gleason_354-U1452B-38F-3 and 4-Spot 21	100	721850	1.5	9.8464	0.7	3.4263	2.7	0.2447	2.6	0.97	1411.0	33.4	1510.5	21.3	1652.8	12.1	1652.8	12.1
Gleason_354-U1452B-38F-3 and 4-Spot 292	601	215059	3.0	9.6831	0.7	3.5095	2.0	0.2465	1.9	0.95	1420.2	24.5	1529.3	16.1	1683.7	12.0	1683.7	12.0
Gleason_354-U1452B-38F-3 and 4-Spot 262	222	2188612	2.7	9.6112	0.5	4.1427	2.3	0.2888	2.2	0.97	1635.4	32.4	1662.8	18.9	1697.5	10.1	1697.5	10.1
Gleason_354-U1452B-38F-3 and 4-Spot 113	405	404994	2.8	9.3722	0.5	3.8415	2.5	0.2611	2.5	0.98	1495.6	32.8	1601.5	20.3	1743.7	9.7	1743.7	9.7
Gleason_354-U1452B-38F-3 and 4-Spot 295	100	87581	3.0	9.2444	0.7	4.8356	3.3	0.3242	3.2	0.98	1810.3	50.9	1791.1	27.7	1768.9	12.3	1768.9	12.3
Gleason_354-U1452B-38F-3 and 4-Spot 150	95	16396	2.6	9.2440	0.8	4.5655	2.9	0.3061	2.7	0.96	1721.4	41.3	1743.0	23.7	1768.9	14.7	1768.9	14.7
Gleason_354-U1452B-38F-3 and 4-Spot 105	410	176804	10.7	9.1024	0.6	4.7583	2.1	0.3141	2.0	0.96	1761.0	31.4	1777.6	17.8	1797.1	10.9	1797.1	10.9
Gleason_354-U1452B-38F-3 and 4-Spot 269	457	1740389	6.8	9.0868	0.7	4.4279	2.2	0.2918	2.1	0.95	1650.6	31.0	1717.6	18.5	1800.2	12.2	1800.2	12.2
Gleason_354-U1452B-38F-3 and 4-Spot 165	376	165595	9.9	9.0542	0.6	4.7734	2.1	0.3135	2.1	0.96	1757.7	31.6	1780.2	18.0	1806.8	11.1	1806.8	11.1
Gleason_354-U1452B-38F-3 and 4-Spot 145	2646	230681	7.8	9.0168	0.6	4.7690	1.7	0.3119	1.6	0.93	1749.9	23.9	1779.5	14.1	1814.3	11.3	1814.3	11.3
Gleason_354-U1452B-38F-3 and 4-Spot 260	513	65761	5.9	8.9585	0.7	4.1192	2.9	0.2676	2.9	0.97	1528.8	38.9	1658.1	24.0	1826.0	12.3	1826.0	12.3
Gleason_354-U1452B-38F-3 and 4-Spot 204	950	26018	2.1	8.9570	0.5	4.4371	2.0	0.2882	2.0	0.96	1632.8	28.3	1719.3	16.9	1826.3	9.9	1826.3	9.9
Gleason_354-U1452B-38F-3 and 4-Spot 207	988	193031	8.6	8.8848	0.6	4.8645	1.9	0.3135	1.8	0.94	1757.7	27.4	1796.1	15.9	1841.0	11.3	1841.0	11.3
Gleason_354-U1452B-38F-3 and 4-Spot 247	462	434531	1.7	8.8832	0.6	4.9671	2.1	0.3200	2.0	0.95	1789.8	31.1	1813.7	17.6	1841.3	11.3	1841.3	11.3
Gleason_354-U1452B-38F-3 and 4-Spot 140	1348	1149261	4.6	8.8108	0.6	4.5200	2.1	0.2888	2.1	0.96	1635.7	29.6	1734.7	17.7	1856.1	10.3	1856.1	10.3
Gleason_354-U1452B-38F-3 and 4-Spot 126	89	1042523	1.6	8.8028	0.6	5.2386	2.9	0.3345	2.9	0.98	1859.9	46.5	1858.9	25.1	1857.8	10.7	1857.8	10.7
Gleason_354-U1452B-38F-3 and 4-Spot 63	399	365298	4.0	8.7213	0.7	5.0372	2.5	0.3186	2.4	0.96	1783.0	37.1	1825.6	21.0	1874.6	12.3	1874.6	12.3
Gleason_354-U1452B-38F-3 and 4-Spot 171	244	361008	4.8	8.6700	0.5	5.3755	2.0	0.3380	2.0	0.97	1877.1	32.3	1881.0	17.5	1885.2	9.1	1885.2	9.1
Gleason_354-U1452B-38F-3 and 4-Spot 285	402	173288	9.3	8.6408	0.7	5.2571	2.0	0.3295	1.9	0.94	1835.8	30.8	1861.9	17.4	1891.3	12.1	1891.3	12.1
Gleason_354-U1452B-38F-3 and 4-Spot 202	377	31104	4.4	8.5000	0.8	5.0619	3.0	0.3121	2.9	0.97	1750.8	45.1	1829.7	25.8	1920.8	13.7	1920.8	13.7
Gleason_354-U1452B-38F-3 and 4-Spot 312	419	200572	5.6	8.4074	0.5	5.6017	1.4	0.3416	1.3	0.94	1894.2	20.8	1916.4	11.7	1940.4	8.6	1940.4	8.6
Gleason_354-U1452B-38F-3 and 4-Spot 270	169	91411	1.7	8.3819	0.6	5.8563	2.2	0.3560	2.1	0.96	1963.2	35.3	1954.8	18.7	1945.8	10.2	1945.8	10.2
Gleason_354-U1452B-38F-3 and 4-Spot 168	112	219720	1.8	7.8863	0.6	6.3877	1.7	0.3654	1.6	0.94	2007.5	27.4	2030.6	14.9	2054.1	10.5	2054.1	10.5
Gleason_354-U1452B-38F-3 and 4-Spot 73	163	65324	1.4	6.6248	0.7	7.4045	2.1	0.3558	2.0	0.95	1962.1	33.0	2161.5	18.4	2356.7	11.4	2356.7	11.4
Gleason_354-U1452B-38F-3 and 4-Spot 267	225	309569	2.9	6.5679	0.5	8.6611	2.4	0.4126	2.4	0.98	2226.7	44.7	2303.0	22.1	2371.4	8.5	2371.4	8.5
Gleason_354-U1452B-38F-3 and 4-Spot 203	363	475532	2.1	6.3409	0.8	7.9534	2.0	0.3658	1.9	0.92	2009.4	32.1	2225.8	18.2	2431.2	13.3	2431.2	13.3
Gleason_354-U1452B-38F-3 and 4-Spot 33	106	24040	1.0	6.2159	0.6	9.1803	2.8	0.4139	2.8	0.98	2232.6	52.4	2356.1	26.0	2464.9	9.5	2464.9	9.5
Gleason_354-U1452B-38F-3 and 4-Spot 287	357	1471199	2.1	6.1044	0.8	9.8651	2.2	0.4368	2.1	0.94	2336.1	41.4	2422.3	20.7	2495.4	12.7	2495.4	12.7
Gleason_354-U1452B-38F-3 and 4-Spot 156	98	79243	1.6	6.0785	0.7	10.7639	2.8	0.4745	2.7	0.97	2503.4	55.7	2503.0	25.7	2502.6	11.4	2502.6	11.4
Gleason_354-U1452B-38F-3 and 4-Spot 166	1837	638625	1.4	6.0021	0.6	10.5402	1.6	0.4588	1.5	0.93	2434.4	30.3	2483.5	14.9	2523.9	9.7	2523.9	9.7
Gleason_354-U1452B-38F-3 and 4-Spot 164	753	1419689	6.9	5.8157	0.6	11.8056	2.0	0.4980	1.9	0.95	2605.0	40.3	2589.1	18.5	2576.7	10.2	2576.7	10.2
Gleason_354-U1452B-38F-3 and 4-Spot 9	689	4320184	1.5	5.7514	0.7	11.1188	2.1	0.4638	2.0	0.94	2456.3	41.1	2533.1	20.0	2595.2	12.4	2595.2	12.4
Gleason_354-U1452B-38F-3 and 4-Spot 264	201	633617	1.1	5.5438	0.5	11.7672	2.1	0.4731	2.1	0.97	2497.3	43.2	2586.1	20.1	2656.4	8.4	2656.4	8.4
Gleason_354-U1452B-38F-3 and 4-Spot 125	53	63545	3.4	5.1252	0.6	13.6896	3.1	0.5089	3.1	0.98	2651.8	66.7	2728.5	29.6	2785.7	9.6	2785.7	9.6
Gleason_354-U1452B-38F-3 and 4-Spot 257	133	314532	2.0	5.1010	0.7	13.2395	2.7	0.4898	2.6	0.97	2569.8	56.2	2696.9	25.9	2793.5	11.7	2793.5	11.7
Gleason_354-U1452B-38F-3 and 4-Spot 136	468	211783	10.0	4.8152	0.5	14.7785	1.6	0.5161	1.5	0.95	2682.7	34.0	2801.1	15.5	2887.5	8.3	2887.5	8.3
Gleason_354-U1452B-38F-3 and 4-Spot 77	105	125162	1.3	3.8577	0.6	18.7696	3.1	0.5252	3.0	0.98	2721.0	67.6	3030.0	30.0	3242.0	10.1	3242.0	10.1

Table ______. U-Pb geochronologic analyses.																		
#8 U1453_32F						Isotope ratios						Apparent ages (Ma)						
Early Pleistocene 1.3 Ma																		
Analysis	U	206Pb	U/Th	206Pb*	±	207Pb*	±	206Pb*	±	error	206Pb*	±	207Pb*	±	206Pb*	±	Best age	±
	(ppm)	204Pb		207Pb*	(%)	235U*	(%)	238U	(%)	corr.	238U*	(Ma)	235U	(Ma)	207Pb*	(Ma)	(Ma)	(Ma)
354-U1453A-32F-2W-0-92-Spot 151	5341	16980	69.4	21.4240	1.0	0.0179	2.3	0.0028	2.0	0.89	17.9	0.4	18.0	0.4	32.7	24.5	17.9	0.4
354-U1453A-32F-2W-0-92-Spot 185	6603	18272	61.0	21.1635	1.2	0.0193	1.9	0.0030	1.5	0.79	19.1	0.3	19.4	0.4	61.9	28.0	19.1	0.3
354-U1453A-32F-2W-0-92-Spot 164	885	5141	1.7	20.4156	1.8	0.0272	2.4	0.0040	1.6	0.68	25.9	0.4	27.2	0.6	147.0	41.1	25.9	0.4
354-U1453A-32F-2W-0-92-Spot 55	654	5300	1.4	21.1036	1.8	0.0400	2.4	0.0061	1.5	0.65	39.4	0.6	39.9	0.9	68.7	42.9	39.4	0.6
354-U1453A-32F-2W-0-92-Spot 105	287	2112	2.1	23.7501	3.2	0.0420	4.0	0.0072	2.3	0.59	46.4	1.1	41.7	1.6	220.2	81.6	46.4	1.1
354-U1453A-32F-2W-0-92-Spot 147	1129	10154	2.1	21.0647	1.7	0.0493	2.6	0.0075	2.0	0.77	48.4	1.0	48.9	1.2	73.1	40.0	48.4	1.0
354-U1453A-32F-2W-0-92-Spot 313	750	6069	120.0	17.8003	1.4	0.0611	3.1	0.0079	2.7	0.89	50.7	1.4	60.2	1.8	459.4	31.0	50.7	1.4
354-U1453A-32F-2W-0-92-Spot 276	848	6177	1.1	21.2463	1.5	0.0522	2.6	0.0080	2.1	0.82	51.7	1.1	51.7	1.3	52.6	35.0	51.7	1.1
354-U1453A-32F-2W-0-92-Spot 100	341	8358	0.9	21.9561	2.1	0.0535	2.8	0.0085	2.0	0.69	54.7	1.1	52.9	1.5	26.4	49.7	54.7	1.1
354-U1453A-32F-2W-0-92-Spot 150	73	485	2.5	40.2303	64.3	0.0296	64.3	0.0086	3.4	0.05	55.4	1.9	29.6	18.8	1779.2	457.5	55.4	1.9
354-U1453A-32F-2W-0-92-Spot 126	494	9560	4.2	20.8565	1.9	0.0575	2.7	0.0087	1.9	0.70	55.8	1.0	56.8	1.5	96.7	45.6	55.8	1.0
354-U1453A-32F-2W-0-92-Spot 183	78	4580	2.4	21.9936	4.1	0.0564	5.2	0.0090	3.3	0.63	57.7	1.9	55.7	2.8	30.5	99.0	57.7	1.9
354-U1453A-32F-2W-0-92-Spot 65	330	64815	2.1	19.8551	1.5	0.0670	2.6	0.0096	2.1	0.82	61.9	1.3	65.8	1.6	211.8	34.4	61.9	1.3
354-U1453A-32F-2W-0-92-Spot 10	1382	17300	4.3	21.1560	0.9	0.0634	2.0	0.0097	1.8	0.89	62.4	1.1	62.4	1.2	62.8	22.2	62.4	1.1
354-U1453A-32F-2W-0-92-Spot 33	326	3055	2.4	23.1899	2.1	0.0579	3.1	0.0097	2.4	0.75	62.5	1.5	57.2	1.7	160.5	51.4	62.5	1.5
354-U1453A-32F-2W-0-92-Spot 229	104	2586	0.7	21.5382	3.9	0.0645	4.7	0.0101	2.7	0.57	64.6	1.7	63.4	2.9	20.0	93.5	64.6	1.7
354-U1453A-32F-2W-0-92-Spot 35	619	3806	2.2	21.9981	1.2	0.0731	1.8	0.0117	1.4	0.78	74.8	1.1	71.7	1.3	31.0	27.9	74.8	1.1
354-U1453A-32F-2W-0-92-Spot 246	112	3153	1.2	21.5671	3.4	0.0767	4.5	0.0120	3.0	0.66	76.9	2.3	75.0	3.2	16.7	80.7	76.9	2.3
354-U1453A-32F-2W-0-92-Spot 86	21	274	1.6	-108.8187	7.8	-0.0155	9.8	0.0122	5.9	0.60	78.3	4.6	15.8	1.6	0.0	0.0	78.3	4.6
354-U1453A-32F-2W-0-92-Spot 63	124	1498	1.7	25.9636	13.2	0.0698	13.4	0.0131	2.4	0.18	84.1	2.0	68.5	8.9	449.6	348.5	84.1	2.0
354-U1453A-32F-2W-0-92-Spot 204	170	2157	8.7	22.7156	2.4	0.0812	3.7	0.0134	2.8	0.76	85.7	2.4	79.3	2.8	109.4	58.6	85.7	2.4
354-U1453A-32F-2W-0-92-Spot 167	64	1508	1.4	22.4039	8.3	0.0826	8.7	0.0134	2.6	0.30	85.9	2.2	80.6	6.7	75.5	203.6	85.9	2.2
354-U1453A-32F-2W-0-92-Spot 296	60	5272	0.8	20.8578	3.3	0.0997	4.7	0.0151	3.4	0.72	96.5	3.2	96.5	4.3	96.5	77.8	96.5	3.2
354-U1453A-32F-2W-0-92-Spot 80	362	3471	1.6	21.2972	1.8	0.0987	3.7	0.0152	3.3	0.87	97.6	3.2	95.6	3.4	46.9	43.8	97.6	3.2
354-U1453A-32F-2W-0-92-Spot 75	1768	71207	1.4	20.3519	0.8	0.1158	2.1	0.0171	2.0	0.93	109.2	2.1	111.2	2.2	154.3	17.9	109.2	2.1
354-U1453A-32F-2W-0-92-Spot 128	4649	176355	4.3	20.4560	0.7	0.1155	2.1	0.0171	2.0	0.95	109.5	2.2	110.9	2.2	142.3	15.9	109.5	2.2
354-U1453A-32F-2W-0-92-Spot 149	3264	28188	1.4	20.4716	0.8	0.1156	1.8	0.0172	1.6	0.91	109.7	1.8	111.1	1.9	140.5	17.9	109.7	1.8
354-U1453A-32F-2W-0-92-Spot 161	1285	33709	1.8	20.8317	0.9	0.1150	1.9	0.0174	1.7	0.89	111.0	1.9	110.5	2.0	99.5	20.7	111.0	1.9
354-U1453A-32F-2W-0-92-Spot 119	352	8033	0.8	20.7584	1.5	0.1163	2.2	0.0175	1.6	0.74	111.9	1.8	111.7	2.4	107.8	35.4	111.9	1.8
354-U1453A-32F-2W-0-92-Spot 208	595	22403	0.9	20.5699	1.0	0.1222	2.2	0.0182	2.0	0.90	116.4	2.3	117.0	2.4	129.3	22.4	116.4	2.3
354-U1453A-32F-2W-0-92-Spot 93	999	5495	3.6	20.1098	1.6	0.1309	2.4	0.0191	1.8	0.76	121.9	2.2	124.9	2.9	182.2	36.8	121.9	2.2
354-U1453A-32F-2W-0-92-Spot 19	2131	43372	301.6	20.3408	0.9	0.1319	3.3	0.0195	3.2	0.96	124.2	3.9	125.8	3.9	155.6	21.8	124.2	3.9
354-U1453A-32F-2W-0-92-Spot 194	1603	59052	7.4	20.0809	0.7	0.1363	2.1	0.0198	2.0	0.94	126.7	2.5	129.7	2.5	185.6	16.6	126.7	2.5
354-U1453A-32F-2W-0-92-Spot 196	1674	65624	2.9	20.2746	0.5	0.1386	1.7	0.0204	1.6	0.95	130.1	2.1	131.8	2.1	163.2	12.7	130.1	2.1
354-U1453A-32F-2W-0-92-Spot 214	493	35572	3.1	19.7698	1.0	0.2286	2.2	0.0328	2.0	0.89	207.9	4.1	209.0	4.2	221.9	23.2	207.9	4.1
354-U1453A-32F-2W-0-92-Spot 242	1305	63457	18.3	17.5005	0.8	0.3438	1.6	0.0436	1.4	0.88	275.4	3.8	300.1	4.1	497.0	17.0	275.4	3.8
354-U1453A-32F-2W-0-92-Spot 174	567	311377	2.1	15.3784	1.2	0.4244	2.7	0.0473	2.4	0.89	298.2	7.0	359.2	8.1	775.2	25.7	298.2	7.0
354-U1453A-32F-2W-0-92-Spot 72	465	337284	3.9	17.5172	0.7	0.4361	1.9	0.0554	1.7	0.92	347.6	5.9	367.5	5.8	494.9	16.4	347.6	5.9
354-U1453A-32F-2W-0-92-Spot 237	8563	1974035	28.8	17.6711	0.6	0.4765	2.0	0.0611	1.9	0.95	382.1	6.9	395.6	6.4	475.6	13.7	382.1	6.9
354-U1453A-32F-2W-0-92-Spot 110	1780	212472	3.7	17.3901	0.6	0.5311	2.6	0.0670	2.5	0.97	418.0	10.3	432.6	9.2	510.9	13.7	418.0	10.3
354-U1453A-32F-2W-0-92-Spot 193	4687	506034	10.1	17.6644	0.5	0.5290	1.5	0.0678	1.5	0.95	422.7	6.0	431.1	5.4	476.4	11.1	422.7	6.0
354-U1453A-32F-2W-0-92-Spot 27	1856	464395	8.9	17.5501	0.8	0.5617	1.9	0.0715	1.8	0.92	445.2	7.7	452.6	7.1	490.8	16.6	445.2	7.7
354-U1453A-32F-2W-0-92-Spot 179	1538	157653	15.8	17.5502	0.6	0.5759	1.7	0.0733	1.6	0.93	456.1	7.0	461.9	6.3	490.8	13.4	456.1	7.0
354-U1453A-32F-2W-0-92-Spot 50	1585	1717676	1.3	17.6769	0.7	0.5720	1.6	0.0733	1.5	0.91	456.2	6.4	459.3	5.9	474.9	14.5	456.2	6.4
354-U1453A-32F-2W-0-92-Spot 257	2153	311059	3.2	17.1016	0.6	0.5994	1.8	0.0743	1.7	0.94	462.3	7.7	476.9	7.0	547.5	14.0	462.3	7.7
354-U1453A-32F-2W-0-92-Spot 120	355	31696	0.8	17.4809	0.8	0.5921	2.1	0.0751	1.9	0.92	466.6	8.5	472.2	7.8	499.4	17.6	466.6	8.5
354-U1453A-32F-2W-0-92-Spot 215	2279	73409	2.5	17.1837	0.5	0.6066	1.8	0.0756	1.8	0.96	469.8	8.0	481.4	7.0	537.1	11.4	469.8	8.0
354-U1453A-32F-2W-0-92-Spot 153	698	58306	2.4	17.5443	0.7	0.5950	2.2	0.0757	2.0	0.94	470.5	9.3	474.1	8.2	491.5	16.2	470.5	9.3
354-U1453A-32F-2W-0-92-Spot 133	978	82617	3.6	17.5590	0.7	0.5947	1.7	0.0757	1.6	0.92	470.6	7.3	473.9	6.6	489.7	14.8	470.6	7.3
354-U1453A-32F-2W-0-92-Spot 192	351	134273	1.6	17.2393	0.9	0.6135	2.0	0.0767	1.8	0.90	476.5	8.1	485.8	7.6	530.0	19.0	476.5	8.1
354-U1453A-32F-2W-0-92-Spot 84	545	102545	3.6	17.3576	0.7	0.6125	1.8	0.0771	1.7	0.93	478.8	7.8	485.1	7.1	515.0	14.8	478.8	7.8
354-U1453A-32F-2W-0-92-Spot 287	1645	375435	2.6	17.3959	0.5	0.6132	2.1	0.0774	2.0	0.97	480.3	9.4	485.5	8.1	510.2	11.5	480.3	9.4
354-U1453A-32F-2W-0-92-Spot 112	939	68106	1.7	17.3793	0.6	0.6173	2.1	0.0778	2.0	0.96	483.0	9.4	488.1	8.1	512.3	12.4	483.0	9.4
354-U1453A-32F-2W-0-92-Spot 156	816	49216	1.0	17.5325	0.7	0.6122	1.6	0.0778	1.4	0.90	483.3	6.6	485.0	6.1	493.0	15.3	483.3	6.6
354-U1453A-32F-2W-0-92-Spot 218	384	67208	1.0	17.2863	0.7	0.6217	2.0	0.0779	1.9	0.93	483.8	8.7	490.9	7.8	524.0	15.9	483.8	8.7
354-U1453A-32F-2W-0-92-Spot 271	1331	175545	1.3	17.4556	0.6	0.6157	1.6	0.0779	1.5	0.92	483.9	6.8	487.2	6.1	502.7	13.9	483.9	6.8
354-U1453A-32F-2W-0-92-Spot 77	375	72437	6.1	17.5916	0.8	0.6113	2.4	0.0780	2.2	0.93	484.2	10.2	484.4	9.1	485.5	18.6	484.2	10.2
354-U1453A-32F-2W-0-92-Spot 270	546	4676231	1.7	17.2300	0.7	0.6290	1.9	0.0786	1.7	0.93	487.8	8.2	495.5	7.4	531.2	15.0	487.8	8.2
354-U1453A-32F-2W-0-92-Spot 46	891	51961	3.9	17.7297	0.5	0.6140	1.5	0.0790	1.4	0.94	489.9	6.8	486.1	6.0	468.3	12.0	489.9	6.8
354-U1453A-32F-2W-0-92-Spot 170	499	364299	2.3	17.0268	0.9	0.6398	1.7	0.0790	1.4	0.84	490.2	6.8	502.2	6.8	557.1	20.5	490.2	6.8
354-U1453A-32F-2W-0-92-Spot 269	412	32778	1.6	17.3832	0.7	0.6270	1.7	0.0791	1.6	0.92	490.5	7.5	494.3	6.8	511.8	14.7	490.5	7.5
354-U1453A-32F-2W-0-92-Spot 92	391	41545	1.7	17.2957	0.8	0.6327	2.3	0.0794	2.2	0.94	492.3	10.2	497.8	9.1	522.8	17.8	492.3	10.2
354-U1453A-32F-2W-0-92-Spot 89	546	30380	2.6	17.4336	0.8	0.6289	2.2	0.0795	2.0	0.93	493.2	9.6	495.4	8.5	505.4	17.0	493.2	9.6
354-U1453A-32F-2W-0-92-Spot 176	718	1312881	1.6	17.3048	0.7	0.6337	2.1	0.0795	1.9	0.94	493.3	9.2	498.4	8.1	521.7	15.7	493.3	9.2
354-U1453A-32F-2W-0-92-Spot 51	189	12496	2.6	17.4205	1.1	0.6300	2.1	0.0796	1.8	0.85	493.7	8.4	496.1	8.1	507.1	23.9	493.7	8.4
354-U1453A-32F-2W-0-92-Spot 23	314	44854	2.2	17.4587	0.9	0.6304	2.2	0.0798	2.0	0.92	495.1	9.5	496.4	8.6	502.3	19.3	495.1	9.5
354-U1453A-32F-2W-0-92-Spot 41	302	44429	1.1	17.2142	0.8	0.6407	2.1	0.0800	1.9	0.92	496.1	9.3	502.7	8.4	533.2	17.8	496.1	9.3
354-U1453A-32F-2W-0-92-Spot 205	195	39434	0.8	17.0349	0.9	0.6494	2.1	0.0802	1.8	0.90	497.5	8.8	508.1	8.2	556.1	19.9	497.5	8.8
354-U1453A-32F-2W-0-92-Spot 12	824	115769	2.6	17.1082	0.7	0.6498	2.0	0.0806	1.9	0.94	499.9	9.0	508.4	8.0	546.7	15.5	499.9	9.0
354-U1453A-32F-2W-0-92-Spot 288	304	19291	1.8	17.3489	1.0	0.6410	2.4	0.0807	2.2	0.91	500.0	10.7	502.9	9.7	516.1	22.3	500.0	10.7
354-U1453A-32F-2W-0-92-Spot 189	285	61062	3.1	17.1072	0.7	0.6508	2.0	0.0807	1.8	0.93	500.6	8.8	509.0	7.9	546.8	16.2	500.6	8.8
354-U1453A-32F-2W-0-92-Spot 307	414	287147	1.7	17.2225	0.6	0.6522	2.0	0.0815	1.9	0.95	504.9	9.2	509.8	8.0	532.2	13.7	504.9	9.2
354-U1453A-32F-2W-0-92-Spot 113	201	16447	0.9	17.3526	0.8	0.6474	2.2	0.0815	2.0	0.93	504.9	9.7	506.9	8.6	515.6	17.9	504.9	9.7
354-U1453A-32F-2W-0-92-Spot 173	311	144948	2.0	17.0534	0.8	0.6623	2.2	0.0819	2.0	0.93	507.5	9.8	516.0	8.8	553.7	17.9	507.5	9.8
354-U1453A-32F-2W-0-92-Spot 292	439	30477	1.2	17.4908	0.7	0.6508	2.0	0.0826	1.9	0.93	511.3	9.2	508.9	8.0	498.2	16.2	511.3	9.2
354-U1453A-32F-2W-0-92-Spot 282	1086	72784	10.1	17.3089	0.5	0.6619	1.4	0.0831	1.3	0.93	514.6	6.7	515.8	5.9	521.2	11.7	514.6	6.7
354-U1453A-32F-2W-0-92-Spot 308	268	28183	0.3	17.2495	0.8	0.6684	2.0	0.0836	1.8	0.92	517.7	9.0	519.8	8.0	528.7	16.8	517.7	9.0
354-U1453A-32F-2W-0-92-Spot 209	557	46031	1.1	17.2947	1.0	0.6732	1.9	0.0844	1.6	0.86	522.6	8.3	522.7	7.9	523.0	21.7	522.6	8.3
354-U1453A-32F-2W-0-92-Spot 202	113	8668	0.5	17.3301	1.5	0.6741	2.5	0.0847	2.0	0.81	524.3	10.3	523.2	10.3	518.5	32.3	524.3	10.3

354-U1453A-32F-2W-0-92-Spot 273	289	27425	1.4	17.1514	0.9	0.6889	2.0	0.0857	1.8	0.89	530.0	9.1	532.1	8.3	541.2	19.9	530.0	9.1
354-U1453A-32F-2W-0-92-Spot 187	248	40196	1.0	16.8995	0.9	0.7081	2.3	0.0868	2.1	0.92	536.5	10.9	543.6	9.8	573.5	20.3	536.5	10.9
354-U1453A-32F-2W-0-92-Spot 250	406	1602048	17.0	16.6678	0.6	0.7596	1.7	0.0918	1.6	0.93	566.3	8.5	573.8	7.4	603.4	13.6	566.3	8.5
354-U1453A-32F-2W-0-92-Spot 306	37	3325	7.6	17.1382	2.3	0.7421	4.5	0.0922	3.9	0.87	568.8	21.3	563.6	19.5	542.9	49.2	568.8	21.3
354-U1453A-32F-2W-0-92-Spot 101	480	101244	8.7	16.5631	0.8	0.7704	1.7	0.0925	1.5	0.88	570.5	8.4	580.0	7.6	617.0	17.4	570.5	8.4
354-U1453A-32F-2W-0-92-Spot 16	224	926152	1.6	16.8129	0.8	0.7642	1.9	0.0932	1.8	0.92	574.4	9.8	576.4	8.6	584.7	16.4	574.4	9.8
354-U1453A-32F-2W-0-92-Spot 227	455	105467	4.0	15.7800	0.6	0.8177	1.9	0.0936	1.8	0.95	576.7	9.8	606.7	8.5	720.7	12.4	576.7	9.8
354-U1453A-32F-2W-0-92-Spot 199	453	27857	0.8	16.7400	0.8	0.7741	2.0	0.0940	1.9	0.91	579.1	10.3	582.1	9.0	594.1	17.9	579.1	10.3
354-U1453A-32F-2W-0-92-Spot 56	817	94514	4.6	16.1622	0.8	0.8099	1.9	0.0949	1.8	0.92	584.7	10.0	602.4	8.8	669.7	16.4	584.7	10.0
354-U1453A-32F-2W-0-92-Spot 302	110	11171	2.3	16.6829	0.9	0.7956	2.3	0.0963	2.2	0.93	592.5	12.3	594.4	10.5	601.4	18.6	592.5	12.3
354-U1453A-32F-2W-0-92-Spot 148	173	27175	2.4	16.2751	0.7	0.8810	2.7	0.1040	2.6	0.96	637.8	16.0	641.5	13.1	654.8	15.9	637.8	16.0
354-U1453A-32F-2W-0-92-Spot 9	797	56888	4.3	16.1494	0.7	0.9123	3.4	0.1069	3.3	0.98	654.4	20.7	658.3	16.4	671.4	14.0	654.4	20.7
354-U1453A-32F-2W-0-92-Spot 195	82	10670	2.7	16.1154	1.3	0.9236	3.4	0.1080	3.1	0.92	660.9	19.5	664.3	16.5	675.9	28.1	660.9	19.5
354-U1453A-32F-2W-0-92-Spot 268	207	26529	2.2	16.0338	1.1	0.9464	1.9	0.1101	1.6	0.83	673.0	10.2	676.2	9.4	686.8	22.5	673.0	10.2
354-U1453A-32F-2W-0-92-Spot 138	999	485581	4.9	15.2874	0.5	1.0188	1.8	0.1130	1.7	0.96	689.9	11.3	713.3	9.2	787.6	10.8	689.9	11.3
354-U1453A-32F-2W-0-92-Spot 298	362	40196	2.6	15.0408	0.7	1.0793	2.0	0.1177	1.9	0.94	717.5	13.0	743.3	10.7	821.7	14.1	717.5	13.0
354-U1453A-32F-2W-0-92-Spot 236	1360	196831	1.8	14.7011	0.6	1.1346	1.6	0.1210	1.5	0.93	736.2	10.5	769.9	8.7	869.2	12.0	736.2	10.5
354-U1453A-32F-2W-0-92-Spot 146	642	72911	6.4	15.3066	0.6	1.1165	2.0	0.1239	1.9	0.95	753.2	13.4	761.3	10.6	785.0	12.6	753.2	13.4
354-U1453A-32F-2W-0-92-Spot 233	796	413729	1.0	14.8820	0.7	1.1707	2.2	0.1264	2.1	0.94	767.1	15.3	787.0	12.3	843.8	15.4	767.1	15.3
354-U1453A-32F-2W-0-92-Spot 82	553	111382	1.1	15.1537	0.7	1.1509	2.2	0.1265	2.1	0.94	767.8	14.9	777.7	11.8	806.1	15.1	767.8	14.9
354-U1453A-32F-2W-0-92-Spot 300	341	275030	2.2	15.0298	0.7	1.1696	3.3	0.1275	3.2	0.98	773.6	23.3	786.5	17.8	823.3	13.9	773.6	23.3
354-U1453A-32F-2W-0-92-Spot 154	1947	1104085	1.4	14.9492	0.5	1.1782	1.8	0.1277	1.7	0.96	775.0	12.4	790.5	9.8	834.4	11.0	775.0	12.4
354-U1453A-32F-2W-0-92-Spot 61	678	39620	0.4	14.6921	0.5	1.2108	1.5	0.1290	1.4	0.93	782.2	10.3	805.5	8.3	870.5	11.4	782.2	10.3
354-U1453A-32F-2W-0-92-Spot 122	744	168005	4.4	14.9520	0.6	1.2223	1.6	0.1326	1.5	0.93	802.4	11.5	810.8	9.2	834.0	12.8	802.4	11.5
354-U1453A-32F-2W-0-92-Spot 213	341	64107	3.1	14.7744	0.7	1.2377	2.0	0.1326	1.9	0.94	802.8	14.4	817.8	11.4	858.9	14.8	802.8	14.4
354-U1453A-32F-2W-0-92-Spot 21	366	55239	1.7	15.2329	0.6	1.2008	1.7	0.1327	1.6	0.94	803.1	12.2	801.0	9.5	795.1	11.9	803.1	12.2
354-U1453A-32F-2W-0-92-Spot 241	399	229766	2.4	14.7619	0.7	1.2405	2.3	0.1328	2.1	0.95	803.9	16.2	819.1	12.7	860.6	15.1	803.9	16.2
354-U1453A-32F-2W-0-92-Spot 295	363	32397	2.1	15.1418	0.7	1.2163	1.8	0.1336	1.7	0.92	808.2	12.9	808.1	10.3	807.7	14.9	808.2	12.9
354-U1453A-32F-2W-0-92-Spot 294	593	111544	15.7	14.8195	0.7	1.2451	1.9	0.1338	1.8	0.94	809.6	13.7	821.2	10.8	852.6	13.7	809.6	13.7
354-U1453A-32F-2W-0-92-Spot 305	124	139193	0.4	14.6723	0.8	1.2580	2.5	0.1339	2.3	0.95	809.9	17.9	827.0	14.0	873.3	16.3	809.9	17.9
354-U1453A-32F-2W-0-92-Spot 247	276	22161	2.9	14.5908	0.7	1.2656	1.6	0.1339	1.4	0.88	810.3	10.7	830.4	9.0	884.8	15.4	810.3	10.7
354-U1453A-32F-2W-0-92-Spot 180	168	503998	2.5	14.7047	0.8	1.2651	2.1	0.1349	2.0	0.92	815.9	15.1	830.2	12.1	868.7	17.5	815.9	15.1
354-U1453A-32F-2W-0-92-Spot 102	128	222657	0.9	14.0694	0.9	1.3338	3.3	0.1361	3.1	0.96	822.6	24.1	860.5	18.9	959.6	18.6	822.6	24.1
354-U1453A-32F-2W-0-92-Spot 111	140	14910	0.7	15.1345	0.8	1.2440	2.6	0.1365	2.4	0.95	825.1	18.9	820.7	14.5	808.7	17.3	825.1	18.9
354-U1453A-32F-2W-0-92-Spot 49	97	12750	0.9	15.1175	1.1	1.2471	2.7	0.1367	2.5	0.92	826.2	19.3	822.1	15.3	811.1	22.4	826.2	19.3
354-U1453A-32F-2W-0-92-Spot 141	323	358790	1.3	14.8830	0.7	1.2711	1.9	0.1372	1.8	0.93	828.8	13.8	832.9	10.8	843.7	14.3	828.8	13.8
354-U1453A-32F-2W-0-92-Spot 272	271	233844	1.5	14.6576	0.7	1.2918	2.3	0.1373	2.2	0.95	829.5	16.9	842.1	13.1	875.4	15.1	829.5	16.9
354-U1453A-32F-2W-0-92-Spot 280	1720	122483	2.0	14.6087	0.6	1.2988	1.4	0.1376	1.3	0.92	831.1	10.4	845.2	8.3	882.3	11.6	831.1	10.4
354-U1453A-32F-2W-0-92-Spot 24	619	121307	10.8	15.1352	0.7	1.2580	2.0	0.1381	1.9	0.93	833.8	14.6	827.0	11.4	808.6	15.7	833.8	14.6
354-U1453A-32F-2W-0-92-Spot 177	116	125430	1.1	14.5456	1.0	1.3223	2.8	0.1395	2.6	0.93	841.8	20.8	855.5	16.4	891.2	21.0	841.8	20.8
354-U1453A-32F-2W-0-92-Spot 70	221	59438	1.5	14.6290	0.8	1.3172	2.6	0.1398	2.4	0.95	843.3	19.1	853.3	14.7	879.4	17.2	843.3	19.1
354-U1453A-32F-2W-0-92-Spot 201	212	32314	1.3	14.7670	0.9	1.3060	2.3	0.1399	2.2	0.93	843.9	17.1	848.4	13.4	859.9	18.0	843.9	17.1
354-U1453A-32F-2W-0-92-Spot 259	214	102376	1.0	14.5574	0.7	1.3411	2.5	0.1416	2.4	0.96	853.7	19.3	863.7	14.7	889.6	15.3	853.7	19.3
354-U1453A-32F-2W-0-92-Spot 22	243	92576	2.0	14.7333	0.6	1.3379	2.1	0.1430	2.0	0.96	861.4	16.5	862.3	12.3	864.7	11.8	861.4	16.5
354-U1453A-32F-2W-0-92-Spot 18	139	25008	3.3	14.3667	1.0	1.4176	3.0	0.1477	2.8	0.94	888.1	23.3	896.4	17.8	916.8	21.3	916.8	21.3
354-U1453A-32F-2W-0-92-Spot 39	228	294821	1.5	14.3435	0.6	1.4269	1.9	0.1484	1.8	0.95	892.2	15.3	900.2	11.5	920.1	11.8	920.1	11.8
354-U1453A-32F-2W-0-92-Spot 118	1094	712667	31.3	14.3367	0.5	1.4140	1.5	0.1470	1.4	0.95	884.3	11.9	894.8	9.1	921.0	10.2	921.0	10.2
354-U1453A-32F-2W-0-92-Spot 140	814	370455	1.8	14.3240	0.6	1.4136	1.8	0.1469	1.7	0.94	883.3	14.1	894.7	10.8	922.9	12.7	922.9	12.7
354-U1453A-32F-2W-0-92-Spot 114	73	36782	1.2	14.2681	0.8	1.4766	2.7	0.1528	2.6	0.96	916.6	22.5	920.8	16.6	930.9	15.9	930.9	15.9
354-U1453A-32F-2W-0-92-Spot 232	152	40166	2.3	14.2065	0.7	1.4372	2.2	0.1481	2.1	0.95	890.2	17.5	904.5	13.3	939.7	14.8	939.7	14.8
354-U1453A-32F-2W-0-92-Spot 200	509	133918	1.3	14.1616	0.6	1.5340	1.6	0.1576	1.5	0.93	943.2	13.3	944.1	10.0	946.3	11.9	946.3	11.9
354-U1453A-32F-2W-0-92-Spot 5	61	4509	4.0	14.1590	1.1	1.5215	3.2	0.1562	3.0	0.94	935.9	26.2	939.1	19.7	946.6	23.2	946.6	23.2
354-U1453A-32F-2W-0-92-Spot 175	1049	216742	116.3	14.1310	0.7	1.5127	1.6	0.1550	1.4	0.89	929.1	12.4	935.5	9.9	950.7	15.0	950.7	15.0
354-U1453A-32F-2W-0-92-Spot 222	328	67068	3.8	14.1227	0.6	1.5157	2.3	0.1553	2.2	0.96	930.3	18.8	936.8	13.8	951.9	13.1	951.9	13.1
354-U1453A-32F-2W-0-92-Spot 42	564	155857	3.4	14.0813	0.7	1.5019	2.1	0.1534	2.0	0.94	919.9	17.1	931.2	12.9	957.9	14.5	957.9	14.5
354-U1453A-32F-2W-0-92-Spot 168	405	98193	1.9	14.0696	0.6	1.5392	1.7	0.1571	1.6	0.94	940.5	13.9	946.2	10.4	959.6	11.9	959.6	11.9
354-U1453A-32F-2W-0-92-Spot 228	656	68652	1.2	13.9303	0.6	1.5745	1.6	0.1591	1.5	0.94	951.6	13.5	960.2	10.1	979.9	11.7	979.9	11.7
354-U1453A-32F-2W-0-92-Spot 190	195	26925	1.3	13.9166	0.7	1.5692	2.2	0.1584	2.1	0.95	947.8	18.7	958.1	13.9	981.9	14.9	981.9	14.9
354-U1453A-32F-2W-0-92-Spot 135	595	79575	2.0	13.8608	0.8	1.4418	1.9	0.1449	1.8	0.91	872.6	14.4	906.5	11.6	990.1	16.0	990.1	16.0
354-U1453A-32F-2W-0-92-Spot 90	500	163416	2.6	13.8528	0.6	1.6168	1.7	0.1624	1.6	0.93	970.3	14.0	976.7	10.5	991.3	12.4	991.3	12.4
354-U1453A-32F-2W-0-92-Spot 264	875	150462	2.0	13.7374	0.6	1.5479	2.1	0.1542	2.0	0.95	924.6	17.2	949.6	13.0	1008.2	13.0	1008.2	13.0
354-U1453A-32F-2W-0-92-Spot 261	173	38674	1.4	13.6831	0.8	1.5574	2.5	0.1546	2.4	0.95	926.4	20.6	953.4	15.6	1016.2	16.1	1016.2	16.1
354-U1453A-32F-2W-0-92-Spot 304	508	40476	1.7	13.6202	0.6	1.6560	1.7	0.1636	1.6	0.93	976.7	14.7	991.9	11.0	1025.6	12.9	1025.6	12.9
354-U1453A-32F-2W-0-92-Spot 31	62	25257	3.9	13.5826	0.9	1.6703	3.7	0.1645	3.6	0.97	981.9	32.4	997.3	23.3	1031.1	17.5	1031.1	17.5
354-U1453A-32F-2W-0-92-Spot 158	366	2275252	1.7	13.4799	0.6	1.7205	1.7	0.1682	1.6	0.94	1002.2	15.2	1016.2	11.2	1046.5	12.1	1046.5	12.1
354-U1453A-32F-2W-0-92-Spot 293	644	535851	1.3	13.4764	0.8	1.4213	2.0	0.1389	1.8	0.91	838.5	14.5	897.9	12.1	1047.0	17.1	1047.0	17.1
354-U1453A-32F-2W-0-92-Spot 256	42	6538	0.5	13.4334	1.2	1.4517	4.6	0.1414	4.4	0.96	852.8	35.3	910.6	27.5	1053.5	24.2	1053.5	24.2
354-U1453A-32F-2W-0-92-Spot 123	196	86477	1.1	13.4182	1.0	1.7466	2.2	0.1700	2.0	0.90	1012.0	19.0	1025.9	14.5	1055.8	19.4	1055.8	19.4
354-U1453A-32F-2W-0-92-Spot 197	147	16924	1.1	13.3998	0.9	1.8623	2.6	0.1810	2.5	0.94	1072.3	24.3	1067.8	17.2	1058.5	17.6	1058.5	17.6
354-U1453A-32F-2W-0-92-Spot 315	1350	993929	3.0	13.3149	0.7	1.6982	1.6	0.1640	1.4	0.91	978.9	12.9	1007.8	10.0	1071.3	13.1	1071.3	13.1
354-U1453A-32F-2W-0-92-Spot 57	803	66870	3.7	13.2898	0.7	1.7593	2.1	0.1696	1.9	0.93	1009.8	18.0	1030.6	13.3	1075.1	14.7	1075.1	14.7
354-U1453A-32F-2W-0-92-Spot 254	330	52682	1.4	13.2831	0.6	1.9433	1.9	0.1872	1.9	0.96	1106.3	18.8	1096.1	13.0	1076.1	11.1	1076.1	11.1
354-U1453A-32F-2W-0-92-Spot 198	237	186112	1.9	13.2676	0.8	1.9125	2.0	0.1840	1.8	0.91	1089.0	18.2	1085.5	13.3	1078.4	16.2	1078.4	16.2
354-U1453A-32F-2W-0-92-Spot 115	305	36318	0.8	13.2549	0.6	1.8607	2.2	0.1789	2.1	0.96	1060.8	20.3	1067.2	14.3	1080.4	12.1	1080.4	12.1
354-U1453A-32F-2W-0-92-Spot 159	195	96505	2.5	13.2015	0.7	1.8508	2.7	0.1772	2.6	0.97	1051.7	25.1	1063.7	17.6	1088.4	13.2	1088.4	13.2
354-U1453A-32F-2W-0-92-Spot 1	707	218972	3.3	13.1497	0.7	1.8773	2.1	0.1790	1.9	0.94	1061.7	19.0	1073.1	13.7	1096.3	13.9	1096.3	13.9
354-U1453A-32F-2W-0-92-Spot 265	208	22273	2.1	13.1185	0.9	1.8373	2.5	0.1748	2.3	0.93	1038.5	22.5	1058.9	16.5	1101.1	17.8	1101.1	17.8
354-U1453A-32F-2W-0-92-Spot 260	384	551792	2.9	13.1010	0.6	1.7979	1.8	0.1708	1.7	0.95	1016.7	16.2	1044.7	11.9	1103.7	11.8	1103.7	11.8
354-U1453A-32F-2W-0-92-Spot 274	478	276926	1.9	13.0741	0.7	1.8536	2.1	0.1758	2.0	0.94	1043.8	19.3	1064.7	14.1	1107.8	14.9	1107.8	14.9
354-U1453A-32F-2W-0-92-Spot 238	377	333157	1.2	13.0549	0.7	1.8455	2.0	0.1747	1.9	0.94	1038.2	18.1	1061.8	13.2	1110.8	13.4	1110.8	13.4
354-U1453A-32F-2W-0-92-Spot 221	137	103559	0.7	13.0497	0.7	1.9032	2.8	0.1801	2.7	0.97	1067.7	26.9	1082.2	18.8	1111.6	14.0	1111.6	14.0
354-U1453A-32F-2W-0-92-Spot 186	532	201637	2.9	13.0109	0.7	1.9208	1.7	0.1813	1.6	0.92	1073.8	15.7	1088.4	11.5	1117.5	13.6	1117.5	13.6
354-U1453A-32F-2W-0-92-Spot 125	293	64268	1.7	13.0029	0.7	1.8665	2.3	0.1760	2.2	0.96	1045.2	21.7	1069.3	15.5	1118.8	13.6	1118.8	13.6
354-U1453A-32F-2W-0-92-Spot 172	311	110279	2.5	13.0023	0.8	1.9900	1.8	0.1877	1.7	0.91	1108.7	16.8	1112.1	12.3	1118.9	15.4	1118.9	15.4
354-U1453A-32F-2W-0-92-Spot 210	894	228883	3.0	12.9325	0.6	1.8032	1.9	0.1691	1.8	0.95	1007.4	17.2	1046.6	12.7	1129.6	12.3	1129.6	12.3
354-U1453A-32F-2W-0-92-Spot 267	131	181304	0.6	12.7992	0.6	2.0623	1.9	0.1914	1.9	0.95	1129.2	19.2	1136.4	13.3	1150.2	11.7	1150.2	11.7

354-U1453A-32F-2W-0-92-Spot 83	499	93652	1.6	12.7728	0.8	2.0192	2.1	0.1871	2.0	0.93	1105.4	20.2	1122.0	14.5	1154.3	15.3	1154.3	15.3
354-U1453A-32F-2W-0-92-Spot 59	107	16554	1.2	12.7335	0.8	2.1040	2.5	0.1943	2.4	0.95	1144.7	24.8	1150.1	17.2	1160.4	15.9	1160.4	15.9
354-U1453A-32F-2W-0-92-Spot 139	89	17076	0.9	12.7293	0.9	2.0536	2.7	0.1896	2.6	0.95	1119.2	26.4	1133.5	18.5	1161.0	17.4	1161.0	17.4
354-U1453A-32F-2W-0-92-Spot 85	195	65767	0.5	12.7248	0.7	1.8127	2.2	0.1673	2.0	0.94	997.2	18.8	1050.1	14.2	1161.7	14.8	1161.7	14.8
354-U1453A-32F-2W-0-92-Spot 45	940	108065	1.0	12.7022	0.7	1.9164	1.8	0.1766	1.7	0.93	1048.1	16.3	1086.8	12.1	1165.2	13.1	1165.2	13.1
354-U1453A-32F-2W-0-92-Spot 206	85	15760	0.9	12.6631	1.2	2.1134	2.9	0.1941	2.6	0.92	1143.5	27.6	1153.2	19.8	1171.4	22.8	1171.4	22.8
354-U1453A-32F-2W-0-92-Spot 253	313	42640	2.4	12.6037	0.7	1.9142	2.0	0.1750	1.8	0.94	1039.5	17.6	1086.0	13.0	1180.7	13.2	1180.7	13.2
354-U1453A-32F-2W-0-92-Spot 99	306	244686	2.0	12.5873	0.7	2.1861	1.9	0.1996	1.7	0.93	1173.0	18.7	1176.6	13.1	1183.3	13.9	1183.3	13.9
354-U1453A-32F-2W-0-92-Spot 79	790	104110	3.7	12.4929	0.7	2.1927	1.9	0.1987	1.7	0.93	1168.2	18.7	1178.7	13.1	1198.1	13.4	1198.1	13.4
354-U1453A-32F-2W-0-92-Spot 310	89	19028	0.9	12.4633	0.8	2.1302	3.0	0.1926	2.9	0.96	1135.2	30.5	1158.6	21.0	1202.8	15.8	1202.8	15.8
354-U1453A-32F-2W-0-92-Spot 3	177	51388	1.5	12.4422	0.7	2.0863	2.3	0.1883	2.2	0.95	1112.0	22.6	1144.3	16.0	1206.1	14.2	1206.1	14.2
354-U1453A-32F-2W-0-92-Spot 290	165	35135	0.8	12.3915	0.7	2.1896	2.4	0.1968	2.3	0.95	1158.0	24.5	1177.7	16.9	1214.1	14.4	1214.1	14.4
354-U1453A-32F-2W-0-92-Spot 163	402	93952	3.2	12.2627	0.7	1.9471	2.0	0.1732	1.9	0.94	1029.5	18.1	1097.4	13.6	1234.7	13.6	1234.7	13.6
354-U1453A-32F-2W-0-92-Spot 62	268	113550	2.8	12.2083	0.6	2.3606	2.1	0.2090	2.0	0.96	1223.6	22.6	1230.8	15.0	1243.4	11.1	1243.4	11.1
354-U1453A-32F-2W-0-92-Spot 87	1039	844118	3.4	12.1885	0.7	2.2951	2.3	0.2029	2.2	0.96	1190.8	23.7	1210.8	16.1	1246.6	12.9	1246.6	12.9
354-U1453A-32F-2W-0-92-Spot 44	124	19713	2.1	12.0540	0.9	2.4595	2.6	0.2150	2.5	0.94	1255.5	28.3	1260.2	19.0	1268.3	17.2	1268.3	17.2
354-U1453A-32F-2W-0-92-Spot 289	311	262605	2.3	11.9245	0.6	2.5469	1.7	0.2203	1.6	0.94	1283.3	18.8	1285.5	12.6	1289.3	11.6	1289.3	11.6
354-U1453A-32F-2W-0-92-Spot 251	141	54565	1.8	11.7285	0.7	2.6790	2.5	0.2279	2.4	0.96	1323.4	28.2	1322.7	18.1	1321.5	12.9	1321.5	12.9
354-U1453A-32F-2W-0-92-Spot 137	247	228840	1.0	11.5598	0.7	2.6971	2.0	0.2261	1.9	0.94	1314.1	22.2	1327.7	14.7	1349.5	12.9	1349.5	12.9
354-U1453A-32F-2W-0-92-Spot 219	525	115946	1.8	11.5218	0.5	2.2950	1.9	0.1918	1.8	0.96	1131.0	18.5	1210.7	13.2	1355.9	10.5	1355.9	10.5
354-U1453A-32F-2W-0-92-Spot 224	220	268270	1.6	11.3881	0.8	2.6779	2.7	0.2212	2.5	0.96	1288.1	29.6	1322.4	19.6	1378.3	14.9	1378.3	14.9
354-U1453A-32F-2W-0-92-Spot 184	340	155374	1.5	11.3373	0.6	2.6885	2.4	0.2211	2.3	0.97	1287.5	26.6	1325.3	17.4	1386.9	11.8	1386.9	11.8
354-U1453A-32F-2W-0-92-Spot 38	308	103117	22.4	11.3272	1.4	2.6926	2.3	0.2212	1.9	0.81	1288.2	21.8	1326.4	17.2	1388.6	26.4	1388.6	26.4
354-U1453A-32F-2W-0-92-Spot 191	566	169762	1.6	11.1539	0.6	2.9345	1.9	0.2374	1.9	0.96	1373.1	22.9	1390.9	14.6	1418.2	10.6	1418.2	10.6
354-U1453A-32F-2W-0-92-Spot 95	221	75943	1.6	10.9915	0.6	3.0612	2.3	0.2440	2.2	0.96	1407.6	28.2	1423.0	17.8	1446.1	12.4	1446.1	12.4
354-U1453A-32F-2W-0-92-Spot 131	177	29754	1.1	10.8797	0.7	2.9084	2.0	0.2295	1.9	0.94	1331.9	23.2	1384.1	15.5	1465.6	13.1	1465.6	13.1
354-U1453A-32F-2W-0-92-Spot 134	165	31455	1.7	10.7967	0.7	3.0784	2.3	0.2411	2.2	0.95	1392.2	27.4	1427.3	17.7	1480.1	13.6	1480.1	13.6
354-U1453A-32F-2W-0-92-Spot 286	72	112180	1.5	10.6883	0.9	3.3016	3.7	0.2559	3.6	0.97	1469.0	46.9	1481.4	28.7	1499.2	17.6	1499.2	17.6
354-U1453A-32F-2W-0-92-Spot 239	137	49428	1.2	10.4170	0.7	3.1764	2.5	0.2400	2.4	0.96	1386.6	29.7	1451.4	19.1	1547.7	12.7	1547.7	12.7
354-U1453A-32F-2W-0-92-Spot 234	203	35823	1.8	10.3921	0.7	3.1348	2.0	0.2363	1.9	0.94	1367.3	23.4	1441.3	15.5	1552.2	12.4	1552.2	12.4
354-U1453A-32F-2W-0-92-Spot 203	550	397050	1.5	10.3871	0.7	3.2171	1.4	0.2424	1.2	0.86	1398.9	15.6	1461.3	11.2	1553.1	13.8	1553.1	13.8
354-U1453A-32F-2W-0-92-Spot 32	297	87515	1.5	10.3628	0.6	3.5359	1.9	0.2657	1.8	0.95	1519.2	24.6	1535.3	15.1	1557.5	10.6	1557.5	10.6
354-U1453A-32F-2W-0-92-Spot 166	396	137682	1.0	10.2811	0.6	3.5735	1.6	0.2665	1.5	0.93	1522.8	20.6	1543.7	12.9	1572.3	10.9	1572.3	10.9
354-U1453A-32F-2W-0-92-Spot 171	1891	228485	0.7	10.2503	0.6	2.9603	2.0	0.2201	1.9	0.95	1282.3	21.7	1397.5	14.9	1577.9	11.6	1577.9	11.6
354-U1453A-32F-2W-0-92-Spot 235	156	40103	1.0	10.2397	0.7	3.6683	2.6	0.2724	2.5	0.96	1553.1	35.0	1564.5	21.0	1579.9	13.2	1579.9	13.2
354-U1453A-32F-2W-0-92-Spot 240	140	48965	0.9	10.1564	0.8	3.7330	2.3	0.2750	2.2	0.94	1566.0	30.3	1578.5	18.6	1595.2	15.0	1595.2	15.0
354-U1453A-32F-2W-0-92-Spot 2	470	396670	1.3	10.1484	0.6	3.5904	2.0	0.2643	1.9	0.96	1511.6	25.4	1547.4	15.6	1596.6	10.7	1596.6	10.7
354-U1453A-32F-2W-0-92-Spot 7	84	15858	1.2	10.1424	0.7	3.7006	3.2	0.2722	3.1	0.98	1552.0	42.6	1571.5	25.3	1597.7	12.6	1597.7	12.6
354-U1453A-32F-2W-0-92-Spot 116	140	133738	1.7	10.1361	0.7	3.5884	2.3	0.2638	2.2	0.96	1509.3	30.2	1547.0	18.6	1598.9	12.6	1598.9	12.6
354-U1453A-32F-2W-0-92-Spot 157	763	382033	2.9	10.1241	0.6	3.7113	2.0	0.2725	1.9	0.95	1553.5	25.6	1573.8	15.6	1601.1	11.4	1601.1	11.4
354-U1453A-32F-2W-0-92-Spot 291	176	282721	1.0	10.1071	0.5	3.6821	1.6	0.2699	1.5	0.94	1540.3	20.4	1567.5	12.6	1604.2	9.8	1604.2	9.8
354-U1453A-32F-2W-0-92-Spot 266	204	4042259	1.3	10.0309	0.8	3.7377	2.3	0.2719	2.2	0.94	1550.5	29.9	1579.5	18.5	1618.3	15.1	1618.3	15.1
354-U1453A-32F-2W-0-92-Spot 66	119	18700	0.8	10.0267	0.7	3.6880	2.1	0.2682	2.0	0.94	1531.6	26.9	1568.8	16.8	1619.1	13.4	1619.1	13.4
354-U1453A-32F-2W-0-92-Spot 17	284	198517	1.4	10.0211	0.8	3.8404	1.9	0.2791	1.7	0.91	1586.9	24.0	1601.3	15.2	1620.2	14.7	1620.2	14.7
354-U1453A-32F-2W-0-92-Spot 107	498	222376	1.5	9.9949	0.6	3.6930	1.7	0.2677	1.6	0.93	1529.2	22.1	1569.9	14.0	1625.0	12.0	1625.0	12.0
354-U1453A-32F-2W-0-92-Spot 130	48	184457	1.1	9.9904	0.8	3.6125	4.0	0.2617	3.9	0.98	1498.8	51.9	1552.3	31.5	1625.9	14.6	1625.9	14.6
354-U1453A-32F-2W-0-92-Spot 60	417	2579062	1.3	9.9714	0.7	3.6931	2.5	0.2671	2.4	0.96	1526.0	32.6	1569.9	19.9	1629.4	12.7	1629.4	12.7
354-U1453A-32F-2W-0-92-Spot 220	1076	1236595	1.1	9.9021	0.6	3.6563	1.8	0.2626	1.7	0.95	1503.0	22.4	1561.9	14.1	1642.3	10.4	1642.3	10.4
354-U1453A-32F-2W-0-92-Spot 162	206	133135	0.9	9.8849	0.5	3.8947	1.6	0.2792	1.5	0.95	1587.4	21.7	1612.6	13.1	1645.6	9.0	1645.6	9.0
354-U1453A-32F-2W-0-92-Spot 129	380	3511261	1.2	9.8814	0.6	3.8211	2.1	0.2738	2.0	0.96	1560.3	27.2	1597.2	16.5	1646.2	11.0	1646.2	11.0
354-U1453A-32F-2W-0-92-Spot 52	300	1264327	1.1	9.8059	0.7	3.8453	1.8	0.2735	1.6	0.92	1558.4	22.7	1602.3	14.3	1660.4	12.7	1660.4	12.7
354-U1453A-32F-2W-0-92-Spot 249	204	114747	3.4	9.7261	0.7	3.5011	2.1	0.2470	1.9	0.94	1422.8	24.8	1527.5	16.4	1675.6	13.6	1675.6	13.6
354-U1453A-32F-2W-0-92-Spot 4	880	295599	5.8	9.6746	0.7	3.7918	2.0	0.2661	1.9	0.94	1520.8	25.7	1591.0	16.2	1685.4	12.5	1685.4	12.5
354-U1453A-32F-2W-0-92-Spot 68	985	3291697	1.9	9.6150	0.6	3.9861	1.7	0.2780	1.6	0.93	1581.1	22.3	1631.4	13.8	1696.8	11.3	1696.8	11.3
354-U1453A-32F-2W-0-92-Spot 165	107	50569	1.8	9.6020	0.6	4.3509	2.5	0.3030	2.4	0.97	1706.2	35.5	1703.1	20.2	1699.2	11.5	1699.2	11.5
354-U1453A-32F-2W-0-92-Spot 178	342	220178	1.1	9.5992	0.5	4.4522	1.3	0.3100	1.2	0.92	1740.5	18.5	1722.1	10.9	1699.8	9.5	1699.8	9.5
354-U1453A-32F-2W-0-92-Spot 127	217	167109	1.2	9.5679	0.8	4.0455	2.3	0.2807	2.2	0.95	1595.0	31.4	1643.4	19.1	1705.8	13.9	1705.8	13.9
354-U1453A-32F-2W-0-92-Spot 104	630	138273	4.0	9.5507	0.6	4.1171	1.7	0.2852	1.5	0.92	1617.4	21.9	1657.7	13.6	1709.1	11.9	1709.1	11.9
354-U1453A-32F-2W-0-92-Spot 58	438	40411	2.1	9.4765	0.7	4.3961	2.2	0.3021	2.1	0.94	1701.9	30.8	1711.6	18.1	1723.4	13.7	1723.4	13.7
354-U1453A-32F-2W-0-92-Spot 121	267	1294209	1.7	9.4750	0.6	4.4388	1.7	0.3050	1.6	0.94	1716.2	24.4	1719.6	14.3	1723.7	11.2	1723.7	11.2
354-U1453A-32F-2W-0-92-Spot 155	96	18009	0.9	9.3905	0.7	4.5754	2.2	0.3116	2.1	0.95	1748.6	32.8	1744.8	18.7	1740.2	12.3	1740.2	12.3
354-U1453A-32F-2W-0-92-Spot 248	273	124736	2.5	9.3570	0.5	4.5854	2.1	0.3112	2.0	0.97	1746.5	31.0	1746.6	17.4	1746.7	9.3	1746.7	9.3
354-U1453A-32F-2W-0-92-Spot 108	430	170805	2.6	9.3283	0.6	4.2710	2.0	0.2890	1.9	0.95	1636.3	26.9	1687.8	16.1	1752.4	10.7	1752.4	10.7
354-U1453A-32F-2W-0-92-Spot 81	263	81471	0.8	9.2714	0.6	4.1884	1.9	0.2816	1.8	0.96	1599.6	26.2	1671.8	15.9	1763.5	10.4	1763.5	10.4
354-U1453A-32F-2W-0-92-Spot 275	228	105449	3.0	9.2560	0.6	4.5747	2.5	0.3071	2.4	0.97	1726.5	36.2	1744.7	20.6	1766.6	11.9	1766.6	11.9
354-U1453A-32F-2W-0-92-Spot 136	185	358357	2.2	9.2441	0.7	4.6358	1.7	0.3108	1.5	0.91	1744.7	23.7	1755.7	14.1	1768.9	12.5	1768.9	12.5
354-U1453A-32F-2W-0-92-Spot 299	171	28026	1.8	9.2390	0.5	4.6165	1.9	0.3093	1.8	0.96	1737.4	26.8	1752.2	15.3	1769.9	9.0	1769.9	9.0
354-U1453A-32F-2W-0-92-Spot 124	179	101110	1.3	9.1543	0.6	4.7117	2.5	0.3128	2.4	0.97	1754.6	36.4	1769.3	20.6	1786.7	11.5	1786.7	11.5
354-U1453A-32F-2W-0-92-Spot 36	408	67654	1.3	9.1467	0.6	4.5050	2.1	0.2989	2.1	0.96	1685.6	30.6	1731.9	17.8	1788.2	10.5	1788.2	10.5
354-U1453A-32F-2W-0-92-Spot 212	289	409525	0.4	9.1459	0.7	4.4960	2.3	0.2982	2.2	0.95	1682.5	32.7	1730.2	19.3	1788.4	13.5	1788.4	13.5
354-U1453A-32F-2W-0-92-Spot 98	397	350525	7.6	9.1376	0.6	4.7854	1.9	0.3171	1.8	0.95	1775.7	27.5	1782.3	15.6	1790.0	10.4	1790.0	10.4
354-U1453A-32F-2W-0-92-Spot 34	305	150867	2.5	9.1233	0.6	4.7870	2.3	0.3167	2.2	0.97	1773.8	34.3	1782.6	19.2	1792.9	10.1	1792.9	10.1
354-U1453A-32F-2W-0-92-Spot 145	208	94742	2.2	8.9603	0.6	4.7783	2.4	0.3105	2.3	0.96	1743.3	34.8	1781.1	19.9	1825.7	11.3	1825.7	11.3
354-U1453A-32F-2W-0-92-Spot 142	312	325719	2.7	8.8691	0.7	4.9637	2.1	0.3193	2.0	0.95	1786.2	31.8	1813.2	18.1	1844.2	12.1	1844.2	12.1
354-U1453A-32F-2W-0-92-Spot 226	345	380213	1.0	8.8661	0.7	4.7861	2.4	0.3078	2.3	0.95	1729.7	34.3	1782.5	20.0	1844.8	13.4	1844.8	13.4
354-U1453A-32F-2W-0-92-Spot 73	336	149726	1.4	8.8370	0.5	5.0821	1.8	0.3257	1.7	0.95	1817.6	27.6	1833.1	15.5	1850.8	9.9	1850.8	9.9
354-U1453A-32F-2W-0-92-Spot 225	580	282692	2.8	8.7935	0.5	5.1412	1.7	0.3279	1.7	0.96	1828.1	26.7	1842.9	14.8	1859.7	8.8	1859.7	8.8
354-U1453A-32F-2W-0-92-Spot 14	477	154868	2.5	8.7854	0.5	5.2012	2.1	0.3314	2.1	0.97	1845.2	33.3	1852.8	18.3	1861.4	9.7	1861.4	9.7
354-U1453A-32F-2W-0-92-Spot 132	659	382219	1.8	8.7844	0.5	5.0705	2.0	0.3230	2.0	0.97	1804.6	31.1	1831.2	17.2	1861.5	8.2	1861.5	8.2
354-U1453A-32F-2W-0-92-Spot 71	457	401271	2.8	8.7828	0.6	4.8201	1.9	0.3070	1.8	0.95	1726.1	27.9	1788.4	16.3	1861.9	10.9	1861.9	10.9
354-U1453A-32F-2W-0-92-Spot 314	196	45507	1.0	8.7822	0.6	5.3146	2.3	0.3385	2.2	0.97	1879.5	36.5	1871.2	19.7	1862.0	10.0	1862.0	10.0
354-U1453A-32F-2W-0-92-Spot 243	381	132791	2.0	8.7657	0.6	5.1301	1.6	0.3261	1.5	0.94	1819.7	23.2	1841.1	13.3	1865.4	10.0	1865.4	10.0
354-U1453A-32F-2W-0-92-Spot 283	298	54264	3.7	8.7360	0.6	5.2753	2.1	0.3342	2.1	0.96	1858.9	33.4	1864.9	18.3	1871.5	10.3	1871.5	10.3
354-U1453A-32F-2W-0-92-Spot 312	227	48333	4.4	8.7247	0.6	5.2472	1.8	0.3320	1.7	0.95	1848.2	27.2	1860.3	15.2	1873.9	10.2	1873.9	10.2
354-U1453A-32F-2W-0-92-Spot 297	219	732452	2.1	8.7134	0.7	5.2692	2.1	0.3330	2.0	0.95	1852.9	31.7	1863.9	17.8	1876.2	12.3	1876.2	12.3
354-U1453A-32F-2W-0-92-Spot 279	391	574457	2.4	8.6751	0.6	5.3012	1.9	0.3335	1.8	0.96	1855.5	29.5	1869.1	16.3	1884.1	10.0	1884.1	10.0

354-U1453A-32F-2W-0-92-Spot 88	444	4011185	7.1	8.6005	0.5	5.1859	2.0	0.3235	1.9	0.96	1806.7	29.8	1850.3	16.7	1899.7	9.5	1899.7	9.5
354-U1453A-32F-2W-0-92-Spot 211	541	1685737	4.2	8.5356	0.6	5.4670	1.8	0.3384	1.7	0.94	1879.1	27.8	1895.4	15.6	1913.3	11.5	1913.3	11.5
354-U1453A-32F-2W-0-92-Spot 309	637	129951	2.4	8.4671	0.6	5.1851	2.0	0.3184	1.9	0.95	1782.0	28.9	1850.2	16.6	1927.7	11.0	1927.7	11.0
354-U1453A-32F-2W-0-92-Spot 277	286	273146	3.9	8.3113	0.7	5.3398	2.0	0.3219	1.9	0.94	1798.9	30.0	1875.3	17.3	1960.9	12.2	1960.9	12.2
354-U1453A-32F-2W-0-92-Spot 29	432	3961070	2.5	8.3073	0.5	5.5401	1.6	0.3338	1.5	0.94	1856.7	24.3	1906.8	13.8	1961.8	9.8	1961.8	9.8
354-U1453A-32F-2W-0-92-Spot 255	294	109566	1.1	8.2989	0.7	5.6914	1.9	0.3426	1.8	0.93	1899.0	29.1	1930.1	16.4	1963.6	12.4	1963.6	12.4
354-U1453A-32F-2W-0-92-Spot 43	655	382556	2.8	8.2656	0.6	5.0579	1.6	0.3032	1.5	0.93	1707.2	22.1	1829.1	13.5	1970.8	10.7	1970.8	10.7
354-U1453A-32F-2W-0-92-Spot 303	533	218252	2.3	8.1086	0.6	5.9149	1.8	0.3479	1.7	0.94	1924.3	27.7	1963.4	15.4	2004.9	11.0	2004.9	11.0
354-U1453A-32F-2W-0-92-Spot 103	706	141996	2.1	7.9932	0.7	5.2528	1.6	0.3045	1.5	0.91	1713.7	22.3	1861.2	13.8	2030.3	11.7	2030.3	11.7
354-U1453A-32F-2W-0-92-Spot 91	254	948564	1.2	7.9718	0.6	5.3933	2.6	0.3118	2.6	0.97	1749.7	39.1	1883.8	22.5	2035.0	10.8	2035.0	10.8
354-U1453A-32F-2W-0-92-Spot 54	225	125670	2.8	7.8094	0.7	6.5340	1.9	0.3701	1.7	0.92	2029.8	30.2	2050.5	16.5	2071.4	12.6	2071.4	12.6
354-U1453A-32F-2W-0-92-Spot 76	454	1056484	1.7	7.3623	0.5	7.4865	1.6	0.3998	1.5	0.94	2167.9	27.3	2171.4	14.1	2174.6	9.2	2174.6	9.2
354-U1453A-32F-2W-0-92-Spot 285	1430	601523	3.7	7.3065	1.1	7.2770	2.5	0.3856	2.3	0.91	2102.5	41.1	2146.0	22.6	2187.9	18.7	2187.9	18.7
354-U1453A-32F-2W-0-92-Spot 144	59	39297	2.0	7.3026	0.8	7.8061	3.4	0.4134	3.3	0.97	2230.6	61.5	2208.9	30.3	2188.8	14.2	2188.8	14.2
354-U1453A-32F-2W-0-92-Spot 94	196	232187	2.1	7.1921	0.6	7.6573	2.1	0.3994	2.1	0.96	2166.4	37.8	2191.6	19.2	2215.3	10.1	2215.3	10.1
354-U1453A-32F-2W-0-92-Spot 117	370	852927	2.8	6.9430	0.6	7.4032	2.2	0.3728	2.2	0.96	2042.5	37.8	2161.4	20.1	2276.2	10.7	2276.2	10.7
354-U1453A-32F-2W-0-92-Spot 67	2021	367060	7.3	6.6983	0.6	7.8210	1.7	0.3800	1.6	0.95	2076.1	29.0	2210.6	15.6	2337.8	9.6	2337.8	9.6
354-U1453A-32F-2W-0-92-Spot 252	1207	1035310	9.1	6.4349	0.8	8.7823	2.0	0.4099	1.8	0.92	2214.3	33.9	2315.7	17.9	2406.2	12.8	2406.2	12.8
354-U1453A-32F-2W-0-92-Spot 96	290	121618	1.5	6.3697	0.7	8.8916	2.3	0.4108	2.2	0.95	2218.4	40.6	2326.9	20.8	2423.5	12.0	2423.5	12.0
354-U1453A-32F-2W-0-92-Spot 74	326	202937	0.9	6.2789	0.6	9.4318	2.3	0.4295	2.2	0.96	2303.5	43.5	2380.9	21.4	2447.8	10.5	2447.8	10.5
354-U1453A-32F-2W-0-92-Spot 207	132	107655	1.3	6.2440	0.7	10.1296	2.7	0.4587	2.6	0.97	2433.9	53.5	2446.7	25.2	2457.3	11.7	2457.3	11.7
354-U1453A-32F-2W-0-92-Spot 20	66	80268	0.6	6.2383	0.6	10.2116	2.8	0.4620	2.8	0.98	2448.4	56.6	2454.1	26.2	2458.8	9.6	2458.8	9.6
354-U1453A-32F-2W-0-92-Spot 169	119	270598	0.9	6.1886	0.6	10.0575	2.3	0.4514	2.2	0.96	2401.6	44.9	2440.1	21.5	2472.3	10.8	2472.3	10.8
354-U1453A-32F-2W-0-92-Spot 13	227	3542	1.3	6.1209	2.6	8.9235	4.0	0.3961	3.0	0.75	2151.2	55.1	2330.2	36.6	2490.9	44.6	2490.9	44.6
354-U1453A-32F-2W-0-92-Spot 109	119	114742	1.0	6.1190	0.5	9.4014	2.2	0.4172	2.1	0.97	2247.9	39.5	2378.0	19.7	2491.4	9.2	2491.4	9.2
354-U1453A-32F-2W-0-92-Spot 217	305	78288	3.0	6.0912	0.6	9.3561	1.9	0.4133	1.8	0.94	2230.1	33.4	2373.5	17.2	2499.1	10.5	2499.1	10.5
354-U1453A-32F-2W-0-92-Spot 262	355	1633237	1.1	6.0446	0.8	9.8123	2.0	0.4302	1.8	0.93	2306.5	35.7	2417.3	18.3	2512.0	12.6	2512.0	12.6
354-U1453A-32F-2W-0-92-Spot 8	277	1559305	1.2	6.0417	0.7	9.6325	2.1	0.4221	2.0	0.94	2269.9	38.2	2400.3	19.5	2512.8	12.1	2512.8	12.1
354-U1453A-32F-2W-0-92-Spot 11	808	1180398	27.1	5.9911	0.6	9.3137	1.6	0.4047	1.4	0.91	2190.6	26.4	2369.4	14.3	2526.9	10.7	2526.9	10.7
354-U1453A-32F-2W-0-92-Spot 301	338	658877	1.9	5.7818	0.5	11.7012	1.9	0.4907	1.8	0.96	2573.6	37.9	2580.8	17.4	2586.5	8.4	2586.5	8.4
354-U1453A-32F-2W-0-92-Spot 64	296	103606	4.9	5.6114	0.6	11.9527	1.8	0.4864	1.7	0.95	2555.3	36.1	2600.7	16.9	2636.3	9.7	2636.3	9.7
354-U1453A-32F-2W-0-92-Spot 47	1170	1475153	2.5	5.4022	0.5	13.7773	1.8	0.5398	1.7	0.95	2782.6	37.8	2734.5	16.6	2699.2	8.7	2699.2	8.7
354-U1453A-32F-2W-0-92-Spot 143	88	125250	1.9	5.3679	0.7	11.9994	2.6	0.4672	2.5	0.97	2471.1	51.5	2604.4	24.3	2709.7	10.8	2709.7	10.8
354-U1453A-32F-2W-0-92-Spot 152	404	412906	2.1	5.3343	0.7	13.5379	1.9	0.5238	1.7	0.93	2715.1	38.0	2717.9	17.5	2720.0	11.5	2720.0	11.5
354-U1453A-32F-2W-0-92-Spot 244	853	647760	11.6	5.1872	0.6	12.7784	1.8	0.4807	1.7	0.94	2530.5	35.8	2663.5	17.2	2766.0	10.4	2766.0	10.4
354-U1453A-32F-2W-0-92-Spot 97	137	802484	1.6	5.1362	0.7	14.2449	2.6	0.5306	2.5	0.96	2744.2	56.4	2766.2	24.8	2782.2	11.4	2782.2	11.4
354-U1453A-32F-2W-0-92-Spot 245	75	45874	1.6	4.9463	0.6	14.6024	2.6	0.5238	2.5	0.97	2715.5	56.4	2789.7	24.9	2843.8	10.2	2843.8	10.2
354-U1453A-32F-2W-0-92-Spot 106	312	88655	1.6	4.7157	0.6	15.6677	2.6	0.5359	2.6	0.97	2766.1	57.6	2856.8	25.2	2921.3	10.4	2921.3	10.4
354-U1453A-32F-2W-0-92-Spot 78	515	3099569	1.2	4.2926	0.8	17.4257	2.1	0.5425	1.9	0.93	2793.9	43.5	2958.6	19.9	3072.5	12.4	3072.5	12.4
354-U1453A-32F-2W-0-92-Spot 311	184	473331	1.9	4.2007	0.7	17.3563	2.9	0.5288	2.8	0.97	2736.3	61.8	2954.7	27.4	3107.0	10.8	3107.0	10.8

Table ______. U-Pb geochronologic analyses.																		
#9 U1449A_29,30,31F_combined						Isotope ratios						Apparent ages (Ma)						
Early Pleistocene 1.5 Ma																		
Analysis	U	206Pb	U/Th	206Pb*	±	207Pb*	±	206Pb*	±	error	206Pb*	±	207Pb*	±	206Pb*	±	Best age	±
	(ppm)	204Pb		207Pb*	(%)	235U*	(%)	238U	(%)	corr.	238U*	(Ma)	235U	(Ma)	207Pb*	(Ma)	(Ma)	(Ma)
Gleason-354-U1449A-29,30,31F_bagged_5May16-Spot 300	323	312	2.1	3.5963	2.0	0.0311	3.8	0.0008	3.2	0.85	5.2	0.2	31.1	1.2	3352.1	31.2	5.2	0.2
Gleason-354-U1449A-29,30,31F_bagged_5May16-Spot 281	305	817	1.2	9.7177	4.1	0.0152	4.8	0.0011	2.5	0.53	6.9	0.2	15.3	0.7	1677.2	75.6	6.9	0.2
Gleason-354-U1449A-29,30,31F_bagged_5May16-Spot 232	360	880	0.4	8.7329	2.4	0.0207	3.8	0.0013	3.0	0.78	8.5	0.3	20.8	0.8	1872.2	42.8	8.5	0.3
Gleason-354-U1449A-29,30,31F_bagged_5May16-Spot 230	280	743	2.9	13.3673	4.0	0.0142	5.3	0.0014	3.4	0.64	8.9	0.3	14.3	0.7	1063.4	81.2	8.9	0.3
Gleason-354-U1449A-29,30,31F_bagged_5May16-Spot 223	219	262	0.8	6.6876	0.8	0.0315	3.0	0.0015	2.9	0.96	9.8	0.3	31.5	0.9	2340.6	13.8	9.8	0.3
Gleason-354-U1449A-29,30,31F_bagged_5May16-Spot 221	219	245	3.8	2.6212	0.0	0.1258	2.3	0.0024	2.3	1.00	15.4	0.4	120.3	2.7	3837.7	0.1	15.4	0.4
Gleason-354-U1449A-29,30,31F_bagged_5May16-Spot 26	183	673	12.8	11.0642	3.0	0.0339	4.4	0.0027	3.2	0.74	17.5	0.6	33.8	1.5	1433.6	56.8	17.5	0.6
Gleason-354-U1449A-29,30,31F_bagged_5May16-Spot 213	229	2477	4.5	6.5372	2.0	0.0574	3.3	0.0027	2.5	0.78	17.5	0.4	56.6	1.8	2379.4	34.8	17.5	0.4
Gleason-354-U1449A-29,30,31F_bagged_5May16-Spot 29	200	533	10.8	8.6333	2.4	0.0496	3.6	0.0031	2.7	0.74	20.0	0.5	49.2	1.7	1892.8	43.0	20.0	0.5
Gleason-354-U1449A-29,30,31F_bagged_5May16-Spot 23	191	847	5.9	7.9176	2.3	0.0545	3.7	0.0031	2.9	0.78	20.1	0.6	53.9	1.9	2047.1	40.9	20.1	0.6
Gleason-354-U1449A-29,30,31F_bagged_5May16-Spot 285	1618	8210	5.6	15.2549	2.6	0.0333	3.2	0.0037	1.9	0.59	23.7	0.5	33.2	1.0	792.1	54.1	23.7	0.5
Gleason-354-U1449A-29,30,31F_bagged_5May16-Spot 158	227	310	0.3	4.4794	1.6	0.1323	2.7	0.0043	2.1	0.80	27.6	0.6	126.1	3.2	3004.2	25.7	27.6	0.6
Gleason-354-U1449A-29,30,31F_bagged_5May16-Spot 48	188	821	1.1	8.0355	2.2	0.0789	3.6	0.0046	2.9	0.80	29.6	0.8	77.1	2.7	2020.9	38.6	29.6	0.8
Gleason-354-U1449A-29,30,31F_bagged_5May16-Spot 270	111	208	0.9	2.7539	0.0	0.2343	3.2	0.0047	3.2	1.00	30.1	1.0	213.7	6.2	3763.0	0.0	30.1	1.0
Gleason-354-U1449A-29,30,31F_bagged_5May16-Spot 296	55	422	6.8	13.5238	8.8	0.0512	9.4	0.0050	3.3	0.35	32.3	1.1	50.7	4.6	1039.9	178.1	32.3	1.1
Gleason-354-U1449A-29,30,31F_bagged_5May16-Spot 99	78	244	0.5	2.5895	0.1	0.3027	3.5	0.0057	3.5	1.00	36.5	1.3	268.5	8.3	3856.1	1.5	36.5	1.3
Gleason-354-U1449A-29,30,31F_bagged_5May16-Spot 283	68	314	0.6	3.4981	1.5	0.2391	3.2	0.0061	2.9	0.88	39.0	1.1	217.6	6.4	3395.3	23.6	39.0	1.1
Gleason-354-U1449A-29,30,31F_bagged_5May16-Spot 306	89	212	1.8	2.5289	0.0	0.3594	3.3	0.0066	3.3	1.00	42.3	1.4	311.7	9.0	3891.8	0.0	42.3	1.4
Gleason-354-U1449A-29,30,31F_bagged_5May16-Spot 198	137	314	1.4	4.5241	1.7	0.2104	3.1	0.0069	2.6	0.84	44.4	1.1	193.9	5.5	2988.3	26.9	44.4	1.1
Gleason-354-U1449A-29,30,31F_bagged_5May16-Spot 94	128	242	4.1	4.0343	1.2	0.2384	2.9	0.0070	2.6	0.90	44.8	1.2	217.1	5.7	3171.2	19.7	44.8	1.2
Gleason-354-U1449A-29,30,31F_bagged_5May16-Spot 289	70	726	4.8	3.2177	2.5	0.3233	3.9	0.0075	3.0	0.77	48.5	1.5	284.5	9.6	3524.8	38.0	48.5	1.5
Gleason-354-U1449A-29,30,31F_bagged_5May16-Spot 252	544	148958	4.0	19.9938	1.3	0.0523	2.2	0.0076	1.8	0.82	48.7	0.9	51.8	1.1	195.7	29.9	48.7	0.9
Gleason-354-U1449A-29,30,31F_bagged_5May16-Spot 21	1451	20459	0.7	20.9854	0.9	0.0502	2.0	0.0076	1.8	0.89	49.1	0.9	49.7	1.0	82.0	22.2	49.1	0.9
Gleason-354-U1449A-29,30,31F_bagged_5May16-Spot 110	94	1178	1.2	9.7990	5.4	0.1176	6.4	0.0084	3.5	0.54	53.6	1.8	112.9	6.8	1661.8	99.7	53.6	1.8
Gleason-354-U1449A-29,30,31F_bagged_5May16-Spot 139	619	7993	2.6	21.3825	1.2	0.0549	2.4	0.0085	2.0	0.85	54.7	1.1	54.3	1.3	37.3	29.9	54.7	1.1
Gleason-354-U1449A-29,30,31F_bagged_5May16-Spot 258	181	4887	5.8	21.9263	3.2	0.0546	3.9	0.0087	2.3	0.59	55.7	1.3	54.0	2.1	23.1	77.0	55.7	1.3
Gleason-354-U1449A-29,30,31F_bagged_5May16-Spot 234	166	1171	1.8	28.0669	5.3	0.0429	5.8	0.0087	2.2	0.39	56.0	1.2	42.6	2.4	659.3	147.3	56.0	1.2
Gleason-354-U1449A-29,30,31F_bagged_5May16-Spot 248	306	4960	1.5	22.1418	2.2	0.0559	3.5	0.0090	2.7	0.78	57.7	1.6	55.3	1.9	46.8	53.1	57.7	1.6
Gleason-354-U1449A-29,30,31F_bagged_5May16-Spot 164	162	677	0.6	6.5030	1.5	0.1982	2.7	0.0093	2.3	0.83	60.0	1.3	183.6	4.5	2388.3	25.6	60.0	1.3
Gleason-354-U1449A-29,30,31F_bagged_5May16-Spot 276	51	1007	1.5	23.9065	8.9	0.0576	9.4	0.0100	3.2	0.34	64.1	2.0	56.9	5.2	236.8	224.8	64.1	2.0
Gleason-354-U1449A-29,30,31F_bagged_5May16-Spot 4	170	1886	1.0	24.1318	2.8	0.0574	3.8	0.0101	2.5	0.68	64.5	1.6	56.7	2.1	260.5	70.3	64.5	1.6
Gleason-354-U1449A-29,30,31F_bagged_5May16-Spot 169	384	8628	4.1	21.5069	1.6	0.0669	2.4	0.0104	1.8	0.75	66.9	1.2	65.7	1.5	23.5	38.1	66.9	1.2
Gleason-354-U1449A-29,30,31F_bagged_5May16-Spot 146	816	30254	1.1	20.7197	0.8	0.0774	1.7	0.0116	1.5	0.90	74.5	1.1	75.7	1.3	112.2	17.7	74.5	1.1
Gleason-354-U1449A-29,30,31F_bagged_5May16-Spot 168	68	250	0.2	3.5472	0.8	0.4533	2.8	0.0117	2.7	0.96	74.7	2.0	379.6	9.0	3373.5	12.7	74.7	2.0
Gleason-354-U1449A-29,30,31F_bagged_5May16-Spot 147	14	3027	8.6	3.6192	9.2	0.4584	11.0	0.0120	5.9	0.54	77.1	4.5	383.2	35.0	3342.1	144.6	77.1	4.5
Gleason-354-U1449A-29,30,31F_bagged_5May16-Spot 86	127	748	2.0	7.3621	1.2	0.2265	2.9	0.0121	2.6	0.90	77.5	2.0	207.3	5.4	2174.7	21.6	77.5	2.0
Gleason-354-U1449A-29,30,31F_bagged_5May16-Spot 255	52	211	13.5	2.9466	0.2	0.5763	2.9	0.0123	2.9	1.00	78.9	2.3	462.1	10.9	3659.9	3.8	78.9	2.3
Gleason-354-U1449A-29,30,31F_bagged_5May16-Spot 224	81	1085	1.8	27.4534	4.1	0.0653	4.9	0.0130	2.6	0.53	83.3	2.1	64.2	3.0	598.9	112.1	83.3	2.1
Gleason-354-U1449A-29,30,31F_bagged_5May16-Spot 226	46	294	0.3	4.0410	1.5	0.4720	3.9	0.0138	3.6	0.92	88.6	3.1	392.5	12.6	3168.6	24.5	88.6	3.1
Gleason-354-U1449A-29,30,31F_bagged_5May16-Spot 257	587	24902	3.6	20.3609	2.3	0.0964	3.7	0.0142	2.9	0.77	91.1	2.6	93.4	3.3	153.3	54.9	91.1	2.6
Gleason-354-U1449A-29,30,31F_bagged_5May16-Spot 55	203	434	2.2	6.2842	2.5	0.3307	3.7	0.0151	2.7	0.74	96.4	2.6	290.1	9.3	2446.4	42.1	96.4	2.6
Gleason-354-U1449A-29,30,31F_bagged_5May16-Spot 199	48	300	1.8	5.3694	1.7	0.4279	3.6	0.0167	3.2	0.88	106.5	3.4	361.7	11.0	2709.2	28.3	106.5	3.4
Gleason-354-U1449A-29,30,31F_bagged_5May16-Spot 104	784	20671	2.2	20.6373	1.1	0.1163	2.1	0.0174	1.8	0.86	111.3	2.0	111.7	2.2	121.6	25.3	111.3	2.0
Gleason-354-U1449A-29,30,31F_bagged_5May16-Spot 35	1502	338183	2.2	19.8033	0.9	0.1229	2.1	0.0177	1.9	0.90	112.8	2.1	117.7	2.3	217.9	21.1	112.8	2.1
Gleason-354-U1449A-29,30,31F_bagged_5May16-Spot 284	118	998	1.0	12.1360	1.1	0.2082	2.8	0.0183	2.5	0.91	117.0	2.9	192.0	4.8	1255.0	22.1	117.0	2.9
Gleason-354-U1449A-29,30,31F_bagged_5May16-Spot 65	131	2983	0.6	18.6970	1.1	0.1419	3.0	0.0192	2.8	0.93	122.9	3.4	134.8	3.8	349.4	24.9	122.9	3.4
Gleason-354-U1449A-29,30,31F_bagged_5May16-Spot 308	445	43942	1.7	20.2373	0.8	0.1335	2.1	0.0196	2.0	0.93	125.1	2.5	127.2	2.6	167.5	18.0	125.1	2.5
Gleason-354-U1449A-29,30,31F_bagged_5May16-Spot 264	2133	57861	2.1	20.5686	0.8	0.1354	1.9	0.0202	1.7	0.90	128.9	2.2	128.9	2.3	129.4	19.4	128.9	2.2
Gleason-354-U1449A-29,30,31F_bagged_5May16-Spot 129	854	108428	1.7	20.3804	1.0	0.1373	2.2	0.0203	1.9	0.88	129.5	2.4	130.6	2.7	151.0	23.8	129.5	2.4
Gleason-354-U1449A-29,30,31F_bagged_5May16-Spot 228	107	820	0.8	7.8596	1.5	0.3638	3.9	0.0207	3.6	0.92	132.3	4.7	315.0	10.5	2060.1	27.1	132.3	4.7
Gleason-354-U1449A-29,30,31F_bagged_5May16-Spot 3	118	1410	2.0	11.7144	2.2	0.2693	3.5	0.0229	2.7	0.77	145.8	3.9	242.1	7.5	1323.8	42.8	145.8	3.9
Gleason-354-U1449A-29,30,31F_bagged_5May16-Spot 67	270	574	0.4	6.2922	1.0	0.5092	2.0	0.0232	1.8	0.88	148.1	2.6	417.9	7.0	2444.3	16.3	148.1	2.6
Gleason-354-U1449A-29,30,31F_bagged_5May16-Spot 200	271	489	1.5	5.1895	18.8	0.6308	20.7	0.0237	8.7	0.42	151.3	13.0	496.6	81.5	2765.3	311.2	151.3	13.0
Gleason-354-U1449A-29,30,31F_bagged_5May16-Spot 154	43	7684	0.2	11.0133	2.1	0.3090	4.0	0.0247	3.4	0.85	157.2	5.3	273.4	9.6	1442.4	40.7	157.2	5.3
Gleason-354-U1449A-29,30,31F_bagged_5May16-Spot 126	83	1499	0.6	10.1033	2.0	0.3626	3.3	0.0266	2.6	0.79	169.1	4.3	314.2	8.8	1604.9	37.3	169.1	4.3
Gleason-354-U1449A-29,30,31F_bagged_5May16-Spot 237	489	93508	5.4	19.6589	1.0	0.1880	2.7	0.0268	2.5	0.93	170.5	4.2	174.9	4.3	234.9	22.0	170.5	4.2
Gleason-354-U1449A-29,30,31F_bagged_5May16-Spot 13	2615	43157	0.6	12.2658	0.8	0.3210	5.9	0.0286	5.9	0.99	181.5	10.5	282.7	14.6	1234.2	15.6	181.5	10.5
Gleason-354-U1449A-29,30,31F_bagged_5May16-Spot 132	828	509929	1.3	19.7643	0.9	0.1999	2.4	0.0286	2.3	0.92	182.1	4.0	185.0	4.1	222.5	21.6	182.1	4.0
Gleason-354-U1449A-29,30,31F_bagged_5May16-Spot 117	59	1626	0.7	10.3882	1.6	0.4069	4.6	0.0307	4.3	0.93	194.7	8.2	346.7	13.4	1552.9	30.5	194.7	8.2
Gleason-354-U1449A-29,30,31F_bagged_5May16-Spot 186	174	619	0.6	6.4278	1.3	0.6671	2.4	0.0311	2.0	0.84	197.4	3.9	518.9	9.8	2408.1	22.0	197.4	3.9
Gleason-354-U1449A-29,30,31F_bagged_5May16-Spot 176	1161	3511	1.0	13.8576	3.8	0.3134	5.0	0.0315	3.3	0.66	199.9	6.6	276.8	12.2	990.6	76.7	199.9	6.6
Gleason-354-U1449A-29,30,31F_bagged_5May16-Spot 236	43	457	0.8	6.7987	1.6	0.6573	2.9	0.0324	2.5	0.84	205.6	5.0	513.0	11.8	2312.3	27.6	205.6	5.0
Gleason-354-U1449A-29,30,31F_bagged_5May16-Spot 145	253	23003	1.0	19.7080	1.0	0.2283	2.1	0.0326	1.8	0.88	207.0	3.7	208.8	3.9	229.1	22.8	207.0	3.7
Gleason-354-U1449A-29,30,31F_bagged_5May16-Spot 171	49	644	1.1	7.4402	4.4	0.6109	5.8	0.0330	3.7	0.64	209.1	7.6	484.2	22.2	2156.3	76.9	209.1	7.6
Gleason-354-U1449A-29,30,31F_bagged_5May16-Spot 78	1923	830	9.1	7.3234	4.1	0.6275	5.8	0.0333	4.0	0.70	211.4	8.4	494.5	22.7	2183.9	72.0	211.4	8.4
Gleason-354-U1449A-29,30,31F_bagged_5May16-Spot 51	214	39773	3.7	19.3006	1.0	0.2422	2.5	0.0339	2.3	0.92	215.0	4.8	220.2	4.9	277.1	22.0	215.0	4.8
Gleason-354-U1449A-29,30,31F_bagged_5May16-Spot 87	658	37194	1.0	19.7234	0.6	0.2385	2.0	0.0341	1.9	0.95	216.3	4.0	217.2	3.9	227.2	14.7	216.3	4.0
Gleason-354-U1449A-29,30,31F_bagged_5May16-Spot 279	51	421	0.1	4.7994	1.2	1.1013	3.1	0.0383	2.9	0.92	242.5	6.9	754.0	16.7	2892.8	19.7	242.5	6.9
Gleason-354-U1449A-29,30,31F_bagged_5May16-Spot 63	154	1378	0.5	10.7682	1.3	0.5264	2.9	0.0411	2.6	0.89	259.7	6.5	429.4	10.1	1485.1	24.8	259.7	6.5
Gleason-354-U1449A-29,30,31F_bagged_5May16-Spot 142	1443	9447	13.6	12.4292	1.1	0.4648	8.6	0.0419	8.5	0.99	264.6	22.0	387.6	27.6	1208.2	22.2	264.6	22.0
Gleason-354-U1449A-29,30,31F_bagged_5May16-Spot 314	24	343	0.6	3.6443	1.2	1.6122	3.6	0.0426	3.4	0.95	269.0	9.1	975.0	22.8	3331.3	18.1	269.0	9.1
Gleason-354-U1449A-29,30,31F_bagged_5May16-Spot 217	49	276	0.8	3.6986	0.9	1.6147	3.4	0.0433	3.3	0.96	273.3	8.7	975.9	21.2	3308.2	14.6	273.3	8.7
Gleason-354-U1449A-29,30,31F_bagged_5May16-Spot 131	1238	1699	5.2	9.5453	2.6	0.6883	4.7	0.0477	4.0	0.83	300.1	11.6	531.8	19.6	1710.1	48.2	300.1	11.6
Gleason-354-U1449A-29,30,31F_bagged_5May16-Spot 149	157	3935	2.1	14.6178	1.1	0.4728	4.8	0.0501	4.6	0.97	315.3	14.2	393.1	15.5	881.0	23.1	315.3	14.2
Gleason-354-U1449A-29,30,31F_bagged_5May16-Spot 274	31	25741	1.3	18.2627	1.8	0.3797	4.6	0.0503	4.2	0.92	316.3	13.1	326.8	12.9	402.3	39.9	316.3	13.1
Gleason-354-U1449A-29,30,31F_bagged_5May16-Spot 82	61	1769	28.8	11.6110	1.7	0.6429	3.0	0.0541	2.5	0.83	339.9	8.3	504.1	12.0	1341.0	32.6	339.9	8.3
Gleason-354-U1449A-29,30,31F_bagged_5May16-Spot 172	175	3221	2.4	12.1604	0.8	0.6511	2.3	0.0574	2.2	0.95	360.0	7.8	509.2	9.4	1251.1	14.8	360.0	7.8
Gleason-354-U1449A-29,30,31F_bagged_5May16-Spot 267	152	80791	0.3	16.5553	1.0	0.4859	2.5	0.0583	2.3	0.91	365.5	8.1	402.1	8.3	618.0	21.8	365.5	8.1
Gleason-354-U1449A-29,30,31F_bagged_5May16-Spot 188	625	46876	1.7	17.4207	0.8	0.5884	2.8	0.0743	2.7	0.96	462.3	12.1	469.9	10.7	507.0	17.6	462.3	12.1
Gleason-354-U1449A-29,30,31F_bagged_5May16-Spot 301	1068	54870	3.8	17.6399	0.7	0.5842	2.0	0.0747	1.9	0.94	464.7	8.5	467.2	7.5	479.5	14.9	464.7	8.5
Gleason-354-U1449A-29,30,31F_bagged_5May16-Spot 47	165	13736	1.1	17.6745	1.0	0.5880	3.3	0.0754	3.1	0.95	468.4	14.1	469.6	12.3	475.2	21.7	468.4	14.1
Gleason-354-U1449A-29,30,31F_bagged_5May16-Spot 84	3881	267027	2.8	17.6251	0.5	0.5937	1.7	0.0759	1.6	0.96	471.5	7.2	473.2	6.3	481.3	10.7	471.5	7.2
Gleason-354-U1449A-29,30,31F_bagged_5May16-Spot 184	444	44776	2.3	17.3697	0.7	0.6085	2.3	0.0767	2.2	0.96	476.2	10.0	482.6	8.8	513.5	14.5	476.2	10.0
Gleason-354-U1449A-29,30,31F_bagged_5May16-Spot 175	207	26561	2.0	17.5169	0.8	0.6094	2.7	0.0774	2.6	0.96	480.7	12.0	483.2	10.5	494.9	17.6	480.7	12.0
Gleason-354-U1449A-29,30,31F_bagged_5May16-Spot 106	462	29268	3.3	16.8863	0.7	0.6529	2.1	0.0800	2.0	0.93	495.9	9.3	510.3	8.4	575.2	16.2	495.9	9.3
Gleason-354-U1449A-29,30,31F_bagged_5May16-Spot 118	132	290016	0.7	17.2185	0.9	0.6501	2.4	0.0812	2.2	0.92	503.2	10.7	508.5	9.6	532.7	20.2	503.2	10.7
Gleason-354-U1449A-29,30,31F_bagged_5May16-Spot 166	1393	468727	6.7	17.5722	0.5	0.6375	1.5	0.0812	1.4	0.93	503.6	6.6	500.8	5.8	487.9	11.6	503.6	6.6
Gleason-354-U1449A-29,30,31F_bagged_5May16-Spot 254	411	233027	12.0	17.1374	0.6	0.6581	1.5	0.0818	1.3	0.91	506.9	6.5	513.5	6.0	543.0	13.6	506.9	6.5
Gleason-354-U1449A-29,30,31F_bagged_5May16-Spot 115	247	25628	0.9	17.2973	0.8	0.6524	2.1	0.0818	1.9	0.93	507.1	9.4	510.0	8.4	522.6	17.0	507.1	9.4
Gleason-354-U1449A-29,30,31F_bagged_5May16-Spot 165	2716	363456	6.1	17.2704	0.6	0.6534	1.8	0.0818	1.7	0.94	507.1	8.3	510.6	7.3	526.1	14.0	507.1	8.3
Gleason-354-U1449A-29,30,31F_bagged_5May16-Spot 25	222	143218	2.2	17.2949	0.6	0.6618	2.1	0.0830	2.0	0.95	514.1	10.0	515.7	8.6	523.0	14.1	514.1	10.0
Gleason-354-U1449A-29,30,31F_bagged_5May16-Spot 71	683	59024	3.7	17.3848	0.7	0.6597	1.9	0.0832	1.8	0.93	515.1	8.7	514.4	7.6	511.6	15.1	515.1	8.7
Gleason-354-U1449A-29,30,31F_bagged_5May16-Spot 177	162	88192	1.2	17.3162	0.8	0.6707	1.8	0.0842	1.6	0.89	521.3	8.0	521.1	7.3	520.3	17.6	521.3	8.0
Gleason-354-U1449A-29,30,31F_bagged_5May16-Spot 137	285	32123	3.4	17.4282	0.9	0.6687	2.3	0.0845	2.1	0.93	523.1	10.5	519.9	9.2	506.1	18.8	523.1	10.5
Gleason-354-U1449A-29,30,31F_bagged_5May16-Spot 112	175	42709	1.2	17.3213	0.8	0.6733	2.4	0.0846	2.3	0.94	523.4	11.6	522.7	10.0	519.6	18.1	523.4	11.6
Gleason-354-U1449A-29,30,31F_bagged_5May16-Spot 97	1488	142009	69.3	17.2522	0.5	0.6794	1.7	0.0850	1.7	0.95	525.9	8.3	526.4	7.1	528.4	11.5	525.9	8.3
Gleason-354-U1449A-29,30,31F_bagged_5May16-Spot 206	100	9421	0.9	17.2134	1.4	0.6816	3.2	0.0851	2.9	0.89	526.4	14.6	527.7	13.2	533.3	31.4	526.4	14.6
Gleason-354-U1449A-29,30,31F_bagged_5May16-Spot 121	329	23477	1.3	17.3462	0.6	0.6803	2.0	0.0856	1.9	0.95	529.4	9.6	527.0	8.2	516.4	14.2	529.4	9.6
Gleason-354-U1449A-29,30,31F_bagged_5May16-Spot 260	417	205828	21.8	17.1936	0.8	0.6890	1.8	0.0859	1.6	0.88	531.4	8.1	532.2	7.4	535.8	18.3	531.4	8.1
Gleason-354-U1449A-29,30,31F_bagged_5May16-Spot 103	1199	80679	23.4	16.2326	0.8	0.7732	2.0	0.0910	1.8	0.92	561.6	9.9	581.6	8.9	660.4	17.0	561.6	9.9

Gleason-354-U1449A-29,30,31F_bagged_5May16-Spot 22	590	72726	10.4	16.6375	0.6	0.7854	1.7	0.0948	1.6	0.94	583.7	9.0	588.5	7.6	607.3	12.2	583.7	9.0
Gleason-354-U1449A-29,30,31F_bagged_5May16-Spot 98	424	464211	4.0	16.8120	0.7	0.7821	2.2	0.0954	2.1	0.95	587.1	11.5	586.7	9.6	584.8	14.9	587.1	11.5
Gleason-354-U1449A-29,30,31F_bagged_5May16-Spot 133	134	31142	1.6	15.8877	0.9	0.8961	2.6	0.1033	2.5	0.94	633.5	14.9	649.7	12.6	706.3	18.2	633.5	14.9
Gleason-354-U1449A-29,30,31F_bagged_5May16-Spot 44	108	102948	0.8	16.1018	0.8	0.8900	2.5	0.1039	2.3	0.94	637.4	14.0	646.4	11.7	677.7	18.0	637.4	14.0
Gleason-354-U1449A-29,30,31F_bagged_5May16-Spot 240	638	98357	7.9	15.2814	0.7	0.9746	2.4	0.1080	2.3	0.96	661.2	14.4	690.8	12.0	788.5	14.7	661.2	14.4
Gleason-354-U1449A-29,30,31F_bagged_5May16-Spot 298	143	51917	3.9	15.1608	0.9	1.0149	2.7	0.1116	2.5	0.95	682.0	16.3	711.4	13.6	805.1	18.1	682.0	16.3
Gleason-354-U1449A-29,30,31F_bagged_5May16-Spot 167	2481	422373	1.9	15.8074	0.6	1.0067	1.8	0.1154	1.7	0.94	704.1	11.5	707.2	9.3	717.0	13.0	704.1	11.5
Gleason-354-U1449A-29,30,31F_bagged_5May16-Spot 36	1855	256480	5.2	14.9998	0.6	1.1017	1.9	0.1199	1.8	0.95	729.7	12.7	754.2	10.3	827.4	12.4	729.7	12.7
Gleason-354-U1449A-29,30,31F_bagged_5May16-Spot 12	280	78828	13.4	15.3043	0.7	1.0832	2.2	0.1202	2.1	0.95	731.9	14.7	745.2	11.8	785.3	14.7	731.9	14.7
Gleason-354-U1449A-29,30,31F_bagged_5May16-Spot 46	182	81574	1.3	15.2953	0.7	1.1158	2.1	0.1238	2.0	0.95	752.3	14.0	761.0	11.1	786.6	13.7	752.3	14.0
Gleason-354-U1449A-29,30,31F_bagged_5May16-Spot 60	291	36815	2.1	15.4109	0.6	1.1262	2.0	0.1259	1.9	0.95	764.3	13.9	765.9	10.9	770.7	13.0	764.3	13.9
Gleason-354-U1449A-29,30,31F_bagged_5May16-Spot 235	647	129305	3.4	14.9805	0.6	1.2306	1.9	0.1337	1.8	0.95	809.0	13.7	814.6	10.7	830.1	12.5	809.0	13.7
Gleason-354-U1449A-29,30,31F_bagged_5May16-Spot 42	200	538380	1.6	14.9165	0.7	1.2448	1.9	0.1347	1.8	0.92	814.4	13.7	821.1	10.9	839.0	15.4	814.4	13.7
Gleason-354-U1449A-29,30,31F_bagged_5May16-Spot 189	290	79189	4.6	15.1325	0.7	1.2487	2.4	0.1371	2.3	0.95	828.0	18.0	822.8	13.7	809.0	15.3	828.0	18.0
Gleason-354-U1449A-29,30,31F_bagged_5May16-Spot 292	149	45720	2.2	14.9667	0.8	1.2633	2.7	0.1371	2.6	0.96	828.4	20.2	829.4	15.4	832.0	16.8	828.4	20.2
Gleason-354-U1449A-29,30,31F_bagged_5May16-Spot 290	111	96709	3.8	14.1818	1.0	1.3486	2.7	0.1387	2.5	0.93	837.4	19.4	866.9	15.5	943.3	19.5	837.4	19.4
Gleason-354-U1449A-29,30,31F_bagged_5May16-Spot 238	513	77586	1.3	14.7062	0.6	1.3056	2.1	0.1393	2.0	0.96	840.5	16.0	848.2	12.2	868.5	12.7	840.5	16.0
Gleason-354-U1449A-29,30,31F_bagged_5May16-Spot 56	666	109597	3.1	14.2825	0.7	1.3737	2.6	0.1423	2.5	0.96	857.6	19.8	877.7	15.1	928.8	15.3	857.6	19.8
Gleason-354-U1449A-29,30,31F_bagged_5May16-Spot 135	339	60530	2.9	14.4075	0.6	1.4659	1.8	0.1532	1.7	0.93	918.7	14.2	916.4	10.7	910.9	13.2	910.9	13.2
Gleason-354-U1449A-29,30,31F_bagged_5May16-Spot 124	198	61605	1.6	14.1176	0.7	1.5800	2.2	0.1618	2.1	0.94	966.7	18.6	962.4	13.7	952.6	15.0	952.6	15.0
Gleason-354-U1449A-29,30,31F_bagged_5May16-Spot 287	588	138331	2.0	14.0594	0.6	1.5659	2.0	0.1597	1.9	0.95	954.9	16.4	956.8	12.1	961.1	12.9	961.1	12.9
Gleason-354-U1449A-29,30,31F_bagged_5May16-Spot 30	378	73868	2.6	13.9806	0.9	1.4239	2.6	0.1444	2.4	0.93	869.4	19.8	899.0	15.5	972.5	19.2	972.5	19.2
Gleason-354-U1449A-29,30,31F_bagged_5May16-Spot 125	440	146752	1.5	13.9089	0.7	1.6368	2.1	0.1651	1.9	0.94	985.1	17.8	984.5	13.0	983.0	14.2	983.0	14.2
Gleason-354-U1449A-29,30,31F_bagged_5May16-Spot 196	356	582268	2.7	13.9089	0.7	1.5521	2.2	0.1566	2.1	0.95	937.7	18.4	951.3	13.7	983.0	14.6	983.0	14.6
Gleason-354-U1449A-29,30,31F_bagged_5May16-Spot 305	131	32000	0.9	13.8496	0.6	1.6817	2.0	0.1689	1.9	0.95	1006.1	17.6	1001.6	12.6	991.7	12.0	991.7	12.0
Gleason-354-U1449A-29,30,31F_bagged_5May16-Spot 291	571	71033	2.6	13.8250	0.8	1.5313	2.1	0.1535	1.9	0.92	920.8	16.2	943.0	12.6	995.3	16.2	995.3	16.2
Gleason-354-U1449A-29,30,31F_bagged_5May16-Spot 16	587	1415140	2.2	13.8190	0.6	1.5083	2.0	0.1512	1.9	0.95	907.5	16.1	933.8	12.2	996.2	12.1	996.2	12.1
Gleason-354-U1449A-29,30,31F_bagged_5May16-Spot 207	218	45425	2.9	13.8125	0.8	1.5782	2.2	0.1581	2.0	0.92	946.2	17.9	961.7	13.7	997.1	17.0	997.1	17.0
Gleason-354-U1449A-29,30,31F_bagged_5May16-Spot 202	93	89563	1.7	13.7639	0.8	1.7466	2.4	0.1744	2.2	0.95	1036.1	21.4	1025.9	15.3	1004.3	15.6	1004.3	15.6
Gleason-354-U1449A-29,30,31F_bagged_5May16-Spot 312	240	1557390	0.5	13.6557	0.7	1.7175	2.3	0.1701	2.2	0.95	1012.7	20.8	1015.1	14.9	1020.3	14.0	1020.3	14.0
Gleason-354-U1449A-29,30,31F_bagged_5May16-Spot 209	247	14806	0.7	13.5961	0.7	1.4615	2.1	0.1441	2.0	0.94	867.9	16.0	914.6	12.6	1029.1	14.0	1029.1	14.0
Gleason-354-U1449A-29,30,31F_bagged_5May16-Spot 18	69	15517	1.2	13.4730	1.5	1.6470	3.4	0.1609	3.0	0.89	962.0	26.8	988.4	21.2	1047.5	30.2	1047.5	30.2
Gleason-354-U1449A-29,30,31F_bagged_5May16-Spot 102	181	97640	2.6	13.3210	0.8	1.9612	2.7	0.1895	2.5	0.95	1118.5	26.1	1102.3	17.9	1070.4	16.0	1070.4	16.0
Gleason-354-U1449A-29,30,31F_bagged_5May16-Spot 170	56	13446	2.4	13.1946	0.8	2.0052	2.7	0.1919	2.5	0.95	1131.6	26.4	1117.3	18.0	1089.5	16.0	1089.5	16.0
Gleason-354-U1449A-29,30,31F_bagged_5May16-Spot 201	211	152198	0.9	13.1849	0.6	1.9123	1.6	0.1829	1.5	0.91	1082.6	14.6	1085.4	10.7	1090.9	13.0	1090.9	13.0
Gleason-354-U1449A-29,30,31F_bagged_5May16-Spot 311	311	191389	2.1	13.1640	0.7	1.7876	2.7	0.1707	2.6	0.96	1015.8	24.1	1041.0	17.3	1094.2	14.2	1094.2	14.2
Gleason-354-U1449A-29,30,31F_bagged_5May16-Spot 309	908	1922515	20.0	13.0430	0.5	1.9289	1.9	0.1825	1.8	0.97	1080.4	18.2	1091.2	12.7	1112.6	9.7	1112.6	9.7
Gleason-354-U1449A-29,30,31F_bagged_5May16-Spot 268	878	112346	1.5	12.9203	0.6	1.9068	1.6	0.1787	1.5	0.93	1059.8	14.4	1083.5	10.6	1131.5	11.7	1131.5	11.7
Gleason-354-U1449A-29,30,31F_bagged_5May16-Spot 244	435	125311	6.2	12.9179	0.9	1.8840	2.7	0.1765	2.6	0.95	1047.9	24.8	1075.5	17.9	1131.8	17.3	1131.8	17.3
Gleason-354-U1449A-29,30,31F_bagged_5May16-Spot 120	1268	171455	107.0	12.8933	0.6	1.6869	2.3	0.1577	2.2	0.96	944.2	19.7	1003.6	14.9	1135.6	12.5	1135.6	12.5
Gleason-354-U1449A-29,30,31F_bagged_5May16-Spot 27	228	72976	5.7	12.8927	0.7	1.8396	2.7	0.1720	2.6	0.97	1023.2	24.5	1059.7	17.6	1135.7	13.5	1135.7	13.5
Gleason-354-U1449A-29,30,31F_bagged_5May16-Spot 130	74	23227	1.3	12.8472	1.4	1.8285	2.8	0.1704	2.4	0.87	1014.2	22.5	1055.7	18.1	1142.7	26.9	1142.7	26.9
Gleason-354-U1449A-29,30,31F_bagged_5May16-Spot 107	200	70257	1.2	12.8057	0.5	2.1840	2.6	0.2028	2.6	0.98	1190.6	27.9	1176.0	18.2	1149.2	10.5	1149.2	10.5
Gleason-354-U1449A-29,30,31F_bagged_5May16-Spot 92	74	54262	1.0	12.7473	0.7	2.1825	2.3	0.2018	2.2	0.95	1184.9	23.9	1175.5	16.2	1158.2	14.4	1158.2	14.4
Gleason-354-U1449A-29,30,31F_bagged_5May16-Spot 157	438	78577	2.5	12.7402	0.8	1.8667	2.4	0.1725	2.2	0.95	1025.8	21.2	1069.4	15.7	1159.3	15.2	1159.3	15.2
Gleason-354-U1449A-29,30,31F_bagged_5May16-Spot 218	88	44842	1.1	12.7295	0.8	2.0884	3.2	0.1928	3.1	0.97	1136.5	32.6	1145.0	22.1	1161.0	15.3	1161.0	15.3
Gleason-354-U1449A-29,30,31F_bagged_5May16-Spot 212	79	26990	1.7	12.6757	0.7	2.1442	2.3	0.1971	2.2	0.95	1159.8	23.0	1163.2	15.8	1169.4	14.2	1169.4	14.2
Gleason-354-U1449A-29,30,31F_bagged_5May16-Spot 62	344	88238	1.0	12.5778	0.8	2.2170	2.5	0.2022	2.4	0.95	1187.3	25.8	1186.4	17.6	1184.8	15.9	1184.8	15.9
Gleason-354-U1449A-29,30,31F_bagged_5May16-Spot 41	386	261135	2.0	12.3640	0.8	2.2308	2.0	0.2000	1.8	0.92	1175.6	19.7	1190.8	13.9	1218.5	14.9	1218.5	14.9
Gleason-354-U1449A-29,30,31F_bagged_5May16-Spot 116	233	243979	6.1	12.3353	0.6	2.4386	2.0	0.2182	1.9	0.95	1272.2	22.3	1254.1	14.6	1223.1	12.1	1223.1	12.1
Gleason-354-U1449A-29,30,31F_bagged_5May16-Spot 134	42	47737	1.1	12.0124	1.1	2.1965	2.9	0.1914	2.7	0.92	1128.8	27.6	1179.9	20.1	1275.0	21.8	1275.0	21.8
Gleason-354-U1449A-29,30,31F_bagged_5May16-Spot 251	160	65326	1.7	11.9926	0.7	2.5157	2.4	0.2188	2.3	0.96	1275.6	26.3	1276.6	17.2	1278.2	12.8	1278.2	12.8
Gleason-354-U1449A-29,30,31F_bagged_5May16-Spot 215	116	17639	1.1	11.9908	0.9	2.1591	2.5	0.1878	2.3	0.93	1109.3	23.2	1168.0	17.1	1278.5	18.1	1278.5	18.1
Gleason-354-U1449A-29,30,31F_bagged_5May16-Spot 295	251	154815	3.8	11.7158	0.9	2.4151	2.7	0.2052	2.5	0.94	1203.3	27.6	1247.1	19.2	1323.6	17.8	1323.6	17.8
Gleason-354-U1449A-29,30,31F_bagged_5May16-Spot 31	68	57502	1.2	11.2707	0.6	2.8762	2.7	0.2351	2.6	0.97	1361.2	31.7	1375.7	20.0	1398.2	12.1	1398.2	12.1
Gleason-354-U1449A-29,30,31F_bagged_5May16-Spot 119	537	309947	8.5	11.2516	0.7	2.7560	2.7	0.2249	2.7	0.97	1307.7	31.5	1343.7	20.5	1401.5	12.9	1401.5	12.9
Gleason-354-U1449A-29,30,31F_bagged_5May16-Spot 52	124	108006	1.3	10.7071	3.8	2.7036	5.2	0.2099	3.5	0.68	1228.5	39.5	1329.4	38.5	1495.9	72.2	1495.9	72.2
Gleason-354-U1449A-29,30,31F_bagged_5May16-Spot 39	339	94361	1.3	10.3940	0.5	3.1463	2.1	0.2372	2.1	0.98	1372.0	25.8	1444.1	16.5	1551.9	8.6	1551.9	8.6
Gleason-354-U1449A-29,30,31F_bagged_5May16-Spot 100	77	179586	1.1	10.3243	0.7	3.5009	2.6	0.2621	2.5	0.97	1500.8	34.0	1527.4	20.8	1564.5	12.6	1564.5	12.6
Gleason-354-U1449A-29,30,31F_bagged_5May16-Spot 96	373	1084927	1.5	10.2196	0.6	3.7670	1.9	0.2792	1.7	0.94	1587.4	24.5	1585.7	14.9	1583.6	12.0	1583.6	12.0
Gleason-354-U1449A-29,30,31F_bagged_5May16-Spot 162	132	65205	2.1	10.2166	0.7	3.7354	2.2	0.2768	2.1	0.94	1575.1	29.4	1579.0	17.9	1584.1	14.0	1584.1	14.0
Gleason-354-U1449A-29,30,31F_bagged_5May16-Spot 79	242	139025	0.7	10.1609	0.7	3.5993	2.4	0.2652	2.3	0.96	1516.6	30.9	1549.4	18.9	1594.3	12.4	1594.3	12.4
Gleason-354-U1449A-29,30,31F_bagged_5May16-Spot 20	111	49261	1.5	10.1404	0.8	3.8464	3.1	0.2829	3.0	0.96	1605.9	42.3	1602.5	24.8	1598.1	15.2	1598.1	15.2
Gleason-354-U1449A-29,30,31F_bagged_5May16-Spot 64	178	45646	0.6	10.1305	0.8	3.2096	3.1	0.2358	3.0	0.97	1364.9	37.0	1459.5	24.0	1599.9	14.3	1599.9	14.3
Gleason-354-U1449A-29,30,31F_bagged_5May16-Spot 299	323	413768	1.9	10.1199	0.6	3.4733	2.2	0.2549	2.1	0.96	1463.8	27.6	1521.2	17.3	1601.9	11.5	1601.9	11.5
Gleason-354-U1449A-29,30,31F_bagged_5May16-Spot 49	235	92215	0.9	10.1170	0.6	3.7002	1.9	0.2715	1.8	0.95	1548.4	24.9	1571.4	15.2	1602.4	10.6	1602.4	10.6
Gleason-354-U1449A-29,30,31F_bagged_5May16-Spot 288	295	390703	2.3	9.9705	0.6	3.7695	2.1	0.2726	2.0	0.95	1553.9	27.4	1586.3	16.7	1629.6	11.5	1629.6	11.5
Gleason-354-U1449A-29,30,31F_bagged_5May16-Spot 190	66	24913	1.0	9.5464	0.7	4.4171	3.3	0.3058	3.2	0.98	1720.1	48.5	1715.6	27.3	1709.9	13.3	1709.9	13.3
Gleason-354-U1449A-29,30,31F_bagged_5May16-Spot 50	238	402860	1.1	9.4853	0.6	4.1193	2.2	0.2834	2.1	0.96	1608.4	29.7	1658.1	17.8	1721.7	11.3	1721.7	11.3
Gleason-354-U1449A-29,30,31F_bagged_5May16-Spot 278	111	62924	1.4	9.4749	0.7	4.4887	2.3	0.3085	2.2	0.95	1733.1	33.0	1728.9	18.9	1723.8	12.5	1723.8	12.5
Gleason-354-U1449A-29,30,31F_bagged_5May16-Spot 38	524	156973	0.4	9.4728	0.7	4.3454	2.3	0.2985	2.2	0.95	1684.1	32.0	1702.0	18.8	1724.2	12.9	1724.2	12.9
Gleason-354-U1449A-29,30,31F_bagged_5May16-Spot 250	1063	400053	6.7	9.2928	0.6	4.6402	2.0	0.3127	1.9	0.95	1754.2	28.8	1756.5	16.6	1759.3	11.6	1759.3	11.6
Gleason-354-U1449A-29,30,31F_bagged_5May16-Spot 181	379	194260	2.9	9.1942	0.6	4.7550	2.1	0.3171	2.1	0.96	1775.4	32.0	1777.0	18.0	1778.8	10.4	1778.8	10.4
Gleason-354-U1449A-29,30,31F_bagged_5May16-Spot 191	434	109486	2.0	8.9141	0.6	4.0649	3.1	0.2628	3.1	0.98	1504.1	41.0	1647.3	25.5	1835.0	11.5	1835.0	11.5
Gleason-354-U1449A-29,30,31F_bagged_5May16-Spot 144	493	1263186	6.0	8.8598	0.8	5.0026	2.2	0.3215	2.1	0.93	1796.8	32.6	1819.8	19.0	1846.1	15.3	1846.1	15.3
Gleason-354-U1449A-29,30,31F_bagged_5May16-Spot 180	514	708854	7.5	8.7102	0.6	5.3375	1.7	0.3372	1.6	0.94	1873.1	25.8	1874.9	14.5	1876.9	10.4	1876.9	10.4
Gleason-354-U1449A-29,30,31F_bagged_5May16-Spot 91	106	376048	3.6	8.6787	0.6	5.4439	2.2	0.3427	2.1	0.96	1899.5	34.4	1891.8	18.6	1883.4	10.4	1883.4	10.4
Gleason-354-U1449A-29,30,31F_bagged_5May16-Spot 57	567	20323	3.7	8.4794	0.9	4.9388	2.2	0.3037	2.0	0.91	1709.8	30.2	1808.9	18.7	1925.1	16.6	1925.1	16.6
Gleason-354-U1449A-29,30,31F_bagged_5May16-Spot 277	153	32244	1.0	8.0408	0.6	6.2397	1.9	0.3639	1.8	0.95	2000.5	30.7	2010.0	16.5	2019.8	10.4	2019.8	10.4
Gleason-354-U1449A-29,30,31F_bagged_5May16-Spot 33	127	34000	1.6	7.4503	0.8	6.8878	3.0	0.3722	2.8	0.96	2039.6	49.8	2097.1	26.3	2153.9	14.5	2153.9	14.5
Gleason-354-U1449A-29,30,31F_bagged_5May16-Spot 88	101	85399	1.6	7.3547	0.6	7.6888	3.7	0.4101	3.7	0.99	2215.5	69.1	2195.3	33.6	2176.4	11.0	2176.4	11.0
Gleason-354-U1449A-29,30,31F_bagged_5May16-Spot 194	11	969	1.8	6.1167	1.0	8.8566	4.6	0.3929	4.5	0.98	2136.3	81.8	2323.3	42.1	2492.0	17.2	2492.0	17.2
Gleason-354-U1449A-29,30,31F_bagged_5May16-Spot 32	48	2771336	0.6	5.9858	0.7	11.3138	3.0	0.4912	3.0	0.97	2575.7	62.7	2549.3	28.3	2528.4	11.6	2528.4	11.6
Gleason-354-U1449A-29,30,31F_bagged_5May16-Spot 303	103	87622	2.2	5.9449	0.8	10.7357	2.7	0.4629	2.6	0.96	2452.3	52.4	2500.5	24.9	2539.9	13.0	2539.9	13.0
Gleason-354-U1449A-29,30,31F_bagged_5May16-Spot 9	35	174527	0.8	5.5028	0.7	13.3707	3.1	0.5336	3.1	0.98	2756.7	68.5	2706.2	29.5	2668.7	11.0	2668.7	11.0
Gleason-354-U1449A-29,30,31F_bagged_5May16-Spot 203	107	62067	1.9	5.0497	0.7	14.6292	3.0	0.5358	2.9	0.97	2765.7	65.0	2791.4	28.3	2810.0	11.5	2810.0	11.5
Gleason-354-U1449A-29,30,31F_bagged_5May16-Spot 178	30	34503	13.1	3.8677	0.8	24.1085	4.0	0.6763	3.9	0.98	3330.1	102.3	3272.8	39.1	3237.9	12.0	3237.9	12.0
-Spot 35	244	256	21.7	51.4941	13.9	0.0020	14.5	0.0007	3.9	0.27	4.8	0.2	2.0	0.3	2767.0	1069.2	4.8	0.2
-Spot 62	200	1654	1.2	24.7890	11.7	0.0218	12.0	0.0039	2.3	0.19	25.2	0.6	21.8	2.6	329.1	302.7	25.2	0.6
-Spot 75	67	1465	1.3	26.8490	6.2	0.0209	7.4	0.0041	4.1	0.55	26.2	1.1	21.0	1.5	538.7	167.2	26.2	1.1
-Spot 65	385	2949	1.3	20.3451	4.4	0.0278	4.9	0.0041	2.3	0.47	26.4	0.6	27.8	1.4	155.1	102.2	26.4	0.6
-Spot 36	66	737	118.5	22.2348	6.3	0.0265	7.4	0.0043	3.9	0.53	27.5	1.1	26.5	1.9	57.0	153.7	27.5	1.1
-Spot 64	187	596	0.5	3.6646	3.7	0.1761	4.2	0.0047	2.0	0.48	30.1	0.6	164.7	6.4	3322.7	57.8	30.1	0.6
-Spot 28	442	103278	1.6	17.8036	2.8	0.0400	3.3	0.0052	1.8	0.54	33.2	0.6	39.8	1.3	459.1	62.1	33.2	0.6
-Spot 96	8064	2742	0.1	15.1010	4.9	0.0478	5.3	0.0052	2.1	0.39	33.6	0.7	47.4	2.5	813.4	102.4	33.6	0.7
-Spot 30	6035	606038	11.2	20.9267	0.8	0.0471	1.7	0.0071	1.5	0.88	45.9	0.7	46.7	0.8	88.7	19.7	45.9	0.7
-Spot 6	2884	953796	2.6	20.6011	1.0	0.0504	3.5	0.0075	3.4	0.96	48.4	1.6	50.0	1.7	125.7	23.1	48.4	1.6
-Spot 94	1228	11074	1.0	21.6152	1.1	0.0497	1.9	0.0078	1.5	0.81	50.0	0.8	49.2	0.9	11.4	27.3	50.0	0.8
-Spot 110	406	15803	15.1	19.8700	2.1	0.0552	2.9	0.0080	2.0	0.69	51.1	1.0	54.5	1.5	210.1	48.1	51.1	1.0
-Spot 11	1720	17329	1.7	21.7049	1.1	0.0514	1.8	0.0081	1.5	0.81	52.0	0.8	50.9	0.9	1.4	25.8	52.0	0.8
-Spot 76	575	3452	3.1	20.3872	3.1	0.0559	3.6	0.0083	1.8	0.50	53.1	1.0	55.2	1.9	150.3	73.3	53.1	1.0
-Spot 84	201	2623	2.1	23.9018	6.9	0.0504	7.0	0.0087	1.5	0.22	56.1	0.9	50.0	3.4	236.3	173.7	56.1	0.9
-Spot 41	124	832	1.5	33.8569	3.7	0.0357	4.4	0.0088	2.4	0.54	56.2	1.3	35.6	1.5	1207.0	115.1	56.2	1.3
-Spot 7	246	6694	2.1	20.1584	1.9	0.0604	3.0	0.0088	2.3	0.77	56.7	1.3	59.6	1.8	176.6	44.9	56.7	1.3
-Spot 42	168	7198	0.8	21.5419	2.1	0.0605	3.5	0.0095	2.8	0.80	60.7	1.7	59.7	2.0	19.5	50.8	60.7	1.7
-Spot 29	406	16419	0.9	20.3338	1.5	0.0720	2.9	0.0106	2.4	0.85	68.1	1.7	70.6	1.9	156.4	34.9	68.1	1.7
-Spot 107	391	2257	0.5	14.9437	2.9	0.1073	3.6	0.0116	2.2	0.61	74.5	1.6	103.5	3.6	835.2	60.1	74.5	1.6

-Spot 52	285	14606	1.1	20.6640	1.5	0.0836	3.4	0.0125	3.1	0.90	80.3	2.5	81.6	2.7	118.6	35.3	80.3	2.5
-Spot 32	1280	21581	1.2	20.0199	1.0	0.1160	2.2	0.0168	2.0	0.89	107.7	2.1	111.5	2.3	192.7	23.0	107.7	2.1
-Spot 18	124	1143	2.7	28.6999	2.0	0.0840	3.3	0.0175	2.6	0.80	111.8	2.9	81.9	2.6	721.1	55.6	111.8	2.9
-Spot 92	123	18569	2.2	20.1158	2.0	0.1203	3.5	0.0175	2.9	0.82	112.1	3.2	115.3	3.9	181.5	47.6	112.1	3.2
-Spot 22	2040	189436	1.3	20.4175	0.6	0.1193	1.4	0.0177	1.2	0.91	112.9	1.4	114.4	1.5	146.8	12.9	112.9	1.4
-Spot 27	669	11783	1.5	20.8841	1.0	0.1198	2.0	0.0182	1.7	0.85	116.0	2.0	114.9	2.2	93.5	24.7	116.0	2.0
-Spot 24	188	19970	1.3	19.3226	2.0	0.1340	2.9	0.0188	2.2	0.74	120.0	2.6	127.7	3.5	274.5	45.2	120.0	2.6
-Spot 50	302	6652	1.5	21.4064	2.3	0.1256	4.3	0.0195	3.6	0.84	124.5	4.4	120.1	4.8	34.7	56.2	124.5	4.4
-Spot 63	869	11012	2.0	21.0597	0.8	0.1289	2.1	0.0197	1.9	0.92	125.7	2.4	123.1	2.4	73.7	19.7	125.7	2.4
-Spot 15	2051	27746	34.0	15.3125	0.7	0.1951	1.8	0.0217	1.7	0.93	138.2	2.3	180.9	3.0	784.2	13.9	138.2	2.3
-Spot 99	610	161357	0.8	18.6708	1.7	0.1620	2.7	0.0219	2.1	0.79	139.9	3.0	152.5	3.8	352.6	37.6	139.9	3.0
-Spot 104	785	1628	1.1	8.6366	10.8	0.3711	11.4	0.0232	3.6	0.31	148.1	5.2	320.4	31.3	1892.1	195.5	148.1	5.2
-Spot 2	106	2077	1.3	21.3873	3.3	0.1714	4.1	0.0266	2.4	0.59	169.2	4.1	160.7	6.2	36.8	80.1	169.2	4.1
-Spot 40	1242	245994	3.8	19.8652	0.6	0.2202	1.4	0.0317	1.3	0.89	201.3	2.5	202.1	2.6	210.7	14.9	201.3	2.5
-Spot 39	1248	35503	3.3	19.7285	0.6	0.2225	1.7	0.0318	1.6	0.94	202.1	3.2	204.0	3.1	226.6	13.6	202.1	3.2
-Spot 71	1073	34301	33.6	16.8375	0.8	0.3147	2.4	0.0384	2.3	0.94	243.1	5.4	277.8	5.9	581.5	18.0	243.1	5.4
-Spot 16	2372	231448	39.8	17.3982	0.6	0.3274	3.0	0.0413	3.0	0.98	260.9	7.6	287.6	7.6	509.9	13.9	260.9	7.6
-Spot 9	1726	99148	28.8	17.4848	1.1	0.3853	8.7	0.0489	8.7	0.99	307.5	26.0	330.9	24.6	498.9	23.2	307.5	26.0
-Spot 25	112	4065	1.0	17.3621	3.5	0.4281	5.2	0.0539	3.8	0.73	338.4	12.5	361.8	15.8	514.4	77.3	338.4	12.5
-Spot 78	908	337277	3.2	16.4905	2.0	0.4673	5.5	0.0559	5.1	0.93	350.6	17.3	389.3	17.7	626.5	43.9	350.6	17.3
-Spot 89	568	55048	1.7	17.4031	0.7	0.4480	2.4	0.0565	2.3	0.96	354.6	7.9	375.9	7.5	509.3	14.7	354.6	7.9
-Spot 21	2393	56764	1.1	17.3566	0.7	0.5245	2.1	0.0660	2.0	0.95	412.2	8.1	428.1	7.5	515.1	14.5	412.2	8.1
-Spot 46	2283	503761	4.5	17.3229	0.7	0.5713	2.0	0.0718	1.8	0.94	446.8	8.0	458.9	7.2	519.4	14.3	446.8	8.0
-Spot 101	683	129874	1.4	17.4462	0.6	0.5995	1.5	0.0759	1.4	0.93	471.3	6.5	476.9	5.8	503.8	12.6	471.3	6.5
-Spot 54	692	132637	1.0	17.3800	0.8	0.6037	1.8	0.0761	1.6	0.90	472.8	7.2	479.6	6.7	512.2	16.9	472.8	7.2
-Spot 87	646	180914	1.3	17.5874	0.7	0.6072	4.8	0.0775	4.8	0.99	480.9	22.1	481.8	18.4	486.1	14.6	480.9	22.1
-Spot 38	660	71641	2.7	17.4458	0.8	0.6160	2.6	0.0779	2.5	0.95	483.8	11.6	487.4	10.1	503.9	17.2	483.8	11.6
-Spot 90	587	17081	1.0	17.2582	1.1	0.6232	3.0	0.0780	2.8	0.93	484.2	13.2	491.8	11.8	527.6	24.1	484.2	13.2
-Spot 37	145	76535	1.6	17.2061	0.9	0.6322	2.9	0.0789	2.8	0.95	489.5	13.0	497.5	11.4	534.3	19.9	489.5	13.0
-Spot 73	262	295358	0.7	17.0221	1.0	0.6549	2.2	0.0809	2.0	0.90	501.2	9.5	511.5	8.8	557.7	20.7	501.2	9.5
-Spot 83	185	281244	2.0	16.7724	1.0	0.6648	2.5	0.0809	2.3	0.92	501.3	11.1	517.6	10.1	589.9	21.3	501.3	11.1
-Spot 91	503	76428	1.7	17.4293	0.6	0.6421	1.7	0.0812	1.6	0.95	503.1	7.8	503.6	6.8	506.0	12.1	503.1	7.8
-Spot 13	425	104905	2.3	17.3422	0.7	0.6520	2.0	0.0820	1.8	0.93	508.1	8.9	509.7	7.9	517.0	16.0	508.1	8.9
-Spot 34	294	45575	1.2	17.2636	0.7	0.6642	2.1	0.0832	2.0	0.93	515.0	9.7	517.2	8.5	527.0	16.4	515.0	9.7
-Spot 66	208	24043	6.4	16.7172	0.7	0.7930	2.8	0.0961	2.7	0.97	591.8	15.5	592.9	12.6	597.0	14.1	591.8	15.5
-Spot 88	181	42458	3.1	15.8572	0.8	0.8875	2.3	0.1021	2.1	0.93	626.5	12.8	645.0	10.9	710.4	17.4	626.5	12.8
-Spot 10	719	35135	6.9	14.9904	0.7	1.0349	2.3	0.1125	2.2	0.95	687.3	14.2	721.4	11.8	828.7	14.5	687.3	14.2
-Spot 26	164	25778	0.3	15.0773	0.9	1.1158	3.0	0.1220	2.8	0.95	742.2	19.8	761.0	15.9	816.7	19.1	742.2	19.8
-Spot 80	299	348412	3.1	14.2426	0.6	1.4259	2.2	0.1473	2.1	0.96	885.8	17.5	899.8	13.2	934.5	12.9	934.5	12.9
-Spot 61	174	77719	0.8	14.2410	0.6	1.4715	2.6	0.1520	2.5	0.97	912.1	21.6	918.7	15.7	934.8	12.1	934.8	12.1
-Spot 58	912	512676	30.5	13.9181	0.6	1.3559	2.3	0.1369	2.2	0.96	826.9	17.2	870.1	13.5	981.7	12.9	981.7	12.9
-Spot 48	753	33433	0.9	13.3772	0.7	1.5619	2.4	0.1515	2.2	0.95	909.6	19.0	955.2	14.6	1061.9	14.4	1061.9	14.4
-Spot 74	131	182468	2.0	13.2830	0.7	1.9070	2.7	0.1837	2.6	0.96	1087.3	26.2	1083.5	18.1	1076.1	14.7	1076.1	14.7
-Spot 14	209	43597	2.0	13.0449	0.7	1.7345	2.6	0.1641	2.5	0.97	979.6	23.1	1021.4	16.9	1112.3	13.1	1112.3	13.1
-Spot 17	41	100361	2.2	12.8768	1.0	1.9766	3.2	0.1846	3.0	0.95	1092.0	30.2	1107.6	21.3	1138.2	19.3	1138.2	19.3
-Spot 85	420	96648	4.6	12.8024	0.6	1.7889	2.1	0.1661	2.0	0.96	990.6	18.7	1041.4	13.8	1149.7	11.6	1149.7	11.6
-Spot 82	88	39713	1.3	12.7472	0.8	1.9659	3.6	0.1818	3.5	0.97	1076.6	34.8	1103.9	24.3	1158.2	16.5	1158.2	16.5
-Spot 19	300	19457	1.8	12.7431	0.6	2.1534	2.2	0.1990	2.1	0.96	1170.1	22.8	1166.1	15.4	1158.9	12.7	1158.9	12.7
-Spot 8	25	5661	0.6	12.7131	1.3	1.9887	4.1	0.1834	3.8	0.95	1085.3	38.4	1111.7	27.4	1163.5	25.8	1163.5	25.8
-Spot 86	53	21493	1.3	12.6838	0.8	2.1900	3.0	0.2015	2.9	0.96	1183.1	31.5	1177.9	21.1	1168.1	16.6	1168.1	16.6
-Spot 53	171	105374	2.0	12.6802	0.7	2.1608	2.4	0.1987	2.3	0.95	1168.4	24.8	1168.5	16.9	1168.7	14.5	1168.7	14.5
-Spot 81	405	85913	1.0	12.6752	0.6	1.8328	2.2	0.1685	2.1	0.97	1003.8	19.9	1057.3	14.5	1169.5	11.1	1169.5	11.1
-Spot 57	148	102495	0.7	12.6632	0.7	2.2011	2.8	0.2022	2.7	0.97	1186.9	29.3	1181.4	19.5	1171.4	14.0	1171.4	14.0
-Spot 98	432	744168	13.3	12.4788	0.6	2.0440	2.1	0.1850	2.0	0.96	1094.2	20.0	1130.3	14.1	1200.3	11.7	1200.3	11.7
-Spot 95	647	400396	1.2	11.1614	0.5	2.7311	1.8	0.2211	1.7	0.96	1287.6	20.1	1336.9	13.3	1416.9	9.4	1416.9	9.4
-Spot 68	33	115372	1.2	10.7352	1.0	3.0810	4.6	0.2399	4.5	0.98	1386.1	56.2	1428.0	35.4	1491.0	18.8	1491.0	18.8
-Spot 105	76	151348	1.3	10.7110	0.9	3.3847	2.8	0.2629	2.6	0.95	1504.8	35.2	1500.9	21.6	1495.2	16.3	1495.2	16.3
-Spot 4	142	46582	1.6	10.5407	0.7	3.3751	2.8	0.2580	2.7	0.96	1479.7	35.8	1498.6	22.0	1525.5	14.1	1525.5	14.1
-Spot 5	283	388494	1.2	10.2365	0.6	3.5540	2.7	0.2639	2.6	0.97	1509.6	34.9	1539.3	21.1	1580.5	11.6	1580.5	11.6
-Spot 77	667	214325	2.7	10.1080	0.4	3.7634	1.7	0.2759	1.6	0.97	1570.7	22.9	1585.0	13.7	1604.1	8.3	1604.1	8.3
-Spot 45	120	49499	1.4	10.0597	0.7	3.7714	2.4	0.2752	2.3	0.96	1566.9	32.1	1586.7	19.3	1613.0	12.5	1613.0	12.5
-Spot 102	248	1939385	1.4	9.7093	0.6	4.0340	2.0	0.2841	1.9	0.95	1611.8	27.4	1641.1	16.4	1678.8	11.4	1678.8	11.4
-Spot 55	222	77024	1.5	9.5818	0.7	4.3870	2.9	0.3049	2.8	0.97	1715.4	42.2	1709.9	23.8	1703.1	12.0	1703.1	12.0
-Spot 108	155	147089	2.3	9.3746	0.5	4.7903	2.6	0.3257	2.5	0.98	1817.5	39.6	1783.2	21.5	1743.3	9.6	1743.3	9.6
-Spot 3	344	119310	4.6	9.0229	0.6	4.6010	2.2	0.3011	2.2	0.97	1696.7	32.4	1749.4	18.7	1813.0	10.0	1813.0	10.0
-Spot 100	378	201821	1.5	8.7809	0.5	4.7705	2.1	0.3038	2.0	0.97	1710.2	30.1	1779.7	17.4	1862.3	9.5	1862.3	9.5
-Spot 1	162	45592	2.6	8.4104	0.7	4.9917	2.3	0.3045	2.2	0.96	1713.5	33.2	1817.9	19.5	1939.7	11.9	1939.7	11.9
-Spot 12	315	145622	7.7	8.0075	0.7	6.1200	2.0	0.3554	1.9	0.94	1960.4	32.5	1993.1	17.8	2027.1	12.1	2027.1	12.1
-Spot 20	133	772730	1.2	6.3540	0.6	9.7156	3.0	0.4477	2.9	0.98	2385.1	58.8	2408.2	27.8	2427.7	10.9	2427.7	10.9
-Spot 44	384	929801	1.4	5.6764	0.6	11.7044	1.6	0.4819	1.5	0.92	2535.4	31.6	2581.1	15.3	2617.1	10.6	2617.1	10.6
-Spot 60	127	532098	1.3	4.2572	0.5	20.5827	2.4	0.6355	2.4	0.98	3171.3	59.9	3119.1	23.7	3085.7	8.5	3085.7	8.5

Table _____. U-Pb geochronologic analyses.																		
#10 U1450A_70F						Isotope ratios						Apparent ages (Ma)						
Late Pliocene 2.9 Ma																		
Analysis	U	206Pb	U/Th	206Pb*	±	207Pb*	±	206Pb*	±	error	206Pb*	±	207Pb*	±	206Pb*	±	Best age	±
	(ppm)	204Pb		207Pb*	(%)	235U*	(%)	238U	(%)	corr.	238U*	(Ma)	235U	(Ma)	207Pb*	(Ma)	(Ma)	(Ma)
354U1450A_70F-Spot 72	873	26160	147.3	19.7631	5.0	0.0055	5.5	0.0008	2.1	0.39	5.0	0.1	5.5	0.3	222.6	116.4	5.0	0.1
354U1450A_70F-Spot 130	3648	26452	171.0	18.1897	2.7	0.0073	3.1	0.0010	1.4	0.46	6.2	0.1	7.4	0.2	411.2	60.8	6.2	0.1
354U1450A_70F-Spot 196	437	45643	4.2	19.9753	5.6	0.0149	6.1	0.0022	2.5	0.40	13.9	0.3	15.0	0.9	197.9	130.8	13.9	0.3
354U1450A_70F-Spot 236	471	29476	0.7	21.2658	4.9	0.0176	5.4	0.0027	2.2	0.42	17.4	0.4	17.7	0.9	50.4	116.0	17.4	0.4
354U1450A_70F-Spot 37	2164	70329	8.8	21.6201	2.6	0.0198	3.0	0.0031	1.4	0.46	20.0	0.3	19.9	0.6	10.9	63.5	20.0	0.3
354U1450A_70F-Spot 146	1053	34163	234.8	19.7429	3.2	0.0222	3.5	0.0032	1.4	0.41	20.5	0.3	22.3	0.8	225.0	73.5	20.5	0.3
354U1450A_70F-Spot 265	242	29566	170.2	20.3596	6.0	0.0229	6.4	0.0034	2.1	0.33	21.8	0.5	23.0	1.4	153.4	140.7	21.8	0.5
354U1450A_70F-Spot 287	1086	108281	107.7	20.8787	3.0	0.0269	3.4	0.0041	1.4	0.43	26.2	0.4	27.0	0.9	94.1	72.0	26.2	0.4
354U1450A_70F-Spot 25	4057	22360		14.4508	7.1	0.0420	7.4	0.0044	2.0	0.26	28.3	0.6	41.7	3.0	904.7	147.5	28.3	0.6
354U1450A_70F-Spot 78	751	36772	1.7	21.5478	2.8	0.0283	3.2	0.0044	1.5	0.47	28.5	0.4	28.4	0.9	18.9	67.7	28.5	0.4
354U1450A_70F-Spot 82	318	16323	0.8	22.1589	4.6	0.0298	4.9	0.0048	1.7	0.35	30.8	0.5	29.8	1.4	48.7	112.1	30.8	0.5
354U1450A_70F-Spot 46	608	15319	268.3	21.1561	3.8	0.0328	4.1	0.0050	1.8	0.42	32.4	0.6	32.8	1.3	62.8	89.6	32.4	0.6
354U1450A_70F-Spot 219	359	13275	0.9	16.6754	5.3	0.0431	5.7	0.0052	1.9	0.34	33.5	0.6	42.9	2.4	602.4	115.4	33.5	0.6
354U1450A_70F-Spot 279	902	31174	15.9	21.4417	2.3	0.0401	2.9	0.0062	1.7	0.61	40.0	0.7	39.9	1.1	30.7	54.5	40.0	0.7
354U1450A_70F-Spot 42	302	42801	3.3	20.5257	4.7	0.0456	5.1	0.0068	2.1	0.40	43.6	0.9	45.3	2.3	134.3	110.8	43.6	0.9
354U1450A_70F-Spot 107	1094	47722	2.3	21.5293	1.7	0.0446	2.6	0.0070	1.9	0.75	44.8	0.9	44.3	1.1	21.0	40.4	44.8	0.9
354U1450A_70F-Spot 58	143	8975	1.7	21.9923	4.8	0.0474	5.0	0.0076	1.5	0.30	48.6	0.7	47.1	2.3	30.4	116.2	48.6	0.7
354U1450A_70F-Spot 235	848	134065	32.8	17.3537	3.2	0.0605	3.6	0.0076	1.6	0.44	48.9	0.8	59.6	2.1	515.5	70.8	48.9	0.8
354U1450A_70F-Spot 246	842	39466	1.4	21.2695	1.9	0.0497	2.6	0.0077	1.8	0.70	49.2	0.9	49.3	1.3	50.0	44.7	49.2	0.9
354U1450A_70F-Spot 294	901	59495	3.0	21.3478	2.5	0.0507	2.9	0.0079	1.6	0.54	50.4	0.8	50.3	1.4	41.3	58.7	50.4	0.8
354U1450A_70F-Spot 271	110	9634	2.4	19.2018	7.1	0.0571	7.4	0.0080	2.1	0.28	51.1	1.1	56.4	4.1	288.9	163.2	51.1	1.1
354U1450A_70F-Spot 39	112	8773	2.0	21.3440	5.5	0.0521	5.9	0.0081	2.2	0.37	51.8	1.1	51.6	3.0	41.7	131.4	51.8	1.1
354U1450A_70F-Spot 177	1359	38883	3.2	21.6838	1.8	0.0522	2.2	0.0082	1.3	0.58	52.7	0.7	51.7	1.1	3.7	42.9	52.7	0.7
354U1450A_70F-Spot 228	179	5944	2.2	21.9596	4.8	0.0533	5.4	0.0085	2.5	0.46	54.5	1.4	52.7	2.8	26.8	116.0	54.5	1.4
354U1450A_70F-Spot 122	318	27413	1.6	21.8164	3.4	0.0568	3.7	0.0090	1.4	0.39	57.6	0.8	56.0	2.0	10.9	82.1	57.6	0.8
354U1450A_70F-Spot 314	1556	70515	17.0	21.2591	1.8	0.0625	2.2	0.0096	1.3	0.58	61.8	0.8	61.6	1.3	51.2	42.7	61.8	0.8
354U1450A_70F-Spot 135	850	43265	1.4	20.8220	1.6	0.0643	2.0	0.0097	1.2	0.61	62.3	0.8	63.3	1.2	100.6	37.3	62.3	0.8
354U1450A_70F-Spot 273	90	29161	2.0	20.1258	5.2	0.0672	5.6	0.0098	2.1	0.37	62.9	1.3	66.0	3.6	180.4	120.3	62.9	1.3
354U1450A_70F-Spot 197	398	55191	1.7	20.7935	2.0	0.0662	2.3	0.0100	1.3	0.55	64.0	0.8	65.1	1.5	103.8	46.2	64.0	0.8
354U1450A_70F-Spot 85	182	5792	0.9	18.6312	5.3	0.0760	5.6	0.0103	1.8	0.33	65.9	1.2	74.4	4.0	357.4	118.8	65.9	1.2
354U1450A_70F-Spot 175	97	5851	1.1	22.4698	5.4	0.0643	5.8	0.0105	2.3	0.39	67.2	1.5	63.3	3.6	82.7	131.2	67.2	1.5
354U1450A_70F-Spot 100	34	1903	2.3	22.8591	8.7	0.0643	9.0	0.0107	2.4	0.26	68.4	1.6	63.3	5.5	124.9	214.9	68.4	1.6
354U1450A_70F-Spot 304	307	155722	1.8	21.4673	2.6	0.0699	3.0	0.0109	1.6	0.53	69.8	1.1	68.6	2.0	27.9	61.8	69.8	1.1
354U1450A_70F-Spot 29	1140	47376	3.3	21.0341	1.5	0.0776	2.0	0.0118	1.2	0.63	75.8	0.9	75.9	1.4	76.5	36.2	75.8	0.9
354U1450A_70F-Spot 38	58	12752	1.7	22.3057	5.4	0.0834	5.8	0.0135	2.1	0.36	86.4	1.8	81.3	4.5	64.8	131.3	86.4	1.8
354U1450A_70F-Spot 34	183	42831	2.0	20.5572	3.1	0.1104	3.6	0.0165	1.8	0.49	105.3	1.8	106.3	3.6	130.7	73.6	105.3	1.8
354U1450A_70F-Spot 241	158	33808	1.8	20.6285	3.1	0.1160	3.5	0.0173	1.7	0.48	110.9	1.8	111.4	3.7	122.6	72.0	110.9	1.8
354U1450A_70F-Spot 31	450	34274	2.9	21.0119	1.8	0.1145	2.6	0.0174	1.8	0.70	111.5	2.0	110.1	2.7	79.0	43.6	111.5	2.0
354U1450A_70F-Spot 64	465	32429	2.0	20.4913	1.8	0.1199	2.5	0.0178	1.7	0.69	113.8	1.9	114.9	2.7	138.3	41.8	113.8	1.9
354U1450A_70F-Spot 158	152	18436	1.5	22.0018	2.9	0.1117	3.2	0.0178	1.5	0.45	113.8	1.6	107.5	3.3	31.4	70.0	113.8	1.6
354U1450A_70F-Spot 137	92	49189	1.5	20.4005	3.1	0.1284	3.7	0.0190	2.0	0.53	121.3	2.4	122.7	4.3	148.7	73.6	121.3	2.4
354U1450A_70F-Spot 299	631	104008	5.6	20.5068	1.9	0.1327	2.2	0.0197	1.2	0.54	126.0	1.5	126.5	2.6	136.5	43.6	126.0	1.5
354U1450A_70F-Spot 3	174	41579	1.3	19.8882	2.5	0.1391	3.1	0.0201	1.7	0.56	128.0	2.2	132.2	3.8	208.0	58.9	128.0	2.2
354U1450A_70F-Spot 291	216	224934	1.5	21.2205	2.6	0.1306	3.2	0.0201	1.8	0.57	128.3	2.3	124.7	3.7	55.5	62.4	128.3	2.3
354U1450A_70F-Spot 81	87	32112	1.3	21.0135	3.7	0.1356	4.0	0.0207	1.3	0.33	131.9	1.7	129.1	4.8	78.8	88.7	131.9	1.7
354U1450A_70F-Spot 240	551	154324	9.2	17.8975	1.4	0.1717	1.9	0.0223	1.3	0.70	142.1	1.9	160.9	2.8	447.3	30.1	142.1	1.9
354U1450A_70F-Spot 127	298	66064	10.6	18.5255	2.8	0.2173	3.7	0.0292	2.4	0.66	185.5	4.4	199.7	6.6	370.2	62.1	185.5	4.4
354U1450A_70F-Spot 221	588	98963	15.0	19.4498	1.2	0.2248	2.2	0.0317	1.8	0.83	201.3	3.6	205.9	4.1	259.4	28.5	201.3	3.6
354U1450A_70F-Spot 222	935	73249	0.8	19.9353	1.4	0.2217	1.9	0.0321	1.4	0.71	203.4	2.8	203.3	3.6	202.5	31.6	203.4	2.8
354U1450A_70F-Spot 249	645	409845	25.8	20.1573	1.4	0.2257	2.0	0.0330	1.4	0.69	209.3	2.8	206.7	3.7	176.7	33.2	209.3	2.8
354U1450A_70F-Spot 208	322	58514	2.9	20.0026	1.8	0.2275	2.2	0.0330	1.3	0.60	209.3	2.7	208.1	4.1	194.7	41.1	209.3	2.7
354U1450A_70F-Spot 97	674	70750	2.9	19.9621	1.5	0.2313	2.0	0.0335	1.3	0.67	212.4	2.8	211.3	3.9	199.4	35.0	212.4	2.8
354U1450A_70F-Spot 49	500	57327	4.3	19.8190	1.6	0.2333	2.3	0.0335	1.7	0.72	212.6	3.5	212.9	4.4	216.1	37.3	212.6	3.5
354U1450A_70F-Spot 121	272	187942	2.0	20.3338	2.3	0.2283	2.7	0.0337	1.5	0.55	213.5	3.1	208.8	5.1	156.4	53.2	213.5	3.1
354U1450A_70F-Spot 52	416	67533	4.3	19.6530	1.0	0.2717	1.7	0.0387	1.4	0.80	245.0	3.3	244.1	3.7	235.5	23.9	245.0	3.3
354U1450A_70F-Spot 14	55	47546	1.4	19.4143	3.2	0.2780	3.8	0.0391	1.9	0.51	247.6	4.7	249.1	8.3	263.7	74.4	247.6	4.7
354U1450A_70F-Spot 194	916	45593	8.3	16.7044	2.8	0.3680	4.0	0.0446	2.8	0.70	281.2	7.7	318.2	10.8	598.7	61.0	281.2	7.7
354U1450A_70F-Spot 105	218	70418	4.7	17.7577	1.8	0.3834	2.4	0.0494	1.6	0.68	310.7	5.0	329.5	6.8	464.8	39.4	310.7	5.0
354U1450A_70F-Spot 174	621	209037	14.6	17.7619	1.0	0.3984	1.8	0.0513	1.5	0.83	322.7	4.7	340.5	5.2	464.2	22.1	322.7	4.7
354U1450A_70F-Spot 187	329	201352	7.5	17.5156	1.4	0.4674	2.3	0.0594	1.8	0.80	371.8	6.6	389.4	7.3	495.1	29.8	371.8	6.6
354U1450A_70F-Spot 237	225	72706	1.7	18.1159	1.5	0.5378	2.0	0.0707	1.3	0.65	440.1	5.4	437.0	7.0	420.3	33.6	440.1	5.4
354U1450A_70F-Spot 65	418	165169	9.1	17.7140	1.1	0.5737	1.9	0.0737	1.6	0.83	458.5	7.0	460.4	7.0	470.2	23.4	458.5	7.0
354U1450A_70F-Spot 10	448	50098	11.4	17.5838	1.0	0.5816	2.2	0.0742	2.0	0.89	461.3	8.7	465.5	8.2	486.5	22.2	461.3	8.7
354U1450A_70F-Spot 93	487	122404	15.0	17.4237	1.1	0.5964	2.0	0.0754	1.7	0.85	468.4	7.6	474.9	7.5	506.7	23.2	468.4	7.6
354U1450A_70F-Spot 159	325	210190	10.3	17.3017	1.2	0.6051	2.0	0.0759	1.6	0.81	471.8	7.3	480.5	7.6	522.1	25.6	471.8	7.3
354U1450A_70F-Spot 155	1331	266287	2.4	17.5147	1.0	0.6017	2.2	0.0764	2.0	0.90	474.8	9.0	478.3	8.3	495.2	21.1	474.8	9.0
354U1450A_70F-Spot 36	205	210070	2.0	17.0779	1.8	0.6191	2.1	0.0767	1.1	0.53	476.3	5.2	489.3	8.2	550.6	38.9	476.3	5.2
354U1450A_70F-Spot 110	1435	214632	3.1	17.4699	1.0	0.6135	1.8	0.0777	1.6	0.84	482.6	7.3	485.7	7.1	500.9	21.9	482.6	7.3
354U1450A_70F-Spot 56	367	64834	8.2	17.2843	1.0	0.6215	1.9	0.0779	1.6	0.85	483.6	7.4	490.8	7.3	524.3	21.9	483.6	7.4
354U1450A_70F-Spot 50	230	61271	2.7	17.6147	1.5	0.6173	2.1	0.0789	1.5	0.69	489.3	7.0	488.2	8.3	482.7	34.0	489.3	7.0
354U1450A_70F-Spot 254	324	57404	1.5	17.2410	1.4	0.6308	2.0	0.0789	1.4	0.71	489.4	6.6	496.6	7.8	529.8	30.9	489.4	6.6
354U1450A_70F-Spot 274	1187	73508	3.1	17.5482	0.9	0.6311	2.2	0.0803	2.0	0.91	498.1	9.5	496.8	8.6	491.0	20.5	498.1	9.5
354U1450A_70F-Spot 201	46	32814	1.5	17.2817	2.5	0.6412	2.8	0.0804	1.4	0.48	498.3	6.6	503.1	11.3	524.6	54.6	498.3	6.6
354U1450A_70F-Spot 156	673	170881	29.3	17.5550	0.9	0.6315	1.9	0.0804	1.6	0.88	498.5	7.9	497.0	7.3	490.1	19.5	498.5	7.9
354U1450A_70F-Spot 62	295	73718	2.5	16.9774	1.2	0.6531	1.7	0.0804	1.3	0.74	498.6	6.2	510.4	7.0	563.4	25.5	498.6	6.2
354U1450A_70F-Spot 132	641	58095	2.1	17.5049	1.2	0.6336	2.8	0.0804	2.5	0.91	498.7	12.2	498.3	11.1	496.4	25.8	498.7	12.2
354U1450A_70F-Spot 48	434	62725	2.3	17.2248	0.9	0.6459	1.6	0.0807	1.4	0.84	500.2	6.6	506.0	6.5	531.9	19.3	500.2	6.6
354U1450A_70F-Spot 151	1419	166709	4.4	17.3620	0.9	0.6413	2.0	0.0808	1.8	0.89	500.6	8.6	503.1	8.0	514.4	20.7	500.6	8.6
354U1450A_70F-Spot 73	587	4769393	2.6	17.3124	1.0	0.6432	2.4	0.0808	2.2	0.90	500.7	10.4	504.3	9.5	520.7	22.5	500.7	10.4

354U1450A_70F-Spot 59	186	62258	2.6	17.2702	1.5	0.6476	2.1	0.0811	1.5	0.70	502.8	7.2	507.0	8.4	526.1	32.9	502.8	7.2
354U1450A_70F-Spot 309	2448	55357	25.8	17.4154	1.0	0.6515	1.9	0.0823	1.6	0.84	509.8	7.8	509.4	7.6	507.7	22.4	509.8	7.8
354U1450A_70F-Spot 26	61	44957	0.8	17.4820	1.9	0.6498	2.5	0.0824	1.6	0.64	510.4	7.7	508.4	9.9	499.3	42.0	510.4	7.7
354U1450A_70F-Spot 77	441	124827	2.2	17.3714	1.2	0.6558	2.2	0.0826	1.8	0.82	511.8	8.7	512.0	8.7	513.3	27.4	511.8	8.7
354U1450A_70F-Spot 245	693	489100	2.5	17.4224	1.0	0.6546	2.2	0.0827	1.9	0.90	512.3	9.6	511.3	8.7	506.8	21.0	512.3	9.6
354U1450A_70F-Spot 160	1396	84198	12.6	17.4000	1.1	0.6628	2.0	0.0836	1.7	0.85	517.9	8.5	516.4	8.1	509.6	23.2	517.9	8.5
354U1450A_70F-Spot 209	1052	89113	3.6	17.3559	0.9	0.6687	2.3	0.0842	2.1	0.92	521.0	10.4	519.9	9.2	515.2	20.0	521.0	10.4
354U1450A_70F-Spot 154	107	25206	1.4	17.4854	2.0	0.6649	2.4	0.0843	1.3	0.54	521.8	6.4	517.6	9.6	498.9	44.1	521.8	6.4
354U1450A_70F-Spot 306	433	78127	2.9	17.4658	1.2	0.6676	1.8	0.0846	1.4	0.76	523.3	6.9	519.2	7.3	501.4	25.8	523.3	6.9
354U1450A_70F-Spot 103	41	63981	0.7	17.4074	2.7	0.6701	3.0	0.0846	1.3	0.44	523.5	6.7	520.8	12.3	508.7	59.6	523.5	6.7
354U1450A_70F-Spot 257	242	88821	1.3	17.3306	1.3	0.6740	1.9	0.0847	1.5	0.76	524.2	7.4	523.2	7.9	518.4	27.6	524.2	7.4
354U1450A_70F-Spot 163	95	37350	3.0	17.2380	1.9	0.6874	2.4	0.0859	1.4	0.59	531.5	7.1	531.2	9.7	530.2	41.5	531.5	7.1
354U1450A_70F-Spot 195	981	155583	23.6	17.0032	0.9	0.7012	1.5	0.0865	1.1	0.79	534.6	5.9	539.5	6.1	560.2	19.4	534.6	5.9
354U1450A_70F-Spot 255	416	81055	3.3	16.7490	0.9	0.7159	1.6	0.0870	1.3	0.81	537.6	6.6	548.3	6.7	592.9	20.4	537.6	6.6
354U1450A_70F-Spot 205	2443	113769	3.9	17.3316	0.7	0.6924	1.6	0.0870	1.5	0.91	537.9	7.6	534.2	6.8	518.3	15.1	537.9	7.6
354U1450A_70F-Spot 167	1975	115252	39.1	17.0182	0.9	0.7155	1.7	0.0883	1.4	0.84	545.6	7.4	548.0	7.2	558.2	20.0	545.6	7.4
354U1450A_70F-Spot 83	365	55211	9.0	16.1319	1.1	0.7585	1.6	0.0887	1.1	0.71	548.1	5.9	573.1	6.9	673.7	23.4	548.1	5.9
354U1450A_70F-Spot 161	500	55704	0.8	17.2219	0.7	0.7115	2.1	0.0889	2.0	0.94	548.9	10.3	545.6	8.9	532.3	16.2	548.9	10.3
354U1450A_70F-Spot 230	44	37825	0.8	17.0595	3.0	0.7310	3.3	0.0904	1.4	0.42	558.2	7.6	557.1	14.4	552.9	66.2	558.2	7.6
354U1450A_70F-Spot 33	1167	456006	64.7	16.8637	0.9	0.7427	1.9	0.0908	1.7	0.87	560.5	9.0	564.0	8.3	578.1	20.1	560.5	9.0
354U1450A_70F-Spot 106	430	138336	16.6	16.9249	1.1	0.7445	1.9	0.0914	1.6	0.82	563.8	8.4	565.0	8.2	570.2	23.4	563.8	8.4
354U1450A_70F-Spot 13	367	83447	10.8	16.5546	1.0	0.8193	2.0	0.0984	1.7	0.85	604.9	9.7	607.7	9.0	618.1	22.1	604.9	9.7
354U1450A_70F-Spot 270	566	148696	9.0	16.1469	1.0	0.8490	1.7	0.0994	1.3	0.79	611.1	7.8	624.1	7.9	671.8	22.4	611.1	7.8
354U1450A_70F-Spot 300	97	54432	3.0	16.2263	2.1	0.8563	2.6	0.1008	1.5	0.58	618.9	8.7	628.1	12.0	661.3	44.7	618.9	8.7
354U1450A_70F-Spot 269	805	973968	1.6	16.5733	0.8	0.8425	1.6	0.1013	1.4	0.88	621.9	8.4	620.5	7.5	615.7	16.8	621.9	8.4
354U1450A_70F-Spot 227	231	234426	2.8	16.4146	1.5	0.8812	2.0	0.1049	1.3	0.66	643.1	8.2	641.6	9.7	636.5	33.2	643.1	8.2
354U1450A_70F-Spot 243	129	71715	7.4	16.0505	1.9	0.9271	3.0	0.1079	2.3	0.78	660.7	14.6	666.1	14.6	684.6	40.1	660.7	14.6
354U1450A_70F-Spot 17	405	54963	1.9	15.9117	1.2	1.0083	1.7	0.1164	1.2	0.71	709.6	8.2	708.0	8.8	703.1	26.0	709.6	8.2
354U1450A_70F-Spot 111	114	142843	2.1	15.8443	1.5	1.0203	2.4	0.1173	1.8	0.77	714.7	12.2	714.1	12.1	712.1	32.2	714.7	12.2
354U1450A_70F-Spot 75	863	230179	37.5	14.8073	0.9	1.1113	2.7	0.1193	2.5	0.94	726.8	17.3	758.8	14.4	854.3	19.5	726.8	17.3
354U1450A_70F-Spot 224	120	60275	3.7	15.8922	1.2	1.0364	1.9	0.1195	1.4	0.78	727.4	9.9	722.1	9.6	705.7	24.9	727.4	9.9
354U1450A_70F-Spot 303	228	99739	2.2	15.7673	1.2	1.0827	2.0	0.1238	1.6	0.80	752.5	11.4	744.9	10.7	722.4	25.9	752.5	11.4
354U1450A_70F-Spot 198	841	192950	0.5	14.9485	0.7	1.1475	1.3	0.1244	1.1	0.86	755.9	8.0	776.1	7.1	834.5	13.9	755.9	8.0
354U1450A_70F-Spot 310	391	147373	2.2	14.5357	1.1	1.1913	1.6	0.1256	1.1	0.73	762.7	8.2	796.6	8.7	892.6	22.4	762.7	8.2
354U1450A_70F-Spot 302	392	187469	3.6	14.8253	1.1	1.1696	2.0	0.1258	1.6	0.83	763.6	11.9	786.4	10.9	851.8	23.0	763.6	11.9
354U1450A_70F-Spot 7	248	50958	0.6	15.3194	1.1	1.1409	1.6	0.1268	1.2	0.75	769.3	8.9	772.9	8.9	783.2	23.0	769.3	8.9
354U1450A_70F-Spot 136	1193	554380	2.4	14.7864	1.0	1.2017	1.6	0.1289	1.3	0.81	781.4	9.9	801.4	9.1	857.2	19.9	781.4	9.9
354U1450A_70F-Spot 76	314	146833	2.3	14.3047	1.6	1.2768	3.2	0.1325	2.7	0.86	801.9	20.5	835.4	18.0	925.6	33.1	801.9	20.5
354U1450A_70F-Spot 140	269	77906	3.3	15.0135	0.9	1.2243	1.4	0.1333	1.1	0.79	806.8	8.7	811.8	8.1	825.5	18.3	806.8	8.7
354U1450A_70F-Spot 259	458	54785	1.2	14.9215	1.1	1.2506	1.8	0.1353	1.5	0.81	818.2	11.4	823.7	10.3	838.3	22.3	818.2	11.4
354U1450A_70F-Spot 290	199	9544485	2.3	15.0866	1.2	1.2426	2.1	0.1360	1.7	0.82	821.8	13.2	820.1	11.8	815.4	25.1	821.8	13.2
354U1450A_70F-Spot 152	1311	134467	1.3	14.9764	1.0	1.2620	2.0	0.1371	1.8	0.88	828.1	14.0	828.8	11.6	830.6	20.1	828.1	14.0
354U1450A_70F-Spot 157	37	182556	0.8	15.1367	1.9	1.2676	2.5	0.1392	1.6	0.64	839.9	12.7	831.3	14.4	808.4	40.8	839.9	12.7
354U1450A_70F-Spot 214	1136	189784	4.8	14.6038	0.8	1.3379	1.6	0.1417	1.4	0.88	854.3	11.2	862.3	9.3	883.0	15.7	854.3	11.2
354U1450A_70F-Spot 142	262	61419	7.2	14.5362	1.1	1.3769	1.8	0.1452	1.5	0.81	873.8	12.0	879.1	10.7	892.6	22.2	873.8	12.0
354U1450A_70F-Spot 277	221	201544	1.5	14.7099	1.0	1.3608	1.8	0.1452	1.4	0.81	873.9	11.8	872.2	10.4	868.0	21.4	873.9	11.8
354U1450A_70F-Spot 307	141	56958	3.3	14.7134	1.3	1.3649	2.0	0.1457	1.5	0.77	876.5	12.7	874.0	11.7	867.5	26.1	876.5	12.7
354U1450A_70F-Spot 199	208	196520	2.0	14.4486	1.4	1.4286	2.1	0.1497	1.6	0.74	899.3	13.3	901.0	12.7	905.0	29.6	905.0	29.6
354U1450A_70F-Spot 114	112	73871	2.2	14.3585	1.2	1.4453	1.7	0.1505	1.3	0.74	903.8	10.8	907.9	10.4	917.9	24.0	917.9	24.0
354U1450A_70F-Spot 282	119	151060	3.2	14.1994	1.4	1.5799	2.0	0.1627	1.4	0.71	971.8	12.6	962.3	12.3	940.8	28.7	940.8	28.7
354U1450A_70F-Spot 143	336	67936	13.6	14.1897	1.4	1.4734	2.1	0.1516	1.5	0.74	910.1	13.0	919.5	12.5	942.2	28.7	942.2	28.7
354U1450A_70F-Spot 261	862	76084	26.0	14.1450	0.9	1.4555	1.8	0.1493	1.5	0.85	897.1	12.8	912.1	10.8	948.7	19.4	948.7	19.4
354U1450A_70F-Spot 53	119	71158	2.8	14.0799	1.3	1.4546	1.9	0.1485	1.5	0.75	892.8	12.1	911.8	11.7	958.1	26.2	958.1	26.2
354U1450A_70F-Spot 286	127	32390	1.8	14.0777	1.2	1.5004	1.8	0.1532	1.3	0.74	918.9	11.5	930.6	11.1	958.4	24.9	958.4	24.9
354U1450A_70F-Spot 98	395	74178	6.1	14.0733	0.8	1.5535	1.8	0.1586	1.6	0.90	948.8	14.5	951.9	11.2	959.0	16.1	959.0	16.1
354U1450A_70F-Spot 284	48	186746	2.8	14.0505	1.6	1.6344	2.1	0.1666	1.3	0.64	993.1	12.4	983.6	13.3	962.4	33.2	962.4	33.2
354U1450A_70F-Spot 164	484	60806	1.5	14.0466	0.9	1.5627	1.5	0.1592	1.2	0.79	952.3	10.5	955.5	9.2	962.9	18.5	962.9	18.5
354U1450A_70F-Spot 181	307	220454	0.9	14.0447	1.0	1.5490	1.7	0.1578	1.4	0.81	944.5	11.9	950.1	10.2	963.2	19.7	963.2	19.7
354U1450A_70F-Spot 204	62	162117	1.9	14.0379	1.9	1.6413	2.4	0.1671	1.4	0.60	996.1	13.2	986.2	15.0	964.2	38.8	964.2	38.8
354U1450A_70F-Spot 218	99	48029	1.6	14.0300	1.3	1.6591	2.1	0.1688	1.6	0.78	1005.6	15.3	993.0	13.4	965.4	27.3	965.4	27.3
354U1450A_70F-Spot 276	241	132124	1.9	14.0274	1.0	1.4843	1.6	0.1510	1.2	0.79	906.6	10.3	924.0	9.4	965.7	19.6	965.7	19.6
354U1450A_70F-Spot 119	554	310221	2.7	13.9957	1.0	1.5739	1.7	0.1598	1.3	0.81	955.5	11.9	960.0	10.3	970.3	19.9	970.3	19.9
354U1450A_70F-Spot 147	141	104762	1.8	13.9910	1.2	1.6038	1.8	0.1627	1.3	0.73	972.0	11.9	971.7	11.3	971.0	25.2	971.0	25.2
354U1450A_70F-Spot 250	451	73786	16.5	13.9830	1.0	1.6000	1.7	0.1623	1.3	0.80	969.3	12.0	970.2	10.3	972.2	20.1	972.2	20.1
354U1450A_70F-Spot 125	74	83249	1.2	13.9789	1.4	1.6431	2.2	0.1666	1.7	0.76	993.3	15.3	986.9	13.8	972.8	29.0	972.8	29.0
354U1450A_70F-Spot 104	400	69367	2.0	13.9083	1.1	1.5500	2.1	0.1564	1.8	0.86	936.5	15.7	950.5	13.0	983.1	22.1	983.1	22.1
354U1450A_70F-Spot 32	1040	343853	5.0	13.8451	1.1	1.6552	2.2	0.1662	1.9	0.87	991.1	17.3	991.5	13.7	992.3	21.7	992.3	21.7
354U1450A_70F-Spot 28	182	55819	2.2	13.8439	1.2	1.6455	1.9	0.1652	1.4	0.76	985.7	12.8	987.8	11.7	992.6	24.6	992.6	24.6
354U1450A_70F-Spot 92	506	89751	2.4	13.8252	1.0	1.6700	1.8	0.1675	1.5	0.83	998.1	13.5	997.2	11.2	995.3	20.1	995.3	20.1
354U1450A_70F-Spot 96	136	234933	1.5	13.8120	1.2	1.7315	1.9	0.1735	1.5	0.76	1031.1	13.9	1020.3	12.4	997.2	25.3	997.2	25.3
354U1450A_70F-Spot 109	79	49051	1.9	13.7824	1.5	1.6533	2.1	0.1653	1.5	0.72	986.0	14.0	990.8	13.5	1001.6	30.1	1001.6	30.1
354U1450A_70F-Spot 54	266	165503	1.7	13.7404	0.9	1.6781	1.5	0.1672	1.2	0.80	996.8	11.4	1000.3	9.8	1007.8	18.6	1007.8	18.6
354U1450A_70F-Spot 47	162	61585	1.5	13.6935	1.5	1.6696	2.2	0.1658	1.6	0.73	989.0	14.7	997.0	13.9	1014.7	30.4	1014.7	30.4
354U1450A_70F-Spot 229	945	565815	3.0	13.6681	0.9	1.7063	1.7	0.1691	1.4	0.84	1007.4	12.9	1010.9	10.6	1018.5	18.4	1018.5	18.4
354U1450A_70F-Spot 251	207	135233	2.0	13.5661	1.3	1.6301	2.1	0.1604	1.6	0.78	958.9	14.5	981.9	13.2	1033.6	26.8	1033.6	26.8
354U1450A_70F-Spot 84	2233	1159383	6.9	13.5093	0.9	1.4247	2.2	0.1396	2.0	0.91	842.4	15.7	899.3	13.1	1042.1	18.8	1042.1	18.8
354U1450A_70F-Spot 68	227	243091	2.8	13.4848	1.2	1.7268	2.0	0.1689	1.6	0.81	1006.0	14.9	1018.6	12.6	1045.8	23.2	1045.8	23.2
354U1450A_70F-Spot 298	389	637707	5.0	13.4451	0.8	1.8864	1.6	0.1839	1.3	0.85	1088.5	13.3	1076.3	10.4	1051.7	16.7	1051.7	16.7
354U1450A_70F-Spot 118	155	112296	1.0	13.4278	1.2	1.7275	2.0	0.1682	1.5	0.79	1002.4	14.3	1018.8	12.6	1054.3	24.5	1054.3	24.5
354U1450A_70F-Spot 285	95	67191	1.1	13.4025	1.9	1.8550	3.5	0.1803	2.9	0.83	1068.7	28.5	1065.2	22.9	1058.1	38.6	1058.1	38.6
354U1450A_70F-Spot 278	304	220770	2.4	13.3809	0.9	1.7434	1.5	0.1692	1.2	0.81	1007.7	11.3	1024.7	9.6	1061.4	17.3	1061.4	17.3
354U1450A_70F-Spot 144	135	3239775	1.7	13.3492	0.9	1.8527	1.6	0.1794	1.3	0.83	1063.5	12.8	1064.4	10.4	1066.1	17.8	1066.1	17.8
354U1450A_70F-Spot 253	696	98149	2.6	13.3426	0.7	1.8091	1.4	0.1751	1.2	0.86	1039.9	12.0	1048.7	9.4	1067.1	14.7	1067.1	14.7
354U1450A_70F-Spot 179	868	120709	5.0	13.2548	1.6	1.7140	3.8	0.1648	3.5	0.91	983.2	31.9	1013.8	24.6	1080.4	31.5	1080.4	31.5
354U1450A_70F-Spot 295	196	163701	1.7	13.2434	1.1	1.9087	1.8	0.1833	1.4	0.80	1085.2	14.3	1084.1	11.9	1082.1	21.8	1082.1	21.8
354U1450A_70F-Spot 176	228	111861	1.0	13.2228	1.0	1.8597	1.6	0.1783	1.3	0.78	1057.9	12.4	1066.9	10.9	1085.2	20.8	1085.2	20.8

354U1450A_70F-Spot 170	228	57859	5.4	13.1850	0.8	1.9353	1.5	0.1851	1.2	0.84	1094.6	12.5	1093.4	9.9	1090.9	16.3	1090.9	16.3
354U1450A_70F-Spot 40	129	85895	3.2	13.0276	1.1	1.7843	1.7	0.1686	1.3	0.77	1004.3	12.3	1039.8	11.2	1114.9	21.8	1114.9	21.8
354U1450A_70F-Spot 190	345	116813	4.5	12.9434	1.1	1.7956	1.6	0.1686	1.2	0.76	1004.2	11.6	1043.9	10.7	1127.9	21.2	1127.9	21.2
354U1450A_70F-Spot 44	108	55229	1.8	12.9278	1.5	1.9128	2.1	0.1793	1.4	0.70	1063.4	14.2	1085.6	13.8	1130.3	29.4	1130.3	29.4
354U1450A_70F-Spot 189	68	94134	1.2	12.8700	1.3	2.0910	2.2	0.1952	1.7	0.80	1149.4	18.0	1145.8	14.8	1139.2	25.9	1139.2	25.9
354U1450A_70F-Spot 193	117	192169	0.8	12.8507	1.2	2.1030	1.7	0.1960	1.2	0.73	1153.8	13.1	1149.8	11.7	1142.2	23.2	1142.2	23.2
354U1450A_70F-Spot 267	62	193159	1.2	12.8256	1.4	2.1302	2.2	0.1982	1.7	0.77	1165.4	18.2	1158.7	15.3	1146.1	27.8	1146.1	27.8
354U1450A_70F-Spot 165	710	72119	3.2	12.7693	1.0	2.0997	1.8	0.1945	1.5	0.85	1145.5	16.2	1148.7	12.5	1154.8	19.1	1154.8	19.1
354U1450A_70F-Spot 88	101	192414	0.8	12.7022	1.2	2.1311	1.7	0.1963	1.2	0.70	1155.6	12.7	1159.0	11.9	1165.2	24.5	1165.2	24.5
354U1450A_70F-Spot 289	91	57988	0.9	12.6892	1.1	2.1381	1.8	0.1968	1.4	0.77	1157.9	14.4	1161.2	12.2	1167.3	22.1	1167.3	22.1
354U1450A_70F-Spot 129	202	76891	1.9	12.6500	1.1	2.1740	1.7	0.1995	1.3	0.75	1172.4	13.6	1172.8	11.7	1173.4	21.9	1173.4	21.9
354U1450A_70F-Spot 188	370	196283	2.6	12.5260	0.7	2.0026	1.6	0.1819	1.4	0.90	1077.5	14.0	1116.4	10.7	1192.9	13.6	1192.9	13.6
354U1450A_70F-Spot 87	521	775438	1.2	12.5049	1.0	2.1514	1.9	0.1951	1.6	0.84	1149.1	17.3	1165.5	13.5	1196.2	20.7	1196.2	20.7
354U1450A_70F-Spot 90	214	47653	3.9	12.4841	2.5	1.9176	2.9	0.1736	1.4	0.48	1032.1	13.2	1087.2	19.3	1199.5	50.1	1199.5	50.1
354U1450A_70F-Spot 74	558	104309	2.2	12.3875	0.8	2.0687	1.4	0.1859	1.2	0.83	1098.9	11.9	1138.5	9.6	1214.8	15.3	1214.8	15.3
354U1450A_70F-Spot 131	168	135468	1.6	12.3629	1.2	2.3729	1.7	0.2128	1.3	0.74	1243.5	14.4	1234.5	12.3	1218.7	22.6	1218.7	22.6
354U1450A_70F-Spot 41	1242	1978319	5.6	12.1582	1.0	2.1866	1.7	0.1928	1.4	0.82	1136.6	14.1	1176.8	11.6	1251.5	18.7	1251.5	18.7
354U1450A_70F-Spot 23	156	219349	1.2	12.0493	1.1	2.4678	1.6	0.2157	1.2	0.75	1258.9	13.7	1262.6	11.6	1269.0	20.8	1269.0	20.8
354U1450A_70F-Spot 293	227	100648	0.7	12.0439	0.9	2.4500	1.5	0.2140	1.2	0.81	1250.1	13.9	1257.4	10.9	1269.9	17.3	1269.9	17.3
354U1450A_70F-Spot 139	118	86507	1.4	11.9823	1.1	2.4989	2.0	0.2172	1.7	0.84	1266.9	19.1	1271.7	14.4	1279.9	21.3	1279.9	21.3
354U1450A_70F-Spot 4	175	898572	1.6	11.8298	1.1	2.5355	1.8	0.2175	1.4	0.77	1268.9	16.2	1282.3	13.2	1304.8	22.2	1304.8	22.2
354U1450A_70F-Spot 12	560	72878	1.7	11.7715	0.9	2.4896	1.8	0.2125	1.6	0.87	1242.4	18.2	1269.0	13.4	1314.4	17.5	1314.4	17.5
354U1450A_70F-Spot 182	298	88136	3.5	11.6335	1.0	2.7110	1.8	0.2287	1.4	0.81	1327.9	17.3	1331.5	13.1	1337.2	19.9	1337.2	19.9
354U1450A_70F-Spot 305	1866	169593	6.3	11.5606	1.0	2.7090	2.1	0.2271	1.8	0.88	1319.5	21.8	1330.9	15.4	1349.4	18.8	1349.4	18.8
354U1450A_70F-Spot 57	593	387068	4.5	11.5478	1.3	2.3353	2.5	0.1956	2.1	0.86	1151.6	22.6	1223.1	17.7	1351.5	24.5	1351.5	24.5
354U1450A_70F-Spot 124	198	95497	7.5	11.4230	1.1	2.6917	1.9	0.2230	1.5	0.80	1297.7	17.6	1326.2	13.8	1372.4	21.5	1372.4	21.5
354U1450A_70F-Spot 108	310	241559	3.5	11.4173	0.9	2.8926	2.0	0.2395	1.7	0.88	1384.2	21.6	1380.0	14.9	1373.4	18.2	1373.4	18.2
354U1450A_70F-Spot 215	174	80091	3.8	11.3224	1.1	2.9420	2.0	0.2416	1.6	0.81	1395.0	19.9	1392.8	14.8	1389.4	21.9	1389.4	21.9
354U1450A_70F-Spot 15	1102	171864	5.8	11.2193	0.8	2.9075	1.9	0.2366	1.7	0.91	1368.9	21.0	1383.9	14.1	1407.0	15.0	1407.0	15.0
354U1450A_70F-Spot 169	321	1669593	4.3	11.2162	0.9	2.9399	2.0	0.2391	1.8	0.89	1382.3	22.7	1392.2	15.5	1407.5	17.7	1407.5	17.7
354U1450A_70F-Spot 91	195	143363	1.7	11.0605	1.2	2.8825	2.5	0.2312	2.2	0.87	1340.9	26.6	1377.3	19.0	1434.2	23.3	1434.2	23.3
354U1450A_70F-Spot 281	340	596577	2.4	10.8945	0.9	3.1900	1.4	0.2521	1.1	0.77	1449.1	14.2	1454.7	11.0	1463.0	17.2	1463.0	17.2
354U1450A_70F-Spot 27	62	35567	1.4	10.8246	1.3	3.2148	2.5	0.2524	2.1	0.85	1450.8	27.8	1460.7	19.5	1475.2	25.1	1475.2	25.1
354U1450A_70F-Spot 128	126	736728	5.8	10.7861	0.9	2.6108	1.7	0.2042	1.4	0.84	1198.0	15.8	1303.7	12.7	1482.0	17.9	1482.0	17.9
354U1450A_70F-Spot 247	225	196771	1.4	10.7585	1.0	3.3368	1.5	0.2604	1.1	0.76	1491.7	15.2	1489.7	11.7	1486.8	18.5	1486.8	18.5
354U1450A_70F-Spot 258	164	52298	1.0	10.5666	0.9	3.3608	1.8	0.2576	1.5	0.86	1477.4	20.3	1495.3	14.1	1520.9	17.5	1520.9	17.5
354U1450A_70F-Spot 185	84	195390	2.4	10.4745	1.0	3.1219	1.6	0.2372	1.3	0.78	1371.9	15.5	1438.1	12.4	1537.3	19.0	1537.3	19.0
354U1450A_70F-Spot 138	420	5347505	2.5	10.4007	0.7	3.3588	1.7	0.2534	1.6	0.92	1455.8	20.3	1494.8	13.2	1550.6	12.5	1550.6	12.5
354U1450A_70F-Spot 6	728	98571	2.6	10.3680	1.2	3.4096	2.2	0.2564	1.9	0.85	1471.3	24.8	1506.6	17.5	1556.6	22.2	1556.6	22.2
354U1450A_70F-Spot 45	122	87770	5.5	10.3062	1.1	3.0039	1.9	0.2245	1.5	0.82	1305.8	18.2	1408.6	14.4	1567.8	20.4	1567.8	20.4
354U1450A_70F-Spot 280	275	1890732	2.4	10.1953	1.0	3.6770	1.7	0.2719	1.4	0.82	1550.4	19.3	1566.4	13.6	1588.0	18.1	1588.0	18.1
354U1450A_70F-Spot 101	486	86988	150.5	10.1836	0.9	3.3075	1.4	0.2443	1.0	0.75	1408.9	13.2	1482.8	10.8	1590.1	17.1	1590.1	17.1
354U1450A_70F-Spot 113	668	82217	2.0	10.1620	0.8	3.7740	1.8	0.2782	1.6	0.90	1582.0	22.8	1587.2	14.5	1594.1	14.4	1594.1	14.4
354U1450A_70F-Spot 266	46	51629	1.2	10.1167	1.4	3.9605	2.0	0.2906	1.5	0.74	1644.5	22.0	1626.1	16.7	1602.5	26.1	1602.5	26.1
354U1450A_70F-Spot 308	152	92976	2.0	10.1068	0.8	3.7205	1.5	0.2727	1.2	0.83	1554.6	17.1	1575.8	11.9	1604.3	15.6	1604.3	15.6
354U1450A_70F-Spot 51	1211	698241	1.9	10.0970	0.6	3.5807	1.8	0.2622	1.7	0.94	1501.2	22.9	1545.3	14.5	1606.1	12.0	1606.1	12.0
354U1450A_70F-Spot 200	641	75244	2.0	10.0713	1.0	3.5205	1.7	0.2572	1.3	0.79	1475.3	17.6	1531.8	13.4	1610.8	19.4	1610.8	19.4
354U1450A_70F-Spot 180	779	462013	2.7	10.0641	0.9	3.5381	1.8	0.2583	1.6	0.87	1480.9	20.5	1535.8	14.1	1612.2	16.4	1612.2	16.4
354U1450A_70F-Spot 168	1098	387375	16.8	10.0600	0.8	3.5269	1.5	0.2573	1.3	0.85	1476.2	16.9	1533.3	12.0	1612.9	15.0	1612.9	15.0
354U1450A_70F-Spot 275	269	140799	1.5	10.0137	1.1	3.4794	2.2	0.2527	1.9	0.86	1452.4	24.9	1522.6	17.5	1621.5	21.1	1621.5	21.1
354U1450A_70F-Spot 66	343	223667	0.7	9.9297	0.7	3.7325	1.3	0.2688	1.1	0.82	1534.7	14.4	1578.4	10.3	1637.2	13.5	1637.2	13.5
354U1450A_70F-Spot 226	179	50304	1.7	9.5551	0.8	4.5357	1.4	0.3143	1.2	0.85	1761.9	18.6	1737.5	11.9	1708.3	14.0	1708.3	14.0
354U1450A_70F-Spot 268	163	207376	2.4	9.5221	1.1	4.5760	1.8	0.3160	1.5	0.82	1770.3	23.2	1744.9	15.3	1714.6	19.5	1714.6	19.5
354U1450A_70F-Spot 30	293	87486	9.0	9.5209	0.8	4.2550	1.4	0.2938	1.1	0.80	1660.6	16.1	1684.7	11.4	1714.9	15.4	1714.9	15.4
354U1450A_70F-Spot 63	132	134650	3.6	9.5170	1.2	4.2750	2.1	0.2951	1.7	0.81	1666.8	24.9	1688.6	17.3	1715.6	22.9	1715.6	22.9
354U1450A_70F-Spot 203	186	195826	3.3	9.5033	1.1	4.3641	1.8	0.3008	1.5	0.81	1695.2	22.1	1705.6	15.1	1718.3	19.8	1718.3	19.8
354U1450A_70F-Spot 301	47	484619	0.8	9.4980	1.3	4.1719	1.9	0.2874	1.4	0.75	1628.4	20.4	1668.5	15.5	1719.3	23.2	1719.3	23.2
354U1450A_70F-Spot 191	220	695766	2.0	9.4184	0.8	4.3479	1.4	0.2970	1.1	0.79	1676.4	15.9	1702.5	11.3	1734.7	15.5	1734.7	15.5
354U1450A_70F-Spot 313	635	184817	1.9	9.3325	0.9	4.7887	1.8	0.3241	1.6	0.87	1809.9	25.1	1782.9	15.3	1751.5	16.5	1751.5	16.5
354U1450A_70F-Spot 145	168	195729	2.4	9.2683	1.1	4.6603	1.7	0.3133	1.4	0.79	1756.8	21.1	1760.1	14.5	1764.2	19.3	1764.2	19.3
354U1450A_70F-Spot 311	216	784312	2.4	9.2487	1.1	4.5841	2.1	0.3075	1.8	0.86	1728.4	27.1	1746.4	17.4	1768.0	19.7	1768.0	19.7
354U1450A_70F-Spot 35	1581	209930	15.2	9.2399	1.4	3.8674	2.1	0.2592	1.6	0.76	1485.6	21.3	1606.9	16.9	1769.8	24.7	1769.8	24.7
354U1450A_70F-Spot 238	83	230965	3.0	9.2204	0.9	4.8427	1.4	0.3238	1.0	0.76	1808.5	16.4	1792.4	11.6	1773.6	16.5	1773.6	16.5
354U1450A_70F-Spot 126	1102	426670	1.6	8.9580	0.7	4.5654	1.5	0.2966	1.3	0.89	1674.5	19.5	1743.0	12.3	1826.1	11.9	1826.1	11.9
354U1450A_70F-Spot 183	296	481842	2.2	8.8984	0.9	5.1324	1.8	0.3312	1.6	0.87	1844.4	25.3	1841.5	15.5	1838.2	16.6	1838.2	16.6
354U1450A_70F-Spot 262	103	55801	1.8	8.8910	0.9	5.1827	1.8	0.3342	1.5	0.87	1858.7	24.9	1849.8	15.0	1839.7	15.6	1839.7	15.6
354U1450A_70F-Spot 171	36	37906	1.6	8.8890	1.3	5.0741	2.0	0.3271	1.5	0.74	1824.4	23.3	1831.8	16.9	1840.2	24.4	1840.2	24.4
354U1450A_70F-Spot 80	231	240425	4.7	8.8182	1.0	5.0465	1.7	0.3228	1.4	0.81	1803.2	21.7	1827.2	14.4	1854.6	17.9	1854.6	17.9
354U1450A_70F-Spot 260	133	78035	1.5	8.7973	1.0	5.3699	1.5	0.3426	1.1	0.75	1899.3	18.4	1880.1	12.9	1858.9	18.1	1858.9	18.1
354U1450A_70F-Spot 94	789	400096	2.9	8.7892	0.8	4.8468	1.5	0.3090	1.2	0.83	1735.6	19.0	1793.1	12.7	1860.6	15.2	1860.6	15.2
354U1450A_70F-Spot 116	191	60818	2.2	8.7781	1.0	5.1496	1.9	0.3278	1.7	0.86	1827.9	26.6	1844.3	16.5	1862.9	17.6	1862.9	17.6
354U1450A_70F-Spot 69	67	45583	2.1	8.7595	1.0	5.3394	1.6	0.3392	1.3	0.81	1882.9	21.7	1875.2	14.1	1866.7	17.6	1866.7	17.6
354U1450A_70F-Spot 60	146	128357	1.9	8.7279	0.9	5.2105	1.6	0.3298	1.3	0.82	1837.5	20.9	1854.3	13.6	1873.2	16.5	1873.2	16.5
354U1450A_70F-Spot 149	230	70728	6.1	8.7234	0.8	5.0593	1.7	0.3201	1.5	0.86	1790.2	22.8	1829.3	14.3	1874.1	15.3	1874.1	15.3
354U1450A_70F-Spot 297	207	72153	8.1	8.2210	0.7	6.0464	1.4	0.3605	1.2	0.85	1984.6	19.8	1982.6	11.9	1980.4	12.7	1980.4	12.7
354U1450A_70F-Spot 315	240	53247	2.1	8.1788	0.8	6.0854	1.5	0.3610	1.3	0.84	1986.8	22.1	1988.2	13.4	1989.6	14.7	1989.6	14.7
354U1450A_70F-Spot 272	169	199394	3.1	8.1608	1.0	6.0089	1.8	0.3557	1.5	0.82	1961.5	24.6	1977.1	15.4	1993.5	17.9	1993.5	17.9
354U1450A_70F-Spot 239	652	753533	8.3	6.6140	0.9	8.0467	1.9	0.3860	1.7	0.88	2104.2	30.3	2236.3	17.3	2359.5	15.3	2359.5	15.3
354U1450A_70F-Spot 231	78	139059	1.3	6.3400	0.9	9.5905	2.1	0.4410	1.9	0.89	2355.1	36.8	2396.3	19.2	2431.4	15.8	2431.4	15.8
354U1450A_70F-Spot 283	139	82694	1.9	6.3292	1.0	9.0597	1.6	0.4159	1.2	0.78	2241.7	23.6	2344.0	14.6	2434.3	17.0	2434.3	17.0
354U1450A_70F-Spot 312	144	131857	2.1	6.2981	0.8	9.4038	1.8	0.4295	1.6	0.90	2303.7	31.6	2378.2	16.7	2442.7	13.4	2442.7	13.4
354U1450A_70F-Spot 18	131	224922	1.9	6.2431	0.8	8.8025	1.6	0.3986	1.5	0.89	2162.4	26.9	2317.7	15.0	2457.5	12.7	2457.5	12.7
354U1450A_70F-Spot 117	285	62998	3.0	6.2094	1.0	8.8524	1.7	0.3987	1.4	0.81	2162.9	24.9	2322.9	15.2	2466.7	16.5	2466.7	16.5
354U1450A_70F-Spot 67	270	142804	1.6	6.1944	0.9	10.1717	1.6	0.4570	1.4	0.85	2426.2	27.4	2450.5	14.8	2470.7	14.4	2470.7	14.4
354U1450A_70F-Spot 184	495	330458	2.5	6.1771	1.0	8.2760	1.8	0.3708	1.5	0.81	2033.0	25.4	2261.7	16.2	2475.5	17.5	2475.5	17.5
354U1450A_70F-Spot 21	616	342870	8.9	6.1113	1.0	9.7643	1.9	0.4328	1.6	0.86	2318.3	31.8	2412.8	17.4	2493.5	16.0	2493.5	16.0

354U1450A_70F-Spot 115	151	90275	2.5	6.0578	0.7	10.1748	1.4	0.4470	1.2	0.85	2382.0	23.2	2450.8	12.7	2508.3	12.3	2508.3	12.3
354U1450A_70F-Spot 79	98	1149424	2.2	5.6340	1.0	12.2087	1.5	0.4989	1.1	0.77	2608.9	24.3	2620.6	13.9	2629.6	15.8	2629.6	15.8
354U1450A_70F-Spot 162	121	224781	1.6	5.4714	0.9	12.9324	1.6	0.5132	1.3	0.81	2670.2	29.1	2674.7	15.4	2678.2	15.7	2678.2	15.7
354U1450A_70F-Spot 2	64	161720	2.2	5.0568	1.0	12.3367	1.7	0.4525	1.4	0.82	2406.2	28.2	2630.4	16.1	2807.7	16.2	2807.7	16.2
354U1450A_70F-Spot 244	230	78877	1.4	4.9923	0.9	12.1658	2.0	0.4405	1.8	0.89	2352.9	34.9	2617.3	18.7	2828.7	14.8	2828.7	14.8
354U1450A_70F-Spot 99	665	223722	7.9	4.8166	1.1	16.3599	2.2	0.5715	1.9	0.87	2914.0	44.1	2898.1	20.7	2887.0	17.5	2887.0	17.5
354U1450A_70F-Spot 148	341	551520	6.4	4.1713	0.8	17.5252	1.3	0.5302	1.0	0.81	2742.3	23.4	2964.0	12.5	3118.2	12.3	3118.2	12.3
354U1450A_70F-Spot 186	392	185876	3.0	4.0252	0.9	20.3766	1.7	0.5949	1.5	0.86	3009.1	35.0	3109.4	16.4	3174.8	13.7	3174.8	13.7

Table ______. U-Pb geochronologic analyses.																		
#11 U1450A_78,79,80F						Isotope ratios					Apparent ages (Ma)							
Late Pliocene 3.2 Ma																		
Analysis	U (ppm)	206Pb 204Pb	U/Th	206Pb* 207Pb*	± (%)	207Pb* 235U*	± (%)	206Pb* 238U	± (%)	error corr.	206Pb* 238U*	± (Ma)	207Pb* 235U	± (Ma)	206Pb* 207Pb*	± (Ma)	Best age (Ma)	± (Ma)
Gleason-354-U1450A-78,80F_bagged-Spot 261	1914	13570	3.3	21.6434	1.0	0.0255	1.8	0.0040	1.6	0.85	25.7	0.4	25.5	0.5	8.2	23.6	25.7	0.4
Gleason-354-U1450A-78,80F_bagged-Spot 139	1144	4579	41.8	21.8411	1.4	0.0253	3.0	0.0040	2.7	0.88	25.7	0.7	25.3	0.8	13.7	34.4	25.7	0.7
Gleason-354-U1450A-78,80F_bagged-Spot 192	43	591	1.2	27.5894	25.1	0.0213	25.4	0.0043	3.9	0.15	27.5	1.1	21.4	5.4	612.3	694.1	27.5	1.1
Gleason-354-U1450A-78,80F_bagged-Spot 116	1682	197946	1.2	21.2731	1.3	0.0297	2.0	0.0046	1.6	0.78	29.5	0.5	29.7	0.6	49.6	30.4	29.5	0.5
Gleason-354-U1450A-78,80F_bagged-Spot 72	128	2448	1.1	24.1139	3.4	0.0302	4.5	0.0053	2.9	0.65	34.0	1.0	30.2	1.3	258.6	86.6	34.0	1.0
Gleason-354-U1450A-78,80F_bagged-Spot 171	5543	20169	7.6	21.5773	0.7	0.0347	5.7	0.0054	5.6	0.99	34.9	2.0	34.7	1.9	15.6	17.6	34.9	2.0
Gleason-354-U1450A-78,80F_bagged-Spot 238	555	168168	1.8	20.7592	1.4	0.0364	2.5	0.0055	2.0	0.82	35.2	0.7	36.3	0.9	107.7	33.3	35.2	0.7
Gleason-354-U1450A-78,80F_bagged-Spot 199	7849	34306	4.5	20.9335	0.8	0.0460	7.6	0.0070	7.5	0.99	44.8	3.4	45.6	3.4	87.9	17.9	44.8	3.4
Gleason-354-U1450A-78,80F_bagged-Spot 120	2082	58897	3.1	21.0694	0.8	0.0473	1.8	0.0072	1.6	0.88	46.4	0.7	46.9	0.8	72.6	19.6	46.4	0.7
Gleason-354-U1450A-78,80F_bagged-Spot 258	2094	90925	1.2	21.2099	0.9	0.0484	2.1	0.0074	1.9	0.90	47.8	0.9	48.0	1.0	56.7	21.3	47.8	0.9
Gleason-354-U1450A-78,80F_bagged-Spot 37	783	34603	1.4	21.7867	0.9	0.0474	2.0	0.0075	1.8	0.88	48.1	0.8	47.0	0.9	7.6	22.8	48.1	0.8
Gleason-354-U1450A-78,80F_bagged-Spot 47	199	12664	1.0	21.2802	2.3	0.0489	3.0	0.0075	1.9	0.64	48.5	0.9	48.5	1.4	48.8	55.0	48.5	0.9
Gleason-354-U1450A-78,80F_bagged-Spot 17	49	2350	1.7	22.5655	4.1	0.0473	5.0	0.0077	2.9	0.58	49.7	1.4	46.9	2.3	93.1	101.0	49.7	1.4
Gleason-354-U1450A-78,80F_bagged-Spot 60	102	7556	1.3	18.6824	3.6	0.0574	4.5	0.0078	2.7	0.61	49.9	1.4	56.6	2.5	351.2	80.9	49.9	1.4
Gleason-354-U1450A-78,80F_bagged-Spot 23	122	2452	1.4	22.6745	2.9	0.0478	3.8	0.0079	2.4	0.64	50.5	1.2	47.4	1.7	105.0	71.0	50.5	1.2
Gleason-354-U1450A-78,80F_bagged-Spot 267	357	7406	4.2	21.6152	1.5	0.0515	2.6	0.0081	2.2	0.83	51.8	1.1	51.0	1.3	11.4	34.9	51.8	1.1
Gleason-354-U1450A-78,80F_bagged-Spot 25	74	2275	0.8	19.6325	4.6	0.0569	5.7	0.0081	3.4	0.59	52.0	1.7	56.2	3.1	237.9	105.6	52.0	1.7
Gleason-354-U1450A-78,80F_bagged-Spot 75	500	22038	1.1	21.5830	1.6	0.0529	2.2	0.0083	1.5	0.70	53.2	0.8	52.4	1.1	15.0	37.7	53.2	0.8
Gleason-354-U1450A-78,80F_bagged-Spot 42	347	3735	1.1	22.9185	2.5	0.0508	3.2	0.0084	2.0	0.63	54.2	1.1	50.3	1.6	131.3	61.2	54.2	1.1
Gleason-354-U1450A-78,80F_bagged-Spot 226	503	9888	13.6	20.1130	1.5	0.0588	3.5	0.0086	3.2	0.90	55.0	1.7	58.0	2.0	181.9	35.1	55.0	1.7
Gleason-354-U1450A-78,80F_bagged-Spot 269	547	7375	2.1	21.4417	1.7	0.0552	3.0	0.0086	2.5	0.82	55.1	1.4	54.6	1.6	30.7	41.9	55.1	1.4
Gleason-354-U1450A-78,80F_bagged-Spot 32	590	6252	7.5	22.0131	2.9	0.0556	3.8	0.0089	2.5	0.66	57.0	1.4	54.9	2.0	32.6	69.9	57.0	1.4
Gleason-354-U1450A-78,80F_bagged-Spot 112	225	7582	1.5	19.7625	3.0	0.0646	3.9	0.0093	2.5	0.63	59.5	1.5	63.6	2.4	222.7	70.1	59.5	1.5
Gleason-354-U1450A-78,80F_bagged-Spot 257	1828	23056	4.0	21.0995	0.9	0.0618	2.2	0.0095	2.0	0.91	60.7	1.2	60.9	1.3	69.2	21.1	60.7	1.2
Gleason-354-U1450A-78,80F_bagged-Spot 175	1805	273049	3.0	20.8497	1.1	0.0633	2.3	0.0096	2.1	0.89	61.4	1.3	62.3	1.4	97.4	24.9	61.4	1.3
Gleason-354-U1450A-78,80F_bagged-Spot 20	217	60376	2.4	20.2935	1.6	0.0683	3.0	0.0101	2.6	0.85	64.5	1.7	67.1	2.0	161.0	36.9	64.5	1.7
Gleason-354-U1450A-78,80F_bagged-Spot 245	1922	78177	12.8	21.1166	0.8	0.0661	2.2	0.0101	2.1	0.93	64.9	1.3	65.0	1.4	67.2	19.2	64.9	1.3
Gleason-354-U1450A-78,80F_bagged-Spot 291	237	11079	0.9	21.3618	1.8	0.0699	2.8	0.0108	2.1	0.77	69.5	1.5	68.6	1.8	39.7	42.4	69.5	1.5
Gleason-354-U1450A-78,80F_bagged-Spot 46	1635	72176	1.4	20.9290	0.9	0.0810	1.8	0.0123	1.5	0.86	78.8	1.2	79.1	1.3	88.4	21.5	78.8	1.2
Gleason-354-U1450A-78,80F_bagged-Spot 234	61	29117	1.2	20.3574	2.5	0.0925	3.5	0.0137	2.5	0.71	87.5	2.1	89.9	3.0	153.7	57.7	87.5	2.1
Gleason-354-U1450A-78,80F_bagged-Spot 4	5	326	17.0	3.0168	8.4	0.6296	12.7	0.0138	9.5	0.75	88.2	8.3	495.9	49.8	3623.9	129.7	88.2	8.3
Gleason-354-U1450A-78,80F_bagged-Spot 64	105	4421	1.3	22.5470	3.2	0.0852	3.9	0.0139	2.1	0.54	89.2	1.8	83.0	3.1	91.1	79.7	89.2	1.8
Gleason-354-U1450A-78,80F_bagged-Spot 241	37	1786	1.4	24.2352	4.5	0.0807	5.5	0.0142	3.1	0.57	90.8	2.8	78.8	4.2	271.3	114.6	90.8	2.8
Gleason-354-U1450A-78,80F_bagged-Spot 237	51	20408	1.9	16.5620	3.7	0.1273	5.2	0.0153	3.7	0.71	97.8	3.6	121.6	5.9	617.2	78.8	97.8	3.6
Gleason-354-U1450A-78,80F_bagged-Spot 73	122	12583	1.8	20.6949	1.7	0.1033	2.8	0.0155	2.3	0.80	99.2	2.2	99.8	2.7	115.0	39.9	99.2	2.2
Gleason-354-U1450A-78,80F_bagged-Spot 268	457	6590	0.8	21.4860	0.9	0.0996	1.6	0.0155	1.4	0.85	99.3	1.4	96.4	1.5	25.8	20.7	99.3	1.4
Gleason-354-U1450A-78,80F_bagged-Spot 27	838	30225	0.9	20.6704	0.8	0.1145	1.7	0.0172	1.5	0.88	109.7	1.6	110.1	1.8	117.8	19.2	109.7	1.6
Gleason-354-U1450A-78,80F_bagged-Spot 299	182	2581	1.2	23.3730	6.0	0.1013	6.2	0.0172	1.7	0.28	109.8	1.9	98.0	5.8	180.1	149.8	109.8	1.9
Gleason-354-U1450A-78,80F_bagged-Spot 40	459	13398	3.2	20.7909	1.2	0.1157	2.4	0.0174	2.0	0.86	111.5	2.3	111.1	2.5	104.1	28.1	111.5	2.3
Gleason-354-U1450A-78,80F_bagged-Spot 14	374	39244	1.5	20.5349	1.2	0.1191	2.6	0.0177	2.2	0.88	113.4	2.5	114.3	2.8	133.3	29.0	113.4	2.5
Gleason-354-U1450A-78,80F_bagged-Spot 265	601	32479	3.4	20.5733	0.8	0.1315	1.8	0.0196	1.6	0.89	125.2	2.0	125.4	2.2	128.9	19.4	125.2	2.0
Gleason-354-U1450A-78,80F_bagged-Spot 109	86	44132	4.6	19.9188	1.8	0.1369	3.2	0.0198	2.7	0.83	126.3	3.3	130.3	3.9	204.5	41.1	126.3	3.3
Gleason-354-U1450A-78,80F_bagged-Spot 248	293	16562	2.1	21.0265	1.3	0.1310	2.1	0.0200	1.6	0.78	127.5	2.1	125.0	2.4	77.4	30.8	127.5	2.1
Gleason-354-U1450A-78,80F_bagged-Spot 200	535	45791	1.6	20.4889	1.0	0.1345	2.0	0.0200	1.8	0.86	127.6	2.2	128.1	2.5	138.6	24.5	127.6	2.2
Gleason-354-U1450A-78,80F_bagged-Spot 76	183	9613	1.3	20.7907	1.4	0.1338	2.6	0.0202	2.2	0.83	128.7	2.7	127.5	3.1	104.1	34.0	128.7	2.7
Gleason-354-U1450A-78,80F_bagged-Spot 215	171	11492	1.1	20.7689	1.8	0.1366	2.4	0.0206	1.6	0.67	131.3	2.1	130.0	2.9	106.6	41.9	131.3	2.1
Gleason-354-U1450A-78,80F_bagged-Spot 10	239	7439	1.6	21.1497	1.3	0.1350	2.5	0.0207	2.1	0.86	132.2	2.8	128.6	3.0	63.5	29.9	132.2	2.8
Gleason-354-U1450A-78,80F_bagged-Spot 243	154	448937	1.5	19.9829	1.4	0.1431	2.6	0.0207	2.2	0.84	132.3	2.9	135.8	3.3	197.0	33.3	132.3	2.9
Gleason-354-U1450A-78,80F_bagged-Spot 12	166	9951	1.8	18.5025	2.2	0.1551	3.3	0.0208	2.5	0.76	132.8	3.3	146.4	4.5	373.0	48.8	132.8	3.3
Gleason-354-U1450A-78,80F_bagged-Spot 88	283	7052	84.1	14.8845	1.9	0.2021	3.1	0.0218	2.4	0.79	139.2	3.3	186.9	5.3	843.5	39.7	139.2	3.3
Gleason-354-U1450A-78,80F_bagged-Spot 95	193	15414	4.2	19.4396	1.3	0.1686	3.3	0.0238	3.0	0.92	151.5	4.5	158.2	4.8	260.7	30.0	151.5	4.5
Gleason-354-U1450A-78,80F_bagged-Spot 150	1160	460	2.0	5.3615	2.5	0.7282	4.1	0.0283	3.2	0.79	180.0	5.7	555.5	17.4	2711.7	41.5	180.0	5.7
Gleason-354-U1450A-78,80F_bagged-Spot 82	390	24654	8.9	15.0354	1.1	0.2613	3.6	0.0285	3.4	0.96	181.1	6.2	235.7	7.6	822.5	22.0	181.1	6.2
Gleason-354-U1450A-78,80F_bagged-Spot 106	83	1044	0.5	8.9286	1.0	0.5453	3.0	0.0353	2.8	0.95	223.7	6.3	441.9	10.8	1832.1	17.8	223.7	6.3
Gleason-354-U1450A-78,80F_bagged-Spot 220	299	21227	0.6	19.8732	1.1	0.2485	2.4	0.0358	2.1	0.89	226.8	4.7	225.3	4.8	209.8	25.2	226.8	4.7
Gleason-354-U1450A-78,80F_bagged-Spot 168	208	13214	9.4	16.4143	1.5	0.3214	3.1	0.0383	2.7	0.88	242.0	6.5	283.0	7.6	636.5	31.4	242.0	6.5
Gleason-354-U1450A-78,80F_bagged-Spot 227	862	79478	4.2	15.3861	0.8	0.3470	2.4	0.0387	2.3	0.94	244.9	5.4	302.5	6.3	774.1	17.2	244.9	5.4
Gleason-354-U1450A-78,80F_bagged-Spot 137	789	44121	5.4	15.2981	1.7	0.4230	5.6	0.0469	5.3	0.95	295.7	15.4	358.2	16.9	786.2	36.0	295.7	15.4
Gleason-354-U1450A-78,80F_bagged-Spot 219	333	205708	1.3	18.9823	1.3	0.3551	3.2	0.0489	2.9	0.91	307.7	8.7	308.5	8.4	315.1	30.0	307.7	8.7
Gleason-354-U1450A-78,80F_bagged-Spot 93	15	209	0.6	2.8611	0.0	2.5784	4.5	0.0535	4.5	1.00	336.0	14.7	1294.5	32.9	3704.9	0.0	336.0	14.7
Gleason-354-U1450A-78,80F_bagged-Spot 262	692	889717	1.4	18.5326	0.8	0.4052	1.9	0.0545	1.8	0.92	341.9	5.9	345.4	5.6	369.3	17.1	341.9	5.9
Gleason-354-U1450A-78,80F_bagged-Spot 117	200	36517	1.8	17.4753	1.0	0.4552	3.1	0.0577	2.9	0.95	361.6	10.3	380.9	9.8	500.2	21.0	361.6	10.3
Gleason-354-U1450A-78,80F_bagged-Spot 178	359	50131	2.5	16.6055	0.8	0.5133	4.4	0.0618	4.4	0.98	386.6	16.4	420.6	15.3	611.5	18.2	386.6	16.4
Gleason-354-U1450A-78,80F_bagged-Spot 211	75	2182	0.5	14.9825	0.9	0.5867	3.6	0.0637	3.5	0.97	398.4	13.6	468.7	13.6	829.8	19.1	398.4	13.6
Gleason-354-U1450A-78,80F_bagged-Spot 157	46	21959	156.5	17.3423	1.6	0.5277	5.3	0.0664	5.0	0.95	414.3	20.2	430.3	18.6	516.9	35.7	414.3	20.2
Gleason-354-U1450A-78,80F_bagged-Spot 302	4212	227975	55.6	17.9434	0.5	0.5178	1.6	0.0674	1.5	0.94	420.4	6.0	423.7	5.5	441.6	12.1	420.4	6.0
Gleason-354-U1450A-78,80F_bagged-Spot 103	861	189324	7.6	17.5375	0.6	0.5304	1.8	0.0675	1.7	0.95	420.9	7.0	432.1	6.4	492.3	12.6	420.9	7.0
Gleason-354-U1450A-78,80F_bagged-Spot 101	948	95442	75.0	17.1965	0.8	0.5664	2.2	0.0706	2.0	0.92	440.0	8.5	455.7	7.9	535.5	18.2	440.0	8.5
Gleason-354-U1450A-78,80F_bagged-Spot 162	2236	488087	2.4	17.4783	0.6	0.5575	2.0	0.0707	1.9	0.96	440.2	8.2	449.9	7.3	499.8	13.0	440.2	8.2
Gleason-354-U1450A-78,80F_bagged-Spot 194	3823	298540	17.2	17.5708	0.8	0.5596	3.8	0.0713	3.7	0.98	444.1	15.9	451.3	13.8	488.2	16.9	444.1	15.9
Gleason-354-U1450A-78,80F_bagged-Spot 218	645	85549	1.3	17.6633	0.7	0.5617	2.0	0.0720	1.9	0.94	447.9	8.3	452.6	7.4	476.5	15.0	447.9	8.3
Gleason-354-U1450A-78,80F_bagged-Spot 128	1086	935148	1.0	17.2634	0.8	0.5765	2.1	0.0722	1.9	0.93	449.3	8.3	462.2	7.7	527.0	16.5	449.3	8.3
Gleason-354-U1450A-78,80F_bagged-Spot 121	4118	1148307	2.5	17.6322	0.5	0.5718	1.9	0.0731	1.8	0.96	454.9	8.1	459.2	7.1	480.4	11.8	454.9	8.1
Gleason-354-U1450A-78,80F_bagged-Spot 18	474	100452	9.3	17.5170	0.7	0.5908	1.6	0.0751	1.5	0.91	466.6	6.6	471.4	6.0	494.9	14.4	466.6	6.6
Gleason-354-U1450A-78,80F_bagged-Spot 256	1884	189304	0.8	17.2222	0.5	0.6021	1.7	0.0752	1.6	0.95	467.4	7.3	478.6	6.5	532.2	11.9	467.4	7.3
Gleason-354-U1450A-78,80F_bagged-Spot 306	394	235385	4.7	17.4537	0.7	0.5998	2.0	0.0759	1.9	0.94	471.7	8.7	477.1	7.8	502.9	14.7	471.7	8.7
Gleason-354-U1450A-78,80F_bagged-Spot 288	258	283111	4.6	17.5981	0.6	0.5987	2.0	0.0764	1.9	0.95	474.7	8.6	476.4	7.5	484.7	13.3	474.7	8.6
Gleason-354-U1450A-78,80F_bagged-Spot 196	330	45252	2.7	17.4280	0.8	0.6061	1.6	0.0766	1.4	0.86	475.9	6.3	481.1	6.1	506.1	17.8	475.9	6.3
Gleason-354-U1450A-78,80F_bagged-Spot 311	1138	88246	2.8	17.3934	0.8	0.6075	1.9	0.0766	1.7	0.91	476.0	8.0	482.0	7.3	510.5	16.9	476.0	8.0
Gleason-354-U1450A-78,80F_bagged-Spot 57	625	687479	4.1	17.6067	0.7	0.6013	2.4	0.0768	2.3	0.96	476.9	10.5	478.0	9.1	483.7	14.6	476.9	10.5
Gleason-354-U1450A-78,80F_bagged-Spot 193	106	20206	2.2	17.8084	1.0	0.5953	2.4	0.0769	2.2	0.91	477.5	10.0	474.2	9.1	458.4	22.5	477.5	10.0
Gleason-354-U1450A-78,80F_bagged-Spot 236	342	109867	1.1	17.4491	0.6	0.6095	1.6	0.0771	1.5	0.92	479.0	6.9	483.2	6.3	503.5	14.1	479.0	6.9
Gleason-354-U1450A-78,80F_bagged-Spot 94	385	53353	4.7	17.6498	0.7	0.6066	2.2	0.0777	2.0	0.94	482.1	9.4	481.4	8.3	478.2	16.5	482.1	9.4
Gleason-354-U1450A-78,80F_bagged-Spot 216	1676	245616	1.5	17.2885	0.7	0.6194	1.8	0.0777	1.7	0.93	482.2	7.9	489.5	7.1	523.8	14.8	482.2	7.9
Gleason-354-U1450A-78,80F_bagged-Spot 206	676	89332	7.9	17.5177	0.8	0.6127	1.7	0.0778	1.5	0.90	483.2	7.2	485.3	6.6	494.8	16.8	483.2	7.2
Gleason-354-U1450A-78,80F_bagged-Spot 280	207	52626	12.0	17.2383	0.8	0.6244	2.6	0.0781	2.5	0.96	484.6	11.7	492.6	10.2	530.2	16.6	484.6	11.7
Gleason-354-U1450A-78,80F_bagged-Spot 111	132	15678	1.0	17.5166	1.1	0.6180	2.5	0.0785	2.2	0.90	487.2	10.5	488.6	9.6	494.9	23.3	487.2	10.5
Gleason-354-U1450A-78,80F_bagged-Spot 201	523	104491	2.7	17.5055	0.8	0.6206	2.1	0.0788	1.9	0.93	488.9	9.0	490.2	8.0	496.4	16.7	488.9	9.0

Gleason-354-U1450A-78,80F_bagged-Spot 205	498	379134	2.2	17.4932	0.6	0.6265	1.9	0.0795	1.8	0.95	493.0	8.7	493.9	7.6	497.9	13.5	493.0	8.7
Gleason-354-U1450A-78,80F_bagged-Spot 22	277	31511	2.6	17.6861	0.8	0.6197	2.4	0.0795	2.3	0.95	493.1	10.8	489.7	9.3	473.7	17.2	493.1	10.8
Gleason-354-U1450A-78,80F_bagged-Spot 174	491	34847	8.3	17.5108	0.6	0.6301	1.6	0.0800	1.5	0.92	496.2	7.1	496.1	6.4	495.7	14.1	496.2	7.1
Gleason-354-U1450A-78,80F_bagged-Spot 177	85	16570	1.5	17.6400	1.3	0.6300	2.5	0.0806	2.1	0.86	499.7	10.2	496.1	9.7	479.4	27.9	499.7	10.2
Gleason-354-U1450A-78,80F_bagged-Spot 51	432	53121	2.2	17.5179	1.0	0.6375	2.5	0.0810	2.3	0.92	502.1	11.0	500.8	9.8	494.8	22.0	502.1	11.0
Gleason-354-U1450A-78,80F_bagged-Spot 135	286	37892	1.4	17.5111	0.6	0.6441	1.9	0.0818	1.8	0.94	506.9	8.6	504.9	7.4	495.7	13.9	506.9	8.6
Gleason-354-U1450A-78,80F_bagged-Spot 179	162	17488	56.0	17.6021	1.0	0.6411	2.1	0.0818	1.9	0.90	507.1	9.4	503.0	8.5	484.2	21.1	507.1	9.4
Gleason-354-U1450A-78,80F_bagged-Spot 274	99	16455	0.7	17.3888	0.8	0.6496	2.8	0.0819	2.7	0.96	507.6	13.0	508.2	11.1	511.1	17.3	507.6	13.0
Gleason-354-U1450A-78,80F_bagged-Spot 232	654	938164	10.2	17.4614	0.7	0.6491	1.7	0.0822	1.6	0.92	509.3	7.8	507.9	6.9	501.9	14.6	509.3	7.8
Gleason-354-U1450A-78,80F_bagged-Spot 169	93	19429	1.5	17.5568	0.9	0.6461	2.5	0.0823	2.3	0.93	509.7	11.3	506.1	9.9	489.9	20.3	509.7	11.3
Gleason-354-U1450A-78,80F_bagged-Spot 303	51	105506	0.8	16.3848	1.2	0.7148	2.7	0.0849	2.5	0.91	525.6	12.5	547.6	11.6	640.4	24.8	525.6	12.5
Gleason-354-U1450A-78,80F_bagged-Spot 24	1051	#######	9.3	16.9175	0.5	0.6982	1.7	0.0857	1.6	0.95	529.9	8.3	537.7	7.1	571.2	11.2	529.9	8.3
Gleason-354-U1450A-78,80F_bagged-Spot 276	165	15527	0.5	17.3218	0.8	0.6917	1.9	0.0869	1.7	0.89	537.1	8.6	533.8	7.8	519.5	18.6	537.1	8.6
Gleason-354-U1450A-78,80F_bagged-Spot 133	506	184277	1.7	17.3435	0.6	0.6945	1.8	0.0874	1.7	0.94	539.9	8.6	535.5	7.4	516.8	13.1	539.9	8.6
Gleason-354-U1450A-78,80F_bagged-Spot 70	389	201741	1.8	17.0929	0.8	0.7106	2.1	0.0881	1.9	0.93	544.3	10.1	545.1	8.8	548.7	16.5	544.3	10.1
Gleason-354-U1450A-78,80F_bagged-Spot 235	419	252033	1.1	16.9719	0.7	0.7278	1.9	0.0896	1.7	0.92	553.1	9.0	555.3	7.9	564.1	15.8	553.1	9.0
Gleason-354-U1450A-78,80F_bagged-Spot 96	884	99276	29.6	16.5337	0.6	0.7601	1.5	0.0911	1.4	0.91	562.3	7.3	574.0	6.5	620.9	13.2	562.3	7.3
Gleason-354-U1450A-78,80F_bagged-Spot 210	286	48284	6.0	16.0969	1.2	0.7858	2.4	0.0917	2.1	0.87	565.8	11.2	588.8	10.6	678.4	24.9	565.8	11.2
Gleason-354-U1450A-78,80F_bagged-Spot 68	56	6458	3.2	16.7365	1.3	0.8281	3.0	0.1005	2.7	0.90	617.5	16.1	612.6	13.9	594.5	28.0	617.5	16.1
Gleason-354-U1450A-78,80F_bagged-Spot 173	158	33553	2.0	16.7642	0.8	0.8285	2.4	0.1007	2.3	0.94	618.7	13.6	612.8	11.3	590.9	18.3	618.7	13.6
Gleason-354-U1450A-78,80F_bagged-Spot 107	642	1398884	9.4	16.0065	0.5	0.9025	2.1	0.1048	2.1	0.97	642.3	12.7	653.1	10.3	690.4	10.5	642.3	12.7
Gleason-354-U1450A-78,80F_bagged-Spot 131	301	86936	1.7	16.3774	0.6	0.9005	1.9	0.1070	1.8	0.95	655.1	11.5	652.0	9.3	641.4	12.6	655.1	11.5
Gleason-354-U1450A-78,80F_bagged-Spot 61	75	72455	2.7	15.7576	1.0	0.9589	4.8	0.1096	4.7	0.98	670.3	29.9	682.7	23.9	723.7	22.1	670.3	29.9
Gleason-354-U1450A-78,80F_bagged-Spot 242	87	57746	0.9	15.7844	0.8	1.0277	2.5	0.1176	2.4	0.95	717.0	16.4	717.8	13.1	720.1	16.7	717.0	16.4
Gleason-354-U1450A-78,80F_bagged-Spot 277	52	81455	1.1	15.6092	0.9	1.0795	3.0	0.1222	2.9	0.96	743.3	20.3	743.4	16.0	743.7	18.9	743.3	20.3
Gleason-354-U1450A-78,80F_bagged-Spot 16	116	76121	1.2	15.6555	0.9	1.0808	3.5	0.1227	3.4	0.96	746.0	24.1	743.9	18.7	737.5	19.9	746.0	24.1
Gleason-354-U1450A-78,80F_bagged-Spot 263	168	31329	1.3	15.2405	0.7	1.1356	2.0	0.1255	1.9	0.93	762.3	13.3	770.4	10.8	794.1	15.4	762.3	13.3
Gleason-354-U1450A-78,80F_bagged-Spot 213	55	308751	2.8	14.8849	0.9	1.1717	3.4	0.1265	3.3	0.97	767.8	23.6	787.5	18.5	843.4	17.7	767.8	23.6
Gleason-354-U1450A-78,80F_bagged-Spot 108	244	54595	4.5	15.3689	0.8	1.1400	2.1	0.1271	1.9	0.93	771.1	13.9	772.5	11.1	776.5	16.1	771.1	13.9
Gleason-354-U1450A-78,80F_bagged-Spot 312	371	558988	21.9	15.0664	0.8	1.1662	2.3	0.1274	2.2	0.94	773.2	15.9	784.9	12.6	818.2	15.9	773.2	15.9
Gleason-354-U1450A-78,80F_bagged-Spot 119	826	392813	3.5	14.9501	0.6	1.2049	1.8	0.1306	1.7	0.95	791.5	12.6	802.8	9.8	834.3	11.5	791.5	12.6
Gleason-354-U1450A-78,80F_bagged-Spot 156	1340	791829	29.2	15.1461	0.7	1.1917	1.8	0.1309	1.7	0.92	793.1	12.7	796.8	10.2	807.1	14.7	793.1	12.7
Gleason-354-U1450A-78,80F_bagged-Spot 9	241	456211	1.6	14.8299	0.9	1.2337	2.4	0.1327	2.2	0.93	803.2	16.8	816.0	13.5	851.1	18.8	803.2	16.8
Gleason-354-U1450A-78,80F_bagged-Spot 90	246	75454	1.7	15.0793	0.7	1.2161	2.2	0.1330	2.1	0.95	805.0	16.1	808.0	12.5	816.4	14.6	805.0	16.1
Gleason-354-U1450A-78,80F_bagged-Spot 52	113	88874	2.7	15.0296	0.7	1.2251	2.8	0.1335	2.7	0.96	808.0	20.3	812.1	15.5	823.3	15.3	808.0	20.3
Gleason-354-U1450A-78,80F_bagged-Spot 271	623	103961	20.5	14.8619	0.5	1.2435	1.7	0.1340	1.7	0.95	810.9	12.7	820.5	9.8	846.6	10.9	810.9	12.7
Gleason-354-U1450A-78,80F_bagged-Spot 134	768	82258	3.2	13.9620	0.6	1.3305	1.7	0.1347	1.6	0.93	814.8	12.0	859.1	9.7	975.2	12.5	814.8	12.0
Gleason-354-U1450A-78,80F_bagged-Spot 185	329	660991	2.6	15.0652	0.6	1.2370	1.8	0.1352	1.6	0.94	817.2	12.6	817.5	9.8	818.3	12.5	817.2	12.6
Gleason-354-U1450A-78,80F_bagged-Spot 100	1218	346858	15.6	15.0123	0.4	1.2438	1.5	0.1354	1.4	0.96	818.7	10.9	820.6	8.3	825.6	8.8	818.7	10.9
Gleason-354-U1450A-78,80F_bagged-Spot 158	250	195476	1.3	15.0013	0.5	1.2449	1.9	0.1354	1.8	0.96	818.8	13.9	821.1	10.6	827.2	10.8	818.8	13.9
Gleason-354-U1450A-78,80F_bagged-Spot 188	202	230281	1.9	14.8436	0.7	1.2727	2.5	0.1370	2.4	0.96	827.8	18.6	833.6	14.2	849.2	15.3	827.8	18.6
Gleason-354-U1450A-78,80F_bagged-Spot 8	230	44349	2.5	14.8996	0.6	1.2696	1.8	0.1372	1.7	0.94	828.8	13.5	832.2	10.5	841.4	12.9	828.8	13.5
Gleason-354-U1450A-78,80F_bagged-Spot 140	292	73315	2.0	14.3645	0.8	1.3247	2.5	0.1380	2.3	0.95	833.4	18.2	856.6	14.2	917.1	16.3	833.4	18.2
Gleason-354-U1450A-78,80F_bagged-Spot 164	96	27567	2.1	14.7469	0.9	1.2910	2.8	0.1381	2.6	0.95	833.8	20.6	841.7	15.9	862.8	18.7	833.8	20.6
Gleason-354-U1450A-78,80F_bagged-Spot 214	155	22597	2.3	15.0480	0.7	1.2708	2.2	0.1387	2.1	0.95	837.2	16.2	832.7	12.3	820.7	13.9	837.2	16.2
Gleason-354-U1450A-78,80F_bagged-Spot 260	277	197315	5.1	15.0177	0.6	1.2839	1.8	0.1398	1.7	0.93	843.8	13.1	838.6	10.2	824.9	13.4	843.8	13.1
Gleason-354-U1450A-78,80F_bagged-Spot 246	77	14090	1.8	15.1446	0.9	1.2742	2.9	0.1400	2.7	0.95	844.4	21.8	834.2	16.4	807.3	18.5	844.4	21.8
Gleason-354-U1450A-78,80F_bagged-Spot 38	759	225982	2.9	14.7958	0.9	1.3050	2.2	0.1400	2.0	0.92	844.9	15.8	847.9	12.5	855.9	17.9	844.9	15.8
Gleason-354-U1450A-78,80F_bagged-Spot 223	174	500156	4.1	14.9664	0.7	1.2975	2.4	0.1408	2.3	0.96	849.4	18.6	844.6	13.9	832.1	14.1	849.4	18.6
Gleason-354-U1450A-78,80F_bagged-Spot 255	194	347642	2.1	14.4742	0.8	1.3787	2.1	0.1447	2.0	0.93	871.4	16.0	879.9	12.5	901.4	16.1	871.4	16.0
Gleason-354-U1450A-78,80F_bagged-Spot 113	117	13309	2.3	14.7179	0.8	1.3591	2.4	0.1451	2.3	0.95	873.3	18.6	871.5	14.1	866.9	15.6	873.3	18.6
Gleason-354-U1450A-78,80F_bagged-Spot 266	286	365171	1.5	14.2895	0.7	1.4160	1.9	0.1468	1.8	0.93	882.7	14.6	895.7	11.3	927.8	14.0	927.8	14.0
Gleason-354-U1450A-78,80F_bagged-Spot 197	1859	331398	12.0	14.2689	0.5	1.4003	1.7	0.1449	1.6	0.95	872.4	13.2	889.1	10.1	930.8	10.9	930.8	10.9
Gleason-354-U1450A-78,80F_bagged-Spot 154	536	153693	10.5	14.2285	0.7	1.4953	1.7	0.1543	1.6	0.91	925.1	13.6	928.5	10.5	936.6	14.4	936.6	14.4
Gleason-354-U1450A-78,80F_bagged-Spot 167	27	22207	0.5	14.1599	1.1	1.4947	4.4	0.1535	4.2	0.97	920.6	36.2	928.2	26.5	946.5	21.7	946.5	21.7
Gleason-354-U1450A-78,80F_bagged-Spot 35	50	30744	0.9	14.1014	1.0	1.5820	3.6	0.1618	3.5	0.96	966.8	31.4	963.2	22.6	954.9	20.1	954.9	20.1
Gleason-354-U1450A-78,80F_bagged-Spot 183	435	154882	14.3	13.9822	0.6	1.5927	1.5	0.1615	1.4	0.92	965.2	12.7	967.4	9.6	972.3	12.0	972.3	12.0
Gleason-354-U1450A-78,80F_bagged-Spot 207	394	141389	1.2	13.9352	0.9	1.5244	2.3	0.1541	2.1	0.92	923.7	18.4	940.3	14.2	979.1	18.4	979.1	18.4
Gleason-354-U1450A-78,80F_bagged-Spot 33	125	61238	2.1	13.9050	0.7	1.6467	2.1	0.1661	2.0	0.94	990.4	18.5	988.3	13.6	983.6	15.0	983.6	15.0
Gleason-354-U1450A-78,80F_bagged-Spot 244	207	267677	2.1	13.8775	0.6	1.6232	2.1	0.1634	2.0	0.95	975.5	18.5	979.2	13.5	987.6	13.1	987.6	13.1
Gleason-354-U1450A-78,80F_bagged-Spot 85	228	51104	1.5	13.7513	0.7	1.6241	2.1	0.1620	2.0	0.94	967.8	17.9	979.6	13.3	1006.2	14.5	1006.2	14.5
Gleason-354-U1450A-78,80F_bagged-Spot 307	103	16537	0.9	13.7023	2.3	1.5328	3.4	0.1523	2.5	0.72	914.0	20.9	943.6	20.8	1013.4	47.4	1013.4	47.4
Gleason-354-U1450A-78,80F_bagged-Spot 124	319	50152	2.9	13.5230	0.7	1.6868	2.3	0.1654	2.2	0.96	987.0	20.5	1003.6	14.9	1040.0	13.9	1040.0	13.9
Gleason-354-U1450A-78,80F_bagged-Spot 259	177	17141	2.0	13.5052	1.1	1.6383	2.2	0.1605	1.9	0.86	959.4	16.9	985.1	13.9	1042.7	22.8	1042.7	22.8
Gleason-354-U1450A-78,80F_bagged-Spot 224	143	433451	2.0	13.3396	0.8	1.7399	2.5	0.1683	2.4	0.95	1002.9	22.3	1023.4	16.2	1067.6	15.2	1067.6	15.2
Gleason-354-U1450A-78,80F_bagged-Spot 283	376	68040	3.9	13.3307	0.6	1.7464	2.3	0.1688	2.2	0.96	1005.7	20.7	1025.8	15.0	1068.9	13.0	1068.9	13.0
Gleason-354-U1450A-78,80F_bagged-Spot 41	252	40592	1.4	13.2914	0.8	1.9099	1.8	0.1841	1.6	0.90	1089.4	15.8	1084.5	11.7	1074.8	15.4	1074.8	15.4
Gleason-354-U1450A-78,80F_bagged-Spot 182	118	40827	2.0	13.2906	0.7	1.8900	2.5	0.1822	2.4	0.96	1078.9	23.7	1077.6	16.4	1075.0	13.3	1075.0	13.3
Gleason-354-U1450A-78,80F_bagged-Spot 146	286	#######	4.6	13.2811	0.6	1.8762	2.0	0.1807	1.9	0.95	1070.9	18.9	1072.7	13.3	1076.4	12.0	1076.4	12.0
Gleason-354-U1450A-78,80F_bagged-Spot 105	373	91731	2.2	13.2798	0.8	1.8631	1.9	0.1794	1.7	0.91	1063.9	16.6	1068.1	12.3	1076.6	15.4	1076.6	15.4
Gleason-354-U1450A-78,80F_bagged-Spot 11	56	7391	1.2	13.2390	0.8	1.9037	2.8	0.1828	2.7	0.96	1082.2	26.9	1082.4	18.7	1082.8	15.4	1082.8	15.4
Gleason-354-U1450A-78,80F_bagged-Spot 160	261	298905	1.8	13.2270	0.7	1.9527	2.1	0.1873	1.9	0.94	1106.9	19.8	1099.4	13.9	1084.6	14.2	1084.6	14.2
Gleason-354-U1450A-78,80F_bagged-Spot 208	430	305558	3.9	13.2095	0.9	1.7074	2.1	0.1636	1.9	0.90	976.6	17.3	1011.3	13.6	1087.2	18.5	1087.2	18.5
Gleason-354-U1450A-78,80F_bagged-Spot 104	776	236537	2.8	13.1758	0.7	1.8030	2.3	0.1723	2.2	0.96	1024.7	21.1	1046.5	15.2	1092.3	13.2	1092.3	13.2
Gleason-354-U1450A-78,80F_bagged-Spot 102	392	106539	1.5	13.0978	0.7	1.9175	1.7	0.1821	1.6	0.92	1078.7	15.8	1087.2	11.5	1104.2	13.1	1104.2	13.1
Gleason-354-U1450A-78,80F_bagged-Spot 132	202	71941	0.6	13.0024	0.7	1.9131	2.2	0.1804	2.1	0.95	1069.2	20.7	1085.7	14.7	1118.9	13.2	1118.9	13.2
Gleason-354-U1450A-78,80F_bagged-Spot 138	212	54228	1.1	12.9957	0.6	2.1021	1.8	0.1981	1.8	0.95	1165.3	18.7	1149.5	12.7	1119.9	11.7	1119.9	11.7
Gleason-354-U1450A-78,80F_bagged-Spot 309	1499	588053	0.6	12.9455	0.5	2.1257	1.8	0.1996	1.8	0.96	1173.1	18.9	1157.2	12.7	1127.6	10.8	1127.6	10.8
Gleason-354-U1450A-78,80F_bagged-Spot 297	71	47170	1.1	12.9041	0.9	1.6441	5.0	0.1539	5.0	0.98	922.6	42.6	987.3	31.8	1133.9	17.5	1133.9	17.5
Gleason-354-U1450A-78,80F_bagged-Spot 264	281	156761	8.4	12.8678	0.8	1.9270	2.0	0.1798	1.8	0.92	1066.1	17.6	1090.5	13.0	1139.6	15.4	1139.6	15.4
Gleason-354-U1450A-78,80F_bagged-Spot 284	1038	217871	7.0	12.8514	0.7	2.0084	1.7	0.1872	1.6	0.91	1106.2	16.0	1118.3	11.8	1142.1	14.7	1142.1	14.7
Gleason-354-U1450A-78,80F_bagged-Spot 7	516	256270	3.0	12.8514	1.1	1.9016	2.0	0.1772	1.6	0.82	1051.9	15.8	1081.7	13.2	1142.1	22.5	1142.1	22.5
Gleason-354-U1450A-78,80F_bagged-Spot 143	285	407587	1.8	12.8059	0.5	2.0370	2.2	0.1892	2.1	0.97	1117.0	21.4	1128.0	14.7	1149.2	10.8	1149.2	10.8
Gleason-354-U1450A-78,80F_bagged-Spot 151	223	175016	1.6	12.8042	0.7	2.0645	2.3	0.1917	2.2	0.95	1130.7	22.7	1137.1	15.8	1149.4	14.7	1149.4	14.7
Gleason-354-U1450A-78,80F_bagged-Spot 49	367	85376	2.0	12.8038	0.7	1.9984	1.9	0.1856	1.7	0.94	1097.4	17.6	1115.0	12.6	1149.5	13.0	1149.5	13.0
Gleason-354-U1450A-78,80F_bagged-Spot 123	94	19042	1.4	12.7880	0.6	2.1300	2.5	0.1976	2.4	0.97	1162.2	25.3	1158.6	17.0	1151.9	12.6	1151.9	12.6
Gleason-354-U1450A-78,80F_bagged-Spot 98	781	168068	10.6	12.7453	0.6	2.1208	1.6	0.1960	1.5	0.94	1154.0	15.7	1155.6	10.9	1158.5	11.1	1158.5	11.1
Gleason-354-U1450A-78,80F_bagged-Spot 21	183	66157	1.7	12.6575	0.9	2.0018	2.1	0.1838	2.0	0.92	1087.5	19.5	1116.1	14.4	1172.3	17.0	1172.3	17.0
Gleason-354-U1450A-78,80F_bagged-Spot 147	139	50545	2.0	12.6548	0.9	2.2000	2.5	0.2019	2.3	0.93	1185.6	25.2	1181.1	17.4	1172.7	17.8	1172.7	17.8
Gleason-354-U1450A-78,80F_bagged-Spot 125	161	40439	0.7	12.6269	0.5	2.2006	2.3	0.2015	2.2	0.97	1183.5	24.1	1181.2	15.9	1177.0	10.1	1177.0	10.1
Gleason-354-U1450A-78,80F_bagged-Spot 69	62	311120	1.2	12.6225	0.8	2.2103	3.0	0.2023	2.8	0.96	1187.9	30.8	1184.3	20.7	1177.7	16.8	1177.7	16.8
Gleason-354-U1450A-78,80F_bagged-Spot 292	320	102822	2.4	12.5236	0.6	2.1058	1.8	0.1913	1.7	0.94	1128.2	17.9	1150.7	12.6	1193.3	12.2	1193.3	12.2
Gleason-354-U1450A-78,80F_bagged-Spot 298	101	107155	1.5	12.4604	0.7	2.1862	2.3	0.1976	2.2	0.96	1162.2	23.0	1176.6	15.8	1203.2	13.1	1203.2	13.1
Gleason-354-U1450A-78,80F_bagged-Spot 176	425	108872	1.9	12.2985	0.6	2.1771	2.1	0.1942	2.0	0.95	1144.1	21.3	1173.8	14.8	1228.9	12.5	1228.9	12.5
Gleason-354-U1450A-78,80F_bagged-Spot 99	257	95638	2.2	12.2954	0.7	2.1741	2.1	0.1939	2.0	0.95	1142.3	20.9	1172.8	14.7	1229.4	13.5	1229.4	13.5

Gleason-354-U1450A-78,80F_bagged-Spot 36	338	1113764	10.2	12.2464	0.7	2.1145	1.9	0.1878	1.7	0.93	1109.5	17.6	1153.5	12.8	1237.3	13.7	1237.3	13.7
Gleason-354-U1450A-78,80F_bagged-Spot 279	138	22101	1.4	12.1824	0.6	2.4601	2.7	0.2174	2.6	0.97	1267.9	29.7	1260.4	19.2	1247.5	12.3	1247.5	12.3
Gleason-354-U1450A-78,80F_bagged-Spot 6	172	143390	1.0	12.1089	0.8	2.4089	2.1	0.2116	2.0	0.94	1237.1	22.4	1245.2	15.2	1259.4	14.7	1259.4	14.7
Gleason-354-U1450A-78,80F_bagged-Spot 251	108	60835	1.2	12.0735	0.7	2.2463	2.5	0.1967	2.4	0.96	1157.6	25.7	1195.6	17.7	1265.1	13.5	1265.1	13.5
Gleason-354-U1450A-78,80F_bagged-Spot 142	120	39953	2.3	12.0732	0.7	2.4940	2.6	0.2184	2.5	0.96	1273.3	28.7	1270.3	18.8	1265.1	14.5	1265.1	14.5
Gleason-354-U1450A-78,80F_bagged-Spot 180	133	21628	2.0	12.0440	0.7	2.5666	1.9	0.2242	1.8	0.94	1304.0	20.8	1291.2	13.8	1269.9	13.0	1269.9	13.0
Gleason-354-U1450A-78,80F_bagged-Spot 83	822	165572	0.9	11.9030	0.6	2.5083	1.8	0.2165	1.7	0.93	1263.6	19.5	1274.4	13.2	1292.8	12.6	1292.8	12.6
Gleason-354-U1450A-78,80F_bagged-Spot 71	604	185855	0.6	11.8306	0.8	2.3705	1.9	0.2034	1.7	0.91	1193.5	18.4	1233.7	13.2	1304.7	14.9	1304.7	14.9
Gleason-354-U1450A-78,80F_bagged-Spot 240	89	92252	2.3	11.6814	0.8	2.6763	2.9	0.2267	2.8	0.96	1317.4	32.8	1321.9	21.3	1329.3	16.0	1329.3	16.0
Gleason-354-U1450A-78,80F_bagged-Spot 293	179	62550	2.4	11.6798	0.7	2.6577	2.0	0.2251	1.9	0.94	1309.0	22.4	1316.8	14.9	1329.6	13.2	1329.6	13.2
Gleason-354-U1450A-78,80F_bagged-Spot 181	165	347968	2.3	11.6689	0.7	2.7328	2.3	0.2313	2.2	0.95	1341.2	26.6	1337.4	17.1	1331.4	13.6	1331.4	13.6
Gleason-354-U1450A-78,80F_bagged-Spot 34	133	84034	1.3	11.6177	0.8	2.6517	2.4	0.2234	2.3	0.94	1300.0	27.1	1315.1	18.0	1339.9	15.9	1339.9	15.9
Gleason-354-U1450A-78,80F_bagged-Spot 59	243	234629	5.6	11.5691	0.8	2.5918	2.8	0.2175	2.7	0.96	1268.5	31.1	1298.3	20.7	1348.0	16.1	1348.0	16.1
Gleason-354-U1450A-78,80F_bagged-Spot 275	172	64965	1.1	11.4342	0.9	2.8364	2.3	0.2352	2.1	0.93	1361.8	26.0	1365.2	17.1	1370.6	16.5	1370.6	16.5
Gleason-354-U1450A-78,80F_bagged-Spot 80	43	12929	0.9	11.3527	0.7	2.9825	3.2	0.2456	3.1	0.97	1415.6	39.6	1403.2	24.3	1384.3	13.7	1384.3	13.7
Gleason-354-U1450A-78,80F_bagged-Spot 78	76	21202	1.8	11.2427	0.7	2.9236	2.3	0.2384	2.2	0.96	1378.3	27.2	1388.0	17.3	1403.0	12.7	1403.0	12.7
Gleason-354-U1450A-78,80F_bagged-Spot 203	61	11663	1.6	10.9516	0.7	2.8908	2.6	0.2296	2.5	0.96	1332.4	30.2	1379.5	19.7	1453.1	13.7	1453.1	13.7
Gleason-354-U1450A-78,80F_bagged-Spot 89	2127	657302	56.8	10.6137	0.7	3.1706	1.8	0.2441	1.7	0.93	1407.8	21.6	1450.0	14.2	1512.5	12.7	1512.5	12.7
Gleason-354-U1450A-78,80F_bagged-Spot 233	99	24832	3.0	10.4955	0.7	3.6263	2.4	0.2760	2.3	0.96	1571.4	32.7	1555.3	19.4	1533.6	12.9	1533.6	12.9
Gleason-354-U1450A-78,80F_bagged-Spot 2	194	165844	1.3	10.2810	0.7	3.5738	2.6	0.2665	2.5	0.96	1522.9	34.5	1543.7	21.0	1572.3	13.7	1572.3	13.7
Gleason-354-U1450A-78,80F_bagged-Spot 221	563	705427	5.4	10.1436	0.5	3.6988	1.7	0.2721	1.6	0.95	1551.5	22.4	1571.1	13.7	1597.5	10.2	1597.5	10.2
Gleason-354-U1450A-78,80F_bagged-Spot 228	392	249640	1.8	10.1334	0.6	3.3804	1.9	0.2484	1.8	0.95	1430.4	22.8	1499.9	14.6	1599.4	10.5	1599.4	10.5
Gleason-354-U1450A-78,80F_bagged-Spot 3	275	316635	1.4	10.0669	0.7	3.5777	2.0	0.2612	1.8	0.93	1496.0	24.3	1544.6	15.5	1611.7	13.3	1611.7	13.3
Gleason-354-U1450A-78,80F_bagged-Spot 15	308	681667	1.3	10.0464	0.7	3.6849	1.8	0.2685	1.6	0.93	1533.1	22.1	1568.1	14.0	1615.5	12.4	1615.5	12.4
Gleason-354-U1450A-78,80F_bagged-Spot 189	901	7130824	4.4	9.9987	0.7	3.7581	1.9	0.2725	1.8	0.93	1553.6	24.6	1583.8	15.3	1624.3	12.7	1624.3	12.7
Gleason-354-U1450A-78,80F_bagged-Spot 186	71	539295	1.2	9.9941	0.8	3.9190	2.6	0.2841	2.4	0.94	1611.8	34.5	1617.6	20.7	1625.2	15.6	1625.2	15.6
Gleason-354-U1450A-78,80F_bagged-Spot 195	1022	222420	1.7	9.8540	0.6	4.0760	1.7	0.2913	1.6	0.94	1648.1	22.8	1649.5	13.6	1651.4	10.6	1651.4	10.6
Gleason-354-U1450A-78,80F_bagged-Spot 118	254	168464	2.6	9.8278	0.8	3.5876	2.2	0.2557	2.1	0.94	1467.9	27.7	1546.8	17.8	1656.3	14.0	1656.3	14.0
Gleason-354-U1450A-78,80F_bagged-Spot 153	202	200996	2.4	9.8126	0.8	4.0398	2.6	0.2875	2.5	0.95	1629.1	36.1	1642.3	21.4	1659.2	14.8	1659.2	14.8
Gleason-354-U1450A-78,80F_bagged-Spot 136	192	204460	1.4	9.6984	0.8	4.0390	2.1	0.2841	2.0	0.93	1612.0	28.3	1642.1	17.4	1680.8	14.7	1680.8	14.7
Gleason-354-U1450A-78,80F_bagged-Spot 166	37	41976	0.4	9.5814	1.0	4.1017	3.4	0.2850	3.3	0.95	1616.7	46.5	1654.6	27.8	1703.2	18.8	1703.2	18.8
Gleason-354-U1450A-78,80F_bagged-Spot 310	97	790609	1.1	9.5385	0.9	4.3357	2.9	0.2999	2.8	0.95	1691.0	41.1	1700.2	24.0	1711.5	16.2	1711.5	16.2
Gleason-354-U1450A-78,80F_bagged-Spot 48	156	260384	2.2	9.5319	0.7	4.1529	1.9	0.2871	1.8	0.92	1627.0	25.2	1664.8	15.6	1712.7	13.5	1712.7	13.5
Gleason-354-U1450A-78,80F_bagged-Spot 122	316	1769182	1.9	9.4593	0.5	4.4823	1.7	0.3075	1.6	0.95	1728.4	24.5	1727.7	14.1	1726.8	9.9	1726.8	9.9
Gleason-354-U1450A-78,80F_bagged-Spot 247	154	262383	1.6	9.3871	0.7	4.4669	2.2	0.3041	2.1	0.95	1711.7	31.8	1724.8	18.4	1740.9	12.4	1740.9	12.4
Gleason-354-U1450A-78,80F_bagged-Spot 287	74	33618	2.5	9.3816	0.6	4.6651	2.6	0.3174	2.5	0.98	1777.1	39.5	1761.0	21.8	1741.9	10.2	1741.9	10.2
Gleason-354-U1450A-78,80F_bagged-Spot 222	95	185548	2.6	9.2577	0.7	4.8394	2.6	0.3249	2.4	0.96	1813.8	38.7	1791.8	21.5	1766.2	13.5	1766.2	13.5
Gleason-354-U1450A-78,80F_bagged-Spot 217	469	2452913	1.9	9.2345	0.6	4.6346	2.0	0.3104	1.9	0.96	1742.7	29.0	1755.5	16.6	1770.8	10.6	1770.8	10.6
Gleason-354-U1450A-78,80F_bagged-Spot 115	223	2220264	2.7	9.1999	0.6	4.5508	1.7	0.3036	1.6	0.93	1709.4	23.7	1740.3	14.1	1777.7	11.2	1777.7	11.2
Gleason-354-U1450A-78,80F_bagged-Spot 286	497	539000	1.6	9.1171	0.5	4.5930	2.0	0.3037	1.9	0.96	1709.6	29.1	1748.0	16.8	1794.2	9.8	1794.2	9.8
Gleason-354-U1450A-78,80F_bagged-Spot 62	556	374621	19.1	9.1164	0.7	4.1611	2.1	0.2751	1.9	0.94	1566.8	27.0	1666.4	16.9	1794.3	12.6	1794.3	12.6
Gleason-354-U1450A-78,80F_bagged-Spot 253	21	41729	1.1	9.0552	0.8	5.1556	3.9	0.3386	3.9	0.98	1879.9	62.8	1845.3	33.5	1806.6	14.6	1806.6	14.6
Gleason-354-U1450A-78,80F_bagged-Spot 170	214	346731	7.7	8.9243	0.6	5.1512	2.1	0.3334	2.0	0.96	1854.9	31.7	1844.6	17.5	1833.0	10.9	1833.0	10.9
Gleason-354-U1450A-78,80F_bagged-Spot 152	479	3962066	12.5	8.9194	0.7	4.9020	2.5	0.3171	2.4	0.96	1775.6	37.1	1802.6	20.9	1834.0	11.9	1834.0	11.9
Gleason-354-U1450A-78,80F_bagged-Spot 148	691	83813	3.1	8.9017	0.6	5.2588	2.3	0.3395	2.3	0.96	1884.3	36.9	1862.2	20.0	1837.6	11.3	1837.6	11.3
Gleason-354-U1450A-78,80F_bagged-Spot 225	171	83971	2.8	8.8661	0.5	5.2659	1.8	0.3386	1.8	0.96	1880.0	28.8	1863.3	15.8	1844.8	9.8	1844.8	9.8
Gleason-354-U1450A-78,80F_bagged-Spot 30	207	4436126	1.8	8.7931	0.6	5.1329	2.1	0.3273	2.0	0.95	1825.5	31.8	1841.6	17.8	1859.8	11.3	1859.8	11.3
Gleason-354-U1450A-78,80F_bagged-Spot 209	308	227892	5.5	8.7856	0.8	5.1877	2.2	0.3306	2.1	0.93	1841.1	33.2	1850.6	18.9	1861.3	14.3	1861.3	14.3
Gleason-354-U1450A-78,80F_bagged-Spot 229	552	801477	15.3	8.7696	0.7	4.4090	1.9	0.2804	1.8	0.94	1593.5	25.6	1714.0	16.0	1864.6	12.3	1864.6	12.3
Gleason-354-U1450A-78,80F_bagged-Spot 67	178	69020	3.7	8.7027	0.6	5.1061	2.4	0.3223	2.3	0.97	1800.9	36.3	1837.1	20.3	1878.4	11.2	1878.4	11.2
Gleason-354-U1450A-78,80F_bagged-Spot 249	694	298058	11.1	8.6630	0.6	5.2567	1.7	0.3303	1.6	0.94	1839.7	25.6	1861.9	14.5	1886.6	10.3	1886.6	10.3
Gleason-354-U1450A-78,80F_bagged-Spot 290	532	272973	1.3	8.5170	0.5	5.6657	1.8	0.3500	1.7	0.96	1934.5	28.7	1926.2	15.5	1917.2	9.3	1917.2	9.3
Gleason-354-U1450A-78,80F_bagged-Spot 86	79	242290	0.9	8.5158	0.6	5.6546	2.3	0.3492	2.2	0.97	1931.0	36.7	1924.5	19.6	1917.4	10.0	1917.4	10.0
Gleason-354-U1450A-78,80F_bagged-Spot 204	230	94964	0.9	8.4870	0.5	5.1039	2.0	0.3142	1.9	0.96	1761.2	29.5	1836.8	16.9	1923.5	9.4	1923.5	9.4
Gleason-354-U1450A-78,80F_bagged-Spot 187	188	194511	1.6	8.3580	0.7	5.9875	2.2	0.3629	2.1	0.95	1996.1	36.2	1974.0	19.3	1950.9	12.4	1950.9	12.4
Gleason-354-U1450A-78,80F_bagged-Spot 45	628	235389	2.5	8.2442	0.8	5.0614	2.1	0.3026	2.0	0.93	1704.3	29.9	1829.7	18.2	1975.4	14.1	1975.4	14.1
Gleason-354-U1450A-78,80F_bagged-Spot 304	224	299196	0.6	8.1772	0.6	5.7424	2.0	0.3406	1.9	0.95	1889.4	30.9	1937.8	17.1	1989.9	10.6	1989.9	10.6
Gleason-354-U1450A-78,80F_bagged-Spot 110	286	76049	2.2	8.1568	0.7	6.0169	1.8	0.3560	1.7	0.93	1963.0	29.1	1978.3	16.1	1994.3	11.8	1994.3	11.8
Gleason-354-U1450A-78,80F_bagged-Spot 198	146	271784	2.4	8.0819	0.7	5.4998	2.4	0.3224	2.3	0.96	1801.3	36.5	1900.6	20.8	2010.7	12.2	2010.7	12.2
Gleason-354-U1450A-78,80F_bagged-Spot 252	66	79663	0.7	8.0737	0.8	6.1928	2.2	0.3626	2.1	0.93	1994.6	35.3	2003.4	19.3	2012.5	14.1	2012.5	14.1
Gleason-354-U1450A-78,80F_bagged-Spot 272	379	127095	1.7	8.0470	0.6	5.7694	2.0	0.3367	1.9	0.96	1870.8	31.3	1941.8	17.4	2018.4	10.6	2018.4	10.6
Gleason-354-U1450A-78,80F_bagged-Spot 184	115	95541	0.6	7.3803	0.7	7.1157	2.9	0.3809	2.9	0.97	2080.4	50.9	2126.0	26.2	2170.4	11.7	2170.4	11.7
Gleason-354-U1450A-78,80F_bagged-Spot 19	1183	5137689	4.8	6.7902	0.6	7.6480	2.3	0.3766	2.2	0.96	2060.6	39.1	2190.5	20.7	2314.5	10.9	2314.5	10.9
Gleason-354-U1450A-78,80F_bagged-Spot 172	416	#######	2.2	6.6816	0.6	8.0871	2.4	0.3919	2.3	0.97	2131.6	41.9	2240.8	21.6	2342.1	10.2	2342.1	10.2
Gleason-354-U1450A-78,80F_bagged-Spot 282	36	27557	0.6	6.5094	0.9	8.2445	2.9	0.3892	2.8	0.95	2119.2	49.9	2258.2	26.3	2386.6	15.1	2386.6	15.1
Gleason-354-U1450A-78,80F_bagged-Spot 161	706	615101	4.1	6.4249	0.6	8.7757	1.7	0.4089	1.6	0.94	2210.0	30.0	2315.0	15.5	2408.9	9.5	2408.9	9.5
Gleason-354-U1450A-78,80F_bagged-Spot 114	132	312108	1.3	6.2974	0.6	9.9226	2.2	0.4532	2.1	0.97	2409.4	42.9	2427.6	20.4	2442.9	9.7	2442.9	9.7
Gleason-354-U1450A-78,80F_bagged-Spot 54	346	483204	2.7	6.2889	0.6	9.4080	2.4	0.4291	2.3	0.97	2301.7	44.4	2378.6	21.7	2445.2	9.8	2445.2	9.8
Gleason-354-U1450A-78,80F_bagged-Spot 305	143	145133	1.3	6.2220	0.6	9.7391	2.7	0.4395	2.6	0.97	2348.4	51.1	2410.4	24.5	2463.2	10.1	2463.2	10.1
Gleason-354-U1450A-78,80F_bagged-Spot 53	152	139112	2.2	6.0259	0.5	9.5707	2.2	0.4183	2.1	0.97	2252.7	40.3	2394.4	20.1	2517.2	9.2	2517.2	9.2
Gleason-354-U1450A-78,80F_bagged-Spot 301	431	89529	1.0	5.7549	0.6	9.7372	1.8	0.4064	1.7	0.94	2198.5	31.0	2410.2	16.4	2594.2	10.4	2594.2	10.4
Gleason-354-U1450A-78,80F_bagged-Spot 28	44	17054	1.9	5.3748	0.6	12.7789	2.6	0.4981	2.5	0.98	2605.8	53.7	2663.5	24.2	2707.6	9.3	2707.6	9.3
Gleason-354-U1450A-78,80F_bagged-Spot 239	490	245159	7.6	5.3449	0.7	13.8743	1.8	0.5378	1.7	0.93	2774.4	38.1	2741.2	17.2	2716.8	10.7	2716.8	10.7
Gleason-354-U1450A-78,80F_bagged-Spot 126	47	36502	1.8	5.0366	0.5	14.6617	3.1	0.5356	3.1	0.99	2764.9	69.0	2793.5	29.6	2814.3	8.2	2814.3	8.2
Gleason-354-U1450A-78,80F_bagged-Spot 296	133	73180	1.7	3.9989	0.5	19.0756	2.3	0.5532	2.3	0.97	2838.7	52.3	3045.6	22.6	3185.2	8.5	3185.2	8.5
Gleason-354-U1450A-78,80F_bagged-Spot 163	362	53962	2.4	3.3734	0.7	23.0599	1.9	0.5642	1.8	0.94	2883.9	42.7	3229.5	19.0	3451.7	10.1	3451.7	10.1

Table ______. U-Pb geochronologic analyses.																		
#12 U1450_98F						Isotope ratios						Apparent ages (Ma)						
Late Pliocene 3.5 Ma																		
Analysis	U	206Pb	U/Th	206Pb*	±	207Pb*	±	206Pb*	±	error	206Pb*	±	207Pb*	±	206Pb*	±	Best age	±
	(ppm)	204Pb		207Pb*	(%)	235U*	(%)	238U	(%)	corr.	238U*	(Ma)	235U	(Ma)	207Pb*	(Ma)	(Ma)	(Ma)
Gleason_354-U1450-A98F-1-Spot 229	5409	40911	7.7	21.2237	1.0	0.0178	1.9	0.0027	1.6	0.86	17.6	0.3	17.9	0.3	55.1	23.2	17.6	0.3
Gleason_354-U1450-A98F-1-Spot 15	3105	43599	92.7	20.8294	0.9	0.0221	2.6	0.0033	2.4	0.93	21.5	0.5	22.2	0.6	99.7	22.4	21.5	0.5
Gleason_354-U1450-A98F-1-Spot 32	780	3058	24.6	22.3255	1.6	0.0210	3.1	0.0034	2.6	0.85	21.9	0.6	21.1	0.6	66.9	39.8	21.9	0.6
Gleason_354-U1450-A98F-1-Spot 275	52	3174	574.7	3.0851	6.6	0.2057	8.8	0.0046	5.8	0.66	29.6	1.7	189.9	15.2	3589.6	101.7	29.6	1.7
Gleason_354-U1450-A98F-1-Spot 161	2098	9631	0.8	21.0101	1.0	0.0315	2.3	0.0048	2.1	0.91	30.9	0.7	31.5	0.7	79.2	23.2	30.9	0.7
Gleason_354-U1450-A98F-1-Spot 206	827	22735	82.7	20.7324	1.4	0.0334	2.3	0.0050	1.8	0.78	32.2	0.6	33.3	0.7	110.8	33.7	32.2	0.6
Gleason_354-U1450-A98F-1-Spot 103	587	30691	0.9	20.5586	2.3	0.0341	3.4	0.0051	2.5	0.74	32.7	0.8	34.1	1.1	130.6	53.5	32.7	0.8
Gleason_354-U1450-A98F-1-Spot 196	16200	58253	103.8	21.3414	0.8	0.0337	2.5	0.0052	2.4	0.94	33.6	0.8	33.7	0.8	42.0	20.1	33.6	0.8
Gleason_354-U1450-A98F-1-Spot 51	2155	6600	4.0	21.9383	1.0	0.0341	2.7	0.0054	2.5	0.93	34.9	0.9	34.1	0.9	24.4	23.9	34.9	0.9
Gleason_354-U1450-A98F-1-Spot 98	1027	5166	157.5	20.5619	1.8	0.0385	9.7	0.0057	9.5	0.98	36.9	3.5	38.3	3.6	130.2	42.8	36.9	3.5
Gleason_354-U1450-A98F-1-Spot 269	254	2553	131.7	21.8010	2.9	0.0366	4.0	0.0058	2.8	0.70	37.2	1.0	36.5	1.4	9.2	69.7	37.2	1.0
Gleason_354-U1450-A98F-1-Spot 35	1037	4020	100.7	17.5753	3.3	0.0518	7.2	0.0066	6.4	0.89	42.4	2.7	51.2	3.6	487.6	73.3	42.4	2.7
Gleason_354-U1450-A98F-1-Spot 8	40	90256	1.6	20.7380	4.3	0.0551	6.2	0.0083	4.4	0.72	53.2	2.4	54.5	3.3	110.1	100.8	53.2	2.4
Gleason_354-U1450-A98F-1-Spot 225	109	2531	4.6	21.8327	2.7	0.0569	4.1	0.0090	3.1	0.76	57.8	1.8	56.2	2.2	12.7	64.8	57.8	1.8
Gleason_354-U1450-A98F-1-Spot 214	72	2343	1.9	22.5987	3.5	0.0553	5.0	0.0091	3.7	0.73	58.1	2.1	54.6	2.7	96.7	85.2	58.1	2.1
Gleason_354-U1450-A98F-1-Spot 168	1376	16161	143.5	18.9979	1.5	0.0781	7.0	0.0108	6.8	0.98	69.0	4.7	76.4	5.1	313.2	34.1	69.0	4.7
Gleason_354-U1450-A98F-1-Spot 272	97	3625	1.8	18.9539	3.3	0.0872	4.9	0.0120	3.6	0.73	76.8	2.8	84.9	4.0	318.4	75.8	76.8	2.8
Gleason_354-U1450-A98F-1-Spot 89	1909	10943	0.6	20.6041	1.0	0.0817	3.4	0.0122	3.2	0.95	78.2	2.5	79.8	2.6	125.4	24.1	78.2	2.5
Gleason_354-U1450-A98F-1-Spot 262	235	7950	26.8	20.5056	1.9	0.0821	2.7	0.0122	2.0	0.72	78.3	1.5	80.1	2.1	136.6	44.5	78.3	1.5
Gleason_354-U1450-A98F-1-Spot 254	193	700051	6.6	20.4855	2.2	0.0837	3.4	0.0124	2.6	0.76	79.6	2.0	81.6	2.6	138.9	51.1	79.6	2.0
Gleason_354-U1450-A98F-1-Spot 20	84	651	1.4	37.9593	12.6	0.0462	13.1	0.0127	3.6	0.28	81.4	2.9	45.8	5.9	1577.7	426.4	81.4	2.9
Gleason_354-U1450-A98F-1-Spot 64	77	2995	1.6	22.8072	6.3	0.0796	7.3	0.0132	3.7	0.51	84.3	3.1	77.8	5.5	119.3	155.3	84.3	3.1
Gleason_354-U1450-A98F-1-Spot 288	822	10704	7.8	20.9818	1.0	0.0895	2.2	0.0136	1.9	0.88	87.2	1.6	87.0	1.8	82.4	24.0	87.2	1.6
Gleason_354-U1450-A98F-1-Spot 286	404	6541	2.3	20.7834	2.6	0.0903	3.4	0.0136	2.2	0.65	87.2	1.9	87.8	2.8	104.9	60.3	87.2	1.9
Gleason_354-U1450-A98F-1-Spot 118	296	11424	2.0	20.3202	1.5	0.1087	2.3	0.0160	1.8	0.77	102.5	1.8	104.8	2.3	157.9	35.0	102.5	1.8
Gleason_354-U1450-A98F-1-Spot 28	38	779	2.9	32.2010	4.7	0.0732	5.9	0.0171	3.5	0.60	109.2	3.8	71.7	4.1	1054.0	141.7	109.2	3.8
Gleason_354-U1450-A98F-1-Spot 7	91	2604	1.0	22.1383	5.1	0.1277	6.0	0.0205	3.2	0.53	130.9	4.1	122.1	6.9	46.4	124.3	130.9	4.1
Gleason_354-U1450-A98F-1-Spot 192	25	894	1.8	29.2615	12.8	0.0998	13.6	0.0212	4.4	0.33	135.1	5.9	96.6	12.5	775.5	363.4	135.1	5.9
Gleason_354-U1450-A98F-1-Spot 299	39	1001	2.1	14.1488	8.1	0.2107	9.0	0.0216	4.0	0.45	137.9	5.5	194.2	16.0	948.1	165.6	137.9	5.5
Gleason_354-U1450-A98F-1-Spot 10	448	12255	10.4	17.6951	1.2	0.2294	4.3	0.0294	4.1	0.96	187.1	7.6	209.7	8.2	472.6	27.6	187.1	7.6
Gleason_354-U1450-A98F-1-Spot 163	818	226049	2.3	19.3959	0.6	0.2428	2.2	0.0342	2.1	0.95	216.5	4.4	220.7	4.3	265.8	14.9	216.5	4.4
Gleason_354-U1450-A98F-1-Spot 162	162	39114	2.6	19.3872	1.5	0.2931	3.0	0.0412	2.6	0.87	260.3	6.6	261.0	6.9	266.9	33.7	260.3	6.6
Gleason_354-U1450-A98F-1-Spot 53	314	12224	1.6	19.3398	0.8	0.2972	2.6	0.0417	2.5	0.95	263.3	6.5	264.2	6.2	272.4	19.0	263.3	6.5
Gleason_354-U1450-A98F-1-Spot 171	120	5720	1.8	20.6706	1.4	0.2846	3.6	0.0427	3.3	0.92	269.4	8.8	254.3	8.1	117.8	32.8	269.4	8.8
Gleason_354-U1450-A98F-1-Spot 235	213	30668	4.3	17.7061	1.0	0.3601	6.5	0.0462	6.4	0.99	291.4	18.2	312.3	17.4	471.2	23.1	291.4	18.2
Gleason_354-U1450-A98F-1-Spot 129	227	8165	1.8	18.9413	0.9	0.3967	2.5	0.0545	2.4	0.93	342.1	7.9	339.3	7.3	320.0	21.3	342.1	7.9
Gleason_354-U1450-A98F-1-Spot 290	346	25788	3.1	17.4025	1.1	0.5270	2.5	0.0665	2.3	0.91	415.1	9.2	429.8	8.8	509.3	23.2	415.1	9.2
Gleason_354-U1450-A98F-1-Spot 65	810	277204	5.1	17.5573	0.7	0.5366	3.3	0.0683	3.3	0.98	426.1	13.4	436.2	11.8	489.9	15.8	426.1	13.4
Gleason_354-U1450-A98F-1-Spot 29	529	2509858	1.9	17.4142	0.8	0.5536	2.3	0.0699	2.1	0.93	435.7	9.1	447.4	8.3	507.9	18.2	435.7	9.1
Gleason_354-U1450-A98F-1-Spot 170	964	52513	0.6	17.4791	0.7	0.5640	2.5	0.0715	2.4	0.96	445.2	10.1	454.1	9.0	499.7	14.7	445.2	10.1
Gleason_354-U1450-A98F-1-Spot 238	747	62102	77.5	17.4194	0.8	0.5735	2.4	0.0725	2.2	0.95	450.9	9.7	460.3	8.7	507.2	16.7	450.9	9.7
Gleason_354-U1450-A98F-1-Spot 278	2919	307179	2.0	17.2620	0.7	0.5826	1.9	0.0729	1.8	0.94	453.9	7.9	466.2	7.2	527.2	14.6	453.9	7.9
Gleason_354-U1450-A98F-1-Spot 40	5398	870747	7.8	17.6690	0.6	0.5727	4.3	0.0734	4.3	0.99	456.5	18.7	459.7	15.9	475.8	14.0	456.5	18.7
Gleason_354-U1450-A98F-1-Spot 194	1718	153592	104.8	17.3086	0.7	0.5859	2.2	0.0736	2.0	0.95	457.5	9.0	468.3	8.1	521.2	15.1	457.5	9.0
Gleason_354-U1450-A98F-1-Spot 47	285	130519	1.3	17.6267	1.0	0.5790	3.5	0.0740	3.4	0.96	460.3	15.2	463.8	13.2	481.1	21.0	460.3	15.2
Gleason_354-U1450-A98F-1-Spot 143	795	36643	2.4	17.4649	1.1	0.5850	2.8	0.0741	2.5	0.92	460.8	11.3	467.7	10.3	501.5	23.4	460.8	11.3
Gleason_354-U1450-A98F-1-Spot 83	3464	446081	7.9	17.3015	0.8	0.5935	2.9	0.0745	2.8	0.96	463.0	12.3	473.1	10.8	522.1	16.5	463.0	12.3
Gleason_354-U1450-A98F-1-Spot 39	1849	261625	0.8	17.4057	0.7	0.5949	2.3	0.0751	2.2	0.95	466.8	10.0	474.0	8.9	509.0	16.5	466.8	10.0
Gleason_354-U1450-A98F-1-Spot 267	302	18965	1.8	17.8010	0.7	0.5826	2.1	0.0752	2.0	0.94	467.5	9.1	466.1	8.0	459.4	16.1	467.5	9.1
Gleason_354-U1450-A98F-1-Spot 142	295	163010	1.4	17.4427	0.7	0.5963	2.3	0.0754	2.2	0.95	468.8	9.9	474.9	8.8	504.3	16.0	468.8	9.9
Gleason_354-U1450-A98F-1-Spot 133	1077	91454	3.7	17.4634	0.6	0.5959	2.0	0.0755	1.9	0.95	469.0	8.5	474.6	7.5	501.7	13.7	469.0	8.5
Gleason_354-U1450-A98F-1-Spot 60	242	61898	0.8	17.1977	0.8	0.6058	2.1	0.0756	2.0	0.92	469.6	9.0	480.9	8.2	535.3	18.0	469.6	9.0
Gleason_354-U1450-A98F-1-Spot 109	615	311040	1.4	17.4385	0.6	0.6002	1.8	0.0759	1.7	0.94	471.7	7.9	477.4	7.0	504.8	14.1	471.7	7.9
Gleason_354-U1450-A98F-1-Spot 181	964	1497527	1.5	17.6593	0.7	0.5956	2.3	0.0763	2.2	0.95	473.9	10.0	474.5	8.7	477.0	15.6	473.9	10.0
Gleason_354-U1450-A98F-1-Spot 285	243	10892	2.7	17.1488	1.3	0.6141	2.7	0.0764	2.4	0.88	474.4	10.9	486.1	10.4	541.5	27.7	474.4	10.9
Gleason_354-U1450-A98F-1-Spot 108	820	41015	31.9	17.7227	0.6	0.5974	1.8	0.0768	1.7	0.95	476.9	7.9	475.6	6.9	469.1	12.7	476.9	7.9
Gleason_354-U1450-A98F-1-Spot 244	452	75676	2.1	17.4807	0.8	0.6064	2.3	0.0769	2.1	0.93	477.5	9.7	481.3	8.7	499.5	17.8	477.5	9.7
Gleason_354-U1450-A98F-1-Spot 197	520	26045	2.3	16.7236	1.5	0.6345	3.2	0.0770	2.8	0.88	477.9	13.1	498.9	12.7	596.2	32.9	477.9	13.1
Gleason_354-U1450-A98F-1-Spot 73	727	716992	2.1	17.3379	0.6	0.6146	1.9	0.0773	1.8	0.96	479.9	8.5	486.5	7.4	517.5	12.3	479.9	8.5
Gleason_354-U1450-A98F-1-Spot 71	496	55419	4.4	17.3754	0.9	0.6142	2.6	0.0774	2.4	0.94	480.6	11.2	486.2	9.9	512.8	19.3	480.6	11.2
Gleason_354-U1450-A98F-1-Spot 17	2509	312070	7.4	17.2907	0.7	0.6174	1.9	0.0774	1.8	0.94	480.7	8.5	488.2	7.5	523.5	14.5	480.7	8.5
Gleason_354-U1450-A98F-1-Spot 243	541	146523	3.6	17.4596	0.6	0.6118	2.8	0.0775	2.7	0.97	481.0	12.7	484.7	10.8	502.2	14.2	481.0	12.7
Gleason_354-U1450-A98F-1-Spot 167	1750	2569696	14.2	17.5663	0.7	0.6096	2.3	0.0777	2.1	0.95	482.2	10.0	483.3	8.7	488.7	15.0	482.2	10.0
Gleason_354-U1450-A98F-1-Spot 246	657	22002	1.3	17.7805	1.0	0.6043	3.0	0.0779	2.8	0.94	483.8	13.1	480.0	11.4	461.9	22.0	483.8	13.1
Gleason_354-U1450-A98F-1-Spot 18	337	1004656	1.7	17.3526	1.0	0.6197	3.0	0.0780	2.8	0.94	484.1	13.2	489.7	11.7	515.6	22.0	484.1	13.2
Gleason_354-U1450-A98F-1-Spot 121	427	36029	24.0	17.4558	0.7	0.6162	2.6	0.0780	2.5	0.96	484.3	11.6	487.5	10.0	502.6	15.7	484.3	11.6
Gleason_354-U1450-A98F-1-Spot 119	1842	296793	5.4	17.2355	0.6	0.6275	2.0	0.0784	1.9	0.96	486.8	9.0	494.6	7.9	530.5	12.9	486.8	9.0
Gleason_354-U1450-A98F-1-Spot 146	508	78992	2.4	17.2797	0.9	0.6282	2.2	0.0787	2.0	0.92	488.6	9.4	495.0	8.5	524.9	19.1	488.6	9.4
Gleason_354-U1450-A98F-1-Spot 37	753	58709	1.5	17.5323	0.8	0.6198	3.5	0.0788	3.4	0.97	489.0	16.0	489.7	13.6	493.0	18.3	489.0	16.0
Gleason_354-U1450-A98F-1-Spot 113	261	585103	3.1	17.6619	1.4	0.6171	4.1	0.0790	3.9	0.94	490.4	18.3	488.0	15.9	476.7	30.2	490.4	18.3
Gleason_354-U1450-A98F-1-Spot 223	620	341424	5.8	17.4571	0.7	0.6288	2.8	0.0796	2.7	0.97	493.8	13.1	495.3	11.1	502.5	16.3	493.8	13.1
Gleason_354-U1450-A98F-1-Spot 250	695	45888	3.2	17.5044	0.7	0.6280	2.2	0.0797	2.1	0.95	494.5	10.1	494.8	8.8	496.5	15.6	494.5	10.1
Gleason_354-U1450-A98F-1-Spot 25	306	49322	1.4	17.5833	0.9	0.6285	2.4	0.0801	2.2	0.93	497.0	10.6	495.1	9.4	486.6	19.8	497.0	10.6
Gleason_354-U1450-A98F-1-Spot 263	386	30366	2.5	17.1456	0.9	0.6473	2.6	0.0805	2.5	0.94	499.1	11.9	506.8	10.5	542.0	19.9	499.1	11.9
Gleason_354-U1450-A98F-1-Spot 220	2211	196662	4.9	17.3653	0.8	0.6449	2.7	0.0812	2.6	0.95	503.4	12.5	505.3	10.8	514.1	18.5	503.4	12.5
Gleason_354-U1450-A98F-1-Spot 297	603	48224	2.4	17.1890	0.9	0.6523	3.3	0.0813	3.2	0.96	504.0	15.3	509.9	13.2	536.4	20.8	504.0	15.3
Gleason_354-U1450-A98F-1-Spot 284	1403	1150355	3.0	17.1055	0.7	0.6597	2.7	0.0818	2.6	0.96	507.1	12.6	514.4	10.9	547.0	16.0	507.1	12.6
Gleason_354-U1450-A98F-1-Spot 218	6286	230278	55.8	17.5783	0.6	0.6434	1.7	0.0820	1.6	0.94	508.2	7.8	504.4	6.7	487.2	12.4	508.2	7.8
Gleason_354-U1450-A98F-1-Spot 54	3263	105149	3.3	17.3328	0.7	0.6532	1.9	0.0821	1.7	0.93	508.7	8.5	510.4	7.5	518.1	14.6	508.7	8.5
Gleason_354-U1450-A98F-1-Spot 24	205	23431	0.6	17.0785	1.0	0.6670	2.8	0.0826	2.6	0.94	511.7	13.0	518.9	11.4	550.5	20.8	511.7	13.0
Gleason_354-U1450-A98F-1-Spot 251	1563	226796	7.0	17.0561	0.8	0.6736	2.5	0.0833	2.4	0.95	516.0	12.0	522.9	10.4	553.4	16.9	516.0	12.0
Gleason_354-U1450-A98F-1-Spot 114	317	65546	2.6	17.2438	1.0	0.6750	2.7	0.0844	2.5	0.93	522.5	12.5	523.8	10.9	529.5	20.9	522.5	12.5
Gleason_354-U1450-A98F-1-Spot 66	116	165713	0.8	17.0369	0.8	0.6837	2.8	0.0845	2.6	0.95	522.8	13.2	529.0	11.4	555.9	18.4	522.8	13.2
Gleason_354-U1450-A98F-1-Spot 237	140	11649	4.7	17.3025	1.4	0.6961	6.5	0.0874	6.3	0.98	539.9	32.8	536.5	27.0	522.0	31.6	539.9	32.8
Gleason_354-U1450-A98F-1-Spot 33	237	128941	1.3	17.0792	0.9	0.7243	2.3	0.0897	2.1	0.92	553.9	11.0	553.2	9.6	550.5	19.3	553.9	11.0
Gleason_354-U1450-A98F-1-Spot 158	1401	7681529	21.9	16.4724	1.2	0.7515	2.8	0.0898	2.6	0.91	554.2	13.6	569.1	12.2	628.9	25.1	554.2	13.6
Gleason_354-U1450-A98F-1-Spot 45	1008	70010	9.7	16.3798	0.9	0.7592	5.7	0.0902	5.7	0.99	556.6	30.1	573.5	25.1	641.0	19.8	556.6	30.1
Gleason_354-U1450-A98F-1-Spot 304	54	6070	292.4	16.7062	1.3	0.7649	3.6	0.0927	3.3	0.93	571.4	18.1	576.8	15.8	598.4	29.1	571.4	18.1

Gleason_354-U1450-A98F-1-Spot 100	92	9403	2.2	16.5992	1.0	0.8136	3.5	0.0980	3.3	0.95	602.4	19.1	604.5	15.8	612.3	22.3	602.4	19.1
Gleason_354-U1450-A98F-1-Spot 137	723	63164	6.8	16.5569	0.6	0.8182	2.2	0.0983	2.1	0.97	604.2	12.2	607.1	10.0	617.8	12.4	604.2	12.2
Gleason_354-U1450-A98F-1-Spot 179	267	106187	1.7	16.4378	0.8	0.8541	2.5	0.1018	2.4	0.95	625.1	14.1	626.9	11.7	633.4	16.3	625.1	14.1
Gleason_354-U1450-A98F-1-Spot 279	430	70245	3.6	16.3322	0.7	0.8744	1.8	0.1036	1.7	0.92	635.3	10.3	638.0	8.7	647.3	15.1	635.3	10.3
Gleason_354-U1450-A98F-1-Spot 147	224	89426	3.3	15.9309	0.9	0.9089	2.5	0.1050	2.3	0.93	643.7	14.1	656.5	11.9	700.5	18.8	643.7	14.1
Gleason_354-U1450-A98F-1-Spot 61	232	123200	0.4	14.8067	0.7	1.1101	2.6	0.1192	2.5	0.96	726.0	16.9	758.2	13.7	854.4	14.5	726.0	16.9
Gleason_354-U1450-A98F-1-Spot 233	63	12609	2.6	15.5121	1.2	1.0615	3.3	0.1194	3.1	0.93	727.2	21.1	734.6	17.2	756.9	24.9	727.2	21.1
Gleason_354-U1450-A98F-1-Spot 136	381	31371	3.8	15.4295	0.7	1.0973	3.0	0.1228	2.9	0.97	746.6	20.8	752.0	16.2	768.2	15.7	746.6	20.8
Gleason_354-U1450-A98F-1-Spot 231	1763	356660	2.5	15.2683	0.7	1.1099	1.9	0.1229	1.8	0.93	747.3	12.5	758.1	10.2	790.3	15.3	747.3	12.5
Gleason_354-U1450-A98F-1-Spot 96	38	25719	1.6	15.1545	1.1	1.1217	4.0	0.1233	3.8	0.96	749.5	27.0	763.8	21.4	805.9	24.0	749.5	27.0
Gleason_354-U1450-A98F-1-Spot 1	2381	586828	1.6	15.1221	0.8	1.1402	2.3	0.1251	2.1	0.94	759.6	15.3	772.6	12.3	810.4	16.7	759.6	15.3
Gleason_354-U1450-A98F-1-Spot 193	1683	238809	22.1	15.2508	0.7	1.1457	2.1	0.1267	2.0	0.95	769.1	14.3	775.2	11.3	792.7	13.9	769.1	14.3
Gleason_354-U1450-A98F-1-Spot 222	218	51537	1.9	14.7313	0.7	1.1898	2.8	0.1271	2.7	0.97	771.4	19.7	795.9	15.5	865.0	15.2	771.4	19.7
Gleason_354-U1450-A98F-1-Spot 62	1307	910992	1.1	14.8586	0.9	1.1833	2.4	0.1275	2.2	0.93	773.7	16.1	792.9	13.1	847.1	18.7	773.7	16.1
Gleason_354-U1450-A98F-1-Spot 111	1888	2905061	1.4	14.7416	0.6	1.2125	1.8	0.1296	1.7	0.95	785.8	12.9	806.3	10.2	863.5	12.3	785.8	12.9
Gleason_354-U1450-A98F-1-Spot 23	1179	62409	1.5	14.8219	0.6	1.2145	2.2	0.1306	2.1	0.96	791.0	15.9	807.2	12.4	852.2	12.4	791.0	15.9
Gleason_354-U1450-A98F-1-Spot 226	168	41342	1.3	15.2322	0.8	1.1819	2.8	0.1306	2.7	0.96	791.1	19.8	792.2	15.3	795.2	16.6	791.1	19.8
Gleason_354-U1450-A98F-1-Spot 144	601	50884	1.8	15.2311	0.6	1.1909	2.2	0.1316	2.1	0.97	796.7	15.7	796.4	12.0	795.4	11.7	796.7	15.7
Gleason_354-U1450-A98F-1-Spot 282	789	104059	1.7	15.0446	0.6	1.2060	2.2	0.1316	2.1	0.96	796.9	15.7	803.3	12.1	821.2	12.5	796.9	15.7
Gleason_354-U1450-A98F-1-Spot 16	596	4504234	1.7	14.9935	0.7	1.2107	2.1	0.1317	2.0	0.95	797.3	15.1	805.5	11.8	828.3	13.7	797.3	15.1
Gleason_354-U1450-A98F-1-Spot 87	1248	759302	2.2	14.8266	0.6	1.2253	1.9	0.1318	1.8	0.95	797.9	13.4	812.2	10.5	851.6	12.2	797.9	13.4
Gleason_354-U1450-A98F-1-Spot 124	428	1845563	1.1	14.7667	0.7	1.2430	2.1	0.1331	1.9	0.94	805.7	14.7	820.2	11.6	860.0	14.8	805.7	14.7
Gleason_354-U1450-A98F-1-Spot 228	360	110242	2.7	14.9905	0.7	1.2247	2.0	0.1332	1.9	0.94	805.8	14.3	811.9	11.2	828.7	14.4	805.8	14.3
Gleason_354-U1450-A98F-1-Spot 300	225	28148	0.8	15.1553	0.7	1.2119	1.9	0.1332	1.8	0.93	806.2	13.4	806.1	10.6	805.8	15.1	806.2	13.4
Gleason_354-U1450-A98F-1-Spot 230	259	119380	6.1	15.0036	1.6	1.2325	3.0	0.1341	2.6	0.86	811.3	19.8	815.5	17.0	826.9	32.6	811.3	19.8
Gleason_354-U1450-A98F-1-Spot 242	265	171440	1.7	15.0163	0.6	1.2326	1.9	0.1342	1.8	0.94	812.0	13.7	815.5	10.7	825.1	13.1	812.0	13.7
Gleason_354-U1450-A98F-1-Spot 227	848	357012	1.2	14.8567	0.7	1.2556	2.0	0.1353	1.8	0.94	818.0	14.2	825.9	11.1	847.4	14.1	818.0	14.2
Gleason_354-U1450-A98F-1-Spot 221	278	322594	5.6	14.8410	0.9	1.2584	2.6	0.1355	2.4	0.94	818.9	18.8	827.2	14.8	849.6	19.1	818.9	18.8
Gleason_354-U1450-A98F-1-Spot 298	1015	117562	0.5	14.6357	0.7	1.2786	2.0	0.1357	1.9	0.93	820.4	14.4	836.2	11.4	878.5	15.0	820.4	14.4
Gleason_354-U1450-A98F-1-Spot 260	167	1303120	2.5	14.8004	0.9	1.2698	3.1	0.1363	2.9	0.95	823.7	22.5	832.3	17.4	855.2	19.4	823.7	22.5
Gleason_354-U1450-A98F-1-Spot 84	168	15488	2.9	15.2341	0.9	1.2413	2.7	0.1372	2.5	0.94	828.5	19.6	819.5	15.0	795.0	18.5	828.5	19.6
Gleason_354-U1450-A98F-1-Spot 67	572	89913	2.5	14.7606	1.0	1.3037	2.4	0.1396	2.2	0.92	842.2	17.1	847.3	13.6	860.8	19.7	842.2	17.1
Gleason_354-U1450-A98F-1-Spot 81	593	165993	16.8	14.8989	0.7	1.2932	2.3	0.1397	2.2	0.95	843.2	17.1	842.7	13.0	841.5	14.8	843.2	17.1
Gleason_354-U1450-A98F-1-Spot 141	914	325735	0.7	14.8579	1.0	1.2996	2.5	0.1400	2.3	0.92	844.9	18.5	845.6	14.6	847.2	20.8	844.9	18.5
Gleason_354-U1450-A98F-1-Spot 131	498	288272	5.9	14.6497	0.6	1.3232	2.1	0.1406	2.0	0.96	848.0	16.1	855.9	12.2	876.5	12.2	848.0	16.1
Gleason_354-U1450-A98F-1-Spot 178	399	94471	1.6	14.7433	0.7	1.3221	2.1	0.1414	2.0	0.95	852.4	15.8	855.4	12.1	863.3	13.6	852.4	15.8
Gleason_354-U1450-A98F-1-Spot 27	347	110984	4.6	14.7091	0.7	1.3292	2.2	0.1418	2.1	0.95	854.8	17.0	858.5	13.0	868.1	14.5	854.8	17.0
Gleason_354-U1450-A98F-1-Spot 104	626	187076	4.4	14.5031	0.7	1.3499	2.5	0.1420	2.4	0.95	855.9	18.9	867.5	14.4	897.2	15.1	855.9	18.9
Gleason_354-U1450-A98F-1-Spot 248	415	143606	4.3	14.8628	0.7	1.3200	2.3	0.1423	2.2	0.96	857.6	17.9	854.5	13.5	846.5	14.1	857.6	17.9
Gleason_354-U1450-A98F-1-Spot 177	339	130679	2.0	14.6479	0.8	1.3507	2.8	0.1435	2.7	0.96	864.4	22.0	867.9	16.5	876.7	15.9	864.4	22.0
Gleason_354-U1450-A98F-1-Spot 78	68	88344	1.9	14.4068	0.9	1.3749	3.6	0.1437	3.5	0.96	865.3	28.0	878.2	21.1	911.0	19.4	865.3	28.0
Gleason_354-U1450-A98F-1-Spot 303	276	73570	2.1	14.4384	0.9	1.3755	2.3	0.1440	2.1	0.92	867.4	17.2	878.5	13.5	906.5	18.0	867.4	17.2
Gleason_354-U1450-A98F-1-Spot 112	186	27259	2.2	14.6660	1.0	1.3787	2.7	0.1467	2.5	0.93	882.2	20.7	879.9	15.9	874.2	20.5	882.2	20.7
Gleason_354-U1450-A98F-1-Spot 130	148	33425	0.7	14.5892	0.8	1.3882	2.4	0.1469	2.3	0.94	883.5	18.6	883.9	14.2	885.0	17.1	883.5	18.6
Gleason_354-U1450-A98F-1-Spot 277	157	108208	1.9	14.6050	0.6	1.3945	2.8	0.1477	2.8	0.98	888.1	23.0	886.6	16.8	882.8	11.7	888.1	23.0
Gleason_354-U1450-A98F-1-Spot 52	202	96009	1.2	14.3742	0.8	1.5131	2.8	0.1577	2.7	0.96	944.2	23.5	935.7	17.1	915.7	16.0	915.7	16.0
Gleason_354-U1450-A98F-1-Spot 72	82	11445	0.8	14.2967	1.1	1.4337	4.6	0.1487	4.5	0.97	893.4	37.7	903.1	27.8	926.8	21.9	926.8	21.9
Gleason_354-U1450-A98F-1-Spot 205	1668	1828701	16.5	14.2390	0.7	1.5715	2.9	0.1623	2.8	0.97	969.5	25.5	959.0	18.1	935.1	14.2	935.1	14.2
Gleason_354-U1450-A98F-1-Spot 14	254	45251	1.5	14.1311	0.7	1.5272	1.8	0.1565	1.7	0.93	937.4	14.8	941.4	11.2	950.7	14.1	950.7	14.1
Gleason_354-U1450-A98F-1-Spot 187	375	47590	4.0	14.1068	0.7	1.5915	2.2	0.1628	2.1	0.94	972.5	18.8	966.9	13.8	954.2	15.0	954.2	15.0
Gleason_354-U1450-A98F-1-Spot 186	165	37540	1.5	14.0574	0.9	1.6543	2.6	0.1687	2.5	0.94	1004.7	23.2	991.2	16.8	961.4	18.1	961.4	18.1
Gleason_354-U1450-A98F-1-Spot 74	132	26188	2.1	14.0132	0.6	1.4173	2.2	0.1440	2.1	0.96	867.5	17.1	896.2	13.1	967.8	12.5	967.8	12.5
Gleason_354-U1450-A98F-1-Spot 90	239	30957	2.4	13.9827	0.6	1.5017	2.3	0.1523	2.2	0.97	913.8	18.8	931.1	13.9	972.2	12.1	972.2	12.1
Gleason_354-U1450-A98F-1-Spot 169	85	26228	1.3	13.9784	0.7	1.6547	3.2	0.1678	3.2	0.98	999.7	29.4	991.4	20.6	972.9	14.0	972.9	14.0
Gleason_354-U1450-A98F-1-Spot 184	599	274276	4.6	13.9220	0.6	1.6238	1.9	0.1640	1.8	0.94	978.8	16.1	979.5	11.8	981.1	12.6	981.1	12.6
Gleason_354-U1450-A98F-1-Spot 240	434	129367	2.7	13.9210	0.7	1.5997	2.0	0.1615	1.9	0.94	965.2	17.1	970.1	12.7	981.3	14.6	981.3	14.6
Gleason_354-U1450-A98F-1-Spot 102	222	29957	1.7	13.8996	0.8	1.6467	2.4	0.1660	2.3	0.94	990.0	21.1	988.3	15.4	984.4	16.5	984.4	16.5
Gleason_354-U1450-A98F-1-Spot 308	456	159252	4.9	13.8725	0.6	1.4349	2.3	0.1444	2.2	0.96	869.3	17.7	903.6	13.5	988.3	12.6	988.3	12.6
Gleason_354-U1450-A98F-1-Spot 126	125	20299	2.1	13.8421	0.8	1.7010	2.2	0.1708	2.0	0.93	1016.4	19.2	1008.9	14.0	992.8	15.9	992.8	15.9
Gleason_354-U1450-A98F-1-Spot 287	395	90481	5.1	13.7890	0.8	1.6312	2.4	0.1631	2.3	0.94	974.1	20.7	982.3	15.4	1000.6	17.0	1000.6	17.0
Gleason_354-U1450-A98F-1-Spot 4	327	70876	3.8	13.7682	0.6	1.6342	2.1	0.1632	2.0	0.96	974.4	18.5	983.5	13.5	1003.7	12.3	1003.7	12.3
Gleason_354-U1450-A98F-1-Spot 148	154	19145	1.4	13.7522	1.1	1.5650	3.8	0.1561	3.7	0.96	935.0	32.0	956.4	23.8	1006.0	22.9	1006.0	22.9
Gleason_354-U1450-A98F-1-Spot 76	799	100482	4.5	13.7463	0.6	1.6132	2.1	0.1608	2.0	0.96	961.4	17.7	975.4	13.0	1006.9	12.4	1006.9	12.4
Gleason_354-U1450-A98F-1-Spot 56	272	322137	1.5	13.7463	0.7	1.6486	2.4	0.1644	2.3	0.96	981.0	20.7	989.0	15.0	1006.9	13.7	1006.9	13.7
Gleason_354-U1450-A98F-1-Spot 219	866	122013	1.1	13.7280	0.8	1.6682	2.4	0.1661	2.3	0.95	990.5	20.9	996.5	15.2	1009.6	15.4	1009.6	15.4
Gleason_354-U1450-A98F-1-Spot 296	462	84074	1.4	13.7166	0.8	1.6234	2.6	0.1615	2.4	0.95	965.1	21.9	979.3	16.2	1011.3	16.7	1011.3	16.7
Gleason_354-U1450-A98F-1-Spot 139	81	8255	0.7	13.6702	2.4	1.4963	4.5	0.1484	3.9	0.86	891.7	32.4	928.9	27.7	1018.1	47.7	1018.1	47.7
Gleason_354-U1450-A98F-1-Spot 309	328	82867	5.2	13.6590	0.6	1.6435	1.8	0.1628	1.7	0.94	972.4	15.0	987.0	11.2	1019.8	12.4	1019.8	12.4
Gleason_354-U1450-A98F-1-Spot 93	88	6546	1.9	13.4523	0.8	1.7482	3.1	0.1706	3.0	0.97	1015.2	27.9	1026.5	19.8	1050.6	15.5	1050.6	15.5
Gleason_354-U1450-A98F-1-Spot 70	222	92690	2.0	13.4067	0.6	1.7996	2.4	0.1750	2.3	0.96	1039.5	21.8	1045.3	15.4	1057.5	12.8	1057.5	12.8
Gleason_354-U1450-A98F-1-Spot 215	1795	491181	5.1	13.3050	0.8	1.5915	4.2	0.1536	4.1	0.98	921.0	34.9	966.9	25.9	1072.8	17.0	1072.8	17.0
Gleason_354-U1450-A98F-1-Spot 257	2481	173127	3.4	13.2807	0.6	1.4985	2.4	0.1443	2.3	0.96	869.2	18.6	929.8	14.5	1076.5	12.8	1076.5	12.8
Gleason_354-U1450-A98F-1-Spot 75	468	80366	3.8	13.2784	0.6	1.9011	1.8	0.1831	1.6	0.94	1083.8	16.4	1081.5	11.7	1076.8	12.5	1076.8	12.5
Gleason_354-U1450-A98F-1-Spot 82	160	26256	1.4	13.2104	0.8	1.9284	2.4	0.1848	2.2	0.94	1093.0	22.5	1091.0	16.0	1087.1	16.8	1087.1	16.8
Gleason_354-U1450-A98F-1-Spot 30	103	22929	2.7	13.2099	0.8	1.9458	2.7	0.1864	2.6	0.95	1101.9	26.0	1097.0	18.1	1087.2	16.6	1087.2	16.6
Gleason_354-U1450-A98F-1-Spot 164	667	106771	2.6	13.2057	0.8	1.6814	2.6	0.1610	2.5	0.95	962.5	22.4	1001.5	16.8	1087.8	16.4	1087.8	16.4
Gleason_354-U1450-A98F-1-Spot 202	304	143663	1.1	13.1785	0.7	1.6813	2.5	0.1607	2.3	0.95	960.6	20.9	1001.5	15.6	1091.9	14.8	1091.9	14.8
Gleason_354-U1450-A98F-1-Spot 59	328	47665	2.2	13.0727	0.8	1.8984	2.5	0.1800	2.4	0.95	1066.9	23.4	1080.5	16.6	1108.0	15.6	1108.0	15.6
Gleason_354-U1450-A98F-1-Spot 210	194	32928	0.7	12.9320	0.6	2.0928	2.4	0.1963	2.4	0.97	1155.4	24.9	1146.5	16.7	1129.6	12.4	1129.6	12.4
Gleason_354-U1450-A98F-1-Spot 268	1322	92813	1.9	12.9048	0.7	1.9221	2.2	0.1799	2.1	0.95	1066.4	20.7	1088.8	14.8	1133.8	13.5	1133.8	13.5
Gleason_354-U1450-A98F-1-Spot 101	184	150826	1.2	12.8852	0.8	2.1204	2.3	0.1982	2.1	0.93	1165.4	22.7	1155.5	15.7	1136.9	16.3	1136.9	16.3
Gleason_354-U1450-A98F-1-Spot 200	1379	332259	3.0	12.7474	0.9	1.8039	2.3	0.1668	2.2	0.92	994.3	19.9	1046.9	15.3	1158.2	18.0	1158.2	18.0
Gleason_354-U1450-A98F-1-Spot 253	200	71125	1.0	12.7308	0.7	2.1542	2.6	0.1989	2.5	0.96	1169.4	26.3	1166.4	17.8	1160.8	14.5	1160.8	14.5
Gleason_354-U1450-A98F-1-Spot 188	72	73302	0.5	12.7246	1.0	2.1713	3.7	0.2004	3.5	0.96	1177.4	38.0	1171.9	25.5	1161.8	19.5	1161.8	19.5
Gleason_354-U1450-A98F-1-Spot 301	756	67704	1.9	12.6699	1.0	2.0130	2.3	0.1850	2.1	0.90	1094.1	21.0	1119.9	15.7	1170.3	19.5	1170.3	19.5
Gleason_354-U1450-A98F-1-Spot 259	268	75948	1.8	12.6585	0.5	2.1548	2.4	0.1978	2.3	0.97	1163.6	24.4	1166.6	16.3	1172.1	10.9	1172.1	10.9
Gleason_354-U1450-A98F-1-Spot 212	513	374602	2.2	12.6313	0.8	2.1809	2.1	0.1998	2.0	0.92	1174.2	21.2	1175.0	14.9	1176.4	16.4	1176.4	16.4
Gleason_354-U1450-A98F-1-Spot 138	464	187178	2.9	12.6174	0.7	2.0209	4.7	0.1849	4.7	0.99	1093.9	47.2	1122.6	32.2	1178.5	13.5	1178.5	13.5
Gleason_354-U1450-A98F-1-Spot 213	489	480431	1.9	12.6145	0.8	2.0381	2.6	0.1865	2.5	0.96	1102.2	24.9	1128.3	17.5	1179.0	14.9	1179.0	14.9
Gleason_354-U1450-A98F-1-Spot 69	95	826234	1.0	12.5974	0.8	2.1224	2.6	0.1939	2.4	0.95	1142.6	25.4	1156.1	17.6	1181.7	15.9	1181.7	15.9
Gleason_354-U1450-A98F-1-Spot 239	820	38591	15.3	12.5904	0.9	2.1014	2.3	0.1919	2.1	0.91	1131.6	21.3	1149.3	15.5	1182.8	18.2	1182.8	18.2
Gleason_354-U1450-A98F-1-Spot 289	656	87909	2.7	12.4879	0.8	1.8479	2.6	0.1674	2.5	0.95	997.6	22.9	1062.7	17.2	1198.9	15.7	1198.9	15.7
Gleason_354-U1450-A98F-1-Spot 191	93	12698	1.0	12.4735	1.0	2.3121	3.0	0.2092	2.9	0.95	1224.4	31.9	1216.0	21.4	1201.2	19.0	1201.2	19.0
Gleason_354-U1450-A98F-1-Spot 314	469	321005	0.8	12.4675	0.6	2.2069	1.9	0.1995	1.8	0.95	1172.9	19.5	1183.2	13.3	1202.1	11.2	1202.1	11.2
Gleason_354-U1450-A98F-1-Spot 255	182	167871	11.7	12.4199	0.6	2.3198	3.0	0.2090	2.9	0.98	1223.3	32.4	1218.4	21.1	1209.7	11.9	1209.7	11.9

Gleason_354-U1450-A98F-1-Spot 195	975	184267	8.1	12.3001	0.8	2.2027	2.2	0.1965	2.0	0.94	1156.5	21.7	1181.9	15.3	1228.7	15.2	1228.7	15.2
Gleason_354-U1450-A98F-1-Spot 122	147	201118	1.5	12.1039	0.8	2.5547	2.3	0.2243	2.1	0.93	1304.4	25.0	1287.8	16.6	1260.2	15.9	1260.2	15.9
Gleason_354-U1450-A98F-1-Spot 94	575	209816	13.8	12.0687	0.6	2.3153	2.3	0.2027	2.2	0.96	1189.6	23.8	1217.0	16.2	1265.9	12.6	1265.9	12.6
Gleason_354-U1450-A98F-1-Spot 173	1049	266143	1.2	11.9507	0.7	2.3259	2.1	0.2016	1.9	0.94	1183.9	21.0	1220.2	14.7	1285.0	13.7	1285.0	13.7
Gleason_354-U1450-A98F-1-Spot 44	486	100704	2.0	11.8455	0.9	2.1578	3.2	0.1854	3.0	0.96	1096.3	30.5	1167.6	21.9	1302.2	17.8	1302.2	17.8
Gleason_354-U1450-A98F-1-Spot 159	268	133992	2.1	11.6190	0.6	2.6902	2.0	0.2267	1.9	0.96	1317.2	23.0	1325.8	14.9	1339.7	11.2	1339.7	11.2
Gleason_354-U1450-A98F-1-Spot 19	179	353147	1.8	11.6014	0.6	2.7932	2.3	0.2350	2.2	0.97	1360.8	27.2	1353.7	17.1	1342.6	11.0	1342.6	11.0
Gleason_354-U1450-A98F-1-Spot 247	139	439975	2.5	11.5464	0.8	2.8805	3.0	0.2412	2.9	0.96	1393.0	36.4	1376.8	22.8	1351.8	16.0	1351.8	16.0
Gleason_354-U1450-A98F-1-Spot 209	103	17743	1.2	11.5031	0.8	2.7702	3.5	0.2311	3.4	0.97	1340.3	41.6	1347.5	26.4	1359.0	16.3	1359.0	16.3
Gleason_354-U1450-A98F-1-Spot 115	231	490059	3.0	11.4750	0.6	2.6277	2.4	0.2187	2.3	0.97	1274.9	26.8	1308.4	17.6	1363.7	11.2	1363.7	11.2
Gleason_354-U1450-A98F-1-Spot 180	359	75530	8.3	11.4704	0.7	2.8639	2.4	0.2383	2.3	0.96	1377.6	28.1	1372.5	17.8	1364.5	13.0	1364.5	13.0
Gleason_354-U1450-A98F-1-Spot 280	1200	174239	22.9	11.3946	0.6	2.3827	1.8	0.1969	1.7	0.95	1158.7	18.2	1237.4	12.9	1377.2	11.0	1377.2	11.0
Gleason_354-U1450-A98F-1-Spot 291	480	1216516	2.1	11.3935	0.5	2.4619	2.0	0.2034	2.0	0.96	1193.8	21.4	1260.9	14.7	1377.4	10.4	1377.4	10.4
Gleason_354-U1450-A98F-1-Spot 49	80	12091	0.9	11.1697	0.8	2.8854	3.4	0.2338	3.3	0.97	1354.1	39.8	1378.1	25.4	1415.5	15.6	1415.5	15.6
Gleason_354-U1450-A98F-1-Spot 79	413	218572	1.8	11.0776	0.9	3.1200	2.1	0.2507	1.9	0.90	1441.9	24.3	1437.6	16.0	1431.3	16.9	1431.3	16.9
Gleason_354-U1450-A98F-1-Spot 63	115	45386	1.1	11.0405	1.2	3.1059	3.6	0.2487	3.4	0.94	1431.8	43.1	1434.2	27.3	1437.7	22.5	1437.7	22.5
Gleason_354-U1450-A98F-1-Spot 127	175	40134	2.0	11.0183	0.7	3.1097	2.1	0.2485	2.0	0.94	1430.8	25.8	1435.1	16.4	1441.5	13.4	1441.5	13.4
Gleason_354-U1450-A98F-1-Spot 36	337	85535	2.1	10.9870	0.8	3.0135	2.8	0.2401	2.7	0.96	1387.4	33.2	1411.0	21.2	1446.9	14.9	1446.9	14.9
Gleason_354-U1450-A98F-1-Spot 183	135	51499	1.0	10.5700	0.9	3.5836	2.4	0.2747	2.2	0.93	1564.7	30.4	1545.9	18.8	1520.2	16.8	1520.2	16.8
Gleason_354-U1450-A98F-1-Spot 315	269	29478	1.5	10.3441	0.7	2.8969	5.4	0.2173	5.3	0.99	1267.8	61.3	1381.1	40.5	1560.9	12.9	1560.9	12.9
Gleason_354-U1450-A98F-1-Spot 306	51	71452	1.0	10.3433	0.7	3.3540	3.3	0.2516	3.2	0.98	1446.8	41.5	1493.7	25.7	1561.0	13.4	1561.0	13.4
Gleason_354-U1450-A98F-1-Spot 11	1681	271577	8.9	10.3337	0.7	3.3134	2.3	0.2483	2.2	0.96	1429.9	28.2	1484.2	17.9	1562.8	12.3	1562.8	12.3
Gleason_354-U1450-A98F-1-Spot 307	333	164491	1.7	10.3084	0.6	3.3700	2.0	0.2520	1.9	0.96	1448.6	24.8	1497.5	15.6	1567.4	11.1	1567.4	11.1
Gleason_354-U1450-A98F-1-Spot 154	219	153456	1.7	10.2692	0.7	3.7874	2.3	0.2821	2.2	0.96	1601.9	31.6	1590.1	18.7	1574.5	12.5	1574.5	12.5
Gleason_354-U1450-A98F-1-Spot 311	342	1255637	0.9	10.2613	0.8	3.4949	2.0	0.2601	1.8	0.92	1490.3	24.2	1526.1	15.6	1575.9	14.1	1575.9	14.1
Gleason_354-U1450-A98F-1-Spot 50	909	288824	2.6	10.2556	0.8	3.6545	2.4	0.2718	2.2	0.94	1550.0	30.6	1561.5	18.8	1577.0	14.8	1577.0	14.8
Gleason_354-U1450-A98F-1-Spot 249	178	78293	2.3	10.1284	0.8	3.8750	3.1	0.2846	3.0	0.96	1614.7	42.4	1608.5	24.9	1600.3	15.8	1600.3	15.8
Gleason_354-U1450-A98F-1-Spot 155	511	1250484	4.2	10.1203	0.7	3.2323	1.9	0.2372	1.8	0.94	1372.4	22.3	1464.9	14.9	1601.8	12.6	1601.8	12.6
Gleason_354-U1450-A98F-1-Spot 42	99	23487	2.0	10.0940	0.7	3.7893	2.2	0.2774	2.1	0.95	1578.3	28.8	1590.5	17.4	1606.6	12.8	1606.6	12.8
Gleason_354-U1450-A98F-1-Spot 165	108	120231	1.3	10.0852	0.8	3.7874	3.4	0.2770	3.3	0.97	1576.4	46.5	1590.1	27.5	1608.3	14.9	1608.3	14.9
Gleason_354-U1450-A98F-1-Spot 68	88	44301	1.3	10.0504	0.5	3.8383	2.5	0.2798	2.4	0.98	1590.3	34.1	1600.8	20.0	1614.7	10.2	1614.7	10.2
Gleason_354-U1450-A98F-1-Spot 46	94	42524	1.3	10.0203	1.0	3.7530	3.8	0.2727	3.6	0.97	1554.7	50.3	1582.7	30.2	1620.3	17.9	1620.3	17.9
Gleason_354-U1450-A98F-1-Spot 48	409	64232	13.8	10.0155	0.9	3.4458	2.5	0.2503	2.4	0.94	1440.0	30.7	1514.9	20.0	1621.2	16.4	1621.2	16.4
Gleason_354-U1450-A98F-1-Spot 276	284	156274	1.8	9.9997	0.6	3.9876	1.8	0.2892	1.7	0.94	1637.5	24.6	1631.7	14.7	1624.1	11.3	1624.1	11.3
Gleason_354-U1450-A98F-1-Spot 166	246	373431	1.4	9.9810	0.7	3.8461	2.1	0.2784	2.0	0.95	1583.4	28.0	1602.5	17.0	1627.6	12.5	1627.6	12.5
Gleason_354-U1450-A98F-1-Spot 55	395	153297	2.2	9.9689	0.9	3.9137	3.5	0.2830	3.4	0.97	1606.3	48.1	1616.5	28.2	1629.9	16.0	1629.9	16.0
Gleason_354-U1450-A98F-1-Spot 85	184	74977	1.6	9.9503	0.9	3.9994	2.8	0.2886	2.7	0.95	1634.6	38.6	1634.1	23.0	1633.3	17.2	1633.3	17.2
Gleason_354-U1450-A98F-1-Spot 313	489	128739	1.4	9.9493	0.6	3.7802	2.1	0.2728	2.0	0.95	1554.9	27.5	1588.5	16.8	1633.5	12.1	1633.5	12.1
Gleason_354-U1450-A98F-1-Spot 99	78	33927	1.9	9.9151	0.9	3.8725	3.2	0.2785	3.1	0.96	1583.7	43.8	1608.0	26.2	1639.9	16.6	1639.9	16.6
Gleason_354-U1450-A98F-1-Spot 140	569	79636	2.1	9.9009	0.7	3.6460	2.5	0.2618	2.4	0.96	1499.1	31.6	1559.6	19.6	1642.6	12.4	1642.6	12.4
Gleason_354-U1450-A98F-1-Spot 261	307	117414	1.6	9.8704	0.6	3.6166	2.2	0.2589	2.1	0.95	1484.2	27.4	1553.2	17.2	1648.3	11.9	1648.3	11.9
Gleason_354-U1450-A98F-1-Spot 294	327	43575	2.2	9.8357	0.8	3.7320	2.8	0.2662	2.6	0.96	1521.6	35.9	1578.3	22.2	1654.8	15.1	1654.8	15.1
Gleason_354-U1450-A98F-1-Spot 217	160	43900	2.2	9.8295	0.7	3.8676	2.3	0.2757	2.2	0.95	1569.8	31.1	1606.9	18.9	1656.0	13.1	1656.0	13.1
Gleason_354-U1450-A98F-1-Spot 185	266	93787	1.7	9.7826	0.7	4.0146	2.4	0.2848	2.3	0.96	1615.7	32.4	1637.2	19.2	1664.8	12.7	1664.8	12.7
Gleason_354-U1450-A98F-1-Spot 190	889	320025	1.7	9.7043	0.8	4.0781	2.3	0.2870	2.2	0.93	1626.6	31.4	1649.9	19.0	1679.7	15.4	1679.7	15.4
Gleason_354-U1450-A98F-1-Spot 160	169	77782	1.4	9.5670	0.7	4.2772	3.1	0.2968	3.0	0.97	1675.3	45.0	1689.0	25.8	1706.0	13.3	1706.0	13.3
Gleason_354-U1450-A98F-1-Spot 252	107	177445	1.8	9.4957	0.8	4.3775	2.7	0.3015	2.6	0.96	1698.6	38.8	1708.1	22.5	1719.7	14.8	1719.7	14.8
Gleason_354-U1450-A98F-1-Spot 258	236	420991	2.3	9.4229	0.8	4.4600	2.4	0.3048	2.2	0.93	1715.1	33.2	1723.6	19.6	1733.9	15.5	1733.9	15.5
Gleason_354-U1450-A98F-1-Spot 58	291	103100	1.5	9.4035	0.9	4.0217	3.2	0.2743	3.1	0.96	1562.5	42.5	1638.6	25.9	1737.6	15.7	1737.6	15.7
Gleason_354-U1450-A98F-1-Spot 95	329	82774	5.2	9.3832	0.7	4.3872	2.3	0.2986	2.2	0.95	1684.2	32.4	1709.9	19.0	1741.6	12.5	1741.6	12.5
Gleason_354-U1450-A98F-1-Spot 274	175	50306	1.8	9.3590	0.8	4.6049	2.9	0.3126	2.8	0.96	1753.4	43.5	1750.2	24.5	1746.3	14.1	1746.3	14.1
Gleason_354-U1450-A98F-1-Spot 273	192	11479	1.0	9.3130	1.1	3.9932	3.4	0.2697	3.2	0.94	1539.4	43.9	1632.8	27.6	1755.4	20.8	1755.4	20.8
Gleason_354-U1450-A98F-1-Spot 189	242	78486	7.9	9.2857	0.7	4.7530	2.5	0.3201	2.4	0.96	1790.2	36.9	1776.6	20.6	1760.7	12.1	1760.7	12.1
Gleason_354-U1450-A98F-1-Spot 149	876	33614	3.6	9.2699	0.8	3.7133	9.3	0.2497	9.3	1.00	1436.7	119.4	1574.2	74.6	1763.8	15.2	1763.8	15.2
Gleason_354-U1450-A98F-1-Spot 176	228	60089	1.4	9.2610	0.7	4.5327	2.5	0.3044	2.4	0.96	1713.3	35.5	1737.0	20.4	1765.6	12.6	1765.6	12.6
Gleason_354-U1450-A98F-1-Spot 203	491	1516820	2.4	9.2371	0.6	4.5636	2.5	0.3057	2.4	0.97	1719.7	36.5	1742.6	20.9	1770.3	11.8	1770.3	11.8
Gleason_354-U1450-A98F-1-Spot 199	1264	523645	38.3	9.2035	0.9	4.2911	2.4	0.2864	2.2	0.92	1623.7	31.6	1691.7	19.6	1777.0	16.8	1777.0	16.8
Gleason_354-U1450-A98F-1-Spot 91	145	51167	1.1	9.1913	0.6	4.7519	2.6	0.3168	2.5	0.97	1773.9	39.4	1776.4	21.9	1779.4	11.4	1779.4	11.4
Gleason_354-U1450-A98F-1-Spot 241	360	112807	4.6	8.9439	1.0	4.6314	2.5	0.3004	2.3	0.92	1693.4	34.6	1754.9	21.0	1829.0	17.4	1829.0	17.4
Gleason_354-U1450-A98F-1-Spot 57	527	88543	1.5	8.9041	0.7	4.9218	2.6	0.3178	2.5	0.97	1779.2	38.7	1806.0	21.8	1837.1	12.1	1837.1	12.1
Gleason_354-U1450-A98F-1-Spot 172	650	155628	3.9	8.8986	0.6	4.7830	2.9	0.3087	2.8	0.98	1734.2	42.8	1781.9	24.2	1838.2	11.3	1838.2	11.3
Gleason_354-U1450-A98F-1-Spot 232	188	422853	2.0	8.8578	0.5	5.2428	2.2	0.3368	2.2	0.97	1871.3	34.9	1859.6	18.9	1846.5	9.9	1846.5	9.9
Gleason_354-U1450-A98F-1-Spot 134	492	805328	3.8	8.8157	0.7	5.0137	2.5	0.3206	2.4	0.96	1792.5	37.4	1821.6	21.1	1855.1	13.1	1855.1	13.1
Gleason_354-U1450-A98F-1-Spot 175	201	594948	4.5	8.7998	0.7	5.2589	2.6	0.3356	2.5	0.96	1865.6	40.0	1862.2	21.9	1858.4	12.4	1858.4	12.4
Gleason_354-U1450-A98F-1-Spot 117	440	109306	6.4	8.7224	0.9	4.9663	2.6	0.3142	2.4	0.94	1761.2	37.7	1813.6	22.0	1874.3	15.8	1874.3	15.8
Gleason_354-U1450-A98F-1-Spot 204	300	84828	3.4	8.7212	0.6	5.2264	2.3	0.3306	2.2	0.96	1841.2	34.9	1856.9	19.3	1874.6	11.1	1874.6	11.1
Gleason_354-U1450-A98F-1-Spot 105	332	113316	2.9	8.5814	0.6	5.3645	2.3	0.3339	2.2	0.97	1857.1	36.1	1879.2	19.8	1903.7	10.9	1903.7	10.9
Gleason_354-U1450-A98F-1-Spot 80	787	124052	8.9	8.5043	0.7	4.7221	3.5	0.2913	3.4	0.98	1647.8	49.5	1771.2	29.2	1919.9	13.2	1919.9	13.2
Gleason_354-U1450-A98F-1-Spot 135	340	130443	4.1	8.2840	0.6	5.5346	1.9	0.3325	1.8	0.95	1850.6	29.7	1906.0	16.7	1966.8	10.7	1966.8	10.7
Gleason_354-U1450-A98F-1-Spot 12	148	112756	2.0	8.2541	0.7	5.3212	2.6	0.3185	2.5	0.96	1782.6	39.3	1872.3	22.4	1973.2	12.3	1973.2	12.3
Gleason_354-U1450-A98F-1-Spot 271	22	17689	3.9	7.8473	1.5	6.6121	4.7	0.3763	4.5	0.95	2059.1	79.0	2061.0	41.8	2062.8	27.3	2062.8	27.3
Gleason_354-U1450-A98F-1-Spot 293	183	328237	1.2	6.9191	0.8	7.9927	2.4	0.4011	2.3	0.94	2174.0	41.7	2230.2	21.7	2282.2	13.9	2282.2	13.9
Gleason_354-U1450-A98F-1-Spot 265	145	34179	2.4	6.8382	0.6	8.7486	2.4	0.4339	2.3	0.96	2323.2	44.4	2312.2	21.6	2302.4	11.1	2302.4	11.1
Gleason_354-U1450-A98F-1-Spot 211	313	140460	2.8	6.6918	0.8	8.9165	2.7	0.4327	2.6	0.95	2318.1	50.3	2329.5	24.7	2339.5	13.9	2339.5	13.9
Gleason_354-U1450-A98F-1-Spot 153	688	460263	7.7	6.4628	0.9	8.2115	2.7	0.3849	2.5	0.94	2099.1	45.1	2254.6	24.2	2398.9	15.5	2398.9	15.5
Gleason_354-U1450-A98F-1-Spot 292	174	97262	0.8	6.3586	0.7	8.7503	2.5	0.4035	2.3	0.96	2185.3	43.5	2312.3	22.4	2426.5	12.1	2426.5	12.1
Gleason_354-U1450-A98F-1-Spot 92	572	170434	2.1	6.3160	0.7	8.8260	2.6	0.4043	2.5	0.97	2188.8	46.2	2320.2	23.5	2437.9	11.3	2437.9	11.3
Gleason_354-U1450-A98F-1-Spot 132	91	31601	1.6	6.2684	0.9	10.4096	2.7	0.4733	2.6	0.95	2497.8	53.4	2471.9	25.3	2450.7	15.0	2450.7	15.0
Gleason_354-U1450-A98F-1-Spot 207	137	135630	0.7	6.1690	1.2	10.1765	3.7	0.4553	3.5	0.95	2418.8	70.6	2450.9	34.2	2477.7	19.9	2477.7	19.9
Gleason_354-U1450-A98F-1-Spot 3	203	322359	1.2	6.1479	0.8	9.9402	2.5	0.4432	2.4	0.95	2365.1	47.8	2429.3	23.4	2483.4	13.2	2483.4	13.2
Gleason_354-U1450-A98F-1-Spot 116	140	103175	2.8	6.0898	0.8	10.7814	2.6	0.4762	2.5	0.96	2510.6	51.0	2504.5	23.8	2499.5	12.7	2499.5	12.7
Gleason_354-U1450-A98F-1-Spot 88	208	141520	2.2	6.0477	0.8	10.0159	2.5	0.4393	2.3	0.94	2347.6	45.8	2436.2	22.8	2511.1	13.7	2511.1	13.7
Gleason_354-U1450-A98F-1-Spot 295	380	96753	4.4	5.9707	0.6	9.9663	2.2	0.4316	2.1	0.96	2312.8	40.8	2431.7	20.1	2532.6	9.8	2532.6	9.8
Gleason_354-U1450-A98F-1-Spot 5	306	140289	1.8	5.8952	0.9	10.2257	2.5	0.4372	2.4	0.94	2338.1	46.2	2455.4	23.2	2554.0	14.5	2554.0	14.5
Gleason_354-U1450-A98F-1-Spot 106	672	38253	1.3	5.3445	0.9	11.3902	3.6	0.4415	3.4	0.97	2357.4	68.0	2555.6	33.2	2716.9	14.4	2716.9	14.4
Gleason_354-U1450-A98F-1-Spot 21	142	140891	1.7	5.0552	0.7	15.1853	2.3	0.5567	2.2	0.96	2853.2	51.2	2826.9	22.1	2808.3	10.7	2808.3	10.7
Gleason_354-U1450-A98F-1-Spot 125	22	8370	1.1	4.8657	2.6	13.0330	10.1	0.4599	9.8	0.97	2439.2	198.7	2682.0	95.6	2870.5	41.7	2870.5	41.7
Gleason_354-U1450-A98F-1-Spot 38	61	107247	0.8	4.4129	0.9	16.2292	3.8	0.5194	3.7	0.97	2696.7	80.9	2890.4	36.1	3028.2	14.0	3028.2	14.0
Gleason_354-U1450-A98F-1-Spot 9	159	144828	1.3	4.2942	0.8	19.2478	3.0	0.5995	2.9	0.97	3027.6	70.1	3054.3	29.0	3071.9	12.3	3071.9	12.3
Gleason_354-U1450-A98F-1-Spot 31	320	305907	3.2	3.8004	0.5	23.4548	1.6	0.6465	1.5	0.95	3214.4	39.1	3246.0	15.8	3265.6	7.8	3265.6	7.8

Table ______. U-Pb geochronologic analyses.																		
#13 U1450A_124F						Isotope ratios						Apparent ages (Ma)						
Early Pliocene 3.6 Ma																		
Analysis	U	206Pb	U/Th	206Pb*	±	207Pb*	±	206Pb*	±	error	206Pb*	±	207Pb*	±	206Pb*	±	Best age	±
	(ppm)	204Pb		207Pb*	(%)	235U*	(%)	238U	(%)	corr.	238U*	(Ma)	235U	(Ma)	207Pb*	(Ma)	(Ma)	(Ma)
354-U1450A-124F-1W-39-138-Spot 252	1626	182344	292.2	20.6426	0.8	0.0305	1.6	0.0046	1.4	0.86	29.4	0.4	30.6	0.5	121.0	19.2	29.4	0.4
354-U1450A-124F-1W-39-138-Spot 279	4120	265120	78.7	19.6004	0.6	0.0438	2.5	0.0062	2.4	0.97	40.0	1.0	43.5	1.0	241.7	14.1	40.0	1.0
354-U1450A-124F-1W-39-138-Spot 30	544	15272	109.2	18.3222	1.7	0.0715	7.6	0.0095	7.4	0.97	61.0	4.5	70.1	5.1	395.0	38.6	61.0	4.5
354-U1450A-124F-1W-39-138-Spot 142	276	4975	1.8	21.2941	1.6	0.0866	2.2	0.0134	1.4	0.66	85.6	1.2	84.3	1.8	47.3	38.8	85.6	1.2
354-U1450A-124F-1W-39-138-Spot 281	5450	369806	403.1	17.7652	0.5	0.2556	1.6	0.0329	1.5	0.94	208.9	3.1	231.1	3.3	463.8	11.7	208.9	3.1
354-U1450A-124F-1W-39-138-Spot 158	778	173586	13.9	15.2021	0.5	0.3505	2.4	0.0386	2.3	0.98	244.4	5.5	305.1	6.2	799.4	10.6	244.4	5.5
354-U1450A-124F-1W-39-138-Spot 215	5850	3328901	42.1	17.9729	0.5	0.3599	6.8	0.0469	6.8	1.00	295.6	19.5	312.1	18.2	438.0	11.1	295.6	19.5
354-U1450A-124F-1W-39-138-Spot 113	1893	63128	23.0	16.7117	0.6	0.4998	1.5	0.0606	1.4	0.93	379.1	5.1	411.6	5.1	597.7	12.0	379.1	5.1
354-U1450A-124F-1W-39-138-Spot 137	319	22015	6.4	17.5148	0.7	0.5356	3.7	0.0680	3.6	0.98	424.4	14.9	435.5	13.1	495.2	15.8	424.4	14.9
354-U1450A-124F-1W-39-138-Spot 19	1186	48824	0.8	17.6877	0.6	0.5435	1.9	0.0697	1.8	0.95	434.5	7.5	440.7	6.7	473.5	13.3	434.5	7.5
354-U1450A-124F-1W-39-138-Spot 72	3257	33289087	18.6	17.6547	0.5	0.5469	1.4	0.0700	1.3	0.92	436.3	5.5	443.0	5.0	477.6	12.0	436.3	5.5
354-U1450A-124F-1W-39-138-Spot 120	413	36506	12.1	17.5932	0.7	0.5505	2.9	0.0702	2.8	0.97	437.6	11.7	445.3	10.3	485.3	15.5	437.6	11.7
354-U1450A-124F-1W-39-138-Spot 49	477	63936	10.0	17.6286	0.5	0.5577	4.9	0.0713	4.8	0.99	444.0	20.7	450.0	17.7	480.9	11.9	444.0	20.7
354-U1450A-124F-1W-39-138-Spot 271	1027	138834	2.6	17.8276	0.6	0.5584	2.1	0.0722	2.0	0.96	449.4	8.7	450.5	7.6	456.1	13.4	449.4	8.7
354-U1450A-124F-1W-39-138-Spot 143	544	152273	1.1	17.7974	0.8	0.5653	1.7	0.0730	1.5	0.89	454.0	6.5	455.0	6.1	459.8	16.6	454.0	6.5
354-U1450A-124F-1W-39-138-Spot 301	797	2495574	4.9	17.5138	0.5	0.5754	1.3	0.0731	1.2	0.93	454.8	5.4	461.5	4.9	495.3	10.8	454.8	5.4
354-U1450A-124F-1W-39-138-Spot 177	894	51212	6.0	17.7473	0.8	0.5750	2.1	0.0740	2.0	0.93	460.3	8.8	461.3	7.9	466.0	17.5	460.3	8.8
354-U1450A-124F-1W-39-138-Spot 37	2021	269688	44.3	17.6224	0.7	0.5825	1.8	0.0745	1.7	0.91	462.9	7.4	466.1	6.8	481.6	16.6	462.9	7.4
354-U1450A-124F-1W-39-138-Spot 79	91	5763	1.6	17.9221	1.4	0.5769	2.6	0.0750	2.2	0.84	466.1	9.7	462.4	9.6	444.3	31.4	466.1	9.7
354-U1450A-124F-1W-39-138-Spot 106	1377	334886	37.8	17.7066	0.5	0.5841	1.3	0.0750	1.2	0.92	466.3	5.3	467.1	4.8	471.2	10.9	466.3	5.3
354-U1450A-124F-1W-39-138-Spot 116	289	17448	3.6	17.5701	0.9	0.5926	2.1	0.0755	1.9	0.90	469.3	8.6	472.5	8.0	488.3	20.8	469.3	8.6
354-U1450A-124F-1W-39-138-Spot 74	561	311842	11.0	17.7914	0.6	0.5862	2.0	0.0756	1.9	0.95	470.0	8.6	468.4	7.5	460.5	14.1	470.0	8.6
354-U1450A-124F-1W-39-138-Spot 276	1439	668722	29.3	17.0053	0.5	0.6136	1.2	0.0757	1.1	0.93	470.3	5.2	485.9	4.7	559.9	9.9	470.3	5.2
354-U1450A-124F-1W-39-138-Spot 266	927	102255	6.9	17.8474	0.5	0.5856	1.4	0.0758	1.3	0.93	471.0	6.0	468.1	5.3	453.6	11.7	471.0	6.0
354-U1450A-124F-1W-39-138-Spot 295	1251	188728	46.6	17.4963	0.7	0.5994	1.8	0.0761	1.7	0.93	472.6	7.6	476.9	6.8	497.5	14.8	472.6	7.6
354-U1450A-124F-1W-39-138-Spot 267	489	273941	22.9	17.6081	0.7	0.5973	1.7	0.0763	1.6	0.91	473.9	7.2	475.5	6.6	483.5	15.8	473.9	7.2
354-U1450A-124F-1W-39-138-Spot 77	186	113155	2.2	17.3868	0.6	0.6052	2.3	0.0763	2.2	0.97	474.1	10.2	480.5	8.8	511.4	13.0	474.1	10.2
354-U1450A-124F-1W-39-138-Spot 209	2558	1427343	4.6	17.5295	0.6	0.6003	1.7	0.0763	1.6	0.94	474.1	7.3	477.4	6.4	493.4	12.8	474.1	7.3
354-U1450A-124F-1W-39-138-Spot 222	214	68320	5.0	17.5798	0.8	0.6022	2.0	0.0768	1.9	0.92	476.9	8.5	478.6	7.7	487.0	17.1	476.9	8.5
354-U1450A-124F-1W-39-138-Spot 91	389	18547	3.8	17.8282	0.7	0.5953	1.5	0.0770	1.3	0.90	478.0	6.0	474.3	5.5	456.0	14.5	478.0	6.0
354-U1450A-124F-1W-39-138-Spot 303	671	191034	17.7	17.5871	0.6	0.6049	1.4	0.0772	1.3	0.91	479.1	6.0	480.3	5.4	486.1	12.9	479.1	6.0
354-U1450A-124F-1W-39-138-Spot 190	394	39925	1.6	17.6228	0.8	0.6047	2.4	0.0773	2.3	0.95	480.0	10.5	480.2	9.2	481.6	17.2	480.0	10.5
354-U1450A-124F-1W-39-138-Spot 131	314	341809	3.6	17.4510	0.8	0.6123	1.9	0.0775	1.8	0.92	481.1	8.2	485.0	7.4	503.2	16.7	481.1	8.2
354-U1450A-124F-1W-39-138-Spot 101	659	330539	1.5	17.4038	0.5	0.6147	1.5	0.0776	1.4	0.94	481.7	6.4	486.5	5.6	509.2	11.0	481.7	6.4
354-U1450A-124F-1W-39-138-Spot 203	762	106944	11.2	17.7442	0.8	0.6036	1.6	0.0777	1.4	0.88	482.2	6.6	479.5	6.2	466.5	17.0	482.2	6.6
354-U1450A-124F-1W-39-138-Spot 6	3002	974313	3.6	17.3673	0.6	0.6168	1.6	0.0777	1.5	0.92	482.3	6.9	487.8	6.2	513.8	14.0	482.3	6.9
354-U1450A-124F-1W-39-138-Spot 25	1843	293304	1.0	17.4052	0.5	0.6191	1.3	0.0782	1.2	0.91	485.1	5.5	489.3	5.0	509.0	11.6	485.1	5.5
354-U1450A-124F-1W-39-138-Spot 100	284	103754	2.1	17.3483	0.8	0.6236	2.3	0.0785	2.2	0.94	486.9	10.2	492.1	9.1	516.2	17.5	486.9	10.2
354-U1450A-124F-1W-39-138-Spot 57	495	469206	12.6	17.5454	0.7	0.6194	1.8	0.0788	1.7	0.93	489.0	8.1	489.4	7.2	491.3	15.3	489.0	8.1
354-U1450A-124F-1W-39-138-Spot 93	511	298421	33.6	17.7273	0.6	0.6131	2.0	0.0788	1.8	0.94	489.1	8.7	485.5	7.5	468.6	14.4	489.1	8.7
354-U1450A-124F-1W-39-138-Spot 64	1253	144261	4.1	17.6807	0.5	0.6161	1.7	0.0790	1.6	0.95	490.2	7.6	487.4	6.5	474.4	11.6	490.2	7.6
354-U1450A-124F-1W-39-138-Spot 180	259	21976	7.4	17.6065	0.6	0.6188	1.8	0.0790	1.7	0.94	490.2	7.9	489.1	6.9	483.7	13.5	490.2	7.9
354-U1450A-124F-1W-39-138-Spot 99	703	109474	11.8	17.4318	0.5	0.6256	1.2	0.0791	1.1	0.91	490.7	5.3	493.3	4.8	505.6	11.5	490.7	5.3
354-U1450A-124F-1W-39-138-Spot 2	158	10768	1.6	17.4167	1.1	0.6262	2.5	0.0791	2.2	0.90	490.8	10.5	493.7	9.7	507.5	23.4	490.8	10.5
354-U1450A-124F-1W-39-138-Spot 35	143	19320	1.6	17.5610	0.9	0.6255	2.2	0.0797	2.1	0.91	494.1	9.8	493.3	8.8	489.4	20.2	494.1	9.8
354-U1450A-124F-1W-39-138-Spot 310	274	169146	6.8	17.2091	0.6	0.6443	1.9	0.0804	1.8	0.95	498.6	8.8	505.0	7.7	533.8	13.6	498.6	8.8
354-U1450A-124F-1W-39-138-Spot 159	394	56167	1.7	17.4663	0.7	0.6388	1.9	0.0809	1.8	0.94	501.6	8.7	501.6	7.6	501.3	14.5	501.6	8.7
354-U1450A-124F-1W-39-138-Spot 192	181	122571	1.9	17.2622	0.8	0.6536	2.0	0.0818	1.8	0.92	507.0	8.9	510.7	8.0	527.1	17.2	507.0	8.9
354-U1450A-124F-1W-39-138-Spot 14	107	8837	51.4	17.4899	1.2	0.6587	2.4	0.0836	2.0	0.85	517.3	10.0	513.8	9.5	498.3	27.0	517.3	10.0
354-U1450A-124F-1W-39-138-Spot 66	113	307687	1.8	17.1537	0.8	0.6724	2.6	0.0837	2.5	0.95	517.9	12.4	522.2	10.7	540.9	17.3	517.9	12.4
354-U1450A-124F-1W-39-138-Spot 212	116	78662	0.8	16.9884	0.9	0.6810	2.0	0.0839	1.8	0.90	519.4	9.0	527.4	8.2	562.0	18.9	519.4	9.0
354-U1450A-124F-1W-39-138-Spot 246	185	33030	0.5	17.1905	0.7	0.7022	1.9	0.0875	1.8	0.93	541.0	9.3	540.1	8.0	536.3	15.3	541.0	9.3
354-U1450A-124F-1W-39-138-Spot 50	154	29651	0.9	16.9251	0.7	0.7141	1.9	0.0877	1.7	0.92	541.6	8.9	547.2	7.9	570.2	15.8	541.6	8.9
354-U1450A-124F-1W-39-138-Spot 27	179	22604	53.2	16.8354	0.9	0.7379	2.3	0.0901	2.2	0.93	556.1	11.6	561.2	10.1	581.8	19.0	556.1	11.6
354-U1450A-124F-1W-39-138-Spot 199	235	35504	5.1	16.5449	0.7	0.7575	2.1	0.0909	1.9	0.93	560.9	10.4	572.6	9.0	619.4	16.1	560.9	10.4
354-U1450A-124F-1W-39-138-Spot 130	889	213311	23.7	16.9437	0.5	0.7449	1.2	0.0915	1.1	0.91	564.6	6.0	565.3	5.3	567.8	11.3	564.6	6.0
354-U1450A-124F-1W-39-138-Spot 225	855	933370	2.5	16.3905	0.7	0.8087	2.6	0.0961	2.5	0.96	591.7	14.4	601.7	12.0	639.6	15.3	591.7	14.4
354-U1450A-124F-1W-39-138-Spot 132	520	195510	9.3	16.4972	0.6	0.8105	1.7	0.0970	1.6	0.94	596.7	9.3	602.8	7.9	625.6	12.8	596.7	9.3
354-U1450A-124F-1W-39-138-Spot 240	342	80463	1.5	16.3261	0.8	0.8374	1.6	0.0992	1.4	0.86	609.5	8.2	617.7	7.6	648.1	17.9	609.5	8.2
354-U1450A-124F-1W-39-138-Spot 59	390	247872	1.2	16.2801	0.6	0.8587	1.9	0.1014	1.8	0.95	622.5	10.7	629.4	9.0	654.1	13.3	622.5	10.7
354-U1450A-124F-1W-39-138-Spot 154	97	16392	1.8	15.7908	1.0	0.8953	2.8	0.1025	2.7	0.94	629.3	16.0	649.2	13.6	719.2	20.6	629.3	16.0
354-U1450A-124F-1W-39-138-Spot 259	85	30008	4.0	16.4088	0.8	0.8619	2.1	0.1026	2.0	0.92	629.5	11.8	631.1	10.1	637.2	18.1	629.5	11.8
354-U1450A-124F-1W-39-138-Spot 226	642	137124	2.4	16.5692	0.6	0.8556	1.8	0.1028	1.7	0.95	630.9	10.5	627.7	8.6	616.2	12.0	630.9	10.5
354-U1450A-124F-1W-39-138-Spot 45	280	54759	3.3	16.4012	0.9	0.8678	2.4	0.1032	2.2	0.93	633.3	13.4	634.4	11.3	638.2	18.6	633.3	13.4
354-U1450A-124F-1W-39-138-Spot 155	68	7744	3.2	16.5900	1.6	0.8685	3.0	0.1045	2.6	0.85	640.7	15.7	634.8	14.2	613.5	34.2	640.7	15.7
354-U1450A-124F-1W-39-138-Spot 146	298	76599	1.5	16.1476	0.5	0.9343	1.3	0.1094	1.2	0.93	669.4	7.6	669.9	6.3	671.7	9.8	669.4	7.6
354-U1450A-124F-1W-39-138-Spot 84	2992	23593650	26.0	15.7726	0.7	0.9621	1.7	0.1101	1.5	0.90	673.1	9.6	684.4	8.3	721.7	15.3	673.1	9.6
354-U1450A-124F-1W-39-138-Spot 39	288	85408	4.4	16.3178	0.6	0.9334	1.5	0.1105	1.4	0.93	675.4	8.7	669.4	7.2	649.2	11.9	675.4	8.7
354-U1450A-124F-1W-39-138-Spot 138	756	95764	0.8	15.1190	0.5	1.0512	1.6	0.1153	1.5	0.95	703.3	10.0	729.5	8.2	810.8	10.7	703.3	10.0
354-U1450A-124F-1W-39-138-Spot 309	152	32398	1.3	15.5061	0.6	1.0313	1.9	0.1160	1.8	0.94	707.4	11.7	719.6	9.6	757.8	13.4	707.4	11.7
354-U1450A-124F-1W-39-138-Spot 197	277	80803	3.4	15.6520	0.7	1.0379	2.2	0.1178	2.1	0.95	718.0	14.4	722.9	11.5	738.0	14.8	718.0	14.4
354-U1450A-124F-1W-39-138-Spot 121	100	8867	1.4	15.0104	0.7	1.0986	2.3	0.1196	2.2	0.95	728.2	15.3	752.7	12.4	825.9	14.6	728.2	15.3
354-U1450A-124F-1W-39-138-Spot 32	551	535675	3.2	15.7186	0.6	1.0587	1.8	0.1207	1.7	0.95	734.6	11.7	733.2	9.3	729.0	12.0	734.6	11.7
354-U1450A-124F-1W-39-138-Spot 283	1124	15886227	1.2	15.2620	0.6	1.1072	1.6	0.1226	1.5	0.92	745.3	10.4	756.8	8.5	791.1	13.0	745.3	10.4
354-U1450A-124F-1W-39-138-Spot 20	21	20754	1.3	15.4732	1.6	1.1001	4.4	0.1235	4.1	0.93	750.4	29.1	753.4	23.5	762.2	34.4	750.4	29.1
354-U1450A-124F-1W-39-138-Spot 109	110	97087	0.8	15.4957	0.7	1.1016	2.8	0.1238	2.7	0.97	752.4	19.0	754.1	14.8	759.2	15.0	752.4	19.0
354-U1450A-124F-1W-39-138-Spot 184	523	58954	2.7	14.7058	1.0	1.1729	4.8	0.1251	4.8	0.98	759.8	34.1	788.0	26.6	868.6	20.1	759.8	34.1
354-U1450A-124F-1W-39-138-Spot 162	255	44839	5.7	15.2405	0.7	1.1375	1.8	0.1257	1.6	0.91	763.5	11.5	771.3	9.5	794.1	15.7	763.5	11.5
354-U1450A-124F-1W-39-138-Spot 98	232	527213	1.7	15.2508	1.6	1.1405	4.6	0.1261	4.3	0.93	765.8	30.8	772.7	24.7	792.7	34.0	765.8	30.8
354-U1450A-124F-1W-39-138-Spot 264	1624	75302	3.4	14.6494	0.6	1.2044	1.3	0.1280	1.2	0.91	776.2	8.8	802.6	7.3	876.5	11.5	776.2	8.8
354-U1450A-124F-1W-39-138-Spot 29	163	9543	2.1	14.2224	2.3	1.2493	3.2	0.1289	2.3	0.70	781.4	16.6	823.1	18.1	937.5	46.9	781.4	16.6
354-U1450A-124F-1W-39-138-Spot 76	86	38039	1.0	15.1412	0.8	1.1754	2.4	0.1291	2.3	0.95	782.6	16.9	789.2	13.3	807.8	16.5	782.6	16.9
354-U1450A-124F-1W-39-138-Spot 112	318	63882	0.9	14.3754	0.6	1.2409	1.3	0.1294	1.2	0.88	784.3	8.5	819.3	7.4	915.5	12.9	784.3	8.5
354-U1450A-124F-1W-39-138-Spot 308	1097	164912	2.9	14.9785	0.7	1.1991	1.8	0.1303	1.7	0.92	789.4	12.4	800.2	10.0	830.4	14.6	789.4	12.4
354-U1450A-124F-1W-39-138-Spot 229	79	58656	1.4	15.1536	0.8	1.1881	2.6	0.1306	2.5	0.95	791.2	18.4	795.1	14.4	806.1	17.8	791.2	18.4
354-U1450A-124F-1W-39-138-Spot 227	50	7595	2.6	15.1793	1.0	1.1896	3.1	0.1310	2.9	0.94	793.3	21.6	795.8	17.0	802.5	21.9	793.3	21.6
354-U1450A-124F-1W-39-138-Spot 17	2811	411734	10.9	14.9632	0.5	1.2123	5.0	0.1316	5.0	0.99	796.8	37.4	806.2	27.9	832.5	10.5	796.8	37.4
354-U1450A-124F-1W-39-138-Spot 8	250	222000	1.7	15.0969	0.7	1.2086	1.9	0.1323	1.8	0.92	801.2	13.3	804.5	10.6	813.9	15.4	801.2	13.3

354-U1450A-124F-1W-39-138-Spot 128	528	151450	5.1	14.7375	0.6	1.2412	2.0	0.1327	1.9	0.96	803.1	14.6	819.4	11.3	864.1	11.5	803.1	14.6
354-U1450A-124F-1W-39-138-Spot 89	124	40461	1.5	15.2238	0.8	1.2019	2.0	0.1327	1.8	0.92	803.3	13.7	801.5	10.9	796.4	16.3	803.3	13.7
354-U1450A-124F-1W-39-138-Spot 168	373	68368	1.2	15.2046	0.5	1.2035	1.8	0.1327	1.8	0.97	803.4	13.4	802.2	10.2	799.0	9.9	803.4	13.4
354-U1450A-124F-1W-39-138-Spot 34	86	105286	1.0	14.9136	0.8	1.2468	2.1	0.1349	2.0	0.93	815.5	15.1	822.0	12.0	839.4	16.6	815.5	15.1
354-U1450A-124F-1W-39-138-Spot 115	159	64557	3.4	14.7044	0.6	1.2729	2.3	0.1358	2.3	0.97	820.6	17.3	833.7	13.2	868.7	11.7	820.6	17.3
354-U1450A-124F-1W-39-138-Spot 280	701	1507403	1.2	14.7521	0.6	1.2778	1.8	0.1367	1.7	0.94	826.1	13.1	835.9	10.2	862.0	12.5	826.1	13.1
354-U1450A-124F-1W-39-138-Spot 213	129	19750	3.8	14.5533	0.7	1.2958	2.5	0.1368	2.4	0.96	826.4	18.5	843.9	14.3	890.1	14.5	826.4	18.5
354-U1450A-124F-1W-39-138-Spot 147	192	523416	1.9	14.8193	0.8	1.2728	3.1	0.1368	3.0	0.96	826.5	23.0	833.6	17.5	852.6	16.8	826.5	23.0
354-U1450A-124F-1W-39-138-Spot 194	292	10706185	3.4	14.8471	0.7	1.2746	1.8	0.1372	1.6	0.92	829.1	12.7	834.4	10.2	848.7	14.8	829.1	12.7
354-U1450A-124F-1W-39-138-Spot 311	1349	1523636	4.7	14.6284	0.8	1.3060	1.8	0.1386	1.6	0.90	836.5	12.7	848.4	10.4	879.5	16.4	836.5	12.7
354-U1450A-124F-1W-39-138-Spot 196	402	107868	1.0	14.9741	0.6	1.2762	1.8	0.1386	1.7	0.94	836.7	13.2	835.2	10.2	831.0	12.7	836.7	13.2
354-U1450A-124F-1W-39-138-Spot 81	709	309547	3.2	14.3861	0.7	1.3333	2.3	0.1391	2.2	0.96	839.7	17.4	860.3	13.4	913.9	13.4	839.7	17.4
354-U1450A-124F-1W-39-138-Spot 164	1056	467731	2.7	14.6950	0.6	1.3055	1.9	0.1391	1.8	0.95	839.8	14.1	848.1	10.8	870.1	11.6	839.8	14.1
354-U1450A-124F-1W-39-138-Spot 314	250	1000199	3.1	14.6504	0.7	1.3141	1.7	0.1396	1.5	0.92	842.6	12.2	851.9	9.7	876.4	13.8	842.6	12.2
354-U1450A-124F-1W-39-138-Spot 187	110	41646	2.7	14.6333	0.8	1.3229	2.4	0.1404	2.2	0.94	846.9	17.8	855.8	13.8	878.8	16.3	846.9	17.8
354-U1450A-124F-1W-39-138-Spot 219	293	88907	1.0	14.6685	0.6	1.3199	1.9	0.1404	1.8	0.95	847.0	14.6	854.5	11.2	873.9	12.3	847.0	14.6
354-U1450A-124F-1W-39-138-Spot 268	832	244204	4.0	14.7974	0.5	1.3141	1.4	0.1410	1.3	0.93	850.5	10.7	851.9	8.4	855.7	11.3	850.5	10.7
354-U1450A-124F-1W-39-138-Spot 255	443	289540	13.0	14.7153	0.6	1.3249	1.8	0.1414	1.7	0.95	852.6	13.9	856.6	10.6	867.2	12.2	852.6	13.9
354-U1450A-124F-1W-39-138-Spot 204	206	2220502	2.0	14.6925	0.7	1.3284	2.2	0.1416	2.1	0.94	853.4	16.5	858.2	12.7	870.5	15.3	853.4	16.5
354-U1450A-124F-1W-39-138-Spot 170	208	91486	2.0	14.8938	0.6	1.3131	1.6	0.1418	1.5	0.93	855.1	12.0	851.5	9.2	842.2	12.0	855.1	12.0
354-U1450A-124F-1W-39-138-Spot 223	152	23179	2.8	14.8864	0.7	1.3290	2.1	0.1435	2.0	0.95	864.3	16.4	858.4	12.4	843.2	13.7	864.3	16.4
354-U1450A-124F-1W-39-138-Spot 94	245	62813	0.5	14.6012	0.6	1.3705	1.7	0.1451	1.5	0.92	873.6	12.5	876.4	9.7	883.4	13.1	873.6	12.5
354-U1450A-124F-1W-39-138-Spot 202	94	311374	1.7	14.3664	0.8	1.4008	2.7	0.1460	2.5	0.95	878.3	20.9	889.3	15.8	916.8	16.7	878.3	20.9
354-U1450A-124F-1W-39-138-Spot 220	366	130721	1.6	14.6582	0.6	1.3867	2.2	0.1474	2.1	0.97	886.5	17.8	883.3	13.1	875.3	11.5	886.5	17.8
354-U1450A-124F-1W-39-138-Spot 156	547	268456	12.0	14.5239	0.6	1.4116	1.9	0.1487	1.8	0.94	893.6	15.2	893.8	11.5	894.3	13.4	893.6	15.2
354-U1450A-124F-1W-39-138-Spot 107	84	104212	1.7	14.5470	0.7	1.4109	2.2	0.1489	2.2	0.96	894.6	18.0	893.5	13.4	891.1	13.6	894.6	18.0
354-U1450A-124F-1W-39-138-Spot 134	272	56983	4.5	14.5782	0.8	1.4093	2.1	0.1490	1.9	0.93	895.4	16.0	892.9	12.3	886.6	15.8	895.4	16.0
354-U1450A-124F-1W-39-138-Spot 124	587	101980	14.3	14.4296	0.6	1.4499	1.6	0.1517	1.4	0.92	910.7	12.2	909.8	9.4	907.7	12.5	907.7	12.5
354-U1450A-124F-1W-39-138-Spot 163	998	1051197	9.5	14.3312	0.6	1.4452	2.5	0.1502	2.4	0.97	902.2	20.2	907.9	14.8	921.8	12.0	921.8	12.0
354-U1450A-124F-1W-39-138-Spot 5	259	144409	1.0	14.2743	0.5	1.5714	1.5	0.1627	1.4	0.95	971.7	13.0	959.0	9.5	930.0	10.2	930.0	10.2
354-U1450A-124F-1W-39-138-Spot 307	329	439261	2.4	14.2659	0.7	1.5017	2.2	0.1554	2.1	0.95	931.0	18.3	931.1	13.5	931.2	14.2	931.2	14.2
354-U1450A-124F-1W-39-138-Spot 231	541	172149	5.7	14.2621	0.6	1.5301	1.1	0.1583	0.9	0.86	947.2	8.4	942.5	6.7	931.8	11.4	931.8	11.4
354-U1450A-124F-1W-39-138-Spot 9	472	103533	2.9	14.2308	0.8	1.4318	2.2	0.1478	2.0	0.93	888.5	16.9	902.3	13.1	936.2	15.9	936.2	15.9
354-U1450A-124F-1W-39-138-Spot 265	52	16564	3.2	14.1663	0.9	1.5452	2.4	0.1588	2.2	0.93	949.9	19.3	948.6	14.5	945.6	18.0	945.6	18.0
354-U1450A-124F-1W-39-138-Spot 152	680	84861	1.9	14.1571	0.6	1.4427	2.1	0.1481	2.1	0.96	890.5	17.1	906.8	12.8	946.9	12.0	946.9	12.0
354-U1450A-124F-1W-39-138-Spot 135	247	210970	2.7	14.1543	0.5	1.5646	1.7	0.1606	1.6	0.96	960.2	14.2	956.3	10.3	947.3	10.0	947.3	10.0
354-U1450A-124F-1W-39-138-Spot 111	367	62402	1.0	14.1094	0.6	1.5126	1.7	0.1548	1.6	0.94	927.8	13.8	935.5	10.4	953.8	11.5	953.8	11.5
354-U1450A-124F-1W-39-138-Spot 68	355	812411	2.6	14.1052	0.6	1.6032	1.8	0.1640	1.7	0.95	979.0	15.2	971.5	11.1	954.4	11.8	954.4	11.8
354-U1450A-124F-1W-39-138-Spot 54	207	31161	0.6	14.0714	0.7	1.5915	1.7	0.1624	1.5	0.90	970.2	13.9	966.9	10.7	959.3	15.0	959.3	15.0
354-U1450A-124F-1W-39-138-Spot 195	339	82062	2.3	14.0698	0.6	1.4694	1.8	0.1499	1.7	0.94	900.7	13.9	917.9	10.6	959.5	12.5	959.5	12.5
354-U1450A-124F-1W-39-138-Spot 193	317	39221	1.9	14.0409	0.4	1.5157	1.4	0.1543	1.4	0.95	925.3	11.8	936.7	8.8	963.8	9.0	963.8	9.0
354-U1450A-124F-1W-39-138-Spot 61	317	68012	5.3	14.0387	0.6	1.5530	1.7	0.1581	1.6	0.94	946.3	14.5	951.7	10.8	964.1	12.1	964.1	12.1
354-U1450A-124F-1W-39-138-Spot 235	1001	98829	26.8	14.0360	0.5	1.5733	1.3	0.1602	1.3	0.94	957.7	11.3	959.7	8.4	964.5	9.2	964.5	9.2
354-U1450A-124F-1W-39-138-Spot 48	172	58060	1.6	14.0325	0.6	1.5512	1.9	0.1579	1.8	0.94	944.9	15.8	951.0	11.7	965.0	12.9	965.0	12.9
354-U1450A-124F-1W-39-138-Spot 70	296	562794	1.6	13.9940	0.6	1.6294	1.6	0.1654	1.5	0.93	986.6	13.6	981.6	10.0	970.6	11.5	970.6	11.5
354-U1450A-124F-1W-39-138-Spot 85	226	181098	3.2	13.9929	0.5	1.5786	2.1	0.1602	2.0	0.97	957.9	18.0	961.8	12.9	970.7	9.8	970.7	9.8
354-U1450A-124F-1W-39-138-Spot 62	141	26367	1.9	13.9682	0.6	1.6255	2.2	0.1647	2.1	0.96	982.7	19.0	980.1	13.6	974.4	11.9	974.4	11.9
354-U1450A-124F-1W-39-138-Spot 92	280	25511	6.4	13.9578	0.7	1.6108	2.1	0.1631	2.0	0.95	973.8	17.9	974.4	13.1	975.8	13.3	975.8	13.3
354-U1450A-124F-1W-39-138-Spot 47	211	37051	1.2	13.9514	0.6	1.6030	1.9	0.1622	1.8	0.94	969.0	15.8	971.4	11.7	976.8	13.1	976.8	13.1
354-U1450A-124F-1W-39-138-Spot 133	613	154903	7.3	13.9272	0.6	1.5532	1.4	0.1569	1.3	0.92	939.4	11.3	951.8	8.8	980.3	11.6	980.3	11.6
354-U1450A-124F-1W-39-138-Spot 18	341	1083523	0.9	13.9208	0.6	1.5811	2.1	0.1596	2.0	0.95	954.7	17.7	962.8	13.0	981.3	13.0	981.3	13.0
354-U1450A-124F-1W-39-138-Spot 286	550	193652	3.4	13.9036	0.5	1.6257	2.0	0.1639	1.9	0.96	978.6	17.0	980.2	12.3	983.8	10.8	983.8	10.8
354-U1450A-124F-1W-39-138-Spot 287	591	852122	5.5	13.8992	0.5	1.6215	1.6	0.1635	1.5	0.94	976.0	13.3	978.6	9.8	984.4	11.0	984.4	11.0
354-U1450A-124F-1W-39-138-Spot 103	205	414959	2.1	13.8948	0.8	1.6073	2.0	0.1620	1.9	0.92	967.7	16.6	973.1	12.6	985.1	15.8	985.1	15.8
354-U1450A-124F-1W-39-138-Spot 118	564	102150	5.4	13.8906	0.5	1.6020	1.5	0.1614	1.4	0.94	964.5	12.8	971.0	9.5	985.7	10.7	985.7	10.7
354-U1450A-124F-1W-39-138-Spot 174	319	181783	0.5	13.8680	0.6	1.6963	1.6	0.1706	1.4	0.92	1015.5	13.6	1007.1	10.0	989.0	12.1	989.0	12.1
354-U1450A-124F-1W-39-138-Spot 169	431	155978	16.1	13.8621	0.7	1.6208	2.1	0.1630	1.9	0.93	973.2	17.5	978.3	13.0	989.8	15.1	989.8	15.1
354-U1450A-124F-1W-39-138-Spot 273	214	71173	4.8	13.8529	0.6	1.6135	1.8	0.1621	1.7	0.93	968.5	15.0	975.5	11.2	991.2	13.2	991.2	13.2
354-U1450A-124F-1W-39-138-Spot 270	114	44808	1.5	13.8405	0.8	1.6547	2.7	0.1661	2.5	0.96	990.6	23.3	991.4	16.8	993.0	15.7	993.0	15.7
354-U1450A-124F-1W-39-138-Spot 237	623	182001	3.5	13.8348	0.4	1.7121	1.7	0.1718	1.6	0.96	1022.0	15.1	1013.1	10.6	993.9	9.1	993.9	9.1
354-U1450A-124F-1W-39-138-Spot 312	538	384558	3.3	13.7947	0.5	1.4962	1.9	0.1497	1.8	0.96	899.2	15.1	928.8	11.4	999.8	10.9	999.8	10.9
354-U1450A-124F-1W-39-138-Spot 315	50	67093	3.6	13.7622	0.8	1.5827	2.5	0.1580	2.3	0.94	945.5	20.5	963.4	15.4	1004.5	17.2	1004.5	17.2
354-U1450A-124F-1W-39-138-Spot 247	118	46217	1.3	13.6771	0.6	1.7308	2.1	0.1717	2.0	0.95	1021.4	19.1	1020.1	13.7	1017.1	12.8	1017.1	12.8
354-U1450A-124F-1W-39-138-Spot 305	69	101114	2.4	13.6343	0.7	1.7215	3.2	0.1702	3.1	0.97	1013.4	29.0	1016.6	20.4	1023.5	14.5	1023.5	14.5
354-U1450A-124F-1W-39-138-Spot 189	189	1138765	1.9	13.5812	0.5	1.7477	2.0	0.1721	1.9	0.97	1023.9	17.9	1026.3	12.6	1031.4	10.4	1031.4	10.4
354-U1450A-124F-1W-39-138-Spot 102	82	37060	3.1	13.4261	0.9	1.7696	2.6	0.1723	2.4	0.94	1024.9	23.0	1034.4	16.8	1054.6	17.8	1054.6	17.8
354-U1450A-124F-1W-39-138-Spot 150	473	91666	4.6	13.4145	0.6	1.6937	1.4	0.1648	1.3	0.92	983.3	12.1	1006.2	9.2	1056.3	11.3	1056.3	11.3
354-U1450A-124F-1W-39-138-Spot 285	69	42731	2.2	13.4122	0.8	1.8056	2.3	0.1756	2.1	0.93	1043.1	20.4	1047.5	14.8	1056.7	16.5	1056.7	16.5
354-U1450A-124F-1W-39-138-Spot 4	1394	359281	5.4	13.3771	0.5	1.6402	1.6	0.1591	1.5	0.95	952.0	13.2	985.8	9.9	1061.9	10.2	1061.9	10.2
354-U1450A-124F-1W-39-138-Spot 245	285	16222	1.1	13.3765	1.5	1.6073	3.0	0.1559	2.5	0.85	934.2	21.9	973.1	18.5	1062.0	31.0	1062.0	31.0
354-U1450A-124F-1W-39-138-Spot 63	245	1358951	2.4	13.3642	0.6	1.7948	1.9	0.1740	1.8	0.95	1033.9	17.3	1043.6	12.4	1063.9	11.6	1063.9	11.6
354-U1450A-124F-1W-39-138-Spot 221	323	58248	1.6	13.2773	0.4	1.8693	1.6	0.1800	1.6	0.97	1067.0	15.4	1070.3	10.7	1077.0	8.3	1077.0	8.3
354-U1450A-124F-1W-39-138-Spot 272	460	155522	8.3	13.2751	0.6	1.6243	2.1	0.1564	2.0	0.96	936.7	17.5	979.7	13.1	1077.3	11.5	1077.3	11.5
354-U1450A-124F-1W-39-138-Spot 22	188	51541	0.9	13.2189	0.7	1.8768	1.9	0.1799	1.8	0.93	1066.6	17.7	1072.9	12.9	1085.8	14.4	1085.8	14.4
354-U1450A-124F-1W-39-138-Spot 200	605	135129	3.2	13.2098	0.5	1.8729	1.5	0.1794	1.4	0.93	1063.9	13.9	1071.6	10.0	1087.2	10.9	1087.2	10.9
354-U1450A-124F-1W-39-138-Spot 242	1450	3050204	1.3	13.1802	0.6	1.8303	3.8	0.1750	3.7	0.99	1039.4	35.6	1056.4	24.6	1091.7	11.0	1091.7	11.0
354-U1450A-124F-1W-39-138-Spot 119	106	17277	2.2	13.1602	1.6	1.5532	3.2	0.1482	2.8	0.87	891.1	23.0	951.8	19.7	1094.7	31.5	1094.7	31.5
354-U1450A-124F-1W-39-138-Spot 214	363	70106	7.0	13.1295	0.5	1.9796	1.9	0.1885	1.9	0.96	1113.3	18.9	1108.6	13.0	1099.4	10.3	1099.4	10.3
354-U1450A-124F-1W-39-138-Spot 198	441	91715	1.8	13.1225	0.7	1.6286	2.0	0.1550	1.9	0.94	928.9	16.2	981.3	12.5	1100.5	13.2	1100.5	13.2
354-U1450A-124F-1W-39-138-Spot 224	103	23249	4.1	13.0632	0.8	1.9706	2.6	0.1867	2.4	0.95	1103.5	24.6	1105.5	17.2	1109.5	16.4	1109.5	16.4
354-U1450A-124F-1W-39-138-Spot 136	386	20408	0.9	13.0002	0.7	1.8744	1.6	0.1767	1.4	0.91	1049.1	13.7	1072.1	10.3	1119.2	13.2	1119.2	13.2
354-U1450A-124F-1W-39-138-Spot 78	482	251015	7.2	12.9649	0.7	1.9901	1.6	0.1871	1.4	0.91	1105.8	14.3	1112.2	10.5	1124.6	13.2	1124.6	13.2
354-U1450A-124F-1W-39-138-Spot 217	120	63650	1.7	12.8044	0.5	2.0588	2.0	0.1912	2.0	0.97	1127.8	20.4	1135.2	13.9	1149.4	10.3	1149.4	10.3
354-U1450A-124F-1W-39-138-Spot 144	306	96227	2.7	12.7699	0.7	2.1992	1.7	0.2037	1.6	0.92	1195.1	17.4	1180.8	12.1	1154.7	13.6	1154.7	13.6
354-U1450A-124F-1W-39-138-Spot 51	505	707945	12.9	12.6890	0.7	2.1229	2.0	0.1954	1.8	0.93	1150.4	19.3	1156.3	13.6	1167.3	14.7	1167.3	14.7
354-U1450A-124F-1W-39-138-Spot 16	40	110292	1.4	12.6699	1.1	2.0705	4.0	0.1903	3.9	0.96	1122.8	40.1	1139.1	27.6	1170.3	21.3	1170.3	21.3
354-U1450A-124F-1W-39-138-Spot 275	134	303495	1.2	12.4810	0.6	2.2281	2.3	0.2017	2.2	0.96	1184.4	23.8	1189.9	16.0	1200.0	12.5	1200.0	12.5
354-U1450A-124F-1W-39-138-Spot 96	341	1138743	2.5	12.4666	0.6	2.3057	1.6	0.2085	1.5	0.93	1220.7	17.1	1214.0	11.7	1202.3	11.8	1202.3	11.8
354-U1450A-124F-1W-39-138-Spot 251	724	31561	2.5	12.3900	0.6	2.0588	1.4	0.1850	1.3	0.90	1094.3	13.0	1135.2	9.8	1214.4	12.4	1214.4	12.4
354-U1450A-124F-1W-39-138-Spot 173	986	262906	7.5	12.2800	0.7	2.2960	2.3	0.2045	2.2	0.95	1199.4	24.1	1211.1	16.3	1231.9	13.9	1231.9	13.9
354-U1450A-124F-1W-39-138-Spot 46	291	5838	2.7	12.0348	3.2	1.9610	3.8	0.1712	2.0	0.53	1018.5	18.8	1102.2	25.3	1271.4	62.2	1271.4	62.2
354-U1450A-124F-1W-39-138-Spot 75	154	51203	1.0	11.9365	0.6	2.6095	2.1	0.2259	2.0	0.96	1313.0	23.4	1303.3	15.1	1287.4	10.9	1287.4	10.9
354-U1450A-124F-1W-39-138-Spot 56	150	25337	1.4	11.8767	0.7	2.6414	1.6	0.2275	1.4	0.90	1321.5	17.0	1312.2	11.6	1297.1	13.1	1297.1	13.1
354-U1450A-124F-1W-39-138-Spot 141	92	35916	1.1	11.1847	0.6	2.9262	2.1	0.2374	2.0	0.96	1373.0	25.1	1388.7	16.0	1412.9	11.8	1412.9	11.8
354-U1450A-124F-1W-39-138-Spot 80	222	70360	1.8	11.1410	0.5	3.1594	1.8	0.2553	1.7	0.95	1465.7	22.3	1447.3	13.8	1420.4	10.1	1420.4	10.1

354-U1450A-124F-1W-39-138-Spot 122	205	34748	1.3	11.0559	0.7	3.0809	2.3	0.2470	2.2	0.95	1423.2	27.5	1427.9	17.3	1435.0	12.9	1435.0	12.9
354-U1450A-124F-1W-39-138-Spot 250	87	92189	1.0	11.0000	0.8	2.8698	2.5	0.2290	2.3	0.94	1329.0	27.7	1374.0	18.5	1444.7	15.6	1444.7	15.6
354-U1450A-124F-1W-39-138-Spot 182	185	285438	2.5	10.9532	0.6	3.0529	2.3	0.2425	2.3	0.97	1399.8	28.4	1421.0	17.8	1452.8	11.1	1452.8	11.1
354-U1450A-124F-1W-39-138-Spot 263	347	118584	1.8	10.5660	0.4	3.4269	1.3	0.2626	1.3	0.95	1503.2	16.9	1510.6	10.5	1521.0	8.2	1521.0	8.2
354-U1450A-124F-1W-39-138-Spot 42	211	673931	1.7	10.4762	0.6	3.4559	2.0	0.2626	1.8	0.95	1503.0	24.8	1517.2	15.4	1537.0	11.9	1537.0	11.9
354-U1450A-124F-1W-39-138-Spot 298	135	178132	1.8	10.4106	0.6	3.4997	2.3	0.2642	2.2	0.97	1511.5	29.7	1527.1	18.0	1548.9	10.8	1548.9	10.8
354-U1450A-124F-1W-39-138-Spot 262	215	61005	2.2	10.3972	0.8	3.5283	2.0	0.2661	1.8	0.92	1520.7	24.6	1533.6	15.5	1551.3	14.1	1551.3	14.1
354-U1450A-124F-1W-39-138-Spot 257	403	89850	1.6	10.3020	0.6	3.6280	1.5	0.2711	1.3	0.91	1546.2	18.4	1555.7	11.7	1568.5	11.3	1568.5	11.3
354-U1450A-124F-1W-39-138-Spot 297	118	88322	1.2	10.2276	0.6	3.8097	2.2	0.2826	2.1	0.96	1604.4	29.7	1594.8	17.5	1582.1	11.1	1582.1	11.1
354-U1450A-124F-1W-39-138-Spot 216	947	376418	7.4	10.1545	0.5	3.5967	1.2	0.2649	1.1	0.89	1514.8	14.5	1548.8	9.6	1595.5	10.2	1595.5	10.2
354-U1450A-124F-1W-39-138-Spot 188	157	299236	1.8	10.1398	0.6	3.8175	2.1	0.2807	2.0	0.95	1595.1	28.3	1596.4	16.9	1598.2	12.0	1598.2	12.0
354-U1450A-124F-1W-39-138-Spot 274	263	643605	3.3	10.0979	0.6	3.8771	1.7	0.2839	1.6	0.93	1611.2	23.0	1608.9	14.0	1605.9	11.7	1605.9	11.7
354-U1450A-124F-1W-39-138-Spot 127	257	52726	2.1	10.0961	0.7	3.3789	2.0	0.2474	1.9	0.94	1425.1	24.2	1499.5	15.7	1606.3	12.3	1606.3	12.3
354-U1450A-124F-1W-39-138-Spot 67	148	50125	1.7	10.0257	0.5	3.9739	2.1	0.2890	2.0	0.97	1636.3	29.3	1628.9	17.0	1619.3	10.2	1619.3	10.2
354-U1450A-124F-1W-39-138-Spot 151	172	710246	1.3	9.7738	0.7	4.2297	2.1	0.2998	1.9	0.95	1690.4	29.0	1679.8	16.9	1666.5	12.3	1666.5	12.3
354-U1450A-124F-1W-39-138-Spot 15	201	151410	1.5	9.7009	0.6	4.4737	1.8	0.3148	1.7	0.94	1764.1	26.4	1726.1	15.0	1680.4	11.1	1680.4	11.1
354-U1450A-124F-1W-39-138-Spot 148	216	58236	1.0	9.6566	0.5	3.6749	3.2	0.2574	3.2	0.99	1476.4	41.6	1565.9	25.5	1688.8	9.5	1688.8	9.5
354-U1450A-124F-1W-39-138-Spot 117	104	30318	1.6	9.6394	0.7	3.7601	2.4	0.2629	2.3	0.95	1504.6	31.3	1584.3	19.6	1692.1	13.7	1692.1	13.7
354-U1450A-124F-1W-39-138-Spot 7	70	542345	0.6	9.5338	0.5	4.2849	2.3	0.2963	2.2	0.97	1672.9	32.6	1690.5	18.8	1712.4	9.8	1712.4	9.8
354-U1450A-124F-1W-39-138-Spot 123	193	126288	1.9	9.4947	0.5	4.3854	1.7	0.3020	1.6	0.96	1701.1	24.6	1709.6	14.2	1719.9	8.7	1719.9	8.7
354-U1450A-124F-1W-39-138-Spot 125	177	40553	2.1	9.4266	0.6	4.5401	1.8	0.3104	1.7	0.94	1742.7	26.5	1738.4	15.4	1733.1	11.7	1733.1	11.7
354-U1450A-124F-1W-39-138-Spot 171	212	107840	3.1	9.4129	0.6	4.6333	1.3	0.3163	1.1	0.88	1771.7	17.8	1755.3	11.0	1735.8	11.7	1735.8	11.7
354-U1450A-124F-1W-39-138-Spot 186	69	15952	1.9	9.4086	0.7	4.7483	2.3	0.3240	2.2	0.95	1809.3	34.8	1775.8	19.4	1736.6	13.0	1736.6	13.0
354-U1450A-124F-1W-39-138-Spot 11	292	353443	1.3	9.3539	0.5	4.0794	2.5	0.2768	2.5	0.98	1575.0	34.7	1650.2	20.7	1747.3	9.9	1747.3	9.9
354-U1450A-124F-1W-39-138-Spot 86	62	22188	1.5	9.2908	0.7	4.4530	2.5	0.3001	2.4	0.96	1691.6	35.1	1722.3	20.4	1759.7	13.0	1759.7	13.0
354-U1450A-124F-1W-39-138-Spot 290	158	95278	5.6	9.1966	0.6	4.6422	1.6	0.3096	1.4	0.93	1738.9	21.9	1756.9	13.0	1778.3	10.7	1778.3	10.7
354-U1450A-124F-1W-39-138-Spot 302	1615	1240927	4.2	9.1863	0.5	4.0844	1.5	0.2721	1.5	0.94	1551.6	20.0	1651.2	12.5	1780.4	9.2	1780.4	9.2
354-U1450A-124F-1W-39-138-Spot 239	586	341859	15.8	9.1758	0.6	3.8713	2.9	0.2576	2.9	0.98	1477.7	38.0	1607.7	23.8	1782.5	11.3	1782.5	11.3
354-U1450A-124F-1W-39-138-Spot 183	409	2494849	7.2	8.9911	0.5	5.0338	1.5	0.3283	1.4	0.93	1829.9	22.8	1825.0	13.0	1819.4	9.9	1819.4	9.9
354-U1450A-124F-1W-39-138-Spot 277	158	50648	3.6	8.9442	0.6	5.1706	2.1	0.3354	2.0	0.96	1864.6	32.9	1847.8	18.0	1828.9	10.8	1828.9	10.8
354-U1450A-124F-1W-39-138-Spot 82	487	117080	8.2	8.9363	0.6	4.8796	1.6	0.3163	1.5	0.94	1771.4	23.6	1798.7	13.7	1830.5	10.1	1830.5	10.1
354-U1450A-124F-1W-39-138-Spot 261	298	209811	2.2	8.9018	0.4	5.1658	1.5	0.3335	1.4	0.96	1855.4	23.0	1847.0	12.7	1837.5	7.8	1837.5	7.8
354-U1450A-124F-1W-39-138-Spot 60	348	66326	6.9	8.8983	0.6	4.8524	2.0	0.3132	1.9	0.96	1756.2	29.2	1794.0	16.7	1838.3	10.4	1838.3	10.4
354-U1450A-124F-1W-39-138-Spot 300	312	92537	9.3	8.8921	0.4	5.2118	1.5	0.3361	1.5	0.96	1868.0	23.7	1854.5	13.0	1839.5	7.8	1839.5	7.8
354-U1450A-124F-1W-39-138-Spot 3	273	135876	6.9	8.8554	0.6	5.1990	1.8	0.3339	1.7	0.95	1857.3	27.7	1852.5	15.4	1847.0	10.1	1847.0	10.1
354-U1450A-124F-1W-39-138-Spot 178	729	258089	3.1	8.8266	0.5	4.4190	1.7	0.2829	1.7	0.96	1605.9	23.6	1715.9	14.3	1852.9	8.5	1852.9	8.5
354-U1450A-124F-1W-39-138-Spot 201	578	126270	3.1	8.8052	0.7	4.9593	1.6	0.3167	1.5	0.91	1773.6	23.2	1812.4	13.9	1857.3	12.4	1857.3	12.4
354-U1450A-124F-1W-39-138-Spot 140	283	218299	5.7	8.7921	0.6	5.2322	1.8	0.3336	1.7	0.94	1856.0	27.6	1857.9	15.5	1860.0	10.9	1860.0	10.9
354-U1450A-124F-1W-39-138-Spot 114	389	250244	1.6	8.7908	0.6	5.1495	1.9	0.3283	1.8	0.95	1830.2	28.4	1844.3	16.0	1860.2	10.5	1860.2	10.5
354-U1450A-124F-1W-39-138-Spot 40	154	39364	2.9	8.7814	0.5	5.3151	2.1	0.3385	2.0	0.97	1879.5	33.1	1871.3	17.9	1862.2	9.0	1862.2	9.0
354-U1450A-124F-1W-39-138-Spot 232	349	846443	1.8	8.7786	0.5	5.3506	1.7	0.3407	1.6	0.96	1889.9	27.0	1877.0	14.7	1862.7	8.5	1862.7	8.5
354-U1450A-124F-1W-39-138-Spot 288	317	47566	1.7	8.7711	0.7	4.1313	1.9	0.2628	1.8	0.92	1504.2	23.6	1660.5	15.6	1864.3	13.1	1864.3	13.1
354-U1450A-124F-1W-39-138-Spot 166	240	652493	4.8	8.7415	0.5	5.2171	1.7	0.3308	1.6	0.94	1842.1	25.0	1855.4	14.1	1870.4	9.8	1870.4	9.8
354-U1450A-124F-1W-39-138-Spot 105	160	91203	3.2	8.6624	0.6	5.2938	2.1	0.3326	2.0	0.96	1850.9	32.6	1867.9	18.1	1886.8	10.8	1886.8	10.8
354-U1450A-124F-1W-39-138-Spot 160	231	156589	2.1	8.6215	0.6	4.4757	2.9	0.2799	2.9	0.98	1590.7	40.3	1726.5	24.3	1895.3	11.3	1895.3	11.3
354-U1450A-124F-1W-39-138-Spot 292	155	65371	1.5	8.6035	0.5	5.5794	2.1	0.3481	2.1	0.97	1925.7	34.8	1912.9	18.5	1899.0	8.7	1899.0	8.7
354-U1450A-124F-1W-39-138-Spot 233	296	44946	24.6	8.5788	0.5	5.0593	1.8	0.3148	1.8	0.96	1764.2	27.2	1829.3	15.6	1904.2	9.3	1904.2	9.3
354-U1450A-124F-1W-39-138-Spot 306	183	61657	1.4	8.5472	1.1	5.0688	3.8	0.3142	3.6	0.96	1761.4	55.9	1830.9	32.1	1910.8	19.0	1910.8	19.0
354-U1450A-124F-1W-39-138-Spot 185	268	79258	1.7	8.4256	0.5	5.3710	1.8	0.3282	1.7	0.95	1829.7	27.1	1880.2	15.3	1936.5	9.7	1936.5	9.7
354-U1450A-124F-1W-39-138-Spot 69	109	1869039	2.3	8.4195	0.6	5.8641	2.2	0.3581	2.1	0.96	1973.1	35.1	1955.9	18.7	1937.8	10.7	1937.8	10.7
354-U1450A-124F-1W-39-138-Spot 90	310	87415	4.0	8.3882	0.7	4.5871	5.1	0.2791	5.0	0.99	1586.7	70.7	1746.9	42.3	1944.5	12.0	1944.5	12.0
354-U1450A-124F-1W-39-138-Spot 256	247	31640474	2.6	8.2245	0.5	6.0410	2.2	0.3603	2.2	0.97	1983.8	36.7	1981.8	19.2	1979.6	8.8	1979.6	8.8
354-U1450A-124F-1W-39-138-Spot 236	102	39214	1.5	8.1162	0.6	5.3764	2.3	0.3165	2.3	0.97	1772.5	35.1	1881.1	20.0	2003.2	10.4	2003.2	10.4
354-U1450A-124F-1W-39-138-Spot 176	325	91095	2.0	8.0598	0.6	4.8977	2.2	0.2863	2.1	0.96	1623.0	30.8	1801.9	18.8	2015.6	10.5	2015.6	10.5
354-U1450A-124F-1W-39-138-Spot 139	271	175485	6.5	8.0230	0.6	6.1153	1.4	0.3558	1.2	0.91	1962.4	21.1	1992.4	11.9	2023.7	9.8	2023.7	9.8
354-U1450A-124F-1W-39-138-Spot 293	523	178549	8.7	8.0149	0.5	5.0088	3.2	0.2912	3.2	0.99	1647.3	46.1	1820.8	27.2	2025.5	9.3	2025.5	9.3
354-U1450A-124F-1W-39-138-Spot 13	61	137423	1.1	7.7200	0.5	7.0388	3.6	0.3941	3.6	0.99	2141.8	65.1	2116.3	32.1	2091.6	9.5	2091.6	9.5
354-U1450A-124F-1W-39-138-Spot 10	532	711861	2.9	7.5831	1.1	6.2372	1.8	0.3430	1.4	0.80	1901.2	23.9	2009.7	15.8	2123.0	18.8	2123.0	18.8
354-U1450A-124F-1W-39-138-Spot 108	106	71912	2.3	7.3980	0.6	7.4736	2.7	0.4010	2.7	0.97	2173.6	49.3	2169.8	24.6	2166.2	10.9	2166.2	10.9
354-U1450A-124F-1W-39-138-Spot 191	42	15017	6.1	7.2728	1.7	6.5442	4.3	0.3452	3.9	0.91	1911.6	64.7	2051.9	37.7	2195.9	30.4	2195.9	30.4
354-U1450A-124F-1W-39-138-Spot 244	236	319543	2.5	6.7270	0.4	8.8838	1.9	0.4334	1.8	0.98	2321.2	35.7	2326.1	17.1	2330.5	6.8	2330.5	6.8
354-U1450A-124F-1W-39-138-Spot 145	320	809656	1.8	6.6059	0.6	8.8756	2.3	0.4252	2.2	0.96	2284.2	42.5	2325.3	20.9	2361.6	10.3	2361.6	10.3
354-U1450A-124F-1W-39-138-Spot 44	181	327553	2.1	6.4401	0.6	9.4075	1.7	0.4394	1.6	0.94	2348.0	31.3	2378.6	15.5	2404.9	9.5	2404.9	9.5
354-U1450A-124F-1W-39-138-Spot 228	32	28092	0.8	6.3443	1.1	9.2672	6.0	0.4264	5.9	0.98	2289.5	113.6	2364.8	55.0	2430.3	18.3	2430.3	18.3
354-U1450A-124F-1W-39-138-Spot 1	330	3465097	1.5	6.2644	0.6	9.9409	1.4	0.4517	1.3	0.91	2402.6	26.0	2429.3	13.1	2451.8	10.0	2451.8	10.0
354-U1450A-124F-1W-39-138-Spot 104	112	80856	0.8	6.2538	0.5	9.5906	1.8	0.4350	1.7	0.95	2328.2	33.8	2396.3	16.6	2454.6	9.1	2454.6	9.1
354-U1450A-124F-1W-39-138-Spot 23	64	90251	1.2	6.2538	0.7	10.2096	2.8	0.4631	2.7	0.97	2453.1	55.2	2453.9	25.8	2454.6	11.2	2454.6	11.2
354-U1450A-124F-1W-39-138-Spot 205	206	70198	3.7	6.2412	0.5	9.5287	1.9	0.4313	1.9	0.97	2311.7	36.2	2390.3	17.6	2458.0	7.8	2458.0	7.8
354-U1450A-124F-1W-39-138-Spot 313	202	292726	0.6	6.2411	0.7	10.0916	1.7	0.4568	1.6	0.91	2425.4	32.1	2443.2	16.1	2458.1	12.2	2458.1	12.2
354-U1450A-124F-1W-39-138-Spot 294	185	64394	1.6	6.2357	0.6	9.8872	2.4	0.4472	2.3	0.97	2382.6	46.0	2424.3	21.9	2459.5	9.3	2459.5	9.3
354-U1450A-124F-1W-39-138-Spot 253	121	40236	1.6	6.2299	0.6	10.4462	1.9	0.4720	1.8	0.96	2492.3	37.3	2475.2	17.5	2461.1	9.3	2461.1	9.3
354-U1450A-124F-1W-39-138-Spot 218	125	48859	2.4	6.2064	0.5	9.7291	2.4	0.4379	2.4	0.98	2341.4	46.8	2409.5	22.5	2467.5	9.1	2467.5	9.1
354-U1450A-124F-1W-39-138-Spot 41	64	19002	1.8	6.1844	0.5	10.3876	2.0	0.4659	2.0	0.96	2465.7	40.3	2469.9	18.9	2473.5	9.2	2473.5	9.2
354-U1450A-124F-1W-39-138-Spot 28	166	258737	1.0	6.1542	0.5	11.0747	2.1	0.4943	2.0	0.97	2589.3	42.6	2529.4	19.2	2481.7	9.0	2481.7	9.0
354-U1450A-124F-1W-39-138-Spot 71	254	570446	4.3	6.0543	0.7	10.8809	1.5	0.4778	1.3	0.88	2517.6	27.0	2513.0	13.7	2509.3	11.9	2509.3	11.9
354-U1450A-124F-1W-39-138-Spot 12	98	241549	3.2	6.0450	0.6	10.6127	2.2	0.4653	2.2	0.96	2462.9	44.1	2489.8	20.8	2511.9	10.7	2511.9	10.7
354-U1450A-124F-1W-39-138-Spot 230	81	42031	2.3	6.0138	0.4	10.8832	2.1	0.4747	2.1	0.98	2504.1	43.4	2513.2	19.9	2520.6	7.5	2520.6	7.5
354-U1450A-124F-1W-39-138-Spot 278	31	354696	1.8	5.9342	0.8	11.0948	3.6	0.4775	3.5	0.97	2516.4	73.0	2531.1	33.5	2542.9	13.4	2542.9	13.4
354-U1450A-124F-1W-39-138-Spot 179	878	716365	6.9	5.9000	0.6	11.0747	1.6	0.4739	1.5	0.93	2500.6	31.9	2529.4	15.4	2552.6	10.0	2552.6	10.0
354-U1450A-124F-1W-39-138-Spot 211	94	242199	1.8	5.7635	0.5	11.8594	2.2	0.4957	2.1	0.97	2595.4	44.7	2593.4	20.2	2591.7	9.0	2591.7	9.0
354-U1450A-124F-1W-39-138-Spot 282	373	24087024	2.8	5.6913	0.5	11.6412	1.6	0.4805	1.5	0.96	2529.5	31.6	2576.0	14.8	2612.7	7.8	2612.7	7.8
354-U1450A-124F-1W-39-138-Spot 88	343	290060	2.4	5.6837	0.4	11.7590	1.5	0.4847	1.5	0.96	2547.8	30.6	2585.4	14.2	2615.0	7.5	2615.0	7.5
354-U1450A-124F-1W-39-138-Spot 234	345	487963	3.4	5.4945	0.5	12.9634	1.7	0.5166	1.6	0.96	2684.7	35.8	2677.0	15.9	2671.2	7.5	2671.2	7.5
354-U1450A-124F-1W-39-138-Spot 36	218	104716	1.9	5.0712	0.5	15.0403	1.6	0.5532	1.5	0.95	2838.4	35.1	2817.8	15.3	2803.1	7.8	2803.1	7.8
354-U1450A-124F-1W-39-138-Spot 129	186	83540	1.6	4.7172	0.5	15.8531	1.8	0.5424	1.7	0.95	2793.4	39.0	2868.0	17.2	2920.8	8.8	2920.8	8.8
354-U1450A-124F-1W-39-138-Spot 258	78	560556	1.0	4.5411	0.5	18.0109	2.3	0.5932	2.2	0.98	3002.4	53.8	2990.3	22.0	2982.2	7.4	2982.2	7.4
354-U1450A-124F-1W-39-138-Spot 21	339	250797	2.8	3.7444	0.9	23.3319	2.0	0.6336	1.8	0.89	3163.9	44.6	3240.9	19.5	3288.9	14.1	3288.9	14.1
354-U1450A-124F-1W-39-138-Spot 58	306	152402	9.8	3.1980	0.7	29.5589	2.7	0.6856	2.6	0.97	3365.8	67.6	3472.3	26.3	3534.3	10.7	3534.3	10.7

Table ______. U-Pb geochronologic analyses.																		
#14 U1451A_47,48,49F_pilot						Isotope ratios						Apparent ages (Ma)						
Late Miocene 7.2 Ma																		
Analysis	U (ppm)	206Pb 204Pb	U/Th	206Pb* 207Pb*	± (%)	207Pb* 235U*	± (%)	206Pb* 238U	± (%)	error corr.	206Pb* 238U*	± (Ma)	207Pb* 235U	± (Ma)	206Pb* 207Pb*	± (Ma)	Best age (Ma)	± (Ma)
354U1451A47F_49F-Spot 229	735	28220	460.6	22.3297	2.7	0.0172	3.1	0.0028	1.5	0.47	17.9	0.3	17.3	0.5	67.4	65.8	17.9	0.3
354U1451A47F_49F-Spot 263	31	9604	373.6	20.6010	7.3	0.0377	7.8	0.0056	2.7	0.34	36.2	1.0	37.6	2.9	125.7	172.0	36.2	1.0
354U1451A47F_49F-Spot 97	143	9537	2.0	20.6381	3.9	0.0388	4.4	0.0058	1.8	0.42	37.3	0.7	38.7	1.7	121.5	93.0	37.3	0.7
354U1451A47F_49F-Spot 28	1353	142148	6.1	21.5186	1.3	0.0427	2.1	0.0067	1.7	0.80	42.8	0.7	42.4	0.9	22.2	30.3	42.8	0.7
354U1451A47F_49F-Spot 261	177	74897	1.1	21.2830	4.3	0.0497	4.6	0.0077	1.7	0.37	49.2	0.8	49.2	2.2	48.5	101.7	49.2	0.8
354U1451A47F_49F-Spot 260	63	99438	2.2	20.2370	5.5	0.0529	5.8	0.0078	1.9	0.33	49.8	0.9	52.3	3.0	167.5	129.3	49.8	0.9
354U1451A47F_49F-Spot 74	60	7840	1.4	20.4573	6.0	0.0529	6.4	0.0079	2.2	0.34	50.4	1.1	52.4	3.3	142.2	140.6	50.4	1.1
354U1451A47F_49F-Spot 119	19	5099	2.9	21.0941	7.5	0.0546	7.9	0.0084	2.6	0.32	53.6	1.4	54.0	4.2	69.8	178.1	53.6	1.4
354U1451A47F_49F-Spot 192	215	40257	1.4	21.4368	3.7	0.0539	4.0	0.0084	1.5	0.39	53.8	0.8	53.3	2.1	31.3	88.8	53.8	0.8
354U1451A47F_49F-Spot 52	18	3264	3.2	22.2494	8.6	0.0525	9.2	0.0085	3.1	0.34	54.3	1.7	51.9	4.7	58.6	210.9	54.3	1.7
354U1451A47F_49F-Spot 300	67	17228	2.1	18.4046	5.7	0.0725	6.0	0.0097	1.9	0.32	62.1	1.2	71.1	4.1	384.9	128.6	62.1	1.2
354U1451A47F_49F-Spot 193	964	81658	7.8	21.0899	1.4	0.0677	2.4	0.0104	1.9	0.79	66.4	1.2	66.5	1.5	70.2	34.4	66.4	1.2
354U1451A47F_49F-Spot 73	77	31107	2.0	21.0302	4.7	0.0887	5.1	0.0135	1.9	0.38	86.6	1.7	86.3	4.2	76.9	111.3	86.6	1.7
354U1451A47F_49F-Spot 86	49	15859	0.9	21.2369	4.7	0.0900	5.2	0.0139	2.0	0.39	88.8	1.8	87.5	4.3	53.7	113.2	88.8	1.8
354U1451A47F_49F-Spot 83	95	27763	1.2	21.0281	3.9	0.0942	4.4	0.0144	2.1	0.47	91.9	1.9	91.4	3.9	77.2	93.0	91.9	1.9
354U1451A47F_49F-Spot 302	46	56145	2.7	20.0647	4.8	0.1063	5.2	0.0155	2.0	0.38	98.9	2.0	102.5	5.0	187.5	111.1	98.9	2.0
354U1451A47F_49F-Spot 13	325	119434	4.0	20.7592	2.0	0.1151	2.6	0.0173	1.7	0.64	110.7	1.8	110.6	2.7	107.7	46.5	110.7	1.8
354U1451A47F_49F-Spot 3	278	70497	2.1	20.6607	1.3	0.1205	2.0	0.0181	1.4	0.73	115.3	1.6	115.5	2.1	118.9	31.7	115.3	1.6
354U1451A47F_49F-Spot 139	772	155639	2.0	20.3965	1.2	0.1319	1.7	0.0195	1.3	0.75	124.5	1.6	125.8	2.1	149.2	27.0	124.5	1.6
354U1451A47F_49F-Spot 125	15	4581	1.7	21.6938	6.4	0.1262	6.8	0.0199	2.4	0.35	126.7	3.0	120.6	7.8	2.7	154.0	126.7	3.0
354U1451A47F_49F-Spot 6	63	79984	3.3	20.5093	2.8	0.2110	3.1	0.0314	1.4	0.46	199.2	2.8	194.4	5.5	136.2	64.9	199.2	2.8
354U1451A47F_49F-Spot 40	505	81267	2.5	19.8444	1.3	0.2314	2.2	0.0333	1.8	0.82	211.2	3.8	211.4	4.2	213.1	29.1	211.2	3.8
354U1451A47F_49F-Spot 233	96	57242	2.2	19.2714	2.1	0.2809	2.4	0.0393	1.2	0.49	248.2	2.9	251.3	5.4	280.6	48.6	248.2	2.9
354U1451A47F_49F-Spot 226	471	193658	10.1	16.0063	1.5	0.4784	2.4	0.0555	1.9	0.80	348.4	6.6	397.0	8.0	690.4	31.2	348.4	6.6
354U1451A47F_49F-Spot 102	30	37774	1.3	16.4798	3.0	0.5069	6.1	0.0606	5.3	0.87	379.2	19.6	416.4	20.8	627.9	63.7	379.2	19.6
354U1451A47F_49F-Spot 67	140	56137	27.5	17.6749	1.5	0.5464	2.7	0.0700	2.2	0.83	436.4	9.4	442.7	9.6	475.1	33.0	436.4	9.4
354U1451A47F_49F-Spot 304	278	134530	1.3	17.4931	1.3	0.5839	2.2	0.0741	1.8	0.81	460.7	8.1	467.0	8.4	497.9	29.3	460.7	8.1
354U1451A47F_49F-Spot 25	233	81524	1.7	17.7730	1.3	0.5835	2.0	0.0752	1.6	0.78	467.5	7.0	466.7	7.5	462.9	27.9	467.5	7.0
354U1451A47F_49F-Spot 127	234	198377	1.2	17.5601	1.5	0.5923	2.4	0.0754	1.9	0.78	468.8	8.5	472.4	9.1	489.5	32.9	468.8	8.5
354U1451A47F_49F-Spot 201	437	155316	7.4	17.5090	1.0	0.5961	1.8	0.0757	1.6	0.85	470.4	7.1	474.7	7.0	495.9	21.1	470.4	7.1
354U1451A47F_49F-Spot 187	121	240361	12.8	17.7194	1.5	0.5966	2.4	0.0767	1.8	0.76	476.2	8.3	475.1	9.0	469.5	33.7	476.2	8.3
354U1451A47F_49F-Spot 163	134	99969	11.2	17.4215	1.5	0.6081	1.9	0.0768	1.1	0.59	477.2	5.3	482.4	7.4	506.9	34.0	477.2	5.3
354U1451A47F_49F-Spot 27	355	53592	1.5	17.7463	1.1	0.5977	2.1	0.0769	1.8	0.85	477.7	8.3	475.8	8.1	466.2	25.0	477.7	8.3
354U1451A47F_49F-Spot 90	287	123341	15.8	17.2832	1.0	0.6177	1.7	0.0774	1.4	0.79	480.7	6.3	488.4	6.6	524.4	22.8	480.7	6.3
354U1451A47F_49F-Spot 267	67	116628	1.3	17.2528	2.0	0.6202	2.5	0.0776	1.5	0.61	481.8	6.9	490.0	9.6	528.3	42.9	481.8	6.9
354U1451A47F_49F-Spot 82	86	39528	2.5	17.5665	1.6	0.6113	2.3	0.0779	1.6	0.70	483.5	7.4	484.4	8.8	488.7	35.8	483.5	7.4
354U1451A47F_49F-Spot 126	177	98819	2.4	17.7023	1.3	0.6089	2.0	0.0782	1.5	0.75	485.2	7.1	482.9	7.7	471.7	29.2	485.2	7.1
354U1451A47F_49F-Spot 80	145	148787	2.2	17.3312	1.3	0.6292	2.3	0.0791	1.9	0.82	490.6	8.9	495.6	9.1	518.3	29.3	490.6	8.9
354U1451A47F_49F-Spot 68	281	84947	11.4	17.4480	1.1	0.6253	1.7	0.0791	1.3	0.76	490.9	6.1	493.1	6.7	503.6	24.6	490.9	6.1
354U1451A47F_49F-Spot 298	728	317401	3.0	17.3469	1.0	0.6321	1.8	0.0795	1.5	0.83	493.3	7.2	497.4	7.3	516.4	22.8	493.3	7.2
354U1451A47F_49F-Spot 191	281	70122	3.5	17.5215	1.0	0.6294	1.5	0.0800	1.2	0.77	496.0	5.7	495.7	6.1	494.3	21.8	496.0	5.7
354U1451A47F_49F-Spot 274	286	52996	3.5	16.9522	1.0	0.6529	1.8	0.0803	1.5	0.82	497.7	7.2	510.2	7.3	566.7	22.6	497.7	7.2
354U1451A47F_49F-Spot 1	60	158889	1.9	17.0427	1.6	0.6526	2.2	0.0807	1.4	0.65	500.1	6.8	510.1	8.7	555.1	35.9	500.1	6.8
354U1451A47F_49F-Spot 31	33	47792	1.0	17.5026	1.9	0.6364	2.5	0.0808	1.7	0.65	500.8	8.0	500.1	10.1	496.7	42.6	500.8	8.0
354U1451A47F_49F-Spot 161	156	83608	1.1	16.8609	1.3	0.6611	2.3	0.0808	1.8	0.81	501.2	8.9	515.3	9.2	578.5	28.7	501.2	8.9
354U1451A47F_49F-Spot 156	35	202001	0.8	17.4619	2.0	0.6451	2.7	0.0817	1.8	0.66	506.3	8.7	505.5	10.8	501.9	44.6	506.3	8.7
354U1451A47F_49F-Spot 100	82	71968	1.8	17.3451	1.4	0.6508	2.0	0.0819	1.5	0.72	507.3	7.1	509.0	8.0	516.6	30.4	507.3	7.1
354U1451A47F_49F-Spot 79	325	1170562	3.7	17.4819	1.1	0.6495	3.0	0.0823	2.8	0.93	510.1	13.6	508.2	11.9	499.3	24.4	510.1	13.6
354U1451A47F_49F-Spot 308	173	100548	4.2	17.2066	1.7	0.6619	2.4	0.0826	1.7	0.69	511.7	8.1	515.8	9.7	534.2	37.7	511.7	8.1
354U1451A47F_49F-Spot 176	154	88331	3.2	17.3890	1.2	0.6551	1.9	0.0826	1.5	0.77	511.7	7.3	511.6	7.7	511.0	26.6	511.7	7.3
354U1451A47F_49F-Spot 224	40	205234	0.9	17.2722	2.4	0.6624	2.9	0.0830	1.7	0.59	513.9	8.6	516.1	11.9	525.8	52.2	513.9	8.6
354U1451A47F_49F-Spot 70	373	57088	2.3	17.4332	1.3	0.6576	2.3	0.0831	1.9	0.84	514.9	9.6	513.2	9.3	505.5	27.7	514.9	9.6
354U1451A47F_49F-Spot 222	859	59706	5.2	17.3606	0.7	0.6627	1.8	0.0834	1.6	0.91	516.7	8.0	516.3	7.2	514.6	15.9	516.7	8.0
354U1451A47F_49F-Spot 247	173	143775	2.7	17.3902	1.3	0.6620	2.5	0.0835	2.1	0.86	516.9	10.5	515.8	10.0	510.9	27.7	516.9	10.5
354U1451A47F_49F-Spot 207	184	55863	1.7	17.5003	1.3	0.6607	2.0	0.0839	1.6	0.78	519.1	7.8	515.1	8.1	497.0	27.6	519.1	7.8
354U1451A47F_49F-Spot 166	42	40026	7.7	17.3029	1.9	0.6688	2.3	0.0839	1.3	0.58	519.6	6.7	520.0	9.3	521.9	41.0	519.6	6.7
354U1451A47F_49F-Spot 200	66	54511	1.5	17.3648	1.6	0.6670	2.2	0.0840	1.6	0.69	520.0	7.8	518.9	9.1	514.1	35.5	520.0	7.8
354U1451A47F_49F-Spot 84	255	143029	1.6	17.3914	0.9	0.6699	2.0	0.0845	1.7	0.88	522.9	8.7	520.6	8.0	510.8	20.2	522.9	8.7
354U1451A47F_49F-Spot 273	64	212131	1.1	17.4103	1.5	0.6701	2.3	0.0846	1.7	0.76	523.6	8.6	520.8	9.2	508.4	32.0	523.6	8.6
354U1451A47F_49F-Spot 171	136	50067	1.8	17.1052	1.2	0.6936	1.7	0.0860	1.2	0.72	532.1	6.1	534.9	6.9	547.1	25.2	532.1	6.1
354U1451A47F_49F-Spot 61	982	72349	104.1	17.1361	1.0	0.6929	1.9	0.0861	1.6	0.84	532.5	8.4	534.5	8.1	543.2	22.9	532.5	8.4
354U1451A47F_49F-Spot 93	525	291039	20.6	16.9004	0.9	0.7071	1.5	0.0867	1.2	0.82	535.8	6.2	543.0	6.2	573.4	18.5	535.8	6.2
354U1451A47F_49F-Spot 110	167	74009	3.1	17.1511	1.2	0.7035	1.8	0.0875	1.3	0.74	540.8	6.8	540.9	7.4	541.3	25.7	540.8	6.8
354U1451A47F_49F-Spot 294	329	96366	193.3	17.0196	1.2	0.7117	1.8	0.0879	1.3	0.73	542.8	6.9	545.8	7.6	558.1	26.8	542.8	6.9
354U1451A47F_49F-Spot 64	1350	203101	5.0	17.2022	1.0	0.7080	1.9	0.0883	1.6	0.84	545.7	8.4	543.6	8.1	534.8	22.7	545.7	8.4
354U1451A47F_49F-Spot 173	115	82191	1.1	16.7148	1.4	0.7506	2.2	0.0910	1.7	0.77	561.4	9.1	568.6	9.5	597.3	29.9	561.4	9.1
354U1451A47F_49F-Spot 278	196	190369	4.1	16.5043	1.3	0.7629	2.0	0.0913	1.5	0.76	563.3	8.1	575.7	8.6	624.7	27.3	563.3	8.1
354U1451A47F_49F-Spot 109	23	28414	0.9	16.0185	2.2	0.7981	2.6	0.0927	1.5	0.56	571.6	8.1	595.8	11.8	688.8	46.4	571.6	8.1
354U1451A47F_49F-Spot 293	180	60146	2.4	16.7398	1.3	0.7796	2.1	0.0946	1.6	0.78	582.9	9.0	585.2	9.3	594.1	28.2	582.9	9.0
354U1451A47F_49F-Spot 155	283	220429	3.1	16.4447	0.9	0.8104	2.4	0.0967	2.2	0.93	594.8	12.5	602.7	10.8	632.5	19.2	594.8	12.5
354U1451A47F_49F-Spot 88	192	239399	18.2	16.3011	1.5	0.8334	2.4	0.0985	1.8	0.76	605.8	10.5	615.5	11.0	651.4	33.1	605.8	10.5
354U1451A47F_49F-Spot 206	292	163081	3.8	16.2676	1.0	0.8685	2.4	0.1025	2.2	0.92	628.9	13.4	634.8	11.5	655.8	20.6	628.9	13.4
354U1451A47F_49F-Spot 29	250	63908	3.4	16.3586	1.4	0.8958	2.4	0.1063	2.0	0.82	651.1	12.2	649.5	11.5	643.8	29.8	651.1	12.2
354U1451A47F_49F-Spot 268	106	89873	2.2	16.2932	1.5	0.9046	2.0	0.1069	1.3	0.65	654.7	8.2	654.2	9.6	652.4	32.5	654.7	8.2
354U1451A47F_49F-Spot 290	338	483609	33.5	15.9880	0.8	0.9231	1.4	0.1070	1.1	0.80	655.5	6.8	664.0	6.7	692.8	17.5	655.5	6.8
354U1451A47F_49F-Spot 309	282	84164	1.0	15.1820	1.0	1.0045	1.6	0.1106	1.3	0.79	676.2	8.1	706.1	8.1	802.1	20.4	676.2	8.1
354U1451A47F_49F-Spot 34	93	196545	1.8	15.9488	1.5	1.0108	2.3	0.1169	1.7	0.74	712.8	11.3	709.3	11.6	698.1	32.6	712.8	11.3
354U1451A47F_49F-Spot 48	34	101389	0.5	15.4321	1.7	1.0464	2.4	0.1171	1.7	0.70	714.0	11.5	727.1	12.6	767.8	36.2	714.0	11.5
354U1451A47F_49F-Spot 172	163	182161	9.7	15.8066	1.1	1.0318	1.7	0.1183	1.3	0.78	720.7	9.0	719.8	8.7	717.1	22.5	720.7	9.0

354U1451A47F_49F-Spot 53	11	13291	2.7	14.5758	3.0	1.1390	3.5	0.1204	1.7	0.49	732.9	11.9	772.0	18.9	886.9	62.8	732.9	11.9
354U1451A47F_49F-Spot 129	460	232994	4.1	14.7692	1.2	1.1311	1.9	0.1212	1.5	0.78	737.2	10.5	768.3	10.4	859.6	25.2	737.2	10.5
354U1451A47F_49F-Spot 117	68	53532	1.9	15.3658	1.5	1.0880	2.1	0.1212	1.4	0.68	737.7	9.9	747.5	11.0	776.9	32.0	737.7	9.9
354U1451A47F_49F-Spot 145	14	13433	1.4	15.2345	2.8	1.1089	3.1	0.1225	1.4	0.45	745.1	9.8	757.7	16.7	794.9	58.7	745.1	9.8
354U1451A47F_49F-Spot 258	153	206140	5.8	14.7289	1.7	1.1580	2.3	0.1237	1.6	0.69	751.8	11.2	781.0	12.6	865.3	34.7	751.8	11.2
354U1451A47F_49F-Spot 115	157	129022	2.3	14.5656	0.9	1.1716	1.6	0.1238	1.3	0.82	752.2	9.6	787.4	9.0	888.4	19.3	752.2	9.6
354U1451A47F_49F-Spot 136	750	1386558	2.6	15.5491	1.0	1.1001	1.6	0.1241	1.2	0.77	753.9	8.7	753.4	8.5	751.9	21.7	753.9	8.7
354U1451A47F_49F-Spot 9	209	73603	6.6	14.3700	0.9	1.2119	1.5	0.1263	1.3	0.82	766.8	9.2	806.1	8.6	916.3	18.0	766.8	9.2
354U1451A47F_49F-Spot 269	136	221496	3.3	14.8769	1.1	1.1724	2.4	0.1265	2.1	0.89	767.9	15.5	787.8	13.2	844.5	22.5	767.9	15.5
354U1451A47F_49F-Spot 313	339	58264	2.4	15.0126	1.2	1.1647	1.9	0.1268	1.4	0.75	769.7	10.1	784.2	10.1	825.6	25.4	769.7	10.1
354U1451A47F_49F-Spot 242	109	145418	1.7	15.3631	1.3	1.1441	2.0	0.1275	1.6	0.78	773.5	11.6	774.5	10.9	777.3	26.3	773.5	11.6
354U1451A47F_49F-Spot 279	116	99840	1.0	15.0760	1.2	1.1687	2.0	0.1278	1.5	0.79	775.2	11.3	786.0	10.7	816.9	25.2	775.2	11.3
354U1451A47F_49F-Spot 134	68	107120	6.5	15.3025	1.2	1.1571	2.0	0.1284	1.6	0.80	778.8	11.9	780.6	11.1	785.6	26.0	778.8	11.9
354U1451A47F_49F-Spot 167	202	164226	2.4	15.1121	1.1	1.1756	1.9	0.1289	1.6	0.84	781.3	12.1	789.3	10.7	811.8	22.0	781.3	12.1
354U1451A47F_49F-Spot 98	23	48205	2.6	14.8693	2.5	1.2022	3.0	0.1296	1.7	0.56	785.8	12.3	801.6	16.6	845.6	51.7	785.8	12.3
354U1451A47F_49F-Spot 203	56	38690	1.0	15.2559	1.4	1.1998	2.2	0.1328	1.8	0.79	803.6	13.4	800.5	12.4	792.0	28.9	803.6	13.4
354U1451A47F_49F-Spot 188	56	167104	1.5	15.2686	1.4	1.2052	2.0	0.1335	1.4	0.72	807.6	10.7	803.0	10.9	790.2	28.6	807.6	10.7
354U1451A47F_49F-Spot 264	433	86688	11.4	14.9459	0.8	1.2364	1.6	0.1340	1.4	0.88	810.8	11.0	817.3	9.2	834.9	16.2	810.8	11.0
354U1451A47F_49F-Spot 77	179	146200	6.2	14.9298	0.9	1.2568	1.7	0.1361	1.5	0.84	822.5	11.3	826.5	9.9	837.2	19.7	822.5	11.3
354U1451A47F_49F-Spot 24	90	60731	1.4	14.8531	1.2	1.2706	2.0	0.1369	1.6	0.82	827.0	12.7	832.7	11.4	847.9	24.1	827.0	12.7
354U1451A47F_49F-Spot 194	159	149307	3.5	14.4646	1.4	1.3061	2.5	0.1370	2.0	0.82	827.8	15.8	848.4	14.2	902.7	29.0	827.8	15.8
354U1451A47F_49F-Spot 103	19	46050	2.5	14.2638	2.0	1.3336	2.7	0.1380	1.9	0.70	833.2	15.1	860.5	15.9	931.5	40.2	833.2	15.1
354U1451A47F_49F-Spot 204	34	33292	1.7	14.9049	1.6	1.2783	2.3	0.1382	1.6	0.72	834.4	12.8	836.1	13.0	840.6	33.1	834.4	12.8
354U1451A47F_49F-Spot 285	102	178920	2.0	14.5201	1.1	1.3157	1.9	0.1386	1.5	0.81	836.5	11.8	852.6	10.7	894.8	22.4	836.5	11.8
354U1451A47F_49F-Spot 128	144	259688	7.0	14.9527	0.8	1.2779	1.5	0.1386	1.3	0.84	836.7	10.0	835.9	8.6	834.0	17.0	836.7	10.0
354U1451A47F_49F-Spot 185	78	189347	2.8	14.0553	1.3	1.3605	2.0	0.1387	1.5	0.74	837.2	11.7	872.1	11.7	961.7	27.3	837.2	11.7
354U1451A47F_49F-Spot 271	186	234226	9.2	14.8762	1.1	1.2968	2.2	0.1399	2.0	0.88	844.2	15.6	844.3	12.9	844.6	22.4	844.2	15.6
354U1451A47F_49F-Spot 45	222	204584	1.6	14.5256	1.2	1.3396	2.0	0.1411	1.5	0.79	851.1	12.3	863.1	11.3	894.0	24.8	851.1	12.3
354U1451A47F_49F-Spot 262	317	66772	16.1	14.8089	1.0	1.3376	1.9	0.1437	1.6	0.84	865.3	12.6	862.2	10.8	854.0	21.1	865.3	12.6
354U1451A47F_49F-Spot 270	83	88918	3.1	14.3771	1.1	1.3812	1.5	0.1440	1.1	0.71	867.3	8.5	880.9	8.8	915.3	21.7	867.3	8.5
354U1451A47F_49F-Spot 305	113	86380	1.7	14.4134	1.3	1.3798	1.9	0.1442	1.4	0.73	868.6	11.4	880.3	11.3	910.0	27.1	868.6	11.4
354U1451A47F_49F-Spot 287	124	80940	2.3	14.7378	1.0	1.3678	1.6	0.1462	1.3	0.79	879.7	10.7	875.2	9.7	864.1	21.2	879.7	10.7
354U1451A47F_49F-Spot 256	151	87811	1.7	14.4508	1.1	1.4089	1.8	0.1477	1.4	0.78	887.9	11.7	892.7	10.7	904.7	23.3	887.9	11.7
354U1451A47F_49F-Spot 234	93	68987	1.8	14.7021	1.2	1.3875	2.2	0.1480	1.8	0.82	889.5	14.8	883.6	12.8	869.1	25.4	889.5	14.8
354U1451A47F_49F-Spot 55	45	42263	1.1	14.4564	1.5	1.4194	2.4	0.1488	1.8	0.77	894.4	15.3	897.1	14.1	903.9	31.0	894.4	15.3
354U1451A47F_49F-Spot 168	509	95155	3.7	14.3809	1.1	1.4921	1.7	0.1556	1.4	0.79	932.4	11.9	927.2	10.6	914.7	22.0	914.7	22.0
354U1451A47F_49F-Spot 123	46	63673	1.1	14.3511	1.7	1.5385	2.3	0.1601	1.6	0.70	957.5	14.3	945.9	14.2	919.0	34.1	919.0	34.1
354U1451A47F_49F-Spot 254	87	145451	5.5	14.3210	1.4	1.4556	2.4	0.1512	1.9	0.80	907.6	15.9	912.2	14.2	923.3	29.1	923.3	29.1
354U1451A47F_49F-Spot 33	63	68348	2.6	14.1889	1.1	1.6109	1.9	0.1658	1.5	0.83	988.8	14.1	974.5	11.7	942.3	21.6	942.3	21.6
354U1451A47F_49F-Spot 216	246	168804	9.3	14.1557	0.7	1.5689	1.3	0.1611	1.1	0.85	962.8	10.3	958.0	8.3	947.1	14.3	947.1	14.3
354U1451A47F_49F-Spot 292	85	45154	2.8	14.1075	1.3	1.5412	2.0	0.1577	1.5	0.75	944.0	13.1	947.0	12.2	954.1	26.8	954.1	26.8
354U1451A47F_49F-Spot 299	33	114639	1.0	14.0904	1.5	1.5364	2.0	0.1570	1.3	0.68	940.2	11.8	945.1	12.2	956.5	29.7	956.5	29.7
354U1451A47F_49F-Spot 211	630	119182	3.4	14.0826	0.9	1.5806	1.7	0.1614	1.4	0.83	964.8	12.4	962.6	10.5	957.7	19.4	957.7	19.4
354U1451A47F_49F-Spot 89	161	77000	1.1	14.0822	1.0	1.5023	1.8	0.1534	1.5	0.82	920.2	12.5	931.3	10.8	957.7	20.4	957.7	20.4
354U1451A47F_49F-Spot 183	144	210630	6.7	14.0591	1.1	1.5268	2.3	0.1557	2.0	0.86	932.7	17.0	941.2	13.9	961.1	23.3	961.1	23.3
354U1451A47F_49F-Spot 99	540	134664	1.1	14.0376	0.8	1.5327	1.7	0.1560	1.6	0.90	934.8	13.5	943.6	10.7	964.2	15.6	964.2	15.6
354U1451A47F_49F-Spot 182	174	200452	10.6	14.0309	1.0	1.5732	1.8	0.1601	1.5	0.83	957.3	13.1	959.7	11.0	965.2	20.3	965.2	20.3
354U1451A47F_49F-Spot 281	361	84983	1.9	14.0214	0.9	1.6475	2.1	0.1675	1.9	0.90	998.5	17.3	988.6	13.2	966.6	18.9	966.6	18.9
354U1451A47F_49F-Spot 57	657	234890	12.6	14.0132	0.9	1.5838	2.4	0.1610	2.2	0.93	962.1	20.0	963.9	15.0	967.8	18.4	967.8	18.4
354U1451A47F_49F-Spot 96	49	370437	1.4	14.0090	1.4	1.5854	2.1	0.1611	1.6	0.75	962.8	14.0	964.5	12.9	968.4	28.0	968.4	28.0
354U1451A47F_49F-Spot 11	445	74354	4.8	14.0036	1.1	1.5533	2.0	0.1578	1.7	0.85	944.3	15.0	951.8	12.4	969.2	21.8	969.2	21.8
354U1451A47F_49F-Spot 250	215	140467	6.6	13.9892	1.1	1.5884	1.8	0.1612	1.5	0.81	963.2	13.2	965.7	11.4	971.3	21.6	971.3	21.6
354U1451A47F_49F-Spot 244	34	52133	2.7	13.9868	1.7	1.5373	2.6	0.1560	2.0	0.76	934.2	17.0	945.4	15.8	971.6	34.0	971.6	34.0
354U1451A47F_49F-Spot 36	460	56947	11.7	13.9836	1.1	1.5338	2.5	0.1556	2.2	0.90	932.0	19.4	944.0	15.3	972.1	22.2	972.1	22.2
354U1451A47F_49F-Spot 169	567	268736	59.7	13.9816	1.0	1.5663	1.7	0.1588	1.3	0.79	950.3	11.8	957.0	10.5	972.4	21.0	972.4	21.0
354U1451A47F_49F-Spot 277	148	106359	2.5	13.9794	0.8	1.6495	1.6	0.1672	1.3	0.84	996.9	12.1	989.3	9.9	972.7	17.3	972.7	17.3
354U1451A47F_49F-Spot 178	771	89243	10.0	13.9578	1.0	1.5933	1.8	0.1613	1.5	0.83	964.0	13.7	967.6	11.5	975.8	20.8	975.8	20.8
354U1451A47F_49F-Spot 5	92	107556	6.8	13.9263	0.9	1.6388	1.9	0.1655	1.6	0.88	987.4	15.1	985.3	11.8	980.4	18.2	980.4	18.2
354U1451A47F_49F-Spot 8	526	172702	2.8	13.8877	0.8	1.5754	2.0	0.1587	1.8	0.91	949.4	16.0	960.6	12.4	986.1	17.1	986.1	17.1
354U1451A47F_49F-Spot 295	45	74247	2.8	13.8538	1.4	1.6476	2.0	0.1655	1.4	0.71	987.5	12.9	988.6	12.5	991.1	28.1	991.1	28.1
354U1451A47F_49F-Spot 20	62	128991	1.6	13.8373	1.3	1.5479	2.0	0.1553	1.5	0.74	930.9	12.6	949.7	12.1	993.5	26.9	993.5	26.9
354U1451A47F_49F-Spot 113	71	36180	2.9	13.8153	1.6	1.4383	2.2	0.1441	1.5	0.68	867.9	12.4	905.0	13.4	996.7	33.3	996.7	33.3
354U1451A47F_49F-Spot 32	149	156651	2.2	13.7907	1.2	1.4980	2.1	0.1498	1.7	0.81	900.0	14.4	929.6	12.8	1000.3	24.8	1000.3	24.8
354U1451A47F_49F-Spot 159	102	136876	2.4	13.7759	1.4	1.7522	2.1	0.1751	1.6	0.74	1040.0	15.0	1028.0	13.6	1002.5	28.7	1002.5	28.7
354U1451A47F_49F-Spot 210	122	54502	2.4	13.7684	1.2	1.7296	2.1	0.1727	1.7	0.81	1027.1	16.2	1019.6	13.5	1003.6	24.8	1003.6	24.8
354U1451A47F_49F-Spot 142	206	73821	9.4	13.7331	1.0	1.6287	1.8	0.1622	1.5	0.83	969.1	13.4	981.3	11.3	1008.8	20.4	1008.8	20.4
354U1451A47F_49F-Spot 124	128	153937	2.7	13.7294	1.0	1.6265	1.7	0.1620	1.3	0.81	967.6	12.1	980.5	10.5	1009.4	20.1	1009.4	20.1
354U1451A47F_49F-Spot 71	76	121797	1.9	13.6860	1.4	1.5863	2.1	0.1575	1.6	0.76	942.6	13.9	964.9	13.0	1015.8	27.4	1015.8	27.4
354U1451A47F_49F-Spot 199	278	303475	0.8	13.6003	0.8	1.7377	1.5	0.1714	1.3	0.84	1019.8	12.2	1022.6	9.8	1028.5	16.6	1028.5	16.6
354U1451A47F_49F-Spot 65	270	113737	6.8	13.5735	0.8	1.4819	2.2	0.1459	2.1	0.93	877.8	17.0	923.0	13.5	1032.5	16.6	1032.5	16.6
354U1451A47F_49F-Spot 18	132	79094	1.9	13.5354	1.1	1.7949	2.2	0.1762	1.9	0.86	1046.2	17.9	1043.6	14.1	1038.2	22.3	1038.2	22.3
354U1451A47F_49F-Spot 147	125	123557	2.1	13.5131	1.0	1.5157	1.9	0.1485	1.6	0.85	892.8	13.6	936.7	11.7	1041.5	20.1	1041.5	20.1
354U1451A47F_49F-Spot 238	322	105229	3.0	13.4652	0.9	1.7483	2.0	0.1707	1.7	0.88	1016.2	16.5	1026.5	12.9	1048.7	19.1	1048.7	19.1
354U1451A47F_49F-Spot 252	1166	2323536	7.4	13.4289	0.9	1.8060	1.8	0.1759	1.6	0.88	1044.5	15.2	1047.6	11.7	1054.1	17.3	1054.1	17.3
354U1451A47F_49F-Spot 44	342	854874	2.8	13.4180	1.0	1.7876	2.0	0.1740	1.8	0.86	1033.9	16.8	1041.0	13.3	1055.8	21.0	1055.8	21.0
354U1451A47F_49F-Spot 149	201	71448	6.8	13.3909	1.1	1.8117	2.1	0.1760	1.7	0.84	1044.8	16.8	1049.7	13.6	1059.9	23.1	1059.9	23.1
354U1451A47F_49F-Spot 153	290	503668	2.7	13.3834	0.8	1.8075	1.4	0.1754	1.1	0.82	1042.0	11.0	1048.2	9.1	1061.0	16.2	1061.0	16.2
354U1451A47F_49F-Spot 311	349	751670	7.3	13.3546	1.1	1.6494	2.2	0.1598	1.8	0.85	955.4	16.4	989.3	13.7	1065.3	22.9	1065.3	22.9
354U1451A47F_49F-Spot 255	147	127304	1.5	13.3387	1.2	1.8269	1.8	0.1767	1.4	0.77	1049.1	13.7	1055.2	12.0	1067.7	23.6	1067.7	23.6
354U1451A47F_49F-Spot 133	214	95389	2.5	13.3337	1.1	1.8002	1.8	0.1741	1.5	0.81	1034.6	14.1	1045.5	11.9	1068.5	21.5	1068.5	21.5
354U1451A47F_49F-Spot 122	118	67492	4.2	13.2869	1.2	1.7576	2.0	0.1694	1.6	0.79	1008.7	14.7	1030.0	12.9	1075.5	24.5	1075.5	24.5
354U1451A47F_49F-Spot 301	572	115464	10.5	13.2214	0.8	1.8977	1.9	0.1820	1.7	0.91	1077.7	16.9	1080.3	12.5	1085.4	15.8	1085.4	15.8
354U1451A47F_49F-Spot 212	150	68616	10.7	13.1462	1.3	1.9762	2.1	0.1884	1.6	0.78	1112.8	16.8	1107.4	14.2	1096.8	26.5	1096.8	26.5
354U1451A47F_49F-Spot 195	20	78528	2.6	13.1380	1.7	1.7382	2.2	0.1656	1.4	0.64	988.0	12.7	1022.8	14.0	1098.1	33.5	1098.1	33.5
354U1451A47F_49F-Spot 118	12	55962	1.4	13.1094	1.9	1.8379	2.6	0.1747	1.8	0.69	1038.2	17.5	1059.1	17.4	1102.5	38.4	1102.5	38.4
354U1451A47F_49F-Spot 21	83	121258	2.0	13.0914	1.3	1.7986	2.4	0.1708	2.0	0.83	1016.4	18.7	1044.9	15.7	1105.2	26.9	1105.2	26.9

354U1451A47F_49F-Spot 181	152	246210	3.2	13.0586	1.1	1.9217	1.7	0.1820	1.3	0.77	1077.9	13.2	1088.7	11.6	1110.2	22.2	1110.2	22.2
354U1451A47F_49F-Spot 62	169	130861	2.7	13.0403	1.0	1.8174	2.1	0.1719	1.8	0.87	1022.5	17.0	1051.7	13.6	1113.0	20.6	1113.0	20.6
354U1451A47F_49F-Spot 265	68	124320	2.3	13.0046	1.5	1.8339	2.5	0.1730	2.0	0.80	1028.5	18.9	1057.7	16.4	1118.5	29.8	1118.5	29.8
354U1451A47F_49F-Spot 248	66	57029	136.3	12.8855	1.5	1.9866	2.2	0.1857	1.6	0.73	1097.8	16.5	1111.0	15.2	1136.8	30.7	1136.8	30.7
354U1451A47F_49F-Spot 30	280	203247	11.8	12.8820	0.8	1.9081	2.2	0.1783	2.1	0.93	1057.5	20.4	1083.9	14.9	1137.4	16.0	1137.4	16.0
354U1451A47F_49F-Spot 95	94	107269	2.1	12.8594	1.0	2.0929	1.7	0.1952	1.4	0.83	1149.5	15.0	1146.5	11.8	1140.8	19.1	1140.8	19.1
354U1451A47F_49F-Spot 26	258	68679	10.6	12.8277	0.9	1.9491	1.5	0.1813	1.2	0.80	1074.3	12.0	1098.1	10.2	1145.8	18.1	1145.8	18.1
354U1451A47F_49F-Spot 272	94	98773	0.8	12.7760	0.9	2.1683	2.1	0.2009	1.9	0.90	1180.2	20.6	1170.9	14.8	1153.8	18.6	1153.8	18.6
354U1451A47F_49F-Spot 232	243	288822	3.6	12.7621	1.1	2.0004	2.0	0.1852	1.7	0.84	1095.1	17.1	1115.6	13.7	1155.9	21.8	1155.9	21.8
354U1451A47F_49F-Spot 310	72	153189	5.0	12.7259	1.2	2.1246	2.2	0.1961	1.8	0.83	1154.3	18.8	1156.8	14.9	1161.6	24.0	1161.6	24.0
354U1451A47F_49F-Spot 225	35	143951	0.7	12.6828	1.2	2.2048	1.9	0.2028	1.5	0.79	1190.4	16.7	1182.6	13.6	1168.3	23.5	1168.3	23.5
354U1451A47F_49F-Spot 202	951	1294538	1.9	12.6756	0.9	2.1298	1.8	0.1958	1.6	0.86	1152.7	16.4	1158.5	12.5	1169.4	18.4	1169.4	18.4
354U1451A47F_49F-Spot 307	213	95735	3.3	12.6500	1.0	2.1234	1.4	0.1948	1.0	0.71	1147.4	10.2	1156.4	9.5	1173.4	19.4	1173.4	19.4
354U1451A47F_49F-Spot 150	311	184731	3.7	12.6488	0.9	2.2060	1.7	0.2024	1.4	0.84	1188.0	15.1	1182.9	11.6	1173.6	17.9	1173.6	17.9
354U1451A47F_49F-Spot 284	229	151354	2.4	12.6131	0.8	1.8878	1.7	0.1727	1.5	0.89	1026.9	14.7	1076.8	11.6	1179.2	15.8	1179.2	15.8
354U1451A47F_49F-Spot 175	95	151886	1.8	12.6035	1.1	2.0895	1.8	0.1910	1.4	0.78	1126.8	14.6	1145.4	12.4	1180.7	22.3	1180.7	22.3
354U1451A47F_49F-Spot 108	66	168268	12.5	12.5640	1.0	2.1814	1.6	0.1988	1.2	0.76	1168.7	12.6	1175.1	10.9	1186.9	20.2	1186.9	20.2
354U1451A47F_49F-Spot 170	107	701601	0.9	12.5589	1.1	2.1731	1.8	0.1979	1.4	0.80	1164.3	15.3	1172.5	12.6	1187.7	21.6	1187.7	21.6
354U1451A47F_49F-Spot 239	278	#######	2.6	12.5379	0.9	2.2597	2.2	0.2055	2.0	0.91	1204.7	21.7	1199.8	15.3	1191.0	18.1	1191.0	18.1
354U1451A47F_49F-Spot 220	233	58880	1.8	12.5116	1.0	2.2464	1.6	0.2038	1.3	0.78	1196.0	14.1	1195.7	11.6	1195.1	20.2	1195.1	20.2
354U1451A47F_49F-Spot 19	41	74860	1.0	12.4885	1.3	2.3236	1.8	0.2105	1.2	0.69	1231.3	13.6	1219.5	12.4	1198.8	24.9	1198.8	24.9
354U1451A47F_49F-Spot 75	63	185497	4.6	12.4685	1.2	2.1361	2.4	0.1932	2.0	0.85	1138.5	21.2	1160.6	16.4	1202.0	24.2	1202.0	24.2
354U1451A47F_49F-Spot 49	243	243750	2.4	12.3872	0.9	2.2268	1.6	0.2001	1.3	0.81	1175.6	13.9	1189.5	11.2	1214.8	18.4	1214.8	18.4
354U1451A47F_49F-Spot 114	168	212806	15.3	12.3473	1.0	2.2569	2.1	0.2021	1.9	0.88	1186.6	20.3	1198.9	14.9	1221.2	19.4	1221.2	19.4
354U1451A47F_49F-Spot 218	89	195945	6.4	12.2273	1.2	2.2618	2.0	0.2006	1.6	0.79	1178.4	17.0	1200.5	14.1	1240.4	24.3	1240.4	24.3
354U1451A47F_49F-Spot 106	455	797816	23.3	12.2061	0.8	2.4106	1.4	0.2134	1.1	0.80	1246.9	12.6	1245.8	10.0	1243.8	16.5	1243.8	16.5
354U1451A47F_49F-Spot 23	146	568538	4.3	12.1764	1.0	2.4308	1.9	0.2147	1.6	0.85	1253.6	18.1	1251.8	13.5	1248.5	19.7	1248.5	19.7
354U1451A47F_49F-Spot 296	381	211557	2.0	12.0053	1.0	2.4650	1.9	0.2146	1.6	0.85	1253.4	18.5	1261.8	13.8	1276.2	19.5	1276.2	19.5
354U1451A47F_49F-Spot 2	264	204353	1.3	11.9981	0.6	2.5484	1.3	0.2218	1.2	0.90	1291.2	14.0	1286.0	9.8	1277.3	11.6	1277.3	11.6
354U1451A47F_49F-Spot 94	129	119851	2.1	11.7870	1.2	2.4833	1.6	0.2123	1.1	0.69	1241.0	12.5	1267.2	11.6	1311.9	22.6	1311.9	22.6
354U1451A47F_49F-Spot 243	129	51479	4.1	11.6554	1.0	2.7115	1.9	0.2292	1.6	0.84	1330.4	19.3	1331.6	14.2	1333.6	20.2	1333.6	20.2
354U1451A47F_49F-Spot 157	77	174454	4.1	11.5727	1.0	2.6908	1.7	0.2258	1.4	0.83	1312.7	17.1	1325.9	12.9	1347.4	18.7	1347.4	18.7
354U1451A47F_49F-Spot 152	210	126845	0.8	11.5213	0.8	2.7507	1.5	0.2299	1.2	0.84	1333.7	15.1	1342.3	11.1	1355.9	15.8	1355.9	15.8
354U1451A47F_49F-Spot 144	80	131540	4.6	11.3681	0.9	2.8710	1.6	0.2367	1.3	0.83	1369.6	16.1	1374.3	11.8	1381.7	16.6	1381.7	16.6
354U1451A47F_49F-Spot 208	137	2997382	4.1	11.3283	1.0	2.9412	2.0	0.2416	1.7	0.86	1395.3	21.6	1392.6	15.2	1388.4	19.7	1388.4	19.7
354U1451A47F_49F-Spot 174	267	52494	1.9	11.2554	2.9	2.7625	3.6	0.2255	2.1	0.58	1310.9	24.9	1345.5	26.9	1400.8	56.3	1400.8	56.3
354U1451A47F_49F-Spot 16	33	26536	1.2	11.1737	1.3	3.0758	2.1	0.2493	1.6	0.77	1434.6	20.8	1426.7	16.1	1414.8	25.8	1414.8	25.8
354U1451A47F_49F-Spot 257	215	86823	2.6	11.1031	1.0	3.1169	2.0	0.2510	1.7	0.87	1443.6	22.3	1436.9	15.3	1426.9	18.8	1426.9	18.8
354U1451A47F_49F-Spot 87	127	102854	2.0	11.0851	1.0	3.0272	1.6	0.2434	1.2	0.76	1404.2	15.3	1414.5	12.2	1430.0	19.9	1430.0	19.9
354U1451A47F_49F-Spot 92	252	55853	5.9	11.0365	0.9	2.8239	1.8	0.2260	1.6	0.86	1313.7	18.6	1361.9	13.7	1438.4	17.9	1438.4	17.9
354U1451A47F_49F-Spot 76	212	222854	2.5	10.9916	1.0	3.1005	1.9	0.2472	1.6	0.86	1423.8	20.5	1432.8	14.4	1446.1	18.5	1446.1	18.5
354U1451A47F_49F-Spot 143	340	2490432	4.0	10.9261	1.2	3.1381	2.0	0.2487	1.6	0.82	1431.6	20.9	1442.1	15.3	1457.5	21.9	1457.5	21.9
354U1451A47F_49F-Spot 289	113	231687	7.3	10.9083	0.8	3.1302	1.5	0.2476	1.3	0.85	1426.3	16.0	1440.1	11.3	1460.6	14.6	1460.6	14.6
354U1451A47F_49F-Spot 137	378	185308	2.3	10.8895	0.9	2.9589	1.6	0.2337	1.3	0.81	1353.8	15.8	1397.1	12.0	1463.9	17.5	1463.9	17.5
354U1451A47F_49F-Spot 66	272	268469	17.3	10.7741	0.8	2.8422	1.9	0.2221	1.7	0.91	1292.9	20.3	1366.8	14.3	1484.1	15.0	1484.1	15.0
354U1451A47F_49F-Spot 245	359	191534	3.0	10.5968	1.1	3.4369	2.0	0.2641	1.7	0.83	1511.0	22.7	1512.9	15.9	1515.5	21.0	1515.5	21.0
354U1451A47F_49F-Spot 275	102	106416	2.2	10.1805	0.9	3.6730	1.7	0.2712	1.4	0.84	1546.9	19.6	1565.5	13.6	1590.7	17.3	1590.7	17.3
354U1451A47F_49F-Spot 288	41	130287	2.5	10.1191	1.2	3.8115	1.8	0.2797	1.4	0.76	1590.0	19.1	1595.2	14.3	1602.0	21.5	1602.0	21.5
354U1451A47F_49F-Spot 58	441	714615	6.2	10.1147	0.9	3.7968	1.5	0.2785	1.2	0.82	1584.0	17.5	1592.1	12.2	1602.8	16.1	1602.8	16.1
354U1451A47F_49F-Spot 164	35	235307	1.3	10.1007	1.3	3.7494	2.0	0.2747	1.6	0.77	1564.5	21.6	1582.0	16.2	1605.4	24.1	1605.4	24.1
354U1451A47F_49F-Spot 131	279	2432421	92.2	10.0365	1.0	3.9121	1.8	0.2848	1.5	0.82	1615.3	21.5	1616.2	14.8	1617.3	19.5	1617.3	19.5
354U1451A47F_49F-Spot 283	83	249168	2.2	10.0338	1.0	3.1853	1.9	0.2318	1.6	0.86	1343.9	19.4	1453.6	14.4	1617.8	18.0	1617.8	18.0
354U1451A47F_49F-Spot 227	109	73533	2.6	10.0282	0.9	3.8938	1.5	0.2832	1.3	0.82	1607.5	18.0	1612.4	12.5	1618.8	16.6	1618.8	16.6
354U1451A47F_49F-Spot 47	50	72643	2.4	10.0222	1.1	3.9274	2.1	0.2855	1.8	0.85	1618.9	26.1	1619.3	17.4	1619.9	21.0	1619.9	21.0
354U1451A47F_49F-Spot 37	84	117641	2.7	9.9790	1.1	3.8214	2.1	0.2766	1.8	0.85	1574.1	24.5	1597.3	16.5	1628.0	19.9	1628.0	19.9
354U1451A47F_49F-Spot 158	496	414008	92.8	9.9622	0.8	3.1103	2.4	0.2247	2.3	0.94	1306.8	26.7	1435.2	18.5	1631.1	15.5	1631.1	15.5
354U1451A47F_49F-Spot 259	51	215437	1.7	9.9221	1.1	3.9888	2.1	0.2870	1.8	0.84	1626.7	25.4	1631.9	17.1	1638.6	21.3	1638.6	21.3
354U1451A47F_49F-Spot 190	334	3216375	2.7	9.8598	0.8	4.0067	1.9	0.2865	1.7	0.90	1624.1	24.1	1635.6	15.1	1650.3	14.8	1650.3	14.8
354U1451A47F_49F-Spot 41	239	126201	13.6	9.8285	1.2	3.9408	2.1	0.2809	1.7	0.81	1596.0	23.4	1622.1	16.6	1656.2	22.5	1656.2	22.5
354U1451A47F_49F-Spot 105	65	170930	3.8	9.8244	1.2	3.8373	2.0	0.2734	1.6	0.79	1558.1	21.9	1600.6	16.1	1656.9	22.5	1656.9	22.5
354U1451A47F_49F-Spot 10	422	234734	2.5	9.7437	1.0	4.0686	1.9	0.2875	1.6	0.84	1629.1	23.1	1648.0	15.6	1672.2	19.2	1672.2	19.2
354U1451A47F_49F-Spot 235	215	59811	2.3	9.7102	1.1	4.2053	2.1	0.2962	1.8	0.87	1672.2	26.9	1675.0	17.3	1678.6	19.5	1678.6	19.5
354U1451A47F_49F-Spot 266	288	114232	1.5	9.6876	0.9	3.8869	1.7	0.2731	1.5	0.85	1556.5	20.3	1611.0	13.9	1682.9	16.7	1682.9	16.7
354U1451A47F_49F-Spot 12	67	168555	2.7	9.6430	1.1	4.1935	1.8	0.2933	1.4	0.79	1657.9	21.2	1672.7	15.1	1691.4	20.9	1691.4	20.9
354U1451A47F_49F-Spot 315	64	159954	23.0	9.6052	1.2	4.0589	1.8	0.2828	1.3	0.73	1605.2	18.5	1646.1	14.5	1698.6	22.4	1698.6	22.4
354U1451A47F_49F-Spot 81	69	134324	3.3	9.4996	1.0	4.4738	1.6	0.3082	1.3	0.79	1732.0	19.1	1726.1	13.3	1719.0	18.1	1719.0	18.1
354U1451A47F_49F-Spot 280	98	246757	2.7	9.4923	0.8	4.3739	1.6	0.3011	1.4	0.87	1696.8	20.9	1707.4	13.3	1720.4	14.7	1720.4	14.7
354U1451A47F_49F-Spot 78	149	160077	1.9	9.4620	1.0	4.3479	1.8	0.2984	1.6	0.86	1683.2	23.4	1702.5	15.2	1726.3	17.6	1726.3	17.6
354U1451A47F_49F-Spot 112	73	185951	3.1	9.4601	1.0	4.3544	1.8	0.2988	1.5	0.83	1685.2	21.7	1703.7	14.5	1726.6	18.0	1726.6	18.0
354U1451A47F_49F-Spot 230	240	61147	11.6	9.4140	1.2	3.8318	2.0	0.2616	1.6	0.80	1498.1	21.5	1599.4	16.2	1735.6	22.2	1735.6	22.2
354U1451A47F_49F-Spot 121	218	166501	8.5	9.3984	0.7	4.5500	1.5	0.3101	1.3	0.88	1741.4	20.2	1740.2	12.6	1738.6	13.3	1738.6	13.3
354U1451A47F_49F-Spot 177	69	435003	0.9	9.1566	1.2	4.2849	2.3	0.2846	1.9	0.83	1614.3	26.8	1690.5	18.6	1786.3	22.7	1786.3	22.7
354U1451A47F_49F-Spot 148	88	167896	6.9	9.0511	1.0	4.8572	1.6	0.3189	1.3	0.80	1784.1	20.3	1794.9	13.8	1807.4	18.0	1807.4	18.0
354U1451A47F_49F-Spot 135	62	166905	2.2	9.0219	1.0	4.9518	2.0	0.3240	1.7	0.87	1809.3	27.0	1811.1	16.6	1813.2	17.5	1813.2	17.5
354U1451A47F_49F-Spot 214	30	200532	1.4	8.9897	1.2	5.1728	2.1	0.3373	1.7	0.81	1873.5	27.2	1848.2	17.6	1819.7	22.1	1819.7	22.1
354U1451A47F_49F-Spot 104	209	106135	2.3	8.8686	0.7	5.0624	1.4	0.3256	1.2	0.85	1817.1	18.3	1829.8	11.6	1844.3	13.1	1844.3	13.1
354U1451A47F_49F-Spot 198	90	54398	2.6	8.8478	0.9	4.7435	1.6	0.3044	1.3	0.81	1713.0	19.9	1775.0	13.6	1848.6	17.1	1848.6	17.1
354U1451A47F_49F-Spot 43	50	140478	2.9	8.7642	1.2	5.3255	2.1	0.3385	1.8	0.83	1879.5	29.2	1873.0	18.4	1865.7	21.4	1865.7	21.4
354U1451A47F_49F-Spot 276	92	199258	8.3	8.7601	1.0	5.2765	1.8	0.3352	1.5	0.82	1863.7	24.6	1865.1	15.7	1866.5	18.8	1866.5	18.8
354U1451A47F_49F-Spot 249	124	80127	6.0	8.7567	0.9	5.3602	1.5	0.3404	1.2	0.81	1888.7	20.0	1878.5	12.8	1867.2	15.8	1867.2	15.8
354U1451A47F_49F-Spot 116	100	107399	4.2	8.7256	0.9	5.3585	1.8	0.3391	1.6	0.88	1882.4	26.0	1878.3	15.6	1873.7	15.8	1873.7	15.8
354U1451A47F_49F-Spot 205	192	221644	4.0	8.6862	0.8	5.3673	2.0	0.3381	1.8	0.90	1877.7	28.9	1879.6	16.8	1881.8	15.3	1881.8	15.3
354U1451A47F_49F-Spot 146	131	55033	2.2	8.3527	1.0	5.7509	1.7	0.3484	1.4	0.80	1926.9	23.3	1939.1	15.0	1952.1	18.4	1952.1	18.4
354U1451A47F_49F-Spot 219	248	125278	4.0	8.3440	1.1	5.3347	2.1	0.3228	1.8	0.85	1803.6	28.3	1874.4	18.1	1953.9	20.0	1953.9	20.0
354U1451A47F_49F-Spot 286	90	113142	6.3	8.3191	1.0	5.6621	1.7	0.3416	1.3	0.79	1894.5	22.0	1925.6	14.6	1959.3	18.4	1959.3	18.4
354U1451A47F_49F-Spot 186	28	66331	3.7	7.6730	1.1	6.6095	1.9	0.3678	1.6	0.81	2019.1	27.4	2060.6	17.2	2102.4	19.9	2102.4	19.9

354U1451A47F_49F-Spot 69	202	228206	1.4	7.5947	0.9	6.8059	1.7	0.3749	1.4	0.85	2052.3	24.9	2086.5	14.7	2120.4	15.3	2120.4	15.3
354U1451A47F_49F-Spot 306	171	152887	2.6	7.5516	1.1	5.8004	2.2	0.3177	2.0	0.88	1778.4	30.7	1946.5	19.4	2130.3	18.6	2130.3	18.6
354U1451A47F_49F-Spot 237	61	240939	2.8	6.8478	1.0	8.7231	1.9	0.4332	1.6	0.86	2320.3	32.1	2309.5	17.4	2300.0	16.5	2300.0	16.5
354U1451A47F_49F-Spot 85	99	68252	1.8	6.6855	1.0	7.0645	2.1	0.3425	1.8	0.88	1898.9	30.4	2119.6	18.6	2341.1	16.8	2341.1	16.8
354U1451A47F_49F-Spot 184	107	131017	2.4	6.5744	0.7	7.8463	1.3	0.3741	1.1	0.86	2048.8	19.7	2213.5	11.8	2369.7	11.6	2369.7	11.6
354U1451A47F_49F-Spot 251	180	53346	3.3	6.3948	0.9	8.4601	1.8	0.3924	1.6	0.88	2133.8	28.7	2281.6	16.4	2416.8	14.8	2416.8	14.8
354U1451A47F_49F-Spot 253	46	105724	1.7	6.3778	0.8	10.0660	1.6	0.4656	1.4	0.87	2464.3	28.1	2440.9	14.6	2421.4	13.4	2421.4	13.4
354U1451A47F_49F-Spot 209	570	6228067	23.3	6.3561	1.7	10.2048	2.5	0.4704	1.8	0.74	2485.4	37.7	2453.5	22.9	2427.2	28.3	2427.2	28.3
354U1451A47F_49F-Spot 213	253	142234	2.8	6.3297	0.7	10.1533	1.5	0.4661	1.3	0.89	2466.5	27.3	2448.8	13.8	2434.2	11.5	2434.2	11.5
354U1451A47F_49F-Spot 154	702	328380	1.4	6.2980	0.8	10.2857	1.5	0.4698	1.3	0.87	2482.8	27.4	2460.8	14.2	2442.7	12.9	2442.7	12.9
354U1451A47F_49F-Spot 189	125	209223	1.5	6.2110	0.8	9.3690	1.8	0.4220	1.6	0.90	2269.7	30.8	2374.8	16.3	2466.2	12.9	2466.2	12.9
354U1451A47F_49F-Spot 54	79	58386	3.3	6.1927	1.1	10.2305	2.1	0.4595	1.8	0.85	2437.3	36.1	2455.8	19.3	2471.2	18.5	2471.2	18.5
354U1451A47F_49F-Spot 7	214	118081	0.9	6.1789	0.9	10.2692	1.9	0.4602	1.7	0.88	2440.4	33.6	2459.3	17.3	2475.0	14.8	2475.0	14.8
354U1451A47F_49F-Spot 38	479	454789	11.9	6.1608	0.7	9.7895	1.6	0.4374	1.4	0.90	2339.1	28.1	2415.2	14.7	2479.9	11.7	2479.9	11.7
354U1451A47F_49F-Spot 138	225	267130	1.8	6.1293	0.9	10.1410	1.7	0.4508	1.4	0.83	2398.8	27.6	2447.7	15.3	2488.6	15.6	2488.6	15.6
354U1451A47F_49F-Spot 141	133	57459	3.7	6.1057	0.8	10.1781	1.9	0.4507	1.7	0.90	2398.4	34.9	2451.1	17.8	2495.1	13.8	2495.1	13.8
354U1451A47F_49F-Spot 236	149	259836	9.5	6.0086	1.0	11.3240	1.9	0.4935	1.6	0.85	2585.7	33.9	2550.2	17.4	2522.0	16.3	2522.0	16.3
354U1451A47F_49F-Spot 240	68	102974	1.6	5.9965	0.9	11.1580	1.6	0.4853	1.3	0.84	2550.2	28.0	2536.4	14.7	2525.4	14.4	2525.4	14.4
354U1451A47F_49F-Spot 223	74	95382	3.7	5.9634	0.9	9.8474	1.8	0.4259	1.5	0.85	2287.2	28.8	2420.6	16.2	2534.7	15.3	2534.7	15.3
354U1451A47F_49F-Spot 60	87	846762	2.2	5.5052	1.2	12.4812	1.9	0.4983	1.4	0.77	2606.7	30.8	2641.3	17.5	2667.9	19.7	2667.9	19.7
354U1451A47F_49F-Spot 217	65	1238712	2.4	5.3697	0.9	10.5672	2.0	0.4115	1.8	0.89	2221.9	33.4	2485.8	18.5	2709.2	15.1	2709.2	15.1
354U1451A47F_49F-Spot 246	68	278853	1.9	5.3632	0.8	13.9685	1.7	0.5433	1.5	0.88	2797.4	34.5	2747.6	16.4	2711.1	13.6	2711.1	13.6
354U1451A47F_49F-Spot 130	42	46217	2.5	5.2710	1.1	11.5012	2.3	0.4397	2.0	0.87	2349.2	39.7	2564.7	21.6	2739.7	18.6	2739.7	18.6
354U1451A47F_49F-Spot 162	117	71917	1.3	5.2151	0.9	13.8942	1.6	0.5255	1.4	0.85	2722.6	31.0	2742.5	15.6	2757.2	14.3	2757.2	14.3
354U1451A47F_49F-Spot 91	238	3199892	1.8	5.0407	0.9	14.3573	1.8	0.5249	1.5	0.87	2719.9	34.1	2773.6	16.8	2812.9	14.1	2812.9	14.1
354U1451A47F_49F-Spot 39	33	73507	2.3	4.9419	1.0	15.3755	1.8	0.5511	1.5	0.82	2829.7	33.3	2838.8	17.0	2845.3	16.7	2845.3	16.7
354U1451A47F_49F-Spot 4	300	#######	11.8	4.3940	1.4	17.0366	2.0	0.5429	1.5	0.74	2795.7	33.9	2936.9	19.4	3035.1	21.7	3035.1	21.7
354U1451A47F_49F-Spot 228	377	171436	6.9	4.3235	1.2	14.9698	3.1	0.4694	2.9	0.92	2481.0	58.7	2813.3	29.4	3061.0	19.0	3061.0	19.0
354U1451A47F_49F-Spot 107	65	217785	13.5	4.0314	1.0	20.3867	1.7	0.5961	1.4	0.83	3014.0	34.9	3109.9	16.9	3172.4	15.5	3172.4	15.5
354U1451A47F_49F-Spot 42	231	1398356	3.1	3.5919	0.9	24.7897	1.8	0.6458	1.5	0.86	3211.8	39.2	3300.0	17.6	3354.0	14.4	3354.0	14.4
354U1451A47F_49F-Spot 231	180	89735	6.4	3.3339	1.2	27.9795	2.0	0.6765	1.6	0.81	3331.0	42.8	3418.4	19.9	3470.0	18.5	3470.0	18.5

Table ______. U-Pb geochronologic analyses.																		
#15 U1451A_102F						Isotope ratios						Apparent ages (Ma)						
Late Miocene 9.8 Ma																		
Analysis	U (ppm)	206Pb 204Pb	U/Th	206Pb* 207Pb*	± (%)	207Pb* 235U*	± (%)	206Pb* 238U	± (%)	error corr.	206Pb* 238U*	± (Ma)	207Pb* 235U	± (Ma)	206Pb* 207Pb*	± (Ma)	Best age (Ma)	± (Ma)
Gleason_354-U1451A-102F-3W-0-102_5May16-Spot 11	1410	871	8.0	33.6204	1.6	0.0088	2.4	0.0022	1.8	0.75	13.9	0.2	8.9	0.2	1185.3	49.0	13.9	0.2
Gleason_354-U1451A-102F-3W-0-102_5May16-Spot 216	114	727	1.6	2.9000	1.4	0.2530	4.0	0.0053	3.7	0.93	34.2	1.3	229.0	8.2	3684.3	22.0	34.2	1.3
Gleason_354-U1451A-102F-3W-0-102_5May16-Spot 292	98	225	5.8	4.0265	1.7	0.2182	4.8	0.0064	4.5	0.94	40.9	1.8	200.4	8.8	3174.3	26.9	40.9	1.8
Gleason_354-U1451A-102F-3W-0-102_5May16-Spot 27	888	7488	0.6	22.4011	2.2	0.0423	3.4	0.0069	2.5	0.75	44.1	1.1	42.0	1.4	75.2	54.6	44.1	1.1
Gleason_354-U1451A-102F-3W-0-102_5May16-Spot 111	442	2876	0.9	19.9767	3.6	0.0495	4.4	0.0072	2.5	0.58	46.0	1.2	49.0	2.1	197.7	82.8	46.0	1.2
Gleason_354-U1451A-102F-3W-0-102_5May16-Spot 309	396	9516	1.2	21.3313	1.6	0.0498	2.9	0.0077	2.4	0.83	49.5	1.2	49.4	1.4	43.1	38.3	49.5	1.2
Gleason_354-U1451A-102F-3W-0-102_5May16-Spot 95	1065	4822	0.6	22.4598	1.9	0.0475	2.8	0.0077	2.0	0.74	49.6	1.0	47.1	1.3	81.6	45.6	49.6	1.0
Gleason_354-U1451A-102F-3W-0-102_5May16-Spot 107	1332	17432	3.1	20.7512	1.1	0.0518	2.1	0.0078	1.8	0.86	50.0	0.9	51.2	1.0	108.6	24.9	50.0	0.9
Gleason_354-U1451A-102F-3W-0-102_5May16-Spot 121	516	32185	1.1	20.5763	2.1	0.0525	3.3	0.0078	2.6	0.78	50.3	1.3	52.0	1.7	128.6	48.4	50.3	1.3
Gleason_354-U1451A-102F-3W-0-102_5May16-Spot 13	1973	19846	1.0	21.4180	1.2	0.0509	2.1	0.0079	1.7	0.83	50.8	0.9	50.4	1.0	33.4	28.1	50.8	0.9
Gleason_354-U1451A-102F-3W-0-102_5May16-Spot 141	649	6765	0.9	22.1003	1.8	0.0500	2.8	0.0080	2.1	0.75	51.4	1.1	49.5	1.3	42.2	44.8	51.4	1.1
Gleason_354-U1451A-102F-3W-0-102_5May16-Spot 211	251	277013	1.1	19.9594	2.5	0.0560	3.2	0.0081	2.1	0.64	52.1	1.1	55.3	1.7	199.7	57.6	52.1	1.1
Gleason_354-U1451A-102F-3W-0-102_5May16-Spot 102	98	1693	2.4	26.3023	4.5	0.0436	5.7	0.0083	3.5	0.61	53.4	1.9	43.4	2.4	483.8	119.5	53.4	1.9
Gleason_354-U1451A-102F-3W-0-102_5May16-Spot 22	76	488	1.0	55.1212	9.0	0.0217	10.2	0.0087	4.7	0.46	55.7	2.6	21.8	2.2	3088.8	1324.6	55.7	2.6
Gleason_354-U1451A-102F-3W-0-102_5May16-Spot 244	389	4016	2.1	21.4063	2.3	0.0584	3.6	0.0091	2.8	0.78	58.1	1.6	57.6	2.0	34.7	54.8	58.1	1.6
Gleason_354-U1451A-102F-3W-0-102_5May16-Spot 29	2091	30273	2.7	20.7274	0.9	0.0638	1.9	0.0096	1.7	0.87	61.5	1.0	62.8	1.2	111.3	22.0	61.5	1.0
Gleason_354-U1451A-102F-3W-0-102_5May16-Spot 214	351	30844	0.9	20.2599	1.5	0.0653	3.1	0.0096	2.7	0.88	61.6	1.7	64.3	1.9	164.9	35.1	61.6	1.7
Gleason_354-U1451A-102F-3W-0-102_5May16-Spot 209	373	2551	1.5	23.9502	3.8	0.0565	4.4	0.0098	2.2	0.51	63.0	1.4	55.8	2.4	241.4	95.3	63.0	1.4
Gleason_354-U1451A-102F-3W-0-102_5May16-Spot 25	103	3323	1.4	10.3895	7.1	0.1314	7.8	0.0099	3.3	0.42	63.5	2.1	125.3	9.2	1552.7	133.1	63.5	2.1
Gleason_354-U1451A-102F-3W-0-102_5May16-Spot 265	86	1780	1.1	19.5411	5.3	0.0783	6.4	0.0111	3.7	0.57	71.1	2.6	76.5	4.7	248.7	121.1	71.1	2.6
Gleason_354-U1451A-102F-3W-0-102_5May16-Spot 126	110	3093	1.1	23.5199	3.0	0.0688	4.1	0.0117	2.8	0.68	75.2	2.1	67.5	2.7	195.8	75.8	75.2	2.1
Gleason_354-U1451A-102F-3W-0-102_5May16-Spot 86	120	11557	1.0	18.3885	3.9	0.0992	5.2	0.0132	3.4	0.66	84.7	2.9	96.0	4.8	386.9	87.8	84.7	2.9
Gleason_354-U1451A-102F-3W-0-102_5May16-Spot 82	267	57515	1.6	20.4914	1.4	0.1134	2.6	0.0169	2.2	0.85	107.8	2.4	109.1	2.7	138.3	31.9	107.8	2.4
Gleason_354-U1451A-102F-3W-0-102_5May16-Spot 273	1042	68566	1.5	20.3644	1.0	0.1180	2.8	0.0174	2.6	0.94	111.4	2.9	113.2	3.0	152.9	22.7	111.4	2.9
Gleason_354-U1451A-102F-3W-0-102_5May16-Spot 258	904	101210	1.9	20.2028	1.3	0.1190	2.6	0.0174	2.3	0.87	111.5	2.5	114.2	2.9	171.5	30.1	111.5	2.5
Gleason_354-U1451A-102F-3W-0-102_5May16-Spot 315	743	15572	0.3	20.5472	1.0	0.1353	2.1	0.0202	1.9	0.88	128.6	2.4	128.8	2.6	131.9	23.6	128.6	2.4
Gleason_354-U1451A-102F-3W-0-102_5May16-Spot 55	261	1850	2.4	9.1688	10.6	0.4304	12.9	0.0286	7.5	0.58	181.9	13.4	363.5	39.6	1783.9	193.2	181.9	13.4
Gleason_354-U1451A-102F-3W-0-102_5May16-Spot 118	3215	482135	33.9	17.6082	2.4	0.2836	9.8	0.0362	9.5	0.97	229.3	21.4	253.5	22.0	483.5	53.2	229.3	21.4
Gleason_354-U1451A-102F-3W-0-102_5May16-Spot 268	2744	2018	4.1	11.2994	1.9	0.4660	2.7	0.0382	1.9	0.70	241.6	4.5	388.4	8.7	1393.4	36.7	241.6	4.5
Gleason_354-U1451A-102F-3W-0-102_5May16-Spot 19	551	30300	2.2	17.5425	0.7	0.5334	3.0	0.0679	2.9	0.97	423.3	11.7	434.1	10.5	491.7	16.5	423.3	11.7
Gleason_354-U1451A-102F-3W-0-102_5May16-Spot 112	5446	467036	1.2	17.3513	0.7	0.5473	2.8	0.0689	2.7	0.97	429.4	11.1	443.2	9.9	515.8	15.0	429.4	11.1
Gleason_354-U1451A-102F-3W-0-102_5May16-Spot 131	2046	105390	0.4	17.4735	0.7	0.5609	2.4	0.0711	2.3	0.96	442.7	10.0	452.1	8.9	500.4	15.7	442.7	10.0
Gleason_354-U1451A-102F-3W-0-102_5May16-Spot 103	1376	99013	3.1	17.6438	0.7	0.5651	2.1	0.0723	2.0	0.94	450.1	8.6	454.8	7.7	479.0	15.5	450.1	8.6
Gleason_354-U1451A-102F-3W-0-102_5May16-Spot 109	787	43641	9.6	17.6148	1.0	0.5788	2.2	0.0739	1.9	0.88	459.9	8.5	463.7	8.1	482.6	23.0	459.9	8.5
Gleason_354-U1451A-102F-3W-0-102_5May16-Spot 162	1152	58132	1.2	17.3205	0.7	0.5925	2.2	0.0744	2.1	0.95	462.8	9.2	472.5	8.2	519.7	15.4	462.8	9.2
Gleason_354-U1451A-102F-3W-0-102_5May16-Spot 160	414	96687	3.5	17.4620	1.0	0.6014	2.6	0.0762	2.4	0.92	473.2	10.8	478.1	9.8	501.8	21.9	473.2	10.8
Gleason_354-U1451A-102F-3W-0-102_5May16-Spot 108	1233	410220	0.3	17.1517	0.7	0.6123	1.9	0.0762	1.8	0.93	473.2	8.0	485.0	7.3	541.2	15.2	473.2	8.0
Gleason_354-U1451A-102F-3W-0-102_5May16-Spot 106	670	61443	11.7	17.5681	0.7	0.6034	1.8	0.0769	1.6	0.92	477.5	7.5	479.4	6.8	488.5	15.4	477.5	7.5
Gleason_354-U1451A-102F-3W-0-102_5May16-Spot 179	1493	459717	13.1	17.6815	0.7	0.6092	2.7	0.0781	2.6	0.96	484.9	11.9	483.0	10.2	474.3	16.3	484.9	11.9
Gleason_354-U1451A-102F-3W-0-102_5May16-Spot 296	2372	472672	5.7	17.1076	0.7	0.6304	2.6	0.0782	2.5	0.97	485.5	11.8	496.3	10.2	546.8	14.4	485.5	11.8
Gleason_354-U1451A-102F-3W-0-102_5May16-Spot 175	348	30119	6.9	17.7651	0.8	0.6085	2.1	0.0784	2.0	0.92	486.5	9.2	482.6	8.2	463.9	18.6	486.5	9.2
Gleason_354-U1451A-102F-3W-0-102_5May16-Spot 205	801	46614	2.0	17.3882	0.6	0.6286	2.1	0.0793	2.0	0.95	491.8	9.3	495.2	8.1	511.1	14.0	491.8	9.3
Gleason_354-U1451A-102F-3W-0-102_5May16-Spot 59	575	115681	2.7	17.4538	0.9	0.6281	2.2	0.0795	2.0	0.91	493.2	9.4	494.9	8.5	502.9	19.4	493.2	9.4
Gleason_354-U1451A-102F-3W-0-102_5May16-Spot 224	778	561113	4.4	17.3875	0.7	0.6364	2.2	0.0803	2.0	0.94	497.6	9.7	500.1	8.5	511.2	16.1	497.6	9.7
Gleason_354-U1451A-102F-3W-0-102_5May16-Spot 200	1906	185132	2.0	17.4184	0.7	0.6385	1.8	0.0807	1.7	0.93	500.0	8.0	501.4	7.1	507.3	14.3	500.0	8.0
Gleason_354-U1451A-102F-3W-0-102_5May16-Spot 56	368	50434	2.6	17.1665	0.9	0.6579	2.7	0.0819	2.5	0.94	507.5	12.4	513.3	10.9	539.3	20.5	507.5	12.4
Gleason_354-U1451A-102F-3W-0-102_5May16-Spot 291	403	81703	1.2	17.2176	0.7	0.6628	2.3	0.0828	2.2	0.95	512.6	10.9	516.3	9.4	532.8	15.8	512.6	10.9
Gleason_354-U1451A-102F-3W-0-102_5May16-Spot 32	411	71882	0.4	17.2012	0.7	0.6672	2.4	0.0832	2.3	0.96	515.4	11.2	519.0	9.6	534.9	15.0	515.4	11.2
Gleason_354-U1451A-102F-3W-0-102_5May16-Spot 18	159	25945	0.5	17.3038	1.0	0.6720	2.6	0.0843	2.4	0.92	521.9	12.2	521.9	10.8	521.8	22.2	521.9	12.2
Gleason_354-U1451A-102F-3W-0-102_5May16-Spot 302	603	39587	1.3	17.1177	0.8	0.6814	2.2	0.0846	2.1	0.94	523.5	10.4	527.6	9.0	545.5	16.6	523.5	10.4
Gleason_354-U1451A-102F-3W-0-102_5May16-Spot 149	347	390794	1.0	17.0732	0.8	0.6852	2.2	0.0848	2.1	0.93	525.0	10.4	529.9	9.1	551.2	17.6	525.0	10.4
Gleason_354-U1451A-102F-3W-0-102_5May16-Spot 96	230	16161	1.0	16.3777	1.4	0.7167	2.9	0.0851	2.6	0.89	526.7	13.2	548.7	12.5	641.3	29.5	526.7	13.2
Gleason_354-U1451A-102F-3W-0-102_5May16-Spot 182	62	8654	0.6	16.7483	1.5	0.7039	3.4	0.0855	3.1	0.90	528.9	15.7	541.1	14.4	593.0	32.2	528.9	15.7
Gleason_354-U1451A-102F-3W-0-102_5May16-Spot 71	330	40325	0.9	17.2084	0.7	0.6874	2.5	0.0858	2.4	0.96	530.6	12.4	531.2	10.5	534.0	15.8	530.6	12.4
Gleason_354-U1451A-102F-3W-0-102_5May16-Spot 267	241	36621	1.1	17.0645	0.9	0.7006	2.5	0.0867	2.3	0.93	536.0	12.0	539.1	10.4	552.3	19.5	536.0	12.0
Gleason_354-U1451A-102F-3W-0-102_5May16-Spot 155	2862	204156	17.8	16.9815	0.7	0.7048	1.6	0.0868	1.5	0.92	536.6	7.7	541.6	6.8	562.9	14.2	536.6	7.7
Gleason_354-U1451A-102F-3W-0-102_5May16-Spot 289	108	47380	3.4	17.1901	1.1	0.6999	3.2	0.0873	3.0	0.94	539.3	15.7	538.7	13.5	536.3	24.2	539.3	15.7
Gleason_354-U1451A-102F-3W-0-102_5May16-Spot 293	2743	538099	7.3	16.9205	0.7	0.7193	2.0	0.0883	1.9	0.93	545.3	9.9	550.3	8.6	570.8	15.7	545.3	9.9
Gleason_354-U1451A-102F-3W-0-102_5May16-Spot 153	606	22807	1.2	17.0548	0.8	0.7148	2.3	0.0884	2.1	0.93	546.2	11.2	547.6	9.7	553.5	17.9	546.2	11.2
Gleason_354-U1451A-102F-3W-0-102_5May16-Spot 312	419	177990	2.2	17.1941	0.8	0.7105	2.4	0.0886	2.2	0.95	547.3	11.7	545.0	9.9	535.8	16.8	547.3	11.7
Gleason_354-U1451A-102F-3W-0-102_5May16-Spot 72	679	109527	5.2	16.8567	0.7	0.7262	1.9	0.0888	1.8	0.92	548.3	9.2	554.3	8.1	579.0	15.8	548.3	9.2
Gleason_354-U1451A-102F-3W-0-102_5May16-Spot 313	553	425956	3.6	16.9190	0.6	0.7323	1.8	0.0899	1.6	0.94	554.7	8.7	557.9	7.5	571.0	13.2	554.7	8.7
Gleason_354-U1451A-102F-3W-0-102_5May16-Spot 231	571	37853	26.2	17.2003	0.9	0.7223	2.5	0.0901	2.4	0.94	556.2	12.7	552.0	10.8	535.0	18.9	556.2	12.7
Gleason_354-U1451A-102F-3W-0-102_5May16-Spot 203	295	23650	4.3	16.1221	1.1	0.8158	6.9	0.0954	6.9	0.99	587.4	38.5	605.7	31.7	675.0	23.7	587.4	38.5
Gleason_354-U1451A-102F-3W-0-102_5May16-Spot 36	721	48573	5.4	16.7968	0.6	0.7925	2.3	0.0965	2.3	0.97	594.1	12.9	592.6	10.5	586.7	12.3	594.1	12.9
Gleason_354-U1451A-102F-3W-0-102_5May16-Spot 140	363	36982	1.3	16.2996	0.8	0.8200	2.4	0.0969	2.3	0.94	596.4	12.8	608.1	11.0	651.5	17.3	596.4	12.8
Gleason_354-U1451A-102F-3W-0-102_5May16-Spot 92	1780	34233	5.1	15.6202	0.8	0.9025	2.1	0.1022	1.9	0.92	627.5	11.4	653.1	10.0	742.3	17.4	627.5	11.4
Gleason_354-U1451A-102F-3W-0-102_5May16-Spot 287	361	39786	1.2	16.5181	0.9	0.8551	2.2	0.1024	2.1	0.92	628.7	12.3	627.4	10.5	622.9	18.8	628.7	12.3
Gleason_354-U1451A-102F-3W-0-102_5May16-Spot 257	65	29549	2.3	16.4329	1.1	0.8900	3.6	0.1061	3.5	0.96	649.9	21.6	646.4	17.4	634.1	22.7	649.9	21.6
Gleason_354-U1451A-102F-3W-0-102_5May16-Spot 199	642	75180	2.2	15.2501	0.8	1.0001	3.9	0.1106	3.8	0.98	676.3	24.5	703.8	19.8	792.8	17.0	676.3	24.5
Gleason_354-U1451A-102F-3W-0-102_5May16-Spot 31	175	18463	1.3	15.9561	0.9	0.9965	2.4	0.1153	2.2	0.93	703.6	14.9	702.0	12.2	697.1	18.4	703.6	14.9
Gleason_354-U1451A-102F-3W-0-102_5May16-Spot 163	1211	232853	36.0	14.4614	1.0	1.1778	2.2	0.1235	2.0	0.90	750.8	14.1	790.3	12.2	903.2	20.3	750.8	14.1
Gleason_354-U1451A-102F-3W-0-102_5May16-Spot 6	509	224207	1.4	15.6418	0.6	1.1167	2.4	0.1267	2.4	0.97	768.9	17.2	761.4	13.1	739.3	12.2	768.9	17.2
Gleason_354-U1451A-102F-3W-0-102_5May16-Spot 26	797	5825758	0.4	15.2511	0.8	1.1583	2.8	0.1281	2.6	0.95	777.2	19.2	781.2	15.0	792.6	17.7	777.2	19.2
Gleason_354-U1451A-102F-3W-0-102_5May16-Spot 213	254	76401	0.4	15.2036	0.9	1.1701	2.5	0.1290	2.3	0.94	782.3	17.1	786.7	13.6	799.2	18.3	782.3	17.1
Gleason_354-U1451A-102F-3W-0-102_5May16-Spot 2	232	158026	0.8	15.2141	0.8	1.1953	2.2	0.1319	2.1	0.93	798.7	15.5	798.4	12.3	797.7	16.9	798.7	15.5
Gleason_354-U1451A-102F-3W-0-102_5May16-Spot 122	890	66647	1.9	14.9424	0.7	1.2320	2.1	0.1335	2.0	0.94	807.9	15.2	815.3	12.0	835.4	15.3	807.9	15.2
Gleason_354-U1451A-102F-3W-0-102_5May16-Spot 94	1046	554008	10.1	14.7412	0.6	1.2493	1.6	0.1336	1.5	0.93	808.2	11.6	823.1	9.2	863.6	12.2	808.2	11.6
Gleason_354-U1451A-102F-3W-0-102_5May16-Spot 181	114	17925	1.0	15.2667	0.9	1.2073	3.1	0.1337	2.9	0.96	808.8	22.3	804.0	17.1	790.5	19.0	808.8	22.3
Gleason_354-U1451A-102F-3W-0-102_5May16-Spot 88	731	72983	4.9	14.6997	0.7	1.2656	2.1	0.1349	2.0	0.94	815.9	15.1	830.4	11.9	869.4	14.9	815.9	15.1
Gleason_354-U1451A-102F-3W-0-102_5May16-Spot 148	292	24240	0.6	14.8754	0.7	1.2514	2.4	0.1350	2.3	0.96	816.4	18.0	824.0	13.7	844.7	13.7	816.4	18.0
Gleason_354-U1451A-102F-3W-0-102_5May16-Spot 266	80	29222	1.2	14.9523	1.0	1.2508	3.2	0.1356	3.0	0.94	820.0	23.0	823.8	17.9	834.0	21.7	820.0	23.0
Gleason_354-U1451A-102F-3W-0-102_5May16-Spot 212	553	145473	2.0	14.8528	0.9	1.2613	2.8	0.1359	2.6	0.94	821.3	20.1	828.5	15.6	847.9	18.9	821.3	20.1
Gleason_354-U1451A-102F-3W-0-102_5May16-Spot 79	480	93539	4.4	14.6901	0.8	1.2793	2.4	0.1363	2.2	0.94	823.7	17.2	836.5	13.5	870.8	16.1	823.7	17.2
Gleason_354-U1451A-102F-3W-0-102_5May16-Spot 138	219	250887	3.2	14.8244	0.9	1.2734	2.5	0.1369	2.3	0.93	827.2	17.9	833.9	14.0	851.9	18.2	827.2	17.9
Gleason_354-U1451A-102F-3W-0-102_5May16-Spot 222	550	204528	4.4	15.0514	0.9	1.2652	2.4	0.1381	2.3	0.93	834.0	17.7	830.2	13.8	820.3	18.7	834.0	17.7
Gleason_354-U1451A-102F-3W-0-102_5May16-Spot 236	551	41557	0.7	14.8503	0.6	1.2826	1.7	0.1381	1.6	0.94	834.1	12.8	838.0	10.0	848.3	12.6	834.1	12.8
Gleason_354-U1451A-102F-3W-0-102_5May16-Spot 53	19	4292	1.8	15.0868	2.2	1.2633	5.0	0.1382	4.5	0.90	834.7	35.6	829.4	28.5	815.3	45.3	834.7	35.6

Gleason_354-U1451A-102F-3W-0-102_5May16-Spot 171	750	168711	1.3	14.6078	0.7	1.3230	2.0	0.1402	1.8	0.94	845.6	14.6	855.8	11.4	882.4	14.3	845.6	14.6
Gleason_354-U1451A-102F-3W-0-102_5May16-Spot 242	262	61025	4.2	14.5237	0.9	1.3393	2.1	0.1411	1.9	0.90	850.7	15.0	862.9	12.2	894.4	19.0	850.7	15.0
Gleason_354-U1451A-102F-3W-0-102_5May16-Spot 28	1562	79811	1.6	14.6242	0.6	1.3309	1.9	0.1412	1.8	0.95	851.2	14.2	859.3	10.9	880.1	12.5	851.2	14.2
Gleason_354-U1451A-102F-3W-0-102_5May16-Spot 218	707	136161	3.0	14.5336	0.8	1.3511	1.9	0.1424	1.7	0.91	858.3	13.8	868.0	11.0	892.9	15.8	858.3	13.8
Gleason_354-U1451A-102F-3W-0-102_5May16-Spot 50	28	21263	2.8	14.6878	1.4	1.3463	5.0	0.1434	4.8	0.96	864.0	38.8	866.0	29.2	871.1	29.2	864.0	38.8
Gleason_354-U1451A-102F-3W-0-102_5May16-Spot 60	460	217833	1.7	14.7656	0.6	1.3420	1.7	0.1437	1.6	0.95	865.6	13.3	864.1	10.1	860.1	11.7	865.6	13.3
Gleason_354-U1451A-102F-3W-0-102_5May16-Spot 114	456	152440	2.9	14.5528	1.0	1.3635	2.8	0.1439	2.6	0.94	866.8	21.3	873.4	16.4	890.2	19.9	866.8	21.3
Gleason_354-U1451A-102F-3W-0-102_5May16-Spot 304	5149	235290	23.7	14.6810	0.7	1.3548	1.9	0.1443	1.8	0.94	868.7	14.6	869.6	11.2	872.1	13.5	868.7	14.6
Gleason_354-U1451A-102F-3W-0-102_5May16-Spot 196	240	29126	0.5	14.6522	0.7	1.3577	2.7	0.1443	2.6	0.97	868.8	21.3	870.9	15.9	876.1	14.7	868.8	21.3
Gleason_354-U1451A-102F-3W-0-102_5May16-Spot 232	443	385125	6.5	14.4247	0.5	1.3840	1.8	0.1448	1.8	0.96	871.7	14.3	882.1	10.8	908.4	10.4	871.7	14.3
Gleason_354-U1451A-102F-3W-0-102_5May16-Spot 85	316	78500	2.6	14.6478	0.7	1.3756	2.1	0.1461	1.9	0.95	879.3	16.0	878.6	12.1	876.7	13.8	879.3	16.0
Gleason_354-U1451A-102F-3W-0-102_5May16-Spot 284	774	206546	2.0	14.4974	0.6	1.3903	1.9	0.1462	1.8	0.94	879.5	14.4	884.8	11.0	898.1	12.7	879.5	14.4
Gleason_354-U1451A-102F-3W-0-102_5May16-Spot 20	220	35366	1.6	14.5453	0.8	1.4447	2.7	0.1524	2.6	0.96	914.4	22.3	907.7	16.4	891.3	16.7	891.3	16.7
Gleason_354-U1451A-102F-3W-0-102_5May16-Spot 15	252	52409	1.1	14.6292	1.0	1.4210	3.0	0.1508	2.8	0.94	905.3	23.7	897.8	17.8	879.4	20.6	905.3	23.7
Gleason_354-U1451A-102F-3W-0-102_5May16-Spot 310	927	279559	2.5	14.6128	0.5	1.4252	2.1	0.1510	2.0	0.97	906.8	17.1	899.5	12.5	881.7	11.3	906.8	17.1
Gleason_354-U1451A-102F-3W-0-102_5May16-Spot 23	138	68364	0.9	14.4179	0.8	1.4699	2.9	0.1537	2.8	0.96	921.7	23.8	918.1	17.4	909.4	16.1	909.4	16.1
Gleason_354-U1451A-102F-3W-0-102_5May16-Spot 169	301	35110	1.5	14.3658	0.9	1.4347	2.6	0.1495	2.4	0.94	898.0	20.4	903.5	15.5	916.9	18.0	916.9	18.0
Gleason_354-U1451A-102F-3W-0-102_5May16-Spot 16	170	164081	0.5	14.3644	0.9	1.4517	2.5	0.1512	2.3	0.93	907.9	19.8	910.6	15.1	917.1	18.7	917.1	18.7
Gleason_354-U1451A-102F-3W-0-102_5May16-Spot 5	411	43871	2.0	14.2703	0.8	1.5193	2.1	0.1572	1.9	0.93	941.5	16.7	938.2	12.6	930.5	15.4	930.5	15.4
Gleason_354-U1451A-102F-3W-0-102_5May16-Spot 100	1535	63106	1.2	14.2591	0.6	1.4499	2.0	0.1499	1.9	0.95	900.6	15.6	909.8	11.8	932.2	12.9	932.2	12.9
Gleason_354-U1451A-102F-3W-0-102_5May16-Spot 226	30	19122	1.7	14.1949	1.1	1.5912	4.3	0.1638	4.2	0.97	978.0	37.7	966.8	26.9	941.5	22.9	941.5	22.9
Gleason_354-U1451A-102F-3W-0-102_5May16-Spot 219	384	24415	0.9	14.1898	0.9	1.4938	2.5	0.1537	2.3	0.94	921.9	20.0	927.9	15.1	942.2	17.5	942.2	17.5
Gleason_354-U1451A-102F-3W-0-102_5May16-Spot 21	166	76180	0.9	14.1666	0.7	1.5857	2.2	0.1629	2.1	0.95	973.0	18.6	964.6	13.5	945.5	14.5	945.5	14.5
Gleason_354-U1451A-102F-3W-0-102_5May16-Spot 58	1154	168695	14.3	14.1214	0.7	1.3803	2.2	0.1414	2.1	0.94	852.4	16.5	880.6	12.9	952.1	14.6	952.1	14.6
Gleason_354-U1451A-102F-3W-0-102_5May16-Spot 215	646	829188	1.9	14.0928	0.9	1.5634	2.0	0.1598	1.8	0.90	955.6	16.0	955.8	12.3	956.2	17.5	956.2	17.5
Gleason_354-U1451A-102F-3W-0-102_5May16-Spot 188	411	507004	2.2	14.0926	0.7	1.5571	1.9	0.1591	1.7	0.92	952.0	15.4	953.3	11.7	956.2	15.0	956.2	15.0
Gleason_354-U1451A-102F-3W-0-102_5May16-Spot 229	235	22736	0.7	14.0758	0.6	1.6230	2.0	0.1657	2.0	0.96	988.3	17.9	979.2	12.9	958.7	12.4	958.7	12.4
Gleason_354-U1451A-102F-3W-0-102_5May16-Spot 77	828	115777	6.8	14.0634	0.8	1.5679	2.4	0.1599	2.3	0.95	956.3	20.4	957.6	15.0	960.5	16.0	960.5	16.0
Gleason_354-U1451A-102F-3W-0-102_5May16-Spot 202	250	148363	1.7	14.0438	0.8	1.5739	2.1	0.1603	1.9	0.92	958.5	16.8	960.0	12.7	963.3	16.6	963.3	16.6
Gleason_354-U1451A-102F-3W-0-102_5May16-Spot 64	328	45892	3.8	14.0304	0.6	1.6209	2.5	0.1649	2.4	0.97	984.2	22.1	978.4	15.7	965.3	12.9	965.3	12.9
Gleason_354-U1451A-102F-3W-0-102_5May16-Spot 249	313	126130	1.1	13.9961	0.8	1.5847	3.0	0.1609	2.9	0.96	961.6	25.7	964.2	18.6	970.3	16.1	970.3	16.1
Gleason_354-U1451A-102F-3W-0-102_5May16-Spot 158	657	298994	6.1	13.9806	0.6	1.4547	1.8	0.1475	1.7	0.94	886.9	14.0	911.8	10.8	972.5	12.3	972.5	12.3
Gleason_354-U1451A-102F-3W-0-102_5May16-Spot 52	160	37335	0.8	13.9795	0.7	1.6081	2.4	0.1630	2.3	0.96	973.7	20.7	973.4	14.9	972.7	14.0	972.7	14.0
Gleason_354-U1451A-102F-3W-0-102_5May16-Spot 161	103	86406	0.6	13.9771	0.9	1.6691	2.9	0.1692	2.8	0.95	1007.7	25.8	996.8	18.5	973.1	18.9	973.1	18.9
Gleason_354-U1451A-102F-3W-0-102_5May16-Spot 57	258	411519	1.4	13.9467	1.0	1.6131	2.6	0.1632	2.4	0.93	974.4	21.8	975.3	16.3	977.5	19.9	977.5	19.9
Gleason_354-U1451A-102F-3W-0-102_5May16-Spot 61	157	227783	1.3	13.9269	0.8	1.5072	2.7	0.1522	2.6	0.95	913.5	22.0	933.3	16.6	980.4	17.1	980.4	17.1
Gleason_354-U1451A-102F-3W-0-102_5May16-Spot 207	682	206086	0.7	13.9254	0.8	1.5792	2.5	0.1595	2.3	0.94	954.0	20.6	962.1	15.3	980.6	16.4	980.6	16.4
Gleason_354-U1451A-102F-3W-0-102_5May16-Spot 128	227	85912	1.6	13.8904	1.0	1.4605	2.6	0.1471	2.4	0.92	884.9	20.0	914.2	15.8	985.7	20.7	985.7	20.7
Gleason_354-U1451A-102F-3W-0-102_5May16-Spot 178	464	559232	1.7	13.8750	0.7	1.5112	2.4	0.1521	2.3	0.95	912.6	19.2	934.9	14.5	988.0	14.8	988.0	14.8
Gleason_354-U1451A-102F-3W-0-102_5May16-Spot 46	937	1232259	5.0	13.8637	0.5	1.6016	1.9	0.1610	1.8	0.96	962.5	16.5	970.8	12.0	989.6	10.4	989.6	10.4
Gleason_354-U1451A-102F-3W-0-102_5May16-Spot 48	490	88385	1.8	13.8446	0.7	1.6241	2.0	0.1631	1.9	0.93	973.8	17.0	979.6	12.6	992.4	14.6	992.4	14.6
Gleason_354-U1451A-102F-3W-0-102_5May16-Spot 124	111	34176	0.7	13.8271	0.7	1.6881	3.2	0.1693	3.1	0.98	1008.2	28.8	1004.0	20.2	995.0	14.1	995.0	14.1
Gleason_354-U1451A-102F-3W-0-102_5May16-Spot 75	51	118086	1.7	13.7978	1.1	1.5085	4.1	0.1510	4.0	0.97	906.3	33.8	933.8	25.2	999.3	21.5	999.3	21.5
Gleason_354-U1451A-102F-3W-0-102_5May16-Spot 67	684	69568	3.2	13.7781	0.7	1.5585	2.4	0.1557	2.3	0.96	933.1	19.9	953.9	14.8	1002.2	13.6	1002.2	13.6
Gleason_354-U1451A-102F-3W-0-102_5May16-Spot 134	636	98862	9.1	13.7493	1.5	1.3479	2.8	0.1344	2.4	0.85	813.0	18.4	866.6	16.6	1006.4	30.6	1006.4	30.6
Gleason_354-U1451A-102F-3W-0-102_5May16-Spot 307	1092	116457	1.2	13.7431	0.6	1.6266	2.0	0.1621	1.9	0.94	968.6	16.8	980.5	12.5	1007.4	13.1	1007.4	13.1
Gleason_354-U1451A-102F-3W-0-102_5May16-Spot 74	302	116423	3.4	13.7055	0.8	1.6105	3.3	0.1601	3.2	0.97	957.3	28.5	974.3	20.7	1012.9	16.3	1012.9	16.3
Gleason_354-U1451A-102F-3W-0-102_5May16-Spot 248	389	2731169	1.5	13.6678	0.8	1.4295	3.2	0.1417	3.1	0.97	854.3	24.8	901.3	19.1	1018.5	15.3	1018.5	15.3
Gleason_354-U1451A-102F-3W-0-102_5May16-Spot 190	1092	314544	28.9	13.6087	0.5	1.6478	2.1	0.1626	2.0	0.97	971.4	18.4	988.7	13.3	1027.3	10.5	1027.3	10.5
Gleason_354-U1451A-102F-3W-0-102_5May16-Spot 247	1087	124865	3.3	13.5784	0.7	1.6974	2.3	0.1672	2.2	0.95	996.4	20.5	1007.5	14.9	1031.8	14.3	1031.8	14.3
Gleason_354-U1451A-102F-3W-0-102_5May16-Spot 3	317	216604	2.4	13.5007	0.7	1.7347	2.4	0.1699	2.3	0.95	1011.3	21.2	1021.5	15.3	1043.4	14.4	1043.4	14.4
Gleason_354-U1451A-102F-3W-0-102_5May16-Spot 151	327	14018	0.8	13.4284	2.6	1.4585	3.5	0.1421	2.3	0.67	856.2	18.8	913.4	21.1	1054.2	52.3	1054.2	52.3
Gleason_354-U1451A-102F-3W-0-102_5May16-Spot 142	38	10086	0.8	13.3713	0.9	1.8858	4.7	0.1829	4.6	0.98	1082.7	46.0	1076.1	31.2	1062.8	18.6	1062.8	18.6
Gleason_354-U1451A-102F-3W-0-102_5May16-Spot 191	490	220250	1.2	13.3543	0.8	1.7328	2.1	0.1678	1.9	0.93	1000.1	17.9	1020.8	13.4	1065.4	15.8	1065.4	15.8
Gleason_354-U1451A-102F-3W-0-102_5May16-Spot 144	783	90711	1.9	13.3341	0.6	1.7623	2.1	0.1704	2.0	0.95	1014.5	18.6	1031.7	13.5	1068.4	12.5	1068.4	12.5
Gleason_354-U1451A-102F-3W-0-102_5May16-Spot 34	350	71479	1.9	13.3259	0.6	1.8701	2.1	0.1807	2.0	0.96	1071.0	20.0	1070.6	14.0	1069.6	12.3	1069.6	12.3
Gleason_354-U1451A-102F-3W-0-102_5May16-Spot 150	490	158529	1.2	13.3203	0.6	1.8657	2.1	0.1802	2.0	0.96	1068.3	20.1	1069.0	14.0	1070.5	11.9	1070.5	11.9
Gleason_354-U1451A-102F-3W-0-102_5May16-Spot 198	331	701824	1.0	13.3069	0.7	1.8794	2.4	0.1814	2.2	0.95	1074.5	22.2	1073.9	15.6	1072.5	14.3	1072.5	14.3
Gleason_354-U1451A-102F-3W-0-102_5May16-Spot 54	594	31389	1.1	13.2858	0.7	1.8273	2.4	0.1761	2.3	0.95	1045.5	21.8	1055.3	15.6	1075.7	14.5	1075.7	14.5
Gleason_354-U1451A-102F-3W-0-102_5May16-Spot 43	835	102882	1.9	13.2684	0.7	1.7244	2.5	0.1659	2.4	0.96	989.7	21.7	1017.7	15.8	1078.3	14.1	1078.3	14.1
Gleason_354-U1451A-102F-3W-0-102_5May16-Spot 194	279	1684671	2.3	13.2530	0.8	1.7667	2.9	0.1698	2.8	0.96	1011.1	25.9	1033.3	18.7	1080.7	15.8	1080.7	15.8
Gleason_354-U1451A-102F-3W-0-102_5May16-Spot 81	365	154325	3.5	13.2238	1.0	1.8678	2.5	0.1791	2.2	0.91	1062.3	22.0	1069.8	16.3	1085.1	20.6	1085.1	20.6
Gleason_354-U1451A-102F-3W-0-102_5May16-Spot 139	537	221698	1.7	13.1934	0.8	1.8861	2.2	0.1805	2.1	0.94	1069.6	20.6	1076.2	14.8	1089.7	15.5	1089.7	15.5
Gleason_354-U1451A-102F-3W-0-102_5May16-Spot 119	699	8995	1.7	13.1599	1.4	1.5456	2.1	0.1475	1.6	0.76	887.0	13.5	948.7	13.2	1094.8	27.9	1094.8	27.9
Gleason_354-U1451A-102F-3W-0-102_5May16-Spot 172	120	119278	1.2	13.1529	0.7	1.8791	2.6	0.1793	2.5	0.97	1062.9	24.5	1073.7	17.2	1095.8	13.3	1095.8	13.3
Gleason_354-U1451A-102F-3W-0-102_5May16-Spot 240	213	1503689	0.8	13.1444	0.7	1.9887	1.7	0.1896	1.5	0.90	1119.2	15.4	1111.7	11.2	1097.1	14.1	1097.1	14.1
Gleason_354-U1451A-102F-3W-0-102_5May16-Spot 116	443	255225	1.4	13.1419	0.6	1.9001	2.1	0.1811	2.0	0.96	1073.0	19.8	1081.1	13.8	1097.5	11.5	1097.5	11.5
Gleason_354-U1451A-102F-3W-0-102_5May16-Spot 235	585	513601	6.5	13.1394	0.7	1.9269	2.0	0.1836	1.9	0.94	1086.8	19.2	1090.5	13.6	1097.9	13.7	1097.9	13.7
Gleason_354-U1451A-102F-3W-0-102_5May16-Spot 303	569	126110	3.3	13.0948	0.7	1.9097	2.1	0.1814	2.0	0.95	1074.5	19.9	1084.5	14.2	1104.7	13.7	1104.7	13.7
Gleason_354-U1451A-102F-3W-0-102_5May16-Spot 173	148	30163	0.5	13.0783	0.8	2.0720	2.9	0.1965	2.8	0.96	1156.7	29.3	1139.6	19.6	1107.2	15.3	1107.2	15.3
Gleason_354-U1451A-102F-3W-0-102_5May16-Spot 251	769	153498	2.7	12.9832	0.6	1.9706	2.1	0.1856	2.0	0.95	1097.2	20.2	1105.5	14.1	1121.8	12.9	1121.8	12.9
Gleason_354-U1451A-102F-3W-0-102_5May16-Spot 305	314	1676624	1.5	12.8159	0.8	2.1288	2.5	0.1979	2.3	0.95	1163.9	25.0	1158.2	17.1	1147.6	15.0	1147.6	15.0
Gleason_354-U1451A-102F-3W-0-102_5May16-Spot 210	88	12282	1.3	12.8042	0.9	2.2079	3.0	0.2050	2.8	0.96	1202.3	30.9	1183.6	20.6	1149.4	17.4	1149.4	17.4
Gleason_354-U1451A-102F-3W-0-102_5May16-Spot 187	389	115066	0.5	12.7696	0.9	2.0290	2.8	0.1879	2.7	0.95	1110.1	27.4	1125.3	19.3	1154.8	18.4	1154.8	18.4
Gleason_354-U1451A-102F-3W-0-102_5May16-Spot 193	311	101947	1.0	12.7053	0.8	2.1926	2.6	0.2020	2.5	0.96	1186.3	27.1	1178.7	18.3	1164.8	15.2	1164.8	15.2
Gleason_354-U1451A-102F-3W-0-102_5May16-Spot 290	718	367958	1.0	12.6882	0.7	2.0988	2.3	0.1931	2.2	0.95	1138.4	22.9	1148.4	15.8	1167.4	14.0	1167.4	14.0
Gleason_354-U1451A-102F-3W-0-102_5May16-Spot 311	128	126306	0.8	12.6596	0.7	2.1401	3.0	0.1965	3.0	0.97	1156.5	31.3	1161.9	21.1	1171.9	13.8	1171.9	13.8
Gleason_354-U1451A-102F-3W-0-102_5May16-Spot 201	211	137162	2.5	12.5865	0.8	2.2032	2.6	0.2011	2.5	0.95	1181.4	26.6	1182.1	18.1	1183.4	15.6	1183.4	15.6
Gleason_354-U1451A-102F-3W-0-102_5May16-Spot 10	129	168898	1.8	12.4309	0.8	2.3755	3.1	0.2142	3.0	0.96	1251.0	34.0	1235.3	22.1	1207.9	16.0	1207.9	16.0
Gleason_354-U1451A-102F-3W-0-102_5May16-Spot 136	121	33026	1.0	12.3951	0.8	2.1819	2.6	0.1961	2.5	0.95	1154.6	26.5	1175.3	18.3	1213.6	16.0	1213.6	16.0
Gleason_354-U1451A-102F-3W-0-102_5May16-Spot 147	444	112984	5.1	12.3265	0.6	2.2858	2.7	0.2044	2.6	0.97	1198.7	28.3	1207.9	18.8	1224.5	12.5	1224.5	12.5
Gleason_354-U1451A-102F-3W-0-102_5May16-Spot 125	350	111116	1.2	12.2758	0.7	2.3781	1.7	0.2117	1.6	0.91	1238.0	17.8	1236.0	12.4	1232.6	13.8	1232.6	13.8
Gleason_354-U1451A-102F-3W-0-102_5May16-Spot 76	251	449979	1.2	12.1586	0.6	2.3947	2.5	0.2112	2.5	0.97	1235.1	27.7	1241.0	18.3	1251.4	12.5	1251.4	12.5
Gleason_354-U1451A-102F-3W-0-102_5May16-Spot 110	486	128486	2.7	11.7683	0.5	2.6849	2.3	0.2292	2.3	0.97	1330.1	27.4	1324.3	17.3	1314.9	10.3	1314.9	10.3
Gleason_354-U1451A-102F-3W-0-102_5May16-Spot 14	322	53607	1.8	11.7482	0.6	2.6642	2.0	0.2270	1.9	0.95	1318.8	22.7	1318.6	14.9	1318.2	12.5	1318.2	12.5
Gleason_354-U1451A-102F-3W-0-102_5May16-Spot 272	189	54657	1.0	11.6470	0.7	2.8497	2.3	0.2407	2.2	0.95	1390.5	27.7	1368.7	17.5	1335.0	14.0	1335.0	14.0
Gleason_354-U1451A-102F-3W-0-102_5May16-Spot 204	219	30500	1.9	11.6110	0.8	2.7672	2.5	0.2330	2.4	0.95	1350.4	28.8	1346.7	18.5	1341.0	14.8	1341.0	14.8
Gleason_354-U1451A-102F-3W-0-102_5May16-Spot 271	244	273812	3.4	11.5774	0.5	2.8705	2.2	0.2410	2.2	0.97	1392.0	27.3	1374.2	16.9	1346.6	10.5	1346.6	10.5
Gleason_354-U1451A-102F-3W-0-102_5May16-Spot 143	594	95183	1.7	11.4403	0.6	2.6531	2.4	0.2201	2.3	0.96	1282.6	26.4	1315.5	17.3	1369.5	12.0	1369.5	12.0
Gleason_354-U1451A-102F-3W-0-102_5May16-Spot 33	480	259228	6.9	11.4170	0.6	2.7907	2.3	0.2311	2.2	0.97	1340.2	27.0	1353.0	17.2	1373.5	11.4	1373.5	11.4
Gleason_354-U1451A-102F-3W-0-102_5May16-Spot 146	361	19146	1.2	10.8826	0.8	2.6686	2.4	0.2106	2.3	0.94	1232.2	25.2	1319.8	17.6	1465.1	15.1	1465.1	15.1
Gleason_354-U1451A-102F-3W-0-102_5May16-Spot 166	1342	1455858	16.4	10.6797	1.1	2.6854	2.3	0.2080	2.0	0.89	1218.2	22.6	1324.4	17.0	1500.8	20.1	1500.8	20.1
Gleason_354-U1451A-102F-3W-0-102_5May16-Spot 152	1682	########	10.2	10.6122	0.9	3.3517	2.4	0.2580	2.3	0.93	1479.4	30.2	1493.2	19.2	1512.7	16.9	1512.7	16.9
Gleason_354-U1451A-102F-3W-0-102_5May16-Spot 39	156	83327	1.4	10.5665	0.8	3.5945	3.0	0.2755	2.9	0.96	1568.5	40.8	1548.3	24.2	1520.9	15.7	1520.9	15.7
Gleason_354-U1451A-102F-3W-0-102_5May16-Spot 154	238	43612	5.4	10.5658	0.6	3.3885	1.9	0.2597	1.8	0.95	1488.1	24.4	1501.7	15.1	1521.0	11.1	1521.0	11.1

Gleason_354-U1451A-102F-3W-0-102_5May16-Spot 189	1820	1039107	7.0	10.4448	0.7	3.2962	2.2	0.2497	2.0	0.94	1436.9	26.4	1480.2	16.9	1542.7	13.7	1542.7	13.7
Gleason_354-U1451A-102F-3W-0-102_5May16-Spot 250	162	488070	1.2	10.3822	0.8	3.6299	3.1	0.2733	3.0	0.97	1557.6	41.8	1556.1	24.9	1554.0	15.2	1554.0	15.2
Gleason_354-U1451A-102F-3W-0-102_5May16-Spot 105	2911	352300	17.0	10.3686	0.5	3.0117	2.0	0.2265	1.9	0.97	1316.0	22.9	1410.6	15.2	1556.4	9.5	1556.4	9.5
Gleason_354-U1451A-102F-3W-0-102_5May16-Spot 184	229	100496	0.9	10.3603	0.6	3.6066	2.4	0.2710	2.3	0.97	1545.9	31.8	1551.0	19.0	1557.9	11.3	1557.9	11.3
Gleason_354-U1451A-102F-3W-0-102_5May16-Spot 239	340	270557	4.3	10.3530	1.2	3.2079	2.9	0.2409	2.6	0.91	1391.2	32.7	1459.1	22.2	1559.3	22.1	1559.3	22.1
Gleason_354-U1451A-102F-3W-0-102_5May16-Spot 156	390	104279	0.9	10.3252	0.7	3.5804	2.3	0.2681	2.2	0.96	1531.3	29.4	1545.2	17.9	1564.3	12.3	1564.3	12.3
Gleason_354-U1451A-102F-3W-0-102_5May16-Spot 41	198	94388	2.3	10.2939	0.6	3.7514	2.6	0.2801	2.6	0.97	1591.7	36.3	1582.4	21.2	1570.0	11.9	1570.0	11.9
Gleason_354-U1451A-102F-3W-0-102_5May16-Spot 135	121	37300	2.1	10.2343	0.7	3.7571	3.2	0.2789	3.1	0.98	1585.7	44.2	1583.6	25.9	1580.9	13.4	1580.9	13.4
Gleason_354-U1451A-102F-3W-0-102_5May16-Spot 288	498	174115	1.4	10.0835	0.7	3.8789	2.4	0.2837	2.3	0.95	1609.8	32.8	1609.3	19.5	1608.6	13.9	1608.6	13.9
Gleason_354-U1451A-102F-3W-0-102_5May16-Spot 91	162	94257	1.4	10.0669	0.6	3.9575	3.0	0.2889	3.0	0.98	1636.3	43.0	1625.5	24.7	1611.6	11.8	1611.6	11.8
Gleason_354-U1451A-102F-3W-0-102_5May16-Spot 283	227	456099	1.5	10.0566	0.6	3.9221	2.7	0.2861	2.6	0.98	1621.8	37.1	1618.2	21.4	1613.6	10.5	1613.6	10.5
Gleason_354-U1451A-102F-3W-0-102_5May16-Spot 308	306	196965	1.6	10.0248	0.7	3.8798	1.9	0.2821	1.8	0.94	1601.9	25.6	1609.5	15.6	1619.5	12.6	1619.5	12.6
Gleason_354-U1451A-102F-3W-0-102_5May16-Spot 63	1496	1632253	3.0	10.0078	0.7	3.9871	2.3	0.2894	2.2	0.96	1638.5	32.0	1631.6	18.8	1622.6	12.5	1622.6	12.5
Gleason_354-U1451A-102F-3W-0-102_5May16-Spot 228	169	92020	1.4	10.0050	0.8	4.0331	2.9	0.2927	2.8	0.96	1654.8	41.2	1640.9	23.9	1623.1	14.8	1623.1	14.8
Gleason_354-U1451A-102F-3W-0-102_5May16-Spot 227	417	202867	0.6	9.9770	0.6	3.8754	1.6	0.2804	1.5	0.93	1593.5	21.1	1608.6	13.0	1628.4	11.2	1628.4	11.2
Gleason_354-U1451A-102F-3W-0-102_5May16-Spot 38	555	91133612	0.5	9.9745	0.7	3.7660	2.2	0.2724	2.1	0.95	1553.2	29.5	1585.5	18.0	1628.8	12.7	1628.8	12.7
Gleason_354-U1451A-102F-3W-0-102_5May16-Spot 255	351	646053	1.5	9.8976	0.7	3.9363	2.4	0.2826	2.3	0.95	1604.3	33.2	1621.2	19.8	1643.2	13.6	1643.2	13.6
Gleason_354-U1451A-102F-3W-0-102_5May16-Spot 44	55	14294	0.6	9.7611	0.8	4.3964	3.9	0.3112	3.8	0.98	1746.8	57.8	1711.7	31.9	1668.9	14.6	1668.9	14.6
Gleason_354-U1451A-102F-3W-0-102_5May16-Spot 157	243	132593	1.5	9.6417	0.5	4.1567	2.1	0.2907	2.0	0.97	1644.9	29.1	1665.5	16.9	1691.6	9.2	1691.6	9.2
Gleason_354-U1451A-102F-3W-0-102_5May16-Spot 93	62	17905	0.5	9.5722	0.8	4.5572	3.2	0.3164	3.1	0.97	1772.0	48.2	1741.5	26.8	1705.0	15.5	1705.0	15.5
Gleason_354-U1451A-102F-3W-0-102_5May16-Spot 133	410	135773	1.6	9.5428	0.6	4.1579	2.8	0.2878	2.7	0.98	1630.4	39.2	1665.8	22.8	1710.6	10.5	1710.6	10.5
Gleason_354-U1451A-102F-3W-0-102_5May16-Spot 8	1484	810073	4.9	9.5114	0.9	4.3769	2.6	0.3019	2.4	0.94	1700.9	36.6	1708.0	21.5	1716.7	16.0	1716.7	16.0
Gleason_354-U1451A-102F-3W-0-102_5May16-Spot 234	935	377607	2.1	9.5030	0.9	4.1093	2.6	0.2832	2.4	0.94	1607.6	34.8	1656.2	21.3	1718.3	16.6	1718.3	16.6
Gleason_354-U1451A-102F-3W-0-102_5May16-Spot 145	864	532462	2.0	9.4808	0.7	4.1513	2.2	0.2854	2.1	0.94	1618.7	29.4	1664.5	17.9	1722.6	13.5	1722.6	13.5
Gleason_354-U1451A-102F-3W-0-102_5May16-Spot 101	326	146455	1.5	9.4092	0.6	4.5884	2.1	0.3131	2.1	0.96	1756.0	31.6	1747.2	17.8	1736.5	10.6	1736.5	10.6
Gleason_354-U1451A-102F-3W-0-102_5May16-Spot 177	307	66979	1.9	9.3925	0.6	4.6745	2.6	0.3184	2.5	0.97	1782.0	38.6	1762.7	21.4	1739.8	11.5	1739.8	11.5
Gleason_354-U1451A-102F-3W-0-102_5May16-Spot 274	338	494488	1.5	9.3593	0.8	4.4661	2.4	0.3032	2.3	0.95	1707.0	33.9	1724.7	19.8	1746.3	13.8	1746.3	13.8
Gleason_354-U1451A-102F-3W-0-102_5May16-Spot 65	783	353048	5.6	9.3073	0.7	4.2457	1.9	0.2866	1.8	0.93	1624.5	25.7	1682.9	15.9	1756.5	13.0	1756.5	13.0
Gleason_354-U1451A-102F-3W-0-102_5May16-Spot 42	60	24052	0.8	9.2101	0.9	4.8497	4.0	0.3239	3.9	0.98	1809.0	61.4	1793.6	33.6	1775.7	15.9	1775.7	15.9
Gleason_354-U1451A-102F-3W-0-102_5May16-Spot 253	937	59959	3.6	9.1840	1.2	4.2913	3.2	0.2858	2.9	0.92	1620.7	42.2	1691.7	26.3	1780.8	22.6	1780.8	22.6
Gleason_354-U1451A-102F-3W-0-102_5May16-Spot 276	272	85807	0.9	8.8781	0.7	5.1013	2.2	0.3285	2.1	0.95	1831.0	33.5	1836.3	18.7	1842.4	11.9	1842.4	11.9
Gleason_354-U1451A-102F-3W-0-102_5May16-Spot 225	555	109497	1.7	8.8525	0.6	4.9282	2.1	0.3164	2.0	0.95	1772.2	31.0	1807.1	17.7	1847.6	11.7	1847.6	11.7
Gleason_354-U1451A-102F-3W-0-102_5May16-Spot 256	391	271632	4.3	8.7969	0.9	5.2884	2.4	0.3374	2.2	0.93	1874.2	35.8	1867.0	20.3	1859.0	16.3	1859.0	16.3
Gleason_354-U1451A-102F-3W-0-102_5May16-Spot 192	389	126959	4.6	8.7919	0.5	5.3063	1.6	0.3384	1.5	0.94	1878.7	24.9	1869.9	13.8	1860.0	9.9	1860.0	9.9
Gleason_354-U1451A-102F-3W-0-102_5May16-Spot 281	275	288841	2.5	8.7423	0.6	5.2413	2.3	0.3323	2.2	0.96	1849.7	36.0	1859.4	19.8	1870.2	11.2	1870.2	11.2
Gleason_354-U1451A-102F-3W-0-102_5May16-Spot 4	515	22220	1.2	8.7354	0.9	5.0218	3.0	0.3182	2.9	0.95	1780.7	45.1	1823.0	25.7	1871.7	16.4	1871.7	16.4
Gleason_354-U1451A-102F-3W-0-102_5May16-Spot 270	277	724547	1.8	8.7211	0.5	5.3665	2.0	0.3394	1.9	0.97	1884.0	31.0	1879.5	16.8	1874.6	8.9	1874.6	8.9
Gleason_354-U1451A-102F-3W-0-102_5May16-Spot 70	382	1428197	2.3	8.7186	0.8	5.3162	2.7	0.3362	2.5	0.95	1868.2	41.0	1871.5	22.7	1875.1	14.2	1875.1	14.2
Gleason_354-U1451A-102F-3W-0-102_5May16-Spot 78	193	82827	1.1	8.6952	0.8	5.1865	2.4	0.3271	2.2	0.95	1824.2	35.7	1850.4	20.2	1880.0	13.5	1880.0	13.5
Gleason_354-U1451A-102F-3W-0-102_5May16-Spot 120	108	1296352	1.1	8.6577	0.6	5.5464	2.4	0.3483	2.3	0.96	1926.3	38.3	1907.8	20.6	1887.8	11.6	1887.8	11.6
Gleason_354-U1451A-102F-3W-0-102_5May16-Spot 97	1327	476291	1.2	8.6214	0.7	5.0162	2.2	0.3137	2.1	0.95	1758.7	31.7	1822.1	18.4	1895.3	12.5	1895.3	12.5
Gleason_354-U1451A-102F-3W-0-102_5May16-Spot 9	450	750418	5.2	8.4936	0.7	5.4301	2.4	0.3345	2.3	0.96	1860.2	36.8	1889.6	20.4	1922.1	12.6	1922.1	12.6
Gleason_354-U1451A-102F-3W-0-102_5May16-Spot 98	556	349407	2.7	8.4235	0.8	4.7126	2.2	0.2879	2.0	0.92	1631.1	29.4	1769.5	18.5	1937.0	15.0	1937.0	15.0
Gleason_354-U1451A-102F-3W-0-102_5May16-Spot 1	5064	7151	4.7	8.3711	1.3	4.5086	3.5	0.2737	3.3	0.93	1559.7	45.5	1732.6	29.5	1948.1	24.0	1948.1	24.0
Gleason_354-U1451A-102F-3W-0-102_5May16-Spot 30	200	83440	1.4	7.9050	0.7	6.2584	2.6	0.3588	2.5	0.96	1976.5	42.2	2012.6	22.5	2049.9	12.0	2049.9	12.0
Gleason_354-U1451A-102F-3W-0-102_5May16-Spot 90	130	683130	0.6	7.4529	0.7	7.3629	2.4	0.3980	2.3	0.95	2159.8	42.0	2156.5	21.5	2153.3	13.1	2153.3	13.1
Gleason_354-U1451A-102F-3W-0-102_5May16-Spot 83	619	252704	2.0	7.3125	0.7	7.4710	2.1	0.3962	2.0	0.95	2151.6	37.2	2169.5	19.2	2186.5	11.9	2186.5	11.9
Gleason_354-U1451A-102F-3W-0-102_5May16-Spot 84	568	514258	0.5	7.1500	0.7	7.1195	2.8	0.3692	2.7	0.97	2025.6	47.5	2126.5	25.1	2225.4	11.8	2225.4	11.8
Gleason_354-U1451A-102F-3W-0-102_5May16-Spot 221	633	127649	1.9	6.8956	0.6	8.1256	2.0	0.4064	1.9	0.94	2198.3	34.5	2245.1	17.7	2288.0	11.1	2288.0	11.1
Gleason_354-U1451A-102F-3W-0-102_5May16-Spot 47	76	308690	1.4	6.7777	0.8	7.2506	4.0	0.3564	4.0	0.98	1965.1	66.9	2142.7	36.0	2317.6	14.0	2317.6	14.0
Gleason_354-U1451A-102F-3W-0-102_5May16-Spot 99	950	96874	1.8	6.7076	2.4	7.0369	7.1	0.3423	6.7	0.94	1897.9	110.5	2116.1	63.6	2335.4	41.7	2335.4	41.7
Gleason_354-U1451A-102F-3W-0-102_5May16-Spot 279	171	83711	1.4	6.6965	0.7	8.4116	2.6	0.4085	2.5	0.96	2208.2	47.2	2276.4	23.8	2338.3	12.0	2338.3	12.0
Gleason_354-U1451A-102F-3W-0-102_5May16-Spot 197	643	775074	2.0	6.6875	0.7	7.6192	2.6	0.3696	2.5	0.96	2027.3	43.6	2187.1	23.4	2340.6	12.1	2340.6	12.1
Gleason_354-U1451A-102F-3W-0-102_5May16-Spot 241	150	53052	2.0	6.6366	0.6	7.0874	2.9	0.3411	2.8	0.98	1892.1	46.4	2122.5	25.8	2353.6	10.4	2353.6	10.4
Gleason_354-U1451A-102F-3W-0-102_5May16-Spot 252	121	238554	0.9	6.6321	0.7	9.3378	3.3	0.4492	3.2	0.98	2391.5	64.6	2371.7	30.3	2354.8	11.6	2354.8	11.6
Gleason_354-U1451A-102F-3W-0-102_5May16-Spot 269	491	1700706	5.4	6.6314	0.7	8.2808	2.4	0.3983	2.3	0.96	2161.0	43.0	2262.2	22.1	2355.0	11.9	2355.0	11.9
Gleason_354-U1451A-102F-3W-0-102_5May16-Spot 37	1068	365887	1.4	6.4040	0.5	9.5720	2.1	0.4446	2.1	0.97	2371.1	41.0	2394.5	19.5	2414.4	8.5	2414.4	8.5
Gleason_354-U1451A-102F-3W-0-102_5May16-Spot 165	366	311880	2.7	6.4009	0.6	8.3332	1.7	0.3869	1.6	0.95	2108.3	29.6	2267.9	15.8	2415.2	9.6	2415.2	9.6
Gleason_354-U1451A-102F-3W-0-102_5May16-Spot 186	1052	984483	4.7	6.3775	0.5	9.3339	1.7	0.4317	1.6	0.95	2313.5	31.3	2371.4	15.5	2421.4	8.8	2421.4	8.8
Gleason_354-U1451A-102F-3W-0-102_5May16-Spot 246	912	210198	1.5	6.3704	0.7	8.7172	2.4	0.4028	2.3	0.96	2181.7	41.8	2308.9	21.6	2423.3	11.9	2423.3	11.9
Gleason_354-U1451A-102F-3W-0-102_5May16-Spot 168	122	985633	1.1	6.3104	0.8	8.7940	3.1	0.4025	2.9	0.96	2180.4	54.4	2316.9	27.8	2439.4	13.8	2439.4	13.8
Gleason_354-U1451A-102F-3W-0-102_5May16-Spot 263	305	173897	1.3	6.2748	0.6	8.8022	2.3	0.4006	2.2	0.97	2171.7	40.4	2317.7	20.7	2449.0	9.9	2449.0	9.9
Gleason_354-U1451A-102F-3W-0-102_5May16-Spot 7	1106	836867	8.3	6.2665	0.7	10.2748	2.2	0.4670	2.1	0.95	2470.3	42.1	2459.8	20.0	2451.2	11.7	2451.2	11.7
Gleason_354-U1451A-102F-3W-0-102_5May16-Spot 243	2122	2966927	5.5	6.2553	0.7	9.9995	2.1	0.4536	2.0	0.94	2411.5	40.2	2434.7	19.6	2454.2	12.4	2454.2	12.4
Gleason_354-U1451A-102F-3W-0-102_5May16-Spot 295	138	720002	1.0	6.1647	0.8	10.9111	2.8	0.4878	2.7	0.96	2561.3	56.8	2515.6	26.1	2478.9	13.8	2478.9	13.8
Gleason_354-U1451A-102F-3W-0-102_5May16-Spot 238	206	162270	0.6	6.1373	0.8	10.6385	2.3	0.4735	2.1	0.94	2499.1	44.4	2492.1	21.1	2486.4	13.0	2486.4	13.0
Gleason_354-U1451A-102F-3W-0-102_5May16-Spot 306	122	626610	1.0	6.1334	0.8	9.5996	3.1	0.4270	2.9	0.96	2292.3	56.8	2397.1	28.2	2487.4	14.3	2487.4	14.3
Gleason_354-U1451A-102F-3W-0-102_5May16-Spot 174	392	140876	1.9	6.1218	0.8	10.0129	2.3	0.4446	2.1	0.94	2371.1	41.9	2436.0	20.9	2490.6	13.4	2490.6	13.4
Gleason_354-U1451A-102F-3W-0-102_5May16-Spot 300	277	81573	1.1	6.0705	0.7	10.6398	2.6	0.4684	2.5	0.97	2476.7	51.9	2492.2	24.3	2504.8	11.5	2504.8	11.5
Gleason_354-U1451A-102F-3W-0-102_5May16-Spot 87	263	79172	1.2	6.0626	0.9	10.5625	2.9	0.4644	2.8	0.95	2459.1	57.2	2485.4	27.3	2507.0	15.1	2507.0	15.1
Gleason_354-U1451A-102F-3W-0-102_5May16-Spot 62	50	198056	0.9	6.0167	0.8	10.9794	3.4	0.4791	3.3	0.97	2523.4	68.7	2521.4	31.6	2519.8	13.7	2519.8	13.7
Gleason_354-U1451A-102F-3W-0-102_5May16-Spot 217	250	292123	0.9	5.9931	0.7	10.5922	2.1	0.4604	2.0	0.95	2441.3	40.6	2488.0	19.6	2526.4	11.5	2526.4	11.5
Gleason_354-U1451A-102F-3W-0-102_5May16-Spot 298	428	222366	1.2	5.8364	0.6	11.1351	2.2	0.4713	2.1	0.96	2489.5	42.8	2534.5	20.2	2570.7	10.4	2570.7	10.4
Gleason_354-U1451A-102F-3W-0-102_5May16-Spot 137	225	186413	1.4	5.6697	0.7	12.2204	2.7	0.5025	2.7	0.97	2624.6	57.2	2621.5	25.6	2619.1	10.8	2619.1	10.8
Gleason_354-U1451A-102F-3W-0-102_5May16-Spot 73	488	278581	5.5	5.6048	0.7	10.9756	2.3	0.4462	2.2	0.95	2378.1	42.8	2521.1	21.0	2638.2	11.6	2638.2	11.6
Gleason_354-U1451A-102F-3W-0-102_5May16-Spot 17	247	141365	1.6	5.3184	0.7	13.3190	3.1	0.5138	3.0	0.97	2672.6	65.5	2702.5	29.1	2725.0	11.7	2725.0	11.7
Gleason_354-U1451A-102F-3W-0-102_5May16-Spot 259	133	34966	1.3	5.2583	0.6	13.5628	2.6	0.5172	2.5	0.97	2687.5	55.0	2719.7	24.4	2743.7	10.1	2743.7	10.1
Gleason_354-U1451A-102F-3W-0-102_5May16-Spot 180	383	140293	1.1	5.1954	0.8	11.6164	3.2	0.4377	3.1	0.97	2340.4	61.8	2574.0	30.3	2763.4	12.9	2763.4	12.9
Gleason_354-U1451A-102F-3W-0-102_5May16-Spot 183	30	38569	1.2	5.1028	0.9	15.3513	4.5	0.5681	4.4	0.98	2900.2	102.6	2837.3	42.8	2792.9	14.8	2792.9	14.8
Gleason_354-U1451A-102F-3W-0-102_5May16-Spot 260	57	311381	1.3	5.1007	0.8	15.5194	3.4	0.5741	3.2	0.97	2924.7	76.4	2847.7	32.0	2793.6	13.8	2793.6	13.8
Gleason_354-U1451A-102F-3W-0-102_5May16-Spot 49	75	54975	1.4	4.9597	0.6	15.3612	3.9	0.5526	3.8	0.99	2835.8	87.5	2837.9	36.7	2839.4	9.0	2839.4	9.0
Gleason_354-U1451A-102F-3W-0-102_5May16-Spot 280	116	73102	1.0	4.7005	0.8	17.6401	2.8	0.6014	2.7	0.96	3035.4	64.2	2970.3	26.6	2926.6	12.6	2926.6	12.6
Gleason_354-U1451A-102F-3W-0-102_5May16-Spot 301	510	246797	1.1	4.6556	0.6	16.7358	2.4	0.5651	2.3	0.96	2887.6	53.6	2919.8	22.9	2942.1	10.3	2942.1	10.3
Gleason_354-U1451A-102F-3W-0-102_5May16-Spot 208	192	149200	2.0	4.3507	0.6	18.4640	2.6	0.5826	2.5	0.97	2959.4	60.4	3014.2	25.2	3051.0	9.9	3051.0	9.9
Gleason_354-U1451A-102F-3W-0-102_5May16-Spot 297	129	8903439	1.3	4.1690	0.8	17.9530	2.9	0.5428	2.8	0.96	2795.3	63.5	2987.2	27.9	3119.1	12.3	3119.1	12.3
Gleason_354-U1451A-102F-3W-0-102_5May16-Spot 286	215	344101	1.4	3.8748	0.8	22.2707	3.0	0.6259	2.9	0.97	3133.2	72.5	3195.6	29.3	3235.0	11.9	3235.0	11.9

Table _____. U-Pb geochronologic analyses.																		
#16 U1451A_37F																		
Late Miocene 6.3 Ma						Isotope ratios						Apparent ages (Ma)						
Analysis	U	206Pb	U/Th	206Pb*	±	207Pb*	±	206Pb*	±	error	206Pb*	±	207Pb*	±	206Pb*	±	Best age	±
	(ppm)	204Pb		207Pb*	(%)	235U*	(%)	238U	(%)	corr.	238U*	(Ma)	235U	(Ma)	207Pb*	(Ma)	(Ma)	(Ma)
354U1451 A37F-Spot 60	569	3002	1.2	22.6214	2.3	0.0178	2.7	0.0029	1.4	0.53	18.8	0.3	17.9	0.5	NA	NA	18.8	0.3
354U1451 A37F-Spot 35	686	6841	1.0	21.9761	1.5	0.0258	2.0	0.0041	1.3	0.66	26.5	0.4	25.9	0.5	NA	NA	26.5	0.4
354U1451 A37F-Spot 4	2647	189756	0.9	19.1273	1.4	0.0329	2.6	0.0046	2.2	0.84	29.3	0.6	32.8	0.8	296.7	32.5	29.3	0.6
354U1451 A37F-Spot 221	169	25640	133.1	22.4648	3.0	0.0282	3.4	0.0046	1.6	0.48	29.5	0.5	28.2	0.9	NA	NA	29.5	0.5
354U1451 A37F-Spot 205	108	1044	1.1	25.3587	6.4	0.0253	6.6	0.0047	1.4	0.21	30.0	0.4	25.4	1.6	NA	NA	30.0	0.4
354U1451 A37F-Spot 174	612	73319	1.6	21.2978	1.6	0.0381	1.9	0.0059	1.2	0.60	37.8	0.4	37.9	0.7	45.8	37.1	37.8	0.4
354U1451 A37F-Spot 120	198	7227	2.3	21.6869	2.3	0.0406	2.8	0.0064	1.6	0.55	41.0	0.6	40.4	1.1	2.4	56.3	41.0	0.6
354U1451 A37F-Spot 200	591	3053	2.0	19.2971	4.1	0.0470	4.3	0.0066	1.1	0.26	42.3	0.5	46.7	2.0	276.6	94.7	42.3	0.5
354U1451 A37F-Spot 238	471	7219	0.4	22.3397	1.5	0.0410	1.9	0.0066	1.2	0.61	42.7	0.5	40.8	0.8	NA	NA	42.7	0.5
354U1451 A37F-Spot 191	662	4696	2.1	13.9438	6.2	0.0670	6.4	0.0068	1.7	0.26	43.6	0.7	65.9	4.1	977.0	126.2	43.6	0.7
354U1451 A37F-Spot 194	912	34368	0.9	20.9746	1.5	0.0486	2.2	0.0074	1.6	0.74	47.5	0.8	48.2	1.0	82.2	35.3	47.5	0.8
354U1451 A37F-Spot 91	355	3553	2.6	22.7395	1.7	0.0492	2.3	0.0081	1.6	0.68	52.1	0.8	48.7	1.1	NA	NA	52.1	0.8
354U1451 A37F-Spot 147	174	2065	1.5	12.0265	10.7	0.0940	11.0	0.0082	2.2	0.20	52.7	1.1	91.2	9.6	1271.9	210.0	52.7	1.1
354U1451 A37F-Spot 215	2994	64328	4.3	20.9277	0.8	0.0554	1.7	0.0084	1.5	0.88	54.0	0.8	54.7	0.9	87.6	19.1	54.0	0.8
354U1451 A37F-Spot 111	144	8344	1.7	21.7566	2.1	0.0543	2.6	0.0086	1.5	0.58	55.0	0.8	53.7	1.3	NA	NA	55.0	0.8
354U1451 A37F-Spot 224	1837	44975	28.0	20.9964	0.9	0.0578	1.8	0.0088	1.5	0.87	56.5	0.9	57.0	1.0	79.7	20.9	56.5	0.9
354U1451 A37F-Spot 172	878	129047	1.8	20.5900	1.0	0.0629	1.5	0.0094	1.2	0.76	60.3	0.7	61.9	0.9	126.0	23.0	60.3	0.7
354U1451 A37F-Spot 176	85	8446	2.9	21.5608	2.9	0.0841	3.3	0.0132	1.6	0.48	84.3	1.3	82.0	2.6	16.4	69.6	84.3	1.3
354U1451 A37F-Spot 161	709	94141	33.8	14.3323	1.1	0.1333	1.9	0.0139	1.5	0.80	88.8	1.3	127.1	2.3	920.8	23.5	88.8	1.3
354U1451 A37F-Spot 151	172	7982	1.9	21.5254	2.0	0.0900	2.4	0.0141	1.4	0.57	90.0	1.2	87.5	2.0	20.4	46.9	90.0	1.2
354U1451 A37F-Spot 287	1792	145592	7.2	20.5116	0.8	0.0961	1.5	0.0143	1.3	0.86	91.6	1.2	93.2	1.3	134.9	18.1	91.6	1.2
354U1451 A37F-Spot 84	918	20227	1.1	20.4033	1.2	0.1018	1.7	0.0151	1.1	0.67	96.4	1.1	98.4	1.6	147.4	29.1	96.4	1.1
354U1451 A37F-Spot 165	250	10189	1.6	21.0440	1.2	0.0998	1.6	0.0152	0.9	0.61	97.5	0.9	96.6	1.4	74.4	29.4	97.5	0.9
354U1451 A37F-Spot 170	227	10035	1.1	20.8585	1.6	0.1445	2.3	0.0219	1.6	0.72	139.4	2.3	137.0	2.9	95.4	37.7	139.4	2.3
354U1451 A37F-Spot 223	719	56021	10.1	17.9971	1.1	0.1875	3.5	0.0245	3.3	0.95	156.0	5.1	174.5	5.6	434.0	24.3	156.0	5.1
354U1451 A37F-Spot 50	1205	104912	2.8	17.6870	0.7	0.3103	2.8	0.0398	2.7	0.97	251.7	6.6	274.4	6.6	472.6	15.7	251.7	6.6
354U1451 A37F-Spot 235	2024	40479	11.2	16.3083	0.7	0.3716	1.4	0.0440	1.2	0.86	277.4	3.3	320.8	3.9	649.5	15.8	277.4	3.3
354U1451 A37F-Spot 310	208	161000	4.6	18.9374	0.8	0.3742	1.4	0.0514	1.1	0.80	323.2	3.5	322.8	3.9	319.5	19.1	323.2	3.5
354U1451 A37F-Spot 44	627	123341	4.0	17.5874	0.8	0.4268	2.0	0.0545	1.8	0.91	341.9	6.0	360.9	6.1	485.1	18.2	341.9	6.0
354U1451 A37F-Spot 241	1160	876484	9.7	17.5087	0.6	0.4802	2.1	0.0610	1.9	0.95	381.7	7.2	398.2	6.8	495.0	14.3	381.7	7.2
354U1451 A37F-Spot 71	1076	145645	9.2	17.3221	0.9	0.5004	2.0	0.0629	1.8	0.90	393.2	7.0	412.0	6.9	518.5	19.9	393.2	7.0
354U1451 A37F-Spot 167	83	47809	1.2	16.0841	1.1	0.5418	2.0	0.0632	1.6	0.84	395.2	6.3	439.6	7.0	679.1	22.9	395.2	6.3
354U1451 A37F-Spot 109	1271	132077	4.2	17.6058	0.7	0.5097	2.0	0.0651	1.9	0.93	406.6	7.4	418.2	6.9	482.8	15.8	406.6	7.4
354U1451 A37F-Spot 305	1762	659254	18.4	17.3922	0.8	0.5709	1.5	0.0720	1.2	0.84	448.5	5.3	458.6	5.4	509.7	17.7	448.5	5.3
354U1451 A37F-Spot 9	1421	92956	11.2	17.6946	0.9	0.5786	1.8	0.0743	1.5	0.85	462.0	6.9	463.6	6.7	471.7	20.9	462.0	6.9
354U1451 A37F-Spot 284	508	120715	10.8	17.4216	1.0	0.5962	1.9	0.0754	1.5	0.83	468.4	7.0	474.8	7.1	506.0	22.9	468.4	7.0
354U1451 A37F-Spot 107	3068	357575	4.4	17.2745	0.8	0.6019	1.6	0.0754	1.4	0.88	468.9	6.4	478.5	6.2	524.6	16.8	468.9	6.4
354U1451 A37F-Spot 78	756	448327	3.7	17.1282	0.8	0.6114	1.5	0.0760	1.3	0.84	472.1	5.8	484.4	5.8	543.2	18.0	472.1	5.8
354U1451 A37F-Spot 122	1509	172291	1.2	17.5385	0.9	0.6038	1.5	0.0768	1.2	0.81	477.2	5.6	479.7	5.7	491.3	19.3	477.2	5.6
354U1451 A37F-Spot 2	145	440465	1.7	17.7059	1.0	0.6017	1.7	0.0773	1.4	0.80	480.0	6.3	478.3	6.5	470.3	22.4	480.0	6.3
354U1451 A37F-Spot 138	713	209721	5.7	17.3208	0.8	0.6159	1.4	0.0774	1.2	0.83	480.6	5.5	487.3	5.5	518.7	17.2	480.6	5.5
354U1451 A37F-Spot 302	807	656011	4.8	17.2585	0.8	0.6197	1.4	0.0776	1.2	0.82	481.8	5.4	489.6	5.6	526.7	18.1	481.8	5.4
354U1451 A37F-Spot 258	33	11760	0.5	17.6882	1.5	0.6048	1.8	0.0776	0.9	0.50	481.9	4.2	480.3	6.8	472.5	34.2	481.9	4.2
354U1451 A37F-Spot 307	534	383856	4.2	17.6041	1.0	0.6089	1.7	0.0778	1.3	0.79	482.9	6.1	482.9	6.4	483.0	22.8	482.9	6.1
354U1451 A37F-Spot 10	227	3479849	2.3	17.0806	0.9	0.6294	1.5	0.0780	1.3	0.82	484.2	5.9	495.7	6.0	549.3	19.2	484.2	5.9
354U1451 A37F-Spot 262	417	61937	1.7	17.5952	0.7	0.6117	1.4	0.0781	1.2	0.85	484.8	5.6	484.7	5.4	484.1	16.2	484.8	5.6
354U1451 A37F-Spot 239	209	27858	3.1	17.4995	0.7	0.6183	1.3	0.0785	1.2	0.86	487.2	5.4	488.8	5.2	496.1	15.0	487.2	5.4
354U1451 A37F-Spot 240	632	74700	1.1	17.2841	0.8	0.6316	1.3	0.0792	1.0	0.79	491.4	4.8	497.1	5.1	523.4	17.4	491.4	4.8
354U1451 A37F-Spot 144	1079	301916	3.0	17.5415	0.8	0.6237	1.4	0.0794	1.1	0.81	492.4	5.4	492.1	5.5	490.8	18.1	492.4	5.4
354U1451 A37F-Spot 24	88	15993	1.7	17.6675	1.0	0.6201	1.7	0.0795	1.3	0.78	493.1	6.2	489.9	6.5	475.1	22.8	493.1	6.2
354U1451 A37F-Spot 45	486	63330	1.7	17.0824	0.9	0.6414	1.5	0.0795	1.2	0.82	493.1	5.9	503.2	6.0	549.0	18.8	493.1	5.9
354U1451 A37F-Spot 152	1059	163644	10.0	17.3025	0.7	0.6334	1.4	0.0795	1.2	0.86	493.2	5.7	498.2	5.5	521.0	15.5	493.2	5.7
354U1451 A37F-Spot 270	1280	2606479	11.2	17.4413	0.7	0.6319	1.7	0.0800	1.5	0.90	495.9	7.3	497.3	6.6	503.5	15.8	495.9	7.3
354U1451 A37F-Spot 121	1354	152090	1.6	17.4675	0.8	0.6311	1.3	0.0800	1.1	0.82	496.1	5.3	496.8	5.3	500.2	16.9	496.1	5.3
354U1451 A37F-Spot 32	540	64286	1.7	17.4787	0.8	0.6359	1.5	0.0806	1.2	0.85	500.0	6.0	499.8	5.8	498.8	17.3	500.0	6.0
354U1451 A37F-Spot 59	287	77670	1.3	17.1266	0.6	0.6505	0.9	0.0808	0.7	0.73	501.1	3.2	508.8	3.6	543.5	13.2	501.1	3.2
354U1451 A37F-Spot 51	73	33739	1.4	17.4886	0.8	0.6380	1.4	0.0810	1.2	0.85	501.9	5.8	501.1	5.6	497.5	16.7	501.9	5.8
354U1451 A37F-Spot 14	220	194283	2.6	17.4933	0.9	0.6383	1.5	0.0810	1.2	0.81	502.2	6.0	501.3	6.1	496.9	20.0	502.2	6.0
354U1451 A37F-Spot 8	429	147303	3.1	17.6111	0.8	0.6357	1.8	0.0812	1.6	0.90	503.4	7.8	499.6	7.1	482.2	17.1	503.4	7.8
354U1451 A37F-Spot 127	181	789465	1.0	16.9759	1.1	0.6658	1.6	0.0820	1.2	0.74	508.1	5.9	518.1	6.6	562.7	23.9	508.1	5.9
354U1451 A37F-Spot 245	348	79082	7.2	17.3497	0.9	0.6524	1.6	0.0821	1.3	0.84	508.8	6.6	510.0	6.4	515.0	19.1	508.8	6.6
354U1451 A37F-Spot 102	477	120984	1.8	17.3828	0.8	0.6554	2.3	0.0827	2.2	0.93	512.0	10.8	511.8	9.4	510.9	18.4	512.0	10.8
354U1451 A37F-Spot 108	462	547160	5.1	17.0658	0.8	0.6679	1.5	0.0827	1.3	0.84	512.3	6.2	519.5	6.1	551.2	17.6	512.3	6.2
354U1451 A37F-Spot 139	207	45258	1.4	17.3200	0.8	0.6590	1.2	0.0828	0.9	0.77	512.9	4.5	514.0	4.7	518.8	16.5	512.9	4.5
354U1451 A37F-Spot 300	375	1297707	4.4	16.8921	0.6	0.6772	1.5	0.0830	1.4	0.92	514.0	7.0	525.1	6.3	573.5	13.5	514.0	7.0
354U1451 A37F-Spot 86	258	81211	1.5	17.3543	0.7	0.6599	1.2	0.0831	1.0	0.80	514.6	4.9	514.6	5.0	514.5	16.2	514.6	4.9
354U1451 A37F-Spot 89	193	36434	1.4	16.8909	1.1	0.6785	1.7	0.0832	1.4	0.79	514.9	6.9	525.9	7.2	573.6	23.1	514.9	6.9
354U1451 A37F-Spot 23	111	15923	0.9	17.3203	1.0	0.6761	1.4	0.0850	0.9	0.67	525.7	4.7	524.4	5.6	518.8	22.3	525.7	4.7
354U1451 A37F-Spot 248	98	47955	1.0	17.0330	1.0	0.6928	1.7	0.0856	1.4	0.80	529.6	6.9	534.5	7.1	555.4	22.5	529.6	6.9
354U1451 A37F-Spot 183	287	3753156	0.9	16.9307	0.6	0.7047	1.3	0.0866	1.1	0.87	535.2	5.7	541.6	5.4	568.5	13.5	535.2	5.7
354U1451 A37F-Spot 312	313	796972	2.3	17.0738	0.9	0.7046	1.7	0.0873	1.4	0.83	539.5	7.3	541.5	7.1	550.2	20.4	539.5	7.3
354U1451 A37F-Spot 214	324	292851	1.2	16.8436	0.8	0.7395	1.5	0.0904	1.3	0.86	557.7	7.1	562.1	6.6	579.7	17.0	557.7	7.1
354U1451 A37F-Spot 145	404	753951	9.8	16.4162	1.4	0.7601	3.0	0.0905	2.6	0.88	558.7	14.0	574.1	13.0	635.3	29.7	558.7	14.0
354U1451 A37F-Spot 196	242	77665	5.9	17.0345	0.8	0.7391	1.3	0.0914	1.1	0.81	563.5	5.8	561.9	5.8	555.2	17.2	563.5	5.8
354U1451 A37F-Spot 157	293	56945	2.3	16.8660	0.7	0.7672	1.5	0.0939	1.3	0.88	578.5	7.2	578.2	6.6	576.9	15.4	578.5	7.2
354U1451 A37F-Spot 177	1042	1063332	10.2	16.2182	1.0	0.8381	1.7	0.0986	1.4	0.83	606.4	8.3	618.1	7.9	661.4	20.4	606.4	8.3
354U1451 A37F-Spot 306	495	188451	10.3	16.0896	0.7	0.8477	1.6	0.0990	1.4	0.90	608.3	8.4	623.4	7.4	678.4	14.8	608.3	8.4
354U1451 A37F-Spot 259	193	88750	5.4	15.8507	1.1	0.8952	1.7	0.1030	1.3	0.76	631.7	7.9	649.1	8.2	710.3	23.6	631.7	7.9
354U1451 A37F-Spot 201	466	343539	3.6	16.2440	0.8	0.8857	1.5	0.1044	1.3	0.85	640.1	7.8	644.0	7.2	658.0	17.1	640.1	7.8
354U1451 A37F-Spot 25	2061	3173111	11.7	15.3994	0.8	0.9615	2.5	0.1074	2.3	0.95	657.8	14.6	684.1	12.2	771.4	16.6	657.8	14.6
354U1451 A37F-Spot 308	405	63051	2.3	15.5425	0.8	0.9576	1.3	0.1080	1.1	0.82	661.1	6.9	682.1	6.7	751.9	16.2	661.1	6.9
354U1451 A37F-Spot 28	587	109317	5.0	15.3865	0.8	0.9804	2.2	0.1095	2.0	0.93	669.6	12.9	693.8	11.0	773.1	16.7	669.6	12.9
354U1451 A37F-Spot 280	92	76726	0.9	16.2225	0.9	0.9395	1.6	0.1106	1.3	0.83	676.2	8.5	672.6	7.9	660.8	18.9	676.2	8.5
354U1451 A37F-Spot 211	377	180362	3.4	15.0011	0.7	1.0327	1.6	0.1124	1.4	0.88	686.7	9.1	720.3	8.2	826.3	15.6	686.7	9.1
354U1451 A37F-Spot 173	252	29428	5.4	15.5018	0.9	1.0085	1.7	0.1134	1.5	0.86	692.7	9.7	708.1	8.7	757.4	18.2	692.7	9.7
354U1451 A37F-Spot 22	237	2916277	1.7	15.7610	0.8	1.0021	1.5	0.1146	1.3	0.85	699.4	8.7	704.9	7.9	722.3	17.3	699.4	8.7
354U1451 A37F-Spot 304	285	103744	8.3	16.1394	1.5	0.9825	2.7	0.1151	2.2	0.82	702.1	14.8	694.9	13.6	671.8	32.8	702.1	14.8
354U1451 A37F-Spot 166	327	374588	1.7	15.7259	0.9	1.0101	1.7	0.1153	1.5	0.85	703.2	9.7	708.9	8.7	727.1	18.8	703.2	9.7
354U1451 A37F-Spot 153	182	57154	2.1	15.6395	0.8	1.0331	1.5	0.1172	1.3	0.85	714.6	8.6	720.5	7.7	738.7	16.5	714.6	8.6
354U1451 A37F-Spot 1	537	111440	4.5	15.4385	0.7	1.0497	1.4	0.1176	1.2	0.85	716.6	8.0	728.7	7.2	766.0	15.4	716.6	8.0
354U1451 A37F-Spot 142	709	90520	1.6	15.2892	0.7	1.0724	1.5	0.1190	1.3	0.88	724.7	9.0	739.9	7.8	786.5	14.8	724.7	9.0
354U1451 A37F-Spot 285	152	35104	1.2	15.1678	0.8	1.0927	1.6	0.1203	1.4	0.86	732.1	9.6	749.8	8.6	803.2	17.5	732.1	9.6
354U1451 A37F-Spot 85	43	16500	4.4	14.9196	1.3	1.1446	1.8	0.1239	1.2	0.68	753.0	8.4	774.7	9.5	837.7	26.9	753.0	8.4
354U1451 A37F-Spot 49	149	96274	1.3	15.2777	1.0	1.1495	1.9	0.1274	1.7	0.86	773.2	12.2	777.0	10.6	788.1	20.8	773.2	12.2

354U1451 A37F-Spot 303	141	41293	2.2	15.3546	0.8	1.1488	1.5	0.1280	1.2	0.82	776.4	8.9	776.7	8.1	777.5	17.8	776.4	8.9
354U1451 A37F-Spot 160	224	82107	4.1	14.9560	0.9	1.1883	1.6	0.1290	1.3	0.83	781.9	9.9	795.2	8.9	832.6	18.6	781.9	9.9
354U1451 A37F-Spot 54	2336	8822456	1.3	14.9743	0.8	1.1952	1.4	0.1299	1.1	0.80	787.1	8.1	798.4	7.6	830.0	17.2	787.1	8.1
354U1451 A37F-Spot 66	610	510409	6.2	14.2210	0.7	1.2597	2.4	0.1300	2.3	0.96	787.7	17.3	827.8	13.8	936.8	14.4	787.7	17.3
354U1451 A37F-Spot 314	663	101766	15.1	14.5055	0.7	1.2610	1.7	0.1327	1.5	0.92	803.4	11.7	828.4	9.6	896.0	14.0	803.4	11.7
354U1451 A37F-Spot 311	497	51446	1.9	14.7197	1.1	1.2438	1.6	0.1328	1.2	0.76	804.1	9.4	820.6	9.2	865.7	22.1	804.1	9.4
354U1451 A37F-Spot 82	357	104210	1.3	14.8490	0.6	1.2426	1.5	0.1339	1.4	0.92	809.9	10.9	820.1	8.7	847.5	12.5	809.9	10.9
354U1451 A37F-Spot 228	813	288017	3.5	14.8822	0.8	1.2513	1.5	0.1351	1.3	0.85	817.0	9.8	824.0	8.5	842.9	16.6	817.0	9.8
354U1451 A37F-Spot 136	282	40421	2.3	15.1459	0.8	1.2327	1.5	0.1355	1.3	0.86	819.0	10.1	815.6	8.5	806.2	16.1	819.0	10.1
354U1451 A37F-Spot 226	404	167047	2.7	14.2447	0.9	1.3191	1.9	0.1363	1.7	0.88	823.9	13.0	854.1	11.0	933.4	18.8	823.9	13.0
354U1451 A37F-Spot 168	74	23250	2.1	14.8218	0.8	1.2686	1.4	0.1364	1.2	0.83	824.4	9.0	831.8	7.9	851.3	15.9	824.4	9.0
354U1451 A37F-Spot 295	103	578058	3.0	15.1102	0.9	1.2463	1.5	0.1366	1.2	0.82	825.7	9.5	821.8	8.4	811.2	17.9	825.7	9.5
354U1451 A37F-Spot 286	410	1718653	2.5	14.7588	0.9	1.2770	1.6	0.1368	1.3	0.82	826.3	10.2	835.5	9.1	860.2	18.8	826.3	10.2
354U1451 A37F-Spot 56	1015	40452	5.3	14.2977	1.1	1.3280	1.8	0.1378	1.5	0.80	832.0	11.3	858.0	10.5	925.7	22.1	832.0	11.3
354U1451 A37F-Spot 184	223	152568	4.3	14.2424	0.9	1.3396	2.2	0.1384	2.0	0.92	835.8	15.7	863.1	12.7	933.7	17.7	835.8	15.7
354U1451 A37F-Spot 116	150	76823	1.9	14.7922	0.8	1.2899	1.5	0.1384	1.2	0.83	835.9	9.6	841.3	8.4	855.5	17.1	835.9	9.6
354U1451 A37F-Spot 279	155	74765	3.2	14.8812	0.8	1.2831	1.3	0.1385	1.0	0.79	836.4	7.9	838.2	7.3	843.0	16.5	836.4	7.9
354U1451 A37F-Spot 244	971	290189	2.4	14.6409	0.6	1.3159	1.3	0.1398	1.2	0.90	843.4	9.2	852.7	7.5	876.8	11.9	843.4	9.2
354U1451 A37F-Spot 52	1199	351662	6.4	14.7486	0.7	1.3300	1.8	0.1423	1.6	0.91	857.8	13.1	858.9	10.4	861.7	15.1	857.8	13.1
354U1451 A37F-Spot 282	37	29796	2.0	14.5153	0.8	1.3732	1.6	0.1446	1.4	0.86	870.8	11.4	877.6	9.6	894.6	17.1	870.8	11.4
354U1451 A37F-Spot 95	424	210987	2.2	14.5632	0.8	1.3871	1.6	0.1466	1.4	0.88	881.7	11.4	883.5	9.3	887.8	15.5	881.7	11.4
354U1451 A37F-Spot 251	297	86553	1.2	14.4740	0.7	1.4015	1.5	0.1472	1.3	0.88	885.2	10.7	889.6	8.8	900.5	14.7	885.2	10.7
354U1451 A37F-Spot 135	305	88241	1.7	14.4848	0.6	1.4020	1.1	0.1473	0.9	0.83	886.1	7.6	889.8	6.6	899.0	13.0	886.1	7.6
354U1451 A37F-Spot 27	254	100151	2.9	14.5490	0.6	1.4118	1.4	0.1490	1.2	0.89	895.6	10.2	893.9	8.2	889.9	13.0	895.6	10.2
354U1451 A37F-Spot 115	199	64848	3.2	14.3829	0.8	1.4308	1.8	0.1493	1.7	0.90	897.1	13.9	901.9	11.0	913.5	16.5	913.5	16.5
354U1451 A37F-Spot 154	226	201710	2.5	14.3438	0.6	1.4841	1.4	0.1545	1.3	0.90	925.9	10.9	923.9	8.4	919.1	12.2	919.1	12.2
354U1451 A37F-Spot 296	86	56962	0.5	14.2814	1.0	1.5123	1.8	0.1567	1.5	0.84	938.5	12.8	935.4	10.7	928.1	19.6	928.1	19.6
354U1451 A37F-Spot 140	371	302754	5.6	14.2811	0.7	1.4929	1.2	0.1547	0.9	0.77	927.2	7.8	927.5	7.1	928.1	15.2	928.1	15.2
354U1451 A37F-Spot 252	98	42809	0.9	14.2137	0.9	1.4686	1.5	0.1515	1.2	0.80	909.1	10.2	917.6	9.1	937.8	18.6	937.8	18.6
354U1451 A37F-Spot 15	52	63919	2.6	14.1828	1.1	1.5084	1.6	0.1552	1.1	0.69	930.2	9.5	933.8	9.6	942.3	23.2	942.3	23.2
354U1451 A37F-Spot 113	1412	240903	1.4	14.0772	0.6	1.5252	1.4	0.1558	1.2	0.91	933.3	10.7	940.6	8.3	957.6	11.6	957.6	11.6
354U1451 A37F-Spot 55	1175	222700	15.9	14.0755	1.0	1.4595	1.8	0.1491	1.5	0.81	895.7	12.2	913.8	10.8	957.8	21.5	957.8	21.5
354U1451 A37F-Spot 137	398	116479	4.0	14.0641	0.6	1.4714	1.4	0.1502	1.2	0.90	901.8	10.3	918.7	8.2	959.5	12.3	959.5	12.3
354U1451 A37F-Spot 13	383	136863	1.0	14.0597	0.7	1.6016	1.5	0.1634	1.3	0.89	975.6	12.1	970.8	9.4	960.1	14.1	960.1	14.1
354U1451 A37F-Spot 34	820	453454	5.0	13.9846	0.8	1.6045	1.5	0.1628	1.3	0.86	972.4	11.5	972.0	9.3	971.1	15.8	971.1	15.8
354U1451 A37F-Spot 197	202	121104	3.1	13.9795	0.8	1.4372	1.6	0.1458	1.3	0.85	877.3	11.0	904.6	9.4	971.8	16.9	971.8	16.9
354U1451 A37F-Spot 123	540	143614	3.2	13.9215	0.8	1.5611	1.4	0.1577	1.2	0.84	943.9	10.6	954.9	8.9	980.3	16.0	980.3	16.0
354U1451 A37F-Spot 64	204	448027	2.3	13.8671	0.8	1.5880	1.8	0.1598	1.6	0.90	955.6	14.6	965.5	11.4	988.3	16.2	988.3	16.2
354U1451 A37F-Spot 40	654	148451	9.2	13.8640	0.6	1.6681	1.2	0.1678	1.1	0.87	1000.0	9.8	996.4	7.7	988.7	12.2	988.7	12.2
354U1451 A37F-Spot 231	580	119575	2.0	13.8487	0.6	1.6045	1.6	0.1612	1.5	0.92	963.6	13.5	971.9	10.3	991.0	13.1	991.0	13.1
354U1451 A37F-Spot 128	195	58537	2.9	13.8376	0.8	1.7007	1.5	0.1708	1.3	0.86	1016.3	12.3	1008.8	9.7	992.6	15.4	992.6	15.4
354U1451 A37F-Spot 69	321	85553	4.3	13.8078	0.9	1.5358	1.5	0.1539	1.1	0.78	922.6	9.7	944.8	9.0	996.9	18.7	996.9	18.7
354U1451 A37F-Spot 158	473	71624	41.9	13.8075	0.6	1.6715	1.4	0.1675	1.2	0.89	998.1	11.3	997.8	8.7	997.0	12.7	997.0	12.7
354U1451 A37F-Spot 290	256	1056459	2.8	13.7316	0.7	1.6557	1.1	0.1650	0.9	0.80	984.3	8.4	991.7	7.2	1008.2	13.8	1008.2	13.8
354U1451 A37F-Spot 143	496	105356	4.5	13.7214	0.8	1.6770	1.6	0.1670	1.4	0.87	995.4	12.6	999.9	10.0	1009.7	15.9	1009.7	15.9
354U1451 A37F-Spot 61	300	102403	2.7	13.6778	0.8	1.7096	1.5	0.1697	1.3	0.86	1010.3	11.9	1012.1	9.6	1016.1	15.6	1016.1	15.6
354U1451 A37F-Spot 17	126	75263	2.4	13.6370	0.8	1.6451	1.5	0.1628	1.3	0.84	972.2	11.7	987.7	9.7	1022.2	16.9	1022.2	16.9
354U1451 A37F-Spot 80	409	690475	5.7	13.5876	0.8	1.6775	2.2	0.1654	2.1	0.94	986.6	19.0	1000.0	14.1	1029.5	15.9	1029.5	15.9
354U1451 A37F-Spot 99	152	73069	4.6	13.5745	1.0	1.7046	2.3	0.1679	2.1	0.91	1000.5	19.3	1010.3	14.7	1031.5	19.5	1031.5	19.5
354U1451 A37F-Spot 119	281	73304	1.4	13.5664	0.8	1.5198	1.5	0.1496	1.2	0.85	898.8	10.4	938.4	9.0	1032.7	15.6	1032.7	15.6
354U1451 A37F-Spot 21	340	8090281	7.4	13.5550	0.7	1.6889	1.6	0.1661	1.5	0.90	990.6	13.4	1004.3	10.3	1034.4	14.5	1034.4	14.5
354U1451 A37F-Spot 88	124	163155	1.9	13.4991	0.6	1.7935	1.4	0.1757	1.3	0.90	1043.3	12.2	1043.1	9.2	1042.7	12.6	1042.7	12.6
354U1451 A37F-Spot 16	216	7469817	5.1	13.4333	0.8	1.6719	1.6	0.1630	1.3	0.86	973.2	12.0	997.9	9.9	1052.6	16.3	1052.6	16.3
354U1451 A37F-Spot 233	1781	2482360	51.0	13.4274	1.3	1.5856	1.9	0.1545	1.4	0.74	926.0	11.9	964.6	11.6	1053.5	25.4	1053.5	25.4
354U1451 A37F-Spot 294	1039	143742	2.1	13.3956	0.6	1.8450	1.3	0.1793	1.2	0.89	1063.3	11.4	1061.7	8.6	1058.3	12.0	1058.3	12.0
354U1451 A37F-Spot 198	293	86676	3.8	13.3754	0.8	1.7882	1.5	0.1735	1.3	0.86	1031.6	12.5	1041.2	9.9	1061.3	15.5	1061.3	15.5
354U1451 A37F-Spot 19	251	73508	2.6	13.3337	0.9	1.8231	1.5	0.1764	1.2	0.79	1047.2	11.6	1053.8	9.9	1067.6	18.4	1067.6	18.4
354U1451 A37F-Spot 272	566	1532123	13.0	13.3325	1.0	1.7925	1.9	0.1734	1.6	0.84	1030.8	15.0	1042.7	12.2	1067.8	20.6	1067.8	20.6
354U1451 A37F-Spot 92	798	71198	2.3	13.3150	0.6	1.6089	1.5	0.1554	1.3	0.90	931.4	11.4	973.7	9.2	1070.4	12.8	1070.4	12.8
354U1451 A37F-Spot 186	28	14119	1.1	13.2657	0.9	1.8688	1.6	0.1799	1.2	0.80	1066.3	12.2	1070.1	10.3	1077.8	18.7	1077.8	18.7
354U1451 A37F-Spot 20	600	555863	2.0	13.2545	0.5	1.8885	1.2	0.1816	1.1	0.90	1075.8	10.6	1077.1	7.9	1079.5	10.6	1079.5	10.6
354U1451 A37F-Spot 38	690	294852	6.6	13.2269	0.7	1.8678	1.9	0.1793	1.8	0.92	1062.9	17.3	1069.7	12.6	1083.7	14.7	1083.7	14.7
354U1451 A37F-Spot 247	471	292748	9.7	13.1635	0.5	1.8508	1.4	0.1768	1.3	0.93	1049.3	13.0	1063.7	9.5	1093.4	10.9	1093.4	10.9
354U1451 A37F-Spot 275	867	623538	2.3	13.1191	0.6	1.8489	1.1	0.1760	1.0	0.86	1045.0	9.5	1063.0	7.5	1100.1	11.5	1100.1	11.5
354U1451 A37F-Spot 46	323	423823	4.3	13.1002	0.8	1.7319	2.0	0.1646	1.8	0.90	982.4	16.4	1020.5	12.8	1103.0	16.9	1103.0	16.9
354U1451 A37F-Spot 146	233	67144	2.0	13.0436	0.8	1.8942	1.4	0.1793	1.1	0.83	1063.0	11.1	1079.0	9.1	1111.6	15.5	1111.6	15.5
354U1451 A37F-Spot 222	1697	299902	3.2	12.9784	0.8	1.7743	1.8	0.1671	1.6	0.89	996.1	15.0	1036.1	11.9	1121.6	16.9	1121.6	16.9
354U1451 A37F-Spot 236	610	349744	7.3	12.9608	0.7	1.9148	1.6	0.1801	1.4	0.88	1067.4	13.5	1086.3	10.4	1124.3	14.7	1124.3	14.7
354U1451 A37F-Spot 219	1130	184793	23.2	12.9396	0.9	1.6807	1.5	0.1578	1.2	0.81	944.5	10.9	1001.2	9.7	1127.6	17.7	1127.6	17.7
354U1451 A37F-Spot 33	59	27393	1.5	12.9319	0.7	2.0677	1.4	0.1940	1.2	0.85	1143.1	12.5	1138.2	9.6	1128.8	14.5	1128.8	14.5
354U1451 A37F-Spot 12	394	389153	1.7	12.8524	0.6	2.0886	1.5	0.1948	1.4	0.90	1147.2	14.3	1145.1	10.4	1141.1	12.9	1141.1	12.9
354U1451 A37F-Spot 204	75	17250	1.5	12.8506	0.8	2.0498	1.7	0.1911	1.5	0.88	1127.5	15.4	1132.2	11.5	1141.4	15.5	1141.4	15.5
354U1451 A37F-Spot 149	30	13461	0.8	12.8315	0.9	2.1573	1.7	0.2008	1.5	0.86	1179.9	16.0	1167.4	12.0	1144.3	17.4	1144.3	17.4
354U1451 A37F-Spot 278	209	73556	1.6	12.8194	0.7	2.1014	1.6	0.1955	1.4	0.88	1150.9	14.6	1149.3	10.8	1146.2	14.7	1146.2	14.7
354U1451 A37F-Spot 232	2282	484273	12.8	12.7713	0.7	2.0128	1.2	0.1865	1.0	0.83	1102.5	10.3	1119.8	8.4	1153.6	13.8	1153.6	13.8
354U1451 A37F-Spot 110	373	96937	12.2	12.7704	0.9	2.0581	1.8	0.1907	1.5	0.86	1125.2	16.0	1135.0	12.3	1153.8	18.1	1153.8	18.1
354U1451 A37F-Spot 156	238	266358	0.8	12.7630	0.7	2.0752	1.3	0.1922	1.1	0.84	1133.2	11.7	1140.7	9.2	1154.9	14.2	1154.9	14.2
354U1451 A37F-Spot 70	1478	3528524	5.3	12.7358	0.8	1.9613	1.9	0.1812	1.8	0.92	1073.8	17.7	1102.3	13.0	1159.2	14.9	1159.2	14.9
354U1451 A37F-Spot 288	55	19495	0.5	12.6996	0.8	2.1562	1.5	0.1987	1.2	0.84	1168.2	13.3	1167.0	10.3	1164.8	16.0	1164.8	16.0
354U1451 A37F-Spot 41	286	249471	2.3	12.6836	0.8	2.1640	1.5	0.1992	1.3	0.85	1170.8	13.6	1169.6	10.3	1167.3	15.3	1167.3	15.3
354U1451 A37F-Spot 254	91	34470	1.5	12.6801	0.8	2.1146	1.5	0.1946	1.3	0.83	1146.0	13.2	1153.6	10.5	1167.8	16.9	1167.8	16.9
354U1451 A37F-Spot 188	719	290490	5.8	12.5916	0.6	2.0087	1.3	0.1835	1.2	0.88	1086.2	11.8	1118.5	9.1	1181.7	12.6	1181.7	12.6
354U1451 A37F-Spot 77	1010	597176	1.1	12.5596	0.7	2.2451	1.5	0.2046	1.3	0.88	1200.0	14.7	1195.3	10.7	1186.8	14.2	1186.8	14.2
354U1451 A37F-Spot 114	100	54985	0.9	12.4977	0.9	1.9632	1.6	0.1780	1.3	0.84	1056.2	12.9	1103.0	10.6	1196.5	17.0	1196.5	17.0
354U1451 A37F-Spot 271	156	46425	0.5	12.4789	1.0	2.2306	1.4	0.2020	1.0	0.72	1185.9	10.8	1190.7	9.8	1199.4	19.1	1199.4	19.1
354U1451 A37F-Spot 68	1059	65714	1.4	12.4480	0.8	1.8277	1.4	0.1651	1.1	0.83	984.9	10.4	1055.5	9.1	1204.4	15.3	1204.4	15.3
354U1451 A37F-Spot 103	131	182031	0.6	12.4289	0.8	2.2338	1.6	0.2014	1.4	0.86	1183.1	14.6	1191.7	11.1	1207.4	16.0	1207.4	16.0
354U1451 A37F-Spot 47	387	188136	2.0	12.3921	0.9	2.2655	1.8	0.2037	1.6	0.87	1195.2	17.2	1201.6	12.7	1213.2	17.5	1213.2	17.5
354U1451 A37F-Spot 230	372	136164	4.4	12.2225	0.6	2.2856	1.4	0.2027	1.3	0.89	1189.8	13.7	1207.8	10.0	1240.3	12.7	1240.3	12.7
354U1451 A37F-Spot 96	125	68386	1.3	12.1229	0.6	2.5537	1.3	0.2246	1.1	0.87	1306.3	13.4	1287.5	9.4	1256.3	12.3	1256.3	12.3
354U1451 A37F-Spot 106	177	124223	2.7	11.7801	0.7	2.6060	1.5	0.2227	1.4	0.90	1296.3	16.0	1302.3	11.1	1312.1	13.0	1312.1	13.0
354U1451 A37F-Spot 117	299	111058	0.8	11.7149	0.7	2.5254	1.3	0.2147	1.1	0.84	1253.6	12.4	1279.4	9.5	1322.9	13.7	1322.9	13.7
354U1451 A37F-Spot 104	248	523232	2.4	11.4864	0.6	2.6999	1.3	0.2250	1.1	0.89	1308.4	13.5	1328.4	9.5	1361.0	11.4	1361.0	11.4
354U1451 A37F-Spot 291	167	73898	2.5	11.2219	0.6	2.9795	1.2	0.2426	1.0	0.86	1400.2	12.9	1402.4	9.1	1405.7	11.8	1405.7	11.8
354U1451 A37F-Spot 261	1777	408479	3.0	11.1251	0.7	2.9300	1.4	0.2365	1.2	0.87	1368.6	14.8	1389.7	10.5	1422.3	13.0	1422.3	13.0
354U1451 A37F-Spot 126	430	111350	1.0	11.1093	0.6	2.9738	1.3	0.2397	1.2	0.89	1385.2	15.0	1400.9	10.3	1425.0	11.6	1425.0	11.6
354U1451 A37F-Spot 42	507	138692	3.1	11.0166	1.1	3.0594	1.8	0.2446	1.4	0.79	1410.3	17.9	1422.6	13.7	1441.0	20.7	1441.0	20.7
354U1451 A37F-Spot 195	316	166244	2.2	10.9678	0.7	2.9713	1.6	0.2365	1.4	0.91	1368.3	17.4	1400.3	11.8	1449.4	12.5	1449.4	12.5
354U1451 A37F-Spot 192	1154	904267	1.2	10.5616	0.7	3.0383	2.4	0.2328	2.3	0.96	1349.3	28.3	1417.3	18.5	1520.9	12.5	1520.9	12.5
354U1451 A37F-Spot 277	409	223828	5.2	10.5275	0.6	3.3111	1.8	0.2529	1.7	0.95	1453.5	21.9	1483.7	13.8	1527.0	10.5	1527.0	10.5
354U1451 A37F-Spot 178	325	97881	1.0	10.2914	0.7	3.4782	2.0	0.2597	1.9	0.94	1488.4	25.2	1522.3	15.9	1569.6	12.7	1569.6	12.7

354U1451 A37F-Spot 187	117	69526	0.6	10.2600	0.7	3.7905	1.2	0.2822	0.9	0.80	1602.3	13.4	1590.7	9.5	1575.4	13.3	1575.4	13.3
354U1451 A37F-Spot 73	240	346924	1.5	10.2432	0.8	3.4608	1.5	0.2572	1.3	0.85	1475.6	17.0	1518.3	12.0	1578.4	15.0	1578.4	15.0
354U1451 A37F-Spot 293	237	108037	1.9	10.2194	0.8	3.6472	1.4	0.2704	1.1	0.80	1543.0	15.1	1559.9	10.9	1582.8	15.3	1582.8	15.3
354U1451 A37F-Spot 43	219	354217	2.1	10.1867	0.8	3.7514	1.6	0.2773	1.3	0.85	1577.6	18.8	1582.4	12.7	1588.8	15.8	1588.8	15.8
354U1451 A37F-Spot 206	453	107753	2.3	10.1420	0.9	3.8097	1.9	0.2804	1.7	0.89	1593.1	24.3	1594.8	15.6	1597.0	16.4	1597.0	16.4
354U1451 A37F-Spot 268	101	54157	1.2	10.1093	0.6	3.8340	1.4	0.2812	1.3	0.92	1597.6	18.7	1599.9	11.6	1603.0	10.8	1603.0	10.8
354U1451 A37F-Spot 273	101	74704	1.1	10.0814	0.7	3.8785	1.2	0.2837	0.9	0.81	1610.0	13.3	1609.2	9.3	1608.2	12.7	1608.2	12.7
354U1451 A37F-Spot 267	305	120461	1.8	10.0003	0.7	3.9564	1.6	0.2871	1.4	0.88	1626.9	19.9	1625.3	12.7	1623.2	13.6	1623.2	13.6
354U1451 A37F-Spot 313	232	190085	2.4	9.9678	0.7	3.8684	1.4	0.2798	1.3	0.88	1590.2	17.8	1607.1	11.5	1629.3	12.3	1629.3	12.3
354U1451 A37F-Spot 256	211	309239	1.5	9.9360	0.7	3.7690	1.6	0.2717	1.5	0.91	1549.5	20.4	1586.2	13.1	1635.2	12.7	1635.2	12.7
354U1451 A37F-Spot 249	1495	124491	3.5	9.9025	0.7	3.4626	1.6	0.2488	1.5	0.90	1432.2	19.0	1518.7	12.9	1641.5	13.0	1641.5	13.0
354U1451 A37F-Spot 253	150	533408	1.8	9.8823	0.7	4.0920	1.3	0.2934	1.1	0.85	1658.6	16.4	1652.7	10.8	1645.3	13.0	1645.3	13.0
354U1451 A37F-Spot 67	189	223884	1.7	9.8305	0.7	3.9213	1.3	0.2797	1.2	0.86	1589.9	16.4	1618.1	10.9	1655.0	12.5	1655.0	12.5
354U1451 A37F-Spot 212	234	156593	3.5	9.8224	0.7	3.9816	1.4	0.2838	1.2	0.85	1610.3	16.7	1630.5	11.2	1656.5	13.3	1656.5	13.3
354U1451 A37F-Spot 227	163	70651	3.9	9.8168	1.0	4.0922	1.6	0.2915	1.3	0.80	1648.9	19.0	1652.8	13.4	1657.6	18.4	1657.6	18.4
354U1451 A37F-Spot 185	302	105569	1.7	9.7950	0.6	4.0395	1.3	0.2871	1.2	0.87	1627.0	16.8	1642.2	10.9	1661.7	12.0	1661.7	12.0
354U1451 A37F-Spot 283	249	94647	2.0	9.7162	1.0	3.6467	3.8	0.2571	3.6	0.96	1474.9	47.7	1559.8	29.9	1676.6	18.3	1676.6	18.3
354U1451 A37F-Spot 31	92	45606	2.7	9.6425	0.7	4.0797	1.6	0.2854	1.4	0.89	1618.7	19.9	1650.3	12.8	1690.7	13.3	1690.7	13.3
354U1451 A37F-Spot 257	731	2801088	1.7	9.5926	0.6	4.1608	1.4	0.2896	1.2	0.90	1639.5	17.8	1666.3	11.2	1700.3	11.0	1700.3	11.0
354U1451 A37F-Spot 6	228	1452723	1.1	9.5846	0.8	4.5447	1.3	0.3161	1.1	0.81	1770.5	16.4	1739.2	10.9	1701.8	14.2	1701.8	14.2
354U1451 A37F-Spot 11	562	976456	2.8	9.5361	0.9	4.4714	1.8	0.3094	1.6	0.87	1737.7	24.5	1725.7	15.3	1711.1	16.6	1711.1	16.6
354U1451 A37F-Spot 75	545	253546	1.7	9.4455	0.9	4.5484	1.7	0.3117	1.4	0.84	1749.2	22.0	1739.9	14.2	1728.7	17.0	1728.7	17.0
354U1451 A37F-Spot 218	273	211144	2.2	9.4219	0.8	4.5639	1.9	0.3120	1.7	0.89	1750.6	26.1	1742.7	15.8	1733.3	15.6	1733.3	15.6
354U1451 A37F-Spot 63	361	239343	3.0	9.3122	0.5	4.6429	1.2	0.3137	1.1	0.90	1759.0	17.3	1757.0	10.4	1754.7	9.9	1754.7	9.9
354U1451 A37F-Spot 97	164	373018	2.8	9.2256	0.7	4.6972	1.3	0.3144	1.1	0.85	1762.5	17.3	1766.7	11.1	1771.8	12.9	1771.8	12.9
354U1451 A37F-Spot 297	266	925634	8.2	9.0865	0.6	4.8174	1.3	0.3176	1.2	0.88	1778.0	18.3	1787.9	11.3	1799.5	11.7	1799.5	11.7
354U1451 A37F-Spot 210	190	708220	3.2	9.0765	0.6	4.8091	1.7	0.3167	1.6	0.94	1773.7	24.8	1786.5	14.4	1801.5	10.9	1801.5	10.9
354U1451 A37F-Spot 155	265	433462	1.7	9.0613	0.7	4.8410	1.7	0.3183	1.5	0.89	1781.3	23.1	1792.1	14.0	1804.5	13.6	1804.5	13.6
354U1451 A37F-Spot 299	294	137958	2.2	9.0337	0.7	4.9523	1.4	0.3246	1.2	0.86	1812.2	19.4	1811.2	12.1	1810.1	13.5	1810.1	13.5
354U1451 A37F-Spot 181	234	7477	2.3	9.0113	2.3	4.1239	2.8	0.2696	1.5	0.55	1539.0	20.9	1659.1	22.7	1814.6	42.2	1814.6	42.2
354U1451 A37F-Spot 189	130	124447	1.8	8.9978	0.8	4.6623	1.4	0.3044	1.2	0.83	1713.0	17.7	1760.5	11.8	1817.3	14.3	1817.3	14.3
354U1451 A37F-Spot 98	335	562516	2.2	8.9691	0.7	4.5425	1.5	0.2956	1.3	0.88	1669.5	19.7	1738.8	12.7	1823.1	13.4	1823.1	13.4
354U1451 A37F-Spot 81	1111	4182717	2.9	8.8418	0.7	4.6375	1.4	0.2975	1.2	0.86	1679.0	17.9	1756.0	11.8	1849.0	13.2	1849.0	13.2
354U1451 A37F-Spot 298	687	310139	5.2	8.8240	0.6	4.8400	1.4	0.3099	1.2	0.88	1740.1	18.2	1791.9	11.4	1852.6	11.7	1852.6	11.7
354U1451 A37F-Spot 62	264	1211041	2.8	8.8154	0.7	4.8359	1.5	0.3093	1.4	0.91	1737.4	21.1	1791.2	12.9	1854.4	11.8	1854.4	11.8
354U1451 A37F-Spot 129	78	217338	0.9	8.7411	0.8	5.2449	1.4	0.3327	1.2	0.84	1851.2	19.1	1859.9	12.0	1869.7	13.6	1869.7	13.6
354U1451 A37F-Spot 130	292	111387	12.0	8.7314	0.6	5.3214	1.4	0.3371	1.3	0.91	1872.8	20.5	1872.3	11.9	1871.7	10.6	1871.7	10.6
354U1451 A37F-Spot 87	2561	955212	8.3	8.3717	0.8	5.5821	1.7	0.3391	1.5	0.88	1882.2	25.0	1913.3	15.1	1947.2	15.1	1947.2	15.1
354U1451 A37F-Spot 315	268	218415	10.2	8.3589	0.8	5.8688	1.4	0.3559	1.1	0.83	1962.9	19.2	1956.6	11.9	1950.0	13.6	1950.0	13.6
354U1451 A37F-Spot 292	1043	667348	4.0	7.9128	0.6	6.8577	1.0	0.3937	0.8	0.81	2140.1	14.4	2093.2	8.6	2047.4	10.0	2047.4	10.0
354U1451 A37F-Spot 237	1444	615211	5.7	7.6930	0.5	6.1910	1.1	0.3456	1.0	0.90	1913.4	16.3	2003.2	9.6	2097.0	8.5	2097.0	8.5
354U1451 A37F-Spot 213	430	805197	2.3	7.5302	0.8	6.0956	1.8	0.3331	1.6	0.90	1853.2	26.2	1989.6	15.8	2134.5	14.0	2134.5	14.0
354U1451 A37F-Spot 281	119	300992	1.5	7.4886	0.6	7.1098	1.2	0.3863	1.1	0.85	2105.7	19.0	2125.3	11.0	2144.2	11.3	2144.2	11.3
354U1451 A37F-Spot 7	52	90406	1.2	7.4733	0.7	7.3079	1.3	0.3963	1.1	0.85	2151.9	21.0	2149.8	12.1	2147.8	12.3	2147.8	12.3
354U1451 A37F-Spot 65	277	198684	1.1	7.2657	0.7	7.0053	2.0	0.3693	1.9	0.95	2026.2	33.0	2112.1	17.8	2196.9	11.3	2196.9	11.3
354U1451 A37F-Spot 255	472	1805105	1.3	6.7174	0.9	8.6811	1.7	0.4231	1.4	0.85	2274.6	27.3	2305.1	15.3	2332.2	15.1	2332.2	15.1
354U1451 A37F-Spot 100	461	530002	1.9	6.5811	0.7	7.9491	1.2	0.3796	1.0	0.84	2074.3	18.1	2225.3	10.9	2367.2	11.2	2367.2	11.2
354U1451 A37F-Spot 263	168	105888	2.7	6.5037	0.9	9.3843	1.7	0.4428	1.4	0.85	2363.4	27.9	2376.3	15.2	2387.4	14.8	2387.4	14.8
354U1451 A37F-Spot 57	466	1383596	1.4	6.4235	0.6	8.3952	1.3	0.3913	1.1	0.88	2128.8	20.5	2274.7	11.7	2408.5	10.6	2408.5	10.6
354U1451 A37F-Spot 199	472	2058681	1.6	6.2872	0.7	8.9848	1.5	0.4099	1.3	0.88	2214.4	25.1	2336.5	13.9	2444.9	12.2	2444.9	12.2
354U1451 A37F-Spot 242	321	2689177	4.3	6.2665	0.6	10.3432	1.4	0.4703	1.3	0.90	2484.8	26.7	2466.0	13.3	2450.5	10.7	2450.5	10.7
354U1451 A37F-Spot 246	240	233784	1.0	6.2488	0.7	10.0059	1.4	0.4537	1.2	0.87	2411.6	24.6	2435.3	12.9	2455.2	11.5	2455.2	11.5
354U1451 A37F-Spot 175	634	66705	2.6	6.2405	0.6	8.4059	1.6	0.3806	1.4	0.92	2079.2	25.5	2275.8	14.2	2457.5	10.6	2457.5	10.6
354U1451 A37F-Spot 274	205	141201	3.6	6.2403	0.8	10.4918	1.4	0.4751	1.1	0.82	2505.7	23.3	2479.2	12.6	2457.5	13.1	2457.5	13.1
354U1451 A37F-Spot 193	531	55327	2.4	6.1573	0.6	9.1311	1.4	0.4079	1.3	0.92	2205.5	24.8	2351.2	13.2	2480.1	9.5	2480.1	9.5
354U1451 A37F-Spot 148	232	482659	1.8	6.1552	0.7	10.4047	1.3	0.4647	1.1	0.86	2460.2	23.3	2471.5	12.2	2480.7	11.2	2480.7	11.2
354U1451 A37F-Spot 18	377	894097	3.3	6.1192	0.6	10.2075	1.2	0.4532	1.1	0.87	2409.5	21.2	2453.8	11.2	2490.6	10.1	2490.6	10.1
354U1451 A37F-Spot 30	184	1628599	1.1	6.1023	0.7	10.0516	1.5	0.4451	1.4	0.89	2373.2	27.4	2439.5	14.3	2495.3	11.8	2495.3	11.8
354U1451 A37F-Spot 150	64	25441	0.9	6.0916	0.8	10.7343	1.9	0.4745	1.7	0.90	2503.1	34.6	2500.4	17.3	2498.2	13.9	2498.2	13.9
354U1451 A37F-Spot 125	383	247540	7.4	6.0493	0.6	9.6039	1.1	0.4215	1.0	0.85	2267.5	18.5	2397.5	10.5	2509.9	10.1	2509.9	10.1
354U1451 A37F-Spot 101	393	810775	2.1	5.9315	0.7	10.8218	1.6	0.4658	1.4	0.90	2464.9	29.4	2507.9	14.8	2543.0	11.6	2543.0	11.6
354U1451 A37F-Spot 133	889	29453	4.8	5.7988	0.8	11.3062	1.3	0.4757	1.1	0.82	2508.5	22.7	2548.7	12.5	2580.8	12.8	2580.8	12.8
354U1451 A37F-Spot 260	137	92242	1.0	5.6467	0.7	12.1961	1.3	0.4997	1.1	0.86	2612.5	24.0	2619.6	12.2	2625.1	11.1	2625.1	11.1
354U1451 A37F-Spot 202	172	93488	1.3	5.3692	0.7	11.7431	1.5	0.4575	1.4	0.90	2428.5	28.2	2584.1	14.4	2708.6	10.9	2708.6	10.9
354U1451 A37F-Spot 3	416	517936	1.4	5.3670	0.8	13.3048	1.7	0.5181	1.5	0.89	2691.2	33.1	2701.5	15.9	2709.3	12.5	2709.3	12.5
354U1451 A37F-Spot 90	108	254168	1.6	5.2815	0.7	13.8093	1.4	0.5292	1.2	0.86	2738.1	26.0	2736.7	12.9	2735.7	11.5	2735.7	11.5
354U1451 A37F-Spot 162	92	78991	2.4	5.2183	0.8	13.4910	1.4	0.5108	1.2	0.84	2660.1	25.9	2714.7	13.4	2755.5	12.7	2755.5	12.7
354U1451 A37F-Spot 124	956	183186	2.4	5.0141	0.7	15.3075	1.3	0.5569	1.1	0.83	2853.8	25.1	2834.6	12.5	2820.9	12.0	2820.9	12.0
354U1451 A37F-Spot 207	115	66031	1.8	5.0105	0.9	13.6763	2.0	0.4972	1.8	0.89	2601.8	38.0	2727.6	18.8	2822.1	14.6	2822.1	14.6
354U1451 A37F-Spot 169	180	148987	2.5	4.5178	0.7	17.9434	1.4	0.5882	1.2	0.86	2982.1	28.4	2986.7	13.3	2989.8	11.2	2989.8	11.2
354U1451 A37F-Spot 182	79	150268	1.1	3.7397	1.0	23.6194	1.8	0.6409	1.4	0.81	3192.6	36.1	3252.8	17.2	3290.1	16.3	3290.1	16.3
354U1451 A37F-Spot 216	182	371566	1.8	3.4103	0.6	28.6352	2.8	0.7086	2.8	0.98	3453.1	74.3	3441.1	27.9	3434.1	9.4	3434.1	9.4

Table _____. U-Pb geochronologic analyses.																		
#17 U1451A_41F						Isotope ratios						Apparent ages (Ma)						
Late Miocene 6.5 Ma																		
Analysis	U	206Pb	U/Th	206Pb*	±	207Pb*	±	206Pb*	±	error	206Pb*	±	207Pb*	±	206Pb*	±	Best age	±
	(ppm)	204Pb		207Pb*	(%)	235U*	(%)	238U	(%)	corr.	238U*	(Ma)	235U	(Ma)	207Pb*	(Ma)	(Ma)	(Ma)
-354-U1451A-41F Spot 189	125	2332	1.3	5.0871	4.7	0.1313	5.3	0.0048	2.4	0.46	31.2	0.8	125.3	6.2	2797.2	76.9	31.2	0.8
-354-U1451A-41F Spot 290	150	1465	0.9	5.4484	3.4	0.1675	4.3	0.0066	2.6	0.60	42.5	1.1	157.3	6.3	2684.4	56.9	42.5	1.1
-354-U1451A-41F Spot 4	910	5144	1.1	20.8024	3.3	0.0541	3.5	0.0082	1.1	0.32	52.4	0.6	53.5	1.8	101.7	77.9	52.4	0.6
-354-U1451A-41F Spot 199	1067	279491	1.0	20.0656	1.6	0.1166	1.9	0.0170	1.0	0.50	108.5	1.0	111.9	2.0	186.3	38.1	108.5	1.0
-354-U1451A-41F Spot 291	1081	26871	2.8	20.2456	1.4	0.1242	2.0	0.0182	1.5	0.72	116.6	1.7	118.9	2.3	165.5	32.9	116.6	1.7
-354-U1451A-41F Spot 273	1254	119227	1.4	20.0185	1.3	0.1441	1.6	0.0209	1.0	0.58	133.6	1.3	136.7	2.1	191.9	30.9	133.6	1.3
-354-U1451A-41F Spot 265	531	258309	2.4	13.8790	2.8	0.5614	3.2	0.0565	1.6	0.50	354.5	5.5	452.4	11.8	986.5	56.8	354.5	5.5
-354-U1451A-41F Spot 140	948	13188	1.3	14.1825	4.5	0.5516	7.2	0.0568	5.5	0.78	355.9	19.2	446.1	25.8	942.3	92.6	355.9	19.2
-354-U1451A-41F Spot 145	372	150382	1.0	17.7241	1.2	0.5746	1.6	0.0739	1.2	0.70	459.5	5.1	461.0	6.1	468.0	26.0	459.5	5.1
-354-U1451A-41F Spot 211	2240	43889	5.5	13.8396	1.2	0.7440	3.9	0.0747	3.7	0.95	464.5	16.7	564.8	16.9	992.3	24.6	464.5	16.7
-354-U1451A-41F Spot 149	1624	290494	0.8	17.7632	0.8	0.5897	1.6	0.0760	1.4	0.86	472.2	6.4	470.7	6.1	463.1	18.3	472.2	6.4
-354-U1451A-41F Spot 215	718	24965	9.3	17.9095	0.8	0.5895	1.4	0.0766	1.2	0.81	475.8	5.3	470.5	5.4	444.9	18.9	475.8	5.3
-354-U1451A-41F Spot 122	1286	38650	33.6	17.5825	0.8	0.6043	1.4	0.0771	1.2	0.82	478.7	5.4	479.9	5.4	485.7	17.8	478.7	5.4
-354-U1451A-41F Spot 99	619	19971	2.4	17.5404	1.1	0.6125	1.7	0.0780	1.3	0.77	483.9	6.3	485.2	6.7	491.0	24.4	483.9	6.3
-354-U1451A-41F Spot 26	729	66342	1.7	17.5455	0.8	0.6164	2.2	0.0785	2.0	0.93	487.0	9.6	487.6	8.5	490.3	18.1	487.0	9.6
-354-U1451A-41F Spot 175	3568	405271	2.7	17.3793	0.9	0.6226	1.5	0.0785	1.2	0.78	487.2	5.5	491.4	5.8	511.3	20.4	487.2	5.5
-354-U1451A-41F Spot 234	359	91833	0.7	16.8259	1.2	0.6442	1.5	0.0786	1.0	0.64	488.0	4.6	504.9	6.1	582.0	25.5	488.0	4.6
-354-U1451A-41F Spot 141	2111	155494	6.6	17.4843	0.8	0.6258	1.5	0.0794	1.3	0.85	492.5	6.0	493.5	5.8	498.0	17.4	492.5	6.0
-354-U1451A-41F Spot 279	674	34738	3.1	17.3995	1.0	0.6291	1.8	0.0794	1.6	0.86	492.7	7.5	495.5	7.2	508.8	20.9	492.7	7.5
-354-U1451A-41F Spot 190	446	110884	2.6	17.1295	1.0	0.6396	1.4	0.0795	0.9	0.67	493.1	4.3	502.1	5.4	543.0	22.2	493.1	4.3
-354-U1451A-41F Spot 241	169	159027	2.8	17.0807	1.6	0.6444	2.1	0.0799	1.2	0.60	495.3	5.9	505.1	8.2	549.3	35.9	495.3	5.9
-354-U1451A-41F Spot 187	266	16250	2.2	17.1905	1.2	0.6414	1.5	0.0800	0.9	0.61	496.1	4.5	503.1	6.1	535.3	26.7	496.1	4.5
-354-U1451A-41F Spot 236	1976	123742	3.1	17.5261	0.8	0.6291	1.2	0.0800	0.9	0.73	496.1	4.2	495.5	4.7	492.8	18.0	496.1	4.2
-354-U1451A-41F Spot 185	570	57652	2.3	17.6420	1.0	0.6255	1.5	0.0801	1.0	0.70	496.5	4.9	493.3	5.7	478.2	23.2	496.5	4.9
-354-U1451A-41F Spot 262	144	24275	1.6	16.7089	1.4	0.6652	1.9	0.0807	1.3	0.68	500.0	6.4	517.8	7.9	597.1	30.8	500.0	6.4
-354-U1451A-41F Spot 101	113	6405	0.5	17.4076	2.1	0.6433	2.4	0.0813	1.2	0.48	503.6	5.6	504.4	9.6	507.8	46.5	503.6	5.6
-354-U1451A-41F Spot 288	130	12115	1.7	16.7715	2.0	0.6683	2.4	0.0813	1.3	0.56	504.1	6.5	519.7	9.8	589.1	43.2	504.1	6.5
-354-U1451A-41F Spot 82	150	91826	1.2	16.9804	1.4	0.6612	1.9	0.0815	1.3	0.69	504.9	6.5	515.4	7.8	562.1	30.2	504.9	6.5
-354-U1451A-41F Spot 213	521	16397	3.8	17.5228	0.9	0.6425	1.4	0.0817	1.0	0.75	506.2	5.0	503.8	5.4	493.2	19.8	506.2	5.0
-354-U1451A-41F Spot 160	292	17102	0.9	17.3784	1.1	0.6527	1.8	0.0823	1.4	0.80	509.8	7.1	510.1	7.3	511.4	24.1	509.8	7.1
-354-U1451A-41F Spot 278	557	151782	2.2	17.2030	0.7	0.6598	1.3	0.0824	1.1	0.84	510.2	5.5	514.5	5.4	533.7	15.6	510.2	5.5
-354-U1451A-41F Spot 143	1251	4192284	12.0	17.2629	1.0	0.6584	1.5	0.0825	1.2	0.77	510.9	5.7	513.6	6.1	526.1	21.2	510.9	5.7
-354-U1451A-41F Spot 298	647	36064	11.3	16.9632	1.1	0.6762	1.7	0.0832	1.4	0.79	515.4	6.8	524.5	7.1	564.3	23.1	515.4	6.8
-354-U1451A-41F Spot 24	675	90490	1.2	16.6963	0.8	0.6891	1.5	0.0835	1.3	0.86	516.9	6.5	532.3	6.3	598.8	16.8	516.9	6.5
-354-U1451A-41F Spot 232	213	9813	0.5	17.3948	1.4	0.6659	1.9	0.0840	1.3	0.69	520.2	6.6	518.2	7.8	509.4	30.5	520.2	6.6
-354-U1451A-41F Spot 274	124	82590	1.2	16.4980	1.3	0.7022	1.7	0.0841	1.1	0.63	520.3	5.4	540.1	7.2	624.6	28.7	520.3	5.4
-354-U1451A-41F Spot 45	3759	128878	2.3	17.3106	0.9	0.6702	1.4	0.0842	1.0	0.73	521.1	5.1	520.9	5.7	520.0	20.8	521.1	5.1
-354-U1451A-41F Spot 134	212	41255	1.3	17.0732	1.4	0.6930	1.8	0.0859	1.0	0.57	531.0	5.1	534.6	7.3	550.3	31.3	531.0	5.1
-354-U1451A-41F Spot 255	161	24840	3.3	16.7775	1.2	0.7066	1.8	0.0860	1.3	0.73	531.9	6.8	542.7	7.6	588.3	26.9	531.9	6.8
-354-U1451A-41F Spot 71	291	55964	3.5	17.0966	1.2	0.6943	2.1	0.0861	1.8	0.82	532.6	9.0	535.4	8.9	547.3	26.9	532.6	9.0
-354-U1451A-41F Spot 282	268	10938	1.1	16.9002	1.7	0.7046	2.1	0.0864	1.2	0.59	534.2	6.2	541.5	8.6	572.5	36.2	534.2	6.2
-354-U1451A-41F Spot 310	82	4896	2.0	16.8717	3.1	0.7058	3.3	0.0864	1.1	0.35	534.3	5.9	542.3	13.8	576.1	67.2	534.3	5.9
-354-U1451A-41F Spot 314	298	29273	2.6	17.0153	1.0	0.7050	1.5	0.0870	1.1	0.72	538.0	5.6	541.8	6.3	557.7	22.8	538.0	5.6
-354-U1451A-41F Spot 67	209	40503	0.6	17.1368	1.2	0.7000	1.8	0.0870	1.2	0.71	538.0	6.4	538.8	7.3	542.1	27.1	538.0	6.4
-354-U1451A-41F Spot 92	314	10940	1.0	17.2601	1.5	0.7001	1.9	0.0877	1.2	0.65	541.8	6.5	538.9	8.0	526.4	32.1	541.8	6.5
-354-U1451A-41F Spot 108	524	24099	2.2	16.9017	1.2	0.7291	1.7	0.0894	1.2	0.70	552.1	6.4	556.0	7.4	572.2	27.1	552.1	6.4
-354-U1451A-41F Spot 105	919	26339	2.3	17.1477	1.4	0.7226	1.8	0.0899	1.0	0.58	555.0	5.5	552.2	7.6	540.7	31.4	555.0	5.5
-354-U1451A-41F Spot 230	119	42139	160.4	16.6581	2.2	0.7452	2.5	0.0901	1.3	0.52	555.9	7.0	565.4	11.0	603.7	46.9	555.9	7.0
-354-U1451A-41F Spot 35	985	1828264	6.7	16.2290	1.2	0.7808	3.0	0.0919	2.7	0.91	567.0	14.8	586.0	13.3	659.9	26.4	567.0	14.8
-354-U1451A-41F Spot 179	272	52976	2.9	16.3138	2.1	0.8183	3.0	0.0969	2.1	0.70	596.0	11.8	607.1	13.5	648.8	45.0	596.0	11.8
-354-U1451A-41F Spot 266	361	36559	1.0	16.5517	1.2	0.8146	1.6	0.0978	1.1	0.70	601.7	6.5	605.0	7.4	617.6	25.0	601.7	6.5
-354-U1451A-41F Spot 157	185	75112	3.2	16.0520	1.3	0.8493	1.7	0.0989	1.1	0.67	608.1	6.6	624.3	7.9	683.4	26.8	608.1	6.6
-354-U1451A-41F Spot 188	211	99902	0.8	16.2420	1.2	0.8530	1.6	0.1005	1.1	0.69	617.5	6.7	626.3	7.7	658.3	25.4	617.5	6.7
-354-U1451A-41F Spot 256	872	112028	3.2	15.2475	0.8	0.9448	2.0	0.1045	1.8	0.92	640.9	11.0	675.4	9.7	792.2	16.2	640.9	11.0
-354-U1451A-41F Spot 46	700	50048	6.0	16.3941	0.7	0.8854	1.2	0.1053	0.9	0.77	645.5	5.5	643.9	5.5	638.2	16.0	645.5	5.5
-354-U1451A-41F Spot 156	362	28613	16.6	16.3396	1.0	0.8929	1.6	0.1059	1.2	0.79	648.7	7.6	647.9	7.5	645.4	20.5	648.7	7.6
-354-U1451A-41F Spot 153	1657	571599	5.0	16.0643	0.6	0.9267	1.2	0.1080	1.0	0.84	661.2	6.3	665.9	5.8	681.8	13.6	661.2	6.3
-354-U1451A-41F Spot 121	707	103915	3.3	16.1533	1.2	0.9222	2.2	0.1081	1.9	0.85	661.7	11.8	663.5	10.8	669.9	24.7	661.7	11.8
-354-U1451A-41F Spot 142	1539	4231685	7.8	16.3269	0.7	0.9346	1.5	0.1107	1.4	0.88	676.9	8.8	670.0	7.6	647.1	15.6	676.9	8.8
-354-U1451A-41F Spot 277	1089	109071	9.2	15.6633	1.1	0.9907	2.3	0.1126	2.0	0.88	687.8	13.3	699.1	11.7	735.6	23.3	687.8	13.3
-354-U1451A-41F Spot 86	1314	2127351	1.7	15.2633	0.9	1.0711	1.8	0.1186	1.5	0.85	722.7	10.2	739.3	9.3	790.0	19.5	722.7	10.2
-354-U1451A-41F Spot 129	318	405883	12.0	15.0357	0.9	1.1205	1.6	0.1222	1.3	0.82	743.4	9.2	763.2	8.5	821.5	19.0	743.4	9.2
-354-U1451A-41F Spot 8	1295	1950068	9.9	14.5506	0.8	1.1631	2.7	0.1228	2.6	0.96	746.6	18.4	783.4	14.9	889.6	16.6	746.6	18.4
-354-U1451A-41F Spot 206	2902	1068498	2.2	15.0143	1.0	1.1316	2.7	0.1233	2.5	0.93	749.4	18.0	768.5	14.8	824.5	21.5	749.4	18.0
-354-U1451A-41F Spot 85	444	101523	3.8	14.8893	1.1	1.1427	1.4	0.1235	0.9	0.62	750.4	6.2	773.8	7.6	841.9	22.9	750.4	6.2
-354-U1451A-41F Spot 60	1306	157575	5.7	14.4239	0.7	1.1885	1.7	0.1244	1.6	0.90	755.7	11.1	795.3	9.5	907.6	15.4	755.7	11.1
-354-U1451A-41F Spot 292	235	10708	6.3	14.6178	1.4	1.1990	2.8	0.1272	2.4	0.86	771.7	17.3	800.1	15.4	880.1	29.8	771.7	17.3
-354-U1451A-41F Spot 47	436	60498	2.6	15.2254	0.7	1.1533	1.2	0.1274	0.9	0.78	773.0	6.5	778.8	6.3	795.3	15.3	773.0	6.5
-354-U1451A-41F Spot 128	1315	81509	21.7	15.4588	0.8	1.1483	1.6	0.1288	1.4	0.87	781.0	10.1	776.4	8.6	763.3	16.5	781.0	10.1
-354-U1451A-41F Spot 271	68	36928	1.6	14.3265	1.4	1.2392	1.7	0.1288	1.0	0.59	781.1	7.4	818.5	9.7	921.6	28.7	781.1	7.4
-354-U1451A-41F Spot 43	49	7652	27.6	15.5078	1.8	1.1499	2.6	0.1294	1.9	0.73	784.4	14.3	777.2	14.3	756.6	38.0	784.4	14.3
-354-U1451A-41F Spot 58	77	7673	2.1	15.5670	1.6	1.1457	1.9	0.1294	1.2	0.59	784.5	8.5	775.2	10.5	748.6	33.0	784.5	8.5
-354-U1451A-41F Spot 242	115	9535	0.9	15.1973	1.2	1.1807	1.8	0.1302	1.4	0.75	789.0	10.2	791.7	10.1	799.1	25.4	789.0	10.2
-354-U1451A-41F Spot 111	249	78711	3.4	14.3784	1.8	1.2486	3.3	0.1303	2.8	0.85	789.4	21.1	822.8	18.8	914.2	36.2	789.4	21.1
-354-U1451A-41F Spot 68	650	142865	2.3	14.7127	0.9	1.2240	1.8	0.1307	1.5	0.86	791.7	11.5	811.6	10.0	866.7	18.8	791.7	11.5
-354-U1451A-41F Spot 289	315	62501	8.3	14.1927	1.1	1.2745	1.8	0.1312	1.4	0.80	795.0	10.8	834.4	10.3	940.9	22.4	795.0	10.8
-354-U1451A-41F Spot 220	572	29956	0.9	15.2447	1.0	1.1890	1.4	0.1315	1.0	0.71	796.5	7.7	795.5	8.0	792.6	21.3	796.5	7.7
-354-U1451A-41F Spot 257	128	20325	1.0	14.6116	0.9	1.2421	1.4	0.1317	1.1	0.76	797.5	8.1	819.8	8.0	881.0	19.1	797.5	8.1
-354-U1451A-41F Spot 299	2080	107590	3.9	14.0757	0.8	1.2972	1.3	0.1325	0.9	0.75	802.0	7.1	844.5	7.3	957.8	17.3	802.0	7.1
-354-U1451A-41F Spot 254	62	7797	2.4	14.8979	2.2	1.2261	2.5	0.1325	1.3	0.50	802.3	9.6	812.6	14.2	840.7	45.8	802.3	9.6
-354-U1451A-41F Spot 138	148	20388	1.3	14.8913	1.4	1.2306	1.7	0.1330	1.0	0.57	804.7	7.5	814.6	9.8	841.7	29.8	804.7	7.5
-354-U1451A-41F Spot 201	175	28689	2.0	15.4221	1.1	1.1902	1.6	0.1332	1.1	0.73	806.0	8.7	796.1	8.6	768.3	22.3	806.0	8.7
-354-U1451A-41F Spot 127	1455	470718	1.3	14.5386	1.2	1.2629	2.6	0.1332	2.3	0.89	806.3	17.4	829.2	14.6	891.3	23.8	806.3	17.4
-354-U1451A-41F Spot 252	319	24308	15.4	15.0076	1.1	1.2271	1.8	0.1336	1.4	0.77	808.5	10.5	813.0	10.0	825.4	23.5	808.5	10.5
-354-U1451A-41F Spot 196	929	640580	1.4	14.8360	1.0	1.2491	1.6	0.1345	1.2	0.75	813.3	9.0	823.0	8.9	849.3	21.6	813.3	9.0
-354-U1451A-41F Spot 61	339	33773	2.4	15.0353	1.1	1.2399	1.7	0.1353	1.3	0.77	817.8	10.1	818.8	9.6	821.6	22.6	817.8	10.1
-354-U1451A-41F Spot 30	394	213368	4.7	14.6826	1.0	1.2782	1.5	0.1362	1.2	0.75	823.0	8.9	836.1	8.8	871.0	21.2	823.0	8.9
-354-U1451A-41F Spot 176	422	22113	2.4	14.5295	1.1	1.2929	1.8	0.1363	1.4	0.78	823.7	10.8	842.6	10.2	892.6	23.3	823.7	10.8
-354-U1451A-41F Spot 243	216	23832	3.6	15.1236	1.3	1.2541	2.5	0.1376	2.2	0.86	831.2	17.0	825.3	14.3	809.3	27.0	831.2	17.0
-354-U1451A-41F Spot 52	456	14308	1.9	14.8804	1.1	1.2777	1.5	0.1380	1.0	0.69	833.1	7.8	835.8	8.3	843.1	22.1	833.1	7.8
-354-U1451A-41F Spot 110	68	7050	2.8	15.0624	1.5	1.2668	1.9	0.1384	1.1	0.60	835.9	8.9	830.9	10.7	817.8	31.7	835.9	8.9
-354-U1451A-41F Spot 120	187	106108	1.2	14.7479	1.2	1.2944	1.8	0.1385	1.4	0.75	836.3	10.6	843.3	10.3	861.8	24.7	836.3	10.6
-354-U1451A-41F Spot 100	65	124058	2.1	14.3478	1.8	1.3462	2.1	0.1402	1.1	0.53	845.5	8.7	865.9	12.1	918.6	36.4	845.5	8.7
-354-U1451A-41F Spot 90	357	49866	1.2	14.8438	1.0	1.3056	1.7	0.1406	1.4	0.81	848.2	11.2	848.2	10.0	848.3	21.2	848.2	11.2

-354-U1451A-41F Spot 147	1236	96070	2.5	14.7551	0.8	1.3193	1.8	0.1412	1.7	0.91	851.7	13.3	854.2	10.6	860.7	16.1	851.7	13.3
-354-U1451A-41F Spot 139	220	23811	1.0	14.2278	1.0	1.3689	4.0	0.1413	3.8	0.97	852.1	30.7	875.7	23.4	935.8	21.4	852.1	30.7
-354-U1451A-41F Spot 155	434	52914	18.5	14.5321	0.8	1.3606	1.5	0.1435	1.2	0.84	864.2	10.0	872.1	8.6	892.2	16.3	864.2	10.0
-354-U1451A-41F Spot 39	3404	2914131	13.6	14.9644	0.9	1.3333	1.4	0.1448	1.0	0.73	871.6	8.2	860.3	8.0	831.4	19.7	871.6	8.2
-354-U1451A-41F Spot 6	374	34522	2.1	14.7914	1.2	1.3582	1.4	0.1458	0.8	0.55	877.2	6.3	871.1	8.2	855.6	24.2	877.2	6.3
-354-U1451A-41F Spot 80	545	42391	7.7	14.4421	1.0	1.3972	1.7	0.1464	1.4	0.80	880.8	11.4	887.8	10.2	905.0	21.3	880.8	11.4
-354-U1451A-41F Spot 41	525	167174	4.2	14.5960	0.9	1.3898	1.4	0.1472	1.0	0.73	885.2	8.1	884.6	8.0	883.2	19.2	885.2	8.1
-354-U1451A-41F Spot 167	715	78796	1.5	14.4452	0.8	1.4112	1.5	0.1479	1.2	0.85	889.2	10.2	893.7	8.6	904.6	15.9	889.2	10.2
-354-U1451A-41F Spot 22	445	104692	1.9	14.4618	1.1	1.4114	1.4	0.1481	0.9	0.66	890.3	7.9	893.8	8.6	902.2	22.4	890.3	7.9
-354-U1451A-41F Spot 247	1079	78712	23.4	14.6989	1.2	1.4098	1.8	0.1504	1.3	0.75	903.0	11.1	893.1	10.5	868.7	24.3	903.0	11.1
-354-U1451A-41F Spot 198	197	18339	1.6	14.5684	0.9	1.4276	1.4	0.1509	1.0	0.76	906.0	8.9	900.5	8.3	887.1	18.6	906.0	8.9
-354-U1451A-41F Spot 93	673	221529	8.7	14.4193	0.7	1.5196	1.4	0.1590	1.2	0.86	951.2	10.5	938.3	8.4	908.3	14.3	908.3	14.3
-354-U1451A-41F Spot 294	519	73660	4.8	14.4168	0.9	1.4185	1.4	0.1484	1.0	0.76	891.9	8.6	896.7	8.1	908.7	18.2	908.7	18.2
-354-U1451A-41F Spot 103	918	96981	7.7	14.4131	0.6	1.4687	1.3	0.1536	1.2	0.88	921.1	10.0	917.6	8.0	909.2	12.9	909.2	12.9
-354-U1451A-41F Spot 184	115	17326	1.3	14.4063	1.3	1.4800	1.9	0.1547	1.3	0.72	927.3	11.6	922.2	11.2	910.2	26.4	910.2	26.4
-354-U1451A-41F Spot 81	138	100749	2.2	14.3450	1.1	1.4127	1.5	0.1470	1.0	0.69	884.4	8.5	894.3	8.9	919.0	22.6	919.0	22.6
-354-U1451A-41F Spot 203	539	38986	1.5	14.3107	0.9	1.5423	1.2	0.1601	0.9	0.72	957.6	7.9	947.4	7.7	923.9	17.9	923.9	17.9
-354-U1451A-41F Spot 170	408	49984	1.9	14.2935	0.8	1.4508	1.4	0.1505	1.1	0.79	903.6	9.1	910.2	8.2	926.4	17.2	926.4	17.2
-354-U1451A-41F Spot 12	245	26735	0.8	14.2909	0.8	1.5374	1.3	0.1594	0.9	0.75	953.6	8.3	945.5	7.7	926.7	17.1	926.7	17.1
-354-U1451A-41F Spot 118	146	37685	2.7	14.2654	1.0	1.4089	1.6	0.1458	1.2	0.75	877.6	9.5	892.7	9.2	930.4	21.0	930.4	21.0
-354-U1451A-41F Spot 204	712	92247	74.3	14.2443	0.7	1.5611	1.5	0.1614	1.3	0.89	964.3	11.6	954.9	9.0	933.4	13.4	933.4	13.4
-354-U1451A-41F Spot 205	346	91452	0.7	14.2006	1.0	1.5778	1.5	0.1626	1.0	0.71	971.0	9.4	961.5	9.2	939.7	21.3	939.7	21.3
-354-U1451A-41F Spot 84	3674	489884	83.8	14.1574	0.9	1.5265	1.6	0.1568	1.3	0.82	939.0	11.2	941.1	9.7	945.9	18.6	945.9	18.6
-354-U1451A-41F Spot 55	323	39650	0.9	14.1225	0.9	1.5779	1.3	0.1617	1.0	0.75	966.1	9.0	961.5	8.3	951.0	18.1	951.0	18.1
-354-U1451A-41F Spot 280	365	33032	1.9	14.1052	1.0	1.4792	1.5	0.1514	1.2	0.77	908.7	10.2	921.9	9.4	953.5	20.1	953.5	20.1
-354-U1451A-41F Spot 306	1469	114879	10.7	14.0997	0.8	1.4626	1.3	0.1496	1.0	0.76	898.9	8.3	915.1	7.8	954.3	17.1	954.3	17.1
-354-U1451A-41F Spot 51	2924	265243	10.3	14.0505	0.8	1.5593	1.6	0.1590	1.3	0.86	951.0	11.8	954.2	9.6	961.5	16.5	961.5	16.5
-354-U1451A-41F Spot 11	831	101168	18.8	14.0197	0.9	1.3675	1.5	0.1391	1.3	0.82	839.6	9.9	875.1	9.0	965.9	17.7	965.9	17.7
-354-U1451A-41F Spot 59	333	42850	4.2	14.0193	0.8	1.5815	1.4	0.1609	1.1	0.82	961.6	10.0	962.9	8.5	966.0	15.8	966.0	15.8
-354-U1451A-41F Spot 95	273	27390	1.7	14.0110	0.9	1.6000	1.3	0.1627	1.0	0.73	971.5	8.8	970.2	8.4	967.2	18.7	967.2	18.7
-354-U1451A-41F Spot 171	155	95045	1.6	14.0058	1.2	1.5299	1.7	0.1555	1.2	0.70	931.6	10.1	942.4	10.2	968.0	24.0	968.0	24.0
-354-U1451A-41F Spot 244	947	103023	29.5	14.0043	0.9	1.4784	1.5	0.1502	1.2	0.78	902.2	9.8	921.6	9.1	968.2	19.0	968.2	19.0
-354-U1451A-41F Spot 258	1058	235199	85.5	14.0030	1.1	1.6025	2.4	0.1628	2.1	0.89	972.4	19.3	971.2	15.0	968.4	22.2	968.4	22.2
-354-U1451A-41F Spot 13	798	123216	1.4	14.0016	0.8	1.6507	1.7	0.1677	1.5	0.87	999.4	13.5	989.8	10.6	968.6	17.2	968.6	17.2
-354-U1451A-41F Spot 74	222	115141	2.4	13.9936	0.8	1.5725	1.3	0.1597	1.0	0.78	954.9	9.2	959.4	8.2	969.7	16.8	969.7	16.8
-354-U1451A-41F Spot 308	347	24677	1.6	13.9600	0.9	1.6193	1.6	0.1640	1.4	0.85	979.1	12.6	977.7	10.3	974.7	17.5	974.7	17.5
-354-U1451A-41F Spot 200	338	6077126	5.5	13.9597	1.0	1.6000	1.3	0.1621	0.8	0.59	968.2	6.8	970.2	7.9	974.7	20.9	974.7	20.9
-354-U1451A-41F Spot 261	1606	345777	1.2	13.9529	0.7	1.6163	1.3	0.1636	1.1	0.85	976.9	10.4	976.5	8.5	975.7	14.5	975.7	14.5
-354-U1451A-41F Spot 276	743	224531	3.1	13.9526	0.6	1.6217	1.4	0.1642	1.2	0.89	980.0	11.2	978.7	8.6	975.7	12.6	975.7	12.6
-354-U1451A-41F Spot 27	1709	99959	2.3	13.9328	0.9	1.4225	1.7	0.1438	1.5	0.86	866.2	11.9	898.4	10.2	978.6	17.4	978.6	17.4
-354-U1451A-41F Spot 214	608	41465	1.8	13.9274	1.0	1.3624	2.9	0.1377	2.7	0.94	831.5	21.0	872.9	16.8	979.4	20.1	979.4	20.1
-354-U1451A-41F Spot 38	397	108353	1.9	13.9250	1.0	1.6027	1.5	0.1619	1.1	0.75	967.5	10.1	971.3	9.4	979.8	20.2	979.8	20.2
-354-U1451A-41F Spot 40	419	74059	2.8	13.9001	0.9	1.6004	1.5	0.1614	1.2	0.79	964.6	10.8	970.4	9.5	983.4	18.7	983.4	18.7
-354-U1451A-41F Spot 293	627	105877	2.0	13.8546	1.1	1.6751	1.5	0.1684	1.0	0.65	1003.3	8.9	999.1	9.3	990.1	22.5	990.1	22.5
-354-U1451A-41F Spot 166	321	41259	2.6	13.8214	1.4	1.6268	1.8	0.1631	1.1	0.62	974.2	10.2	980.6	11.3	995.0	28.7	995.0	28.7
-354-U1451A-41F Spot 33	211	67214	2.1	13.8095	0.9	1.6984	1.5	0.1702	1.1	0.77	1013.1	10.5	1007.9	9.3	996.7	18.7	996.7	18.7
-354-U1451A-41F Spot 56	1250	73195	4.7	13.7810	0.9	1.6289	1.5	0.1629	1.2	0.79	972.7	10.5	981.4	9.3	1000.9	18.4	1000.9	18.4
-354-U1451A-41F Spot 137	787	831376	1.1	13.7751	0.7	1.6099	1.1	0.1609	0.9	0.82	961.8	8.3	974.1	7.1	1001.8	13.3	1001.8	13.3
-354-U1451A-41F Spot 311	271	48020	2.7	13.7724	2.5	1.4421	2.7	0.1441	1.0	0.38	867.8	8.3	906.6	16.2	1002.2	50.8	1002.2	50.8
-354-U1451A-41F Spot 272	197	56193	3.8	13.7541	1.2	1.5635	1.8	0.1560	1.3	0.74	934.7	11.4	955.9	10.9	1004.9	24.0	1004.9	24.0
-354-U1451A-41F Spot 245	1020	319863	8.3	13.7423	0.9	1.6833	1.6	0.1678	1.3	0.83	1000.2	12.3	1002.2	10.2	1006.6	17.8	1006.6	17.8
-354-U1451A-41F Spot 228	174	87916	2.2	13.6823	1.2	1.6058	1.6	0.1594	1.0	0.62	953.6	8.6	972.5	9.8	1015.5	24.7	1015.5	24.7
-354-U1451A-41F Spot 66	189	93399	2.1	13.6727	1.1	1.4754	1.5	0.1464	1.0	0.68	880.6	8.5	920.3	9.1	1016.9	22.4	1016.9	22.4
-354-U1451A-41F Spot 223	898	15399502	14.5	13.6121	0.9	1.7413	1.4	0.1720	1.0	0.75	1023.0	9.7	1023.9	8.9	1025.9	18.6	1025.9	18.6
-354-U1451A-41F Spot 197	137	26719	3.9	13.5752	1.0	1.8045	1.5	0.1777	1.2	0.77	1054.6	11.5	1047.1	10.1	1031.4	20.0	1031.4	20.0
-354-U1451A-41F Spot 295	296	84477	4.3	13.5441	0.8	1.7286	1.3	0.1699	1.0	0.77	1011.5	9.4	1019.2	8.4	1036.0	16.9	1036.0	16.9
-354-U1451A-41F Spot 65	703	379085	1.7	13.5045	1.0	1.7615	1.5	0.1726	1.2	0.78	1026.4	11.4	1031.4	9.9	1041.9	19.3	1041.9	19.3
-354-U1451A-41F Spot 32	687	2495756	2.0	13.3599	1.1	1.7064	2.5	0.1654	2.3	0.90	986.8	20.7	1010.9	16.0	1063.6	21.4	1063.6	21.4
-354-U1451A-41F Spot 287	498	91120	2.1	13.3426	0.9	1.7289	1.6	0.1674	1.3	0.83	997.7	12.0	1019.3	10.0	1066.2	17.2	1066.2	17.2
-354-U1451A-41F Spot 297	105	287416	1.4	13.3389	1.3	1.6666	1.9	0.1613	1.4	0.73	964.0	12.5	995.9	12.1	1066.8	26.0	1066.8	26.0
-354-U1451A-41F Spot 259	484	107492	2.1	13.3298	0.8	1.8611	1.2	0.1800	0.9	0.76	1067.0	8.7	1067.4	7.6	1068.2	15.1	1068.2	15.1
-354-U1451A-41F Spot 54	291	141578	6.1	13.2773	1.1	1.8689	1.5	0.1800	1.0	0.65	1067.2	9.7	1070.2	10.0	1076.1	23.1	1076.1	23.1
-354-U1451A-41F Spot 64	634	59977	10.6	13.2565	1.1	1.9162	1.5	0.1843	1.1	0.69	1090.5	10.6	1086.7	10.2	1079.2	22.3	1079.2	22.3
-354-U1451A-41F Spot 75	678	45154	2.1	13.2521	1.1	1.6744	1.9	0.1610	1.5	0.81	962.3	13.8	998.9	12.2	1079.9	22.7	1079.9	22.7
-354-U1451A-41F Spot 69	850	118669	2.0	13.2390	1.2	1.7188	1.8	0.1651	1.3	0.75	985.1	12.1	1015.6	11.3	1081.9	23.2	1081.9	23.2
-354-U1451A-41F Spot 181	1159	205220	4.8	13.1716	0.7	1.8823	1.2	0.1799	1.0	0.80	1066.4	9.7	1074.9	8.2	1092.1	14.7	1092.1	14.7
-354-U1451A-41F Spot 158	523	47979	2.6	13.1603	0.9	1.9033	2.1	0.1817	1.9	0.91	1076.5	19.3	1082.3	14.3	1093.8	18.3	1093.8	18.3
-354-U1451A-41F Spot 231	299	115664	0.7	13.0856	1.1	1.8915	2.0	0.1796	1.7	0.85	1064.7	16.5	1078.1	13.2	1105.2	21.1	1105.2	21.1
-354-U1451A-41F Spot 300	3179	356427	15.2	13.0182	0.7	1.6745	1.5	0.1582	1.3	0.88	946.6	11.5	998.9	9.4	1115.6	14.3	1115.6	14.3
-354-U1451A-41F Spot 109	496	54412	1.2	12.9609	0.9	2.0062	1.5	0.1887	1.2	0.81	1114.1	12.3	1117.6	10.0	1124.3	17.3	1124.3	17.3
-354-U1451A-41F Spot 240	242	58842	1.9	12.9583	0.9	2.0796	1.4	0.1955	1.1	0.79	1151.3	11.6	1142.1	9.6	1124.7	17.3	1124.7	17.3
-354-U1451A-41F Spot 89	1222	430764	82.2	12.9441	0.7	1.9895	1.4	0.1869	1.3	0.89	1104.3	12.9	1112.0	9.7	1126.9	13.3	1126.9	13.3
-354-U1451A-41F Spot 107	1187	158516	2.5	12.8338	0.8	1.8796	2.8	0.1750	2.7	0.96	1039.7	26.0	1073.9	18.7	1143.9	15.6	1143.9	15.6
-354-U1451A-41F Spot 151	1495	574535	17.9	12.8250	0.8	2.0563	1.6	0.1914	1.4	0.88	1128.7	14.8	1134.4	11.1	1145.3	15.3	1145.3	15.3
-354-U1451A-41F Spot 104	1537	506428	1.9	12.8213	0.6	2.1193	1.4	0.1972	1.2	0.89	1160.0	13.1	1155.1	9.6	1145.9	12.6	1145.9	12.6
-354-U1451A-41F Spot 91	758	70519	1.4	12.7669	0.9	2.0532	1.5	0.1902	1.2	0.80	1122.4	12.0	1133.4	9.9	1154.3	17.3	1154.3	17.3
-354-U1451A-41F Spot 191	303	80797	2.5	12.7625	0.8	2.1092	1.4	0.1953	1.1	0.80	1150.1	12.0	1151.8	9.8	1155.0	16.8	1155.0	16.8
-354-U1451A-41F Spot 283	492	102638	2.1	12.6790	0.9	2.1936	1.7	0.2018	1.4	0.84	1185.0	15.2	1179.0	11.7	1168.0	18.3	1168.0	18.3
-354-U1451A-41F Spot 29	498	35840	0.9	12.6436	0.7	2.1217	1.7	0.1946	1.5	0.91	1146.5	16.2	1155.9	11.7	1173.6	14.0	1173.6	14.0
-354-U1451A-41F Spot 307	199	220210	0.9	12.5481	0.8	2.0650	1.2	0.1880	0.9	0.78	1110.6	9.7	1137.3	8.3	1188.5	14.9	1188.5	14.9
-354-U1451A-41F Spot 250	120	37349	1.6	12.4898	0.9	2.2319	1.5	0.2023	1.1	0.76	1187.5	12.0	1191.1	10.2	1197.8	18.7	1197.8	18.7
-354-U1451A-41F Spot 87	196	75087	1.8	12.4316	0.7	2.1415	1.4	0.1932	1.2	0.84	1138.5	12.3	1162.3	9.6	1206.9	14.6	1206.9	14.6
-354-U1451A-41F Spot 284	328	73297	2.1	12.3629	0.9	2.0959	1.6	0.1880	1.2	0.80	1110.6	12.7	1147.5	10.7	1217.9	18.5	1217.9	18.5
-354-U1451A-41F Spot 77	422	70947	3.6	12.2263	0.9	2.3907	1.3	0.2121	1.0	0.73	1239.9	10.8	1239.8	9.4	1239.7	17.6	1239.7	17.6
-354-U1451A-41F Spot 20	274	44178	2.3	12.1387	0.8	2.4654	1.6	0.2171	1.4	0.87	1266.8	16.0	1261.9	11.6	1253.8	15.7	1253.8	15.7
-354-U1451A-41F Spot 159	96	54071	1.8	12.0883	1.3	2.3972	1.9	0.2103	1.4	0.73	1230.2	15.5	1241.8	13.5	1261.9	25.1	1261.9	25.1
-354-U1451A-41F Spot 235	199	99886	1.9	11.9708	1.1	2.4597	1.5	0.2136	1.1	0.71	1248.2	12.5	1260.3	11.1	1280.9	21.1	1280.9	21.1
-354-U1451A-41F Spot 53	366	245053	1.8	11.9387	0.8	2.5269	1.5	0.2189	1.2	0.83	1276.0	14.3	1279.8	10.9	1286.1	16.3	1286.1	16.3
-354-U1451A-41F Spot 229	563	54709	78.0	11.8793	1.2	2.4177	1.8	0.2084	1.4	0.74	1220.3	15.1	1247.9	13.1	1295.9	23.7	1295.9	23.7
-354-U1451A-41F Spot 25	326	90555	2.9	11.6354	0.8	2.7435	1.4	0.2316	1.1	0.80	1343.0	13.8	1340.3	10.6	1336.1	16.4	1336.1	16.4
-354-U1451A-41F Spot 144	2765	3615307	13.7	11.6218	1.2	2.5482	1.9	0.2149	1.4	0.74	1254.8	15.9	1285.9	13.7	1338.3	24.2	1338.3	24.2
-354-U1451A-41F Spot 178	329	59632	89.4	11.4974	1.0	2.7857	1.6	0.2324	1.2	0.76	1347.0	14.4	1351.7	11.6	1359.1	19.3	1359.1	19.3
-354-U1451A-41F Spot 44	428	94784	8.7	11.3851	1.8	2.7488	2.7	0.2271	2.0	0.75	1319.2	24.4	1341.8	20.2	1378.0	34.2	1378.0	34.2
-354-U1451A-41F Spot 309	244	46708	2.9	11.2661	1.0	2.9549	1.6	0.2416	1.3	0.80	1394.8	16.3	1396.1	12.4	1398.2	18.8	1398.2	18.8
-354-U1451A-41F Spot 251	264	271705	2.7	11.1806	0.9	3.0558	1.4	0.2479	1.1	0.79	1427.7	14.1	1421.7	10.7	1412.8	16.4	1412.8	16.4
-354-U1451A-41F Spot 152	547	207612	3.4	11.1344	1.5	2.7807	2.7	0.2247	2.2	0.82	1306.4	25.7	1350.4	19.8	1420.7	29.0	1420.7	29.0
-354-U1451A-41F Spot 3	448	93602	2.4	11.0938	0.7	3.0929	1.2	0.2490	1.0	0.83	1433.1	13.1	1430.9	9.5	1427.7	13.2	1427.7	13.2
-354-U1451A-41F Spot 31	1747	473468	7.0	10.9793	0.8	2.9634	1.4	0.2361	1.2	0.81	1366.3	14.3	1398.3	10.8	1447.4	15.8	1447.4	15.8
-354-U1451A-41F Spot 285	450	30251	3.6	10.9791	1.9	2.5772	2.3	0.2053	1.3	0.54	1203.8	13.8	1294.2	17.0	1447.5	37.1	1447.5	37.1
-354-U1451A-41F Spot 114	486	807236	1.7	10.9205	1.0	3.1237	1.7	0.2475	1.4	0.80	1425.6	17.3	1438.5	13.0	1457.7	19.1	1457.7	19.1
-354-U1451A-41F Spot 168	785	106751	2.4	10.8455	0.8	2.9474	1.6	0.2319	1.4	0.87	1344.6	16.8	1394.2	12.1	1470.8	15.0	1470.8	15.0

-354-U1451A-41F Spot 49	1547	115706	1.8	10.7956	0.7	3.0838	1.7	0.2416	1.5	0.91	1394.8	19.3	1428.7	13.1	1479.5	13.7	1479.5	13.7
-354-U1451A-41F Spot 313	537	86471	1.8	10.6334	1.9	2.7926	2.5	0.2155	1.5	0.61	1257.8	17.2	1353.5	18.4	1508.1	36.7	1508.1	36.7
-354-U1451A-41F Spot 248	1150	96732	2.9	10.4909	0.7	3.0806	1.9	0.2345	1.8	0.93	1358.0	21.7	1427.9	14.6	1533.6	13.3	1533.6	13.3
-354-U1451A-41F Spot 150	1191	169282	7.7	10.4162	0.7	3.5225	1.8	0.2662	1.7	0.92	1521.6	22.8	1532.3	14.4	1547.0	13.1	1547.0	13.1
-354-U1451A-41F Spot 70	1138	133657	1.6	10.2441	0.9	3.5781	1.7	0.2660	1.5	0.85	1520.3	20.0	1544.7	13.8	1578.3	17.3	1578.3	17.3
-354-U1451A-41F Spot 183	314	102316	2.2	10.2437	0.9	3.1600	2.2	0.2349	2.0	0.91	1360.0	24.7	1447.4	17.2	1578.3	17.6	1578.3	17.6
-354-U1451A-41F Spot 233	150	24198	1.5	10.2366	1.0	3.2285	2.1	0.2398	1.8	0.87	1385.6	22.5	1464.0	16.0	1579.6	18.9	1579.6	18.9
-354-U1451A-41F Spot 269	422	699902	2.0	10.1717	0.9	3.7477	1.5	0.2766	1.2	0.81	1574.2	16.8	1581.6	11.8	1591.5	16.0	1591.5	16.0
-354-U1451A-41F Spot 48	1047	107459	3.9	10.0586	0.9	3.4603	1.4	0.2525	1.2	0.80	1451.6	15.1	1518.2	11.3	1612.4	15.9	1612.4	15.9
-354-U1451A-41F Spot 207	595	111644	33.5	9.7152	0.6	4.3068	1.1	0.3036	0.9	0.86	1709.1	14.1	1694.7	9.0	1676.8	10.4	1676.8	10.4
-354-U1451A-41F Spot 146	560	989245	1.7	9.6137	0.9	4.3267	1.4	0.3018	1.1	0.78	1700.3	16.7	1698.5	11.8	1696.2	16.4	1696.2	16.4
-354-U1451A-41F Spot 260	372	122760	2.4	9.6069	0.7	4.2699	1.3	0.2976	1.0	0.81	1679.6	15.4	1687.6	10.5	1697.5	13.7	1697.5	13.7
-354-U1451A-41F Spot 97	507	139898	2.3	9.5873	0.8	4.1238	1.4	0.2869	1.1	0.79	1625.8	15.6	1659.0	11.3	1701.3	15.6	1701.3	15.6
-354-U1451A-41F Spot 130	175	151903	3.1	9.5554	1.1	3.9056	1.8	0.2708	1.5	0.80	1544.8	20.3	1614.8	14.9	1707.4	20.3	1707.4	20.3
-354-U1451A-41F Spot 193	721	12088	1.1	9.5431	2.6	3.5385	3.1	0.2450	1.6	0.53	1412.7	20.8	1535.9	24.6	1709.8	48.6	1709.8	48.6
-354-U1451A-41F Spot 21	1466	150817	1.6	9.4336	1.0	3.9968	2.5	0.2736	2.3	0.92	1558.9	32.0	1633.5	20.5	1731.0	18.7	1731.0	18.7
-354-U1451A-41F Spot 172	339	327146	2.0	9.4249	0.9	4.5809	1.2	0.3133	0.9	0.71	1756.8	13.1	1745.8	10.1	1732.7	15.8	1732.7	15.8
-354-U1451A-41F Spot 165	164	84374	3.3	9.4150	0.9	4.7331	1.4	0.3233	1.0	0.72	1806.0	15.6	1773.1	11.5	1734.6	17.4	1734.6	17.4
-354-U1451A-41F Spot 98	543	946959	1.0	9.4104	0.8	4.3625	1.2	0.2979	0.9	0.74	1680.8	13.6	1705.3	10.2	1735.5	15.3	1735.5	15.3
-354-U1451A-41F Spot 132	462	496538	2.8	9.3951	1.0	4.4230	1.7	0.3015	1.4	0.81	1698.8	20.3	1716.7	13.9	1738.5	18.1	1738.5	18.1
-354-U1451A-41F Spot 9	311	87550	3.8	9.3189	0.7	4.5705	1.8	0.3090	1.7	0.92	1736.0	25.8	1743.9	15.4	1753.4	13.5	1753.4	13.5
-354-U1451A-41F Spot 116	377	224102	1.2	9.1151	1.0	4.7155	2.8	0.3119	2.6	0.93	1749.9	39.9	1770.0	23.4	1793.7	18.6	1793.7	18.6
-354-U1451A-41F Spot 62	543	113980	1.1	9.0775	0.6	4.3624	1.5	0.2873	1.3	0.91	1628.2	19.2	1705.2	12.0	1801.3	10.7	1801.3	10.7
-354-U1451A-41F Spot 302	1052	199947	2.5	9.0618	0.9	4.4342	2.3	0.2916	2.1	0.91	1649.3	30.8	1718.8	19.2	1804.4	17.1	1804.4	17.1
-354-U1451A-41F Spot 173	271	141000	4.0	9.0344	0.8	4.9475	1.5	0.3243	1.3	0.87	1810.8	21.2	1810.4	13.0	1809.9	13.9	1809.9	13.9
-354-U1451A-41F Spot 16	4518	6273446	28.8	9.0267	0.8	4.3784	1.4	0.2868	1.1	0.81	1625.4	15.9	1708.3	11.2	1811.5	14.4	1811.5	14.4
-354-U1451A-41F Spot 124	157	43090	2.3	8.9857	0.9	5.1070	1.3	0.3330	1.0	0.75	1852.8	15.6	1837.3	11.0	1819.8	15.5	1819.8	15.5
-354-U1451A-41F Spot 131	2031	547057	57.6	8.9637	1.0	4.0576	1.9	0.2639	1.6	0.84	1509.8	21.5	1645.8	15.4	1824.2	18.5	1824.2	18.5
-354-U1451A-41F Spot 57	445	112135	3.5	8.9384	0.8	5.1495	1.5	0.3340	1.3	0.85	1857.6	20.3	1844.3	12.6	1829.3	14.3	1829.3	14.3
-354-U1451A-41F Spot 5	393	20147	2.1	8.7976	1.0	4.6165	1.7	0.2947	1.3	0.81	1664.9	19.7	1752.3	13.8	1858.0	17.6	1858.0	17.6
-354-U1451A-41F Spot 154	166	4217	1.0	8.7889	3.4	4.6235	5.8	0.2948	4.7	0.81	1665.7	69.0	1753.5	48.7	1859.8	62.2	1859.8	62.2
-354-U1451A-41F Spot 50	610	312748	11.1	8.7438	0.8	5.3186	1.3	0.3374	1.0	0.78	1874.3	16.2	1871.9	10.9	1869.1	14.3	1869.1	14.3
-354-U1451A-41F Spot 42	616	9369927	1.1	8.5776	0.9	5.6327	1.6	0.3506	1.3	0.83	1937.3	21.6	1921.1	13.4	1903.7	15.6	1903.7	15.6
-354-U1451A-41F Spot 78	247	228682	5.2	8.1728	0.8	6.1288	1.5	0.3634	1.3	0.83	1998.5	21.8	1994.4	13.3	1990.1	15.0	1990.1	15.0
-354-U1451A-41F Spot 186	215	267982	1.5	8.0981	0.6	5.8618	1.1	0.3444	0.9	0.83	1907.9	14.8	1955.6	9.4	2006.4	10.7	2006.4	10.7
-354-U1451A-41F Spot 119	819	116110	11.1	7.7798	0.7	6.2543	1.6	0.3530	1.4	0.91	1949.1	24.0	2012.1	13.8	2077.3	11.7	2077.3	11.7
-354-U1451A-41F Spot 222	197	8717	1.9	7.0299	1.6	7.6159	2.0	0.3885	1.1	0.58	2115.7	20.3	2186.7	17.5	2254.0	27.6	2254.0	27.6
-354-U1451A-41F Spot 225	128	57335	1.7	6.9081	0.8	8.9195	1.7	0.4471	1.5	0.87	2382.3	29.8	2329.8	15.6	2284.1	14.4	2284.1	14.4
-354-U1451A-41F Spot 202	4145	693738	1.1	6.8376	1.5	7.2965	2.7	0.3620	2.3	0.84	1991.6	39.2	2148.4	24.2	2301.8	25.1	2301.8	25.1
-354-U1451A-41F Spot 126	532	1624456	2.7	6.7396	0.8	8.7939	1.3	0.4300	1.1	0.80	2305.9	20.6	2316.9	12.2	2326.5	13.7	2326.5	13.7
-354-U1451A-41F Spot 73	454	459946	3.3	6.7252	2.2	8.3577	2.8	0.4078	1.6	0.59	2205.0	30.6	2270.6	25.2	2330.2	38.5	2330.2	38.5
-354-U1451A-41F Spot 106	146	59112	1.0	6.4686	0.9	8.2055	1.9	0.3851	1.7	0.89	2100.2	30.9	2254.0	17.5	2396.6	14.9	2396.6	14.9
-354-U1451A-41F Spot 210	669	168430	3.0	6.3595	0.7	9.1632	1.3	0.4228	1.1	0.84	2273.3	21.7	2354.4	12.4	2425.5	12.5	2425.5	12.5
-354-U1451A-41F Spot 301	366	88623	8.6	6.3442	0.8	9.1844	1.5	0.4228	1.2	0.86	2273.1	23.8	2356.6	13.3	2429.6	12.8	2429.6	12.8
-354-U1451A-41F Spot 37	947	183245	1.0	6.3043	0.8	9.8432	1.6	0.4503	1.4	0.89	2396.4	29.0	2420.2	15.1	2440.3	12.9	2440.3	12.9
-354-U1451A-41F Spot 79	417	281110	2.3	6.2872	0.8	8.3911	1.5	0.3828	1.3	0.83	2089.3	22.7	2274.2	13.8	2444.9	14.2	2444.9	14.2
-354-U1451A-41F Spot 209	313	98174	1.5	6.2760	0.9	8.5674	1.7	0.3901	1.5	0.86	2123.5	26.3	2293.1	15.4	2447.9	14.6	2447.9	14.6
-354-U1451A-41F Spot 221	1100	286653	12.9	6.2623	0.8	9.0176	1.7	0.4097	1.5	0.87	2213.8	27.9	2339.8	15.6	2451.6	14.1	2451.6	14.1
-354-U1451A-41F Spot 15	127	44031	0.9	6.1875	0.8	10.0355	1.5	0.4506	1.3	0.84	2397.7	25.1	2438.1	13.8	2471.9	13.8	2471.9	13.8
-354-U1451A-41F Spot 162	111	344022	1.2	6.1338	0.8	10.2381	1.4	0.4557	1.2	0.83	2420.3	24.0	2456.5	13.3	2486.6	13.5	2486.6	13.5
-354-U1451A-41F Spot 216	193	1218486	2.0	6.1245	0.8	8.5883	1.4	0.3817	1.1	0.80	2084.0	19.6	2295.3	12.6	2489.1	14.1	2489.1	14.1
-354-U1451A-41F Spot 180	303	106063	1.4	6.1045	0.8	11.2454	1.4	0.4981	1.1	0.81	2605.6	24.5	2543.7	13.2	2494.7	14.2	2494.7	14.2
-354-U1451A-41F Spot 208	1308	619326	15.3	6.0704	0.7	9.8847	1.2	0.4354	1.0	0.84	2329.9	20.1	2424.1	11.2	2504.1	11.0	2504.1	11.0
-354-U1451A-41F Spot 303	232	316890	1.1	6.0494	0.8	10.5048	1.4	0.4611	1.2	0.84	2444.4	24.1	2480.3	13.1	2509.9	13.1	2509.9	13.1
-354-U1451A-41F Spot 305	188	50344	0.7	6.0249	0.7	9.9202	1.4	0.4337	1.3	0.88	2322.3	24.5	2427.4	13.2	2516.7	11.4	2516.7	11.4
-354-U1451A-41F Spot 148	363	96877	2.7	5.9993	0.8	10.9161	1.3	0.4752	1.0	0.75	2506.2	19.9	2516.0	11.9	2523.9	14.2	2523.9	14.2
-354-U1451A-41F Spot 19	106	487490	1.9	5.9762	1.0	11.1109	1.5	0.4818	1.2	0.78	2535.1	24.7	2532.5	14.1	2530.4	16.0	2530.4	16.0
-354-U1451A-41F Spot 18	458	112296	2.8	5.7908	0.9	11.0153	1.4	0.4628	1.1	0.77	2452.0	22.0	2524.4	13.0	2583.1	14.8	2583.1	14.8
-354-U1451A-41F Spot 125	354	1351681	2.0	5.5071	0.9	12.2076	1.6	0.4878	1.4	0.85	2561.2	29.4	2620.5	15.3	2666.7	14.2	2666.7	14.2
-354-U1451A-41F Spot 164	371	96213	1.3	5.4805	0.9	11.5369	1.5	0.4588	1.2	0.81	2434.1	25.1	2567.6	14.2	2674.7	14.6	2674.7	14.6
-354-U1451A-41F Spot 10	338	135611	1.4	5.4468	0.8	12.2575	1.6	0.4844	1.4	0.85	2546.5	29.2	2624.3	15.3	2684.9	14.0	2684.9	14.0
-354-U1451A-41F Spot 270	145	227889	1.5	5.3480	0.8	12.8355	1.7	0.4981	1.5	0.86	2605.5	31.1	2667.7	15.8	2715.1	14.0	2715.1	14.0
-354-U1451A-41F Spot 174	140	48019	3.3	5.1702	1.0	13.4380	1.4	0.5041	1.0	0.69	2631.5	21.2	2710.9	13.5	2770.7	16.9	2770.7	16.9
-354-U1451A-41F Spot 135	73	40582	2.4	4.8789	0.7	14.8144	1.3	0.5244	1.1	0.86	2718.0	25.3	2803.4	12.6	2865.4	11.0	2865.4	11.0
-354-U1451A-41F Spot 253	314	714869	267.9	3.7933	0.8	23.6166	1.4	0.6500	1.1	0.81	3228.2	28.6	3252.7	13.6	3267.8	13.0	3267.8	13.0
-354-U1451A-41F Spot 17	830	374957	14.0	3.7157	0.9	20.6096	1.5	0.5556	1.2	0.80	2848.6	27.3	3120.4	14.4	3300.2	14.1	3300.2	14.1
-354-U1451A-41F Spot 182	754	852685	1.8	3.6621	0.9	24.8827	1.7	0.6612	1.4	0.83	3271.7	35.8	3303.6	16.4	3323.0	14.6	3323.0	14.6
-354-U1451A-41F Spot 94	153	1090179	2.1	3.5342	0.7	26.9745	1.4	0.6917	1.2	0.85	3389.1	31.3	3382.5	13.7	3378.6	11.5	3378.6	11.5

Table _____. U-Pb geochronologic analyses.																		
#18 U1451A_60F						Isotope ratios						Apparent ages (Ma)						
Late Miocene 7.5 Ma																		
Analysis	U	206Pb	U/Th	206Pb*	±	207Pb*	±	206Pb*	±	error	206Pb*	±	207Pb*	±	206Pb*	±	Best age	±
	(ppm)	204Pb		207Pb*	(%)	235U*	(%)	238U	(%)	corr.	238U*	(Ma)	235U	(Ma)	207Pb*	(Ma)	(Ma)	(Ma)
354U1451A-60F-100P #34 -Spot 35	1368	14722	2.3	21.4954	1.6	0.0163	2.0	0.0025	1.1	0.55	16.3	0.2	16.4	0.3	23.7	39.2	16.3	0.2
354U1451A-60F-100P #34 -Spot 174	477	2781	1.1	21.9350	3.2	0.0321	3.4	0.0051	1.2	0.35	32.8	0.4	32.1	1.1	NA	NA	32.8	0.4
354U1451A-60F-100P #34 -Spot 127	644	9480	3.8	21.9039	2.3	0.0364	2.6	0.0058	1.3	0.48	37.2	0.5	36.3	0.9	NA	NA	37.2	0.5
354U1451A-60F-100P #34 -Spot 50	270	2041	2.4	24.4608	1.7	0.0431	2.3	0.0076	1.5	0.67	49.1	0.7	42.8	1.0	NA	NA	49.1	0.7
354U1451A-60F-100P #34 -Spot 99	167	29622	1.1	21.4907	2.1	0.0494	2.4	0.0077	1.1	0.48	49.5	0.6	49.0	1.1	24.2	49.7	49.5	0.6
354U1451A-60F-100P #34 -Spot 305	43	7206	0.9	20.0984	3.7	0.0575	4.3	0.0084	2.2	0.50	53.8	1.2	56.7	2.4	182.5	87.2	53.8	1.2
354U1451A-60F-100P #34 -Spot 98	456	5423	2.0	22.3514	1.5	0.0557	2.1	0.0090	1.4	0.69	57.9	0.8	55.0	1.1	NA	NA	57.9	0.8
354U1451A-60F-100P #34 -Spot 255	101	2003	1.6	22.4614	4.9	0.0565	5.1	0.0092	1.5	0.28	59.1	0.9	55.8	2.8	NA	NA	59.1	0.9
354U1451A-60F-100P #34 -Spot 88	198	2999	59.3	17.3756	4.3	0.0753	6.0	0.0095	4.1	0.69	60.9	2.5	73.7	4.2	511.8	94.6	60.9	2.5
354U1451A-60F-100P #34 -Spot 314	365	37806	1.5	20.6359	1.6	0.0683	2.0	0.0102	1.1	0.56	65.6	0.7	67.1	1.3	120.7	38.3	65.6	0.7
354U1451A-60F-100P #34 -Spot 91	165	3628	1.0	21.7808	2.4	0.0810	2.7	0.0128	1.3	0.49	82.0	1.1	79.1	2.1	NA	NA	82.0	1.1
354U1451A-60F-100P #34 -Spot 214	1138	26808	1.5	19.9811	0.9	0.1127	1.2	0.0163	0.8	0.68	104.4	0.9	108.4	1.3	196.2	21.1	104.4	0.9
354U1451A-60F-100P #34 -Spot 276	598	12801	0.7	20.6995	0.9	0.1111	1.5	0.0167	1.2	0.81	106.7	1.3	107.0	1.5	113.5	20.8	106.7	1.3
354U1451A-60F-100P #34 -Spot 87	4039	90255	3.4	19.9056	1.6	0.1157	6.0	0.0167	5.8	0.97	106.8	6.1	111.1	6.3	205.0	36.0	106.8	6.1
354U1451A-60F-100P #34 -Spot 126	1138	68456	3.0	15.3766	3.6	0.2181	6.3	0.0243	5.2	0.82	155.0	8.0	200.4	11.5	774.5	75.6	155.0	8.0
354U1451A-60F-100P #34 -Spot 213	840	144117	7.5	17.7801	0.8	0.1950	2.9	0.0252	2.8	0.96	160.2	4.4	180.9	4.8	461.0	17.5	160.2	4.4
354U1451A-60F-100P #34 -Spot 148	270	75413	0.5	19.3717	1.1	0.1826	1.7	0.0257	1.4	0.78	163.4	2.2	170.3	2.7	267.7	25.1	163.4	2.2
354U1451A-60F-100P #34 -Spot 49	4557	1253872	49.1	18.2709	0.9	0.2268	4.9	0.0301	4.8	0.98	191.0	9.1	207.5	9.2	400.3	20.3	191.0	9.1
354U1451A-60F-100P #34 -Spot 46	1092	104211	3.5	17.3415	0.8	0.3776	2.8	0.0475	2.6	0.96	299.3	7.7	325.3	7.7	516.1	17.7	299.3	7.7
354U1451A-60F-100P #34 -Spot 195	1517	191102	3.5	17.1789	0.8	0.4365	1.6	0.0544	1.4	0.86	341.6	4.5	367.8	4.9	536.8	17.6	341.6	4.5
354U1451A-60F-100P #34 -Spot 159	3735	208847	5.6	17.5876	0.7	0.4842	4.3	0.0618	4.3	0.99	386.5	16.0	400.9	14.3	485.1	14.8	386.5	16.0
354U1451A-60F-100P #34 -Spot 236	4557	109062	1.4	17.7477	0.7	0.5119	2.3	0.0659	2.2	0.96	411.6	8.9	419.8	8.0	465.0	14.8	411.6	8.9
354U1451A-60F-100P #34 -Spot 96	4557	550655	10.1	18.1343	0.7	0.5035	1.6	0.0662	1.4	0.88	413.5	5.5	414.1	5.3	417.1	16.3	413.5	5.5
354U1451A-60F-100P #34 -Spot 65	301	27889	1.0	18.0004	0.9	0.5106	1.3	0.0667	0.9	0.73	416.2	3.8	418.8	4.4	433.6	19.3	416.2	3.8
354U1451A-60F-100P #34 -Spot 226	976	70732	2.7	17.7294	0.8	0.5249	2.7	0.0675	2.6	0.95	421.2	10.6	428.4	9.5	467.3	17.8	421.2	10.6
354U1451A-60F-100P #34 -Spot 67	971	472363	4.4	17.5527	1.1	0.5513	2.7	0.0702	2.5	0.92	437.4	10.4	445.8	9.7	489.5	23.2	437.4	10.4
354U1451A-60F-100P #34 -Spot 70	221	49035	5.0	17.8105	0.9	0.5492	1.8	0.0710	1.5	0.86	442.0	6.5	444.5	6.3	457.2	19.6	442.0	6.5
354U1451A-60F-100P #34 -Spot 262	1310	66552	1.2	17.2693	0.7	0.5668	1.2	0.0710	1.0	0.83	442.3	4.3	456.0	4.5	525.2	15.2	442.3	4.3
354U1451A-60F-100P #34 -Spot 60	1985	441823	1.7	17.3600	0.8	0.5657	1.9	0.0713	1.7	0.91	443.7	7.4	455.3	6.9	513.7	16.8	443.7	7.4
354U1451A-60F-100P #34 -Spot 150	1808	178675	17.6	17.8531	0.7	0.5636	1.2	0.0730	1.0	0.81	454.2	4.3	453.8	4.4	451.9	15.5	454.2	4.3
354U1451A-60F-100P #34 -Spot 193	1660	120627	6.5	17.1228	0.6	0.5897	1.2	0.0733	1.1	0.86	455.8	4.6	470.7	4.6	543.9	13.7	455.8	4.6
354U1451A-60F-100P #34 -Spot 267	785	93781	3.6	17.4470	0.7	0.5835	1.7	0.0739	1.6	0.91	459.4	7.0	466.7	6.5	502.7	15.4	459.4	7.0
354U1451A-60F-100P #34 -Spot 253	2038	147542	9.5	17.5021	0.7	0.5823	1.3	0.0739	1.1	0.82	459.9	4.7	465.9	4.8	495.8	16.2	459.9	4.7
354U1451A-60F-100P #34 -Spot 143	973	81833	3.4	17.3670	0.8	0.5931	2.4	0.0747	2.3	0.94	464.6	10.3	472.8	9.2	512.9	18.0	464.6	10.3
354U1451A-60F-100P #34 -Spot 85	387	35174	2.4	17.8814	0.7	0.5770	1.4	0.0749	1.2	0.86	465.4	5.6	462.5	5.4	448.4	16.1	465.4	5.6
354U1451A-60F-100P #34 -Spot 289	422	780575	2.5	17.5770	0.7	0.5919	1.4	0.0755	1.2	0.87	469.1	5.6	472.1	5.4	486.4	15.7	469.1	5.6
354U1451A-60F-100P #34 -Spot 211	565	107133	9.7	17.4502	0.7	0.5993	1.3	0.0759	1.1	0.84	471.5	5.0	476.8	5.0	502.4	15.7	471.5	5.0
354U1451A-60F-100P #34 -Spot 11	493	58639	1.9	17.8077	0.8	0.5877	1.4	0.0759	1.2	0.81	471.8	5.4	469.4	5.4	457.6	18.7	471.8	5.4
354U1451A-60F-100P #34 -Spot 56	1568	187766	11.0	17.5373	0.6	0.5971	1.2	0.0760	1.0	0.85	472.1	4.8	475.4	4.7	491.4	14.2	472.1	4.8
354U1451A-60F-100P #34 -Spot 178	974	219988	0.7	17.5824	0.6	0.6000	1.3	0.0765	1.1	0.89	475.4	5.2	477.2	4.8	485.7	12.6	475.4	5.2
354U1451A-60F-100P #34 -Spot 7	1539	100203	3.1	17.5542	0.8	0.6018	1.8	0.0766	1.7	0.90	476.1	7.6	478.4	7.0	489.3	17.8	476.1	7.6
354U1451A-60F-100P #34 -Spot 90	1320	262297	1.1	17.4815	0.7	0.6060	1.3	0.0769	1.1	0.83	477.4	5.0	481.0	5.0	498.4	16.2	477.4	5.0
354U1451A-60F-100P #34 -Spot 161	2581	755092	3.9	17.6860	0.5	0.5996	1.1	0.0769	1.0	0.91	477.8	4.6	477.0	4.2	472.7	10.4	477.8	4.6
354U1451A-60F-100P #34 -Spot 221	600	90525	1.3	17.4403	0.6	0.6098	1.5	0.0772	1.4	0.91	479.2	6.4	483.4	5.8	503.6	13.9	479.2	6.4
354U1451A-60F-100P #34 -Spot 315	1220	191964	2.5	17.2265	0.6	0.6205	1.3	0.0776	1.1	0.88	481.5	5.1	490.2	4.9	530.7	12.8	481.5	5.1
354U1451A-60F-100P #34 -Spot 14	679	38268	2.7	17.0013	1.1	0.6320	2.5	0.0780	2.3	0.90	484.0	10.6	497.4	9.9	559.5	23.6	484.0	10.6
354U1451A-60F-100P #34 -Spot 45	1397	115791	1.5	17.4564	0.7	0.6191	1.3	0.0784	1.0	0.82	486.6	4.8	489.3	4.9	501.6	16.1	486.6	4.8
354U1451A-60F-100P #34 -Spot 146	678	514009	1.5	17.4742	0.8	0.6199	1.2	0.0786	0.9	0.76	487.7	4.2	489.8	4.6	499.4	16.8	487.7	4.2
354U1451A-60F-100P #34 -Spot 101	1022	259572	6.5	17.7303	0.7	0.6134	1.5	0.0789	1.3	0.89	489.6	6.3	485.7	5.7	467.2	14.7	489.6	6.3
354U1451A-60F-100P #34 -Spot 177	694	73665	1.3	17.6153	0.7	0.6211	1.2	0.0794	0.9	0.81	492.5	4.5	490.5	4.5	481.6	14.8	492.5	4.5
354U1451A-60F-100P #34 -Spot 234	575	108714	1.5	17.3662	0.7	0.6302	1.6	0.0794	1.4	0.89	492.6	6.8	496.2	6.4	513.0	16.2	492.6	6.8
354U1451A-60F-100P #34 -Spot 103	1350	70773	5.1	17.1207	0.7	0.6406	1.2	0.0796	1.0	0.81	493.6	4.7	502.6	4.9	544.2	15.7	493.6	4.7
354U1451A-60F-100P #34 -Spot 247	461	60969	3.6	17.6623	0.8	0.6211	1.4	0.0796	1.1	0.80	493.7	5.2	490.5	5.3	475.7	18.3	493.7	5.2
354U1451A-60F-100P #34 -Spot 102	349	52851	1.8	17.4026	0.6	0.6311	1.9	0.0797	1.8	0.94	494.3	8.5	496.8	7.4	508.4	13.9	494.3	8.5
354U1451A-60F-100P #34 -Spot 71	1429	66559	1.3	17.4067	0.7	0.6336	1.4	0.0800	1.2	0.86	496.3	5.7	498.4	5.5	507.9	15.5	496.3	5.7
354U1451A-60F-100P #34 -Spot 6	837	61807	2.0	17.4415	0.6	0.6353	1.2	0.0804	1.1	0.88	498.5	5.1	499.4	4.8	503.4	12.7	498.5	5.1
354U1451A-60F-100P #34 -Spot 295	529	147822	1.9	17.3854	0.9	0.6380	1.3	0.0805	1.0	0.75	499.0	4.8	501.1	5.3	510.5	19.6	499.0	4.8
354U1451A-60F-100P #34 -Spot 279	782	63164	5.0	17.5367	0.6	0.6374	1.2	0.0811	1.0	0.84	502.7	4.8	500.7	4.7	491.5	14.2	502.7	4.8
354U1451A-60F-100P #34 -Spot 220	260	32893	2.0	17.4222	0.9	0.6423	1.8	0.0812	1.6	0.87	503.2	7.6	503.7	7.1	505.9	19.3	503.2	7.6
354U1451A-60F-100P #34 -Spot 81	459	49408	0.4	17.2686	0.7	0.6529	1.4	0.0818	1.2	0.85	506.9	5.7	510.2	5.5	525.4	15.7	506.9	5.7
354U1451A-60F-100P #34 -Spot 243	281	32271	1.9	17.2072	1.1	0.6554	2.0	0.0818	1.6	0.82	507.0	7.9	511.8	7.9	533.1	24.5	507.0	7.9
354U1451A-60F-100P #34 -Spot 105	833	70666	1.1	17.4925	0.7	0.6494	2.4	0.0824	2.3	0.96	510.6	11.2	508.1	9.5	497.0	15.0	510.6	11.2
354U1451A-60F-100P #34 -Spot 120	928	310515	1.9	17.2403	0.7	0.6640	1.3	0.0831	1.1	0.84	514.4	5.4	517.1	5.3	529.0	15.4	514.4	5.4
354U1451A-60F-100P #34 -Spot 63	662	102002	23.9	16.9725	0.8	0.6763	1.8	0.0833	1.6	0.89	515.7	7.9	524.6	7.4	563.1	18.1	515.7	7.9
354U1451A-60F-100P #34 -Spot 252	175	30377	0.6	17.0886	0.7	0.6769	1.2	0.0839	0.9	0.78	519.5	4.5	524.9	4.8	548.3	16.0	519.5	4.5
354U1451A-60F-100P #34 -Spot 139	481	60799	1.1	17.0929	0.7	0.6799	1.2	0.0843	1.0	0.80	521.9	4.9	526.7	5.0	547.8	16.0	521.9	4.9
354U1451A-60F-100P #34 -Spot 94	569	497539	2.0	17.1036	0.5	0.6927	1.1	0.0860	0.9	0.86	531.6	4.7	534.4	4.4	546.3	11.8	531.6	4.7
354U1451A-60F-100P #34 -Spot 133	291	64706	7.5	16.9089	0.7	0.7080	1.5	0.0869	1.3	0.87	537.0	6.7	543.6	6.3	571.3	16.3	537.0	6.7
354U1451A-60F-100P #34 -Spot 199	795	168592	4.6	16.7345	0.6	0.7333	1.1	0.0890	0.9	0.82	549.8	4.6	558.5	4.6	593.8	13.1	549.8	4.6
354U1451A-60F-100P #34 -Spot 196	91	30758	0.6	16.6678	0.9	0.7435	1.3	0.0899	1.0	0.73	555.0	5.2	564.4	5.7	602.5	19.5	555.0	5.2
354U1451A-60F-100P #34 -Spot 145	1283	936954	58.8	16.5108	0.8	0.7613	1.6	0.0912	1.4	0.87	562.7	7.4	574.8	6.9	622.9	16.9	562.7	7.4
354U1451A-60F-100P #34 -Spot 144	733	92798	1.9	16.0555	0.8	0.8069	1.4	0.0940	1.1	0.81	579.1	6.3	600.7	6.4	683.0	17.9	579.1	6.3
354U1451A-60F-100P #34 -Spot 311	128	41890	2.1	16.4539	1.1	0.7890	1.8	0.0942	1.3	0.76	580.3	7.4	590.6	7.9	630.4	24.7	580.3	7.4
354U1451A-60F-100P #34 -Spot 224	343	473909	2.1	16.6012	0.7	0.7841	1.3	0.0945	1.1	0.85	581.8	6.0	587.8	5.7	611.1	14.7	581.8	6.0
354U1451A-60F-100P #34 -Spot 209	161	62117	1.1	16.5780	1.0	0.7947	1.5	0.0956	1.2	0.77	588.6	6.7	593.9	6.9	614.1	21.3	588.6	6.7
354U1451A-60F-100P #34 -Spot 242	383	4181740	2.3	16.4977	0.8	0.8319	1.5	0.0996	1.2	0.86	611.9	7.3	614.6	6.7	624.6	16.2	611.9	7.3
354U1451A-60F-100P #34 -Spot 261	173	133325	393.1	16.6464	1.1	0.8320	1.5	0.1005	1.1	0.70	617.3	6.3	614.7	7.0	605.2	23.6	617.3	6.3
354U1451A-60F-100P #34 -Spot 272	1631	85235	41.9	15.4197	2.0	0.9047	3.3	0.1012	2.7	0.80	621.6	16.0	654.2	16.2	768.6	41.9	621.6	16.0
354U1451A-60F-100P #34 -Spot 34	420	387765	2.3	16.0262	0.8	0.9036	1.4	0.1051	1.2	0.85	644.1	7.3	653.7	6.8	686.8	16.0	644.1	7.3
354U1451A-60F-100P #34 -Spot 69	439	141505	21.4	15.8352	1.4	0.9313	2.4	0.1070	1.9	0.80	655.4	12.0	668.3	11.7	712.4	30.3	655.4	12.0
354U1451A-60F-100P #34 -Spot 97	334	67415	4.8	15.8558	0.9	0.9563	1.3	0.1100	1.0	0.75	672.9	6.5	681.4	6.7	709.6	18.9	672.9	6.5
354U1451A-60F-100P #34 -Spot 28	470	69439	6.0	15.9861	0.8	0.9535	1.5	0.1106	1.3	0.85	676.2	8.2	679.9	7.4	692.2	16.7	676.2	8.2
354U1451A-60F-100P #34 -Spot 112	418	65484	4.0	15.4016	1.0	1.0044	1.5	0.1122	1.1	0.74	685.8	7.4	706.0	7.9	771.1	22.1	685.8	7.4
354U1451A-60F-100P #34 -Spot 217	145	32684	1.9	15.8865	1.0	0.9749	1.4	0.1124	1.0	0.71	686.5	6.5	691.0	7.1	705.5	21.3	686.5	6.5
354U1451A-60F-100P #34 -Spot 51	176	55008	2.0	15.7126	0.9	1.0177	1.6	0.1160	1.4	0.85	707.7	9.1	712.8	8.2	728.9	18.2	707.7	9.1
354U1451A-60F-100P #34 -Spot 26	699	92037	3.3	15.0688	1.0	1.0631	1.7	0.1162	1.3	0.79	708.9	8.9	735.3	8.9	816.9	21.8	708.9	8.9
354U1451A-60F-100P #34 -Spot 299	199	28304	1.6	15.8233	0.7	1.0156	1.4	0.1166	1.2	0.88	711.0	8.2	711.7	7.1	714.0	14.2	711.0	8.2
354U1451A-60F-100P #34 -Spot 297	1542	313151	3.1	14.5076	0.5	1.1272	2.2	0.1186	2.2	0.97	722.8	14.7	766.4	11.9	895.7	10.9	722.8	14.7
354U1451A-60F-100P #34 -Spot 21	274	18370	2.4	14.5705	1.2	1.1375	1.7	0.1203	1.3	0.74	732.1	8.7	771.3	9.2	886.8	23.8	732.1	8.7
354U1451A-60F-100P #34 -Spot 44	333	49689	0.9	15.0932	0.9	1.1266	2.2	0.1234	2.1	0.92	750.0	14.6	766.2	12.1	813.6	18.3	750.0	14.6
354U1451A-60F-100P #34 -Spot 162	1064	40725	1.1	14.3673	0.7	1.1856	1.2	0.1236	0.9	0.80	751.2	6.6	793.9	6.4	915.8	14.2	751.2	6.6
354U1451A-60F-100P #34 -Spot 171	700	393896	11.6	14.8219	1.3	1.1497	2.5	0.1236	2.1	0.84	751.5	15.0	777.1	13.6	851.3	28.1	751.5	15.0
354U1451A-60F-100P #34 -Spot 33	673	81652	22.8	15.2606	0.6	1.1580	1.4	0.1282	1.2	0.88	777.8	8.8	781.0	7.4	790.4	13.5	777.8	8.8
354U1451A-60F-100P #34 -Spot 246	399	206126	4.2	15.3274	1.0	1.1622	2.4	0.1292	2.1	0.90	783.6	15.8	783.0	12.9	781.2	21.6	783.6	15.8

354U1451A-60F-100P #34 -Spot 13	33	9201	0.4	15.0491	1.7	1.1887	2.3	0.1298	1.5	0.66	786.7	11.4	795.4	12.8	819.7	36.3	786.7	11.4
354U1451A-60F-100P #34 -Spot 147	59	11248	1.0	14.9832	0.9	1.1971	1.4	0.1301	1.0	0.76	788.7	7.7	799.2	7.6	828.8	18.7	788.7	7.7
354U1451A-60F-100P #34 -Spot 108	127	102209	1.3	15.1101	1.0	1.1904	1.4	0.1305	1.1	0.74	790.8	7.9	796.2	8.0	811.2	20.4	790.8	7.9
354U1451A-60F-100P #34 -Spot 131	196	66953	1.8	15.2834	0.8	1.1770	1.2	0.1305	1.0	0.78	790.8	7.2	789.9	6.8	787.3	16.2	790.8	7.2
354U1451A-60F-100P #34 -Spot 270	504	79342	1.8	14.9474	0.6	1.2056	1.4	0.1307	1.3	0.89	792.1	9.4	803.1	7.8	833.8	13.4	792.1	9.4
354U1451A-60F-100P #34 -Spot 110	1192	2084913	18.8	13.9906	0.7	1.2934	1.2	0.1313	1.0	0.82	795.3	7.5	842.8	7.0	970.2	14.4	795.3	7.5
354U1451A-60F-100P #34 -Spot 184	298	108557	2.9	14.6291	0.8	1.2444	1.3	0.1321	1.0	0.76	799.8	7.4	820.9	7.2	878.5	17.2	799.8	7.4
354U1451A-60F-100P #34 -Spot 86	796	222349	0.7	14.7876	0.7	1.2389	1.3	0.1329	1.1	0.83	804.6	8.0	818.4	7.2	856.1	14.9	804.6	8.0
354U1451A-60F-100P #34 -Spot 237	123	20061	1.5	15.2494	0.8	1.2074	1.4	0.1336	1.1	0.80	808.3	8.4	804.0	7.8	791.9	17.8	808.3	8.4
354U1451A-60F-100P #34 -Spot 42	288	67847	2.2	15.1074	0.9	1.2190	1.5	0.1336	1.2	0.81	808.5	9.1	809.3	8.2	811.6	17.9	808.5	9.1
354U1451A-60F-100P #34 -Spot 52	228	160972	1.2	14.7708	0.8	1.2479	1.3	0.1337	1.1	0.81	809.2	8.0	822.5	7.3	858.5	15.9	809.2	8.0
354U1451A-60F-100P #34 -Spot 194	1941	1187739	9.0	14.2931	0.6	1.2982	2.2	0.1346	2.1	0.96	814.3	16.1	844.9	12.5	926.4	11.8	814.3	16.1
354U1451A-60F-100P #34 -Spot 271	111	18390	2.7	14.7422	1.3	1.2597	1.8	0.1347	1.3	0.72	814.9	10.0	827.8	10.3	862.6	26.2	814.9	10.0
354U1451A-60F-100P #34 -Spot 223	262	69358	1.0	14.8159	0.7	1.2719	1.3	0.1367	1.1	0.84	826.2	8.7	833.3	7.6	852.2	15.1	826.2	8.7
354U1451A-60F-100P #34 -Spot 89	299	32910	1.7	14.9841	0.8	1.2672	1.6	0.1378	1.3	0.85	832.1	10.4	831.2	8.9	828.7	17.5	832.1	10.4
354U1451A-60F-100P #34 -Spot 300	1525	508450	3.7	14.5524	0.7	1.3126	1.6	0.1386	1.5	0.90	836.7	11.6	851.3	9.4	889.4	14.6	836.7	11.6
354U1451A-60F-100P #34 -Spot 219	424	104845	0.8	14.6144	0.8	1.3239	1.6	0.1404	1.4	0.88	846.9	11.5	856.2	9.5	880.6	15.8	846.9	11.5
354U1451A-60F-100P #34 -Spot 302	27	49181	0.5	14.2754	1.2	1.3564	1.7	0.1405	1.2	0.70	847.5	9.4	870.3	9.8	928.9	24.5	847.5	9.4
354U1451A-60F-100P #34 -Spot 189	444	756797	8.3	14.7816	0.7	1.3164	1.2	0.1412	1.0	0.80	851.3	7.7	852.9	7.0	857.0	15.0	851.3	7.7
354U1451A-60F-100P #34 -Spot 293	105	20626	2.8	14.2494	0.9	1.3764	1.5	0.1423	1.2	0.81	857.7	10.0	878.9	9.1	932.7	18.7	857.7	10.0
354U1451A-60F-100P #34 -Spot 186	237	118707	1.9	14.6134	0.8	1.3440	1.1	0.1425	0.8	0.70	858.8	6.2	865.0	6.5	880.7	16.4	858.8	6.2
354U1451A-60F-100P #34 -Spot 66	486	181784	1.7	14.8741	0.9	1.3255	1.3	0.1431	1.0	0.73	861.9	7.8	856.9	7.7	844.0	19.1	861.9	7.8
354U1451A-60F-100P #34 -Spot 210	340	397924	6.0	14.9610	0.7	1.3240	1.2	0.1437	1.0	0.83	865.7	8.1	856.3	6.9	831.9	13.8	865.7	8.1
354U1451A-60F-100P #34 -Spot 265	639	193399	2.5	14.7968	1.0	1.3391	1.5	0.1438	1.2	0.78	866.0	9.7	862.9	8.9	854.8	19.8	866.0	9.7
354U1451A-60F-100P #34 -Spot 73	526	615188	13.5	14.5348	0.7	1.4348	1.3	0.1513	1.1	0.82	908.4	8.9	903.6	7.7	891.8	15.4	891.8	15.4
354U1451A-60F-100P #34 -Spot 164	1902	445461	25.4	14.4576	0.7	1.4146	1.4	0.1484	1.2	0.86	891.9	10.0	895.1	8.3	902.8	14.8	891.9	10.0
354U1451A-60F-100P #34 -Spot 18	228	78743	1.6	14.5219	1.0	1.4127	1.4	0.1489	1.0	0.73	894.5	8.8	894.3	8.5	893.7	20.2	894.5	8.8
354U1451A-60F-100P #34 -Spot 29	26	12147	0.8	14.7244	1.4	1.3977	1.8	0.1493	1.0	0.59	897.2	8.8	888.0	10.5	865.1	29.9	897.2	8.8
354U1451A-60F-100P #34 -Spot 38	342	120852	2.4	14.4889	0.9	1.4405	1.6	0.1514	1.3	0.85	909.0	11.4	905.9	9.6	898.4	17.6	898.4	17.6
354U1451A-60F-100P #34 -Spot 298	731	76235	9.5	14.4677	0.7	1.4383	1.4	0.1510	1.2	0.86	906.5	10.0	905.0	8.2	901.4	14.7	901.4	14.7
354U1451A-60F-100P #34 -Spot 156	61	16747	67.9	14.2829	0.9	1.4702	1.5	0.1524	1.2	0.79	914.2	10.0	918.2	9.0	927.9	18.9	927.9	18.9
354U1451A-60F-100P #34 -Spot 129	352	68937	2.6	14.2748	1.0	1.4151	1.5	0.1466	1.1	0.75	881.7	9.2	895.3	8.9	929.0	20.5	929.0	20.5
354U1451A-60F-100P #34 -Spot 230	176	81957	0.8	14.2353	1.0	1.4645	1.8	0.1513	1.5	0.82	908.0	12.6	915.9	11.0	934.7	21.4	934.7	21.4
354U1451A-60F-100P #34 -Spot 115	235	90453	1.2	14.1286	0.6	1.5628	1.0	0.1602	0.8	0.82	957.9	7.2	955.6	6.1	950.1	11.7	950.1	11.7
354U1451A-60F-100P #34 -Spot 309	823	341821	15.4	14.1052	0.7	1.5003	1.4	0.1535	1.3	0.89	920.8	10.9	930.5	8.7	953.5	13.3	953.5	13.3
354U1451A-60F-100P #34 -Spot 142	129	93559	0.4	14.0694	0.8	1.5798	1.3	0.1613	1.0	0.75	963.8	8.7	962.3	8.0	958.7	17.3	958.7	17.3
354U1451A-60F-100P #34 -Spot 84	752	1789071	4.6	14.0605	0.5	1.5676	1.3	0.1599	1.2	0.92	956.4	11.0	957.5	8.3	960.0	10.5	960.0	10.5
354U1451A-60F-100P #34 -Spot 32	267	75571	1.2	14.0527	0.6	1.5255	1.5	0.1555	1.3	0.90	932.0	11.5	940.7	9.0	961.2	12.9	961.2	12.9
354U1451A-60F-100P #34 -Spot 106	207	48265	3.5	14.0358	0.7	1.5841	1.3	0.1613	1.2	0.87	964.2	10.5	964.0	8.4	963.6	13.6	963.6	13.6
354U1451A-60F-100P #34 -Spot 154	338	406293	1.9	14.0300	1.4	1.5614	2.2	0.1590	1.7	0.79	950.9	15.4	955.0	13.6	964.4	27.7	964.4	27.7
354U1451A-60F-100P #34 -Spot 22	284	3523756	1.9	14.0266	0.8	1.5863	1.2	0.1614	0.9	0.78	964.8	8.5	964.9	7.6	965.0	15.7	965.0	15.7
354U1451A-60F-100P #34 -Spot 301	664	290425	1.4	14.0105	0.7	1.4595	2.0	0.1484	1.9	0.94	891.8	15.8	913.8	12.1	967.3	14.0	967.3	14.0
354U1451A-60F-100P #34 -Spot 310	107	35383	0.8	13.9202	1.1	1.5681	2.8	0.1584	2.6	0.92	947.8	22.9	957.7	17.5	980.5	22.7	980.5	22.7
354U1451A-60F-100P #34 -Spot 12	112	174407	2.7	13.9021	0.9	1.4393	1.5	0.1452	1.2	0.80	873.9	9.8	905.4	9.0	983.1	18.3	983.1	18.3
354U1451A-60F-100P #34 -Spot 92	296	32481	1.5	13.8943	0.5	1.5904	1.2	0.1603	1.1	0.91	958.6	9.9	966.5	7.6	984.2	10.0	984.2	10.0
354U1451A-60F-100P #34 -Spot 58	323	59786	1.9	13.8889	0.8	1.4901	2.5	0.1502	2.4	0.95	901.9	20.0	926.3	15.2	985.1	16.1	985.1	16.1
354U1451A-60F-100P #34 -Spot 215	214	75967	2.5	13.8776	0.8	1.6242	1.3	0.1635	1.0	0.77	976.4	9.2	979.6	8.3	986.7	17.0	986.7	17.0
354U1451A-60F-100P #34 -Spot 79	1338	391123	16.5	13.8555	0.7	1.4071	1.3	0.1415	1.1	0.83	852.9	8.4	891.9	7.6	989.9	14.6	989.9	14.6
354U1451A-60F-100P #34 -Spot 274	292	1199683	1.3	13.8341	0.7	1.5503	1.2	0.1556	1.1	0.85	932.4	9.2	950.6	7.7	993.1	13.5	993.1	13.5
354U1451A-60F-100P #34 -Spot 282	346	259242	1.1	13.8327	0.7	1.6114	1.4	0.1617	1.2	0.86	966.4	10.8	974.6	8.7	993.3	14.3	993.3	14.3
354U1451A-60F-100P #34 -Spot 280	627	516926	5.4	13.8201	0.8	1.5993	1.4	0.1604	1.2	0.81	958.9	10.3	969.9	8.9	995.1	17.0	995.1	17.0
354U1451A-60F-100P #34 -Spot 39	575	270407	2.9	13.8134	0.7	1.6110	1.2	0.1615	1.0	0.81	964.9	8.7	974.5	7.5	996.1	14.1	996.1	14.1
354U1451A-60F-100P #34 -Spot 93	366	54185	1.2	13.8000	0.7	1.5883	1.3	0.1590	1.1	0.86	951.4	10.0	965.6	8.2	998.1	13.9	998.1	13.9
354U1451A-60F-100P #34 -Spot 200	87	64219	3.3	13.7779	0.8	1.5687	1.3	0.1568	1.1	0.82	939.1	9.6	957.9	8.3	1001.3	15.6	1001.3	15.6
354U1451A-60F-100P #34 -Spot 31	2582	146220	9.4	13.7697	0.5	1.5213	1.3	0.1520	1.1	0.91	912.1	9.7	939.0	7.7	1002.6	10.6	1002.6	10.6
354U1451A-60F-100P #34 -Spot 2	132	140583	1.4	13.7250	0.8	1.6847	1.4	0.1678	1.2	0.82	999.8	10.9	1002.8	9.1	1009.2	16.5	1009.2	16.5
354U1451A-60F-100P #34 -Spot 15	833	170776	12.5	13.7067	0.6	1.5232	1.3	0.1515	1.1	0.89	909.3	9.5	939.8	7.7	1011.9	11.5	1011.9	11.5
354U1451A-60F-100P #34 -Spot 235	214	22655	2.3	13.6070	1.8	1.6575	2.8	0.1636	2.1	0.75	977.0	18.8	992.4	17.5	1026.6	36.8	1026.6	36.8
354U1451A-60F-100P #34 -Spot 165	435	228595	3.8	13.5481	0.6	1.7838	1.7	0.1753	1.6	0.92	1041.5	15.1	1039.6	11.1	1035.4	13.1	1035.4	13.1
354U1451A-60F-100P #34 -Spot 188	191	28191	1.8	13.5449	1.2	1.6889	1.8	0.1660	1.4	0.76	989.9	12.7	1004.3	11.6	1035.9	23.6	1035.9	23.6
354U1451A-60F-100P #34 -Spot 24	282	116393	1.4	13.5102	0.8	1.6502	1.8	0.1618	1.6	0.89	966.6	14.2	989.6	11.3	1041.1	16.6	1041.1	16.6
354U1451A-60F-100P #34 -Spot 16	62	42740	1.1	13.5037	1.0	1.8691	1.7	0.1831	1.4	0.82	1084.1	14.2	1070.2	11.5	1042.0	20.3	1042.0	20.3
354U1451A-60F-100P #34 -Spot 233	937	148152	4.5	13.3041	0.8	1.7003	1.2	0.1641	0.9	0.77	979.7	8.4	1008.7	7.8	1072.1	15.7	1072.1	15.7
354U1451A-60F-100P #34 -Spot 264	124	21535	1.8	13.3015	0.8	1.7782	1.2	0.1716	0.9	0.75	1021.0	8.4	1037.5	7.7	1072.5	15.9	1072.5	15.9
354U1451A-60F-100P #34 -Spot 283	290	469407	2.0	13.2453	0.6	1.8485	1.4	0.1776	1.3	0.91	1054.1	12.7	1062.9	9.5	1080.9	12.0	1080.9	12.0
354U1451A-60F-100P #34 -Spot 136	927	71600	1.5	13.2216	1.1	1.6688	2.0	0.1601	1.7	0.84	957.3	15.2	996.7	12.9	1084.5	22.1	1084.5	22.1
354U1451A-60F-100P #34 -Spot 123	240	151178	0.7	13.1679	0.9	1.8978	1.6	0.1813	1.4	0.84	1074.2	13.4	1080.3	10.8	1092.7	17.7	1092.7	17.7
354U1451A-60F-100P #34 -Spot 30	816	436023	1.0	13.1402	0.8	1.8677	1.6	0.1781	1.5	0.88	1056.4	14.1	1069.7	10.9	1096.9	15.5	1096.9	15.5
354U1451A-60F-100P #34 -Spot 251	357	53344	2.0	13.1334	0.6	1.8895	1.4	0.1801	1.2	0.89	1067.3	11.9	1077.4	9.0	1097.9	12.2	1097.9	12.2
354U1451A-60F-100P #34 -Spot 308	275	77138	1.8	13.1234	0.6	1.8989	1.0	0.1808	0.8	0.77	1071.4	7.6	1080.7	6.7	1099.5	13.0	1099.5	13.0
354U1451A-60F-100P #34 -Spot 77	137	73162	1.6	13.1029	1.0	2.0240	1.6	0.1924	1.2	0.76	1134.5	12.8	1123.6	10.9	1102.6	20.8	1102.6	20.8
354U1451A-60F-100P #34 -Spot 173	304	157147	14.5	13.0986	0.7	1.9155	1.2	0.1821	1.0	0.82	1078.2	9.5	1086.5	7.8	1103.3	13.3	1103.3	13.3
354U1451A-60F-100P #34 -Spot 125	1251	238343	8.0	13.0953	0.7	1.8779	1.3	0.1784	1.0	0.82	1058.4	10.2	1073.3	8.4	1103.7	14.6	1103.7	14.6
354U1451A-60F-100P #34 -Spot 296	92	67434	0.9	12.9750	0.7	2.0570	1.3	0.1937	1.2	0.87	1141.1	12.2	1134.6	9.1	1122.2	13.1	1122.2	13.1
354U1451A-60F-100P #34 -Spot 80	166	49160	1.6	12.9438	0.8	2.0148	1.7	0.1892	1.5	0.88	1117.2	15.8	1120.5	11.9	1127.0	16.4	1127.0	16.4
354U1451A-60F-100P #34 -Spot 119	130	32987	1.2	12.9385	0.7	2.0825	1.3	0.1955	1.1	0.83	1151.1	11.6	1143.1	9.1	1127.8	14.7	1127.8	14.7
354U1451A-60F-100P #34 -Spot 266	542	223857	1.5	12.9340	0.6	2.0720	1.3	0.1945	1.2	0.88	1145.4	12.1	1139.6	8.9	1128.5	12.3	1128.5	12.3
354U1451A-60F-100P #34 -Spot 207	524	244785	1.9	12.8564	0.7	1.7651	1.3	0.1647	1.1	0.85	982.6	9.9	1032.7	8.3	1140.5	13.3	1140.5	13.3
354U1451A-60F-100P #34 -Spot 175	287	240771	0.8	12.7521	0.7	2.0502	1.5	0.1897	1.3	0.88	1119.8	13.2	1132.4	10.0	1156.6	14.0	1156.6	14.0
354U1451A-60F-100P #34 -Spot 227	46	14346	1.0	12.7034	0.9	2.0742	1.3	0.1912	0.9	0.71	1127.8	9.6	1140.3	9.0	1164.2	18.3	1164.2	18.3
354U1451A-60F-100P #34 -Spot 36	1454	262953	10.6	12.6786	0.6	1.9953	1.2	0.1836	1.0	0.83	1086.4	9.8	1113.9	7.9	1168.1	12.8	1168.1	12.8
354U1451A-60F-100P #34 -Spot 160	22	190587	0.5	12.6507	0.9	2.0844	1.7	0.1913	1.5	0.85	1128.6	15.1	1143.7	11.9	1172.5	18.2	1172.5	18.2
354U1451A-60F-100P #34 -Spot 290	1163	1959041	4.0	12.5981	0.8	1.9917	1.3	0.1821	1.0	0.79	1078.2	10.1	1112.7	8.7	1180.7	15.7	1180.7	15.7
354U1451A-60F-100P #34 -Spot 140	294	194776	1.2	12.4475	0.8	2.0671	1.5	0.1867	1.2	0.82	1103.4	12.3	1138.0	10.1	1204.4	16.5	1204.4	16.5
354U1451A-60F-100P #34 -Spot 291	250	130438	16.8	12.3660	0.8	2.3329	1.7	0.2093	1.5	0.88	1225.2	16.3	1222.4	11.8	1217.3	15.5	1217.3	15.5
354U1451A-60F-100P #34 -Spot 204	373	128732	2.4	12.2487	0.8	2.1881	1.7	0.1945	1.5	0.88	1145.5	15.3	1177.3	11.6	1236.1	15.5	1236.1	15.5
354U1451A-60F-100P #34 -Spot 135	56	18688	2.3	12.1661	1.0	2.2935	1.6	0.2025	1.2	0.75	1188.5	12.7	1210.3	11.1	1249.3	20.5	1249.3	20.5
354U1451A-60F-100P #34 -Spot 182	1508	110428	13.4	12.0229	0.8	2.1896	1.4	0.1910	1.1	0.83	1126.9	11.6	1177.7	9.4	1272.4	14.7	1272.4	14.7
354U1451A-60F-100P #34 -Spot 285	355	117049	1.4	11.9998	0.4	2.4578	1.0	0.2140	1.0	0.91	1250.1	10.8	1259.7	7.6	1276.2	8.6	1276.2	8.6
354U1451A-60F-100P #34 -Spot 288	2566	70187	5.2	11.6669	0.5	2.1694	1.0	0.1836	0.9	0.85	1086.9	8.8	1171.3	7.2	1330.8	10.6	1330.8	10.6
354U1451A-60F-100P #34 -Spot 240	988	140508	1.0	11.6217	0.6	2.8053	1.4	0.2366	1.2	0.88	1368.8	14.7	1357.0	10.1	1338.4	12.2	1338.4	12.2
354U1451A-60F-100P #34 -Spot 201	1501	531214	7.8	11.4941	0.7	2.6696	1.7	0.2226	1.6	0.92	1295.8	18.2	1320.1	12.5	1359.7	12.8	1359.7	12.8
354U1451A-60F-100P #34 -Spot 151	596	501245	2.1	11.4365	0.7	2.7159	1.3	0.2254	1.1	0.86	1310.2	13.5	1332.8	9.8	1369.3	13.0	1369.3	13.0
354U1451A-60F-100P #34 -Spot 82	452	25713	1.2	11.3356	1.0	2.6123	1.4	0.2149	1.0	0.73	1254.6	11.8	1304.1	10.4	1386.4	18.4	1386.4	18.4
354U1451A-60F-100P #34 -Spot 197	246	299625	1.6	11.2636	0.8	2.9098	1.5	0.2378	1.3	0.85	1375.3	16.0	1384.5	11.5	1398.6	15.5	1398.6	15.5
354U1451A-60F-100P #34 -Spot 59	557	132050	7.3	11.2148	0.8	2.6524	1.9	0.2158	1.7	0.90	1259.8	20.0	1315.3	14.3	1406.9	16.2	1406.9	16.2
354U1451A-60F-100P #34 -Spot 263	749	826281	0.9	11.1947	0.7	2.8283	1.4	0.2297	1.2	0.86	1333.1	14.3	1363.1	10.3	1410.4	13.4	1410.4	13.4
354U1451A-60F-100P #34 -Spot 216	628	105894	2.1	10.9191	0.7	3.0361	1.4	0.2405	1.3	0.88	1389.5	15.9	1416.7	11.1	1457.9	13.0	1457.9	13.0
354U1451A-60F-100P #34 -Spot 17	729	202951	3.3	10.6344	0.7	3.3860	1.3	0.2613	1.1	0.85	1496.3	14.9	1501.2	10.4	1508.0	13.3	1508.0	13.3
354U1451A-60F-100P #34 -Spot 250	97	49437	1.5	10.3805	0.7	3.5784	1.3	0.2695	1.1	0.84	1538.4	14.5	1544.8	10.0	1553.5	12.7	1553.5	12.7
354U1451A-60F-100P #34 -Spot 191	68	24660	1.4	10.2870	0.9	3.4186	1.5	0.2552	1.2	0.80	1465.1	16.2	1508.7	12.2	1570.4	17.4	1570.4	17.4

354U1451A-60F-100P #34 -Spot 306	273	153057	2.6	10.2384	0.6	3.6802	1.5	0.2734	1.4	0.92	1558.0	19.1	1567.1	12.0	1579.3	11.2	1579.3	11.2
354U1451A-60F-100P #34 -Spot 138	516	683902	1.2	10.2159	0.7	3.5443	1.5	0.2627	1.3	0.87	1503.7	17.2	1537.2	11.7	1583.4	13.5	1583.4	13.5
354U1451A-60F-100P #34 -Spot 292	80	27386	2.3	10.1878	0.7	3.6864	1.1	0.2725	0.9	0.81	1553.5	12.8	1568.4	9.2	1588.6	12.7	1588.6	12.7
354U1451A-60F-100P #34 -Spot 111	278	580364	1.7	10.1075	0.6	3.6259	1.3	0.2659	1.2	0.88	1520.0	15.6	1555.2	10.5	1603.3	11.8	1603.3	11.8
354U1451A-60F-100P #34 -Spot 269	553	236277	1.7	10.0781	1.1	3.4357	3.3	0.2512	3.1	0.94	1444.9	40.4	1512.6	26.0	1608.8	20.6	1608.8	20.6
354U1451A-60F-100P #34 -Spot 218	293	72339	3.3	10.0450	0.7	3.5891	2.6	0.2616	2.5	0.97	1498.0	34.0	1547.1	20.9	1614.9	12.2	1614.9	12.2
354U1451A-60F-100P #34 -Spot 20	553	144847	1.6	9.9970	0.9	3.7889	1.7	0.2748	1.5	0.87	1565.3	20.7	1590.4	13.8	1623.8	16.0	1623.8	16.0
354U1451A-60F-100P #34 -Spot 232	2425	332247	1.1	9.9312	0.8	3.6328	1.5	0.2618	1.2	0.84	1498.9	16.4	1556.7	11.6	1636.1	14.7	1636.1	14.7
354U1451A-60F-100P #34 -Spot 27	306	2276674	0.8	9.8870	0.9	4.0080	1.5	0.2875	1.2	0.82	1629.2	17.7	1635.8	12.2	1644.4	16.1	1644.4	16.1
354U1451A-60F-100P #34 -Spot 47	973	144489	15.2	9.8573	0.8	3.9159	1.4	0.2801	1.2	0.82	1591.8	16.8	1617.0	11.7	1649.9	15.1	1649.9	15.1
354U1451A-60F-100P #34 -Spot 206	251	133289	2.9	9.7943	0.7	3.9824	1.2	0.2830	1.0	0.82	1606.5	13.6	1630.6	9.5	1661.8	12.3	1661.8	12.3
354U1451A-60F-100P #34 -Spot 68	1018	1166003	0.8	9.7706	0.6	3.9632	1.3	0.2810	1.1	0.87	1596.2	15.9	1626.7	10.5	1666.3	11.9	1666.3	11.9
354U1451A-60F-100P #34 -Spot 62	970	774330	5.0	9.7142	0.7	4.4380	1.6	0.3128	1.5	0.89	1754.5	22.5	1719.5	13.6	1677.0	13.5	1677.0	13.5
354U1451A-60F-100P #34 -Spot 155	265	1358596	2.0	9.5654	0.6	4.2607	1.2	0.2957	1.0	0.85	1670.0	15.4	1685.8	10.1	1705.5	11.8	1705.5	11.8
354U1451A-60F-100P #34 -Spot 107	211	160995	1.1	9.5545	0.7	4.2571	1.5	0.2951	1.3	0.88	1667.1	19.3	1685.1	12.3	1707.6	13.1	1707.6	13.1
354U1451A-60F-100P #34 -Spot 134	404	1358885	5.3	9.5086	0.6	4.6448	1.4	0.3205	1.2	0.90	1792.0	19.0	1757.4	11.3	1716.4	11.0	1716.4	11.0
354U1451A-60F-100P #34 -Spot 222	43	17235	0.6	9.5070	0.7	4.5169	1.3	0.3116	1.1	0.85	1748.5	16.7	1734.1	10.7	1716.7	12.5	1716.7	12.5
354U1451A-60F-100P #34 -Spot 181	100	57123	0.9	9.4755	0.8	4.5523	1.7	0.3130	1.5	0.89	1755.4	23.4	1740.6	14.3	1722.8	14.4	1722.8	14.4
354U1451A-60F-100P #34 -Spot 163	2797	734229	13.3	9.4634	0.7	4.3718	1.2	0.3002	1.1	0.85	1692.3	15.7	1707.0	10.3	1725.2	12.1	1725.2	12.1
354U1451A-60F-100P #34 -Spot 284	310	282737	2.0	9.3360	0.7	4.4167	1.6	0.2992	1.4	0.89	1687.3	20.5	1715.5	12.9	1750.0	12.9	1750.0	12.9
354U1451A-60F-100P #34 -Spot 312	468	322125	6.0	9.1752	0.6	4.7929	1.2	0.3191	1.1	0.86	1785.2	16.6	1783.6	10.4	1781.8	11.6	1781.8	11.6
354U1451A-60F-100P #34 -Spot 130	423	286552	2.4	9.1591	0.7	4.6175	1.2	0.3069	1.0	0.81	1725.3	15.0	1752.4	10.2	1785.0	13.0	1785.0	13.0
354U1451A-60F-100P #34 -Spot 287	404	1848749	6.1	8.9447	0.7	4.9547	1.8	0.3216	1.7	0.92	1797.4	26.2	1811.6	15.3	1828.1	12.9	1828.1	12.9
354U1451A-60F-100P #34 -Spot 41	424	456218	5.9	8.9236	0.8	4.3707	1.6	0.2830	1.4	0.87	1606.4	20.0	1706.8	13.4	1832.3	14.5	1832.3	14.5
354U1451A-60F-100P #34 -Spot 8	1008	135040	17.3	8.8749	0.9	4.1643	1.9	0.2682	1.7	0.89	1531.5	23.2	1667.0	15.7	1842.2	15.8	1842.2	15.8
354U1451A-60F-100P #34 -Spot 172	246	935026	4.2	8.8592	0.5	5.1471	1.1	0.3309	1.0	0.87	1842.5	15.6	1843.9	9.5	1845.4	9.8	1845.4	9.8
354U1451A-60F-100P #34 -Spot 248	157	118760	2.1	8.8071	0.6	4.8240	1.8	0.3083	1.7	0.95	1732.2	26.3	1789.1	15.3	1856.1	10.2	1856.1	10.2
354U1451A-60F-100P #34 -Spot 307	654	520944	2.3	8.7672	0.8	5.0833	1.5	0.3234	1.3	0.84	1806.2	20.0	1833.3	12.8	1864.3	14.7	1864.3	14.7
354U1451A-60F-100P #34 -Spot 124	544	152201	3.0	8.7567	0.6	5.0532	1.5	0.3211	1.3	0.91	1794.9	21.0	1828.3	12.5	1866.5	11.1	1866.5	11.1
354U1451A-60F-100P #34 -Spot 83	387	432410	2.6	8.7378	0.7	4.9853	1.4	0.3161	1.2	0.87	1770.5	18.6	1816.8	11.6	1870.4	12.2	1870.4	12.2
354U1451A-60F-100P #34 -Spot 192	60	57639	1.4	8.7279	0.7	4.5311	1.4	0.2869	1.2	0.86	1626.3	17.4	1736.7	11.7	1872.4	12.8	1872.4	12.8
354U1451A-60F-100P #34 -Spot 43	532	266189	3.4	8.7070	0.5	5.2893	1.3	0.3342	1.2	0.93	1858.5	19.8	1867.1	11.2	1876.7	8.6	1876.7	8.6
354U1451A-60F-100P #34 -Spot 122	223	164294	3.1	8.6998	0.7	5.2323	1.4	0.3303	1.2	0.86	1839.8	19.1	1857.9	11.8	1878.2	12.5	1878.2	12.5
354U1451A-60F-100P #34 -Spot 185	363	235615	9.4	8.6130	0.6	5.2747	1.3	0.3296	1.2	0.89	1836.6	18.8	1864.8	11.3	1896.3	11.0	1896.3	11.0
354U1451A-60F-100P #34 -Spot 198	167	198851	1.9	8.6100	0.7	5.3659	1.0	0.3352	0.7	0.72	1863.7	11.7	1879.4	8.5	1896.9	12.4	1896.9	12.4
354U1451A-60F-100P #34 -Spot 278	649	532257	5.5	8.5341	0.7	5.5271	1.4	0.3422	1.3	0.88	1897.5	20.8	1904.8	12.3	1912.8	12.1	1912.8	12.1
354U1451A-60F-100P #34 -Spot 238	96	479845	1.0	8.5076	0.7	5.6809	2.2	0.3507	2.0	0.94	1937.8	34.2	1928.5	18.7	1918.4	12.8	1918.4	12.8
354U1451A-60F-100P #34 -Spot 260	386	185811	18.3	8.3311	0.8	5.3236	1.5	0.3218	1.3	0.86	1798.5	19.8	1872.7	12.6	1955.9	13.6	1955.9	13.6
354U1451A-60F-100P #34 -Spot 244	478	137259	3.0	8.1291	0.7	5.8292	1.3	0.3438	1.1	0.85	1905.0	17.6	1950.8	10.9	1999.6	11.6	1999.6	11.6
354U1451A-60F-100P #34 -Spot 100	669	159634	3.8	8.0911	0.6	5.1913	1.1	0.3048	1.0	0.84	1714.9	14.3	1851.2	9.6	2007.9	10.8	2007.9	10.8
354U1451A-60F-100P #34 -Spot 245	152	87287	3.4	7.6733	0.6	7.0758	1.2	0.3940	1.0	0.85	2141.2	18.8	2121.0	10.9	2101.5	11.4	2101.5	11.4
354U1451A-60F-100P #34 -Spot 9	70	37375	1.2	7.6381	0.8	6.9916	1.4	0.3875	1.2	0.83	2111.1	20.9	2110.4	12.4	2109.6	13.6	2109.6	13.6
354U1451A-60F-100P #34 -Spot 132	152	81355	1.4	7.4282	0.6	7.1112	1.2	0.3833	1.1	0.89	2091.6	19.9	2125.5	11.1	2158.4	9.8	2158.4	9.8
354U1451A-60F-100P #34 -Spot 109	199	1674757	2.0	7.4221	0.7	7.4264	1.3	0.3999	1.1	0.86	2168.8	20.7	2164.2	11.7	2159.8	11.7	2159.8	11.7
354U1451A-60F-100P #34 -Spot 286	1023	19546529	67.4	7.1750	0.8	7.4446	1.5	0.3876	1.2	0.81	2111.6	21.3	2166.3	13.0	2218.7	14.7	2218.7	14.7
354U1451A-60F-100P #34 -Spot 256	205	86466	1.9	6.9700	0.5	8.3510	1.0	0.4223	0.8	0.85	2271.1	15.5	2269.9	8.6	2268.8	8.6	2268.8	8.6
354U1451A-60F-100P #34 -Spot 19	150	502484	1.8	6.7814	0.8	8.6942	1.4	0.4278	1.2	0.81	2295.8	22.4	2306.5	13.0	2316.0	14.4	2316.0	14.4
354U1451A-60F-100P #34 -Spot 57	470	389602	2.2	6.5686	0.8	8.9161	1.5	0.4249	1.3	0.85	2282.9	24.4	2329.5	13.7	2370.5	13.5	2370.5	13.5
354U1451A-60F-100P #34 -Spot 3	299	163137	3.3	6.3789	0.7	9.2857	1.6	0.4298	1.5	0.90	2304.7	28.5	2366.6	15.0	2420.3	12.2	2420.3	12.2
354U1451A-60F-100P #34 -Spot 37	327	211000	1.6	6.3138	0.8	10.1081	1.5	0.4631	1.3	0.85	2453.1	25.9	2444.7	13.9	2437.7	13.5	2437.7	13.5
354U1451A-60F-100P #34 -Spot 121	441	512265	1.5	6.3052	0.6	9.8495	1.3	0.4506	1.1	0.87	2398.0	22.6	2420.8	12.0	2440.0	10.9	2440.0	10.9
354U1451A-60F-100P #34 -Spot 157	299	419222	1.6	6.2688	0.7	10.3749	1.4	0.4719	1.2	0.88	2491.9	25.7	2468.8	13.0	2449.8	11.2	2449.8	11.2
354U1451A-60F-100P #34 -Spot 168	292	236182	2.0	6.1623	0.6	10.0265	1.3	0.4483	1.2	0.89	2387.8	23.8	2437.2	12.3	2478.8	10.1	2478.8	10.1
354U1451A-60F-100P #34 -Spot 187	133	76018	1.1	6.0620	0.7	10.2348	1.2	0.4502	1.0	0.82	2396.0	20.3	2456.2	11.4	2506.4	11.8	2506.4	11.8
354U1451A-60F-100P #34 -Spot 149	1343	391902	0.9	6.0248	0.7	9.6481	1.5	0.4218	1.3	0.87	2268.5	24.3	2401.8	13.5	2516.8	12.2	2516.8	12.2
354U1451A-60F-100P #34 -Spot 241	1088	54965	5.4	5.9933	0.7	11.2735	1.2	0.4902	1.0	0.83	2571.8	21.2	2546.0	11.2	2525.6	11.3	2525.6	11.3
354U1451A-60F-100P #34 -Spot 141	159	1046485	1.2	5.9479	0.7	10.9566	1.5	0.4729	1.3	0.89	2496.1	27.7	2519.5	14.0	2538.3	11.7	2538.3	11.7
354U1451A-60F-100P #34 -Spot 117	114	108913	2.4	5.9083	0.8	9.7873	1.3	0.4196	1.0	0.76	2258.6	18.9	2415.0	12.0	2549.5	14.0	2549.5	14.0
354U1451A-60F-100P #34 -Spot 170	1166	1469831	7.2	5.8431	0.8	11.2397	1.5	0.4765	1.3	0.85	2512.1	27.0	2543.2	14.2	2568.1	13.3	2568.1	13.3
354U1451A-60F-100P #34 -Spot 275	1021	1499896	21.3	5.7535	0.7	10.5813	1.2	0.4417	1.0	0.80	2358.4	18.8	2487.1	11.0	2593.9	11.8	2593.9	11.8
354U1451A-60F-100P #34 -Spot 303	289	275811	2.2	5.6771	0.6	11.1708	2.2	0.4601	2.1	0.96	2440.2	42.9	2537.5	20.5	2616.2	10.4	2616.2	10.4
354U1451A-60F-100P #34 -Spot 268	651	176662	2.8	5.5625	0.7	12.9375	1.3	0.5222	1.0	0.81	2708.3	22.4	2675.1	11.8	2650.1	12.2	2650.1	12.2
354U1451A-60F-100P #34 -Spot 95	190	86310	2.9	5.5429	0.8	11.6897	1.4	0.4701	1.2	0.81	2484.2	24.0	2579.9	13.4	2655.9	13.7	2655.9	13.7
354U1451A-60F-100P #34 -Spot 4	143	238510	1.2	5.3943	0.7	13.2510	1.2	0.5186	1.0	0.83	2693.4	22.3	2697.7	11.5	2700.9	11.3	2700.9	11.3
354U1451A-60F-100P #34 -Spot 281	169	64682	1.4	5.2285	0.6	13.8731	1.0	0.5263	0.8	0.77	2725.9	16.7	2741.1	9.2	2752.3	10.1	2752.3	10.1
354U1451A-60F-100P #34 -Spot 75	98	238057	1.6	5.0445	0.9	14.9235	1.5	0.5462	1.2	0.82	2809.5	28.1	2810.4	14.4	2811.0	14.2	2811.0	14.2
354U1451A-60F-100P #34 -Spot 176	353	1098804	1.0	4.9458	0.5	14.8281	1.2	0.5321	1.1	0.92	2750.4	23.9	2804.3	11.1	2843.3	7.6	2843.3	7.6
354U1451A-60F-100P #34 -Spot 128	418	945700	22.9	4.6861	0.8	16.2360	1.4	0.5520	1.2	0.82	2833.7	26.8	2890.8	13.6	2930.8	13.2	2930.8	13.2
354U1451A-60F-100P #34 -Spot 229	477	345139	1.6	2.9074	0.7	33.9627	1.4	0.7165	1.2	0.85	3482.8	32.2	3609.0	13.9	3679.7	11.2	3679.7	11.2

Table _____. U-Pb geochronologic analyses.																		
#19 U1451A_66F						Isotope ratios							Apparent ages (Ma)					
Late Miocene 8.4 Ma																		
Analysis	U	206Pb	U/Th	206Pb*	±	207Pb*	±	206Pb*	±	error	206Pb*	±	207Pb*	±	206Pb*	±	Best age	±
	(ppm)	204Pb		207Pb*	(%)	235U*	(%)	238U	(%)	corr.	238U*	(Ma)	235U	(Ma)	207Pb*	(Ma)	(Ma)	(Ma)
-354-U1451A-66F-CCW Spot 50	204	930	0.4	3.1893	1.3	0.1603	1.9	0.0037	1.4	0.72	23.9	0.3	151.0	2.7	3537.8	20.4	23.9	0.3
-354-U1451A-66F-CCW Spot 193	840	4205	186.3	22.7359	1.9	0.0234	2.2	0.0039	1.0	0.45	24.8	0.2	23.5	0.5	NA	NA	24.8	0.2
-354-U1451A-66F-CCW Spot 118	20	1855	12.0	6.1038	14.6	0.0973	15.1	0.0043	3.8	0.25	27.7	1.0	94.3	13.6	2494.9	247.7	27.7	1.0
-354-U1451A-66F-CCW Spot 265	2885	274151	2.4	21.4660	1.1	0.0345	1.5	0.0054	1.0	0.70	34.6	0.4	34.5	0.5	27.0	26.0	34.6	0.4
-354-U1451A-66F-CCW Spot 44	343	11851	0.8	21.9917	2.5	0.0459	2.7	0.0073	1.1	0.39	47.1	0.5	45.6	1.2	NA	NA	47.1	0.5
-354-U1451A-66F-CCW Spot 82	603	56895	1.3	21.3727	1.4	0.0476	1.6	0.0074	0.8	0.48	47.5	0.4	47.3	0.7	37.4	34.0	47.5	0.4
-354-U1451A-66F-CCW Spot 0	551	14114	0.9	20.7791	1.4	0.0506	1.8	0.0076	1.0	0.56	49.0	0.5	50.2	0.9	104.4	34.3	49.0	0.5
-354-U1451A-66F-CCW Spot 89	604	60438	1.3	21.1379	1.4	0.0502	1.7	0.0077	1.0	0.57	49.4	0.5	49.7	0.8	63.8	33.5	49.4	0.5
-354-U1451A-66F-CCW Spot 245	151	432367	1.5	19.5095	2.6	0.0594	2.9	0.0084	1.1	0.39	54.0	0.6	58.6	1.6	251.4	60.9	54.0	0.6
-354-U1451A-66F-CCW Spot 236	589	11116	1.3	21.1105	1.9	0.0598	2.1	0.0092	1.0	0.48	58.7	0.6	58.9	1.2	66.9	44.1	58.7	0.6
-354-U1451A-66F-CCW Spot 60	73	1717	1.3	19.5982	6.4	0.0666	7.2	0.0095	3.3	0.46	60.8	2.0	65.5	4.5	241.0	147.1	60.8	2.0
-354-U1451A-66F-CCW Spot 249	105	1989	1.3	24.1757	2.9	0.0681	3.2	0.0119	1.3	0.40	76.5	1.0	66.9	2.1	NA	NA	76.5	1.0
-354-U1451A-66F-CCW Spot 30	693	54500	1.1	20.8932	1.0	0.0875	1.4	0.0133	1.0	0.72	85.0	0.9	85.2	1.2	91.4	23.8	85.0	0.9
-354-U1451A-66F-CCW Spot 303	110	4196	1.4	22.5212	5.0	0.0826	5.2	0.0135	1.4	0.27	86.4	1.2	80.6	4.0	NA	NA	86.4	1.2
-354-U1451A-66F-CCW Spot 299	886	102725	47.0	15.7768	1.1	0.1246	1.8	0.0143	1.5	0.80	91.3	1.3	119.3	2.1	720.2	23.2	91.3	1.3
-354-U1451A-66F-CCW Spot 150	101	8369	1.7	21.7533	2.3	0.1295	2.6	0.0204	1.1	0.42	130.5	1.4	123.7	3.0	NA	NA	130.5	1.4
-354-U1451A-66F-CCW Spot 224	690	41188	2.0	19.3239	0.8	0.2971	1.1	0.0417	0.8	0.73	263.1	2.1	264.1	2.6	273.3	17.8	263.1	2.1
-354-U1451A-66F-CCW Spot 283	2014	784650	5.3	17.5546	0.6	0.3285	1.3	0.0418	1.2	0.89	264.2	3.1	288.4	3.4	489.2	13.3	264.2	3.1
-354-U1451A-66F-CCW Spot 38	223	70931	2.2	18.2235	0.9	0.4816	1.3	0.0637	0.9	0.68	398.0	3.4	399.2	4.2	406.1	21.0	398.0	3.4
-354-U1451A-66F-CCW Spot 292	1328	128796	18.6	17.5983	0.6	0.5641	1.0	0.0720	0.8	0.80	448.4	3.3	454.2	3.5	483.8	12.7	448.4	3.3
-354-U1451A-66F-CCW Spot 8	620	69310	9.2	17.6326	0.8	0.5732	1.5	0.0733	1.3	0.87	456.2	5.9	460.1	5.7	479.4	16.8	456.2	5.9
-354-U1451A-66F-CCW Spot 247	2289	1076653	33.4	17.4688	0.7	0.5803	1.3	0.0736	1.1	0.84	457.5	4.9	464.7	4.9	500.0	15.5	457.5	4.9
-354-U1451A-66F-CCW Spot 73	636	43561	9.1	17.7898	0.7	0.5703	1.0	0.0736	0.6	0.64	457.9	2.7	458.2	3.5	459.8	16.4	457.9	2.7
-354-U1451A-66F-CCW Spot 221	4545	1146135	489.7	17.3703	0.5	0.5891	1.0	0.0742	0.8	0.84	461.7	3.6	470.3	3.6	512.5	11.5	461.7	3.6
-354-U1451A-66F-CCW Spot 159	1640	212733	29.3	17.3656	0.7	0.6019	1.2	0.0758	1.0	0.82	471.3	4.6	478.5	4.7	513.1	15.4	471.3	4.6
-354-U1451A-66F-CCW Spot 12	589	55410	8.4	17.5954	0.8	0.5954	1.1	0.0760	0.7	0.68	472.3	3.4	474.3	4.1	484.1	17.4	472.3	3.4
-354-U1451A-66F-CCW Spot 164	431	121213	1.2	17.5310	0.7	0.6024	1.8	0.0766	1.7	0.93	475.9	7.7	478.7	6.9	492.2	15.0	475.9	7.7
-354-U1451A-66F-CCW Spot 40	428	100363	4.7	17.5829	0.6	0.6027	1.2	0.0769	1.0	0.85	477.5	4.7	478.9	4.6	485.7	13.9	477.5	4.7
-354-U1451A-66F-CCW Spot 83	604	1103579	9.5	17.5695	0.7	0.6038	1.1	0.0770	0.9	0.78	478.0	4.1	479.6	4.4	487.3	15.8	478.0	4.1
-354-U1451A-66F-CCW Spot 139	1286	46013	0.7	17.0448	1.2	0.6247	2.2	0.0773	1.8	0.84	479.7	8.5	492.8	8.5	553.9	25.6	479.7	8.5
-354-U1451A-66F-CCW Spot 223	708	171022	10.7	17.4883	0.6	0.6109	1.3	0.0775	1.1	0.87	481.3	5.2	484.1	5.0	497.5	14.0	481.3	5.2
-354-U1451A-66F-CCW Spot 129	849	442211	8.9	17.3697	0.6	0.6178	1.1	0.0779	0.8	0.80	483.4	3.9	488.5	4.1	512.5	13.9	483.4	3.9
-354-U1451A-66F-CCW Spot 170	204	36691	2.9	17.2403	1.1	0.6243	1.6	0.0781	1.1	0.73	484.8	5.3	492.6	6.1	529.0	23.3	484.8	5.3
-354-U1451A-66F-CCW Spot 168	1155	224482	3.3	17.7427	0.7	0.6107	1.6	0.0786	1.4	0.91	487.9	6.8	484.0	6.1	465.7	14.6	487.9	6.8
-354-U1451A-66F-CCW Spot 93	349	45813	1.9	17.6557	0.8	0.6183	1.2	0.0792	0.9	0.75	491.4	4.2	488.8	4.6	476.5	17.3	491.4	4.2
-354-U1451A-66F-CCW Spot 289	57	38329	0.6	16.9403	1.3	0.6527	1.6	0.0802	0.9	0.55	497.5	4.1	510.1	6.3	567.3	28.3	497.5	4.1
-354-U1451A-66F-CCW Spot 108	1391	1266397	12.7	17.4453	0.7	0.6353	1.2	0.0804	1.0	0.80	498.6	4.6	499.4	4.7	503.0	15.7	498.6	4.6
-354-U1451A-66F-CCW Spot 288	281	131414	0.4	17.3210	0.7	0.6408	1.3	0.0805	1.1	0.86	499.3	5.4	502.8	5.2	518.7	15.0	499.3	5.4
-354-U1451A-66F-CCW Spot 142	156	94993	0.8	17.3432	0.8	0.6403	1.1	0.0806	0.8	0.73	499.5	4.0	502.5	4.5	515.9	17.0	499.5	4.0
-354-U1451A-66F-CCW Spot 246	421	82056	0.8	17.2256	0.7	0.6471	1.3	0.0809	1.1	0.84	501.4	5.2	506.7	5.1	530.8	15.2	501.4	5.2
-354-U1451A-66F-CCW Spot 78	449	1646844	1.2	17.1937	0.7	0.6524	1.3	0.0814	1.1	0.86	504.4	5.4	510.0	5.2	534.9	14.4	504.4	5.4
-354-U1451A-66F-CCW Spot 26	337	59336	0.6	17.1877	0.9	0.6543	2.0	0.0816	1.8	0.89	505.7	8.8	511.1	8.1	535.7	20.0	505.7	8.8
-354-U1451A-66F-CCW Spot 105	219	27831	0.5	17.5449	1.0	0.6426	1.4	0.0818	1.0	0.71	506.9	4.9	503.9	5.6	490.4	21.9	506.9	4.9
-354-U1451A-66F-CCW Spot 206	1945	537423	1.7	17.2941	0.5	0.6552	0.9	0.0822	0.8	0.88	509.3	4.0	511.6	3.8	522.1	10.0	509.3	4.0
-354-U1451A-66F-CCW Spot 131	168	30545	1.4	17.3172	0.9	0.6730	1.5	0.0846	1.1	0.78	523.3	5.7	522.6	5.9	519.2	19.9	523.3	5.7
-354-U1451A-66F-CCW Spot 287	299	73705	1.2	17.2512	0.7	0.6757	1.1	0.0846	0.8	0.79	523.4	4.2	524.2	4.4	527.6	14.4	523.4	4.2
-354-U1451A-66F-CCW Spot 204	790	107242	3.4	16.9437	0.5	0.6888	0.8	0.0847	0.6	0.75	524.0	2.8	532.1	3.1	566.9	10.9	524.0	2.8
-354-U1451A-66F-CCW Spot 192	261	170833	1.9	17.1342	0.7	0.6819	1.0	0.0848	0.8	0.76	524.6	3.9	527.9	4.2	542.5	14.3	524.6	3.9
-354-U1451A-66F-CCW Spot 158	697	109091	1.6	17.2897	0.7	0.6828	1.2	0.0857	1.0	0.83	529.8	5.1	528.4	5.0	522.7	15.0	529.8	5.1
-354-U1451A-66F-CCW Spot 157	248	92072	0.5	17.0773	0.8	0.6985	1.3	0.0866	1.0	0.77	535.1	5.1	537.9	5.4	549.7	17.8	535.1	5.1
-354-U1451A-66F-CCW Spot 174	3251	24120455	8.5	16.6134	0.5	0.7334	1.1	0.0884	0.9	0.87	546.1	4.8	558.6	4.6	609.5	11.3	546.1	4.8
-354-U1451A-66F-CCW Spot 36	629	135823	2.0	17.1868	0.6	0.7172	1.2	0.0894	1.0	0.85	552.2	5.2	549.0	5.0	535.7	13.6	552.2	5.2
-354-U1451A-66F-CCW Spot 226	1464	632583	2.5	17.0945	0.6	0.7230	1.0	0.0897	0.8	0.81	553.6	4.4	552.4	4.4	547.5	13.2	553.6	4.4
-354-U1451A-66F-CCW Spot 143	972	399865	0.7	17.1978	0.7	0.7198	1.4	0.0898	1.2	0.86	554.5	6.4	550.6	6.0	534.4	15.7	554.5	6.4
-354-U1451A-66F-CCW Spot 219	952	146313	6.8	16.0079	1.3	0.7861	1.7	0.0913	1.2	0.68	563.3	6.4	589.0	7.7	689.3	26.9	563.3	6.4
-354-U1451A-66F-CCW Spot 120	167	34320	0.4	16.7323	0.9	0.7598	1.2	0.0922	0.9	0.71	568.8	4.7	573.9	5.3	594.1	18.5	568.8	4.7
-354-U1451A-66F-CCW Spot 259	859	178640	7.4	16.8413	0.6	0.7723	1.0	0.0944	0.9	0.82	581.3	4.8	581.1	4.6	580.0	13.0	581.3	4.8
-354-U1451A-66F-CCW Spot 286	1103	124699	4.5	16.1885	0.6	0.8147	0.9	0.0957	0.7	0.79	589.1	4.1	605.1	4.2	665.3	11.8	589.1	4.1
-354-U1451A-66F-CCW Spot 239	507	1384602	26.9	16.5856	0.7	0.8024	1.4	0.0966	1.2	0.85	594.3	6.5	598.2	6.1	613.2	15.5	594.3	6.5
-354-U1451A-66F-CCW Spot 176	331	548534	6.6	16.6656	0.7	0.8214	1.3	0.0993	1.1	0.84	610.5	6.6	608.9	6.2	602.8	15.8	610.5	6.6
-354-U1451A-66F-CCW Spot 187	2194	3288855	44.2	16.2216	0.6	0.8463	1.1	0.0996	0.9	0.86	612.1	5.5	622.6	5.1	660.9	12.0	612.1	5.5
-354-U1451A-66F-CCW Spot 309	901	148030	2.3	16.2803	0.6	0.8576	1.2	0.1013	1.0	0.86	622.1	6.0	628.8	5.5	653.2	13.1	622.1	6.0
-354-U1451A-66F-CCW Spot 308	353	602595	3.9	16.4524	0.6	0.8547	1.0	0.1020	0.8	0.80	626.3	4.8	627.3	4.7	630.6	13.0	626.3	4.8
-354-U1451A-66F-CCW Spot 114	38	12137	41.3	15.9573	2.1	0.8993	2.9	0.1041	2.0	0.69	638.5	12.3	651.3	14.0	696.0	44.7	638.5	12.3
-354-U1451A-66F-CCW Spot 225	3440	1634530	39.1	15.8963	0.6	0.9155	0.9	0.1056	0.6	0.69	647.1	3.8	660.0	4.4	704.2	13.8	647.1	3.8
-354-U1451A-66F-CCW Spot 127	113	18722	2.5	16.0276	0.8	0.9399	1.1	0.1093	0.8	0.68	668.7	5.0	672.8	5.7	686.7	18.0	668.7	5.0
-354-U1451A-66F-CCW Spot 65	230	79753	3.2	16.1314	0.7	0.9474	1.3	0.1109	1.1	0.85	677.9	7.4	676.7	6.6	672.9	14.9	677.9	7.4
-354-U1451A-66F-CCW Spot 242	1440	652278	1.4	15.3522	0.5	1.0473	0.9	0.1167	0.7	0.80	711.3	4.8	727.5	4.7	777.8	11.4	711.3	4.8
-354-U1451A-66F-CCW Spot 173	277	117136	2.9	15.8602	0.7	1.0155	1.3	0.1169	1.1	0.87	712.5	7.6	711.6	6.7	709.0	13.9	712.5	7.6
-354-U1451A-66F-CCW Spot 49	1258	167423	31.4	15.4087	0.6	1.0583	1.2	0.1183	1.1	0.86	720.9	7.3	733.0	6.5	770.1	13.2	720.9	7.3
-354-U1451A-66F-CCW Spot 134	483	133665	1.0	15.5194	0.7	1.0792	1.5	0.1215	1.3	0.87	739.4	9.0	743.3	7.8	755.0	15.6	739.4	9.0
-354-U1451A-66F-CCW Spot 96	510	820829	1.3	15.2702	0.6	1.1288	1.3	0.1251	1.2	0.87	759.7	8.3	767.2	7.1	789.1	13.5	759.7	8.3
-354-U1451A-66F-CCW Spot 161	124	42937	0.9	15.1092	0.7	1.1699	1.1	0.1283	0.8	0.76	777.9	6.1	786.6	6.1	811.3	15.1	777.9	6.1
-354-U1451A-66F-CCW Spot 7	342	79433	0.7	14.9330	0.5	1.1939	0.9	0.1294	0.7	0.80	784.2	5.5	797.8	5.1	835.8	11.4	784.2	5.5
-354-U1451A-66F-CCW Spot 47	831	715750	6.9	14.9838	0.6	1.1977	1.2	0.1302	1.1	0.86	789.1	7.9	799.5	6.8	828.7	12.9	789.1	7.9
-354-U1451A-66F-CCW Spot 313	177	158681	2.1	15.0747	0.7	1.1918	1.0	0.1304	0.8	0.77	789.9	6.0	796.8	5.8	816.1	14.1	789.9	6.0
-354-U1451A-66F-CCW Spot 280	261	2322208	1.5	15.1923	0.5	1.1850	0.9	0.1306	0.8	0.84	791.5	5.6	793.7	5.0	799.8	10.4	791.5	5.6
-354-U1451A-66F-CCW Spot 125	991	282740	2.2	14.9273	0.6	1.2109	1.3	0.1312	1.2	0.90	794.5	8.7	805.6	7.2	836.6	11.9	794.5	8.7
-354-U1451A-66F-CCW Spot 77	501	348448	7.5	14.9385	0.6	1.2128	1.2	0.1315	1.1	0.89	796.2	8.2	806.5	6.9	835.0	11.6	796.2	8.2
-354-U1451A-66F-CCW Spot 248	102	429309	0.9	15.1463	0.7	1.1963	1.0	0.1315	0.7	0.71	796.3	5.4	798.9	5.6	806.2	15.1	796.3	5.4
-354-U1451A-66F-CCW Spot 152	368	149852	2.1	14.8813	0.5	1.2249	1.0	0.1323	0.8	0.84	800.7	6.2	812.0	5.5	843.0	10.9	800.7	6.2
-354-U1451A-66F-CCW Spot 75	768	758104	2.4	15.2006	0.5	1.2001	1.0	0.1324	0.8	0.83	801.3	6.1	800.6	5.4	798.7	11.5	801.3	6.1
-354-U1451A-66F-CCW Spot 20	78	15916	0.8	15.2837	1.3	1.1957	1.5	0.1326	0.8	0.55	802.6	6.3	798.6	8.5	787.2	27.1	802.6	6.3
-354-U1451A-66F-CCW Spot 207	320	270014	2.3	14.7426	0.6	1.2597	1.2	0.1348	1.0	0.88	814.9	7.8	827.8	6.6	862.5	11.6	814.9	7.8
-354-U1451A-66F-CCW Spot 39	777	216917	1.0	15.2819	0.5	1.2173	1.0	0.1350	0.8	0.82	816.2	6.1	808.6	5.4	787.5	11.4	816.2	6.1
-354-U1451A-66F-CCW Spot 25	1330	287297	69.6	14.9933	0.8	1.2564	1.3	0.1367	1.1	0.81	825.9	8.2	826.3	7.4	827.4	16.0	825.9	8.2
-354-U1451A-66F-CCW Spot 3	861	184145	10.7	14.7070	0.6	1.2877	1.5	0.1374	1.4	0.92	830.0	10.6	840.3	8.4	867.5	11.7	830.0	10.6
-354-U1451A-66F-CCW Spot 171	407	1451410	3.1	14.5248	0.5	1.3067	1.3	0.1377	1.2	0.92	831.7	9.5	848.7	7.6	893.3	10.5	831.7	9.5
-354-U1451A-66F-CCW Spot 294	92	362209	2.4	14.9938	0.8	1.2668	1.2	0.1378	0.8	0.72	832.3	6.6	831.0	6.6	827.3	16.9	832.3	6.6
-354-U1451A-66F-CCW Spot 295	2306	403750	2.1	14.3086	0.5	1.3435	1.1	0.1395	1.0	0.89	841.7	7.9	864.7	6.6	924.2	10.8	841.7	7.9
-354-U1451A-66F-CCW Spot 153	321	121140	2.4	14.7330	0.7	1.3207	1.2	0.1412	1.0	0.80	851.3	7.8	854.8	7.0	863.8	14.9	851.3	7.8
-354-U1451A-66F-CCW Spot 293	58	22464	1.2	14.8758	1.0	1.3122	1.4	0.1416	1.0	0.70	853.9	7.9	851.1	8.2	843.8	21.3	853.9	7.9
-354-U1451A-66F-CCW Spot 194	918	929115	4.1	14.5660	0.5	1.3425	1.6	0.1419	1.5	0.95	855.3	11.9	864.3	9.1	887.4	9.7	855.3	11.9
-354-U1451A-66F-CCW Spot 195	577	168164	3.3	14.3196	0.6	1.3735	1.1	0.1427	1.0	0.86	860.0	7.8	877.7	6.7	922.6	12.1	860.0	7.8

-354-U1451A-66F-CCW Spot 276	116	1473134	1.6	14.5743	0.5	1.3516	0.8	0.1429	0.6	0.75	861.2	4.9	868.3	4.7	886.2	11.1	861.2	4.9
-354-U1451A-66F-CCW Spot 106	52	29983	0.4	14.9690	1.2	1.3195	1.6	0.1433	1.0	0.64	863.4	8.1	854.3	9.0	830.8	25.0	863.4	8.1
-354-U1451A-66F-CCW Spot 137	846	158785	18.0	14.6931	0.5	1.3496	1.0	0.1439	0.8	0.83	866.6	6.6	867.4	5.7	869.4	11.1	866.6	6.6
-354-U1451A-66F-CCW Spot 300	194	96646	1.6	14.6879	0.6	1.3533	1.1	0.1442	0.8	0.79	868.5	6.8	869.0	6.2	870.2	13.4	868.5	6.8
-354-U1451A-66F-CCW Spot 55	51	33110	1.2	14.8136	1.2	1.3442	1.5	0.1445	0.9	0.62	869.9	7.7	865.0	8.9	852.5	24.8	869.9	7.7
-354-U1451A-66F-CCW Spot 24	609	377821	1.0	14.4922	0.5	1.3862	1.0	0.1458	0.8	0.87	877.2	6.8	883.1	5.7	897.9	9.9	877.2	6.8
-354-U1451A-66F-CCW Spot 177	164	81012	2.5	14.7131	0.6	1.3757	1.1	0.1469	0.9	0.83	883.3	7.8	878.6	6.7	866.7	13.3	883.3	7.8
-354-U1451A-66F-CCW Spot 67	315	213991	4.0	14.6584	0.8	1.3816	1.3	0.1469	1.1	0.81	883.8	8.7	881.1	7.7	874.4	16.0	883.8	8.7
-354-U1451A-66F-CCW Spot 111	124	190277	1.7	14.5832	0.9	1.3921	1.3	0.1473	1.0	0.75	885.8	8.2	885.6	7.8	885.0	18.0	885.8	8.2
-354-U1451A-66F-CCW Spot 264	295	233474	2.2	14.6696	0.6	1.3931	1.0	0.1483	0.8	0.80	891.3	6.7	886.0	6.0	872.8	12.6	891.3	6.7
-354-U1451A-66F-CCW Spot 6	301	1179186	2.8	14.4379	0.5	1.4374	1.0	0.1506	0.9	0.87	904.2	7.6	904.6	6.2	905.6	10.7	905.6	10.7
-354-U1451A-66F-CCW Spot 209	426	152168	3.9	14.3279	0.6	1.5157	1.0	0.1576	0.8	0.80	943.3	7.2	936.8	6.2	921.4	12.4	921.4	12.4
-354-U1451A-66F-CCW Spot 275	193	64763	2.5	14.3070	0.8	1.4499	1.2	0.1505	0.9	0.74	903.9	7.8	909.8	7.5	924.4	17.2	924.4	17.2
-354-U1451A-66F-CCW Spot 202	1466	1804484	4.1	14.2528	0.7	1.4611	1.2	0.1511	1.0	0.83	907.1	8.3	914.5	7.1	932.2	13.4	932.2	13.4
-354-U1451A-66F-CCW Spot 230	81	88769	0.9	14.2473	0.8	1.5703	1.1	0.1623	0.7	0.66	969.7	6.6	958.6	6.8	933.0	16.9	933.0	16.9
-354-U1451A-66F-CCW Spot 126	750	781035	6.1	14.1575	0.5	1.5666	1.0	0.1609	0.8	0.86	961.9	7.6	957.1	6.1	945.9	10.3	945.9	10.3
-354-U1451A-66F-CCW Spot 102	376	96574	1.5	14.1148	0.6	1.4943	1.0	0.1530	0.9	0.83	918.0	7.4	928.1	6.3	952.1	11.8	952.1	11.8
-354-U1451A-66F-CCW Spot 169	655	1657905	2.5	14.1013	0.4	1.5678	1.0	0.1604	0.9	0.90	959.1	7.8	957.6	6.0	954.1	8.6	954.1	8.6
-354-U1451A-66F-CCW Spot 222	1753	287185	14.8	14.0651	0.6	1.4872	1.2	0.1518	1.1	0.88	910.9	9.1	925.2	7.4	959.4	12.0	959.4	12.0
-354-U1451A-66F-CCW Spot 99	749	148702	2.4	14.0577	0.6	1.5644	1.2	0.1596	1.1	0.89	954.4	9.7	956.2	7.6	960.4	11.6	960.4	11.6
-354-U1451A-66F-CCW Spot 15	598	302417	1.7	14.0502	0.6	1.4413	1.1	0.1469	0.9	0.83	883.7	7.4	906.2	6.5	961.5	12.5	961.5	12.5
-354-U1451A-66F-CCW Spot 10	549	113416	2.8	14.0356	0.6	1.5604	1.1	0.1589	1.0	0.83	950.7	8.4	954.6	7.1	963.6	12.9	963.6	12.9
-354-U1451A-66F-CCW Spot 254	1035	473932	5.2	14.0324	0.5	1.5625	0.9	0.1591	0.7	0.80	951.7	6.4	955.5	5.6	964.1	11.1	964.1	11.1
-354-U1451A-66F-CCW Spot 178	864	261012	6.3	14.0122	0.5	1.6281	0.9	0.1655	0.8	0.85	987.4	7.1	981.1	5.7	967.0	9.9	967.0	9.9
-354-U1451A-66F-CCW Spot 74	389	101280	0.9	13.9979	0.6	1.6033	1.0	0.1628	0.8	0.82	972.5	7.5	971.5	6.3	969.1	11.9	969.1	11.9
-354-U1451A-66F-CCW Spot 151	582	225161	1.1	13.9976	0.5	1.6251	1.3	0.1651	1.2	0.92	984.8	11.3	980.0	8.4	969.2	10.5	969.2	10.5
-354-U1451A-66F-CCW Spot 160	190	57364	1.0	13.9959	0.7	1.6338	1.0	0.1659	0.8	0.76	989.6	7.1	983.3	6.4	969.4	13.5	969.4	13.5
-354-U1451A-66F-CCW Spot 231	912	1599604	1.3	13.9934	0.5	1.5920	0.9	0.1616	0.8	0.84	965.9	6.8	967.1	5.6	969.8	9.9	969.8	9.9
-354-U1451A-66F-CCW Spot 262	308	32167590	1.9	13.9767	0.5	1.6213	0.8	0.1644	0.7	0.82	981.3	6.1	978.5	5.1	972.2	9.3	972.2	9.3
-354-U1451A-66F-CCW Spot 232	515	214437	3.6	13.9669	0.6	1.5753	0.9	0.1596	0.7	0.77	954.8	6.1	960.5	5.5	973.7	11.4	973.7	11.4
-354-U1451A-66F-CCW Spot 228	273	135995	3.3	13.9572	0.5	1.5724	1.0	0.1592	0.9	0.87	952.5	7.8	959.4	6.3	975.1	10.3	975.1	10.3
-354-U1451A-66F-CCW Spot 17	157	22584	1.2	13.9543	0.8	1.6447	1.1	0.1665	0.7	0.68	993.0	6.6	987.5	6.6	975.5	15.7	975.5	15.7
-354-U1451A-66F-CCW Spot 71	224	71187	0.6	13.9447	0.6	1.6612	1.2	0.1681	1.0	0.87	1001.5	9.5	993.8	7.4	976.9	11.9	976.9	11.9
-354-U1451A-66F-CCW Spot 56	49	103742	2.3	13.9376	1.0	1.6256	1.3	0.1644	0.8	0.64	981.1	7.6	980.1	8.2	977.9	20.4	977.9	20.4
-354-U1451A-66F-CCW Spot 21	802	231881	1.9	13.9341	0.5	1.6330	1.1	0.1651	1.0	0.88	985.1	9.0	983.0	7.0	978.4	10.8	978.4	10.8
-354-U1451A-66F-CCW Spot 307	587	163699	1.1	13.9301	0.5	1.5952	1.1	0.1612	1.0	0.88	963.6	8.9	968.3	7.1	979.0	11.0	979.0	11.0
-354-U1451A-66F-CCW Spot 199	161	38488	1.4	13.9244	0.7	1.5418	1.1	0.1558	0.9	0.80	933.2	7.9	947.2	7.0	979.9	14.0	979.9	14.0
-354-U1451A-66F-CCW Spot 260	131	34069	2.4	13.8674	1.0	1.4167	1.7	0.1425	1.4	0.82	859.1	11.1	896.0	10.0	988.2	19.5	988.2	19.5
-354-U1451A-66F-CCW Spot 80	495	46123	9.4	13.8562	0.9	1.4375	1.2	0.1445	0.8	0.66	870.2	6.2	904.7	6.9	989.9	17.6	989.9	17.6
-354-U1451A-66F-CCW Spot 115	323	166698	2.3	13.8493	0.6	1.6097	0.9	0.1618	0.8	0.81	966.5	6.8	974.0	5.9	990.9	11.3	990.9	11.3
-354-U1451A-66F-CCW Spot 256	229	140977	1.3	13.8491	0.6	1.6825	1.1	0.1691	0.9	0.84	1007.0	8.3	1001.9	6.8	990.9	11.8	990.9	11.8
-354-U1451A-66F-CCW Spot 92	469	218089	0.7	13.8334	0.6	1.6815	1.4	0.1688	1.3	0.89	1005.3	11.8	1001.5	9.1	993.2	13.2	993.2	13.2
-354-U1451A-66F-CCW Spot 70	125	95990	2.2	13.7889	0.7	1.6163	1.1	0.1617	0.9	0.80	966.3	8.2	976.5	7.2	999.7	14.0	999.7	14.0
-354-U1451A-66F-CCW Spot 217	185	442858	1.0	13.7244	0.8	1.6602	1.3	0.1653	1.1	0.80	986.3	9.7	993.5	8.4	1009.2	15.9	1009.2	15.9
-354-U1451A-66F-CCW Spot 210	92	61447	0.7	13.6852	0.8	1.7349	1.2	0.1723	0.8	0.72	1024.6	7.9	1021.6	7.4	1015.0	16.2	1015.0	16.2
-354-U1451A-66F-CCW Spot 285	691	371030	2.2	13.6757	0.5	1.7058	0.9	0.1693	0.8	0.84	1008.0	7.3	1010.7	6.0	1016.5	10.3	1016.5	10.3
-354-U1451A-66F-CCW Spot 16	245	175327	1.9	13.5695	0.6	1.6878	1.1	0.1662	0.9	0.83	991.0	8.2	1003.9	6.9	1032.2	12.2	1032.2	12.2
-354-U1451A-66F-CCW Spot 205	783	3292330	1.7	13.5154	0.5	1.7504	0.9	0.1717	0.8	0.85	1021.2	7.5	1027.3	6.0	1040.3	9.8	1040.3	9.8
-354-U1451A-66F-CCW Spot 220	66	42404	2.8	13.4921	1.0	1.8662	1.3	0.1827	0.9	0.69	1081.7	9.1	1069.2	8.8	1043.8	19.5	1043.8	19.5
-354-U1451A-66F-CCW Spot 191	696	3534224	5.1	13.4507	0.5	1.4711	1.4	0.1436	1.3	0.93	864.8	10.3	918.6	8.2	1050.0	9.9	1050.0	9.9
-354-U1451A-66F-CCW Spot 51	935	742876	1.4	13.4256	0.5	1.8330	1.2	0.1786	1.1	0.89	1059.1	10.4	1057.3	7.9	1053.8	11.1	1053.8	11.1
-354-U1451A-66F-CCW Spot 29	1952	221419	8.8	13.4133	0.5	1.6198	1.0	0.1576	0.8	0.84	943.7	7.3	977.9	6.2	1055.6	10.8	1055.6	10.8
-354-U1451A-66F-CCW Spot 163	434	252219	1.7	13.3829	0.6	1.7411	1.1	0.1691	0.9	0.85	1007.0	8.7	1023.9	7.1	1060.2	11.9	1060.2	11.9
-354-U1451A-66F-CCW Spot 213	373	250805	8.8	13.3362	0.5	1.8787	1.0	0.1818	0.8	0.85	1076.8	8.4	1073.6	6.6	1067.2	10.4	1067.2	10.4
-354-U1451A-66F-CCW Spot 181	676	528325	19.1	13.2868	1.4	1.6552	1.8	0.1596	1.1	0.64	954.4	10.1	991.5	11.3	1074.7	27.5	1074.7	27.5
-354-U1451A-66F-CCW Spot 251	187	117048	0.8	13.2769	0.7	1.7422	1.2	0.1678	0.9	0.81	1000.2	8.6	1024.3	7.4	1076.1	13.6	1076.1	13.6
-354-U1451A-66F-CCW Spot 72	2340	13068360	1.8	13.2702	0.7	1.7482	1.0	0.1683	0.8	0.76	1002.9	7.2	1026.5	6.5	1077.2	13.2	1077.2	13.2
-354-U1451A-66F-CCW Spot 37	438	109514	1.1	13.2479	0.7	1.9058	1.2	0.1832	1.0	0.84	1084.4	10.1	1083.1	8.1	1080.5	13.4	1080.5	13.4
-354-U1451A-66F-CCW Spot 19	1062	247682	4.4	13.2334	0.5	1.9906	0.9	0.1911	0.7	0.80	1127.5	7.3	1112.3	6.0	1082.8	10.7	1082.8	10.7
-354-U1451A-66F-CCW Spot 272	676	987558	1.4	13.1976	0.6	1.9244	1.0	0.1843	0.8	0.79	1090.3	8.0	1089.6	6.8	1088.2	12.4	1088.2	12.4
-354-U1451A-66F-CCW Spot 306	1597	138368	1.8	13.1871	0.7	1.8650	1.3	0.1784	1.1	0.85	1058.5	10.9	1068.8	8.7	1089.7	13.9	1089.7	13.9
-354-U1451A-66F-CCW Spot 166	252	152720	2.2	13.1477	0.7	1.9306	1.3	0.1842	1.1	0.84	1089.8	11.2	1091.8	8.8	1095.7	14.2	1095.7	14.2
-354-U1451A-66F-CCW Spot 32	337	361203	3.5	13.1452	0.7	1.9490	1.2	0.1859	1.0	0.83	1099.1	10.1	1098.1	8.1	1096.1	13.3	1096.1	13.3
-354-U1451A-66F-CCW Spot 90	473	176093	2.0	13.1259	0.6	2.0527	1.1	0.1955	1.0	0.87	1151.1	10.2	1133.2	7.6	1099.1	11.2	1099.1	11.2
-354-U1451A-66F-CCW Spot 147	488	110199	5.1	13.1257	0.4	1.9313	0.9	0.1839	0.8	0.88	1088.4	8.4	1092.0	6.3	1099.1	8.9	1099.1	8.9
-354-U1451A-66F-CCW Spot 64	827	29461	1.2	13.1150	0.8	1.5818	1.1	0.1505	0.8	0.71	903.9	6.7	963.1	7.0	1100.8	15.8	1100.8	15.8
-354-U1451A-66F-CCW Spot 301	479	4694652	1.7	13.1072	0.7	1.9271	1.8	0.1833	1.6	0.92	1084.8	16.3	1090.5	11.8	1101.9	13.4	1101.9	13.4
-354-U1451A-66F-CCW Spot 88	281	239747	1.3	13.0822	0.7	1.9454	1.0	0.1847	0.8	0.76	1092.4	7.8	1096.8	6.8	1105.7	13.3	1105.7	13.3
-354-U1451A-66F-CCW Spot 162	1574	276286	2.7	13.0800	0.4	1.6282	1.1	0.1545	1.0	0.94	926.3	8.8	981.2	6.8	1106.1	7.1	1106.1	7.1
-354-U1451A-66F-CCW Spot 212	93	58563	1.5	13.0497	0.7	1.9292	1.1	0.1827	0.8	0.74	1081.5	8.0	1091.3	7.3	1110.7	14.8	1110.7	14.8
-354-U1451A-66F-CCW Spot 189	888	667973	2.2	13.0373	0.5	1.7398	1.0	0.1646	0.9	0.87	982.2	7.8	1023.4	6.4	1112.6	9.7	1112.6	9.7
-354-U1451A-66F-CCW Spot 119	72	58027	1.4	12.9333	0.7	2.0624	1.2	0.1935	1.0	0.80	1140.5	10.1	1136.4	8.3	1128.6	14.6	1128.6	14.6
-354-U1451A-66F-CCW Spot 28	138	164870	0.9	12.9264	0.7	1.9235	1.1	0.1804	0.9	0.78	1069.2	8.7	1089.3	7.6	1129.6	14.0	1129.6	14.0
-354-U1451A-66F-CCW Spot 179	515	573026	4.1	12.9028	1.4	1.8991	1.8	0.1778	1.2	0.65	1054.9	11.3	1080.8	11.9	1133.3	27.2	1133.3	27.2
-354-U1451A-66F-CCW Spot 110	1380	210038	1.6	12.8742	0.6	1.9859	1.1	0.1855	0.9	0.83	1097.0	9.3	1110.7	7.5	1137.7	12.1	1137.7	12.1
-354-U1451A-66F-CCW Spot 22	999	210318	2.0	12.8654	0.6	1.8520	1.3	0.1729	1.2	0.88	1028.0	11.1	1064.1	8.7	1139.0	12.3	1139.0	12.3
-354-U1451A-66F-CCW Spot 116	522	538790	2.3	12.8645	0.5	2.0895	1.0	0.1950	0.9	0.86	1148.6	9.4	1145.4	7.1	1139.2	10.3	1139.2	10.3
-354-U1451A-66F-CCW Spot 180	1104	1056018	1.4	12.8335	0.6	1.9098	2.2	0.1778	2.1	0.96	1055.1	20.2	1084.5	14.4	1144.0	11.6	1144.0	11.6
-354-U1451A-66F-CCW Spot 273	560	25205651	1.8	12.8313	0.7	2.0700	1.4	0.1927	1.3	0.87	1136.1	13.1	1138.9	9.9	1144.3	14.4	1144.3	14.4
-354-U1451A-66F-CCW Spot 244	1254	2577191	11.0	12.8146	0.7	2.0780	1.1	0.1932	0.9	0.78	1138.8	9.1	1141.6	7.6	1146.9	13.8	1146.9	13.8
-354-U1451A-66F-CCW Spot 297	595	569493	0.7	12.7955	0.4	2.0862	0.9	0.1937	0.7	0.86	1141.3	7.7	1144.3	5.8	1149.9	8.5	1149.9	8.5
-354-U1451A-66F-CCW Spot 208	233	118984	2.5	12.7564	0.5	2.0708	1.0	0.1917	0.9	0.87	1130.4	9.2	1139.2	7.0	1155.9	9.9	1155.9	9.9
-354-U1451A-66F-CCW Spot 281	2214	865926	10.3	12.7451	0.6	1.9835	1.1	0.1834	0.9	0.84	1085.7	9.1	1109.9	7.3	1157.7	11.5	1157.7	11.5
-354-U1451A-66F-CCW Spot 237	513	2168758	1.7	12.7415	0.7	2.0357	1.3	0.1882	1.1	0.85	1111.6	10.8	1127.5	8.5	1158.3	13.3	1158.3	13.3
-354-U1451A-66F-CCW Spot 27	834	527995	7.8	12.6972	0.7	1.7550	1.6	0.1617	1.4	0.89	966.1	12.5	1029.0	10.1	1165.2	13.9	1165.2	13.9
-354-U1451A-66F-CCW Spot 284	466	997623	3.2	12.6616	0.7	2.2416	1.3	0.2059	1.1	0.83	1207.1	11.8	1194.2	9.1	1170.8	14.5	1170.8	14.5
-354-U1451A-66F-CCW Spot 182	254	111239	3.8	12.6507	0.6	2.1420	1.0	0.1966	0.8	0.80	1157.1	8.2	1162.5	6.7	1172.5	11.4	1172.5	11.4
-354-U1451A-66F-CCW Spot 103	69	86039	0.5	12.6127	0.7	2.1602	1.3	0.1977	1.1	0.83	1162.9	11.8	1168.3	9.3	1178.4	14.8	1178.4	14.8
-354-U1451A-66F-CCW Spot 255	393	208062	1.3	12.5416	0.6	2.2465	1.2	0.2044	1.1	0.89	1199.1	12.1	1195.7	8.7	1189.6	11.2	1189.6	11.2
-354-U1451A-66F-CCW Spot 35	1141	45665	6.1	12.5287	1.2	1.8636	1.8	0.1694	1.3	0.75	1008.9	12.5	1068.3	11.8	1191.6	23.2	1191.6	23.2
-354-U1451A-66F-CCW Spot 87	161	60657	2.6	12.5131	1.3	2.0811	1.6	0.1889	1.0	0.62	1115.7	10.3	1142.6	11.1	1194.1	25.1	1194.1	25.1
-354-U1451A-66F-CCW Spot 310	218	20430702	2.3	12.4942	0.6	2.2236	0.9	0.2016	0.7	0.80	1183.8	8.0	1188.5	6.5	1197.0	10.9	1197.0	10.9
-354-U1451A-66F-CCW Spot 57	1325	1165399	2.9	12.4036	0.6	2.1488	1.1	0.1934	0.9	0.83	1139.7	9.2	1164.7	7.3	1211.4	11.4	1211.4	11.4
-354-U1451A-66F-CCW Spot 58	280	197348	3.2	12.2749	0.7	2.3648	1.3	0.2106	1.1	0.83	1232.1	12.2	1232.0	9.3	1231.9	14.1	1231.9	14.1
-354-U1451A-66F-CCW Spot 18	240	154289	1.7	11.9365	0.6	2.4312	1.1	0.2106	0.9	0.82	1231.8	10.0	1251.9	7.8	1286.5	11.9	1286.5	11.9
-354-U1451A-66F-CCW Spot 252	195	427236	5.9	11.8266	0.7	2.7252	1.4	0.2339	1.2	0.87	1354.6	14.7	1335.3	10.3	1304.5	13.2	1304.5	13.2
-354-U1451A-66F-CCW Spot 267	720	8645224	1.4	11.8116	0.7	2.4911	1.1	0.2135	0.9	0.79	1247.4	10.2	1269.5	8.3	1307.0	13.5	1307.0	13.5
-354-U1451A-66F-CCW Spot 33	1492	146433	2.8	11.7763	0.5	2.3675	1.1	0.2023	1.0	0.88	1187.6	10.5	1232.8	7.8	1312.8	10.0	1312.8	10.0
-354-U1451A-66F-CCW Spot 113	427	494998	1.2	11.4164	0.4	2.9062	0.9	0.2407	0.8	0.90	1390.5	10.6	1383.5	7.1	1372.7	7.7	1372.7	7.7
-354-U1451A-66F-CCW Spot 98	227	237997	2.1	11.3638	0.5	2.8391	1.1	0.2341	0.9	0.89	1355.9	11.5	1365.9	8.0	1381.6	9.4	1381.6	9.4
-354-U1451A-66F-CCW Spot 94	259	440899	2.4	11.1275	0.7	2.9406	1.2	0.2374	1.0	0.82	1373.3	12.5	1392.4	9.3	1421.9	13.3	1421.9	13.3

-354-U1451A-66F-CCW Spot 46	352	118799	5.8	11.0860	0.5	3.1198	1.3	0.2510	1.2	0.92	1443.4	15.3	1437.6	9.9	1429.0	9.6	1429.0	9.6
-354-U1451A-66F-CCW Spot 101	927	4256365	4.0	11.0577	1.8	2.5133	2.0	0.2016	1.0	0.49	1184.2	10.8	1275.9	14.9	1433.9	34.1	1433.9	34.1
-354-U1451A-66F-CCW Spot 61	181	1942186	1.7	10.9870	0.5	3.0709	1.1	0.2448	0.9	0.87	1411.7	11.8	1425.5	8.2	1446.1	10.1	1446.1	10.1
-354-U1451A-66F-CCW Spot 91	401	97475	2.3	10.9653	0.6	3.1006	1.3	0.2467	1.2	0.90	1421.4	15.1	1432.8	10.1	1449.9	10.9	1449.9	10.9
-354-U1451A-66F-CCW Spot 41	486	305707	5.2	10.9462	0.6	3.1432	1.4	0.2496	1.3	0.90	1436.7	16.2	1443.3	10.8	1453.2	11.6	1453.2	11.6
-354-U1451A-66F-CCW Spot 62	915	2083612	1.4	10.6514	0.5	3.4005	1.3	0.2628	1.2	0.92	1504.2	16.0	1504.5	10.2	1505.0	9.8	1505.0	9.8
-354-U1451A-66F-CCW Spot 81	1173	504934	1.0	10.6188	0.6	3.2579	1.3	0.2510	1.1	0.87	1443.7	14.7	1471.1	10.2	1510.7	12.2	1510.7	12.2
-354-U1451A-66F-CCW Spot 104	219	113151	0.8	10.3500	0.6	3.5138	1.2	0.2639	1.0	0.84	1509.7	13.3	1530.3	9.3	1559.0	11.9	1559.0	11.9
-354-U1451A-66F-CCW Spot 133	297	502065	2.2	10.2357	0.6	3.5682	0.9	0.2650	0.7	0.78	1515.4	9.9	1542.5	7.5	1579.8	11.0	1579.8	11.0
-354-U1451A-66F-CCW Spot 190	727	583344	1.4	10.1464	0.6	3.7081	1.0	0.2730	0.8	0.82	1556.0	11.1	1573.1	7.8	1596.2	10.6	1596.2	10.6
-354-U1451A-66F-CCW Spot 107	381	161144	2.6	10.1122	0.5	3.9697	1.2	0.2913	1.1	0.90	1647.8	16.1	1628.0	10.0	1602.5	10.2	1602.5	10.2
-354-U1451A-66F-CCW Spot 97	439	155519	1.8	10.1106	0.5	3.8878	1.1	0.2852	0.9	0.88	1617.6	13.3	1611.1	8.6	1602.8	9.5	1602.8	9.5
-354-U1451A-66F-CCW Spot 269	229	184680	1.3	10.0759	0.7	3.6992	1.1	0.2704	0.9	0.82	1543.1	12.9	1571.2	9.2	1609.2	12.2	1609.2	12.2
-354-U1451A-66F-CCW Spot 155	137	322429	2.1	10.0755	0.7	3.6765	1.0	0.2688	0.8	0.77	1534.6	10.7	1566.3	8.1	1609.3	12.1	1609.3	12.1
-354-U1451A-66F-CCW Spot 296	165	180055	2.2	10.0266	0.6	3.9713	1.1	0.2889	0.9	0.83	1636.1	12.7	1628.4	8.6	1618.3	11.0	1618.3	11.0
-354-U1451A-66F-CCW Spot 52	159	153925	0.9	9.9816	0.6	4.0220	0.9	0.2913	0.7	0.79	1648.0	10.7	1638.6	7.6	1626.7	10.7	1626.7	10.7
-354-U1451A-66F-CCW Spot 291	604	511679	1.1	9.9376	0.6	4.0870	1.0	0.2947	0.8	0.81	1665.0	11.4	1651.7	7.8	1634.9	10.6	1634.9	10.6
-354-U1451A-66F-CCW Spot 149	112	439923	1.5	9.9043	0.6	4.0232	1.0	0.2891	0.8	0.79	1637.1	11.2	1638.9	8.0	1641.1	11.1	1641.1	11.1
-354-U1451A-66F-CCW Spot 140	563	1027009	1.5	9.8300	0.6	3.9503	1.0	0.2818	0.8	0.81	1600.2	11.8	1624.1	8.3	1655.1	11.0	1655.1	11.0
-354-U1451A-66F-CCW Spot 2	1413	1782988	1.2	9.7818	0.6	3.7675	1.0	0.2674	0.9	0.84	1527.6	11.9	1585.9	8.4	1664.2	10.6	1664.2	10.6
-354-U1451A-66F-CCW Spot 278	397	336970	1.9	9.5161	0.7	4.3574	1.1	0.3009	0.9	0.78	1695.6	13.0	1704.3	9.2	1715.0	12.7	1715.0	12.7
-354-U1451A-66F-CCW Spot 165	254	263459	1.5	9.4904	0.7	4.1646	1.3	0.2868	1.0	0.82	1625.4	14.9	1667.1	10.4	1719.9	13.5	1719.9	13.5
-354-U1451A-66F-CCW Spot 9	585	354491	2.2	9.4504	0.4	4.5227	0.8	0.3101	0.7	0.89	1741.3	11.4	1735.2	7.0	1727.7	6.9	1727.7	6.9
-354-U1451A-66F-CCW Spot 136	1956	755538	48.0	9.4124	0.7	4.0588	1.0	0.2772	0.8	0.76	1577.2	11.0	1646.1	8.4	1735.1	12.2	1735.1	12.2
-354-U1451A-66F-CCW Spot 13	644	825010	1.6	9.3906	0.6	4.4498	1.1	0.3032	1.0	0.85	1707.1	14.3	1721.7	9.2	1739.4	10.7	1739.4	10.7
-354-U1451A-66F-CCW Spot 146	210	104678	2.1	9.3149	0.6	4.6044	1.1	0.3112	0.9	0.82	1746.6	13.6	1750.1	9.1	1754.2	11.5	1754.2	11.5
-354-U1451A-66F-CCW Spot 69	553	594075	3.0	9.2652	0.5	4.6221	1.0	0.3107	0.9	0.86	1744.3	13.2	1753.3	8.4	1764.0	9.5	1764.0	9.5
-354-U1451A-66F-CCW Spot 45	765	1882353	3.2	9.2099	0.6	4.6343	0.9	0.3097	0.7	0.80	1739.2	11.3	1755.5	7.8	1774.9	10.2	1774.9	10.2
-354-U1451A-66F-CCW Spot 218	533	334174	5.7	9.1386	0.5	4.8837	1.0	0.3238	0.9	0.86	1808.4	13.6	1799.4	8.4	1789.1	9.4	1789.1	9.4
-354-U1451A-66F-CCW Spot 277	1741	521767	1.5	9.1237	0.6	4.4391	1.0	0.2939	0.9	0.82	1660.8	12.5	1719.7	8.7	1792.0	10.9	1792.0	10.9
-354-U1451A-66F-CCW Spot 282	380	15078	1.9	9.1139	2.9	4.3313	3.3	0.2864	1.5	0.47	1623.6	21.9	1699.3	26.9	1794.0	52.3	1794.0	52.3
-354-U1451A-66F-CCW Spot 311	299	104414	0.9	8.8235	0.6	5.1587	1.3	0.3303	1.2	0.90	1839.7	19.2	1845.8	11.4	1852.7	10.8	1852.7	10.8
-354-U1451A-66F-CCW Spot 263	305	112349	1.8	8.8064	0.6	5.0008	1.2	0.3195	1.0	0.84	1787.5	15.1	1819.5	9.8	1856.2	11.4	1856.2	11.4
-354-U1451A-66F-CCW Spot 215	838	1027708	1.6	8.7615	0.6	4.9770	1.1	0.3164	0.9	0.83	1772.1	14.1	1815.4	9.2	1865.5	10.9	1865.5	10.9
-354-U1451A-66F-CCW Spot 117	240	223599	2.8	8.7441	0.5	5.3636	0.8	0.3403	0.7	0.84	1888.1	11.4	1879.1	7.1	1869.1	8.2	1869.1	8.2
-354-U1451A-66F-CCW Spot 100	273	91120	4.3	8.6950	0.6	5.4355	1.2	0.3429	1.0	0.84	1900.7	16.0	1890.5	9.9	1879.2	11.3	1879.2	11.3
-354-U1451A-66F-CCW Spot 68	220	114183	2.8	8.6026	0.5	5.4262	0.9	0.3387	0.7	0.82	1880.4	11.4	1889.0	7.3	1898.4	8.7	1898.4	8.7
-354-U1451A-66F-CCW Spot 243	761	8753835	3.4	8.5814	0.6	5.3123	1.4	0.3308	1.2	0.89	1842.1	19.4	1870.8	11.6	1902.9	11.2	1902.9	11.2
-354-U1451A-66F-CCW Spot 261	672	434666	4.0	8.4459	0.5	5.4475	1.0	0.3338	0.9	0.86	1856.9	13.9	1892.4	8.6	1931.4	9.0	1931.4	9.0
-354-U1451A-66F-CCW Spot 203	877	646950	10.6	8.4169	0.6	5.4577	1.1	0.3333	0.9	0.85	1854.4	14.5	1894.0	9.1	1937.6	9.9	1937.6	9.9
-354-U1451A-66F-CCW Spot 86	566	333793	1.9	8.3188	0.6	5.7913	1.3	0.3496	1.2	0.89	1932.5	19.3	1945.1	11.2	1958.6	10.4	1958.6	10.4
-354-U1451A-66F-CCW Spot 235	930	898365	1.0	8.2013	0.7	5.8584	1.3	0.3486	1.1	0.82	1928.0	17.6	1955.1	11.2	1983.9	13.0	1983.9	13.0
-354-U1451A-66F-CCW Spot 298	572	5956982	6.4	8.1467	0.6	5.6059	1.0	0.3314	0.8	0.82	1845.0	12.8	1917.0	8.4	1995.8	9.8	1995.8	9.8
-354-U1451A-66F-CCW Spot 253	428	35708400	8.7	8.0604	0.6	6.1397	1.1	0.3591	0.9	0.86	1977.8	15.8	1995.9	9.5	2014.7	9.9	2014.7	9.9
-354-U1451A-66F-CCW Spot 5	1418	178699	21.5	7.7479	1.4	5.5473	1.7	0.3119	1.0	0.57	1749.8	14.7	1908.0	14.6	2084.5	24.5	2084.5	24.5
-354-U1451A-66F-CCW Spot 4	305	1734003	2.3	7.7304	0.5	6.2367	1.0	0.3498	0.9	0.87	1933.7	14.6	2009.6	8.8	2088.5	8.7	2088.5	8.7
-354-U1451A-66F-CCW Spot 234	171	114804	2.8	7.6982	0.5	6.8887	1.2	0.3848	1.0	0.89	2098.6	18.6	2097.2	10.3	2095.8	9.1	2095.8	9.1
-354-U1451A-66F-CCW Spot 23	764	212037	1.8	7.2002	0.6	7.5785	1.2	0.3959	1.0	0.86	2150.2	18.2	2182.3	10.4	2212.6	10.2	2212.6	10.2
-354-U1451A-66F-CCW Spot 42	236	118048	1.8	6.8063	0.6	8.1975	1.2	0.4048	1.0	0.85	2191.3	18.6	2253.1	10.6	2309.6	10.5	2309.6	10.5
-354-U1451A-66F-CCW Spot 240	561	8227671	1.3	6.7037	0.5	8.7833	0.9	0.4272	0.8	0.84	2293.2	14.9	2315.8	8.4	2335.7	8.5	2335.7	8.5
-354-U1451A-66F-CCW Spot 270	1450	1764337	13.5	6.5999	1.0	7.9522	1.3	0.3808	0.8	0.63	2080.1	14.6	2225.6	11.8	2362.4	17.4	2362.4	17.4
-354-U1451A-66F-CCW Spot 84	69	56265	2.0	6.5161	0.5	7.9440	1.1	0.3756	0.9	0.87	2055.6	16.3	2224.7	9.7	2384.2	9.2	2384.2	9.2
-354-U1451A-66F-CCW Spot 95	326	1397153	0.8	6.2841	0.6	8.1921	1.4	0.3735	1.3	0.90	2046.0	22.2	2252.5	12.7	2445.7	10.1	2445.7	10.1
-354-U1451A-66F-CCW Spot 279	455	335745	2.0	6.2507	0.4	9.4551	1.1	0.4288	1.0	0.92	2300.4	19.3	2383.2	9.9	2454.7	7.2	2454.7	7.2
-354-U1451A-66F-CCW Spot 14	357	984282	0.8	6.2456	0.6	9.4401	1.2	0.4278	1.0	0.87	2295.8	19.8	2381.7	10.8	2456.1	9.9	2456.1	9.9
-354-U1451A-66F-CCW Spot 200	351	215346	0.5	6.2087	0.5	8.9422	1.0	0.4028	0.8	0.85	2182.1	15.3	2332.1	8.9	2466.1	8.7	2466.1	8.7
-354-U1451A-66F-CCW Spot 124	269	2947991	2.1	6.1969	0.8	9.4540	1.4	0.4251	1.2	0.83	2283.5	23.0	2383.1	13.3	2469.3	13.6	2469.3	13.6
-354-U1451A-66F-CCW Spot 156	35	349389	1.5	6.1671	0.7	10.7149	1.1	0.4795	0.9	0.80	2524.9	18.6	2498.7	10.4	2477.5	11.4	2477.5	11.4
-354-U1451A-66F-CCW Spot 290	105	144143	1.1	6.1480	0.6	10.1559	1.0	0.4530	0.8	0.80	2408.8	15.9	2449.1	9.1	2482.7	10.0	2482.7	10.0
-354-U1451A-66F-CCW Spot 266	359	616533	0.7	6.1238	0.6	10.4499	1.1	0.4643	0.9	0.80	2458.6	17.9	2475.5	10.1	2489.4	10.9	2489.4	10.9
-354-U1451A-66F-CCW Spot 112	225	281097	1.7	6.0857	0.7	10.9017	1.7	0.4814	1.6	0.92	2533.3	33.3	2514.8	16.1	2499.9	11.6	2499.9	11.6
-354-U1451A-66F-CCW Spot 34	1353	1657896	8.2	6.0839	0.5	8.5259	0.9	0.3764	0.8	0.85	2059.3	13.3	2288.7	8.1	2500.4	7.9	2500.4	7.9
-354-U1451A-66F-CCW Spot 121	302	1226563	1.8	6.0734	0.6	10.5236	1.0	0.4638	0.8	0.80	2456.1	15.9	2482.0	9.0	2503.3	9.7	2503.3	9.7
-354-U1451A-66F-CCW Spot 1	854	342545	2.8	6.0676	0.8	9.0603	1.7	0.3989	1.5	0.88	2163.9	27.1	2344.1	15.3	2504.9	13.5	2504.9	13.5
-354-U1451A-66F-CCW Spot 185	679	770059	1.8	6.0242	0.6	10.4493	1.0	0.4567	0.8	0.81	2425.2	17.1	2475.4	9.6	2516.9	10.1	2516.9	10.1
-354-U1451A-66F-CCW Spot 43	346	410928	1.3	5.9732	0.7	10.6995	1.1	0.4637	0.8	0.75	2456.0	16.8	2497.4	10.1	2531.2	12.0	2531.2	12.0
-354-U1451A-66F-CCW Spot 183	728	280310	14.0	5.9638	1.1	9.8454	2.6	0.4260	2.3	0.90	2287.8	44.8	2420.4	23.9	2533.9	19.2	2533.9	19.2
-354-U1451A-66F-CCW Spot 198	191	547633	1.9	5.7001	0.5	11.8277	1.1	0.4892	0.9	0.86	2567.1	19.2	2590.9	9.8	2609.5	8.8	2609.5	8.8
-354-U1451A-66F-CCW Spot 145	108	93369	1.7	5.0519	0.5	13.2646	1.0	0.4862	0.9	0.86	2554.3	18.5	2698.7	9.6	2808.6	8.5	2808.6	8.5
-354-U1451A-66F-CCW Spot 257	336	262757	7.2	5.0239	0.9	14.7169	2.0	0.5365	1.8	0.90	2768.6	41.1	2797.1	19.3	2817.7	14.2	2817.7	14.2
-354-U1451A-66F-CCW Spot 54	98	139473	1.2	4.9707	0.5	15.3723	1.0	0.5544	0.9	0.89	2843.5	21.2	2838.6	9.9	2835.1	7.9	2835.1	7.9
-354-U1451A-66F-CCW Spot 128	163	143111	2.4	4.9362	0.5	15.0680	1.0	0.5397	0.8	0.83	2782.1	18.1	2819.5	9.2	2846.4	8.7	2846.4	8.7
-354-U1451A-66F-CCW Spot 122	660	533374	2.4	4.2963	0.5	16.2306	0.9	0.5060	0.7	0.80	2639.4	15.5	2890.5	8.5	3070.4	8.5	3070.4	8.5
-354-U1451A-66F-CCW Spot 31	460	1748690	2.5	4.1471	0.6	17.7623	1.4	0.5345	1.2	0.91	2760.3	27.5	2976.9	13.0	3126.8	9.0	3126.8	9.0
-354-U1451A-66F-CCW Spot 250	56	438102	1.3	4.0575	0.5	22.5395	0.9	0.6636	0.7	0.85	3281.0	18.9	3207.3	8.4	3161.4	7.1	3161.4	7.1
-354-U1451A-66F-CCW Spot 305	21	413430	9.2	3.7776	0.9	19.2762	2.9	0.5284	2.7	0.95	2734.5	60.6	3055.7	27.8	3274.3	14.8	3274.3	14.8
-354-U1451A-66F-CCW Spot 135	201	382079	0.8	3.6346	0.5	24.9462	0.9	0.6579	0.8	0.82	3258.9	19.3	3306.1	9.0	3334.9	8.4	3334.9	8.4
-354-U1451A-66F-CCW Spot 59	659	843910	1.4	3.5024	0.5	25.2667	0.9	0.6421	0.8	0.84	3197.2	19.5	3318.6	9.0	3392.7	7.8	3392.7	7.8
-354-U1451A-66F-CCW Spot 109	289	565707	1.5	3.2933	0.5	28.9234	0.9	0.6912	0.7	0.85	3387.0	19.6	3450.9	8.6	3488.2	7.2	3488.2	7.2
-354-U1451A-66F-CCW Spot 304	171	655483	3.5	3.2622	0.5	28.2620	1.0	0.6690	0.9	0.87	3301.9	22.2	3428.2	9.7	3502.9	7.5	3502.9	7.5

Table _____. U-Pb geochronologic analyses.																		
#20 U1451A_80F						Isotope ratios						Apparent ages (Ma)						
Late Miocene 8.7 Ma																		
Analysis	U	206Pb	U/Th	206Pb*	±	207Pb*	±	206Pb*	±	error	206Pb*	±	207Pb*	±	206Pb*	±	Best age	±
	(ppm)	204Pb		207Pb*	(%)	235U*	(%)	238U	(%)	corr.	238U*	(Ma)	235U	(Ma)	207Pb*	(Ma)	(Ma)	(Ma)
354U1451A-80F-4-W #35 -Spot 125	191	11006	344.9	19.5106	2.1	0.0364	2.5	0.0051	1.4	0.56	33.1	0.5	36.3	0.9	251.3	48.0	33.1	0.5
354U1451A-80F-4-W #35 -Spot 10	304	4310	1.2	21.2460	1.6	0.0427	2.1	0.0066	1.4	0.65	42.3	0.6	42.4	0.9	51.6	38.7	42.3	0.6
354U1451A-80F-4-W #35 -Spot 14	124	1833	0.9	24.6773	2.9	0.0414	3.1	0.0074	1.1	0.35	47.6	0.5	41.2	1.3	NA	NA	47.6	0.5
354U1451A-80F-4-W #35 -Spot 129	1128	16798	0.6	20.8156	1.8	0.0498	4.6	0.0075	4.3	0.92	48.3	2.1	49.3	2.2	100.2	41.9	48.3	2.1
354U1451A-80F-4-W #35 -Spot 33	234	60221	1.2	20.3373	1.8	0.0518	2.2	0.0076	1.4	0.62	49.1	0.7	51.3	1.1	155.0	41.0	49.1	0.7
354U1451A-80F-4-W #35 -Spot 230	184	1135	1.6	8.7155	6.5	0.1314	6.7	0.0083	1.5	0.22	53.4	0.8	125.4	7.9	1875.0	117.7	53.4	0.8
354U1451A-80F-4-W #35 -Spot 306	78	1410	1.1	23.3875	12.3	0.0493	12.4	0.0084	2.0	0.16	53.7	1.1	48.9	5.9	NA	NA	53.7	1.1
354U1451A-80F-4-W #35 -Spot 276	885	3089	0.9	9.2698	8.8	0.1312	9.0	0.0088	1.9	0.21	56.6	1.1	125.1	10.6	1763.1	160.9	56.6	1.1
354U1451A-80F-4-W #35 -Spot 208	3163	28758	10.2	19.6787	1.0	0.0621	1.6	0.0089	1.2	0.79	56.9	0.7	61.2	0.9	231.5	21.9	56.9	0.7
354U1451A-80F-4-W #35 -Spot 24	92	2992	1.4	21.8214	5.9	0.0567	6.2	0.0090	2.0	0.32	57.7	1.1	56.0	3.4	NA	NA	57.7	1.1
354U1451A-80F-4-W #35 -Spot 147	1199	28420	0.6	20.4228	1.0	0.0635	1.5	0.0094	1.1	0.72	60.4	0.7	62.6	0.9	145.1	24.6	60.4	0.7
354U1451A-80F-4-W #35 -Spot 99	230	13931	1.6	20.5606	1.8	0.0640	2.4	0.0095	1.6	0.65	61.2	1.0	63.0	1.5	129.3	43.3	61.2	1.0
354U1451A-80F-4-W #35 -Spot 80	2147	26949	1.2	20.9213	1.0	0.0651	1.5	0.0099	1.1	0.76	63.3	0.7	64.0	0.9	88.3	23.5	63.3	0.7
354U1451A-80F-4-W #35 -Spot 56	368	16847	0.7	20.8262	1.5	0.0657	2.0	0.0099	1.3	0.63	63.7	0.8	64.7	1.2	99.0	36.5	63.7	0.8
354U1451A-80F-4-W #35 -Spot 254	366	1002203	2.2	19.9245	1.6	0.0694	2.1	0.0100	1.4	0.67	64.4	0.9	68.1	1.4	202.7	36.3	64.4	0.9
354U1451A-80F-4-W #35 -Spot 31	152	30637	1.6	20.5188	2.2	0.0677	2.7	0.0101	1.5	0.57	64.7	1.0	66.6	1.7	134.1	51.5	64.7	1.0
354U1451A-80F-4-W #35 -Spot 185	215	3899	2.0	21.5078	1.6	0.0704	2.1	0.0110	1.4	0.67	70.4	1.0	69.1	1.4	22.3	37.8	70.4	1.0
354U1451A-80F-4-W #35 -Spot 78	718	64325	6.5	20.3155	1.6	0.0799	2.3	0.0118	1.7	0.74	75.5	1.3	78.0	1.8	157.5	36.8	75.5	1.3
354U1451A-80F-4-W #35 -Spot 275	149	68615	1.3	19.8683	1.6	0.0849	1.9	0.0122	1.0	0.54	78.4	0.8	82.7	1.5	209.3	36.2	78.4	0.8
354U1451A-80F-4-W #35 -Spot 209	822	13120	1.1	20.8942	1.1	0.0807	1.5	0.0122	1.0	0.68	78.4	0.8	78.8	1.2	91.3	26.3	78.4	0.8
354U1451A-80F-4-W #35 -Spot 200	604	23279	1.4	20.3100	0.9	0.0988	1.4	0.0146	1.1	0.78	93.2	1.0	95.7	1.3	158.1	20.9	93.2	1.0
354U1451A-80F-4-W #35 -Spot 233	61	4492	2.7	20.8438	4.5	0.0978	4.8	0.0148	1.4	0.30	94.6	1.3	94.7	4.3	97.0	107.6	94.6	1.3
354U1451A-80F-4-W #35 -Spot 152	1326	700565	2.5	20.0411	0.6	0.1117	1.2	0.0162	1.1	0.86	103.9	1.1	107.5	1.3	189.2	14.5	103.9	1.1
354U1451A-80F-4-W #35 -Spot 241	557	14493	3.3	20.1083	1.3	0.1154	1.6	0.0168	0.8	0.54	107.7	0.9	110.9	1.6	181.4	30.4	107.7	0.9
354U1451A-80F-4-W #35 -Spot 16	458	152138	1.6	19.5712	2.1	0.1195	2.5	0.0170	1.3	0.53	108.5	1.4	114.7	2.7	244.1	48.0	108.5	1.4
354U1451A-80F-4-W #35 -Spot 182	607	12803	1.6	15.7777	4.0	0.1534	4.2	0.0176	1.2	0.28	112.2	1.3	144.9	5.6	720.1	84.8	112.2	1.3
354U1451A-80F-4-W #35 -Spot 4	441	11438	0.9	20.6166	0.9	0.1177	1.6	0.0176	1.3	0.81	112.5	1.5	112.9	1.7	122.9	22.2	112.5	1.5
354U1451A-80F-4-W #35 -Spot 165	144	5014	1.6	20.9204	2.1	0.1166	2.7	0.0177	1.7	0.62	113.1	1.9	112.0	2.8	88.4	50.1	113.1	1.9
354U1451A-80F-4-W #35 -Spot 224	206	12901	1.2	20.1333	1.4	0.1300	2.1	0.0190	1.6	0.76	121.2	1.9	124.1	2.5	178.5	32.1	121.2	1.9
354U1451A-80F-4-W #35 -Spot 285	303	21941	1.5	19.8626	1.0	0.1409	1.6	0.0203	1.2	0.76	129.6	1.6	133.8	2.0	210.0	24.3	129.6	1.6
354U1451A-80F-4-W #35 -Spot 302	34	3394	1.7	20.6369	2.9	0.1386	3.2	0.0207	1.2	0.37	132.4	1.5	131.8	3.9	120.6	69.2	132.4	1.5
354U1451A-80F-4-W #35 -Spot 193	3442	26020	6.2	9.3728	0.6	0.3414	4.8	0.0232	4.8	0.99	148.0	7.0	298.2	12.4	1742.8	11.1	148.0	7.0
354U1451A-80F-4-W #35 -Spot 229	638	43549	3.3	19.7981	0.8	0.2170	1.5	0.0312	1.3	0.84	197.9	2.5	199.4	2.8	217.5	19.5	197.9	2.5
354U1451A-80F-4-W #35 -Spot 207	1810	41215	4.6	13.6405	0.7	0.4197	1.6	0.0415	1.4	0.89	262.4	3.6	355.8	4.7	1021.7	14.4	262.4	3.6
354U1451A-80F-4-W #35 -Spot 113	1907	82314	2.1	18.5224	1.0	0.3183	1.4	0.0428	1.0	0.69	270.1	2.6	280.6	3.5	369.6	23.4	270.1	2.6
354U1451A-80F-4-W #35 -Spot 103	3442	40698	1.0	17.3505	0.8	0.4085	4.2	0.0514	4.2	0.98	323.3	13.1	347.8	12.5	514.9	17.3	323.3	13.1
354U1451A-80F-4-W #35 -Spot 124	673	65972	1.9	17.5337	0.8	0.4170	1.9	0.0530	1.8	0.92	333.2	5.7	353.9	5.8	491.9	17.1	333.2	5.7
354U1451A-80F-4-W #35 -Spot 50	703	161210	6.1	10.8124	0.9	0.7342	3.4	0.0576	3.3	0.97	361.0	11.7	559.0	14.8	1476.6	16.3	361.0	11.7
354U1451A-80F-4-W #35 -Spot 166	897	429581	12.6	17.6518	0.8	0.5597	1.6	0.0717	1.4	0.86	446.3	6.0	451.3	5.9	477.0	18.0	446.3	6.0
354U1451A-80F-4-W #35 -Spot 75	1005	215085	5.3	17.7484	1.0	0.5609	2.0	0.0722	1.8	0.88	449.6	7.8	452.1	7.5	465.0	21.2	449.6	7.8
354U1451A-80F-4-W #35 -Spot 2	947	291314	1.4	17.5641	0.8	0.5676	1.3	0.0723	1.0	0.78	450.2	4.3	456.4	4.7	488.0	17.5	450.2	4.3
354U1451A-80F-4-W #35 -Spot 146	271	64357	1.1	17.3801	0.9	0.5757	1.9	0.0726	1.7	0.87	451.8	7.3	461.7	7.1	511.2	20.5	451.8	7.3
354U1451A-80F-4-W #35 -Spot 65	1394	334388	1.4	17.7231	0.6	0.5709	1.2	0.0734	1.1	0.89	456.7	4.9	458.6	4.6	468.1	12.8	456.7	4.9
354U1451A-80F-4-W #35 -Spot 204	938	89488	10.8	17.5060	0.9	0.5824	1.5	0.0740	1.2	0.82	460.1	5.4	466.0	5.6	495.3	19.0	460.1	5.4
354U1451A-80F-4-W #35 -Spot 105	931	100992	5.7	17.4999	0.6	0.5848	2.1	0.0743	2.0	0.96	461.8	8.9	467.6	7.8	496.1	13.3	461.8	8.9
354U1451A-80F-4-W #35 -Spot 268	536	76413	2.7	17.4002	0.6	0.5979	1.5	0.0755	1.3	0.90	469.1	5.9	475.9	5.6	508.7	14.2	469.1	5.9
354U1451A-80F-4-W #35 -Spot 162	269	43137	1.6	16.7541	1.5	0.6291	1.9	0.0765	1.1	0.59	475.1	5.1	495.5	7.4	591.3	33.0	475.1	5.1
354U1451A-80F-4-W #35 -Spot 54	597	142241	16.0	17.4314	0.8	0.6064	1.5	0.0767	1.3	0.86	476.4	6.1	481.3	5.9	504.7	17.0	476.4	6.1
354U1451A-80F-4-W #35 -Spot 212	1424	283381	6.2	17.2174	0.6	0.6143	1.4	0.0767	1.2	0.89	476.6	5.6	486.3	5.3	531.9	13.9	476.6	5.6
354U1451A-80F-4-W #35 -Spot 108	645	75919	10.8	17.5332	0.8	0.6041	1.4	0.0769	1.1	0.80	477.3	5.0	479.8	5.2	491.9	17.7	477.3	5.0
354U1451A-80F-4-W #35 -Spot 71	669	248428	2.7	17.7943	0.8	0.5996	1.4	0.0774	1.1	0.79	480.7	5.1	477.0	5.3	459.2	18.8	480.7	5.1
354U1451A-80F-4-W #35 -Spot 314	1097	231622	5.4	17.5199	0.6	0.6130	1.3	0.0779	1.1	0.88	483.7	5.3	485.4	5.0	493.6	13.9	483.7	5.3
354U1451A-80F-4-W #35 -Spot 128	1047	325086	1.4	17.0073	0.9	0.6357	1.4	0.0784	1.1	0.77	486.8	5.2	499.6	5.6	558.7	19.7	486.8	5.2
354U1451A-80F-4-W #35 -Spot 295	700	184039	7.2	16.6860	0.9	0.6542	1.5	0.0792	1.3	0.83	491.4	6.0	511.1	6.2	600.1	18.6	491.4	6.0
354U1451A-80F-4-W #35 -Spot 9	138	24841	1.0	17.1368	0.9	0.6420	1.8	0.0798	1.5	0.86	495.1	7.2	503.5	7.0	542.1	19.5	495.1	7.2
354U1451A-80F-4-W #35 -Spot 45	171	24778	2.9	16.9536	1.8	0.6548	2.4	0.0805	1.5	0.62	499.4	7.0	511.4	9.5	565.6	40.2	499.4	7.0
354U1451A-80F-4-W #35 -Spot 62	128	26719	0.4	17.2032	0.9	0.6486	1.4	0.0810	1.1	0.76	501.9	5.2	507.6	5.7	533.6	20.2	501.9	5.2
354U1451A-80F-4-W #35 -Spot 289	232	125749	1.1	17.0677	0.9	0.6591	1.7	0.0816	1.4	0.85	505.8	6.8	514.1	6.7	550.9	18.8	505.8	6.8
354U1451A-80F-4-W #35 -Spot 49	60	25020	0.7	16.9682	1.1	0.6698	1.8	0.0825	1.4	0.78	510.8	6.8	520.6	7.3	563.7	24.5	510.8	6.8
354U1451A-80F-4-W #35 -Spot 35	1264	1301805	1.8	17.0934	0.6	0.6706	1.1	0.0832	0.9	0.85	515.0	4.6	521.1	4.4	547.7	12.5	515.0	4.6
354U1451A-80F-4-W #35 -Spot 239	951	303497	0.7	16.8326	0.7	0.6859	1.3	0.0838	1.1	0.85	518.6	5.5	530.3	5.4	581.2	15.1	518.6	5.5
354U1451A-80F-4-W #35 -Spot 37	859	2263265	2.7	17.3394	0.7	0.6800	1.3	0.0856	1.1	0.84	529.2	5.7	526.8	5.5	516.3	16.0	529.2	5.7
354U1451A-80F-4-W #35 -Spot 249	44	10437	0.6	16.7173	1.6	0.7279	2.0	0.0883	1.2	0.59	545.4	6.0	555.3	8.4	596.1	34.6	545.4	6.0
354U1451A-80F-4-W #35 -Spot 74	230	35621	1.0	16.8379	1.4	0.7309	2.5	0.0893	2.0	0.83	551.4	10.8	557.1	10.6	580.5	29.7	551.4	10.8
354U1451A-80F-4-W #35 -Spot 174	512	75333	7.7	17.0584	0.6	0.7292	1.2	0.0903	1.0	0.85	557.1	5.5	556.1	5.2	552.2	13.9	557.1	5.5
354U1451A-80F-4-W #35 -Spot 199	1755	360720	13.7	16.2339	0.7	0.7975	1.6	0.0939	1.4	0.89	578.8	7.8	595.4	7.1	659.3	15.8	578.8	7.8
354U1451A-80F-4-W #35 -Spot 269	338	384229	3.0	16.5150	0.7	0.8393	1.4	0.1006	1.2	0.85	617.8	7.0	618.8	6.5	622.4	15.8	617.8	7.0
354U1451A-80F-4-W #35 -Spot 280	271	62137	2.2	16.2978	0.7	0.8531	1.4	0.1009	1.2	0.86	619.5	6.8	626.3	6.3	650.9	14.9	619.5	6.8
354U1451A-80F-4-W #35 -Spot 215	61	86812	367.8	16.6501	1.1	0.8439	1.9	0.1020	1.6	0.83	625.8	9.6	621.3	8.9	604.8	22.9	625.8	9.6
354U1451A-80F-4-W #35 -Spot 58	144	50440	4.8	16.0529	1.1	0.9105	1.6	0.1061	1.2	0.72	649.8	7.2	657.3	7.8	683.3	24.1	649.8	7.2
354U1451A-80F-4-W #35 -Spot 180	364	68589	1.9	15.6943	0.7	1.0229	1.3	0.1165	1.1	0.85	710.3	7.4	715.4	6.7	731.4	14.5	710.3	7.4
354U1451A-80F-4-W #35 -Spot 245	148	25505	2.2	15.5468	0.8	1.0573	1.5	0.1193	1.3	0.86	726.4	8.7	732.5	7.7	751.3	16.1	726.4	8.7
354U1451A-80F-4-W #35 -Spot 186	185	34040	3.5	15.5541	0.7	1.0577	1.4	0.1194	1.2	0.85	727.0	8.2	732.7	7.3	750.3	15.4	727.0	8.2
354U1451A-80F-4-W #35 -Spot 93	535	135206	6.1	15.6039	0.6	1.0770	1.4	0.1219	1.2	0.89	741.7	8.6	742.2	7.3	743.6	13.5	741.7	8.6
354U1451A-80F-4-W #35 -Spot 157	111	55833	1.1	15.4262	0.8	1.1020	1.3	0.1233	1.0	0.77	749.8	7.0	754.3	6.8	767.7	17.1	749.8	7.0
354U1451A-80F-4-W #35 -Spot 120	856	33306	4.4	14.6106	0.8	1.1704	1.4	0.1241	1.1	0.81	754.0	8.1	786.8	7.7	881.1	17.0	754.0	8.1
354U1451A-80F-4-W #35 -Spot 117	478	366951	1.8	15.3003	0.7	1.1273	1.3	0.1251	1.1	0.84	760.1	7.6	766.5	6.8	784.9	14.6	760.1	7.6
354U1451A-80F-4-W #35 -Spot 123	232	61270	1.4	15.0897	0.9	1.1802	1.5	0.1292	1.3	0.82	783.4	9.3	791.4	8.5	814.0	18.7	783.4	9.3
354U1451A-80F-4-W #35 -Spot 12	410	3389256	0.9	14.9704	1.0	1.1980	1.8	0.1301	1.5	0.82	788.6	10.8	799.7	9.8	830.6	21.0	788.6	10.8
354U1451A-80F-4-W #35 -Spot 278	97	15966	1.1	15.1082	0.8	1.1911	1.4	0.1306	1.1	0.81	791.1	8.2	796.5	7.6	811.5	16.9	791.1	8.2
354U1451A-80F-4-W #35 -Spot 15	161	221211	1.3	14.9975	0.8	1.2030	1.3	0.1309	1.1	0.80	793.0	7.9	802.0	7.4	826.8	16.8	793.0	7.9
354U1451A-80F-4-W #35 -Spot 27	3569	292102	0.8	14.9942	0.9	1.2169	1.6	0.1324	1.3	0.83	801.5	10.1	808.4	9.0	827.3	18.6	801.5	10.1
354U1451A-80F-4-W #35 -Spot 206	615	824106	2.7	14.0142	0.7	1.3097	2.1	0.1332	2.0	0.95	806.0	15.3	850.0	12.3	966.7	14.2	806.0	15.3
354U1451A-80F-4-W #35 -Spot 203	258	38808	2.3	14.6706	0.7	1.2539	1.5	0.1335	1.3	0.88	807.6	10.0	825.1	8.4	872.6	14.7	807.6	10.0
354U1451A-80F-4-W #35 -Spot 240	166	44945	1.7	14.6284	1.1	1.2654	1.8	0.1343	1.5	0.80	812.4	11.2	830.3	10.4	878.6	22.9	812.4	11.2
354U1451A-80F-4-W #35 -Spot 135	204	78168	1.6	15.0104	0.8	1.2386	1.3	0.1349	1.1	0.79	815.7	8.2	818.2	7.6	825.0	17.1	815.7	8.2
354U1451A-80F-4-W #35 -Spot 244	256	66933	2.2	14.3222	0.7	1.3014	1.4	0.1352	1.2	0.87	817.7	9.1	846.3	7.8	922.2	13.7	817.7	9.1
354U1451A-80F-4-W #35 -Spot 256	99	142143	2.0	14.7272	0.9	1.2822	1.5	0.1370	1.2	0.78	827.8	9.1	837.8	8.6	864.7	19.6	827.8	9.1
354U1451A-80F-4-W #35 -Spot 154	351	89269	1.2	14.8937	0.8	1.2696	1.4	0.1372	1.2	0.82	828.8	9.1	832.2	8.1	841.3	17.1	828.8	9.1
354U1451A-80F-4-W #35 -Spot 242	260	50084	0.7	14.7509	0.6	1.2872	1.3	0.1378	1.1	0.87	832.0	8.8	840.0	7.4	861.3	13.2	832.0	8.8
354U1451A-80F-4-W #35 -Spot 112	1268	240687	0.9	14.0644	0.7	1.3623	2.2	0.1390	2.0	0.95	839.1	16.1	872.9	12.6	959.5	14.1	839.1	16.1
354U1451A-80F-4-W #35 -Spot 85	543	111942	6.1	14.7585	0.9	1.3160	1.6	0.1409	1.3	0.82	849.9	10.2	852.7	9.1	860.2	18.8	849.9	10.2
354U1451A-80F-4-W #35 -Spot 247	125	95694	2.5	14.6221	0.7	1.3308	1.3	0.1412	1.0	0.82	851.4	8.2	859.2	7.3	879.5	15.1	851.4	8.2
354U1451A-80F-4-W #35 -Spot 238	197	187137	1.5	14.3866	0.7	1.3552	1.5	0.1415	1.4	0.90	853.0	10.8	869.8	8.8	913.0	13.5	853.0	10.8

354U1451A-80F-4-W #35 -Spot 210	112	253701	1.5	14.6825	0.7	1.3329	1.3	0.1420	1.1	0.83	855.9	8.8	860.1	7.7	871.0	15.5	855.9	8.8
354U1451A-80F-4-W #35 -Spot 164	581	147351	1.0	14.5973	0.8	1.3434	1.6	0.1423	1.4	0.86	857.6	10.9	864.7	9.1	883.0	16.4	857.6	10.9
354U1451A-80F-4-W #35 -Spot 132	140	122228	1.5	14.6578	0.9	1.3415	1.4	0.1427	1.1	0.77	859.8	8.6	863.9	8.1	874.4	18.2	859.8	8.6
354U1451A-80F-4-W #35 -Spot 72	314	130111	4.6	14.8234	0.7	1.3298	1.4	0.1430	1.2	0.88	861.8	10.0	858.8	8.2	851.1	14.2	861.8	10.0
354U1451A-80F-4-W #35 -Spot 151	91	32785	1.5	14.6370	0.9	1.3789	1.7	0.1464	1.4	0.84	881.0	11.5	880.0	9.9	877.4	19.1	881.0	11.5
354U1451A-80F-4-W #35 -Spot 121	371	51683	7.0	14.7328	0.8	1.3776	1.8	0.1473	1.6	0.90	885.6	13.5	879.4	10.7	863.9	16.8	885.6	13.5
354U1451A-80F-4-W #35 -Spot 284	57	85414	1.1	14.4406	1.0	1.4069	1.6	0.1474	1.2	0.78	886.5	10.2	891.9	9.4	905.2	20.5	886.5	10.2
354U1451A-80F-4-W #35 -Spot 8	309	65683	5.4	14.4034	0.7	1.4152	1.5	0.1479	1.3	0.86	889.2	10.4	895.3	8.7	910.6	15.1	889.2	10.4
354U1451A-80F-4-W #35 -Spot 133	472	215523	7.9	14.7873	0.6	1.3911	1.2	0.1493	1.1	0.89	896.8	9.2	885.2	7.3	856.2	12.0	896.8	9.2
354U1451A-80F-4-W #35 -Spot 262	1258	359168	12.7	14.2910	0.7	1.4012	1.3	0.1453	1.1	0.85	874.5	8.9	889.4	7.6	926.7	13.6	926.7	13.6
354U1451A-80F-4-W #35 -Spot 17	141	57668	3.7	14.2698	0.8	1.5596	1.3	0.1615	1.0	0.79	965.0	9.3	954.3	8.2	929.8	16.7	929.8	16.7
354U1451A-80F-4-W #35 -Spot 310	2040	65330	2.1	14.2694	0.8	1.4138	1.7	0.1464	1.4	0.86	880.6	11.7	894.7	9.8	929.8	17.2	929.8	17.2
354U1451A-80F-4-W #35 -Spot 100	546	82009	20.6	14.2635	0.7	1.4876	1.3	0.1540	1.1	0.83	923.1	9.3	925.3	8.0	930.6	15.2	930.6	15.2
354U1451A-80F-4-W #35 -Spot 311	2863	403504	49.6	14.2519	0.8	1.5506	1.3	0.1603	1.0	0.80	958.7	9.1	950.7	7.9	932.3	15.5	932.3	15.5
354U1451A-80F-4-W #35 -Spot 301	235	1070501	1.1	14.2480	0.9	1.4592	1.6	0.1509	1.4	0.85	905.7	11.5	913.7	9.7	932.9	17.5	932.9	17.5
354U1451A-80F-4-W #35 -Spot 227	261	183544	4.3	14.2397	1.0	1.3976	1.8	0.1444	1.5	0.83	869.5	12.3	887.9	10.9	934.1	21.2	934.1	21.2
354U1451A-80F-4-W #35 -Spot 309	178	74535	1.3	14.1799	0.7	1.4637	1.1	0.1506	0.9	0.81	904.3	7.9	915.6	6.9	942.7	13.7	942.7	13.7
354U1451A-80F-4-W #35 -Spot 18	868	510041	3.1	14.1519	0.7	1.5353	1.4	0.1576	1.2	0.88	943.7	10.7	944.6	8.6	946.8	13.8	946.8	13.8
354U1451A-80F-4-W #35 -Spot 5	360	711724	0.5	14.1475	0.9	1.5832	1.5	0.1625	1.2	0.79	970.7	10.7	963.6	9.3	947.4	18.8	947.4	18.8
354U1451A-80F-4-W #35 -Spot 76	346	129714	2.6	14.0875	0.6	1.6378	1.4	0.1674	1.2	0.89	997.8	11.2	984.9	8.6	956.1	12.9	956.1	12.9
354U1451A-80F-4-W #35 -Spot 48	384	71944	1.3	14.0750	0.9	1.5628	1.5	0.1596	1.2	0.79	954.6	10.7	955.6	9.4	957.9	19.0	957.9	19.0
354U1451A-80F-4-W #35 -Spot 142	200	100794	1.3	14.0639	1.1	1.5586	1.9	0.1591	1.6	0.83	951.5	14.3	953.9	12.1	959.5	22.2	959.5	22.2
354U1451A-80F-4-W #35 -Spot 218	412	586149	7.3	14.0519	0.8	1.5601	1.5	0.1591	1.2	0.85	951.6	10.9	954.5	9.0	961.3	15.7	961.3	15.7
354U1451A-80F-4-W #35 -Spot 217	263	391807	2.5	14.0412	0.8	1.5832	1.6	0.1613	1.4	0.87	964.0	12.5	963.6	10.0	962.8	16.2	962.8	16.2
354U1451A-80F-4-W #35 -Spot 271	128	103930	1.1	14.0318	0.8	1.5519	1.5	0.1580	1.2	0.83	945.6	10.7	951.2	9.0	964.2	16.7	964.2	16.7
354U1451A-80F-4-W #35 -Spot 13	762	215472	6.3	14.0317	0.8	1.5997	1.6	0.1629	1.4	0.86	972.7	12.3	970.1	9.9	964.2	16.4	964.2	16.4
354U1451A-80F-4-W #35 -Spot 145	277	1741062	0.8	14.0246	0.8	1.6073	1.5	0.1636	1.3	0.84	976.5	11.4	973.1	9.3	965.2	16.3	965.2	16.3
354U1451A-80F-4-W #35 -Spot 292	809	4786411	6.6	14.0055	0.7	1.5736	1.4	0.1599	1.2	0.86	956.3	10.4	959.9	8.4	968.0	14.0	968.0	14.0
354U1451A-80F-4-W #35 -Spot 28	225	331724	3.0	13.9943	0.9	1.5931	1.8	0.1618	1.6	0.88	966.6	14.5	967.5	11.4	969.6	17.3	969.6	17.3
354U1451A-80F-4-W #35 -Spot 29	941	544395	49.4	13.9900	0.7	1.5719	1.3	0.1596	1.1	0.83	954.3	9.4	959.2	7.9	970.3	14.7	970.3	14.7
354U1451A-80F-4-W #35 -Spot 300	464	77986	18.8	13.9820	0.5	1.6254	1.1	0.1649	1.0	0.88	983.9	8.7	980.1	6.8	971.5	10.3	971.5	10.3
354U1451A-80F-4-W #35 -Spot 26	479	46614	0.9	13.9789	1.2	1.4759	1.9	0.1497	1.4	0.77	899.3	12.1	920.6	11.4	971.9	24.7	971.9	24.7
354U1451A-80F-4-W #35 -Spot 223	903	510364	3.7	13.9479	0.6	1.5330	1.3	0.1551	1.1	0.86	929.8	9.4	943.7	7.8	976.4	13.2	976.4	13.2
354U1451A-80F-4-W #35 -Spot 315	232	885402	1.7	13.9402	0.7	1.5833	1.3	0.1601	1.1	0.82	957.6	9.4	963.7	8.1	977.5	15.2	977.5	15.2
354U1451A-80F-4-W #35 -Spot 288	125	70207	1.6	13.9221	0.7	1.7091	1.3	0.1726	1.1	0.85	1026.7	10.8	1011.9	8.6	980.2	14.5	980.2	14.5
354U1451A-80F-4-W #35 -Spot 25	1713	681959	1.1	13.9013	0.7	1.6825	1.2	0.1697	1.0	0.81	1010.5	9.4	1001.9	7.9	983.2	14.8	983.2	14.8
354U1451A-80F-4-W #35 -Spot 46	571	121045	3.3	13.8943	0.8	1.6181	1.8	0.1631	1.6	0.88	974.2	14.0	977.3	11.1	984.2	17.0	984.2	17.0
354U1451A-80F-4-W #35 -Spot 88	16	15502	3.9	13.8903	1.4	1.6092	2.0	0.1622	1.4	0.72	968.9	12.8	973.8	12.3	984.9	27.7	984.9	27.7
354U1451A-80F-4-W #35 -Spot 171	115	62731	3.7	13.8786	0.8	1.5767	1.4	0.1588	1.2	0.82	949.9	10.3	961.1	8.8	986.5	16.4	986.5	16.4
354U1451A-80F-4-W #35 -Spot 222	181	143050	2.0	13.8696	0.6	1.6726	1.1	0.1683	1.0	0.83	1002.9	8.8	998.2	7.3	987.9	13.0	987.9	13.0
354U1451A-80F-4-W #35 -Spot 294	372	76446	1.4	13.8674	0.7	1.6355	1.1	0.1646	0.9	0.78	982.1	7.8	984.0	7.0	988.2	14.1	988.2	14.1
354U1451A-80F-4-W #35 -Spot 198	535	139875	25.6	13.8641	0.5	1.5505	1.0	0.1560	0.8	0.86	934.4	7.4	950.7	6.1	988.7	10.2	988.7	10.2
354U1451A-80F-4-W #35 -Spot 109	625	389586	6.5	13.8268	0.7	1.6583	1.5	0.1664	1.3	0.89	992.1	11.9	992.7	9.2	994.2	13.6	994.2	13.6
354U1451A-80F-4-W #35 -Spot 122	127	227979	0.6	13.8169	0.9	1.5159	1.3	0.1520	1.0	0.73	912.0	8.2	936.8	8.0	995.6	18.2	995.6	18.2
354U1451A-80F-4-W #35 -Spot 232	354	60537	4.5	13.7768	0.8	1.5468	1.4	0.1546	1.2	0.83	926.8	10.2	949.2	8.8	1001.5	16.1	1001.5	16.1
354U1451A-80F-4-W #35 -Spot 170	243	194143	1.9	13.7697	0.8	1.7184	1.6	0.1717	1.4	0.87	1021.4	13.1	1015.4	10.2	1002.6	15.8	1002.6	15.8
354U1451A-80F-4-W #35 -Spot 266	538	247407	2.2	13.7303	0.9	1.6935	1.5	0.1687	1.2	0.81	1005.0	11.4	1006.1	9.7	1008.4	18.1	1008.4	18.1
354U1451A-80F-4-W #35 -Spot 44	402	77237	1.4	13.7293	0.8	1.5160	1.7	0.1510	1.5	0.89	906.7	12.9	936.9	10.5	1008.5	15.9	1008.5	15.9
354U1451A-80F-4-W #35 -Spot 188	158	64498	1.5	13.6949	0.7	1.6935	1.3	0.1683	1.1	0.84	1002.6	9.9	1006.1	8.1	1013.6	14.1	1013.6	14.1
354U1451A-80F-4-W #35 -Spot 70	1164	74105	11.1	13.6767	1.2	1.5308	2.0	0.1519	1.6	0.80	911.6	13.7	942.8	12.5	1016.3	24.9	1016.3	24.9
354U1451A-80F-4-W #35 -Spot 116	486	455827	2.4	13.6500	0.7	1.7483	1.3	0.1732	1.1	0.85	1029.5	10.5	1026.5	8.4	1020.3	14.1	1020.3	14.1
354U1451A-80F-4-W #35 -Spot 79	248	180698	1.6	13.6390	0.6	1.7520	1.2	0.1734	1.1	0.86	1030.7	10.2	1027.9	8.0	1021.9	12.6	1021.9	12.6
354U1451A-80F-4-W #35 -Spot 160	557	492301	4.2	13.6055	0.8	1.6413	1.7	0.1620	1.5	0.90	968.0	13.9	986.2	10.9	1026.9	15.6	1026.9	15.6
354U1451A-80F-4-W #35 -Spot 23	656	1104738	29.8	13.5937	1.0	1.5405	1.8	0.1519	1.5	0.84	911.8	12.6	946.7	10.9	1028.6	19.5	1028.6	19.5
354U1451A-80F-4-W #35 -Spot 19	46	13363	1.7	13.5861	2.2	1.3976	2.8	0.1378	1.8	0.63	832.1	13.8	887.9	16.6	1029.8	43.9	1029.8	43.9
354U1451A-80F-4-W #35 -Spot 287	67	23950	2.0	13.5844	1.0	1.6963	1.5	0.1672	1.2	0.77	996.6	10.7	1007.1	9.6	1030.0	19.5	1030.0	19.5
354U1451A-80F-4-W #35 -Spot 234	32	136871	1.3	13.5786	1.4	1.6618	1.9	0.1637	1.3	0.66	977.5	11.6	994.1	12.2	1030.9	29.3	1030.9	29.3
354U1451A-80F-4-W #35 -Spot 126	448	304358	3.2	13.5704	0.9	1.6675	1.5	0.1642	1.1	0.77	980.0	10.4	996.2	9.4	1032.1	19.0	1032.1	19.0
354U1451A-80F-4-W #35 -Spot 57	551	253713	2.8	13.5267	0.8	1.4642	1.6	0.1437	1.4	0.86	865.6	11.0	915.8	9.5	1038.6	16.1	1038.6	16.1
354U1451A-80F-4-W #35 -Spot 194	330	252423	5.8	13.5235	0.8	1.7298	1.1	0.1697	0.7	0.67	1010.7	6.8	1019.7	7.0	1039.1	16.2	1039.1	16.2
354U1451A-80F-4-W #35 -Spot 1	688	310526	7.9	13.4463	0.8	1.6613	1.3	0.1621	1.1	0.81	968.3	9.6	993.9	8.4	1050.7	15.8	1050.7	15.8
354U1451A-80F-4-W #35 -Spot 87	1222	1578943	0.6	13.3815	0.7	1.8042	1.5	0.1752	1.4	0.89	1040.6	13.1	1047.0	10.1	1060.4	14.3	1060.4	14.3
354U1451A-80F-4-W #35 -Spot 298	810	198979	2.7	13.3696	0.7	1.5772	1.4	0.1530	1.3	0.87	917.8	10.7	961.3	9.0	1062.2	14.4	1062.2	14.4
354U1451A-80F-4-W #35 -Spot 312	489	203374	2.2	13.2826	0.6	1.8380	1.3	0.1771	1.2	0.90	1051.3	11.7	1059.1	8.8	1075.3	11.6	1075.3	11.6
354U1451A-80F-4-W #35 -Spot 98	228	87741	1.9	13.2817	0.6	1.9342	1.3	0.1864	1.1	0.88	1101.9	11.5	1093.0	8.6	1075.4	12.3	1075.4	12.3
354U1451A-80F-4-W #35 -Spot 111	178	153874	1.4	13.2205	0.6	1.9166	1.3	0.1838	1.1	0.87	1088.0	11.3	1086.9	8.7	1084.7	13.0	1084.7	13.0
354U1451A-80F-4-W #35 -Spot 156	105	114793	2.1	13.2159	0.6	1.8898	1.2	0.1812	1.1	0.86	1073.6	10.5	1077.5	8.3	1085.4	12.9	1085.4	12.9
354U1451A-80F-4-W #35 -Spot 248	282	82636	3.2	13.1973	0.5	1.8722	1.1	0.1793	0.9	0.87	1063.0	8.9	1071.3	7.0	1088.2	10.5	1088.2	10.5
354U1451A-80F-4-W #35 -Spot 21	730	269955	2.1	13.1780	0.8	1.8125	1.5	0.1733	1.3	0.86	1030.3	12.4	1050.0	9.9	1091.2	15.7	1091.2	15.7
354U1451A-80F-4-W #35 -Spot 81	342	414896	1.6	13.1616	0.7	1.9652	1.5	0.1877	1.3	0.88	1108.7	13.1	1103.7	9.8	1093.6	13.7	1093.6	13.7
354U1451A-80F-4-W #35 -Spot 281	404	504214	2.5	13.1398	0.8	1.8472	1.3	0.1761	1.1	0.82	1045.7	10.2	1062.4	8.6	1096.9	15.0	1096.9	15.0
354U1451A-80F-4-W #35 -Spot 55	100	104345	1.9	13.1344	1.0	1.8944	1.8	0.1805	1.5	0.84	1069.9	14.7	1079.1	11.8	1097.8	19.1	1097.8	19.1
354U1451A-80F-4-W #35 -Spot 187	390	55241	2.5	13.1171	0.7	1.6613	1.4	0.1581	1.1	0.84	946.3	10.0	993.9	8.6	1100.4	14.8	1100.4	14.8
354U1451A-80F-4-W #35 -Spot 183	394	69530	7.0	13.0970	0.7	1.9298	1.2	0.1834	1.0	0.83	1085.5	9.7	1091.5	7.8	1103.5	13.3	1103.5	13.3
354U1451A-80F-4-W #35 -Spot 40	53	13138	1.5	13.0601	1.5	1.8100	1.9	0.1715	1.1	0.60	1020.5	10.8	1049.1	12.6	1109.1	30.9	1109.1	30.9
354U1451A-80F-4-W #35 -Spot 237	296	103859	3.5	12.9924	0.8	1.9198	1.6	0.1810	1.4	0.87	1072.3	13.5	1088.0	10.5	1119.5	15.3	1119.5	15.3
354U1451A-80F-4-W #35 -Spot 282	53	41804	0.5	12.9161	0.8	2.0061	1.3	0.1880	1.0	0.80	1110.6	10.7	1117.6	8.9	1131.2	15.8	1131.2	15.8
354U1451A-80F-4-W #35 -Spot 283	468	1180183	5.9	12.7919	1.0	2.0267	1.6	0.1881	1.3	0.81	1111.1	13.6	1124.5	11.2	1150.4	19.0	1150.4	19.0
354U1451A-80F-4-W #35 -Spot 86	24	55990	2.3	12.7554	1.5	1.7141	1.9	0.1586	1.2	0.62	949.2	10.5	1013.8	12.3	1156.1	29.9	1156.1	29.9
354U1451A-80F-4-W #35 -Spot 114	171	588344	2.2	12.7521	0.6	2.1403	1.2	0.1980	1.0	0.85	1164.8	11.1	1161.9	8.5	1156.6	12.6	1156.6	12.6
354U1451A-80F-4-W #35 -Spot 38	96	810553	1.1	12.7499	0.9	1.7226	1.8	0.1594	1.5	0.86	953.2	13.4	1017.0	11.3	1157.0	17.7	1157.0	17.7
354U1451A-80F-4-W #35 -Spot 36	194	101712	3.7	12.7264	0.8	2.1744	1.4	0.2008	1.1	0.81	1179.6	11.9	1172.9	9.6	1160.6	16.2	1160.6	16.2
354U1451A-80F-4-W #35 -Spot 214	195	77479	1.1	12.7258	0.7	2.1471	1.6	0.1983	1.5	0.89	1166.0	15.5	1164.1	11.3	1160.7	14.8	1160.7	14.8
354U1451A-80F-4-W #35 -Spot 305	268	251381	2.0	12.6346	0.6	2.2039	1.4	0.2020	1.2	0.89	1186.3	13.3	1182.3	9.7	1175.0	12.6	1175.0	12.6
354U1451A-80F-4-W #35 -Spot 43	316	79399	1.5	12.6111	0.7	2.0556	1.7	0.1881	1.5	0.91	1111.1	15.7	1134.2	11.6	1178.7	14.2	1178.7	14.2
354U1451A-80F-4-W #35 -Spot 63	298	397246	0.8	12.5528	0.8	2.0638	1.8	0.1880	1.6	0.89	1110.4	16.3	1136.9	12.2	1187.8	15.9	1187.8	15.9
354U1451A-80F-4-W #35 -Spot 263	1051	235487	5.8	12.5022	1.0	1.9871	1.6	0.1803	1.3	0.79	1068.4	12.8	1111.1	11.1	1195.8	19.7	1195.8	19.7
354U1451A-80F-4-W #35 -Spot 92	709	46107	16.7	12.3459	0.8	2.2418	1.3	0.2008	1.0	0.78	1179.7	10.6	1194.2	8.8	1220.5	15.4	1220.5	15.4
354U1451A-80F-4-W #35 -Spot 138	708	235163	19.7	12.0668	1.6	2.0988	2.0	0.1838	1.3	0.64	1087.5	13.2	1148.4	14.1	1265.4	30.5	1265.4	30.5
354U1451A-80F-4-W #35 -Spot 107	647	290202	3.5	11.9078	0.8	2.4733	1.5	0.2137	1.3	0.86	1248.5	14.5	1264.2	10.7	1291.2	14.8	1291.2	14.8
354U1451A-80F-4-W #35 -Spot 219	494	360803	3.7	11.7056	0.5	2.6561	1.1	0.2256	1.0	0.88	1311.4	11.5	1316.3	8.1	1324.4	10.3	1324.4	10.3
354U1451A-80F-4-W #35 -Spot 213	170	138537	1.2	11.6576	0.6	2.6512	1.3	0.2243	1.1	0.87	1304.3	13.4	1315.0	9.6	1332.4	12.4	1332.4	12.4
354U1451A-80F-4-W #35 -Spot 261	1075	2123343	10.2	11.6253	0.7	2.6150	1.4	0.2206	1.2	0.84	1284.9	13.4	1304.9	10.0	1337.8	14.1	1337.8	14.1
354U1451A-80F-4-W #35 -Spot 131	877	2833954	11.5	11.5525	1.0	2.1761	1.4	0.1824	1.0	0.73	1080.1	10.2	1173.4	9.8	1349.9	18.5	1349.9	18.5
354U1451A-80F-4-W #35 -Spot 176	516	546119	2.5	11.5366	0.5	2.8246	1.1	0.2364	1.0	0.88	1368.2	11.7	1362.1	8.1	1352.5	9.8	1352.5	9.8
354U1451A-80F-4-W #35 -Spot 251	1026	210129	14.8	11.5154	1.5	2.3748	1.9	0.1984	1.2	0.61	1166.8	12.6	1235.0	13.8	1356.1	29.4	1356.1	29.4
354U1451A-80F-4-W #35 -Spot 82	384	143662	1.0	11.3967	0.8	2.7809	1.4	0.2300	1.1	0.83	1334.3	13.6	1350.4	10.2	1376.0	14.7	1376.0	14.7
354U1451A-80F-4-W #35 -Spot 246	302	257913	1.5	11.3365	0.8	2.9249	1.2	0.2406	1.0	0.78	1389.8	12.0	1388.4	9.4	1386.2	15.0	1386.2	15.0
354U1451A-80F-4-W #35 -Spot 161	3486	204912	1.7	11.2588	0.8	2.6758	1.8	0.2186	1.6	0.89	1274.4	18.3	1321.8	13.2	1399.4	15.8	1399.4	15.8
354U1451A-80F-4-W #35 -Spot 259	517	13605717	2.8	11.1819	1.0	2.9253	1.5	0.2373	1.2	0.78	1372.9	14.7	1388.5	11.6	1412.5	18.5	1412.5	18.5
354U1451A-80F-4-W #35 -Spot 149	361	159770	1.8	11.1352	0.7	3.0147	1.5	0.2436	1.4	0.88	1405.2	17.2	1411.3	11.8	1420.5	13.7	1420.5	13.7

354U1451A-80F-4-W #35 -Spot 97	62	44477	0.3	11.1005	0.7	3.0249	1.6	0.2436	1.4	0.90	1405.6	18.0	1413.9	12.1	1426.5	13.4	1426.5	13.4
354U1451A-80F-4-W #35 -Spot 101	242	62511	0.7	10.9389	0.7	2.7135	1.6	0.2154	1.4	0.88	1257.4	15.9	1332.1	11.7	1454.4	14.1	1454.4	14.1
354U1451A-80F-4-W #35 -Spot 115	296	199303	1.6	10.5208	0.7	3.4866	1.4	0.2662	1.2	0.85	1521.2	15.9	1524.2	10.9	1528.2	13.7	1528.2	13.7
354U1451A-80F-4-W #35 -Spot 153	940	70140	1.5	10.5009	0.6	3.1658	1.3	0.2412	1.1	0.86	1393.0	13.5	1448.9	9.7	1531.8	12.0	1531.8	12.0
354U1451A-80F-4-W #35 -Spot 6	619	2118900	1.1	10.1296	0.7	3.6207	1.4	0.2661	1.2	0.87	1521.1	16.4	1554.1	11.0	1599.3	12.5	1599.3	12.5
354U1451A-80F-4-W #35 -Spot 201	388	208154	2.3	10.0957	0.8	3.8706	1.5	0.2835	1.2	0.83	1609.1	17.1	1607.6	11.7	1605.5	15.1	1605.5	15.1
354U1451A-80F-4-W #35 -Spot 84	137	402786	2.4	10.0443	0.6	3.9242	1.3	0.2860	1.1	0.88	1621.5	15.7	1618.7	10.1	1615.0	11.2	1615.0	11.2
354U1451A-80F-4-W #35 -Spot 69	292	122483	1.3	9.9798	0.8	3.9881	1.8	0.2888	1.6	0.88	1635.5	22.9	1631.8	14.6	1627.0	15.5	1627.0	15.5
354U1451A-80F-4-W #35 -Spot 59	366	2552623	3.3	9.9548	0.6	3.9510	1.2	0.2854	1.0	0.87	1618.4	14.7	1624.2	9.6	1631.7	10.7	1631.7	10.7
354U1451A-80F-4-W #35 -Spot 181	227	159791	1.5	9.9341	0.7	3.4738	1.4	0.2504	1.2	0.87	1440.5	16.1	1521.3	11.4	1635.6	13.3	1635.6	13.3
354U1451A-80F-4-W #35 -Spot 211	119	1264748	0.7	9.9255	0.6	4.0799	1.3	0.2938	1.1	0.88	1660.6	16.8	1650.3	10.7	1637.2	11.6	1637.2	11.6
354U1451A-80F-4-W #35 -Spot 96	266	75271	1.3	9.8358	0.8	3.9523	1.5	0.2821	1.3	0.86	1601.7	18.2	1624.5	12.1	1654.0	14.3	1654.0	14.3
354U1451A-80F-4-W #35 -Spot 130	208	233614	0.5	9.8104	0.7	4.1251	1.6	0.2936	1.4	0.89	1659.7	21.1	1659.3	13.2	1658.8	13.6	1658.8	13.6
354U1451A-80F-4-W #35 -Spot 64	595	193488	2.0	9.7350	0.8	3.8815	1.4	0.2742	1.2	0.82	1562.0	16.0	1609.8	11.4	1673.1	15.2	1673.1	15.2
354U1451A-80F-4-W #35 -Spot 95	663	18296	1.2	9.6602	0.9	3.8214	1.7	0.2679	1.4	0.83	1529.9	18.8	1597.3	13.4	1687.3	17.2	1687.3	17.2
354U1451A-80F-4-W #35 -Spot 7	45	36390	0.8	9.6458	0.9	4.3075	1.3	0.3015	1.0	0.75	1698.6	14.7	1694.8	10.8	1690.1	16.0	1690.1	16.0
354U1451A-80F-4-W #35 -Spot 20	291	184709	4.6	9.5040	0.7	4.2525	1.6	0.2933	1.4	0.90	1657.8	20.9	1684.2	13.1	1717.3	12.6	1717.3	12.6
354U1451A-80F-4-W #35 -Spot 66	372	353158	6.6	9.4417	0.7	4.1873	1.3	0.2869	1.1	0.82	1625.8	15.5	1671.5	10.8	1729.4	13.7	1729.4	13.7
354U1451A-80F-4-W #35 -Spot 77	244	205290	0.8	9.4014	0.6	4.5956	1.3	0.3135	1.1	0.87	1757.8	17.1	1748.5	10.7	1737.3	11.6	1737.3	11.6
354U1451A-80F-4-W #35 -Spot 89	450	327825	1.6	9.3838	0.6	4.4702	1.2	0.3044	1.0	0.85	1712.9	15.4	1725.5	10.0	1740.7	11.5	1740.7	11.5
354U1451A-80F-4-W #35 -Spot 205	165	114601	1.4	9.3317	0.8	4.6071	1.7	0.3119	1.5	0.88	1750.3	22.4	1750.6	13.9	1750.9	14.6	1750.9	14.6
354U1451A-80F-4-W #35 -Spot 267	106	62040	0.9	9.2588	0.7	4.6209	1.6	0.3104	1.5	0.91	1742.8	22.8	1753.1	13.7	1765.2	12.3	1765.2	12.3
354U1451A-80F-4-W #35 -Spot 221	1265	484651	1.7	9.2389	0.9	4.7644	1.5	0.3194	1.2	0.79	1786.7	18.1	1778.6	12.2	1769.2	16.2	1769.2	16.2
354U1451A-80F-4-W #35 -Spot 250	459	24759	15.2	9.1802	0.7	4.4174	1.6	0.2942	1.5	0.90	1662.7	21.5	1715.6	13.5	1780.8	13.0	1780.8	13.0
354U1451A-80F-4-W #35 -Spot 141	190	144441	2.2	9.1626	0.8	3.9114	1.8	0.2600	1.5	0.88	1490.0	20.6	1616.0	14.2	1784.3	15.2	1784.3	15.2
354U1451A-80F-4-W #35 -Spot 136	619	225082	11.0	9.1558	0.9	4.8154	1.6	0.3199	1.3	0.83	1789.3	21.1	1787.6	13.6	1785.6	16.2	1785.6	16.2
354U1451A-80F-4-W #35 -Spot 150	155	92311	1.6	9.1415	0.8	4.7855	1.6	0.3174	1.4	0.86	1777.1	21.4	1782.4	13.4	1788.5	14.8	1788.5	14.8
354U1451A-80F-4-W #35 -Spot 243	1062	3228941	3.6	9.0936	0.7	4.9343	1.3	0.3256	1.1	0.84	1816.9	17.0	1808.1	10.8	1798.1	12.5	1798.1	12.5
354U1451A-80F-4-W #35 -Spot 191	678	1117238	2.3	8.9437	0.7	5.0282	1.2	0.3263	1.0	0.83	1820.4	16.2	1824.1	10.4	1828.3	12.3	1828.3	12.3
354U1451A-80F-4-W #35 -Spot 202	355	1927920	2.4	8.9323	0.6	4.9039	1.3	0.3178	1.1	0.89	1779.1	17.9	1802.9	10.9	1830.6	10.9	1830.6	10.9
354U1451A-80F-4-W #35 -Spot 226	453	116729	3.8	8.8981	0.8	4.9735	1.4	0.3211	1.2	0.82	1795.1	18.2	1814.8	12.0	1837.5	14.9	1837.5	14.9
354U1451A-80F-4-W #35 -Spot 272	294	196349	1.8	8.8772	0.7	5.0726	1.6	0.3267	1.4	0.90	1822.5	22.5	1831.5	13.3	1841.8	12.3	1841.8	12.3
354U1451A-80F-4-W #35 -Spot 307	441	1790727	3.7	8.7821	0.6	5.2992	1.3	0.3377	1.2	0.89	1875.4	18.9	1868.7	11.1	1861.2	10.6	1861.2	10.6
354U1451A-80F-4-W #35 -Spot 168	437	990460	3.0	8.7806	0.6	5.1987	1.3	0.3312	1.1	0.87	1844.2	17.7	1852.4	10.8	1861.6	11.1	1861.6	11.1
354U1451A-80F-4-W #35 -Spot 273	298	1588236	2.9	8.7767	0.6	5.2582	1.3	0.3349	1.1	0.89	1861.9	18.5	1862.1	11.0	1862.3	10.8	1862.3	10.8
354U1451A-80F-4-W #35 -Spot 265	796	516560	2.2	8.7570	0.6	5.4876	1.2	0.3487	1.0	0.85	1928.3	16.4	1898.7	9.9	1866.4	11.0	1866.4	11.0
354U1451A-80F-4-W #35 -Spot 195	219	94428	2.0	8.7526	0.7	5.3315	1.4	0.3386	1.2	0.85	1879.9	19.4	1873.9	11.9	1867.3	13.2	1867.3	13.2
354U1451A-80F-4-W #35 -Spot 94	207	37016	3.3	8.7061	0.7	5.1138	1.7	0.3230	1.5	0.92	1804.6	23.8	1838.4	14.1	1876.9	12.0	1876.9	12.0
354U1451A-80F-4-W #35 -Spot 167	443	7917389	1.3	8.4510	0.8	5.5434	1.3	0.3399	1.0	0.80	1886.3	16.8	1907.4	11.0	1930.4	13.8	1930.4	13.8
354U1451A-80F-4-W #35 -Spot 197	385	985742	3.2	8.0783	1.0	5.0076	2.1	0.2935	1.8	0.88	1659.1	26.7	1820.6	17.6	2010.7	17.5	2010.7	17.5
354U1451A-80F-4-W #35 -Spot 51	32	39140	2.0	7.8765	0.8	6.6244	1.2	0.3786	1.0	0.79	2069.7	17.1	2062.6	10.9	2055.5	13.4	2055.5	13.4
354U1451A-80F-4-W #35 -Spot 158	283	211340	2.5	7.2871	0.7	7.6062	1.4	0.4022	1.2	0.86	2179.0	21.8	2185.6	12.3	2191.7	12.0	2191.7	12.0
354U1451A-80F-4-W #35 -Spot 290	425	171128	11.8	6.8260	0.7	8.3028	1.3	0.4112	1.1	0.82	2220.5	19.9	2264.6	11.7	2304.7	12.8	2304.7	12.8
354U1451A-80F-4-W #35 -Spot 196	483	956022	2.3	6.7837	0.5	7.2261	1.2	0.3557	1.1	0.90	1961.7	17.8	2139.7	10.5	2315.4	8.8	2315.4	8.8
354U1451A-80F-4-W #35 -Spot 11	1131	1077048	10.8	6.7291	0.8	9.1298	1.3	0.4458	1.0	0.78	2376.4	20.8	2351.1	12.3	2329.2	14.5	2329.2	14.5
354U1451A-80F-4-W #35 -Spot 264	306	244359	2.6	6.4871	0.7	9.7114	1.3	0.4571	1.1	0.86	2426.8	23.2	2407.8	12.2	2391.7	11.4	2391.7	11.4
354U1451A-80F-4-W #35 -Spot 61	382	146859	2.3	6.3936	0.8	8.4518	1.4	0.3921	1.1	0.80	2132.5	20.1	2280.8	12.6	2416.4	14.0	2416.4	14.0
354U1451A-80F-4-W #35 -Spot 225	289	168540	0.6	6.3650	0.7	8.9582	1.2	0.4137	1.0	0.80	2231.9	18.9	2333.8	11.4	2424.0	12.6	2424.0	12.6
354U1451A-80F-4-W #35 -Spot 148	443	248522	1.8	6.2972	0.8	9.8229	1.5	0.4488	1.3	0.85	2390.0	25.2	2418.3	13.7	2442.2	13.2	2442.2	13.2
354U1451A-80F-4-W #35 -Spot 144	25	7682	1.9	6.1789	1.1	9.9126	1.7	0.4444	1.3	0.78	2370.4	26.8	2426.7	16.0	2474.2	18.4	2474.2	18.4
354U1451A-80F-4-W #35 -Spot 32	306	267254	1.6	6.1522	0.6	11.1048	1.2	0.4957	1.0	0.84	2595.4	21.1	2532.0	10.9	2481.5	10.6	2481.5	10.6
354U1451A-80F-4-W #35 -Spot 159	291	111654	1.2	6.1449	0.5	10.6334	1.1	0.4741	1.0	0.89	2501.5	20.8	2491.6	10.5	2483.5	8.8	2483.5	8.8
354U1451A-80F-4-W #35 -Spot 137	176	3796181	3.5	6.1419	0.7	10.4377	1.4	0.4652	1.2	0.85	2462.3	23.8	2474.4	12.7	2484.4	12.1	2484.4	12.1
354U1451A-80F-4-W #35 -Spot 216	224	1403753	1.7	6.0873	0.8	9.3370	1.5	0.4124	1.3	0.87	2225.9	24.6	2371.7	13.8	2499.4	12.7	2499.4	12.7
354U1451A-80F-4-W #35 -Spot 39	162	419767	2.1	6.0864	0.7	11.1280	1.4	0.4914	1.3	0.89	2576.9	27.1	2533.9	13.4	2499.7	11.1	2499.7	11.1
354U1451A-80F-4-W #35 -Spot 22	402	198503	0.5	6.0794	1.0	10.4886	1.6	0.4627	1.3	0.79	2451.3	26.1	2478.9	15.0	2501.6	16.8	2501.6	16.8
354U1451A-80F-4-W #35 -Spot 231	122	142613	2.1	6.0746	0.8	10.3191	1.3	0.4548	1.1	0.81	2416.7	21.3	2463.8	12.1	2502.9	12.9	2502.9	12.9
354U1451A-80F-4-W #35 -Spot 277	454	27854	2.7	6.0086	0.7	10.5483	1.6	0.4599	1.4	0.90	2439.0	29.4	2484.2	14.9	2521.3	11.7	2521.3	11.7
354U1451A-80F-4-W #35 -Spot 228	605	76449	3.9	5.7394	0.6	10.9602	1.1	0.4564	0.9	0.81	2423.8	18.3	2519.8	10.3	2598.0	10.7	2598.0	10.7
354U1451A-80F-4-W #35 -Spot 260	813	416746	25.3	5.6200	0.6	12.8224	1.2	0.5229	1.1	0.89	2711.4	24.5	2666.7	11.7	2633.0	9.5	2633.0	9.5
354U1451A-80F-4-W #35 -Spot 91	1215	623266	2.8	5.5996	0.7	12.6433	1.3	0.5137	1.1	0.85	2672.4	24.8	2653.4	12.6	2639.0	11.9	2639.0	11.9
354U1451A-80F-4-W #35 -Spot 83	339	370600	1.7	5.5835	0.5	11.8067	1.2	0.4783	1.0	0.89	2520.0	21.4	2589.2	10.8	2643.8	8.8	2643.8	8.8
354U1451A-80F-4-W #35 -Spot 118	398	258633	2.7	5.5406	0.9	13.0038	1.6	0.5228	1.4	0.83	2710.9	30.2	2679.9	15.4	2656.6	15.0	2656.6	15.0
354U1451A-80F-4-W #35 -Spot 127	158	421720	2.3	5.3591	0.7	13.2379	1.3	0.5148	1.1	0.84	2676.9	24.0	2696.8	12.3	2711.7	11.7	2711.7	11.7
354U1451A-80F-4-W #35 -Spot 252	30	50862	2.3	5.2192	0.7	14.1256	1.2	0.5349	1.0	0.82	2762.2	23.1	2758.2	11.9	2755.2	11.7	2755.2	11.7
354U1451A-80F-4-W #35 -Spot 257	307	3590746	1.0	5.1251	0.7	14.3148	1.4	0.5323	1.1	0.84	2751.2	25.5	2770.8	12.9	2785.1	12.2	2785.1	12.2
354U1451A-80F-4-W #35 -Spot 270	150	225720	1.8	4.3375	0.7	19.1914	1.1	0.6040	0.8	0.77	3045.9	19.7	3051.5	10.2	3055.1	10.9	3055.1	10.9
354U1451A-80F-4-W #35 -Spot 297	91	286938	1.7	4.3298	0.8	18.2079	1.3	0.5720	1.0	0.81	2916.1	24.2	3000.8	12.3	3058.0	12.2	3058.0	12.2
354U1451A-80F-4-W #35 -Spot 184	599	576801	1.9	3.6077	0.6	27.0254	1.2	0.7074	1.1	0.87	3448.8	29.0	3384.4	12.2	3346.4	9.5	3346.4	9.5

Table _____. U-Pb geochronologic analyses.																		
#21 U1451B_3X						Isotope ratios						Apparent ages (Ma)						
Late Miocene 10.2 Ma																		
Analysis	U (ppm)	206Pb 204Pb	U/Th	206Pb* 207Pb*	± (%)	207Pb* 235U*	± (%)	206Pb* 238U	± (%)	error corr.	206Pb* 238U*	± (Ma)	207Pb* 235U	± (Ma)	206Pb* 207Pb*	± (Ma)	Best age (Ma)	± (Ma)
-354-U1451-B3X-1W-13-28 Spot 277	386	23332	2.1	20.8984	2.5	0.0174	2.7	0.0026	1.0	0.36	17.0	0.2	17.5	0.5	90.8	60.0	17.0	0.2
-354-U1451-B3X-1W-13-28 Spot 54	1296	1964	1.5	8.4912	10.9	0.0466	11.3	0.0029	3.0	0.27	18.5	0.6	46.3	5.1	1921.8	196.5	18.5	0.6
-354-U1451-B3X-1W-13-28 Spot 45	162	4609	########	23.4216	4.2	0.0176	5.1	0.0030	3.0	0.58	19.3	0.6	17.7	0.9	NA	NA	19.3	0.6
-354-U1451-B3X-1W-13-28 Spot 205	637	11916	1.0	21.5023	2.1	0.0255	2.3	0.0040	1.1	0.47	25.6	0.3	25.6	0.6	22.9	49.2	25.6	0.3
-354-U1451-B3X-1W-13-28 Spot 256	90	12951	513.6	16.5252	7.1	0.0345	9.3	0.0041	6.0	0.65	26.6	1.6	34.5	3.2	621.0	153.4	26.6	1.6
-354-U1451-B3X-1W-13-28 Spot 188	970	20062	1.7	21.7321	1.3	0.0415	1.5	0.0065	0.8	0.53	42.0	0.3	41.3	0.6	NA	NA	42.0	0.3
-354-U1451-B3X-1W-13-28 Spot 58	678	40383	1.8	21.2343	1.4	0.0446	1.7	0.0069	1.0	0.57	44.2	0.4	44.3	0.7	52.9	32.8	44.2	0.4
-354-U1451-B3X-1W-13-28 Spot 214	1312	20090	1.4	20.7451	2.5	0.0472	2.7	0.0071	1.0	0.38	45.6	0.5	46.8	1.2	108.3	59.4	45.6	0.5
-354-U1451-B3X-1W-13-28 Spot 183	396	73433	1.4	20.3665	2.6	0.0496	2.8	0.0073	0.9	0.34	47.0	0.4	49.1	1.3	151.6	61.1	47.0	0.4
-354-U1451-B3X-1W-13-28 Spot 218	639	18210	1.5	21.4414	1.3	0.0493	1.9	0.0077	1.3	0.69	49.2	0.6	48.9	0.9	29.7	32.2	49.2	0.6
-354-U1451-B3X-1W-13-28 Spot 168	415	9694	1.1	16.6029	2.8	0.0651	3.1	0.0078	1.1	0.37	50.3	0.6	64.0	1.9	610.9	61.3	50.3	0.6
-354-U1451-B3X-1W-13-28 Spot 189	268	2916	1.2	23.4566	5.0	0.0464	5.1	0.0079	1.1	0.21	50.7	0.5	46.0	2.3	NA	NA	50.7	0.5
-354-U1451-B3X-1W-13-28 Spot 63	61	3020	1.2	23.5499	5.1	0.0475	5.7	0.0081	2.6	0.46	52.1	1.4	47.2	2.6	NA	NA	52.1	1.4
-354-U1451-B3X-1W-13-28 Spot 77	137	309211	1.4	21.2131	2.7	0.0534	2.9	0.0082	1.2	0.42	52.8	0.6	52.9	1.5	55.3	63.4	52.8	0.6
-354-U1451-B3X-1W-13-28 Spot 139	573	98125	8.5	21.5192	1.5	0.0538	1.8	0.0084	1.1	0.58	53.9	0.6	53.2	1.0	21.0	35.9	53.9	0.6
-354-U1451-B3X-1W-13-28 Spot 194	282	5454	1.6	21.5101	1.5	0.0545	1.9	0.0085	1.2	0.62	54.6	0.7	53.9	1.0	22.0	36.7	54.6	0.7
-354-U1451-B3X-1W-13-28 Spot 220	160	10664	1.2	22.0612	2.0	0.0588	2.2	0.0094	0.9	0.43	60.4	0.6	58.0	1.2	NA	NA	60.4	0.6
-354-U1451-B3X-1W-13-28 Spot 44	324	16631	1.6	15.4192	5.6	0.0851	5.7	0.0095	1.3	0.23	61.1	0.8	82.9	4.6	768.7	117.1	61.1	0.8
-354-U1451-B3X-1W-13-28 Spot 111	92	7100	0.9	21.1712	3.1	0.0642	3.3	0.0099	1.0	0.31	63.3	0.7	63.2	2.0	60.0	75.0	63.3	0.7
-354-U1451-B3X-1W-13-28 Spot 0	757	213425	11.6	19.8015	1.1	0.0696	5.5	0.0100	5.4	0.98	64.1	3.4	68.3	3.6	217.1	25.4	64.1	3.4
-354-U1451-B3X-1W-13-28 Spot 28	545	16216	1.4	21.6260	1.1	0.0668	1.4	0.0105	0.9	0.65	67.3	0.6	65.7	0.9	9.1	25.7	67.3	0.6
-354-U1451-B3X-1W-13-28 Spot 14	357	14099	1.3	20.9606	1.7	0.0696	1.9	0.0106	0.9	0.46	67.9	0.6	68.4	1.3	83.8	39.9	67.9	0.6
-354-U1451-B3X-1W-13-28 Spot 237	86	2866	2.2	22.0522	3.4	0.0685	3.7	0.0110	1.3	0.35	70.3	0.9	67.3	2.4	NA	NA	70.3	0.9
-354-U1451-B3X-1W-13-28 Spot 137	377	20847	1.8	21.2384	1.4	0.0712	1.9	0.0110	1.3	0.69	70.3	0.9	69.8	1.3	52.5	33.4	70.3	0.9
-354-U1451-B3X-1W-13-28 Spot 133	339	20316	3.2	21.5046	1.6	0.0782	2.6	0.0122	2.0	0.80	78.2	1.6	76.4	1.9	22.7	37.3	78.2	1.6
-354-U1451-B3X-1W-13-28 Spot 149	80	10376	1.3	22.2273	2.6	0.0797	2.8	0.0128	1.0	0.35	82.3	0.8	77.8	2.1	NA	NA	82.3	0.8
-354-U1451-B3X-1W-13-28 Spot 136	110	2242	1.1	22.4689	2.1	0.0872	2.2	0.0142	0.8	0.36	91.0	0.7	84.9	1.8	NA	NA	91.0	0.7
-354-U1451-B3X-1W-13-28 Spot 10	1877	283739	2.7	20.7344	0.6	0.0972	1.0	0.0146	0.7	0.76	93.6	0.7	94.2	0.9	109.5	15.2	93.6	0.7
-354-U1451-B3X-1W-13-28 Spot 227	616	18551	2.0	20.6673	1.1	0.1083	1.4	0.0162	0.9	0.63	103.8	0.9	104.4	1.4	117.1	25.5	103.8	0.9
-354-U1451-B3X-1W-13-28 Spot 190	163	14507	2.8	21.2410	1.8	0.1071	2.1	0.0165	1.0	0.48	105.6	1.0	103.3	2.0	52.2	43.6	105.6	1.0
-354-U1451-B3X-1W-13-28 Spot 93	1355	195699	2.4	20.8975	0.6	0.1109	1.0	0.0168	0.8	0.78	107.5	0.9	106.8	1.0	90.9	15.2	107.5	0.9
-354-U1451-B3X-1W-13-28 Spot 174	967	55012	7.0	20.7615	0.7	0.1123	1.1	0.0169	0.9	0.79	108.1	0.9	108.0	1.1	106.4	16.3	108.1	0.9
-354-U1451-B3X-1W-13-28 Spot 299	291	90696	5.2	20.6922	1.6	0.1150	2.3	0.0173	1.7	0.73	110.4	1.8	110.5	2.4	114.3	36.7	110.4	1.8
-354-U1451-B3X-1W-13-28 Spot 22	298	46659	2.3	21.2754	1.0	0.1128	1.3	0.0174	0.9	0.67	111.3	1.0	108.6	1.4	48.3	23.8	111.3	1.0
-354-U1451-B3X-1W-13-28 Spot 228	104	30374	1.3	20.7324	2.4	0.1339	2.5	0.0201	0.9	0.34	128.6	1.1	127.6	3.0	109.7	56.4	128.6	1.1
-354-U1451-B3X-1W-13-28 Spot 209	746	1301479	2.9	18.8692	0.6	0.2192	1.0	0.0300	0.8	0.79	190.6	1.5	201.2	1.9	327.7	14.2	190.6	1.5
-354-U1451-B3X-1W-13-28 Spot 264	5	2020	24.7	1.9692	3.3	2.1504	5.3	0.0307	4.1	0.78	195.1	7.9	1165.2	36.6	4263.5	NA	195.1	7.9
-354-U1451-B3X-1W-13-28 Spot 171	507	61534	2.2	19.0795	0.8	0.2894	1.1	0.0401	0.7	0.69	253.2	1.8	258.1	2.5	302.4	17.7	253.2	1.8
-354-U1451-B3X-1W-13-28 Spot 251	1356	133420	56.7	17.5475	0.5	0.3636	1.1	0.0463	1.0	0.88	291.7	2.9	314.9	3.1	490.1	11.8	291.7	2.9
-354-U1451-B3X-1W-13-28 Spot 35	1266	340004	2.7	17.7066	0.6	0.4507	1.9	0.0579	1.8	0.96	362.9	6.3	377.8	5.9	470.2	12.3	362.9	6.3
-354-U1451-B3X-1W-13-28 Spot 166	370	370684	6.6	17.3450	0.6	0.4618	1.2	0.0581	1.0	0.86	364.2	3.7	385.5	3.8	515.6	13.4	364.2	3.7
-354-U1451-B3X-1W-13-28 Spot 59	516	428135	4.2	16.0396	0.7	0.5060	5.3	0.0589	5.2	0.99	368.8	18.7	415.7	17.9	685.1	14.1	368.8	18.7
-354-U1451-B3X-1W-13-28 Spot 152	1323	352924	1.9	17.5016	0.6	0.5675	1.6	0.0721	1.4	0.92	448.6	6.3	456.4	5.8	495.9	13.8	448.6	6.3
-354-U1451-B3X-1W-13-28 Spot 276	3581	1068645	2.0	16.9236	0.6	0.6065	0.9	0.0745	0.7	0.77	463.1	3.0	481.3	3.3	569.5	12.0	463.1	3.0
-354-U1451-B3X-1W-13-28 Spot 244	419	74558	11.6	17.8105	0.7	0.5842	1.3	0.0755	1.1	0.84	469.2	5.0	467.1	4.9	457.2	15.7	469.2	5.0
-354-U1451-B3X-1W-13-28 Spot 187	242	67902	2.3	17.4230	0.8	0.6026	1.5	0.0762	1.3	0.83	473.3	5.7	478.9	5.8	505.8	18.5	473.3	5.7
-354-U1451-B3X-1W-13-28 Spot 274	657	114800	3.9	17.7080	0.6	0.5938	1.1	0.0763	0.9	0.84	474.0	4.1	473.3	4.1	470.0	13.0	474.0	4.1
-354-U1451-B3X-1W-13-28 Spot 124	1036	147329	4.9	17.6532	0.5	0.6105	1.0	0.0782	0.9	0.87	485.3	4.2	483.9	3.9	476.8	11.1	485.3	4.2
-354-U1451-B3X-1W-13-28 Spot 290	258	90302	2.4	17.5592	0.6	0.6200	1.1	0.0790	0.9	0.80	490.1	4.1	489.9	4.2	488.6	14.3	490.1	4.1
-354-U1451-B3X-1W-13-28 Spot 157	301	902169	2.2	17.6119	0.7	0.6213	1.2	0.0794	1.0	0.80	492.5	4.5	490.7	4.7	482.0	16.1	492.5	4.5
-354-U1451-B3X-1W-13-28 Spot 184	229	199280	1.5	16.5665	0.9	0.6623	1.1	0.0796	0.7	0.61	493.8	3.2	516.0	4.4	615.6	18.7	493.8	3.2
-354-U1451-B3X-1W-13-28 Spot 253	492	299880	2.1	17.2048	0.5	0.6455	1.0	0.0806	0.9	0.87	499.6	4.2	505.7	4.0	533.5	10.9	499.6	4.2
-354-U1451-B3X-1W-13-28 Spot 215	230	126749	0.7	17.3457	0.7	0.6442	1.1	0.0811	0.8	0.76	502.6	4.0	504.9	4.4	515.5	15.6	502.6	4.0
-354-U1451-B3X-1W-13-28 Spot 84	191	148998	0.6	16.9153	0.7	0.6660	0.9	0.0817	0.6	0.67	506.5	3.0	518.3	3.7	570.5	14.8	506.5	3.0
-354-U1451-B3X-1W-13-28 Spot 1	771	95305	4.1	16.6365	1.5	0.6824	4.0	0.0824	3.8	0.93	510.3	18.4	528.2	16.6	606.5	31.5	510.3	18.4
-354-U1451-B3X-1W-13-28 Spot 301	79	36196	1.5	17.4566	0.9	0.6529	1.6	0.0827	1.3	0.81	512.2	6.5	510.2	6.5	501.6	20.9	512.2	6.5
-354-U1451-B3X-1W-13-28 Spot 76	245	42303	1.3	17.2092	0.6	0.6664	1.2	0.0832	1.0	0.86	515.3	5.1	518.5	4.8	532.9	13.3	515.3	5.1
-354-U1451-B3X-1W-13-28 Spot 302	381	55024	1.9	17.1268	0.6	0.6718	1.1	0.0835	1.0	0.87	516.9	4.8	521.8	4.5	543.4	12.1	516.9	4.8
-354-U1451-B3X-1W-13-28 Spot 219	1726	4803596	47.2	17.1973	0.6	0.6752	0.9	0.0842	0.7	0.72	521.4	3.3	523.9	3.7	534.4	13.9	521.4	3.3
-354-U1451-B3X-1W-13-28 Spot 232	467	145015	1.1	17.1383	0.5	0.6856	0.9	0.0853	0.8	0.86	527.4	3.9	530.1	3.7	541.9	10.1	527.4	3.9
-354-U1451-B3X-1W-13-28 Spot 250	383	659020	2.3	16.9899	1.0	0.6982	2.6	0.0861	2.3	0.92	532.3	12.0	537.7	10.7	560.9	22.4	532.3	12.0
-354-U1451-B3X-1W-13-28 Spot 216	419	156870	3.0	17.0519	0.7	0.7097	1.1	0.0878	0.8	0.75	542.6	4.3	544.6	4.6	553.0	15.6	542.6	4.3
-354-U1451-B3X-1W-13-28 Spot 285	338	113084	5.4	17.2186	0.5	0.7049	1.3	0.0881	1.2	0.91	544.1	6.4	541.7	5.6	531.7	11.8	544.1	6.4
-354-U1451-B3X-1W-13-28 Spot 88	350	157485	10.1	17.0808	0.5	0.7159	0.9	0.0887	0.8	0.83	548.0	4.1	548.2	4.0	549.3	11.7	548.0	4.1
-354-U1451-B3X-1W-13-28 Spot 33	823	101315	8.3	16.6607	0.9	0.7482	2.9	0.0904	2.8	0.95	558.2	14.9	567.2	12.7	603.4	19.0	558.2	14.9
-354-U1451-B3X-1W-13-28 Spot 284	463	242753	9.2	16.7379	0.8	0.7872	2.4	0.0956	2.3	0.94	588.6	12.8	589.6	10.8	593.4	18.3	588.6	12.8
-354-U1451-B3X-1W-13-28 Spot 265	191	45523	0.9	16.2743	0.8	0.8221	1.1	0.0971	0.7	0.70	597.2	4.2	609.2	4.9	653.9	16.3	597.2	4.2
-354-U1451-B3X-1W-13-28 Spot 153	316	53750	3.2	16.6740	0.5	0.8443	0.9	0.1021	0.7	0.79	627.0	4.0	621.5	4.0	601.7	11.3	627.0	4.0
-354-U1451-B3X-1W-13-28 Spot 87	2092	242284	13.8	15.7813	0.7	0.8939	1.1	0.1024	0.8	0.79	628.2	5.0	648.5	5.1	719.6	13.9	628.2	5.0
-354-U1451-B3X-1W-13-28 Spot 243	412	1662471	10.4	16.3247	0.5	0.8667	0.9	0.1027	0.7	0.81	630.0	4.3	633.8	4.1	647.4	10.9	630.0	4.3
-354-U1451-B3X-1W-13-28 Spot 177	1174	166918	7.0	16.2259	0.5	0.8727	0.9	0.1027	0.7	0.78	630.5	4.1	637.0	4.1	660.4	11.7	630.5	4.1
-354-U1451-B3X-1W-13-28 Spot 195	438	401479	22.7	15.7319	0.6	0.9072	1.3	0.1036	1.1	0.86	635.2	6.6	655.5	6.1	726.2	13.7	635.2	6.6
-354-U1451-B3X-1W-13-28 Spot 41	1374	101354	6.9	15.6588	1.1	0.9137	1.8	0.1038	1.4	0.80	636.7	8.5	659.0	8.5	736.1	22.5	636.7	8.5
-354-U1451-B3X-1W-13-28 Spot 263	417	169843	3.9	16.2530	0.6	0.8805	1.0	0.1038	0.8	0.78	636.9	4.7	641.3	4.8	656.8	13.5	636.9	4.7
-354-U1451-B3X-1W-13-28 Spot 175	68	12318	73.6	15.8988	1.2	0.9086	2.4	0.1048	2.0	0.87	642.6	12.5	656.3	11.4	703.8	25.2	642.6	12.5
-354-U1451-B3X-1W-13-28 Spot 43	491	246780	5.2	16.1261	0.5	0.9037	0.8	0.1057	0.6	0.79	647.9	3.9	653.7	3.8	673.6	10.4	647.9	3.9
-354-U1451-B3X-1W-13-28 Spot 128	607	142097	7.8	16.4041	0.6	0.8911	1.2	0.1061	1.0	0.88	649.8	6.4	646.9	5.7	636.9	12.2	649.8	6.4
-354-U1451-B3X-1W-13-28 Spot 222	421	181434	2.3	16.0997	0.6	0.9244	1.0	0.1080	0.8	0.80	661.0	4.9	664.7	4.8	677.1	12.7	661.0	4.9
-354-U1451-B3X-1W-13-28 Spot 34	803	445854	3.8	15.0906	0.5	0.9889	1.7	0.1083	1.6	0.96	662.7	10.1	698.2	8.4	813.9	9.9	662.7	10.1
-354-U1451-B3X-1W-13-28 Spot 130	576	50470	2.0	15.6449	1.2	0.9943	1.9	0.1129	1.5	0.77	689.4	9.6	700.9	9.7	738.0	26.0	689.4	9.6
-354-U1451-B3X-1W-13-28 Spot 146	146	163956	1.4	15.7758	0.6	0.9982	1.1	0.1143	0.9	0.82	697.4	5.7	702.9	5.4	720.3	13.0	697.4	5.7
-354-U1451-B3X-1W-13-28 Spot 249	439	328743	2.1	15.8293	0.7	0.9958	1.1	0.1144	0.9	0.80	698.1	5.7	701.7	5.5	713.2	14.0	698.1	5.7
-354-U1451-B3X-1W-13-28 Spot 107	288	256163	1.6	15.6208	0.7	1.0358	1.4	0.1174	1.2	0.88	715.6	8.2	721.8	7.1	741.3	14.1	715.6	8.2
-354-U1451-B3X-1W-13-28 Spot 258	44	78135	433596.0	15.3994	1.0	1.0515	1.3	0.1175	0.9	0.68	716.1	6.0	729.6	6.8	771.4	20.0	716.1	6.0
-354-U1451-B3X-1W-13-28 Spot 97	267	218289	3.2	15.7217	0.6	1.0473	0.9	0.1195	0.7	0.78	727.5	4.9	727.5	4.8	727.7	12.4	727.5	4.9
-354-U1451-B3X-1W-13-28 Spot 180	167	185966	1.8	15.4288	0.6	1.0741	1.1	0.1202	0.9	0.81	732.0	6.0	740.7	5.7	767.4	13.4	732.0	6.0
-354-U1451-B3X-1W-13-28 Spot 48	651	951635	1.0	15.5950	0.6	1.0717	1.2	0.1213	1.0	0.88	737.8	7.2	739.6	6.2	744.8	11.8	737.8	7.2
-354-U1451-B3X-1W-13-28 Spot 270	286	92492	0.8	15.2051	0.5	1.1113	0.9	0.1226	0.7	0.81	745.5	5.0	758.8	4.7	798.1	11.0	745.5	5.0
-354-U1451-B3X-1W-13-28 Spot 262	164	57539	0.6	15.4198	0.9	1.1146	1.2	0.1247	0.9	0.72	757.6	6.4	760.4	6.7	768.6	18.2	757.6	6.4
-354-U1451-B3X-1W-13-28 Spot 125	369	531371	1.7	15.6118	0.5	1.1288	0.9	0.1279	0.8	0.82	775.7	5.5	767.2	5.0	742.5	11.1	775.7	5.5
-354-U1451-B3X-1W-13-28 Spot 230	206	63196	1.2	15.2248	0.6	1.1585	1.1	0.1280	0.9	0.82	776.3	6.3	781.3	5.7	795.3	12.7	776.3	6.3
-354-U1451-B3X-1W-13-28 Spot 185	2630	847821	2.3	14.5755	0.5	1.2124	0.9	0.1282	0.8	0.84	777.7	5.6	806.3	5.0	886.1	10.0	777.7	5.6
-354-U1451-B3X-1W-13-28 Spot 313	299	72626	0.7	15.1452	0.6	1.1704	1.3	0.1286	1.1	0.87	780.0	8.3	786.8	7.1	806.3	13.5	780.0	8.3
-354-U1451-B3X-1W-13-28 Spot 122	81	60184	1.1	15.1576	0.5	1.1730	1.7	0.1290	1.6	0.95	782.2	12.1	788.1	9.4	804.6	11.0	782.2	12.1

-354-U1451-B3X-1W-13-28 Spot 16	667	1535509	4.5	14.7648	0.9	1.2131	2.7	0.1300	2.5	0.95	787.7	18.8	806.6	14.9	859.3	17.7	787.7	18.8
-354-U1451-B3X-1W-13-28 Spot 199	814	333916	1.6	13.9591	0.5	1.2993	1.2	0.1316	1.1	0.91	797.0	8.1	845.4	6.8	974.8	10.0	797.0	8.1
-354-U1451-B3X-1W-13-28 Spot 206	65	25258	0.9	15.0934	0.9	1.2067	1.2	0.1322	0.8	0.64	800.1	5.9	803.7	6.8	813.5	19.7	800.1	5.9
-354-U1451-B3X-1W-13-28 Spot 39	1448	166675	4.4	14.6690	0.6	1.2459	1.3	0.1326	1.1	0.88	802.8	8.5	821.6	7.2	872.9	12.6	802.8	8.5
-354-U1451-B3X-1W-13-28 Spot 32	149	54930	2.1	14.6892	0.7	1.2646	2.6	0.1348	2.5	0.97	815.1	19.0	830.0	14.5	870.0	13.6	815.1	19.0
-354-U1451-B3X-1W-13-28 Spot 23	438	127781	1.4	14.5028	0.7	1.2889	1.2	0.1356	1.0	0.83	819.9	7.8	840.8	7.1	896.4	14.4	819.9	7.8
-354-U1451-B3X-1W-13-28 Spot 267	387	194286	1.8	14.8353	0.6	1.2778	1.3	0.1375	1.1	0.86	830.7	8.6	835.9	7.3	849.5	13.5	830.7	8.6
-354-U1451-B3X-1W-13-28 Spot 121	157	276045	0.8	14.8540	1.0	1.2769	1.5	0.1376	1.1	0.72	831.2	8.2	835.5	8.3	846.9	21.2	831.2	8.2
-354-U1451-B3X-1W-13-28 Spot 208	590	118724	1.4	14.4323	0.7	1.3284	1.1	0.1391	0.8	0.74	839.6	6.2	858.2	6.2	906.4	14.7	839.6	6.2
-354-U1451-B3X-1W-13-28 Spot 145	214	251491	3.2	14.6142	0.7	1.3361	1.1	0.1417	0.9	0.79	854.1	7.0	861.5	6.4	880.6	14.2	854.1	7.0
-354-U1451-B3X-1W-13-28 Spot 26	109	75366	2.0	14.7918	0.7	1.3220	1.2	0.1419	1.0	0.80	855.3	7.8	855.4	7.0	855.6	15.2	855.3	7.8
-354-U1451-B3X-1W-13-28 Spot 6	307	1099186	2.3	14.4161	0.7	1.3565	1.3	0.1419	1.1	0.84	855.3	8.6	870.4	7.5	908.8	14.4	855.3	8.6
-354-U1451-B3X-1W-13-28 Spot 246	493	247182	1.7	14.5963	0.5	1.3562	1.3	0.1436	1.2	0.93	865.2	10.1	870.3	7.8	883.2	9.8	865.2	10.1
-354-U1451-B3X-1W-13-28 Spot 36	490	701595	1.4	14.7463	0.5	1.3626	0.9	0.1458	0.8	0.83	877.4	6.2	873.0	5.3	862.0	10.4	877.4	6.2
-354-U1451-B3X-1W-13-28 Spot 241	607	223919	10.1	14.4902	0.5	1.3911	1.0	0.1463	0.8	0.86	880.0	7.0	885.2	5.8	898.2	10.1	880.0	7.0
-354-U1451-B3X-1W-13-28 Spot 13	72	93539	3.0	14.4360	1.0	1.3983	1.7	0.1465	1.4	0.82	881.1	11.5	888.2	10.1	905.9	20.2	881.1	11.5
-354-U1451-B3X-1W-13-28 Spot 160	165	113079	2.4	14.4770	0.6	1.4019	0.9	0.1473	0.8	0.81	885.6	6.3	889.7	5.6	900.1	11.4	885.6	6.3
-354-U1451-B3X-1W-13-28 Spot 307	600	10132349	1.4	14.5526	0.5	1.4048	1.0	0.1483	0.9	0.85	891.6	7.4	891.0	6.2	889.3	11.3	891.6	7.4
-354-U1451-B3X-1W-13-28 Spot 60	640	147872	2.5	14.4256	0.6	1.4720	1.0	0.1541	0.8	0.83	923.7	7.2	918.9	6.0	907.4	11.4	907.4	11.4
-354-U1451-B3X-1W-13-28 Spot 103	1099	389178	6.5	14.4194	0.4	1.4724	0.9	0.1540	0.8	0.89	923.6	6.6	919.1	5.2	908.3	8.2	908.3	8.2
-354-U1451-B3X-1W-13-28 Spot 30	116	82070	1.1	14.3693	0.7	1.4800	1.2	0.1543	0.9	0.78	925.0	7.9	922.2	7.2	915.5	15.4	915.5	15.4
-354-U1451-B3X-1W-13-28 Spot 275	576	161714	6.1	14.2451	0.5	1.4657	0.9	0.1515	0.8	0.81	909.3	6.4	916.4	5.6	933.3	11.0	933.3	11.0
-354-U1451-B3X-1W-13-28 Spot 66	30	109005	1.4	14.2381	0.8	1.5063	1.3	0.1556	1.1	0.80	932.4	9.4	932.9	8.2	934.3	16.6	934.3	16.6
-354-U1451-B3X-1W-13-28 Spot 309	235	100960	2.9	14.2283	0.5	1.4566	1.0	0.1504	0.8	0.84	903.1	6.9	912.6	5.9	935.7	11.1	935.7	11.1
-354-U1451-B3X-1W-13-28 Spot 314	406	361189	8.8	14.1844	0.6	1.4867	1.1	0.1530	0.9	0.85	917.8	8.1	925.0	6.8	942.1	12.0	942.1	12.0
-354-U1451-B3X-1W-13-28 Spot 306	112	34477	1.3	14.1596	0.7	1.5154	1.2	0.1557	0.9	0.78	932.8	7.9	936.6	7.2	945.6	15.2	945.6	15.2
-354-U1451-B3X-1W-13-28 Spot 129	254	46419	1.6	14.1418	0.6	1.5000	1.1	0.1539	0.9	0.85	922.9	7.7	930.4	6.4	948.2	11.4	948.2	11.4
-354-U1451-B3X-1W-13-28 Spot 310	281	219771	1.2	14.1343	0.6	1.5753	0.8	0.1616	0.6	0.73	965.4	5.3	960.5	5.1	949.3	11.5	949.3	11.5
-354-U1451-B3X-1W-13-28 Spot 91	359	115670	3.6	14.1252	0.5	1.6010	1.0	0.1641	0.9	0.84	979.5	7.8	970.6	6.4	950.6	11.2	950.6	11.2
-354-U1451-B3X-1W-13-28 Spot 140	536	114255	2.4	14.0931	0.6	1.5916	0.9	0.1627	0.6	0.73	972.0	5.8	966.9	5.4	955.3	12.1	955.3	12.1
-354-U1451-B3X-1W-13-28 Spot 95	547	192392	1.8	14.0899	0.5	1.4948	1.2	0.1528	1.0	0.90	916.8	9.0	928.3	7.0	955.7	10.1	955.7	10.1
-354-U1451-B3X-1W-13-28 Spot 260	39	18633	2.3	14.0724	1.1	1.5842	1.3	0.1618	0.7	0.53	966.5	6.2	964.0	8.1	958.3	22.7	958.3	22.7
-354-U1451-B3X-1W-13-28 Spot 248	767	311026	6.6	14.0472	0.5	1.3896	1.1	0.1416	0.9	0.89	853.9	7.5	884.5	6.2	962.0	9.9	962.0	9.9
-354-U1451-B3X-1W-13-28 Spot 165	97	556395	2.2	14.0462	0.8	1.5735	1.0	0.1604	0.7	0.65	958.8	5.8	959.8	6.2	962.1	15.5	962.1	15.5
-354-U1451-B3X-1W-13-28 Spot 176	148	69007	0.9	14.0311	0.7	1.5522	1.0	0.1580	0.8	0.76	945.8	6.9	951.4	6.4	964.3	14.0	964.3	14.0
-354-U1451-B3X-1W-13-28 Spot 304	685	907782	8.0	13.9958	0.7	1.6668	1.2	0.1693	0.9	0.79	1008.1	8.8	996.0	7.5	969.4	14.8	969.4	14.8
-354-U1451-B3X-1W-13-28 Spot 113	361	702816	2.1	13.9835	0.6	1.5972	1.3	0.1621	1.2	0.91	968.2	11.0	969.1	8.4	971.2	11.4	971.2	11.4
-354-U1451-B3X-1W-13-28 Spot 53	888	92879	2.2	13.9603	0.5	1.4332	0.8	0.1452	0.6	0.78	873.9	5.1	902.9	4.8	974.6	10.1	974.6	10.1
-354-U1451-B3X-1W-13-28 Spot 289	377	107458	3.5	13.9536	0.6	1.6021	1.3	0.1622	1.2	0.89	969.0	10.5	971.0	8.2	975.6	12.1	975.6	12.1
-354-U1451-B3X-1W-13-28 Spot 281	161	45969	0.8	13.9440	0.7	1.6001	0.9	0.1619	0.6	0.66	967.3	5.5	970.2	5.8	977.0	14.1	977.0	14.1
-354-U1451-B3X-1W-13-28 Spot 57	1015	794340	3.1	13.9181	0.5	1.6060	0.7	0.1622	0.5	0.68	968.9	4.3	972.5	4.4	980.8	10.4	980.8	10.4
-354-U1451-B3X-1W-13-28 Spot 280	181	111706	1.3	13.8997	0.6	1.5854	0.9	0.1599	0.7	0.77	956.2	6.2	964.5	5.6	983.5	11.8	983.5	11.8
-354-U1451-B3X-1W-13-28 Spot 311	682	231196	2.5	13.8977	0.5	1.5412	0.7	0.1554	0.6	0.79	931.3	5.1	947.0	4.6	983.7	9.3	983.7	9.3
-354-U1451-B3X-1W-13-28 Spot 150	164	20249	4.3	13.8865	0.7	1.4215	1.0	0.1432	0.7	0.73	862.9	5.8	898.0	5.9	985.4	13.7	985.4	13.7
-354-U1451-B3X-1W-13-28 Spot 117	375	101689	2.8	13.8628	0.6	1.4548	1.3	0.1463	1.1	0.88	880.4	9.5	911.9	7.9	988.9	12.9	988.9	12.9
-354-U1451-B3X-1W-13-28 Spot 51	295	325121	4.8	13.8610	0.5	1.6376	1.0	0.1647	0.9	0.87	982.8	8.2	984.8	6.5	989.2	10.1	989.2	10.1
-354-U1451-B3X-1W-13-28 Spot 138	275	159040	1.5	13.8568	0.6	1.5970	1.4	0.1606	1.3	0.90	959.9	11.6	969.1	9.0	989.8	12.5	989.8	12.5
-354-U1451-B3X-1W-13-28 Spot 178	100	155888	2.7	13.8567	0.7	1.5763	1.0	0.1585	0.7	0.71	948.4	6.3	960.9	6.3	989.8	14.3	989.8	14.3
-354-U1451-B3X-1W-13-28 Spot 9	315	406243	1.4	13.8502	0.5	1.6958	0.9	0.1704	0.8	0.86	1014.4	7.5	1007.0	5.9	990.7	9.5	990.7	9.5
-354-U1451-B3X-1W-13-28 Spot 202	395	1791306	5.1	13.8093	0.5	1.6327	0.9	0.1636	0.7	0.81	976.7	6.4	982.9	5.5	996.7	10.5	996.7	10.5
-354-U1451-B3X-1W-13-28 Spot 71	417	580840	2.4	13.8019	0.6	1.6262	1.2	0.1629	1.0	0.86	972.6	9.1	980.4	7.4	997.8	12.5	997.8	12.5
-354-U1451-B3X-1W-13-28 Spot 268	1197	202823	3.1	13.7585	0.6	1.6012	1.4	0.1598	1.2	0.90	955.9	10.9	970.7	8.5	1004.2	12.1	1004.2	12.1
-354-U1451-B3X-1W-13-28 Spot 52	104	32888	0.9	13.7530	0.8	1.5782	1.2	0.1575	1.0	0.77	942.8	8.4	961.7	7.7	1005.0	16.0	1005.0	16.0
-354-U1451-B3X-1W-13-28 Spot 201	64	118457	2.0	13.7175	0.9	1.6245	1.2	0.1617	0.9	0.72	966.2	8.0	979.7	7.8	1010.3	17.7	1010.3	17.7
-354-U1451-B3X-1W-13-28 Spot 303	127	89594	2.4	13.7095	0.5	1.4848	0.8	0.1477	0.7	0.82	888.1	5.6	924.2	5.0	1011.4	9.6	1011.4	9.6
-354-U1451-B3X-1W-13-28 Spot 286	424	304972	2.8	13.6485	0.6	1.6064	1.1	0.1591	0.9	0.82	951.7	7.9	972.7	6.8	1020.5	12.7	1020.5	12.7
-354-U1451-B3X-1W-13-28 Spot 158	537	497326	78.1	13.5960	0.5	1.7886	1.6	0.1764	1.6	0.95	1047.5	15.1	1041.3	10.7	1028.3	10.5	1028.3	10.5
-354-U1451-B3X-1W-13-28 Spot 210	280	149743	3.8	13.4305	0.6	1.7722	1.2	0.1727	1.0	0.85	1027.0	9.4	1035.3	7.6	1053.0	12.4	1053.0	12.4
-354-U1451-B3X-1W-13-28 Spot 169	288	224129	2.2	13.4285	0.7	1.9006	1.1	0.1852	0.9	0.80	1095.2	8.8	1081.3	7.3	1053.3	13.4	1053.3	13.4
-354-U1451-B3X-1W-13-28 Spot 305	117	47611	4.1	13.3625	0.7	1.8749	1.2	0.1818	1.0	0.81	1076.7	10.0	1072.2	8.2	1063.2	14.5	1063.2	14.5
-354-U1451-B3X-1W-13-28 Spot 235	220	75320	2.4	13.3565	0.4	1.6504	1.3	0.1599	1.3	0.95	956.5	11.3	989.7	8.5	1064.2	8.3	1064.2	8.3
-354-U1451-B3X-1W-13-28 Spot 164	317	347220	3.5	13.3214	0.5	1.8053	1.0	0.1745	0.8	0.85	1036.8	7.9	1047.4	6.4	1069.5	10.4	1069.5	10.4
-354-U1451-B3X-1W-13-28 Spot 102	752	688154	5.9	13.3096	0.6	1.8961	1.5	0.1831	1.3	0.90	1084.0	13.3	1079.7	9.8	1071.2	13.0	1071.2	13.0
-354-U1451-B3X-1W-13-28 Spot 82	791	1069186	21.4	13.3059	0.4	1.8667	0.9	0.1802	0.8	0.88	1068.2	7.7	1069.3	5.9	1071.8	8.6	1071.8	8.6
-354-U1451-B3X-1W-13-28 Spot 85	550	5017868	4.8	13.2734	0.6	1.9453	1.3	0.1873	1.2	0.89	1107.0	11.8	1096.8	8.7	1076.7	11.9	1076.7	11.9
-354-U1451-B3X-1W-13-28 Spot 156	379	178184	5.1	13.1992	0.5	1.6574	1.1	0.1587	0.9	0.87	949.7	8.3	992.4	6.9	1087.9	10.6	1087.9	10.6
-354-U1451-B3X-1W-13-28 Spot 2	413	228743	38.9	13.1926	0.9	1.8890	1.9	0.1808	1.7	0.88	1071.5	16.8	1077.2	12.9	1088.9	18.8	1088.9	18.8
-354-U1451-B3X-1W-13-28 Spot 172	378	161702	2.6	13.1277	0.5	1.9104	1.1	0.1820	0.9	0.87	1077.7	9.1	1084.7	7.0	1098.8	10.4	1098.8	10.4
-354-U1451-B3X-1W-13-28 Spot 134	90	53276	1.0	13.1240	0.7	1.8496	1.1	0.1761	0.8	0.73	1045.8	7.8	1063.3	7.2	1099.4	14.9	1099.4	14.9
-354-U1451-B3X-1W-13-28 Spot 229	144	6700336	2.5	13.1119	0.9	1.8470	1.4	0.1757	1.1	0.77	1043.5	10.4	1062.3	9.2	1101.2	17.6	1101.2	17.6
-354-U1451-B3X-1W-13-28 Spot 7	705	175437	1.8	13.1087	0.4	1.9880	0.8	0.1891	0.7	0.89	1116.4	7.2	1111.4	5.3	1101.7	7.1	1101.7	7.1
-354-U1451-B3X-1W-13-28 Spot 291	343	787384	3.2	13.1065	0.6	1.9572	1.1	0.1861	0.9	0.83	1100.4	8.9	1100.9	7.1	1102.0	11.9	1102.0	11.9
-354-U1451-B3X-1W-13-28 Spot 109	345	9550950	4.9	13.0914	0.5	2.0731	1.0	0.1969	0.9	0.88	1158.8	9.5	1140.0	7.0	1104.4	9.7	1104.4	9.7
-354-U1451-B3X-1W-13-28 Spot 108	75	359621	1.6	13.0678	0.8	1.8406	1.1	0.1745	0.7	0.69	1037.0	7.0	1060.1	7.0	1107.9	15.4	1107.9	15.4
-354-U1451-B3X-1W-13-28 Spot 252	421	143244	4.2	13.0535	0.7	1.9395	1.2	0.1837	1.0	0.82	1087.2	10.1	1094.8	8.2	1110.1	14.0	1110.1	14.0
-354-U1451-B3X-1W-13-28 Spot 61	94	395502	1.9	12.9829	0.7	1.9968	1.0	0.1881	0.7	0.71	1111.1	7.6	1114.4	7.0	1121.0	14.5	1121.0	14.5
-354-U1451-B3X-1W-13-28 Spot 255	234	81659	1.2	12.8696	0.7	2.0642	1.2	0.1927	1.0	0.83	1136.3	10.5	1137.0	8.2	1138.4	13.2	1138.4	13.2
-354-U1451-B3X-1W-13-28 Spot 148	232	171586	1.9	12.8606	0.5	2.0309	0.9	0.1895	0.8	0.87	1118.7	8.4	1125.9	6.4	1139.8	9.4	1139.8	9.4
-354-U1451-B3X-1W-13-28 Spot 96	808	88894	1.2	12.7918	0.5	1.8075	1.0	0.1678	0.9	0.89	999.8	8.5	1048.2	6.8	1150.4	9.4	1150.4	9.4
-354-U1451-B3X-1W-13-28 Spot 114	151	3922042	2.1	12.7299	0.6	2.1033	0.9	0.1943	0.7	0.76	1144.5	7.1	1149.9	6.2	1160.1	11.7	1160.1	11.7
-354-U1451-B3X-1W-13-28 Spot 159	828	250369	3.4	12.6643	0.6	2.1893	1.0	0.2012	0.8	0.82	1181.7	9.1	1177.7	7.2	1170.3	11.7	1170.3	11.7
-354-U1451-B3X-1W-13-28 Spot 254	287	2121876	2.2	12.6132	0.5	2.2285	0.9	0.2040	0.7	0.82	1196.5	7.7	1190.0	6.1	1178.3	9.9	1178.3	9.9
-354-U1451-B3X-1W-13-28 Spot 191	301	193680	6.3	12.5352	0.6	2.2336	1.1	0.2031	0.9	0.84	1192.2	9.9	1191.6	7.6	1190.6	11.7	1190.6	11.7
-354-U1451-B3X-1W-13-28 Spot 161	273	150969	2.4	12.5225	0.4	2.1795	1.1	0.1980	1.0	0.92	1164.8	10.8	1174.5	7.7	1192.6	8.7	1192.6	8.7
-354-U1451-B3X-1W-13-28 Spot 94	315	176030	2.3	12.2688	0.6	2.1696	1.3	0.1931	1.1	0.90	1138.3	12.0	1171.3	8.9	1232.9	11.0	1232.9	11.0
-354-U1451-B3X-1W-13-28 Spot 211	49	658444	1.4	12.2022	0.7	2.4351	1.1	0.2156	0.8	0.72	1258.6	8.7	1253.0	7.7	1243.5	14.5	1243.5	14.5
-354-U1451-B3X-1W-13-28 Spot 239	824	295387	4.4	12.0514	0.7	2.4537	0.9	0.2146	0.6	0.66	1253.1	6.9	1258.5	6.6	1267.8	13.4	1267.8	13.4
-354-U1451-B3X-1W-13-28 Spot 238	146	49584	1.2	11.9994	0.5	2.4431	0.9	0.2127	0.8	0.82	1243.3	8.7	1255.4	6.8	1276.3	10.5	1276.3	10.5
-354-U1451-B3X-1W-13-28 Spot 279	267	154097	1.8	11.8672	0.5	2.5846	1.1	0.2225	1.0	0.90	1295.3	11.8	1296.3	8.2	1297.8	9.7	1297.8	9.7
-354-U1451-B3X-1W-13-28 Spot 81	1344	904472	6.2	11.7546	0.5	2.4806	0.9	0.2116	0.7	0.84	1237.2	8.3	1266.4	6.3	1316.3	9.1	1316.3	9.1
-354-U1451-B3X-1W-13-28 Spot 104	1023	23729703	2.2	11.7266	0.7	2.4359	2.2	0.2073	2.1	0.95	1214.2	23.5	1253.3	16.1	1321.0	13.8	1321.0	13.8
-354-U1451-B3X-1W-13-28 Spot 101	769	331587	12.6	11.5652	0.5	2.7973	1.0	0.2347	0.8	0.85	1359.3	10.0	1354.8	7.1	1347.8	9.6	1347.8	9.6
-354-U1451-B3X-1W-13-28 Spot 112	303	100836	1.7	11.5569	0.4	2.5804	0.7	0.2164	0.6	0.82	1262.7	6.4	1295.1	5.0	1349.2	7.6	1349.2	7.6
-354-U1451-B3X-1W-13-28 Spot 78	121	266270	2.6	11.3374	0.5	2.8093	0.8	0.2311	0.6	0.81	1340.3	7.5	1358.0	5.7	1386.1	8.7	1386.1	8.7
-354-U1451-B3X-1W-13-28 Spot 31	495	429210	3.5	11.2903	1.5	2.7319	2.0	0.2238	1.2	0.64	1301.9	14.7	1337.2	14.5	1394.1	28.8	1394.1	28.8
-354-U1451-B3X-1W-13-28 Spot 221	178	204372	2.1	10.9966	0.5	2.9759	0.8	0.2374	0.7	0.80	1373.4	8.1	1401.5	6.2	1444.4	9.3	1444.4	9.3
-354-U1451-B3X-1W-13-28 Spot 193	261	114814	1.6	10.9001	0.5	3.2175	1.2	0.2545	1.1	0.90	1461.5	13.8	1461.4	9.1	1461.2	9.9	1461.2	9.9
-354-U1451-B3X-1W-13-28 Spot 282	57	364159	1.8	10.8039	0.7	3.2783	1.0	0.2570	0.8	0.73	1474.4	10.1	1475.9	8.1	1478.0	13.5	1478.0	13.5
-354-U1451-B3X-1W-13-28 Spot 24	428	105754	1.7	10.4609	0.4	3.4019	0.9	0.2582	0.8	0.88	1480.7	10.7	1504.8	7.2	1539.0	8.1	1539.0	8.1
-354-U1451-B3X-1W-13-28 Spot 144	421	173344	4.0	10.4466	0.5	3.2337	1.4	0.2451	1.3	0.93	1413.2	16.9	1465.3	11.1	1541.5	10.0	1541.5	10.0
-354-U1451-B3X-1W-13-28 Spot 288	277	977371	0.7	10.4335	0.5	3.3612	0.8	0.2545	0.6	0.75	1461.4	8.0	1495.4	6.4	1543.9	10.2	1543.9	10.2

-354-U1451-B3X-1W-13-28 Spot 181	255	299519	1.0	10.4068	0.5	3.5420	1.1	0.2675	0.9	0.86	1527.9	12.5	1536.6	8.5	1548.7	10.2	1548.7	10.2
-354-U1451-B3X-1W-13-28 Spot 297	969	223507	2.0	10.3446	0.6	3.0539	1.6	0.2292	1.5	0.93	1330.4	18.3	1421.2	12.5	1560.0	11.5	1560.0	11.5
-354-U1451-B3X-1W-13-28 Spot 245	177	242624	4.6	10.3311	0.7	3.2350	2.4	0.2425	2.3	0.96	1399.7	29.1	1465.6	18.8	1562.4	13.1	1562.4	13.1
-354-U1451-B3X-1W-13-28 Spot 49	449	2803728	1.2	10.2299	0.4	3.8242	1.2	0.2839	1.1	0.95	1610.8	16.0	1597.9	9.5	1580.9	6.9	1580.9	6.9
-354-U1451-B3X-1W-13-28 Spot 19	236	140813	3.2	10.1468	0.4	3.6365	1.0	0.2677	0.9	0.92	1529.3	12.6	1557.6	8.0	1596.1	7.6	1596.1	7.6
-354-U1451-B3X-1W-13-28 Spot 29	1244	282218	2.7	10.0924	0.5	3.2653	0.8	0.2391	0.7	0.81	1382.1	8.3	1472.8	6.4	1606.1	8.9	1606.1	8.9
-354-U1451-B3X-1W-13-28 Spot 198	253	367226	8.0	10.0752	0.4	3.6127	1.0	0.2641	0.9	0.93	1510.8	12.7	1552.3	8.1	1609.3	7.2	1609.3	7.2
-354-U1451-B3X-1W-13-28 Spot 110	225	256776	1.2	10.0630	0.5	3.9055	1.0	0.2852	0.8	0.87	1617.3	12.1	1614.8	7.8	1611.6	8.8	1611.6	8.8
-354-U1451-B3X-1W-13-28 Spot 75	166	229936	2.2	10.0308	0.5	4.0109	0.9	0.2919	0.7	0.82	1651.1	10.5	1636.4	7.2	1617.5	9.5	1617.5	9.5
-354-U1451-B3X-1W-13-28 Spot 50	742	98585	2.0	9.9478	0.6	3.5013	1.7	0.2527	1.6	0.93	1452.5	20.3	1527.5	13.2	1633.0	11.1	1633.0	11.1
-354-U1451-B3X-1W-13-28 Spot 18	296	421110	1.6	9.9395	0.4	4.0942	0.9	0.2953	0.8	0.88	1667.8	11.1	1653.2	7.0	1634.5	7.5	1634.5	7.5
-354-U1451-B3X-1W-13-28 Spot 294	266	308706	1.8	9.8766	0.5	4.2519	1.0	0.3047	0.9	0.88	1714.6	13.0	1684.1	8.1	1646.3	8.7	1646.3	8.7
-354-U1451-B3X-1W-13-28 Spot 212	813	572540	14.5	9.6008	0.8	3.4009	1.3	0.2369	1.1	0.79	1370.6	13.1	1504.6	10.5	1698.7	15.3	1698.7	15.3
-354-U1451-B3X-1W-13-28 Spot 127	370	366388	1.6	9.4813	0.4	4.4960	0.9	0.3093	0.8	0.89	1737.3	12.4	1730.2	7.6	1721.7	7.8	1721.7	7.8
-354-U1451-B3X-1W-13-28 Spot 151	539	653629	1.5	9.4694	0.5	4.5435	1.0	0.3122	0.9	0.86	1751.4	13.3	1739.0	8.4	1724.0	9.4	1724.0	9.4
-354-U1451-B3X-1W-13-28 Spot 40	1212	101507	0.9	9.3322	0.4	3.7804	0.7	0.2560	0.6	0.87	1469.3	8.3	1588.6	5.8	1750.8	6.5	1750.8	6.5
-354-U1451-B3X-1W-13-28 Spot 217	128	98157	4.3	9.2722	0.6	4.6515	1.0	0.3129	0.9	0.82	1755.2	13.1	1758.6	8.7	1762.6	10.8	1762.6	10.8
-354-U1451-B3X-1W-13-28 Spot 119	213	45162	1.0	9.1683	0.6	4.0450	1.0	0.2691	0.8	0.81	1536.2	11.1	1643.3	8.1	1783.1	10.5	1783.1	10.5
-354-U1451-B3X-1W-13-28 Spot 295	270	370749	2.1	9.0036	0.6	4.8432	1.1	0.3164	0.9	0.83	1772.1	14.0	1792.4	9.1	1816.1	10.9	1816.1	10.9
-354-U1451-B3X-1W-13-28 Spot 106	114	114521	0.8	8.8499	0.4	5.0895	0.8	0.3268	0.6	0.83	1822.9	10.1	1834.4	6.5	1847.3	7.7	1847.3	7.7
-354-U1451-B3X-1W-13-28 Spot 226	440	2789495	2.2	8.8300	0.5	4.9615	1.1	0.3179	1.0	0.88	1779.4	15.1	1812.8	9.3	1851.4	9.4	1851.4	9.4
-354-U1451-B3X-1W-13-28 Spot 55	398	634132	12.7	8.8261	0.4	5.2674	1.1	0.3373	1.0	0.92	1873.8	16.4	1863.6	9.4	1852.2	8.0	1852.2	8.0
-354-U1451-B3X-1W-13-28 Spot 99	301	301370	8.0	8.8117	0.4	4.9730	0.9	0.3180	0.7	0.87	1779.7	11.5	1814.7	7.2	1855.2	7.7	1855.2	7.7
-354-U1451-B3X-1W-13-28 Spot 155	289	1236776	3.2	8.8019	0.5	5.4309	0.8	0.3468	0.7	0.84	1919.5	11.5	1889.8	7.1	1857.2	8.2	1857.2	8.2
-354-U1451-B3X-1W-13-28 Spot 259	281	816350	3.3	8.7921	0.5	5.2721	0.8	0.3363	0.6	0.76	1869.0	9.3	1864.4	6.4	1859.2	8.9	1859.2	8.9
-354-U1451-B3X-1W-13-28 Spot 116	1204	179379	3.8	8.7878	0.6	4.3902	1.5	0.2799	1.3	0.91	1591.0	18.7	1710.5	12.0	1860.1	10.6	1860.1	10.6
-354-U1451-B3X-1W-13-28 Spot 120	257	219557	2.0	8.7568	0.6	5.5658	1.1	0.3536	1.0	0.85	1951.9	16.1	1910.8	9.6	1866.4	10.5	1866.4	10.5
-354-U1451-B3X-1W-13-28 Spot 213	578	1081094	12.0	8.7444	0.6	5.1732	1.1	0.3282	0.9	0.82	1829.8	13.7	1848.2	9.0	1869.0	10.9	1869.0	10.9
-354-U1451-B3X-1W-13-28 Spot 203	788	463055	1.3	8.7421	0.4	5.1285	0.8	0.3253	0.8	0.90	1815.6	12.0	1840.8	7.1	1869.5	6.5	1869.5	6.5
-354-U1451-B3X-1W-13-28 Spot 173	852	1710388	2.2	8.7326	0.5	5.1363	1.0	0.3254	0.9	0.89	1816.3	14.2	1842.1	8.6	1871.4	8.4	1871.4	8.4
-354-U1451-B3X-1W-13-28 Spot 186	135	132695	2.0	8.6869	0.5	5.2364	0.8	0.3301	0.6	0.81	1838.6	10.2	1858.6	6.7	1880.9	8.2	1880.9	8.2
-354-U1451-B3X-1W-13-28 Spot 200	713	14034466	1.9	8.6795	0.5	5.1719	1.0	0.3257	0.9	0.87	1817.6	13.6	1848.0	8.3	1882.4	8.6	1882.4	8.6
-354-U1451-B3X-1W-13-28 Spot 261	140	134361	3.3	8.6749	0.6	5.2657	1.1	0.3314	0.9	0.82	1845.4	13.9	1863.3	9.1	1883.4	11.0	1883.4	11.0
-354-U1451-B3X-1W-13-28 Spot 70	993	414390	5.3	8.5515	0.5	5.2978	1.0	0.3287	0.8	0.85	1832.2	12.9	1868.5	8.2	1909.2	9.1	1909.2	9.1
-354-U1451-B3X-1W-13-28 Spot 65	1521	176603	12.2	8.4427	0.6	4.7635	0.9	0.2918	0.7	0.75	1650.5	9.8	1778.5	7.5	1932.1	10.7	1932.1	10.7
-354-U1451-B3X-1W-13-28 Spot 12	308	240107	4.6	6.9090	0.4	8.3183	1.1	0.4170	1.0	0.92	2246.9	19.4	2266.3	10.0	2283.9	7.4	2283.9	7.4
-354-U1451-B3X-1W-13-28 Spot 278	201	2977004	1.9	6.8482	0.5	8.0361	1.1	0.3993	1.0	0.88	2165.9	17.6	2235.1	9.8	2299.1	8.9	2299.1	8.9
-354-U1451-B3X-1W-13-28 Spot 98	356	470350	1.0	6.4512	0.6	8.6774	1.2	0.4062	1.0	0.88	2197.4	19.4	2304.7	10.8	2401.2	9.7	2401.2	9.7
-354-U1451-B3X-1W-13-28 Spot 47	165	240674	1.8	6.4079	0.5	9.7657	1.3	0.4541	1.2	0.91	2413.2	23.2	2412.9	11.6	2412.6	8.8	2412.6	8.8
-354-U1451-B3X-1W-13-28 Spot 11	90	110539	1.2	6.3045	0.6	8.7434	0.9	0.4000	0.7	0.76	2168.8	12.9	2311.6	8.4	2440.2	10.0	2440.2	10.0
-354-U1451-B3X-1W-13-28 Spot 312	265	1204334	3.7	6.2638	0.5	9.2542	1.3	0.4206	1.2	0.91	2263.2	22.3	2363.5	11.7	2451.2	8.7	2451.2	8.7
-354-U1451-B3X-1W-13-28 Spot 154	764	448581	36.3	6.2145	0.7	9.6602	2.7	0.4356	2.6	0.97	2330.9	50.7	2402.9	24.7	2464.5	11.8	2464.5	11.8
-354-U1451-B3X-1W-13-28 Spot 225	420	95082	3.5	6.2099	0.5	9.2241	1.0	0.4156	0.8	0.85	2240.6	15.9	2360.5	9.1	2465.8	8.9	2465.8	8.9
-354-U1451-B3X-1W-13-28 Spot 83	179	148411	1.3	6.1812	0.5	9.9928	0.9	0.4482	0.7	0.82	2387.1	14.2	2434.1	8.0	2473.6	8.3	2473.6	8.3
-354-U1451-B3X-1W-13-28 Spot 73	255	230727	1.1	6.1798	0.6	10.6785	1.1	0.4788	1.0	0.86	2522.1	20.4	2495.6	10.5	2474.0	9.6	2474.0	9.6
-354-U1451-B3X-1W-13-28 Spot 283	422	577068	2.8	6.1579	0.5	9.3435	1.1	0.4175	1.0	0.88	2249.0	19.0	2372.3	10.5	2480.0	9.2	2480.0	9.2
-354-U1451-B3X-1W-13-28 Spot 192	342	417864	5.0	6.1576	0.4	11.0192	0.8	0.4923	0.7	0.89	2580.7	15.9	2524.8	7.8	2480.1	6.5	2480.1	6.5
-354-U1451-B3X-1W-13-28 Spot 308	766	445919	6.0	6.1383	0.5	8.6118	2.0	0.3836	2.0	0.97	2092.9	35.2	2297.8	18.4	2485.4	7.7	2485.4	7.7
-354-U1451-B3X-1W-13-28 Spot 182	135	283278	2.9	6.1212	0.7	9.7207	1.5	0.4317	1.3	0.86	2313.5	24.7	2408.7	13.5	2490.1	12.4	2490.1	12.4
-354-U1451-B3X-1W-13-28 Spot 197	791	184536	4.5	6.1118	0.4	8.8402	1.0	0.3920	0.9	0.90	2132.2	17.1	2321.6	9.5	2492.7	7.5	2492.7	7.5
-354-U1451-B3X-1W-13-28 Spot 273	128	712878	2.0	6.0985	0.5	10.1057	1.0	0.4472	0.9	0.85	2382.7	17.3	2444.5	9.4	2496.3	9.0	2496.3	9.0
-354-U1451-B3X-1W-13-28 Spot 90	185	718998	1.8	6.0803	0.6	10.5687	1.4	0.4663	1.2	0.90	2467.2	25.5	2486.0	12.8	2501.3	10.2	2501.3	10.2
-354-U1451-B3X-1W-13-28 Spot 100	1263	635701	14.2	5.8904	0.5	9.3508	0.8	0.3996	0.7	0.82	2167.4	12.7	2373.0	7.7	2554.6	8.1	2554.6	8.1
-354-U1451-B3X-1W-13-28 Spot 236	230	318766	3.2	5.6218	0.4	11.0411	2.4	0.4504	2.4	0.99	2396.9	47.1	2526.6	22.2	2632.5	6.7	2632.5	6.7
-354-U1451-B3X-1W-13-28 Spot 266	216	313672	3.6	5.4960	0.4	12.0695	1.0	0.4813	0.9	0.91	2533.0	19.4	2609.8	9.5	2670.0	6.9	2670.0	6.9
-354-U1451-B3X-1W-13-28 Spot 3	160	16567	7.7	5.3247	0.9	12.7793	1.7	0.4937	1.5	0.86	2586.8	31.8	2663.5	16.3	2722.3	14.4	2722.3	14.4
-354-U1451-B3X-1W-13-28 Spot 86	112	144666	1.9	5.3182	0.5	12.9267	0.9	0.4988	0.8	0.84	2608.7	16.7	2674.3	8.7	2724.3	8.1	2724.3	8.1
-354-U1451-B3X-1W-13-28 Spot 64	468	1367715	1.7	5.2346	0.5	12.4421	1.3	0.4726	1.2	0.92	2494.8	24.8	2638.4	12.2	2750.4	8.3	2750.4	8.3
-354-U1451-B3X-1W-13-28 Spot 247	75	252134	2.0	5.0123	0.6	15.4233	1.3	0.5609	1.1	0.89	2870.4	26.3	2841.8	12.2	2821.5	9.6	2821.5	9.6
-354-U1451-B3X-1W-13-28 Spot 141	142	223875	1.8	4.9654	0.4	14.7216	0.9	0.5304	0.8	0.88	2743.1	18.6	2797.4	9.0	2836.8	7.2	2836.8	7.2
-354-U1451-B3X-1W-13-28 Spot 42	399	721486	7.2	3.6235	0.4	24.2595	1.0	0.6378	0.9	0.90	3180.5	23.2	3278.9	10.0	3339.6	6.9	3339.6	6.9

Table ______. U-Pb geochronologic analyses.																		
#22 U1451B_22R							Isotope ratios						Apparent ages (Ma)					
Late Miocene 11.5 Ma																		
Analysis	U (ppm)	206Pb/204Pb	U/Th	206Pb*/207Pb*	± (%)	207Pb*/235U*	± (%)	206Pb*/238U	± (%)	error corr.	206Pb*/238U*	± (Ma)	207Pb*/235U	± (Ma)	206Pb*/207Pb*	± (Ma)	Best age (Ma)	± (Ma)
-354-U1451B-22R-I-W Spot 134	972	13822	0.4	13.7363	4.4	0.0279	4.6	0.0028	1.3	0.29	17.9	0.2	28.0	1.3	1007.5	89.1	17.9	0.2
-354-U1451B-22R-I-W Spot 236	52	1152	237.7	30.9258	15.4	0.0185	15.5	0.0041	1.9	0.12	26.7	0.5	18.6	2.9	NA	NA	26.7	0.5
-354-U1451B-22R-I-W Spot 37	65	9566	97.2	19.4314	4.5	0.0354	4.7	0.0050	1.3	0.28	32.1	0.4	35.3	1.6	260.6	103.5	32.1	0.4
-354-U1451B-22R-I-W Spot 253	484	12490	1.2	21.4358	1.5	0.0373	1.9	0.0058	1.1	0.57	37.3	0.4	37.2	0.7	30.4	37.0	37.3	0.4
-354-U1451B-22R-I-W Spot 141	724	45128	0.8	21.1295	1.2	0.0459	1.5	0.0070	0.9	0.61	45.2	0.4	45.6	0.7	64.7	27.6	45.2	0.4
-354-U1451B-22R-I-W Spot 248	515	46373	17.7	22.0007	1.4	0.0452	1.8	0.0072	1.2	0.66	46.3	0.5	44.9	0.8	NA	NA	46.3	0.5
-354-U1451B-22R-I-W Spot 188	821	96202	0.9	21.2785	1.2	0.0473	1.5	0.0073	0.9	0.60	46.9	0.4	46.9	0.7	48.0	29.2	46.9	0.4
-354-U1451B-22R-I-W Spot 93	143	2612	7.0	4.7939	1.4	0.2189	1.8	0.0076	1.2	0.64	48.9	0.6	201.0	3.3	2894.0	22.8	48.9	0.6
-354-U1451B-22R-I-W Spot 121	394	10305	1.9	21.6131	1.8	0.0489	2.1	0.0077	1.1	0.53	49.2	0.6	48.5	1.0	10.5	43.6	49.2	0.6
-354-U1451B-22R-I-W Spot 136	182	23680	1.1	20.4727	2.5	0.0519	2.7	0.0077	1.2	0.44	49.5	0.6	51.3	1.4	139.4	57.7	49.5	0.6
-354-U1451B-22R-I-W Spot 205	195	19283	1.4	20.9605	2.1	0.0516	2.3	0.0078	0.9	0.42	50.3	0.5	51.0	1.1	83.8	48.9	50.3	0.5
-354-U1451B-22R-I-W Spot 148	1380	156777	0.6	20.7649	1.0	0.0549	1.3	0.0083	0.8	0.63	53.1	0.4	54.3	0.7	106.0	23.0	53.1	0.4
-354-U1451B-22R-I-W Spot 80	172	26218	0.8	15.6721	1.8	0.0793	2.1	0.0090	1.1	0.50	57.9	0.6	77.5	1.6	734.4	39.0	57.9	0.6
-354-U1451B-22R-I-W Spot 252	197	10102	1.0	20.9857	2.1	0.0600	2.5	0.0091	1.3	0.53	58.7	0.8	59.2	1.4	80.9	50.2	58.7	0.8
-354-U1451B-22R-I-W Spot 274	328	12036	1.2	20.9641	1.7	0.0645	2.1	0.0098	1.2	0.57	62.9	0.8	63.5	1.3	83.4	41.0	62.9	0.8
-354-U1451B-22R-I-W Spot 216	192	14062	1.0	20.5916	1.9	0.0667	2.1	0.0100	1.0	0.47	64.0	0.6	65.6	1.4	125.8	44.3	64.0	0.6
-354-U1451B-22R-I-W Spot 278	207	239821	1.3	20.3668	1.6	0.0734	1.8	0.0108	0.9	0.50	69.6	0.6	71.9	1.3	151.6	36.6	69.6	0.6
-354-U1451B-22R-I-W Spot 241	782	39614	1.1	21.2154	1.1	0.0709	1.5	0.0109	1.0	0.66	70.0	0.7	69.6	1.0	55.1	26.6	70.0	0.7
-354-U1451B-22R-I-W Spot 311	182	25349	2.1	20.7746	1.3	0.0731	1.5	0.0110	0.7	0.49	70.7	0.5	71.7	1.1	104.9	31.5	70.7	0.5
-354-U1451B-22R-I-W Spot 179	97	6328	1.3	19.9414	2.6	0.0845	2.7	0.0122	0.6	0.23	78.3	0.5	82.3	2.1	200.8	59.9	78.3	0.5
-354-U1451B-22R-I-W Spot 14	75	6928	1.3	21.2322	2.9	0.0804	3.1	0.0124	1.0	0.32	79.3	0.8	78.5	2.3	53.2	69.9	79.3	0.8
-354-U1451B-22R-I-W Spot 312	85	5597	1.0	22.3532	2.4	0.0819	2.6	0.0133	1.2	0.45	85.0	1.0	79.9	2.0	NA	NA	85.0	1.0
-354-U1451B-22R-I-W Spot 275	260	17107	1.6	21.0727	1.3	0.0909	1.7	0.0139	1.0	0.63	89.0	0.9	88.3	1.4	71.2	30.6	89.0	0.9
-354-U1451B-22R-I-W Spot 163	128	34661	2.2	21.2578	1.8	0.0996	2.0	0.0154	0.9	0.44	98.3	0.9	96.4	1.8	50.3	42.7	98.3	0.9
-354-U1451B-22R-I-W Spot 230	1078	861119	2.5	20.5834	1.0	0.1087	1.4	0.0162	1.0	0.71	103.8	1.0	104.8	1.4	126.7	22.7	103.8	1.0
-354-U1451B-22R-I-W Spot 132	360	109983	1.6	20.7168	1.0	0.1083	1.4	0.0163	0.9	0.67	104.1	0.9	104.4	1.4	111.5	24.2	104.1	0.9
-354-U1451B-22R-I-W Spot 125	1178	497618	2.1	20.3291	0.9	0.1112	1.3	0.0164	1.0	0.76	104.9	1.0	107.0	1.3	155.9	19.9	104.9	1.0
-354-U1451B-22R-I-W Spot 164	99	1201	1.4	3.3218	1.0	0.7380	1.5	0.0178	1.1	0.75	113.7	1.3	561.2	6.5	3474.9	15.5	113.7	1.3
-354-U1451B-22R-I-W Spot 261	369	242834	1.0	19.8590	1.2	0.1249	1.7	0.0180	1.1	0.66	115.0	1.2	119.5	1.9	210.4	28.9	115.0	1.2
-354-U1451B-22R-I-W Spot 151	182	14479	1.5	20.6663	2.1	0.1268	2.4	0.0190	1.1	0.45	121.4	1.3	121.2	2.7	117.3	49.9	121.4	1.3
-354-U1451B-22R-I-W Spot 83	34	181723	1.9	21.0221	3.7	0.1389	3.8	0.0212	0.9	0.23	135.1	1.2	132.0	4.7	76.8	87.1	135.1	1.2
-354-U1451B-22R-I-W Spot 249	281	114444	1.5	20.4848	1.3	0.1647	1.7	0.0245	1.1	0.66	155.9	1.7	154.8	2.4	138.0	29.6	155.9	1.7
-354-U1451B-22R-I-W Spot 251	413	34595	0.8	20.4200	0.8	0.1691	1.3	0.0250	1.0	0.77	159.5	1.6	158.6	1.9	145.5	19.5	159.5	1.6
-354-U1451B-22R-I-W Spot 45	188	31188	1.5	19.3740	1.1	0.2991	1.4	0.0420	0.9	0.63	265.5	2.3	265.7	3.2	267.4	24.6	265.5	2.3
-354-U1451B-22R-I-W Spot 313	1648	315632	2.5	19.0972	0.7	0.3193	1.1	0.0442	0.8	0.73	279.0	2.1	281.3	2.6	300.4	16.7	279.0	2.1
-354-U1451B-22R-I-W Spot 290	458	165129	1.9	18.9105	0.6	0.3263	1.2	0.0448	1.0	0.85	282.4	2.8	286.8	3.0	322.7	14.5	282.4	2.8
-354-U1451B-22R-I-W Spot 157	243	34331	3.8	19.1650	0.9	0.3609	1.2	0.0502	0.9	0.70	315.7	2.6	312.9	3.3	292.2	19.7	315.7	2.6
-354-U1451B-22R-I-W Spot 161	121	267436	0.5	15.3199	3.0	0.5040	3.5	0.0560	1.8	0.50	351.4	6.0	414.4	11.9	782.3	63.4	351.4	6.0
-354-U1451B-22R-I-W Spot 19	603	435913	1.6	17.7501	0.6	0.5359	1.1	0.0690	0.9	0.82	430.2	3.7	435.7	3.9	464.8	14.0	430.2	3.7
-354-U1451B-22R-I-W Spot 239	383	167850	1.6	18.1327	0.8	0.5281	1.3	0.0695	1.1	0.80	433.0	4.4	430.6	4.6	417.3	17.5	433.0	4.4
-354-U1451B-22R-I-W Spot 260	1518	189791	3.9	17.6809	0.7	0.5584	1.4	0.0716	1.2	0.86	446.0	5.1	450.5	5.0	473.4	15.2	446.0	5.1
-354-U1451B-22R-I-W Spot 118	359	600541	1.6	17.4391	0.6	0.5974	1.1	0.0756	0.9	0.83	469.8	4.2	475.6	4.3	503.8	13.6	469.8	4.2
-354-U1451B-22R-I-W Spot 98	1757	846630	24.5	17.8277	0.7	0.5868	1.1	0.0759	0.9	0.79	471.6	3.9	468.8	4.1	455.0	15.0	471.6	3.9
-354-U1451B-22R-I-W Spot 299	233	144214	2.8	16.8777	0.7	0.6221	1.3	0.0762	1.1	0.85	473.3	5.2	491.2	5.2	575.3	15.2	473.3	5.2
-354-U1451B-22R-I-W Spot 24	153	42209	0.4	17.6695	0.8	0.6217	1.2	0.0797	0.9	0.75	494.4	4.2	490.9	4.6	474.8	17.3	494.4	4.2
-354-U1451B-22R-I-W Spot 88	312	554976	2.5	17.5574	0.8	0.6297	1.3	0.0802	1.0	0.79	497.5	4.9	495.9	5.1	488.8	17.4	497.5	4.9
-354-U1451B-22R-I-W Spot 270	112	50182	0.6	17.5678	0.7	0.6295	1.0	0.0802	0.8	0.76	497.6	3.7	495.8	4.0	487.6	14.5	497.6	3.7
-354-U1451B-22R-I-W Spot 29	116	95375	0.8	17.5842	0.6	0.6402	1.1	0.0817	0.9	0.84	506.1	4.3	502.4	4.2	485.5	12.7	506.1	4.3
-354-U1451B-22R-I-W Spot 237	72	28869	0.7	17.3197	1.1	0.6590	1.4	0.0828	0.9	0.65	512.9	4.6	514.0	5.8	518.8	24.2	512.9	4.6
-354-U1451B-22R-I-W Spot 158	273	350802	1.1	16.9500	0.9	0.6735	1.6	0.0828	1.3	0.83	513.0	6.4	522.9	6.3	566.1	18.8	513.0	6.4
-354-U1451B-22R-I-W Spot 108	890	161964	1.0	16.9480	0.7	0.6882	1.2	0.0846	1.0	0.83	523.7	4.9	531.7	4.9	566.3	14.4	523.7	4.9
-354-U1451B-22R-I-W Spot 292	112	157900	1.5	16.7282	1.2	0.7062	1.5	0.0857	0.9	0.59	530.2	4.5	542.5	6.3	594.6	26.0	530.2	4.5
-354-U1451B-22R-I-W Spot 232	77	165037	1.2	16.7376	1.2	0.7084	1.4	0.0860	0.8	0.56	532.0	4.1	543.8	6.0	593.4	25.5	532.0	4.1
-354-U1451B-22R-I-W Spot 218	750	5112333	2.0	17.2680	0.7	0.6916	1.1	0.0867	0.8	0.74	535.7	4.2	533.8	4.6	525.5	16.3	535.7	4.2
-354-U1451B-22R-I-W Spot 5	146	75673	0.6	17.0376	0.9	0.7045	1.4	0.0871	1.1	0.77	538.3	5.6	541.4	5.9	554.8	19.7	538.3	5.6
-354-U1451B-22R-I-W Spot 211	239	255654	0.8	17.2333	0.8	0.6969	1.5	0.0871	1.3	0.86	538.6	6.8	536.9	6.3	529.8	16.8	538.6	6.8
-354-U1451B-22R-I-W Spot 72	470	432637	3.1	17.0300	0.9	0.7206	1.3	0.0890	1.0	0.72	549.9	5.1	551.0	5.7	555.8	20.2	549.9	5.1
-354-U1451B-22R-I-W Spot 281	304	1645308	0.9	16.4609	0.8	0.7996	1.2	0.0955	1.0	0.77	588.0	5.4	596.6	5.6	629.5	17.1	588.0	5.4
-354-U1451B-22R-I-W Spot 257	511	11267837	4.8	16.2688	0.7	0.8119	1.6	0.0958	1.4	0.91	590.0	8.1	603.5	7.2	654.7	14.5	590.0	8.1
-354-U1451B-22R-I-W Spot 21	335	158163	3.0	16.6710	0.7	0.8020	1.1	0.0970	0.9	0.77	596.9	4.9	598.0	5.0	602.0	15.1	596.9	4.9
-354-U1451B-22R-I-W Spot 174	75	1905608	1.1	16.3913	0.9	0.8158	1.2	0.0970	0.8	0.66	597.0	4.7	605.7	5.7	638.6	20.0	597.0	4.7
-354-U1451B-22R-I-W Spot 25	1056	199883	5.9	16.0981	0.7	0.8322	1.4	0.0972	1.2	0.86	598.0	6.8	614.8	6.4	677.3	15.1	598.0	6.8
-354-U1451B-22R-I-W Spot 155	490	1393115	1.8	16.4799	0.7	0.8318	1.3	0.0995	1.1	0.84	611.3	6.5	614.6	6.2	626.9	15.8	611.3	6.5
-354-U1451B-22R-I-W Spot 307	1044	2119133	2.2	16.4906	0.5	0.8520	1.2	0.1019	1.0	0.88	625.8	6.1	625.7	5.4	625.6	11.6	625.8	6.1
-354-U1451B-22R-I-W Spot 68	338	123271	5.2	15.4125	1.5	0.9270	2.2	0.1037	1.5	0.71	635.8	9.4	666.0	10.6	769.6	32.0	635.8	9.4
-354-U1451B-22R-I-W Spot 243	227	335422	4.4	16.5012	0.8	0.8862	1.5	0.1061	1.2	0.84	650.1	7.6	644.3	6.9	624.2	16.8	650.1	7.6
-354-U1451B-22R-I-W Spot 156	277	142404	1.5	16.1610	0.7	0.9094	1.1	0.1066	0.9	0.82	653.2	5.9	656.8	5.5	668.9	14.0	653.2	5.9
-354-U1451B-22R-I-W Spot 181	922	5889109	4.6	16.1389	0.8	0.9324	1.1	0.1092	0.7	0.70	668.0	4.7	668.9	5.2	671.9	16.2	668.0	4.7
-354-U1451B-22R-I-W Spot 305	67	62184	2.5	15.7903	0.9	0.9811	1.2	0.1124	0.8	0.66	686.7	5.3	694.2	6.2	718.4	19.6	686.7	5.3
-354-U1451B-22R-I-W Spot 36	77	65067	0.6	15.2926	1.2	1.0435	2.2	0.1158	1.8	0.84	706.3	12.3	725.7	11.3	786.0	24.7	706.3	12.3
-354-U1451B-22R-I-W Spot 289	616	3733613	1.2	15.1954	0.7	1.0725	1.5	0.1182	1.3	0.89	720.4	9.1	739.9	7.9	799.4	14.6	720.4	9.1
-354-U1451B-22R-I-W Spot 135	3112	968685	3.7	15.3065	1.1	1.0665	3.7	0.1184	3.5	0.96	721.6	24.0	737.0	19.3	784.1	22.7	721.6	24.0
-354-U1451B-22R-I-W Spot 41	1475	255312	0.5	15.0077	0.7	1.0957	1.5	0.1193	1.3	0.88	726.6	8.9	751.3	7.8	825.4	14.7	726.6	8.9
-354-U1451B-22R-I-W Spot 116	178	181963	1.2	15.3044	0.9	1.0848	1.2	0.1205	0.7	0.64	733.2	5.2	746.0	6.2	784.4	18.8	733.2	5.2
-354-U1451B-22R-I-W Spot 69	253	155057	0.8	15.4400	0.7	1.1234	0.9	0.1259	0.6	0.64	764.2	4.1	764.6	4.8	765.8	14.4	764.2	4.1
-354-U1451B-22R-I-W Spot 183	157	201176	2.1	15.1661	0.7	1.1692	1.5	0.1287	1.3	0.87	780.2	9.4	786.3	8.1	803.4	15.2	780.2	9.4
-354-U1451B-22R-I-W Spot 245	331	325812	1.0	15.3074	0.7	1.1610	1.3	0.1290	1.1	0.85	781.9	7.9	782.4	6.9	784.0	13.8	781.9	7.9
-354-U1451B-22R-I-W Spot 101	1601	1061310	26.3	15.1902	0.6	1.1776	1.6	0.1298	1.5	0.94	786.7	11.3	790.2	8.9	800.1	11.7	786.7	11.3
-354-U1451B-22R-I-W Spot 224	37	34209	1.6	15.4573	1.1	1.1586	1.5	0.1299	1.0	0.70	787.5	7.7	781.3	8.1	763.5	22.5	787.5	7.7
-354-U1451B-22R-I-W Spot 300	699	708635	4.9	15.2198	0.6	1.1832	1.8	0.1307	1.7	0.95	791.7	12.7	792.8	9.9	796.0	11.8	791.7	12.7
-354-U1451B-22R-I-W Spot 55	89	44304	1.1	14.7592	0.9	1.2308	1.4	0.1318	1.1	0.77	798.1	7.9	814.7	7.7	860.1	18.3	798.1	7.9
-354-U1451B-22R-I-W Spot 206	55	90180	1.9	15.3781	0.9	1.1826	1.3	0.1320	0.9	0.72	799.0	7.1	792.5	7.2	774.3	19.1	799.0	7.1
-354-U1451B-22R-I-W Spot 195	925	472308	3.5	15.0559	0.6	1.2225	1.0	0.1335	0.8	0.83	808.1	6.4	810.9	5.7	818.7	11.9	808.1	6.4
-354-U1451B-22R-I-W Spot 97	193	78632	0.9	14.1139	1.0	1.3103	1.4	0.1342	1.0	0.69	811.7	7.4	850.3	8.1	952.3	20.8	811.7	7.4
-354-U1451B-22R-I-W Spot 0	225	361114	1.1	15.0907	0.8	1.2284	1.1	0.1345	0.8	0.67	813.5	5.7	813.6	6.3	813.9	17.3	813.5	5.7
-354-U1451B-22R-I-W Spot 9	231	195799	0.7	15.2524	0.7	1.2166	1.2	0.1346	1.0	0.84	814.3	8.0	808.2	6.9	791.5	14.3	814.3	8.0
-354-U1451B-22R-I-W Spot 122	990	893031	2.8	15.0184	0.7	1.2412	1.1	0.1353	0.9	0.76	817.8	6.7	819.4	6.4	823.9	15.3	817.8	6.7
-354-U1451B-22R-I-W Spot 113	248	100453	1.5	14.9244	0.9	1.2494	1.4	0.1353	1.1	0.77	818.0	8.2	823.1	7.8	837.0	18.2	818.0	8.2
-354-U1451B-22R-I-W Spot 250	354	502295	4.3	14.4081	0.7	1.2967	1.2	0.1356	1.0	0.84	819.5	7.8	844.3	7.0	909.9	13.7	819.5	7.8
-354-U1451B-22R-I-W Spot 124	106	70303	2.6	14.9534	1.2	1.2516	1.6	0.1358	1.0	0.67	820.9	8.0	824.1	8.8	833.0	24.2	820.9	8.0
-354-U1451B-22R-I-W Spot 103	107	128324	1.4	14.8817	0.9	1.2582	1.2	0.1359	0.8	0.67	821.2	6.3	827.1	6.9	842.9	18.8	821.2	6.3
-354-U1451B-22R-I-W Spot 215	233	111171	1.2	14.6821	0.7	1.2760	1.2	0.1359	1.0	0.83	821.6	7.9	835.1	7.0	871.0	14.0	821.6	7.9
-354-U1451B-22R-I-W Spot 283	378	188267	6.2	14.7078	0.6	1.2775	1.3	0.1363	1.1	0.87	823.9	8.7	835.8	7.3	867.4	13.0	823.9	8.7
-354-U1451B-22R-I-W Spot 165	153	166515	1.8	14.7624	0.7	1.2794	1.1	0.1370	0.9	0.81	827.9	7.2	836.6	6.5	859.7	13.7	827.9	7.2

-354-U1451B-22R-I-W Spot 276	440	290112	2.4	14.5347	0.5	1.3040	1.1	0.1375	1.0	0.90	830.6	7.9	847.5	6.5	891.9	10.3	830.6	7.9
-354-U1451B-22R-I-W Spot 268	968	406439	3.2	14.0717	0.7	1.3524	1.2	0.1381	1.0	0.84	833.8	8.1	868.6	7.2	958.4	13.7	833.8	8.1
-354-U1451B-22R-I-W Spot 43	430	369225	1.7	14.7140	0.7	1.3001	1.2	0.1388	1.0	0.82	837.9	7.8	845.8	6.9	866.5	14.2	837.9	7.8
-354-U1451B-22R-I-W Spot 187	77	109575	1.4	14.9426	0.6	1.2841	1.0	0.1392	0.8	0.79	840.2	6.1	838.7	5.6	834.5	12.6	840.2	6.1
-354-U1451B-22R-I-W Spot 175	1052	597460	1.6	14.8050	0.7	1.3001	1.1	0.1397	0.9	0.81	842.8	7.3	845.8	6.5	853.7	13.8	842.8	7.3
-354-U1451B-22R-I-W Spot 199	458	443947	18.1	14.8474	0.6	1.2969	1.1	0.1397	1.0	0.83	843.1	7.5	844.4	6.6	847.7	13.3	843.1	7.5
-354-U1451B-22R-I-W Spot 56	1518	1113677	2.4	14.6133	0.6	1.3258	1.1	0.1406	0.8	0.79	848.0	6.6	857.1	6.1	880.7	13.4	848.0	6.6
-354-U1451B-22R-I-W Spot 7	274	388914	1.4	14.5910	0.6	1.3291	2.1	0.1407	2.0	0.95	848.7	15.5	858.5	11.9	883.9	13.3	848.7	15.5
-354-U1451B-22R-I-W Spot 4	59	172301	1.3	15.0018	1.0	1.3037	1.4	0.1419	1.0	0.70	855.5	7.8	847.4	7.9	826.2	20.6	855.5	7.8
-354-U1451B-22R-I-W Spot 190	606	380261	2.0	14.5960	0.6	1.3439	1.2	0.1423	1.0	0.86	857.8	8.1	864.9	6.8	883.2	12.3	857.8	8.1
-354-U1451B-22R-I-W Spot 225	611	910097	27.5	14.4567	0.7	1.3572	1.4	0.1424	1.2	0.87	858.0	9.6	870.7	8.0	903.0	13.8	858.0	9.6
-354-U1451B-22R-I-W Spot 231	266	183111	3.2	14.7632	0.7	1.3373	1.4	0.1433	1.2	0.85	863.0	9.5	862.1	8.1	859.6	15.1	863.0	9.5
-354-U1451B-22R-I-W Spot 284	1045	623883	6.1	14.5529	0.7	1.3739	1.2	0.1451	1.0	0.80	873.3	8.1	877.8	7.3	889.3	15.4	873.3	8.1
-354-U1451B-22R-I-W Spot 119	513	495860	1.3	14.5644	0.7	1.3731	1.3	0.1451	1.2	0.87	873.5	9.5	877.5	7.8	887.7	13.5	873.5	9.5
-354-U1451B-22R-I-W Spot 31	241	884240	1.2	14.7352	0.7	1.3605	1.3	0.1455	1.1	0.85	875.5	9.1	872.1	7.6	863.5	14.2	875.5	9.1
-354-U1451B-22R-I-W Spot 176	193	108780	3.3	14.4840	0.7	1.3843	1.3	0.1455	1.1	0.86	875.6	9.1	882.3	7.6	899.1	13.5	875.6	9.1
-354-U1451B-22R-I-W Spot 48	137	50348	0.6	14.6942	0.8	1.3750	1.3	0.1466	1.1	0.79	881.9	8.8	878.3	7.9	869.3	17.0	881.9	8.8
-354-U1451B-22R-I-W Spot 171	716	458905	1.9	14.4702	0.8	1.4005	1.3	0.1470	0.9	0.75	884.4	7.8	889.2	7.5	901.0	17.4	884.4	7.8
-354-U1451B-22R-I-W Spot 197	291	4352141	1.2	14.7230	0.7	1.3773	1.3	0.1471	1.1	0.82	884.9	8.8	879.3	7.7	865.3	15.4	884.9	8.8
-354-U1451B-22R-I-W Spot 193	177	764459	1.4	14.4751	0.7	1.4689	1.3	0.1543	1.1	0.86	924.9	9.9	917.7	8.0	900.3	13.9	900.3	13.9
-354-U1451B-22R-I-W Spot 160	963	992331	5.8	14.3141	0.6	1.4126	2.0	0.1467	1.9	0.96	882.5	15.5	894.2	11.7	923.4	11.8	923.4	11.8
-354-U1451B-22R-I-W Spot 85	404	1582037	1.8	14.2566	0.7	1.4671	1.2	0.1518	1.0	0.82	910.8	8.4	916.9	7.3	931.6	14.0	931.6	14.0
-354-U1451B-22R-I-W Spot 54	318	14932388	1.3	14.2485	0.6	1.5336	1.2	0.1585	1.0	0.87	948.7	9.1	943.9	7.4	932.8	12.3	932.8	12.3
-354-U1451B-22R-I-W Spot 107	39	67888	2.0	14.2405	1.0	1.5239	1.5	0.1575	1.1	0.75	942.6	10.0	940.0	9.4	934.0	20.8	934.0	20.8
-354-U1451B-22R-I-W Spot 70	194	92201	1.9	14.2389	0.7	1.5001	2.1	0.1550	2.0	0.94	928.8	17.4	930.4	13.0	934.2	14.4	934.2	14.4
-354-U1451B-22R-I-W Spot 209	235	394873	2.8	14.2165	0.7	1.4520	1.1	0.1498	0.8	0.75	899.7	6.6	910.7	6.4	937.4	14.5	937.4	14.5
-354-U1451B-22R-I-W Spot 309	1739	186790	42.1	14.2101	0.5	1.4326	1.0	0.1477	0.9	0.85	888.1	7.1	902.6	6.1	938.4	11.1	938.4	11.1
-354-U1451B-22R-I-W Spot 59	148	203510	0.5	14.1900	0.7	1.5203	1.1	0.1565	0.9	0.76	937.5	7.5	938.6	7.0	941.3	15.3	941.3	15.3
-354-U1451B-22R-I-W Spot 308	1495	2319822	36.3	14.1808	0.7	1.4026	1.5	0.1443	1.3	0.89	869.1	10.9	890.1	8.9	942.6	13.7	942.6	13.7
-354-U1451B-22R-I-W Spot 234	86	135208	1.0	14.1660	0.8	1.5308	1.9	0.1573	1.7	0.90	942.0	14.8	942.8	11.6	944.7	17.0	944.7	17.0
-354-U1451B-22R-I-W Spot 166	1110	514466	23.5	14.1212	0.8	1.5062	1.2	0.1543	0.9	0.75	925.2	7.9	932.9	7.4	951.2	16.4	951.2	16.4
-354-U1451B-22R-I-W Spot 306	760	1344037	7.1	14.1002	0.7	1.5469	1.2	0.1583	1.0	0.83	947.1	9.1	949.3	7.7	954.2	14.3	954.2	14.3
-354-U1451B-22R-I-W Spot 47	885	1482277	4.8	14.0992	0.6	1.5534	1.2	0.1589	1.0	0.84	950.7	8.5	951.8	7.1	954.4	12.9	954.4	12.9
-354-U1451B-22R-I-W Spot 266	153	909364	1.4	14.0934	0.7	1.5541	1.1	0.1589	0.9	0.80	950.8	8.1	952.1	7.1	955.2	13.9	955.2	13.9
-354-U1451B-22R-I-W Spot 81	221	72848	1.8	14.0926	0.7	1.5467	1.1	0.1582	0.9	0.80	946.5	8.0	949.2	7.0	955.3	14.0	955.3	14.0
-354-U1451B-22R-I-W Spot 170	426	927855	1.9	14.0874	0.6	1.5936	1.1	0.1629	0.9	0.84	972.8	8.1	967.7	6.6	956.1	11.6	956.1	11.6
-354-U1451B-22R-I-W Spot 282	526	947811	2.8	14.0687	0.8	1.5853	1.4	0.1618	1.1	0.81	966.9	10.1	964.4	8.6	958.8	16.4	958.8	16.4
-354-U1451B-22R-I-W Spot 145	281	186069	2.7	14.0569	1.4	1.6226	2.6	0.1655	2.2	0.84	987.3	20.0	979.0	16.3	960.5	28.3	960.5	28.3
-354-U1451B-22R-I-W Spot 150	1347	348719	1.4	14.0410	0.6	1.5305	1.4	0.1559	1.2	0.90	934.1	10.5	942.7	8.3	962.9	12.1	962.9	12.1
-354-U1451B-22R-I-W Spot 273	430	10640409	1.4	14.0341	0.7	1.6656	1.3	0.1696	1.1	0.86	1009.9	10.3	995.5	8.1	963.9	13.5	963.9	13.5
-354-U1451B-22R-I-W Spot 86	233	293739	0.8	14.0331	0.6	1.5719	1.2	0.1601	1.0	0.84	957.1	9.1	959.2	7.5	964.0	13.3	964.0	13.3
-354-U1451B-22R-I-W Spot 213	254	422073	1.5	14.0210	0.6	1.5717	1.1	0.1599	0.9	0.85	956.2	8.1	959.1	6.6	965.8	11.4	965.8	11.4
-354-U1451B-22R-I-W Spot 154	441	260802	2.2	14.0061	0.5	1.5963	1.3	0.1622	1.2	0.92	969.2	10.6	968.8	8.0	967.9	10.0	967.9	10.0
-354-U1451B-22R-I-W Spot 303	292	2110462	1.5	13.9943	0.6	1.6300	1.2	0.1655	1.1	0.86	987.3	9.7	981.9	7.8	969.6	13.0	969.6	13.0
-354-U1451B-22R-I-W Spot 100	1395	1431850	4.2	13.9839	0.5	1.4763	1.2	0.1498	1.1	0.90	899.8	9.2	920.7	7.4	971.2	10.7	971.2	10.7
-354-U1451B-22R-I-W Spot 11	285	135861	2.7	13.9820	1.3	1.3830	1.8	0.1403	1.2	0.69	846.4	9.6	881.7	10.4	971.5	26.1	971.5	26.1
-354-U1451B-22R-I-W Spot 258	745	965855	9.6	13.9795	0.5	1.5114	1.0	0.1533	0.9	0.89	919.4	7.7	935.0	6.2	971.8	9.2	971.8	9.2
-354-U1451B-22R-I-W Spot 152	189	1212935	1.1	13.9688	0.6	1.5179	1.0	0.1538	0.7	0.76	922.5	6.4	937.6	6.0	973.4	13.1	973.4	13.1
-354-U1451B-22R-I-W Spot 94	86	119269	0.4	13.9357	0.7	1.6451	1.1	0.1663	0.9	0.79	992.0	8.2	987.7	7.2	978.2	14.3	978.2	14.3
-354-U1451B-22R-I-W Spot 131	524	377940	1.7	13.9252	0.8	1.6448	1.2	0.1662	1.0	0.80	991.1	9.1	987.6	7.9	979.8	15.4	979.8	15.4
-354-U1451B-22R-I-W Spot 40	1209	866853	3.3	13.9249	0.7	1.6334	1.4	0.1650	1.3	0.88	984.7	11.4	983.2	8.9	979.8	13.6	979.8	13.6
-354-U1451B-22R-I-W Spot 23	214	656308	0.9	13.8975	0.5	1.5975	0.8	0.1611	0.6	0.79	962.8	5.7	969.2	5.1	983.8	10.2	983.8	10.2
-354-U1451B-22R-I-W Spot 114	1015	585711	11.8	13.8934	0.8	1.6737	1.2	0.1687	0.9	0.76	1005.1	8.5	998.6	7.6	984.4	15.8	984.4	15.8
-354-U1451B-22R-I-W Spot 104	487	357064	3.1	13.8870	0.6	1.5862	1.2	0.1598	1.1	0.86	955.8	9.4	964.8	7.7	985.3	12.9	985.3	12.9
-354-U1451B-22R-I-W Spot 38	323	6000518	1.4	13.8236	0.7	1.6617	1.3	0.1667	1.1	0.83	993.7	9.7	994.0	8.1	994.6	14.6	994.6	14.6
-354-U1451B-22R-I-W Spot 220	633	169206	2.5	13.8175	0.6	1.5572	0.9	0.1561	0.7	0.77	935.2	6.3	953.3	5.8	995.5	12.1	995.5	12.1
-354-U1451B-22R-I-W Spot 111	624	1409011	1.9	13.7863	0.7	1.6559	1.3	0.1656	1.1	0.86	988.0	10.2	991.8	8.2	1000.1	13.5	1000.1	13.5
-354-U1451B-22R-I-W Spot 8	1136	804472	2.1	13.7777	0.7	1.6249	1.3	0.1624	1.2	0.87	970.3	10.6	979.9	8.5	1001.4	13.5	1001.4	13.5
-354-U1451B-22R-I-W Spot 62	308	168870	1.4	13.7273	0.7	1.6230	1.1	0.1617	0.9	0.77	966.0	7.7	979.1	7.0	1008.8	14.4	1008.8	14.4
-354-U1451B-22R-I-W Spot 233	1906	1100731	23.0	13.7263	0.6	1.6173	1.0	0.1611	0.8	0.78	962.7	6.9	976.9	6.2	1009.0	12.5	1009.0	12.5
-354-U1451B-22R-I-W Spot 46	805	703548	15.4	13.7241	1.4	1.5365	1.7	0.1530	1.1	0.63	917.8	9.4	945.1	10.7	1009.3	27.4	1009.3	27.4
-354-U1451B-22R-I-W Spot 159	251	93052	0.6	13.7039	1.2	1.6003	2.1	0.1591	1.7	0.83	951.9	15.2	970.3	13.0	1012.3	23.7	1012.3	23.7
-354-U1451B-22R-I-W Spot 262	274	357891	2.1	13.6881	0.7	1.6632	1.6	0.1652	1.5	0.91	985.5	13.6	994.6	10.4	1014.6	13.8	1014.6	13.8
-354-U1451B-22R-I-W Spot 192	677	3229716	5.2	13.6734	0.7	1.6365	1.2	0.1624	1.0	0.83	969.9	8.9	984.4	7.6	1016.8	13.7	1016.8	13.7
-354-U1451B-22R-I-W Spot 42	956	1391445	0.8	13.6389	0.6	1.7717	1.1	0.1753	0.9	0.83	1041.4	8.5	1035.1	6.9	1021.9	11.9	1021.9	11.9
-354-U1451B-22R-I-W Spot 169	567	2291209	1.4	13.6266	0.6	1.7269	1.1	0.1707	0.9	0.84	1016.2	8.7	1018.6	7.1	1023.7	12.0	1023.7	12.0
-354-U1451B-22R-I-W Spot 1	130	51094	3.0	13.5734	0.8	1.7774	1.3	0.1750	1.0	0.80	1039.9	9.9	1037.2	8.4	1031.6	15.7	1031.6	15.7
-354-U1451B-22R-I-W Spot 168	425	6612828	3.4	13.5589	0.7	1.8055	1.1	0.1776	0.9	0.77	1054.0	8.6	1047.4	7.5	1033.8	14.8	1033.8	14.8
-354-U1451B-22R-I-W Spot 143	388	1806978	2.2	13.5454	0.7	1.6719	1.1	0.1643	0.8	0.77	980.8	7.3	997.9	6.7	1035.8	13.7	1035.8	13.7
-354-U1451B-22R-I-W Spot 44	190	4792662	1.2	13.4967	0.7	1.7847	1.2	0.1748	1.0	0.82	1038.4	9.6	1039.9	7.9	1043.1	14.2	1043.1	14.2
-354-U1451B-22R-I-W Spot 73	554	582931	4.7	13.4640	0.8	1.7393	1.2	0.1699	0.9	0.74	1011.7	8.5	1023.2	7.9	1048.0	16.6	1048.0	16.6
-354-U1451B-22R-I-W Spot 267	441	300917	8.1	13.4475	0.6	1.8370	1.2	0.1792	1.1	0.88	1062.8	10.6	1058.8	8.1	1050.5	11.7	1050.5	11.7
-354-U1451B-22R-I-W Spot 226	2176	1289075	2.8	13.4402	0.8	1.7514	1.4	0.1708	1.1	0.79	1016.5	10.1	1027.7	8.7	1051.6	16.6	1051.6	16.6
-354-U1451B-22R-I-W Spot 128	516	865980	6.8	13.2424	0.7	1.8539	1.4	0.1781	1.3	0.89	1056.8	12.2	1064.8	9.3	1081.4	13.1	1081.4	13.1
-354-U1451B-22R-I-W Spot 191	1289	65487	6.4	13.2394	1.3	1.6722	1.5	0.1606	0.8	0.54	960.3	7.3	998.0	9.7	1081.8	25.6	1081.8	25.6
-354-U1451B-22R-I-W Spot 71	931	429555	2.9	13.1939	0.6	1.8493	1.0	0.1770	0.8	0.76	1050.8	7.3	1063.2	6.6	1088.7	12.9	1088.7	12.9
-354-U1451B-22R-I-W Spot 219	688	1536022	0.9	13.1894	0.7	1.9586	1.0	0.1874	0.8	0.76	1107.5	7.7	1101.4	6.7	1089.4	13.1	1089.4	13.1
-354-U1451B-22R-I-W Spot 235	985	6934289	7.0	13.1818	0.6	1.8616	1.4	0.1781	1.3	0.90	1056.4	12.7	1067.6	9.6	1090.5	12.6	1090.5	12.6
-354-U1451B-22R-I-W Spot 22	579	2117833	4.5	13.1791	0.6	1.9184	1.1	0.1834	0.9	0.84	1085.8	9.1	1087.5	7.2	1091.0	11.7	1091.0	11.7
-354-U1451B-22R-I-W Spot 288	113	37427	0.9	13.1615	0.9	1.6988	1.3	0.1622	0.9	0.69	969.2	7.9	1008.1	8.2	1093.6	18.7	1093.6	18.7
-354-U1451B-22R-I-W Spot 198	290	789464	2.9	13.1403	0.9	1.9035	1.3	0.1815	0.9	0.74	1075.1	9.4	1082.3	8.5	1096.9	17.3	1096.9	17.3
-354-U1451B-22R-I-W Spot 109	816	147671	1.0	13.1357	1.0	1.5925	1.9	0.1518	1.7	0.87	911.0	14.1	967.3	12.0	1097.6	19.1	1097.6	19.1
-354-U1451B-22R-I-W Spot 140	106	70994	2.9	13.1322	1.5	1.6760	1.8	0.1597	1.0	0.55	955.1	8.8	999.4	11.5	1098.1	30.3	1098.1	30.3
-354-U1451B-22R-I-W Spot 78	76	219517	1.9	13.1246	1.0	1.9332	1.4	0.1841	1.1	0.74	1089.3	10.6	1092.6	9.5	1099.3	19.1	1099.3	19.1
-354-U1451B-22R-I-W Spot 214	433	1801602	6.8	12.9232	1.0	1.9507	1.4	0.1829	1.0	0.73	1082.9	10.1	1098.7	9.3	1130.1	19.0	1130.1	19.0
-354-U1451B-22R-I-W Spot 304	227	23603	1.6	12.9092	1.7	1.6626	2.2	0.1557	1.4	0.62	933.0	12.1	994.4	14.2	1132.3	34.8	1132.3	34.8
-354-U1451B-22R-I-W Spot 127	2393	3329004	6.7	12.8473	0.6	1.8986	0.9	0.1770	0.7	0.78	1050.5	6.8	1080.6	5.9	1141.8	11.1	1141.8	11.1
-354-U1451B-22R-I-W Spot 32	218	99960	2.8	12.7847	0.7	2.1050	1.0	0.1953	0.7	0.74	1149.9	7.8	1150.4	6.9	1151.5	13.4	1151.5	13.4
-354-U1451B-22R-I-W Spot 50	62	176542	1.3	12.7432	0.7	2.1505	1.0	0.1988	0.7	0.69	1169.1	7.5	1165.2	7.0	1158.0	14.5	1158.0	14.5
-354-U1451B-22R-I-W Spot 254	278	164017	2.1	12.7191	0.7	2.1074	1.1	0.1945	0.9	0.80	1145.7	9.4	1151.2	7.7	1161.8	13.3	1161.8	13.3
-354-U1451B-22R-I-W Spot 204	637	1178882	1.4	12.7173	0.7	1.9842	1.5	0.1831	1.3	0.88	1083.9	12.9	1110.2	9.9	1162.0	13.6	1162.0	13.6
-354-U1451B-22R-I-W Spot 30	279	123866	2.4	12.7007	0.9	1.9150	1.4	0.1765	1.2	0.80	1047.7	11.2	1086.3	9.6	1164.6	17.2	1164.6	17.2
-354-U1451B-22R-I-W Spot 117	331	422223	3.3	12.6836	0.7	1.9645	1.9	0.1808	1.8	0.93	1071.3	17.9	1103.4	13.1	1167.3	14.0	1167.3	14.0
-354-U1451B-22R-I-W Spot 18	406	311860	2.8	12.6097	0.6	2.2133	1.4	0.2025	1.3	0.90	1188.8	14.0	1185.3	10.1	1178.9	12.7	1178.9	12.7
-354-U1451B-22R-I-W Spot 212	121	478733	1.7	12.5405	0.7	2.1511	1.2	0.1957	1.0	0.82	1152.4	10.5	1165.4	8.4	1189.7	13.5	1189.7	13.5
-354-U1451B-22R-I-W Spot 180	169	268380	2.1	12.5307	0.8	2.2315	1.2	0.2029	0.9	0.76	1190.8	10.3	1191.0	8.7	1191.3	15.9	1191.3	15.9
-354-U1451B-22R-I-W Spot 2	89	85822	0.8	12.5113	1.1	2.0978	1.4	0.1904	0.8	0.59	1123.8	8.2	1148.1	9.3	1194.4	21.7	1194.4	21.7
-354-U1451B-22R-I-W Spot 112	1112	14814238	41.1	12.4897	0.6	2.0535	1.1	0.1861	0.9	0.83	1100.2	9.0	1133.5	7.3	1197.8	11.7	1197.8	11.7
-354-U1451B-22R-I-W Spot 286	944	375867	1.9	12.4856	0.6	2.0661	1.2	0.1872	1.0	0.86	1106.0	10.4	1137.6	8.2	1198.4	12.1	1198.4	12.1
-354-U1451B-22R-I-W Spot 61	596	1221479	2.2	12.4307	0.4	2.2714	1.3	0.2049	1.2	0.94	1201.4	12.9	1203.4	8.8	1207.1	8.6	1207.1	8.6
-354-U1451B-22R-I-W Spot 178	78	147630	1.3	12.3159	0.8	2.4068	1.2	0.2151	0.9	0.74	1255.8	9.8	1244.6	8.3	1225.3	15.4	1225.3	15.4

-354-U1451B-22R-I-W Spot 227	503	685016	3.5	12.3071	0.7	2.2374	1.3	0.1998	1.1	0.85	1174.2	12.2	1192.8	9.4	1226.7	13.8	1226.7	13.8
-354-U1451B-22R-I-W Spot 149	435	211349	1.5	11.9267	0.5	2.5168	1.1	0.2178	1.0	0.90	1270.3	11.6	1276.9	8.1	1288.1	9.5	1288.1	9.5
-354-U1451B-22R-I-W Spot 210	328	456871	1.9	11.7542	0.6	2.5765	1.1	0.2197	1.0	0.87	1280.5	11.6	1294.0	8.4	1316.4	10.9	1316.4	10.9
-354-U1451B-22R-I-W Spot 133	63	61454	0.6	11.6960	1.1	2.4158	1.7	0.2050	1.4	0.78	1202.2	15.0	1247.3	12.5	1326.0	21.0	1326.0	21.0
-354-U1451B-22R-I-W Spot 60	110	3417623	1.3	11.4608	0.8	2.5889	1.4	0.2153	1.1	0.80	1256.9	12.6	1297.5	10.0	1365.3	15.7	1365.3	15.7
-354-U1451B-22R-I-W Spot 129	217	167522	6.4	11.3412	0.8	2.9161	1.4	0.2400	1.1	0.81	1386.5	14.0	1386.1	10.5	1385.4	15.6	1385.4	15.6
-354-U1451B-22R-I-W Spot 26	886	151944	2.8	11.1144	0.8	2.4541	1.2	0.1979	0.9	0.77	1164.1	9.8	1258.6	8.6	1424.1	14.4	1424.1	14.4
-354-U1451B-22R-I-W Spot 162	68	86403	1.1	10.9644	0.8	3.2208	1.2	0.2562	0.9	0.76	1470.5	11.7	1462.2	9.1	1450.0	14.5	1450.0	14.5
-354-U1451B-22R-I-W Spot 182	1169	678823	1.5	10.9154	0.5	3.0562	1.1	0.2420	0.9	0.86	1397.3	11.4	1421.8	8.1	1458.5	10.4	1458.5	10.4
-354-U1451B-22R-I-W Spot 96	590	9707649	1.7	10.6218	0.6	3.4808	1.1	0.2683	0.9	0.86	1532.0	12.7	1522.9	8.6	1510.2	10.5	1510.2	10.5
-354-U1451B-22R-I-W Spot 51	104	165269	0.8	10.6203	0.6	3.2981	1.0	0.2542	0.7	0.75	1459.8	9.5	1480.6	7.5	1510.5	12.1	1510.5	12.1
-354-U1451B-22R-I-W Spot 65	362	1168362	1.7	10.6198	0.6	3.3858	1.2	0.2609	1.0	0.85	1494.4	13.8	1501.1	9.5	1510.6	12.1	1510.6	12.1
-354-U1451B-22R-I-W Spot 240	559	1585520	1.6	10.5922	0.8	3.3413	1.3	0.2568	1.1	0.79	1473.4	13.9	1490.8	10.4	1515.5	15.3	1515.5	15.3
-354-U1451B-22R-I-W Spot 12	442	2520039	2.8	10.4242	0.8	3.5448	1.6	0.2681	1.3	0.86	1531.2	18.3	1537.3	12.4	1545.6	15.2	1545.6	15.2
-354-U1451B-22R-I-W Spot 189	43	79078	1.0	10.2769	0.9	3.8955	1.3	0.2905	1.0	0.75	1643.9	14.6	1612.7	10.9	1572.3	16.8	1572.3	16.8
-354-U1451B-22R-I-W Spot 184	284	10285672	0.6	10.2273	0.7	3.7004	1.3	0.2746	1.1	0.85	1564.1	16.0	1571.5	10.8	1581.3	13.1	1581.3	13.1
-354-U1451B-22R-I-W Spot 293	143	583491	1.6	10.1541	0.6	3.8901	1.1	0.2866	1.0	0.85	1624.5	13.9	1611.6	9.3	1594.8	11.4	1594.8	11.4
-354-U1451B-22R-I-W Spot 82	218	1042581	1.7	10.0716	1.1	3.8553	1.9	0.2817	1.5	0.81	1600.1	21.2	1604.4	14.9	1610.0	20.3	1610.0	20.3
-354-U1451B-22R-I-W Spot 35	310	257780	1.4	10.0546	0.7	3.9483	1.3	0.2880	1.1	0.83	1631.8	16.1	1623.6	10.9	1613.1	13.8	1613.1	13.8
-354-U1451B-22R-I-W Spot 256	47	80550	1.0	9.7270	0.6	4.0676	1.0	0.2871	0.8	0.81	1626.9	12.2	1647.8	8.5	1674.6	11.3	1674.6	11.3
-354-U1451B-22R-I-W Spot 115	414	5534098	3.6	9.6076	0.6	4.4919	1.1	0.3131	1.0	0.85	1756.1	15.0	1729.5	9.5	1697.4	11.2	1697.4	11.2
-354-U1451B-22R-I-W Spot 242	71	167256	0.8	9.5422	0.8	4.4664	1.2	0.3092	0.9	0.76	1736.9	13.3	1724.7	9.6	1709.9	13.9	1709.9	13.9
-354-U1451B-22R-I-W Spot 74	676	2491713	1.4	9.5033	0.8	4.3537	1.4	0.3002	1.2	0.85	1692.3	17.8	1703.6	11.7	1717.5	13.9	1717.5	13.9
-354-U1451B-22R-I-W Spot 298	271	484460	2.0	9.4095	0.7	4.3918	1.4	0.2998	1.2	0.87	1690.5	18.4	1710.8	11.8	1735.7	12.9	1735.7	12.9
-354-U1451B-22R-I-W Spot 89	182	546451	10.2	9.3760	0.8	4.5271	1.6	0.3080	1.4	0.86	1730.8	21.1	1736.0	13.5	1742.2	15.4	1742.2	15.4
-354-U1451B-22R-I-W Spot 202	915	263950	1.2	9.3328	0.6	4.3864	1.2	0.2970	1.1	0.88	1676.6	15.6	1709.8	10.0	1750.7	10.6	1750.7	10.6
-354-U1451B-22R-I-W Spot 144	339	93150	1.7	9.3275	1.0	4.5012	1.9	0.3046	1.7	0.86	1714.3	25.0	1731.2	16.1	1751.7	18.1	1751.7	18.1
-354-U1451B-22R-I-W Spot 223	1367	791629	16.2	9.2916	0.6	4.2846	1.0	0.2889	0.8	0.81	1635.8	11.6	1690.4	8.2	1758.8	10.6	1758.8	10.6
-354-U1451B-22R-I-W Spot 196	116	151376	1.0	9.2067	0.7	4.9376	1.2	0.3298	0.9	0.79	1837.6	14.5	1808.7	9.8	1775.5	13.0	1775.5	13.0
-354-U1451B-22R-I-W Spot 138	217	212790	2.4	9.1938	1.4	4.8105	1.7	0.3209	1.0	0.58	1794.1	15.2	1786.7	14.0	1778.1	24.8	1778.1	24.8
-354-U1451B-22R-I-W Spot 137	231	114552	1.9	9.1337	0.8	4.1026	2.0	0.2719	1.8	0.91	1550.4	24.8	1654.8	16.2	1790.1	15.2	1790.1	15.2
-354-U1451B-22R-I-W Spot 285	265	1461629	7.7	9.1254	0.6	4.8200	1.2	0.3191	1.0	0.87	1785.5	15.9	1788.4	9.8	1791.7	10.4	1791.7	10.4
-354-U1451B-22R-I-W Spot 75	64	55675	0.9	8.9724	0.6	5.0018	0.8	0.3256	0.6	0.73	1817.1	9.7	1819.6	7.1	1822.4	10.3	1822.4	10.3
-354-U1451B-22R-I-W Spot 217	272	1278381	1.8	8.9630	0.5	4.9674	1.1	0.3230	1.0	0.88	1804.6	15.8	1813.8	9.6	1824.3	9.9	1824.3	9.9
-354-U1451B-22R-I-W Spot 314	885	323018	1.7	8.9438	0.6	5.0029	1.3	0.3247	1.1	0.87	1812.5	17.6	1819.8	10.9	1828.2	11.7	1828.2	11.7
-354-U1451B-22R-I-W Spot 79	349	2830832	5.9	8.9272	0.6	5.2107	1.2	0.3375	1.1	0.86	1874.7	17.2	1854.4	10.5	1831.6	11.3	1831.6	11.3
-354-U1451B-22R-I-W Spot 238	312	3080033	1.5	8.9205	0.7	5.0718	1.2	0.3283	1.0	0.83	1830.0	15.8	1831.4	10.1	1833.0	12.1	1833.0	12.1
-354-U1451B-22R-I-W Spot 77	528	1110549	3.3	8.9061	0.5	5.2124	1.1	0.3368	0.9	0.87	1871.4	15.2	1854.6	9.2	1835.9	9.9	1835.9	9.9
-354-U1451B-22R-I-W Spot 229	530	424594	2.8	8.8955	0.6	4.9583	1.3	0.3200	1.1	0.88	1789.9	17.6	1812.2	10.9	1838.0	11.2	1838.0	11.2
-354-U1451B-22R-I-W Spot 110	371	190403	4.4	8.8844	0.6	5.2704	1.4	0.3397	1.2	0.88	1885.5	19.7	1864.1	11.6	1840.3	11.5	1840.3	11.5
-354-U1451B-22R-I-W Spot 120	712	894075	3.3	8.8725	0.4	5.0333	1.1	0.3240	1.0	0.94	1809.4	16.2	1824.9	9.3	1842.7	6.7	1842.7	6.7
-354-U1451B-22R-I-W Spot 291	190	397024	3.6	8.8474	1.1	4.9194	2.6	0.3158	2.4	0.91	1769.2	36.6	1805.6	21.9	1847.8	19.6	1847.8	19.6
-354-U1451B-22R-I-W Spot 106	258	167832	6.7	8.8338	0.5	5.2675	0.9	0.3376	0.7	0.81	1875.2	11.9	1863.6	7.7	1850.6	9.5	1850.6	9.5
-354-U1451B-22R-I-W Spot 280	237	1586501	0.9	8.8302	0.6	4.9783	1.0	0.3190	0.9	0.82	1784.7	13.3	1815.6	8.8	1851.4	10.9	1851.4	10.9
-354-U1451B-22R-I-W Spot 255	116	271695	1.8	8.8104	0.8	5.0857	1.2	0.3251	1.0	0.77	1814.6	15.2	1833.7	10.6	1855.4	14.4	1855.4	14.4
-354-U1451B-22R-I-W Spot 247	657	3882659	2.9	8.7866	0.8	5.2649	1.4	0.3357	1.1	0.80	1865.8	17.8	1863.2	11.8	1860.3	15.0	1860.3	15.0
-354-U1451B-22R-I-W Spot 297	193	180323	1.6	8.7588	0.6	5.0850	1.1	0.3232	0.9	0.83	1805.2	14.2	1833.6	9.3	1866.0	11.1	1866.0	11.1
-354-U1451B-22R-I-W Spot 142	218	203254	3.1	8.7582	0.7	5.3081	1.3	0.3373	1.0	0.84	1873.8	17.0	1870.2	10.7	1866.2	12.3	1866.2	12.3
-354-U1451B-22R-I-W Spot 76	146	185305	1.2	8.7526	0.7	5.3403	1.2	0.3391	1.0	0.81	1882.6	15.6	1875.3	10.1	1867.3	12.3	1867.3	12.3
-354-U1451B-22R-I-W Spot 310	289	379813	1.9	8.2055	0.6	6.0087	1.3	0.3577	1.1	0.89	1971.5	19.4	1977.1	11.2	1983.0	10.5	1983.0	10.5
-354-U1451B-22R-I-W Spot 203	237	9094	1.9	7.8283	3.6	5.4883	4.6	0.3117	2.7	0.60	1749.3	42.1	1898.8	39.2	2066.3	64.2	2066.3	64.2
-354-U1451B-22R-I-W Spot 27	108	161900	1.2	7.8147	0.7	6.8884	1.1	0.3906	0.9	0.79	2125.5	16.0	2097.2	9.9	2069.4	12.0	2069.4	12.0
-354-U1451B-22R-I-W Spot 279	279	638617	2.6	7.6281	1.1	6.8803	1.9	0.3808	1.5	0.81	2080.1	26.7	2096.1	16.4	2111.9	19.1	2111.9	19.1
-354-U1451B-22R-I-W Spot 173	203	370244	2.1	6.8173	0.7	8.6698	1.1	0.4289	0.9	0.80	2300.5	17.5	2303.9	10.3	2306.9	11.7	2306.9	11.7
-354-U1451B-22R-I-W Spot 167	133	210928	2.5	6.7538	1.8	6.9728	2.4	0.3417	1.7	0.68	1894.8	27.2	2108.0	21.6	2322.9	30.6	2322.9	30.6
-354-U1451B-22R-I-W Spot 53	433	570593	5.0	6.6864	0.6	8.9412	1.2	0.4338	1.1	0.88	2322.8	20.5	2332.0	10.9	2340.1	9.8	2340.1	9.8
-354-U1451B-22R-I-W Spot 146	4	110075	1.3	6.4786	1.2	9.5288	1.4	0.4479	0.8	0.59	2386.0	16.9	2390.3	13.1	2394.0	19.6	2394.0	19.6
-354-U1451B-22R-I-W Spot 87	794	668015	1.7	6.3922	0.9	8.5855	1.5	0.3982	1.2	0.80	2160.8	21.8	2295.0	13.5	2416.8	15.2	2416.8	15.2
-354-U1451B-22R-I-W Spot 130	534	932490	1.6	6.3727	0.6	9.5790	1.1	0.4429	0.9	0.86	2363.7	18.4	2395.2	9.9	2422.0	9.4	2422.0	9.4
-354-U1451B-22R-I-W Spot 222	6	43789	2.4	6.3416	1.1	10.0977	1.6	0.4646	1.1	0.72	2460.0	23.0	2443.8	14.4	2430.3	18.3	2430.3	18.3
-354-U1451B-22R-I-W Spot 10	700	848244	1.4	6.2896	0.5	8.7013	0.9	0.3971	0.8	0.88	2155.6	15.0	2307.2	8.5	2444.2	7.6	2444.2	7.6
-354-U1451B-22R-I-W Spot 277	268	293160	1.4	6.2492	0.6	10.2251	1.4	0.4636	1.2	0.89	2455.6	25.0	2455.4	12.7	2455.1	10.6	2455.1	10.6
-354-U1451B-22R-I-W Spot 208	195	425444	0.9	6.2056	0.6	10.4589	1.2	0.4709	1.0	0.86	2487.6	20.4	2476.3	10.7	2467.0	10.0	2467.0	10.0
-354-U1451B-22R-I-W Spot 177	208	338927	1.6	6.1896	0.7	10.4250	1.6	0.4682	1.4	0.89	2475.6	29.0	2473.3	14.7	2471.3	12.1	2471.3	12.1
-354-U1451B-22R-I-W Spot 295	450	2975308	1.3	6.1765	0.6	10.2450	1.1	0.4591	0.9	0.85	2435.8	18.9	2457.1	10.1	2474.9	9.7	2474.9	9.7
-354-U1451B-22R-I-W Spot 201	34	145169	0.8	6.1580	0.7	11.0177	1.1	0.4923	0.8	0.75	2580.6	17.2	2524.6	10.0	2479.9	12.0	2479.9	12.0
-354-U1451B-22R-I-W Spot 3	144	850376	1.2	6.1547	0.7	10.7189	1.1	0.4787	0.9	0.79	2521.5	18.4	2499.1	10.4	2480.9	11.5	2480.9	11.5
-354-U1451B-22R-I-W Spot 20	164	216307	0.8	6.1499	0.5	10.4720	1.0	0.4673	0.9	0.85	2471.6	17.8	2477.4	9.5	2482.2	9.2	2482.2	9.2
-354-U1451B-22R-I-W Spot 92	95	192678	1.7	6.1444	0.7	10.5365	1.1	0.4697	0.9	0.81	2482.4	18.7	2483.1	10.4	2483.7	11.1	2483.7	11.1
-354-U1451B-22R-I-W Spot 57	77	298381	1.4	6.1279	0.7	10.7376	1.0	0.4774	0.7	0.71	2516.1	15.2	2500.7	9.5	2488.2	12.1	2488.2	12.1
-354-U1451B-22R-I-W Spot 147	355	3809045	0.8	6.1152	0.6	8.8459	1.1	0.3925	1.0	0.84	2134.4	17.5	2322.2	10.5	2491.7	10.5	2491.7	10.5
-354-U1451B-22R-I-W Spot 105	168	1927473	1.8	6.0729	0.8	11.1955	1.3	0.4933	1.0	0.79	2585.0	21.4	2539.5	11.8	2503.4	13.0	2503.4	13.0
-354-U1451B-22R-I-W Spot 63	459	666531	5.7	5.9998	1.0	10.3256	1.4	0.4495	1.0	0.73	2393.1	20.3	2464.4	12.9	2523.8	16.1	2523.8	16.1
-354-U1451B-22R-I-W Spot 34	677	212201	1.7	5.9781	0.6	9.9155	1.3	0.4301	1.1	0.88	2306.1	21.9	2427.0	11.8	2529.8	10.1	2529.8	10.1
-354-U1451B-22R-I-W Spot 264	383	915283	2.1	5.9009	0.6	11.1964	1.3	0.4794	1.2	0.89	2524.6	24.6	2539.6	12.4	2551.6	10.3	2551.6	10.3
-354-U1451B-22R-I-W Spot 228	68	188956	1.1	5.8934	0.7	11.3224	1.0	0.4842	0.8	0.75	2545.4	16.5	2550.1	9.7	2553.8	11.5	2553.8	11.5
-354-U1451B-22R-I-W Spot 95	198	308399	1.8	5.6954	0.7	11.6702	1.0	0.4823	0.8	0.76	2537.2	16.7	2578.3	9.8	2610.8	11.4	2610.8	11.4
-354-U1451B-22R-I-W Spot 49	398	841316	1.7	5.6790	0.6	12.0457	1.5	0.4964	1.3	0.91	2598.1	28.8	2608.0	13.9	2615.6	10.4	2615.6	10.4
-354-U1451B-22R-I-W Spot 172	591	3367258	5.4	5.6580	0.6	12.1524	1.1	0.4989	0.9	0.83	2609.1	19.3	2616.2	10.2	2621.8	10.2	2621.8	10.2
-354-U1451B-22R-I-W Spot 244	131	1269002	1.1	5.6445	0.7	12.4291	2.2	0.5090	2.1	0.95	2652.5	46.3	2637.4	21.0	2625.8	11.3	2625.8	11.3
-354-U1451B-22R-I-W Spot 64	432	727187	2.7	5.6158	0.5	12.0962	0.9	0.4929	0.8	0.85	2583.2	16.7	2611.9	8.7	2634.2	8.2	2634.2	8.2
-354-U1451B-22R-I-W Spot 28	122	184864	1.6	5.5770	0.5	13.0022	1.3	0.5261	1.2	0.93	2725.2	26.3	2679.8	12.0	2645.7	7.8	2645.7	7.8
-354-U1451B-22R-I-W Spot 126	370	557208	2.0	5.4513	1.9	12.1769	2.8	0.4816	2.0	0.74	2534.4	42.8	2618.1	26.0	2683.5	31.0	2683.5	31.0
-354-U1451B-22R-I-W Spot 259	127	386220	1.9	5.4509	0.6	11.8433	1.1	0.4684	0.9	0.82	2476.6	17.9	2592.1	9.9	2683.6	10.0	2683.6	10.0
-354-U1451B-22R-I-W Spot 207	910	1089718	2.0	5.3139	0.5	14.1096	1.0	0.5440	0.8	0.83	2800.3	18.8	2757.1	9.4	2725.6	9.0	2725.6	9.0
-354-U1451B-22R-I-W Spot 287	120	227110	2.2	4.9660	0.7	12.8151	1.4	0.4618	1.2	0.86	2447.3	24.9	2666.2	13.4	2836.6	11.9	2836.6	11.9
-354-U1451B-22R-I-W Spot 13	253	601226	1.7	3.8376	0.5	23.4830	1.4	0.6539	1.3	0.92	3243.3	32.0	3247.2	13.3	3249.5	8.4	3249.5	8.4

Table _____. U-Pb geochronologic analyses.																		
#23 U1451B_41R						Isotope ratios					Apparent ages (Ma)							
Mld Miocene 14.0 Ma																		
Analysis	U	206Pb	U/Th	206Pb*	±	207Pb*	±	206Pb*	±	error	206Pb*	±	207Pb*	±	206Pb*	±	Best age	±
	(ppm)	204Pb		207Pb*	(%)	235U*	(%)	238U	(%)	corr.	238U*	(Ma)	235U	(Ma)	207Pb*	(Ma)	(Ma)	(Ma)
354U1451 B41R1W #33 -Spot 74	72	3985	2.3	19.6641	3.3	0.0890	3.6	0.0127	1.4	0.40	81.4	1.2	86.6	3.0	233.2	75.7	81.4	1.2
354U1451 B41R1W #33 -Spot 75	303	40590	2.8	20.3767	1.2	0.0868	1.9	0.0128	1.4	0.77	82.2	1.2	84.6	1.5	150.4	28.5	82.2	1.2
354U1451 B41R1W #33 -Spot 128	1947	73335	2.5	20.6425	0.7	0.1260	1.3	0.0189	1.1	0.83	120.5	1.3	120.5	1.5	120.0	17.1	120.5	1.3
354U1451 B41R1W #33 -Spot 4	23	4205	2.4	22.5971	4.1	0.1224	4.5	0.0201	2.1	0.45	128.1	2.6	117.3	5.0	NA	NA	128.1	2.6
354U1451 B41R1W #33 -Spot 119	297	53647	1.6	20.2859	1.2	0.1397	1.8	0.0206	1.4	0.75	131.2	1.8	132.8	2.2	160.9	27.6	131.2	1.8
354U1451 B41R1W #33 -Spot 185	149	15094	1.6	20.4029	1.7	0.1589	2.0	0.0235	1.1	0.54	149.9	1.6	149.7	2.8	147.4	39.4	149.9	1.6
354U1451 B41R1W #33 -Spot 303	1683	15971	1.2	14.8639	1.4	0.4853	1.8	0.0523	1.0	0.59	328.9	3.4	401.7	5.9	845.4	30.0	328.9	3.4
354U1451 B41R1W #33 -Spot 256	191	23763	1.2	16.9303	0.7	0.6225	1.5	0.0765	1.3	0.88	475.0	6.0	491.4	5.8	568.6	15.0	475.0	6.0
354U1451 B41R1W #33 -Spot 40	1239	547647	3.8	17.4108	0.6	0.6112	1.5	0.0772	1.3	0.90	479.4	6.2	484.3	5.7	507.4	14.2	479.4	6.2
354U1451 B41R1W #33 -Spot 60	3304	285621	2.0	17.3534	0.7	0.6164	1.6	0.0776	1.4	0.89	481.9	6.5	487.6	6.1	514.6	15.4	481.9	6.5
354U1451 B41R1W #33 -Spot 255	98	63817	0.5	16.8206	0.9	0.6621	1.5	0.0808	1.2	0.78	500.9	5.5	515.9	6.0	582.7	20.3	500.9	5.5
354U1451 B41R1W #33 -Spot 223	149	101649	0.6	17.0869	1.0	0.6658	1.9	0.0825	1.6	0.86	511.3	8.1	518.2	7.8	548.5	21.4	511.3	8.1
354U1451 B41R1W #33 -Spot 136	141	86296	0.6	17.3644	0.7	0.6605	1.6	0.0832	1.5	0.91	515.3	7.3	514.9	6.5	513.2	14.8	515.3	7.3
354U1451 B41R1W #33 -Spot 208	48	17527	0.9	17.4795	1.2	0.6589	1.6	0.0836	1.1	0.67	517.4	5.4	513.9	6.6	498.7	26.7	517.4	5.4
354U1451 B41R1W #33 -Spot 212	206	21678	0.7	17.2386	0.8	0.6682	1.4	0.0836	1.1	0.79	517.5	5.4	519.6	5.6	529.2	18.4	517.5	5.4
354U1451 B41R1W #33 -Spot 229	315	32416	4.0	17.0489	0.8	0.6852	1.6	0.0848	1.4	0.87	524.5	7.1	529.9	6.7	553.3	17.5	524.5	7.1
354U1451 B41R1W #33 -Spot 213	201	37858	1.7	17.0609	0.9	0.6882	1.4	0.0852	1.1	0.75	527.0	5.4	531.7	5.8	551.8	20.1	527.0	5.4
354U1451 B41R1W #33 -Spot 26	470	414110	3.5	17.2458	1.1	0.6820	1.8	0.0853	1.4	0.80	527.9	7.2	528.0	7.3	528.3	23.0	527.9	7.2
354U1451 B41R1W #33 -Spot 254	532	70129	3.0	16.9152	0.9	0.6980	1.6	0.0857	1.4	0.84	529.8	6.9	537.6	6.7	570.5	18.8	529.8	6.9
354U1451 B41R1W #33 -Spot 141	234	199298	2.3	17.1186	1.0	0.6903	1.3	0.0857	0.9	0.67	530.3	4.4	533.0	5.4	544.5	21.3	530.3	4.4
354U1451 B41R1W #33 -Spot 277	158	146800	1.8	16.7798	0.9	0.7136	1.6	0.0869	1.4	0.84	537.0	7.1	546.9	7.0	587.9	19.2	537.0	7.1
354U1451 B41R1W #33 -Spot 68	665	378742	2.1	17.2872	0.7	0.6928	1.4	0.0869	1.2	0.88	537.1	6.4	534.5	5.9	523.0	15.0	537.1	6.4
354U1451 B41R1W #33 -Spot 43	2798	123347	3.4	16.8558	0.7	0.7416	1.7	0.0907	1.5	0.91	559.7	8.1	563.3	7.2	578.2	14.9	559.7	8.1
354U1451 B41R1W #33 -Spot 23	444	367486	0.3	16.7303	0.6	0.7637	1.2	0.0927	1.0	0.84	571.6	5.4	576.2	5.1	594.4	13.6	571.6	5.4
354U1451 B41R1W #33 -Spot 70	2398	623977	34.3	16.8904	0.8	0.7610	1.6	0.0933	1.4	0.87	574.8	7.8	574.6	7.2	573.7	17.4	574.8	7.8
354U1451 B41R1W #33 -Spot 288	97	17630	4.1	16.7506	0.9	0.7966	1.4	0.0968	1.1	0.76	595.7	6.0	594.9	6.2	591.7	19.2	595.7	6.0
354U1451 B41R1W #33 -Spot 217	1358	79173	3.6	16.3504	0.6	0.8172	1.3	0.0970	1.2	0.88	596.5	6.8	606.5	6.2	644.0	13.5	596.5	6.8
354U1451 B41R1W #33 -Spot 35	144	23488	3.6	16.5889	0.9	0.8124	1.5	0.0978	1.1	0.78	601.4	6.6	603.8	6.7	612.7	19.8	601.4	6.6
354U1451 B41R1W #33 -Spot 231	1487	13027598	27.0	15.9652	0.8	0.8933	1.6	0.1035	1.4	0.86	634.8	8.3	648.2	7.7	695.0	17.7	634.8	8.3
354U1451 B41R1W #33 -Spot 188	869	208830	3.5	16.3413	0.7	0.8830	1.3	0.1047	1.1	0.83	641.9	6.6	642.6	6.2	645.2	15.6	641.9	6.6
354U1451 B41R1W #33 -Spot 33	94	19557	3.1	16.3660	0.9	0.9130	1.7	0.1084	1.5	0.85	663.5	9.2	658.6	8.3	641.9	19.2	663.5	9.2
354U1451 B41R1W #33 -Spot 193	166	56278	1.8	15.4711	0.7	0.9673	1.1	0.1086	0.9	0.77	664.6	5.5	687.1	5.7	761.6	15.6	664.6	5.5
354U1451 B41R1W #33 -Spot 215	163	35019	2.2	15.5919	1.0	1.0467	1.8	0.1184	1.5	0.83	721.5	10.1	727.3	9.3	745.2	21.1	721.5	10.1
354U1451 B41R1W #33 -Spot 63	362	55414	3.0	15.0909	0.9	1.0873	1.8	0.1191	1.5	0.85	725.1	10.4	747.2	9.4	813.9	19.3	725.1	10.4
354U1451 B41R1W #33 -Spot 138	431	62398	7.8	15.6574	0.8	1.0563	1.6	0.1200	1.4	0.86	730.6	9.4	732.0	8.2	736.3	16.7	730.6	9.4
354U1451 B41R1W #33 -Spot 236	539	158395	4.8	15.3797	0.7	1.0828	1.3	0.1208	1.1	0.84	735.4	7.4	745.0	6.7	774.1	14.7	735.4	7.4
354U1451 B41R1W #33 -Spot 90	73	48309	1.6	15.1142	1.0	1.1158	1.6	0.1224	1.2	0.76	744.2	8.4	761.0	8.4	810.6	21.5	744.2	8.4
354U1451 B41R1W #33 -Spot 121	60	50379	0.8	15.4957	1.2	1.0985	1.7	0.1235	1.1	0.68	750.7	8.0	752.6	8.8	758.2	25.6	750.7	8.0
354U1451 B41R1W #33 -Spot 250	95	46981	1.6	15.1393	0.9	1.1273	1.7	0.1238	1.5	0.86	752.6	10.4	766.5	9.2	807.1	18.1	752.6	10.4
354U1451 B41R1W #33 -Spot 251	1370	182303	10.8	14.2630	0.8	1.2092	3.2	0.1251	3.1	0.97	760.1	22.3	804.8	17.8	930.7	16.2	760.1	22.3
354U1451 B41R1W #33 -Spot 110	118	29627	1.6	15.3207	0.9	1.1496	1.6	0.1278	1.3	0.82	775.3	9.3	777.1	8.5	782.1	18.6	775.3	9.3
354U1451 B41R1W #33 -Spot 216	225	122710	1.5	14.8569	0.7	1.1861	1.4	0.1279	1.3	0.87	775.6	9.2	794.1	7.9	846.4	14.5	775.6	9.2
354U1451 B41R1W #33 -Spot 287	1778	146583	3.4	14.9030	1.8	1.1889	2.4	0.1286	1.6	0.66	779.7	11.9	795.5	13.5	840.0	38.1	779.7	11.9
354U1451 B41R1W #33 -Spot 102	360	3374441	1.4	14.8961	0.7	1.2102	1.5	0.1308	1.3	0.87	792.5	9.6	805.3	8.2	841.0	15.4	792.5	9.6
354U1451 B41R1W #33 -Spot 130	492	1381409	3.0	15.3664	0.6	1.1766	1.3	0.1312	1.1	0.88	794.6	8.4	789.7	7.0	775.9	12.8	794.6	8.4
354U1451 B41R1W #33 -Spot 163	179	159077	1.4	14.5742	0.8	1.2462	1.6	0.1318	1.3	0.84	798.0	9.9	821.7	8.8	886.3	17.4	798.0	9.9
354U1451 B41R1W #33 -Spot 228	158	41211	1.2	15.0223	0.6	1.2362	1.5	0.1347	1.4	0.91	814.9	10.7	817.1	8.6	823.3	13.2	814.9	10.7
354U1451 B41R1W #33 -Spot 84	933	377578	12.1	14.9422	0.6	1.2529	1.2	0.1358	1.0	0.85	821.1	7.6	824.7	6.6	834.5	12.9	821.1	7.6
354U1451 B41R1W #33 -Spot 105	647	267017	0.8	14.4008	0.8	1.3041	1.5	0.1363	1.2	0.83	823.5	9.6	847.5	8.5	910.9	16.8	823.5	9.6
354U1451 B41R1W #33 -Spot 171	430	160275	3.9	14.8405	0.6	1.2656	1.7	0.1363	1.6	0.94	823.6	12.4	830.4	9.7	848.8	12.4	823.6	12.4
354U1451 B41R1W #33 -Spot 92	667	212433	16.9	14.4595	0.8	1.3077	1.8	0.1372	1.5	0.88	828.8	12.1	849.1	10.1	902.6	16.8	828.8	12.1
354U1451 B41R1W #33 -Spot 13	682	159927	2.4	14.4792	0.9	1.3122	1.5	0.1379	1.2	0.79	832.5	9.1	851.1	8.4	899.8	18.4	832.5	9.1
354U1451 B41R1W #33 -Spot 205	231	156208	2.1	15.1667	0.7	1.2537	1.4	0.1380	1.3	0.89	833.1	10.1	825.1	8.2	803.3	13.7	833.1	10.1
354U1451 B41R1W #33 -Spot 95	67	166735	1.7	14.7788	0.8	1.2893	1.3	0.1383	1.0	0.80	834.8	8.2	841.0	7.5	857.4	16.4	834.8	8.2
354U1451 B41R1W #33 -Spot 97	305	89472	1.8	14.9393	0.8	1.2787	1.8	0.1386	1.7	0.91	836.8	13.2	836.3	10.5	834.9	15.6	836.8	13.2
354U1451 B41R1W #33 -Spot 232	131	101275	2.1	14.4374	1.0	1.3336	1.6	0.1397	1.2	0.75	843.0	9.4	860.4	9.2	905.7	21.5	843.0	9.4
354U1451 B41R1W #33 -Spot 2	123	275595	1.4	14.5874	0.8	1.3238	1.1	0.1401	0.8	0.71	845.3	6.5	856.2	6.6	884.4	16.6	845.3	6.5
354U1451 B41R1W #33 -Spot 230	479	153356	0.7	14.5259	0.9	1.3295	1.6	0.1401	1.4	0.84	845.4	11.0	858.7	9.5	893.1	18.2	845.4	11.0
354U1451 B41R1W #33 -Spot 242	145	77824	0.7	14.6690	1.0	1.3192	2.4	0.1404	2.2	0.91	847.0	17.7	854.2	14.1	872.9	20.7	847.0	17.7
354U1451 B41R1W #33 -Spot 168	316	348348	7.6	14.9920	0.7	1.2929	1.5	0.1406	1.3	0.88	848.3	10.3	842.6	8.5	827.6	14.9	848.3	10.3
354U1451 B41R1W #33 -Spot 111	2177	398143	2.7	14.7610	0.7	1.3155	1.4	0.1409	1.2	0.86	849.7	9.8	852.5	8.3	859.9	15.5	849.7	9.8
354U1451 B41R1W #33 -Spot 8	72	19755	1.3	14.5674	1.0	1.3348	2.3	0.1411	2.1	0.90	850.8	16.5	861.0	13.4	887.2	21.0	850.8	16.5
354U1451 B41R1W #33 -Spot 151	181	975155	1.7	14.7750	1.1	1.3229	1.7	0.1418	1.4	0.79	854.9	11.0	855.8	10.0	857.9	21.8	854.9	11.0
354U1451 B41R1W #33 -Spot 313	131	28219	2.6	14.7676	0.9	1.3387	1.4	0.1434	1.1	0.77	864.1	8.5	862.7	7.9	859.0	18.0	864.1	8.5
354U1451 B41R1W #33 -Spot 3	362	756168	2.1	14.5054	0.8	1.3691	1.7	0.1441	1.5	0.88	867.8	12.0	875.8	9.9	896.0	16.5	867.8	12.0
354U1451 B41R1W #33 -Spot 285	66	12711	1.2	14.5519	1.0	1.3855	2.6	0.1463	2.4	0.93	880.1	19.6	882.8	15.1	889.5	19.7	880.1	19.6
354U1451 B41R1W #33 -Spot 245	609	95935	4.4	14.6184	0.8	1.3849	1.5	0.1469	1.3	0.86	883.5	10.8	882.5	9.0	880.0	16.2	883.5	10.8
354U1451 B41R1W #33 -Spot 174	267	72189	1.8	14.4212	0.7	1.4047	1.0	0.1470	0.7	0.69	884.0	5.6	890.9	5.9	908.0	14.8	884.0	5.6
354U1451 B41R1W #33 -Spot 182	180	52334	1.3	14.6099	0.8	1.3898	1.8	0.1473	1.6	0.89	886.0	13.3	884.6	10.7	881.2	17.1	886.0	13.3
354U1451 B41R1W #33 -Spot 82	284	187431	0.9	14.7857	0.9	1.3752	1.5	0.1475	1.2	0.82	887.1	10.3	878.4	8.9	856.4	17.8	887.1	10.3
354U1451 B41R1W #33 -Spot 56	154	88080	2.7	14.4991	0.9	1.4234	1.5	0.1497	1.2	0.81	899.5	10.3	898.8	9.1	896.9	18.4	899.5	10.3
354U1451 B41R1W #33 -Spot 280	164	69407	2.3	14.4641	0.8	1.4298	1.3	0.1501	1.1	0.81	901.3	9.1	901.5	8.0	901.9	16.0	901.9	16.0
354U1451 B41R1W #33 -Spot 101	137	61267	1.7	14.4453	0.7	1.4226	1.7	0.1491	1.5	0.90	896.0	12.8	898.5	10.2	904.6	15.4	904.6	15.4
354U1451 B41R1W #33 -Spot 98	479	322691	7.8	14.4077	0.9	1.4230	2.3	0.1488	2.1	0.92	894.0	17.6	898.6	13.7	910.0	19.1	910.0	19.1
354U1451 B41R1W #33 -Spot 116	531	205832	4.0	14.4062	0.8	1.4311	1.7	0.1496	1.5	0.89	898.7	12.6	902.0	10.0	910.2	15.6	910.2	15.6
354U1451 B41R1W #33 -Spot 127	106	67534	2.7	14.3271	0.9	1.4616	1.5	0.1519	1.3	0.82	911.8	10.7	914.7	9.3	921.5	18.2	921.5	18.2
354U1451 B41R1W #33 -Spot 184	593	432435	4.0	14.3233	0.7	1.4503	1.5	0.1507	1.3	0.87	905.1	10.8	910.0	8.8	922.1	14.7	922.1	14.7
354U1451 B41R1W #33 -Spot 219	580	299090	3.5	14.2607	0.7	1.4704	1.4	0.1521	1.2	0.88	913.0	10.6	918.3	8.6	931.0	13.7	931.0	13.7
354U1451 B41R1W #33 -Spot 218	99	71014	1.6	14.2245	0.9	1.4155	1.6	0.1461	1.4	0.84	879.0	11.2	895.5	9.7	936.3	18.4	936.3	18.4
354U1451 B41R1W #33 -Spot 293	201	50796	2.1	14.2188	0.7	1.4898	1.6	0.1537	1.5	0.90	921.7	12.5	926.3	9.9	937.1	14.6	937.1	14.6
354U1451 B41R1W #33 -Spot 276	142	175363	0.8	14.2124	0.9	1.4228	1.6	0.1467	1.3	0.82	882.6	10.8	898.5	9.5	938.0	18.7	938.0	18.7
354U1451 B41R1W #33 -Spot 69	389	120900	6.3	14.1702	0.7	1.5224	1.4	0.1565	1.2	0.84	937.5	10.1	939.4	8.4	944.1	15.2	944.1	15.2
354U1451 B41R1W #33 -Spot 272	266	383370	7.7	14.1496	0.9	1.4804	1.7	0.1520	1.5	0.85	912.1	12.4	922.4	10.3	947.1	18.3	947.1	18.3
354U1451 B41R1W #33 -Spot 172	96	67806	1.1	14.1454	1.0	1.5154	1.6	0.1555	1.2	0.76	931.9	10.5	936.6	9.8	947.7	21.2	947.7	21.2
354U1451 B41R1W #33 -Spot 73	317	71213	1.5	14.1132	0.7	1.5649	1.4	0.1602	1.2	0.85	958.1	10.6	956.4	8.7	952.4	15.2	952.4	15.2
354U1451 B41R1W #33 -Spot 5	247	559492	1.3	14.1101	0.8	1.5301	1.4	0.1567	1.2	0.85	938.2	10.6	942.5	8.8	952.8	15.7	952.8	15.7
354U1451 B41R1W #33 -Spot 44	1837	1464545	4.2	14.0895	0.8	1.4672	1.6	0.1500	1.4	0.88	900.9	12.0	917.0	9.8	955.8	15.7	955.8	15.7
354U1451 B41R1W #33 -Spot 12	573	106305	2.5	14.0766	0.9	1.5122	1.8	0.1545	1.6	0.87	925.9	13.6	935.3	11.0	957.7	18.0	957.7	18.0
354U1451 B41R1W #33 -Spot 48	304	62517	1.9	14.0736	0.8	1.5007	1.5	0.1532	1.3	0.87	919.1	11.4	930.7	9.4	958.1	15.7	958.1	15.7
354U1451 B41R1W #33 -Spot 266	677	964845	3.0	14.0632	0.7	1.4969	1.2	0.1527	0.9	0.77	916.3	7.6	929.1	7.0	959.6	15.2	959.6	15.2
354U1451 B41R1W #33 -Spot 268	1161	126150	12.6	14.0412	0.7	1.4239	1.7	0.1451	1.6	0.92	873.3	12.8	899.0	10.2	962.8	14.0	962.8	14.0
354U1451 B41R1W #33 -Spot 299	128	46854	1.2	14.0332	0.7	1.5721	1.3	0.1601	1.1	0.85	957.2	9.6	959.3	7.9	964.0	13.8	964.0	13.8
354U1451 B41R1W #33 -Spot 99	404	1080850	3.5	14.0261	0.7	1.5798	1.4	0.1608	1.2	0.87	961.1	10.8	962.3	8.6	965.0	14.0	965.0	14.0
354U1451 B41R1W #33 -Spot 189	306	70800	1.7	14.0188	0.5	1.4140	1.5	0.1438	1.4	0.93	866.3	11.2	894.8	8.8	966.1	11.1	966.1	11.1

354U1451 B41R1W #33 -Spot 158	348	140165	2.9	14.0100	0.7	1.6346	1.4	0.1662	1.3	0.88	991.0	11.5	983.6	9.0	967.4	14.0	967.4	14.0
354U1451 B41R1W #33 -Spot 164	776	251057	20.0	14.0041	0.7	1.5754	1.4	0.1601	1.2	0.86	957.2	11.1	960.6	8.9	968.2	14.8	968.2	14.8
354U1451 B41R1W #33 -Spot 226	361	33282	2.0	14.0009	1.3	1.3597	2.1	0.1381	1.7	0.80	834.1	13.2	871.7	12.3	968.7	25.7	968.7	25.7
354U1451 B41R1W #33 -Spot 159	648	89551	7.4	13.9932	0.8	1.6151	1.6	0.1640	1.3	0.85	978.9	12.2	976.1	9.9	969.8	16.9	969.8	16.9
354U1451 B41R1W #33 -Spot 131	513	87348	1.1	13.9901	0.6	1.5858	1.3	0.1610	1.1	0.90	962.2	10.2	964.6	8.0	970.2	11.5	970.2	11.5
354U1451 B41R1W #33 -Spot 180	460	715319	2.0	13.9721	0.8	1.5405	1.4	0.1562	1.2	0.83	935.5	10.3	946.7	8.8	972.9	16.3	972.9	16.3
354U1451 B41R1W #33 -Spot 278	426	78681	1.5	13.9335	0.7	1.4232	1.6	0.1439	1.4	0.88	866.6	11.2	898.7	9.3	978.5	14.8	978.5	14.8
354U1451 B41R1W #33 -Spot 118	93	71269	1.7	13.8971	0.7	1.5196	1.3	0.1532	1.1	0.83	919.1	9.0	938.3	7.8	983.9	14.5	983.9	14.5
354U1451 B41R1W #33 -Spot 207	671	79608	1.2	13.8890	0.7	1.5360	1.6	0.1548	1.5	0.91	927.8	12.6	944.9	9.9	985.0	13.6	985.0	13.6
354U1451 B41R1W #33 -Spot 153	350	105488	2.1	13.8869	0.7	1.5926	1.7	0.1605	1.6	0.91	959.4	13.8	967.3	10.7	985.3	14.7	985.3	14.7
354U1451 B41R1W #33 -Spot 275	407	387087	2.9	13.8743	0.7	1.6398	1.5	0.1651	1.3	0.89	984.9	12.0	985.6	9.3	987.2	13.4	987.2	13.4
354U1451 B41R1W #33 -Spot 238	209	50412	1.7	13.8734	0.7	1.6421	1.6	0.1653	1.4	0.88	986.2	12.8	986.5	10.0	987.3	15.0	987.3	15.0
354U1451 B41R1W #33 -Spot 165	144	407288	1.2	13.8696	0.7	1.4910	1.2	0.1500	1.0	0.83	901.2	8.6	926.7	7.5	987.9	14.0	987.9	14.0
354U1451 B41R1W #33 -Spot 281	437	67782	2.2	13.8485	0.8	1.5876	1.6	0.1595	1.4	0.87	954.1	12.1	965.3	9.8	991.0	16.0	991.0	16.0
354U1451 B41R1W #33 -Spot 32	375	62219	1.7	13.8442	0.7	1.6115	1.9	0.1619	1.8	0.94	967.2	16.3	974.7	12.1	991.6	13.4	991.6	13.4
354U1451 B41R1W #33 -Spot 198	160	63981	1.2	13.8359	0.8	1.6543	1.8	0.1661	1.6	0.89	990.5	14.6	991.2	11.4	992.8	16.8	992.8	16.8
354U1451 B41R1W #33 -Spot 54	488	850293	4.4	13.7993	0.7	1.6283	1.5	0.1630	1.4	0.89	973.6	12.2	981.2	9.6	998.2	14.4	998.2	14.4
354U1451 B41R1W #33 -Spot 176	433	219321	1.9	13.7955	0.6	1.6039	1.6	0.1605	1.5	0.92	959.8	13.3	971.7	10.2	998.8	13.1	998.8	13.1
354U1451 B41R1W #33 -Spot 246	225	50906	1.6	13.7827	0.8	1.5781	1.7	0.1578	1.4	0.87	944.7	12.7	961.6	10.4	1000.6	16.9	1000.6	16.9
354U1451 B41R1W #33 -Spot 310	197	286163	1.6	13.7599	0.8	1.5990	1.4	0.1596	1.2	0.84	954.8	10.6	969.8	8.9	1004.0	15.8	1004.0	15.8
354U1451 B41R1W #33 -Spot 196	855	723890	1.3	13.7397	0.8	1.6668	1.7	0.1662	1.5	0.89	991.0	13.9	996.0	10.8	1007.0	15.7	1007.0	15.7
354U1451 B41R1W #33 -Spot 195	1741	1831900	14.0	13.7388	0.8	1.6026	1.4	0.1598	1.2	0.84	955.5	10.8	971.2	9.0	1007.1	15.9	1007.1	15.9
354U1451 B41R1W #33 -Spot 191	160	114919	2.5	13.7218	1.5	1.5460	3.1	0.1539	2.7	0.87	922.9	23.1	948.9	19.0	1009.6	30.4	1009.6	30.4
354U1451 B41R1W #33 -Spot 302	3189	1374799	6.3	13.7136	0.6	1.7390	1.4	0.1730	1.3	0.91	1028.8	12.0	1023.1	8.9	1010.8	11.6	1010.8	11.6
354U1451 B41R1W #33 -Spot 132	260	316488	2.7	13.6641	0.8	1.6831	1.3	0.1669	1.1	0.80	994.8	9.7	1002.2	8.4	1018.2	16.2	1018.2	16.2
354U1451 B41R1W #33 -Spot 24	364	68981	3.6	13.6498	0.7	1.5707	1.8	0.1556	1.6	0.93	932.1	14.3	958.7	11.0	1020.3	13.2	1020.3	13.2
354U1451 B41R1W #33 -Spot 248	1172	820689	4.0	13.6306	0.6	1.6618	1.4	0.1644	1.3	0.90	981.0	11.6	994.1	8.9	1023.1	12.4	1023.1	12.4
354U1451 B41R1W #33 -Spot 203	595	105862	13.1	13.6210	1.0	1.6195	1.8	0.1601	1.5	0.84	957.1	13.4	977.8	11.2	1024.6	19.5	1024.6	19.5
354U1451 B41R1W #33 -Spot 117	349	106591	2.5	13.5778	0.8	1.7741	1.6	0.1748	1.3	0.85	1038.4	12.7	1036.0	10.1	1031.0	16.4	1031.0	16.4
354U1451 B41R1W #33 -Spot 298	165	76378	2.0	13.5761	0.7	1.6875	1.5	0.1662	1.3	0.87	991.3	12.2	1003.8	9.7	1031.2	15.1	1031.2	15.1
354U1451 B41R1W #33 -Spot 261	1025	188063	6.1	13.2984	0.8	1.7409	1.7	0.1680	1.5	0.87	1001.0	14.0	1023.8	11.1	1072.9	17.0	1072.9	17.0
354U1451 B41R1W #33 -Spot 307	982	26521	2.9	13.1830	1.1	1.6686	1.7	0.1596	1.3	0.77	954.6	11.6	996.7	10.8	1090.4	21.9	1090.4	21.9
354U1451 B41R1W #33 -Spot 17	609	85138	1.9	13.1599	0.7	1.9686	1.3	0.1880	1.1	0.86	1110.4	11.2	1104.8	8.6	1093.9	13.1	1093.9	13.1
354U1451 B41R1W #33 -Spot 139	270	58510	4.2	13.1597	0.6	1.9444	1.3	0.1857	1.2	0.90	1097.8	11.8	1096.5	8.7	1093.9	11.5	1093.9	11.5
354U1451 B41R1W #33 -Spot 53	766	252474	10.5	13.1421	0.7	1.8878	1.7	0.1800	1.5	0.92	1067.0	15.1	1076.8	11.1	1096.6	13.0	1096.6	13.0
354U1451 B41R1W #33 -Spot 294	462	412567	1.3	13.1337	0.8	1.9419	1.5	0.1851	1.2	0.83	1094.6	12.2	1095.7	9.8	1097.9	16.5	1097.9	16.5
354U1451 B41R1W #33 -Spot 25	482	115162	7.4	13.1291	0.9	1.8966	1.5	0.1807	1.2	0.81	1070.6	12.1	1079.9	10.1	1098.6	18.0	1098.6	18.0
354U1451 B41R1W #33 -Spot 45	1058	409820	15.5	13.1157	0.9	1.9048	1.5	0.1813	1.2	0.81	1073.9	11.6	1082.8	9.7	1100.6	17.3	1100.6	17.3
354U1451 B41R1W #33 -Spot 257	599	157553	2.1	13.1111	0.5	1.8758	1.1	0.1784	0.9	0.87	1058.5	9.2	1072.6	7.2	1101.3	10.8	1101.3	10.8
354U1451 B41R1W #33 -Spot 225	191	60913	1.0	13.0911	0.9	2.0102	1.7	0.1909	1.4	0.84	1126.5	14.8	1119.0	11.6	1104.4	18.7	1104.4	18.7
354U1451 B41R1W #33 -Spot 304	249	77060	7.4	13.0849	0.7	1.9967	1.3	0.1896	1.1	0.87	1119.0	11.7	1114.4	8.9	1105.3	13.1	1105.3	13.1
354U1451 B41R1W #33 -Spot 270	773	364717	3.7	13.0433	0.7	1.8372	1.4	0.1739	1.2	0.87	1033.4	11.8	1058.8	9.3	1111.7	13.7	1111.7	13.7
354U1451 B41R1W #33 -Spot 290	51	51503	0.8	13.0382	1.0	1.9763	2.5	0.1870	2.3	0.91	1104.9	23.5	1107.5	17.1	1112.4	20.6	1112.4	20.6
354U1451 B41R1W #33 -Spot 264	350	447339	1.2	13.0144	0.6	1.9237	1.3	0.1817	1.1	0.88	1076.0	11.2	1089.4	8.6	1116.1	12.1	1116.1	12.1
354U1451 B41R1W #33 -Spot 274	41	6626	1.2	13.0066	1.2	1.9666	1.8	0.1856	1.3	0.75	1097.5	13.6	1104.1	12.1	1117.3	24.0	1117.3	24.0
354U1451 B41R1W #33 -Spot 86	171	51728	1.9	12.9911	1.0	1.9434	1.9	0.1832	1.6	0.84	1084.4	15.9	1096.2	12.7	1119.7	20.2	1119.7	20.2
354U1451 B41R1W #33 -Spot 259	1917	7356299	5.9	12.9821	0.8	1.9514	2.0	0.1838	1.8	0.90	1087.8	17.8	1098.9	13.2	1121.1	16.8	1121.1	16.8
354U1451 B41R1W #33 -Spot 234	964	425695	46.3	12.9524	1.2	1.7519	1.7	0.1646	1.1	0.69	982.6	10.4	1027.9	10.7	1125.7	23.8	1125.7	23.8
354U1451 B41R1W #33 -Spot 201	353	592599	1.8	12.8436	0.9	2.0893	1.8	0.1947	1.5	0.87	1146.8	16.0	1145.3	12.1	1142.4	17.6	1142.4	17.6
354U1451 B41R1W #33 -Spot 145	1159	671488	22.4	12.8374	0.6	2.0974	1.5	0.1954	1.3	0.91	1150.4	14.2	1148.0	10.2	1143.4	12.2	1143.4	12.2
354U1451 B41R1W #33 -Spot 52	250	823990	1.5	12.8139	0.7	2.1742	1.3	0.2021	1.1	0.85	1186.8	11.9	1172.8	9.0	1147.0	13.6	1147.0	13.6
354U1451 B41R1W #33 -Spot 124	319	358000	2.2	12.7905	0.7	2.1264	1.7	0.1973	1.5	0.90	1161.0	15.8	1157.4	11.4	1150.6	14.6	1150.6	14.6
354U1451 B41R1W #33 -Spot 27	145	77375	0.6	12.7799	0.7	2.1365	1.6	0.1981	1.4	0.91	1165.2	15.4	1160.7	11.0	1152.3	13.0	1152.3	13.0
354U1451 B41R1W #33 -Spot 179	201	272144	1.0	12.7598	0.9	2.0834	1.7	0.1929	1.5	0.86	1137.0	15.7	1143.4	12.0	1155.4	17.6	1155.4	17.6
354U1451 B41R1W #33 -Spot 129	197	52848	3.1	12.7595	0.8	2.1098	1.7	0.1953	1.5	0.88	1150.2	15.8	1152.0	11.8	1155.5	16.4	1155.5	16.4
354U1451 B41R1W #33 -Spot 152	293	139827	1.8	12.7577	0.6	2.0461	1.4	0.1894	1.3	0.91	1118.1	13.1	1131.0	9.5	1155.7	11.2	1155.7	11.2
354U1451 B41R1W #33 -Spot 284	897	846436	5.7	12.7243	0.8	2.0734	1.6	0.1914	1.4	0.87	1129.1	14.6	1140.0	11.1	1160.9	15.9	1160.9	15.9
354U1451 B41R1W #33 -Spot 237	199	92749	1.4	12.7053	0.8	2.0854	1.3	0.1922	1.1	0.80	1133.5	11.0	1144.0	9.1	1163.9	15.9	1163.9	15.9
354U1451 B41R1W #33 -Spot 58	842	561807	3.2	12.6518	0.8	1.9856	1.7	0.1823	1.5	0.88	1079.4	15.0	1110.6	11.6	1172.3	16.3	1172.3	16.3
354U1451 B41R1W #33 -Spot 38	402	28778	2.1	12.6394	1.1	1.7313	1.7	0.1588	1.2	0.73	950.0	10.7	1020.2	10.7	1174.2	22.7	1174.2	22.7
354U1451 B41R1W #33 -Spot 309	391	563919	2.5	12.6228	0.6	2.1683	1.2	0.1986	1.0	0.85	1167.7	10.5	1170.9	8.1	1176.8	12.0	1176.8	12.0
354U1451 B41R1W #33 -Spot 135	78	31541	1.8	12.5515	1.6	2.1558	1.8	0.1963	0.9	0.51	1155.6	9.8	1166.9	12.6	1188.0	30.8	1188.0	30.8
354U1451 B41R1W #33 -Spot 144	611	244745	3.2	12.5135	0.8	2.0304	1.6	0.1843	1.5	0.89	1090.7	14.7	1125.7	11.2	1194.0	15.0	1194.0	15.0
354U1451 B41R1W #33 -Spot 67	252	117691	2.4	12.3032	0.8	2.1166	1.7	0.1889	1.5	0.87	1115.7	14.9	1154.2	11.6	1227.4	16.5	1227.4	16.5
354U1451 B41R1W #33 -Spot 173	220	564424	3.3	12.2812	0.8	2.2605	1.5	0.2014	1.3	0.84	1183.0	13.6	1200.0	10.6	1230.9	16.1	1230.9	16.1
354U1451 B41R1W #33 -Spot 146	307	104009	1.0	12.2324	0.9	2.2785	1.6	0.2022	1.3	0.82	1187.3	14.6	1205.7	11.6	1238.7	18.3	1238.7	18.3
354U1451 B41R1W #33 -Spot 206	178	186630	1.6	12.1574	0.8	2.3509	1.4	0.2074	1.2	0.83	1214.8	12.7	1227.8	9.9	1250.7	15.3	1250.7	15.3
354U1451 B41R1W #33 -Spot 239	212	28209	2.5	12.1184	0.9	2.1545	1.5	0.1894	1.3	0.82	1118.3	13.0	1166.5	10.7	1257.0	17.4	1257.0	17.4
354U1451 B41R1W #33 -Spot 29	164	153365	1.5	11.9478	0.6	2.5385	1.3	0.2201	1.2	0.87	1282.2	13.4	1283.1	9.6	1284.7	12.5	1284.7	12.5
354U1451 B41R1W #33 -Spot 96	345	86675	2.4	11.9334	0.7	2.5391	1.4	0.2199	1.2	0.85	1281.1	13.6	1283.3	10.0	1287.0	14.0	1287.0	14.0
354U1451 B41R1W #33 -Spot 187	804	199132	1.6	11.9182	0.7	2.5142	1.5	0.2174	1.3	0.88	1268.3	14.8	1276.2	10.6	1289.5	13.6	1289.5	13.6
354U1451 B41R1W #33 -Spot 126	491	423501	2.9	11.9074	0.8	2.3690	1.7	0.2047	1.5	0.87	1200.4	16.0	1233.3	12.0	1291.3	16.0	1291.3	16.0
354U1451 B41R1W #33 -Spot 252	872	178843	1.9	11.8703	0.7	2.3842	3.0	0.2054	2.9	0.97	1204.0	32.1	1237.9	21.5	1297.3	13.2	1297.3	13.2
354U1451 B41R1W #33 -Spot 39	192	56832	1.8	11.7956	0.9	2.4923	1.9	0.2133	1.6	0.86	1246.4	18.2	1269.8	13.5	1309.6	18.4	1309.6	18.4
354U1451 B41R1W #33 -Spot 199	474	198353	2.1	11.6536	0.8	2.7719	1.3	0.2344	1.0	0.80	1357.4	12.4	1348.0	9.4	1333.1	14.6	1333.1	14.6
354U1451 B41R1W #33 -Spot 109	244	40893	4.7	11.5655	0.9	2.4721	1.5	0.2075	1.2	0.81	1215.2	13.1	1263.9	10.5	1347.7	16.5	1347.7	16.5
354U1451 B41R1W #33 -Spot 204	116	34063	1.4	11.4197	0.8	2.9207	1.3	0.2420	1.0	0.78	1397.1	13.1	1387.3	10.1	1372.2	16.2	1372.2	16.2
354U1451 B41R1W #33 -Spot 55	230	536458	1.3	11.0744	0.8	3.0705	2.1	0.2467	1.9	0.91	1421.6	23.9	1425.4	15.7	1431.0	16.0	1431.0	16.0
354U1451 B41R1W #33 -Spot 134	619	71015	2.8	10.9853	0.9	3.1474	1.9	0.2509	1.7	0.88	1443.0	21.6	1444.4	14.6	1446.4	17.0	1446.4	17.0
354U1451 B41R1W #33 -Spot 80	358	173891	0.7	10.9844	0.7	3.1324	1.6	0.2497	1.5	0.90	1436.7	19.1	1440.7	12.6	1446.6	13.5	1446.6	13.5
354U1451 B41R1W #33 -Spot 18	521	854831	1.3	10.9818	0.8	2.8876	1.4	0.2301	1.2	0.82	1335.0	14.1	1378.7	10.8	1447.0	15.5	1447.0	15.5
354U1451 B41R1W #33 -Spot 22	316	91317	1.6	10.9148	0.8	3.2191	1.6	0.2549	1.4	0.85	1463.9	17.8	1461.8	12.4	1458.6	15.9	1458.6	15.9
354U1451 B41R1W #33 -Spot 59	519	1870243	4.0	10.6355	0.8	3.1085	1.6	0.2399	1.3	0.86	1386.1	16.6	1434.8	11.9	1507.8	15.1	1507.8	15.1
354U1451 B41R1W #33 -Spot 91	334	51863	2.3	10.4166	0.7	3.1575	1.4	0.2386	1.2	0.86	1379.7	15.0	1446.8	10.8	1547.0	13.2	1547.0	13.2
354U1451 B41R1W #33 -Spot 286	81	54760	0.9	10.3640	0.6	3.4844	1.5	0.2620	1.4	0.91	1500.2	18.2	1523.7	11.8	1556.5	11.5	1556.5	11.5
354U1451 B41R1W #33 -Spot 6	229	126544	1.4	10.1474	0.8	3.7092	1.6	0.2731	1.4	0.88	1556.5	19.9	1573.4	13.1	1596.0	14.6	1596.0	14.6
354U1451 B41R1W #33 -Spot 51	231	131970	1.9	10.1219	0.7	3.6190	1.5	0.2658	1.3	0.89	1519.4	17.7	1553.7	11.6	1600.7	12.4	1600.7	12.4
354U1451 B41R1W #33 -Spot 178	116	33309	2.1	10.1009	1.7	3.5975	2.2	0.2637	1.4	0.62	1508.5	18.2	1549.0	17.4	1604.6	32.1	1604.6	32.1
354U1451 B41R1W #33 -Spot 197	527	141170	1.8	10.0851	0.7	3.6368	1.3	0.2661	1.1	0.86	1521.1	15.5	1557.6	10.7	1607.5	12.9	1607.5	12.9
354U1451 B41R1W #33 -Spot 273	116	66987	0.7	10.0147	0.7	3.7167	1.6	0.2701	1.4	0.89	1541.2	19.5	1575.0	12.7	1620.5	13.2	1620.5	13.2
354U1451 B41R1W #33 -Spot 181	296	90684	1.7	10.0091	0.9	3.4533	1.9	0.2508	1.7	0.89	1442.6	22.4	1516.6	15.2	1621.6	16.1	1621.6	16.1
354U1451 B41R1W #33 -Spot 148	230	6385572	2.6	10.0052	0.6	4.0519	1.9	0.2942	1.8	0.95	1662.2	26.4	1644.7	15.4	1622.3	10.8	1622.3	10.8
354U1451 B41R1W #33 -Spot 47	242	10751114	1.3	9.9904	0.8	3.9107	1.6	0.2835	1.3	0.86	1608.9	19.1	1615.9	12.6	1625.0	14.6	1625.0	14.6
354U1451 B41R1W #33 -Spot 78	913	696886	7.4	9.7961	0.6	3.7613	1.5	0.2674	1.4	0.91	1527.3	19.1	1584.5	12.4	1661.5	11.9	1661.5	11.9
354U1451 B41R1W #33 -Spot 301	2925	709309	1.9	9.7945	0.6	3.7335	1.3	0.2653	1.1	0.86	1517.0	14.6	1578.6	10.1	1661.8	12.0	1661.8	12.0
354U1451 B41R1W #33 -Spot 211	132	184956	1.1	9.7498	0.8	4.2560	1.4	0.3011	1.1	0.82	1696.7	17.1	1684.9	11.5	1670.3	14.6	1670.3	14.6
354U1451 B41R1W #33 -Spot 122	272	114328	1.1	9.6405	0.8	4.3121	1.7	0.3016	1.6	0.89	1699.4	23.3	1695.7	14.4	1691.1	14.5	1691.1	14.5
354U1451 B41R1W #33 -Spot 112	265	138671	2.7	9.6357	0.7	4.4815	1.2	0.3133	0.9	0.77	1757.0	13.9	1727.5	9.7	1692.0	13.7	1692.0	13.7
354U1451 B41R1W #33 -Spot 244	613	2671535	2.3	9.5677	0.8	4.3566	1.3	0.3024	1.1	0.81	1703.4	16.1	1704.1	10.9	1705.0	14.2	1705.0	14.2
354U1451 B41R1W #33 -Spot 315	155	91726	1.9	9.5072	0.6	4.3543	1.4	0.3004	1.3	0.91	1693.2	18.9	1703.7	11.6	1716.7	10.9	1716.7	10.9
354U1451 B41R1W #33 -Spot 15	754	94601	1.4	9.5022	0.7	4.0000	1.4	0.2758	1.2	0.86	1570.1	16.3	1634.2	11.0	1717.7	12.5	1717.7	12.5

354U1451 B41R1W #33 -Spot 295	547	349171	0.7	9.4981	0.7	4.3603	1.3	0.3005	1.0	0.82	1693.8	15.3	1704.8	10.4	1718.5	13.2	1718.5	13.2
354U1451 B41R1W #33 -Spot 214	404	207771	14.2	9.4797	0.8	4.3041	1.6	0.2961	1.4	0.87	1671.7	20.8	1694.2	13.4	1722.0	14.6	1722.0	14.6
354U1451 B41R1W #33 -Spot 177	566	72185	4.1	9.4771	0.9	4.0880	1.9	0.2811	1.6	0.87	1597.0	22.9	1651.9	15.2	1722.5	17.1	1722.5	17.1
354U1451 B41R1W #33 -Spot 194	245	127797	1.9	9.4729	0.6	4.2172	1.3	0.2899	1.1	0.90	1640.9	16.6	1677.4	10.5	1723.4	10.4	1723.4	10.4
354U1451 B41R1W #33 -Spot 140	797	212521	34.7	9.4648	0.7	4.2011	1.3	0.2885	1.1	0.86	1634.1	16.4	1674.2	10.8	1724.9	12.2	1724.9	12.2
354U1451 B41R1W #33 -Spot 210	162	81980	2.6	9.4508	0.7	4.4949	1.4	0.3082	1.2	0.85	1732.0	17.6	1730.0	11.3	1727.6	13.0	1727.6	13.0
354U1451 B41R1W #33 -Spot 150	206	196625	2.4	9.3936	0.7	4.5883	1.2	0.3127	1.1	0.85	1754.1	16.3	1747.1	10.4	1738.8	12.0	1738.8	12.0
354U1451 B41R1W #33 -Spot 269	255	177276	4.8	9.3896	0.8	4.4874	1.6	0.3057	1.4	0.87	1719.6	21.5	1728.6	13.5	1739.5	14.4	1739.5	14.4
354U1451 B41R1W #33 -Spot 14	176	526587	1.9	9.3820	0.7	4.3580	1.6	0.2967	1.4	0.89	1674.8	20.4	1704.4	12.8	1741.0	12.9	1741.0	12.9
354U1451 B41R1W #33 -Spot 291	191	153427	1.7	9.3738	0.8	4.4558	1.2	0.3031	1.0	0.80	1706.5	14.9	1722.8	10.3	1742.6	13.8	1742.6	13.8
354U1451 B41R1W #33 -Spot 289	285	62862	3.0	9.3685	0.7	3.6701	1.8	0.2495	1.7	0.92	1435.8	21.7	1564.9	14.7	1743.7	13.5	1743.7	13.5
354U1451 B41R1W #33 -Spot 142	128	571833	1.6	9.3260	0.7	4.6554	1.3	0.3150	1.1	0.82	1765.4	16.4	1759.3	10.8	1752.0	13.4	1752.0	13.4
354U1451 B41R1W #33 -Spot 133	219	111270	2.5	9.2743	0.7	4.8203	1.4	0.3244	1.2	0.85	1811.0	18.6	1788.4	11.6	1762.2	13.3	1762.2	13.3
354U1451 B41R1W #33 -Spot 166	301	128608	1.8	9.2653	0.5	4.6969	1.1	0.3158	1.0	0.88	1769.0	15.5	1766.7	9.5	1763.9	9.7	1763.9	9.7
354U1451 B41R1W #33 -Spot 161	161	614947	1.3	9.2117	0.8	4.5971	1.4	0.3073	1.2	0.82	1727.2	17.7	1748.7	11.9	1774.5	14.9	1774.5	14.9
354U1451 B41R1W #33 -Spot 89	209	122251	3.1	9.1910	0.8	4.5324	1.6	0.3023	1.5	0.89	1702.5	21.8	1736.9	13.6	1778.6	13.7	1778.6	13.7
354U1451 B41R1W #33 -Spot 93	106	11964	1.0	9.1222	1.1	4.1619	3.1	0.2755	2.9	0.94	1568.5	39.8	1666.6	25.0	1792.3	19.4	1792.3	19.4
354U1451 B41R1W #33 -Spot 79	351	65527	2.0	9.0911	0.7	4.6081	1.7	0.3040	1.6	0.92	1711.0	24.1	1750.7	14.5	1798.6	12.5	1798.6	12.5
354U1451 B41R1W #33 -Spot 160	873	526268	18.0	9.0440	0.6	4.9949	1.5	0.3278	1.3	0.91	1827.6	21.2	1818.5	12.4	1808.0	11.0	1808.0	11.0
354U1451 B41R1W #33 -Spot 104	97	46318	1.0	9.0015	0.7	4.8692	1.5	0.3180	1.3	0.88	1780.1	20.1	1796.9	12.4	1816.6	12.6	1816.6	12.6
354U1451 B41R1W #33 -Spot 243	264	94399	1.9	8.9286	0.6	4.6167	1.4	0.2991	1.2	0.89	1686.8	18.2	1752.3	11.5	1831.3	11.2	1831.3	11.2
354U1451 B41R1W #33 -Spot 37	140	118951	1.8	8.9208	0.8	5.1526	1.3	0.3335	1.0	0.77	1855.4	16.2	1844.8	11.1	1832.9	15.1	1832.9	15.1
354U1451 B41R1W #33 -Spot 31	548	1220624	8.3	8.8832	0.7	4.9486	1.3	0.3190	1.0	0.82	1784.6	16.4	1810.6	10.8	1840.5	13.3	1840.5	13.3
354U1451 B41R1W #33 -Spot 107	312	424620	2.6	8.8684	0.7	5.2854	1.5	0.3401	1.3	0.88	1887.2	21.3	1866.5	12.7	1843.6	12.9	1843.6	12.9
354U1451 B41R1W #33 -Spot 11	494	422000	2.1	8.8365	0.7	5.0268	1.5	0.3223	1.3	0.90	1800.9	21.2	1823.8	12.7	1850.1	11.9	1850.1	11.9
354U1451 B41R1W #33 -Spot 306	820	449381	5.1	8.8102	0.6	4.9661	1.4	0.3175	1.2	0.89	1777.3	18.7	1813.6	11.4	1855.5	11.1	1855.5	11.1
354U1451 B41R1W #33 -Spot 120	220	90872	3.4	8.7980	0.8	5.1227	1.5	0.3270	1.2	0.82	1823.9	19.2	1839.9	12.5	1858.0	15.2	1858.0	15.2
354U1451 B41R1W #33 -Spot 200	260	131019	6.3	8.7765	0.6	4.9236	1.3	0.3135	1.1	0.88	1758.1	17.6	1806.3	11.0	1862.4	11.3	1862.4	11.3
354U1451 B41R1W #33 -Spot 57	367	135259	2.3	8.7730	0.7	4.8416	2.1	0.3082	1.9	0.94	1731.8	29.6	1792.2	17.5	1863.1	12.8	1863.1	12.8
354U1451 B41R1W #33 -Spot 115	190	229735	2.1	8.6678	0.6	5.3719	1.2	0.3379	1.1	0.86	1876.3	17.5	1880.4	10.7	1884.9	11.4	1884.9	11.4
354U1451 B41R1W #33 -Spot 16	332	572600	2.8	8.6359	0.9	5.3472	1.8	0.3351	1.6	0.87	1862.9	25.4	1876.4	15.4	1891.5	15.9	1891.5	15.9
354U1451 B41R1W #33 -Spot 311	143	92998	0.9	8.2875	0.6	5.7657	1.3	0.3467	1.2	0.91	1918.9	19.9	1941.3	11.4	1965.3	9.8	1965.3	9.8
354U1451 B41R1W #33 -Spot 265	547	34761	2.5	8.1931	0.8	5.5880	1.4	0.3322	1.2	0.85	1849.0	19.6	1914.2	12.4	1985.7	13.6	1985.7	13.6
354U1451 B41R1W #33 -Spot 175	135	643111	1.3	8.1468	0.7	5.8984	1.4	0.3487	1.2	0.87	1928.2	19.8	1961.0	11.9	1995.8	12.0	1995.8	12.0
354U1451 B41R1W #33 -Spot 143	40	43355	1.5	8.0434	0.8	6.3420	1.7	0.3701	1.5	0.89	2030.0	26.1	2024.3	14.7	2018.4	13.6	2018.4	13.6
354U1451 B41R1W #33 -Spot 202	320	446049	3.1	8.0258	0.8	5.9762	1.6	0.3480	1.4	0.88	1925.1	23.0	1972.4	13.7	2022.3	13.5	2022.3	13.5
354U1451 B41R1W #33 -Spot 108	370	4114	2.1	7.9328	4.4	5.2380	4.8	0.3015	2.0	0.42	1698.7	30.4	1858.8	41.1	2042.9	77.3	2042.9	77.3
354U1451 B41R1W #33 -Spot 157	1026	479455	5.9	7.7256	0.9	6.7537	1.4	0.3786	1.1	0.77	2069.6	19.5	2079.7	12.7	2089.6	16.1	2089.6	16.1
354U1451 B41R1W #33 -Spot 21	485	1275432	1.3	7.6578	0.6	6.8363	1.3	0.3799	1.2	0.88	2075.6	20.4	2090.4	11.6	2105.1	10.8	2105.1	10.8
354U1451 B41R1W #33 -Spot 297	856	247105	19.9	7.1585	0.8	8.2486	1.3	0.4284	1.0	0.79	2298.7	19.8	2258.7	11.8	2222.7	13.9	2222.7	13.9
354U1451 B41R1W #33 -Spot 77	817	173830	4.1	7.0362	0.8	7.8216	1.5	0.3993	1.3	0.86	2165.9	23.1	2210.7	13.2	2252.5	13.2	2252.5	13.2
354U1451 B41R1W #33 -Spot 36	581	512311	15.5	6.6950	0.6	8.1304	1.3	0.3950	1.1	0.87	2145.8	19.9	2245.6	11.4	2337.9	10.8	2337.9	10.8
354U1451 B41R1W #33 -Spot 312	2643	727264	1.7	6.6825	0.6	8.8974	1.2	0.4314	1.1	0.88	2312.1	20.9	2327.5	11.1	2341.1	9.9	2341.1	9.9
354U1451 B41R1W #33 -Spot 106	185	75327	2.2	6.6541	0.7	8.7525	1.4	0.4226	1.3	0.89	2272.2	24.5	2312.6	13.1	2348.4	11.2	2348.4	11.2
354U1451 B41R1W #33 -Spot 83	639	263503	3.4	6.5249	0.7	8.9965	1.2	0.4259	1.0	0.82	2287.3	18.9	2337.7	11.0	2381.8	11.7	2381.8	11.7
354U1451 B41R1W #33 -Spot 267	295	188286	3.4	6.3645	0.7	9.1031	2.3	0.4204	2.2	0.95	2262.2	41.6	2348.4	20.9	2424.2	11.6	2424.2	11.6
354U1451 B41R1W #33 -Spot 154	633	2335058	1.7	6.3627	0.7	9.8513	1.6	0.4548	1.4	0.89	2416.6	28.9	2421.0	14.9	2424.6	12.5	2424.6	12.5
354U1451 B41R1W #33 -Spot 71	517	33892945	9.8	6.3350	0.7	9.8422	1.3	0.4524	1.1	0.84	2405.9	21.3	2420.1	11.7	2432.1	11.8	2432.1	11.8
354U1451 B41R1W #33 -Spot 9	66	60762	1.3	6.2946	0.8	10.2953	1.5	0.4702	1.3	0.87	2484.5	27.1	2461.7	14.0	2442.9	12.8	2442.9	12.8
354U1451 B41R1W #33 -Spot 279	442	881151	2.5	6.2760	0.7	9.6211	1.7	0.4381	1.6	0.90	2342.2	30.7	2399.2	15.9	2447.9	12.6	2447.9	12.6
354U1451 B41R1W #33 -Spot 100	151	168560	1.0	6.2718	0.8	9.8123	1.8	0.4465	1.6	0.90	2379.8	31.6	2417.3	16.2	2449.0	12.8	2449.0	12.8
354U1451 B41R1W #33 -Spot 258	1002	213794	3.3	6.2700	1.0	8.2003	1.8	0.3731	1.5	0.83	2043.8	26.0	2253.4	16.1	2449.5	16.7	2449.5	16.7
354U1451 B41R1W #33 -Spot 147	263	270821	3.5	6.2619	0.7	10.1948	1.7	0.4632	1.5	0.90	2453.7	31.4	2452.6	15.7	2451.7	12.3	2451.7	12.3
354U1451 B41R1W #33 -Spot 19	143	507046	1.4	6.2518	0.9	10.1904	1.8	0.4623	1.6	0.88	2449.5	32.1	2452.2	16.6	2454.4	14.4	2454.4	14.4
354U1451 B41R1W #33 -Spot 94	104	86979	0.8	6.2445	0.7	10.1125	1.5	0.4582	1.3	0.89	2431.5	26.5	2445.1	13.6	2456.4	11.4	2456.4	11.4
354U1451 B41R1W #33 -Spot 305	415	227839	2.7	6.2347	0.7	9.9361	1.5	0.4495	1.4	0.90	2393.0	27.3	2428.9	14.0	2459.1	11.2	2459.1	11.2
354U1451 B41R1W #33 -Spot 186	316	288228	1.8	6.2328	0.7	9.5416	1.6	0.4315	1.5	0.91	2312.5	28.5	2391.6	14.9	2459.6	11.5	2459.6	11.5
354U1451 B41R1W #33 -Spot 260	279	159698	1.0	6.2179	0.6	10.2070	1.3	0.4605	1.1	0.88	2441.8	22.4	2453.7	11.6	2463.6	10.1	2463.6	10.1
354U1451 B41R1W #33 -Spot 62	279	1707163	1.9	6.1713	0.6	10.5310	1.7	0.4716	1.6	0.94	2490.4	32.3	2482.7	15.4	2476.3	9.7	2476.3	9.7
354U1451 B41R1W #33 -Spot 227	34	51753	3.3	6.1467	0.7	10.1399	1.3	0.4522	1.1	0.84	2405.2	22.6	2447.6	12.4	2483.1	12.4	2483.1	12.4
354U1451 B41R1W #33 -Spot 283	168	111903	1.2	6.1353	0.8	10.0590	1.6	0.4478	1.4	0.88	2385.4	28.2	2440.2	14.9	2486.2	13.1	2486.2	13.1
354U1451 B41R1W #33 -Spot 192	506	581129	6.4	6.0161	0.8	10.7051	2.1	0.4673	1.9	0.92	2471.7	39.6	2497.9	19.6	2519.2	14.2	2519.2	14.2
354U1451 B41R1W #33 -Spot 209	54	28377	2.4	6.0045	1.0	10.8619	1.8	0.4732	1.5	0.83	2497.7	30.2	2511.4	16.3	2522.4	16.2	2522.4	16.2
354U1451 B41R1W #33 -Spot 113	59	111385	1.3	5.8800	0.8	11.2442	1.5	0.4797	1.3	0.84	2526.1	26.6	2543.6	14.1	2557.6	13.7	2557.6	13.7
354U1451 B41R1W #33 -Spot 292	259	113915	4.4	5.8029	0.7	11.6902	1.6	0.4922	1.5	0.91	2580.3	31.6	2579.9	15.3	2579.6	11.4	2579.6	11.4
354U1451 B41R1W #33 -Spot 220	815	552602	3.5	5.6705	0.7	12.7452	1.3	0.5244	1.1	0.83	2717.8	23.6	2661.0	12.0	2618.1	11.8	2618.1	11.8
354U1451 B41R1W #33 -Spot 235	338	541054	2.8	5.6103	0.6	10.7944	1.3	0.4394	1.2	0.90	2348.0	23.6	2505.6	12.4	2635.9	9.7	2635.9	9.7
354U1451 B41R1W #33 -Spot 123	113	37129	2.5	5.5087	0.8	12.3084	1.6	0.4920	1.4	0.88	2579.2	29.8	2628.2	15.0	2666.2	12.7	2666.2	12.7
354U1451 B41R1W #33 -Spot 296	106	181310	2.5	5.3114	0.8	10.8973	1.7	0.4200	1.4	0.87	2260.3	27.5	2514.4	15.4	2726.4	13.3	2726.4	13.3
354U1451 B41R1W #33 -Spot 190	533	72056	1.7	5.2837	0.8	11.7659	2.1	0.4511	2.0	0.93	2400.0	39.6	2586.0	19.9	2735.0	12.8	2735.0	12.8
354U1451 B41R1W #33 -Spot 87	69	80072	2.1	5.0601	0.9	14.3946	1.5	0.5285	1.2	0.80	2735.1	27.0	2776.1	14.4	2806.0	14.7	2806.0	14.7
354U1451 B41R1W #33 -Spot 66	102	12743653	1.5	5.0418	0.8	14.5032	1.3	0.5306	1.1	0.79	2743.8	23.5	2783.2	12.7	2811.9	13.4	2811.9	13.4
354U1451 B41R1W #33 -Spot 61	68	553679	2.1	4.9424	0.8	15.2433	1.8	0.5466	1.7	0.91	2811.2	37.9	2830.6	17.4	2844.4	12.3	2844.4	12.3
354U1451 B41R1W #33 -Spot 222	109	164342	1.8	4.6951	0.6	15.6701	1.2	0.5338	1.1	0.89	2757.6	24.0	2856.9	11.5	2927.7	9.0	2927.7	9.0
354U1451 B41R1W #33 -Spot 50	210	937	1.3	4.3658	4.0	15.6758	5.5	0.4966	3.7	0.67	2599.1	78.6	2857.2	52.1	3044.7	64.6	3044.7	64.6
354U1451 B41R1W #33 -Spot 34	206	196098	1.9	4.0164	0.7	21.2509	1.4	0.6193	1.2	0.88	3107.2	29.9	3150.1	13.4	3177.6	10.5	3177.6	10.5
354U1451 B41R1W #33 -Spot 282	1318	116464	6.1	3.6683	1.7	22.2613	2.1	0.5925	1.1	0.54	2999.7	26.6	3195.2	20.0	3320.4	27.1	3320.4	27.1

Table _____. U-Pb geochronologic analyses.																		
#24 U1451B_51_54R						Isotope ratios						Apparent ages (Ma)						
Early Miocene 16 Ma																		
Analysis	U (ppm)	206Pb 204Pb	U/Th	206Pb* 207Pb*	± (%)	207Pb* 235U*	± (%)	206Pb* 238U	± (%)	error corr.	206Pb* 238U*	± (Ma)	207Pb* 235U	± (Ma)	206Pb* 207Pb*	± (Ma)	Best age (Ma)	± (Ma)
-3541451B51R2 Spot 209	96	807	161002.2	21.3403	12.9	0.0253	13.9	0.0039	5.1	0.37	25.2	1.3	25.4	3.5	41.1	309.2	25.2	1.3
-3541451B51R2 Spot 124	147	8534	0.7	19.6511	4.1	0.1308	4.5	0.0187	1.9	0.41	119.1	2.2	124.8	5.3	234.8	95.6	119.1	2.2
-3541451B51R2 Spot 62	55	1046	1.3	18.9646	15.7	0.1509	15.8	0.0208	2.3	0.14	132.5	3.0	142.7	21.1	316.2	357.9	132.5	3.0
-3541451B51R2 Spot 215	57	1130	1.4	30.7759	8.3	0.0938	8.6	0.0209	2.1	0.24	133.6	2.8	91.0	7.5	NA	NA	133.6	2.8
-3541451B51R2 Spot 162	32	893	4.3	2.6679	2.5	2.1263	4.5	0.0412	3.8	0.83	260.0	9.6	1157.4	31.3	3810.4	38.0	260.0	9.6
-3541451B51R2 Spot 165	20000	572836	10.0	17.1432	0.8	0.5111	2.1	0.0636	1.9	0.92	397.4	7.3	419.2	7.1	541.3	17.5	397.4	7.3
-3541451B51R2 Spot 201	13216	385309	4.2	17.5076	0.8	0.5171	1.5	0.0657	1.3	0.84	410.2	5.1	423.2	5.3	495.2	18.3	410.2	5.1
-3541451B51R2 Spot 11	1446	53695	2.2	17.3398	0.8	0.6044	1.3	0.0760	1.0	0.77	472.5	4.7	480.0	5.1	516.3	18.4	472.5	4.7
-3541451B51R2 Spot 127	695	56223	1.1	17.2440	1.0	0.6351	1.4	0.0795	1.0	0.72	492.9	4.9	499.3	5.6	528.5	21.7	492.9	4.9
-3541451B51R2 Spot 294	6082	4738653	36.7	17.1795	0.8	0.6387	1.5	0.0796	1.3	0.87	493.8	6.3	501.5	6.0	536.7	16.4	493.8	6.3
-3541451B51R2 Spot 290	1902	996598	1.4	17.4456	0.9	0.6349	1.5	0.0804	1.2	0.79	498.3	5.7	499.1	6.0	503.0	20.6	498.3	5.7
-3541451B51R2 Spot 286	737	416769	0.6	17.3558	1.0	0.6386	1.5	0.0804	1.0	0.72	498.6	5.0	501.4	5.8	514.3	22.1	498.6	5.0
-3541451B51R2 Spot 119	4426	1368155	17.5	17.2204	0.8	0.6711	1.6	0.0838	1.3	0.86	519.0	6.7	521.4	6.4	531.5	17.7	519.0	6.7
-3541451B51R2 Spot 132	1995	2394490	7.0	17.1424	0.8	0.6759	1.5	0.0841	1.2	0.83	520.4	6.1	524.3	6.0	541.4	17.8	520.4	6.1
-3541451B51R2 Spot 0	280	14814	0.6	17.1226	1.4	0.6788	2.1	0.0843	1.6	0.76	521.9	7.9	526.0	8.5	544.0	29.7	521.9	7.9
-3541451B51R2 Spot 190	754	70132	2.5	17.3393	1.0	0.6808	1.4	0.0856	1.0	0.72	529.7	5.3	527.2	6.0	516.4	22.1	529.7	5.3
-3541451B51R2 Spot 176	5950	184679	0.9	16.9583	0.6	0.7050	1.5	0.0867	1.3	0.91	536.3	6.9	541.8	6.2	564.9	13.6	536.3	6.9
-3541451B51R2 Spot 303	205	49359	10.9	16.6762	1.4	0.7256	1.7	0.0878	1.0	0.58	542.5	5.1	553.9	7.3	601.4	30.1	542.5	5.1
-3541451B51R2 Spot 37	400	187462	1.2	17.0238	1.1	0.7231	1.4	0.0893	0.9	0.64	551.5	4.7	552.5	5.9	556.6	23.4	551.5	4.7
-3541451B51R2 Spot 152	121	12547	1.2	16.4885	1.7	0.8337	2.0	0.0997	1.2	0.59	612.9	7.0	615.7	9.5	625.8	35.8	612.9	7.0
-3541451B51R2 Spot 72	2531	206769	12.3	16.3495	0.7	0.8664	1.2	0.1028	1.0	0.83	630.7	6.0	633.6	5.6	644.1	14.4	630.7	6.0
-3541451B51R2 Spot 51	432	52716	2.7	16.4775	1.1	0.8810	1.5	0.1053	1.0	0.66	645.6	6.1	641.5	7.2	627.3	24.3	645.6	6.1
-3541451B51R2 Spot 189	2835	831632	2.0	16.2186	0.9	0.8984	1.5	0.1057	1.1	0.76	647.9	6.9	650.9	7.0	661.3	20.1	647.9	6.9
-3541451B51R2 Spot 312	252	45717	1.0	15.9790	1.3	0.9724	1.9	0.1127	1.3	0.71	688.7	8.7	689.7	9.5	693.2	28.6	688.7	8.7
-3541451B51R2 Spot 42	1659	121013	22.8	15.6694	0.8	1.0092	1.4	0.1147	1.2	0.84	700.2	7.8	708.5	7.1	734.7	16.0	700.2	7.8
-3541451B51R2 Spot 17	184	35283	2.0	15.5591	1.0	1.0212	1.4	0.1153	1.0	0.68	703.4	6.5	714.5	7.4	749.6	22.2	703.4	6.5
-3541451B51R2 Spot 241	531	22756	1.2	15.7042	1.2	1.0426	1.9	0.1188	1.5	0.76	723.6	10.0	725.2	9.9	730.0	26.4	723.6	10.0
-3541451B51R2 Spot 154	579	111215	1.0	15.3219	0.8	1.0762	1.4	0.1196	1.2	0.83	728.5	8.1	741.8	7.5	782.0	16.6	728.5	8.1
-3541451B51R2 Spot 270	2234	161568	6.8	14.6375	0.9	1.1458	1.2	0.1217	0.8	0.68	740.3	5.8	775.3	6.6	877.3	18.4	740.3	5.8
-3541451B51R2 Spot 103	3022	158520	3.3	14.2850	0.7	1.2015	1.9	0.1245	1.8	0.93	756.6	12.9	801.3	10.7	927.6	14.5	756.6	12.9
-3541451B51R2 Spot 43	231	34778	1.0	15.3568	1.1	1.1179	1.6	0.1246	1.1	0.73	756.8	8.1	762.0	8.4	777.2	22.5	756.8	8.1
-3541451B51R2 Spot 56	425	32913	1.2	15.5328	1.1	1.1070	1.6	0.1248	1.1	0.71	757.9	8.0	756.7	8.4	753.2	23.4	757.9	8.0
-3541451B51R2 Spot 277	246	27022	1.1	15.4097	1.2	1.1275	1.6	0.1261	1.0	0.66	765.4	7.5	766.6	8.5	770.0	24.9	765.4	7.5
-3541451B51R2 Spot 253	387	35433	0.6	15.3386	0.9	1.1608	1.6	0.1292	1.4	0.84	783.3	10.2	782.3	9.0	779.7	19.0	783.3	10.2
-3541451B51R2 Spot 174	480	161749	0.9	15.1106	1.0	1.1861	1.6	0.1300	1.3	0.78	788.1	9.4	794.1	8.9	811.1	21.4	788.1	9.4
-3541451B51R2 Spot 107	1264	139212	2.0	15.2130	0.7	1.1927	1.1	0.1317	0.8	0.78	797.3	6.2	797.2	5.9	797.0	13.8	797.3	6.2
-3541451B51R2 Spot 117	1936	129987	9.0	13.9994	0.8	1.3043	5.2	0.1325	5.1	0.99	802.0	38.6	847.6	29.7	968.9	15.8	802.0	38.6
-3541451B51R2 Spot 254	1787	122991	2.5	15.0468	0.8	1.2149	1.3	0.1326	1.0	0.78	802.9	7.8	807.4	7.4	820.0	17.2	802.9	7.8
-3541451B51R2 Spot 221	451	56376	1.5	15.0574	0.9	1.2188	1.6	0.1332	1.3	0.82	805.8	9.9	809.2	8.9	818.5	19.2	805.8	9.9
-3541451B51R2 Spot 227	259	307653	1.1	14.9267	1.3	1.2381	1.7	0.1341	1.1	0.65	811.1	8.6	818.0	9.7	836.7	27.4	811.1	8.6
-3541451B51R2 Spot 222	3856	2853552	4.1	14.6073	0.9	1.2657	1.5	0.1341	1.2	0.80	811.5	9.2	830.5	8.6	881.6	19.0	811.5	9.2
-3541451B51R2 Spot 48	678	36264	2.9	14.5014	1.3	1.2858	1.9	0.1353	1.4	0.74	818.0	10.9	839.4	10.9	896.6	26.5	818.0	10.9
-3541451B51R2 Spot 167	402	56912	3.9	14.9586	0.9	1.2469	1.3	0.1353	0.9	0.70	818.3	6.8	822.0	7.1	832.2	18.6	818.3	6.8
-3541451B51R2 Spot 231	193	82258	0.8	14.6312	1.7	1.2752	2.1	0.1354	1.3	0.61	818.5	10.0	834.7	12.0	878.2	34.6	818.5	10.0
-3541451B51R2 Spot 57	162	271056	0.8	14.9283	1.2	1.2519	1.8	0.1356	1.3	0.71	819.8	9.6	824.3	10.0	836.5	25.9	819.8	9.6
-3541451B51R2 Spot 157	630	112547	2.1	15.0953	1.0	1.2422	1.5	0.1361	1.1	0.76	822.3	8.8	819.9	8.4	813.3	20.2	822.3	8.8
-3541451B51R2 Spot 108	870	115195	2.9	14.4349	1.1	1.3004	1.7	0.1362	1.3	0.76	823.1	9.9	845.9	9.7	906.1	22.8	823.1	9.9
-3541451B51R2 Spot 9	762	263873	1.3	14.6145	0.8	1.2881	1.4	0.1366	1.2	0.82	825.4	9.2	840.5	8.3	880.6	17.2	825.4	9.2
-3541451B51R2 Spot 49	320	14172	2.2	15.1670	0.8	1.2471	1.3	0.1372	1.1	0.82	829.1	8.4	822.1	7.4	803.3	15.9	829.1	8.4
-3541451B51R2 Spot 289	384	245168	23.4	14.7501	1.2	1.2824	1.7	0.1372	1.2	0.72	829.1	9.5	837.9	9.6	861.5	24.2	829.1	9.5
-3541451B51R2 Spot 22	367	24879	1.5	14.9180	1.2	1.2680	2.5	0.1373	2.2	0.88	829.1	17.1	831.5	14.2	837.9	24.9	829.1	17.1
-3541451B51R2 Spot 314	392	59060	1.0	14.7872	0.8	1.2827	1.4	0.1376	1.2	0.84	831.2	9.3	838.0	8.1	856.2	16.0	831.2	9.3
-3541451B51R2 Spot 163	747	110650	3.3	14.8475	1.0	1.3281	1.4	0.1431	1.0	0.72	862.0	8.1	858.1	8.1	847.7	20.2	862.0	8.1
-3541451B51R2 Spot 147	902	93408	2.8	14.7903	1.0	1.3419	1.5	0.1440	1.1	0.75	867.3	9.0	864.1	8.5	855.7	20.0	867.3	9.0
-3541451B51R2 Spot 299	614	1009097	2.4	14.6374	0.7	1.3582	1.4	0.1442	1.1	0.85	868.6	9.3	871.1	7.9	877.3	14.9	868.6	9.3
-3541451B51R2 Spot 268	273	3233307	1.9	14.4901	1.0	1.3911	1.5	0.1463	1.0	0.71	879.9	8.6	885.2	8.7	898.2	21.5	879.9	8.6
-3541451B51R2 Spot 159	1251	138968	1.6	14.8269	0.7	1.3617	1.4	0.1465	1.2	0.87	881.3	9.8	872.6	8.0	850.6	13.8	881.3	9.8
-3541451B51R2 Spot 285	497	344269	2.4	14.6527	0.8	1.3976	1.4	0.1486	1.1	0.83	893.0	9.3	887.9	8.0	875.1	15.8	893.0	9.3
-3541451B51R2 Spot 10	361	140796	2.1	14.4573	1.2	1.4211	1.6	0.1491	1.1	0.68	895.8	9.2	897.8	9.6	902.9	24.2	895.8	9.2
-3541451B51R2 Spot 54	233	37441	1.5	14.5543	1.2	1.4216	1.8	0.1501	1.3	0.76	901.7	11.3	898.0	10.6	889.1	23.8	901.7	11.3
-3541451B51R2 Spot 67	1177	147946	1.8	14.3331	0.9	1.4963	1.4	0.1556	1.1	0.79	932.4	9.6	928.9	8.5	920.7	17.7	920.7	17.7
-3541451B51R2 Spot 139	504	26725	1.4	14.2812	0.8	1.5433	1.3	0.1599	1.1	0.81	956.3	9.5	947.8	8.2	928.1	16.2	928.1	16.2
-3541451B51R2 Spot 181	685	34837	4.9	14.2742	0.9	1.5148	1.3	0.1569	1.0	0.76	939.5	9.0	936.4	8.2	929.1	17.9	929.1	17.9
-3541451B51R2 Spot 284	819	140928	3.3	14.2717	0.8	1.4654	1.3	0.1517	1.1	0.79	910.7	8.9	916.2	8.0	929.5	16.5	929.5	16.5
-3541451B51R2 Spot 246	532	93230	1.4	14.2572	0.9	1.4333	1.3	0.1483	1.0	0.73	891.2	8.1	902.9	7.9	931.6	18.5	931.6	18.5
-3541451B51R2 Spot 27	413	145016	1.6	14.2388	0.8	1.4801	1.9	0.1529	1.8	0.91	917.3	15.0	922.3	11.6	934.2	16.0	934.2	16.0
-3541451B51R2 Spot 55	1178	44959	2.6	14.1676	0.9	1.5009	1.3	0.1543	1.0	0.76	925.0	8.6	930.8	8.0	944.5	17.5	944.5	17.5
-3541451B51R2 Spot 66	1673	176991	0.9	14.1620	0.8	1.5752	1.5	0.1619	1.3	0.86	967.1	11.4	960.5	9.2	945.3	15.5	945.3	15.5
-3541451B51R2 Spot 23	1256	296402	1.2	14.1534	1.0	1.5120	1.7	0.1553	1.5	0.83	930.5	12.6	935.2	10.7	946.6	19.8	946.6	19.8
-3541451B51R2 Spot 113	1143	353289	5.1	14.0853	0.8	1.5271	1.3	0.1561	1.1	0.80	934.9	9.2	941.3	8.1	956.4	16.3	956.4	16.3
-3541451B51R2 Spot 19	670	101532	2.3	14.0451	1.0	1.5983	1.4	0.1629	1.0	0.71	972.8	8.8	969.5	8.5	962.3	19.5	962.3	19.5
-3541451B51R2 Spot 125	742	134948	2.2	14.0211	0.8	1.5675	1.5	0.1595	1.2	0.82	953.8	10.6	957.4	9.0	965.8	16.9	965.8	16.9
-3541451B51R2 Spot 213	1394	208203	10.9	14.0143	0.7	1.5453	1.7	0.1571	1.5	0.90	940.8	13.4	948.6	10.5	966.7	14.8	966.7	14.8
-3541451B51R2 Spot 207	738	37207	3.5	14.0088	1.0	1.6690	1.4	0.1697	1.0	0.71	1010.2	9.2	996.8	8.8	967.5	19.8	967.5	19.8
-3541451B51R2 Spot 278	1989	158602	1.5	13.9745	1.0	1.4098	2.3	0.1430	2.1	0.89	861.3	16.6	893.1	13.7	972.5	21.0	972.5	21.0
-3541451B51R2 Spot 84	1520	480047	1.8	13.9534	1.0	1.5280	1.6	0.1547	1.2	0.79	927.3	10.7	941.7	9.6	975.6	19.5	975.6	19.5
-3541451B51R2 Spot 29	1242	531572	1.8	13.9362	0.8	1.5643	1.3	0.1582	1.1	0.80	946.6	9.3	956.2	8.2	978.1	16.3	978.1	16.3
-3541451B51R2 Spot 243	692	124066	2.0	13.9166	0.9	1.5752	1.2	0.1591	0.8	0.63	951.5	6.7	960.5	7.4	981.0	19.0	981.0	19.0
-3541451B51R2 Spot 94	1643	387705	7.1	13.9054	0.6	1.6393	1.3	0.1654	1.1	0.87	986.7	10.1	985.5	8.1	982.6	13.0	982.6	13.0
-3541451B51R2 Spot 122	1045	527716	1.5	13.8643	0.9	1.5551	1.8	0.1564	1.5	0.86	937.0	13.2	952.5	10.9	988.7	18.1	988.7	18.1
-3541451B51R2 Spot 63	784	791180	82.9	13.8508	0.7	1.6676	1.3	0.1676	1.1	0.83	998.8	9.9	996.3	8.2	990.6	14.9	990.6	14.9
-3541451B51R2 Spot 77	849	255938	2.5	13.7943	0.7	1.6830	1.8	0.1685	1.6	0.93	1003.6	15.3	1002.1	11.3	998.9	13.4	998.9	13.4
-3541451B51R2 Spot 193	2018	2883641	4.3	13.7936	0.8	1.5790	1.7	0.1580	1.5	0.89	945.9	13.0	962.0	10.3	999.0	15.6	999.0	15.6
-3541451B51R2 Spot 177	1023	87048	0.9	13.7925	1.0	1.3737	1.9	0.1375	1.7	0.85	830.4	12.9	877.8	11.4	999.2	20.9	999.2	20.9
-3541451B51R2 Spot 21	1964	152135	2.2	13.7836	0.8	1.6467	1.5	0.1647	1.3	0.86	982.8	11.5	988.3	9.4	1000.5	15.6	1000.5	15.6
-3541451B51R2 Spot 184	660	95583	3.8	13.7694	0.9	1.6825	1.3	0.1681	1.0	0.75	1001.6	9.0	1001.9	8.3	1002.6	17.6	1002.6	17.6
-3541451B51R2 Spot 230	2628	242422	1.5	13.7456	0.7	1.6921	2.5	0.1688	2.3	0.96	1005.3	21.9	1005.5	15.7	1006.1	14.5	1006.1	14.5
-3541451B51R2 Spot 38	626	152315	1.4	13.6950	0.9	1.6029	1.4	0.1593	1.1	0.79	952.8	9.9	971.4	8.9	1013.6	17.6	1013.6	17.6
-3541451B51R2 Spot 32	892	580808	4.2	13.6465	0.8	1.6983	1.4	0.1682	1.2	0.84	1001.9	11.3	1007.9	9.2	1020.8	15.7	1020.8	15.7
-3541451B51R2 Spot 64	1165	129094	2.3	13.5775	0.8	1.8231	1.2	0.1796	0.9	0.73	1064.8	8.4	1053.8	7.7	1031.0	16.4	1031.0	16.4
-3541451B51R2 Spot 187	395	96944	45.8	13.2765	0.9	1.7744	3.0	0.1709	2.9	0.95	1017.2	27.0	1036.1	19.5	1076.2	17.9	1076.2	17.9
-3541451B51R2 Spot 169	818	663326	1.8	13.2710	1.0	1.9407	1.4	0.1869	1.0	0.74	1104.4	10.5	1095.3	9.4	1077.0	19.1	1077.0	19.1
-3541451B51R2 Spot 228	2071	739971	14.8	13.2682	0.6	1.8476	1.3	0.1779	1.1	0.87	1055.4	10.6	1062.6	8.3	1077.4	12.5	1077.4	12.5
-3541451B51R2 Spot 95	305	71661	1.3	13.2316	1.1	1.8476	1.9	0.1774	1.5	0.79	1052.6	14.3	1062.6	12.3	1083.0	22.8	1083.0	22.8

-3541451B51R2 Spot 4	725	37046	0.8	13.2008	0.9	1.8722	1.7	0.1793	1.4	0.85	1063.3	14.0	1071.3	11.2	1087.7	17.9	1087.7	17.9
-3541451B51R2 Spot 151	1115	225815	2.6	13.2003	1.0	1.9243	1.7	0.1843	1.4	0.83	1090.4	13.9	1089.5	11.2	1087.7	19.0	1087.7	19.0
-3541451B51R2 Spot 249	415	93687	1.9	13.1746	0.7	1.9504	1.2	0.1864	1.0	0.80	1102.1	9.9	1098.6	8.2	1091.7	14.8	1091.7	14.8
-3541451B51R2 Spot 47	284	15282	1.6	13.1516	1.0	1.8928	1.3	0.1806	0.9	0.66	1070.4	8.4	1078.6	8.6	1095.2	19.4	1095.2	19.4
-3541451B51R2 Spot 98	1913	436236	20.1	13.1029	0.8	1.8897	1.4	0.1797	1.2	0.85	1065.1	11.9	1077.5	9.5	1102.6	15.3	1102.6	15.3
-3541451B51R2 Spot 58	182	107755	1.0	13.1018	1.1	1.9836	1.9	0.1886	1.6	0.82	1113.6	16.3	1109.9	13.0	1102.7	21.9	1102.7	21.9
-3541451B51R2 Spot 280	1150	246252	1.5	13.1005	1.0	1.8924	1.5	0.1799	1.1	0.76	1066.3	10.9	1078.4	9.8	1103.0	19.2	1103.0	19.2
-3541451B51R2 Spot 211	362	57619	0.8	13.0808	0.9	1.9246	1.8	0.1827	1.5	0.87	1081.5	15.2	1089.7	11.7	1105.9	17.4	1105.9	17.4
-3541451B51R2 Spot 245	1058	151196	1.9	13.0620	0.9	1.9434	1.5	0.1842	1.2	0.80	1089.8	11.7	1096.2	9.8	1108.8	17.7	1108.8	17.7
-3541451B51R2 Spot 74	353	523022	1.1	13.0430	0.7	1.9849	1.4	0.1878	1.2	0.86	1109.7	12.7	1110.4	9.8	1111.7	14.7	1111.7	14.7
-3541451B51R2 Spot 13	805	223416	5.5	12.8342	0.7	1.9867	1.4	0.1850	1.2	0.85	1094.3	11.8	1111.0	9.3	1143.9	14.5	1143.9	14.5
-3541451B51R2 Spot 116	174	17372	1.2	12.7937	1.1	2.0826	1.7	0.1933	1.4	0.79	1139.3	14.3	1143.1	12.0	1150.1	21.5	1150.1	21.5
-3541451B51R2 Spot 1	1145	690526	3.0	12.7089	1.5	1.8377	2.4	0.1695	1.9	0.80	1009.2	18.1	1059.1	15.9	1163.3	28.9	1163.3	28.9
-3541451B51R2 Spot 136	817	75585	0.9	12.5650	0.8	2.1576	1.4	0.1967	1.2	0.83	1157.6	12.5	1167.5	9.9	1185.9	15.9	1185.9	15.9
-3541451B51R2 Spot 283	209	22940859	1.4	12.5328	0.8	2.1836	1.3	0.1986	1.0	0.79	1167.6	11.0	1175.8	9.1	1191.0	15.8	1191.0	15.8
-3541451B51R2 Spot 28	1335	159638	5.2	12.3187	0.7	2.2777	1.8	0.2036	1.6	0.91	1194.6	17.8	1205.4	12.6	1224.9	14.6	1224.9	14.6
-3541451B51R2 Spot 257	774	53459	6.8	12.0648	0.9	2.3125	1.4	0.2024	1.1	0.78	1188.4	12.0	1216.1	10.1	1265.7	17.3	1265.7	17.3
-3541451B51R2 Spot 185	959	131460	10.3	12.0515	0.7	2.3677	1.3	0.2070	1.1	0.83	1213.0	12.2	1232.9	9.4	1267.8	14.2	1267.8	14.2
-3541451B51R2 Spot 121	666	42813	3.2	11.9962	0.9	2.4207	1.4	0.2107	1.1	0.77	1232.6	11.8	1248.8	9.8	1276.8	17.0	1276.8	17.0
-3541451B51R2 Spot 112	939	326578	5.5	11.9219	1.5	2.1281	2.1	0.1841	1.5	0.71	1089.3	14.8	1158.0	14.4	1288.9	28.7	1288.9	28.7
-3541451B51R2 Spot 225	511	123506	2.4	11.7902	0.7	2.5713	1.0	0.2200	0.8	0.74	1281.7	8.9	1292.5	7.6	1310.5	13.6	1310.5	13.6
-3541451B51R2 Spot 92	1050	316402	2.5	11.7818	0.8	2.5059	1.7	0.2142	1.4	0.86	1251.3	16.2	1273.7	12.0	1311.9	16.3	1311.9	16.3
-3541451B51R2 Spot 68	644	371462	1.6	11.4864	0.7	2.9309	1.2	0.2443	0.9	0.77	1408.9	11.3	1389.9	8.7	1361.0	14.1	1361.0	14.1
-3541451B51R2 Spot 282	1818	510911	11.0	11.4161	0.7	2.6548	1.4	0.2199	1.2	0.86	1281.4	13.6	1316.0	10.0	1372.8	13.2	1372.8	13.2
-3541451B51R2 Spot 3	171	37038	1.9	11.3925	0.9	2.9485	1.5	0.2437	1.1	0.76	1406.1	14.1	1394.5	11.1	1376.8	18.2	1376.8	18.2
-3541451B51R2 Spot 198	903	561245	1.6	11.1306	0.9	2.7509	1.4	0.2222	1.1	0.78	1293.3	13.2	1342.3	10.8	1421.3	17.3	1421.3	17.3
-3541451B51R2 Spot 89	532	57558	4.0	11.0567	0.7	3.1682	1.3	0.2542	1.0	0.82	1459.9	13.7	1449.4	9.9	1434.0	14.0	1434.0	14.0
-3541451B51R2 Spot 203	957	100960	1.1	10.6214	0.7	2.9189	1.6	0.2250	1.5	0.90	1308.0	17.7	1386.8	12.5	1510.3	13.2	1510.3	13.2
-3541451B51R2 Spot 220	363	142030	2.4	10.5569	0.6	3.4202	1.1	0.2620	0.9	0.82	1500.0	11.7	1509.0	8.4	1521.8	11.6	1521.8	11.6
-3541451B51R2 Spot 16	257	222822	2.9	10.4085	0.9	3.3505	1.6	0.2530	1.2	0.79	1454.1	16.0	1492.9	12.1	1548.4	17.7	1548.4	17.7
-3541451B51R2 Spot 100	233	137704	1.6	10.3101	0.9	3.3017	1.4	0.2470	1.1	0.77	1423.0	13.5	1481.5	10.7	1566.2	16.3	1566.2	16.3
-3541451B51R2 Spot 88	489	86583	2.2	10.2073	0.7	3.7529	1.0	0.2779	0.8	0.75	1581.0	10.5	1582.7	8.1	1585.0	12.5	1585.0	12.5
-3541451B51R2 Spot 196	573	62051	1.7	10.1850	0.9	3.6982	1.3	0.2733	1.0	0.72	1557.5	13.2	1571.0	10.6	1589.1	17.4	1589.1	17.4
-3541451B51R2 Spot 36	343	157321	1.2	10.0634	0.9	3.9940	1.4	0.2916	1.1	0.77	1649.7	16.0	1633.0	11.6	1611.5	17.0	1611.5	17.0
-3541451B51R2 Spot 138	538	51770	0.8	10.0351	0.9	3.8334	1.4	0.2791	1.0	0.73	1586.9	13.9	1599.8	10.9	1616.7	17.3	1616.7	17.3
-3541451B51R2 Spot 256	741	458141	1.3	9.9566	0.8	3.6976	1.3	0.2671	1.0	0.79	1526.2	13.7	1570.8	10.2	1631.3	14.4	1631.3	14.4
-3541451B51R2 Spot 247	121	350191	2.2	9.9529	1.3	3.9936	1.8	0.2884	1.3	0.71	1633.5	18.2	1632.9	14.5	1632.0	23.3	1632.0	23.3
-3541451B51R2 Spot 144	1145	194596	3.9	9.8079	0.8	4.0724	1.4	0.2898	1.1	0.82	1640.6	16.2	1648.8	11.1	1659.3	14.3	1659.3	14.3
-3541451B51R2 Spot 14	249	209977	1.1	9.5472	0.9	4.3981	1.6	0.3047	1.3	0.83	1714.4	19.3	1712.0	12.8	1709.0	16.0	1709.0	16.0
-3541451B51R2 Spot 166	357	59348	2.2	9.5149	0.9	4.4226	1.4	0.3053	1.1	0.76	1717.7	16.7	1716.6	12.0	1715.2	17.2	1715.2	17.2
-3541451B51R2 Spot 172	453	122403	4.3	9.4938	1.2	4.5449	1.7	0.3131	1.3	0.73	1755.8	19.6	1739.2	14.5	1719.3	21.8	1719.3	21.8
-3541451B51R2 Spot 160	995	592394	4.3	9.4576	0.7	4.3745	1.3	0.3002	1.1	0.84	1692.3	16.3	1707.5	10.7	1726.3	12.7	1726.3	12.7
-3541451B51R2 Spot 180	1234	2785925	1.8	9.4499	0.7	4.4787	1.4	0.3071	1.2	0.86	1726.4	17.6	1727.0	11.2	1727.8	12.7	1727.8	12.7
-3541451B51R2 Spot 183	320	96537	2.8	9.4391	0.7	4.4588	1.2	0.3054	1.0	0.84	1717.9	15.8	1723.3	10.3	1729.9	12.4	1729.9	12.4
-3541451B51R2 Spot 2	905	665233	1.9	9.4052	0.7	4.3319	1.3	0.2956	1.1	0.84	1669.6	16.4	1699.5	11.0	1736.5	13.3	1736.5	13.3
-3541451B51R2 Spot 60	189	62179	0.9	9.4006	0.9	4.7664	1.4	0.3251	1.1	0.75	1814.6	16.7	1779.0	11.8	1737.4	17.0	1737.4	17.0
-3541451B51R2 Spot 12	849	69194	2.2	9.3775	0.9	4.6392	1.5	0.3157	1.2	0.79	1768.5	18.9	1756.3	12.9	1741.9	17.3	1741.9	17.3
-3541451B51R2 Spot 90	498	105023	2.1	9.3666	0.8	4.6197	1.6	0.3140	1.4	0.87	1760.2	21.0	1752.8	13.2	1744.1	14.5	1744.1	14.5
-3541451B51R2 Spot 110	735	313476	1.4	9.3478	1.0	4.3689	1.6	0.2963	1.3	0.78	1673.1	18.5	1706.5	13.2	1747.7	18.2	1747.7	18.2
-3541451B51R2 Spot 40	167	77591	2.3	9.3216	0.9	4.6259	1.5	0.3129	1.2	0.79	1754.9	18.1	1754.0	12.4	1752.9	16.6	1752.9	16.6
-3541451B51R2 Spot 262	757	156542	5.0	9.2184	0.7	4.6930	1.7	0.3139	1.5	0.90	1759.9	22.9	1766.0	13.9	1773.2	13.2	1773.2	13.2
-3541451B51R2 Spot 258	461	293849	2.3	9.1269	1.0	4.5103	1.5	0.2987	1.2	0.78	1684.8	17.6	1732.9	12.7	1791.4	17.5	1791.4	17.5
-3541451B51R2 Spot 111	1210	178874	2.4	8.9877	0.8	4.8554	1.5	0.3166	1.3	0.87	1773.3	20.9	1794.5	13.0	1819.3	13.9	1819.3	13.9
-3541451B51R2 Spot 134	208	54181	1.3	8.9182	0.8	5.2397	1.7	0.3391	1.5	0.89	1882.1	24.1	1859.1	14.2	1833.4	13.9	1833.4	13.9
-3541451B51R2 Spot 229	2233	468400	5.2	8.8828	0.8	4.8173	1.4	0.3105	1.2	0.84	1743.1	18.6	1787.9	12.1	1840.6	14.1	1840.6	14.1
-3541451B51R2 Spot 18	446	121811	0.9	8.8282	0.7	5.0217	1.2	0.3217	1.0	0.83	1797.9	16.1	1823.0	10.5	1851.8	12.6	1851.8	12.6
-3541451B51R2 Spot 141	1042	432115	4.7	8.8069	0.8	5.2365	1.5	0.3346	1.3	0.85	1860.7	20.3	1858.6	12.6	1856.1	14.2	1856.1	14.2
-3541451B51R2 Spot 80	555	302196	2.9	8.7917	0.7	5.1995	1.1	0.3317	0.9	0.76	1846.5	13.9	1852.5	9.7	1859.3	13.4	1859.3	13.4
-3541451B51R2 Spot 128	1103	447340	2.4	8.7773	0.7	5.1263	1.3	0.3265	1.2	0.86	1821.3	18.3	1840.5	11.4	1862.2	12.2	1862.2	12.2
-3541451B51R2 Spot 8	2790	4024346	4.5	8.7722	0.6	5.1270	1.1	0.3263	0.9	0.83	1820.6	14.8	1840.6	9.6	1863.3	11.5	1863.3	11.5
-3541451B51R2 Spot 75	553	996572	2.2	8.6910	0.9	5.4522	1.4	0.3438	1.1	0.77	1905.0	17.5	1893.1	11.8	1880.0	15.7	1880.0	15.7
-3541451B51R2 Spot 263	1985	737480	19.1	8.6729	0.7	4.3932	1.3	0.2765	1.1	0.85	1573.5	15.5	1711.1	10.7	1883.8	12.2	1883.8	12.2
-3541451B51R2 Spot 216	1453	1875206	0.6	8.6661	1.1	5.0694	1.8	0.3188	1.5	0.81	1783.7	22.7	1831.0	15.3	1885.2	19.0	1885.2	19.0
-3541451B51R2 Spot 44	328	61216	1.5	8.6358	0.8	5.2413	1.3	0.3284	1.0	0.81	1830.7	16.6	1859.4	11.0	1891.5	13.6	1891.5	13.6
-3541451B51R2 Spot 218	337	183948	1.9	8.6058	0.7	5.3309	1.3	0.3329	1.1	0.84	1852.3	17.6	1873.8	11.1	1897.8	12.7	1897.8	12.7
-3541451B51R2 Spot 191	532	139444	1.3	8.5487	0.6	5.3764	1.2	0.3335	1.0	0.86	1855.3	16.3	1881.1	10.1	1909.7	11.0	1909.7	11.0
-3541451B51R2 Spot 50	1509	2469917	11.2	8.4479	0.8	5.0419	1.3	0.3091	1.0	0.80	1736.0	15.8	1826.4	11.0	1931.0	13.8	1931.0	13.8
-3541451B51R2 Spot 307	358	158982	1.3	8.3781	0.7	5.6349	1.4	0.3425	1.1	0.84	1898.9	18.8	1921.5	11.7	1945.8	13.2	1945.8	13.2
-3541451B51R2 Spot 308	646	176190	1.3	8.2850	0.8	4.6368	1.4	0.2787	1.1	0.81	1585.0	15.9	1755.9	11.6	1965.8	14.4	1965.8	14.4
-3541451B51R2 Spot 35	923	601728	5.7	8.1966	1.2	5.7859	2.2	0.3441	1.9	0.84	1906.4	30.8	1944.3	19.1	1984.9	21.1	1984.9	21.1
-3541451B51R2 Spot 288	543	32307	2.3	8.1467	1.1	5.1625	1.6	0.3052	1.2	0.74	1716.9	18.2	1846.5	13.9	1995.8	19.5	1995.8	19.5
-3541451B51R2 Spot 31	267	51591	1.1	7.9728	0.9	6.2580	1.4	0.3620	1.1	0.77	1991.8	18.8	2012.6	12.4	2034.0	15.9	2034.0	15.9
-3541451B51R2 Spot 45	326	135502	3.0	7.7445	1.1	6.8035	1.8	0.3823	1.4	0.80	2087.0	25.6	2086.2	15.8	2085.3	18.7	2085.3	18.7
-3541451B51R2 Spot 148	907	627962	6.3	7.7046	0.8	6.9997	1.6	0.3913	1.4	0.87	2128.9	25.8	2111.4	14.5	2094.4	14.2	2094.4	14.2
-3541451B51R2 Spot 255	1853	480314	20.5	7.6939	0.9	6.9966	1.3	0.3906	0.9	0.72	2125.6	16.4	2111.0	11.3	2096.8	15.6	2096.8	15.6
-3541451B51R2 Spot 292	1159	603190	2.4	7.4210	2.6	6.3818	3.0	0.3436	1.6	0.53	1904.1	26.0	2029.8	26.4	2160.0	44.6	2160.0	44.6
-3541451B51R2 Spot 304	442	40398047	2.7	7.3802	1.0	6.2826	1.7	0.3364	1.3	0.78	1869.5	21.3	2016.0	14.7	2169.6	18.3	2169.6	18.3
-3541451B51R2 Spot 137	913	489328	4.1	7.1761	0.8	7.2617	1.3	0.3781	1.1	0.81	2067.4	19.1	2144.1	12.0	2218.4	13.7	2218.4	13.7
-3541451B51R2 Spot 5	515	66335	2.1	6.8974	0.9	7.9867	2.0	0.3997	1.8	0.88	2167.7	32.6	2229.5	18.1	2286.8	16.2	2286.8	16.2
-3541451B51R2 Spot 252	202	161342	1.7	6.4788	0.6	9.5159	1.4	0.4473	1.3	0.90	2383.4	25.7	2389.1	13.2	2393.9	10.6	2393.9	10.6
-3541451B51R2 Spot 219	181	47729	1.5	6.3452	0.7	9.0025	1.1	0.4145	0.8	0.79	2235.3	15.9	2338.3	9.8	2429.3	11.2	2429.3	11.2
-3541451B51R2 Spot 153	395	142705	1.9	6.2901	1.0	10.2335	1.5	0.4671	1.2	0.76	2470.6	24.2	2456.1	14.3	2444.1	16.9	2444.1	16.9
-3541451B51R2 Spot 146	2344	628816	13.1	6.2445	0.8	8.7393	1.7	0.3960	1.5	0.88	2150.5	27.9	2311.2	15.7	2456.4	13.5	2456.4	13.5
-3541451B51R2 Spot 186	587	77673	1.1	6.2359	0.8	8.8864	1.4	0.4021	1.1	0.83	2178.6	20.7	2326.4	12.4	2458.7	12.9	2458.7	12.9
-3541451B51R2 Spot 53	162	54932	0.8	6.2310	0.6	10.4088	1.2	0.4706	1.1	0.87	2486.2	22.5	2471.8	11.6	2460.1	10.4	2460.1	10.4
-3541451B51R2 Spot 73	87	129460	4.6	6.2030	0.8	10.8981	1.3	0.4905	1.0	0.77	2572.8	21.2	2514.5	12.1	2467.7	14.0	2467.7	14.0
-3541451B51R2 Spot 175	804	641931	2.5	6.1763	0.7	10.7892	1.2	0.4835	1.0	0.83	2542.5	21.3	2505.1	11.4	2475.0	11.6	2475.0	11.6
-3541451B51R2 Spot 102	350	189411	0.8	6.1707	0.6	9.6273	1.4	0.4310	1.2	0.89	2310.4	23.8	2399.8	12.6	2476.5	10.5	2476.5	10.5
-3541451B51R2 Spot 223	618	79277	14.2	6.1554	0.7	10.3193	1.1	0.4609	0.9	0.79	2443.5	18.2	2463.8	10.6	2480.7	11.9	2480.7	11.9
-3541451B51R2 Spot 155	498	645801	3.5	6.1488	0.9	10.7564	1.2	0.4799	0.8	0.69	2526.8	17.1	2502.3	11.1	2482.5	14.6	2482.5	14.6
-3541451B51R2 Spot 287	2929	668839	8.7	6.1146	0.7	10.0995	1.5	0.4481	1.3	0.88	2386.7	26.4	2443.9	13.9	2491.9	12.0	2491.9	12.0
-3541451B51R2 Spot 97	680	1012721	1.9	6.0853	0.7	10.3153	1.3	0.4555	1.1	0.86	2419.5	23.0	2463.5	12.3	2500.0	11.6	2500.0	11.6
-3541451B51R2 Spot 130	307	156551	1.7	5.9915	1.0	10.0413	2.5	0.4365	2.3	0.92	2335.1	46.0	2438.6	23.4	2526.1	16.2	2526.1	16.2
-3541451B51R2 Spot 20	223	226650	1.1	5.9915	0.7	11.3155	1.3	0.4919	1.1	0.86	2579.0	24.2	2549.5	12.4	2526.1	11.4	2526.1	11.4
-3541451B51R2 Spot 61	1415	213422	1.6	5.9838	0.9	9.9248	1.6	0.4309	1.3	0.82	2309.8	26.1	2427.8	15.0	2528.2	15.5	2528.2	15.5
-3541451B51R2 Spot 302	184	105686	1.4	5.9731	0.8	11.0791	1.6	0.4802	1.3	0.85	2528.0	28.0	2529.8	14.6	2531.2	13.9	2531.2	13.9
-3541451B51R2 Spot 46	1367	291512	14.2	5.9357	1.1	10.1406	2.2	0.4367	1.9	0.86	2336.1	38.0	2447.7	20.8	2541.8	18.9	2541.8	18.9
-3541451B51R2 Spot 182	874	1200013	6.2	5.8370	0.6	11.1844	1.2	0.4737	1.1	0.86	2499.7	22.3	2538.6	11.6	2569.9	10.6	2569.9	10.6
-3541451B51R2 Spot 15	1715	298833	3.3	5.8369	0.9	11.5172	1.5	0.4878	1.2	0.81	2561.0	25.1	2566.0	13.7	2569.9	14.3	2569.9	14.3
-3541451B51R2 Spot 291	447	249543	2.5	5.6586	0.8	10.7725	1.8	0.4423	1.7	0.91	2360.9	32.7	2503.7	16.9	2621.6	12.5	2621.6	12.5
-3541451B51R2 Spot 81	1723	730720	22.5	5.4918	0.8	11.9697	1.5	0.4770	1.3	0.85	2514.0	27.4	2602.0	14.5	2671.3	13.5	2671.3	13.5

-3541451B51R2 Spot 168	726	3307282	3.9	5.4022	0.8	13.0379	1.4	0.5111	1.2	0.82	2661.1	25.7	2682.4	13.6	2698.4	13.6	2698.4	13.6
-3541451B51R2 Spot 99	841	130336	1.5	5.3782	0.7	13.1561	1.3	0.5134	1.1	0.85	2671.1	24.8	2690.9	12.6	2705.8	11.6	2705.8	11.6
-3541451B51R2 Spot 161	229	424432	2.0	5.2292	0.8	13.1398	1.2	0.4986	0.9	0.74	2607.6	18.3	2689.7	10.9	2752.1	12.7	2752.1	12.7
-3541451B51R2 Spot 33	116	323381	1.4	4.4428	1.1	14.2748	1.5	0.4602	1.0	0.66	2440.3	20.4	2768.1	14.4	3016.7	18.2	3016.7	18.2
-3541451B51R2 Spot 150	586	291132	0.7	3.4215	0.8	26.7223	1.3	0.6634	1.1	0.81	3280.3	27.5	3373.3	13.0	3429.0	12.2	3429.0	12.2
-3541451B51R2 Spot 120	245	191998	3.3	3.2897	0.6	29.6547	1.3	0.7078	1.1	0.88	3450.3	29.9	3475.4	12.5	3490.0	9.3	3490.0	9.3

Table _____. U-Pb geochronologic analyses.																		
#25 U1451B_62R						Isotope ratios						Apparent ages (Ma)						
Early Miocene 18 Ma																		
Analysis	U	206Pb	U/Th	206Pb*	±	207Pb*	±	206Pb*	±	error	206Pb*	±	207Pb*	±	206Pb*	±	Best age	±
	(ppm)	204Pb		207Pb*	(%)	235U*	(%)	238U	(%)	corr.	238U*	(Ma)	235U	(Ma)	207Pb*	(Ma)	(Ma)	(Ma)
-354-U1451-B-62R-1W-7-24 Spot 38	2493	65125	0.7	20.6418	1.2	0.0153	1.5	0.0023	0.9	0.59	14.8	0.1	15.4	0.2	120.1	28.3	14.8	0.1
-354-U1451-B-62R-1W-7-24 Spot 128	2396	16734	0.8	18.8163	1.8	0.0170	1.9	0.0023	0.7	0.38	14.9	0.1	17.1	0.3	334.0	40.8	14.9	0.1
-354-U1451-B-62R-1W-7-24 Spot 162	1457	2683	0.9	18.3352	2.9	0.0177	3.1	0.0024	0.9	0.29	15.2	0.1	17.8	0.5	392.4	65.7	15.2	0.1
-354-U1451-B-62R-1W-7-24 Spot 173	1969	13162	0.9	20.5811	1.2	0.0160	1.5	0.0024	0.9	0.59	15.3	0.1	16.1	0.2	127.0	27.7	15.3	0.1
-354-U1451-B-62R-1W-7-24 Spot 137	172	1116	1.0	28.1584	5.9	0.0118	6.1	0.0024	1.9	0.30	15.5	0.3	11.9	0.7	NA	NA	15.5	0.3
-354-U1451-B-62R-1W-7-24 Spot 67	862	2895	1.2	21.9662	2.5	0.0151	2.7	0.0024	1.1	0.40	15.5	0.2	15.2	0.4	NA	NA	15.5	0.2
-354-U1451-B-62R-1W-7-24 Spot 263	535	5212	1.5	17.6537	3.7	0.0534	4.4	0.0068	2.3	0.53	43.9	1.0	52.8	2.3	476.8	82.0	43.9	1.0
-354-U1451-B-62R-1W-7-24 Spot 290	1535	21237	3.0	20.6576	1.1	0.0498	1.4	0.0075	0.9	0.62	48.0	0.4	49.4	0.7	118.2	26.1	48.0	0.4
-354-U1451-B-62R-1W-7-24 Spot 43	330	3648	1.1	17.0372	2.1	0.0634	2.3	0.0078	0.9	0.41	50.3	0.5	62.4	1.4	554.9	46.2	50.3	0.5
-354-U1451-B-62R-1W-7-24 Spot 234	918	26484	3.8	19.5273	1.2	0.0570	1.4	0.0081	0.8	0.55	51.8	0.4	56.3	0.8	249.3	27.8	51.8	0.4
-354-U1451-B-62R-1W-7-24 Spot 258	759	5930	2.0	19.5752	2.8	0.0579	3.0	0.0082	1.0	0.34	52.8	0.5	57.1	1.6	243.7	64.4	52.8	0.5
-354-U1451-B-62R-1W-7-24 Spot 136	253	2794	1.4	19.3988	5.9	0.0630	6.0	0.0089	1.3	0.21	56.9	0.7	62.0	3.6	264.5	135.6	56.9	0.7
-354-U1451-B-62R-1W-7-24 Spot 226	61	2256	2.9	19.9484	8.4	0.0736	8.6	0.0106	1.7	0.20	68.3	1.2	72.1	6.0	200.0	194.9	68.3	1.2
-354-U1451-B-62R-1W-7-24 Spot 20	677	27881	1.1	18.0309	1.5	0.0930	1.7	0.0122	0.8	0.48	77.9	0.6	90.3	1.4	429.8	32.7	77.9	0.6
-354-U1451-B-62R-1W-7-24 Spot 159	307	3125	1.1	17.4140	2.3	0.1014	2.5	0.0128	0.8	0.34	82.1	0.7	98.1	2.3	506.9	51.2	82.1	0.7
-354-U1451-B-62R-1W-7-24 Spot 286	1372	36753	0.7	20.4997	0.7	0.0959	1.1	0.0143	0.9	0.78	91.3	0.8	93.0	1.0	136.3	16.2	91.3	0.8
-354-U1451-B-62R-1W-7-24 Spot 13	482	53871	2.3	18.1711	1.3	0.1262	1.6	0.0166	0.9	0.60	106.4	1.0	120.7	1.8	412.5	28.3	106.4	1.0
-354-U1451-B-62R-1W-7-24 Spot 299	1222	35148	15.3	16.1357	1.0	0.1594	2.0	0.0187	1.7	0.85	119.2	2.0	150.2	2.7	672.3	22.0	119.2	2.0
-354-U1451-B-62R-1W-7-24 Spot 268	6	849	24.8	3.1487	7.3	1.1752	8.6	0.0268	4.5	0.53	170.8	7.6	789.1	47.3	3557.5	113.1	170.8	7.6
-354-U1451-B-62R-1W-7-24 Spot 266	532	34909	2.7	19.1549	1.1	0.2007	2.2	0.0279	1.9	0.85	177.3	3.3	185.7	3.7	293.4	26.2	177.3	3.3
-354-U1451-B-62R-1W-7-24 Spot 193	43	23724	302497.3	7.9473	8.6	0.4981	8.9	0.0287	2.3	0.26	182.6	4.1	410.4	29.9	2039.7	151.7	182.6	4.1
-354-U1451-B-62R-1W-7-24 Spot 171	396	532462	1.7	18.8045	0.8	0.2495	1.1	0.0340	0.8	0.68	215.8	1.7	226.2	2.3	335.4	19.0	215.8	1.7
-354-U1451-B-62R-1W-7-24 Spot 41	1689	5275	1.7	11.8992	1.4	0.4278	1.6	0.0369	0.8	0.51	233.8	1.9	361.6	5.0	1292.6	27.4	233.8	1.9
-354-U1451-B-62R-1W-7-24 Spot 302	973	53859	0.9	18.4538	1.7	0.2983	1.9	0.0399	0.9	0.47	252.5	2.2	265.1	4.4	377.9	37.7	252.5	2.2
-354-U1451-B-62R-1W-7-24 Spot 94	140	6615	1.2	18.3969	2.0	0.3007	2.3	0.0401	1.2	0.52	253.7	3.0	266.9	5.5	384.9	44.7	253.7	3.0
-354-U1451-B-62R-1W-7-24 Spot 121	432	30666	2.6	16.8712	3.0	0.3319	3.2	0.0406	1.0	0.32	256.7	2.6	291.0	8.1	576.2	65.6	256.7	2.6
-354-U1451-B-62R-1W-7-24 Spot 7	1724	64587	1.8	19.2433	1.0	0.2963	1.4	0.0414	1.0	0.69	261.3	2.5	263.5	3.3	282.9	23.5	261.3	2.5
-354-U1451-B-62R-1W-7-24 Spot 127	65	58151	0.5	10.7231	2.6	0.5410	2.9	0.0421	1.2	0.41	265.8	3.1	439.1	10.2	1492.3	49.3	265.8	3.1
-354-U1451-B-62R-1W-7-24 Spot 298	5203	5369	13.6	13.5039	3.1	0.4831	3.6	0.0473	1.8	0.50	298.1	5.2	400.2	11.9	1042.0	63.2	298.1	5.2
-354-U1451-B-62R-1W-7-24 Spot 39	816	1438	2.7	8.2008	5.9	0.8176	6.0	0.0487	1.0	0.16	306.2	2.9	606.7	27.3	1984.0	105.0	306.2	2.9
-354-U1451-B-62R-1W-7-24 Spot 18	2906	28511	1.1	16.6885	0.4	0.4219	0.8	0.0511	0.6	0.84	321.2	2.0	357.4	2.3	599.8	8.8	321.2	2.0
-354-U1451-B-62R-1W-7-24 Spot 104	92	6656	1.3	19.6643	1.4	0.3886	1.7	0.0554	1.0	0.58	347.9	3.4	333.4	5.0	233.2	32.8	347.9	3.4
-354-U1451-B-62R-1W-7-24 Spot 158	1385	182359	2.7	18.4872	0.6	0.4405	1.0	0.0591	0.8	0.79	370.1	2.8	370.6	3.0	373.9	13.5	370.1	2.8
-354-U1451-B-62R-1W-7-24 Spot 120	3170	5765	0.9	6.8969	0.5	1.2769	1.6	0.0639	1.5	0.95	399.3	5.8	835.5	9.0	2286.9	8.6	399.3	5.8
-354-U1451-B-62R-1W-7-24 Spot 187	972	60029	1.9	17.4741	0.8	0.5546	2.9	0.0703	2.8	0.96	438.1	11.7	448.0	10.4	499.4	17.3	438.1	11.7
-354-U1451-B-62R-1W-7-24 Spot 248	406	56932	1.8	17.2245	1.3	0.6048	1.6	0.0756	0.9	0.57	469.7	4.3	480.3	6.3	531.0	29.5	469.7	4.3
-354-U1451-B-62R-1W-7-24 Spot 22	1054	371225	1.4	17.0929	0.5	0.6123	0.9	0.0759	0.8	0.84	471.8	3.5	485.0	3.6	547.7	10.9	471.8	3.5
-354-U1451-B-62R-1W-7-24 Spot 250	3516	333565	1.4	17.5099	0.7	0.6039	1.1	0.0767	0.8	0.78	476.6	3.9	479.7	4.2	494.8	15.2	476.6	3.9
-354-U1451-B-62R-1W-7-24 Spot 53	710	67478	1.5	17.4861	0.7	0.6114	1.2	0.0776	0.9	0.78	481.6	4.3	484.4	4.5	497.8	16.1	481.6	4.3
-354-U1451-B-62R-1W-7-24 Spot 278	940	132751	9.0	17.5607	0.6	0.6204	1.0	0.0790	0.7	0.74	490.4	3.3	490.1	3.7	488.5	14.0	490.4	3.3
-354-U1451-B-62R-1W-7-24 Spot 35	2217	283842	4.1	17.5218	0.5	0.6299	1.0	0.0801	0.9	0.88	496.6	4.3	496.0	4.0	493.3	10.8	496.6	4.3
-354-U1451-B-62R-1W-7-24 Spot 300	543	41287	3.1	16.8343	0.7	0.6616	1.3	0.0808	1.1	0.82	501.0	5.1	515.6	5.2	581.0	16.2	501.0	5.1
-354-U1451-B-62R-1W-7-24 Spot 142	1077	621342	3.0	17.3913	0.5	0.6412	1.0	0.0809	0.9	0.86	501.6	4.2	503.1	4.0	509.8	11.6	501.6	4.2
-354-U1451-B-62R-1W-7-24 Spot 188	765	271397	3.6	17.4340	0.6	0.6451	1.0	0.0816	0.9	0.84	505.7	4.2	505.5	4.1	504.4	12.5	505.7	4.2
-354-U1451-B-62R-1W-7-24 Spot 308	254	231379	1.5	17.1037	0.9	0.6614	1.5	0.0821	1.1	0.77	508.5	5.6	515.5	5.9	546.3	20.3	508.5	5.6
-354-U1451-B-62R-1W-7-24 Spot 205	460	124901	1.4	16.7766	0.7	0.6776	1.1	0.0825	0.9	0.77	510.9	4.3	525.3	4.7	588.4	15.6	510.9	4.3
-354-U1451-B-62R-1W-7-24 Spot 71	929	32494	0.5	16.7642	1.0	0.6812	1.6	0.0829	1.2	0.77	513.1	6.0	527.5	6.6	590.0	22.1	513.1	6.0
-354-U1451-B-62R-1W-7-24 Spot 60	809	62726	1.4	17.4079	0.6	0.6655	0.9	0.0841	0.7	0.76	520.3	3.6	518.0	3.8	507.7	13.5	520.3	3.6
-354-U1451-B-62R-1W-7-24 Spot 210	459	96438	1.5	16.9462	0.7	0.6854	1.2	0.0843	1.0	0.81	521.6	4.8	530.0	4.9	566.5	14.9	521.6	4.8
-354-U1451-B-62R-1W-7-24 Spot 264	2651	1527867	102.7	17.3345	0.5	0.6727	0.9	0.0846	0.8	0.82	523.6	3.8	522.4	3.8	517.0	11.7	523.6	3.8
-354-U1451-B-62R-1W-7-24 Spot 6	840	71925	1.0	16.9637	0.5	0.6895	0.9	0.0849	0.7	0.80	525.1	3.6	532.5	3.7	564.3	11.7	525.1	3.6
-354-U1451-B-62R-1W-7-24 Spot 200	647	174597	1.0	17.2695	0.5	0.6784	1.1	0.0850	0.9	0.88	525.9	4.8	525.8	4.4	525.2	11.3	525.9	4.8
-354-U1451-B-62R-1W-7-24 Spot 177	183	27812	1.2	16.8032	0.6	0.6999	1.0	0.0853	0.8	0.82	527.9	4.2	538.7	4.2	585.0	12.5	527.9	4.2
-354-U1451-B-62R-1W-7-24 Spot 295	929	48743	2.1	16.3579	0.6	0.7201	1.3	0.0855	1.1	0.87	528.7	5.7	550.7	5.4	643.0	13.4	528.7	5.7
-354-U1451-B-62R-1W-7-24 Spot 59	338	35132	3.1	17.2329	0.8	0.6840	1.1	0.0855	0.8	0.70	529.1	4.0	529.2	4.6	529.9	17.4	529.1	4.0
-354-U1451-B-62R-1W-7-24 Spot 213	149	35395	3.9	16.3317	0.9	0.7221	1.1	0.0856	0.7	0.64	529.3	3.7	551.9	4.8	646.4	18.4	529.3	3.7
-354-U1451-B-62R-1W-7-24 Spot 97	666	298978	4.2	17.2997	0.7	0.6835	1.1	0.0858	0.9	0.80	530.6	4.6	528.9	4.6	521.4	14.7	530.6	4.6
-354-U1451-B-62R-1W-7-24 Spot 23	868	110158	8.2	17.1098	0.6	0.6934	1.2	0.0861	1.1	0.87	532.3	5.5	534.8	5.2	545.6	13.5	532.3	5.5
-354-U1451-B-62R-1W-7-24 Spot 214	1191	58053	10.2	17.1261	0.6	0.7174	1.2	0.0892	1.1	0.88	550.5	5.5	549.2	5.1	543.5	12.5	550.5	5.5
-354-U1451-B-62R-1W-7-24 Spot 122	3166	61733	1.1	16.6296	0.5	0.7622	1.0	0.0920	0.8	0.85	567.2	4.5	575.3	4.3	607.4	11.0	567.2	4.5
-354-U1451-B-62R-1W-7-24 Spot 65	601	37872	1.3	16.2077	0.5	0.7845	1.0	0.0923	0.8	0.83	568.9	4.3	588.1	4.3	662.7	11.5	568.9	4.3
-354-U1451-B-62R-1W-7-24 Spot 126	1089	132611	11.5	16.7619	0.7	0.8091	1.3	0.0984	1.1	0.86	605.0	6.4	601.9	5.9	590.3	14.3	605.0	6.4
-354-U1451-B-62R-1W-7-24 Spot 169	501	160794	1.4	16.5500	0.6	0.8202	1.2	0.0985	1.0	0.85	605.6	5.7	608.2	5.3	617.8	13.2	605.6	5.7
-354-U1451-B-62R-1W-7-24 Spot 12	476	1694612	2.7	16.4751	0.8	0.8282	1.3	0.0990	1.0	0.78	608.6	6.0	612.6	6.1	627.6	17.6	608.6	6.0
-354-U1451-B-62R-1W-7-24 Spot 218	189	69189	3.3	16.2303	0.7	0.8564	1.1	0.1009	0.9	0.81	619.4	5.4	628.1	5.3	659.8	13.9	619.4	5.4
-354-U1451-B-62R-1W-7-24 Spot 27	1131	64744	7.9	16.4226	0.7	0.8596	1.3	0.1024	1.1	0.87	628.6	6.9	629.9	6.2	634.5	14.0	628.6	6.9
-354-U1451-B-62R-1W-7-24 Spot 175	450	47538	3.2	15.8231	0.7	0.9037	1.2	0.1038	0.9	0.79	636.4	5.6	653.7	5.6	714.0	15.2	636.4	5.6
-354-U1451-B-62R-1W-7-24 Spot 1	2162	76254	6.7	16.0944	0.4	0.8947	0.9	0.1045	0.8	0.89	640.6	4.8	648.9	4.2	677.8	8.5	640.6	4.8
-354-U1451-B-62R-1W-7-24 Spot 4	3737	277904	2.0	15.5856	0.4	0.9422	0.8	0.1065	0.7	0.86	652.7	4.4	674.0	4.1	746.0	8.8	652.7	4.4
-354-U1451-B-62R-1W-7-24 Spot 235	591	52004	44.7	16.0445	0.7	0.9167	1.2	0.1067	1.0	0.83	653.7	6.1	660.6	5.8	684.4	14.2	653.7	6.1
-354-U1451-B-62R-1W-7-24 Spot 157	575	127684	4.1	15.4666	0.8	0.9897	1.9	0.1111	1.7	0.90	678.9	11.0	698.6	9.6	762.2	17.2	678.9	11.0
-354-U1451-B-62R-1W-7-24 Spot 73	376	181564	1.8	15.4485	0.6	1.0215	1.1	0.1145	0.9	0.82	698.8	5.8	714.7	5.5	764.7	13.1	698.8	5.8
-354-U1451-B-62R-1W-7-24 Spot 84	119	22239	0.8	15.3388	0.6	1.0768	0.9	0.1198	0.7	0.76	729.7	4.7	742.1	4.8	779.7	12.4	729.7	4.7
-354-U1451-B-62R-1W-7-24 Spot 260	573	172948	1.8	15.5261	0.5	1.0715	1.0	0.1207	0.9	0.88	734.6	6.3	739.5	5.4	754.1	10.2	734.6	6.3
-354-U1451-B-62R-1W-7-24 Spot 55	666	97066	5.8	15.1620	0.5	1.1211	1.0	0.1233	0.9	0.85	749.7	6.2	763.5	5.5	804.0	11.5	749.7	6.2
-354-U1451-B-62R-1W-7-24 Spot 130	597	76145	4.2	14.8897	1.0	1.1504	1.8	0.1243	1.5	0.83	755.2	10.7	777.4	9.9	841.8	21.1	755.2	10.7
-354-U1451-B-62R-1W-7-24 Spot 288	610	532542	7.3	14.0416	2.2	1.2475	3.1	0.1271	2.1	0.69	771.3	15.4	822.3	17.3	962.8	45.4	771.3	15.4
-354-U1451-B-62R-1W-7-24 Spot 244	326	53806	1.1	15.1509	0.5	1.1654	1.0	0.1281	0.9	0.88	777.1	6.7	784.5	5.7	805.5	10.4	777.1	6.7
-354-U1451-B-62R-1W-7-24 Spot 80	257	113572	0.7	15.1785	2.0	1.1711	3.3	0.1290	2.7	0.81	782.0	19.8	787.2	18.2	801.7	41.0	782.0	19.8
-354-U1451-B-62R-1W-7-24 Spot 49	476	71899	0.6	15.3441	0.7	1.1638	1.2	0.1296	1.0	0.84	785.4	7.4	783.8	6.5	778.9	13.8	785.4	7.4
-354-U1451-B-62R-1W-7-24 Spot 289	304	80537	0.5	15.1222	0.5	1.2119	0.9	0.1330	0.8	0.82	804.8	5.9	806.1	5.3	809.5	11.4	804.8	5.9
-354-U1451-B-62R-1W-7-24 Spot 273	818	1195869	3.8	14.3985	0.4	1.2883	1.4	0.1346	1.4	0.96	814.0	10.6	840.5	8.3	911.3	8.4	814.0	10.6
-354-U1451-B-62R-1W-7-24 Spot 306	1132	2051524	7.9	15.1081	0.6	1.2411	1.2	0.1361	1.0	0.87	822.3	7.9	819.4	6.6	811.5	12.0	822.3	7.9
-354-U1451-B-62R-1W-7-24 Spot 185	1181	1089705	9.9	14.8588	0.7	1.2761	1.1	0.1376	0.9	0.80	831.0	6.8	835.1	6.2	846.2	13.6	831.0	6.8
-354-U1451-B-62R-1W-7-24 Spot 145	234	1867192	1.9	14.6620	1.0	1.2944	1.8	0.1377	1.4	0.81	831.7	11.1	843.3	10.0	873.9	21.3	831.7	11.1
-354-U1451-B-62R-1W-7-24 Spot 164	523	49292	5.5	14.8902	0.5	1.2776	1.1	0.1380	1.0	0.91	833.5	8.1	835.8	6.5	841.8	10.0	833.5	8.1
-354-U1451-B-62R-1W-7-24 Spot 313	229	76219	2.9	14.5546	0.7	1.3184	1.2	0.1392	1.0	0.83	840.3	7.7	853.8	6.8	889.1	13.6	840.3	7.7
-354-U1451-B-62R-1W-7-24 Spot 178	313	64462	1.9	14.7519	0.7	1.3160	1.0	0.1409	0.7	0.74	849.5	6.0	852.8	5.8	861.2	14.1	849.5	6.0
-354-U1451-B-62R-1W-7-24 Spot 76	1423	344080	37.3	14.2918	0.7	1.3595	1.2	0.1410	1.0	0.83	850.2	7.9	871.7	7.0	926.6	13.8	850.2	7.9
-354-U1451-B-62R-1W-7-24 Spot 179	1565	295063	2.5	14.8665	0.6	1.3164	1.1	0.1420	0.9	0.83	855.9	7.2	852.9	6.2	845.1	12.4	855.9	7.2
-354-U1451-B-62R-1W-7-24 Spot 204	47	14941	0.8	14.4489	0.9	1.3741	1.4	0.1441	1.0	0.74	867.6	8.5	877.9	8.3	904.1	19.5	867.6	8.5
-354-U1451-B-62R-1W-7-24 Spot 107	381	29548	5.2	14.4467	0.8	1.3807	1.2	0.1447	0.9	0.75	871.3	7.5	880.7	7.2	904.4	16.5	871.3	7.5
-354-U1451-B-62R-1W-7-24 Spot 47	77	10641	2.0	14.5211	0.8	1.4138	1.2	0.1490	0.8	0.69	895.1	6.7	894.7	6.9	893.8	17.3	895.1	6.7
-354-U1451-B-62R-1W-7-24 Spot 270	365	47590	2.6	14.5610	0.7	1.4283	1.1	0.1509	0.9	0.77	906.0	7.4	900.9	6.8	888.1	15.0	906.0	7.4

-354-U1451-B-62R-1W-7-24 Spot 191	120	28653	2.5	14.3995	0.7	1.5233	1.1	0.1592	0.9	0.77	952.1	7.8	939.8	7.0	911.1	14.8	911.1	14.8
-354-U1451-B-62R-1W-7-24 Spot 293	1765	197618	6.0	14.2295	0.5	1.5395	0.8	0.1590	0.6	0.78	951.0	5.6	946.3	5.0	935.6	10.4	935.6	10.4
-354-U1451-B-62R-1W-7-24 Spot 24	978	22070850	2.2	14.2202	0.4	1.5637	1.2	0.1613	1.1	0.93	964.2	9.7	955.9	7.2	936.9	8.7	936.9	8.7
-354-U1451-B-62R-1W-7-24 Spot 11	1026	112559	7.4	14.1383	0.4	1.6262	1.0	0.1668	0.9	0.90	994.6	8.0	980.4	6.1	948.7	8.9	948.7	8.9
-354-U1451-B-62R-1W-7-24 Spot 78	561	117145	11.9	14.1304	0.6	1.5327	1.1	0.1571	0.9	0.84	940.9	8.0	943.6	6.6	949.9	11.9	949.9	11.9
-354-U1451-B-62R-1W-7-24 Spot 52	725	223223	4.3	14.1290	0.5	1.5405	0.8	0.1579	0.7	0.83	945.3	6.0	946.7	5.1	950.1	9.4	950.1	9.4
-354-U1451-B-62R-1W-7-24 Spot 124	783	150428	0.7	14.1129	0.5	1.5689	0.9	0.1607	0.8	0.86	960.4	7.2	958.0	5.8	952.4	9.8	952.4	9.8
-354-U1451-B-62R-1W-7-24 Spot 190	1999	38107	7.7	14.0714	0.6	1.3999	0.9	0.1429	0.7	0.72	861.2	5.4	888.9	5.5	958.4	13.3	958.4	13.3
-354-U1451-B-62R-1W-7-24 Spot 62	953	89388	2.9	14.0422	0.4	1.4232	1.0	0.1450	0.9	0.90	872.9	7.5	898.7	6.1	962.7	9.2	962.7	9.2
-354-U1451-B-62R-1W-7-24 Spot 294	461	61503	1.1	14.0040	0.6	1.6445	1.0	0.1671	0.8	0.81	996.1	7.3	987.4	6.2	968.2	11.9	968.2	11.9
-354-U1451-B-62R-1W-7-24 Spot 202	174	19400	4.5	13.9683	0.7	1.4706	1.1	0.1491	0.9	0.80	895.7	7.5	918.4	6.8	973.4	13.7	973.4	13.7
-354-U1451-B-62R-1W-7-24 Spot 259	456	109718	2.8	13.8683	0.7	1.7141	1.0	0.1725	0.6	0.66	1025.8	6.0	1013.8	6.2	988.1	14.7	988.1	14.7
-354-U1451-B-62R-1W-7-24 Spot 195	266	99458	2.5	13.8661	0.6	1.5868	1.1	0.1596	1.0	0.86	954.8	8.6	965.0	7.1	988.4	12.0	988.4	12.0
-354-U1451-B-62R-1W-7-24 Spot 45	82	20826	3.2	13.8581	0.8	1.5443	1.2	0.1553	0.9	0.75	930.5	8.0	948.2	7.6	989.6	16.7	989.6	16.7
-354-U1451-B-62R-1W-7-24 Spot 48	1226	96800	3.0	13.7728	0.5	1.4757	1.2	0.1475	1.0	0.90	886.8	8.6	920.5	7.0	1002.1	10.3	1002.1	10.3
-354-U1451-B-62R-1W-7-24 Spot 57	245	37899	2.1	13.7360	0.8	1.6464	1.3	0.1641	1.0	0.81	979.5	9.5	988.2	8.2	1007.5	15.4	1007.5	15.4
-354-U1451-B-62R-1W-7-24 Spot 211	112	278958	0.5	13.7284	0.8	1.6380	1.1	0.1632	0.8	0.72	974.3	7.2	984.9	7.0	1008.6	15.7	1008.6	15.7
-354-U1451-B-62R-1W-7-24 Spot 255	252	34457	1.3	13.7279	0.7	1.6700	1.1	0.1663	0.8	0.74	992.0	7.5	997.2	7.0	1008.7	15.0	1008.7	15.0
-354-U1451-B-62R-1W-7-24 Spot 201	264	661272	2.4	13.7020	0.5	1.4634	1.8	0.1455	1.8	0.97	875.6	14.6	915.4	11.1	1012.5	9.7	1012.5	9.7
-354-U1451-B-62R-1W-7-24 Spot 32	447	92537	5.2	13.6905	0.5	1.7878	0.9	0.1776	0.8	0.82	1053.8	7.3	1041.0	6.0	1014.3	10.5	1014.3	10.5
-354-U1451-B-62R-1W-7-24 Spot 297	393	61907	1.2	13.6762	0.6	1.6884	1.0	0.1675	0.8	0.83	998.6	7.7	1004.2	6.4	1016.4	11.2	1016.4	11.2
-354-U1451-B-62R-1W-7-24 Spot 167	2161	59415	52.0	13.6645	0.5	1.5670	1.0	0.1554	0.9	0.85	931.0	7.4	957.2	6.2	1018.1	10.8	1018.1	10.8
-354-U1451-B-62R-1W-7-24 Spot 96	751	119314	1.8	13.6381	0.4	1.6883	0.9	0.1671	0.7	0.86	995.9	6.9	1004.1	5.5	1022.0	9.1	1022.0	9.1
-354-U1451-B-62R-1W-7-24 Spot 42	43	7995	1.1	13.6235	1.3	1.6668	1.7	0.1648	1.1	0.65	983.2	10.0	996.0	10.6	1024.2	25.6	1024.2	25.6
-354-U1451-B-62R-1W-7-24 Spot 112	207	62495	2.7	13.6135	0.8	1.6413	1.3	0.1621	1.1	0.81	968.6	9.8	986.2	8.5	1025.7	16.1	1025.7	16.1
-354-U1451-B-62R-1W-7-24 Spot 240	322	27378	5.8	13.6084	0.8	1.5091	1.2	0.1490	0.9	0.73	895.4	7.3	934.1	7.4	1026.4	16.7	1026.4	16.7
-354-U1451-B-62R-1W-7-24 Spot 224	656	74061	3.0	13.5890	0.6	1.7815	1.1	0.1757	0.9	0.83	1043.2	8.8	1038.7	7.1	1029.3	12.2	1029.3	12.2
-354-U1451-B-62R-1W-7-24 Spot 31	885	165257	4.5	13.5589	0.6	1.8417	0.9	0.1812	0.7	0.79	1073.5	7.0	1060.5	5.9	1033.8	11.3	1033.8	11.3
-354-U1451-B-62R-1W-7-24 Spot 155	844	139985	2.7	13.5503	0.6	1.6881	1.1	0.1660	0.9	0.86	989.9	8.5	1004.0	6.9	1035.1	11.3	1035.1	11.3
-354-U1451-B-62R-1W-7-24 Spot 186	1438	140694	2.3	13.5074	0.6	1.5703	1.2	0.1539	1.0	0.86	922.8	9.0	958.6	7.5	1041.5	12.5	1041.5	12.5
-354-U1451-B-62R-1W-7-24 Spot 282	424	53549	8.1	13.5041	0.8	1.7798	3.2	0.1744	3.1	0.97	1036.2	29.5	1038.1	20.7	1042.0	16.0	1042.0	16.0
-354-U1451-B-62R-1W-7-24 Spot 262	220	53099	1.1	13.4673	0.7	1.7938	2.5	0.1753	2.4	0.96	1041.2	23.4	1043.2	16.5	1047.5	14.0	1047.5	14.0
-354-U1451-B-62R-1W-7-24 Spot 176	1069	612863	5.5	13.4668	0.8	1.8548	1.3	0.1812	1.0	0.80	1073.7	10.3	1065.1	8.6	1047.6	15.6	1047.6	15.6
-354-U1451-B-62R-1W-7-24 Spot 141	573	31140	1.3	13.4176	0.6	1.8037	1.6	0.1756	1.5	0.94	1042.9	14.7	1046.8	10.6	1055.0	11.1	1055.0	11.1
-354-U1451-B-62R-1W-7-24 Spot 64	752	17839	7.5	13.4142	0.7	1.4622	1.0	0.1423	0.8	0.75	857.7	6.1	914.9	6.1	1055.5	13.6	1055.5	13.6
-354-U1451-B-62R-1W-7-24 Spot 50	303	54210	3.5	13.4116	0.5	1.8508	1.0	0.1801	0.8	0.84	1067.6	8.1	1063.7	6.5	1055.9	10.8	1055.9	10.8
-354-U1451-B-62R-1W-7-24 Spot 26	1234	1206254	2.7	13.3826	0.6	1.8738	1.2	0.1819	1.0	0.85	1077.6	9.8	1071.9	7.7	1060.2	12.3	1060.2	12.3
-354-U1451-B-62R-1W-7-24 Spot 229	395	62229	1.7	13.2763	0.6	1.9704	1.1	0.1898	0.9	0.82	1120.4	9.1	1105.5	7.2	1076.2	12.2	1076.2	12.2
-354-U1451-B-62R-1W-7-24 Spot 129	1434	21572	1.8	13.2747	0.8	1.5373	1.0	0.1481	0.6	0.62	890.2	5.0	945.4	5.9	1076.5	15.2	1076.5	15.2
-354-U1451-B-62R-1W-7-24 Spot 46	336	356255	4.7	13.1586	0.6	1.8731	0.9	0.1788	0.6	0.68	1060.6	5.7	1071.6	5.7	1094.1	12.7	1094.1	12.7
-354-U1451-B-62R-1W-7-24 Spot 131	416	166013	2.5	13.1219	0.6	1.9244	1.1	0.1832	0.9	0.84	1084.5	9.2	1089.6	7.3	1099.7	12.0	1099.7	12.0
-354-U1451-B-62R-1W-7-24 Spot 143	5203	531154	2.0	13.0653	0.5	1.8034	1.0	0.1710	0.8	0.87	1017.4	7.7	1046.7	6.2	1108.3	9.6	1108.3	9.6
-354-U1451-B-62R-1W-7-24 Spot 90	333	193600	2.6	13.0193	0.5	2.0758	1.0	0.1961	0.9	0.85	1154.3	9.3	1140.8	7.1	1115.4	10.9	1115.4	10.9
-354-U1451-B-62R-1W-7-24 Spot 15	547	74763	2.4	12.9361	0.5	2.0936	0.9	0.1965	0.8	0.86	1156.5	8.4	1146.7	6.4	1128.2	9.5	1128.2	9.5
-354-U1451-B-62R-1W-7-24 Spot 280	416	15696	1.6	12.9106	1.0	1.9353	4.3	0.1813	4.1	0.97	1074.1	41.0	1093.4	28.6	1132.1	20.3	1132.1	20.3
-354-U1451-B-62R-1W-7-24 Spot 123	1742	416521	1.2	12.8335	0.5	2.0184	1.0	0.1879	0.9	0.87	1110.2	9.2	1121.7	7.0	1144.0	10.2	1144.0	10.2
-354-U1451-B-62R-1W-7-24 Spot 30	1145	393871	0.8	12.7956	0.4	2.2163	0.8	0.2058	0.7	0.85	1206.2	7.2	1186.2	5.4	1149.9	8.0	1149.9	8.0
-354-U1451-B-62R-1W-7-24 Spot 98	665	97511	4.5	12.7475	0.9	2.1128	1.4	0.1954	1.1	0.77	1150.7	11.2	1153.0	9.5	1157.3	17.4	1157.3	17.4
-354-U1451-B-62R-1W-7-24 Spot 275	686	36606	1.4	12.7147	0.7	1.8771	1.0	0.1732	0.8	0.77	1029.6	7.5	1073.0	6.8	1162.4	13.1	1162.4	13.1
-354-U1451-B-62R-1W-7-24 Spot 196	1615	69931	4.2	12.7140	0.9	1.8248	1.4	0.1683	1.0	0.76	1002.9	9.6	1054.4	9.0	1162.5	17.8	1162.5	17.8
-354-U1451-B-62R-1W-7-24 Spot 146	365	68003	1.7	12.6613	0.5	2.1032	0.9	0.1932	0.7	0.81	1138.8	7.8	1149.9	6.3	1170.8	10.5	1170.8	10.5
-354-U1451-B-62R-1W-7-24 Spot 198	74	181282	1.5	12.6298	0.8	2.1873	1.3	0.2004	1.0	0.79	1177.7	11.1	1177.0	9.0	1175.7	15.6	1175.7	15.6
-354-U1451-B-62R-1W-7-24 Spot 312	692	348189	3.8	12.6002	0.5	2.2816	1.2	0.2086	1.0	0.90	1221.3	11.5	1206.6	8.2	1180.4	10.2	1180.4	10.2
-354-U1451-B-62R-1W-7-24 Spot 3	427	172551	1.3	12.5838	0.4	2.1921	1.0	0.2002	0.9	0.91	1176.2	9.5	1178.5	6.8	1183.0	8.2	1183.0	8.2
-354-U1451-B-62R-1W-7-24 Spot 269	693	212418	1.6	12.5675	0.5	1.7617	1.1	0.1606	1.0	0.89	960.4	9.0	1031.5	7.3	1185.5	10.4	1185.5	10.4
-354-U1451-B-62R-1W-7-24 Spot 144	96	69147	1.2	12.4070	0.7	2.1703	0.9	0.1954	0.6	0.67	1150.4	6.3	1171.6	6.2	1210.8	13.0	1210.8	13.0
-354-U1451-B-62R-1W-7-24 Spot 37	1284	314426	1.7	12.3771	0.4	2.2908	0.8	0.2057	0.7	0.85	1206.0	7.8	1209.4	5.9	1215.6	8.7	1215.6	8.7
-354-U1451-B-62R-1W-7-24 Spot 223	361	108596	2.8	12.3673	1.6	1.8410	1.7	0.1652	0.6	0.36	985.6	5.7	1060.2	11.4	1217.1	31.8	1217.1	31.8
-354-U1451-B-62R-1W-7-24 Spot 304	284	268947	1.7	12.3637	0.7	2.2122	1.0	0.1984	0.7	0.74	1167.0	7.7	1184.9	6.9	1217.7	13.0	1217.7	13.0
-354-U1451-B-62R-1W-7-24 Spot 156	999	196577	13.2	12.3339	0.5	1.9671	1.2	0.1760	1.1	0.92	1045.3	10.4	1104.3	7.9	1222.4	9.3	1222.4	9.3
-354-U1451-B-62R-1W-7-24 Spot 189	1063	48605	1.9	12.1656	0.6	2.1213	1.0	0.1873	0.9	0.84	1106.5	8.9	1155.8	7.1	1249.4	10.9	1249.4	10.9
-354-U1451-B-62R-1W-7-24 Spot 236	308	104985	1.4	12.0491	0.6	2.3912	1.0	0.2091	0.8	0.79	1223.8	8.8	1240.0	7.1	1268.2	11.8	1268.2	11.8
-354-U1451-B-62R-1W-7-24 Spot 150	1063	162625	9.4	11.8822	1.1	2.4764	1.7	0.2135	1.4	0.79	1247.5	15.5	1265.2	12.6	1295.4	21.0	1295.4	21.0
-354-U1451-B-62R-1W-7-24 Spot 161	447	217597	2.6	11.1654	0.5	2.4750	2.9	0.2005	2.8	0.98	1178.1	30.3	1264.8	20.7	1415.4	9.6	1415.4	9.6
-354-U1451-B-62R-1W-7-24 Spot 82	2371	11387113	2.5	11.0333	0.6	2.9946	0.9	0.2397	0.6	0.74	1385.3	8.0	1406.3	6.5	1438.1	11.0	1438.1	11.0
-354-U1451-B-62R-1W-7-24 Spot 170	394	22952	0.9	10.9614	0.7	2.5851	1.9	0.2056	1.8	0.92	1205.4	19.4	1296.4	14.0	1450.5	14.3	1450.5	14.3
-354-U1451-B-62R-1W-7-24 Spot 285	694	60276	2.7	10.8498	0.8	2.7875	1.3	0.2194	1.1	0.80	1278.9	12.5	1352.2	10.1	1470.0	15.3	1470.0	15.3
-354-U1451-B-62R-1W-7-24 Spot 194	741	397663	2.2	10.7203	0.6	3.3626	1.2	0.2616	1.0	0.86	1497.8	13.4	1495.7	9.1	1492.8	11.3	1492.8	11.3
-354-U1451-B-62R-1W-7-24 Spot 225	373	65378	2.0	10.6853	0.4	3.4003	0.9	0.2636	0.8	0.88	1508.4	10.2	1504.5	6.7	1498.9	7.7	1498.9	7.7
-354-U1451-B-62R-1W-7-24 Spot 279	613	62937	3.1	10.5918	0.6	3.2952	1.0	0.2532	0.9	0.85	1455.2	11.5	1479.9	8.1	1515.5	10.4	1515.5	10.4
-354-U1451-B-62R-1W-7-24 Spot 291	1744	196033	5.8	10.4999	0.6	3.0783	1.1	0.2345	0.9	0.85	1358.2	11.5	1427.3	8.5	1532.0	11.1	1532.0	11.1
-354-U1451-B-62R-1W-7-24 Spot 10	352	94820	0.9	10.4912	0.8	2.9924	1.7	0.2278	1.5	0.89	1322.9	17.9	1405.7	12.8	1533.5	14.1	1533.5	14.1
-354-U1451-B-62R-1W-7-24 Spot 287	326	81250	5.1	10.4513	1.0	3.4023	1.7	0.2580	1.4	0.82	1479.6	18.2	1504.9	13.1	1540.7	17.9	1540.7	17.9
-354-U1451-B-62R-1W-7-24 Spot 301	533	75103	1.5	10.3699	0.5	3.5518	1.1	0.2672	1.0	0.87	1526.8	12.9	1538.8	8.7	1555.4	10.2	1555.4	10.2
-354-U1451-B-62R-1W-7-24 Spot 217	302	388042	1.6	10.3233	0.6	3.7956	1.0	0.2843	0.8	0.83	1613.0	12.1	1591.8	8.2	1563.8	10.6	1563.8	10.6
-354-U1451-B-62R-1W-7-24 Spot 274	308	133905	2.6	10.3021	0.6	3.6021	1.0	0.2693	0.9	0.83	1537.0	11.9	1550.0	8.3	1567.7	11.0	1567.7	11.0
-354-U1451-B-62R-1W-7-24 Spot 284	254	1061446	1.2	10.2874	0.6	3.2524	1.8	0.2428	1.7	0.95	1401.1	21.7	1469.7	14.1	1570.4	10.4	1570.4	10.4
-354-U1451-B-62R-1W-7-24 Spot 106	171	67940	0.5	10.2836	0.5	3.5087	1.4	0.2618	1.3	0.94	1499.1	17.7	1529.2	11.1	1571.1	8.7	1571.1	8.7
-354-U1451-B-62R-1W-7-24 Spot 66	333	228056	1.9	10.1793	0.4	3.7087	1.0	0.2739	0.9	0.90	1560.7	12.6	1573.2	8.1	1590.1	8.4	1590.1	8.4
-354-U1451-B-62R-1W-7-24 Spot 110	335	340565	3.6	10.1733	0.7	3.2168	2.2	0.2375	2.1	0.94	1373.4	25.7	1461.2	17.1	1591.2	13.8	1591.2	13.8
-354-U1451-B-62R-1W-7-24 Spot 72	102	66071	2.8	10.0424	0.5	3.8254	0.7	0.2787	0.6	0.76	1585.0	8.0	1598.1	6.0	1615.4	9.0	1615.4	9.0
-354-U1451-B-62R-1W-7-24 Spot 276	581	29772	1.5	10.0111	0.7	3.7086	1.5	0.2694	1.3	0.87	1537.7	17.5	1573.2	11.8	1621.2	13.7	1621.2	13.7
-354-U1451-B-62R-1W-7-24 Spot 249	139	240627	2.9	9.8415	0.6	3.9952	0.9	0.2853	0.6	0.73	1618.0	9.0	1633.2	7.0	1652.9	11.0	1652.9	11.0
-354-U1451-B-62R-1W-7-24 Spot 138	446	65318	8.5	9.8388	1.0	3.5963	1.3	0.2567	0.8	0.60	1473.1	10.1	1548.7	10.1	1653.4	18.9	1653.4	18.9
-354-U1451-B-62R-1W-7-24 Spot 183	2584	149817	1.9	9.7549	0.5	3.2574	1.2	0.2306	1.0	0.89	1337.4	12.7	1470.9	9.2	1669.3	10.2	1669.3	10.2
-354-U1451-B-62R-1W-7-24 Spot 51	825	11255086	1.6	9.7091	0.5	4.3464	1.0	0.3062	0.9	0.86	1722.0	13.1	1702.2	8.4	1678.0	9.7	1678.0	9.7
-354-U1451-B-62R-1W-7-24 Spot 296	475	150614	1.9	9.6363	0.6	4.3360	1.2	0.3032	1.0	0.87	1707.0	15.7	1700.2	9.9	1691.9	11.0	1691.9	11.0
-354-U1451-B-62R-1W-7-24 Spot 89	100	61620	3.0	9.6259	0.5	4.3878	1.0	0.3065	0.9	0.85	1723.3	13.2	1710.0	8.5	1693.9	10.0	1693.9	10.0
-354-U1451-B-62R-1W-7-24 Spot 61	158	26275	1.5	9.5530	0.6	4.1748	1.0	0.2894	0.8	0.80	1638.4	11.8	1669.1	8.4	1707.9	11.4	1707.9	11.4
-354-U1451-B-62R-1W-7-24 Spot 95	270	1330421	3.2	9.3849	0.5	4.5167	0.8	0.3076	0.6	0.80	1728.7	9.2	1734.1	6.3	1740.5	8.4	1740.5	8.4
-354-U1451-B-62R-1W-7-24 Spot 16	237	100183	1.5	9.3731	0.5	4.6670	1.2	0.3174	1.0	0.89	1777.0	16.2	1761.3	9.8	1742.8	9.6	1742.8	9.6
-354-U1451-B-62R-1W-7-24 Spot 221	620	155584	3.3	9.3248	0.5	4.0883	1.1	0.2766	1.0	0.89	1574.3	13.3	1652.0	8.7	1752.2	8.8	1752.2	8.8
-354-U1451-B-62R-1W-7-24 Spot 172	239	171854	4.3	9.1447	1.0	4.0638	2.2	0.2696	2.0	0.89	1539.0	27.3	1647.1	18.2	1787.9	18.3	1787.9	18.3
-354-U1451-B-62R-1W-7-24 Spot 256	2037	273823	2.3	9.0629	0.5	4.2579	0.9	0.2800	0.8	0.84	1591.3	11.1	1685.3	7.7	1804.2	9.1	1804.2	9.1
-354-U1451-B-62R-1W-7-24 Spot 199	388	110120	2.4	8.9117	0.4	4.9206	1.6	0.3182	1.5	0.97	1780.8	24.1	1805.8	13.4	1834.7	6.9	1834.7	6.9
-354-U1451-B-62R-1W-7-24 Spot 8	124	41765	4.6	8.3489	0.7	4.8060	3.0	0.2911	2.9	0.97	1647.2	42.0	1786.0	24.9	1952.1	12.0	1952.1	12.0
-354-U1451-B-62R-1W-7-24 Spot 19	143	154105	2.8	7.9552	0.5	6.2916	1.0	0.3632	0.9	0.89	1997.1	15.5	2017.3	8.9	2037.9	8.2	2037.9	8.2
-354-U1451-B-62R-1W-7-24 Spot 100	558	117182	2.9	7.6475	0.6	7.2151	1.2	0.4004	1.0	0.87	2170.7	19.1	2138.4	10.6	2107.5	10.1	2107.5	10.1
-354-U1451-B-62R-1W-7-24 Spot 29	775	480035	6.3	7.1071	0.5	7.5870	1.0	0.3912	0.8	0.84	2128.6	14.9	2183.3	8.8	2235.1	9.3	2235.1	9.3
-354-U1451-B-62R-1W-7-24 Spot 28	439	161578	1.3	6.5436	0.5	8.1472	1.0	0.3868	0.9	0.87	2108.1	16.0	2247.5	9.3	2377.0	8.7	2377.0	8.7
-354-U1451-B-62R-1W-7-24 Spot 212	537	206259	1.8	6.4607	0.6	9.3324	1.5	0.4375	1.4	0.93	2339.4	28.0	2371.2	14.1	2398.7	9.5	2398.7	9.5

-354-U1451-B-62R-1W-7-24 Spot 184	177	196460	1.6	6.4290	0.5	9.4454	1.1	0.4406	0.9	0.88	2353.4	18.7	2382.3	9.9	2407.0	8.6	2407.0	8.6
-354-U1451-B-62R-1W-7-24 Spot 81	394	892253	1.3	6.3705	0.5	10.1257	1.1	0.4680	0.9	0.86	2475.0	19.0	2446.3	9.9	2422.6	9.3	2422.6	9.3
-354-U1451-B-62R-1W-7-24 Spot 239	267	358955	1.3	6.2697	0.4	10.0001	0.9	0.4549	0.8	0.89	2417.1	16.6	2434.8	8.6	2449.6	7.1	2449.6	7.1
-354-U1451-B-62R-1W-7-24 Spot 33	227	144196	3.0	6.2425	0.5	10.1792	0.9	0.4611	0.8	0.83	2444.2	15.8	2451.2	8.6	2456.9	8.8	2456.9	8.8
-354-U1451-B-62R-1W-7-24 Spot 140	272	152350	1.1	6.2328	0.5	10.3142	1.0	0.4665	0.8	0.84	2468.0	16.7	2463.4	9.0	2459.6	8.8	2459.6	8.8
-354-U1451-B-62R-1W-7-24 Spot 243	669	227611	1.5	6.2247	0.5	10.7427	0.9	0.4852	0.8	0.83	2549.9	16.1	2501.1	8.5	2461.8	8.6	2461.8	8.6
-354-U1451-B-62R-1W-7-24 Spot 116	644	249440	6.9	5.8082	1.8	9.1222	2.2	0.3844	1.2	0.54	2097.0	20.7	2350.3	19.7	2578.1	30.4	2578.1	30.4
-354-U1451-B-62R-1W-7-24 Spot 232	642	184621	2.1	5.7616	0.4	9.4863	1.1	0.3966	1.0	0.91	2153.2	18.2	2386.2	10.0	2591.6	7.4	2591.6	7.4
-354-U1451-B-62R-1W-7-24 Spot 228	291	178475	1.1	5.6056	0.4	11.2263	1.2	0.4566	1.1	0.93	2424.6	22.0	2542.1	10.9	2637.2	7.3	2637.2	7.3
-354-U1451-B-62R-1W-7-24 Spot 208	558	198670	2.5	5.4160	0.5	13.3040	1.0	0.5228	0.8	0.87	2711.1	18.6	2701.5	9.1	2694.2	7.7	2694.2	7.7
-354-U1451-B-62R-1W-7-24 Spot 257	111	122559	2.1	5.0029	0.6	14.5134	0.9	0.5268	0.7	0.78	2728.1	16.3	2783.9	8.9	2824.5	9.5	2824.5	9.5
-354-U1451-B-62R-1W-7-24 Spot 0	434	593211	1.9	4.4290	0.4	19.4491	0.9	0.6250	0.8	0.88	3129.9	19.8	3064.4	8.8	3021.7	7.0	3021.7	7.0
-354-U1451-B-62R-1W-7-24 Spot 281	1329	500303	29.6	4.4142	0.4	16.9099	2.4	0.5416	2.4	0.99	2790.1	53.7	2929.7	23.0	3027.1	5.9	3027.1	5.9
-354-U1451-B-62R-1W-7-24 Spot 21	1064	1185122	41.6	4.1899	0.5	17.5502	1.0	0.5336	0.9	0.86	2756.4	19.2	2965.4	9.5	3110.4	8.0	3110.4	8.0
-354-U1451-B-62R-1W-7-24 Spot 163	534	6115805	1.1	3.5629	0.4	26.4738	1.1	0.6844	1.1	0.94	3361.2	27.9	3364.2	11.1	3366.0	6.2	3366.0	6.2

Table _____. U-Pb geochronologic analyses.

M01 Ganges River						Isotope ratios							Apparent ages (Ma)					
Analysis	U	206Pb	U/Th	206Pb*	±	207Pb*	±	206Pb*	±	error	206Pb*	±	207Pb*	±	206Pb*	±	Best age	±
	(ppm)	204Pb		207Pb*	(%)	235U*	(%)	238U	(%)	corr.	238U*	(Ma)	235U	(Ma)	207Pb*	(Ma)	(Ma)	(Ma)
M01 -Spot 259	4759	53266	154.2	20.9731	0.9	0.0197	1.4	0.0030	1.1	0.77	19.3	0.2	19.8	0.3	82.4	21.6	19.3	0.2
M01 -Spot 59	2421	78870	28.7	21.2820	1.3	0.0235	1.6	0.0036	1.0	0.58	23.4	0.2	23.6	0.4	47.6	31.6	23.4	0.2
M01 -Spot 22	242	1585	2.4	25.6006	2.0	0.0448	2.5	0.0083	1.4	0.58	53.4	0.8	44.5	1.1	NA	NA	53.4	0.8
M01 -Spot 222	2819	177994	72.0	9.4206	0.7	0.3163	1.5	0.0216	1.4	0.90	137.9	1.9	279.1	3.7	1733.5	12.5	137.9	1.9
M01 -Spot 171	4604	1272363	69.4	17.9604	0.6	0.3926	1.1	0.0512	0.9	0.85	321.7	2.9	336.3	3.1	438.6	12.8	321.7	2.9
M01 -Spot 122	2042	3708	5.9	10.7228	1.3	0.6627	1.6	0.0516	0.9	0.60	324.1	3.0	516.3	6.4	1492.3	23.9	324.1	3.0
M01 -Spot 251	387	45174	3.5	11.4297	5.6	0.6900	6.0	0.0572	2.2	0.36	358.7	7.5	532.8	25.0	1370.5	108.3	358.7	7.5
M01 -Spot 287	1722	52751	2.3	10.2549	0.9	0.7989	1.7	0.0594	1.4	0.85	372.2	5.2	596.2	7.6	1576.3	16.5	372.2	5.2
M01 -Spot 32	1192	30639	8.7	16.5523	1.9	0.5152	3.2	0.0619	2.5	0.80	387.0	9.5	422.0	10.9	617.5	40.7	387.0	9.5
M01 -Spot 194	2042	281993	18.2	17.3816	0.6	0.5427	2.2	0.0684	2.1	0.96	426.8	8.7	440.2	7.9	511.0	13.7	426.8	8.7
M01 -Spot 67	524	81091	6.6	17.4260	0.7	0.5490	3.1	0.0694	3.0	0.97	432.6	12.6	444.3	11.1	505.4	16.4	432.6	12.6
M01 -Spot 224	245	36475	2.8	17.5519	0.7	0.5681	1.4	0.0723	1.2	0.84	450.3	5.0	456.8	5.1	489.6	16.5	450.3	5.0
M01 -Spot 159	1513	197542	1.2	17.7119	0.6	0.5659	1.2	0.0727	1.0	0.84	452.5	4.2	455.3	4.2	469.5	14.0	452.5	4.2
M01 -Spot 211	1426	649932	16.0	17.6737	2.0	0.5723	3.3	0.0734	2.6	0.79	456.6	11.5	459.5	12.2	474.3	44.7	456.6	11.5
M01 -Spot 277	2532	1337563	5.1	17.5278	0.9	0.5780	3.4	0.0735	3.3	0.96	457.3	14.4	463.2	12.6	492.6	20.8	457.3	14.4
M01 -Spot 80	1764	830766	3.2	17.4525	0.7	0.5880	1.3	0.0745	1.1	0.85	463.0	4.9	469.6	4.8	502.1	14.6	463.0	4.9
M01 -Spot 88	1368	855858	18.4	17.6343	0.5	0.5828	1.1	0.0746	1.0	0.89	463.6	4.4	466.3	4.2	479.2	11.5	463.6	4.4
M01 -Spot 233	669	59712	2.5	17.2733	0.8	0.5960	1.4	0.0747	1.1	0.82	464.4	5.0	474.7	5.2	524.7	17.1	464.4	5.0
M01 -Spot 60	469	45753	3.8	16.9915	0.8	0.6065	1.6	0.0748	1.4	0.86	464.9	6.3	481.4	6.3	560.7	18.1	464.9	6.3
M01 -Spot 116	766	263330	10.8	17.6428	0.6	0.5845	1.2	0.0748	1.0	0.85	465.1	4.7	467.3	4.6	478.1	14.2	465.1	4.7
M01 -Spot 57	1919	458428	11.3	17.2598	0.7	0.5997	1.3	0.0751	1.0	0.81	466.9	4.7	477.1	4.9	526.5	16.4	466.9	4.7
M01 -Spot 114	851	541101	6.4	17.4999	0.6	0.5917	1.1	0.0751	0.9	0.84	467.0	4.0	472.0	4.0	496.1	12.8	467.0	4.0
M01 -Spot 278	739	44709	1.1	17.6392	0.9	0.5874	1.6	0.0752	1.3	0.84	467.3	5.9	469.2	5.8	478.6	18.8	467.3	5.9
M01 -Spot 10	275	29807	4.8	17.5891	1.0	0.5917	1.6	0.0755	1.2	0.77	469.3	5.5	471.9	5.9	484.9	22.0	469.3	5.5
M01 -Spot 75	342	33627	4.5	17.5586	0.9	0.6000	1.4	0.0764	1.1	0.79	474.9	5.1	477.2	5.4	488.7	19.4	474.9	5.1
M01 -Spot 208	908	346474	5.1	17.3899	0.7	0.6061	2.8	0.0765	2.7	0.97	475.0	12.4	481.1	10.7	510.0	15.7	475.0	12.4
M01 -Spot 183	1297	112824	3.4	17.6510	0.6	0.5983	1.0	0.0766	0.9	0.85	476.0	4.1	476.2	4.0	477.1	12.3	476.0	4.1
M01 -Spot 95	574	245912	4.4	17.2851	0.6	0.6117	1.2	0.0767	1.1	0.86	476.5	4.9	484.6	4.8	523.2	14.1	476.5	4.9
M01 -Spot 216	1046	134856	2.0	17.2141	0.6	0.6185	1.1	0.0773	0.8	0.80	479.7	3.9	488.9	4.1	532.3	14.1	479.7	3.9
M01 -Spot 213	775	59419	3.2	17.4286	0.8	0.6118	1.6	0.0774	1.3	0.86	480.4	6.2	484.7	6.0	505.1	17.6	480.4	6.2
M01 -Spot 115	657	68985	1.6	17.5729	0.5	0.6079	1.0	0.0775	0.8	0.83	481.2	3.7	482.2	3.7	486.9	11.7	481.2	3.7
M01 -Spot 170	590	280289	2.7	17.6077	0.7	0.6072	1.0	0.0776	0.8	0.73	481.6	3.5	481.8	4.0	482.6	15.6	481.6	3.5
M01 -Spot 147	348	44907	3.5	17.3083	0.7	0.6234	1.3	0.0783	1.2	0.87	485.9	5.4	492.0	5.2	520.3	14.7	485.9	5.4
M01 -Spot 100	614	58690	1.3	16.8260	0.8	0.6442	1.6	0.0787	1.3	0.84	488.1	6.2	504.9	6.2	582.0	18.3	488.1	6.2
M01 -Spot 237	674	131456	2.9	17.3329	0.8	0.6267	1.4	0.0788	1.1	0.80	489.0	5.2	494.0	5.4	517.2	18.4	489.0	5.2
M01 -Spot 182	894	450971	14.1	17.5399	0.6	0.6260	1.1	0.0797	0.9	0.84	494.1	4.3	493.6	4.2	491.1	12.9	494.1	4.3
M01 -Spot 161	98	496851	0.6	17.1281	1.1	0.6552	1.6	0.0814	1.2	0.72	504.7	5.6	511.7	6.4	543.2	24.1	504.7	5.6
M01 -Spot 26	77	23771	0.5	17.3112	1.2	0.6631	1.8	0.0833	1.3	0.74	515.7	6.5	516.5	7.2	519.9	26.5	515.7	6.5
M01 -Spot 220	307	1136059	2.3	17.2742	0.6	0.6675	1.7	0.0837	1.5	0.93	517.9	7.7	519.2	6.8	524.6	13.8	517.9	7.7
M01 -Spot 5	837	199456	8.4	17.0262	0.7	0.6794	1.3	0.0839	1.1	0.87	519.5	5.6	526.4	5.4	556.2	14.3	519.5	5.6
M01 -Spot 221	80	57524	0.8	17.3331	1.3	0.6777	1.8	0.0852	1.2	0.66	527.3	6.0	525.4	7.3	517.1	29.2	527.3	6.0
M01 -Spot 299	89	105865	0.8	17.1657	1.2	0.6866	1.6	0.0855	1.1	0.69	529.0	5.7	530.8	6.7	538.4	25.9	529.0	5.7
M01 -Spot 71	377	161413	1.7	17.0711	0.9	0.6951	1.6	0.0861	1.3	0.84	532.5	6.7	535.9	6.6	550.5	18.8	532.5	6.7
M01 -Spot 118	2891	82302	24.7	16.4682	0.7	0.7270	1.3	0.0869	1.1	0.83	537.0	5.5	554.8	5.5	628.5	15.5	537.0	5.5
M01 -Spot 14	1128	67326	23.2	16.8629	0.6	0.7379	1.6	0.0903	1.5	0.93	557.2	7.9	561.2	6.8	577.3	12.7	557.2	7.9
M01 -Spot 27	420	26920	6.6	15.6203	0.8	0.8505	1.5	0.0964	1.3	0.84	593.3	7.3	624.9	7.1	741.3	17.6	593.3	7.3
M01 -Spot 19	1936	132420	4.9	16.1970	0.8	0.8318	1.4	0.0978	1.1	0.80	601.2	6.4	614.6	6.5	664.2	18.1	601.2	6.4
M01 -Spot 263	811	2763990	1.3	16.6840	0.6	0.8223	1.2	0.0995	1.0	0.85	611.7	5.8	609.3	5.4	600.4	13.7	611.7	5.8
M01 -Spot 295	314	73586	2.6	16.5664	0.7	0.8476	1.1	0.1019	0.8	0.74	625.5	4.8	623.4	5.1	615.7	15.9	625.5	4.8
M01 -Spot 215	760	154753	1.5	16.0248	0.9	0.8815	1.6	0.1025	1.3	0.83	629.0	8.0	641.8	7.7	687.0	19.0	629.0	8.0
M01 -Spot 69	223	39391	1.0	16.4806	0.7	0.8617	1.1	0.1030	0.9	0.79	632.2	5.3	631.0	5.2	626.9	14.9	632.2	5.3
M01 -Spot 39	86	118595	0.9	16.4005	0.8	0.8987	1.1	0.1069	0.8	0.70	655.0	4.9	651.0	5.4	637.4	17.2	655.0	4.9
M01 -Spot 24	183	49010	5.1	15.9750	0.9	0.9444	1.3	0.1095	0.9	0.71	669.7	5.9	675.2	6.5	693.7	19.9	669.7	5.9
M01 -Spot 210	248	45471	4.5	15.5277	0.9	1.0099	1.7	0.1138	1.4	0.83	694.7	9.2	708.8	8.6	753.9	19.5	694.7	9.2
M01 -Spot 167	251	107204	1.7	15.0440	0.8	1.1023	1.6	0.1203	1.4	0.86	732.4	9.4	754.4	8.4	820.4	17.0	732.4	9.4
M01 -Spot 15	242	76844	2.3	14.4847	0.9	1.1497	4.2	0.1208	4.1	0.98	735.4	28.6	777.1	22.8	899.0	17.6	735.4	28.6
M01 -Spot 193	454	396019	1.4	15.2497	0.7	1.1156	1.1	0.1234	0.9	0.80	750.4	6.3	760.9	6.0	791.9	14.2	750.4	6.3
M01 -Spot 29	211	76336	5.3	15.2943	0.8	1.1160	1.3	0.1238	1.1	0.82	752.7	7.6	761.1	7.0	785.8	15.8	752.7	7.6
M01 -Spot 212	153	175554	1.7	15.4250	0.8	1.1156	1.3	0.1249	1.0	0.80	758.4	7.5	760.8	7.0	767.9	16.3	758.4	7.5
M01 -Spot 68	3246	1013528	5.0	15.0564	0.7	1.1539	1.5	0.1261	1.3	0.88	765.3	9.6	779.1	8.2	818.7	15.1	765.3	9.6
M01 -Spot 308	1022	199960	1.4	15.1684	0.6	1.1481	1.3	0.1264	1.1	0.87	767.1	8.2	776.4	7.1	803.1	13.4	767.1	8.2
M01 -Spot 158	631	145389	2.0	15.3195	0.5	1.1464	1.3	0.1274	1.2	0.91	773.2	8.7	775.5	7.1	782.3	11.4	773.2	8.7
M01 -Spot 174	307	89286	1.0	15.3666	0.7	1.1457	1.5	0.1277	1.4	0.90	775.0	10.1	775.2	8.3	775.9	13.7	775.0	10.1
M01 -Spot 104	290	206478	1.3	15.0361	0.7	1.1737	1.7	0.1280	1.5	0.92	776.7	11.3	788.4	9.2	821.5	14.1	776.7	11.3
M01 -Spot 257	188	35799	1.1	15.1823	0.7	1.1669	1.4	0.1285	1.2	0.86	779.5	8.5	785.2	7.4	801.2	14.7	779.5	8.5
M01 -Spot 254	86	265277	1.8	15.0341	0.9	1.1807	1.4	0.1288	1.0	0.74	781.0	7.4	791.6	7.4	821.7	18.9	781.0	7.4
M01 -Spot 197	812	268737	1.6	14.8365	0.7	1.1965	2.6	0.1288	2.5	0.96	781.1	18.6	799.0	14.5	849.3	15.2	781.1	18.6
M01 -Spot 293	466	78878	1.9	15.1953	0.7	1.1694	1.4	0.1289	1.2	0.86	781.8	9.1	786.4	7.9	799.4	15.7	781.8	9.1
M01 -Spot 248	1686	592120	1.1	14.7817	0.7	1.2092	1.4	0.1297	1.2	0.85	786.1	8.7	804.8	7.7	857.0	15.0	786.1	8.7
M01 -Spot 273	160	65751	1.8	15.3324	0.8	1.1728	1.6	0.1305	1.4	0.87	790.6	10.1	787.9	8.5	780.6	15.9	790.6	10.1
M01 -Spot 153	130	64116	1.3	15.1156	0.7	1.1976	1.3	0.1313	1.1	0.84	795.5	8.0	799.5	7.1	810.4	14.6	795.5	8.0
M01 -Spot 188	183	81580	0.7	14.9677	0.9	1.2105	1.5	0.1315	1.2	0.82	796.2	9.1	805.4	8.3	831.0	18.0	796.2	9.1
M01 -Spot 113	1259	814545	8.9	14.0344	0.7	1.2957	1.5	0.1319	1.3	0.87	798.9	9.5	843.8	8.3	963.8	14.4	798.9	9.5
M01 -Spot 102	146	32697	1.0	15.2992	1.0	1.1919	1.4	0.1323	0.9	0.70	801.1	7.1	796.9	7.5	785.1	20.4	801.1	7.1
M01 -Spot 53	119	88920	2.1	14.8720	0.8	1.2429	1.5	0.1341	1.3	0.85	811.3	9.9	820.2	8.6	844.3	16.7	811.3	9.9
M01 -Spot 195	83	20568	1.6	15.1630	1.0	1.2216	1.5	0.1344	1.2	0.76	812.9	9.0	810.5	8.6	803.9	20.9	812.9	9.0
M01 -Spot 227	863	98003	0.7	15.2148	0.6	1.2189	1.0	0.1346	0.8	0.81	813.8	6.0	809.3	5.4	796.7	11.9	813.8	6.0
M01 -Spot 151	183	62106	1.8	15.0499	0.8	1.2570	1.7	0.1373	1.5	0.89	829.2	11.9	826.6	9.7	819.6	16.4	829.2	11.9
M01 -Spot 124	363	179349	1.8	14.8598	0.6	1.2741	1.4	0.1374	1.2	0.90	829.8	9.6	834.2	7.7	846.0	12.1	829.8	9.6
M01 -Spot 44	259	70120	1.2	14.7639	0.9	1.2885	1.7	0.1380	1.4	0.86	833.5	11.1	840.6	9.5	859.5	17.8	833.5	11.1
M01 -Spot 38	847	556653	0.8	14.6298	0.7	1.3024	1.3	0.1383	1.1	0.82	834.8	8.3	846.8	7.4	878.4	15.0	834.8	8.3
M01 -Spot 13	797	196064	1.0	14.8295	0.6	1.2879	1.3	0.1386	1.1	0.89	836.6	8.9	840.4	7.3	850.3	12.0	836.6	8.9
M01 -Spot 300	923	449821	194.9	14.7818	0.8	1.2970	1.2	0.1391	1.0	0.78	839.6	7.7	844.4	7.2	856.9	16.2	839.6	7.7
M01 -Spot 250	315	195143	1.3	14.7943	0.8	1.2976	1.5	0.1393	1.2	0.82	840.7	9.6	844.7	8.5	855.2	17.5	840.7	9.6
M01 -Spot 132	1247	281687	8.4	14.6821	0.6	1.3117	1.0	0.1397	0.8	0.81	843.2	6.2	850.9	5.6	871.0	11.9	843.2	6.2
M01 -Spot 150	153	17205	0.9	14.7845	0.8	1.3179	1.3	0.1414	1.0	0.76	852.4	7.8	853.6	7.4	856.6	17.3	852.4	7.8
M01 -Spot 81	992	103832	28.6	14.3112	0.9	1.3683	1.5	0.1421	1.1	0.77	856.5	9.1	875.4	8.7	923.8	19.4	856.5	9.1
M01 -Spot 209	246	95880	1.0	14.9337	0.6	1.3151	1.3	0.1425	1.2	0.89	858.8	9.3	852.4	7.5	835.7	12.2	858.8	9.3
M01 -Spot 236	404	330943	1.7	14.4312	0.6	1.3685	1.2	0.1433	1.0	0.84	863.3	8.2	875.5	7.0	906.6	13.2	863.3	8.2
M01 -Spot 79	279	129848	1.5	14.8113	0.9	1.3404	1.5	0.1441	1.2	0.79	867.5	9.8	863.4	8.9	852.8	19.3	867.5	9.8
M01 -Spot 11	224	118816	0.8	14.9373	0.6	1.3294	1.1	0.1441	0.9	0.83	867.7	7.6	858.6	6.5	835.2	12.9	867.7	7.6
M01 -Spot 219	873	117755	12.7	14.6222	0.6	1.3659	1.5	0.1449	1.4	0.93	872.4	11.3	874.4	8.7	879.5	11.5	872.4	11.3
M01 -Spot 28	149	104356	1.5	14.6467	0.8	1.3718	1.3	0.1458	1.0	0.78	877.3	8.5	876.9	7.8	876.0	17.0	877.3	8.5
M01 -Spot 144	529	61734	1.4	14.7083	0.5	1.3764	1.2	0.1469	1.1	0.90	883.5	8.8	878.9	6.9	867.3	10.5	883.5	8.8

M01 -Spot 297	340	264061	3.0	14.4973	0.7	1.4081	1.4	0.1481	1.2	0.85	890.4	9.8	892.4	8.2	897.2	14.7	890.4	9.8
M01 -Spot 23	253	49713	5.2	14.5369	0.9	1.4285	1.5	0.1507	1.2	0.81	904.7	10.0	900.9	8.8	891.5	17.9	904.7	10.0
M01 -Spot 17	494	70321	1.5	14.2825	0.6	1.4490	0.9	0.1502	0.7	0.73	901.9	5.7	909.5	5.6	927.9	13.0	927.9	13.0
M01 -Spot 94	173	67532	2.5	14.2808	0.7	1.4615	1.1	0.1514	0.9	0.79	909.0	7.3	914.6	6.6	928.2	13.7	928.2	13.7
M01 -Spot 205	943	572602	5.4	14.1259	0.6	1.4819	1.4	0.1519	1.3	0.91	911.6	10.7	923.0	8.4	950.5	11.9	950.5	11.9
M01 -Spot 223	198	241941	1.1	14.0606	0.9	1.5266	1.6	0.1557	1.2	0.81	933.1	10.9	941.1	9.5	960.0	18.8	960.0	18.8
M01 -Spot 141	1202	174491	12.1	14.0572	0.5	1.5684	1.1	0.1600	0.9	0.86	956.6	8.3	957.8	6.7	960.5	11.1	960.5	11.1
M01 -Spot 109	278	70821	2.3	14.0400	0.9	1.4292	1.8	0.1456	1.5	0.86	876.2	12.5	901.2	10.6	963.0	18.5	963.0	18.5
M01 -Spot 49	220	46033	1.2	14.0201	0.8	1.6214	1.5	0.1649	1.3	0.83	984.2	11.4	978.6	9.5	965.9	17.1	965.9	17.1
M01 -Spot 162	525	168516	1.0	13.9845	0.8	1.6283	1.3	0.1652	1.0	0.77	985.7	9.3	981.2	8.3	971.1	17.3	971.1	17.3
M01 -Spot 243	626	71170	1.7	13.9810	0.5	1.4733	1.1	0.1495	1.0	0.90	897.9	8.2	919.5	6.6	971.6	9.8	971.6	9.8
M01 -Spot 266	163	63470	1.1	13.9779	0.8	1.5910	1.2	0.1614	1.0	0.78	964.3	8.6	966.7	7.7	972.0	15.8	972.0	15.8
M01 -Spot 112	588	217260	3.6	13.9759	0.4	1.6075	1.0	0.1630	0.9	0.89	973.5	7.9	973.1	6.1	972.3	9.1	972.3	9.1
M01 -Spot 146	437	384754	2.4	13.9483	0.6	1.5750	1.2	0.1594	1.0	0.86	953.5	8.8	960.4	7.1	976.4	12.0	976.4	12.0
M01 -Spot 154	102	1123213	0.4	13.9408	0.8	1.5523	1.2	0.1570	0.8	0.73	940.2	7.4	951.4	7.2	977.4	16.2	977.4	16.2
M01 -Spot 83	329	408198	2.4	13.9348	0.6	1.6095	1.2	0.1627	1.1	0.87	971.9	9.8	973.9	7.8	978.3	12.5	978.3	12.5
M01 -Spot 87	550	407812	7.0	13.9333	0.8	1.6629	1.7	0.1681	1.5	0.89	1001.7	13.8	994.5	10.6	978.5	15.6	978.5	15.6
M01 -Spot 18	1262	286706	1.2	13.8952	0.7	1.6050	1.2	0.1618	0.9	0.79	966.9	8.2	972.2	7.2	984.1	14.4	984.1	14.4
M01 -Spot 313	977	135811	6.1	13.8935	0.5	1.5819	1.1	0.1595	1.0	0.89	953.8	8.7	963.1	6.9	984.4	10.4	984.4	10.4
M01 -Spot 43	748	32137	4.9	13.8932	0.8	1.4290	1.3	0.1441	1.0	0.80	867.5	8.5	901.1	7.8	984.4	16.1	984.4	16.1
M01 -Spot 291	348	205240	2.6	13.8874	0.8	1.5840	1.4	0.1596	1.2	0.84	954.6	10.6	964.0	8.9	985.3	16.0	985.3	16.0
M01 -Spot 181	117	78889	0.7	13.8852	0.7	1.5442	1.2	0.1556	1.0	0.81	932.1	8.3	948.2	7.3	985.6	14.3	985.6	14.3
M01 -Spot 165	230	503520	1.2	13.7849	0.9	1.6182	1.4	0.1619	1.1	0.78	967.1	9.5	977.3	8.6	1000.3	17.3	1000.3	17.3
M01 -Spot 258	1004	100169	12.7	13.7793	0.7	1.3295	1.5	0.1329	1.3	0.87	804.5	9.9	858.7	8.7	1001.1	14.9	1001.1	14.9
M01 -Spot 204	677	195077	3.3	13.7721	0.9	1.6057	1.5	0.1605	1.2	0.79	959.3	10.3	972.4	9.2	1002.2	18.4	1002.2	18.4
M01 -Spot 85	68	36801	3.5	13.7675	0.8	1.6615	1.3	0.1660	1.1	0.81	989.9	9.8	993.9	8.3	1002.9	15.4	1002.9	15.4
M01 -Spot 93	629	62032	2.8	13.6947	0.7	1.6086	1.5	0.1598	1.3	0.87	955.9	11.8	973.6	9.5	1013.6	15.1	1013.6	15.1
M01 -Spot 289	576	74943	2.4	13.6915	0.8	1.6680	1.3	0.1657	1.0	0.80	988.4	9.3	996.4	8.1	1014.1	15.6	1014.1	15.6
M01 -Spot 305	218	72363	1.7	13.6634	0.8	1.7719	1.3	0.1757	1.0	0.75	1043.2	9.3	1035.2	8.3	1018.3	17.1	1018.3	17.1
M01 -Spot 288	875	136342	1.4	13.5480	0.6	1.5066	1.0	0.1481	0.8	0.79	890.3	6.5	933.1	6.0	1035.4	12.4	1035.4	12.4
M01 -Spot 164	750	315131	1.8	13.5209	0.5	1.6874	0.9	0.1655	0.7	0.79	987.5	6.5	1003.8	5.7	1039.5	11.1	1039.5	11.1
M01 -Spot 268	295	73162	1.1	13.4616	0.6	1.7851	1.2	0.1744	1.0	0.84	1036.1	9.3	1040.0	7.5	1048.4	12.7	1048.4	12.7
M01 -Spot 256	47	16028	3.4	13.4096	1.0	1.7121	1.4	0.1666	1.0	0.70	993.3	9.1	1013.1	9.0	1056.2	20.1	1056.2	20.1
M01 -Spot 61	282	329790	1.5	13.2820	0.8	1.8297	1.5	0.1763	1.3	0.86	1046.9	12.4	1056.2	9.8	1075.4	15.6	1075.4	15.6
M01 -Spot 231	336	71217	4.2	13.2736	0.7	1.9481	1.5	0.1876	1.3	0.87	1108.5	13.2	1097.8	9.9	1076.6	14.5	1076.6	14.5
M01 -Spot 74	492	106624	2.3	13.2334	0.7	1.8455	1.3	0.1772	1.1	0.83	1051.7	10.3	1061.8	8.4	1082.8	14.3	1082.8	14.3
M01 -Spot 255	852	461061	1.8	13.1668	0.6	1.8579	1.2	0.1775	1.1	0.86	1053.3	10.4	1066.2	8.2	1092.9	12.7	1092.9	12.7
M01 -Spot 307	694	348743	2.7	13.1626	0.8	1.7158	1.7	0.1639	1.5	0.89	978.3	13.5	1014.5	10.8	1093.5	15.5	1093.5	15.5
M01 -Spot 127	714	296638	1.7	13.1594	0.6	1.6477	1.2	0.1573	1.0	0.84	941.9	8.5	988.7	7.3	1094.0	12.5	1094.0	12.5
M01 -Spot 72	1542	99105	1.2	13.1451	0.7	1.6889	1.5	0.1611	1.3	0.87	962.8	11.4	1004.3	9.4	1096.2	14.7	1096.2	14.7
M01 -Spot 178	497	154750	0.7	13.1395	0.7	1.8772	1.4	0.1790	1.2	0.87	1061.3	11.7	1073.1	9.1	1097.0	13.4	1097.0	13.4
M01 -Spot 101	877	590207	4.1	13.0282	0.6	1.8675	1.2	0.1765	1.0	0.83	1048.0	9.5	1069.6	7.8	1114.0	12.9	1114.0	12.9
M01 -Spot 303	830	21839	1.8	12.8925	1.4	1.6697	2.0	0.1562	1.3	0.68	935.6	11.5	997.1	12.4	1134.9	28.7	1134.9	28.7
M01 -Spot 281	192	109603	0.7	12.8924	0.5	1.9928	1.3	0.1864	1.2	0.91	1101.9	12.1	1113.1	8.9	1134.9	10.9	1134.9	10.9
M01 -Spot 41	206	41834	1.7	12.8825	0.7	2.0512	1.5	0.1917	1.3	0.87	1130.8	13.3	1132.7	10.1	1136.4	14.7	1136.4	14.7
M01 -Spot 176	733	243385	5.3	12.7459	0.9	2.0067	1.4	0.1856	1.0	0.71	1097.4	9.8	1117.8	9.2	1157.6	18.9	1157.6	18.9
M01 -Spot 20	213	554323	1.1	12.6229	0.7	2.1769	1.2	0.1994	1.0	0.83	1172.0	11.1	1173.7	8.7	1176.8	13.7	1176.8	13.7
M01 -Spot 90	2176	2195256	6.1	12.5573	0.8	1.9829	1.5	0.1807	1.3	0.86	1070.6	13.1	1109.7	10.4	1187.1	15.3	1187.1	15.3
M01 -Spot 70	960	27386	3.3	12.4888	0.6	1.9279	1.2	0.1747	1.1	0.89	1038.0	10.4	1090.8	8.2	1197.9	11.3	1197.9	11.3
M01 -Spot 64	472	19863490	2.7	12.3573	0.8	2.1853	1.3	0.1959	1.0	0.80	1153.5	10.7	1176.4	8.8	1218.7	14.8	1218.7	14.8
M01 -Spot 36	639	875174	4.9	12.2564	0.9	1.9296	1.6	0.1716	1.3	0.81	1020.9	11.9	1091.4	10.4	1234.9	17.9	1234.9	17.9
M01 -Spot 8	301	48298	1.8	11.8988	0.7	2.5092	1.3	0.2166	1.1	0.84	1264.1	12.7	1274.7	9.6	1292.7	14.1	1292.7	14.1
M01 -Spot 298	938	102518	2.3	11.5356	0.6	2.5188	1.0	0.2108	0.8	0.80	1233.2	9.1	1277.5	7.4	1352.7	11.9	1352.7	11.9
M01 -Spot 126	522	858882	1.7	11.4778	0.6	2.3146	1.1	0.1928	0.9	0.80	1136.3	9.1	1216.8	7.7	1362.4	12.4	1362.4	12.4
M01 -Spot 108	728	33317210	1.8	11.4651	0.7	2.5647	1.4	0.2134	1.3	0.87	1246.7	14.2	1290.6	10.5	1364.5	13.5	1364.5	13.5
M01 -Spot 156	748	331509	2.1	11.0438	0.5	3.1703	1.0	0.2540	0.9	0.85	1459.3	11.4	1450.0	7.9	1436.3	10.4	1436.3	10.4
M01 -Spot 239	289	83572	2.3	10.8908	0.7	3.2446	1.3	0.2564	1.1	0.83	1471.4	14.7	1467.9	10.4	1462.8	14.0	1462.8	14.0
M01 -Spot 134	1227	343650	26.3	10.4315	0.7	3.4852	1.1	0.2638	0.9	0.75	1509.2	11.5	1523.9	8.9	1544.3	14.0	1544.3	14.0
M01 -Spot 172	674	872664	1.5	10.3985	0.6	3.5721	1.1	0.2695	0.9	0.84	1538.3	12.6	1543.4	8.6	1550.2	10.9	1550.2	10.9
M01 -Spot 97	43	66963	0.9	10.2939	1.0	3.3140	1.5	0.2475	1.2	0.78	1425.7	15.0	1484.3	11.8	1569.2	17.9	1569.2	17.9
M01 -Spot 86	795	2819178	1.5	10.0002	0.6	3.9365	1.1	0.2856	0.9	0.85	1619.6	13.2	1621.2	8.8	1623.2	10.8	1623.2	10.8
M01 -Spot 123	354	1945030	2.4	9.9133	0.6	3.9919	1.2	0.2871	1.0	0.85	1627.2	14.8	1632.5	9.8	1639.4	11.8	1639.4	11.8
M01 -Spot 180	362	93509	1.5	9.8898	0.6	3.9923	1.1	0.2865	1.0	0.85	1623.9	13.9	1632.6	9.2	1643.8	11.0	1643.8	11.0
M01 -Spot 214	183	226137	1.1	9.6455	0.6	4.1972	1.2	0.2938	1.1	0.88	1660.2	16.0	1673.5	10.1	1690.1	10.6	1690.1	10.6
M01 -Spot 202	859	369802	2.8	9.6006	0.6	4.2929	1.3	0.2990	1.2	0.90	1686.6	17.6	1692.0	10.9	1698.7	10.8	1698.7	10.8
M01 -Spot 285	469	112129	1.0	9.5363	0.6	4.1703	1.2	0.2886	1.1	0.89	1634.3	15.9	1668.2	10.1	1711.1	10.5	1711.1	10.5
M01 -Spot 261	307	240019	2.9	9.5059	0.5	4.4056	0.8	0.3039	0.7	0.78	1710.5	9.8	1713.4	6.9	1717.0	9.5	1717.0	9.5
M01 -Spot 296	1353	880916	8.1	9.4839	0.6	4.5824	1.1	0.3153	1.0	0.86	1766.9	14.9	1746.1	9.4	1721.2	10.7	1721.2	10.7
M01 -Spot 244	390	14155	1.2	9.4611	0.6	3.9948	1.4	0.2742	1.3	0.90	1562.3	18.0	1633.1	11.7	1725.6	11.7	1725.6	11.7
M01 -Spot 315	218	144493	0.9	9.4175	0.6	4.4273	1.1	0.3025	1.0	0.87	1703.8	14.6	1717.5	9.3	1734.1	10.2	1734.1	10.2
M01 -Spot 301	246	32557	1.6	9.4052	0.9	4.2957	1.6	0.2931	1.2	0.80	1657.3	18.1	1692.5	12.8	1736.5	17.3	1736.5	17.3
M01 -Spot 138	452	1586780	1.7	9.3713	0.6	4.0322	1.0	0.2742	0.9	0.84	1562.0	12.0	1640.7	8.4	1743.1	10.2	1743.1	10.2
M01 -Spot 225	2127	544505	10.2	9.3455	0.6	4.6273	1.1	0.3138	0.9	0.84	1759.3	14.2	1754.2	9.2	1748.2	11.1	1748.2	11.1
M01 -Spot 137	1035	4988347	11.6	9.2682	0.7	3.7658	3.9	0.2532	3.8	0.98	1455.2	49.7	1585.5	31.2	1763.4	13.4	1763.4	13.4
M01 -Spot 31	707	1594776	4.6	9.2573	0.8	4.8402	1.5	0.3251	1.2	0.82	1814.7	19.1	1791.9	12.4	1765.5	15.5	1765.5	15.5
M01 -Spot 284	402	141546	6.4	9.2467	0.6	4.8181	1.3	0.3233	1.1	0.87	1805.6	17.5	1788.1	10.8	1767.6	11.7	1767.6	11.7
M01 -Spot 91	518	982692	7.5	9.2002	0.7	4.5684	1.5	0.3050	1.3	0.87	1715.9	20.0	1743.5	12.7	1776.8	13.5	1776.8	13.5
M01 -Spot 128	897	1459853	14.2	9.1316	0.6	4.6904	1.1	0.3108	0.9	0.84	1744.5	13.7	1765.5	8.9	1790.5	10.6	1790.5	10.6
M01 -Spot 117	681	366277	7.7	9.1302	0.6	4.5410	1.0	0.3008	0.8	0.83	1695.4	12.1	1738.5	8.2	1790.8	10.1	1790.8	10.1
M01 -Spot 143	1782	929361	6.6	9.1270	0.6	4.7254	1.2	0.3129	1.0	0.84	1755.1	15.4	1771.7	10.0	1791.4	11.7	1791.4	11.7
M01 -Spot 206	340	102207	4.8	9.1074	0.7	3.8754	1.5	0.2561	1.3	0.86	1469.8	16.6	1608.6	11.8	1795.3	13.4	1795.3	13.4
M01 -Spot 3	574	3771643	1.4	9.0918	0.9	4.7118	1.7	0.3108	1.4	0.83	1744.8	21.0	1769.3	13.9	1798.4	16.8	1798.4	16.8
M01 -Spot 92	419	122998	2.1	9.0888	0.7	4.2997	1.4	0.2836	1.2	0.85	1609.2	17.3	1693.3	11.7	1799.0	13.6	1799.0	13.6
M01 -Spot 234	1259	1310417	9.3	9.0862	0.8	4.8888	1.6	0.3223	1.3	0.85	1801.0	20.8	1800.3	13.1	1799.5	14.8	1799.5	14.8
M01 -Spot 77	1452	270440	11.7	9.0408	0.6	3.8841	1.1	0.2548	1.0	0.85	1463.1	12.6	1610.4	9.1	1808.6	10.8	1808.6	10.8
M01 -Spot 25	1512	439293	3.0	9.0297	0.6	4.7590	1.1	0.3118	0.9	0.83	1749.5	14.3	1777.7	9.5	1810.9	11.5	1810.9	11.5
M01 -Spot 191	448	532149	2.5	8.9956	0.7	4.9586	1.3	0.3237	1.1	0.86	1807.5	17.6	1812.3	11.0	1817.7	11.9	1817.7	11.9
M01 -Spot 120	1035	2212573	6.9	8.9946	0.8	4.8700	1.3	0.3178	1.1	0.82	1779.1	17.2	1797.1	11.3	1817.9	13.8	1817.9	13.8
M01 -Spot 269	714	606758	7.1	8.9730	0.7	5.1552	1.4	0.3356	1.2	0.88	1865.6	20.0	1845.2	11.9	1822.3	11.8	1822.3	11.8
M01 -Spot 133	917	368768	3.2	8.9646	0.5	4.8273	1.1	0.3140	1.0	0.88	1760.3	14.8	1789.7	9.2	1824.0	9.4	1824.0	9.4
M01 -Spot 163	903	847343	6.1	8.9626	0.5	5.0647	1.0	0.3294	0.9	0.88	1835.3	14.2	1830.2	8.6	1824.4	8.5	1824.4	8.5
M01 -Spot 168	766	425157	5.3	8.9471	0.6	4.7641	1.0	0.3093	0.8	0.79	1737.2	12.6	1778.6	8.8	1827.6	11.7	1827.6	11.7
M01 -Spot 47	513	1351566	3.5	8.9421	0.8	4.7108	1.6	0.3056	1.4	0.85	1719.2	20.7	1769.2	13.5	1828.6	15.3	1828.6	15.3
M01 -Spot 142	435	79037	0.7	8.9366	0.8	4.8761	1.6	0.3162	1.3	0.85	1771.0	20.5	1798.1	13.1	1829.7	14.9	1829.7	14.9
M01 -Spot 280	330	354565	1.0	8.9138	0.5	4.9828	1.2	0.3223	1.1	0.90	1800.8	17.3	1816.4	10.3	1834.3	9.5	1834.3	9.5
M01 -Spot 50	472	1714511	7.7	8.9132	0.6	5.0655	1.1	0.3276	0.9	0.84	1826.7	14.1	1830.3	9.0	1834.4	10.4	1834.4	10.4
M01 -Spot 267	315	160729	1.2	8.9123	0.7	5.0306	1.5	0.3253	1.3	0.89	1815.6	20.7	1824.5	12.5	1834.6	12.2	1834.6	12.2
M01 -Spot 48	322	350115	1.7	8.9104	0.8	4.8758	1.3	0.3152	1.0	0.79	1766.4	15.6	1798.1	10.8	1835.0	14.2	1835.0	14.2
M01 -Spot 105	479	668560	7.9	8.8898	0.6	5.1315	1.3	0.3310	1.2	0.90	1843.2	18.6	1841.3	10.9	1839.2	10.2	1839.2	10.2
M01 -Spot 196	870	201404	7.1	8.8853	0.7	4.6597	1.1	0.3004	0.9	0.81	1693.3	13.8	1760.0	9.6	1840.1	12.3	1840.1	12.3
M01 -Spot 66	250	103837	2.6	8.8595	0.8	4.7476	1.6	0.3052	1.4	0.86	1717.0	21.1	1775.7	13.6	1845.4	14.7	1845.4	14.7

M01 -Spot 34	220	84448	2.4	8.8467	0.7	5.3068	1.4	0.3406	1.3	0.89	1889.8	20.5	1870.0	12.1	1848.0	11.9	1848.0	11.9
M01 -Spot 249	753	495176	9.5	8.8373	0.7	5.2600	1.7	0.3373	1.6	0.90	1873.6	25.3	1862.4	14.7	1849.9	13.2	1849.9	13.2
M01 -Spot 271	732	137508	1.2	8.8346	0.7	4.9774	1.2	0.3191	1.0	0.82	1785.2	15.3	1815.5	10.1	1850.5	12.4	1850.5	12.4
M01 -Spot 111	608	127963	4.2	8.8307	0.6	5.1118	1.4	0.3275	1.3	0.91	1826.4	20.2	1838.1	11.9	1851.3	10.5	1851.3	10.5
M01 -Spot 187	231	232335	1.1	8.8305	0.5	4.8634	1.0	0.3116	0.9	0.85	1748.6	13.4	1795.9	8.7	1851.3	9.8	1851.3	9.8
M01 -Spot 46	562	269256	2.9	8.8264	0.6	5.3172	1.2	0.3405	1.0	0.86	1889.2	16.7	1871.6	10.1	1852.2	10.7	1852.2	10.7
M01 -Spot 35	619	378581	6.2	8.8217	0.5	5.3461	1.1	0.3422	1.0	0.88	1897.2	16.4	1876.3	9.7	1853.1	9.7	1853.1	9.7
M01 -Spot 54	403	394182	2.9	8.8205	0.6	4.4739	1.4	0.2863	1.2	0.89	1623.2	17.6	1726.1	11.4	1853.4	11.3	1853.4	11.3
M01 -Spot 286	1198	306840	47.8	8.8178	0.6	4.7454	1.1	0.3036	0.9	0.82	1709.2	13.4	1775.3	9.1	1853.9	11.0	1853.9	11.0
M01 -Spot 42	327	2416438	3.0	8.8091	0.7	5.2888	1.4	0.3380	1.2	0.88	1877.3	19.4	1867.1	11.6	1855.7	11.8	1855.7	11.8
M01 -Spot 148	927	293784	7.8	8.8070	0.5	4.8423	0.9	0.3094	0.8	0.82	1737.9	11.6	1792.3	7.8	1856.1	9.6	1856.1	9.6
M01 -Spot 309	232	448742	1.6	8.8062	0.7	5.1520	1.2	0.3292	0.9	0.80	1834.5	15.1	1844.7	10.0	1856.3	12.7	1856.3	12.7
M01 -Spot 149	703	1462712	5.6	8.8010	0.5	4.8474	1.1	0.3095	0.9	0.87	1738.5	14.0	1793.2	8.9	1857.4	9.4	1857.4	9.4
M01 -Spot 84	581	403999	2.1	8.8002	0.6	5.1921	1.1	0.3315	0.9	0.84	1845.8	15.0	1851.3	9.5	1857.5	11.0	1857.5	11.0
M01 -Spot 129	317	445208	3.6	8.7993	0.5	5.2979	1.2	0.3383	1.1	0.91	1878.3	18.2	1868.5	10.4	1857.7	8.9	1857.7	8.9
M01 -Spot 185	870	321033	7.9	8.7990	0.7	5.2401	1.1	0.3346	0.8	0.77	1860.4	13.6	1859.2	9.3	1857.8	12.6	1857.8	12.6
M01 -Spot 106	329	115319	2.3	8.7798	0.5	5.3326	1.1	0.3397	0.9	0.87	1885.3	15.1	1874.1	9.1	1861.7	9.5	1861.7	9.5
M01 -Spot 107	273	334082	2.8	8.7788	0.6	5.1799	1.2	0.3299	1.1	0.85	1838.1	16.8	1849.3	10.5	1861.9	11.6	1861.9	11.6
M01 -Spot 40	354	687296	2.4	8.7649	0.7	5.1489	1.4	0.3275	1.2	0.87	1826.0	19.3	1844.2	11.9	1864.8	12.6	1864.8	12.6
M01 -Spot 218	232	238700	7.3	8.7497	0.8	5.2651	1.5	0.3343	1.3	0.85	1859.0	20.4	1863.2	12.7	1867.9	14.3	1867.9	14.3
M01 -Spot 21	458	123348	2.6	8.7220	0.8	5.1053	1.4	0.3231	1.2	0.84	1804.8	19.1	1837.0	12.3	1873.6	14.2	1873.6	14.2
M01 -Spot 260	250	263051	1.7	8.7083	0.9	5.3229	1.5	0.3363	1.2	0.81	1869.0	19.2	1872.5	12.5	1876.5	15.5	1876.5	15.5
M01 -Spot 265	818	689493	17.3	8.7025	0.8	4.4936	1.5	0.2837	1.2	0.83	1610.2	17.5	1729.8	12.2	1877.7	14.7	1877.7	14.7
M01 -Spot 245	308	485819	5.6	8.6871	0.6	5.3254	1.3	0.3357	1.2	0.88	1865.8	18.9	1873.0	11.3	1880.8	11.4	1880.8	11.4
M01 -Spot 58	332	90230	3.9	8.6796	0.7	5.3537	1.0	0.3372	0.8	0.75	1873.0	12.6	1877.5	8.8	1882.4	12.3	1882.4	12.3
M01 -Spot 240	593	63641	1.4	8.6372	0.7	5.1651	1.6	0.3237	1.4	0.88	1807.7	21.8	1846.9	13.3	1891.2	13.2	1891.2	13.2
M01 -Spot 232	678	268882	21.4	8.5813	0.6	5.1538	1.4	0.3209	1.2	0.91	1794.1	19.4	1845.0	11.6	1902.9	10.2	1902.9	10.2
M01 -Spot 272	673	766386	3.6	8.5292	0.8	5.5301	1.7	0.3422	1.5	0.88	1897.4	24.1	1905.3	14.3	1913.8	13.9	1913.8	13.9
M01 -Spot 262	452	2477980	1.7	8.4523	0.6	5.4476	1.3	0.3341	1.1	0.86	1858.2	17.6	1892.4	10.8	1930.1	11.4	1930.1	11.4
M01 -Spot 121	124	64450	3.6	8.3463	0.6	5.8153	1.0	0.3522	0.8	0.79	1945.0	13.7	1948.7	8.9	1952.6	11.2	1952.6	11.2
M01 -Spot 203	159	104353	0.5	7.9628	0.7	6.1717	1.2	0.3566	1.0	0.85	1966.0	17.7	2000.4	10.7	2036.3	11.5	2036.3	11.5
M01 -Spot 229	358	1540453	3.2	7.6105	0.7	6.3504	1.3	0.3507	1.1	0.84	1937.8	17.8	2025.4	11.1	2115.9	11.9	2115.9	11.9
M01 -Spot 56	176	627046	2.4	7.5745	0.6	6.2420	1.3	0.3431	1.1	0.86	1901.4	18.2	2010.4	11.2	2124.2	11.3	2124.2	11.3
M01 -Spot 276	653	1134070	1.9	7.5177	0.6	7.2919	1.1	0.3978	1.0	0.87	2158.7	18.4	2147.8	10.3	2137.4	9.9	2137.4	9.9
M01 -Spot 241	596	281845	1.3	7.4709	0.7	7.1802	1.5	0.3892	1.3	0.87	2119.2	23.8	2134.0	13.4	2148.3	12.9	2148.3	12.9
M01 -Spot 186	314	93033	1.9	7.3484	0.8	7.6404	1.3	0.4074	1.0	0.78	2202.9	19.0	2189.6	11.7	2177.2	14.0	2177.2	14.0
M01 -Spot 199	252	218537	1.8	7.2930	0.8	7.5399	1.6	0.3990	1.4	0.88	2164.4	25.9	2177.7	14.4	2190.3	13.3	2190.3	13.3
M01 -Spot 4	2472	140533	13.6	6.9251	0.6	7.5592	1.4	0.3798	1.2	0.88	2075.5	21.3	2180.0	12.2	2279.9	11.1	2279.9	11.1
M01 -Spot 96	382	163167	0.7	6.9090	0.8	8.0114	1.5	0.4016	1.2	0.84	2176.5	23.0	2232.3	13.4	2283.9	13.8	2283.9	13.8
M01 -Spot 279	1473	1109991	8.9	6.8709	0.7	7.1583	1.9	0.3569	1.8	0.93	1967.3	29.8	2131.3	16.8	2293.4	11.9	2293.4	11.9
M01 -Spot 252	131	62955	1.2	6.8524	0.7	8.0824	1.8	0.4019	1.6	0.92	2177.6	30.0	2240.3	16.0	2298.1	12.0	2298.1	12.0
M01 -Spot 89	1194	271574	59.7	6.8023	1.0	8.7182	1.7	0.4303	1.4	0.82	2307.1	26.2	2309.0	15.1	2310.7	16.3	2310.7	16.3
M01 -Spot 98	208	145947	1.9	6.7486	1.0	8.3571	1.4	0.4092	1.0	0.70	2211.4	18.5	2270.5	12.8	2324.3	17.4	2324.3	17.4
M01 -Spot 62	1367	1166242	15.0	6.7318	0.7	9.3047	1.2	0.4545	1.0	0.80	2415.2	19.8	2368.5	11.3	2328.5	12.8	2328.5	12.8
M01 -Spot 110	429	441487	3.6	6.7047	0.7	7.0170	1.3	0.3414	1.1	0.84	1893.2	18.1	2113.6	11.6	2335.4	12.0	2335.4	12.0
M01 -Spot 37	1441	404110	2.8	6.6811	0.8	9.2250	1.3	0.4472	1.0	0.79	2382.8	20.8	2360.6	12.0	2341.5	13.6	2341.5	13.6
M01 -Spot 275	1613	452539	6.7	6.5969	0.7	9.4510	1.1	0.4524	0.9	0.76	2405.8	17.2	2382.8	10.3	2363.1	12.3	2363.1	12.3
M01 -Spot 294	514	219669	5.2	6.5653	0.7	8.1497	1.2	0.3882	1.0	0.82	2114.6	18.4	2247.8	11.2	2371.3	12.1	2371.3	12.1
M01 -Spot 78	921	1691537	1.4	6.4769	0.6	9.6106	1.4	0.4517	1.2	0.89	2402.6	24.7	2398.2	12.8	2394.4	10.9	2394.4	10.9
M01 -Spot 207	1522	441958	2.0	6.4277	0.8	9.9150	1.3	0.4624	1.0	0.80	2450.2	21.1	2426.9	11.9	2407.4	13.0	2407.4	13.0
M01 -Spot 7	580	317919	1.6	6.3939	0.6	9.5311	1.1	0.4422	0.9	0.83	2360.4	17.8	2390.5	9.9	2416.3	10.2	2416.3	10.2
M01 -Spot 125	951	839025	0.6	6.3365	0.8	9.8171	1.3	0.4514	1.1	0.81	2401.3	21.3	2417.8	12.1	2431.6	13.1	2431.6	13.1
M01 -Spot 201	508	193168	1.4	6.3253	0.6	9.5884	1.3	0.4401	1.1	0.86	2350.9	21.5	2396.1	11.6	2434.6	10.8	2434.6	10.8
M01 -Spot 152	807	1069004	1.6	6.2956	0.6	9.7671	1.1	0.4462	1.0	0.84	2378.2	19.1	2413.0	10.5	2442.6	10.3	2442.6	10.3
M01 -Spot 131	446	75458	1.6	6.2792	0.8	9.5957	1.5	0.4372	1.2	0.85	2338.0	24.3	2396.8	13.4	2447.0	12.8	2447.0	12.8
M01 -Spot 2	149	111802	1.5	6.2440	0.8	10.2880	1.4	0.4661	1.1	0.82	2466.4	23.4	2461.0	13.0	2456.5	13.6	2456.5	13.6
M01 -Spot 312	154	71366	1.4	6.2266	0.5	9.6719	1.1	0.4370	1.0	0.89	2337.1	19.8	2404.0	10.5	2461.3	8.9	2461.3	8.9
M01 -Spot 310	90	40096	1.2	6.1858	0.6	10.5107	1.3	0.4718	1.2	0.89	2491.2	24.3	2480.9	12.2	2472.4	10.1	2472.4	10.1
M01 -Spot 55	198	314750	1.7	6.1794	0.8	10.5595	1.2	0.4735	1.0	0.78	2498.7	20.1	2485.2	11.6	2474.1	13.3	2474.1	13.3
M01 -Spot 177	323	267508	1.6	6.1747	0.6	10.4157	1.1	0.4667	0.9	0.82	2468.9	18.6	2472.4	10.2	2475.4	10.6	2475.4	10.6
M01 -Spot 33	59	49021	1.0	6.1650	0.6	10.2344	1.2	0.4578	1.0	0.85	2429.9	20.1	2456.2	10.8	2478.0	10.4	2478.0	10.4
M01 -Spot 63	327	212957	2.7	6.1629	0.6	10.1444	1.5	0.4536	1.4	0.92	2411.4	28.0	2448.0	14.0	2478.6	10.2	2478.6	10.2
M01 -Spot 139	568	122108	1.3	6.1539	0.7	9.9306	1.2	0.4434	1.1	0.85	2365.9	21.0	2428.4	11.5	2481.1	11.1	2481.1	11.1
M01 -Spot 140	852	781597	3.4	6.1537	0.5	9.8653	0.8	0.4405	0.7	0.83	2352.8	13.8	2422.3	7.7	2481.1	7.8	2481.1	7.8
M01 -Spot 292	208	297749	1.6	6.1413	0.6	9.5914	1.1	0.4274	0.9	0.85	2294.0	18.0	2396.3	10.1	2484.5	9.7	2484.5	9.7
M01 -Spot 189	441	300794	2.0	6.0905	0.5	10.6757	1.1	0.4718	1.0	0.90	2491.4	20.6	2495.3	10.3	2498.5	8.4	2498.5	8.4
M01 -Spot 65	217	163069	0.7	6.0806	0.5	10.0996	1.2	0.4456	1.0	0.91	2375.6	20.8	2443.9	10.7	2501.3	8.2	2501.3	8.2
M01 -Spot 238	228	232436	1.6	6.0710	0.6	10.6523	1.2	0.4692	1.1	0.86	2480.2	21.9	2493.3	11.5	2503.9	10.7	2503.9	10.7
M01 -Spot 9	206	5248792	1.7	6.0130	0.8	10.8804	1.6	0.4747	1.4	0.88	2504.1	29.4	2513.0	15.0	2520.1	12.9	2520.1	12.9
M01 -Spot 184	54	52629	1.0	5.8704	0.6	10.9042	1.2	0.4645	1.0	0.88	2459.2	21.4	2515.0	11.1	2560.3	9.7	2560.3	9.7
M01 -Spot 16	950	1034240	1.0	5.8585	0.6	12.0101	1.2	0.5105	1.0	0.86	2658.9	22.2	2605.2	11.2	2563.7	10.3	2563.7	10.3
M01 -Spot 119	1656	462621	2.9	5.8260	0.7	11.3302	1.3	0.4790	1.1	0.86	2522.7	23.1	2550.7	12.0	2573.0	10.9	2573.0	10.9
M01 -Spot 6	284	366478	1.4	5.4391	0.7	12.9779	1.5	0.5122	1.4	0.90	2665.9	30.5	2678.1	14.6	2687.2	10.9	2687.2	10.9
M01 -Spot 235	103	115965	2.1	5.2744	0.6	13.4520	1.1	0.5148	1.0	0.87	2677.1	21.9	2711.9	10.8	2737.9	9.2	2737.9	9.2
M01 -Spot 247	513	334104	1.8	5.2367	0.7	14.1259	1.4	0.5367	1.2	0.85	2769.8	26.3	2758.2	13.0	2749.7	11.7	2749.7	11.7
M01 -Spot 270	339	559119	6.9	5.0043	0.5	12.7781	1.3	0.4640	1.2	0.92	2457.1	25.0	2663.4	12.5	2824.1	8.5	2824.1	8.5
M01 -Spot 200	213	364357	2.1	4.4421	0.6	17.7489	1.3	0.5721	1.1	0.86	2916.3	25.2	2976.2	12.0	3016.9	10.3	3016.9	10.3
M01 -Spot 198	105	53883	1.7	4.2708	0.7	19.7650	1.1	0.6125	0.9	0.78	3080.0	21.1	3079.9	10.7	3079.9	11.1	3079.9	11.1
M01 -Spot 136	535	218589	1.6	4.0692	0.7	21.0317	1.2	0.6210	0.9	0.79	3113.8	23.2	3140.1	11.6	3156.9	11.6	3156.9	11.6

Table _____. U-Pb geochronologic analyses.

J03 Brahmaputra R.						Isotope ratios							Apparent ages (Ma)					
Analysis	U (ppm)	206Pb 204Pb	U/Th	206Pb* 207Pb*	± (%)	207Pb* 235U*	± (%)	206Pb* 238U	± (%)	error corr.	206Pb* 238U*	± (Ma)	207Pb* 235U	± (Ma)	206Pb* 207Pb*	± (Ma)	Best age (Ma)	± (Ma)
-Spot 60	2020	2879	37.3	23.2369	4.1	0.0053	4.3	0.0009	1.2	0.28	5.8	0.1	5.4	0.2	NA	NA	5.8	0.1
-Spot 153	295	606	1.7	2.9549	2.0	0.1096	2.6	0.0023	1.7	0.66	15.1	0.3	105.6	2.6	3655.0	30.0	15.1	0.3
-Spot 296	259	1270	0.5	3.8682	3.6	0.0860	4.4	0.0024	2.6	0.58	15.5	0.4	83.8	3.6	3237.0	57.1	15.5	0.4
-Spot 44	445	2423	69.9	24.8991	2.9	0.0140	3.3	0.0025	1.6	0.48	16.3	0.3	14.2	0.5	NA	NA	16.3	0.3
-Spot 302	3015	30814	9.7	21.7391	1.0	0.0177	1.5	0.0028	1.2	0.77	18.0	0.2	17.8	0.3	NA	NA	18.0	0.2
-Spot 100	4436	319649	0.6	20.3125	1.2	0.0194	1.8	0.0029	1.3	0.75	18.4	0.2	19.5	0.3	157.8	27.5	18.4	0.2
-Spot 108	104	425	0.9	101.9907	190.0	0.0048	190.0	0.0036	1.3	0.01	22.9	0.3	4.9	9.2	NA	NA	22.9	0.3
-Spot 131	4333	15198	1.4	19.3809	1.5	0.0265	2.1	0.0037	1.4	0.68	24.0	0.3	26.6	0.5	266.6	34.8	24.0	0.3
-Spot 99	4436	47232	2.9	21.2514	0.6	0.0281	1.4	0.0043	1.3	0.92	27.9	0.4	28.1	0.4	51.0	13.6	27.9	0.4
-Spot 51	192	813	0.9	2.8010	4.0	0.2135	5.7	0.0043	4.0	0.71	27.9	1.1	196.5	10.1	3736.6	60.8	27.9	1.1
-Spot 312	556	5366	2.0	22.0628	2.1	0.0281	2.5	0.0045	1.2	0.49	28.9	0.3	28.1	0.7	NA	NA	28.9	0.3
-Spot 236	4436	67328	83.5	20.4085	0.8	0.0311	1.4	0.0046	1.1	0.81	29.6	0.3	31.1	0.4	146.8	19.5	29.6	0.3
-Spot 222	1992	24498	1.9	21.4615	1.2	0.0311	1.8	0.0048	1.3	0.73	31.2	0.4	31.1	0.5	27.5	29.2	31.2	0.4
-Spot 61	2539	24672	22.8	21.5053	1.4	0.0328	1.9	0.0051	1.3	0.66	32.9	0.4	32.8	0.6	22.6	34.1	32.9	0.4
-Spot 33	918	20999	1.5	19.4620	1.9	0.0400	2.1	0.0056	1.0	0.48	36.3	0.4	39.8	0.8	257.0	43.0	36.3	0.4
-Spot 284	750	8669	60.4	21.0963	1.9	0.0376	2.2	0.0058	1.0	0.48	37.0	0.4	37.5	0.8	68.5	45.2	37.0	0.4
-Spot 171	974	100761	2.4	21.3290	1.3	0.0389	1.6	0.0060	0.9	0.58	38.7	0.4	38.7	0.6	42.3	30.7	38.7	0.4
-Spot 253	752	10958	5.1	20.7716	1.3	0.0495	1.9	0.0075	1.4	0.72	47.9	0.7	49.1	0.9	105.2	31.5	47.9	0.7
-Spot 186	2139	30517	8.3	21.2865	1.0	0.0488	1.6	0.0075	1.3	0.78	48.4	0.6	48.4	0.8	47.1	24.0	48.4	0.6
-Spot 88	1445	19523	5.3	21.1869	1.0	0.0506	1.7	0.0078	1.3	0.78	49.9	0.6	50.1	0.8	58.3	24.9	49.9	0.6
-Spot 130	564	3734	2.2	23.0279	1.3	0.0476	1.9	0.0079	1.3	0.69	51.0	0.7	47.2	0.9	NA	NA	51.0	0.7
-Spot 167	1097	7724	17.4	21.8350	1.1	0.0510	1.5	0.0081	1.1	0.71	51.9	0.6	50.5	0.8	NA	NA	51.9	0.6
-Spot 28	144	3147	2.7	23.6784	4.5	0.0501	4.8	0.0086	1.5	0.32	55.3	0.8	49.7	2.3	NA	NA	55.3	0.8
-Spot 10	330	2299	1.6	24.1034	1.7	0.0494	2.2	0.0086	1.3	0.61	55.5	0.7	49.0	1.0	NA	NA	55.5	0.7
-Spot 136	133	68322	2.9	21.0203	3.1	0.0580	3.5	0.0089	1.6	0.47	56.8	0.9	57.3	1.9	77.0	73.2	56.8	0.9
-Spot 214	2139	42960	42.5	21.1239	0.9	0.0578	1.3	0.0089	0.9	0.71	56.8	0.5	57.0	0.7	65.4	22.6	56.8	0.5
-Spot 227	668	10779	1.4	21.2103	1.3	0.0584	1.8	0.0090	1.3	0.69	57.7	0.7	57.7	1.0	55.6	31.5	57.7	0.7
-Spot 203	275	4207	2.4	19.6435	2.7	0.0637	2.9	0.0091	1.1	0.39	58.3	0.7	62.7	1.8	235.6	61.9	58.3	0.7
-Spot 187	66	7007	1.3	20.3757	2.8	0.0617	3.2	0.0091	1.6	0.50	58.5	0.9	60.8	1.9	150.6	64.6	58.5	0.9
-Spot 120	142	2877	1.6	23.7193	3.8	0.0610	4.0	0.0105	1.4	0.34	67.3	0.9	60.1	2.3	NA	NA	67.3	0.9
-Spot 237	3317	12480	0.4	17.9163	1.2	0.0841	1.6	0.0109	1.1	0.67	70.1	0.7	81.9	1.2	444.0	25.7	70.1	0.7
-Spot 83	338	2853	1.1	22.2139	1.6	0.0708	2.2	0.0114	1.5	0.68	73.2	1.1	69.5	1.5	NA	NA	73.2	1.1
-Spot 188	409	30099	3.8	21.2895	1.1	0.0828	1.6	0.0128	1.2	0.72	81.9	0.9	80.7	1.3	46.7	27.2	81.9	0.9
-Spot 202	261	5292	5.9	21.5035	3.3	0.0833	3.6	0.0130	1.5	0.43	83.3	1.3	81.3	2.8	22.8	78.8	83.3	1.3
-Spot 78	1721	46665	19.2	15.8630	1.0	0.1167	2.3	0.0134	2.1	0.90	86.0	1.8	112.0	2.5	708.7	21.8	86.0	1.8
-Spot 151	315	55973	2.0	20.8705	1.2	0.0900	1.9	0.0136	1.4	0.76	87.3	1.2	87.5	1.6	94.0	28.9	87.3	1.2
-Spot 275	127	7981	1.4	21.6560	1.9	0.0876	2.1	0.0138	1.0	0.46	88.1	0.9	85.3	1.8	5.8	45.9	88.1	0.9
-Spot 213	96	23634	2.4	20.4775	1.8	0.1020	2.3	0.0152	1.4	0.64	96.9	1.4	98.6	2.1	138.9	41.1	96.9	1.4
-Spot 162	32	79327	47.7	18.7797	3.9	0.1141	4.1	0.0155	1.2	0.30	99.4	1.2	109.7	4.3	338.4	88.4	99.4	1.2
-Spot 102	95	1694	1.6	23.8309	2.6	0.0962	2.9	0.0166	1.2	0.43	106.4	1.3	93.3	2.6	NA	NA	106.4	1.3
-Spot 18	415	12737	1.5	20.8184	1.5	0.1125	2.2	0.0170	1.7	0.76	108.7	1.8	108.3	2.3	99.9	34.5	108.7	1.8
-Spot 13	1582	458310	3.6	20.4742	0.9	0.1150	1.4	0.0171	1.1	0.79	109.2	1.2	110.6	1.5	139.2	20.1	109.2	1.2
-Spot 314	2917	101932	2.3	20.5136	0.6	0.1194	1.1	0.0178	0.9	0.84	113.6	1.0	114.5	1.2	134.7	14.1	113.6	1.0
-Spot 139	350	7546	8.7	20.8911	1.9	0.1187	2.1	0.0180	1.1	0.51	115.0	1.2	113.9	2.3	91.7	43.9	115.0	1.2
-Spot 155	166	2876	1.5	23.3149	2.7	0.1065	3.0	0.0180	1.4	0.47	115.1	1.6	102.7	3.0	NA	NA	115.1	1.6
-Spot 158	1147	62294	1.3	20.7308	0.9	0.1207	1.5	0.0182	1.2	0.80	116.0	1.4	115.7	1.6	109.9	20.9	116.0	1.4
-Spot 265	629	11877	1.7	20.5385	1.1	0.1235	1.7	0.0184	1.2	0.74	117.6	1.4	118.3	1.9	131.9	26.7	117.6	1.4
-Spot 288	340	8813	0.8	18.0525	1.7	0.1420	2.2	0.0186	1.4	0.64	118.8	1.6	134.8	2.7	427.2	37.2	118.8	1.6
-Spot 48	83	2311	1.3	24.4896	2.4	0.1052	2.8	0.0187	1.4	0.50	119.3	1.6	101.5	2.7	NA	NA	119.3	1.6
-Spot 67	1466	50691	3.6	20.2581	0.8	0.1305	1.2	0.0192	1.0	0.78	122.4	1.2	124.5	1.5	164.1	18.1	122.4	1.2
-Spot 229	342	74087	0.2	20.2465	1.3	0.1322	1.8	0.0194	1.2	0.67	124.0	1.5	126.0	2.1	165.4	30.5	124.0	1.5
-Spot 298	371	53036	0.8	19.7324	1.1	0.1393	1.6	0.0199	1.1	0.70	127.3	1.4	132.4	2.0	225.2	26.5	127.3	1.4
-Spot 141	115	9370	1.1	20.2567	1.8	0.1406	2.1	0.0207	1.1	0.50	131.8	1.4	133.6	2.7	164.2	43.2	131.8	1.4
-Spot 244	219	96982	1.8	18.2167	1.7	0.1653	2.0	0.0219	1.1	0.56	139.4	1.6	155.4	2.9	406.9	37.4	139.4	1.6
-Spot 128	15	601	0.4	3.6163	3.0	0.9421	5.0	0.0247	3.9	0.79	157.4	6.1	674.0	24.4	3342.7	47.4	157.4	6.1
-Spot 53	23	703	4.4	33.6312	4.2	0.1018	4.6	0.0248	1.9	0.40	158.2	2.9	98.5	4.4	NA	NA	158.2	2.9
-Spot 106	141	12541	1.5	15.7152	5.5	0.2272	5.7	0.0259	1.7	0.30	164.9	2.8	207.9	10.8	728.5	115.9	164.9	2.8
-Spot 285	929	32031	3.2	19.9171	0.5	0.2323	1.2	0.0336	1.1	0.92	212.8	2.3	212.1	2.3	203.6	11.1	212.8	2.3
-Spot 39	230	29661	2.4	17.5097	1.2	0.2852	1.8	0.0362	1.3	0.73	229.5	2.9	254.8	4.0	494.9	26.6	229.5	2.9
-Spot 19	474	16199	0.9	17.8853	1.2	0.2823	1.9	0.0366	1.5	0.79	231.9	3.4	252.5	4.3	447.9	26.2	231.9	3.4
-Spot 45	4233	2688	10.2	10.7017	3.7	0.5266	4.0	0.0409	1.4	0.37	258.4	3.7	429.6	13.9	1496.1	69.7	258.4	3.7
-Spot 248	1087	63875	23.8	6.3378	1.5	1.0012	3.6	0.0460	3.3	0.91	290.2	9.3	704.4	18.3	2431.3	25.1	290.2	9.3
-Spot 172	536	77403	7.5	16.9120	1.7	0.3837	2.9	0.0471	2.4	0.82	296.6	7.0	329.7	8.3	570.9	36.1	296.6	7.0
-Spot 58	396	7532	3.8	10.5823	5.9	0.6496	6.8	0.0499	3.4	0.50	313.8	10.5	508.2	27.3	1517.2	111.4	313.8	10.5
-Spot 81	1601	50530	18.9	17.7824	0.9	0.3978	1.8	0.0513	1.6	0.87	322.7	4.9	340.1	5.1	460.7	19.3	322.7	4.9
-Spot 247	1860	141835	15.3	18.3261	0.8	0.4302	1.4	0.0572	1.2	0.85	358.6	4.3	363.3	4.4	393.5	17.1	358.6	4.3
-Spot 233	1185	132682	3.9	17.3656	0.8	0.4722	1.3	0.0595	1.1	0.82	372.5	4.0	392.7	4.4	513.1	17.1	372.5	4.0
-Spot 138	3937	76436	16.5	17.2311	0.8	0.4931	1.6	0.0616	1.3	0.86	385.6	5.0	407.0	5.3	530.1	17.7	385.6	5.0
-Spot 144	146	22138	2.1	14.9792	1.1	0.5739	2.4	0.0624	2.2	0.90	390.0	8.2	460.5	8.9	829.3	22.0	390.0	8.2
-Spot 110	689	78201	2.9	17.7278	0.8	0.4964	1.5	0.0639	1.2	0.84	399.0	4.8	409.3	5.0	467.5	17.7	399.0	4.8
-Spot 84	4436	1482724	0.6	17.8453	1.2	0.5078	4.6	0.0658	4.5	0.97	410.5	17.9	417.0	15.9	452.9	25.7	410.5	17.9
-Spot 177	71	5040	1.1	17.4188	1.5	0.5282	2.5	0.0668	1.9	0.79	416.6	7.9	430.6	8.7	506.3	33.5	416.6	7.9
-Spot 190	1033	109430	1.3	17.2989	0.9	0.5623	1.5	0.0706	1.1	0.78	439.7	4.9	453.0	5.3	521.5	19.8	439.7	4.9
-Spot 278	2019	4983431	137.2	17.0298	0.6	0.5900	1.3	0.0729	1.2	0.88	453.7	5.2	470.9	5.1	555.8	14.0	453.7	5.2
-Spot 175	1745	566102	11.1	17.3115	0.8	0.5893	1.3	0.0740	1.0	0.79	460.3	4.5	470.4	4.9	519.9	17.6	460.3	4.5
-Spot 85	1553	256985	1.6	17.0401	0.6	0.5996	1.3	0.0741	1.2	0.88	461.0	5.3	477.0	5.1	554.5	13.9	461.0	5.3
-Spot 308	265	25376	1.2	17.5360	0.6	0.5899	1.3	0.0751	1.1	0.86	466.6	4.8	470.8	4.7	491.6	14.3	466.6	4.8
-Spot 182	185	16268	1.1	17.8724	1.2	0.5814	1.8	0.0754	1.3	0.72	468.6	5.7	465.4	6.5	449.5	27.2	468.6	5.7
-Spot 62	2943	532429	6.5	17.7680	0.9	0.5857	2.0	0.0755	1.8	0.90	469.3	8.3	468.1	7.7	462.5	20.1	469.3	8.3
-Spot 16	1715	60805	2.2	17.2203	0.9	0.6075	1.5	0.0759	1.2	0.80	471.6	5.6	482.0	5.9	531.5	20.1	471.6	5.6
-Spot 189	411	65877	3.0	17.3156	0.6	0.6073	1.4	0.0763	1.2	0.88	474.0	5.6	481.8	5.3	519.4	14.1	474.0	5.6
-Spot 70	357	32069	1.4	17.5790	0.8	0.5987	1.6	0.0764	1.4	0.85	474.4	6.2	476.4	6.1	486.2	18.5	474.4	6.2
-Spot 280	632	104815	2.2	17.4194	0.7	0.6077	1.5	0.0768	1.3	0.88	477.1	5.9	482.1	5.6	506.3	15.5	477.1	5.9
-Spot 135	1080	76029	2.0	17.8012	0.8	0.5955	1.3	0.0769	1.0	0.80	477.7	4.7	474.4	4.8	458.3	16.8	477.7	4.7
-Spot 17	792	122187	1.9	17.4447	0.8	0.6083	1.6	0.0770	1.4	0.86	478.2	6.5	482.5	6.3	503.1	18.5	478.2	6.5
-Spot 173	69	36212	1.8	17.3665	1.2	0.6116	1.7	0.0771	1.2	0.72	478.6	5.7	484.6	6.7	512.9	26.6	478.6	5.7
-Spot 59	339	32941	1.1	17.7108	0.8	0.6016	1.8	0.0773	1.6	0.89	480.0	7.5	478.2	6.9	469.6	18.3	480.0	7.5
-Spot 93	971	210813	14.5	17.5176	0.7	0.6082	1.5	0.0773	1.3	0.87	480.1	5.9	482.5	5.6	493.9	15.7	480.1	5.9
-Spot 82	271	26815	1.1	17.5762	1.0	0.6084	1.6	0.0776	1.2	0.78	481.7	5.6	482.5	6.0	486.5	21.7	481.7	5.6
-Spot 242	3123	916211	1.2	17.7330	0.9	0.6064	1.6	0.0780	1.4	0.84	484.3	6.5	481.3	6.3	466.9	19.6	484.3	6.5
-Spot 191	3222	476329	15.4	17.5179	0.7	0.6138	1.2	0.0780	1.0	0.83	484.3	4.7	486.0	4.7	493.8	15.0	484.3	4.7
-Spot 299	2870	3884769	2.3	17.6850	0.6	0.6091	1.1	0.0782	0.9	0.83	485.1	4.4	483.0	4.4	472.9	14.2	485.1	4.4
-Spot 47	2512	75571	1.8	17.3117	0.9	0.6270	1.5	0.0788	1.2	0.82	488.7	5.8	494.2	5.9	519.9	19.0	488.7	5.8
-Spot 5	2264	117860	3.1	17.3775	0.7	0.6250	1.3	0.0788	1.1	0.83	489.0	5.1	493.0	5.1	511.5	16.2	489.0	5.1

-Spot 260	1512	1100305	2.5	17.5157	0.7	0.6201	1.7	0.0788	1.6	0.91	489.0	7.4	489.9	6.7	494.1	16.2	489.0	7.4
-Spot 283	868	65636	3.7	17.5199	0.6	0.6242	1.4	0.0794	1.3	0.90	492.3	5.9	492.5	5.4	493.6	13.1	492.3	5.9
-Spot 55	240	30810	3.8	17.4618	1.0	0.6324	1.4	0.0801	1.0	0.69	496.9	4.6	497.6	5.5	500.9	22.4	496.9	4.6
-Spot 90	799	920799	1.3	17.3420	0.7	0.6370	1.4	0.0802	1.2	0.87	497.0	5.7	500.4	5.4	516.0	14.7	497.0	5.7
-Spot 91	948	81261	1.8	17.2005	0.5	0.6439	1.0	0.0804	0.9	0.85	498.3	4.3	504.7	4.2	534.0	12.0	498.3	4.3
-Spot 300	1716	336945	21.4	17.4499	0.4	0.6359	1.0	0.0805	0.9	0.91	499.2	4.4	499.8	3.9	502.4	9.3	499.2	4.4
-Spot 204	426	30998	6.2	17.3074	0.8	0.6425	1.5	0.0807	1.2	0.84	500.2	5.9	503.9	5.8	520.4	17.5	500.2	5.9
-Spot 37	1003	124384	28.5	17.4806	0.8	0.6364	1.3	0.0807	1.0	0.78	500.4	4.8	500.1	5.1	498.5	17.8	500.4	4.8
-Spot 290	319	28572	2.2	17.4895	0.7	0.6382	1.1	0.0810	0.8	0.73	502.0	3.8	501.2	4.3	497.4	16.2	502.0	3.8
-Spot 66	460	35440	2.1	17.6188	0.7	0.6335	1.3	0.0810	1.1	0.84	502.0	5.4	498.3	5.2	481.2	16.0	502.0	5.4
-Spot 134	3520	868837	8.7	17.5686	0.7	0.6410	1.6	0.0817	1.4	0.89	506.4	6.7	502.9	6.2	487.5	16.0	506.4	6.7
-Spot 184	264	373327	1.5	17.1061	0.8	0.6591	1.5	0.0818	1.2	0.83	506.9	5.9	514.1	5.9	546.0	18.1	506.9	5.9
-Spot 251	530	71979	3.4	17.4240	0.9	0.6480	1.5	0.0819	1.1	0.76	507.6	5.5	507.2	5.9	505.7	20.9	507.6	5.5
-Spot 261	629	435681	2.6	17.1948	0.7	0.6583	1.3	0.0821	1.2	0.86	508.9	5.6	513.6	5.4	534.7	14.7	508.9	5.6
-Spot 309	449	752148	2.2	17.3457	0.7	0.6554	1.3	0.0825	1.1	0.84	510.9	5.5	511.8	5.4	515.6	16.0	510.9	5.5
-Spot 291	2382	365351	3.4	17.3655	0.6	0.6561	1.0	0.0827	0.8	0.81	512.0	4.0	512.2	4.1	513.1	13.3	512.0	4.0
-Spot 98	854	70087	1.3	16.6843	0.7	0.6856	1.3	0.0830	1.1	0.84	514.0	5.4	530.1	5.4	600.3	15.2	514.0	5.4
-Spot 179	208	24038	4.1	17.4602	0.8	0.6602	1.4	0.0836	1.1	0.78	517.8	5.2	514.7	5.5	501.1	18.6	517.8	5.2
-Spot 125	929	1397096	1.6	17.3452	0.8	0.6651	1.4	0.0837	1.2	0.84	518.2	5.9	517.7	5.7	515.6	16.5	518.2	5.9
-Spot 220	876	77793	11.0	16.7971	0.9	0.7384	1.6	0.0900	1.3	0.83	555.5	7.0	561.5	6.9	585.8	19.3	555.5	7.0
-Spot 95	1442	577382	2.3	16.3570	0.7	0.8045	1.3	0.0955	1.1	0.87	587.9	6.4	599.4	5.9	643.1	14.1	587.9	6.4
-Spot 73	154	18103	2.6	16.9244	1.0	0.7789	1.7	0.0956	1.4	0.81	588.9	7.6	584.8	7.4	569.3	21.3	588.9	7.6
-Spot 197	373	648516	0.7	16.6439	0.7	0.8066	1.4	0.0974	1.2	0.86	599.2	6.8	600.6	6.3	605.6	15.5	599.2	6.8
-Spot 57	233	402581	2.8	15.1336	0.8	0.9943	1.5	0.1092	1.2	0.85	668.0	7.8	700.9	7.3	807.9	16.1	668.0	7.8
-Spot 268	290	24361	2.2	15.9497	0.6	0.9869	1.4	0.1142	1.2	0.89	697.2	8.1	697.1	7.0	697.1	13.2	697.2	8.1
-Spot 127	367	109877	0.6	15.8604	0.8	1.0116	1.5	0.1164	1.3	0.87	709.9	9.0	709.7	7.9	709.0	16.3	709.9	9.0
-Spot 240	1702	68884	1.0	15.1782	0.8	1.0933	1.4	0.1204	1.1	0.81	732.9	7.7	750.1	7.3	801.8	17.0	732.9	7.7
-Spot 132	2777	56520	48.9	14.8692	0.8	1.1391	1.4	0.1229	1.2	0.81	747.2	8.3	772.1	7.8	844.7	17.5	747.2	8.3
-Spot 193	561	102905	3.0	14.6329	0.6	1.2163	1.5	0.1291	1.4	0.91	783.0	10.3	808.1	8.6	877.9	13.4	783.0	10.3
-Spot 68	293	711011	2.2	14.6816	0.9	1.2179	1.8	0.1297	1.6	0.87	786.4	11.7	808.8	10.1	871.1	18.3	786.4	11.7
-Spot 305	502	142003	1.4	15.1055	0.7	1.2058	1.1	0.1322	0.8	0.78	800.2	6.3	803.3	5.9	811.8	13.8	800.2	6.3
-Spot 238	807	82666	2.2	14.2084	0.9	1.2903	1.8	0.1330	1.6	0.86	805.1	11.9	841.4	10.4	938.6	18.7	805.1	11.9
-Spot 232	88	12578	1.6	14.8629	0.9	1.2527	1.2	0.1351	0.9	0.72	816.9	6.9	824.6	7.0	845.6	18.1	816.9	6.9
-Spot 159	238	47279	3.0	15.0569	0.7	1.2410	1.6	0.1356	1.4	0.90	819.6	10.8	819.4	8.8	818.6	14.5	819.6	10.8
-Spot 269	425	63328	4.3	14.8226	0.7	1.2631	1.0	0.1358	0.8	0.75	821.2	5.9	829.3	5.8	851.2	14.1	821.2	5.9
-Spot 9	531	314647	2.2	14.6425	0.7	1.2802	1.5	0.1360	1.3	0.87	822.1	10.0	836.9	8.4	876.6	14.9	822.1	10.0
-Spot 241	136	29117	1.2	14.8410	0.8	1.2656	1.5	0.1363	1.3	0.84	823.6	9.9	830.4	8.6	848.7	17.0	823.6	9.9
-Spot 166	153	32192	2.0	14.2305	0.9	1.3214	1.6	0.1364	1.3	0.82	824.5	10.4	855.1	9.5	935.4	19.3	824.5	10.4
-Spot 87	472	59198	3.9	14.9656	0.7	1.2612	1.4	0.1369	1.2	0.85	827.4	9.4	828.4	8.1	831.3	15.6	827.4	9.4
-Spot 218	472	62596	8.7	14.6316	0.8	1.2943	1.6	0.1374	1.4	0.88	830.0	11.1	843.2	9.3	878.1	16.1	830.0	11.1
-Spot 306	669	1003406	2.4	15.0067	0.8	1.2639	1.3	0.1376	1.1	0.82	831.2	8.4	829.6	7.5	825.5	15.9	831.2	8.4
-Spot 178	678	109375	3.1	14.9746	0.7	1.2739	1.4	0.1384	1.2	0.88	835.7	9.7	834.1	8.0	830.0	14.0	835.7	9.7
-Spot 310	828	201493	14.3	14.2767	0.6	1.3951	1.1	0.1445	1.0	0.84	870.2	7.8	886.9	6.8	928.7	12.6	870.2	7.8
-Spot 146	462	76812	7.7	14.4452	0.7	1.4709	1.3	0.1542	1.1	0.84	924.3	9.2	918.5	7.7	904.6	14.1	904.6	14.1
-Spot 196	399	7987916	2.3	14.4343	0.7	1.4323	1.1	0.1500	0.9	0.81	901.0	7.7	902.5	6.7	906.2	13.5	906.2	13.5
-Spot 34	158	72115	1.0	13.9467	1.0	1.5808	1.5	0.1600	1.2	0.77	956.6	10.4	962.7	9.5	976.6	20.0	976.6	20.0
-Spot 234	372	73464	2.4	13.9050	1.1	1.4664	1.8	0.1480	1.4	0.78	889.5	11.4	916.7	10.6	982.7	22.4	982.7	22.4
-Spot 165	405	262257	1.6	13.8475	0.6	1.6526	1.0	0.1660	0.9	0.83	990.3	8.0	990.5	6.6	991.1	11.8	991.1	11.8
-Spot 77	193	58564	1.7	13.8179	0.7	1.7406	1.4	0.1745	1.2	0.87	1036.9	11.4	1023.7	8.8	995.5	13.5	995.5	13.5
-Spot 56	255	45052	1.8	13.8059	0.9	1.4598	1.6	0.1462	1.3	0.83	879.8	11.0	913.9	9.7	997.2	18.0	997.2	18.0
-Spot 174	121	22374	2.0	13.7760	0.8	1.5847	1.7	0.1584	1.5	0.89	947.9	13.3	964.2	10.5	1001.6	15.5	1001.6	15.5
-Spot 154	284	157467	1.6	13.7238	0.9	1.6159	1.7	0.1609	1.5	0.85	961.8	13.1	976.4	10.8	1009.3	18.4	1009.3	18.4
-Spot 206	554	348127	2.5	13.7114	0.9	1.5601	1.9	0.1552	1.7	0.88	930.1	14.7	954.5	12.0	1011.2	19.0	1011.2	19.0
-Spot 199	1509	729829	1.7	13.6647	0.8	1.6800	1.4	0.1666	1.1	0.83	993.2	10.4	1001.0	8.7	1018.1	15.4	1018.1	15.4
-Spot 198	657	63954	3.7	13.6348	0.7	1.7230	1.3	0.1705	1.1	0.86	1014.7	10.8	1017.2	8.5	1022.5	13.5	1022.5	13.5
-Spot 121	740	47739	1.5	13.5797	0.7	1.5136	1.3	0.1491	1.1	0.86	896.1	9.4	935.9	7.9	1030.7	13.4	1030.7	13.4
-Spot 169	208	33396	0.5	13.5699	0.9	1.8471	1.3	0.1819	1.0	0.74	1077.2	9.6	1062.4	8.6	1032.2	17.7	1032.2	17.7
-Spot 273	361	91119	3.1	13.4128	0.7	1.8123	1.4	0.1764	1.3	0.89	1047.1	12.1	1049.9	9.2	1055.7	13.1	1055.7	13.1
-Spot 41	330	37450	1.9	13.4042	0.8	1.7042	1.5	0.1657	1.3	0.84	988.6	11.8	1010.1	9.8	1057.0	17.0	1057.0	17.0
-Spot 257	981	91086	7.8	13.3180	0.6	1.8560	1.4	0.1794	1.2	0.90	1063.4	12.0	1065.6	8.9	1070.0	11.6	1070.0	11.6
-Spot 21	452	72380	5.7	13.2978	0.9	1.4863	1.5	0.1434	1.2	0.82	863.9	9.8	924.8	9.0	1073.0	17.1	1073.0	17.1
-Spot 143	712	308137	1.8	13.2676	0.7	1.8508	1.4	0.1782	1.2	0.87	1057.0	12.0	1063.7	9.3	1077.5	14.1	1077.5	14.1
-Spot 228	705	129562	1.6	13.2425	0.7	1.6988	1.3	0.1632	1.1	0.84	974.7	10.1	1008.1	8.5	1081.3	14.3	1081.3	14.3
-Spot 292	767	124686	20.3	13.2098	0.6	1.8799	1.2	0.1802	1.1	0.88	1068.0	10.7	1074.0	8.2	1086.3	11.9	1086.3	11.9
-Spot 212	33	5416	0.5	13.1652	1.2	1.9617	1.5	0.1874	0.9	0.62	1107.2	9.3	1102.5	10.0	1093.1	23.4	1093.1	23.4
-Spot 38	348	79462	1.5	13.1188	0.8	1.9368	1.6	0.1844	1.3	0.85	1090.7	13.4	1093.9	10.4	1100.2	16.2	1100.2	16.2
-Spot 254	1072	112911	1.7	13.0609	0.7	1.9360	1.3	0.1835	1.1	0.84	1085.9	10.9	1093.6	8.7	1109.0	14.1	1109.0	14.1
-Spot 180	790	1224496	12.9	13.0313	1.3	1.8853	1.8	0.1783	1.2	0.70	1057.5	12.2	1075.9	11.8	1113.5	25.2	1113.5	25.2
-Spot 6	398	95297	11.7	13.0174	0.9	1.6231	1.4	0.1533	1.1	0.76	919.5	9.1	979.2	8.8	1115.7	18.0	1115.7	18.0
-Spot 207	572	86017	7.5	13.0052	0.7	1.8768	1.3	0.1771	1.1	0.82	1051.1	10.3	1072.9	8.6	1117.5	14.8	1117.5	14.8
-Spot 243	235	66022	1.1	12.9989	0.7	1.9662	1.4	0.1855	1.2	0.86	1096.7	11.7	1104.0	9.1	1118.5	13.8	1118.5	13.8
-Spot 4	1398	114861	1.8	12.9528	0.7	1.9049	1.5	0.1790	1.3	0.87	1061.7	12.7	1082.8	9.9	1125.6	14.7	1125.6	14.7
-Spot 209	538	35218	2.8	12.9336	0.7	1.8209	1.5	0.1709	1.3	0.88	1017.0	12.5	1053.0	9.9	1128.5	14.1	1128.5	14.1
-Spot 122	105	21123	0.4	12.9323	0.9	2.1281	1.3	0.1997	1.0	0.75	1173.6	10.4	1158.0	9.0	1128.8	17.2	1128.8	17.2
-Spot 133	125	53149	0.7	12.8903	0.7	1.9719	1.2	0.1844	1.0	0.79	1091.1	9.8	1106.0	8.3	1135.2	14.9	1135.2	14.9
-Spot 185	1008	321226	2.5	12.8664	0.7	2.0210	1.3	0.1887	1.1	0.85	1114.2	11.1	1122.6	8.6	1138.9	13.1	1138.9	13.1
-Spot 129	93	16575	1.2	12.8372	0.9	2.0439	1.4	0.1904	1.1	0.76	1123.4	11.4	1130.3	9.8	1143.4	18.5	1143.4	18.5
-Spot 271	72	12114	1.8	12.8294	0.8	2.0030	1.4	0.1865	1.1	0.80	1102.1	11.2	1116.5	9.3	1144.6	16.2	1144.6	16.2
-Spot 266	72	192207	0.7	12.7678	0.9	2.0552	1.4	0.1904	1.0	0.74	1123.5	10.4	1134.0	9.2	1154.2	17.9	1154.2	17.9
-Spot 231	75	45609	1.3	12.7607	1.0	2.1033	1.3	0.1947	0.9	0.66	1147.0	9.3	1149.9	9.2	1155.3	19.8	1155.3	19.8
-Spot 145	738	458138	2.0	12.7559	0.6	2.1523	1.1	0.1992	0.9	0.84	1171.0	10.1	1165.8	7.8	1156.0	12.3	1156.0	12.3
-Spot 3	271	117826	0.8	12.7236	1.0	2.0533	1.8	0.1896	1.5	0.85	1119.0	15.7	1133.4	12.3	1161.0	19.1	1161.0	19.1
-Spot 96	416	360704	2.1	12.6996	0.7	2.0137	1.5	0.1856	1.4	0.89	1097.2	13.8	1120.1	10.5	1164.8	14.2	1164.8	14.2
-Spot 116	177	39127	1.1	12.6696	1.0	2.2342	1.4	0.2054	1.1	0.74	1204.2	11.7	1191.8	10.1	1169.5	19.0	1169.5	19.0
-Spot 7	102	18078	1.9	12.6606	0.9	2.1597	1.5	0.1984	1.3	0.81	1166.7	13.4	1168.2	10.7	1170.9	17.9	1170.9	17.9
-Spot 79	239	67778	2.1	12.6189	0.7	2.1257	1.5	0.1946	1.3	0.87	1146.4	13.4	1157.2	10.2	1177.4	14.5	1177.4	14.5
-Spot 221	727	114846	1.5	12.5762	0.7	2.1938	1.3	0.2002	1.1	0.87	1176.3	12.2	1179.1	9.1	1184.1	12.9	1184.1	12.9
-Spot 211	2250	541077	3.0	12.5468	0.9	2.2862	1.4	0.2081	1.1	0.80	1218.9	12.8	1208.0	10.1	1188.8	17.0	1188.8	17.0
-Spot 20	669	106561	1.3	12.5327	0.8	2.0392	1.3	0.1854	1.0	0.78	1096.6	10.5	1128.7	9.1	1191.0	16.5	1191.0	16.5
-Spot 160	196	184424	1.3	12.5140	0.9	2.0153	1.7	0.1830	1.4	0.86	1083.3	14.3	1120.7	11.4	1193.9	17.1	1193.9	17.1
-Spot 80	170	33987	0.5	12.5010	0.6	2.1415	1.4	0.1942	1.3	0.89	1144.3	13.2	1162.3	9.8	1196.0	12.6	1196.0	12.6
-Spot 74	181	70586	10.3	12.4462	0.7	2.0658	1.3	0.1866	1.1	0.84	1102.7	11.0	1137.6	8.9	1204.6	14.1	1204.6	14.1
-Spot 226	200	55807	3.6	12.4314	0.8	2.1456	1.5	0.1935	1.2	0.83	1140.5	12.6	1163.6	10.1	1207.0	15.9	1207.0	15.9
-Spot 23	374	30444	1.8	12.3124	0.9	2.1503	1.4	0.1921	1.1	0.78	1132.8	11.1	1165.2	9.5	1225.9	16.8	1225.9	16.8
-Spot 277	249	33863	2.1	12.2910	0.8	1.9508	3.3	0.1740	3.2	0.97	1034.0	30.3	1098.7	21.9	1229.3	14.8	1229.3	14.8
-Spot 35	257	123458	1.5	12.2730	0.8	2.3517	1.5	0.2094	1.3	0.86	1225.7	14.0	1228.1	10.4	1232.2	14.8	1232.2	14.8
-Spot 224	686	120851	3.9	12.2236	0.9	1.9135	1.8	0.1697	1.5	0.87	1010.5	14.4	1085.8	11.8	1240.1	17.3	1240.1	17.3
-Spot 181	310	226585	0.9	12.1818	0.9	2.2332	2.1	0.1974	1.9	0.90	1161.3	20.4	1191.5	15.0	1246.8	18.3	1246.8	18.3
-Spot 29	2018	129459	14.7	12.0227	0.7	2.2561	1.3	0.1968	1.1	0.86	1158.1	11.9	1198.7	9.2	1272.5	12.9	1272.5	12.9
-Spot 250	111	13388	1.0	11.8931	1.0	2.5645	1.4	0.2213	1.0	0.71	1288.7	11.3	1290.6	10.0	1293.6	18.7	1293.6	18.7
-Spot 286	309	66512	1.1	11.8722	0.7	2.4689	1.2	0.2127	1.0	0.83	1243.1	11.2	1263.0	8.6	1297.0	13.0	1297.0	13.0

-Spot 295	1965	363006	12.4	11.6857	0.6	2.3948	1.0	0.2031	0.7	0.78	1191.7	8.1	1241.0	6.8	1327.7	11.6	1327.7	11.6
-Spot 42	303	20619	2.2	11.6681	1.2	2.3447	1.7	0.1985	1.3	0.72	1167.3	13.4	1226.0	12.3	1330.6	23.2	1330.6	23.2
-Spot 217	370	73478	61.5	11.6458	0.9	2.4513	2.1	0.2071	1.9	0.90	1213.6	21.2	1257.8	15.4	1334.3	18.0	1334.3	18.0
-Spot 252	254	43332	2.1	11.6414	0.9	2.3029	1.3	0.1945	0.9	0.72	1145.8	9.5	1213.2	8.9	1335.1	16.9	1335.1	16.9
-Spot 289	538	91050	38.3	11.6068	0.9	2.3428	1.3	0.1973	1.0	0.76	1160.8	10.9	1225.4	9.6	1340.8	17.0	1340.8	17.0
-Spot 103	454	217227	5.2	11.4262	0.6	2.4967	1.6	0.2070	1.5	0.92	1212.8	16.5	1271.1	11.8	1371.1	12.2	1371.1	12.2
-Spot 32	587	13974797	2.6	11.2724	0.8	2.7434	1.4	0.2244	1.2	0.83	1305.0	13.8	1340.3	10.4	1397.1	14.8	1397.1	14.8
-Spot 258	207	163326	2.6	11.2550	1.0	2.8867	1.4	0.2357	0.9	0.68	1364.5	11.6	1378.4	10.4	1400.1	19.3	1400.1	19.3
-Spot 111	82	34399	1.1	11.0073	1.0	3.1791	1.5	0.2539	1.2	0.77	1458.6	15.1	1452.1	11.7	1442.6	18.6	1442.6	18.6
-Spot 256	772	149559	2.1	10.8530	0.8	3.1012	1.6	0.2442	1.4	0.88	1408.6	18.3	1433.0	12.6	1469.4	14.6	1469.4	14.6
-Spot 50	1358	650915	5.9	10.8180	1.1	3.1444	1.6	0.2468	1.2	0.74	1422.0	15.2	1443.6	12.4	1475.6	20.3	1475.6	20.3
-Spot 168	648	315021	1.9	10.7929	0.9	3.0646	1.7	0.2400	1.4	0.86	1386.7	18.0	1423.9	12.8	1480.0	16.3	1480.0	16.3
-Spot 2	371	33353	3.7	10.7547	2.4	2.9190	2.7	0.2278	1.2	0.46	1322.9	14.7	1386.9	20.1	1486.7	44.7	1486.7	44.7
-Spot 76	67	32603	1.7	10.5216	0.7	3.0054	1.3	0.2294	1.1	0.82	1331.6	12.8	1409.0	9.9	1528.1	14.0	1528.1	14.0
-Spot 313	196	57561	1.0	10.5130	0.7	3.4233	1.2	0.2611	1.0	0.84	1495.6	13.7	1509.8	9.6	1529.6	12.7	1529.6	12.7
-Spot 43	2550	72496	41.5	10.4402	0.6	3.7486	1.1	0.2840	0.9	0.84	1611.3	13.3	1581.8	8.9	1542.7	11.3	1542.7	11.3
-Spot 64	161	797278	0.9	10.4076	0.6	3.6893	1.3	0.2786	1.2	0.89	1584.3	16.6	1569.1	10.6	1548.6	11.4	1548.6	11.4
-Spot 115	1065	1014632	4.3	10.3263	1.0	3.7297	2.5	0.2795	2.3	0.92	1588.6	32.4	1577.8	20.0	1563.3	18.2	1563.3	18.2
-Spot 249	182	73941	0.9	10.2783	0.7	3.5266	1.3	0.2630	1.1	0.83	1505.2	14.4	1533.2	10.2	1572.0	13.4	1572.0	13.4
-Spot 148	600	75700	2.0	10.2460	0.6	3.0050	1.2	0.2234	1.0	0.85	1299.8	11.5	1408.9	8.8	1577.9	11.5	1577.9	11.5
-Spot 114	541	260412	2.3	10.2004	0.7	3.5693	1.4	0.2642	1.2	0.86	1511.1	16.5	1542.7	11.4	1586.3	13.7	1586.3	13.7
-Spot 119	395	207915	3.7	10.1683	0.7	3.0749	1.5	0.2269	1.3	0.86	1318.0	15.1	1426.4	11.3	1592.1	13.9	1592.1	13.9
-Spot 54	161	62468	1.9	10.1441	0.8	3.7615	1.2	0.2769	1.0	0.77	1575.5	13.4	1584.6	9.9	1596.6	14.6	1596.6	14.6
-Spot 176	217	82233	1.2	10.1434	0.6	3.4321	1.5	0.2526	1.3	0.91	1451.9	17.4	1511.8	11.6	1596.7	11.5	1596.7	11.5
-Spot 71	387	119587	1.3	10.1434	0.7	3.3156	1.5	0.2440	1.3	0.88	1407.6	17.0	1484.7	11.9	1596.7	13.2	1596.7	13.2
-Spot 279	229	539181	4.5	10.0297	1.9	3.7562	2.0	0.2734	0.6	0.32	1557.8	8.6	1583.4	15.7	1617.7	34.5	1617.7	34.5
-Spot 287	1588	631021	14.8	10.0273	0.6	3.3699	1.2	0.2452	1.0	0.86	1413.6	12.6	1497.4	9.1	1618.2	11.0	1618.2	11.0
-Spot 75	81	568926	1.2	10.0249	0.9	3.7166	1.6	0.2703	1.3	0.82	1542.5	17.8	1574.9	12.6	1618.6	16.6	1618.6	16.6
-Spot 11	264	437096	1.5	10.0233	0.6	3.8074	1.2	0.2769	1.0	0.85	1575.7	14.2	1594.3	9.6	1618.9	11.5	1618.9	11.5
-Spot 104	182	49952	1.6	10.0227	1.7	3.4499	2.1	0.2509	1.2	0.58	1443.0	15.4	1515.8	16.2	1619.0	31.2	1619.0	31.2
-Spot 245	1251	223062	18.0	9.9997	0.6	3.9292	1.2	0.2851	1.0	0.86	1616.9	14.3	1619.7	9.4	1623.3	11.0	1623.3	11.0
-Spot 52	269	142291	1.6	9.9533	0.7	3.6912	1.5	0.2666	1.3	0.88	1523.4	18.2	1569.5	12.1	1632.0	13.1	1632.0	13.1
-Spot 94	462	102945	1.7	9.9471	0.7	4.0405	1.5	0.2916	1.3	0.90	1649.6	19.1	1642.4	11.9	1633.1	12.1	1633.1	12.1
-Spot 276	127	59602	1.3	9.8113	0.8	3.3684	1.2	0.2398	0.9	0.76	1385.6	11.7	1497.1	9.7	1658.6	14.9	1658.6	14.9
-Spot 24	961	299138	1.6	9.8102	0.7	3.9681	1.5	0.2825	1.3	0.89	1603.7	18.6	1627.7	11.9	1658.8	12.3	1658.8	12.3
-Spot 157	228	201077	0.9	9.6921	0.8	3.9550	1.4	0.2781	1.2	0.83	1581.9	16.4	1625.0	11.3	1681.2	14.2	1681.2	14.2
-Spot 164	595	350261	5.0	9.6707	1.1	3.9119	1.8	0.2745	1.4	0.80	1563.6	20.0	1616.1	14.6	1685.3	20.2	1685.3	20.2
-Spot 149	1064	793294	2.7	9.5902	0.9	3.9133	1.6	0.2723	1.4	0.82	1552.5	18.8	1616.4	13.3	1700.7	17.2	1700.7	17.2
-Spot 40	717	380925	21.8	9.3815	0.7	4.4537	1.6	0.3032	1.5	0.90	1707.0	22.1	1722.4	13.6	1741.1	13.0	1741.1	13.0
-Spot 118	605	379961	3.0	9.3804	0.7	4.5359	1.3	0.3087	1.1	0.84	1734.5	17.0	1737.6	11.1	1741.3	13.3	1741.3	13.3
-Spot 200	694	106288	6.4	9.2722	0.6	4.2250	1.3	0.2842	1.1	0.87	1612.7	15.5	1678.9	10.3	1762.6	11.2	1762.6	11.2
-Spot 223	214	178745	2.7	9.2466	0.7	4.7300	1.3	0.3173	1.1	0.82	1776.8	16.4	1772.6	10.8	1767.6	13.4	1767.6	13.4
-Spot 267	344	109067	1.2	9.2107	0.7	4.1318	1.2	0.2761	1.0	0.81	1571.9	13.4	1660.6	9.7	1774.7	12.8	1774.7	12.8
-Spot 31	288	368628	2.4	9.1766	0.9	4.6981	2.4	0.3128	2.2	0.92	1754.6	33.4	1766.9	19.8	1781.5	16.7	1781.5	16.7
-Spot 215	603	339097	2.2	9.1441	0.6	5.0365	1.3	0.3342	1.2	0.89	1858.5	19.1	1825.5	11.2	1788.0	10.8	1788.0	10.8
-Spot 183	251	78126	1.9	8.9168	0.7	4.9923	1.4	0.3230	1.2	0.85	1804.3	19.1	1818.0	12.1	1833.7	13.5	1833.7	13.5
-Spot 293	688	963360	1.9	8.8869	0.8	4.8476	2.5	0.3126	2.3	0.95	1753.4	36.0	1793.2	20.8	1839.8	13.9	1839.8	13.9
-Spot 105	646	19102	2.6	8.8720	0.8	4.2217	1.3	0.2718	1.0	0.78	1549.8	13.6	1678.2	10.3	1842.8	14.1	1842.8	14.1
-Spot 63	149	45134	1.6	8.8126	0.8	5.1785	1.5	0.3311	1.3	0.86	1843.8	20.6	1849.1	12.7	1855.0	13.6	1855.0	13.6
-Spot 219	944	715861	38.4	8.7949	0.9	4.5495	1.8	0.2903	1.5	0.86	1643.1	22.0	1740.1	14.8	1858.6	16.6	1858.6	16.6
-Spot 49	138	122909	1.7	8.7718	0.9	5.1701	1.4	0.3291	1.1	0.79	1833.8	18.2	1847.7	12.2	1863.4	15.7	1863.4	15.7
-Spot 255	436	75100	0.6	8.7158	0.8	4.6626	1.8	0.2949	1.5	0.88	1665.8	22.7	1760.5	14.7	1874.9	14.8	1874.9	14.8
-Spot 126	627	344268	2.1	7.2336	0.8	7.0706	1.5	0.3711	1.2	0.84	2034.6	21.4	2120.3	13.0	2204.6	13.8	2204.6	13.8
-Spot 259	780	250312	2.1	6.5408	0.8	8.4509	1.6	0.4011	1.4	0.85	2174.0	25.0	2280.7	14.4	2377.7	14.1	2377.7	14.1
-Spot 281	1019	253361	9.1	6.5336	0.6	9.2469	1.3	0.4384	1.2	0.90	2343.3	23.3	2362.8	12.1	2379.6	10.0	2379.6	10.0
-Spot 235	565	164392	1.8	6.2870	0.7	10.1589	1.5	0.4634	1.4	0.90	2454.7	27.8	2449.3	14.0	2444.9	11.2	2444.9	11.2
-Spot 195	287	55047	1.4	6.1690	0.8	10.2218	1.6	0.4575	1.3	0.87	2428.7	27.3	2455.1	14.4	2476.9	13.0	2476.9	13.0
-Spot 46	347	343052	4.5	6.0836	0.9	10.6389	1.5	0.4696	1.2	0.82	2481.9	25.2	2492.1	13.9	2500.4	14.4	2500.4	14.4
-Spot 15	402	251967	1.4	6.0790	0.6	9.4667	1.3	0.4176	1.2	0.89	2249.4	21.9	2384.3	12.0	2501.7	10.2	2501.7	10.2
-Spot 311	500	328885	3.7	5.7669	0.4	11.9185	1.0	0.4987	0.9	0.91	2608.3	19.7	2598.0	9.4	2590.0	6.8	2590.0	6.8
-Spot 65	130	95439	0.9	5.7053	0.8	10.8928	1.5	0.4509	1.2	0.83	2399.4	25.0	2514.0	14.0	2607.9	13.9	2607.9	13.9
-Spot 1	280	428291	2.1	5.5191	0.6	10.8417	1.6	0.4342	1.5	0.92	2324.5	29.4	2509.6	15.3	2663.0	10.8	2663.0	10.8
-Spot 216	367	137257	2.4	5.4487	0.8	12.1310	2.5	0.4796	2.4	0.95	2525.5	50.6	2614.6	23.8	2684.3	12.5	2684.3	12.5
-Spot 97	71	84019	2.5	5.0495	0.8	13.8654	1.3	0.5080	1.0	0.79	2648.1	22.3	2740.6	12.3	2809.4	13.0	2809.4	13.0
-Spot 263	104	67300	1.9	4.9628	0.8	15.0073	1.2	0.5404	0.9	0.77	2785.1	21.2	2815.7	11.6	2837.6	12.7	2837.6	12.7
-Spot 264	154	101059	0.8	4.4959	0.7	13.8304	1.2	0.4512	0.9	0.80	2400.4	18.9	2738.2	11.2	2997.6	11.4	2997.6	11.4
